# Transcriptome Analysis of the Emerald Ash Borer (EAB), *Agrilus planipennis*: *De Novo* Assembly, Functional Annotation and Comparative Analysis

**DOI:** 10.1371/journal.pone.0134824

**Published:** 2015-08-05

**Authors:** Jun Duan, Tim Ladd, Daniel Doucet, Michel Cusson, Kees vanFrankenhuyzen, Omprakash Mittapalli, Peter J. Krell, Guoxing Quan

**Affiliations:** 1 Great Lakes Forestry Centre, Canadian Forest Service, Natural Resources Canada, Sault Ste. Marie, Ontario, Canada; 2 Laurentian Forestry Centre, Canadian Forest Service, Natural Resources Canada, Québec City, Québec, Canada; 3 Département de biochimie, de microbiologie et bio-informatique, Université Laval Québec City, Québec, Canada; 4 Department of Entomology, Ohio Agricultural and Research Development Center, The Ohio State University, Wooster, Ohio, United States of America; 5 Department of Molecular and Cellular Biology, University of Guelph, Guelph, Ontario, Canada; Kansas State University, UNITED STATES

## Abstract

**Background:**

The Emerald ash borer (EAB), *Agrilus planipennis*, is an invasive phloem-feeding insect pest of ash trees. Since its initial discovery near the Detroit, US- Windsor, Canada area in 2002, the spread of EAB has had strong negative economic, social and environmental impacts in both countries. Several transcriptomes from specific tissues including midgut, fat body and antenna have recently been generated. However, the relatively low sequence depth, gene coverage and completeness limited the usefulness of these EAB databases.

**Methodology and Principal Findings:**

High-throughput deep RNA-Sequencing (RNA-Seq) was used to obtain 473.9 million pairs of 100 bp length paired-end reads from various life stages and tissues. These reads were assembled into 88,907 contigs using the Trinity strategy and integrated into 38,160 unigenes after redundant sequences were removed. We annotated 11,229 unigenes by searching against the public nr, Swiss-Prot and COG. The EAB transcriptome assembly was compared with 13 other sequenced insect species, resulting in the prediction of 536 unigenes that are Coleoptera-specific. Differential gene expression revealed that 290 unigenes are expressed during larval molting and 3,911 unigenes during metamorphosis from larvae to pupae, respectively (FDR< 0.01 and log2 FC>2). In addition, 1,167 differentially expressed unigenes were identified from larval and adult midguts, 435 unigenes were up-regulated in larval midgut and 732 unigenes were up-regulated in adult midgut. Most of the genes involved in RNA interference (RNAi) pathways were identified, which implies the existence of a system RNAi in EAB.

**Conclusions and Significance:**

This study provides one of the most fundamental and comprehensive transcriptome resources available for EAB to date. Identification of the tissue- stage- or species- specific unigenes will benefit the further study of gene functions during growth and metamorphosis processes in EAB and other pest insects.

## Introduction

The emerald ash borer (EAB), *Agrilus planipennis*, a coleopteran insect, is a destructive forest pest of ash trees [1]. It is thought to have been introduced from Asia in wood packing material in the mid-1990s and was first detected in the Detroit-Windsor area of Michigan, USA and Ontario, Canada in 2002 [2]. So far, EAB has killed or infested approximately 70 million ash trees in North America. It is still continuing to spread into new areas, which poses a threat to an estimated 10 billion ash trees in Canada and the US [3]. It was estimated that the costs of treatment, removal and replacement of trees affected by EAB in Canadian municipalities may reach $2 billion over a 30-year period [4]. In the 25 US states where EAB is present, the costs are estimated to reach approximately $10.7 billion for the period of 2009–2019 [5]. In addition to negative economic impacts, the loss of ash component is accompanied by ecological impacts such as altered forest succession dynamics and biogeochemical cycling [6].

The EAB life cycle is usually completed in one or two years. Larvae feed under the bark of ash trees, on phloem and cambial tissues, making s-shaped galleries in the process. Under high larval densities this activity disrupts the flow of nutrients and water leading to tree mortality within 2–4 years. At the end of the fourth larval instar, the larvae carve a pupal chamber into the heartwood of the tree to overwinter as pre-pupae. Following pupal development, adult EAB bore out of the trees in the spring-early summer to feed on leaves. The EAB adult life is relatively short, lasting 2 to 3 weeks [2].

Efforts have been made on several fronts to slow the spread of EAB, including the establishment of quarantine zones to limit the transport of unfinished wood products such as firewood, the characterization of entomopathogens and the release of parasitoids introduced from China for control [7–9]. A few chemical control products are effective against EAB. Up until now, three systemic insecticides, TreeAzin, Confidor, Acecap97 are fully registered in Canada for EAB control. TreeAzin, produced from extracts of neem tree seeds, is currently the most widely used, because of its reduced impacts on non-target organisms [10, 11]. However, it is more expensive than the other two. While these three injectable products afford EAB control with favorable cost of application/environmental effects trade-offs, there is still a need for additional control approaches.

Recent advances in biotechnology may offer new EAB control options. For instance, RNA interference (RNAi) provides a new tool for pest control [12]. Knock down of essential genes with specifically tailored double-stranded RNA (dsRNA) molecules can trigger lethality in a pest insect, and these molecules are being considered for field deployment in pest insect management [13–15]. Although several factors may affect the effectiveness of RNAi, a key step is in the selection of the target gene. Identification of target genes of a pest insect requires extensive and detailed molecular knowledge of its genome and/or transcriptome, particularly the essential genes implicated in insect growth, development or survival. However, until recently limited knowledge on EAB molecular biology has hindered such research and development.

EAB transcriptomes from specific tissues including midgut, fat body and antenna were previously sequenced using the Roche 454 pyrosequencing platform [16, 17]. However, because of limited sequence depth, the obtained sequences displayed relatively low gene coverage and completeness. Sequencing-by-synthesis technology (e.g. Illumina) makes it possible to recover full transcripts at high depths of coverages and at a low cost. In this study, we used the RNA-Seq technology to sequence entire EAB transcriptomes from four developmental stages (larvae, pre-pupae, pupae, adults) and two tissues (midguts from larvae and adults). A combined reference transcriptome was assembled by *de novo* assembly of all the transcriptomes followed by gene annotation. The relationship between EAB and 13 other insect species, in terms of orthologous gene content, was assessed. A large number of genes showing stage- or tissue-specific expression were observed. These data provide a valuable reference of expressed genes for molecular studies in EAB and the management of this pest. This gene resource should also be highly valuable in the comparative molecular biology of Coleopterans and other insect pests.

## Materials and Methods

### EAB collection and RNA preparation

The study was carried out on private land. The owner of the land gave permission to collect the material. Transport of the isolated material was done with appropriate permission from the Canadian Food Inspection Agency. EAB larvae, pre-pupae and pupae were collected from infested ash trees in Dutton, Ontario (42.65882N, 81.55498W), while adult males and females were obtained from infested logs from those locations that were incubated at room temperature. Four EAB individuals or four midguts were pooled for total RNA isolation. The developmental stages were distinguished using the criteria of Chamorro et al. [18]. Gut contents were removed from all samples prior to RNA isolation. PureLink RNA Micro Scale Kit (Life Technologies) was used to isolate RNA. The quality of RNA was assessed by running samples on agarose gel.

### Sequencing and *de novo* assembly

RNA samples were sent to LC Science (Houston, TX, USA) and poly (A) RNAs were sequenced in a paired-end pattern on Illumina HiSeq 2000 (Illumina Inc., San Diego, CA, USA). FastQC was used for quality checking [19]. The raw sequencing reads were filtered stringently before assembly. All the reads were aligned against the ncRNA database of rRNA, tRNA and mtDNA using Bowtie 2 [20] to filter out non-coding RNAs. Raw reads were then processed by trimming the adaptor sequences and filtering low quality sequences with Trimmomatic (LEADING: 20, TRAILING: 20, SLIDINGWINDOW: 4:15) [21]. Reads that were longer than 50 bp and passed quality control were assembled with the Trinity *de novo* transcriptome assembly software with a default k-mer of 25 [22]. To remove the redundancy of Trinity results, only the longest transcript contig was selected for each gene. These transcripts were assembled into unigenes using TGICL [23]. Unigenes longer than 300 bp were used for subsequent assembly and analysis.

The raw paired-end reads of each sample were aligned back onto the unigenes using Bowtie 2 to check for quality of the assembly. The correct pairs of reads were calculated by perl script of alignReads.pl in Trinity. Open reading frames (ORFs) in the unigenes were predicted by TransDecoder (http://transdecoder.sourceforge.net/), with 300 bp set as the minimum ORF length.

### Rarefaction analysis

Rarefaction analysis was used to evaluate whether sufficient sequencing depth was achieved. Seven libraries with reads from 0.1 to 100 million pairs of reads were randomly generated from the combined EAB RNA-Seq libraries. These libraries were aligned against the official *T*. *castaneum* gene dataset by RAPSearch2 [24] using an e-value threshold of 10^−6^. The number of *T*. *castaneum* genes that were hit by reads at least 1, 10, 50, 100 and 200 times were recorded.

### Functional annotation

Assembled unigenes were compared to the NCBI non-redundant (nr) database, Swiss-Prot database and COG (Eukaryotic Orthologous Groups) database using BLASTx with a cut-off e-value of 10^−6^. Putative gene functions were assigned based on the most similar genes with the lowest BLAST e-value and highest bit score in those databases. Gene ontology analysis was performed with the Blast2GO software [25]. Only the top 10 BLAST hits against the nr database (e-value < 10^−6^) were considered for assigning GO terms. BiNGO was used to perform hypergeometric statistical test of significance enrichment GO term (p-value < 0.05) [26].

### Comparative analysis of unigenes

Orthologs between EAB and *Tribolum castaneum* were identified via a reciprocal best-hits BLAST method using a cut-off E-value of 10^−6^. To obtain an overview of EAB gene density on the *T*. *castaneum* genome, the predicted transcripts were used to BLAST against the *T*. *castaneum* genome. The regions of best hits were used for analysis of density. A customized PERL script was written to analyze the density of best hit with a window of 10 kb. The PERL script is available from the authors upon request. The graph of density distribution was drawn using the Circos program [27].

To identify species-specific unigenes, the predicted transcripts were compared with two vertebrates, *Homo sapiens* and *Gallus gallus*, and thirteen insect species, including: *T*. *castaneum*. *Bombyx mori*, *Drosophila melanogaster*, *Anopheles gambiae*, *Apis mellifera*, *Acyrthosiphon pisum*, *Pediculus humanus*, *Nasonia vitripennis*, *Harpegnatos saltator*, *Plutella xylostella*, *Camponotus floridanus* and *Danaus plexippus*. The orthologous relationships were analyzed by OrthoMCL [28]. To determine the phylogenetic relationship with other species, the 1:1:1 conserved genes were combined together and analyzed using the Hal pipeline [29], which can find conserved regions in protein sequences. ProTest [30] was used to predict optimal evolutionary models for maximum likelihood analysis. RAxML [31] was used to infer a phylogenetic tree based on the conserved region. The phylogenetic tree was further visualized by figtree (http://tree.bio.ed.ac.uk/software/figtree/).

### Identification of RNAi related genes

Core RNAi components present in EAB contigs were searched using previously annotated RNAi genes in the *T*. *castaneum* genome [32], including TcSid-A (EF688527), TcSid-B (EF688528), TcSid-C (EF688529), Tc_Dcr-1 (EU273918), Tc_Dcr-2 (EU273919), Tc_R2D2 (EU273920), Tc_C3PO (EU273921), Tc-Loqs (XP_966668), Tc_Pasha (XP_971282), Tc_ago1 (EU273915), Tc_ago2 (EU273916) and Tc_Snp (EF688530). Textual and sequence similarity searches were performed to identify the homologs of these genes in *D*. *melanogaster*, *C*. *elegans* and *B*. *mori* through database of WormBase [33], FlyBase [34] and SilkDB [35]. Candidate genes in EAB were manually validated by BLASTing against nr database at the NCBI web site.

### Unigene abundance analysis

The filtered RNA-Seq reads for each sample were mapped against the unigenes generated from all libraries using the Bowtie 2 package. The BAM files generated by Bowtie 2 were then used to estimate the unigene abundance in each library using RSEM software [36]. Unigene qualification was measured by read counts and FPKM (Fragments Per Kilobase per Million) [37]. FPKM expression values were normalized by TMM (Transcripts Per Million) methods. The differential expression unigenes were analyzed based on read counts using the edgeR in R Bioconductor [36]. The threshold was set as FDR < 0.01 and the absolute value of Log_2_^fold change^ (Log_2_^FC^> 2).

### Quantitative Real-Time Polymerase Chain Reaction (qRT-PCR)

Total RNA was isolated using a Purelink RNA Micro kit, according to the manufacturer’s instructions (Invitrogen, life technologies) as described above. The absence of DNA contamination in the RNA samples was confirmed by conducting PCR with no reverse transcription RNA as a template. First-strand cDNA samples were generated from 4 μg of total RNA, using the Ready-To-Go T-Primed First-Strand kit (Amersham Biosciences). Primers for each selected gene were designed using Primer3 (http://bioinfo.ut.ee/primer3/). Primer sequences and product sizes are provided in S5 Table. PCR reactions were performed in a final volume of 20 μL using 2.0 μL of cDNA (diluted 1:5), 10 μL of Takara SYBR ExTaq premix reagent on a Rotor-Gene RG-3000 thermal cycler with technical replicates (Corbett Research). The PCR conditions were as follow: 40 cycles at 95°C for 10s, and 65°C for 20s. After melting curve analysis, the relative quantities of each transcript were assessed using the 2^-∆∆CT^ method [38], with the housekeeping gene translation elongation factor 1α (TEF-1α) as the reference gene [39].

## Results

### Illumina sequencing

RNA samples prepared from four developmental stages (larvae, pre-pupae, pupae, adults) and two tissues (larval and adult midgut) were sequenced on an illumina HiSeq 2000 platform. A total of 473.9 million pairs of 100 base length paired-end reads were generated (Table 1). These reads were subjected to a quality filter prior to subsequent transcript assembly and analysis. After discarding low quality sequences and filtering ncRNA including rRNA, tRNA and mitochondrial RNA, a total of 332.3 million pairs of reads (≥ 50 bases) were retained. The percent of bases with a quality of over 20 (base call accuracy over 99%) reached 98.9%. The raw reads in FASTQ format were deposited in the Short Read Archive (SRA) database of the National Centre for Biotechnology Information (NCBI) under the accession number SRP048676.

**Table 1:** Summary statistics of the RNA-Seq data for assembly.

		Raw data		After filtering					Aligning to Unigenes		
Library name	Source	Pairs of Read (#)	GC%	Pairs of Read (#)	GC%	Insert Size (bp)	%bases with quality score >20	AveragePhred score	No. of aligned Reads	Reads in Proper pairs	%
EAB3M	Third instar molting larvae	78,333,353	40	66,867,193	40	165±43	98.9	36.4	89,595,908	80,960,794	90.4
EAB4F	Fourth instar feeding larvae	71,653,629	39	61,031,838	39	170±44	99.0	36.5	90,000,926	82,411,672	91.6
EABPP	Pre-pupae	95,813,260	40	46,253,178	40	178±41	97.9	35.5	75,238,260	70,730,656	94.0
EABP0	Pupa at 0 hour	107,175,780	40	50,121,042	39	176±40	97.9	35.5	84,511,516	79,953,482	94.6
EABLM	Larval midguts	39,302,489	41	36,219,832	41	184±46	99.4	37.1	42,355,565	38,615,954	91.2
EABAM	Adult Midguts	42,646,390	40	38,745,997	41	171±38	99.5	37.2	46,871,897	42,612,406	90.9
EABA	Adult	38,950,441	40	33,035,686	40	174±39	99.5	37.2	52,944,831	49,734,610	93.9
Total		473,875,342		332,274,766					481,518,903	445,019,574	

**Fig 1:**
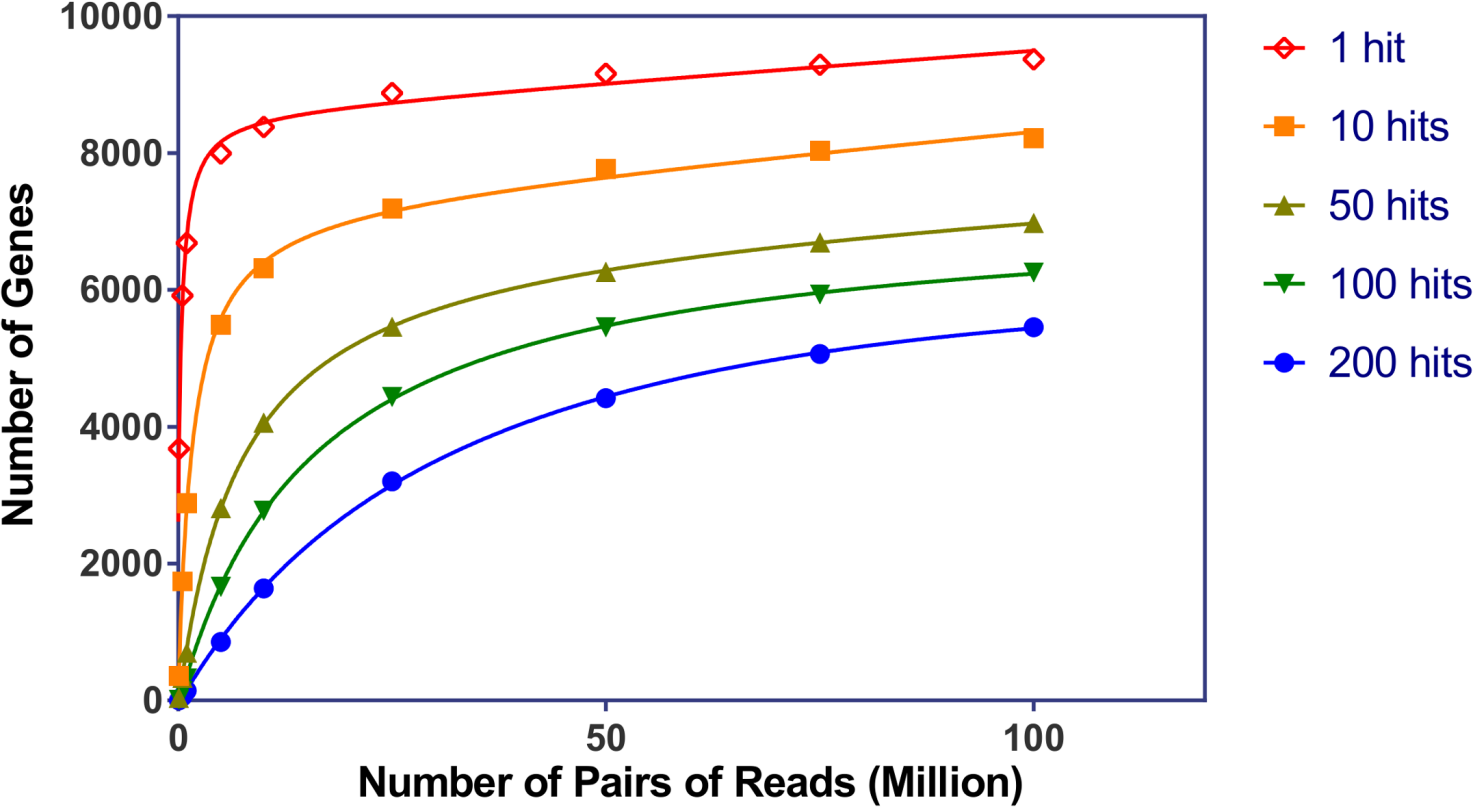
Rarefaction analysis of the gene representation. Randomly sampled reads from the combined seven sequenced libraries were aligned against the *T*. *castaneum* official gene dataset. The number of genes that were hit by reads at least 1, 10, 50, 100 and 200 times was recorded.

To evaluate whether sufficient sequencing depth was achieved to cover EAB genes, the relationship between sequencing depth and the number of gene discoveries was examined by a rarefaction analysis. Because the complete genomic sequence of EAB is not available, we adopted a random re-sampling method and compared these reads with the closely related species *T*. *castaneum*. Increments of random sampled reads from 0.1 to 100 million pairs of reads were aligned to the *T*. *castaneum* official genes dataset. As shown in Fig 1, 9,292 genes can be detected with at least one pair of reads using the random dataset of 75 million paired reads, while 6,695 genes are represented by at least 50 pairs of reads. Increasing the dataset to 100 million pairs of reads, resulted in the detection of 9,373 while 6,976 genes are represented by at least 50 pairs of reads. These results indicate that the gene discovery rate is saturated above 75 million pairs of reads. Since the total numbers of pre-filtered reads exceeds 332.3 million pairs, the dataset is considered sufficient to detect most of the genes in the samples.

### *De novo* assembly

All pre-filtered reads were combined and assembled using the Trinity software. A summary of the assembly statistics is provided in Table 2. The assembly generated 88,907 contigs (201.8 Mb) longer than 300 bp. After discarding redundant contigs, the list was narrowed to 38,160 unigenes (47.3 Mb) (S1 Text). The average GC content of the unigenes is approximately 35%. The unigene dataset has an N50 of 2.5 kb and 220 unigenes are longer than 10 kb. The longest one, EABT33086, is 32.1 kb. This gene displays similarity to a microtubule actin cross-linking factor (MACF, 25.4 kb long) gene from *T*. *castaneum*. EABT33086 covers more than 95% of the coding region of the MACF gene. The MACF gene encodes a large linker protein that interacts with the actin and microtubule cytoskeleton [40]. These results indicate that our assembly strategy performed well in covering long transcripts.

**Table 2:** Summary statistics of the *de novo* EAB transcriptome assembly.

	Statistics
Number of Reads Used in Assembly	598,478,160
Number of Contigs	88,907
Total Size of Contigs (Mb)	201.8
Number of Unigenes	38,160
Total Size of Unigenes (Mb)	47.3
Mean Unigene size (bp)	1,241
Median Unigene size (bp)	874
Min Unigene Size (bp)	300
Max Unigene Size (bp)	32,109
N25 (bp)	5,350
N50 (bp)	2,516
N75 (bp)	848
Number of Unigene > 1 Kb	11,251
Number of Unigene > 5 Kb	1,722
Number of Unigene > 10 Kb	225
Number of Predicted ORFs	15,079
NR Database Nnnotated Unigenes	10,975
Swissprot Database Annotated Unigenes	8,446
COG Classified Unigenes	7,080

More than 481.5 million pre-filtered reads could be mapped back to unigenes (Table 1). Of these, 445.0 million (92.4%) could be mapped to the unigenes in proper paired relationships, while in 24.9 million (5.2%) paired reads only one side of the reads could be aligned, and a further 11.6 million (2.4%) had improper paired relationships. These results indicate that most of the unigenes were correctly assembled. Frequency distribution of the number of reads showed that 99.5% of the unigenes were supported by more than 10 reads (S1 Fig). Approximately 70% of these unigenes had 15 to 250 reads mapped to them, which implies that there are sufficient reads to support a majority of the unigenes. There was also a minor peak around 2,500 reads per unigene (S1 Fig). These unigenes with higher reads may belong to house-keeping genes or other highly expressed genes.

### Assessment of completeness

**Fig 2:**
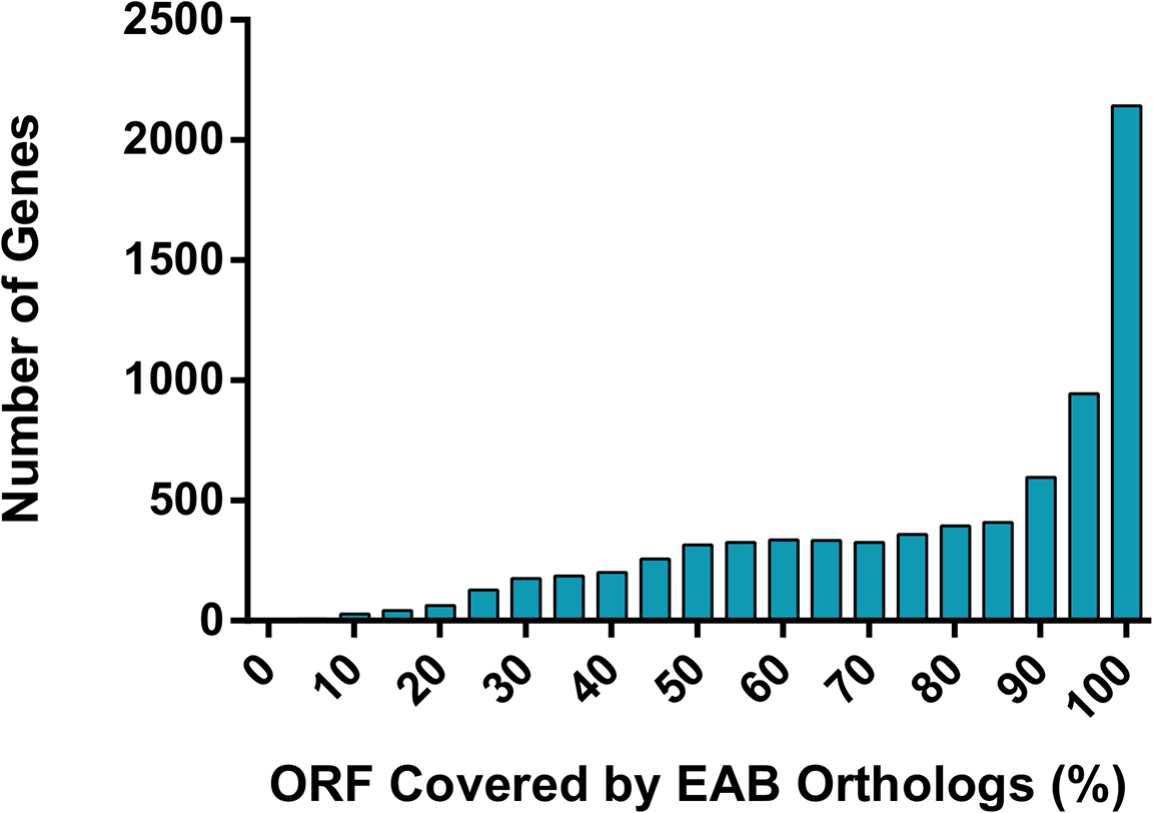
Completeness analysis of the assembly. A total of 7,580 *T*. *castaneum* genes orthologs can be found in EAB with cut-off E-value of 10^−6^ by reciprocal best-hits BLAST method. Of these, 2,143 (28.3%) *T*. *castaneum* genes can be matched with 100% alignment coverage, while 5,505 (72.6%) can be matched with > 70% coverage.

**Fig 3:**
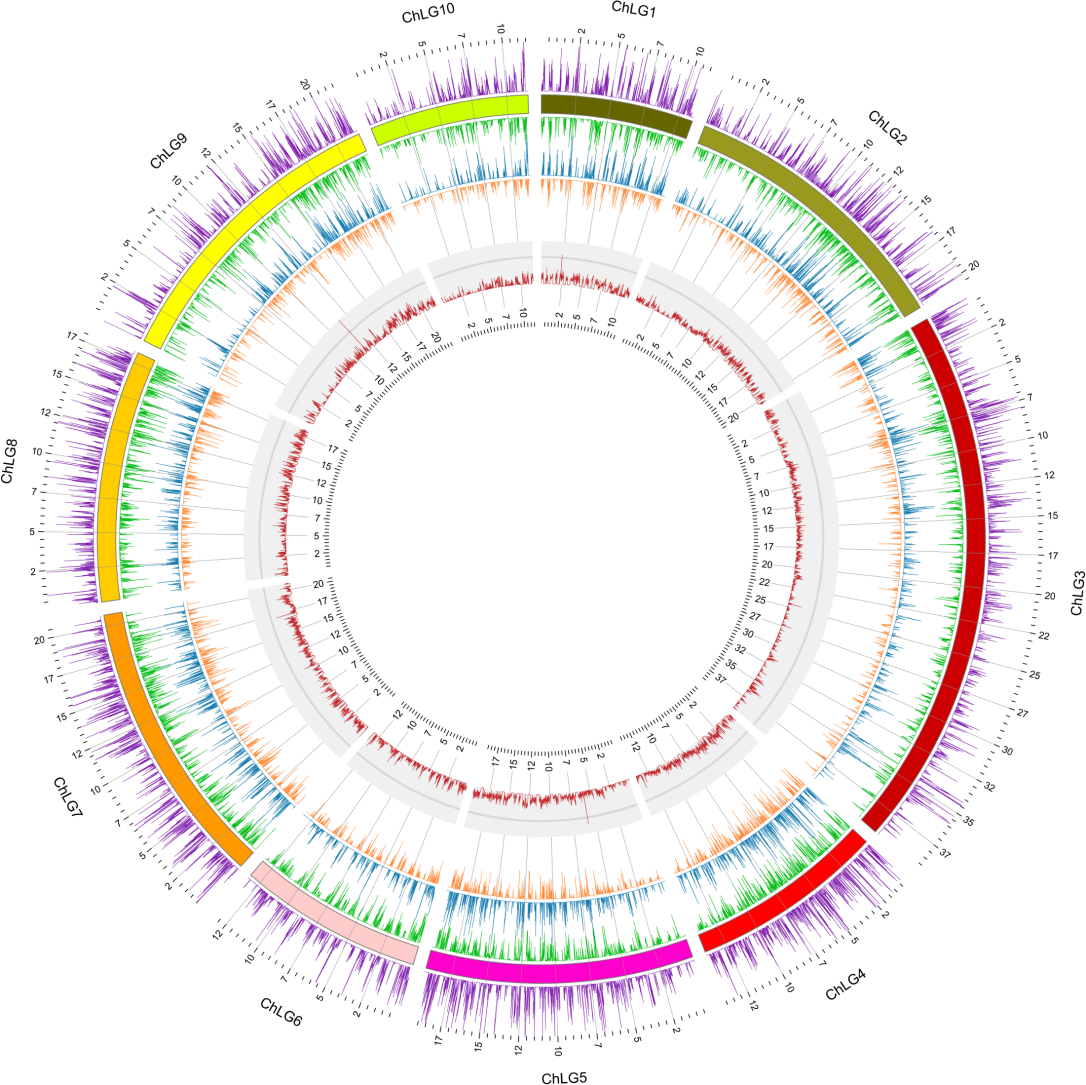
Abundance and distribution of the EAB transcripts compared to *T*. *castaneum* genome. The transcripts with ORFs longer than 300 bp were compared to the *T*. *castaneum* genome. A window of 10 kb was adopted to analyze the density distribution of the best hit region. External track shows *T*. *castaneum* gene density in both + (outside) and—(inside) strands. The middle track shows the density of the alignments of EAB transcripts to *T*. *castaneum* genome, in both + (outside) and—(inside) strands. Inner-most track shows the GC profile of *T*. *castaneum* genome.

The completeness of the transcriptome assembly was evaluated in two ways. The CEGMA (Core Eukaryotic Genes Mapping Approach) program was used to assess the representation of core eukaryotic proteins in the assembly. CEGMA defines a representative set of 248 ultra-conserved Core Eukaryotic Genes (CEGs), which are mostly housekeeping genes [41], and therefore are expected to be expressed in EAB. Analysis of the assembled unigenes identified 229 of the 248 core proteins (92.3%) as complete (defined as covering more than 75% of the length of the core proteins by global alignment). Completeness was also evaluated by the ability of reconstructing of the unigenes with full-length proteins. All unigenes were scanned for potential open reading frames (ORFs). A total of 15,079 transcripts with ORFs longer than 300 bp were predicted. These transcripts were compared to the *T*. *castaneum* official gene set by reciprocal best-hits BLAST method. We identified 7,580 EAB transcripts with corresponding homologs in *T*. *castaneum* with a cut-off E-value of 10^−6^ (Fig 2). Of these, 2,143 (28.3%) EAB transcripts can be matched with 100% alignment coverage, while 5,505 (72.6%) can be matched with > 70% coverage. All predicted transcripts were further compared to the *T*. *castaneum* genome. The density distribution of the best hit region was analyzed. A high correlation (R = 0.81) of the best hit regions with *T*. *castaneum* was observed (Fig 3), which indicates that most of the predicted EAB transcripts are represented in *T*. *castaneum*. Taken together, both analyses suggest that our EAB transcriptome assembly had a broad representation.

### Functional annotation and classification

**Fig 4:**
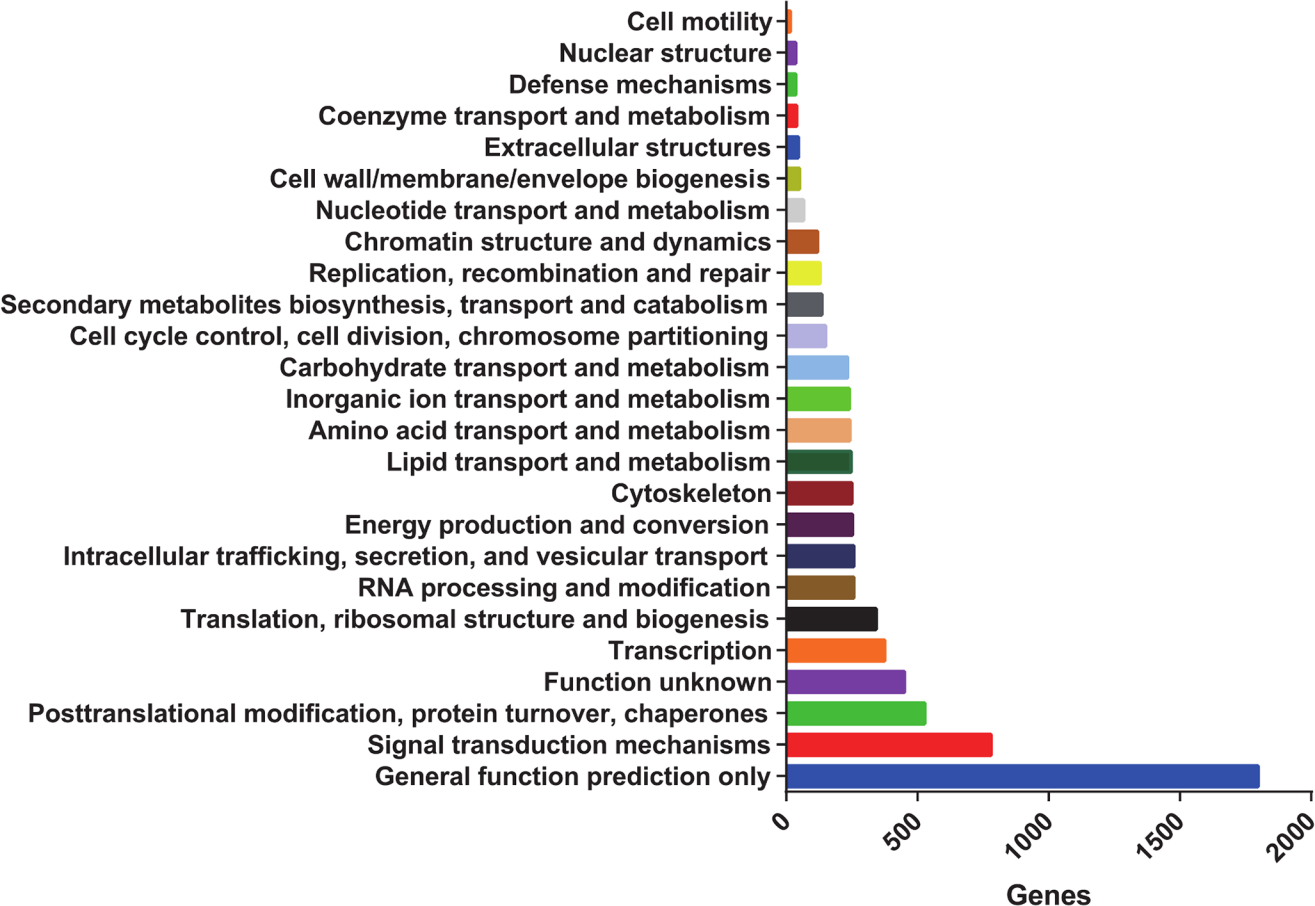
COG function classification of the EAB transcriptome. The EAB unigenes were also aligned to the Clusters of Orthologous Groups (COG) database to predict and classify potential functions. A total of 7,080 unigenes were classified in 25 categories. The categories are shown in the order of increasing numbers of genes/category.

To annotate the EAB unigenes, a homology search was performed by BLASTing them against the Swiss-Port public protein database. Significant hits were obtained for 8,446 (22.1%) unigenes that displayed an E-value of 10^−6^. 10,975 unigenes (28.8%) had corresponding homologs identified by searching against the NCBI nr database, of which, 6,893 exhibited significant similarity to *T*. *castaneum* genes (S2 Fig), indicating the close relationship between EAB and *T*. *castaneum*. The EAB unigenes were also aligned to the Clusters of Orthologous Groups (COG) database to predict and classify potential functions (Fig 4). A total of 7,080 unigenes were COG classified. Among the 24 COG categories, the cluster for ‘general function prediction’ represented the largest group (1,798 unigenes, 25.4%) followed by ‘signal transduction mechanisms’ (780 unigenes, 11.0%) and ‘posttranslational modification, protein turnover and chaperones’ (528 unigenes, 7.5%). Cell motility (36 unigenes, 0.5%) and nuclear structure (16 unigenes, 0.2%) represented the smallest groups. Gene Ontology assignments were also used to classify the functions of the unigenes. In total, 5,325 EAB unigenes were categorized into GO functional groups. The terms “cellular process”, “biological regulation”, “metabolic process” were the most represented in the main category of biological process. In summary, 11,229 unigenes were annotated using nr, Swiss-Prot, COG, and KEGG databases.

Among the unigenes, 26, 931 remain unannotated. A comparison between these unigenes with the 454 sequencing data from Mittapalli *et al* [16, 17, 42] showed that only 3, 298 of our unannotated unigenes (12.2%) could be supported by 454 data. This implies that most of the unannotated unigenes still could not be validated. The mean length of the unannotated unigenes was 628 bp, which is much shorter than the mean length of the 11, 229 annotated unigenes. The unannotated unigenes were assumed to be non-coding RNA or unknown genes.

### Analysis of orthologs

**Fig 5:**
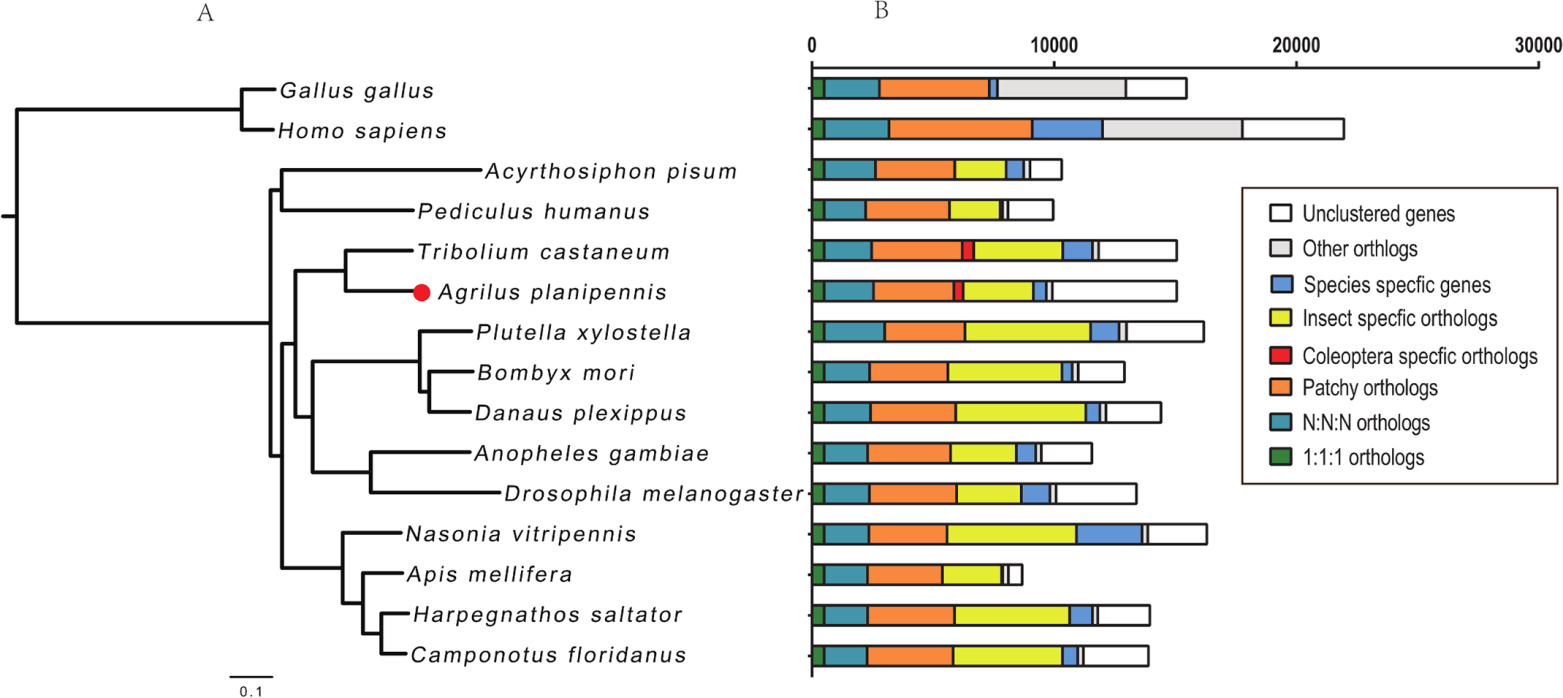
Phylogenetic relationships of orthologs between EAB and other insect species. (A) Phylogenetic relations of EAB with other insect species. A maximum likelihood evolutionary tree was inferred based on concatenated single copy orthologs among EAB and 13 insect species. The vertebrates H. sapiens and G. gallus were used as outgroups. Bar shows a genetic distance of 0.1. (B) The comparison of orthologous gene among EAB and other species. 1:1:1 orthologs include the common orthologs with only one copy in all surveyed species, N:N:N orthologs include the common orthologs with different copy numbers in the different species, patchy orthologs include the orthologs existing in at least one species of vertebrates and insects, Coleoptera-specific orthologs include the orthologs existing only in EAB and *T*. *castaneum*, insect-specific orthologs includes the orthologs existing only in insects, species specific orthologs represent the genes from only one species, other orthologs include the unclassified orthologs, and unclustered orthologs include these that cannot be clustered into known gene families.

To identify the species-specific genes of EAB, the 15,079 transcripts containing an ORF longer than 300 bp were compared against 13 other sequenced insect species (Materials and Methods). Two vertebrate genomes, *G*. *gallus* and *H*. *sapiens*, were used as outgroups. Orthologous relationships among these species were classified based on their sequence similarity (Fig 5). A total of 2,541 EAB unigenes belonging to the ancient group have orthologous relations among all the surveyed insects and vertebrates, of which 507 unigenes contain only one copy in all examined species. These unigenes may belong to essential gene categories and may be subject to strict evolutionary constraints. A maximum likelihood evolutionary tree was inferred based these single copy orthologs. The results showed that EAB has the closest relationship with *T*. *castaneum* (Fig 5). In the comparison against *G*. *gallus* and *H*. *sapiens*, 2,896 EAB unigenes were predicted as insect-specific genes because they had orthologs only in other examined insects but not in *G*. *gallus* and *H*. *sapiens*. We found 536 EAB unigenes that only had orthologs in *T*. *castaneum* but not found in other insect species, suggesting they might be Coleoptera-specfic orthologs (Fig 5). Most of the identified Coleoptera-specfic genes were new and predicted as hypothetical proteins. Only a few of the hypothetical proteins had a predicted function, such as the ecdysis triggering hormone preprotein gene (NP_001165744), odorant binding protein C20, serine protease P72 (EFA09224) and EMSY (XP_001811996), but little is known about their functions in beetles.

### Differentially expressed transcripts during molting and metamorphosis

To analyze the transcript abundance between different development stages, the RNA-Seq reads from third-instar molting larvae, fourth-instar feeding larvae, pre-pupae and pupae were mapped onto the assembled unigenes. The abundance of each transcript was calculated based on the number of reads mapping onto it (S1 Table). The total number of mapped reads in each library ranged from 42.4 to 90 million. As shown in S2 Table, 290 unigenes were identified as differentially expressed between third molt and fourth feeding larvae (Log_2_^FC^ >2; FDR ≤0.01), of which, 97 transcripts were up-regulated and 193 transcripts were down-regulated during the molt between third and fourth instars. Functional enrichment analysis showed that the up-regulated transcripts in the third molt were implicated mainly in metabolic processes, such as oxidoreductase activity, antioxidant activity, peroxidase activity and hydrolase activity. Although we did not observe dramatically enriched transcripts in fourth-instar feeding larvae, a few of the up-regulated transcripts belonged to categories associated with digestion or feeding behavior, such as lipase, chemosensory protein and odorant binding protein were observed.

In this analysis, a total of 3,911 transcripts were differentially expressed between pre-pupae and pupae (S3 Table). Of these, 2,812 were up-regulated and 1,099 were down-regulated, indicating that more genes were activated during the metamorphosis. In the pre-pupal stage, transcripts involved in the processes of anatomical structure morphogenesis, organ development, chitin metabolic process, dendrite morphogenesis, regulation of developmental process and other processes were enriched. In pupae, only transcripts involved in lipid metabolic processes were detected as significantly enriched, indicating that lipid metabolic processes have an important role in this process.

### Differentially expressed transcripts between larval and adult midguts

**Fig 6:**
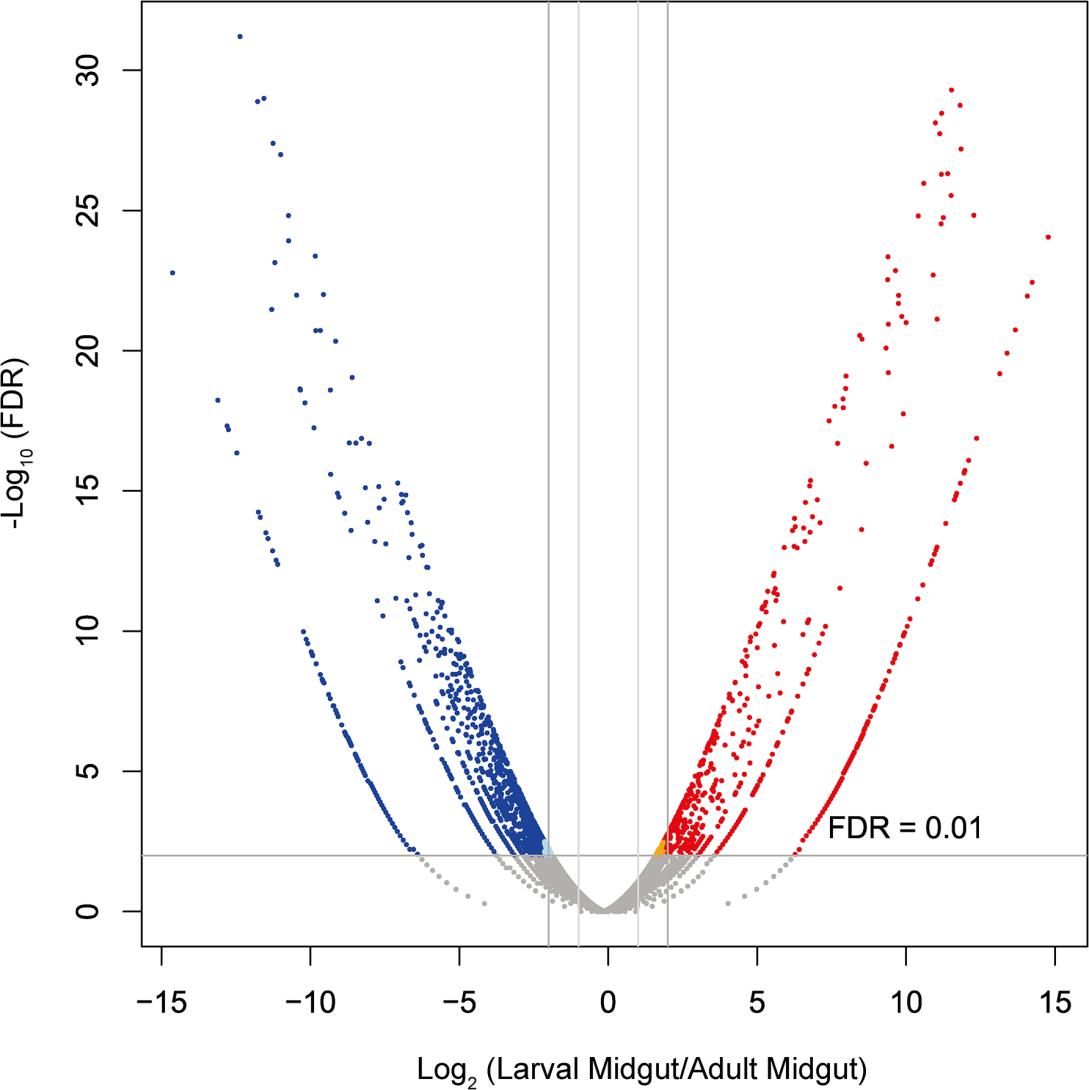
Differential gene expression analysis between larval midguts and adult midguts. The volcano plot shows the magnitude of differential expression of transcripts between larval and adult midguts. Each dot represents a transcript that had detectable expression. The horizontal line marks the threshold (FDR < 0.01) for defining a transcript as up-regulated in larval midguts (blue) or adult midguts (red), with a combined change > 4-fold.

Both the EAB larvae and adults feed, but they consume different parts of the ash tree. The larvae feed exclusively on phloem and cambial tissues, while the adults feed exclusively on foliage. To explore potential factors contributing to EAB diet selection, we compared the midgut transcriptomes of larvae and adults. An overall view of the expression pattern is depicted in Fig 6. A total of 1,167 unigenes were detected as being differentially expressed (S4 Table). Of these, 732 unigenes were up-regulated in adult midgut, and 435 unigenes were up-regulated in larval midgut (Log_2_^FC^>2; FDR <0.01). Functional annotation of these unigenes showed that many genes were associated with digestive physiology and related metabolic processes, including serine protease, trypsin, glucose dehydrogenase, sugar transporter, endoglucanase. The laccase transcript was 22-fold higher in adult midgut than in larval midgut. Transcripts corresponding to alcohol dehydrogenases, aldehyde dehydrogenases, cytochrome P450s were also highly expressed in the adult midgut.

### qRT-PCR validation of differentially expressed transcripts

**Fig 7:**
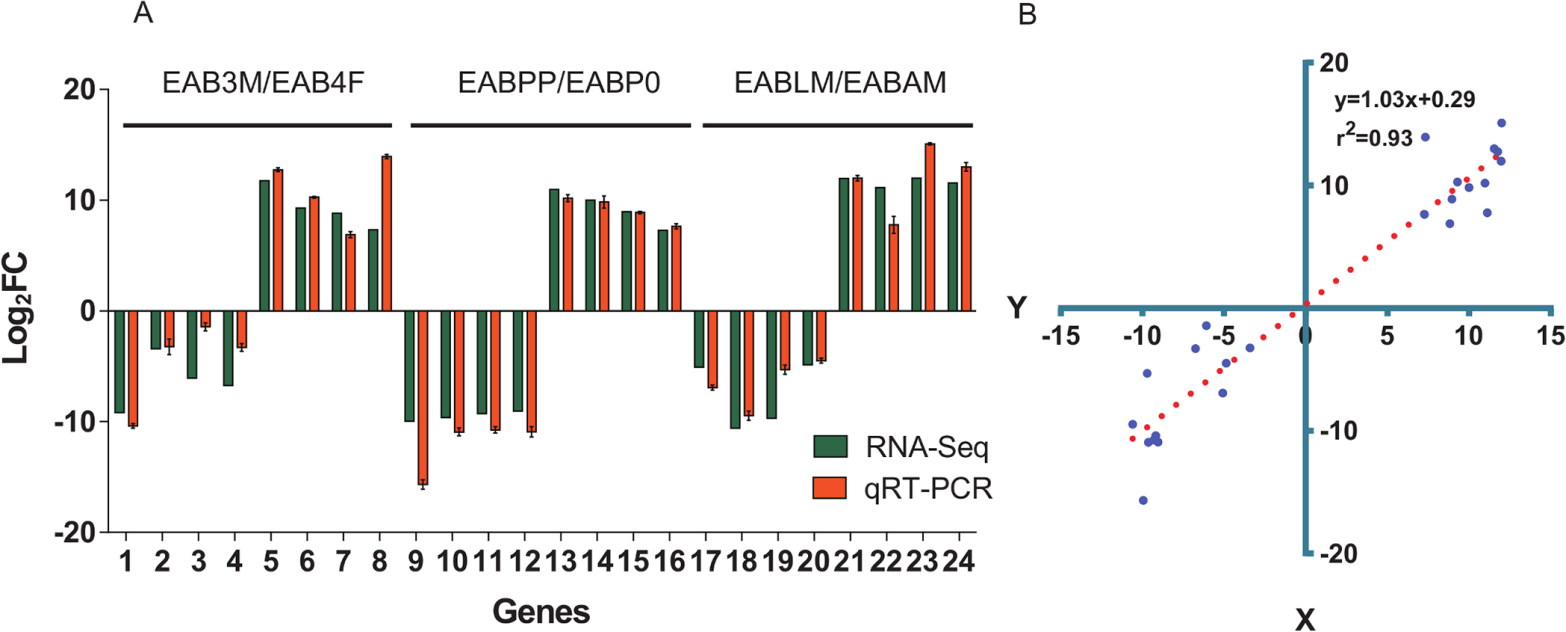
Correlation between the gene expression ratios obtained from RNA-Seq data and qRT-PCR. (A) Expression ratios (Log_2_^FC^) obtained by RNA-seq and qRT-PCR. The expression ratio change was calculated by the 2^-ΔΔCT^ method. TEF-1α was used as a reference gene to normalize the qRT-PCR data. Error bars represent the standard error of the mean (n = 4). Investigated genes were listed in S5 Table, including 1, EABT36748; 2, EABT26334; 3, EABT1664; 4, EABT37717; 5, EABT755; 6, EABT22472; 7, EABT11324; 8, EABT14053; 9, EABT30570; 10,EABT27511; 11, EABT36884; 12, EABT35689; 13, EABT19583; 14, EABT23189; 15, EABT23473; 16, EABT4817; 17, EABT14338; 18, EABT16135; 19, EABT21639; 20, EABT4315; 21, EABT37729; 22, EABT33854; 23, EABT7214 and 24, EABT36743. (B) Lineage analysis between RNA-Seq and qRT-PCR. The Log_2_^FC^ obtained by qRT-PCR (x-axis) are plotted against the Log_2_^FC^ obtained by RNA-Seq (y-axis).

In order to validate the RNA-seq expression analysis, we selected 24 differentially-expressed unigenes and verified their expression between different developmental stages and tissues by qRT-PCR. As shown in Fig 7A and S5 Table, fold-changes obtained by qRT-PCR were compared with those from the RNA-Seq expression analysis. A high correlation coefficient of R = 0.96 was observed between qRT-PCR and RNA-Seq expression data. Linear regression analysis of the correlation (Fig 7B) shows an R^2^ (goodness of fit) value of 0.93, with a corresponding slope of 1.03, suggesting a strong positive correlation between qRT-PCR and RNA-Seq data. These results confirm that fold-changes by qRT-PCR were consistent with the fold changes obtained by RNA-Seq.

### Identification of RNAi related genes

The core RNAi pathway-related proteins were identified from the assembled unigenes by using homology searches. As shown in Table 3 and S2 Text, 11 unigenes implicated in the following process were identified: dsRNA uptake and spread, dsRNA cleavage, endonuclease activity, dsRNA binding and degradation. The uptake and spreading of dsRNA is an important process related to system RNAi. It has been suggested that sid-1 is a core gene that was required for system RNAi in *Caenorhabditis elegans*. By using sid-1 as a query sequence to search against the EAB unigene dataset, we identified two sid-1 homologs (EABsid-1a and EABsid-1b) from EAB. Phylogenetic analysis showed that EABsid-1a and EABsid-1b display close relationships with Tcsid-1a and Tcsid-1c from *T*. *castaneum* (S3 Fig). The presence of sid-1 homologs in EAB suggests that dsRNA uptake mechanisms are similar in EAB and *T*. *castaneum*. For the dsRNA cleaving process, two members of the Dicer gene family were identified in EAB. Dicer, which contains two RNase III-like domains, plays an important role in cleaving long dsRNA molecules into small RNAs (siRNAs). Another enzyme that participates in the cleaving process is Drosha, which is implicated in the process of cleaving long RNA primary transcript (pri-miRNA) into a 70 base pairs pre-miRNA with stem-loop structure. This transcript was found in EAB and *D*. *melanogaster*, but not in the genomes of *B*. *mori* and *T*. *castaneum*.

**Table 3:** Comparative analysis of the transcripts/genes involved in RNAi.

Gene	EAB	*T*.*castaneum*	*D*.*melanogaster*	*B*.*mori*	*C*.*elegans*	Function
*Sid*	*EABSid-1*,*EABSid-2*	*TcSid-A*, *TcSid-B*, *TcSid-C*	*-*	*Bm_sid-like1*,*Bm_sid-like2*, *Bm_sid-like2*	*Ce_Sid-1*, *Ce_Sid-2*	dsRNA uptaking and spreading through the tissues
*Dicer*	*EAB_Dcr-1*, *EAB_Dcr-2*, *EAB_Drosha*	*Tc_Dcr-1*, *Tc_Dcr-2*	*Dr_Dicer-1*,*Dr_Dicer-2*,*Dr_Drosha*	*Bm_Dicer-1*, *Bm_Dicer-2*	*Ce_Dcr_1*	Cleavage of dsRNA
*R2D2*	*EAB_R2D2*	*Tc_R2D2Tc_C3PO*	*Dr_R2D2*	*Bm_R2D2*	*-*	dsRNA Binding
*Rde-4*	*-*	*-*	*-*	*-*	*Ce_Rde-4*	dsRNA Binding
*Loquacious*	*EAB_loqs*	*Tc-Loqs*	*Dr_Loqs*	*Bm_Loqs*	*-*	dsRNA Binding
*Pasha*	*EAB_Pasha*	*Tc_Pasha*	*Dr_Pasha*	*Bm_Pasha*	*Ce_pash-1*	dsRNA Binding
Ago	*EAB_ago1*, *EAB_ago2*	*Tc_ago1*,*Tc_ago2*	*Dr_ago1*, *Dr_ago2*, *Dr_ago3*	*Bm_ago1*, *Bm_ago2*, *Bm_ago3*		Endonuclease Activity
PIWI	*-*	*-*	*-*	*-*	*Ce_PIWI*	Endonuclease Activity
RdRP	*-*	*-*	*-*	*-*	*Ego-1*, *RRF-1*, *RRF-3*	Amplification
Eri-1 exonuclearse	*-*	*-*	*-*	*-*	*Eri-1*	Degradation
	*EAB_Snp*	*Tc_Snp*	*Dr_Snp*	*-*	*Snp*	Degradation
	*-*	*-*	*-*	*-*	*Eri-3*	Degradation

After the dsRNA cleaving process, siRNA is loaded onto the RNA-induced silencing complexes (RISC), and proceeds to recognize mRNAs targeted for degradation. We observed that the dsRNA binding motif (dsRBM) in the proteins involved in the loading of siRNA into RISC were conserved among all examined insect species including EAB. The R2D2, Loquacious and Pasha proteins are present in the EAB with a 1:1:1 orthologous relationship with *D*. *melanogaster* and *B*. *mori* (Table 3). The Argonaute gene family is thought to be the main component of silencing complexes and can mediate target recognition and silencing. Two members of the Argonaute family, EABago1 and EABago2 were identified from EAB. These genes were found in *D*. *melanogaster*, *B*. *mori* and *T*. *castaneum*, but not in *C*. *elegans*. RdRP was thought to be responsible for RNAi signal amplification in plants and *C*. *elegans*, however, it could be found in neither EAB nor other insects.

## Discussion

Using high-throughput deep RNA-sequencing technology, we sequenced EAB transcriptomes from larvae, pre-pupae, pupae and adults, as well as midguts from larvae and adults. Due to the lack of genome sequence for EAB, we adopted a *de novo* assembly strategy to construct the reference transcriptome. RNA-Seq effectively increased the depth of sequencing compared to the previous sequence data from Roche 454 sequencing [17, 42]. This made it possible to cover most of the EAB transcripts in the examined samples, and was especially effective for transcripts expressed at low levels. In previous studies, the average size for midgut and fat body transcripts was only 259 bp and 688 bp, respectively [17, 42]. In this study, the N50 of the unigene dataset is 2.5 kb and the average sequence length is 1.2 kb. The transcriptome assembly obtained in this study has been dramatically improved.

In this study, a large number of tissue- or stage- specific unigenes were identified. These results were validated by qRT-PCR experiments (Fig 7 and S5 Table). We observed 290 transcripts were differentially expressed between the third instar molting larvae and fourth instar feeding larvae, while 3,911 unigenes were differentially expressed during the transition from pre-pupae to pupae. More unigenes show variations in expression levels during metamorphosis than during molting. During molting, the larvae exchange their cuticle for a new flexible one and stop feeding; during metamorphosis, some larval tissues and organs break down and are replaced or are extensively remodeled. We also observed 1,167 unigenes differentially expressed between larval and adult midguts. Larval and adult midguts digest different plant materials. One gene with high expression levels attracted our attention; the endogenous laccase was expressed at a 22-fold higher level in adult midguts than in larval midguts. An endogenous laccase that degrades lignin alkali and lignin phenolics was recently characterized in termites [43]. The high expression of laccase in adult midguts may contribute to the degradation of lignin within leaves. During the lignin degradation process, phenylpropanoids which are often toxic to insects, are released and can provide protection to the plant against insects and pathogens [44].

RNAi is a gene-silencing technique that uses double-stranded RNA (dsRNA) to inhibit homologous gene expression at the RNA level. This technology has been widely used in developing RNAi-based pesticides or transgenic plants producing dsRNAs directed against genes of target pests [14, 45, 46]. We found that our EAB transcriptome assembly had the closest relationship with *T*. *castaneum*. This species is known to be responsive to RNAi [32, 47–49]. We found that the core RNAi components related to dsRNA uptake, binding, cleavage, and endonuclease activity of the RNAi pathway were well conserved in EAB (Table 3). Recently, Zhao et al (2015) identified three RNAi pathway core component genes *Dicer-2*, *Argnaute-2* and *R2D2* from the EAB genome sequence and demonstrated that introducing an EAB gene dsRNA corresponding to a β-fructofuranosidase-encoding gene *AplaScrB-2* into adults down regulated the expression of target gene [50].

The differentially expressed genes identified in this study will help better understand the molting and metamorphosis processes happening not only in EAB, but also in other Buprestidae beetles. This knowledge paves the way for developing improved strategies in the management of this important family of insects. For instance, insect molting and metamorphosis can be targeted by organic compounds such as the diacylhydrazines (DAHs). DAHs are molecules that interfere in an agonistic fashion with the natural hormone ecdysone, leading to ecdysone receptor activation and precocious molting [51]. Halofenozide is one such DAH that displays good activity against Coleoptera, but the molecular basis of its higher activity compared to other available DAHs (e.g. tebufenozide, methoxyfenozide) is not entirely understood. An approach comparing the transcriptomic signature of halofenozide treatment with the natural molting process would be worthy of further investigation, to determine if halofenozide would be of value in EAB control. We provide here an exhaustive catalog of genes to support further work on the ecdysone- or DAH-triggered gene expression in EAB.

Our EAB transcriptome assembly also provides a wide range of sequences for the functional screening of genes by RNAi. RNAi may provide an avenue to target molting and metamorphosis processes in EAB. Important work in *T*. *castaneum* has revealed that several genes encoding cuticular proteins, chitin metabolism enzymes or enzymes involved in melanization can be knocked down and lead to lethal phenotypes at the larval-pupal and/or pupal-adult molts [52–54]. Several genes belonging to these Gene Ontology functional classes have been discovered in our EAB transcriptomic data, and could systematically be investigated for their insecticidal potential by knockdown via double stranded RNA treatment.

## Conclusions

This work presents the transcriptome assembly of EAB, assembled from RNA-Seq data including four different developmental stages and midguts from larval and adult stages. It appears to broadly represent the transcripts of the EAB life cycle except for the embryo stage. Based on the comparison of the transcriptome assembly with 13 other insect species, EAB exhibits a close relationship with *T*. *castaneum*. There are a few hundred transcripts predicted to be Coleoptera-specific or EAB species-specific. Large numbers of transcripts were up or down regulated during EAB molting and metamorphosis or expressed differentially between larval and adult midguts. The RNAi-related proteins were also identified indicating the potential of system RNAi to exist in this coleopteran forestry pest. This study provides the most comprehensive transcriptome of EAB to date and will benefit further studies of EAB and other related pest insects.

## Supporting Information

S1 Fig Frequency distribution of the number of mapped reads per reference transcript. Filtered reads from all the examined samples were combined together to map against the unigene dataset.
(PDF)

S2 Fig Species distribution by comparison Unigenes with NR database. The Unigenes were used to BLAST against nr database by BLASTX. The species from top hits were used in statistical analysis. Only the top 10 species are shown.
(PDF)

S3 Fig Phylogenetic tree analysis of *sid* gene. Phylogenetic analyses were inferred using the neighbor-joining algorithm and Poisson model in MEGA software. Bootstrap values (%) for 500 replicates are indicated at the nodes.
(PDF)

S1 Table Transcript abundance among examined samples. (PDF)

S2 Table Differentially expressed transcripts detected between molting (EAB3M) and feeding larvae (EAB4F). (PDF)

S3 Table Differentially expressed transcripts detected between pre-pupae (EABPP) and pupae (EABP0). (PDF)

S4 Table Differentially expressed transcripts detected between larval (EABLM) and adult midguts (EABAM). (PDF)

S5 Table Primers used for validation of RNA-seq data by qRT-PCR. (DOCX)

S1 Text Unigene sequences in FASTA format. (ZIP)

S2 Text Genes possibly involved in RNAi pathway. (TXT)
