# Supplementary figures and images for: Transcriptome Analysis of the Emerald Ash Borer (EAB), Agrilus planipennis: De Novo Assembly, Functional Annotation and Comparative Analysis
Source: PLoS One. 2015 Aug 5;10(8):e0134824. doi: 10.1371/journal.pone.0134824 (PMC4526369; doi:10.1371/journal.pone.0134824)

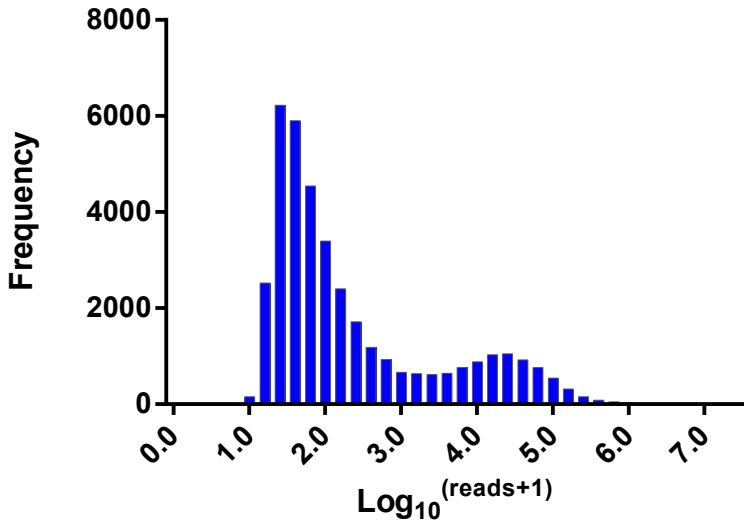

Supplement: S1 Fig — Filtered reads from all the examined samples were combined together to map against the unigene dataset. (PDF) [file pone.0134824.s001.pdf]

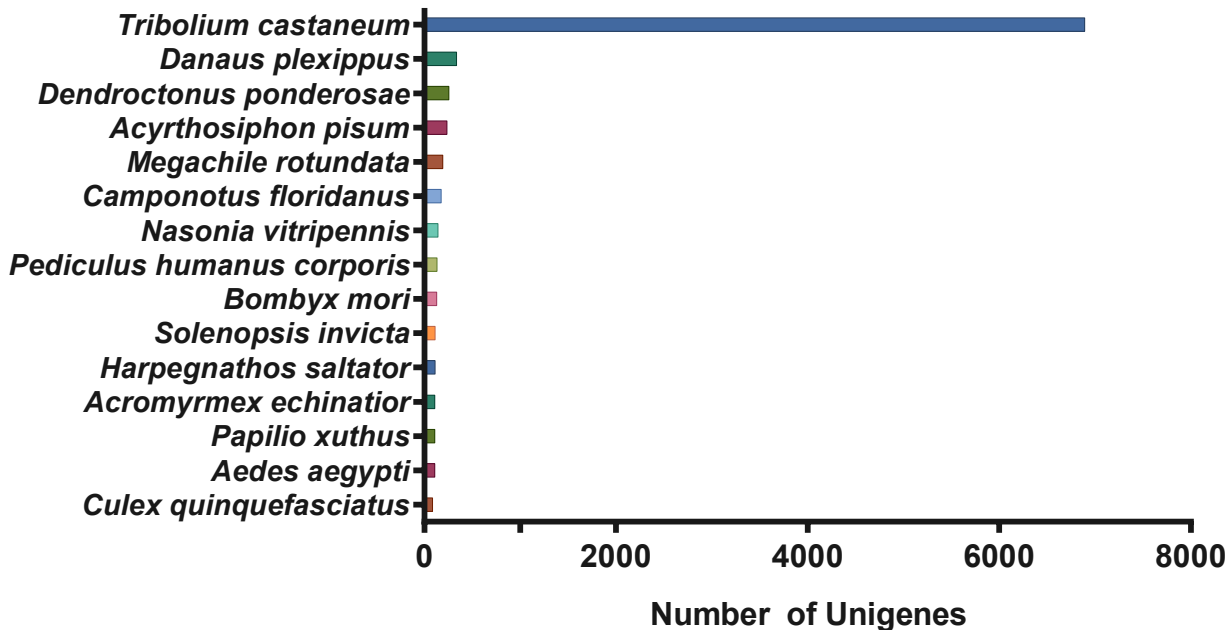

Supplement: S2 Fig — The Unigenes were used to BLAST against nr database by BLASTX. The species from top hits were used in statistical analysis. Only the top 10 species are shown. (PDF) [file pone.0134824.s002.pdf]

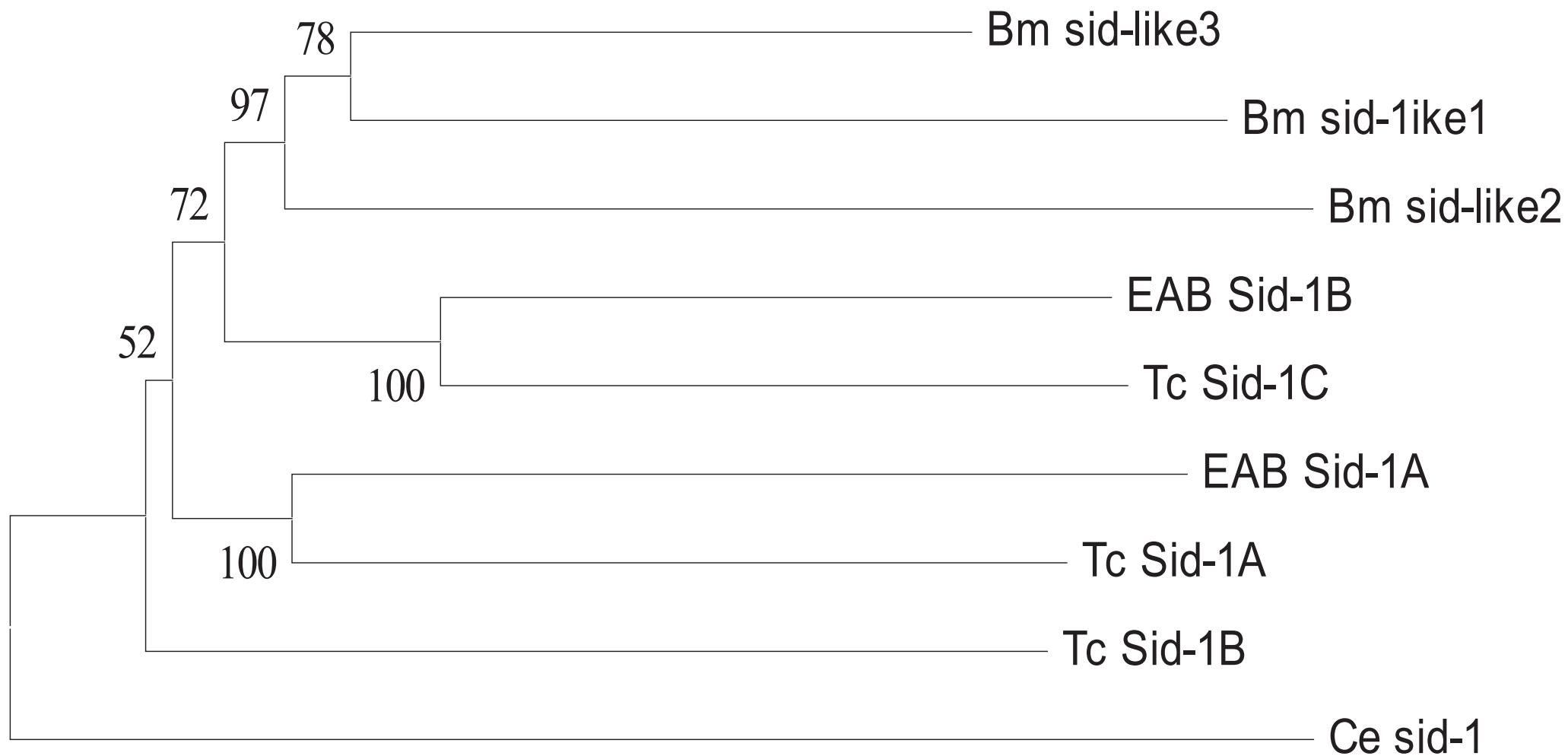

50

Supplement: S3 Fig — Phylogenetic analyses were inferred using the neighbor-joining algorithm and Poisson model in MEGA software. Bootstrap values (%) for 500 replicates are indicated at the nodes. (PDF) [file pone.0134824.s003.pdf]
