# Supplementary material for: Transcriptome Analysis of the Emerald Ash Borer (EAB), Agrilus planipennis: De Novo Assembly, Functional Annotation and Comparative Analysis
Source: PLoS One. 2015 Aug 5;10(8):e0134824. doi: 10.1371/journal.pone.0134824 (PMC4526369; doi:10.1371/journal.pone.0134824)
Supplement: S1 Table — (PDF) [file pone.0134824.s004.pdf]

	EAB3M	EAB4F	EABPP	EABP0	EABA	EABLM	EABAM
EABT1	1	0	20	2	1	1	0
EABT10	1	7	8	16	1	1	1
EABT100	0	0	66	0	0	0	0
EABT1000	0	0	6	0	0	0	0
EABT10000	0	0	7	9	0	0	0
EABT10001	5	7	2	1	2	1	4
EABT10002	247.57	330	518	644.18	478.76	318.86	275.38
EABT10003	5	15	9	11.44	2	2	2
EABT10004	1	7	8	13	10	3	10
EABT10005	7195.21	1896	4075.92	2431.09	3297.94	1172.84	2092.32
EABT10006	0	0	4	1	0	0	0
EABT10007	1231.8	684.99	1401	4078.54	699	703	786.92
EABT10008	0	2	14	1	0	0	2
EABT10009	0	0	10	1	0	0	0
EABT1001	497.93	725.67	717.82	996.43	679.83	491.45	387.12
EABT10010	0	0	7	2	0	1	0
EABT10011	50	69	53	17	23	9	18
EABT10012	0	0	6	1	0	0	0
EABT10013	791.99	1455.92	1612.54	3370.07	1414.35	813.02	772.87
EABT10014	3	7	12	4	3	4	4
EABT10015	1	2	17	32	4	6	6
EABT10016	0	4	0	3	2	0	1
EABT10017	0	1	7.13	7	1	0	0
EABT10018	0	4	6	2	0	1	0
EABT10019	5	36	194	111	112	14	56
EABT1002	2	1	3	5	3	0	0
EABT10020	179137	216494	155129	112888	104410	173233	72049.5
EABT10021	0	5	9	0	0	0	0
EABT10022	10	70	65	266	151	20	0
EABT10023	1	3	0	1	0	0	0
EABT10024	1362.93	60	10	22	454	134	98
EABT10025	1	4	8	3	1	28	9
EABT10026	0	2	42	3	2	2	0
EABT10027	0	0	0	38	1	0	1
EABT10028	2	1	6	0	1	3	1
EABT10029	3	9	23	7	4	0	2
EABT1003	14	15	0	0	0	0	0
EABT10030	28	8	22.98	36	288	8	9
EABT10031	1	2	9	0	3	0	1
EABT10032	2	8	3	3	3	0	4
EABT10033	117	124	199.32	251.5	319.86	56	47.99
EABT10034	1	3	44	8	1	3	5
EABT10035	4	24	8	25	15	4	4
EABT10036	1	3	3	2	1	0	2
EABT10037	1	1	9	21	10	1	0
EABT10038	3	6	3	7	0	1	0
EABT10039	0	1	11	1	0	0	0
EABT1004	0	2	2	6	1	0	0
EABT10040	0	2	2	1	0	0	0

EABT10041	65	138.99	653	140	40	74	66
EABT10042	481.93	1579	2538	5545.47	1837.96	214	448.9
EABT10043	2199.49	3556.07	2063.05	4769.75	3419.58	2114.21	2743.01
EABT10044	254307	62459.2	572.43	5	60202.7	164895	413886
EABT10045	0	2	6	0	0	0	0
EABT10046	0	2	4	0	2	2	2
EABT10047	5	1	1	17	2	0	0
EABT10048	1	0	5	0	0	0	2
EABT10049	2530	3796.66	5676.13	6118.99	2358	3029.93	3033
EABT1005	1	1	10	1	0	0	0
EABT10050	0	5	18	6	0	2	0
EABT10051	6	3	2	5	3	1	0
EABT10052	714.73	1046	1267.22	1398.89	822	724	721
EABT10053	1	2	5	0	0	0	0
EABT10054	0	2	4	2	0	1	0
EABT10055	78	147	133	1697.02	880.1	56	31
EABT10056	313.5	179	29	1	32	36	75
EABT10057	318.78	897.03	421	446.09	422	182	301.08
EABT10058	3	0	3	0	0	3	0
EABT10059	0	0	1	0	27	1	0
EABT1006	4783.71	12747.9	9814.19	494.56	2045.59	1049.87	5860.73
EABT10060	17	4	10	6	2	46	19
EABT10061	2	1	4	4	0	0	0
EABT10062	10	10	10.51	24	16	5	18.33
EABT10063	0	0	1	4	2	0	0
EABT10064	0	0	6	1	0	0	0
EABT10065	2059.45	2213	1157.78	824.68	7084.01	1806.94	1571.09
EABT10066	0	0	31	1	0	0	0
EABT10067	1	1	0	0	0	0	0
EABT10068	0	0	12	2	0	0	0
EABT10069	1	4	20	2	0	0	0
EABT1007	1	2	0	1	2	0	0
EABT10070	30	59	71	85	77	14	20
EABT10071	3762.3	4987.81	3110.1	7371.81	2986.35	3485.9	2795.49
EABT10072	1	11	11	3	2	0	2
EABT10073	2	7	11	1	1	0	0
EABT10074	0	0	10	2	1	1	0
EABT10075	1	2	79	0	0	0	2
EABT10076	1	3	11	7	2	0	1
EABT10077	146	574.03	527.99	2493.75	615.06	28	164
EABT10078	1	2	7.11	3	0	7	20
EABT10079	0	1	7	0	0	0	0
EABT1008	0	0	5	0	0	0	0
EABT10080	0	1	1	0	0	0	0
EABT10081	220.01	392.86	384.41	369	237.25	233.05	261.73
EABT10082	6	18	96	14	1	3	3
EABT10083	73	15	168	114	112	5	0
EABT10084	6	6	48	9	1	1	0
EABT10085	3	15	9	2	0	1	1
EABT10086	0	0	0	1	0	0	1
EABT10087	0	1	3	0	6	0	7

EABT10088	0	0	7	1	0	0	0
EABT10089	1	0	30	5	1	0	0
EABT1009	7	9	88	3	0	21	3.99
EABT10090	19	56	65	81	10	15	8
EABT10091	0	3	4	0	0	0	0
EABT10092	1	8	36	1	4	1	0
EABT10093	11	18	177	3	2	5	3
EABT10094	1	0	4	0	1	0	0
EABT10095	0	5	2	0	1	0	1
EABT10096	21	1	0	0	0	17	0
EABT10097	11	38	47	10479	739.02	4	3757.01
EABT10098	1	3	3	0	0	0	0
EABT10099	0	3	3	6	2	1	1
EABT101	1	5	0	24	2	0	0
EABT1010	3	3	62.88	1	0	0	0
EABT10100	0	0	1	0	2	0	1
EABT10101	4	4	2	2	0	4	0
EABT10102	0	4	6	6	0	0	0
EABT10103	3	1	51.39	37	0	1	0
EABT10104	1	0	5	0	1	0	0
EABT10105	0	1	5	3	1	2	0
EABT10106	0	1	6	10	2	2	0
EABT10107	2	1	12	3	1	0	0
EABT10108	1	1	27	2	0	0	0
EABT10109	0	0	1	3	6	0	2
EABT1011	0	1	3	0	1	1	3
EABT10110	0	1	1	0	0	0	0
EABT10111	0	0	1	0	2	4	3
EABT10112	4	4	32	5	3	1	0
EABT10113	557.13	772.2	389.94	564.35	477.75	205.61	313.96
EABT10114	43	101.01	40	61	65	24	68
EABT10115	25	33	5	6	11	27	25.39
EABT10116	0	1	5	3	12	0	2
EABT10117	1	0	13	1	0	1	0
EABT10118	2	0	4	6	4	0	0
EABT10119	9	14	33	6	10	0	1
EABT1012	11	40	37	24	24	6	14
EABT10120	0	0	4	0	0	0	0
EABT10121	2	4	0	10	20	2	0
EABT10122	0	0	3	3	0	0	0
EABT10123	1	0	6	8	3	1	3
EABT10124	4	3	30	1	1	3	4
EABT10125	1	3	1	3	2	1	0
EABT10126	0	2	12	6	0	0	1
EABT10127	4	13	24	16	4	2	1
EABT10128	688.01	952.03	1046.84	1027.91	710	498	650
EABT10129	2	1	9	0	6	0	0
EABT1013	0	0	9	0	0	0	0
EABT10130	0	4	4	11	0	1	0
EABT10131	0	0	3	3	0	0	1
EABT10132	2266.1	3650.86	3910.02	9867.33	3821.04	3290.97	2975.18

EABT10133	1	11	4	7	0	0	8
EABT10134	0	0	3	0	1	2	0
EABT10135	2	0	1	2	1	0	5
EABT10136	0	2	25	1	0	2	1
EABT10137	0	4	4	4	2	0	0
EABT10138	242	337	79	121	159	59	493
EABT10139	1	4	17	0	0	2	0
EABT1014	6	31	299	147.06	161	11	1
EABT10140	0	1	15	0	0	0	0
EABT10141	1	3	9	5	3	0	1
EABT10142	1	0	4	0	6	0	5
EABT10143	0	0	0	0	0	9	0
EABT10144	9	6	25	6	6	1	15
EABT10145	13	10	14	24	9	3	68
EABT10146	1	0	3	0	1	2	0
EABT10147	4	5	44	7	0	3	4
EABT10148	1	3	4	0	0	0	0
EABT10149	345.25	713.23	2493.32	8145.81	17	5	2
EABT1015	5	4	30	12	5	4	2
EABT10150	1	0	5	0	0	1	0
EABT10151	298.05	580.82	831.94	1095.56	759.07	320	486.02
EABT10152	64	200.09	320	323.05	86.81	77.01	70
EABT10153	125	225	61	157.99	9	344	124
EABT10154	2701.46	2882	17	3	1171.99	3513.99	5367
EABT10155	26	36	33	2	9	50	8
EABT10156	14	191	23	52.99	8	18	17
EABT10157	5	3	14	3	2	0	2
EABT10158	2845.53	2433.1	1293.65	1892.01	2290.8	1271	989.61
EABT10159	0	3	0	1	1	0	0
EABT1016	68	92	24	152	69	73	302
EABT10160	10	18	6	15	2	5	6
EABT10161	0	0	1	5	0	0	1
EABT10162	3032.92	3413.02	3247.71	2463.81	1450.02	637.97	846.06
EABT10163	0	0	0	0	0	0	0
EABT10164	4	4	1	0	0	1	4
EABT10165	21	49	16	243.99	156.94	17	7
EABT10166	0	1	0	2	2	0	0
EABT10167	0	3	5	0	0	0	0
EABT10168	532.98	928.65	963.71	1326.35	680.41	636.89	525.59
EABT10169	924	2518.97	3042.78	2070.93	1232.59	873.04	888
EABT1017	2	5	67	4	0	2	0
EABT10170	31	50	0	0	3	61	45
EABT10171	21	61	69	121	80	6	14
EABT10172	65	37	26	1	2	0	1
EABT10173	1	4	8	7	0	0	0
EABT10174	0	1	4	0	0	0	0
EABT10175	84	61	0	0	0	21	75
EABT10176	1	4	2	2	5	0	0
EABT10177	0	1	4	0	1	0	0
EABT10178	708.99	1832.25	507	2192.85	666	1321	399
EABT10179	110	253.77	38	29	44	38	15

EABT1018	4	1	6.97	0	0	2	0
EABT10180	0	2	24	0	0	0	2
EABT10181	11	17.12	13.95	7.98	9	12	70.01
EABT10182	1	4	2	3	3	1	3
EABT10183	932.28	1392.16	2045.94	1787.78	1533.7	1291.98	974.62
EABT10184	5	1	4	1	1	0	0
EABT10185	0	0	10	1	0	0	2
EABT10186	0	0	0	0	6	0	0
EABT10187	0	5	1	7	2	2	1
EABT10188	2226.79	3821.87	6087.74	6480.22	3685.52	2203.69	2073.77
EABT10189	19	30.06	39.9	38	21	9	12
EABT1019	40	127	67	57	10.56	11	18
EABT10190	5	1	4	3	2	0	1
EABT10191	0	1	8	0	42	0	0
EABT10192	0	1	3	1	0	0	0
EABT10193	0	1	23	0	0	0	0
EABT10194	0	0	1	6	2	1	0
EABT10195	18	43	1	5	1	4	0
EABT10196	530.15	919.64	1155.68	2026.04	659.24	358.63	339.35
EABT10197	6894.85	9000.08	6160.14	8742.9	20898.9	3588.21	4242
EABT10198	0	0	1	4	0	0	0
EABT10199	0	3	0	0	0	0	0
EABT102	0	2.15	1	5	1	0	0
EABT1020	5	8	78	3	3	14	7
EABT10200	26	46	34	64	30	8	22
EABT10201	0	1	6	0	0	0	0
EABT10202	997.12	776.09	25	461.76	115.03	717.9	272.98
EABT10203	0	1	0	4	8	0	8
EABT10204	2357.93	3553.67	3520.23	5257.13	1451	1568.44	1404.01
EABT10205	0	3	3.98	10	0	0	0
EABT10206	29	33	124	20942.6	25	12	14
EABT10207	4246.67	5026.84	1756	5046.86	2144.21	2511.16	1998.17
EABT10208	2	1	4	0	3	0	1
EABT10209	1	5	1	1	2	6	24
EABT1021	0	0	3	1	9	0	0
EABT10210	4	7	4	0	0	2	3
EABT10211	4	13	74	16	5	15	17
EABT10212	0	1	4	61	883.01	1	0
EABT10213	0	3	16	0	2	3	1
EABT10214	1005.06	1862.31	1977.95	3372.1	1861.05	904.93	1585.42
EABT10215	1364.17	3795.6	1362.66	5051.98	1004.47	963.7	976.65
EABT10216	5	11	17	33	7	6	6
EABT10217	392.53	566.22	333.96	772.22	330.47	197.9	204.05
EABT10218	229.99	492.52	777.47	496	148.56	136.97	81
EABT10219	16	13	95	33	10	3	6
EABT1022	2	10	34	6	1	2	1
EABT10220	30	30.34	36	31	27	2	26
EABT10221	480.32	797.17	439	1408.23	720.53	343.3	594.35
EABT10222	1	9	10	3	0	1	0
EABT10223	15	5	16	1	0	2	1
EABT10224	0	0	3	1	0	0	1

EABT10225	2	2	7	3	0	0	0
EABT10226	109	558	871.33	7	57	4	0
EABT10227	0	7	22	9	33	6	3
EABT10228	1	0	4	4	10	3	12
EABT10229	5	1	4	5	1	0	0
EABT1023	1	4	4	15	3	1	1
EABT10230	1	1	7	1	0	0	0
EABT10231	0	0	3	1	1	0	1
EABT10232	0	5	3	3	0	0	1
EABT10233	8	16	3	1	2	25	54
EABT10234	1	0	1	1	1	14	0
EABT10235	12	18	43	6	3	5	4
EABT10236	0	0	3	3	0	0	0
EABT10237	0	0	1	0	12	2	5
EABT10238	2	2	15	4	0	2	1
EABT10239	4	6	18	1	0	0	0
EABT1024	0	0	8	44	0	0	0
EABT10240	0	5	4	3	0	0	0
EABT10241	0	1	6	1	0	0	1
EABT10242	1	6	3	1	1	0	0
EABT10243	125.58	277.6	289.23	385.53	241.06	149.75	264.68
EABT10244	20	17	1	0	0	10	0
EABT10245	1	2	25	8	3	0	0
EABT10246	0	1	14	2	0	2	0
EABT10247	0	0	17	1	0	0	0
EABT10248	360	459.81	342	519.17	360	353	356
EABT10249	1	1	0	1	0	1	0
EABT1025	4	1	10	0	0	0	0
EABT10250	2	3	1	3	0	0	2
EABT10251	0	2	3	2	1	0	2
EABT10252	915.92	1862.13	1817.13	6434.92	1130.08	738.78	595
EABT10253	37.08	46	31.99	168	30	80	49.01
EABT10254	824.32	1827.99	677.32	323.74	828	2161.75	2446.93
EABT10255	8	12	1	1	0	17	1
EABT10256	2	1	26	11	0	0	2
EABT10257	0	0	13.99	3.93	0	0	0
EABT10258	1	1	7	7	0	0	0
EABT10259	0	1	4	0	0	1	1
EABT1026	0	0	4	1	0	0	0
EABT10260	0	1	5	0	0	0	0
EABT10261	1	0	8	0	0	0	0
EABT10262	0	1	7	1	0	0	0
EABT10263	0	0	14	0	0	0	0
EABT10264	26	30	4	22	23	5	2
EABT10265	10173	9247.11	6697.51	6633.88	7261.26	10983.2	8297.12
EABT10266	1	2	3	0	0	0	0
EABT10267	711.63	874.01	812.13	1771.67	882.82	786.02	556.22
EABT10268	2	8	4	2	7	4	86
EABT10269	1509.52	1526.34	407.71	1175.77	1089.31	1750.34	1565.74
EABT1027	0	4	9	6	1	2	4
EABT10270	0	3	11	1	0	5	2

EABT10271	11000.9	6941.79	8509.37	11274.7	13527.9	10570.5	2892.97
EABT10272	4	6	18	4	0	2	5
EABT10273	113	234.56	201	151	94.12	162.01	115.79
EABT10274	0	1	4	0	1	0	1
EABT10275	0	6	23	7	1	3	6
EABT10276	1	2	6	9	1	0	0
EABT10277	0	2	3	5	2	0	1
EABT10278	5	10	4	10	8	6	6
EABT10279	0	1	0	1	0	4	0
EABT1028	7	16	16	40	1	11	10
EABT10280	1	1	10	8	0	0	0
EABT10281	15	20	36	44	7	20	6
EABT10282	66.29	221	113.94	234.28	1	191.74	46
EABT10283	0	3	4	0	0	0	0
EABT10284	4	19	21	6	3	4	14
EABT10285	4	16	20	433	5	0	1
EABT10286	0	0	5	3	0	1	0
EABT10287	0	2	6	1	2	0	2
EABT10288	0	0	4	0	1	0	0
EABT10289	1681	1891.36	1297.47	2817.47	1126	1137.57	768.99
EABT1029	0	2	5	0	0	0	0
EABT10290	5	5	13	16	3	3	6
EABT10291	0	0	0	1	0	0	0
EABT10292	4	7	3	0	0	0	3
EABT10293	6	14	2	0	0	0	0
EABT10294	30	121	38	708.08	20	34	68
EABT10295	5	9	24	3	5	7	3
EABT10296	1	9	32	19	1	10	4
EABT10297	1	0	15	1	0	0	0
EABT10298	0	0	0	0	0	0	0
EABT10299	1	0	16	1	1	0	0
EABT103	0	1	6	1	1	4	0
EABT1030	230	89	114	361	180.6	8	151.05
EABT10300	1	6	5	0	2	0	0
EABT10301	1	3	9	1	0	1	1
EABT10302	13.96	31.03	47	49	7	33	1
EABT10303	815.97	1397.78	1460.19	2385.38	816.95	826.69	641.56
EABT10304	5	5	37	27	1	3	1
EABT10305	302.44	450.47	267.9	893.06	477	126	230.2
EABT10306	24	16	0	0	0	53	0
EABT10307	0	1	8	0	0	0	1
EABT10308	2	4	40	3	2	2	0
EABT10309	0	2	6	2	0	0	0
EABT1031	0	1	8	2	0	0	0
EABT10310	0	1	5	0	1	0	0
EABT10311	1828.51	3178.11	3276.99	6452.12	2213.55	1872.2	1928.28
EABT10312	7781.6	7583.15	4475.17	9905.75	6869.5	10865	7133.87
EABT10313	2	2	0	0	0	0	0
EABT10314	2	0	0	0	0	12	0
EABT10315	2791.99	4376.91	4321.44	4326.82	1533.31	624	688.25
EABT10316	2	8	1	0	0	1	2

EABT10317	3	5	2	2	0	1	1
EABT10318	1	3	4	1	0	2	0
EABT10319	2	2	0	0	0	1	0
EABT1032	1294	1314	478	1147	1201	874.99	716
EABT10320	199.5	577.22	1270.36	7850.27	1014.01	97	212.95
EABT10321	1	0	6	0	0	2	0
EABT10322	0	1	0	2	2	0	0
EABT10323	0	1	2	0	5	0	3
EABT10324	0	0	5	1	0	0	0
EABT10325	1	2	3	2	0	0	1
EABT10326	0	0	10	1	0	1	0
EABT10327	1	0	7	1	0	0	0
EABT10328	0	0	3	1	4	0	0
EABT10329	15	44	40	77	9	13	15
EABT1033	1	1	0	4	1	0	0
EABT10330	0	5	8	10	4	0	0
EABT10331	5	18	19	1	5	4	18
EABT10332	2631.63	4654.27	7987.3	2460.33	2402.05	1827.74	1901.9
EABT10333	0	0	3	6	0	0	2
EABT10334	1	0	3	2	1	1	0
EABT10335	0	3	7	32	2	0	0
EABT10336	19	35	14.66	22	12	31	21
EABT10337	245	357.06	1015.92	2873.61	221	6	30
EABT10338	10	6	3	0	0	1	1
EABT10339	0	0	0	2	0	0	1
EABT1034	0	4	0	0	0	3	0
EABT10340	1271.84	2371.03	1691.86	325	1349	4	64
EABT10341	0	0	5	0	0	1	0
EABT10342	1	6	6	1	1	1	2
EABT10343	17423.8	21715.8	5791.4	8	7927.66	34037.5	61012.5
EABT10344	1	2	4	0	0	0	0
EABT10345	0	1	0	0	3	0	0
EABT10346	2548.06	6170.89	8267.41	2398.42	1287.72	1056.24	2073.05
EABT10347	1	4	1	1	0	0	1
EABT10348	12	16	14	40	10	5	10
EABT10349	52	117	185.25	320	23	49	39
EABT1035	17	11	25	15	23	30	45
EABT10350	2	0	10	4	0	0	0
EABT10351	37.1	43.01	102.24	67	37.1	29.59	18
EABT10352	0	1	6	5	0	0	0
EABT10353	1	7	14	2	0	2	1
EABT10354	43.98	15	9	6	16.99	2	24.84
EABT10355	1	4	6	3	0	0	0
EABT10356	0	1	0	1	4	0	0
EABT10357	0	0	3	6	0	1	1
EABT10358	0	0	0	0	1	0	0
EABT10359	5	3	2	3	0	0	5
EABT1036	3	0	1	2	0	1	0
EABT10360	3	5	32	13	5	6	6
EABT10361	0	2	6	3	0	0	0
EABT10362	11	36	29	32	42	9	10

EABT10363	51	153	21	48	26	5	41
EABT10364	25	10	0	6	6	4	6
EABT10365	3379	1156.92	139	1637	2093.58	2788.25	5142.78
EABT10366	2135.44	2053.88	1415.88	2341.22	1161.3	2485.56	1678.38
EABT10367	285.68	338.4	186.01	630.9	590.1	229	208.94
EABT10368	5	10	11	10	5	4	2
EABT10369	11	17	0	0	0	3	1
EABT1037	15	39	6	118	12	9	36
EABT10370	44	159	187.55	451.26	35	32	40
EABT10371	0	5	11	9	2	0	2
EABT10372	0	1	1	2	0	0	0
EABT10373	1	1	47	0	0	0	0
EABT10374	338	179	74.83	29	61	188	159
EABT10375	0	1	17	2	1	2	2
EABT10376	1	5	0	0	0	0	0
EABT10377	10831.5	7846.15	2296	10498.2	2561.18	7962.95	6739.81
EABT10378	2	6	4	1	9	0	34
EABT10379	3	5	5	2	1	0	0
EABT1038	2285.15	3170.71	3205.5	4367.05	1364.67	1377.67	1494.89
EABT10380	2103.17	3872.65	4146.82	9229.83	1628.02	798.98	716.15
EABT10381	2	2	3	3	0	0	0
EABT10382	3704.5	4931.49	6183.74	7714.12	3712.47	2616.61	2683.13
EABT10383	1	0	5	0	1	1	3
EABT10384	6293.29	7562.27	22631.1	9169.28	3241.98	6745.03	3526.73
EABT10385	417.99	905.75	1350.01	2786.99	533.95	407	380.09
EABT10386	0	1	0	0	0	1	1
EABT10387	7	2	10	13	10	0	9
EABT10388	0	0	10	0	0	0	0
EABT10389	0	4	2	1	0	0	0
EABT1039	0	1	2	4	2	0	0
EABT10390	302.82	462.5	452.93	1009.08	341.45	132	306
EABT10391	6	53	21	14	296.95	3	252
EABT10392	0	1	2	3	0	0	0
EABT10393	0	1	12	1	0	0	0
EABT10394	0	0	0	0	0	0	6
EABT10395	1	5	2	10	0	0	2
EABT10396	2	5	34	3	0	0	2
EABT10397	0	0	13	0	0	0	0
EABT10398	2	0	5	0	3	1	1
EABT10399	3	3	7	9	3	0	0
EABT104	0	0	7	0	0	0	0
EABT1040	2	0	5	0	0	2	0
EABT10400	226	286	157	491	262	187	128.28
EABT10401	0	1	3	1	1	0	0
EABT10402	0	0	2	0	0	0	17
EABT10403	3026.9	3058.98	1890.87	3327.81	1553.64	1766.92	1184.28
EABT10404	613.99	2636.07	1204	651.94	2831	266	1640
EABT10405	6	13	6	5	0	8	1
EABT10406	0	1	0	0	0	15	0
EABT10407	76	46	35	1193	555	4.97	9
EABT10408	12.99	42	24	8	8.88	59	3

EABT10409	378.99	578	2146.2	403.44	2781	618	1753
EABT1041	0	0	0	0	0	0	8
EABT10410	1	0	0	0	0	0	0
EABT10411	5	2	13	11	1	2	1
EABT10412	0	0	8	1	1	1	0
EABT10413	0	1	4	0	6	0	4
EABT10414	5	9	31	24	0	2	3
EABT10415	1	0	1	1	0	0	0
EABT10416	0	0	7	4	1	0	0
EABT10417	101	156	100	213.93	82	36	53
EABT10418	1	0	5	4	0	0	0
EABT10419	1	0	8	2	0	0	1
EABT1042	1449.92	2878.05	3352.67	6185.12	2446.55	1156.96	1353.9
EABT10420	7534.35	6107.63	2996.22	5269.99	3620.64	110	646
EABT10421	0	2	4	12	3	1	0
EABT10422	3	2	1	0	0	0	0
EABT10423	1968.51	2563.17	408	904.11	428.86	2661.99	1532.06
EABT10424	1.99	0	2	0	0	0	1
EABT10425	1	2	8	0	0	0	0
EABT10426	1234.96	2739.96	2598.28	7095.48	4154.82	260	1830.77
EABT10427	374.37	451.95	175.98	556.92	360.25	326.01	212.41
EABT10428	0	1	2	1	0	1	1
EABT10429	1	8	86	13.06	0	1	1
EABT1043	87	138	483	1657.01	232	33	49
EABT10430	9	27	16	13	5	6	9
EABT10431	14	34.99	4	0	2	7	4
EABT10432	0	1	5	1	0	0	1
EABT10433	0	4	9	1	0	2	1
EABT10434	0	4	9	0	0	0	0
EABT10435	558.32	705	693.25	1207.99	479.91	574.39	508.48
EABT10436	0	1	7	0	1	0	0
EABT10437	1	4	11	5	4	2	2
EABT10438	0	3	2	0	0	2	0
EABT10439	5217.36	8729.82	11289.9	12226	4293.63	5469.34	4495.09
EABT1044	10	17	0	0	0	8	0
EABT10440	0	0	4	0	0	1	0
EABT10441	2	1	7	12	5	0	1
EABT10442	8	7	46	3	0	18	1
EABT10443	0	0	3	1	0	0	0
EABT10444	4	8	7	17	0	3	0
EABT10445	0	2	2	0	2	0	1
EABT10446	2	5	7	8	9	6	13
EABT10447	5	27	47	55	10	0	8
EABT10448	564.67	669	626	696	532.81	505.98	358.99
EABT10449	0	1	48	5	1	1	0
EABT1045	1	7.26	3	76	7	0	0
EABT10450	1	4	5	1	1	0	0
EABT10451	3	3	7	3	0	0	2
EABT10452	230	296	162	377	275	188	199
EABT10453	0	0	78	3	0	0	0
EABT10454	0	0	10	0	0	1	0

EABT10455	1	0	4	1	0	0	1
EABT10456	1	0	12	2	0	0	1
EABT10457	1044.85	1458.96	2340.49	1997.73	1097.47	719.48	997
EABT10458	0	3	4	0	0	0	1
EABT10459	1	0	3	0	0	0	0
EABT1046	0	1	6	0	0	0	0
EABT10460	284.92	497.02	2601.47	211	385	15	32
EABT10461	4	10	12	7	1	2	3
EABT10462	13	17	55	147	58	9	14
EABT10463	0	4	0	1	0	1	0
EABT10464	0	2	15	6	0	0	0
EABT10465	814.99	934.48	2782.99	831.75	1189.94	1775.43	1696.8
EABT10466	0	3	16	14	0	0	0
EABT10467	2	2	2	4	2	1	1
EABT10468	5	11	2	1	0	2	13
EABT10469	0	3	6	13	0	1	1
EABT1047	0	0	7	8	0	0	0
EABT10470	4	5	0	0	1	0	0
EABT10471	1	1	5	5	1	2	1
EABT10472	0	0	8	3	0	0	0
EABT10473	969	1328	1301.93	3434.28	1090	872	754
EABT10474	2	2	29	9	9	3	0
EABT10475	10	53	2	4	2	3	0
EABT10476	11	6	5	8	7	3	7
EABT10477	2	2	0	1	8	1	0
EABT10478	6	12	54	127	87	8	8
EABT10479	6	12	23	55	4	0	1
EABT1048	5	4	0	0	0	0	2
EABT10480	5	10	3	1	1	1	0
EABT10481	0	1	8	1	0	0	0
EABT10482	17	23	161	62	10	2	9
EABT10483	2	16	1	0	2	6	6
EABT10484	3	4	2	1	0	6	0
EABT10485	1026.73	1461.12	860.16	2478.82	955.33	481.77	903
EABT10486	0	0	4	6	1	0	1
EABT10487	1	4	1	1	1	1	0
EABT10488	1	4	4	0	1	0	2
EABT10489	3	4	10	27	14	0	6
EABT1049	3	2	9	0	1	1	1
EABT10490	1	8	9	7	4	1	3
EABT10491	5	15	2	1	1	1	2
EABT10492	4	37	11	14	5	32	124
EABT10493	0	1	6	11	0	1	1
EABT10494	2390.51	3793.29	4068.93	8637.97	2836.5	1808.62	1714.98
EABT10495	0	0	1	1	7	0	0
EABT10496	1807.23	2822.53	1811.41	5500.35	1632.87	1248.06	1651.85
EABT10497	0	0	9	0	0	1	0
EABT10498	0	0	9	0	0	0	0
EABT10499	117271	142128	48615.7	113418	87956.5	65422.6	71639.6
EABT105	1	0	3	8	7	1	1
EABT1050	1	0	3	9	0	0	0

EABT10500	1	1	3	0	0	0	0
EABT10501	0	0	9	0	0	0	0
EABT10502	0	1	6	2	0	0	0
EABT10503	4264.38	5097.37	4456.79	3626.25	2580.88	2974.29	4503.93
EABT10504	13	41	21	13	17	1	1
EABT10505	2273.16	4071.65	3610.33	5661.93	2286.47	2469.83	2704.75
EABT10506	110	125	107	166	64	153	123.04
EABT10507	5	8	1	0	0	3	15
EABT10508	0	1	4	15	1	1	0
EABT10509	0	1	16	4	1	2	0
EABT1051	5	6	5	5	0	23	5
EABT10510	2.06	2	4	0	6	6	53.03
EABT10511	8	3	0	2	2	0	0
EABT10512	664.18	1222.64	2578.11	11118.9	1168.01	374	453
EABT10513	8512.19	11070.6	8050.56	5725.37	4444.37	7420.13	7021.24
EABT10514	0	0	8	1	0	0	0
EABT10515	1	1	3	3	0	0	0
EABT10516	582	820	693.88	1523.02	571	316	566.99
EABT10517	0	4	3	4	0	0	2
EABT10518	0	0	14	2	0	2	1
EABT10519	5	6	3	7	22	5	3
EABT1052	7709.84	5318.5	787.02	335	3533.16	785.07	3493.32
EABT10520	0	2	26	0	0	0	0
EABT10521	11	15	38.06	18	4	5	4
EABT10522	7	8	9	12	3	0	2
EABT10523	12	53	24	24	3	0	0
EABT10524	0	0	5	0	0	0	0
EABT10525	1	1	10	15	2	0	2
EABT10526	1665.72	2262.05	2600.84	4962.59	1942.46	1406	1232.05
EABT10527	1	2	0	14	3	1	0
EABT10528	187.79	579.27	286.94	185.1	196	219	103.95
EABT10529	0	4	1	5	1	0	2
EABT1053	0	1	8	1	1	0	0
EABT10530	0	1	3	1	0	0	2
EABT10531	3	3	10	0	0	0	1
EABT10532	0	3	17	3	2	1	1
EABT10533	2	2	15	2	0	2	1
EABT10534	9	11	1	15	7	1	3
EABT10535	6434.8	13097	6918.02	9110.49	20711.9	379	5949.08
EABT10536	2	5	5	0	0	1	0
EABT10537	1	3	5	0	0	0	0
EABT10538	4	16	6	7	4	0	0
EABT10539	3	10	5	17	5	0	9
EABT1054	23	25	19	53	21	0	0
EABT10540	1	1	13	1	0	0	0
EABT10541	2	7	0	5	1	0	3
EABT10542	0	1	1	4	0	1	0
EABT10543	194	269	0	0	53	173	68
EABT10544	1	1	12	1	0	0	0
EABT10545	1	3	2	1	0	1	0
EABT10546	0	3	7	0	1	1	0

EABT10547	7	5	32	14	2	3	8
EABT10548	2	18	29	33	2	1	0
EABT10549	1	1	6	1	0	0	0
EABT1055	0	3	8	2	0	1	0
EABT10550	147	178	39	1825	327	10	90
EABT10551	477.99	883.39	598.56	1191.82	182.74	330.05	555.74
EABT10552	272	358.46	435.79	2165.98	191	15	290
EABT10553	0	0	3	1	0	0	1
EABT10554	2	14	123	11	4	1	1
EABT10555	0	3	3	22	1	1	5
EABT10556	0	0	6	1	0	0	0
EABT10557	5571.49	8818.1	3901.67	6001.47	3686.16	3385.67	3598.27
EABT10558	0	1	7	0	0	0	0
EABT10559	0	6	17	9	0	0	1
EABT1056	47950.3	25174.8	776	709.86	13334.8	18063.1	4085.24
EABT10560	0	7	0	1	0	1	1
EABT10561	2	6	11	9	4	2	2
EABT10562	55292.8	180761	722.14	4	23235.7	3153	79203.1
EABT10563	0	2	2	2	2	0	0
EABT10564	593	522.8	203	135	373.05	675	977.81
EABT10565	1	7	17	20	3	10	1
EABT10566	0	1	4	3	0	0	0
EABT10567	1	0	8	0	0	0	0
EABT10568	3	3	1	0	0	0	2
EABT10569	3	2	11	5	3	0	0
EABT1057	0	3	7	7	0	0	0
EABT10570	1908.11	2697.94	1745.52	3294.61	2029.34	1550.85	1236.89
EABT10571	0	0	1	3	1	0	0
EABT10572	0	0	10	0	0	0	0
EABT10573	4	3	9	9	2	3	3
EABT10574	0	1	15	15	4	0	0
EABT10575	3	1	10	11	29	0	2
EABT10576	1178.45	2566.65	1411.06	633	870.19	122	275
EABT10577	0	0	4	0	0	0	0
EABT10578	0	0	0	0	3	0	1
EABT10579	111497	45203.5	2020.27	1507.74	5536.83	33992.2	23656.3
EABT1058	3	13	14	9	20	0	2
EABT10580	0	1	3	0	1	3	4
EABT10581	0	1	3	3	1	0	3
EABT10582	8	6	0	0	0	4	1
EABT10583	1	3	17	29	1	6	2
EABT10584	53	148	154	43	16	101	17
EABT10585	0	0	4	4	0	0	0
EABT10586	0	1	18	0	0	0	0
EABT10587	2	1	0	0	0	0	0
EABT10588	210	367	229	5	8	5	1
EABT10589	1	3	1	4	0	0	0
EABT1059	20	55	43	3	10	1	4
EABT10590	0	1	0	0	0	1	3
EABT10591	0	0	1	7	0	0	0
EABT10592	1	1	4	4	1	1	0

EABT10593	2013.41	2247.83	1780.35	674.04	3722.96	1571.21	10976.9
EABT10594	1	1	10	0	1	0	0
EABT10595	1	4	8	3	0	1	1
EABT10596	55	59	19	40	133	5	28
EABT10597	0	2	4	6	4	0	0
EABT10598	0	0	6	0	0	0	0
EABT10599	0	2	20	0	1	1	2
EABT106	1	1	24	4	1	0	0
EABT1060	57.99	9.8	1	2	4	515.23	0
EABT10600	681.72	1570.72	1587.01	649.61	890.09	144.84	519.87
EABT10601	127	218	595	568	70	91	34
EABT10602	5287.9	8685.55	5078.86	4638.1	5531.07	5175.91	8969.96
EABT10603	66	319.17	1384.55	26826.3	169	134	256
EABT10604	3	4	5	2	0	0	0
EABT10605	4	2	9	11	1	0	9
EABT10606	3	6	4	12	0	0	4
EABT10607	4	2	9	0	0	0	1
EABT10608	5	8	26	0	7	2	10
EABT10609	44	90	53	368	179	15	13
EABT1061	0	0	16	0	0	0	0
EABT10610	2	2	4	3	0	0	1
EABT10611	5064.35	6813.87	3212.64	8936.09	6196.94	2490.55	4156.59
EABT10612	2	1	17	0	1	1	0
EABT10613	0	1	6	0	0	0	0
EABT10614	0	0	1	2	4	0	0
EABT10615	0	1	6	2	0	0	0
EABT10616	0	0	4	1	0	0	0
EABT10617	0	1	9	2	3	2	0
EABT10618	0	0	6	2	3	0	1
EABT10619	2	2	0	0	0	5	0
EABT1062	7	20	36	22	7	3	5
EABT10620	0	1	0	5	1	0	5
EABT10621	8	16	8	4	2	3	10
EABT10622	2524.21	2399.39	590.86	1302.25	7019.47	1795.3	2028.77
EABT10623	2455.26	3915.11	3502.73	4043.72	3440.96	4221.53	5262.03
EABT10624	62	169	300	378	177	59	50
EABT10625	0	2	4	5	0	0	1
EABT10626	0	0	18	3	1	2	0
EABT10627	2	0	2	2	0	0	0
EABT10628	2	1	2	1	0	0	0
EABT10629	1	1	2	0	3	0	0
EABT1063	0	0	6	2	0	0	0
EABT10630	2	1	4	3	0	0	0
EABT10631	3	14	12	17	1	1	0
EABT10632	3	1	13	3	3	0	4
EABT10633	1	3	7	3	1	3	2
EABT10634	23	43	79	28	7.03	1	2
EABT10635	8365.15	10296.1	7999.23	20950.5	6774.54	6022.03	5119.8
EABT10636	4	5	5	11	2	0	3
EABT10637	0	1	7	1	0	0	0
EABT10638	2	2	3	0	1	0	0

EABT10639	1448.2	3164.68	4908.57	9605.01	2862.86	2472.98	1916.09
EABT1064	0	2	1	2	0	0	2
EABT10640	1	2	3	6	0	0	0
EABT10641	0	1	5	0	0	0	0
EABT10642	0	2	22	4	0	0	1
EABT10643	2	5	3	5	0	2	0
EABT10644	868.04	1196.65	445.96	802.11	946	259	378
EABT10645	2927.41	6561.85	5891.62	4728.96	4333.99	2129.91	2364.99
EABT10646	7	5	2	7	4	11	2
EABT10647	0	2	1	1	7	0	4
EABT10648	3	0	9	1	0	0	0
EABT10649	3086.37	5033.68	4555.27	4390.99	1976.06	2233.39	2589.99
EABT1065	2	2	8	0	1	0	0
EABT10650	0	1	0	5	0	0	0
EABT10651	193	279.99	663.1	3280	676	14	81
EABT10652	0	0	3	1	3	0	0
EABT10653	2	4	12	13	1	1	4
EABT10654	23	61	115	128	20	34	43
EABT10655	1343	2047.83	167.96	675	804.57	1999.05	3809
EABT10656	63.08	2	2	3	9	42	64.8
EABT10657	0	2	9	2	1	0	0
EABT10658	0	1	2	2	2	0	1
EABT10659	3	4	4	63	2	2	5
EABT1066	0	2	3	2	3	0	0
EABT10660	256	360.6	456.89	462	142.04	162	93.99
EABT10661	0	0	1	2	0	2	1
EABT10662	4	32	67.02	10	0	1	1
EABT10663	8045.42	6706.07	2988.24	3045.97	2342.55	7295.84	6088.97
EABT10664	6390.66	5402.42	6032.53	3508.52	3619	6973.24	3219.47
EABT10665	6	4	4	7	16	1	2
EABT10666	0	2	23	2	0	0	0
EABT10667	1777.75	3813.98	7539.51	2949.83	3113.5	1956.43	3404.75
EABT10668	1399.47	1937.01	1627.84	1153.74	979.6	1273.89	1402.18
EABT10669	31	46	28	79	28	4	164
EABT1067	2	3	3	1	0	0	0
EABT10670	4	6	20	1	0	0	1
EABT10671	14	19	70	1	1	2	1
EABT10672	0	1	3	1	0	0	0
EABT10673	1	5	30	148	22	0	0
EABT10674	0	4	17	113	0	1	0
EABT10675	0	2	5	0	0	0	0
EABT10676	0	2	3	4	1	1	0
EABT10677	0	1	7	3	0	0	0
EABT10678	0	4	2	2	1	2	0
EABT10679	1	1	5	2	0	0	0
EABT1068	1	0	9	2	0	0	0
EABT10680	1	5	20	0	0	1	0
EABT10681	30	30	1	3	15	59	131
EABT10682	0	0	4	0	0	0	0
EABT10683	8138.17	19897.6	15137.4	7803.82	7562.13	5196.39	5360.38
EABT10684	1	2	7	6	0	2	3

EABT10685	0	1	6	1	0	0	0
EABT10686	7	17	5	15	6	0	0
EABT10687	0	2	5	0	0	0	1
EABT10688	79	125	458.33	737	526.62	3	218
EABT10689	20016.7	23636	14932.6	18960.7	16770	24339.2	23875
EABT1069	0	19	33	12	13	0	0
EABT10690	0	5	87	1	1	0	1
EABT10691	0	0	5	1	0	4	0
EABT10692	0	1	1	3	1	0	0
EABT10693	0	0	5	1	1	0	0
EABT10694	1	3	47	5	0	1	0
EABT10695	9	4	16	9	4	13	2
EABT10696	48843.9	41610.3	1974.9	4837.73	923.42	8632.26	2257.33
EABT10697	12.04	9	15	44	4	4	12
EABT10698	0	0	2	0	1	1	2.19
EABT10699	0	2	24.01	5	0	3	0
EABT107	0	1	6	1	0	0	0
EABT1070	2	2	10	4	0	0	1
EABT10700	1	1	4	0	1	1	0
EABT10701	0	2	10	0	0	0	0
EABT10702	3	13	83	47	5	0	1
EABT10703	1	7	8	7	0	0	0
EABT10704	0	2	1	5	0	0	3
EABT10705	0	2	7	0	2	0	0
EABT10706	296	622	838.09	1320.92	791.95	364	442.96
EABT10707	0	1	2	4	1	0	0
EABT10708	0	0	4	2	0	0	0
EABT10709	0	0	2	0	0	1	0
EABT1071	0	0	10	0	1	0	0
EABT10710	1289.02	1304	1257.97	1469.86	952	1	113
EABT10711	4	11	9	24	4	5	7
EABT10712	4146	8693.59	9771.57	15329.3	6611.68	3791.82	4466.07
EABT10713	0	1	4	0	2	0	0
EABT10714	0	0	5	3	0	1	0
EABT10715	4	8	4	0	0	0	0
EABT10716	5	12	55	1	1	1	1
EABT10717	1	1	6	0	1	2	0
EABT10718	6	16	105	19	5	3	1
EABT10719	1	9	32	4	1	8	1
EABT1072	1514.59	2374.94	2031.99	5056.41	2339.96	955.89	1358.91
EABT10720	3145	2842	1830.01	3702.05	2008.1	2134.14	1777.34
EABT10721	0	0	1	10	2	0	1
EABT10722	4	6	5	0	1	8	2
EABT10723	936	67	62	116.9	114	205	75
EABT10724	0	1	5	0	0	1	0
EABT10725	16	31	80	73	9	6	10
EABT10726	0	0	17	2	1	0	0
EABT10727	0	0	2	13	2	0	0
EABT10728	0	3	2	1	0	2	1
EABT10729	19.59	404.98	156.51	37.26	22.9	0	3
EABT1073	9	21.01	54	3	0	1	0

EABT10730	1	0	32	1	1	0	0
EABT10731	1	0	12	0	0	0	1
EABT10732	1	3	1	3	2	0	3
EABT10733	5	2	26	2	1	1	2
EABT10734	0	0	5	0	0	0	0
EABT10735	3	4	0	0	0	0	0
EABT10736	1942.01	3249.99	4190.99	2635.93	1439.03	1252	1161.15
EABT10737	1	1	6	0	1	1	0
EABT10738	4	13	32	20	35	7	4
EABT10739	4	5	25	2	3	1	0
EABT1074	0	1	5	0	0	0	0
EABT10740	1963.43	2926.93	4183.61	2771.63	2208.66	1895.96	2412.43
EABT10741	10	16.01	77	2	3	0	0
EABT10742	2	0	3	0	0	1	1
EABT10743	0	4	6	99.35	5	5	7
EABT10744	0	0	0	1	0	0	0
EABT10745	4	10	4	8	0	0	2
EABT10746	8	4	0	6	1	5	8
EABT10747	0	1	4	2	0	0	0
EABT10748	17	22	26	1	8	15	5
EABT10749	7562.32	15434.4	31614.2	24494.1	3050.12	3	342
EABT1075	13	19	34	26	10	8	18
EABT10750	0	0	0	0	2	1	8
EABT10751	1	1	7	0	0	2	0
EABT10752	160	191	142	25	109	120	91
EABT10753	2661.2	3535.61	4750.21	7428.43	3060.9	2509.01	2442.81
EABT10754	853	1124.76	1102.57	1691.08	1003.65	1050	808
EABT10755	126.67	29	40	3	4	49	19
EABT10756	1	7	139	1	2	1	0
EABT10757	7	4	65	7	4	14	14
EABT10758	0	1	10	1	0	0	0
EABT10759	742.53	914.54	43.99	519.13	441.31	1	42.73
EABT1076	11	27	9	6	1	5	10
EABT10760	2	1	5	3	3	0	3
EABT10761	1	0	3	3	0	1	1
EABT10762	55	28	0	0	0	13	3
EABT10763	2	1	3	1	1	1	1
EABT10764	0	2	1	0	0	1	1
EABT10765	0	1	3	0	0	0	0
EABT10766	18	32	27	51	15	9	14
EABT10767	2	2	19	8	3	5	3
EABT10768	0	1	5	2	0	1	2
EABT10769	3	5	3	3	0	0	1
EABT1077	1	2	7	0	0	0	0
EABT10770	2901.28	4185.92	6958.06	1624.38	907.63	202.99	105.14
EABT10771	1	0	3	0	0	0	0
EABT10772	1123.78	1671.55	2130.74	2196.87	1243.9	2159.97	1413.31
EABT10773	0	1	1	3	4	0	0
EABT10774	577.99	757.78	672.01	1561.94	645.26	278.43	476.33
EABT10775	295	970.92	2281.04	194	332	298.19	36.77
EABT10776	0	2	2	1	1	2	0

EABT10777	548.02	1413.33	168	6668.91	1379.17	10	603.06
EABT10778	281.05	497.82	1191.4	1277.53	631.21	405.31	527.24
EABT10779	390	449	479	846	460	365	310
EABT1078	2	2	3	0	1	1	0
EABT10780	6	10	0	0	3	5	12
EABT10781	2	3	24.99	0	2	7	3
EABT10782	0	5	49	2	2	0	1
EABT10783	0	2	4	1	0	0	0
EABT10784	0	1	44	1	0	0	0
EABT10785	0	0	7	0	0	0	0
EABT10786	1	4	21	0	2	0	1
EABT10787	1	2	6	1	0	0	0
EABT10788	3	13	20	2	0	0	0
EABT10789	21	53	16	41	10	6	11
EABT1079	34	29	59	2958.71	125	4	9
EABT10790	1	5	1	4	1	0	0
EABT10791	0	3	1	1	1	0	0
EABT10792	2425.93	3509.17	3078.34	4756.06	3289.09	2435.01	1979.97
EABT10793	0	1	7	0	0	0	0
EABT10794	0	1	22	0	0	0	0
EABT10795	2	0	16	4	0	0	0
EABT10796	4	9	12	14	12	4	2
EABT10797	0	7	1	1	0	1	1
EABT10798	16	14	0	0	0	0	0
EABT10799	4	4	0	0	0	2	7
EABT108	0	1	4	0	1	2	0
EABT1080	0	0	0	1	0	0	1
EABT10800	1	2	10	3	1	5	1
EABT10801	2	1	2	3	3	0	2
EABT10802	5	7	3	4	4	0	0
EABT10803	0	0	3	4	0	0	0
EABT10804	0	2	14	0	1	0	1
EABT10805	0	2	2	12	1	0	1
EABT10806	1228	2773.61	5483	15779.8	7213.62	523.66	2171.84
EABT10807	109.99	333	206	79	109.96	32	90
EABT10808	0	0	9	1	0	0	0
EABT10809	0	0	2	3	0	0	0
EABT1081	0	3	0	0	0	0	0
EABT10810	15	23	64	23	115	2	3
EABT10811	16	5	8	0	0	36	5
EABT10812	0	0	2	2	0	0	0
EABT10813	5	8	12	16	3	6	5
EABT10814	2	0	8	1	0	1	0
EABT10815	3	5	15	3	3	3	2
EABT10816	127	436	310	172	116	4	16
EABT10817	87	573	914	4868.59	700	36	101
EABT10818	0	0	4	16	0	0	0
EABT10819	4	0	0	0	2	0	4
EABT1082	1	2	3	1	0	1	1
EABT10820	694	187	51	52	117	37	52
EABT10821	3087.92	3915.52	6018.57	4714.31	2500.9	2293.96	1680.33

EABT10822	1	1	12	1	1	0	0
EABT10823	8631.09	10790.8	14560.9	18763.2	12834.5	7178.05	5835.28
EABT10824	0	5	3	2	0	0	0
EABT10825	2	2	27	4	4	0	8
EABT10826	1	8	1	1	1	1	3
EABT10827	1477.27	2209.2	2121.53	3864.98	1545.71	894.76	1048.73
EABT10828	1	2	0	3	0	0	0
EABT10829	405	401	824	605.06	86	785.7	831.88
EABT1083	1	0	1	6	0	0	0
EABT10830	3	3	4	2	0	4	0
EABT10831	2	7	55	2	4	1	3
EABT10832	1	1	38	5	70.69	1	11
EABT10833	3	1	2	4	0	1	0
EABT10834	101	194	283.02	344.95	186	50	193
EABT10835	7059.09	13952.7	19715.5	29140.1	8953.82	13671.5	11693.6
EABT10836	0	0	4	4	0	0	1
EABT10837	1906.05	2916.26	2134.91	5045.84	1481.44	1869.91	2085.06
EABT10838	4	3	10	4	1	2	3
EABT10839	3619.74	6184.19	8517.63	4458.83	3422.14	3257.1	4497.48
EABT1084	2	0	11	0	0	0	0
EABT10840	1143.05	1667.77	1391.23	3240.43	1247.99	811.07	1181.23
EABT10841	4242.22	3330.75	1734.45	3918.69	2172.76	5287.67	3606.4
EABT10842	0	4	13	3	0	1	0
EABT10843	0	0	3	1	0	0	0
EABT10844	2082.91	3838.52	3666.5	5709.21	6006.02	1016.89	1380.99
EABT10845	0	6	1	2	0	4	0
EABT10846	0	2	2	0	0	0	0
EABT10847	0	0	0	4	13	0	0
EABT10848	1	1	5	4	706	1	9
EABT10849	0	0	0	0	6	0	2
EABT1085	1	1	21	1	0	0	0
EABT10850	1	1	5	2	0	0	0
EABT10851	3082.8	7274.53	4378.45	4763.69	2687.3	2587.88	3251.69
EABT10852	0	1	2	2	0	0	0
EABT10853	0	3	21	2	0	0	0
EABT10854	2	5	27	0	16	0	2
EABT10855	42	31	14	61	730	4	6
EABT10856	12	7	10	11	8	1	2
EABT10857	3	41	0	2	3	4	14
EABT10858	1	2	17	6	1	1	1
EABT10859	3	4	12	7	1	0	0
EABT1086	2	1	3	0	0	0	0
EABT10860	4	3	0	0	0	0	2
EABT10861	57	143	662	3130.62	41	178.86	7
EABT10862	3139.12	3910.39	3013.24	3833.43	2434.08	2798.22	3883.78
EABT10863	0	2	4	17	7	0	1
EABT10864	7	12	14	7	2	3	10
EABT10865	3	0	0	0	0	20	0
EABT10866	706.8	1118.5	513.98	200	1667.84	405.3	5874.41
EABT10867	6	32	70	36.17	62.01	2	299.69
EABT10868	0	0	2	3	14	1	2

EABT10869	0	4	7	1	0	0	1
EABT1087	1	1	0	1	0	1	0
EABT10870	0	0	69	1	2	1	0
EABT10871	31	82	38	161	32	43	49.01
EABT10872	0	3	3	0	0	0	0
EABT10873	13	26	0	0	0	0	0
EABT10874	353.18	1067.96	2147.23	2991.22	887.51	37	331.18
EABT10875	3	10	9.99	13	3	3	10
EABT10876	1	0	5	7	0	0	0
EABT10877	1	3	3	3	0	3	3
EABT10878	81	167.01	247.43	171.25	125.03	109.3	131.36
EABT10879	0	0	12	0	0	0	0
EABT1088	0	0	0	5	0	2	1
EABT10880	2	2	1	2	0	1	0
EABT10881	0	0	7	2	1	0	0
EABT10882	0	0	2	0	1	0	0
EABT10883	869	1499.93	1679.99	4042.72	1676.96	786.95	880.12
EABT10884	0	0	0	2	1	0	0
EABT10885	0	2	5	3	0	1	0
EABT10886	10	14	29	11	41	28	27
EABT10887	4	5	0	3	3	0	0
EABT10888	43	75	90	28	92	112	47
EABT10889	1	3	1	19	1	2	2
EABT1089	0	0	1	0	0	0	0
EABT10890	2	0	4	1	0	2	0
EABT10891	1	0	0	2	2	0	0
EABT10892	3	0	42	1	3	2	0
EABT10893	8	13	68	11	2	9	2
EABT10894	0	0	29	0	3	1	2
EABT10895	226	387	639.87	1588.95	536	200	204
EABT10896	1286.6	274	2	2	6	1507.74	20
EABT10897	1588.96	2035	2557.49	3358.99	1981.81	1634.17	1535.46
EABT10898	0	1	2	1	1	0	0
EABT10899	0	1	9	16	0	0	0
EABT109	4	1	9	9	2	3	5
EABT1090	0	1	4	0	1	0	0
EABT10900	0	2	30	3	0	0	0
EABT10901	217.02	352	250	685	264	132	124
EABT10902	2	0	9	0	4	1	1
EABT10903	1214.99	2567.26	1371.03	4302.56	750.99	640	819.34
EABT10904	429.93	681.91	450.74	598.75	377.3	305.05	560.08
EABT10905	1677.72	2722.56	2234.1	3275.88	1754.35	1127.8	1567.91
EABT10906	1	3	2	0	0	1	2
EABT10907	549.02	832.05	1403.59	1913.43	922.2	804.98	526.85
EABT10908	4	7	25	9	3	1	4
EABT10909	123.05	144.19	117	97.99	39.47	847.09	255.08
EABT1091	0	0	13	2	0	0	0
EABT10910	9	0	1	0	0	17	1
EABT10911	325	338	453	122	343	234	275
EABT10912	2	6	2	1	1	1	1
EABT10913	0	1	3	0	0	0	0

EABT10914	45	58	5	14	6	30	5
EABT10915	0	4	26	9	1	0	0
EABT10916	2	7	7	1	2	6	9
EABT10917	0	0	3	4	0	0	0
EABT10918	4783.01	6915.38	5639	7965.66	5094.02	2377.23	4591.23
EABT10919	900.7	1461.8	1334.72	1460.08	767.08	1047.06	1058.3
EABT1092	3	12	6	5	2	2	3
EABT10920	14	23	81.05	68	16	13	16
EABT10921	0	0	5	2	0	0	0
EABT10922	1	0	2	10	1	1	4
EABT10923	6	8	3	7	9	7	8
EABT10924	1	3	5	0	0	0	0
EABT10925	1	2	2	1	1	0	0
EABT10926	0	2	1	3	0	0	0
EABT10927	6	90	15	6	1	0	0
EABT10928	86	103	244	389.32	97	22	61.99
EABT10929	2935.51	3061.81	2383.95	2441.84	1678.82	3537.35	2620.82
EABT1093	1	0	4	0	0	1	0
EABT10930	1	1	0	2	3	0	0
EABT10931	3	7	37	3	0	3	1
EABT10932	54.9	12.96	15	17	3	1	0
EABT10933	2	5	152	10	0	5	1
EABT10934	0	2	5	3	1	3	0
EABT10935	488	367	90	266	147.85	331.02	111
EABT10936	0	2	2	7	1	1	2
EABT10937	90	22	42	35	80	62	45
EABT10938	0	0	0	5	1	0	0
EABT10939	9	3	1	0	1	20	0
EABT1094	0	3	11	1	0	1	0
EABT10940	2	0	3	0	3	1	0
EABT10941	3885.79	5946.96	7559.28	9131.02	3761.92	3964.13	4129.06
EABT10942	0	2	26	0	1	0	0
EABT10943	0	0	120	0	0	0	0
EABT10944	2	0	0	0	0	1	2
EABT10945	0	4	27	1	0	0	0
EABT10946	0	3	8	12	1	0	0
EABT10947	0	4	28	3	1	0	0
EABT10948	7	6	0	0	0	0	0
EABT10949	19453.6	20935.8	16554.3	18682.4	16301.2	22354.1	18408.7
EABT1095	1	10	13	11	0	0	1
EABT10950	1116	2138.01	2598.98	2911.02	3201.54	268	1172
EABT10951	2	6	23	2	5	3	10
EABT10952	2538.01	3644.18	3021.54	6254.45	2987.74	3722.48	3070.87
EABT10953	1	1	0	1	4	3	7
EABT10954	0	0	9	1	4	1	1
EABT10955	469.99	603	319.15	1655.62	624	526.31	376.01
EABT10956	0	5	0	1	2	0	0
EABT10957	0	0	3	0	0	2	0
EABT10958	1	0	40	1	0	0	0
EABT10959	32	27	16	2	9	0	1
EABT1096	0	0	2	14	0	0	0

EABT10960	0	0	0	25	3	0	1
EABT10961	0	2	4	0	1	0	0
EABT10962	10	19	0	0	0	0	6
EABT10963	0	0	11	1	0	0	0
EABT10964	0	1	18	0	0	1	0
EABT10965	0	0	4	2	0	0	0
EABT10966	0	0	0	0	0	0	677.92
EABT10967	26	60.08	26.98	11	0	0	0
EABT10968	5	2	15	3	1	3	0
EABT10969	4	2	17	0	0	1	0
EABT1097	35	28	2	1	126	15	597.04
EABT10970	0	1	81	1	1	1	0
EABT10971	1	1	0	2	0	0	2
EABT10972	0	0	14	1	1	0	1
EABT10973	2	2	14	7	0	4	1
EABT10974	3	14	8	15	8	2	2
EABT10975	25	26	4	11	1	0	0
EABT10976	0	9	134	2	206	1	0
EABT10977	2242.85	3727.99	3868.12	6312.8	2184.05	1232.01	1311.98
EABT10978	1534.15	2156.64	1275.2	2679.72	2015.98	1065.33	1241.08
EABT10979	222	216	141	38	300	306	1712
EABT1098	2	6	2	7	0	0	1
EABT10980	594.98	724.45	789.01	851.35	533.59	512.03	504.12
EABT10981	6	15	28	2	7	0	10
EABT10982	0	1	8	0	0	0	0
EABT10983	0	0	0	0	0	0	0
EABT10984	0	1	8	4	0	0	0
EABT10985	0	1	10	0	1	0	0
EABT10986	0	2	10	1	0	0	0
EABT10987	293.29	433.63	469	689.22	356.43	157	368.14
EABT10988	0	7	7	7	0	5	0
EABT10989	12	27	18	43	17	5	9
EABT1099	13	3	7	9	1	33	6
EABT10990	2	10	5	5	0	1	1
EABT10991	0	0	2	3	0	0	0
EABT10992	3	3	0	3	3	0	6
EABT10993	0	5	0	1	0	1	0
EABT10994	3	6	2	5	0	1	0
EABT10995	1	1	4	8	1	0	1
EABT10996	0	2	3	3	0	0	0
EABT10997	0	0	0	1	2	0	0
EABT10998	5	1	5	0	0	0	0
EABT10999	4	10	4	46	34	1	1
EABT11	23	40	142	16	10	10	4
EABT110	731.45	942	1618.16	1504.02	814.74	336	384
EABT1100	3	3	0	4	0	0	0
EABT11000	2	0	0	0	1	3	3
EABT11001	0	1	6	0	0	0	2
EABT11002	8	14	38	36	5	3	3
EABT11003	0	5	10	0	0	0	0
EABT11004	0	0	7	1	0	0	0

EABT11005	12	103	26	43	13	1	12
EABT11006	0	0	1	0	0	0	0
EABT11007	5	15	98.22	4	1	0	1
EABT11008	1	2	7	2	0	0	0
EABT11009	277.59	249	109	213.32	113	174	187
EABT1101	10	10	14	12	5	11	11
EABT11010	1	5	31	3	0	1	0
EABT11011	1	0	2	0	2	0	2
EABT11012	2	1	0	0	1	1	0
EABT11013	1	2	35	1	1	0	1
EABT11014	16	34	0	0	0	0	1
EABT11015	0	0	15	4	0	5	1
EABT11016	0	5	0	0	0	0	2
EABT11017	5	4	2	367	23	1	4
EABT11018	0	0	3	0	0	0	0
EABT11019	10099.7	12811.3	9070.3	15677.8	4354.72	6882.82	3983.43
EABT1102	1	0	0	5	0	0	0
EABT11020	3	3	2	4	3	1	0
EABT11021	12	5	48	1	2	53	9
EABT11022	2035.06	2674.35	3172.13	4694.6	2172.13	1778.28	2365.58
EABT11023	12	6	37.99	15	13	1	4
EABT11024	1	0	4	6	11	0	1
EABT11025	69	80	122	166	65	56	68
EABT11026	2838.27	3339.69	82	11	77	1056.17	13
EABT11027	12	23	8	7	3	1	4
EABT11028	1	2	1	0	1	14	0
EABT11029	0	0	2	1	0	1	0
EABT1103	0	1	0	0	0	0	10
EABT11030	0	0	8	0	0	0	0
EABT11031	1	0	11	2	2	1	2
EABT11032	2	2	1	9	0	0	1
EABT11033	0	0	3	0	16	0	0
EABT11034	2	30	38	65	17	3	7
EABT11035	1	11	1	8	1	0	1
EABT11036	391	736.93	665.04	2401.13	512	291	283
EABT11037	0	2	5.01	1	1	0	0
EABT11038	0	0	10	0	0	0	0
EABT11039	0	0	6	0	0	0	1
EABT1104	0	8	23	5	2	0	1
EABT11040	6	17	14	393.07	164	3	8
EABT11041	0	2	16.84	1	0	0	0
EABT11042	45	24	18	74	16	61	15
EABT11043	1768.91	1739.85	664.8	178.73	360.98	1613.45	354.61
EABT11044	0	0	1	4	0	0	0
EABT11045	2	2	6	15	3	1	0
EABT11046	10	26	12	105	17	11	57
EABT11047	0	1	1	0	6	0	0
EABT11048	0	0	0	5	0	0	1
EABT11049	2	7	4	17	4	1	0
EABT1105	1	0	9	0	1	1	0
EABT11050	0	2	5	0	1	0	0

EABT11051	1206.03	1755.33	7316.59	16547	3737.77	1615.99	279
EABT11052	4	0	7	17	3	1	0
EABT11053	3	3	8.72	4	1	0	0
EABT11054	5	1	2	0	0	1	0
EABT11055	2	6	6	11	1	0	1
EABT11056	6	8	0	0	0	8	3
EABT11057	1656.06	2204.03	1476.9	4664.32	1815.81	1537.92	1061.82
EABT11058	6572.94	5415.38	2815.75	5115.92	4103.21	8459.92	4528.31
EABT11059	0	0	6	1	0	0	0
EABT1106	1	0	15	1	0	0	0
EABT11060	136	51.97	4	2	0	38	0
EABT11061	0	5	18	3	4	1	0
EABT11062	394	335	40	56.78	40	279	216
EABT11063	0	1	3	0	4	0	0
EABT11064	4	11	5	14	0	0	0
EABT11065	0	0	1	3	0	1	1
EABT11066	0	0	6	0	0	0	1
EABT11067	2	4	0	1	0	0	0
EABT11068	0	0	0	0	1	0	5
EABT11069	0	2	6	0	0	1	0
EABT1107	9611	5734.15	3045.76	5483	2617.07	10867.1	6808.02
EABT11070	1	5	36	3.13	0	0	1
EABT11071	0	0	2	0	0	2	0
EABT11072	0	4	1	1.99	0	1	1
EABT11073	2	1	16	5	2	1	2
EABT11074	0	1	2	0	0	1	0
EABT11075	0	0	3	5	11	0	0
EABT11076	6	3	3	2	1	9	4
EABT11077	1	1	16	3	0	0	0
EABT11078	15	36	69	38	12	18	22
EABT11079	1	3	2	1	0	7	1
EABT1108	0	0	12	0	0	0	0
EABT11080	0	6	11	5	3	0	1
EABT11081	1	1	2	4	25	0	0
EABT11082	0	0	0	0	1	0	1
EABT11083	0	5	9	82	18	0	2
EABT11084	4	3	4	0	1	0	0
EABT11085	0	0	8	0	0	0	0
EABT11086	0	3	7	1	1	0	0
EABT11087	0	0	3	1	3	0	1
EABT11088	1188.04	1373.51	1136	1378.56	976.25	1053.61	961.65
EABT11089	2	3	124	4	0	4	0
EABT1109	4	2	1	1	0	3	1
EABT11090	20	33	87.74	7	1	18	4
EABT11091	2	2	4	2	3	0	1
EABT11092	1	1	14	1	2	1	1
EABT11093	1	0	4	9	0	1	1
EABT11094	1	0	8	0	0	0	1
EABT11095	1	6	40	20	5	2	3
EABT11096	1	1	1	1	3	1	0
EABT11097	0	0	7	0	0	3	1

EABT11098	1	4	1	2	0	1	0
EABT11099	0	1	4	7	0	0	2
EABT111	0	2	3	4	5	0	1
EABT1110	9	4	18	6	1	2	1
EABT11100	2	2	39	34	59	3	7
EABT11101	0	2	0	12	2	0	1
EABT11102	0	0	4	11	1	0	2
EABT11103	0	0	19	0	0	0	0
EABT11104	0	5	7	9	12	0	7
EABT11105	1	0	9	0	0	0	1
EABT11106	10	15	45	79	29.1	1	2
EABT11107	201.61	331.5	217	545.04	352.73	95.01	125.86
EABT11108	1	3	9	1	0	1	2
EABT11109	19	26	63	64	12	32	34
EABT1111	0	2	5	0	0	1	0
EABT11110	0	1	2	4	3	0	0
EABT11111	15	7	51.21	15	165	6	43
EABT11112	4272.28	5804.33	3798.52	3414.61	2215.6	2233.66	2312.23
EABT11113	0	0	3	0	0	0	0
EABT11114	0	1	20	0	13	0	0
EABT11115	1	7	0	2	1	0	0
EABT11116	403.99	528.46	245.01	890.07	414.98	353.29	461
EABT11117	0	0	7	5	4	0	0
EABT11118	40607	252579	83648.2	15933.5	39719.8	1290	18875
EABT11119	1	5	6	4	2	0	0
EABT1112	872.18	1390.98	2557.86	2487.47	3882.98	660.75	735.7
EABT11120	2007.01	4163.01	3897.52	11156.4	1794.96	1315.97	1431.75
EABT11121	4	2	0	0	0	0	0
EABT11122	0	5	6	0	0	1	0
EABT11123	8	36	10	14	7	4	5
EABT11124	0	1	17	1	0	0	0
EABT11125	6	2	9	6	3	9	8
EABT11126	0	0	5	3	0	0	1
EABT11127	128.06	166	0	0	0	11	5
EABT11128	3	1	3	1	0	1	0
EABT11129	67	128.93	285	146	106	50	89
EABT1113	2	1	0	0	1	5	4
EABT11130	1	1	3	1	0	0	0
EABT11131	5	3	13	2	0	4	2
EABT11132	0	1	15	2	1	0	0
EABT11133	1	4	52.84	11	2	0	0
EABT11134	1580.98	2511.47	2073	4344.87	1868.09	1334.95	1122
EABT11135	2368.99	3699.74	2580.38	3904.01	2436.96	1841	2078.77
EABT11136	2	4	19	10	5	2	2
EABT11137	0	1	8	3	0	0	1
EABT11138	5	2	23	3	0	16	2
EABT11139	20570.7	2891.35	325	6	28	18615.6	51
EABT1114	869.42	1180	1525.04	2173.84	724.96	501	580.83
EABT11140	2800.36	5183.09	9785.02	16018.2	2932.88	2133.86	2259.12
EABT11141	5620.99	6477.55	6533.05	5679.29	6533.85	3894.35	4620.13
EABT11142	1	0	4	0	0	0	0

EABT11143	27	25	39	58	31	0	6
EABT11144	372.92	674.83	1874.9	1365.53	470.92	669.54	310.99
EABT11145	149	227.73	174	607.23	141.07	19	122.97
EABT11146	0	2	0	1	2	0	1
EABT11147	1	8	0	3	0	0	1
EABT11148	7	10	0	0	0	0	0
EABT11149	29	37	17	126	83.95	0	3
EABT1115	1	5	13.26	10	28	0	9
EABT11150	2	7	23	0	0	1	4
EABT11151	2	1	15	2	3	0	2
EABT11152	14952.3	10313.6	5823.97	8625.63	7664.65	7389.65	7245.79
EABT11153	5	6	6	1	16	2	81
EABT11154	5	5	3	56	5	0	1
EABT11155	2	3	2	0	0	0	0
EABT11156	2	3	0	0	0	0	0
EABT11157	8	15	21	28	18	1	8
EABT11158	2	4	0	0	1	10	6
EABT11159	0	1	9	0	2	1	2
EABT1116	2	8	4	8	0	0	0
EABT11160	1	4	0	2	1	0	0
EABT11161	0	2	17	2	1	1	0
EABT11162	0	6	4	1	0	0	0
EABT11163	0	0	12	0	0	0	0
EABT11164	0	1	13	1	0	0	0
EABT11165	1	4	7	6	1	5	3
EABT11166	1	2	12	2	0	2	0
EABT11167	0	0	4	0	2	0	0
EABT11168	0	1	8	1	0	0	0
EABT11169	0	2	8	1	1	1	0
EABT1117	0	1	3	1	0	0	0
EABT11170	0	0	6	0	1	0	0
EABT11171	1	1	1	1	0	0	0
EABT11172	1	2	1	2	0	1	0
EABT11173	0	4	0	0	0	0	0
EABT11174	10928.4	16569.2	14979.2	24420.2	16952.9	7594.1	14449.6
EABT11175	0	10	14	16	3	0	4
EABT11176	1	5	15	2	1	1	3
EABT11177	0	3	5	2	0	0	1
EABT11178	3	7	7	2	4	4	4
EABT11179	221.74	271	170	498.45	227	241	141
EABT1118	1	1	2	0	4	1	6
EABT11180	3	6	3	1	6	1	2
EABT11181	1	0	3	0	3	1	3
EABT11182	1904.23	2533.69	9085.97	196.16	1736.55	985.89	4450.32
EABT11183	0	0	5	1	0	1	0
EABT11184	0	3	18	0	0	0	0
EABT11185	5	6	0	0	0	1	2
EABT11186	1	0	2	2	5	2	1
EABT11187	18.66	27.92	14	77	54	5.01	29
EABT11188	577.74	754	214	484.02	1711	21	64
EABT11189	9	6.4	71.18	67	8	1	21

EABT1119	8	0	1	0	1	43	0
EABT11190	16639.1	15682.2	14613.6	3188.99	5711.66	5655.86	3364.03
EABT11191	4	5	7	16	2	5	3
EABT11192	0	0	2	5	0	0	1
EABT11193	0	8	29	8	3	0	2
EABT11194	5	1	0	0	0	2	4
EABT11195	0	3	71	2	0	2	2
EABT11196	0	0	3	0	1	8	0
EABT11197	0	3	6	2	1	0	1
EABT11198	16419	49166.8	25281.4	8067.76	7810.8	56	2178.95
EABT11199	0	4	2	10	1	1	0
EABT112	0	1	2	4	1	0	1
EABT1120	1	1	3	4	3	1	2
EABT11200	11	38	57	99.01	2	3	6
EABT11201	0	0	2	2	1	0	0
EABT11202	1	1	7	8	4	0	0
EABT11203	2	10	9.13	9.94	4	0	3
EABT11204	211.38	230.06	52	140	143.15	5	13
EABT11205	3556.12	4746.01	5639.99	4516.98	1815	2092	1953.59
EABT11206	0	2	5	0	0	0	0
EABT11207	0	1	10.77	44	6	1	0
EABT11208	0	4	3	7	1	0	0
EABT11209	0	1	6	1	0	0	0
EABT1121	2	6	4	6	3	1	5
EABT11210	1597.63	2115.97	2357.85	3395.19	1920.28	1357.23	1287
EABT11211	0	0	12	0	3	2	0
EABT11212	0	0	11	1	0	2	0
EABT11213	10209.2	19302.3	14082.7	21169.4	4996.23	6073.75	2745.8
EABT11214	912.22	1309.96	1679.21	3286.94	2144.81	715.22	804.66
EABT11215	4	3	0	2	0	1	0
EABT11216	0	1	3	4	1	0	1
EABT11217	6	7	18	17	5	3	3
EABT11218	272	387	290.85	507	394	91	253
EABT11219	0	0	6	6	0	0	0
EABT1122	0	0	9	0	0	0	0
EABT11220	3	3	4	0	5	0	0
EABT11221	2	0	5	0	0	0	0
EABT11222	0	5	11	18	2	1	1
EABT11223	0	0	0	1	4	0	1
EABT11224	1067.44	2664.37	5267.6	5429.49	3707.62	776.69	1686.63
EABT11225	43.11	263	106	324.65	106.06	4	59
EABT11226	5	8	8	3	1	0	1
EABT11227	179	111	49.39	14	73	0	2
EABT11228	0	0	1	0	0	0	2
EABT11229	0	3	19	1	0	0	0
EABT1123	6	24	7	5.92	3	1	1
EABT11230	0	4	4	3	0	2	1
EABT11231	8	4	0	0	0	11	2
EABT11232	8	9	15	22	247	8	7
EABT11233	0	5	2	6	0	1	1
EABT11234	7	8	22	1	3	2	3

EABT11235	9	25	75	22	2	13	7
EABT11236	1095.41	2195.79	1597.9	3375.92	918.79	25	101
EABT11237	1768	2329.68	1777.01	2781.98	1515.15	1360.97	1332
EABT11238	2	1	4	11	1	1	1
EABT11239	2	7	18	12	1.05	0	4
EABT1124	1	1	15	0	1	2	1
EABT11240	386.26	444.51	129.12	367.41	179.02	181	148.89
EABT11241	1090.92	1098.81	598.56	1466.23	1300.86	1390.22	1024.81
EABT11242	9	23	5	3	0	13	15
EABT11243	0	8	3	46	6	0	4
EABT11244	650.82	946.06	537.16	1661.66	614	446	491
EABT11245	0	0	6	0	0	0	0
EABT11246	1	1	17	2	0	0	0
EABT11247	0	2	4	0	1	0	3
EABT11248	13	18	11	6	0	22	0
EABT11249	454.68	725.01	768.89	964.32	516.35	462.94	537.67
EABT1125	3	6	7	7	0	0	1
EABT11250	605	657.97	849	921.75	640	434	294
EABT11251	891.64	1265.02	929.64	2029.38	1219.16	1075.14	1044.9
EABT11252	898.05	1394.44	2216.42	4782.89	872.98	1121.16	854.77
EABT11253	7	5	173.93	1	0	1	1
EABT11254	97.12	289.83	651.61	376.07	80.8	73	94.92
EABT11255	12	12	1	2	1	0	0
EABT11256	0	0	1	2	1	0	5
EABT11257	2	5	11	0	1	0	0
EABT11258	1631.55	6718.63	28762.3	1256.33	3713.12	1575.07	559.6
EABT11259	13	223	176	29	16	13	9
EABT1126	1	8	131	1	0	2	1
EABT11260	0	1	9	0	0	0	1
EABT11261	3	6	12	10	1	0	0
EABT11262	7	9	8	4	1	0	0
EABT11263	1	13	9	19.03	5	2	3
EABT11264	3623.26	6329.87	4646.48	13158	2833.74	1968.97	1886.51
EABT11265	0	0	5	6	0	0	1
EABT11266	578	2842.33	285	2472.01	652	21	17
EABT11267	5	20	25	52	3	0	2
EABT11268	2	1	3	1	0	0	0
EABT11269	0	6	8	1	0	0	0
EABT1127	3	6	4	2	4	0	0
EABT11270	13	29	15	20	7	4	11
EABT11271	0	4	13	12	2	0	0
EABT11272	0	0	9	4	0	0	0
EABT11273	1	1	0	2	1	0	0
EABT11274	3	7	29	9.99	4	2	1
EABT11275	144	192	112	466.64	242.04	171	161
EABT11276	1	3	21	272	157	0	8
EABT11277	0	0	1	9	57	0	2
EABT11278	0	0	3	0	0	0	2
EABT11279	0	1	2	4	0	0	0
EABT1128	4	3.94	1	0	1	0	1
EABT11280	2	1	5	0	0	0	1

EABT11281	1	3	2	2	0	1	0
EABT11282	1	2	1	4	1	0	0
EABT11283	2	8	15	5	9	0	7
EABT11284	0	3	5	0	1	0	0
EABT11285	8900.97	11698.6	29554.9	16987.3	8727.58	6310.25	9342.65
EABT11286	0	0	31	3	1	1	0
EABT11287	14.09	32.89	88	331	35	8	59.98
EABT11288	0	6	8	2	1	0	0
EABT11289	0	0	0	1	1	0	0
EABT1129	176305	33582.4	271.05	18	2403.03	70080.7	6966.47
EABT11290	2	2	3	3	0	1	1
EABT11291	0	1	19	0	1	2	0
EABT11292	1	3	6	1	6	2	8
EABT11293	1	0	1	0	0	1	2
EABT11294	5	0	0	0	0	19	0
EABT11295	1	0	2	3	0	2	0
EABT11296	517	763	780.67	1622.63	569.93	389.7	528.48
EABT11297	1	1	10	2	2	0	0
EABT11298	3	4.01	6	18	3	4	3
EABT11299	1	3	2	1	1	0	0
EABT113	12	16	0	4	1	0	0
EABT1130	0	2	48	20	21	6	88
EABT11300	1	11	26	2	0	3	7
EABT11301	0	1	7	0	0	0	0
EABT11302	5	8	32	43.11	67	0	22
EABT11303	0	2	2	3	4	3	10
EABT11304	47	24	7	18	2	14	2
EABT11305	0	6	2	11	0	7	5
EABT11306	0	1	0	9	1	0	0
EABT11307	7	6	8	7	3	2	1
EABT11308	1	3	1	0	0	0	0
EABT11309	1	7	36	5	1	0	0
EABT1131	0	3	54	6	3	4	3
EABT11310	1.08	2	42	0	0	3	0
EABT11311	1	9	50	19	0	4	1
EABT11312	2	2	10	0	0	2	0
EABT11313	52.98	60	47	74.21	52	38	23
EABT11314	1	2	2	8	1	0	0
EABT11315	1	4	1	0	0	1	0
EABT11316	0	1	1	1	1	0	0
EABT11317	1	7	4	22	2	0	0
EABT11318	0	0	13	1	1	0	0
EABT11319	0	0	10	0	0	0	0
EABT1132	0	1	16	3	0	0	0
EABT11320	2	2	2	2	4	6	1
EABT11321	1	0	4	0	0	0	0
EABT11322	0	0	5	1	0	0	0
EABT11323	2367.27	3107.76	1620.03	1452	1408	1734.61	1531.86
EABT11324	3075	10	0	1	0	295.35	264
EABT11325	3	5	5	7	1	1	4
EABT11326	402	604.71	577.65	1271.78	622.05	429.29	418.49

EABT11327	1	1	8	0	0	0	0
EABT11328	0	1	50	47	0	0	1
EABT11329	5	7	4	2	0	2	1
EABT1133	16	16	49	508	21	0	2
EABT11330	0	2	3	0	0	0	0
EABT11331	1362.97	2539.37	2406.15	5642.24	889	1075.55	630.96
EABT11332	0	0	0	2	1	0	7
EABT11333	2	2	0	0	0	4	0
EABT11334	30.98	71	226	1006.99	68	22	53
EABT11335	17	81.11	116.03	74.79	143.92	4	37
EABT11336	1	2	6	2	1	3	1
EABT11337	3	3	1	25	2	2.53	2
EABT11338	8	8	8	0	2	4	9
EABT11339	0	2	34	3	1	0	0
EABT1134	0	0	4	2	1	1	0
EABT11340	0	0	5	0	9	1	0
EABT11341	3715.62	3179.65	822	4205.64	1874.84	2458.64	3466.19
EABT11342	1	1	22	1	0	1	0
EABT11343	2	0	7	70	31	2	5
EABT11344	3	14	2	2	2	6	5
EABT11345	0	3	11	0	0	1	0
EABT11346	1	0	1	2	0	1	1
EABT11347	12	25	54	89	2	0	0
EABT11348	0	1	4	2	1	0	0
EABT11349	1	8	41	6	1	2	1
EABT1135	1	3	19	3	2	3	3
EABT11350	532.68	670.35	634.41	552.1	472.94	522.13	459
EABT11351	7	6	10	2	7	12	5
EABT11352	1	2	4	0	0	0	0
EABT11353	0	0	7	1	0	0	0
EABT11354	5	7	73	8	2	1	8
EABT11355	3867	3132	2011.95	2839	2580.01	5486.98	3127
EABT11356	0	0	9	9	3	1	3
EABT11357	0	0	27	0	0	0	0
EABT11358	1	2	2	101	0	0	0
EABT11359	0	6	10	12	15	0	1
EABT1136	1	1	9	3	1	1	0
EABT11360	0	0	8	2	0	0	0
EABT11361	1	3	1	0	0	0	0
EABT11362	4	10	18	14	1	2	3
EABT11363	0	0	3	5	1	0	1
EABT11364	11	9	6	6	0	0	0
EABT11365	20	12	42	8	5	2	17
EABT11366	1	1	6	1	0	0	0
EABT11367	0	1	8	2	0	1	0
EABT11368	1142	1432.9	1090	1744.03	1007	766.01	655.93
EABT11369	6	32	65	312	22.28	5	14
EABT1137	0	0	3.01	0	0	0	0
EABT11370	58	304.4	190	864.7	57	3	18
EABT11371	9	46	37	25	28	0	17
EABT11372	2	2.89	29	4	1	0	0

EABT11373	0	1	10	11	0	0	0
EABT11374	6	4	20	15	1	6	2
EABT11375	2	6	55	4	3	1	1
EABT11376	3	3	12	4	2	1	1
EABT11377	0	0	33	0	0	1	0
EABT11378	1	14	8	0	2	0	2
EABT11379	2412.93	4214.9	6659.48	11059.5	3875.3	1956.44	2078.55
EABT1138	0	5	5	1	0	2	3
EABT11380	2	4	1	8	4	0	8
EABT11381	687.8	1033.45	1041	1196	454	412	275
EABT11382	714.04	1415.3	2188.95	5245.59	1177.9	403.01	623.4
EABT11383	1468.49	2069.59	1728.36	3954.25	1576.93	1014.05	962.45
EABT11384	43377.3	41779.5	2271.99	2978.16	16757	67859.5	75667.8
EABT11385	0	2	12	0	1	0	1
EABT11386	1719.78	3328.34	8929.98	5223.01	1648	834.36	1883.4
EABT11387	0	1	11	0	0	0	0
EABT11388	10.01	22	32	35	11	0	38
EABT11389	185.02	287.52	267.85	717.45	443.43	36	95.61
EABT1139	54	60	34	99.01	58.61	37.02	57
EABT11390	34	33	0	0	7	58	58
EABT11391	0	2	2	1	1	0	2
EABT11392	4	2	3	1	0	1	0
EABT11393	1	4	1	7	0	0	0
EABT11394	174	182	100	274	150	92	104
EABT11395	4121.36	3371.7	771.63	62	576.55	21	37
EABT11396	2	0	3	5	0	0	0
EABT11397	0	1	3	2	1	0	2
EABT11398	2	8	1	0	0	0	0
EABT11399	0	0	13	2	1	1	0
EABT114	1119.27	1449.18	1731.21	1482.88	1251.51	339	1377
EABT1140	1	1	6	2	0	0	1
EABT11400	2	9	8	6	4	0	0
EABT11401	3100.91	4215.09	3778.88	7917.64	5332.11	2257.89	2104.59
EABT11402	0	6	1	16	0	0	0
EABT11403	128	109	11	165	37	134.98	169
EABT11404	0	0	2	2	0	0	0
EABT11405	0	0	5	0	0	0	0
EABT11406	9099.92	12646.9	3321.39	1825.16	8881.64	5238.81	14377.2
EABT11407	3	5	7	26	2	2	6
EABT11408	8	2	22	3	4	1	3
EABT11409	2	13	264	37	5	0	7
EABT1141	2	1	3	9.91	3	1	1
EABT11410	3	45	4	21	3	4	13
EABT11411	1	3	8	1	0	2	0
EABT11412	0	1	12	1	0	0	0
EABT11413	1	3	17	1	2	0	2
EABT11414	802.86	1509.77	2732.63	3217.16	2174.58	1263	1065.34
EABT11415	2	4	24	10	1	0	0
EABT11416	9	10	8	2	2	0	0
EABT11417	2	1	21	0	2	1	2
EABT11418	15	19	50	13	24	3	19

EABT11419	0	0	1	0	1	0	2
EABT1142	49	81	184.38	265.76	170	28.81	56
EABT11420	0	0	1	4	0	0	0
EABT11421	0	1	13	5	1	0	0
EABT11422	0	2	2	14	1	1	6
EABT11423	1707.94	2056.04	1449.59	1196.83	1061	1566.47	886.92
EABT11424	0	2	23	6	2	2	0
EABT11425	1	2	2	4	0	1	0
EABT11426	0	0	0	3	1	1	1
EABT11427	1	2	4	2	1	0	0
EABT11428	1	11	7	2	0	0	0
EABT11429	42	49	21	5	60	1	4
EABT1143	0	1	11	1	0	0	0
EABT11430	2	7	4	5	0	3	1
EABT11431	36	52	117	98	2	19	12
EABT11432	0	2	5	0	0	0	0
EABT11433	0	0	10	2	0	0	1
EABT11434	4	1	3	5	1	0	6
EABT11435	0	0	3	2	1	0	0
EABT11436	1	1	4	1	0	1	0
EABT11437	170	471.66	3525.18	2501.92	2598.37	18	120
EABT11438	0	0	1	2	2.95	0	0
EABT11439	1	3	9	53	1	0	0
EABT1144	3	5	16	9	0	2	0
EABT11440	0	4	9.9	0	0	0	1
EABT11441	14	41	5	3	1	0	1
EABT11442	27.89	4	26	2	1	7	7
EABT11443	4	5	9	6	1	2	0
EABT11444	0	0	5	0	6	0	0
EABT11445	1	5	5	6	4	1	3
EABT11446	1	0	1	2	0	2	0
EABT11447	8	23	42	3	10	1	15
EABT11448	0	0	0	1	0	4	6
EABT11449	1	2	4	0	0	1	0
EABT1145	63	101	51	799.54	412.97	8	111.95
EABT11450	8	20	36	37	9	4	15
EABT11451	0	4	5	6	13	2	1
EABT11452	0	2	0	1	1	0	2
EABT11453	594	788	274.99	2500.73	511.04	390	483.28
EABT11454	0	3	7	4	0	0	0
EABT11455	2645.94	6080.04	9597.4	8068.62	2898	3174.96	2865
EABT11456	0	0	10	1	1	0	0
EABT11457	0	1	2	0	0	1	1
EABT11458	1	7	1	0	0	0	4
EABT11459	1	1	2	6	2	0	0
EABT1146	0	0	11	3	2	0	0
EABT11460	0	0	4	0	0	0	0
EABT11461	1	2	5	0	0	1	1
EABT11462	56	97.91	37	267.05	77.03	11	18
EABT11463	1	0	3	0	0	0	1
EABT11464	1	1	4	1	0	2	0

EABT11465	68.01	47	37	527.4	2281.23	347.51	8723.37
EABT11466	2080.05	5010.08	3100.79	5011.01	3179.4	1779.54	3284.9
EABT11467	1541.06	2178.28	3126.65	1480.85	317.82	118.03	150
EABT11468	2968.2	3789.25	2570.5	5337.47	2333.26	1757.82	1539
EABT11469	7	7	5	1	2	1	2
EABT1147	0	3	1	4	0	0	0
EABT11470	0	0	6	0	0	0	0
EABT11471	21	32	260	25	11	8	4
EABT11472	13	33	22	22	7	10	10
EABT11473	0	2	4	1	0	0	0
EABT11474	0	2	1	0	0	0	0
EABT11475	1	3	17	0	1	1	0
EABT11476	1032.99	1119	1096.04	1785.09	1046	769	612.99
EABT11477	1	2	1	2	0	0	2
EABT11478	29	15.49	248	21	17	12	25
EABT11479	860.14	99	14	25	27	791.02	190
EABT1148	2866.43	5161.91	13888.4	20686.2	5456.03	2267.44	6668.95
EABT11480	0	1	6	1	0	0	0
EABT11481	5309.37	8191.92	6191.68	7977.69	3615.45	9982.94	7289.61
EABT11482	2	1	7	0	0	1	1
EABT11483	1	2	19	0	0	2	0
EABT11484	2	0	0	0	11	0	0
EABT11485	102	73	80	16	415	5	6
EABT11486	0	1	9	3	1	0	0
EABT11487	2	6	74.01	12	7	3	4
EABT11488	0	0	18	6	0	0	0
EABT11489	0	2	16	3	3	0	2
EABT1149	0	2	16	0	0	0	0
EABT11490	75.98	56.97	33	3	0	20	6
EABT11491	1	0	0	4	0	2	0
EABT11492	1	0	16	0	2	0	4
EABT11493	0	3	10	4	0	0	1
EABT11494	0	6	2	4	1	3	8
EABT11495	0	1	2	4	0	1	1
EABT11496	2	1	13	0	0	0	0
EABT11497	10	36	15	2	12	13	10
EABT11498	0	2	2	6	2	1	3
EABT11499	5	1	19	4	1	2	2
EABT115	0	0	12	1	1	2	0
EABT1150	0	1	0	0	0	0	2
EABT11500	0	2	1	3	0	0	0
EABT11501	1	1	0	0	1	7	15
EABT11502	1	1	10	1	0	0	0
EABT11503	0	0	0	3	0	0	0
EABT11504	1	0	3	2	2	1	1
EABT11505	3196.74	2221.9	165.87	161	3072.42	4284.69	14542.9
EABT11506	199.78	339.24	790.21	417.29	263	108	168.99
EABT11507	3	7	19	1	3	2	5
EABT11508	7	2	34	6	3	2	1
EABT11509	7	1	1	0	0	21	0
EABT1151	239.07	311.11	405.89	800	949.95	97.94	80

EABT11510	2	1	9	0	0	0	1
EABT11511	1301	2424.13	1335.2	4866.7	2410.98	2028.36	1329.05
EABT11512	25	45	9	2	7	0	3
EABT11513	5372.99	6142	4605.21	4193.11	4956.03	5024.77	5578.1
EABT11514	643.81	1077.99	1449.01	1299.95	1045.96	568	631
EABT11515	11	3	47	0	3	0	1.42
EABT11516	0	13	15	7	7	1	6
EABT11517	441	567.99	513	1512.28	733.01	481.01	687.57
EABT11518	3	17	4	11	8	5	6
EABT11519	0	0	0	1	1	1	4
EABT1152	62	154	101.13	38	7.09	21	10
EABT11520	1	4	0	0	0	0	0
EABT11521	24	27	12	31.94	4	18.01	5
EABT11522	3	12	7	13	4	4	7
EABT11523	5	4	7	13	2	3	6
EABT11524	0	0	19	0	0	1	0
EABT11525	0	3	0	0	0	2	0
EABT11526	1	2	3	9	4	1	1
EABT11527	1692.11	2218.85	1105.47	2579.49	1548.8	1812.26	2087.71
EABT11528	9	16	9	46	0	0	4
EABT11529	0	0	6	4	1	0	0
EABT1153	1701.38	2077.7	2151.32	3418.04	1785.23	1743.32	1357.35
EABT11530	9947.19	10776.2	3551.01	7338.26	32556	9145.36	8095.75
EABT11531	1	4	2	5	0	0	0
EABT11532	1600.04	1951.77	1121.01	2085.88	1365.04	1616.62	1115.9
EABT11533	2	5	6	6	3	0	0
EABT11534	3	1	26.08	1	0	0	0
EABT11535	0	0	4	0	5	1	5
EABT11536	0	0	9	0	0	0	0
EABT11537	0	0	15	0	0	0	0
EABT11538	406.98	852.23	356.99	969.52	517.27	401.93	569.09
EABT11539	0	0	3	1	0	1	0
EABT1154	2256.68	3141.03	1992.8	5970.64	1746.67	1586.54	1665.02
EABT11540	3	10	59	4	6	4	9
EABT11541	0	2	2	0	1	0	0
EABT11542	89	173	102	106	183.45	11	273.98
EABT11543	4	10	9	27	13	3	0
EABT11544	0	1	0	0	0	2	0
EABT11545	3014.8	5166.68	2321.07	8218.14	2852.71	2111.13	2416.92
EABT11546	2	0	8	2	0	1	0
EABT11547	0	0	10	0	0	0	0
EABT11548	0	1	1	1	0	3	4
EABT11549	3	5	0	0	2	1	12
EABT1155	0	1	11	0	0	0	0
EABT11550	0	0	1	4	1	0	0
EABT11551	21	45	155	13	143	18	17
EABT11552	1	1	13	2	0	2	0
EABT11553	1	3	20	2	1	0	1
EABT11554	2	3	6	0	1	0	0
EABT11555	4.97	7	1	12	0	2	1
EABT11556	0	0	6	0	0	1	1

EABT11557	5	4	9	13	4	3	2
EABT11558	17	50	96.26	83	11	29	22
EABT11559	1	0	9	8	1	1	1
EABT1156	2	14	26	12	1	1	4
EABT11560	0	3	21	2	0	0	0
EABT11561	871.14	854.91	1123.99	2346.87	943.47	721.94	757.56
EABT11562	3	16	49	17	19	19	9
EABT11563	5009.91	8925.38	7332.13	19438.8	9207.02	5920.24	6232.37
EABT11564	0	1	6	0	0	0	0
EABT11565	1487.74	1861.18	1525.88	3604.21	1599.52	651.89	639.09
EABT11566	16	32	4	27	5	2	5
EABT11567	4	0	0	0	0	6	5
EABT11568	3	19	33	15	15	4	12
EABT11569	12	19	4	5	19	0	25
EABT1157	22	57	247	62	12	25	28
EABT11570	2	3	2	0	0	3	0
EABT11571	4	5	10	5	2	5	1
EABT11572	518	797.02	880	981	639	499	486
EABT11573	0	0	3	2	3	0	0
EABT11574	6984.18	6662.66	18657.4	10728.7	2678.39	6121.99	1763.45
EABT11575	476.91	675.08	573.91	2361.88	900.42	169	621
EABT11576	4	35	88	283.14	12	1	4
EABT11577	1	3	4	0	2	1	0
EABT11578	93	111	115	263.77	187.01	111.69	267.83
EABT11579	0	1	0	1	1	0	0
EABT1158	0	0	10	0	1	0	0
EABT11580	0	2	55	3	1	2	1
EABT11581	1064.26	1826.46	1983.44	3047.33	967.59	874	1213.01
EABT11582	2	1	8	4	1	0	0
EABT11583	112.57	211	330.51	385.11	119	87	86
EABT11584	2	1	3	4	3	0	0
EABT11585	2	10	13	7	0	4	4
EABT11586	3	8	9	14	3	5	2
EABT11587	0	4	13	0	2	0	0
EABT11588	1	1	0	2	0	3	3
EABT11589	12	11	86	15	7	12	13
EABT1159	0	1	4	6	0	0	0
EABT11590	1606.57	2576.6	1631.28	3762.48	2012.47	1386.15	1592.36
EABT11591	6	8	8	5	15	0	7
EABT11592	1	21	10	4	18	0	4
EABT11593	946	1944.34	1571.93	363	1361.86	150	477
EABT11594	0	4	102	1	2	6	1
EABT11595	0	3	0	6	2	2	0
EABT11596	7	3	0	0	0	0	0
EABT11597	1	8	13	7	1	0	2
EABT11598	0	9	10	0	2	1	1
EABT11599	0	5	2	7	0	1	0
EABT116	0	0	5	0	0	0	0
EABT1160	4	7	0	1	1	3	2
EABT11600	8	4	3	1	2	8	4
EABT11601	1078.96	1737.23	1123.67	4397.78	1530.17	1136	1560.9

EABT11602	2	2	6	4	0	0	0
EABT11603	0	2	7	1	1	1	1
EABT11604	0	0	9	5	1	0	0
EABT11605	0	0	3	0	1	1	0
EABT11606	9	13	7	44	3	0	7
EABT11607	6059.82	11991.5	17518.2	18305.1	8082.51	6942.24	6387.6
EABT11608	42	115	21	205	15	8	15
EABT11609	332	657	584	507	694.99	317	295
EABT1161	1183.77	1605.65	1436.82	1630.8	1071.73	1316.32	1214.87
EABT11610	10	22	37	105	97	1	21
EABT11611	1334	1452.96	1698	2569.19	709	1740.25	914
EABT11612	5	0	1	1	2	5	0
EABT11613	0	5	11	11	3	0	1
EABT11614	0	0	19	0	0	0	0
EABT11615	1	7	10	94	2	0	1
EABT11616	20	44	23	7	13	20	25
EABT11617	2	11	28.45	44	3	3	4
EABT11618	0	0	5	0	0	0	0
EABT11619	0	0	2	0	0	0	15
EABT1162	5.66	12.4	49	2	2	0	2
EABT11620	2	0	6	2	1	0	1
EABT11621	0	1	17	2	0	0	0
EABT11622	1	2	7	0	3	0	0
EABT11623	13937.2	21100.7	6766.84	928.32	1132.36	809.69	255.9
EABT11624	0	0	2	0	0	0	0
EABT11625	3	8	62	38	1	1	0
EABT11626	16	133.06	857.09	606	36	12	51
EABT11627	2	4	1	2	3	3	6
EABT11628	0	10	5.7	1	1	0	1
EABT11629	15	16	9	66	81	2	54
EABT1163	4	2	0	0	1	10	6
EABT11630	1	5	20.96	3	1	0	1
EABT11631	483	428	152	254	454.78	190	580.1
EABT11632	0	1	1	12	6	0	0
EABT11633	0	2	5	0	0	0	0
EABT11634	1	0	2.95	1	0	11	2
EABT11635	244	245	98.99	85	101	310	372
EABT11636	0	1	4	0	0	0	0
EABT11637	14	10	64	23.17	13	9	4
EABT11638	121	133	259.1	4	60	48	93.03
EABT11639	23	41	56	21	1	4	6
EABT1164	0	1	4	0	0	0	0
EABT11640	0	9	20	8	7	0	1
EABT11641	0	1	20	0	0	0	0
EABT11642	1	0	3	0	2	2	0
EABT11643	5	8	2	15	4	0	1
EABT11644	0	3	3	7	15	0	0
EABT11645	4	3	0	0	0	8	0
EABT11646	1642.31	2476	2541.95	3798.86	1264.96	1561.02	1405.28
EABT11647	1	1	5	2.01	0	0	0
EABT11648	0	1	11	10	1	1	0

EABT11649	150	233	361.87	906.3	447	98.04	136
EABT1165	19214.6	21483.6	18931.5	14607.7	13714	12373.8	17055
EABT11650	142.96	174.51	228.34	338.01	161.93	159.04	131
EABT11651	0	1	1	0	0	0	3
EABT11652	1	2	6	1	0	0	0
EABT11653	7	8	3	3	2	0	1
EABT11654	0	1	3	0	0	0	1
EABT11655	1	1	2	1	0	0	1
EABT11656	255	388	132	691.9	173	72	140
EABT11657	5	1	17	3	2	2	8
EABT11658	150	191	278	448.03	281	126	211
EABT11659	1	0	10	0	0	2	0
EABT1166	3	4	5	15	3	2	5
EABT11660	0	0	0	0	4	2	4
EABT11661	3	2	11	2	2	0	0
EABT11662	0	2	33	5	0	0	0
EABT11663	3	22.13	21	30	24	7	5
EABT11664	1343.99	1758.08	1149.06	2505.38	1312	1233.39	970
EABT11665	0	8	1	1	4	1	10
EABT11666	5856.9	7109.07	8845.81	7798.99	3179.54	5714.68	3236.94
EABT11667	3	3	57	0	1	8	2
EABT11668	0	1	8	0	0	0	0
EABT11669	1	4	12	3	4	4	0
EABT1167	0	0	9	0	0	0	0
EABT11670	1	4	9	10	1	0	0
EABT11671	21	5	1	0	0	0	0
EABT11672	2	4	6	10	2	0	0
EABT11673	825.97	1158.63	820.73	1548.64	815.97	774.96	712.17
EABT11674	0	2	3	3	0	1	0
EABT11675	1	0	1	8	0	0	0
EABT11676	0	0	5	1	0	1	0
EABT11677	30	55	330.22	2.01	3	25	9
EABT11678	0	0	8	0	0	0	1
EABT11679	2167.12	4879.4	13096.4	5527.9	5546.21	506.24	1367.74
EABT1168	0	1	0	9	1	0	0
EABT11680	0	1	1	1	1	0	0
EABT11681	0	3	7	0	0	0	0
EABT11682	2	1	6	0	0	0	0
EABT11683	1	2	0	2	1	3	1
EABT11684	1	8	34	8	0	0	4
EABT11685	0	3	9	5	2	0	2
EABT11686	2	7	37	28	10	2	2
EABT11687	502.41	787.92	9301.52	9494.96	18	13.99	8
EABT11688	6	2	4	3	1	3	13
EABT11689	30	99	315	473.93	223.98	26	51
EABT1169	2	1	33	2	1	2	0
EABT11690	1	1	20	0	1	0	0
EABT11691	0	4	26.82	3	0	0	0
EABT11692	348.19	599.56	288	265.86	166.02	643.5	603.71
EABT11693	18	29	0	0	0	6	1
EABT11694	13	9	13	79	10	0	0

EABT11695	1539.88	2021.8	2894.64	1332.62	992.43	1171.38	1113.13
EABT11696	2179.89	2500.65	650.03	1920.1	1272.13	927.3	1316.32
EABT11697	0	1	0	3	1	0	0
EABT11698	0	0	5	0	0	0	1
EABT11699	0	0	5	1	0	11.11	97
EABT117	0	0	0	0	93	0	9
EABT1170	11	15	22	46	25	6	5
EABT11700	0	1	6	1	0	1	0
EABT11701	0	1	4	0	0	1	0
EABT11702	0	3	1	4	3	1	2
EABT11703	1	2	2	1	0	0	0
EABT11704	698	1340.94	617.06	2427.99	1046	380.27	764
EABT11705	47	0	0	0	0	1	3
EABT11706	15	59	21	20	52	7	20
EABT11707	6	2	2	13	0	0	0
EABT11708	3	9	10	0	1	1	5
EABT11709	2639.48	3839.21	1243.83	4600.51	1466.96	2430.29	2851.02
EABT1171	1479	6478.09	2492.9	1616	610	1910.12	812.06
EABT11710	519.1	662	569.98	1165.29	1393.91	410	311.02
EABT11711	1	2	1	37	3	1	3
EABT11712	111.73	296.23	2957.04	2170	571.97	135	204
EABT11713	0	1	11	0	1	0	0
EABT11714	0	2	1	0	1	1	0
EABT11715	1	1	1	0	0	3	0
EABT11716	0	3	7	3	2	0	5
EABT11717	0	1	8	1	0	1	0
EABT11718	198	358	78	107	783.07	339.76	571.65
EABT11719	0	0	6	0	0	0	1
EABT1172	2	3	2	9	1	1	0
EABT11720	0	2	14	2	0	0	0
EABT11721	199	441	356	3575.51	682.61	84	63
EABT11722	4801.9	6191.47	383.05	1288.79	635	49	18
EABT11723	0	4	4	3	0	1	0
EABT11724	2	0	4	4	1	0	3
EABT11725	0	0	7	0	0	0	0
EABT11726	0	4	3	13	2	0	1
EABT11727	19	51	77	426.46	508.01	15	15
EABT11728	0	0	1	1	7	0	0
EABT11729	13	12	34	64	8	0	3
EABT1173	48	62.98	53	31	29	152	173.34
EABT11730	3513.83	240	91	107	441	663.36	257
EABT11731	0	2	4	0	1	0	0
EABT11732	0	0	3	3	0	0	0
EABT11733	225.99	304.99	360.67	1359.87	88	96	35
EABT11734	1	1	2	3	0	0	1
EABT11735	105	195.93	14	190	499.01	6	3
EABT11736	1679.34	3153.05	2113.45	10988.9	1512.83	1145.52	840
EABT11737	0	1	3	1	4	0	0
EABT11738	2	6	15	5	0	6	4
EABT11739	0	0	10	1	1	0	1
EABT1174	0	1	26	0	0	1	0

EABT11740	1141.02	1668.21	1481.9	4322.63	1191.65	1173.01	1239.76
EABT11741	1173.99	1590.87	2230.44	3378	817.6	1136.85	899.01
EABT11742	0	5	2	2	1	0	1
EABT11743	1	0	1	1	1	0	2
EABT11744	1	1	19	0	0	2	1
EABT11745	2	0	11	1	0	0	1
EABT11746	0	0	13	96	3	0	1
EABT11747	2	5	18	8	6	0	1
EABT11748	0	2	4	4	2	0	4
EABT11749	7251.95	3454.26	4148.33	17179.8	2246.56	3041.99	3098.74
EABT1175	0	1	84	0	1	0	0
EABT11750	0	0	6	0	0	0	0
EABT11751	100	200	271	912.75	446	71	70
EABT11752	1749.72	2252.79	2514.01	4026	1827.08	1421	1062.01
EABT11753	0	1	49	2	1	0	0
EABT11754	776	911.28	506.35	1174.01	700.61	843.99	505
EABT11755	811.14	816	421	2097.85	759	166	318
EABT11756	2	1	3	2	1	0	1
EABT11757	1	4	3	94	6	1	1
EABT11758	0	0	0	6	0	0	0
EABT11759	0	0	7	0	0	0	0
EABT1176	46	66	119.83	118	28	36	33
EABT11760	0	1	4	0	109	2	0
EABT11761	1	0	6	0	1	1	0
EABT11762	0	0	6	0	0	0	0
EABT11763	0	5	25	1	2	0	0
EABT11764	162	199	125	380	136	90	122.31
EABT11765	1	0	2	0	1	0	0
EABT11766	1	5	13	1	0	0	2
EABT11767	0	3	3	0	1	1	2
EABT11768	2325.41	3396.13	4554.92	6106.72	2577.06	1342.61	1490.74
EABT11769	0	0	13	1	0	0	1
EABT1177	1905.7	2747.77	1836.89	3952.35	2726.83	1081.44	1538.99
EABT11770	461.99	649.81	767	1425	639	260	381.91
EABT11771	1	2	4	2	5	0	0
EABT11772	2	1	6	4	0	1	6
EABT11773	0	2	3	0	0	4	0
EABT11774	0	0	13	0	0	0	0
EABT11775	0	3	5	0	1	5	1
EABT11776	22	74	6	1	68	7	42
EABT11777	1	0	4	1	0	0	0
EABT11778	6	21	64	52	8	6	10
EABT11779	1	5	3	3	3	0	0
EABT1178	2	2	18	1	0	1	1
EABT11780	2157.49	2946.42	1415.7	4475.72	2148.89	1395.98	1785.56
EABT11781	0	0	8	2	2	0	0
EABT11782	0	0	7	5	0	0	0
EABT11783	0	2	4	16	2	0	2
EABT11784	0	0	0	1	1	0	0
EABT11785	0	0	0	0	0	0	0
EABT11786	0	3	20	0	0	0	1

EABT11787	12	16	18	18	4	0	6
EABT11788	2	1	3	2	0	0	0
EABT11789	0	0	1	0	1	0	1
EABT1179	1	0	4	0	0	0	0
EABT11790	1	0	3	2	0	0	0
EABT11791	7	3	32	1	1	1	1
EABT11792	1105.15	1572.75	808.9	2185.36	824.6	536.21	845.07
EABT11793	0	0	0	0	0	0	0
EABT11794	1	9	15	154	29	2	0
EABT11795	2	2	131	0	0	1	0
EABT11796	1187.13	1952.6	2878.98	3178.29	1301.71	931	916.94
EABT11797	1835.99	1656.62	846.44	1934	1323.78	443	921.34
EABT11798	0	0	4	3	0	0	0
EABT11799	68	67	195	835	255	489.71	19.79
EABT118	0	2	1	1	1	1	0
EABT1180	0	0	3	1	0	0	0
EABT11800	2	11	8	3	1	1	0
EABT11801	0	0	5	0	2	0	3
EABT11802	0	0	4	0	0	0	0
EABT11803	430.99	1043.59	934.96	130	628	6	8
EABT11804	0	1	1	2	0	0	0
EABT11805	1	3	0	2	0	0	0
EABT11806	0	5	2	3	2	0	0
EABT11807	0	0	10	5	0	0	1
EABT11808	27	19	98	63	23	43	35
EABT11809	3459.63	2593.71	4	0	2.04	775	7.46
EABT1181	19	20.94	22	23	4	9	9
EABT11810	4	3	7	10	1	3	4
EABT11811	1949.16	3377.11	1278.23	8101.59	3413.32	2074.02	1580.75
EABT11812	0	0	4	2	0	0	0
EABT11813	0	2	11	1	5	0	14
EABT11814	0	3	2	5	0	2	0
EABT11815	80	112	86	108	181	55	59
EABT11816	1	0	16	0	1	3	3
EABT11817	1	2	0	0	2	0	1
EABT11818	14103.3	1550.95	102	0	58	0	0
EABT11819	10	7	36	2	2	4	2
EABT1182	1	3	10	12	0	0	0
EABT11820	0	2	6	3	0	2	1
EABT11821	0	1	8	0	0	0	0
EABT11822	0	0	3	15.93	1	1	0
EABT11823	0	0	0	1	0	0	0
EABT11824	0	1	12	0	0	0	0
EABT11825	3	9	21	0	0	7	1
EABT11826	16	30	93	11	13	0	2
EABT11827	0	1	1	1	0	0	0
EABT11828	1	1	3	1	0	0	1
EABT11829	0	2	6	1	0	0	0
EABT1183	1	3	6	2	0	2.02	0
EABT11830	0	2	5	0	1	0	0
EABT11831	3	3	4	2	0	0	0

EABT11832	1	0	1	0	3	2	0
EABT11833	1	2	9	0	0	0	0
EABT11834	0	1	13	4	0	0	0
EABT11835	0	0	5	0	0	0	0
EABT11836	0	2	8	0	0	0	0
EABT11837	1	2	2	3	1	1	7
EABT11838	7	3	2	0	0	0	0
EABT11839	46	43	0	0	4	15	19
EABT1184	14	207	96	74	101	25	64
EABT11840	2	9	6	1	0	0	0
EABT11841	0	0	10	0	0	0	0
EABT11842	0	13	37	215	4	1	3
EABT11843	1528.28	2103.5	1235	1837.91	3027.65	595.55	835.94
EABT11844	22	46.32	39	68	25	29	18
EABT11845	0	4	6	0	2	1	0
EABT11846	1	2	37	17	10	0	6
EABT11847	14	22	102.17	35	8	0	0
EABT11848	0	0	13	0	0	0	0
EABT11849	8	18	0	16	13	0	0
EABT1185	0	0	4	2	0	1	0
EABT11850	0	0	7	1	0	0	0
EABT11851	4	6	27	8	3	13	8
EABT11852	11	37	18	47	11	7	16
EABT11853	169	77	21	6	35	0	2
EABT11854	20	8	22	29	5	4	8
EABT11855	1	0	5	2	2	0	0
EABT11856	3	10	63.99	2	52	22	126
EABT11857	13	23	227.24	60	251.82	1	1
EABT11858	7	12	11	5	6	20	31
EABT11859	0	2	5	3	1	0	1
EABT1186	9	7	33	17	9	8	13
EABT11860	0	2	3	5	0	2	0
EABT11861	16	32	105	74.04	14	5	1
EABT11862	1	3	1	0	2	1	1
EABT11863	31.94	68	55	124	40	23	30
EABT11864	7	16	90.97	11.05	1	1	10
EABT11865	96	117	41	35	108	1	4
EABT11866	0	1	2	1	0	0	2
EABT11867	0	0	0	4	0	0	0
EABT11868	29	77	158.57	64	48	127	63
EABT11869	1810.52	1950.83	3643.75	4042.29	1590.81	1521.31	1814.32
EABT1187	1	1	8	0	1	0	0
EABT11870	3	1	1	1	0	4	0
EABT11871	0	2	2	4	3	0	1
EABT11872	0	55	40	2	4	2	1
EABT11873	0	1	7	3	0	0	0
EABT11874	0	0	7	1	0	0	0
EABT11875	4	13	69	27	2	0	4
EABT11876	0	0	3	0	3	0	3
EABT11877	2	6	5	3	2	2	1
EABT11878	6	2	46	1	1	0	2

EABT11879	0	1	10	0	0	0	0
EABT1188	0	1	8	0	0	0	0
EABT11880	1	4	87	0	1	0	0
EABT11881	24	24	99	11	6	8	15
EABT11882	12	13	0	0	1	2	4
EABT11883	2	0	5	0	2	0	0
EABT11884	8014.02	14476	10230	13880.9	7492.12	7455.75	9176.92
EABT11885	805	1166.91	1144.8	1330.89	942	577	555
EABT11886	3	19	7	9	0	0	1
EABT11887	766	1207.06	810.07	3714.78	460.02	317	311.44
EABT11888	188	286	299	1297.91	445.05	216	98
EABT11889	0	0	16	0	0	0	0
EABT1189	0	0	5	2	0	0	0
EABT11890	22	79	108	173	34	19	30
EABT11891	0	3	8	2	0	0	2
EABT11892	0	0	0	0	3	0	2
EABT11893	0	0	5	1	0	0	0
EABT11894	2	0	0	0	1	4	13
EABT11895	13	14	32.4	24	21	13	17
EABT11896	0	1	4	8	0	0	0
EABT11897	5	9	0	0	0	13	3
EABT11898	0	0	5	23	2	2	1
EABT11899	0	4	15	0	0	1	0
EABT119	815.21	1206.74	1033.71	2015.14	827.41	966.98	793.97
EABT1190	0	2	18	3	1	0	0
EABT11900	0	1	12.99	0	0	2	1
EABT11901	0	0	13	0	0	0	1
EABT11902	0	0	3	2	4	3	11
EABT11903	6	16	20	34	6	17	2
EABT11904	1	0	6	2	1	0	0
EABT11905	0	0	0	0	0	0	0
EABT11906	0	0	0	0	4	0	0
EABT11907	0	0	1	2	0	6	1
EABT11908	302.95	865	1129	4441.94	770.95	120	220.67
EABT11909	17	34	261	116	7	7	3
EABT1191	0	6	12	3	1	0	0
EABT11910	3	0	3	2	0	0	0
EABT11911	12	9	2	6	8	0	0
EABT11912	3	3	17	10	3	3	2
EABT11913	12	29	48	0	21	2	5
EABT11914	2	8	8	2	3	0	0
EABT11915	20	45.11	53	85	15	15	24
EABT11916	1	8	10	16	2	2	3
EABT11917	0	0	2	9	0	1	2
EABT11918	1	1.02	5	5	1	1	0
EABT11919	45	103	494	97	102	67	101.26
EABT1192	0	0	5	0	0	0	0
EABT11920	3	7	12	1	0	32	37
EABT11921	10	12	0	0	0	0	0
EABT11922	686.7	1229.91	2589.16	2366.95	1634.5	682	667
EABT11923	574.79	670	189	834.86	427.06	280	297

EABT11924	0	0	8	0	0	0	0
EABT11925	754.74	720.18	741.43	392	2874.08	926.3	4724.86
EABT11926	144	86	11	25	68	0	4
EABT11927	0	1	1	0	0	0	0
EABT11928	19	26	5	3	5	5	64
EABT11929	0	0	7	1	0	0	0
EABT1193	2295.57	3081.77	2622.75	3180.13	3860.8	2175.84	2097.65
EABT11930	2046.09	3878.5	2478.25	8671.35	2492.83	2029.02	2389.68
EABT11931	0	0	10	1	0	0	0
EABT11932	25	46	53	60	122	23	13
EABT11933	1	0	7	47	3	0	0
EABT11934	1	1	2	0	0	1	0
EABT11935	165	366.5	565.28	124	107	29	128
EABT11936	1	1	7	1	0	0	2
EABT11937	1	1	21	1	0	0	1
EABT11938	0	0	0	0	0	0	0
EABT11939	11	49	65	29	1	6	6
EABT1194	5	44	11	3	4	8	13
EABT11940	0	3	4	0	0	0	0
EABT11941	0	4	4	3	2	0	0
EABT11942	0	0	7	0	0	0	0
EABT11943	8	19	0	3	8	29	39
EABT11944	4	17	19	19	1	1	1
EABT11945	0	0	1	2	0	2	1
EABT11946	0	0	22	1	0	1	1
EABT11947	0	0	0	0	0	0	0
EABT11948	4	2	22	2	1	0	1
EABT11949	506	1252.01	1420	3999.7	727	603.98	483
EABT1195	0	0	11	0	1	0	0
EABT11950	1	2	0	19	1	0	0
EABT11951	59	133.3	111	151	47	72	46
EABT11952	1	1	7	2	0	2	0
EABT11953	0	4	0	5	0	1	2
EABT11954	0	1	7	1	1	0	1
EABT11955	2	2	0	1	1	2	1
EABT11956	0	3	4	8	1	0	0
EABT11957	5	9	26	9	7	4	4
EABT11958	8940.27	8015.51	2215.5	314.87	1431.28	16033.7	5981.72
EABT11959	1	3	2	5	3	0	3
EABT1196	562	1082.49	3056.3	2196.32	614.97	399	574.83
EABT11960	0	3	4	0	0	0	0
EABT11961	1	3	9	11	5	1	5
EABT11962	2	1	3	0	0	0	4
EABT11963	0	0	7	3	24	8	4
EABT11964	0	1	0	1	1.01	1	7
EABT11965	2	2	2	4	2	0	0
EABT11966	0	2	1	3	4	0	1
EABT11967	0	0	8	0	1	0	0
EABT11968	968.96	1447.44	1705.21	2665.88	1561.1	804	865.09
EABT11969	17	26	57	57	85	2	3
EABT1197	2	2	3	2	1	0	2

EABT11970	1	6	21	6	0	0	1
EABT11971	0	1	7	0	0	0	1
EABT11972	1	0	3	0	1	0	0
EABT11973	2	1	1	2	1	0	0
EABT11974	1	0	10	2	0	0	0
EABT11975	0	0	0	0	0	2	20
EABT11976	0	0	0	10	0	0	0
EABT11977	0	0	1	2	1	0	0
EABT11978	2	0	11	0	0	0	1
EABT11979	0	4	3	2	0	0	2
EABT1198	12530	12881.5	8034.5	6390.95	10818	7869.08	5450.98
EABT11980	14	49	175.37	237.19	136.04	2	5
EABT11981	24	46	3	7	0	0	22
EABT11982	1584.57	1789.69	957.1	2247.9	1515.54	919.71	1005.56
EABT11983	8	2	2	0	0	2	0
EABT11984	7	14	5	0	2	2	11
EABT11985	0	3	12	2	2	0	0
EABT11986	0	2	11	1	2	0	1
EABT11987	1	3	5	10	2	1	1
EABT11988	601	646.46	526.06	986.03	365	498.99	398
EABT11989	2449.09	5876.49	7933.3	11178.4	3013.26	3200.08	3063.08
EABT1199	2	5	2	5	0	1	2
EABT11990	2765.57	3865.11	7629.48	681.53	2811.94	1144.41	1046.01
EABT11991	122	70.55	39.88	18	194.4	44	1999.05
EABT11992	24.36	45	18	92.04	51.79	25.54	38
EABT11993	3	5	6	1	1	1	1
EABT11994	0	0	6	0	1	0	0
EABT11995	2	0	2	1	1	0	2
EABT11996	61	367.09	208	15	45	3	6
EABT11997	173	322.89	186	417	126	131	171.48
EABT11998	0	0	2	17	1	0	0
EABT11999	1	1	3	0	0	1	1
EABT12	1	2	21	15	9	2.67	9
EABT120	13	18	22	1	0	0	0
EABT1200	2	0	0	59	822.08	2	4
EABT12000	3	0	12	0	0	8	1
EABT12001	0	0	1	1	0	0	2
EABT12002	0	2	2	0	0	0	1
EABT12003	0	0	0	2	2	0	0
EABT12004	0	0	0	0	1	22	10
EABT12005	877.28	1897.2	84.1	249.04	62	906.38	261.26
EABT12006	48	117.98	134.06	122.76	11	6	3
EABT12007	2	4	63	9	2	1	0
EABT12008	0	2	6	2	1	1	0
EABT12009	0	1	16	0	1	0	0
EABT1201	0	2	10	0	1	0	3
EABT12010	1	3	20	6	0	1	4
EABT12011	914.02	1565.17	1751.03	2930.4	1401.87	1196	1059.57
EABT12012	0	4	1	12	1	0	0
EABT12013	774.49	1170	1644	1715	1138.95	706.99	689.01
EABT12014	2	7	36	16	1	0	0

EABT12015	5	12.87	10	27	16	1	9
EABT12016	0	3	2	0	0	1	0
EABT12017	2	8	24	5	2	11	3
EABT12018	0	11	36	9	11	1	2
EABT12019	2	6	13	16	1	1	4
EABT1202	0	0	0	0	1	0	3
EABT12020	2	7	4	11	3	6	2
EABT12021	0	0	1	1	3	0	9
EABT12022	10	15	0	0	0	4	5
EABT12023	0	1	0	0	0	10	2
EABT12024	1	1	9	0	1	0	0
EABT12025	4	4	3	14	10	0	9
EABT12026	1	9	2	4	1	2	1
EABT12027	6	11	15	38	14	13	2
EABT12028	0	0	15	0	0	0	0
EABT12029	5	7	5	12	5	0	2
EABT1203	0	0	1	1	1	0	3
EABT12030	2	1	66.99	0	0	2	1
EABT12031	5	8.03	10	7	1	0	0
EABT12032	3939.96	13597.8	7189.86	21700.3	5356.9	631.64	1739.15
EABT12033	0	0	2	0	0	0	0
EABT12034	6	6	20	26	3	1	1
EABT12035	150	170	98	647	282	69	63
EABT12036	1	0	12	3	0	0	0
EABT12037	9	15.45	6	21	0	0	37
EABT12038	6	34	6	40	5	0	1
EABT12039	1	2	3	0	1	0	1
EABT1204	0	0	2	0	0	0	0
EABT12040	0	2	3	0	0	0	0
EABT12041	8	6	1	30	61	14	10
EABT12042	0	2	6	1	0	0	0
EABT12043	2	0	1	0	0	0	1
EABT12044	0	4	5	1	0	0	0
EABT12045	23258.9	12893	31926.3	13623.1	12786.3	13120.3	16088.5
EABT12046	1	2	1	0	3	0	0
EABT12047	19	5	6	49	1	0	0
EABT12048	656	1036.6	952.24	1236.13	1008	294	469.21
EABT12049	4	9	11	22	2	0	7
EABT1205	6	15	32	16	4	6	4
EABT12050	2	6	2	0	0	0	1
EABT12051	2	3	0	1	0	2	0
EABT12052	57	152	164	80	55	7	14
EABT12053	1	2	9	2	1	0	2
EABT12054	0	0	4	1	0	0	2
EABT12055	196.96	270	2084.76	366	1406.99	101	522
EABT12056	22	10	28	210	65	21	15
EABT12057	0	4	0	0	2	2	4
EABT12058	2495	3299.88	1803	1298.04	2951.02	2132.85	4777.95
EABT12059	0	0	23	9	0	0	0
EABT1206	0	0	11	0	2	0	1
EABT12060	2	6	0	0	0	11	2

EABT12061	0	1	1	1	0	3	0
EABT12062	3	7	106	13	90	2	22
EABT12063	28	24	0	2	0	1	0
EABT12064	458	585	418.62	1179.77	504.23	462.94	381.07
EABT12065	0	1	14	955.71	1	0	1
EABT12066	0	1	0	0	7	0	2
EABT12067	3	1	3	0	0	0	0
EABT12068	0	0	14	1	0	1	0
EABT12069	3236.67	4166.17	53	3	865	441	1765
EABT1207	3	0	6	7	1	1	0
EABT12070	0	0	6	1	0	0	0
EABT12071	0	0	9	1	0	0	0
EABT12072	270.98	468.98	441.37	1677.73	369	281	526.33
EABT12073	0	0	2	0	0	0	0
EABT12074	0	4	8	0	0	0	0
EABT12075	10	18	22	19	6	4	22
EABT12076	0	1	17	0	0	0	0
EABT12077	1	3	2	0	0	0	1
EABT12078	0	0	0	0	9	0	0
EABT12079	7	10	69	14	4	2	6
EABT1208	2	6	5	9	6	0	9
EABT12080	0	2	16	0	1	1	0
EABT12081	3	12	86.64	38	4	27	11
EABT12082	18	9	0	0	0	3	0
EABT12083	1403.96	1848.39	4265.34	2697.68	1585.02	1566.91	886.66
EABT12084	28	28	47	72	32	23	26
EABT12085	0	1	3	0	0	0	1
EABT12086	6	7	7	20	11	10	1
EABT12087	450.05	834.98	503.07	1142.01	702.03	255	358
EABT12088	8	15	33	14	11	3	4
EABT12089	4	10	5	3	2	8	1
EABT1209	0	0	5	1	0	0	0
EABT12090	2	5	17	0	0	0	1
EABT12091	0	0	8	0	0	0	0
EABT12092	6	0	1	0	0	0	0
EABT12093	0	0	21	2	0	0	0
EABT12094	34492.8	30755	12836.3	15962.9	14472.7	4871.14	4842.1
EABT12095	0	0	13	0	0	0	0
EABT12096	3	4	12	42	3	3	15
EABT12097	0	2	39	1	0	0	0
EABT12098	0	1	1	3	0	0	0
EABT12099	20	46	450	69.03	2	0	0
EABT121	371	525.99	1039.96	2259.59	518	109	210
EABT1210	0	2	1	1	1	0	1
EABT12100	0	2	6	0	0	0	0
EABT12101	1	11	5	5	7	0	0
EABT12102	1116.71	2511.87	2163.92	2852.95	1403.96	557	1330.64
EABT12103	0	4	8	1	0	0	0
EABT12104	1	1	4	7	4	0	2
EABT12105	1818.35	2566.18	2242.15	3807.34	1580.28	980.97	1342.39
EABT12106	1031	1906.2	898.94	4638.36	1602.66	344	737

EABT12107	0	3	3	0	0	11	0
EABT12108	0	4	16	0	0	0	1
EABT12109	2	6	5	2	1	0	0
EABT1211	17	18	38	8	1	15	7
EABT12110	0	1	6	1	0	21	0
EABT12111	10	16	14	11	8	4	12
EABT12112	1	1	8	22	1	0	0
EABT12113	172.58	245	1016.24	282	90	177.01	139
EABT12114	17	239.01	167.7	474.04	86	6	39
EABT12115	1	1	2	1	1	0	1
EABT12116	0	1	51	2	0	0	0
EABT12117	2	1	3	27	2	1	1
EABT12118	0	0	4	3	1	0	0
EABT12119	2	2	0	1	1	0	1
EABT1212	1	5	17	3	0	0	0
EABT12120	4	7	62	5	1	3	2
EABT12121	3	3	6	4	6	1	2
EABT12122	3	2	3	8	0	0	0
EABT12123	68	118	2380.74	69	23	59	41
EABT12124	0	2	3	0	2	1	0
EABT12125	2	4	56	0	1	0	0
EABT12126	0	8	0	1	2	35	1
EABT12127	0	1	21	1	0	0	0
EABT12128	0	5	19	1	0	0	0
EABT12129	1	21	23	73	0	0	0
EABT1213	0	1	0	2	4	0	2
EABT12130	248	51	25.13	2	42	401.55	152
EABT12131	0	4	13	0	0	1	0
EABT12132	6172.86	10020.1	10626.9	16344.3	7005.13	8593.81	5411.94
EABT12133	922.88	1642.47	1672	4586.04	1390	583.01	621.72
EABT12134	2560.16	6399.86	13780.6	7205.79	1699.46	3597.93	2213.47
EABT12135	0	0	8	1	0	0	0
EABT12136	770.01	1073.23	1308.07	1725.73	503.86	575	370.15
EABT12137	0	3	1	7	2	0	3
EABT12138	2	1	9	2	0	1	0
EABT12139	0	2	5	0	0	0	0
EABT1214	6	2	2	5	4	0	0
EABT12140	0	3	4	1	3	1	5
EABT12141	146	409	463	232	81	236	177.01
EABT12142	1	2	16	0	3	5	2
EABT12143	3.01	12.99	10	8	0	7.01	3
EABT12144	0	3	1	0	2	0	3
EABT12145	17	59.62	64.66	27	1	2	7
EABT12146	731.17	816.99	472.6	1227.15	686.56	641	561
EABT12147	1	2	13	1	0	0	0
EABT12148	1	3	24	1	1	0	0
EABT12149	19	0	1	0	0	12	0
EABT1215	0	1	13	12	1	0	2
EABT12150	180	224	166	54.03	13	83	29
EABT12151	0	1	7	1	0	0	0
EABT12152	174	579.91	1241	1805.82	1082	46	254

EABT12153	2725.41	4082.55	8228.77	6341.28	3601.86	3369.6	2919.73
EABT12154	1	10	8	3	1	3	5
EABT12155	4	6	6	2	2	4	0
EABT12156	3	8	18	74	65	2	0
EABT12157	1	1	5	2	1	5	1
EABT12158	728	1117.14	1708.21	1890.53	1270	497	562.04
EABT12159	8	3	4	3	3	0	0
EABT1216	14	18	0	0	0	0	1
EABT12160	4	3	1	0	3	0	0
EABT12161	6	35	14	313.46	2416.05	1	74
EABT12162	22	39	22	139	26	15	109
EABT12163	0	3	10	1	0	0	0
EABT12164	1286.11	1956.55	2752.02	2652.55	3173.1	657	1351.3
EABT12165	7	11	2	1	3	1	4
EABT12166	0	1	2	0	0	4	0
EABT12167	1	5	3	48	1	1	1
EABT12168	1704	2278.53	2570.56	3430.05	2112.75	1559	1460.42
EABT12169	8304.61	8791.28	5129.1	11234.8	4167.43	5284.99	8833.69
EABT1217	1	4	36	2	2	1	0
EABT12170	2	6	2	0	0	0	0
EABT12171	6	5	23	7	8.19	0	6
EABT12172	1	2	6	8	9	2	2
EABT12173	5	0	6	2	0	0	1
EABT12174	28	29	79	362.88	27	2	22
EABT12175	1353.58	2518.74	2301.33	3519.43	1825.2	1412.9	1682.23
EABT12176	31	24	23	7	2	57	5
EABT12177	2	9	24	5	3	6	9
EABT12178	4	11	0	1	0	1	0
EABT12179	0	3	2	7	3	3	1
EABT1218	0	2	4	2	0	0	0
EABT12180	1910	2130.04	1036.27	1335.78	1468.47	2887.27	2139.02
EABT12181	0	9	13	13	6	0	0
EABT12182	0	0	2	1	0	0	0
EABT12183	0	0	25.06	2	1	0	0
EABT12184	2	4	8	0	1	0	1
EABT12185	0	1	5	8	0	4	4
EABT12186	1155.81	6744.65	72.89	72	31.62	2304.57	121.03
EABT12187	1	0	5	1	0	1	1
EABT12188	0	2	17	3	1	1	0
EABT12189	706.86	918	316	1457.33	443.06	434	594.65
EABT1219	1	0	3	3	0	1	0
EABT12190	17	44.13	18	60	28	1	1
EABT12191	7765.48	14564.6	16595.4	18884.1	11428.9	4250.57	10859.6
EABT12192	698.02	1601.89	325.02	287.15	105.54	1137.13	354
EABT12193	0	1	1	0	1	0	2
EABT12194	7	1	0	1	1	0	0
EABT12195	120	412	342.99	1038.65	138	116	129
EABT12196	2	0	1	0	1	1	0
EABT12197	4	2	8	73	2	6	2
EABT12198	0	1	0	3	600.99	0	1
EABT12199	272.99	584	931	5119.84	803.28	249	470.99

EABT122	0	0	11	0	0	0	0
EABT1220	0	2	40	6	0	1	2
EABT12200	0	1	3	2	0	3	0
EABT12201	174	760.01	448	174.67	85	222	744.93
EABT12202	370	469	142.99	451.99	559.01	241	240
EABT12203	1	3	8	0	2	0	1
EABT12204	0	0	0	0	1	0	1
EABT12205	1	1	15	0	0	0	0
EABT12206	7	3	1	1	0	8	1
EABT12207	561.32	670	281	687	757	526.8	488
EABT12208	1	1	8	1	0	0	0
EABT12209	0	3	5	3	0	1	0
EABT1221	4	6	0	1	3	1	0
EABT12210	0	0	0	0	7	0	0
EABT12211	934	1328.33	4939.91	2323.84	820	117	179
EABT12212	333	71	77	53.8	119	17	15
EABT12213	0	2	3	2	0	0	0
EABT12214	0	0	5	7	0	0	0
EABT12215	2	7	7	8	2	0	1
EABT12216	1	5	2	6	2	0	0
EABT12217	1	0	13	1	0	0	0
EABT12218	13	21	47	10	0	1	4
EABT12219	431	536.07	547.55	739	217	369	367.01
EABT1222	0	0	20	2	0	0	0
EABT12220	11005.6	12163.2	8424.02	12588.7	5687.31	11199.3	8865.69
EABT12221	0	1	1	2	2	2	0
EABT12222	106	143	117	192	128.96	90	115
EABT12223	0	1	15	0	0	0	0
EABT12224	1037.73	1505.4	1091.79	2090.98	1495.75	1294.07	1292.98
EABT12225	0	1	5	4	0	0	1
EABT12226	25	47	61.97	95	23	16	24
EABT12227	1	0	7	1	0	0	0
EABT12228	39	79.96	84	84	23	3	2
EABT12229	151	207	162	326.08	132	66	73.91
EABT1223	0	0	2	0	14	0	0
EABT12230	3	3	0	0	17	0	1
EABT12231	684.31	3348.88	3053.15	1526.98	1970.26	167.97	1274.97
EABT12232	245	518	500.63	3650.04	1451.1	186	152
EABT12233	597.89	522.73	649.99	730.41	175.38	402.98	405
EABT12234	26	52	64	156	91	24	49
EABT12235	0	0	12	3	3	0	0
EABT12236	7	15	30	34	71	4	11.32
EABT12237	3	2	3	5	6	1	3
EABT12238	1	0	5	1	0	1	1
EABT12239	0	1	7	0	0	0	0
EABT1224	523.99	1099.06	2451.3	2136.18	800.21	259.02	373
EABT12240	2	4	0	0	0	3	5
EABT12241	2	4	5	5	2	2	1
EABT12242	1	1	5	0	0	0	0
EABT12243	0	0	13	0	1	1	0
EABT12244	0	2	16	1	1	1	0

EABT12245	0	0	5.01	0	0	2	0
EABT12246	0	1	0	0	0	0	3
EABT12247	1	6	17	5	2	0	0
EABT12248	20	123	619.86	2	2	8	3
EABT12249	71	128	254	1	4	17	0
EABT1225	8575.25	8660.53	5923.99	3890.14	16202.7	6110.89	5306.59
EABT12250	3	5	37	18	4	1	1
EABT12251	2365.91	1754.98	70.97	65	1223.36	4859.62	7906.97
EABT12252	0	1	1	47	212.59	0	0
EABT12253	0	1	5	0	1	0	0
EABT12254	3	3	40	2	1	4	3
EABT12255	1	3	1	2	0	0	0
EABT12256	11	12	66	14	10	11	7
EABT12257	16	22	19	22	2	3	3
EABT12258	1	2	0	1	0	0	0
EABT12259	498.58	430.48	347.85	616.21	269.96	384	197
EABT1226	1	1	2	3	0	2	0
EABT12260	1107.13	2399.57	1266.61	2573.99	1259.57	1035.91	1887.88
EABT12261	0	0	3	0	0	1	0
EABT12262	0	4.03	0	0	4	0	3
EABT12263	1771.74	2315.95	1426.26	2186.95	3049.77	614.74	800.93
EABT12264	0	0	2	3	0	0	0
EABT12265	0	2	8	0	0	0	0
EABT12266	2	0	32	6	0	1	0
EABT12267	0	0	3	72	1	0	0
EABT12268	3	0	0	0	0	4	0
EABT12269	9	10	4	62	24	15	68
EABT1227	1	0	0	1	0	2	0
EABT12270	0	1	5	0	0	0	0
EABT12271	0	17	8	8	1	1	2
EABT12272	5321.93	11246.9	18010.3	4299.29	2482.63	407.07	1284
EABT12273	2	0	15	1	0	1	0
EABT12274	0	2	6	6	0	0	0
EABT12275	12	19	0	0	8	0	26
EABT12276	3	1	8	0	2	0	6
EABT12277	9	17	37	5	16	5	12
EABT12278	0	4	10	1	0	0	0
EABT12279	1	0	5	0	1	0	0
EABT1228	9	9	58	9	25	4	10
EABT12280	2	9	12	28	3	15	3
EABT12281	1013.34	2709.15	6008.75	2868.02	2533.37	722.31	1904.18
EABT12282	7773.01	4455.08	1098	2041.01	1868.99	217	498
EABT12283	2	6	20	13	13	0	0
EABT12284	3356.84	5249.25	7486.79	14404.3	4058.21	3056.06	2303
EABT12285	0	0	3	0	0	0	0
EABT12286	0	2	19	1	1	0	0
EABT12287	2	5	21	4	3	0	1
EABT12288	40	52	35	48.22	9	19	12.81
EABT12289	2	0	1	17	1	0	0
EABT1229	1738.45	1200.95	74.01	40	1740.86	5	59
EABT12290	0	0	0	0	1	0	3

EABT12291	2	3	4	6	2	3	6
EABT12292	15	2093.32	49896.4	7505.62	549.68	1	87
EABT12293	0	0	2	3	0	0	0
EABT12294	0	0	0	11	0	0	0
EABT12295	8	46	203	407.14	86	11	19
EABT12296	13	8	0	0	2	32	16
EABT12297	2	2	3	1	0	1	1
EABT12298	28	39	41	102.05	32	24	11
EABT12299	2	2	0	0	0	1	5
EABT123	2	6	20	7	0	1	3
EABT1230	1	0	0	0	7	19.04	10
EABT12300	1416.33	2634.54	2249.3	5553.14	893.06	658.39	663
EABT12301	308	395	535	889	438	221	280
EABT12302	2	2	16	101.95	10	4	11
EABT12303	0	5	8	2	0	5	0
EABT12304	0	3	2	5	0	0	1
EABT12305	0	1	8	0	0	0	0
EABT12306	0	0	5	1	0	0	0
EABT12307	17	39	13	76	44	1	1
EABT12308	1	1	0	4	0	0	0
EABT12309	0	1	11	0	0	0	0
EABT1231	0	1	5	1	0	0	0
EABT12310	7	8	18	14	5	1	1
EABT12311	2	6	14	11	6	1	1
EABT12312	4	8	15	15	6	4	6
EABT12313	1554	1994.99	1210	2805.11	1062	1725.9	1065
EABT12314	20	25	1	3	105	23	8
EABT12315	7	52	10	4	0	4	0
EABT12316	1	2	19	6	0	0	0
EABT12317	3	5	0	0	1	20	4
EABT12318	1	1	1	1	0	0	0
EABT12319	0	4	17	0	0	1	0
EABT1232	7	1	10	4	14	1	12
EABT12320	0	0	3	1	1	0	0
EABT12321	273	471.98	1042.81	1141.24	958.08	213.16	304.3
EABT12322	0	3	17	1	0	1	1
EABT12323	3	1	5	1	1	0	0
EABT12324	4	10	9	4	2	1	5
EABT12325	3	10	9	6	0	3	7
EABT12326	42	150	71	204	110	0	0
EABT12327	0	0	10	1	0	1	0
EABT12328	6	4	0	0	0	1	0
EABT12329	5	11	0	0	1	44	30
EABT1233	3	6	3	11	1	0	2
EABT12330	0	2	4	5	9	2	1
EABT12331	2	3	53	4	1	1	2
EABT12332	0	9	7	0	5	0	0
EABT12333	4802.01	5999.71	7100.65	12932.2	3944.43	4819.07	3228.88
EABT12334	6	11	47	2	0	3	0
EABT12335	1	5	7	15	1	1	2
EABT12336	0	2	6	4	0	0	0

EABT12337	5	1	67	2	3	7	3
EABT12338	0	1	33	3	1	0	0
EABT12339	1	1	55	3	3	0	1
EABT1234	16	39	88.99	49.99	13	22	18
EABT12340	0	2	4	2	0	0	0
EABT12341	12	7	2	0	2	10	28
EABT12342	0	2	8	5	1	3	0
EABT12343	530	747.89	426.07	825.28	687.34	381.51	459.25
EABT12344	1205.93	2033.23	1549.07	1407	1767.75	2098.85	2045.82
EABT12345	1	7	68	7	0	1	1
EABT12346	388.97	468.98	350.16	642.01	403.84	210	214
EABT12347	1	2	0	0	1	6	1
EABT12348	1	1	11	1	0	0	0
EABT12349	5	13	45	11	3	5	7
EABT1235	0	0	1	0	0	0	0
EABT12350	7	5	0	0	2	0	0
EABT12351	69	173.07	52.65	72	57.06	32.51	63
EABT12352	0	1	4	2	0	0	0
EABT12353	0	0	10	0	0	0	0
EABT12354	0	1	6	4	0	0	0
EABT12355	9249.44	6366.51	182	313	979.16	1202.17	133
EABT12356	1	22	12	1	0	1	0
EABT12357	0	5	3	2	6	0	0
EABT12358	0	0	10	4	0	0	0
EABT12359	0	0	4	1	0	0	0
EABT1236	5	4	3	8	1	4	5
EABT12360	223.32	216	293.42	1	197	24	339
EABT12361	0	0	2	20	1	0	0
EABT12362	11	9	1	9	3	0	0
EABT12363	10	0	1	0	0	48	0
EABT12364	20	35	31	37	20	18	49
EABT12365	29534.9	14888.7	662	58	1353.68	46334.9	4910.49
EABT12366	5715.79	5859.5	5340.54	8006.83	2776.09	6139.51	4650.67
EABT12367	0	0	6	1	1	0	0
EABT12368	1	5	8	13	1	1	0
EABT12369	0	0	1	13	1	0	0
EABT1237	4	16	66	22	7	6	14
EABT12370	4	1	0	0	0	0	8
EABT12371	1	4	2	11	10	0	14
EABT12372	0	3	6	0	0	0	0
EABT12373	8	9	17	32	7	2	1
EABT12374	2	1	9	1	0	1	0
EABT12375	1	0	0	0	0	7	0
EABT12376	13	16	34	242	100	0	0
EABT12377	0	2	2	11	0	0	0
EABT12378	7875.59	17349.2	798.02	783	1212.62	685	2379
EABT12379	1	0	3	2	1	0	1
EABT1238	4	6	33	2	0	0	1
EABT12380	1026.32	1243.88	884.92	956.07	699.62	2209.85	844.34
EABT12381	0	1	2	13	0	0	0
EABT12382	0	0	4	0	0	0	1

EABT12383	2	3	3	2	10	1	0
EABT12384	661	1442.95	368	1326	337.22	48	130
EABT12385	1	7	13	2	0	2	1
EABT12386	1	3	26	1	0	1	0
EABT12387	0	0	1	0	3	0	0
EABT12388	1	11	4	13	4	13	5
EABT12389	4	16	31	5	2	2	2
EABT1239	0	1	2	1	3	0	1
EABT12390	0	2	31	1	1	0	0
EABT12391	118	469.96	59	12	16	414	92
EABT12392	1	1	37	1	0	2	1
EABT12393	2	2	1	3	0	0	0
EABT12394	0	0	9	0	0	0	0
EABT12395	0	1	2	2	1	4	0
EABT12396	1	6	6	21	4	0	0
EABT12397	37	22	7	0	6	24	43
EABT12398	4	3	1	1	0	3	0
EABT12399	940.35	1380.78	877.11	1528.1	691.98	962.98	686.06
EABT124	0	0	8	0	0	0	0
EABT1240	134	170.15	51	268	93.8	132	78
EABT12400	0	0	4	0	0	1	0
EABT12401	0	0	0	6	0	0	0
EABT12402	2	1	16	31	0	1	1
EABT12403	5	9	12	0	2	6	7
EABT12404	0	1	9	0	0	0	0
EABT12405	0	0	10.01	0	0	1	0
EABT12406	0	1	4	0	0	0	0
EABT12407	1	1	5	2	0	0	0
EABT12408	4	12	8	0	0	5	2
EABT12409	14	19	3	7	4	7	6
EABT1241	1	2	12	0	0	0	0
EABT12410	2	1	0	2	1	0	0
EABT12411	5200.71	4950.99	902.99	1443.44	1734.09	1152.96	6521.07
EABT12412	2	9	5	2	2	0	0
EABT12413	4	6	2	1	0	0	0
EABT12414	0	5	6	2	1	2	0
EABT12415	0	2	7	99	206	3	19
EABT12416	4	6	8	3	1	0	2
EABT12417	0	3	19	0	0	0	0
EABT12418	0	2	18	2	1	0	2
EABT12419	2	1	1	0	2	5	9
EABT1242	29	52	44	9	20	96	99
EABT12420	0	1	8	5	1	0	1
EABT12421	1	1	2	0	0	0	1
EABT12422	1	1	12	1	0	0	1
EABT12423	1	3	3	0	2	0	0
EABT12424	1	1	6	1	0	2	0
EABT12425	0	0	4	1	1	0	0
EABT12426	1976.17	3463.16	2513.1	493.47	420.66	558.56	816.69
EABT12427	0	2	8	3	3	0	0
EABT12428	0	0	8	1	0	0	0

EABT12429	1	1	3	5	0	0	1
EABT1243	0	0	1	6	0	0	0
EABT12430	0	0	12	0	0	0	0
EABT12431	0	5	9	13	1	0	0
EABT12432	0	2	0	1	0	1	0
EABT12433	2107.19	2485.7	2939.45	2574.1	2839.41	1905.34	2493.79
EABT12434	6	15	26	28	4	3	2
EABT12435	0	0	10	1	0	0	0
EABT12436	6	4	44	4	3	12	7
EABT12437	1335.6	1803.31	1437	2455	785	1599	1051.2
EABT12438	0	1	0	2	1	0	3
EABT12439	2	5	6	6	2	2	5
EABT1244	1	10	9	37	13	6	7
EABT12440	8	14	73	25	2	0	0
EABT12441	10	7	8	3	1	2	1
EABT12442	0	0	16	0	0	0	0
EABT12443	0	1	0	3	0	1	0
EABT12444	0	0	1	0	2	0	0
EABT12445	2137.83	3607.62	2132.41	6724.38	2062.62	2425.16	1659.82
EABT12446	0	0	9	1	0	0	0
EABT12447	1	0	0	0	1	0	0
EABT12448	8	20	256	84	18	3	5
EABT12449	0	0	0	0	0	12	0
EABT1245	0	0	2	0	0	0	0
EABT12450	1	5	49	11	2	0	5
EABT12451	0	0	10	3	0	1	0
EABT12452	23	24	50	50	7.01	15	23
EABT12453	4	4	10	32	3	3	1
EABT12454	1	0	11	0	0	0	0
EABT12455	0	0	0	1	0	1	0
EABT12456	1	0	6	0	4	0	0
EABT12457	6865.66	7083.41	6584.34	3052.95	2367.17	11082.8	4030.93
EABT12458	0	4	0	0	0	0	1
EABT12459	49.51	37.83	40	104	36.96	34.48	47.42
EABT1246	14	18	62	25	18	11	11
EABT12460	0	2	1	1	0	0	0
EABT12461	1	0	6	0	3	0	0
EABT12462	0	32	2	0	0	9	0
EABT12463	0	1	2	1	0	0	0
EABT12464	0	1	0	2	1	0	1
EABT12465	517	750.08	385	877	646.99	496.21	413
EABT12466	1	4	57	3	0	1	3
EABT12467	0	0	6	0	0	0	1
EABT12468	1	8	32	92	38	3	2
EABT12469	0	0	10	1	1	1	0
EABT1247	1	0	9	0	1	1	0
EABT12470	4	3	0	1	5	0	1
EABT12471	8	12	8	0	0	0	0
EABT12472	32	34.01	76.09	220.94	70	4	34
EABT12473	1	4	55	3	5	2	1
EABT12474	0	0	4	0	11	0	0

EABT12475	1	4	1	5	1	1	0
EABT12476	1	4	6	13	7	0	0
EABT12477	26.88	43	42.97	77	113	47	148
EABT12478	0	0	1	2	2	0	0
EABT12479	0	1	4	7	1	0	0
EABT1248	2	1	79.64	1	16	2	0
EABT12480	0	1	15.96	5	0	1	2
EABT12481	1	0	5	3	0	0	0
EABT12482	0	1	17	0	0	0	1
EABT12483	332	426	1138.02	722	943	301	437
EABT12484	23	59.89	22.07	12	17	16	31
EABT12485	736.72	946.05	619.42	1446.14	681.99	1176.88	693
EABT12486	2	3	0	2	0	0	1
EABT12487	0	0	4	0	0	1	1
EABT12488	1240	1884	1078	2052.7	907.22	892	1156
EABT12489	0	0	8	2	0	0	0
EABT1249	2607.99	5399.4	7350.99	13058.4	4749.32	3110.55	5406.62
EABT12490	3371.65	6835.68	14501.5	11026.2	4359.23	5611.37	5506.39
EABT12491	1	1	0	0	4	1	6
EABT12492	0	2	0	1	1	1	1
EABT12493	23	19	33	39	15	0	41
EABT12494	3	6	2	0	0	6	0
EABT12495	0	0	4	0	0	0	0
EABT12496	57	135	15	42	20	1	2
EABT12497	957.08	1219.92	729.03	1811.96	921.54	759.02	666.03
EABT12498	276	380	239	854	159	58	172
EABT12499	2	2	31	0	1	2	1
EABT125	0	0	3	17	0	0	0
EABT1250	3	1	5	4	11	8	25
EABT12500	1524	2195.83	573.01	1090.01	1159	1326.42	1509.57
EABT12501	0	2	1	5	2	0	0
EABT12502	1	1	2	3.85	8	0	1
EABT12503	0	0	8	0	0	1	0
EABT12504	3207.02	3729.06	1344.41	735.01	313	4964.13	1368.09
EABT12505	14	6	5	0	7	16	4
EABT12506	0	4	5	1	4	3	2
EABT12507	1	2	8	3	2	0	1
EABT12508	1	1	6	1	2	3	4
EABT12509	1	2	6	1	0	0	0
EABT1251	0	1	1	1	0	1	1
EABT12510	0	1	5	3	11.99	4	0
EABT12511	13911.1	18543.1	19556.7	11441.4	10894.8	8186.56	13292.2
EABT12512	0	1	6	0	0	0	0
EABT12513	0	0	5	1	0	0	0
EABT12514	1	1	7	0	0	0	0
EABT12515	1	2	8	1	0	0	1
EABT12516	1	4	4	8	3	0	1
EABT12517	8	21	30	66	7	8	12
EABT12518	0	1	1	0	1	2	1
EABT12519	360.46	352.03	880.05	1817.84	258	133	159
EABT1252	1	3	1	2	0	0	0

EABT12520	7	4	0	2	1	0	0
EABT12521	0	1	3	1	0	1	0
EABT12522	0	0	7	1	0	0	0
EABT12523	2	3	1	4	0	0	0
EABT12524	21994.1	25993.7	51412.3	8778.67	8629.16	1829.17	2621.31
EABT12525	0	0	15	6	0	0	1
EABT12526	0	0	4	0	0	0	1
EABT12527	0	1	1	1	3	0	0
EABT12528	888	1078	944	1200	548	712	541
EABT12529	0	2	11	0	0	0	0
EABT1253	4	10	44	17	3	6	8
EABT12530	8	18	8	0	16	15	25
EABT12531	10	5	0	1	0	0	0
EABT12532	0	0	17	1	0	0	1
EABT12533	0	2	3	2	4	0	0
EABT12534	0	1	4	4	2	0	0
EABT12535	2	5	12	4	3	0	7
EABT12536	1	5	3	0	1	0	0
EABT12537	609	1879.72	2141.95	7117.6	2519.01	444.45	921
EABT12538	4248.07	5338.36	3043.56	4671.06	2146.25	4306.3	3827.54
EABT12539	34	63	85	194	40	1	14
EABT1254	31	45	55	119	32	15	17
EABT12540	37	75	70	66	10	44	19
EABT12541	0	0	22	2	0	0	0
EABT12542	1	5	32	8	0	4	2
EABT12543	0	0	2	1	0	3	2
EABT12544	1	3	4	8	9	0	13
EABT12545	3	1	45	15	3	0	1
EABT12546	6	2	29	1	2	0	0
EABT12547	1011.95	1908.96	2843.67	1557.84	341.97	1	19
EABT12548	6	6	91	52.01	357	0	3
EABT12549	8	8	19	2	12	2	5
EABT1255	0	4	10	5	2	2	0
EABT12550	0	5	5	0	0	2	0
EABT12551	4	12	40	5	5	4	4
EABT12552	4	8	7	0	2	18	2
EABT12553	0	5	4	0	3	0	0
EABT12554	0	0	1	6	3	0	0
EABT12555	1	7	15.2	17	8	1	2
EABT12556	0	4	2	5	0	2	1
EABT12557	0	1	8	0	0	0	0
EABT12558	636.5	984.15	586.73	541	367.03	887.51	1364.57
EABT12559	0	3	8	6	2	0	2
EABT1256	4	3	5	0	0	0	5
EABT12560	3	1	0	0	2	27	7
EABT12561	10	1	0	5	2	3	2
EABT12562	0	4	10	0	3	0	2
EABT12563	4	1	4	2	3	0	0
EABT12564	0	0	16	1	1	1	0
EABT12565	1	6	2	3	2	0	1
EABT12566	0	3	8	2	0	1	0

EABT12567	3	14	3	2	1	0	5
EABT12568	0	3	12	2	4	0	2
EABT12569	3	10	11	0	1	8	0
EABT1257	1	0	2	5	1	0	0
EABT12570	1	2	5	0	0	0	0
EABT12571	5	11	12	29.5	3	2	12
EABT12572	0	0	4	0	0	0	0
EABT12573	0	6	8	2	1	4	1
EABT12574	156	164.94	13	2	41.18	142.91	334.95
EABT12575	1	0	1	1	2	0	0
EABT12576	0	0	4	1	0	0	0
EABT12577	2781.29	5054.1	2156.87	6788.31	3853.98	1369.54	1503.22
EABT12578	3	3	0	0	2	2	12
EABT12579	2	0	8	1	0	0	0
EABT1258	0	7	5	1	3	2	0
EABT12580	3	4	0	0	0	0	0
EABT12581	1138.89	1090.03	502	1094	710	800	409.85
EABT12582	1239	1666.29	1112	1806.2	914.33	840	707
EABT12583	6	3	33	3	2	3	1
EABT12584	0	2	3	0	0	0	1
EABT12585	1	2	17	13	3	0	1
EABT12586	1	0	0	0	0	4	1
EABT12587	5	6	2	9	2	1	0
EABT12588	0	1	6	0	0	0	0
EABT12589	5	6	13	4	0	2	2
EABT1259	0	0	2	1	2	0	5
EABT12590	0	6.2	0	0	0	0	5
EABT12591	0	0	8	7	2	0	0
EABT12592	1	2	18	2	1	0	0
EABT12593	0	4	1	0	0	1	0
EABT12594	2	0	2	0	1	1	4
EABT12595	7845.01	8537.76	5713.61	5295.63	4109.33	3523.65	3093.13
EABT12596	671.99	5	30	5638.71	77	1	4
EABT12597	15	21	299	10	4	11	9
EABT12598	2	7	3	1	0	0	0
EABT12599	1	6	10	7	0	1	0
EABT126	1	1	42	0	0	1	0
EABT1260	2844.6	3321.7	7	2	328.98	418.96	319.56
EABT12600	775.85	1101.92	877.08	1706.59	752.23	590.13	578.88
EABT12601	4	2	5	0	1	0	0
EABT12602	32	15.14	48	141.97	1	1	2
EABT12603	1	2	21	111	5	3	4
EABT12604	2	25	13	13	156	0	294.73
EABT12605	1385.05	1512.31	1535.5	2020.96	1060.37	1102.81	1107.56
EABT12606	23688.9	3997.54	5600.86	1834.21	16.99	1	2
EABT12607	0	1	12	0	0	0	0
EABT12608	0	2	2	0	0	0	1
EABT12609	3	8	19	4	5	0	0
EABT1261	2	2	26	11	0	1	4
EABT12610	0	7	0	0	0	0	0
EABT12611	2	4	7	1	0	0	1

EABT12612	1	2	4	5	0	0	0
EABT12613	1	4	2	1	0	0	0
EABT12614	8	18	63	8	8	6	13
EABT12615	0	1	4	1	1	1	12
EABT12616	0	0	1	2	2	1	0
EABT12617	0	0	0	3	0	0	1
EABT12618	0	1	7	0	0	0	0
EABT12619	1	2	11	3	1	8	3
EABT1262	1	0	0	0	82	0	0
EABT12620	2543.82	4839.4	3950.72	8182.51	3150.81	2161.69	2464.17
EABT12621	74	148.72	112	77.01	125	12	118.01
EABT12622	438.09	574	587	1173.97	349	417	449
EABT12623	22885.5	25104.2	6322.83	2031.94	5051.61	3607.3	2849.62
EABT12624	0	1	1	13	0	0	0
EABT12625	3	6	1	4	1	3	0
EABT12626	2	4	26	8	1	2	0
EABT12627	0	1	4	0	0	0	0
EABT12628	6	4	4	17	1	1	0
EABT12629	7	8	16	20	19	1	21
EABT1263	1	5	9	0	0	0	1
EABT12630	4	7	5	8	8	2	2
EABT12631	31906.6	27383.2	31527.8	34548	9475	24666.1	18154
EABT12632	0	2	14	0	0	0	0
EABT12633	0	3	5	17	1	0	3
EABT12634	0	7	0	6	0	1	0
EABT12635	1	4	5	20	6.05	2	4
EABT12636	3476.4	6368.41	9093.12	10422.6	5636.27	5447.4	5855.04
EABT12637	601.97	1095.96	858.38	2081.64	792.02	406.27	419.94
EABT12638	0	0	1	2	9	0	1
EABT12639	0	0	14	1	0	0	0
EABT1264	2	5	2	0	0	5	0
EABT12640	4	9	22	26	13	11	0
EABT12641	17	80	72	652.64	20	2	3
EABT12642	0	1	0	0	0	0	0
EABT12643	1	6	14	0	0	0	0
EABT12644	3	1	3	0	0	0	0
EABT12645	1108.87	1323.65	622.28	1965.09	519.01	903	853
EABT12646	1	1	9	1	1	2	1
EABT12647	8	6	24	6	5	3	2
EABT12648	6	1	0	1	0	4	0
EABT12649	83	278	281.12	226	171.99	5	8
EABT1265	4	3	0	0	0	0	0
EABT12650	267	1044	898.72	1331.55	1197.54	276.66	352.37
EABT12651	3	12	4	9	4	3	4
EABT12652	0	2	5	0	0	0	0
EABT12653	0	1	1	0	5	0	0
EABT12654	1	0	7	2	0	1	0
EABT12655	6	1	15	1	0	0	0
EABT12656	5	21.16	27	75	123	1	5
EABT12657	0	0	5	1	0	0	0
EABT12658	0	1	1	1	0	0	0

EABT12659	2	5	5	12	2	2	1
EABT1266	0	4	58	37	40	0	0
EABT12660	308	564	607.17	1618.99	460	150	235
EABT12661	26300.1	43854.5	20377.3	26234.7	8578.45	31567.7	24598.2
EABT12662	0	2	7	1	24	0	0
EABT12663	0	0	10	0	0	0	0
EABT12664	0	0	7	0	0	0	0
EABT12665	1	0	2	1	1	0	0
EABT12666	0	2	25	7	0	0	0
EABT12667	0	0	0	2	2	0	0
EABT12668	0	1	2	0	2	0	2
EABT12669	4	2	14.92	9	3	1	0
EABT1267	1	0	0	2	2	1	3
EABT12670	0	2	4	6	1	0	0
EABT12671	0	0	3	1	0	0	0
EABT12672	0	0	10	0	0	0	0
EABT12673	4	1	0	0	0	3	2
EABT12674	0	0	1	5	0	0	0
EABT12675	0	0	7	4	2	2	6
EABT12676	1	2	5	0	0	0	0
EABT12677	0	0	0	0	0	0	49
EABT12678	4	0	2	2	0	0	0
EABT12679	2	4	17	8	0	1	5
EABT1268	1	1	5	6	0	0	0
EABT12680	1	0	9	0	1	0	0
EABT12681	0	0	5	1	1	0	0
EABT12682	0	1	10	1	0	0	0
EABT12683	8	9	41	27	6	6	20
EABT12684	0	4	11	2	0	1	0
EABT12685	1	0	6	0	0	0	0
EABT12686	4	14	8	12	3	10	8
EABT12687	729.88	1016.44	1184.15	2633.76	966.63	1159.7	957
EABT12688	4	19	60	19	6	6	2
EABT12689	2	0	6	9	7	1	12
EABT1269	75	214	350	15950.2	359	31	39
EABT12690	3	11	25	16	5	6	6
EABT12691	1	3	1	6	0	0	0
EABT12692	0	2	32	0	0	0	0
EABT12693	1	1	18	10	2	2	0
EABT12694	1	1	7	3	1	0	0
EABT12695	0	1	12	0	0	0	0
EABT12696	0	0	5	2	1	0	0
EABT12697	649.25	919.92	1033.01	1424	485	668	658.03
EABT12698	1627.74	2099.33	326.78	2562.81	1412.61	242.59	605.16
EABT12699	0	2	3	6	0	0	0
EABT127	3	0	9	4	0	0	0
EABT1270	0	0	15	3	1	0	0
EABT12700	0	2	3	3	2	0	1
EABT12701	52	120.99	150	2108.49	241	10	48
EABT12702	0	0	3	1	1	6	1
EABT12703	1	10	1	0	3	0	1

EABT12704	0	2	0	0	0	25	8
EABT12705	0	4	0	2	1	0	2
EABT12706	0	2	2	4	0	1	2
EABT12707	369.16	530.77	445.89	983.19	441.48	222	273.62
EABT12708	0	0	0	4	0	0	0
EABT12709	0	4	5	4	0	0	1
EABT1271	0	1	4	7	0	0	0
EABT12710	2	1	3	0	0	0	0
EABT12711	0	1	6	2	0	0	0
EABT12712	11	24	160.17	18	5	5	5
EABT12713	161	198.1	150.33	201.01	205	141	162
EABT12714	7156.57	10445.7	14737.8	14917.1	9219.25	7185.32	8790.94
EABT12715	0	2	25	0	1	0	0
EABT12716	1	1	0	2	0	0	0
EABT12717	3387.49	4449.08	9370.31	2884.97	1028.84	621.54	1470.87
EABT12718	3943.08	7349.78	11848.5	9586.83	4796.44	4638.43	4742.01
EABT12719	1	3	3	2	1	0	0
EABT1272	10	27	42	39	10	8	8
EABT12720	3	2	13	33	4	1	8
EABT12721	1	1	6	0	0	1	0
EABT12722	0	0	3	1	0	0	0
EABT12723	12	60	66	19	1	16	10
EABT12724	1	5	16	3	0	0	0
EABT12725	23	97	144	233	219	35	160.01
EABT12726	23	156	28	226	51	1	20
EABT12727	7	6	7	37	6	0	0
EABT12728	0	2	1	6	0	0	0
EABT12729	0	0	4	1	1	0	0
EABT1273	1	1	6	6	0	0	0
EABT12730	0	0	10	1	0	0	0
EABT12731	1	13	2	0	2	1	5
EABT12732	0	0	0	9	0	0	0
EABT12733	17	7	2	4	0	15	2
EABT12734	3	3	2	2	2	5	2
EABT12735	0	5	30	6	1	1	4
EABT12736	0	1	4	0	0	0	1
EABT12737	1	4	16	17	6	1	1
EABT12738	1	2	8	2	0	0	0
EABT12739	0	1	2	2	0	0	1
EABT1274	1	14	23	27	2	7	2
EABT12740	30	62.01	108.02	211.99	73.88	16.68	102.66
EABT12741	1626.25	2807.89	10295.3	8211.07	1291.99	2727.46	1852.57
EABT12742	34	53.23	40	54	20	9	11
EABT12743	0	1	3	0	2	0	2
EABT12744	13443.8	11451.5	2403.22	1146.37	9243.95	710.21	1381.94
EABT12745	2	2	8	5	0	1	0
EABT12746	26	26	39.89	6	3	13	8
EABT12747	512.06	933.51	381.21	1248.97	500.16	382.17	567.42
EABT12748	0	0	2	1	0	0	3
EABT12749	3366.01	4170.28	2643.35	5780.35	2942.74	2703.83	2063.47
EABT1275	3	18	25	18	3	3	5

EABT12750	65898.5	50046.6	23082.6	24943.2	23439.3	31355	34202.5
EABT12751	2	7	28	0	1	1	1
EABT12752	3	7	0	1	1	1	0
EABT12753	53	48.01	20	11	4	2	16
EABT12754	4	11	6	20	3	6	2
EABT12755	4	2	15	1	1	4	2
EABT12756	278.06	7	2	2	13	24	240.06
EABT12757	0	2	17.02	2	1	0	0
EABT12758	0	3	5	5	4	0	0
EABT12759	2871.22	3289.8	3880.42	4618.98	1943.48	2271	1207
EABT1276	2	5	19	8.83	3	3	0
EABT12760	0	0	5	0	3	0	0
EABT12761	1	5	3	89	5	1	1
EABT12762	0	2	0	3	1	0	0
EABT12763	2	15	2	2	24	22	43
EABT12764	1	1	131	82.52	0	0	0
EABT12765	0	0	4	1	1	0	1
EABT12766	1	3	6	9	1	0	5
EABT12767	0	3	4	0	0	0	0
EABT12768	6239.11	1920.44	6	25	3	5202.1	6
EABT12769	0	0	2	0	2	0	0
EABT1277	0	0	0	0	0	0	0
EABT12770	3	8	10	1	1	4	0
EABT12771	0	1	4	1	1	2	0
EABT12772	1	4	1	14	1	0	2
EABT12773	1	2	7	2	0	0	0
EABT12774	0	2	26	1	2	0	0
EABT12775	0	1	34	3	0	0	0
EABT12776	0	1	16	2	0	1	0
EABT12777	0	2	3	3	4	1	1
EABT12778	0	0	1	0	0	0	0
EABT12779	1	0	2	3	1	0	0
EABT1278	0	3	9	10	1	1	3
EABT12780	1	1	5	2	1	1	0
EABT12781	0	0	2	6	0	0	0
EABT12782	0	0	5	0	0	1	0
EABT12783	2542.92	2495.96	757.66	1573.12	1700.13	2241.73	1474.82
EABT12784	1	1	2	22	0	0	1
EABT12785	1	2	3	4	0	0	1
EABT12786	1	8	0	12	6	0	4
EABT12787	0	1	7	0	0	0	0
EABT12788	1	0	30	4	0	0	0
EABT12789	0	1	2	0	1	0	0
EABT1279	1	3	22	0	3	1	1
EABT12790	144	301	340.23	0	912.2	0	5
EABT12791	2	5	2	4	3	0	0
EABT12792	2	1	13	0	0	0	0
EABT12793	0	0	11	0	0	0	1
EABT12794	1	9	12	0	1	1	6
EABT12795	2	9	22	0	1	0	1
EABT12796	0	0	4	0	0	0	0

EABT12797	667	785	512	1445.17	413	443.52	380.3
EABT12798	1	2	8	0	1	0	0
EABT12799	0	2	37	13	1	0	1
EABT128	0	5	4	0	1	0	0
EABT1280	1	1	2	3	0	1	0
EABT12800	2724.99	2907.09	2143.19	974	1221.84	66	122.99
EABT12801	0	0	8	1	1	0	0
EABT12802	2	7	2	4	0	2	2
EABT12803	4	6	9	7	1	1	4
EABT12804	547	614	818	979.91	533.38	560	519
EABT12805	2	5	87	2	3	16	1
EABT12806	0	0	4	1	0	0	1
EABT12807	0	4	10	1	0	0	0
EABT12808	11	41	97.95	323	103	3	16
EABT12809	1	2	17	53.09	77	1	43
EABT1281	0	0	5	1	0	0	0
EABT12810	6583.73	6932.09	4493.59	5371.7	4625.51	9239.31	6749.85
EABT12811	1	1	1	5	1	1	1
EABT12812	9	27	2	1	0	0	1
EABT12813	1	4	14	1	19	1	0
EABT12814	55	59	0	0	0	2	6
EABT12815	25	126.99	185	203.99	28.04	24	40
EABT12816	3	6	14	0	0	1	1
EABT12817	3	1	0	0	0	2	1
EABT12818	0	4	31	6	1	0	0
EABT12819	1634.33	2767.04	1138	4183.88	1858.06	1327	1910.07
EABT1282	0	0	19	0	0	0	0
EABT12820	474	1185.98	8	133	8	1817	110
EABT12821	1	0	5	8	0	0	0
EABT12822	334.74	407.5	265	295.99	387.57	284.18	295
EABT12823	2	6	3	2	0	1	1
EABT12824	0	1	5	2	2	0	0
EABT12825	7	10	24	7	0	2	0
EABT12826	0	2	5	2	0	1	0
EABT12827	1	2	6	0	0	0	0
EABT12828	1	65.99	18	2	1	52	13.53
EABT12829	0	6	76	1	1	0	0
EABT1283	10674	6092.83	3299.3	2464.49	2912.09	20058.7	9924.8
EABT12830	0	0	4	0	0	0	1
EABT12831	2	1	0	1	0	0	0
EABT12832	0	1	0	0	1	1	5
EABT12833	4287.08	8405.16	5015.06	7431.86	4873.48	2974.08	4390.52
EABT12834	0	0	4	2	0	0	0
EABT12835	0	1	1	1	2	0	0
EABT12836	155.53	265	369.29	285	470	131	248.95
EABT12837	3127.05	4261.92	2000.99	4180.99	2523	1699.01	2391.83
EABT12838	195.53	307.61	288	837.23	474.1	243.7	259
EABT12839	2	1	3	7	0	0	3
EABT1284	2	1	10	13	2	3	2
EABT12840	4	5	29	9	1	0	5
EABT12841	2	2	2	0	0	0	0

EABT12842	11	4	0	0	0	0	3
EABT12843	2206.11	3611.21	1947.99	361.99	1509.74	770	1534.99
EABT12844	1	7	29	4	0	0	0
EABT12845	46.99	45	53	4	13	32	40
EABT12846	4490.8	5281.66	3124.3	3929.4	3820.48	2413.45	3108.16
EABT12847	34	53	72	137	76	8	18
EABT12848	694.81	1053.46	1349.01	1784.99	891.97	297.98	767.6
EABT12849	5	22	7	16	11	0	1
EABT1285	0	0	4	5	0	3	3
EABT12850	0	1	0	1	0	3	8
EABT12851	0	0	25	2	0	0	0
EABT12852	5	0	1	4	0	0	0
EABT12853	1789.73	2801.94	2547.23	3501.53	3201.81	2152.04	1627.98
EABT12854	4	3	1	0	0	15	0
EABT12855	3	3	2	0	0	9	5
EABT12856	2	7	110	6	1	3	6
EABT12857	1	2	32	5	0	2	0
EABT12858	128.95	446	314.84	676	278	200	290.78
EABT12859	420	780.16	842.88	2138.49	409	264.53	279.43
EABT1286	20	53	39	18	2	3	0
EABT12860	0	1	5	4	2	0	1
EABT12861	27	30	3	3	3	0	3
EABT12862	2	0	2	0	0	4	6
EABT12863	12	3	0	0	0	1	0
EABT12864	0	2	5	0	0	2	0
EABT12865	0	4	2	0	0	0	1
EABT12866	2	3	4	1	2	4	2
EABT12867	0	2	3	8	1	0	1
EABT12868	3	0	0	0	0	7	0
EABT12869	456	890	2143.14	7932	1273	204	928
EABT1287	0	1	1	7	18	0	1
EABT12870	1	5	15	1	1	1	0
EABT12871	0	2	0	4	4	0	3
EABT12872	10	0	0	0	1	31	0
EABT12873	0	3	29	7	21	0	0
EABT12874	2004.33	4594.49	4744.38	3430.88	3885.74	915.32	1377.46
EABT12875	1	1	0	6	0	0	0
EABT12876	0	0	1	0	6	0	0
EABT12877	2	2	12	2	1	0	0
EABT12878	1	56.52	71	1	3	3	11
EABT12879	3	4	26	4	4	10	4
EABT1288	0	4	1	3	1	2	3
EABT12880	0	1	9	0	0	0	1
EABT12881	974.04	1153.02	3178.53	1800.75	604.24	567.99	292
EABT12882	1132	1579	1666.89	2172	918	1009	968.96
EABT12883	13	11	4	1	2	0	1
EABT12884	0	2	13	3	0	0	0
EABT12885	0	6	3	1	0	2	6
EABT12886	0	1	2	5	0	0	1
EABT12887	0	0	0	4	1	0	1
EABT12888	32	159	348	568	439	21	97.2

EABT12889	0	4	1	1	0	0	0
EABT1289	1	0	5	0	2	0	0
EABT12890	3	2	1	7	11	2	5
EABT12891	1	0	4	1	0	0	0
EABT12892	20	13.01	3	2	9	5	11
EABT12893	1	0	15	2	1	0	7
EABT12894	2218.81	2575.51	2417.95	2830.56	2440.75	2381.85	2002.17
EABT12895	0	1	2	5	0	0	1
EABT12896	437	532.93	268	661.92	432	257	223
EABT12897	1	2	4	0	0	0	0
EABT12898	0	1	1	58	0	0	0
EABT12899	0	0	10	0	0	1	1
EABT129	4	6	2	1	3	2	3
EABT1290	3	3	11	1	1	0	0
EABT12900	6	7	21	13	2	3	0
EABT12901	0	3	0	0	0	6	0
EABT12902	0	0	8	0	0	3	1
EABT12903	1	1	22	4.11	0	0	0
EABT12904	10	26	44	60	13	7	4
EABT12905	1409.27	2507.75	1092.99	2312.5	1255.33	967.96	1263.11
EABT12906	1	5	6	13	3	0	1
EABT12907	0	0	1	0	0	0	2
EABT12908	3	11	4	3	0	0	0
EABT12909	0	2	0	0	0	0	0
EABT1291	1	1	12	1	3	0	6
EABT12910	0	0	9	0	0	0	0
EABT12911	4	0	0	0	1	11	1
EABT12912	2	0	3	0	0	0	0
EABT12913	2490.83	4457.51	5287.89	6301.74	1519.97	2508.24	1870.94
EABT12914	83	346.19	227	193	48	54	8
EABT12915	316	390	160	588	278.48	210	193
EABT12916	7	9	10	1	1	3	7.99
EABT12917	43	29	32	23	13	14	17
EABT12918	9797.43	8216.14	4763.13	2444	3257.44	12327.5	5210.8
EABT12919	2168.66	13409.7	7012.73	12667.4	25856.5	538.91	3724.25
EABT1292	1	13	2	7	1	0	0
EABT12920	0	1	3	5	1	0	1
EABT12921	1834.14	2163.12	1595.82	3605.47	1533.46	1443.26	1339
EABT12922	15	5	6	12	10	17	25
EABT12923	304	1136.99	489.49	894.99	626.02	757.98	1406.09
EABT12924	0	0	1	0	33	0	0
EABT12925	0	0	2	2	5	0	1
EABT12926	499	569.59	455.48	801.22	465.04	365	269.32
EABT12927	3590.96	10101	2398	399	3540.91	159	3019.93
EABT12928	115.05	202	228.9	1519.04	1167.73	46	47
EABT12929	0	0	0	0	0	0	0
EABT1293	20	38.82	33	91	17	15	34
EABT12930	0	1	6	0	0	3	0
EABT12931	2	2	7	3	1	0	1
EABT12932	0	4	127	3	1	1	0
EABT12933	2	5	3	1	2	0	1

EABT12934	0	0	11	0	0	0	0
EABT12935	27	75.9	320.84	228.32	385.12	23	449.73
EABT12936	4	4	0	0	0	14	3
EABT12937	1	2	4	5	6	13	0
EABT12938	8	12	11	14	5	9	12
EABT12939	1	2	0	0	0	1	2
EABT1294	0	0	21	0	0	0	0
EABT12940	2898.97	3516.23	2328.2	2851.63	2082.82	1825	1848.99
EABT12941	4	1	0	0	0	2	6
EABT12942	1871.43	516.95	42	772.13	1821.33	3	146.58
EABT12943	6	6	10	4	3	6	2
EABT12944	0	2	0	0	3	0	9
EABT12945	1	2	13	17	6	0	0
EABT12946	1	6	1	15	2	0	1
EABT12947	2070.92	89	0	0	4	1047.87	9.34
EABT12948	31.28	67.8	33	50	11	24	26.14
EABT12949	3	4	84	0	0	4	1
EABT1295	3838.99	3089.56	3187.15	27543.5	2656.03	725	1232.01
EABT12950	4	15	5	13	8	7	33
EABT12951	1	2	3	2	0	0	1
EABT12952	2	0	23	6	0	1	0
EABT12953	0	4	1	0	1	0	1
EABT12954	0	3	1	2	0	1	0
EABT12955	0	5	18	90.1	16	1	4
EABT12956	0	0	1	1	0	0	1
EABT12957	6	16	87	14	5	7	7
EABT12958	1	0	1	3	0	1	0
EABT12959	2	3	5	8	2	4	2
EABT1296	29	89.01	469	32	42.02	20	29
EABT12960	1	10	10	18	1	0	1
EABT12961	216	184	74	858.04	58	2	100
EABT12962	0	4	4	6	0	1	1
EABT12963	3569.04	6167.56	9710.69	9539.57	5105.43	1614.91	2303.03
EABT12964	1	2	0	1	1	0	0
EABT12965	14	7	9	6	5	1	2
EABT12966	0	1	8	12	0	0	1
EABT12967	5	17	23	17	4	6	1
EABT12968	2	0	4	3	14	0	0
EABT12969	385	1688.91	643.7	156.93	470.02	230.97	245.61
EABT1297	8	23	41.07	49.99	13	10	17
EABT12970	0	3	2	0	3	2	3
EABT12971	0	4	5	3	2	1	0
EABT12972	40	223	509.02	995.96	234	3	63
EABT12973	1	7	5	3	0	0	0
EABT12974	703.78	912.22	714	1645	805	490	527
EABT12975	2	1	6	0	0	1	1
EABT12976	1	5	19	7	0	0	0
EABT12977	2	5	0	3	0	0	0
EABT12978	3	19	3	2	6	0	0
EABT12979	7	6	15	18	13	1	3
EABT1298	6	1	2	0	1	4	0

EABT12980	0	2	0	0	0	1	5
EABT12981	2	1	7	0	1	0	0
EABT12982	7	4	5	0	0	1	1
EABT12983	0	0	13	4	0	0	0
EABT12984	0	7	8.93	2	0	0	0
EABT12985	1	0	19	0	0	0	0
EABT12986	2937.01	4878.83	4602.37	7274.15	2171.88	3893.06	3956.02
EABT12987	182	282.3	207.12	541.21	255.81	93.98	230.89
EABT12988	0	0	62	0	0	0	0
EABT12989	3	1	5	1	6	3	13.01
EABT1299	0	0	13	23	1	0	1
EABT12990	0	1	8	1	0	1	3
EABT12991	0	0	1	2	1	0	0
EABT12992	1868.76	2793.12	2622.63	5129.5	1520.56	1489.38	1148.99
EABT12993	784.82	1074.24	1270.1	1442	1110	466	666.91
EABT12994	403	624.99	552.82	1080.66	377.82	357.41	406.79
EABT12995	8	9	23	5	1	1	0
EABT12996	2	8	3	2	2	0	2
EABT12997	199	391	2532.57	205	848	23	180
EABT12998	65	208	211	328	242.78	15	54
EABT12999	5	10	0	0	9	1	13
EABT13	2	7	6	3	4	0	15
EABT130	13	28	3	1	1	0	0
EABT1300	0	1	5	0	0	0	0
EABT13000	12	31.04	103	10.99	16	10	80.06
EABT13001	1875.03	5608.6	5139.09	8313.02	3777.27	1297.09	2644.68
EABT13002	0	0	10	0	0	0	0
EABT13003	3	2	12	1	14	0	0
EABT13004	0	5	0	4	3	1	1
EABT13005	1	15	2	70.07	3854.92	1	14
EABT13006	0	0	1	1	0	0	0
EABT13007	0	0	12	1	0	0	3
EABT13008	2	1	7	11	1	0	1
EABT13009	24	72	20	59	18	5	19
EABT1301	2	10	17	25	2	5	6
EABT13010	695.99	1207.02	880.15	1203.98	999	276	371
EABT13011	0	0	0	2	2	0	0
EABT13012	3426.6	3497.19	3514.84	1352.04	1894.23	2106.26	2923.72
EABT13013	11	13	29	21	3	6	4
EABT13014	2	3	15	3	2	3	1
EABT13015	0	1	2	1	1	0	1
EABT13016	1	2	20	0	1	2	0
EABT13017	1	1	20	0	1	0	0
EABT13018	58	229	606	3461	126	13	175
EABT13019	1	5	1	22	2	1	6
EABT1302	1	1	5	1	1	0	2
EABT13020	0	0	2	7	0	1	0
EABT13021	0	3	3	4	1	0	0
EABT13022	1	2	2	6	22	0	0
EABT13023	6	9	3	0	3	7	2
EABT13024	2	5	5	1	1	0	1

EABT13025	0	0	19	3	0	0	0
EABT13026	0	0	2	33	0	0	1
EABT13027	1	0	2	0	3	1	0
EABT13028	0	8.49	10	8	2	0	1
EABT13029	5	1	0	0	0	1	3
EABT1303	0	0	7	0	0	1	0
EABT13030	0	4	0	0	0	0	0
EABT13031	0	2	7	0	2	3	1
EABT13032	3	4	3	2	3	0	0
EABT13033	1	5	106	12	2	8	1
EABT13034	0	3	3	0	0	4	2
EABT13035	1	4	8	4	1	1	1
EABT13036	3	6	6	9	9	1	7
EABT13037	0	0	3	0	0	0	0
EABT13038	3	4	18	5	1	2	2
EABT13039	0	1	1	4	0	0	0
EABT1304	1	4	25	5	2	0	2
EABT13040	26	30	89	52	8	14	22
EABT13041	457.99	569.68	306.56	691.4	501.71	365.55	301.91
EABT13042	2	2	5	5	0	2	6
EABT13043	1	2	0	0	1	0	2
EABT13044	0	0	4	4	0	0	0
EABT13045	1	7	50	174	12	0	0
EABT13046	0	0	0	7	0	0	0
EABT13047	1219	1454.89	2380.43	2233.01	1413.45	621.01	651
EABT13048	2179.02	3601.15	4087.22	19304.7	5335.16	519	855
EABT13049	90	124	17	51	40	25	16
EABT1305	0	0	4	4	1	0	0
EABT13050	11	11	121	1022.81	15	5	64
EABT13051	242.08	371.4	229.8	1161.68	303.97	161.96	230.93
EABT13052	3	4	3	15	6	2	1
EABT13053	0	13	12	0	16	68	1780.97
EABT13054	50	121.4	178	152.96	58.02	78	53
EABT13055	1	0	9	19	20	0	1
EABT13056	4	3	83	18	9	1	3
EABT13057	2	2	7	3	2	2	0
EABT13058	0	1	0	12	0	0	0
EABT13059	265.1	493	724	3152.18	727	74	72
EABT1306	0	0	8	5	2	0	1
EABT13060	0	0	9	2	0	0	0
EABT13061	1	2	1	1	27	2	1
EABT13062	0	0	22	0	0	0	0
EABT13063	2	0	1	5	0	0	2
EABT13064	0	0	2	0	4	0	2.97
EABT13065	5	8	37	18	2	12.77	8
EABT13066	0	0	12	0	0	0	0
EABT13067	1677.89	1027.62	366.95	79.16	802.44	1928.34	3774.31
EABT13068	1	1	4	2	0	0	0
EABT13069	0	7	0	4	10	1	2
EABT1307	1	5	4	0	0	7	1
EABT13070	1275.09	1901.11	2617.9	3808.92	2736.85	1275.9	1664

EABT13071	0	3	2	6	0	0	1
EABT13072	0	2	8	4	1	0	2
EABT13073	1	3	4	2	1	0	0
EABT13074	1	3	7	21	0	0	2
EABT13075	1	0	1	0	2	1	0
EABT13076	1	0	8	0	0	0	0
EABT13077	0	7	29	7	1	9	2
EABT13078	3	2	12	2	1	0	0
EABT13079	1	3	2	0	1	0	0
EABT1308	0	0	8	2	0	0	0
EABT13080	56	85	238.95	17	84	8	12
EABT13081	5	1	42	1	0	5	0
EABT13082	72.57	161	166	471.56	214.02	21	91
EABT13083	1	1	5	1	4	2	4
EABT13084	5	13	18	53	5	8	10
EABT13085	0	1	0	3	0	3	0
EABT13086	507.63	996.64	2965.07	14331.4	2636.09	52	358.6
EABT13087	0	3	7	0	0	1	0
EABT13088	2	4	6	4	2	6	3
EABT13089	0	0	16	4	0	1	0
EABT1309	14	17	16	14	10	6	40.8
EABT13090	0	0	25	0	0	0	0
EABT13091	0	2	7	1	0	1	2
EABT13092	1	0	2	2	0	0	0
EABT13093	3	7	6	2	1	1	0
EABT13094	3	7	45	2	2	3	5
EABT13095	0	2	18.94	31	5	0	0
EABT13096	1	0	11	9	0	0	2
EABT13097	28	71	96	68	10	17	25
EABT13098	0	1	45	0	0	0	0
EABT13099	0	2	2	1	2	0	0
EABT131	0	0	0	0	0	0	6
EABT1310	0	1	4	0	0	0	0
EABT13100	0	0	6	0	0	0	0
EABT13101	0	0	15	0	0	0	0
EABT13102	12563.4	10409.1	4852.36	5718.3	15867.6	5913.47	5612.78
EABT13103	3	11	10	1	0	2	4
EABT13104	1576.98	3846.16	4696.82	9222.64	3374.99	255	821.62
EABT13105	6348.42	13274.1	28716.5	17952.3	9587.75	5635.09	5535.01
EABT13106	0	4	27	1	0	0	1
EABT13107	0	0	1	0	7	0	1
EABT13108	1451.96	1890.01	1746.99	2022.96	1542.01	2760.98	1910.08
EABT13109	1	0	8	3	2	0	1
EABT1311	6	5	2	7	3	0	0
EABT13110	0	2	7	2	0	1	0
EABT13111	1	1	6	3	1	0	0
EABT13112	2303.17	4033.58	3146.95	7975.73	1994.85	87.02	455.04
EABT13113	10	24	6	12	1	8.01	2
EABT13114	0	1	19	4	1	0	1
EABT13115	115	259	275	653.72	258.09	101	160
EABT13116	3	7	72	2	0	2	1

EABT13117	1	0	9	3	0	0	1
EABT13118	3	0	4	1	0	1	0
EABT13119	4	2	45	0	0	0	0
EABT1312	0	0	2	3	0	0	1
EABT13120	0	0	3	2	1	0	0
EABT13121	232	298	358.99	1390.73	798.08	312.12	759
EABT13122	29	81	122	1798.21	2230.01	2	25
EABT13123	1	0	23	2	0	1	0
EABT13124	329.03	489.98	609.57	514.94	691.58	330.41	365.12
EABT13125	29	53.63	119	6	10	49	17
EABT13126	2	5	0	11	0	1	0
EABT13127	540	496.01	310.15	549.99	464	186.99	258
EABT13128	0	0	11	0	0	1	0
EABT13129	2	2	8.31	6	1	4	2
EABT1313	4	0	1	0	0	9	0
EABT13130	0	3	3	11	164	2	2
EABT13131	33	76	42	106	20	24	26
EABT13132	1	9	12	9	1	2	1
EABT13133	1201.12	384.83	2	2	29	313.28	60
EABT13134	0	1	8	2	2	0	0
EABT13135	0	0	2	3	0	0	1
EABT13136	1	5	3	26	0	3	5
EABT13137	273	405.99	492.16	790.12	462.62	181.7	196
EABT13138	0	3	3	3	0	0	0
EABT13139	4	5	24	1	0	3	0
EABT1314	1	1	0	0	1	4	5
EABT13140	0	0	8	0	0	0	0
EABT13141	133	780.95	3092.39	4610.26	531	28	100.89
EABT13142	0	2	1	2	5	1	1
EABT13143	0	2	1	1	0	0	0
EABT13144	0	0	2	5	3	0	5
EABT13145	0	2	2	0	0	0	1
EABT13146	1	0	4	1	0	1	0
EABT13147	0	0	4	1	2	0	1
EABT13148	9	37	68	499.99	82	4	21
EABT13149	3576.15	4584.98	4716.17	6598.86	2088.99	4151	2147.67
EABT1315	0	1	74	0	0	0	0
EABT13150	1	5	6	6	1	0	1
EABT13151	0	0	4	0	0	2	0
EABT13152	0	2	5	1	1	0	0
EABT13153	5	4	4	3	4	2	1
EABT13154	2	3	1	4	10	0	0
EABT13155	1	14	0	62	1	1	3
EABT13156	0	0	3	1	0	0	0
EABT13157	0	0	16	0	1	1	0
EABT13158	0	0	1	0	1	0	4
EABT13159	4866.34	6509.04	7970.14	16939.1	4550.44	5139.3	5167.13
EABT1316	6	9.58	8	94	10	4	12
EABT13160	20	31	51	87	108	9	15.7
EABT13161	423	491.8	33	68	151.89	9	89.47
EABT13162	1	0	5	0	0	0	1

EABT13163	1	0	8	0	1	0	0
EABT13164	3	5	5	1	1	0	0
EABT13165	7097.58	11207.1	8059.22	18610.4	5869.81	6299.49	6583.41
EABT13166	0	3	6	1	1	0	0
EABT13167	101.96	234.87	138	147	108.01	79	90
EABT13168	954.55	1395	1411	2553	986.57	736	648.17
EABT13169	1	4	9	1	1	0	0
EABT1317	0	10	52	5	0	4	1
EABT13170	0	3	6	0	0	0	0
EABT13171	0	0	0	0	0	5	3
EABT13172	42	48	33	26	33	28	41
EABT13173	0	0	9	2	0	0	0
EABT13174	2	4	8	2	1	0	0
EABT13175	0	11	0	0	0	1	0
EABT13176	1412.02	2017.01	2170.07	3038.62	2115.98	1453	1123.45
EABT13177	2782.93	3400.44	3823.9	4444.13	1715	3853.01	3691.02
EABT13178	0	0	0	0	7	0	8
EABT13179	0	0	0	0	2	1	3
EABT1318	4	0	1	0	0	0	0
EABT13180	1	0	5	3	2	1	0
EABT13181	9	49.45	33	73	17	15	19
EABT13182	0	1	0	0	0	1	3
EABT13183	9	11	17	40	1	3	3
EABT13184	0	0	3	3	2	0	1
EABT13185	0	0	0	2	8	0	0
EABT13186	0	0	1	18	3	0	1
EABT13187	1	4	14	1	3	0	1
EABT13188	0	3	58	0	0	0	0
EABT13189	2	6	17	12	0	4	1
EABT1319	0	0	1	39	2	0	0
EABT13190	1	0	7	0	0	0	0
EABT13191	1008	1266.1	709.35	1150.06	484.23	677.27	362
EABT13192	0	2	3	0	0	2	0
EABT13193	2	9	24	2	9	1	15
EABT13194	1	1	4	0	1	0	1
EABT13195	2	3	1	4	0	2	0
EABT13196	48	73	8	91	110	31	105
EABT13197	0	11	8	39	12	0	2
EABT13198	0	0	7	1	0	0	0
EABT13199	1	2	5	23	13	1	2
EABT132	0	4	16	8	2	0	1
EABT1320	2	27	41	38	3	20	2
EABT13200	415.05	519.99	1024.21	1437.5	526.8	507.18	568
EABT13201	606.03	658.87	533.54	1099.54	435.82	595.8	771.18
EABT13202	1	13	34	28	19	1	16
EABT13203	0	1	6	0	1	0	1
EABT13204	0	1	3	0	0	1	0
EABT13205	1	1	2	3	2	0	1
EABT13206	11	8	2	0	0	0	1
EABT13207	12	9	0	0	5	30	26
EABT13208	2	2	10	0	0	0	0

EABT13209	13	25	20	0	7	43.92	37
EABT1321	0	0	3	2	0	0	1
EABT13210	3	4	1	1	0	0	0
EABT13211	5	5	3	43	25	0	1
EABT13212	0	4	2	0	0	0	0
EABT13213	1767.62	2260.75	642.9	639.83	267.64	2813.59	733
EABT13214	0	0	15	0	2	0	0
EABT13215	453.88	584.99	590.24	1073.93	593.49	248.98	324.1
EABT13216	2243.99	4410.09	3586.35	10267.2	2455.67	2079.31	1990.9
EABT13217	2	12	11	17	1	2	0
EABT13218	0	5	5.03	0	0	2	0
EABT13219	1	1	3	0	0	0	1
EABT1322	1	2	1	0	0	0	0
EABT13220	130.98	183.91	119	644	331	65	133
EABT13221	0	3	7	0	1	1	0
EABT13222	0	0	2	0	0	0	0
EABT13223	2	17	2	10	0	1	1
EABT13224	0	0	5	0	0	0	0
EABT13225	8811.46	13958.5	4850.67	13212.7	38032.6	9893.96	10775.3
EABT13226	3	3	13	8	0	2	4
EABT13227	0	0	8	2	0	1	0
EABT13228	25	4	0	1	0	55	0
EABT13229	1	1	5	2	1	1	1
EABT1323	0	0	22	3	0	1	2
EABT13230	1	0	3	3	0	0	0
EABT13231	87	139	180	143	189	97	62
EABT13232	1323.01	1475.58	1599.09	2755.29	1185	949	760
EABT13233	12	21	3	0	4	24	22
EABT13234	2	0	2	3	0	1	0
EABT13235	8	29	46	41	8	3	6
EABT13236	3	0	1	0	1	0	2
EABT13237	2	0	3	5	0	0	1
EABT13238	0	0	11	2	1	0	1
EABT13239	11	27	33	16	3	1	4
EABT1324	5	21	3	16	14	5	9
EABT13240	0	1	2	3	1	0	3
EABT13241	1	1	8	3	0	4	2
EABT13242	870	1076	1119	1987	682	712	636
EABT13243	0	0	1	4	3	0	0
EABT13244	0	0	5.99	2	0	0	0
EABT13245	201.18	141.06	4143.24	256	114.15	113	85.85
EABT13246	2814.71	3863.33	2939.61	3941.75	1366.98	2768.21	2436.57
EABT13247	1	1	3	1	0	1	0
EABT13248	3	1	1	0	0	0	0
EABT13249	1	0	0	0	2	0	2
EABT1325	0	0	3	3	1	1	0
EABT13250	7995.97	14305.3	3634.09	5210.52	1578.64	247.83	419.54
EABT13251	1	0	1	5	0	0	2
EABT13252	0	1	1	1	1	0	1
EABT13253	2	0	6	1	0	0	0
EABT13254	0	0	2	5	0	0	0

EABT13255	1	1	3	0	2	0	1
EABT13256	2	0	9	7	11	2	28
EABT13257	0	0	2	1	0	4	0
EABT13258	4988.36	9059.44	6439.43	10009.3	3786.3	2533.46	2695
EABT13259	2	2	84	2	2	1	5
EABT1326	0	4	7	0	2	1	3
EABT13260	6	5	7	11	12	2	0
EABT13261	0	1	2	2	0	0	0
EABT13262	5	12	3	2	5	5	1
EABT13263	0	1	1	8	1	3	1
EABT13264	3	0	1	1	0	0	0
EABT13265	0	3	12	2	0	1	1
EABT13266	0	1	7	0	1	0	0
EABT13267	12	13	140	1	1	10	2
EABT13268	20	25	0	0	4	63	46
EABT13269	0	0	5	0	0	0	0
EABT1327	0	0	5	5	1	4	4
EABT13270	1	6	32	3	1	1	0
EABT13271	8	19	87	3	1	21	9
EABT13272	1	3	3	10	9	0	3
EABT13273	1	0	1	0	1	0	0
EABT13274	0	0	3	3	1	0	1
EABT13275	0	1	1	0	0	5	0
EABT13276	2	0	0	5	0	1	0
EABT13277	6	9	9	15	1	0	3
EABT13278	1593.87	2321.2	2129.84	2429.21	2011.38	1381.06	2017.09
EABT13279	10890.4	11132.3	4765.94	676.51	1605.44	1939.95	1585.28
EABT1328	2	21	2	7	3	2	6
EABT13280	1	0	4	1	0	0	1
EABT13281	0	4	4	4	0	0	0
EABT13282	0	0	13	2	0	0	0
EABT13283	0	1	0	3	1	0	0
EABT13284	2	0	6	0	0	0	0
EABT13285	2472.8	1532	145	25	989.93	1156.01	2983.47
EABT13286	195	304.09	272.97	21	55	42.83	78
EABT13287	0	1	4	9	4	0	0
EABT13288	238.52	362.75	361.32	662.41	443.76	182	289.35
EABT13289	0	0	23	2	0	0	0
EABT1329	8	8	44	39	17	2	4
EABT13290	4	10	5	0	4	0	1
EABT13291	38	213.12	209	297	16	7	9
EABT13292	2	0	3	8	0	0	1
EABT13293	4	12	0	1	0	9	0
EABT13294	0	0	0	0	0	0	1
EABT13295	0	1	47	1	0	1	1
EABT13296	0	1	5	0	0	0	0
EABT13297	20	2	0	1	10	52	263
EABT13298	18	58	118	158	8	15	17.08
EABT13299	0	0	23	2	0	4	3
EABT133	4	4	0	1	14	0	1
EABT1330	271.36	1897.06	477	457.83	3491.77	87	455

EABT13300	0	0	0	6.99	1	0	8
EABT13301	0	0	1	1	0	0	0
EABT13302	0	0	15	2	2	0	1
EABT13303	0	5	26.67	14	0	0	0
EABT13304	0	4	2	28	3	0	0
EABT13305	0	1	11	0	0	0	0
EABT13306	0	0	7	2	0	0	2
EABT13307	6	13	31.39	3	9	2	5
EABT13308	7202	10416	10714.9	14737.2	4033.01	7003.71	4135.99
EABT13309	0	4	8	2	0	0	0
EABT1331	1	1	9	0	0	1	0
EABT13310	1	0	12	1	0	1	1
EABT13311	0	4	13	0	1	2	1
EABT13312	5	2	1	1	0	3	0
EABT13313	9	5	25	49	4	3	5
EABT13314	5	40	16	11	17	7	2
EABT13315	1	0	16	2	0	0	0
EABT13316	0	0	0	6	0	0	1
EABT13317	179.98	474.33	731.05	972.02	501.46	166	145
EABT13318	0	0	8	0	0	0	0
EABT13319	0	1	3	0	0	0	1
EABT1332	0	0	9	0	0	0	0
EABT13320	0	1	6	1	2	2	0
EABT13321	1	7	5	3	1	3	3
EABT13322	105	177	35	73	63	14	161
EABT13323	610.99	748	1096.53	2000.01	1496	232	591
EABT13324	0	11	3	1	371	0	1
EABT13325	6	14	39	168	0	0	0
EABT13326	1	8	8	5	2	0	3
EABT13327	8	20	28	10	0	4	5
EABT13328	17	9	4	17.16	13	3	8
EABT13329	1062	1280	773	892	2608.59	679	770.6
EABT1333	1	3	3	0	1	1	0
EABT13330	0	10	18	0	0	0	0
EABT13331	2405.61	3801.82	2820.61	6960.19	5030.11	2162.83	3336.93
EABT13332	0	1	2	8	1	5	2
EABT13333	1	10	19	2	7	2	2
EABT13334	2	2	1	0	1	1	0
EABT13335	2	0	6	2	1	1	2
EABT13336	0	3	47	0	0	0	0
EABT13337	2	12	14	10	3	5	4
EABT13338	9	33.01	20	44	0	8	2
EABT13339	1	2	1	11	0	0	0
EABT1334	0	3	19	2	0	1	0
EABT13340	1	4	6	2	0	1	0
EABT13341	0	1	0	3	1	0	3
EABT13342	3	4	3	0	0	3	5
EABT13343	0	0	4	8	1	0	0
EABT13344	22	8	5	12	2	1	15
EABT13345	0	0	5	0	4	0	0
EABT13346	0	0	2	0	0	0	0

EABT13347	1862.47	3637.13	2336.54	884.94	103	0	4
EABT13348	13	35	45	177	500	6	3
EABT13349	1717.95	3208.6	2174.96	3330.3	1236.86	1683.68	1854.68
EABT1335	42	45	8	20	9	8	5
EABT13350	41	75	135	107.12	101.05	52	75
EABT13351	906.02	1472.94	974.61	1495.02	720.22	2052.78	345
EABT13352	0	2	3	15	0	0	2
EABT13353	0	1	7	5	1	0	0
EABT13354	0	0	5	2	0	0	0
EABT13355	1	0	0	0	0	0	2
EABT13356	0	0	18	1	0	0	0
EABT13357	0	0	12	0	0	0	0
EABT13358	2	24	13	2	0	2	1
EABT13359	33365.6	82498.1	6038.42	6842.37	36785	120	565.3
EABT1336	8	65	0	1	0	0	1
EABT13360	303	483	146	44	314	612	1241
EABT13361	16	22	27	16	18	6	7
EABT13362	1	1	7	0	0	0	0
EABT13363	138	336.29	187.02	523.33	94	147	103
EABT13364	0	0	4	0	1	1	0
EABT13365	118.07	127	160	104	123	438	116
EABT13366	6	6	6	2	0	0	0
EABT13367	1	2	1	10	2	0	6
EABT13368	1	3	4	3	0	1	1
EABT13369	0	4	62	9	1	2	0
EABT1337	1	0	6	0	0	0	0
EABT13370	1	6	2	4	1	5	2
EABT13371	0	0	18	4	1	0	0
EABT13372	1208.06	1961.56	1056	1992	2591.34	293	465.6
EABT13373	19	25	19	18	6	9.03	9
EABT13374	0	0	3	4	2	0	0
EABT13375	0	0	3	1	1	0	0
EABT13376	1271.64	987	239	1038	359.72	1712	962
EABT13377	0	0	2	3	5	0	0
EABT13378	1355.66	1866.4	1338.55	2618.17	1175.76	947.47	674.07
EABT13379	0	2	0	0	0	3	1
EABT1338	1	0	1	0	0	0	4
EABT13380	4	2	10	0	0	0	1
EABT13381	758.95	794.07	355.08	1105.2	311	337.59	435
EABT13382	0	11	110	10	2	1	0
EABT13383	2	3	16	13	2	1	9
EABT13384	3	2	0	0	0	2	1
EABT13385	2	0	0	0	0	15	0
EABT13386	1	2	28	1	1	1	0
EABT13387	2995.35	3894	938	848	2572	3159.18	6676.37
EABT13388	1704.23	2034.95	3069.63	3330.9	2143.49	1680.88	1413.31
EABT13389	12.1	2	0	1	0	2	0
EABT1339	407.25	851.82	1153.68	2152.31	641.69	446.87	382.4
EABT13390	0	2	5	1	1	0	0
EABT13391	0	0	4	0	0	0	0
EABT13392	0	0	0	0	1	0	2

EABT13393	5	8	6	15	1	1	3
EABT13394	3	1	2	1	0	0	2
EABT13395	0	0	2	1	0	0	0
EABT13396	0	0	5	4	0	0	2
EABT13397	4	7	8	17	6	2	13
EABT13398	0	0	0	0	2	0	5
EABT13399	0	5	17	11	4	0	1
EABT134	1	6	3	8	5	0	1
EABT1340	470.93	397.77	247	270.92	198	294.62	374.4
EABT13400	0	0	8	0	0	0	0
EABT13401	0	1	17	1	0	0	0
EABT13402	0	0	2	2	5	0	0
EABT13403	0	0	3	2	2	2	1
EABT13404	32	59	64	67	28	17	20
EABT13405	806	2615.98	1139.64	793	997	5	218
EABT13406	0	2	2	0	0	1	3
EABT13407	2664	5131.89	5294.14	9434.9	3884.97	2428	2689
EABT13408	0	3	5	1	0	0	0
EABT13409	1	4	1	0	0	0	1
EABT1341	65	24	12	9	7	19	5
EABT13410	1470.42	2228.26	2823	3494.25	1354.58	1346.39	1290.78
EABT13411	0	8	15	1	57	0	1
EABT13412	0	0	4	0	2	0	0
EABT13413	21	29	47	25	8	1	2
EABT13414	2	13	14	77	10	0	0
EABT13415	6	6	1	0	0	0	1
EABT13416	0	0	6	2	0	0	0
EABT13417	0	1	0	3	0	0	1
EABT13418	1	0	1	2	2	2	2
EABT13419	225	868.56	1179.42	1570.9	206.91	144	107
EABT1342	1	2	0	0	1	0	1
EABT13420	285.25	422.7	489.49	807.99	363.06	148	265
EABT13421	0	1	13	0	0	0	0
EABT13422	0	2	33	0	3	3	1
EABT13423	3	4	0	0	0	4	12
EABT13424	0	0	33	1	0	0	0
EABT13425	0	0	10	0	0	0	0
EABT13426	6890.05	6407.35	5504.03	9234.95	4246.47	6176.49	4369.05
EABT13427	215	976	3606.01	1543	1779.38	156	296
EABT13428	0	4	0	0	0	3	0
EABT13429	1	2	3	4	10	0	0
EABT1343	279	420.05	867.28	1037.03	566.96	242	275
EABT13430	0	0	4	1	0	0	0
EABT13431	928.01	1400.96	1604.01	1829.36	1054	443.89	593.98
EABT13432	14227.2	15628.3	3517.42	3868.4	5867.26	27004.9	24583
EABT13433	1	6	14	6	3	0	0
EABT13434	3	2	14	8	0	1	3
EABT13435	3	15	77	1	1	0	0
EABT13436	0	1	3	0	0	1	4
EABT13437	0	0	15	0	0	0	0
EABT13438	0	3	21	2	1	0	0

EABT13439	9	2	0	0	0	6	0
EABT1344	0	1	20	5	1	0	0
EABT13440	5	9	13	12	1	0	2
EABT13441	0	0	6	5	1	0	0
EABT13442	1	2	9	0	0	0	0
EABT13443	4772.92	8222.05	6115.14	8181.43	7359.99	2743.1	2988.66
EABT13444	2	14	39	1	0	3	1
EABT13445	1	3	10	1	1	0	0
EABT13446	0	1	8	5	1	0	1
EABT13447	5	20	124.97	79	0	1	1
EABT13448	4	5	2	0	0	0	0
EABT13449	0	7	0	1	0	0	1
EABT1345	3	3	6	12	1	4	2
EABT13450	1	2	30.99	5	1	1	5
EABT13451	0	1	3	16	0	0	0
EABT13452	3	6	5	5	1	0	1
EABT13453	1	2	6	6	2	2	0
EABT13454	0	0	0	18	1	0	0
EABT13455	1	1	29	3	0	0	0
EABT13456	0	1	3	2	1	0	0
EABT13457	1334	2564.95	4994.6	3222.5	3289.63	1390.83	1650.55
EABT13458	0	0	15	1	0	0	0
EABT13459	3	11	75	143	29	2	15
EABT1346	2	0	6	0	0	0	0
EABT13460	0	0	11	20	8	0	0
EABT13461	0	0	1	4	1	0	0
EABT13462	0	1	6	1	0	0	1
EABT13463	963.02	514.27	35	50	196	1337.09	1060
EABT13464	2	4	2	4	3	9	10
EABT13465	1365	1957	2762.93	2385	1833	1205	955
EABT13466	0	1	5	1	1	0	1
EABT13467	0	3	19	2	6	2	3
EABT13468	0	0	1	1	1	0	0
EABT13469	1792.25	2969.67	4518.67	6681.58	1648.94	1867.97	1593.97
EABT1347	1	6	13	10	8	0	2
EABT13470	45	29	34	13	10	0	2
EABT13471	112	136	49	262	133.94	92	57
EABT13472	0	6	1	0	34	1	49
EABT13473	3522.01	4882.42	4042.67	8442.66	2723.38	3306.11	3277.1
EABT13474	0	0	8	0	1	0	0
EABT13475	5	5	10	0	10	1	9
EABT13476	0	0	0	2	2	0	0
EABT13477	4	3	21	0	8	0	1
EABT13478	1	2	5	32	1	2	5
EABT13479	0	3	12	0	0	0	1
EABT1348	580	1641.7	1163	801.99	859.7	11	34
EABT13480	0	9	16	2	3	1	0
EABT13481	554	670.16	312.97	593.93	267	428	475
EABT13482	0	1	0	0	2	1	2
EABT13483	3145	2976.98	1103	1514	11486.9	1775	1706.03
EABT13484	0	0	0	5	0	0	0

EABT13485	0	0	8	1	0	0	3
EABT13486	11	11	0	0	0	2	6
EABT13487	0	1	15	0	1	0	1
EABT13488	26	59	178	68.08	39	107	27
EABT13489	2489.11	5216.98	4880.95	9625.31	5328.63	3767.43	3546.71
EABT1349	0	4	6	8	4	1	0
EABT13490	0	0	0	11	0	0	0
EABT13491	0	0	0	0	0	2	3
EABT13492	0	0	7	0	0	0	1
EABT13493	8	5	0	0	0	4	1
EABT13494	0	5	14	4	0	0	0
EABT13495	485.3	745.08	1021.35	982	575.14	397.32	422.5
EABT13496	2	5	1	2	1	2	2
EABT13497	17	45	3	0	0	22	1
EABT13498	0	3	5	8	5	3	1
EABT13499	1	11	5	4	0	0	0
EABT135	10	40	62	1	3	8	2
EABT1350	12	111.17	281.86	710.03	988	11	14
EABT13500	0	2	22	1	0	7	0
EABT13501	13	10	18	29	7	0	1
EABT13502	0	1	10	2	2	0	1
EABT13503	14	4	1	1	3	13	8
EABT13504	26	124.01	205	378	144	4	46.91
EABT13505	13	30	8	2	1	5	19.03
EABT13506	0	0	0	0	0	2	1
EABT13507	2	2	0	4	2	0	1
EABT13508	1	0	6	0	0	0	1
EABT13509	0	0	1	0	0	0	0
EABT1351	3	2	4	2	2	8	2
EABT13510	2	0	7	0	0	0	1
EABT13511	141	343.38	399.08	944.63	179.01	73	136
EABT13512	1	2	0	0	0	1	3
EABT13513	0	1	1	3	0	0	0
EABT13514	4	5	3	0	0	0	0
EABT13515	1	1	25	1	2	1	0
EABT13516	0	0	5	4	0	0	0
EABT13517	151	71	3	1	20	0	3
EABT13518	0	0	3	3	0	0	0
EABT13519	30	162	215.95	399	57	11	35
EABT1352	1	13	14	104	4	1	7
EABT13520	2	0	24	1	2	0	1
EABT13521	0	1	5	2	0	2	0
EABT13522	9599.47	11746.4	6337.5	28	95	57	14
EABT13523	2	6	2	14	1	1	0
EABT13524	2	0	0	0	0	2	2
EABT13525	0	2	5	0	0	0	2
EABT13526	1	0	1	0	2	0	5
EABT13527	2	9	82	14	2	10	10
EABT13528	1	2	12	3	0	0	0
EABT13529	6344.75	4321.63	1149.45	3801.55	5402.36	666.45	1039.43
EABT1353	2	1	1	3	0	0	0

EABT13530	0	3	13	5	0	0	0
EABT13531	1	0	3	1	3	0	0
EABT13532	0	0	15	0	1	0	0
EABT13533	0	4	35.99	9	0	0	0
EABT13534	2	2	1	0	0	1	1
EABT13535	2	3	2	4	1	2	1
EABT13536	0	0	4	0	9	1	0
EABT13537	0	2	11	0	0	1	0
EABT13538	0	1	0	1	2	0	0
EABT13539	1	6	18	8	0	1	0
EABT1354	1	0	8	1	0	0	0
EABT13540	8	12	152	8	2	6	5
EABT13541	0	0	2	0	5	1	0
EABT13542	1743.81	2173.54	3875.98	5107.12	1920.01	2064.48	1723.37
EABT13543	0	1	6	1	4	1	0
EABT13544	1	7	18	4	1	1	1
EABT13545	0	0	3	0	2	0	0
EABT13546	0	0	3	10	0	0	0
EABT13547	0	2	0	3	1	0	0
EABT13548	2	4	2	22	0	0	1
EABT13549	819	1667	2170	4624.98	1287.96	960.01	944
EABT1355	1	4	27.1	0	3	4	6
EABT13550	0	1	12	0	0	0	0
EABT13551	1	2	15	1	3	0	4
EABT13552	2	0	2	1	3.95	0	0
EABT13553	8	12	12	3	3	12	9
EABT13554	0	0	9	2	0	0	0
EABT13555	16	24	29	89	15	11	14
EABT13556	2	0	2	1	13	0	5
EABT13557	3	2	11	4	1	1	1
EABT13558	2	1	22	3	0	1	0
EABT13559	0	0	7	0	1	0	0
EABT1356	0	0	12	0	0	0	0
EABT13560	0	5	2	8	0	0	0
EABT13561	154	105	60.01	159	131	15	95
EABT13562	3	5	2	0	1	7	0
EABT13563	0	7	150.05	4	2	0	2
EABT13564	1	2	3	0	0	1	0
EABT13565	5359.13	6106	9	12	147	6	1
EABT13566	0	0	4	1	0	1	0
EABT13567	115	164	529.87	306.14	267	19	28
EABT13568	0	3	55	1	1	0	1
EABT13569	793.56	1196.28	1428.06	3707.01	523.02	461.59	595
EABT1357	1602.87	705	108	7	426	75	1279
EABT13570	1968.95	4025.24	2382.04	4277.69	3401.74	868	4096.34
EABT13571	6205	12148.2	6981.98	1396.64	2821.53	6510.47	3534.91
EABT13572	0	0	4	1	0	0	0
EABT13573	0	1	17	1	0	1	0
EABT13574	0	2	6	2	2	1	1
EABT13575	0	0	5	1	1	0	4
EABT13576	1	0	1	8	0	0	0

EABT13577	0	4	19	10	0	0	0
EABT13578	0	2	3	4	1	0	0
EABT13579	0	0	0	0	2	0	4
EABT1358	2	0	10	4	0	2	1
EABT13580	0	0	12	0	0	0	0
EABT13581	3	16	84	6	2	1	4
EABT13582	3	3	10	0	0	1	0
EABT13583	49	202.07	69	241.88	364.26	29	61
EABT13584	0	0	22	1	0	0	1
EABT13585	879.01	1249.52	1406	2525.25	1244	1020	903.67
EABT13586	2	2	9	2	0	0	0
EABT13587	2869.1	5024.18	3561.32	1688.55	706.09	7	62
EABT13588	0	1	2	2	0	0	0
EABT13589	1	3	3	4	0	2	0
EABT1359	0	4	10	2	0	2	0
EABT13590	3	5	12	6	7	2	0
EABT13591	14	69	165.93	459	32	9	14
EABT13592	0	0	16	0	0	0	1
EABT13593	9125.64	9472.58	6216.53	6803.25	7537.91	8151.67	8775.49
EABT13594	3	4	8	10	0	0	0
EABT13595	2	5	2	2	2	1	6
EABT13596	3	5	5	4	3	2	1
EABT13597	370.18	461.34	273	1366	2682.39	15	980.73
EABT13598	1	1	4	0	2	0	0
EABT13599	0	0	13	1	0	0	3
EABT136	4047.31	7493.7	14069.8	4170.3	2919.86	2801.36	2579.76
EABT1360	1	0	8	3	0	0	1
EABT13600	0	0	1	0	18	0	0
EABT13601	0	0	0	18	1	0	0
EABT13602	0	1	9	0	2	1	5
EABT13603	0	1	11	2.01	1	1	4
EABT13604	1	0	4	2	0	2	0
EABT13605	136	146	37	251	158	70	114
EABT13606	1	3	7.06	2	0	4	1
EABT13607	5	12	0	0	0	0	0
EABT13608	16	70	304	321	67	0	3
EABT13609	0	0	11	0	0	0	0
EABT1361	26987.2	100213	93018.7	63250.1	26082.3	14138.9	20299.9
EABT13610	2	2	22	0	1	0	2
EABT13611	0	1	6	2	0	0	0
EABT13612	0	0	15	0	0	0	0
EABT13613	0	0	0	7	0	0	0
EABT13614	0	2	4	0	0	0	0
EABT13615	0	0	0	0	0	0	0
EABT13616	31	74	51	81	18	29	20
EABT13617	341.09	625.8	554.01	2646.72	883	304.68	415.04
EABT13618	132	150	102.14	240.03	120.83	42	53
EABT13619	24	21	0	0	6	67	31
EABT1362	3532.96	5227.79	5219.42	8701.07	3809.06	3221.08	2868.99
EABT13620	2	16	40	4	5	0	1
EABT13621	0	2	157.01	4	5	0	2

EABT13622	236	329	169	457	399	183	157
EABT13623	3909.25	2724.86	1166.53	3026.67	1962.02	5726.16	4723.94
EABT13624	0	2	105	2	0	0	1
EABT13625	0	2	0	0	5	1	3
EABT13626	0	2	7	4	1	0	0
EABT13627	0	3	4	2	0	0	0
EABT13628	0	0	3	0	1	0	0
EABT13629	1753	2239.01	1431.03	2755.99	1873.02	1428.26	1521
EABT1363	0	2	4	4	2	0	1
EABT13630	3	3	8	4	4	0	3
EABT13631	2	2	1	9	2	0	1
EABT13632	3298.55	4719.98	5772.9	19504.8	5393.86	3043.12	3860.63
EABT13633	151	290	359.29	895	200.23	106	126
EABT13634	55	70	120	248	60	23	51
EABT13635	2	5	8	2	0	3	1
EABT13636	672.79	1204.92	1386.8	2655	1413.85	481.27	634.78
EABT13637	0	4	1	0	0	0	1
EABT13638	4	4	1	0	0	92	1
EABT13639	35	4	0	0	0	8	0
EABT1364	0	0	4	0	0	0	0
EABT13640	1	2	2	1	1	0	0
EABT13641	0	0	5	0	0	0	2
EABT13642	0	0	5	6	0	0	4
EABT13643	4	4	0	0	0	0	1
EABT13644	0	3	7	10.97	0	1	1
EABT13645	2	43	55.98	60	6	3	1
EABT13646	2	8	14	5	0	1	1
EABT13647	4	11	109	4	6	4	4
EABT13648	2	5	7	8	9	0	2
EABT13649	6	7	0	0	3	18	10
EABT1365	0	4	39	1	0	0	0
EABT13650	86	59	115	189	124	35	88
EABT13651	30	82	80	20	6	20	14
EABT13652	0	1	3	4	9	0	3
EABT13653	0	1	4	6	1	0	0
EABT13654	0	0	0	0	0	0	0
EABT13655	0	1	2	0	0	1	0
EABT13656	2	1	4	3	0	0	0
EABT13657	3	9	26	6	4	29	0
EABT13658	0	0	5	0	0	0	0
EABT13659	0	1	12	1	1	1	1
EABT1366	8	11	32	22	4	5	8
EABT13660	5	1	7	1	0	3	1
EABT13661	1	0	1	0	1	0	0
EABT13662	1	1	3	3	2	0	0
EABT13663	0	4	22	6	1	0	1
EABT13664	0	1	5	0	0	1	0
EABT13665	14	29	0	0	2	53	34
EABT13666	1	1	5	1	0	0	0
EABT13667	17	25	0	0	1	39	20
EABT13668	0	1	23	1	0	0	1

EABT13669	0	0	11	7	1	0	0
EABT1367	6	2	30	2	0	1	1
EABT13670	4042.68	4782.17	2484.71	5658.69	4482.96	3802.7	3320.4
EABT13671	0	6	11	0	0	0	0
EABT13672	0	2	7	6	1	1	0
EABT13673	3	3	65	6	1	1	0
EABT13674	24	44	159.99	57	34	55	34
EABT13675	3	4	35	0	4	2	8
EABT13676	25	31	264	10	5	25	9
EABT13677	0	0	17	0	0	0	0
EABT13678	3	4	24	4	2	2	1
EABT13679	1	2	2	1	2	0	4
EABT1368	3	10	9	25	5.66	4	8
EABT13680	3	6	0	0	1	0	0
EABT13681	2	0	2	0	0	0	2
EABT13682	1242.13	1603.08	1059.23	2464.38	1438.25	838.49	1000.37
EABT13683	0	1	0	2	2	0	8
EABT13684	0	0	3	1	0	0	0
EABT13685	3	5	7	0	1	10	3
EABT13686	0	1	2	8	0	0	0
EABT13687	11	4	0	0	4	22	32
EABT13688	0	1	1	3	1	0	0
EABT13689	3	0	0	0	0	12	0
EABT1369	0	1	8	3	1	0	0
EABT13690	0	1	5	1	0	0	2
EABT13691	1	1	6	4	0	0	2
EABT13692	15	40	9	240	9	0	7
EABT13693	4708.23	6604.04	4354.33	4545.4	3751.57	3156.39	3248.9
EABT13694	0	3	4	0	0	0	0
EABT13695	1	1	13	1	3	1	0
EABT13696	4	8	10	14	4	3	4
EABT13697	1	3	2	3	126	0	1054
EABT13698	1	0	0	0	2	0	0
EABT13699	0	0	12	0	1	0	0
EABT137	0	8	1	1	1	1	10
EABT1370	917.32	1412.81	3020.43	2522.13	1217.98	1041.45	1195.18
EABT13700	0	0	9	0	0	0	0
EABT13701	0	0	0	1	0	0	0
EABT13702	0	0	4	1	0	0	0
EABT13703	1	6	25	5	0	3	1
EABT13704	2291.95	2948	4413.32	4983.03	2057.4	3146.02	2284
EABT13705	2	3	1	0	1	0	2
EABT13706	4	2	11	5	2	4	2
EABT13707	2	1	0	3	4	0	0
EABT13708	20	14	0	0	0	3	2
EABT13709	9	10	14	22	0	0	0
EABT1371	2	15	5	5	4	0	1
EABT13710	0	2	6	2	0	0	0
EABT13711	0	2	0	1	0	2	0
EABT13712	0	0	38	1	0	0	1
EABT13713	5415.04	9699.67	9790.77	20559.3	4145.94	3538.21	3624.95

EABT13714	0	0	3	0	0	0	0
EABT13715	0	0	1	0	10	1	3
EABT13716	464	740.45	683.96	1854.97	847.15	311.02	469.65
EABT13717	264.58	889.77	971.45	2253.49	1020.9	106	346.19
EABT13718	3	4	13	6	2	1	1
EABT13719	3	4	5	4	6	2	3
EABT1372	1	5	5	0	3	0	3
EABT13720	0	3	2	0	0	0	1
EABT13721	1149.35	1643.96	1543.02	1951.01	2797.08	193.3	1243.31
EABT13722	0	9	3	4	2	0	0
EABT13723	0	2	3	2	2	0	1
EABT13724	0	2	19	2	1	0	0
EABT13725	1	1	29	0	0	0	1
EABT13726	1	0	4	1	4	1	5
EABT13727	2	7	2	5	4	3	1
EABT13728	0	1	1	1	0	1	2
EABT13729	1	1	3	8	0	0	0
EABT1373	0	10	51	5	1	0	0
EABT13730	1	0	7	0	0	0	1
EABT13731	16.01	31	29	3	3	1	3
EABT13732	25890.8	45543.8	48052.7	23317.1	23328.9	10349.4	17431.9
EABT13733	0	0	0	0	1	0	4
EABT13734	7339.9	8931.44	8357.72	13228.7	3580.02	1266.1	1608.41
EABT13735	14	24	9	13	6	4	31
EABT13736	1	3	7	2	1	1	2
EABT13737	0	1	2	0	1	0	2
EABT13738	0	2	6	3	0	0	1
EABT13739	404.06	580.13	436.87	803.86	278.11	302.77	266.29
EABT1374	0	0	17	4	1527	10	16
EABT13740	1	1	1	0	0	0	1
EABT13741	199	291	318.43	738.36	295	181	222
EABT13742	1996.41	1922.95	2229.4	3969.46	1527.71	1619.27	1646.55
EABT13743	0	0	7	2	0	0	0
EABT13744	1	0	11	2	0	0	0
EABT13745	0	2	13	0	0	0	0
EABT13746	617.97	852	901.25	1251	789.62	538	512.72
EABT13747	1	0	4	3	0	0	0
EABT13748	1	0	3	6	0	0	3
EABT13749	0	1	5	2	0	1	0
EABT1375	1.28	2	37	34	0	0	0
EABT13750	3172.99	10468.7	1413.95	54	1497.96	4396.74	3821.36
EABT13751	0	7	0	0	0	1	0
EABT13752	4	1	4	3	0	3	0
EABT13753	4	28	22	19	10.09	2	11
EABT13754	4654.48	35335.7	360251	228089	68470.4	664.36	2025.91
EABT13755	10	22	157	10207.2	7	13	4
EABT13756	0	0	0	7	0	0	0
EABT13757	0	2	0	2	6	6	14
EABT13758	6	1	13	3	1	0	0
EABT13759	0	1	1	3	2	0	0
EABT1376	3	4	3	2	0	2	0

EABT13760	9	21	28.6	17	2	23	4
EABT13761	7	1	0	0	0	5	0
EABT13762	2	3	27	3	1	0	1
EABT13763	11	11	20	16	3	5	5
EABT13764	4	6	13	6	1	1	7
EABT13765	30	32.75	43	79	57	59.11	71
EABT13766	0	2	20	1	0	0	0
EABT13767	11	12	7	1	4	8	5
EABT13768	1	3	20	16	5	2	5
EABT13769	0	0	3	1	0	0	0
EABT1377	162	245	189	509.21	203	89	125
EABT13770	22	38	17	40	7	19	5
EABT13771	1218.12	1288.57	945.24	3599.98	969.98	892.01	610.52
EABT13772	0	2	7	4	0	0	0
EABT13773	1	5	12	4	0	1	0
EABT13774	2	2	3	0	2	0	1
EABT13775	69	132	202	233	69	58	75
EABT13776	0	0	9	1	0	0	0
EABT13777	12.99	2	2	5	0	16	5
EABT13778	4	15	50	197.63	38	7	18
EABT13779	1	7	23.8	9	0	0	0
EABT1378	1	2	20	1	0	1	0
EABT13780	15	3	10	1	51708.8	31	164
EABT13781	0	0	4	1	0	0	0
EABT13782	1	1	51	0	0	0	0
EABT13783	792.76	1624.98	1074	2316.87	1561.01	721.09	1069
EABT13784	5	10	1	1	7	23	6
EABT13785	6	2	10	1	1	0	0
EABT13786	2	2	7	2	1	1	0
EABT13787	0	2	2	3	0	0	0
EABT13788	9	2	0	0	3	13	22
EABT13789	0	0	3	4	0	0	0
EABT1379	21	4	92.99	0	2	0	0
EABT13790	4375.58	4778.97	4916.37	5553.94	3334.75	3907.7	4206.17
EABT13791	0	1	3	0	1	0	0
EABT13792	55.56	60	174.89	3484.4	13	9	0
EABT13793	0	0	3	0	0	1	0
EABT13794	0	0	6	1	0	3	1
EABT13795	0	0	8	1	1	0	0
EABT13796	0	1	11	13	13	0	2
EABT13797	9	24.75	16	16	12	14	11.96
EABT13798	0	0	0	0	29	1	1
EABT13799	0	7	15	1	2	0	0
EABT138	1	5	1	3	3	1	3
EABT1380	1	3	1	0	0	3	0
EABT13800	0	0	12	0	1	0	0
EABT13801	3	10	23	5	4	1	11
EABT13802	1	16	15	7	7	3	17
EABT13803	65	100	149	183.91	96	83.93	77.99
EABT13804	112	617	2018.85	1570.18	420.01	104	241
EABT13805	1	7	0	11	5	0	0

EABT13806	955.76	1331.41	989.06	2909.53	1001.39	580	659.98
EABT13807	450	601	393	63	171	395	453
EABT13808	0	0	23	2	0	2	0
EABT13809	558	810.05	421	531	208.68	84	146
EABT1381	7	9	2	0	0	22	8
EABT13810	0	1	19	3	0	0	0
EABT13811	1	3	6	1	0	0	0
EABT13812	5	11	14	14	8	12	4
EABT13813	0	9	26	8	1	2	1
EABT13814	3	8	10	81	14	1	2
EABT13815	0	1	2	6	10	0	2
EABT13816	6	5	37	6	0	0	0
EABT13817	1019.79	1660.72	3158.33	4076.7	1593.28	1097.63	883.79
EABT13818	0	0	2	0	0	0	77
EABT13819	0	2	5	0	0	0	0
EABT1382	0	0	5	4	2	0	0
EABT13820	2336.19	5235.91	7292.4	4878.91	1691.7	2727.18	2802.52
EABT13821	1	5	5	2	1	2	1
EABT13822	1	7	14	1	2	1	1
EABT13823	1	4	1	0	0	0	0
EABT13824	8	74	0	7	30	0	1
EABT13825	1940.59	30.96	0	5	1	3653.15	0
EABT13826	0	0	5	3	0	0	0
EABT13827	1	6	3	3	1	1	0
EABT13828	0	0	1	0	2	0	1
EABT13829	3	12	21	73	29	2	6
EABT1383	2119.72	4106.43	5015.69	2663.89	2458.92	790.7	823
EABT13830	2118.16	2411.43	319.98	5	476	158	316.01
EABT13831	0	3	2	0	2	0	1
EABT13832	0	1	2	13	2	1	0
EABT13833	0	1	51	4	1	0	0
EABT13834	1294.84	1868.11	820.03	1068.67	2123.25	548.96	818.1
EABT13835	1	1	0	0	0	0	1
EABT13836	14	6	36	31	16	11	30
EABT13837	2	0	4	0	0	0	0
EABT13838	0	1	11	2	0	1	0
EABT13839	1	1	12	1	0	3	0
EABT1384	2	2	21	11	10	3	12
EABT13840	5	1	5	6	1	1	1
EABT13841	209	691.05	685.02	1073.01	513	542.08	596.99
EABT13842	0	0	1	0	0	0	0
EABT13843	1	3	4	2	1	0	0
EABT13844	3920.56	3106.71	4481.77	8	1120.03	1776.18	1892.17
EABT13845	3	2	3	4	1	0	1
EABT13846	0	0	21	0	0	0	0
EABT13847	0	0	0	0	1	2	11
EABT13848	0	13	12	5	0	0	0
EABT13849	1	1	3	23	2	1	3
EABT1385	6	50	22	3	11	1	1
EABT13850	1	2	6	1	0	0	0
EABT13851	1919.55	4575.62	2030.66	3916.57	2986.35	1682.94	3153.08

EABT13852	4	12	126	16	2	7	1
EABT13853	1	3	0	0	0	1	2
EABT13854	2	1	0	0	0	0	2
EABT13855	0	1	16	2	0	0	3
EABT13856	1288.23	1532.42	1301.22	1575.93	1672.27	1103.36	1016.61
EABT13857	41	121	11	106	3	0	3
EABT13858	2	3	21	7	0	1	1
EABT13859	0	0	2	8	2	0	1
EABT1386	250	701	35461.9	1923.2	25020.4	59	505.45
EABT13860	0	11	0	0	0	0	0
EABT13861	20	27	58	33	15	6	7
EABT13862	0	0	0	1	2	0	4
EABT13863	0	1	6	0	0	3	4
EABT13864	0	3	24	1	1	0	1
EABT13865	0	8	1	5	0	0	1
EABT13866	532.1	706.19	1271.96	523	518.2	527.1	426
EABT13867	1	1	9	0	0	0	0
EABT13868	1	4	71	1	0	3	2
EABT13869	0	4	0	4	3	2	0
EABT1387	289.2	559.81	1956.53	221.89	431.99	40	338
EABT13870	2	6	20	4	2	0	0
EABT13871	3	36	55	413	95	57.34	5
EABT13872	2031.44	2467.78	1723.78	3538.83	1951.23	1647.05	1526.43
EABT13873	1	7	0	1	0	1	1
EABT13874	1163.61	1600.42	1607.42	2081.81	1304.18	1089.18	1237.78
EABT13875	2	2	0	0	0	0	1
EABT13876	0	0	4	1	0	0	0
EABT13877	3	12	44	3	1	0	1
EABT13878	1	0	1	10	2	0	0
EABT13879	0	3	4	2	1	0	2
EABT1388	0	0	2	0	1	1	0
EABT13880	2	2	3	1	2	1	1
EABT13881	875.07	1278.56	1006.63	2604.9	1171.05	388.03	747.01
EABT13882	6	6	12	9	4	1	0
EABT13883	2	2	0	0	0	0	0
EABT13884	1	5	7	0	0	0	0
EABT13885	4	3	0	0	0	16	0
EABT13886	710	1760	855	1030.14	491.93	78	183.73
EABT13887	0	5	19	3	1	0	3
EABT13888	33	1	1	3	10	172.07	88.7
EABT13889	1	0	24	0	1	3	0
EABT1389	77	301	167.96	299	919	2	36
EABT13890	1147.58	1215.04	985	1102	683	1147	501
EABT13891	4	12	24	15	2	10	6
EABT13892	1	3	92	0	0	0	0
EABT13893	1	4	7	2	0	1	0
EABT13894	2	2	32	6	0	0	3
EABT13895	778	1272.59	929.01	716.08	1039	324	796
EABT13896	1.74	5	7	6	1	0	1
EABT13897	24	38	70	108	17	12	25
EABT13898	0	1	7	1	0	0	0

EABT13899	2	1	2	1	0	1	0
EABT139	0	3	4	0	1	1	2
EABT1390	0	4	4	0	0	0	0
EABT13900	1436	2935.92	7249.63	2869.04	1396.99	196	375
EABT13901	2	4	4	1	0	0	0
EABT13902	1696	2909.03	1564	6250.41	1631.68	1067.02	1463
EABT13903	7	5	10	0	0	1	2
EABT13904	82985.5	84241.8	27172	97294.9	53851.2	26017.9	25949.7
EABT13905	13	34	77.03	5	0	5	0
EABT13906	6	56	6	0	10	36	34
EABT13907	610.99	747.23	1573.16	930.2	720.23	415.93	434.82
EABT13908	1838.16	3365.66	3517.63	4963.07	4071.66	958	1831.11
EABT13909	3	6	10	7	0	0	8
EABT1391	0	0	2	0	0	0	0
EABT13910	1	1	4	1	0	0	0
EABT13911	14	14	0	0	0	6	3
EABT13912	0	2	7	1	0	0	0
EABT13913	5	3	11	8	3	4	2
EABT13914	1	4	0	0	0	0	1
EABT13915	0	3.02	2	2	0	1	1
EABT13916	1	2	6	3	0	0	0
EABT13917	2	4	33	20.96	5	0	0
EABT13918	0	1	14	6	2	0	0
EABT13919	0	0	4	0	1	0	0
EABT1392	2	9	17	0	0	1	2
EABT13920	0	1	5	1	1	6	0
EABT13921	4	2	4	5	4	2	4
EABT13922	5	20	9	4	3	7	1
EABT13923	58	70	30	7	223	3	21
EABT13924	1892.94	1885.52	3414.6	10662.6	2625.12	310.01	516.08
EABT13925	2	1	4	3	1	0	2
EABT13926	0	0	11	0	5	0	1
EABT13927	1	3	10	0	0	0	1
EABT13928	0	1	0	0	1	1	0
EABT13929	14	16	20	1	5	21	22
EABT1393	0	0	4	2	0	0	0
EABT13930	2	1	5	1	0	6	1
EABT13931	4	9	7	4	2	2	0
EABT13932	1	6	20	15	4	2	6
EABT13933	0	2	6	0	2	0	0
EABT13934	1	2	20	2	0	1	1
EABT13935	0	1	3	8	4	2	0
EABT13936	1	5	8	2	1	5	0
EABT13937	0	1	3	1	1	0	0
EABT13938	0	0	0	4	0	1	3
EABT13939	686	5763.56	554	405	111	8.03	15
EABT1394	6	27	72	22	11	7	12
EABT13940	0	0	0	0	0	0	0
EABT13941	0	0	15	2	0	0	0
EABT13942	0	2	3	2	2	0	2
EABT13943	2989.99	314	12	0	1	2102	3

EABT13944	0	4	4	1	1	0	0
EABT13945	46	43	58.99	37	15	204	97
EABT13946	58	78	25	65	41	27	14
EABT13947	1	1	6	1	0	0	2
EABT13948	0	0	15	0	0	1	0
EABT13949	0	15	0	5	3	0	0
EABT1395	3	4	69	30	4	0	0
EABT13950	0	1	6	2	0	0	0
EABT13951	0	3	2	5	2	0	1
EABT13952	0	0	8	0	1	0	0
EABT13953	2	2	7	14	1	0	0
EABT13954	23	23	102	114	57.99	8	23
EABT13955	1	0	6	4	2	0	1
EABT13956	0	4	5	1	0	0	0
EABT13957	1	1	16	1	3	2	1
EABT13958	1	1	9	0	2	0	1
EABT13959	2	12	8	23	4	4	0
EABT1396	0	1	7	0	0	0	0
EABT13960	0	4	6	1	1	0	1
EABT13961	2	1	6	1	0	0	0
EABT13962	13	19	23	50	14	8	6
EABT13963	55	142.49	52	776.67	443.09	39	8
EABT13964	105.91	261.23	306.98	300.13	223.26	52	35
EABT13965	2	3	6	29	1	0	0
EABT13966	498	767	615.79	1523.47	792.14	345	443
EABT13967	0	1	36	0	1	0	0
EABT13968	1	1	4	4	0	2	1
EABT13969	4	12	7	98	7	0	0
EABT1397	0	0	0	2	1	0	0
EABT13970	0	2	3	4	0	0	0
EABT13971	89	197.56	164.78	355.48	35	32.26	38
EABT13972	11	15	1	9	4	1	2
EABT13973	3	13	27.84	0	6	1	0
EABT13974	4	2	3	4	4	0	0
EABT13975	30	5	1	0	1	0	1
EABT13976	0	5	1	1	0	0	0
EABT13977	1304.51	1510.04	119	585.93	364	0	25
EABT13978	2	1	4	3	0	2	0
EABT13979	2873.45	5095.65	5103.04	5960.16	6991.52	3069.34	4482.81
EABT1398	4	1	2	1	2	2	0
EABT13980	0	1	3	10	0	0	1
EABT13981	1	4	8	4	2	1	1
EABT13982	10	20	27	26	29	6	6
EABT13983	376.99	607.02	654	1185	690	352.71	411
EABT13984	3	8	16	18	5	4	7
EABT13985	0	1	4	0	5	0	1
EABT13986	58	35	0	0	0	11	1
EABT13987	6	16	103	451.97	36	0	0
EABT13988	0	0	0	0	22	0	0
EABT13989	3	1	1	0	0	0	3
EABT1399	1147.45	1585.3	4360.45	4080.41	1679.5	997.29	1552.49

EABT13990	253.15	375.79	634.3	693.08	392	297.06	158.56
EABT13991	3	14	63	4	0	0	1
EABT13992	145	338.97	1435.99	64	15	29	26
EABT13993	0	0	8	0	0	0	0
EABT13994	0	1	10	1	0	2	0
EABT13995	2587	1397.94	548.27	284.75	868.06	2700.42	3537.08
EABT13996	6	17	0	0	1	1	0
EABT13997	0	0	10	0	0	0	0
EABT13998	1	0	7	5	2	0	0
EABT13999	6477.23	5558.49	3505.58	4088.07	3691.22	4702.56	6587.87
EABT14	16885.9	28447.9	22125.5	57812.3	34878.5	24	622
EABT140	4	7	2	0	1	2	5
EABT1400	0	1	4	4	0	1	0
EABT14000	863	1150.05	869	1546.07	927.91	648.62	729
EABT14001	0	1	8	0	0	0	0
EABT14002	0	7	6	1	0	1	0
EABT14003	5572.29	8552.76	17944.1	11196.8	6059.54	7366.53	4492.57
EABT14004	0	2	23	6	4	0	1
EABT14005	0	0	0	0	2	0	0
EABT14006	2	5	24	3	1	6	3
EABT14007	11	6	0	0	0	4	0
EABT14008	0	0	8	6	0	0	0
EABT14009	359.9	456	828.12	533.98	372.84	360.93	417.12
EABT1401	504.93	746.1	109	670.97	437.64	271	414
EABT14010	13	13	23	12	1	29	10
EABT14011	0	1	1	6	3	0	2
EABT14012	1263.5	1852.65	1818.99	2443.84	1462.17	1085.77	957.31
EABT14013	0	2	4	0	0	2	0
EABT14014	1	0	6	0	0	1	1
EABT14015	1	1	2	2	0	0	0
EABT14016	0	0	10	1	0	0	0
EABT14017	1	2	1	12	2	0	0
EABT14018	7	14	22	33	34	3	32
EABT14019	7	11	20	21	3	6	6
EABT1402	3	2	6	1	0	2	1
EABT14020	1	28	11	32	5	3	5
EABT14021	2	0	1	5	0	1	0
EABT14022	0	1	8	2	0	0	0
EABT14023	0	1	3	1	0	0	0
EABT14024	1	1	12	3	13	0	1
EABT14025	4	6	7	9	1	1	6
EABT14026	3	9.21	22	27	3	1	6
EABT14027	4	11	67	20	4	3	8
EABT14028	16	9	1	0	2	3	10
EABT14029	2	10	15	4	2	3	1
EABT1403	2	5	0	0	0	2	3
EABT14030	0	3	2	1	1	0	0
EABT14031	0	0	10	0	0	0	0
EABT14032	0	4	16	1	0	0	0
EABT14033	0	1	5	9	2	0	0
EABT14034	0	0	6	0	0	0	0

EABT14035	0	1	5	2	0	3	0
EABT14036	0	0	5	13	0	0	0
EABT14037	0	0	11	0	0	0	0
EABT14038	0	1	0	0	1	0	0
EABT14039	984.88	1242.48	880.01	1397.98	858	1232.07	814.01
EABT1404	2520.22	4016.88	2801.95	3246.25	2895.57	2407.34	4453.01
EABT14040	2976.17	7055.68	7333.59	7779.56	5044.41	2230.96	2449.36
EABT14041	0	3	12	15	16	0	0
EABT14042	193	359.42	332	1027	227.11	114	267.02
EABT14043	0	2	4	6	1	0	2
EABT14044	4	33	64	41	1	1	4
EABT14045	8	7	7	3	1	1	0
EABT14046	0	1	48	12	3	0	0
EABT14047	98.5	124	77	182.98	68	18	42
EABT14048	28	29	10	302	82	15	12
EABT14049	1	4	19	0	0	1	0
EABT1405	0	1	0	0	1	0	1
EABT14050	19	67	72	242	150	3	4
EABT14051	5	6	5	8	3	1	0
EABT14052	3	6	99	5	4	2	1
EABT14053	86776.6	810.99	1	2	0	5560.25	1
EABT14054	553.95	1091.94	996	1500.99	662.16	649	585.13
EABT14055	0	3	3	0	0	0	1
EABT14056	122	132	94	126	143	114	79
EABT14057	1	10	51	3	0	2	1
EABT14058	406	628	895	1119.89	591.12	271	228
EABT14059	1472.99	2173.88	1742.06	2314.09	622.29	1098.31	1071.77
EABT1406	6	16	33	47	15	3	15
EABT14060	7	12	44	0	0	2.99	4
EABT14061	15	18	3	21	20	0	19
EABT14062	571.24	1197.59	1314	1345.21	1127.72	174.85	462.68
EABT14063	1	4	0	0	1	1	2
EABT14064	6	17.47	17	81	4	7	5
EABT14065	0	1	13	7	1	0	0
EABT14066	0	2	4	1	0	0	0
EABT14067	3151.91	8358.42	4122.06	19162.8	2926.16	2772.33	3757.1
EABT14068	1316.84	1617.37	1062.99	2631.99	1011.9	917.56	867.27
EABT14069	3476	5876.67	4686.68	5478.68	3137.04	5152	5393.75
EABT1407	2	1	2	1	3	0	1
EABT14070	0	0	0	0	3	0	2
EABT14071	2	7	0	0	0	4	4
EABT14072	0	0	11	0	0	0	0
EABT14073	0	13	36	1	5	3	1
EABT14074	0	1	3	1	0	0	0
EABT14075	4	13	69.06	76	8	3	8
EABT14076	1298.61	1580.28	1199.09	1983.21	904.1	1065.25	819.37
EABT14077	1	5	11	10	25	1	11
EABT14078	0	0	6	0	0	0	0
EABT14079	537.01	1078.93	1030.61	3750.07	1064.39	518.12	755.08
EABT1408	1	4	9	4	0	3	6
EABT14080	2	5	0	1	2	9	20

EABT14081	2	13	3	0	2	9	0
EABT14082	0	6	1	2	1	13	1
EABT14083	7	9	18	12.95	7.15	5	6
EABT14084	1	3	1	24	0	1	0
EABT14085	0	4	32	0	0	0	0
EABT14086	425.78	607	472	832.99	796.85	296	383
EABT14087	1	3	11	3	0	0	0
EABT14088	77.99	244	241.59	158	859	28	15
EABT14089	10	26	25	4	0	3	3
EABT1409	4	6	6	0	0	0	2
EABT14090	1549.41	2586.73	2684	3389.78	2364.12	2510.45	2725.82
EABT14091	2	2	2	4	2	0	2
EABT14092	18	69	60	48	247	5	3
EABT14093	0	0	10	4	0	1	0
EABT14094	0	0	19	4	2	0	0
EABT14095	1	3	4	0	1	0	0
EABT14096	1	2	2	0	2	1	1
EABT14097	0	0	0	0	3	12	8
EABT14098	5779.43	7667.82	7752.23	11248.1	4521.06	6993.84	4721.21
EABT14099	0	0	11	0	0	1	1
EABT141	2910.94	4356.26	13853.1	2907.56	1608.54	85.87	1243.54
EABT1410	0	0	0	1	1	0	1
EABT14100	1	0	5	0	0	0	0
EABT14101	0	0	0	0	2	0	0
EABT14102	0	0	110.02	1	3	2	5
EABT14103	0	1	8	0	0	0	0
EABT14104	1	0	7	5	0	0	1
EABT14105	0	1	13	5	0	0	0
EABT14106	1	4	57	4	1	0	0
EABT14107	0	1	10	1	1	0	0
EABT14108	433.61	545.68	400.07	473	534	412	323
EABT14109	2	2	0	1	0	0	0
EABT1411	184	335	376	671	461.01	269	72
EABT14110	1	1	3	2	0	1	1
EABT14111	0	9	29	21	0	7	4
EABT14112	0	1	6	0	2	0	0
EABT14113	2	0	2	0	0	0	1
EABT14114	8	14	32	163	70	2	18
EABT14115	34	96	60	458.74	452.89	28	19
EABT14116	1598.9	2478.26	2584.19	4453.44	1948.35	1069.64	1352.75
EABT14117	16	19.02	1	0	1	3	1
EABT14118	7	7	2	128	9	0	0
EABT14119	0	23	36.34	9	8	0	3
EABT1412	2	1	1	1	0	0	0
EABT14120	316.18	454.47	381.49	938.29	371.02	184.27	196.09
EABT14121	895.27	1509.01	1002	3884.82	1122.26	433.15	339
EABT14122	0	1	8	13	1	0	1.59
EABT14123	0	0	1	0	0	0	4
EABT14124	0	2	7	1	0	0	0
EABT14125	4318.58	6096.65	7949.68	21969.1	6178.8	3891.58	3091.31
EABT14126	23	36	4	2	3	0	0

EABT14127	1731.87	3197.55	2035.67	4260.16	2291.99	1542.07	2518.92
EABT14128	186	1079	481.89	1554	174.97	98	270
EABT14129	1	3	16	0	2	2	3
EABT1413	1	0	3	0	1	1	0
EABT14130	2	2	3	4	3	1	1
EABT14131	7	1	0	1	1	0	1
EABT14132	224.01	165	399	730	204	156	122.97
EABT14133	1126.44	1955.95	1169	641.76	718.55	1371.82	676
EABT14134	1	1	12	5	2	1	2
EABT14135	0	0	1	3	0	0	2
EABT14136	0	2	3	6	0	0	0
EABT14137	84	152	88	14	21	181	81.14
EABT14138	1150.91	2133.3	2434.67	3061.25	1819.85	1066.11	1208.83
EABT14139	0	2	1	1	3	0	1
EABT1414	2	10	17	30	10	2	8
EABT14140	1	2	3	7	1	0	0
EABT14141	1	2	1	0	1	0	0
EABT14142	3	22	14	14	11	1	0
EABT14143	8438.22	9986.99	2485.79	5513.03	2419.29	6544.94	3011.88
EABT14144	234	385	472	2580	296	312	332
EABT14145	1	2	1	0	1	0	0
EABT14146	0	0	2	1	0	0	0
EABT14147	469.02	792.88	383.38	831.14	373.16	258.68	353.72
EABT14148	6	4	8	0	1	6	2
EABT14149	0	3	4	0	1	3	0
EABT1415	0	1	3	0	0	1	2
EABT14150	2436.49	4554.93	5174.65	4032.55	3611.42	1654.64	2374.13
EABT14151	1	11	28	14	1	0	0
EABT14152	0	0	5	1	0	0	0
EABT14153	0	0	2	2	1	0	1
EABT14154	10	28	6	0	0	8	0
EABT14155	0	3	0	13	0	0	0
EABT14156	0	12	9	57	0	4	4
EABT14157	0	0	0	0	0	2	0
EABT14158	15	76	163.46	112	57	38	10
EABT14159	0	0	35	8	1	2	1
EABT1416	0	1	13	1	0	0	0
EABT14160	4002.39	9110.96	2977.07	5526.18	5169.07	26	442
EABT14161	1	1	6	1	4	0	0
EABT14162	511	546.21	5	5	34	502	314.92
EABT14163	0	1	4	1	0	1	0
EABT14164	2	4	4	3	3	0	11
EABT14165	14	305	151.05	54.76	73.01	9	952.88
EABT14166	0	2	0	4	0	0	0
EABT14167	3	7	6	9	0	2	5
EABT14168	21498.9	21101.1	16637.5	24187.3	8660.21	12191.9	8826.7
EABT14169	3416.64	6113.58	1763.7	5478.83	3028.17	2448.19	2868.73
EABT1417	6	4	10	26	3	0	0
EABT14170	0	2	10	1	0	0	0
EABT14171	0	0	7	0	1	2	0
EABT14172	0	0	16	0	2	0	1

EABT14173	203	360.65	41	102.13	413.87	2	20
EABT14174	13	19	1	0	6	27	127
EABT14175	51	38	0	0	5	41	22
EABT14176	3	5	5	4	1	4	7
EABT14177	598.02	761.4	706.2	1057.73	831.76	342.03	492.06
EABT14178	2054	3129.12	4739.88	6258.64	2349.03	1884	1449.99
EABT14179	0	0	11	0	0	1	0
EABT1418	0	0	0	0	0	0	0
EABT14180	0	1	0	5	0	0	0
EABT14181	1	3	10	5	0	0	0
EABT14182	0	0	0	1	0	1	0
EABT14183	0	3	4	1	0	1	1
EABT14184	0	0	10	1	0	0	0
EABT14185	18	44	127	4	4	5	0
EABT14186	0	0	1	1	0	0	0
EABT14187	409.94	493.04	556.05	581	371.56	548.03	409.98
EABT14188	0	0	0	5	0	0	0
EABT14189	14	20	30	5	7	5	7
EABT1419	0	2	5	0	0	0	0
EABT14190	7	10	28	31	4	1	3
EABT14191	0	0	9	0	1	0	0
EABT14192	2123.02	4598.62	4235.61	10875.8	2196.81	1961.3	1907.21
EABT14193	5	2	1	5	0	0	0
EABT14194	0	2	23	3	0	2	0
EABT14195	0	2	6	1	1	0	4
EABT14196	1	1	17	1	0	0	0
EABT14197	0	1	2	6	3	0	0
EABT14198	2	5	9	6	0	0	0
EABT14199	0	0	6	0	0	0	0
EABT142	2066.31	3446.3	2516.92	3329.52	1514.17	1659.45	2793.4
EABT1420	1	1	4	4	0	1	0
EABT14200	0	1	6	0	0	0	0
EABT14201	809	1075.68	1145	3609.6	1046	732	628
EABT14202	0	2	7	0	0	0	2
EABT14203	1	7	4	13	1	6	1
EABT14204	577.02	1047.32	1713.97	3437.87	1535.52	362	1086.02
EABT14205	0	3	5	0	0	0	2
EABT14206	0	1	7	4	0	0	0
EABT14207	18	20	23	49	427	6	8
EABT14208	2	1	5	0	1	0	0
EABT14209	1	1	7	0	0	0	1
EABT1421	5	0	3	0	0	1	2
EABT14210	3	10	8	13	4	4	2
EABT14211	0	0	8	0	0	0	0
EABT14212	2313.82	3339.76	4570.3	4823.95	2798	2404.09	2421
EABT14213	1	4	1	1	2	0	0
EABT14214	2247.01	3269.08	4549.47	3614.94	2243	1958.37	1935
EABT14215	1	0	0	0	2	1	5
EABT14216	1	2	3	6	2	0	2
EABT14217	0	7	1	4	1	2	0
EABT14218	0	4	7	0	4	0	0

EABT14219	12	13	7	0	0	0	0
EABT1422	10555.5	23380.9	39082.8	13230.7	13076.7	2905.44	3186.9
EABT14220	71	111	226	23	21	2	8
EABT14221	0	0	1	2	0	0	0
EABT14222	702.73	591.54	54.99	59.03	251	613.29	1457.55
EABT14223	0	1	8	4	0	1	1
EABT14224	0	0	30	0	0	0	0
EABT14225	0	0	4	0	1	0	0
EABT14226	1584.07	2310.8	636.6	311.99	2472.48	51	306
EABT14227	1	3	26	6	0	0	0
EABT14228	0	6	0	0	5	8	15
EABT14229	10	22	16	35	4	7	8
EABT1423	2	3	1	1	0	1	0
EABT14230	431.74	555.97	419.14	1047.11	366.03	337.03	198.22
EABT14231	0	0	11	8	0	1	0
EABT14232	21	16	20	1	62	72	1939.55
EABT14233	23914.4	23765.2	11786.6	14640.7	62936.6	21436.5	18516.6
EABT14234	0	1	2	0	0	1	0
EABT14235	1	6	9	2	0	0	0
EABT14236	4	1	71	3	3	1	2
EABT14237	2154.01	2680.42	674	907.54	1297.02	2767.58	3319.5
EABT14238	1301	1645.55	2703.05	1892.66	1555.75	898	1120
EABT14239	0	0	0	4	3	2	0
EABT1424	0	3	7	1	1	2	1
EABT14240	1	1	11	1	0	0	0
EABT14241	1565.57	2158.02	2433.2	4682.86	1953.07	1427.05	1705.83
EABT14242	3	1	60	0	1	0	1
EABT14243	831	1148	770	1474.95	877.44	978.27	762
EABT14244	3	7	8	1	1	2	0
EABT14245	0	0	1	0	2	0	0
EABT14246	0	0	10	0	0	0	1
EABT14247	0	1	4	1	3	1	21
EABT14248	11	0	0	0	0	7	26
EABT14249	0	2	35	37	3	1	1
EABT1425	0	0	0	0	7	0	1
EABT14250	0	1	17	2	1	0	0
EABT14251	262	68.98	1	0	20	239	111
EABT14252	22609.9	17579.6	972	254	1642.01	11809.3	5902.99
EABT14253	0	3	1	3	1	1	0
EABT14254	0	0	3	1	1	0	1
EABT14255	3	6	4	49	7	0	1
EABT14256	0	5	5	6	0	3	5
EABT14257	3	12	21	4	11	3	3
EABT14258	4	3	9	4	2	7	1
EABT14259	0	4	2	3	2	3	0
EABT1426	633	1270	962	1836.1	774	645	647.01
EABT14260	3220.69	4973.19	10073	3953.52	3818.86	2937.24	3941.6
EABT14261	66.58	118.71	81.38	120.27	77.64	35.96	68.37
EABT14262	2	5	8	1	1	3	7
EABT14263	1	1	0	0	1	0	0
EABT14264	0	1	0	1	4	0	1

EABT14265	1	2	3	1	0	0	1
EABT14266	2705.61	4559.27	3931.31	6384.44	2376.5	1978.67	2784.91
EABT14267	1	1	6	1	0	0	0
EABT14268	4	3	18	1	0	2	0
EABT14269	0	4	14	26	2	0	1
EABT1427	0	7	3	0	1	0	0
EABT14270	0	0	1	0	0	51	1
EABT14271	0	0	3	0	0	1	0
EABT14272	5462.39	2648	1373.6	3651.02	1574.92	4726.08	3469.04
EABT14273	1	5	3	2	0	0	0
EABT14274	1	3	3	1	0	2	0
EABT14275	1	0	2	1.54	1	1	1
EABT14276	2821.21	712.55	52	13	8	3621.78	47
EABT14277	4	169	91	3	4	0	7
EABT14278	0	5	11	1	0	0	0
EABT14279	8	11	39.02	3	2	6	0
EABT1428	0	0	0	0	0	0	1
EABT14280	1	2	9	14	2	1	1
EABT14281	0	2	1	1	0	0	0
EABT14282	0	4	6	2	1	3	3
EABT14283	1716.07	2417.56	2100.11	1455.97	978	2483.19	2069.73
EABT14284	0	1	19	0	0	0	0
EABT14285	0	0	2	1	0	0	0
EABT14286	145	283	297	805	469	75	279
EABT14287	0	1	1	0	1	0	0
EABT14288	7	11	5	0	1	0	0
EABT14289	8	6	7	14	159	1	0
EABT1429	0	0	42	2	3	0	1
EABT14290	0	0	24	2	1	0	0
EABT14291	0	3	10	7	3	1	2
EABT14292	3	39	25	30	2	15	4
EABT14293	10	17	140	35	5	0	0
EABT14294	61	105	257	1067.92	141	1	51
EABT14295	1	2	17	5	1	0	0
EABT14296	6295.99	14089.7	37648.3	4091.92	5044.12	6897.32	8301.55
EABT14297	0	0	7	2	0	0	0
EABT14298	0	1	36	0	0	0	0
EABT14299	0	1	19	0	0	0	0
EABT143	0	1	3	0	2	0	1
EABT1430	226	366.88	302.89	584.21	259.17	252.26	298.02
EABT14300	1	2	6	1	0	0	3
EABT14301	0	1	1	0	1	1	4
EABT14302	0	1	2	2	1	0	1
EABT14303	0	0	7	0	1	0	0
EABT14304	12	7	0	0	0	0	0
EABT14305	1	5	5	1	0	0	1
EABT14306	6	1	0	2	0	4	1
EABT14307	0	0	4	0	2	1	0
EABT14308	18	0	0	0	0	7	0
EABT14309	0	0	0	0	4	117	61
EABT1431	11	35	33	65	7	8	6

EABT14310	278	429.25	369	1181.99	439	274	236
EABT14311	2	3	3	2	1	0	0
EABT14312	9	20	54	35	9	9	11
EABT14313	0	0	1	0	0	0	0
EABT14314	2514.23	5510.63	8457.05	14943	2998.28	3746.95	3095.2
EABT14315	1	6	9	5	2	0	2
EABT14316	0	0	6	2	0	0	0
EABT14317	0	1	9	1	0	0	0
EABT14318	0	0	3	6	0	0	0
EABT14319	4	4	13	4	1	3	2
EABT1432	4	13	0	2	1	8	1
EABT14320	2	5	15	6	0	2	1
EABT14321	0	0	11	0	1	0	1
EABT14322	1	1	13	0	6	1	5
EABT14323	3	12	31	4	0	19	9
EABT14324	1	2	2	0	0	0	0
EABT14325	5	12	47	10	1	4	3
EABT14326	1192.03	1755.95	731.86	775.44	437.01	784	375
EABT14327	0	0	8	0	0	0	0
EABT14328	0	1	24	1	0	0	1
EABT14329	1005.27	1397.17	2262.22	924.92	610	964	781.34
EABT1433	11	12	0	0	0	0	1
EABT14330	0	0	8	0	0	0	0
EABT14331	2	3	26	6	1	2	0
EABT14332	0	0	9	0	0	0	0
EABT14333	2	3	7	14	4	1	5
EABT14334	0	0	118	2	0	0	0
EABT14335	0	1	16	1	0	0	1
EABT14336	304.95	451.34	377	694.92	370.97	310.01	248
EABT14337	0	2	23	1	0	4	2
EABT14338	7634.11	33558.5	6751.64	4736.35	27517.8	210	7096.18
EABT14339	0	0	9	0	0	0	0
EABT1434	295.38	2217.93	1520.63	3092.8	1084.94	1168.2	668.94
EABT14340	11	20	6	10	5	5	6
EABT14341	0	1	1	0	0	0	4
EABT14342	2	3	1	6	0	0	2
EABT14343	0	0	6	1	0	0	1
EABT14344	12	6	4	1	2	0	0
EABT14345	681	833	568.2	806	765.94	667	651
EABT14346	3	20	30	10	7	4	3
EABT14347	0	0	1	1	1	0	0
EABT14348	0	0	15	0	0	1	1
EABT14349	1	2	24	19	58	1	4
EABT1435	4	1	4	1	29	2	0
EABT14350	0	1	3	1	2	0	0
EABT14351	0	1	2	3	2	0	1
EABT14352	0	2	12	6	2	5	0
EABT14353	0	0	2	2	0	0	1
EABT14354	0	1	454.26	1	0	0	0
EABT14355	1	5	11	40	11	3	2
EABT14356	1	0	7	0	0	2	0

EABT14357	0	1	8	2	0	1	0
EABT14358	0	6	57	3	2	1	0
EABT14359	1	0	9	1	0	1	0
EABT1436	1	3	27	3	0	2	1
EABT14360	0	0	0	5	0	0	1
EABT14361	0	1	1	8	0	2	0
EABT14362	73	67	9	5	4	0	0
EABT14363	9	24	23	74	2	11	19
EABT14364	0	2	0	5	0	0	1
EABT14365	0	1	16	1	0	1	3
EABT14366	6	18	48	96	5	2	10.15
EABT14367	1	3	12	5	0	0	1
EABT14368	1	3	12	39	7	2	1
EABT14369	204	118	71	489	138	60	95
EABT1437	4	5	0	6	0	0	0
EABT14370	16	0	0	0	0	1	0
EABT14371	0	1	25	3	0	1	1
EABT14372	11	25	14	16	18	5	4
EABT14373	0	0	11	3	1	1	2
EABT14374	0	0	7	0	2	0	1
EABT14375	3769	3539.98	299.52	212	2421	8171.93	16672.2
EABT14376	1	0	3	0	0	0	0
EABT14377	0	1	3	3	0	0	0
EABT14378	0	0	1	1	2	0	1
EABT14379	1.09	2	0	0	0	4	1
EABT1438	0	0	2	2	0	2	1
EABT14380	1	0	14	7	3	0	2
EABT14381	1	0	2	0	2	1	0
EABT14382	7	18	5	0	4	10	23
EABT14383	62	117	134	3008.03	59	4	10
EABT14384	5878.73	8493.06	7960.39	1928.72	3941.44	3881.87	3550.83
EABT14385	2	2	25	0	2	3	0
EABT14386	2055	3060.1	2414.87	3206	1603.49	2276	1870
EABT14387	76	113.15	0	0	2	7	26
EABT14388	0	0	2	4	7	0	0
EABT14389	47	84	35	114	8	43.42	27
EABT1439	3	4	15	3	0	2	4
EABT14390	3	27	53	167.56	382.24	2	3
EABT14391	2	3	21	8	8	4	10
EABT14392	3180.75	2673.32	218.41	366.11	224.09	5991.96	267.75
EABT14393	12	43	27	59	7	6	6
EABT14394	1	0	5	5	0	0	0
EABT14395	6	2	17	0	0	1	0
EABT14396	10	35	7	5	4	3	1
EABT14397	1	5	28	5	5	3	3
EABT14398	1	0	1	0	0	0	0
EABT14399	1	9	9	0	0	0	0
EABT144	3031.19	1287	1460.36	4270.2	221	3389.98	1245.93
EABT1440	6879.85	2433.97	12	1	58	7066.63	199
EABT14400	2	7	0	0	0	0	0
EABT14401	3	1	3	1	0	1	0

EABT14402	4	3	6	4	2	11	4
EABT14403	1	6	1	0	0	8	1
EABT14404	0	0	6	10	10	0	2
EABT14405	0	13	65.99	13	0	6	4
EABT14406	5	7	27	0	0	1	0
EABT14407	4	8	11	17	3	10	5
EABT14408	31	11	1	15	2	10	7
EABT14409	205	294.07	415.82	530.81	374	127	144
EABT1441	1862.99	4742.02	10795.8	2533.94	172	22	280.58
EABT14410	1	0	3	1	1	2	1
EABT14411	0	0	0	1	1	0	5
EABT14412	5	2	2	2	2	0	0
EABT14413	0	0	9	1	1	0	0
EABT14414	1	2	16	1	1	1	1
EABT14415	0	5	11	10	2	0	10
EABT14416	0	5	3	19	0	0	1
EABT14417	3	0	0	0	0	9	0
EABT14418	1	3	48.06	7	0	0	1
EABT14419	5	34	5	2	3	21	16
EABT1442	2546.5	301.07	60	1	1	1181.29	0
EABT14420	31	359.16	1106.87	118	254	22.68	569
EABT14421	0	0	0	4	2	1	0
EABT14422	307.77	1068.51	972.84	493.82	955.12	438.99	859.75
EABT14423	5	10	31	9	2	1	4
EABT14424	10	44	75	163	38	6	5
EABT14425	0	4	10	5	1	0	1
EABT14426	1637.44	1855.42	912.64	1177.19	961.01	2348.65	1646.76
EABT14427	0	0	13	0	2	1	0
EABT14428	53	153	200.12	198	62	48	177.69
EABT14429	8652.1	7829.78	2717.72	3496.77	5358.35	9599.82	8331.25
EABT1443	4	1	1	1	7	1	2
EABT14430	0	1	5	1	0	0	0
EABT14431	471	606	142	262	455	233	740.48
EABT14432	0	1	1	5	0	0	1
EABT14433	0	5	2	3	0	1	1
EABT14434	1	10	19	5	5	4	7
EABT14435	1	1	2	5	0	1	1
EABT14436	1	2	3	3	0	0	1
EABT14437	4654.49	6049.2	4805.13	2017.82	2667.53	1902.15	2367.58
EABT14438	13	28	448.87	42	2	1	0
EABT14439	311	316	155	380	286	102	189
EABT1444	1	1	19	4	0	0	0
EABT14440	0	2	22	4	2	0	7
EABT14441	6	8	19	3	1	0	5
EABT14442	3083.32	2168	1054	2265.92	1083	2760.92	2237.38
EABT14443	1	12	35	1	4	0	2
EABT14444	2	3	1	0	0	0	0
EABT14445	0	0	9	1	0	0	0
EABT14446	2	1	0	0	4	0	5
EABT14447	0	4	2	4	2	0	2
EABT14448	2	0	14	2	3	0	0

EABT14449	0	0	4	2	1	1	0
EABT1445	0	0	1	2	0	0	2
EABT14450	0	0	14	6	0	0	1
EABT14451	3	3	11	15	6	2	3
EABT14452	2	4	5	5	0	0	12
EABT14453	0	2	3	53	1	2	1
EABT14454	0	2	1	3	0	0	0
EABT14455	141	244	498	2222.06	361.19	201	675
EABT14456	0	2	9	3	0	0	1
EABT14457	0	2	2	0	0	6	1
EABT14458	4	5	18	5	1	3	2
EABT14459	1	6	7	20.01	0	1	0
EABT1446	0	0	6	0	0	0	1
EABT14460	6	17	28	12	41	2	22
EABT14461	0	0	6	4	4	0	0
EABT14462	2	7	43	27	2	0	3
EABT14463	1	1	15	4	1	0	0
EABT14464	414	644.28	755.45	1217.78	569.1	383.06	290.6
EABT14465	1	2	3	3	0	15	3
EABT14466	1	0	5	0	1	1	0
EABT14467	0	3	2	1	0	1	1
EABT14468	7	48	60	98	9	11	5
EABT14469	38	28	18	12	5	6	16
EABT1447	0	4	6	2	1	0	1
EABT14470	10	19.01	24.91	21	22	18	13
EABT14471	4	9	13	17	10	2	3
EABT14472	0	1	6	26	3	1	3
EABT14473	8	15	8	20	15	1	0
EABT14474	1358	1172	1	0	0	689	0
EABT14475	0	2	42	7	10	3	5
EABT14476	1	2	6	2	0	0	0
EABT14477	0	5	12	2	0	0	0
EABT14478	3	1	0	0	1	7	0
EABT14479	0	2	16	3	0	0	0
EABT1448	0	1	4	3	0	0	1
EABT14480	1	5	6	7	2	1	2.31
EABT14481	0	0	24	0	0	0	0
EABT14482	0	0	19	24	0	0	0
EABT14483	4196.15	6994.53	5945.99	8514.74	1097.02	474.08	820.97
EABT14484	1	1	0	0	2	0	0
EABT14485	3	5	8	11	1	4	4
EABT14486	5	13	22	20	37	6	13
EABT14487	6	3	86	35	0	0	0
EABT14488	2145.95	2937.96	3045.18	3010.87	1992.82	1528.37	1798.27
EABT14489	59.01	124.62	58.73	108	94.16	56	52.08
EABT1449	0	0	7	0	0	0	0
EABT14490	0	0	7	0	0	0	0
EABT14491	1	1	4	0	0	0	0
EABT14492	150.93	597.99	760	270.01	203.99	33	71
EABT14493	95.01	74.04	79	88	63.01	39	54
EABT14494	0	0	0	1	3	0	1

EABT14495	5	16	44	24	8	17	8
EABT14496	0	2	6	0	1	0	0
EABT14497	0	3	3	5	2	1	1
EABT14498	0	2	9	1	1	1	4
EABT14499	0	0	4	1	1	0	3
EABT145	1287	1235.41	1758.97	1342	718	40	481
EABT1450	1	0	2	4	0	1	0
EABT14500	2	4	12	0	4	0	2
EABT14501	1	0	1	3	1	0	0
EABT14502	0	3	0	4	1	0	0
EABT14503	6	6	2	10	7	4	7
EABT14504	3	4	26	3	2	2	0
EABT14505	15	46	72.58	60	18.96	8	6
EABT14506	425.34	1520.06	1805	721	572	336	400.99
EABT14507	6	3	4	0	2	5	3
EABT14508	11	32.18	29	12	1	0	2
EABT14509	197	262	506	1082.99	263	90.01	91
EABT1451	0	0	5	1	1	2	0
EABT14510	0	3	7	0	0	0	0
EABT14511	1	4	90	16	1	2	0
EABT14512	0	1	17	0	2	0	0
EABT14513	0	0	0	3	0	0	0
EABT14514	11	35	16	29	0	0	0
EABT14515	0	0	6	0	0	0	0
EABT14516	1	6	11	0	4	0	3
EABT14517	0	1	11	1	0	0	0
EABT14518	1	2	41	0	1	0	4
EABT14519	551.52	920.37	785.6	2323.35	948.89	465.83	523.59
EABT1452	380	426	241	504.47	199	199.01	322
EABT14520	0	2	5	3	2	1	0
EABT14521	0	1	0	0	0	2	5
EABT14522	1	0	6	0	0	0	0
EABT14523	1	6	14	0	0	3	1
EABT14524	667	1002.45	161	216	348.18	1148	1116.07
EABT14525	358.79	522	451.01	1140.47	443	523	445.36
EABT14526	0	3	9	0	1	0	0
EABT14527	0	0	1	0	0	0	0
EABT14528	31	16	3	5	10	3	25
EABT14529	345	371.99	540.03	208	106	244.29	234
EABT1453	2	8	26	14	7	1	8
EABT14530	0	4	4	4	4	0	5
EABT14531	0	5	2	0	0	0	6
EABT14532	489	391.24	5	0	0	196	2
EABT14533	1808.97	3177.86	3145.87	5248.35	2890.65	1968.97	2745.76
EABT14534	81	16	1	1	0	0	0
EABT14535	0	0	4	0	3	0	0
EABT14536	0	0	18	2	1	0	0
EABT14537	0	0	2	3	1	0	0
EABT14538	1	1	6	2	2	3	1
EABT14539	2	1	2	1	0	0	0
EABT1454	3	4	19	5	3	0	1

EABT14540	0	1	3	3	0	1	0
EABT14541	528	473	2071	643.01	168.05	1	3
EABT14542	1	4	7	7	0	5	0
EABT14543	0	2	2	0	1	0	0
EABT14544	0	2	9	2	0	0	1
EABT14545	1299.76	1219	234.17	170	1603.01	1	37
EABT14546	2	2	38	6	0	0	0
EABT14547	0	1	2	0	1	0	1
EABT14548	6	10	9	11	7	4	9
EABT14549	3	5	4	0	0	16	3
EABT1455	1	0	0	4	1	0	0
EABT14550	0	0	0	0	0	0	0
EABT14551	0	0	0	0	0	0	0
EABT14552	66	24	22	67	3257.48	24	15
EABT14553	0	3	3	2	2	0	0
EABT14554	508	1049.93	734	117	3804.68	425.01	366
EABT14555	0	0	13	0	0	0	0
EABT14556	2	3	2	1	0	0	0
EABT14557	0	0	0	0	0	0	0
EABT14558	0	2	5	0	0	1	0
EABT14559	0	6	32	34	1	1	0
EABT1456	6	32	58	1	1	14	6
EABT14560	0	0	0	1	1	0	0
EABT14561	58	82	140	33	65	79	108
EABT14562	0	3	26	2	0	0	0
EABT14563	1	1	9	1	0	0	0
EABT14564	0	1	0	0	0	8	0
EABT14565	1	3	0	2	0	0	0
EABT14566	1	7.98	29	2	1	1	0
EABT14567	1	11	2	0	2	2	11
EABT14568	12	28	189	199.95	67	16	10
EABT14569	2	3	6.04	6	1	4	2
EABT1457	0	0	20	0	1	0	0
EABT14570	2	7	2	13	7	0	3
EABT14571	0	0	1	0	0	0	0
EABT14572	5668	8716.96	10036.9	9000.94	8110.01	5594.99	4872.31
EABT14573	0	1	2	3	1	0	0
EABT14574	1	1	0	1	3	1	16
EABT14575	0	2	17	0	2	0	0
EABT14576	0	0	0	0	1	0	0
EABT14577	1086.01	156.5	4	0	4	599.15	74
EABT14578	15	19	15	35	4	8	16
EABT14579	0	0	4	0	0	0	0
EABT1458	2	2	9	7	1	2	1
EABT14580	2	7	11	56	12	0	0
EABT14581	0	4	9	65	3	5	0
EABT14582	862.98	1839.78	3811.54	5178.24	2697.37	1013.69	1419.58
EABT14583	1	36	20	23	1	14	5
EABT14584	1	1	3	1	0	2	0
EABT14585	3	3	16	4	0	0	1
EABT14586	0	0	6	1	0	0	0

EABT14587	1	7	8	13	5	0	0
EABT14588	5	16	4	14	2	0	3
EABT14589	2	7	8	0	0	4	1
EABT1459	0	2	2	3	0	0	1
EABT14590	121.01	373.77	23	50	29	495.97	87
EABT14591	6318.87	3933.01	2197.03	8260.25	1010.99	118.48	268
EABT14592	30	49	562.15	19	7	60	26
EABT14593	0	0	10	1	0	0	0
EABT14594	0	0	5	2	0	0	0
EABT14595	2	0	3	5	0	0	0
EABT14596	0	0	3	0	1	0	0
EABT14597	0	0	6	0	0	0	2
EABT14598	0	1	3	0	0	0	0
EABT14599	0	1	16	1	0	0	0
EABT146	734	1114.53	929.99	193	240	6	43
EABT1460	0	1	6	0	0	0	0
EABT14600	7	6	2	2	0	1	1
EABT14601	0	2	6	2	3	0	1
EABT14602	0	0	9	0	0	0	0
EABT14603	2201.57	3567.37	2845.44	4329.34	2594.02	2617.98	1999
EABT14604	4	4	0	2	0	11	0
EABT14605	101	149	114	260	248	60.83	78
EABT14606	10	20	39	8	8	4	5
EABT14607	2	0	4	2	0	0	0
EABT14608	2	3	0	0	3	16	29
EABT14609	7	11	8	31	17	2	3
EABT1461	0	3	2	6	12	0	1
EABT14610	2	2	1	0	0	0	8
EABT14611	1	2	52	2	1	1	3
EABT14612	1	0	0	7	2	0	1
EABT14613	0	0	11	0	0	0	1
EABT14614	0	1	0	0	0	0	20
EABT14615	0	1	0	2	2	0	0
EABT14616	1431.82	2119.39	478.49	1312.52	2232.99	1729.98	135.05
EABT14617	6388.96	4611.46	186	7	562.97	5237.04	929.01
EABT14618	1	3	16	3	0	0	2
EABT14619	1	3	7	3	0	0	0
EABT1462	8	21	1	6	1	7	31
EABT14620	1	4	167.7	2	0	0	0
EABT14621	0	7	4	30	0	0	10
EABT14622	1	2	15	2	27	0	2
EABT14623	3	3	0	0	0	1	0
EABT14624	5	6	32	5	4	0	3
EABT14625	0	2	3	13	1	0	2
EABT14626	0	0	4	8	0	0	0
EABT14627	49	108	43	57	56	48	81
EABT14628	39	102	188	278	26	0	0
EABT14629	0	0	6	3	1	2	6
EABT1463	1	0	5	2	0	0	0
EABT14630	2498.64	3685.53	4398.84	5559.44	3598.38	1247.97	1484.23
EABT14631	4	1	22	3	1	2	5

EABT14632	0	0	6	0	5	1	0
EABT14633	1831.6	1803.78	1277.49	2693.2	1288	1873.64	1877.9
EABT14634	1	0	2	4	0	0	0
EABT14635	0	0	5	0	0	0	0
EABT14636	0	1	8	0	0	0	0
EABT14637	19	12	61	26	9	136	1
EABT14638	7	6	3	3	2	2	54
EABT14639	5	7	9	5	4	9	2
EABT1464	0	3	3.03	3	1	1	2
EABT14640	1	1	2	3	1	0	1
EABT14641	0	2	13	2	0	0	1
EABT14642	2	4	10	8	0	1	2
EABT14643	582.62	1137.9	516	1159.78	465.96	471.01	518.1
EABT14644	1595	2763.25	6665.94	3359.74	3722.17	2280.29	3276.36
EABT14645	0	3	6	2	1	0	0
EABT14646	383.02	442.45	149	1870.64	241.99	342.39	152.19
EABT14647	11	24	6	5	3	36	4
EABT14648	1	4	2	0	1	0	0
EABT14649	1	5	0	2	0	0	1
EABT1465	2	6	8	3	0	0	0
EABT14650	0	0	16	0	0	0	1
EABT14651	1	0	7	2	0	0	1
EABT14652	0	3	4	10	0	1	2
EABT14653	2	8	7	11	0	1	1
EABT14654	3	12	4	3	2	10	0
EABT14655	0	0	16	0	0	0	0
EABT14656	0	0	4	5	0	0	0
EABT14657	8	0	0	0	0	18	3
EABT14658	1	1	1	3	6	0	2
EABT14659	0	4	3	0	0	0	0
EABT1466	1	1	5	2	0	1	0
EABT14660	2	9	0	0	0	10	6
EABT14661	4	1	1	3	0	5	0
EABT14662	2	2	8	5	0	6	1
EABT14663	0	1	7	1	2	1	0
EABT14664	0	0	6	0	0	0	0
EABT14665	13400.9	6479.32	3197.18	8017.25	3125.26	578	1048.03
EABT14666	0	0	11	1	0	1	0
EABT14667	1	4	7	0	0	0	2
EABT14668	0	0	3	0	0	3	0
EABT14669	1	4	0	13	3	0	2
EABT1467	0	0	12	0	0	0	0
EABT14670	4	2	24	0	1	0	0
EABT14671	1	4	0	4	1	1	14
EABT14672	0	3	3	2	0	0	0
EABT14673	2	3	4	4	1	1	0
EABT14674	38	79	117.51	1150.04	338	10	51
EABT14675	1	5	18	1	2	2	2
EABT14676	1	1	4	0	2	1	0
EABT14677	0	2	3	3	0	0	0
EABT14678	15	13	0	0	1	21	3

EABT14679	2	1	1	0	4	0	0
EABT1468	13.99	36	39	71.02	17	18	13
EABT14680	13400.5	11167.6	6962.99	9390.07	6619.87	11076.7	12028.8
EABT14681	2	8	66	0	1	5	0
EABT14682	2	5	19	17	9	0	1
EABT14683	3	0	1	0	0	2	1
EABT14684	600.52	1828.56	2559.1	2805.53	547.93	427	564.98
EABT14685	2103.62	2977.56	2618.58	3379.25	2448.54	1316.66	1766.95
EABT14686	2	22	56	19	2	9	10
EABT14687	4	5	174	23	1	3	0
EABT14688	0	0	9	0	0	0	2
EABT14689	3	0	3	0	0	1	0
EABT1469	1	11	1	2	2	2	0
EABT14690	10	4	2	1	0	10	1
EABT14691	15	0	0	0	0	16	0
EABT14692	0	2	2	14	4	0	0
EABT14693	0	6	13	2	0	0	1
EABT14694	1	1	21.14	14	3	0	1
EABT14695	15	7	1	0	1	17	81
EABT14696	1489.19	2403.01	1707.96	2480.73	893.29	1421.12	690
EABT14697	1	3	16	0	0	2	0
EABT14698	2	6	10	18	3	2	1
EABT14699	0	2	1	1	0	0	4
EABT147	103	161.12	164.01	351.51	138.38	79.45	112
EABT1470	2	1	2	0	0	2	2
EABT14700	2	2	7	0	0	1	0
EABT14701	2	11	3	2	0	1	2
EABT14702	2	5	3	1	0	6	0
EABT14703	1	5	25	4	2	2	0
EABT14704	2	8	9	10	4	3	1
EABT14705	5	5	18	1	7	1	4
EABT14706	0	0	0	2	0	0	5
EABT14707	0	0	2	0	0	0	0
EABT14708	0	0	28	0	0	0	0
EABT14709	1	1	1	367	119	0	5
EABT1471	0	0	0	0	0	0	0
EABT14710	1	0	7	0	0	0	0
EABT14711	390	570	583	690	293	295.56	275
EABT14712	1	3	28.04	0	0	0	0
EABT14713	0	0	3	2	0	0	0
EABT14714	0	0	9	3	0	0	0
EABT14715	713.95	1436.54	2201.93	3828.45	654.49	536	471
EABT14716	2	0	1	7	0	0	0
EABT14717	0	1	2	0	0	0	0
EABT14718	1485.82	2476.17	1733.01	3924.23	1810.04	928.14	1223.38
EABT14719	1	1	0	1	0	4	0
EABT1472	582.36	253.23	12	1	0	174.82	0
EABT14720	0	5	2	1	1	0	0
EABT14721	0	0	7	4	0	1	0
EABT14722	0	0	7	0	0	0	0
EABT14723	1	9	130.98	116	9	3	13

EABT14724	0	0	0	0	0	0	0
EABT14725	0	7	11	4	0	2	2
EABT14726	72	140.87	104	36.88	48.02	19	22
EABT14727	0	1	7	0	0	0	0
EABT14728	227.94	133.99	49	79	20	291	6
EABT14729	0	7	7	2	0	0	5
EABT1473	3	1	1	0	0	6	0
EABT14730	19846.8	28291.5	14024.9	7975.4	6339.1	2879.77	1534.77
EABT14731	4	3	7	1	3	5	8
EABT14732	0	0	36	0	0	0	0
EABT14733	0	1	4	0	0	0	0
EABT14734	0	0	8	6	0	0	0
EABT14735	5	6	6	15	19	6	3
EABT14736	2	3	0	0	0	0	0
EABT14737	0	3	50	5	0	0	0
EABT14738	889.34	1429.51	2035.16	2750.16	1904.91	899.85	1060.16
EABT14739	1	3	3	0	0	0	0
EABT1474	5283.93	7970.93	10804.3	16542.5	5760.39	4717.05	5046.28
EABT14740	3	25	50	114	51	2	5
EABT14741	9	2	25	3	0	5	3
EABT14742	0	0	6	1	0	0	0
EABT14743	0	1	0	3	0	0	6
EABT14744	2114.18	4160.29	3555.16	6638.72	2201.94	1528.44	1863.93
EABT14745	0	4	8	1	0	0	0
EABT14746	5	9	10	4	0	0	0
EABT14747	157.05	263	285.97	294.07	97.17	164.11	135.75
EABT14748	647.99	1203	1789.92	1925.2	821	647	645
EABT14749	2	7	5	24	3	3	1
EABT1475	19	18	0	0	0	3	3
EABT14750	2	2	0	0	0	0	14
EABT14751	1	3	2	1	0	1	0
EABT14752	0	4	21	4.99	0	2	3
EABT14753	1	3	1	8	6	0	0
EABT14754	3447.76	5521.13	3974.81	7646.67	5205.91	2307.57	3671.43
EABT14755	84	49	1	0	1	40	54
EABT14756	0	1	0	2	3	0	1
EABT14757	2	12	5	6	1	5	2
EABT14758	1	0	1	4	1	0	2
EABT14759	1174.16	721.07	727.44	2947.38	352.61	226	201.72
EABT1476	2	3	8	1	1	0	0
EABT14760	1	1	7	0	0	0	0
EABT14761	193.91	540	436.24	867.14	219	176	685
EABT14762	1	0	1	0	0	0	0
EABT14763	7	5	10	2	3	2	1.96
EABT14764	0	0	0	0	0	0	0
EABT14765	0	2	9	0	0	0	1
EABT14766	0	1	7	0	0	1	1
EABT14767	1	2.01	5	1	1	1	3
EABT14768	0	2	2	2	4	1	1
EABT14769	0	1	12	2	1	0	1
EABT1477	0	3	37	0	0	0	0

EABT14770	2367.73	4976.28	12426	10043	2845.4	2675.74	2223.54
EABT14771	829	4035.96	354	12	450.98	55	2689.84
EABT14772	0	0	1	0	0	2	0
EABT14773	1	0	4	6	0	0	0
EABT14774	1	0	9	1	0	2	1
EABT14775	0	1	5	6	1	0	2
EABT14776	1	1	2	0	1	2	0
EABT14777	425.91	896.42	1005.45	4602.09	638.22	353.17	436.34
EABT14778	6	63	1	0	0	0	1
EABT14779	0	0	0	0	0	0	11
EABT1478	2	2	5	1	7	0	2
EABT14780	2	5	10	5	2	3	1
EABT14781	2471.41	2809.96	1806.24	2502.28	2461.15	1615.23	1980.67
EABT14782	3	5	0	1	0	4	3
EABT14783	1	2	4	0	0	1	1
EABT14784	3	6	0	0	0	3	5
EABT14785	0	2	0	3	11	0	0
EABT14786	19	27	15	17.97	3	19	11
EABT14787	0	0	0	0	1	0	1
EABT14788	4	3	7	26	4	2	3
EABT14789	7	4	16	3	0	4	5
EABT1479	0	0	1	0	0	0	0
EABT14790	3	1	2	0	0	2	1
EABT14791	1	0	1	0	3	0	0
EABT14792	0	1	5	1	3	0	1
EABT14793	21	36	133.01	275	14	13	17
EABT14794	0	1	3	4	2	0	3
EABT14795	0	0	4	0	0	1	0
EABT14796	0	1	14	2	1	1	4
EABT14797	7	16.99	0	11	6	3	4
EABT14798	6	18	34	3	0	1	1
EABT14799	0	0	1	1	6	0	3
EABT148	10	2	3	1	0	4	0
EABT1480	0	0	5	0	0	1	1
EABT14800	2	1	1	15	3	0	0
EABT14801	1	2	37	0	2	1	2
EABT14802	1	0	14	2	0	3	0
EABT14803	2	0	1	0	0	1	0
EABT14804	5	14	23	23	9	1	1
EABT14805	0	4	2	5	1	0	0
EABT14806	0	0	3	1	0	1	0
EABT14807	0	1	3	2	0	0	0
EABT14808	3	0	11	2	0	0	0
EABT14809	3	1	1	1	2	1	1
EABT1481	0	0	5	1	0	0	0
EABT14810	5	8	1	0	0	4	0
EABT14811	3187	4625.42	4211	3187.99	4694	1948	2897
EABT14812	0	1	0	4	0	1	1
EABT14813	1	2	2	3	2	0	0
EABT14814	2	3	10	8	1	0	0
EABT14815	0	1	7	0	0	0	0

EABT14816	1	0	12	2	0	0	0
EABT14817	0	1	2	0	0	0	0
EABT14818	18	11	75	4	12	1	5
EABT14819	2169.31	3773.53	3624.98	5593.07	3275.41	1607.88	1809.13
EABT1482	12	50	158	15	4	26	9
EABT14820	2	3	29	3	0	5	5
EABT14821	4	8	5	5	1	6	1
EABT14822	0	1	5	1	0	0	0
EABT14823	1	0	14	4	2	2	0
EABT14824	1	0	1	4	0	0	0
EABT14825	0	0	5	0	1	0	0
EABT14826	0	2	4	1	0	0	0
EABT14827	159	219	181	594	276.88	179	672.02
EABT14828	0	0	5	0	0	0	0
EABT14829	1360.54	1828.13	2306.51	3564.24	1696.97	1080.11	1046.03
EABT1483	455.34	661.96	936.97	1478	584.43	333.99	352.99
EABT14830	2859.37	4058.02	4817	1989	1875	2605.18	3292.54
EABT14831	749.85	869.81	883.47	446.21	1049.01	31	206
EABT14832	2	3	2	5	4906.01	4	3
EABT14833	0	0	1	2	0	0	0
EABT14834	1218	1572.97	1585	4077.52	1076	1103.02	1190
EABT14835	1	1	5	1	1	0	0
EABT14836	0	2	11	3	0	0	0
EABT14837	119	942	88	432	30	49	24
EABT14838	0	0	3	2	0	0	0
EABT14839	1	2	7	1	0	1	1
EABT1484	1	21	28.03	10	6.96	2	3
EABT14840	0	0	10	0	0	0	0
EABT14841	5	14	11	53	4	2	4
EABT14842	4	5	3	3	3	1	0
EABT14843	1	15	49	8	0	1	1
EABT14844	0	0	5	0	0	0	0
EABT14845	0	3	0	5	2	0	0
EABT14846	0	0	8	0	0	0	0
EABT14847	77	120	192	323.01	112.6	12	33
EABT14848	10	33	188	37	12	14	11
EABT14849	54198.1	48105	30290.2	28207.1	16935.8	52362.1	36590.8
EABT1485	0	0	0	7	1	0	0
EABT14850	0	0	6	2	0	0	0
EABT14851	1	3	4	0	0	4	0
EABT14852	3	1	9	12	2	1	0
EABT14853	0	5	7	19	2	2	1
EABT14854	1	0	3	3	1	0	0
EABT14855	0	0	2	1	1	0	0
EABT14856	0	3	45	2	3481	2	1
EABT14857	2	1	2	2	12	1	8.99
EABT14858	2	12	133	597	31	2	1
EABT14859	10	8	31	4	1	4	2
EABT1486	4	1	0	2	2	1	1
EABT14860	0	0	7	0	0	0	0
EABT14861	4	27	35	37	40	1	1

EABT14862	31780.3	37543.3	21129.3	16350.2	80120.7	13721.7	13904.2
EABT14863	1886.25	2538.69	1196.96	2250.14	2234.03	1189.21	2129
EABT14864	577.79	1275.16	2070.75	2612.1	1202.5	824.08	807.05
EABT14865	464	722	970.35	922.11	557.01	11	389.9
EABT14866	4	19	116.69	9	69.29	5.98	15.36
EABT14867	0	3	39	4	1	0	2
EABT14868	1	8	3	1	1	0	0
EABT14869	0	1	1	1	4	0	0
EABT1487	1	5	7	10	1	0	0
EABT14870	0	8	6	1	0	0	2
EABT14871	1	0	2	0	0	0	2
EABT14872	1	2	6	6	0	1	1
EABT14873	1	2	12	3	0	2	0
EABT14874	601	673.06	910	399	478	4	114
EABT14875	1	5	9	11	0	0	0
EABT14876	0	2	7	0	0	0	0
EABT14877	2	3	3	0	1	2	3
EABT14878	0	2	10	2	1	0	0
EABT14879	0	1	9	0	1	0	0
EABT1488	0	1	1	0	5	0	0
EABT14880	2	1	3	0	1	0	1
EABT14881	2	6	14	5	4	0	2
EABT14882	1	0	2	2	0	0	0
EABT14883	2	2	1	1	2	2	9
EABT14884	479.45	736.65	6618.66	1793.28	1088.23	46	70.79
EABT14885	0	0	7	0	0	0	0
EABT14886	5	0	28	5	0	3	2
EABT14887	2	3	21	11	2	1	2
EABT14888	0	0	9	3	0	1	1
EABT14889	0	2	4	2	0	1	0
EABT1489	1	0	8	0	0	0	0
EABT14890	0	2	6	3	3	0	1
EABT14891	4767.96	3380.65	5431.99	3579.67	3382.72	3250.1	3579.14
EABT14892	0	0	3	0	1	0	0
EABT14893	3	7	40	5	5	1	3
EABT14894	0	0	22	0	0	0	0
EABT14895	175	366.12	877.18	606.34	577.28	249.89	326.29
EABT14896	9	28	26	11	18	6	7
EABT14897	446.01	667.67	366.97	1158.12	562.74	295.22	291.07
EABT14898	5	12	23	8	41	2	21
EABT14899	1124	2815.52	3686.9	287	672.2	325	122
EABT149	0	1	3	0	0	1	1
EABT1490	112416	85486.5	134	15	52	36607.7	904.5
EABT14900	1	2	0	0	0	9	3
EABT14901	1	8	1	3	2	3	9
EABT14902	1	0	8	4	0	1.99	0
EABT14903	351.59	509.54	31	7	54.02	829.09	233.84
EABT14904	4	1	10	0	0	1	0
EABT14905	4	9	12	1	0	3	2
EABT14906	18	5	20	2	0	0	0
EABT14907	1	3	20	1	1	0	1

EABT14908	2723.16	4834.07	4472.66	6712.19	3489.15	3264.92	3934.56
EABT14909	790	751.81	462	794	558	363	451.25
EABT1491	0	5	5	1	4	0	0
EABT14910	1	1	1	0	0	0	1
EABT14911	0	6	4	2	4	2	0
EABT14912	251	266	411	427.2	426	117	120
EABT14913	2	4	1	4	0	3	2
EABT14914	0	0	1	1	0	0	3
EABT14915	1	0	3	0	2	0	0
EABT14916	1	0	10	2	0	0	0
EABT14917	0	20	2	0	4	2	1
EABT14918	9	7.98	1	12	3	1	1
EABT14919	2	0	0	4	0	2	1
EABT1492	4	5	1	5	1	0	2
EABT14920	172	201	96	400	174	44	98
EABT14921	40	44	49	37	14	34	32
EABT14922	0	0	2	3	0	0	0
EABT14923	1	5	4	1	0	1	0
EABT14924	0	2	30	4	0	0	0
EABT14925	0	0	6	7	1	1	0
EABT14926	1648.03	2370.97	1971.99	1941.99	1724.34	1401.17	1313.89
EABT14927	0	1	8	4	0	0	0
EABT14928	0	0	2	2	4500.94	2	118
EABT14929	0	0	7	0	0	0	0
EABT1493	503	1050	2132.77	7946.18	3027.52	27	54
EABT14930	41	32	23	19	127	3	3
EABT14931	1	2	10	5	0	5	0
EABT14932	3	0	7	4	1	2	1
EABT14933	684	893	235	1286.88	425	583	793
EABT14934	36	53	0	0	1	19	30
EABT14935	10	3	1	0	0	0	0
EABT14936	0	1	1	0	0	0	0
EABT14937	0	1	4	2	0	0	0
EABT14938	906	1275	1221	2824	1210.96	825	1497.89
EABT14939	167	411	614.5	555	220	54	370
EABT1494	5466.61	7677.39	3758.41	12617.8	6242.21	3186.19	4380.25
EABT14940	1679	2174.7	835.69	3009.01	774	798.01	772.19
EABT14941	0	2	6	2	0	0	0
EABT14942	0	1	8	3	0	0	0
EABT14943	129.38	248.23	198.94	613.99	180.02	19	88
EABT14944	40	64	310.68	942.02	178	217	424
EABT14945	7	6	21	4	0	2	0
EABT14946	0	0	4	4	1	0	0
EABT14947	37	139	111	252.19	13	6	16
EABT14948	3	2	2	1	0	5	8
EABT14949	117.97	37	5	4	7	94	51
EABT1495	0	2	36	1	0	0	0
EABT14950	0	2	25	2	1	4	0
EABT14951	0	3	92	4	1	2	2
EABT14952	0	2	1	0	0	0	1
EABT14953	0	2	4	0	0	0	1

EABT14954	1	1	4	2	0	0	1
EABT14955	0	1	7	0	1	5	1
EABT14956	7	14	135	3	5	12	1
EABT14957	487.99	615.02	397.91	868.82	725.01	363.1	370.41
EABT14958	0	1	22	5	3	0	0
EABT14959	0	0	0	0	2	0	2
EABT1496	0	7	16	1	3	0	1
EABT14960	0	0	33	0	0	3	0
EABT14961	7	21	26	16	2	9	12
EABT14962	0	0	8	0	0	1	1
EABT14963	0	1	0	0	20	14	0
EABT14964	32	41.85	25	12	1	6	0
EABT14965	0	0	11	8	0	0	0
EABT14966	3	9	3	3	5	2	5
EABT14967	2	4	7	4	3	0	2
EABT14968	1044	2088.75	778.93	2555.89	2513.54	143	1907
EABT14969	45164.6	11744.6	1254.8	9448.66	12919.6	286.01	717
EABT1497	32	222.03	20	0	11	0	0
EABT14970	1	1	2	4	0	0	0
EABT14971	5	15	0	0	4	42	35
EABT14972	242	363.99	282	1008.96	567.95	243	252.96
EABT14973	2	5	20	16	11	1	6
EABT14974	168	336.99	437.03	88.01	89	125	280
EABT14975	0	4	1	0	1	0	1
EABT14976	2	5	0	1	0	3	0
EABT14977	2	0	9	4	0	0	1
EABT14978	1	1	2	1	3	1	1
EABT14979	1	0	2	0	0	2	0
EABT1498	0	0	0	11	3	0	0
EABT14980	11	28	23	17	3	54	33.96
EABT14981	4343.3	9353.69	6179.57	6474.17	3808.77	3757.3	4022.89
EABT14982	27	44	54	118	39	16	23
EABT14983	0	1	0	0	1	20	14
EABT14984	1	3	2	0	0	0	2
EABT14985	0	0	25	0	0	0	0
EABT14986	0	2	25	4	44	0	0
EABT14987	8	21	12	45.98	119	14	33
EABT14988	46	22	20	43	7	7	11.19
EABT14989	989.16	9638.82	148	13017.1	1638.14	91.59	246.01
EABT1499	0	0	13	0	0	0	0
EABT14990	17	35	161.88	181.92	50	27.91	50
EABT14991	0	1	4	6	0	0	0
EABT14992	18	15	22	2	3	14	13
EABT14993	2	10	15	16	0	0	1
EABT14994	12	12	20	8	41.99	21	26
EABT14995	1	2	3	2	3	1	0
EABT14996	1	2	12	3	0	0	0
EABT14997	0	1	1	2	0	0	0
EABT14998	3	5	183.96	16	1	4	1
EABT14999	0	1	5	0	1	2	0
EABT15	0	4	16	1	1	1	0

EABT150	0	1	9	2	0	0	0
EABT1500	236	950.07	584	2760.61	271.41	980	1197.42
EABT15000	0	2	2	0	2	0	0
EABT15001	3851.01	6045.9	6506.63	8126.15	3338.99	3994.94	3647.01
EABT15002	0	5	0	3	0	0	0
EABT15003	5	3	1	8	1	0	1
EABT15004	5	9	17	15	2	0	3
EABT15005	21	79	41	512	50	15	23
EABT15006	0	3	38	0	0	0	0
EABT15007	1	5	73	0	0	0	0
EABT15008	1	3	6	0	0	2	0
EABT15009	0	0	1	0	33	0	49
EABT1501	7	10	20	6	0	3	0
EABT15010	0	0	0	0	3	0	0
EABT15011	23	25	85	111	309.1	0	92.05
EABT15012	0	2	8	2	6	2	4
EABT15013	3	3	6	3	0	3	13
EABT15014	1	0	7	0	0	0	0
EABT15015	7	5	4	0	0	0	0
EABT15016	0	1	1	0	1	0	0
EABT15017	0	2	0	1	0	0	0
EABT15018	379	572	1332.84	1070.59	563	372	451
EABT15019	6549.56	7455.2	2830	6459.2	2900.74	5381.06	4612.36
EABT1502	1	4	11	12	2	0	0
EABT15020	0	0	4	1	0	0	0
EABT15021	3	9	9	25	0	1	3
EABT15022	0	1	6	13	0	0	0
EABT15023	1	4	14	3	0	1	1
EABT15024	34	29.46	144.99	65.88	25	0	2
EABT15025	326.02	444	319	301	338.58	257	275
EABT15026	775.64	871.46	809.82	1382.12	681.59	687.94	621.47
EABT15027	5	4	0	0	1	0	0
EABT15028	640.61	813.62	656.49	939.49	449.15	908.77	464.42
EABT15029	0	3	4	0	0	0	0
EABT1503	28	4	3	0	0	23	0
EABT15030	3	5	24	0	0	1	2
EABT15031	0	2	9	5	12542	6	12
EABT15032	0	0	0	0	0	0	0
EABT15033	1	4	10	1	1	1	2
EABT15034	3	0	26	2	2	1	2
EABT15035	1	1	4	1	0	0	0
EABT15036	0	3	1	1	1	0	0
EABT15037	0	0	14	0	0	0	0
EABT15038	3	6	3	9	13	3	17
EABT15039	0	1	3	4	23	0	1
EABT1504	0	0	0	0	2	0	3.01
EABT15040	1	4	2	0	0	0	0
EABT15041	1	1	12	1	0	4	2
EABT15042	0	0	14	0	0	0	0
EABT15043	1	9	27	1	0	2	4
EABT15044	1	2	1	1	0	1	1

EABT15045	0	1	41	0	0	2	1
EABT15046	2840.81	5331.04	8674.56	3305	2364.87	3361.58	2599.12
EABT15047	16	23	5	1	1	3	7
EABT15048	1	2	9	0	1	0	1
EABT15049	5	2	15	1	0	12	2
EABT1505	1	2	2	0	0	7	3
EABT15050	5	21	19	15	3	2	1
EABT15051	0	0	39	1	0	0	0
EABT15052	911	2319.79	3519.59	5363.17	1556.95	221.02	528.94
EABT15053	1	2	0	0	1	3	1
EABT15054	623.14	1028.03	1144.51	1749.52	693	675.32	647
EABT15055	5	4	62	6	0	1	2
EABT15056	20	20	11	1	7	3	19
EABT15057	280	332	294	626	254.72	308	216
EABT15058	578	1944	2335.18	13506.2	807.1	160	642.64
EABT15059	1	0	2	0	1	1	0
EABT1506	2	4	11	6	1	2	1
EABT15060	1	1	12	1	0	0	0
EABT15061	0	0	4	0	0	1	2
EABT15062	3	0	13	0	0	0	1
EABT15063	0	1	7	0	0	0	0
EABT15064	6	0	0	0	0	2	0
EABT15065	5	20	19	66	5	15	11
EABT15066	2	0	6	24	12	0	2
EABT15067	8	21	8	2	0	5	5
EABT15068	0	0	3	3	1	1	0
EABT15069	0	1	11	2	1	2	2
EABT1507	0	0	4	1	2	2	0
EABT15070	1	2	0	16	3	0	0
EABT15071	0	0	0	0	0	0	0
EABT15072	1	0	0	0	2	1	2
EABT15073	0	5	0	0	0	8	0
EABT15074	0	0	2	3	2	0	2
EABT15075	0	2	9	2	1	0	0
EABT15076	3	124	7	4	11	5	5
EABT15077	176.99	284.52	95.51	423.29	79.21	97.92	105.88
EABT15078	0	3	3	2	1	1	4
EABT15079	8	14	13	13	1	7	6
EABT1508	1094	1109	412	1083.88	1178.08	716.01	620
EABT15080	19	1	0	0	1	5	3
EABT15081	0	0	5	0	0	0	0
EABT15082	1	1	5	1	0	0	0
EABT15083	1	0	13	1	0	3	0
EABT15084	1	3	2	4	3	2	3
EABT15085	0	0	15	4	0	0	0
EABT15086	4	8	0	5	3	7	7
EABT15087	0	14	8	0	0	11	0
EABT15088	0	0	14	3	0	1	0
EABT15089	1	0	8	1	0	0	0
EABT1509	119	275	230	294	365	98	74
EABT15090	2	2	6	7	0	1	1

EABT15091	4	0	0	0	0	8	0
EABT15092	6	8	53	3	2	2	5
EABT15093	1	3	12	7	0	1	6
EABT15094	1	4	72	61	1	0	1
EABT15095	3	14	55	10	7	5	0
EABT15096	2	0	1	0	1	6	4
EABT15097	0	3	2	1	1	0	0
EABT15098	7	3	17	1	1	3.99	10
EABT15099	2	7	6	0	2	0	1
EABT151	0	1	7	1	0	0	0
EABT1510	1133	2021.21	1603	1973.54	1369.98	804.97	1124
EABT15100	1	0	11	0	0	1	1
EABT15101	0	0	2	1	0	1	0
EABT15102	3	0	0	0	0	2	1
EABT15103	2	1	3	1	0	0	0
EABT15104	27	24	38	19	8	21	9
EABT15105	0	0	1	0	2	1	1
EABT15106	808.88	1124.92	1194.04	1665.87	663.98	798.1	672.67
EABT15107	11	15	12	20	9	17	28
EABT15108	1	3	19	10	4	1	1
EABT15109	0	0	25	22408.3	10	0	2
EABT1511	1146.84	1787.37	652.98	1874.06	1228.9	716.06	999.95
EABT15110	0	11	2	1	1	2	4
EABT15111	5	13	79	7	1	9	4
EABT15112	0	1	12	1	1	0	1
EABT15113	13	59	497	65	27	15	7
EABT15114	19	68	63	53	49	8	16
EABT15115	0	2	2	4	1	1	2
EABT15116	3	15	116	18	10	1	1
EABT15117	1	1	3	1	0	0	0
EABT15118	3	10	6	51	7	1	13
EABT15119	0	0	6	0	1	0	0
EABT1512	1	1	1	4	2	0	1
EABT15120	443	844.54	3035.18	3297.71	1862.4	41	241
EABT15121	0	0	7	0	2	0	2
EABT15122	0	3	2	1	0	0	0
EABT15123	4301.58	5904.79	4578.34	9206.91	2418.97	3667.07	2618.5
EABT15124	4	4	16	3	3	19	7
EABT15125	0	0	7	1	0	0	0
EABT15126	368.36	844	733.97	12495.2	368	76	516.94
EABT15127	0	1	16	4	1	0	0
EABT15128	2	3	0	0	0	0	0
EABT15129	1	0	8	13	1131.62	1	1
EABT1513	0	0	4	1	0	0	0
EABT15130	4	8	40	7	0	1	0
EABT15131	14	2	0	1	9	10	38
EABT15132	2362.57	3581.2	2877.11	3038.06	2420.01	1914.42	2668
EABT15133	0	2	3	1	0	0	1
EABT15134	2	0	5	1	1	0	0
EABT15135	10	17	22	3	3	10	5
EABT15136	482	713.01	224	846	304	217	460.18

EABT15137	0	2	13	0	0	1	0
EABT15138	332.8	773	384	1633	356.02	274	310
EABT15139	0	3	4	1	3	0	0
EABT1514	621	938.73	1132.22	2302	780	509.18	376
EABT15140	16	28	44	41	9	16	15
EABT15141	0	0	12	0	0	0	0
EABT15142	15	25.62	60	68	12	5	14
EABT15143	0	5	2	4	0	0	1
EABT15144	0	0	0	5.99	0	0	0
EABT15145	10	19	0	0	0	2	6
EABT15146	4	40	36	23	10	12	10
EABT15147	0	0	2	0	1	0	2
EABT15148	1	6	1	0	1	0	2
EABT15149	1	0	10	1	0	0	0
EABT1515	0	3	2	0	0	0	1
EABT15150	0	0	1	0	1	0	1
EABT15151	0	0	2	0	0	0	0
EABT15152	0	5	5	13	0	1	0
EABT15153	151	171	356	1072	109.01	26	48
EABT15154	1945.35	3824.87	4389.34	11791.6	1687.58	1481.29	1169.55
EABT15155	0	0	1	1	0	0	3
EABT15156	48	27	66	24	27	0	7
EABT15157	0	1	26	6	1	0	4
EABT15158	258.15	1257.95	1251.01	1185.68	298	91	176.34
EABT15159	1	2	9	2	2	0	2
EABT1516	0	0	2	1	1	0	0
EABT15160	0	0	0	5	5	0	2
EABT15161	0	1	5.94	3	0	0	0
EABT15162	0	0	4	2	0	0	0
EABT15163	2	5	37	0	0	0	0
EABT15164	0	0	14	1	0	0	0
EABT15165	509	814	5326	1467	1178	820	279
EABT15166	3	9	13	3	1	3	2
EABT15167	0	2	4	9	0	0	0
EABT15168	9060.65	16143.4	24351.4	76731.4	3401.11	11	56
EABT15169	0	0	4	1	0	0	0
EABT1517	685.26	1761.17	724.53	494.34	936.82	45	175
EABT15170	5	9	35	3	0	7	0
EABT15171	0	1	9.04	42	0	1	0
EABT15172	12	66	7	8	15	0	1
EABT15173	0	1	5	0	2	0	0
EABT15174	5	10	4	8	0	8	1
EABT15175	0	0	11	1	0	0	0
EABT15176	6	6	8	4	0	1	2
EABT15177	5	3	1	6	2	3	13
EABT15178	37	15	12	49	14	41	55
EABT15179	0	2	1	3	0	1	0
EABT1518	0	2	3	0	1	0	0
EABT15180	1	1	17	4	7	1	3
EABT15181	0	4	6	0	0	0	0
EABT15182	2598	3712	2431.64	5870.7	1418.83	2697	2151.53

EABT15183	127	276	383	557.99	343	62	31
EABT15184	1969.98	3836.43	2690.83	14695.4	4272.2	799.7	1808.97
EABT15185	1	3	7	9	2	2	1
EABT15186	0	0	20	4	0	1	0
EABT15187	4	74	8	1	6	0	2
EABT15188	2082.06	4052.56	4047.14	6421.6	2414.95	2340.92	2025.46
EABT15189	0	20	2	16	1	0	1
EABT1519	0	3	11	0	1	0	0
EABT15190	0	1	5	0	1	0	0
EABT15191	0	2	16	0	0	0	1
EABT15192	0	1	2	1	0	0	0
EABT15193	59	37	4	0	1	35.98	14
EABT15194	6617.47	7605.86	4888.12	3771.72	4092.38	6271.11	5564.81
EABT15195	41	34	10	16	10	6	26
EABT15196	3	3	0	0	0	0	0
EABT15197	13	45	64.99	64	36	6	4
EABT15198	0	1	4	0	0	0	0
EABT15199	1	2	0	0	0	0	0
EABT152	5	2	100	3	1	12	2
EABT1520	0	0	4	1	1	0	0
EABT15200	0	1	3	8	1	0	0
EABT15201	9	3	1	3	8	21	33
EABT15202	3	3	8	1	1	18	4
EABT15203	0	5	16	8	0	0	1
EABT15204	1006	1582	2166	2560	1522	754.99	634
EABT15205	0	1	9	1	0	1	1
EABT15206	0	0	1	7	1	0	0
EABT15207	185.43	485.01	840.08	659	350.52	291	94
EABT15208	1	2	7	1	0	1	0
EABT15209	0	4	12	3	2	7	0
EABT1521	3	0	4	3	1	1	0
EABT15210	59	56	10	55.47	11	11	9
EABT15211	3244.6	5887.54	4235.32	9059.73	5132.94	1788.08	3444.95
EABT15212	3	1	2	6	0	0	0
EABT15213	8429.4	7973.11	7353.77	3001.35	4555.39	3449.82	4172.01
EABT15214	2136.03	4087.52	917.82	1545.6	937.57	3082.22	2352.72
EABT15215	3643.81	3689.22	1605.84	2763.95	2234.44	3327.39	2653.86
EABT15216	0	3	1	0	0	0	0
EABT15217	0	0	1	4	0	0	0
EABT15218	2	1	30	1	0	1	0
EABT15219	2	1	3	1	0	3	1
EABT1522	1091.01	1430.43	1049.85	1612.78	1033.45	1393.53	1073.72
EABT15220	0	0	5	3	0	1	1
EABT15221	0	0	17	0	0	0	0
EABT15222	23	30	0	0	2	34	30
EABT15223	2	2	9	0	0	0	0
EABT15224	2	2	1	7	3	0	0
EABT15225	1	1	9	6	2	4	2
EABT15226	0	0	19	1	7	1	1
EABT15227	1997.89	2832.21	2151.57	4211.4	2199.62	2240.36	2661.98
EABT15228	0	1	22	3	2	0	0

EABT15229	205.04	225.01	138.77	414	182	124	91
EABT1523	10703.3	18329.2	7774.86	26140.7	9254.84	9456.57	10125.4
EABT15230	6	1	12	1	1	2	3
EABT15231	0	0	3	2	0	0	1
EABT15232	11	55	170	71	97	7	436
EABT15233	1819.72	85	1	0	5	1301.6	10
EABT15234	45	74	13	8	1	39	1
EABT15235	0	0	7	1	0	1	0
EABT15236	1051.93	975	338.99	1850.63	917.87	1254.42	1184.05
EABT15237	0	0	6	0	0	0	0
EABT15238	1	0	0	4	0	0	0
EABT15239	1	3	2	1	0	0	0
EABT1524	0	2	6	0	1	1	0
EABT15240	0	4	0	1	0	0	0
EABT15241	5	4	15	5	0	1	1
EABT15242	2	0	6	0	1	0	0
EABT15243	6	23	4	5	39	6	14
EABT15244	0	0	15	1	0	0	0
EABT15245	0	1	6	0	0	1	0
EABT15246	0	0	2	0	0	0	0
EABT15247	0	2	9	0	3	1	3
EABT15248	2	3	13	1	0	5	0
EABT15249	1	0	3	2	3	1	0
EABT1525	6	34.83	13	32	5	0	1
EABT15250	1	1	0	0	1	8	2
EABT15251	6387.35	1875.24	1	0	0	1697.79	0
EABT15252	1	1	3	5	0	0	0
EABT15253	0	2	4	2	2	0	0
EABT15254	5	7	4	1	0	3	5
EABT15255	398.53	815.89	1660.96	1099.82	1003.3	401.58	484.61
EABT15256	0	2	2	0	2	1	1
EABT15257	1319.93	1991.53	2131.43	2305.11	887.11	934.98	512
EABT15258	647	1138.09	915.03	1310.16	674.93	227	277
EABT15259	0	0	4	1	0	1	0
EABT1526	26	77	81.02	122	28	21	16
EABT15260	6	4	4	13	2	5	2
EABT15261	31	27	18	7	18	0	0
EABT15262	3	14	4	16	2	7	5
EABT15263	0	1	3	6	17	0	0
EABT15264	298	714.04	1135.75	876	616	198	231
EABT15265	14429.6	22789.1	15536	10974.1	11184.9	21481.4	25271.9
EABT15266	23	22.99	13	12	9	24	39
EABT15267	0	2	20	0	1	1	0
EABT15268	1	0	17	3	0	1	1
EABT15269	0	1	1	2	1	0	1
EABT1527	44.99	93	392.01	53.02	53	90	53.8
EABT15270	1646.81	2067.34	1269.25	3286.53	1527.45	1165.45	980.26
EABT15271	0	2	17	0	0	0	1
EABT15272	6	10	24	3	4	0	15
EABT15273	6	7	10	3	1	3	0
EABT15274	0	0	7	0	0	0	0

EABT15275	6	8	22	3	2	23	2
EABT15276	4747.99	5840.95	4537.6	8774.35	4151.11	4697.82	4261.27
EABT15277	0	0	8	0	0	0	0
EABT15278	6	7	41	28	3	2	4
EABT15279	0	0	7	0	1	0	0
EABT1528	0	0	5	2	0	0	0
EABT15280	1	6	0	0	0	0	0
EABT15281	0	1	1	22	2	0	0
EABT15282	1	1	12	10	0	2	1
EABT15283	3	1	3	0	0	0	0
EABT15284	8	4	1	0	1	7	1
EABT15285	1	0	1	0	1	1	2
EABT15286	4	2	24	10	2	4	3
EABT15287	0	0	3	4	0	0	1
EABT15288	2	7	25	7	2	2	0
EABT15289	2	5	16	2	8	0	1
EABT1529	0	10	2	1	1	1	1
EABT15290	0	0	2	6	0	0	0
EABT15291	1	0	2	4	5	0	6
EABT15292	0	1	10	0	2	0	2
EABT15293	0	0	4	0	0	0	1
EABT15294	229	214.04	106	519.4	77	228	67
EABT15295	5	12	21	25	12	2	1
EABT15296	0	1	1	1	0	1	1
EABT15297	0	1	2	2	1	0	0
EABT15298	1	3	1	1	0	1	1
EABT15299	0	0	2	8	1	0	1
EABT153	0	0	25	0	0	0	0
EABT1530	8	24	54	40	5	8	10
EABT15300	24	47	5	1	5	27	51
EABT15301	6	7	0	0	0	1	2
EABT15302	0	1	0	3	0	0	0
EABT15303	0	1	1	0	0	0	2
EABT15304	23	64	457	736	438.58	12	86
EABT15305	0	0	5	0	0	0	0
EABT15306	0	0	13.42	0	0	0	0
EABT15307	9	9	31	15	1	2	0
EABT15308	24	48	54	12	25	163.02	16
EABT15309	0	1	1	6	2	0	0
EABT1531	0	0	0	8	0	0	0
EABT15310	0	3	27	2	0	0	0
EABT15311	10860.7	16094.5	1188.44	1894.97	2839.39	10146.6	13767.1
EABT15312	1	6	34	9	0	1	3
EABT15313	447161	86914.1	74	81	60474.3	609289	416487
EABT15314	623.59	638.27	225.47	223.17	395.48	416.95	471.58
EABT15315	7	11	0	0	7	51	16
EABT15316	0	4	16	0	1	0	2
EABT15317	523	682.62	602.34	987.94	539.01	585	277.98
EABT15318	9	9	0	0	3	27.03	15
EABT15319	28371	29584.3	21109.1	23926	24123.3	33403.4	28412.8
EABT1532	27	20	131	63	12	14	13

EABT15320	0	8	0	9	0	0	0
EABT15321	3	3	5	5	3	1	4
EABT15322	1	0	0	1	1	0	0
EABT15323	2	2	3	1	0	0	0
EABT15324	1	0	4	24	84	0	2
EABT15325	2	3	2	1	0	2	0
EABT15326	0	0	20	0	0	0	0
EABT15327	1492.49	4584.09	6928.2	4297.51	1573.44	776	967
EABT15328	2	5	21	3	6	5	6
EABT15329	0	3	0	0	0	0	1
EABT1533	1865	3302.37	8494.32	10601.3	2613.21	1486.51	1517.61
EABT15330	0	2	2	11	0	0	0
EABT15331	0	3	3	2	1	2	1
EABT15332	2	3	35	4	0	3	2
EABT15333	1	0	6	0	0	0	0
EABT15334	0	1	9	0	0	0	0
EABT15335	0	2	18	6	1	1	0
EABT15336	0	0	6	0	5	1	0
EABT15337	149.62	153.41	240.02	515.74	238.22	112.07	236.42
EABT15338	1	3	1	6	1	0	0
EABT15339	5	7	23	0	1	6	1
EABT1534	2	3	8	2	0	2	1
EABT15340	1	4	0	0	0	0	0
EABT15341	0	7	0	0	0	4	2
EABT15342	2	5	14	5	2	0	0
EABT15343	35	21	3	130	3	2	0
EABT15344	0	2	8	0	0	0	0
EABT15345	589.98	811.57	1118	1129	451.93	405	520
EABT15346	0	0	14	0	1	0	0
EABT15347	1586.3	4187.36	5300.97	6617.93	2622.86	3919.34	3032.11
EABT15348	14	10	0	0	0	0	1
EABT15349	2275.27	2952.93	3414.89	5160.68	2135.32	2021.16	1899.28
EABT1535	2	0	2	3	0	0	0
EABT15350	2	3	159	1	1	7	1
EABT15351	2	0	5	7	1	0	0
EABT15352	2280.39	3232.56	31559.9	5336.97	5195.87	2231.1	1732.99
EABT15353	0	2	4	0	0	0	1
EABT15354	0	0	10	1	0	0	0
EABT15355	20	18	1	0	2	0	0
EABT15356	648.99	1773.99	507.03	537	1075.78	226	427
EABT15357	110	394.01	747	8394.86	455.84	5	180
EABT15358	450	495	147	17	1003	3	15
EABT15359	0	9	1	1	0	0	0
EABT1536	2924.73	3738.15	4380.66	4874.66	2695	2403.99	1604
EABT15360	1248.6	1838.95	911.23	1373.9	1304.75	1050.29	1290.23
EABT15361	1	3	0	0	0	0	0
EABT15362	113	393.22	249.33	190.44	333.03	142.98	210.7
EABT15363	7	6	8	18	8	2	3
EABT15364	0	2	0	4	0	0	0
EABT15365	0	2	41	4	2	1	2
EABT15366	0	2	13	1	1	0	1

EABT15367	1	3	0	0	0	0	0
EABT15368	2701	1952.93	279	640	2615.55	1	141
EABT15369	4	2	1	1	0	6	2.97
EABT1537	3	0	7	1	0	3	1
EABT15370	0	0	2	1	0	0	0
EABT15371	17	137	11	3	83	29	271
EABT15372	1100.85	1333.49	2165.51	737.11	807.81	979.27	1189.4
EABT15373	19	16	28	5	1	56	10
EABT15374	1	1	19	1	0	0	1
EABT15375	0	2	3	0	0	0	0
EABT15376	333.81	458.23	280.68	649.93	320.98	130	319
EABT15377	7	17	30	15	0	3	2
EABT15378	0	0	8	1	0	0	0
EABT15379	0	0	10	5	0	0	0
EABT1538	0	0	8	0	0	0	1
EABT15380	18	25	36	72	9	10	9
EABT15381	0	1	0	1	0	1	0
EABT15382	1	1	9	1	10	2	7
EABT15383	0	0	4	1	0	4	0
EABT15384	0	1	8	2	0	0	0
EABT15385	4	6	4	8	2	0	0
EABT15386	0	5	2	0	1	0	0
EABT15387	7	4	10	3	4	1	2
EABT15388	1	6	1	0	0	0	0
EABT15389	4477.34	8507.21	5210.6	4895.24	2379.09	2877.29	3084.94
EABT1539	548.82	796.53	593.94	1864.3	765	428.03	478.27
EABT15390	804.16	901.73	30	0	716.13	97	920.18
EABT15391	11799.3	15686.4	17438.1	17371	7187.51	10758.5	8660.08
EABT15392	0	2	2	8	2	1	0
EABT15393	0	0	6	1	0	0	0
EABT15394	0	8	2	1	1	1	0
EABT15395	0	2	13	12	1	1	3
EABT15396	0	1	3	2	0	0	1
EABT15397	3	4	21	7	2	6	1
EABT15398	0	1	20	1	0	0	0
EABT15399	0	0	0	8	1	0	0
EABT154	0	0	9	2	0	0	0
EABT1540	7	5	14	1	1	1	0
EABT15400	1	1	34	0	0	0	0
EABT15401	141	240.47	260	571.15	69.46	120.02	103
EABT15402	2	3	7	1	2	0	0
EABT15403	32	45	0	0	3	100	59
EABT15404	5	8	21.99	3	0	4	3
EABT15405	3	2	4	0	1	3	1
EABT15406	2	4	0	0	2	0	0
EABT15407	23	26	0	0	0	13	15
EABT15408	0	1	32	1	0	0	0
EABT15409	3	2	7	2	6	3	2
EABT1541	1	4	6	5	108	1	9
EABT15410	39	69	28	252	43	63	314
EABT15411	0	0	1	3	0	2	1

EABT15412	0	3	11	1	1	3	11
EABT15413	0	1	2	7	0	0	0
EABT15414	0	0	0	0	0	1	3
EABT15415	1	3	20	5	3	1	4
EABT15416	1	4	6	2	1	0	0
EABT15417	2	5	11	3	0	2	1
EABT15418	0	1	2	14	1	0	0
EABT15419	0	4	40	1	0	3	2
EABT1542	0	0	17.02	1	0	0	0
EABT15420	17	65.95	59	238.34	48	4	19
EABT15421	1	2	3	8	0	0	0
EABT15422	0	0	3	1	1	0	1
EABT15423	0	1	1	3	2	0	0
EABT15424	2	8	1	0	0	7	4
EABT15425	10528.5	18740.5	29106.2	3182.8	8568	1736.62	3012
EABT15426	0	2	1	4	1	0	0
EABT15427	1697.94	2469.79	3422.6	1873.03	1600.97	1393.3	1319.67
EABT15428	0	0	7	1	0	0	0
EABT15429	3	3	6	6	6	0	18
EABT1543	541.56	895.14	464.01	858.05	595	500.89	385.72
EABT15430	0	0	0	0	0	2	0
EABT15431	1063.48	1535.07	1269.04	2921.21	1043.83	1017.48	948.98
EABT15432	396	1004.24	2321.65	2451.43	654.57	256	896.07
EABT15433	14	8	2	15	5	0	2
EABT15434	1	4	9	0	0	1	2
EABT15435	0	0	8	1	0	1	0
EABT15436	0	0	3	1	0	0	2
EABT15437	560.99	507.79	65	381.07	183	573.05	32
EABT15438	1	0	8	3	0	0	0
EABT15439	1	3	0	0	3	6	4
EABT1544	2	0	3	2	0	4	0
EABT15440	1	1	7	2	0	1	1
EABT15441	2487.03	4395.33	6433.99	6539.79	4835.31	2905.05	5459.51
EABT15442	1098.28	1063.25	94	68	863.1	1738.01	260
EABT15443	3637.45	6925.05	4991.94	7142.84	2277.85	4887.35	3719.99
EABT15444	2662.45	3331.24	1628.79	3594.46	1495.23	4043.21	2878.22
EABT15445	2	6	12	13	14	0	4
EABT15446	0	0	9	0	0	0	0
EABT15447	0	2	0	2	2	1	1
EABT15448	7	8	41	2	1	2	3
EABT15449	4	5	9	18	1	4	0
EABT1545	5	13	8	5	6	0	32
EABT15450	0	2	9	4	1	0	1
EABT15451	6	8	0	0	2	0	4
EABT15452	0	1	0	0	0	0	0
EABT15453	0	0	9	4	2	0	0
EABT15454	0	2	10	1	0	0	0
EABT15455	1	1	10	1	0	1	1
EABT15456	517.26	743.95	711.05	1276.77	527.11	336	539.43
EABT15457	0	2	16	1	1	0	1
EABT15458	1733.96	1698.01	1098.13	1372.89	2785.13	1443.96	1377.89

EABT15459	1	10	12	7	13	8	3
EABT1546	11	7	1	1	1	9	0
EABT15460	1	2	1	3	2	0	1
EABT15461	0	0	7	4	1	1	0
EABT15462	0	1	3	0	4	0	0
EABT15463	0	0	6	3	0	1	0
EABT15464	2603.83	3548.35	12090	3355.24	1681.39	685.21	1173.83
EABT15465	2	0	3	1	0	0	0
EABT15466	3	12	2	0	0	2	0
EABT15467	0	1	1	1	0	0	0
EABT15468	20	54	73.9	4	1	4	0
EABT15469	556.27	772.08	289	458	335	313.58	443
EABT1547	2	15	26	47	22.49	13	19
EABT15470	379.17	937.9	303	239.53	226.1	35.99	136
EABT15471	1	5	9	1	0	2	0
EABT15472	0	1	10	3	1	0	1
EABT15473	0	0	11	0	0	0	0
EABT15474	0	0	4	10	0	0	0
EABT15475	0	0	3	1	0	0	0
EABT15476	5	25	6	7	7	0	21
EABT15477	1	6	32	6	3	1	2
EABT15478	4	0	7	1	2	6	3
EABT15479	5	21	19	47	11	15	4
EABT1548	170.3	203.57	180.91	217.38	195.54	174	158
EABT15480	3	41	25	0	6	3	0
EABT15481	1	2	0	0	0	9	8
EABT15482	1937.21	3026.45	1995.4	4247.92	2019.14	1504.09	1642.36
EABT15483	0	4	2	1	0	0	1
EABT15484	2	5	18	4	0	0	1
EABT15485	71.61	100.47	206.95	352.85	139.05	46	38.02
EABT15486	2	0	2	0	0	5	0
EABT15487	2	3	11	2	1	5	0
EABT15488	0	3	2	2	0	0	0
EABT15489	711.29	1356.2	1474.38	1834.96	1067.47	518.99	684.03
EABT1549	1	0	3	0	0	0	0
EABT15490	0	1	10	0	7	0	0
EABT15491	3	1	0	0	0	0	0
EABT15492	4	4	13	1	2	8	3
EABT15493	0	2	3	2	2	0	0
EABT15494	2	2	9	1	0	0	0
EABT15495	2	4	29	1	1	8	0
EABT15496	10	10	0	0	0	19	17
EABT15497	280.89	388.5	450.32	710.67	480.03	100.09	167
EABT15498	0	0	0	0	0	0	0
EABT15499	0	2	3	0	1	0	0
EABT155	62.78	78	332.01	70	22	17	18
EABT1550	1	4	8	13	4	0	0
EABT15500	0	2	24	2	0	1	0
EABT15501	0	4	10	1	1	0	0
EABT15502	0	0	5	3	0	0	0
EABT15503	13	35	97	1355.16	63.99	28	74.38

EABT15504	5	27	54	2	1	8	9
EABT15505	7	39	89	20.26	2	5	4
EABT15506	1	0	6	1	0	0	1
EABT15507	337	514.01	416.55	576.99	812.06	463.99	353
EABT15508	0	4	29	1	0	1	1
EABT15509	0	2	1	4	0	1	1
EABT1551	6	11	6	0	0	49	0
EABT15510	0	1	47	0	0	2	0
EABT15511	262.01	306.65	486.72	563.31	356	186.94	261.91
EABT15512	32	18	4	1	4	5	37
EABT15513	5	6	17	5	0	1	6
EABT15514	832.01	984.93	398	1609.59	1089.26	1284.24	706.65
EABT15515	0	1	10	3	1	0	0
EABT15516	0	3	5	3	20	1	13
EABT15517	4	7	20.33	12	13	10	8
EABT15518	3	10	88.89	4	2	3	5
EABT15519	1375.14	1194.54	711.14	768	780.34	1146.6	859.64
EABT1552	0	8	2	12	0	2	2
EABT15520	0	3	7	3	0	0	0
EABT15521	0	1	0	0	11.21	0	2
EABT15522	404	671.23	600.52	1238.93	693	168	440
EABT15523	2	0	6	0	0	0	0
EABT15524	0	0	0	2	2	0	4
EABT15525	18	25	23	29	13	3	6
EABT15526	1518.1	2199	2143.91	2602	1852.9	1741	1577.82
EABT15527	1078.98	1673.39	1079.15	4610.37	2659.27	770	628.19
EABT15528	0	0	9	8	1	0	0
EABT15529	326	454	407	928.99	463.66	329.01	327.52
EABT1553	46	79.97	57	12	5	9	4.01
EABT15530	2	3	18	1	0	1	0
EABT15531	1	2	28	14	2	2	3
EABT15532	1	0	0	0	0	4	0
EABT15533	2	4	10	1	0	0	0
EABT15534	0	0	0	3	4	0	2
EABT15535	3941.62	5068.07	2774.4	2809.28	2940.02	2643.49	5738.73
EABT15536	2	3	22	15	0	1	2
EABT15537	3658.1	3218	62	315	1604.02	2447.9	2932.97
EABT15538	0	1	1	1	0	0	0
EABT15539	0	2	13	0	0	1	0
EABT1554	1	2	0	0	0	0	9
EABT15540	0	6	6	0	0	0	0
EABT15541	25	56	627.62	839.09	1357.56	3	273.37
EABT15542	1350	1625.91	2063.28	3459.93	775.18	888.16	806
EABT15543	210	289	256.06	394.72	79	169.98	145.26
EABT15544	2467.11	2657.03	433.01	2574.03	1347.07	1238	2398.22
EABT15545	0	2	3	4	1	0	1
EABT15546	6	0	0	0	0	3	0
EABT15547	0	0	5	0	1	0	0
EABT15548	2	0	3	2	0	0	0
EABT15549	11	8	15	12	5	0	0
EABT1555	0	18	13	63	2	1	5

EABT15550	9	6	6	6	6	0	2
EABT15551	2	0	5	0	0	0	0
EABT15552	2	15	11	90	22	4	1
EABT15553	3	3	1	2	2	0	0
EABT15554	0	1	7	0	0	0	0
EABT15555	0	1	13	0	1	0	1
EABT15556	2	2	0	2	0	0	0
EABT15557	1	5	4	3	0	1	0
EABT15558	3	1	4	3	1	0	0
EABT15559	1	1	25	0	0	0	0
EABT1556	4	7	0	0	0	6	4
EABT15560	1019	3020.99	3876.83	9444.21	465.98	931.5	626
EABT15561	0	1	4	0	0	0	0
EABT15562	3	6	6	12	0	0	2
EABT15563	59	224	151	70	68.77	275	1935
EABT15564	0	1	5	0	0	0	1
EABT15565	0	6	2	2	0	0	0
EABT15566	4	4	11	1	0	11	5
EABT15567	2	7	38	3	0	0	1
EABT15568	453.58	576.25	415.97	1087.53	480.53	334.97	476.29
EABT15569	0	4	0	2	0	1	0
EABT1557	1	5	3	1	1	2	2
EABT15570	2	2	0	11	0	4	2
EABT15571	0	0	9	0	0	0	0
EABT15572	1553.82	2492.1	1977.45	2365.74	1263.65	1362.26	1150.93
EABT15573	0	4	6	6	3	9	12
EABT15574	235.72	388.1	331.14	574	357.69	128	209.03
EABT15575	3	1	9	2	0	0	0
EABT15576	0	6	14	16	3	0	1
EABT15577	1	1	28	0	0	0	0
EABT15578	2339.98	6995.23	267	16	1147.91	2710.03	3053.75
EABT15579	374	681	1054.99	708	1012	102.01	109
EABT1558	0	0	0	0	1	3	1
EABT15580	0	0	42	0	0	1	0
EABT15581	0	0	2	0	0	0	0
EABT15582	0	10	608	2	0	0	0
EABT15583	1	0	2	0	1	0	4
EABT15584	0	0	26	1	0	0	0
EABT15585	0	3	1	0	7	0	6
EABT15586	4443.32	4176.68	3267.23	5324.95	2089.95	2278.57	2378.05
EABT15587	21782.9	13325.8	3277.8	5797.76	7900.47	2890.64	2912.34
EABT15588	1	13	75	9	4	0	0
EABT15589	0	1	1	3	0	0	0
EABT1559	23	13	336	2433	131	0	1
EABT15590	370	439.04	651.78	957	550.1	355.06	378.01
EABT15591	9	7	2	3	1	0	1
EABT15592	1	2	12	2	0	0	1
EABT15593	0	1	1	1	1	0	0
EABT15594	1438.38	2268.73	1607.62	6836.48	2552.93	1719.05	1812.11
EABT15595	5911.89	3288.61	149	2	4706.58	8903.75	29031.2
EABT15596	0	0	16	0	54.1	1	0

EABT15597	20	32	291.92	70	48	8	56
EABT15598	0	0	1	1	0	0	0
EABT15599	206	270.08	246	850	294.99	72	91
EABT156	0	2	11	0	4	1	4
EABT1560	727.98	1039.73	1046.26	1460.22	968.39	639.08	644.33
EABT15600	44	23	27	40	19	1	1
EABT15601	2494.51	2562.35	2226.83	1464	1824.04	4155.27	3971.57
EABT15602	14	38	19	0	1	9	1
EABT15603	1	2	10	1	0	0	1
EABT15604	0	0	13	2	3	0	1
EABT15605	0	0	5	0	0	0	0
EABT15606	1	5	12	7	3	2	1
EABT15607	11	49	253	89.02	337	4	20
EABT15608	8	29	57	55.97	338	7	10
EABT15609	767.06	921.99	507.43	1306.37	917.88	606.47	597.99
EABT1561	0	0	38	4	0	0	0
EABT15610	1	1	30	1	1	0	1
EABT15611	0	1	6	1	0	0	0
EABT15612	0	1	4	0	0	0	2
EABT15613	3	2	2	2	1	1	1
EABT15614	0	1	10	1	0	0	1
EABT15615	6	12	2	18	7	7	7
EABT15616	1	0	11	0	2	0	1
EABT15617	1281.72	1130.42	899.14	931.49	509.45	946.75	832.82
EABT15618	1	1	26	10	1	1	0
EABT15619	46	76	177.09	566.12	136.79	65	73
EABT1562	0	10	1	7	0	1	2
EABT15620	473	824	1327.91	2436	540	333	363
EABT15621	1	2	2	2	3	0	0
EABT15622	1	2	10	1	3	0	0
EABT15623	13	25.67	7	17	13	0	2
EABT15624	33	46.35	0	0	5	12	16
EABT15625	0	2	12	3	3	1	0
EABT15626	0	1	3	2	1	3	0
EABT15627	12	22	25	165.95	23	12	6
EABT15628	0	3	5	11	3	2	0
EABT15629	15	62	96	68	18	50	18
EABT1563	0	1	1	1	6	0	1
EABT15630	3	4	35	14	0	9	3
EABT15631	2	1	10	0	2	1	0
EABT15632	0	0	2	4	0	0	0
EABT15633	5776.44	27155.6	83616.4	907	3635.79	1616.28	10051.5
EABT15634	0	1	48	1	0	1	1
EABT15635	0	1	3	3	3	0	1
EABT15636	0	2	6	0	1	0	0
EABT15637	0	0	0	0	0	0	7
EABT15638	1	0	10	2	1	0	1
EABT15639	40	205	430.21	1736.13	37	12	9
EABT1564	2	1	5	5	1	1	0
EABT15640	1	1	9	1	1	0	1
EABT15641	4	1	3	6	1	2	0

EABT15642	47	185	103	97	780	2	45
EABT15643	1	3	9	3	2	0	4
EABT15644	2057	3390.14	2383.39	3912.21	1857	2308.99	1650
EABT15645	0	0	0	3	2	0	0
EABT15646	5	0	0	0	0	0	1
EABT15647	1	1	15.99	2	1	1	0
EABT15648	11	1	0	0	0	11	0
EABT15649	4	5	6	9	1	4	6
EABT1565	0	0	11	0	0	0	0
EABT15650	0	0	0	0	0	0	0
EABT15651	2	4	0	0	0	1	0
EABT15652	1695.8	3263.58	3995.82	6611.13	1894.35	2389.15	1762.4
EABT15653	0	1	3	1	0	1	0
EABT15654	0	0	3	6	0	0	0
EABT15655	5	9	0	0	0	7	0
EABT15656	9	14	20	41.41	45.01	1	9
EABT15657	0	0	2	0	1	0	2
EABT15658	3	2	13	1	1	0	0
EABT15659	416	652	491	965.01	259	247	247
EABT1566	1	1	7	3	0	0	1
EABT15660	1	0	9	5	1	1	1
EABT15661	0	0	3	0	0	1	2
EABT15662	0	15	0	1	5	0	1
EABT15663	3	5	7	16	1	2	0
EABT15664	0	2	0	3	0	1	0
EABT15665	5830.36	10814.6	11412.5	13092.3	4255.09	5276.03	4947.08
EABT15666	39	118	67.02	22	170	20	13
EABT15667	2899.58	3112.33	939	595	1449.81	3879.28	2897.9
EABT15668	0	4	2	6	0	0	1
EABT15669	1801.32	2386.46	1747.92	2662.25	1508.21	1461.68	1627.24
EABT1567	2	2	2	2	2	0	1
EABT15670	0	3	0	2	0	0	1
EABT15671	0	2	8	0	0	0	0
EABT15672	1	1	8	3	0	1	0
EABT15673	0	1	8	0	0	0	1
EABT15674	1	0	3	4	3	0	1
EABT15675	199.32	284.82	370.34	861.26	301.11	116	114
EABT15676	84013.4	54000.2	81902.6	80097.4	58045.9	13365.1	9689.78
EABT15677	0	1	6	0	0	0	0
EABT15678	1	19	64	11	4	3	1
EABT15679	892.84	2199.41	1809.43	1381.51	469	90.12	176
EABT1568	31	65	121	56	314	32	46
EABT15680	9	6	0	0	0	0	0
EABT15681	1581.86	2270.08	3729.34	4977.8	1454.13	1656.02	1156.09
EABT15682	0	0	1	0	0	0	1
EABT15683	0	3	1	0	0	0	3
EABT15684	0	0	3	0	0	0	1
EABT15685	2	4	6	6	1	1	3
EABT15686	10	33	34	16	7	5	5
EABT15687	4	1	7	0	0	1	0
EABT15688	0	22	73	10	10	2	1

EABT15689	2	3	28	3	1	3	3
EABT1569	3398.28	5768.75	5342.24	8883.93	2568.78	1930.98	1701.66
EABT15690	3109.35	4214.64	2475.55	4381.02	1970.72	3383.41	3415.75
EABT15691	100	150.04	55	20	35	18	36
EABT15692	2	1	7	1	0	0	1
EABT15693	16	3	2	10	4	3	2
EABT15694	910	1058.36	822.12	1426	1051	701.71	550.01
EABT15695	3	21	5	0	40	1	1
EABT15696	3	11.2	42.3	10	7	1	7
EABT15697	3	14	0	0	8	0	2
EABT15698	42	12	0	0	0	0	5
EABT15699	0	0	7	0	0	0	0
EABT157	0	1	0	2	3	1	1
EABT1570	28	29	93	48	61	7	37
EABT15700	2	4	7	0	0	0	0
EABT15701	2	5	19	4	2	1	4
EABT15702	2	0	3	2	1	6	4
EABT15703	0	0	1	2	4	2	2
EABT15704	4	1	0	0	0	1	0
EABT15705	0	5	23	4	0	0	0
EABT15706	10	1	2	0	0	0	0
EABT15707	22	33	103	28	67	37	51.01
EABT15708	1774.6	2963.58	3157.75	6537.33	3363.97	1779.68	1886.08
EABT15709	2	0	27.98	1	0	0	1
EABT1571	6	3	6	15	19	1	7
EABT15710	0	1	37	1	0	0	0
EABT15711	348.85	1341.89	399.38	306.06	674.02	299.99	674.32
EABT15712	0	3	3	1	0	0	0
EABT15713	11	19	16	10	3	1	3
EABT15714	0	4	27	2	3	2	1
EABT15715	492	622	759	1605.06	589	1	14
EABT15716	1	5	89	12	1	9	2
EABT15717	0	2	2	1	2	2	2
EABT15718	0	1	6	4	0	5	3
EABT15719	846	1057.64	1042.75	2038	892	688	639
EABT1572	8	7	0	0	0	2	1
EABT15720	1432.84	2099.58	913.53	1341.47	854.35	943.62	1625.44
EABT15721	1595.03	2188.1	2128.04	1802.12	949.01	1443.15	1043.99
EABT15722	4	14	2	2	1	3	1
EABT15723	0	1	4	0	0	0	1
EABT15724	0	1	2	2	0	1	1
EABT15725	0	0	13	0	1	0	1
EABT15726	6	3	0	4	0	0	0
EABT15727	12	5	3	1	8	0	0
EABT15728	7773.08	9743.79	2007.5	455	5050.7	6798.26	13120.7
EABT15729	18	8	10	58	4	1	1
EABT1573	0	22	0	0	0	0	5
EABT15730	6	6	11	8	0	4	3
EABT15731	0	2	1	0	0	1	0
EABT15732	5	8	2	1	2	1	17
EABT15733	24	30	0	0	2	31	17

EABT15734	7	11	2	10	17	0	0
EABT15735	0	0	8	0	0	0	0
EABT15736	0	0	9	5	0	2	1
EABT15737	0	0	3	5	0	0	0
EABT15738	1322.01	2528.7	2208.25	1671.51	969.92	740.18	707.28
EABT15739	3	4	21	12	2	0	5
EABT1574	0	3	3	1	0	0	0
EABT15740	1	1	4	1	0	0	0
EABT15741	0	0	6	0	0	0	0
EABT15742	0	0	6	3	0	0	0
EABT15743	1543.01	1295.95	499.01	867	1078.17	1732	833.05
EABT15744	56	89	157	186.08	112	46	119
EABT15745	0	1	4	0	1	0	0
EABT15746	35	35	3	4	22	6	37
EABT15747	3	13	22	1	3	10	2
EABT15748	0	0	20	0	0	0	0
EABT15749	0	1	5	9	1	2	0
EABT1575	6	57.37	256.07	594	4	0	0
EABT15750	1	5	1	4	0	0	0
EABT15751	0	0	10	0	0	0	0
EABT15752	530	745	575	1132.69	563	450	398
EABT15753	54.96	86	97	201.01	83	46	61
EABT15754	50.96	127	98	65	271.01	55.94	96
EABT15755	2	3	10	8	0	0	1
EABT15756	741.01	838.01	425	513.92	592.99	397.16	349
EABT15757	5	11	0	0	4	0	1
EABT15758	1	6	8	53	3	0	2
EABT15759	1	0	8	1	1	0	0
EABT1576	24	18	34	1	5	12	44
EABT15760	0	5	14	0	0	2	0
EABT15761	7	10	42	39	3	6	2
EABT15762	1	3	50	0	1	3	0
EABT15763	8	26	14	105	20	0	1
EABT15764	12411.3	16300.3	11294.7	11434.6	4676.66	12395.4	11195.7
EABT15765	4663	4823	297	1347	1046.36	6217.93	1689.99
EABT15766	9	11	59	26	4	15	7
EABT15767	0	3	4	0	2	0	2
EABT15768	3	0	67	0	1	1	0
EABT15769	0	0	0	0	0	0	0
EABT1577	3	4	4	4	1	2	2
EABT15770	19	13	154.7	12.43	21.84	21	11
EABT15771	0	0	0	2	1	0	1
EABT15772	27538	23677	21002.2	11668.8	34353.7	24286.3	16593.9
EABT15773	1978.27	3083.47	3348.16	5933.55	1966.22	1515.49	1371.09
EABT15774	0	0	10	0	0	0	0
EABT15775	1	3	1	8	7	5	1
EABT15776	0	0	4	1	0	0	0
EABT15777	0	1	9	0	0	0	0
EABT15778	187.81	115	121	184.04	110.98	70.67	30
EABT15779	2	1	7	0	0	0	1
EABT1578	0	0	7	1	1	0	1

EABT15780	0	1	0	3	1	0	0
EABT15781	0	0	21	0	0	0	0
EABT15782	12	19.25	87	647.01	10	9	11
EABT15783	486.96	1154.2	1348.09	4592.92	575	512.99	311.92
EABT15784	0	0	1	1	0	0	0
EABT15785	2	2	22	2	0	1	0
EABT15786	33.99	159	75	150	149.99	7	16.67
EABT15787	10241	5549.97	2037.2	9825.4	565	461	276
EABT15788	0	0	12	0	0	0	0
EABT15789	0	0	8	0	0	0	0
EABT1579	0	0	3	0	0	1	0
EABT15790	8	23	15	21	16	0	1
EABT15791	0	0	7	0	0	0	2
EABT15792	6	13	14	14	15	2	2
EABT15793	48	147	57	262	28	115	197
EABT15794	8	7	11	0	0	13	11
EABT15795	0	4	14	8	2	0	0
EABT15796	4608.75	5624.6	4016.1	3494.28	3521.7	7978.1	4509.37
EABT15797	1	5	1	13	1	1	8
EABT15798	0	1	4	1	0	0	0
EABT15799	10069.6	91432.1	150870	66293.9	2074.19	141	130
EABT158	3	13	3	0	0	3	4
EABT1580	1	1	0	0	0	4	0
EABT15800	9	15	99.95	8	37	9	12
EABT15801	0	5	10	1	1	1	3
EABT15802	3188.04	3986.49	2157.88	5205.07	1946.27	2634.11	2046.84
EABT15803	0	7	30	1	0	1	0
EABT15804	2	6	13	10	6	0	0
EABT15805	1	2	4	9	0	5	2
EABT15806	0	0	1	0	3	0	0
EABT15807	27	39	33	151	13	9	36
EABT15808	1	0	6	2	0	0	0
EABT15809	1	2	1	3	0	1	0
EABT1581	19	0	2	8	4	36	6
EABT15810	0	0	10	1	0	0	0
EABT15811	160	226.93	196	382.05	168	99	113
EABT15812	4	15	53.68	587.89	93	0	5
EABT15813	1	0	5	0	1	0	0
EABT15814	0	0	5	1	0	1	0
EABT15815	62.87	82	94	120	76	25	42
EABT15816	8	10	7	11	0	0	3
EABT15817	12387.8	7876.99	15057.2	5830.37	3968.4	4180.56	2539.09
EABT15818	99	37	90.27	54	12	145	18
EABT15819	2127.96	779	39	33	604	1610	1125.98
EABT1582	0	0	13	0	2	0	0
EABT15820	0	2	18	0	1	0	0
EABT15821	4	13	21	26	9	3	8
EABT15822	4	4	0	0	0	1	0
EABT15823	1	4	6	20	22	0	0
EABT15824	0	1	25	0	1	0	0
EABT15825	3	14	12	16	0	3	1

EABT15826	0	2	28	10	0	0	1
EABT15827	0	0	18	5	2	0	0
EABT15828	2	3	57	9	13	74	14
EABT15829	3981.14	3208.23	4400.03	3547.64	2794.88	2439.31	2206.98
EABT1583	11	44.22	7	2.02	0	54	28.07
EABT15830	0	0	4	0	1	0	0
EABT15831	1	2	7	21	2	0	1
EABT15832	0	3	16	2	2	1	0
EABT15833	2	0	0	0	1	3	1
EABT15834	2	0	4	2	0	0	0
EABT15835	1391.05	1939.01	996.96	391.56	674	1055.37	1525.27
EABT15836	1	10	14	13	9	0	4
EABT15837	3	3	6	4	1	17	2
EABT15838	1	3	4	0	1	0	0
EABT15839	1	0	3	0	1	0	0
EABT1584	3	20	57	70	69	0	1
EABT15840	0	5	4	2	0	2	1
EABT15841	0	2	12	1	0	1	0
EABT15842	2	0	11	7	1	1	1
EABT15843	23	11	1	3	0	12	2
EABT15844	1	5	39	4	1	3	1
EABT15845	2	4	7	10	4	3	0
EABT15846	0	2	11	1	0	0	0
EABT15847	5	6.98	6	10	5	2	1
EABT15848	1	0	24	3	0	0	0
EABT15849	0	0	4	0	2	0	0
EABT1585	5	14	26	41	1	2	3
EABT15850	0	1	6	1	0	2	0
EABT15851	1	2	4	1	0	1	1
EABT15852	289.17	372.07	123	27	122	208.34	91
EABT15853	2	3	6	0	1	0	0
EABT15854	25	4	0	0	0	2	2
EABT15855	0	1	4	1	0	2	0
EABT15856	13	2	6	1	0	2	0
EABT15857	201	396.63	1247.54	647.79	977.98	290.57	592.2
EABT15858	6	3	0	0	0	0	0
EABT15859	0	0	0	0	15	0	0
EABT1586	941	1116	664.84	1130.15	889.66	798.62	500.78
EABT15860	4	4	3	17	4	0	8
EABT15861	2	4	4	6	5	0	1
EABT15862	2	8	11	0	0	1	0
EABT15863	1	0	13	0	0	0	0
EABT15864	1	0	3	0	0	0	1
EABT15865	2	6	2	35	4	1	1
EABT15866	5	5	4	8	0	1	0
EABT15867	0	1	4	1	1	1	0
EABT15868	16	6	8	0	0	6	1
EABT15869	531.78	932.57	1275.6	1403.95	508.54	322.6	491.82
EABT1587	0	0	4	2	0	1	0
EABT15870	2	3	6	0	0	0	1
EABT15871	8	3	16	5	14	2	2

EABT15872	18845.1	401.94	6601.57	37010.5	7843.99	49	56
EABT15873	36	55.01	31	30	22	14	10
EABT15874	6	14	259	8	2	21	2
EABT15875	6	28	19	21	15	1	12
EABT15876	5577.15	7915.93	9075.71	18545.5	5659.87	3496.75	5504.95
EABT15877	76	176	208	1453.92	312.47	29	69
EABT15878	5208.05	10717	12855.7	24017.1	8597.88	3471.05	4946.77
EABT15879	5	6	15	4	1	0	3
EABT1588	0	2	6	0	0	0	0
EABT15880	2594.93	3897.78	7089.69	3950.56	1986	1632.85	1703.42
EABT15881	1700	1880.61	468.02	337.01	948	2014.59	2045
EABT15882	0	0	6	0	0	1	0
EABT15883	0	3	4	1	1	0	1
EABT15884	12353.3	10200.4	3174.19	3630.31	2264.29	1275.57	1497.43
EABT15885	0	2	9	0	0	1	0
EABT15886	0	2	8	0	1	0	1
EABT15887	10	18	23	25	15	19	2
EABT15888	8	14	14	25	21	8	15
EABT15889	0	1	38	4	1	0	0
EABT1589	0	24	28	9	0	2	4
EABT15890	4	9	0	1	0	0	0
EABT15891	1671	14421.4	7699.86	10895.8	988.47	120	155
EABT15892	0	7	4	18	3	0	7
EABT15893	18	10	69	7	3	10	9
EABT15894	0	0	7	0	0	0	0
EABT15895	0	1	3	1	0	0	0
EABT15896	5	13	0	0	0	0	1
EABT15897	0	0	1	1	2	0	4
EABT15898	0	1	14	19	3	1	4
EABT15899	10	29	51	8	10	19	27
EABT159	1461.05	2037.05	1283	4341.86	1631.2	1095.83	1208.78
EABT1590	1478.86	1784.42	284.99	607	780	762	2487.14
EABT15900	202.48	754.44	529	1444.24	652.6	296	619.69
EABT15901	2	4	22	14	3	1	3
EABT15902	0	2	3	1	0	0	1
EABT15903	3204.09	5001.55	5208.47	10655.6	2709.43	2109.99	2044.66
EABT15904	1473.99	1543.97	1013.93	1319.47	1482.62	1149.55	929.87
EABT15905	45.11	28	8	2.9	1	1	0
EABT15906	5	4	3	7	1	1	0
EABT15907	0	8	2	4	1	2	0
EABT15908	1309.74	1904.05	2574.04	1975.63	1023.91	1190	1305.59
EABT15909	5741.51	6746.05	8492.23	3664.38	16727.4	5120.81	3671.6
EABT1591	0	1	3	0	0	2	1
EABT15910	0	1	12	1	0	0	0
EABT15911	1	1	27	0	1	0	0
EABT15912	933.8	586.39	136	181	285	780.89	2458.52
EABT15913	0	0	10	0	0	2	0
EABT15914	18493.6	9649.99	10315.8	2488.16	5421.12	2925.03	1339.88
EABT15915	1356.03	1856.81	1717.85	3666.55	1552.26	756.3	1330.74
EABT15916	8	44	77	129.98	35	20	16
EABT15917	0	1	3	5	0	0	0

EABT15918	0	0	8	5	0	2	0
EABT15919	50.78	502	1753.92	95	210	95	202.97
EABT1592	0	3	0	0	0	0	5
EABT15920	3	4	7	6	1	0	0
EABT15921	10	17	10	32	12	21	20
EABT15922	7	19	103	19	4	6	3
EABT15923	1	1	0	0	0	14	0
EABT15924	94.01	349.14	143.31	637	75.78	179.82	279.21
EABT15925	0	0	3	1	0	0	0
EABT15926	1	0	1	0	0	46	11
EABT15927	160	414.2	87	173.95	283.47	423.1	233
EABT15928	0	1	7	7	0	0	0
EABT15929	6293.73	9401.52	11132.8	12630.6	7742.96	9572.51	9771.91
EABT1593	0	0	4	0	0	0	1
EABT15930	0	1	0	8	1	1	0
EABT15931	0	0	4	0	0	0	0
EABT15932	0	3	12	0	0	2	0
EABT15933	23	27	89.04	123	17	31.27	21
EABT15934	2	0	1	0	0	1	8
EABT15935	45.03	82.01	290.91	17	33.99	6	67
EABT15936	4	3	2	0	0	2	3
EABT15937	4	8	8	115	1	13	7
EABT15938	0	0	7	0	0	0	0
EABT15939	0	0	4	3	1	0	1
EABT1594	0	0	8	0	0	1	0
EABT15940	1	0	5	0	0	0	3
EABT15941	2	2	2	8	5	0	2
EABT15942	0	3	3	4	1	0	0
EABT15943	7	20	32	17	12	22	15
EABT15944	1	0	5	1	0	0	1
EABT15945	0	0	1	2	0	0	0
EABT15946	2194	2196	1012	19902.8	1643	46	104
EABT15947	0	3	2	0	39	1	1
EABT15948	8951.56	2447.7	72	16	78.31	48502.8	1030.99
EABT15949	54	22	92	171	100	1	7
EABT1595	1	4	7	2	42	0	2
EABT15950	1	4	4	8	1	0	0
EABT15951	0	1	2	1	0	0	0
EABT15952	601.05	935.69	954.43	1621.82	905.45	318.1	484.03
EABT15953	1	0	1	0	2	0	0
EABT15954	4	2	8	1	1	2	3
EABT15955	2243.75	2454.97	1399	3252.32	1752.29	2809.98	1980.77
EABT15956	0	3	5	15	1	0	0
EABT15957	0	0	1	4	0	0	0
EABT15958	16	50	36	24.03	3	9.99	2
EABT15959	139	51	244	143	429.01	0	4
EABT1596	5485.71	10062.9	6550.8	16403.9	2498.9	637	438.97
EABT15960	2	1	0	0	0	15	0
EABT15961	1	1	5	1	2	4	1
EABT15962	4	2	1	0	0	0	1
EABT15963	6	9	1	0	2	6	15

EABT15964	3.39	9	7	5	3	0	1
EABT15965	0	0	11	0	0	0	0
EABT15966	2	8	17	7	5	1	2
EABT15967	2342.29	2396.91	536.32	396	672	430	900
EABT15968	0	2	1	2	2	2	6
EABT15969	6	9	33	15	17	4	9
EABT1597	18	93	77	94	12	4	18
EABT15970	0	0	12	0	0	0	0
EABT15971	5	8	7	14	16	0	2
EABT15972	11	38	41	28	1	2	7
EABT15973	13	40	3	6	1	1	1
EABT15974	2311.14	2392.81	475	595	684.1	15	189
EABT15975	1	2	5	2	0	0	0
EABT15976	1	6	0	2	0	0	2
EABT15977	3	6	6	2	236	1	3
EABT15978	3	1	15	0	0	0	0
EABT15979	1	16	107	1	991	13	27
EABT1598	0	3	0	0	0	1	2
EABT15980	0	7	60	8	0	0	0
EABT15981	25	62	81	61	122	6	8
EABT15982	123	240.06	195	347.99	319	135	227
EABT15983	0	1	7	0	0	0	0
EABT15984	2	2	3	0	1	0	0
EABT15985	1041.98	1536.49	708	2187.97	758.08	581.4	756
EABT15986	3	6	20	25	2	6	5
EABT15987	3	2	0	1	1	0	0
EABT15988	1	2	1	1	1	0	0
EABT15989	0	0	6	0	0	0	0
EABT1599	2	2	0	2	1	0	5
EABT15990	0	1	18	0	1	0	0
EABT15991	4	3	3	13	3	3	4
EABT15992	0	0	0	1	0	2	3
EABT15993	2	3	30	2	2	2	0
EABT15994	3	6	15	1	1	0	0
EABT15995	2	7	11	43	22.08	0	0
EABT15996	0	1	2	3	2	1	0
EABT15997	8	13	196	4	2	7	4
EABT15998	79	42	4	26	3	25	21
EABT15999	0	3	5	3	0	0	0
EABT16	0	2	4	9	2	0	1
EABT160	2	3	1	4	0	1	2
EABT1600	3	7	69	3	1	0	2
EABT16000	14889	15889.6	6982.19	14267.9	7310.31	11890	12992.5
EABT16001	49428.3	1196	0	0	0	3794.28	4
EABT16002	1	0	35	0	2008.61	10	17431.8
EABT16003	5149.8	12301.1	21148.8	26905.5	7746.14	2996.64	2898
EABT16004	3	8	18	42	7	1	1
EABT16005	2151.6	3704.84	2800.72	8063.56	3066.33	1147.93	2068.07
EABT16006	19240.4	30927.8	30929.8	26337.1	18788.5	8252.14	19112
EABT16007	4	3	10	4	2	1	1
EABT16008	1	2	4	1	0	0	0

EABT16009	1602.4	3536.9	3007.67	1884.01	2004.93	932.51	935.66
EABT1601	1	1	14	2	4	6	2
EABT16010	1	1	0	1	2	0	0
EABT16011	1	5	34	3	6	2	1
EABT16012	0	3	2	0	2	1	0
EABT16013	31	44.93	0	0	3	28	13.98
EABT16014	1316.5	3619.69	1862.77	1185.01	4059.01	526.36	2735.87
EABT16015	0	0	8	0	0	0	0
EABT16016	0	4	1	4	0	1	0
EABT16017	0	0	17	0	1	0	0
EABT16018	233	293	83	378.85	188	244	177.04
EABT16019	1	2	0	1	0	0	1
EABT1602	1557	2855.95	1882.83	5066.82	920.05	1340	929.57
EABT16020	1	2	15	3	0	1	0
EABT16021	1	3	3	1	0	0	1
EABT16022	2	5	0	4	1	1	0
EABT16023	1	1	14	2	0	0	2
EABT16024	2	1	4	1	0	0	1
EABT16025	1	3	4	1	0	1	1
EABT16026	12	26	43	10	67	2	5
EABT16027	1	0	1	0	2	0	12
EABT16028	2	0	3	3	0	0	0
EABT16029	0	3	12	2	0	0	0
EABT1603	5	6	6	15	2	0	0
EABT16030	2	3	9	1	0	5	0
EABT16031	7	11	84	6	7	10	3
EABT16032	15	35	29	59	12	17	18.37
EABT16033	0	0	1	5	1	0	0
EABT16034	1	0	11	0	2	1	0
EABT16035	0	0	4	0	0	1	0
EABT16036	1	2	9	14	3	0	6
EABT16037	1	2	2	1	0	0	1
EABT16038	0	0	7	0	0	0	0
EABT16039	0	1	1	12	1	0	2
EABT1604	2	3	73	6	3	6	4
EABT16040	0	0	0	0	7	0	10
EABT16041	0	0	2	0	0	0	0
EABT16042	1	2	3	8	24	2	3
EABT16043	1	2	21	0	0	1	0
EABT16044	0	0	43	0	0	0	0
EABT16045	0	3	0	0	1	2	2
EABT16046	1	4	5	1	1277.08	1	616
EABT16047	0	0	5	1	0	1	2
EABT16048	1	6	2	7	0	0	0
EABT16049	5	5	76	2	47	28	51
EABT1605	5654.46	3746.78	803.95	1054.47	849.96	4589.92	1256.52
EABT16050	0	2	28	27	1	2	0
EABT16051	1	3	7	7	0	1	0
EABT16052	4	2	0	0	0	9	2
EABT16053	1	4	13	5	3	2	2
EABT16054	567	3859.02	4022.13	19797	2996	111	2848

EABT16055	0	0	7	8	0	0	1
EABT16056	1	1	5	4	9	0	0
EABT16057	7	23	9	85	19	21	48
EABT16058	0	0	0	2	1	0	0
EABT16059	0	4	2	4	0	1	1
EABT1606	0	0	0	0	0	6	0
EABT16060	1	1	2	0	1	11	2
EABT16061	0	0	1	0	3	1	1
EABT16062	1	1	5	1	0	0	0
EABT16063	971.86	1338.11	3059.01	4215.86	1091	928	689
EABT16064	0	0	8	1	0	0	1
EABT16065	53	173.17	492	140	200	12	70
EABT16066	15	65	37	161	8	13	8
EABT16067	0	0	6	3	1	0	1
EABT16068	21	40	43	25	0	1	2
EABT16069	124	208	140	411.97	152	9	143
EABT1607	1	1	65	7	2	1	3
EABT16070	3	2	0	3	0	1	0
EABT16071	11	10	4	2	3	16	0
EABT16072	5	17	24	2	5	24	20
EABT16073	0	6	8.01	0	1	0	0
EABT16074	1	1	11	1	0	0	0
EABT16075	127	192	215	438	257	150.71	131
EABT16076	2	4	5	2	2	0	0
EABT16077	0	9	71	995.03	7	0	0
EABT16078	4	0	5	2	2	0	0
EABT16079	2	4	1	77	5	1	2
EABT1608	836.02	1742.35	1210.09	1530.04	1162.01	122	378.92
EABT16080	2	1	4	8	2	0	0
EABT16081	0	3	6	4	0	1	1
EABT16082	5	20	21	3	10	11	10
EABT16083	0	2	11	4	1	0	0
EABT16084	0	0	7	0	0	0	0
EABT16085	0	0	3	3	0	0	0
EABT16086	828.77	1452.11	1704.87	858	522.92	222.01	271.99
EABT16087	0	2	7	3	1	0	1
EABT16088	0	4	2	0	1	1	0
EABT16089	16	13	6	26	2	3	5
EABT1609	14	1	1	2	0	9	0
EABT16090	2	8	6	2	3	0	1
EABT16091	5	33	22	31	1	1	4
EABT16092	1740.49	2433.66	4102.72	5107.4	3513.61	2485.56	4152.56
EABT16093	0	1	7	1	2	0	2
EABT16094	1	1	24	2	1	2	2
EABT16095	7123.73	8635.72	5860.11	8115.49	3932.35	5076.41	4861.84
EABT16096	7	23	30	11	3	0	6
EABT16097	195	242	419	497.06	335.69	150.37	168.99
EABT16098	7	16	32	2	5	0	0
EABT16099	0	0	5	1	1	1	2
EABT161	0	0	3	43	5	0	0
EABT1610	1	1	4	4	6	0	4

EABT16100	0	0	3	2	1	0	0
EABT16101	1	2	14	2	1	1	1
EABT16102	3	6	8	12	4	0	3
EABT16103	87	96	31	1	7	2	4
EABT16104	133	168.17	92	137	188	64.81	279.98
EABT16105	616	902	530	1166.02	768	324	370
EABT16106	0	0	7	1	0	0	0
EABT16107	7	29	18	5	2	4	2
EABT16108	5	2	31	3	1	2	0
EABT16109	0	1	7	0	0	0	0
EABT1611	553.77	814.84	1002.38	1773.59	1053.13	653.79	607.14
EABT16110	2	11	1	0	2	7	2
EABT16111	0	0	14	0	0	0	0
EABT16112	1	0	3	5	0	0	0
EABT16113	509	636.99	467.01	881	548	293	348
EABT16114	17	29	0	0	0	3	0
EABT16115	0	7	3	4	0	0	0
EABT16116	0	2	2	3	0	0	0
EABT16117	0	0	0	0	0	0	0
EABT16118	1	0	6	1	0	0	0
EABT16119	1	8	7	6	4	0	2
EABT1612	0	1	5	1	0	1	0
EABT16120	0	0	4	13	1	0	0
EABT16121	2	8	0	0	7	0	0
EABT16122	0	1	7	0	0	0	2
EABT16123	0	2	10	3	1	0	0
EABT16124	0	0	3	0	2	0	0
EABT16125	0	2	0	0	0	43	0
EABT16126	0	0	2	1	0	0	0
EABT16127	2	3	4	30	1	3	1
EABT16128	0	0	1	1	3	0	2
EABT16129	0	12	3	10	0	1	4
EABT1613	351.03	186.03	467	138.94	592.91	193	858.6
EABT16130	2	0	4	0	0	0	0
EABT16131	0	1	4.99	0	1	0	0
EABT16132	0	0	0	1	1	2	0
EABT16133	0	4	15	2	4	4	1
EABT16134	5	12	21	23	7	1	2
EABT16135	20	39	47	464	4533.88	5	8791.87
EABT16136	11	23	3	0	0	0	0
EABT16137	30	90	258	321	177	15	26
EABT16138	0	0	7	1	1	1	6
EABT16139	1	3	28	2	0	0	0
EABT1614	1	0	3	12	0	0	1
EABT16140	1	0	1	0	1	6	3
EABT16141	0	2	13	5	0	2	0
EABT16142	65	74.12	98	80	34	3	47
EABT16143	46	295	423	499	362	32	111
EABT16144	0	2	2	0	0	2	0
EABT16145	1	0	5.05	0	0	0	0
EABT16146	0	3	4	0	2	0	1

EABT16147	0	1	5	1	0	0	0
EABT16148	0	3	6	1	0	1	0
EABT16149	0	0	17	4	1	0	0
EABT1615	0	1	6	0	0	0	0
EABT16150	1	2	6	3	0	1	1
EABT16151	0	0	2	0	13	0	4
EABT16152	68	97	69	91	77	12	23
EABT16153	0	0	4	0	0	0	0
EABT16154	62	241	1378.86	258	316	28	246.28
EABT16155	9.33	14	37.98	3	2	2	0
EABT16156	0	2	3	4	0	0	0
EABT16157	628.06	1051.84	1014.95	1331.16	715	569.68	622.45
EABT16158	2	7	207.94	8	8	4	2
EABT16159	81	180	198	1395.9	832.99	79	53
EABT1616	449.84	773.85	744.18	2141.99	776.99	101.93	535.45
EABT16160	1465.48	678	87	354.59	310.96	990.21	742.99
EABT16161	2	17	17	15	2	0	0
EABT16162	0	1	2	0	0	0	8
EABT16163	10	3	2	0	0	0	0
EABT16164	2	5	10	3	1	0	0
EABT16165	28	26	9	5	1	0	1
EABT16166	0	4	10	7	1	2	2
EABT16167	1	1	8	4	0	0	0
EABT16168	0	0	0	3	0	0	0
EABT16169	2	4	9	3	1	0	3
EABT1617	3144.79	2775.04	1396	5001.37	1123.02	1095.35	1044.86
EABT16170	84	253	2088.68	331.63	302	47	200
EABT16171	1	3	5	14	23	0	5
EABT16172	0	0	3	0	0	2	0
EABT16173	3	0	2	1	0	1	0
EABT16174	0	0	9	1	0	0	0
EABT16175	0	4	0	0	2	0	0
EABT16176	0	0	1	0	1	0	0
EABT16177	0	2	7	72	1	0	0
EABT16178	2	2	76	1	0	0	0
EABT16179	3	0	11	5	21	4	1
EABT1618	0	1	5	0	0	0	0
EABT16180	0	1	5	2	0	0	1
EABT16181	0	0	7	1	3	0	6
EABT16182	9	10	0	0	0	0	0
EABT16183	1	2	16	2	0	0	0
EABT16184	16093.9	23742.7	15833.6	24251.7	6387.13	8727.73	6889.99
EABT16185	39	124	1	7	2	6	10
EABT16186	1	3	2	1	2	0	0
EABT16187	4679.92	11480.3	31679.1	21811.9	11687.9	8796.17	7783.96
EABT16188	1	6	12	2	3	0	2
EABT16189	1	1	0	0	0	0	1
EABT1619	308.87	478.84	379.04	779.94	444.3	142	209.98
EABT16190	5	5	3	6	2	3	7
EABT16191	0	0	2	7	2	2	0
EABT16192	9	21.88	55.29	0	1	3.61	9

EABT16193	0	0	2	0	1	0	0
EABT16194	14	38	6	8	1	0	0
EABT16195	1123.19	1501.78	1317.4	2873.53	1320.33	674.86	637.32
EABT16196	0	1	3	4	1	0	0
EABT16197	0	1	7	0	1	0	0
EABT16198	1	3	10	18	0	0	0
EABT16199	166	263.01	251.13	394.63	341.57	129	181
EABT162	1796.38	2086.52	2532.11	2503.95	1618	1522.96	1018.15
EABT1620	0	0	0	4	0	0	0
EABT16200	10644.7	18884.1	29289.8	19031.4	15473.9	10512.6	11091.1
EABT16201	434.25	651.51	632.79	881.07	687.25	280	437
EABT16202	0	4	4	0	0	0	0
EABT16203	1687.6	917.94	713.54	954.6	849	2179	1912.98
EABT16204	0	2	19	2	0	0	0
EABT16205	1	8	4	4	0	0	0
EABT16206	4158.8	7437.01	7960.95	5943.1	6759.05	3172.18	3558.79
EABT16207	0	0	5	4	0	0	0
EABT16208	3	10	3	3	1	0	4
EABT16209	2	3	34	0	24	9	36
EABT1621	6700.35	8001.76	44460.7	2637.97	41119.1	5963.7	5961.52
EABT16210	18	2	0	1	0	38	0
EABT16211	26	17	0	0	1	10	0
EABT16212	137	200	659	493.75	418.9	175	238.91
EABT16213	3	3	7	10	0	1	1
EABT16214	0	0	1	0	1	0	3
EABT16215	0	1	3	2	1	0	1
EABT16216	759.36	840.35	2220.16	1804.73	788.6	361.49	406
EABT16217	0	1	2	3	0	0	0
EABT16218	0	0	5	0	0	0	0
EABT16219	1	1	31	3	3	1	1
EABT1622	0	3	0	0	1	1	0
EABT16220	0	0	0	0	1	0	20
EABT16221	1	9	11	1	49.48	0	1
EABT16222	2289.85	2806.61	2125.83	76.01	457.36	19	248.92
EABT16223	1	12	18	16	4	2	6
EABT16224	1755.96	2363.31	1827.95	3214.76	1240.28	1516.97	1187.25
EABT16225	0	1	1	0	0	1	0
EABT16226	3	6	4	10.54	5	4	0
EABT16227	1795.05	3167.28	2610.49	3774.56	1263.83	1859.37	1589.71
EABT16228	1315	1816.13	990	1886	1675	937.01	1086.01
EABT16229	0	0	4	1	0	0	0
EABT1623	0	0	7	1	1	0	2
EABT16230	2	4	7	17	14	1	3
EABT16231	3	0	46	2	0	2	2
EABT16232	0	0	2	2	0	0	0
EABT16233	0	5	0	0	0	0	0
EABT16234	1	2	9	3	1	0	0
EABT16235	0	0	11	4	0	2	0
EABT16236	1	7	8	7.25	5	0	1
EABT16237	404.17	532.02	414.61	506.21	515.69	414.01	433.98
EABT16238	0	2	6	1	1	0	0

EABT16239	0	1	0	5	1	0	0
EABT1624	0	0	0	0	27	0	1
EABT16240	0	3	23	4	1	3	1
EABT16241	0	0	4	9	0	1	1
EABT16242	0	1	3	1	0	0	0
EABT16243	1	1	5	0	0	0	0
EABT16244	0	1	5	6	0	0	1
EABT16245	2	8	19.99	34	3	5	5
EABT16246	1	1	8	0	1	0	1
EABT16247	0	2	4	2	0	0	0
EABT16248	3	16	92	24	2	2	1
EABT16249	0	0	4	2	0	0	1
EABT1625	10	2	0	1	4	0	8
EABT16250	218.14	530.6	687	1790.99	387	94	196
EABT16251	6078.07	6525.75	6203.96	4687.02	3648.52	5667.19	4224.77
EABT16252	8	31	46	83	1	0	0
EABT16253	0	3	4	0	0	0	0
EABT16254	22	6	0	0	0	7	0
EABT16255	1	5	1	0	27	5	24
EABT16256	1	0	1	1	0	0	0
EABT16257	8	9	0	0	3	29	25
EABT16258	3	1	0	0	2	2	6
EABT16259	27	74	130	49.46	69	9	2
EABT1626	489.6	594.63	419.93	941	683.54	272.4	326.61
EABT16260	0	0	1	3	0	0	0
EABT16261	5	4	6	1	0	1	0
EABT16262	1694.54	3052.38	2767.1	3641.94	1539.5	884.67	1098.37
EABT16263	2928.79	3697.25	3615.91	2545.74	3859.78	246	967.91
EABT16264	0	0	3	0	1	0	0
EABT16265	0	0	11	1	0	0	0
EABT16266	13	19	31	30	3	13	12
EABT16267	711.93	1148.4	840.03	4467.74	1883.68	856.79	995.05
EABT16268	2	1	4	0	0	0	2
EABT16269	2	2	14	5	7	0	3
EABT1627	2	3	21	0	0	0	1
EABT16270	1	1	234	2	0	2	1
EABT16271	0	1	5	1	0	2	0
EABT16272	0	0	11	0	0	0	0
EABT16273	1	0	7	1	0	0	1
EABT16274	1	1	4	0	1	0	0
EABT16275	159	120	34	17	354.95	381	2195.88
EABT16276	1	4	6	2	1	1	0
EABT16277	0	0	0	19	0	0	0
EABT16278	32	24	31	14	141	38	71
EABT16279	10	16	97	6	6	4	4
EABT1628	5	12	36	12	2	2	3
EABT16280	0	0	4	0	0	0	0
EABT16281	1	4	24	2	2	0	0
EABT16282	6342.43	25877.4	49116	27504.3	10281.8	621	4527.38
EABT16283	1	5	9	0	6	3	15
EABT16284	5	8	4	7	0	0	1

EABT16285	0	2	4	2	1	0	0
EABT16286	2478.11	3258.58	6019.95	4115.09	3639.98	2292.81	1998.39
EABT16287	0	2	6	0	0	0	0
EABT16288	1	8	5	33	3	1	0
EABT16289	46	123	134	99.21	55	14	91
EABT1629	0	0	10	0	0	0	0
EABT16290	4	1	3	1	3	1	1
EABT16291	0	3	29	0	0	0	0
EABT16292	1	3	5	1	0	0	1
EABT16293	0	3	7	0	2	1	1
EABT16294	0	2	4	1	2	0	0
EABT16295	0	0	1	0	2	0	0
EABT16296	14653.7	6407.51	1501.88	484.92	1941.56	39	4682
EABT16297	2	6	3	1	0	1	0
EABT16298	0	1	11	0	1	0	0
EABT16299	1	1	8	20	11	1	1
EABT163	1	0	2	0	800.59	1	5
EABT1630	0	1	27	2	2	1	1
EABT16300	0	2	2	0	0	0	0
EABT16301	3	0	17	7	3	0	3
EABT16302	285.04	321.97	187	580.79	406	203	215
EABT16303	2287.28	3432.96	3094.36	6197.08	1828.23	2885.22	2110.03
EABT16304	3138.73	5940.4	6062.71	5565.86	3404.77	3273.26	4935.69
EABT16305	5.02	0	4	0	4	3	0
EABT16306	0	1	7	0	0	1	0
EABT16307	0	1	19	0	0	0	0
EABT16308	0	0	6	1	8	0	0
EABT16309	0	0	2	1	2	0	1
EABT1631	0	0	5	3584.68	124251	30	267
EABT16310	0	0	0	0	0	0	1
EABT16311	780.93	1508.87	2229.03	4507.47	1229.97	542.87	683.06
EABT16312	0	0	8	0	0	1	0
EABT16313	1	10	4	13	3	3	5
EABT16314	0	4	11	38	0	0	0
EABT16315	1	6	0	0	0	0	0
EABT16316	0	0	6	2	0	0	0
EABT16317	76	142	495.67	67	226	4	78
EABT16318	0	2	2	1	4	1	3
EABT16319	2	1	1	0	1	0	0
EABT1632	1714.95	1859.05	775.82	1941.81	1010.1	1750.3	2631.15
EABT16320	3	3	5	2	2	1	4
EABT16321	1	0	2	3	1	4	1
EABT16322	755.09	1354.92	1471.77	3048.06	1331.77	737.68	810.33
EABT16323	0	1	4	3	1	0	0
EABT16324	0	0	7	0	0	1	0
EABT16325	4350.28	6699.19	6725.8	9758.01	3738.63	2117.71	2990.03
EABT16326	16	90	1077.41	356	88	17	8
EABT16327	935.57	1250.7	745	1571.81	917.98	1140	620.54
EABT16328	0	0	1	18	0	0	0
EABT16329	12	18	69	53	1	13	7
EABT1633	6	24	35	9	4	0	2

EABT16330	1	1	0	5	0	0	0
EABT16331	0	3	9	10	1	1	3
EABT16332	0	0	19	14	0	0	0
EABT16333	284	389	829.98	2408.44	421.56	128.98	125.01
EABT16334	1	0	11	1	0	0	0
EABT16335	0	0	8	3	1	0	0
EABT16336	1	1	4	0	0	1	0
EABT16337	2	4	124	0	0	1	5
EABT16338	733	852.92	738	896	796	615	566.88
EABT16339	0	1	6	1	3	0	0
EABT1634	0	0	2	1	0	0	0
EABT16340	0	1	0	2	2	0	2
EABT16341	0	1	1	8	11	0	3
EABT16342	5634.02	12102.2	5889.14	3927.59	8747.92	5327.26	10521.3
EABT16343	2	0	1	1	26	0	0
EABT16344	4	16	8	28	4	3	3
EABT16345	0	0	1	1	0	0	0
EABT16346	0	1	2	1	0	1	0
EABT16347	5	10	42	8	2	7	7
EABT16348	0	0	11.76	0	1	0	0
EABT16349	0	1	0	0	0	2	5
EABT1635	2	1	0	2	1	0	3
EABT16350	0	8	9	10	9	4	5
EABT16351	2	3	1	0	0	0	0
EABT16352	1	8	14	2	2	23	14
EABT16353	9	23	25	98	5	5	2
EABT16354	0	1	31	0	1	0	0
EABT16355	4172.68	5307.49	3124.18	2916.84	2166	2277.94	2558.02
EABT16356	3	0	1	7	0	2	1
EABT16357	0	0	0	0	0	0	0
EABT16358	1	7	5	13	2	0	0
EABT16359	2	0	4	2	1	1	2
EABT1636	0	2	3	4	1	0	0
EABT16360	3	0	1	0	0	1	1
EABT16361	26	93	101.02	101.99	17	15	26
EABT16362	0	0	3	2	0	0	0
EABT16363	9	4	9	3	1	1	7
EABT16364	2	5	6	6	1	2	1
EABT16365	2	0	2	7	7	0	0
EABT16366	6	3	0	11	6	0	2
EABT16367	9	5	21	9	0	0	9
EABT16368	0	4	6	0	0	0	0
EABT16369	0	1	7	1	0	0	0
EABT1637	1	0	1	0	0	1	1
EABT16370	48	136	133	102	122.22	19	115
EABT16371	3	14	15.98	3	67	1	1
EABT16372	9	33	61	75	24	1	17
EABT16373	1	3	1	8	1	0	1
EABT16374	0	2	12	5	3	1	1
EABT16375	0	2	4	0	0	0	0
EABT16376	17	16.75	9	30	4	6.99	8

EABT16377	1	1	7	0	0	0	0
EABT16378	0	1	3	11	0	1	2
EABT16379	0	0	9	1	0	0	0
EABT1638	1	2	7	0	1	0	1
EABT16380	8	66	48	58.79	208	2	7
EABT16381	1576.02	2438	740.03	156	1253.01	964.89	1463.66
EABT16382	0	1	3	0	0	0	0
EABT16383	0	14	1	0	1	0	0
EABT16384	1	2	3	3	1	0	0
EABT16385	1	6	5	3	0	2	1
EABT16386	676	1026	1823.03	2766.98	1041	504	471
EABT16387	0	1	4	1	0	0	1
EABT16388	1	16	9	1	1	1	0
EABT16389	0	1	7	0	11	1	0
EABT1639	0	0	2.99	1	1	0	0
EABT16390	14	15	10	77	5	6	10
EABT16391	75.83	125.03	81	223	253	40	206
EABT16392	0	4	3	1	4	3	1
EABT16393	2	5	3	3	1	0	2
EABT16394	2	0	13	1	0	0	0
EABT16395	0	3	4	4	1	2	0
EABT16396	8	22	212	20	11	21	9
EABT16397	0	0	2	0	0	0	0
EABT16398	247.03	426	286	692.01	283.33	239	271.87
EABT16399	1	2	0	3	1	0	0
EABT164	5268.56	6774.46	3967.46	6185.53	3205.5	4845.28	4275.3
EABT1640	0	2	5	4	0	1	1
EABT16400	2542.23	2908.57	1718.18	227	3766.61	1735.16	14002.5
EABT16401	0	0	1	2	5	0	3
EABT16402	61	207	98	69	23	54	81
EABT16403	45	49	1	0	1	70	6
EABT16404	2	1	8	21	3	0	0
EABT16405	1	0	22	7	1	0	0
EABT16406	3	13	18	14	5	1	0
EABT16407	4695.22	10057.7	3879.86	2828.06	7315.12	2540.77	3254.9
EABT16408	2	3	1	2	1	41	2
EABT16409	1	2	2	0	1	0	0
EABT1641	1	0	25	10	1	0	0
EABT16410	607.99	884.95	837.76	1543.6	481	391.79	361
EABT16411	0	0	6	1	0	0	0
EABT16412	24	45.27	196	59	378	27	54
EABT16413	0	0	32	0	0	0	0
EABT16414	48	126	40	221	64	57	61
EABT16415	1	1	35	16	14	0	0
EABT16416	0	1	13	1	0	0	0
EABT16417	4	0	4	0	0	6	9
EABT16418	0	3	0	0	1	0	1
EABT16419	1	0	36	3	15	0	7
EABT1642	0	0	0	19	2	0	4
EABT16420	7	7	177	14	4	6	3
EABT16421	0	2	1	3	10	0	0

EABT16422	1	4	9	1	0	0	0
EABT16423	5	19	116.03	31	12	9	13
EABT16424	0	2	2	6	4	0	0
EABT16425	858.94	1785.33	2325.49	3085.05	1788.49	1093.8	1360.09
EABT16426	0	0	7	0	0	0	0
EABT16427	16	43	18	14	6	29	25
EABT16428	3	10	0	2	1	4	7
EABT16429	1	1	4	0	0	0	0
EABT1643	1	2	12	0	2	0	1
EABT16430	0	2	3	6	2	2	1
EABT16431	0	0	6	0	0	0	0
EABT16432	95.79	158	384.02	1104.01	604.55	18	168
EABT16433	257.19	64.92	46	47	84.02	975.19	447.22
EABT16434	6	7	18	9	5	1	4
EABT16435	5489.08	9395.55	1335.29	573.04	3226.54	149.99	1410.73
EABT16436	1	0	8	3	0	0	0
EABT16437	3117.8	5434.55	11766.6	11400.9	4268	4479.24	3786.5
EABT16438	2	8	8	1	4	0	8
EABT16439	19	44	431.06	391.7	399.57	16	9
EABT1644	2699.88	4090.21	5905.3	9687.84	4801.24	2962.06	2764.84
EABT16440	1	2	2	2	0	0	1
EABT16441	0	2	5	7	2	0	0
EABT16442	1	2	28	2	0	0	0
EABT16443	177	825.67	2317.96	2212.63	863.1	81	222.06
EABT16444	2188	3804.16	3723.05	6600.06	1967.14	913	470
EABT16445	1	8	7	6	2	1	1
EABT16446	0	1	0	4	2	1	0
EABT16447	2312.5	4366.05	1675.24	4784.71	2540	2655.08	7595.82
EABT16448	6	6	23	8	1	0	0
EABT16449	0	0	3	0	2	0	3
EABT1645	2	8	14	1	1	2	2
EABT16450	67	77	621	33	127	126	121
EABT16451	0	0	0	9	0	0	0
EABT16452	12	15	42	13	4	4	2
EABT16453	3	2	9	13	2	1	4
EABT16454	5891.07	3471.91	570	246.01	1427	7608.41	6186.71
EABT16455	1047.57	1121.04	485.61	814	1029.5	800.06	696.98
EABT16456	2	2	5	0	0	1	1
EABT16457	568	804.07	1302.93	1142.01	647.3	539.05	396.99
EABT16458	17	46	32	57.09	14	18	10
EABT16459	3	2	7	1	0	0	0
EABT1646	0	1	6	0	0	0	0
EABT16460	8	30	152	399	11	3	3
EABT16461	0	0	6	0	1	2	2
EABT16462	0	3	24	19	0	1	1
EABT16463	1	2	0	2	1	0	1
EABT16464	6	1	14	0	0	0	0
EABT16465	1294.14	1754.34	1159.53	2794.77	848.61	932.83	690.77
EABT16466	0	1	4	0	1	0	2
EABT16467	156.8	305	274.01	373	256.73	17	200
EABT16468	1	0	12	0	0	0	0

EABT16469	4	8	0	1	2	2	8
EABT1647	6	14	39	11	1	1	0
EABT16470	0	0	0	0	0	0	1
EABT16471	5	5	26	45	9	7	4
EABT16472	0	0	2	2	0	0	0
EABT16473	0	1	5	6	23	0	0
EABT16474	0	1	4	0	0	0	0
EABT16475	0	0	6	0	0	0	0
EABT16476	0	0	11	1	0	0	1
EABT16477	0	0	2	5	1	0	1
EABT16478	0	0	27	0	0	0	0
EABT16479	7	21	14	238	11	0	23
EABT1648	4	3	4	5	0	2	0
EABT16480	5	11	116	7	1	0	4
EABT16481	5	10	3	0	0	1	0
EABT16482	0	0	7	0	1	0	1
EABT16483	2	2	55	0	3	3	2
EABT16484	1	0	7	0	3	0	0
EABT16485	15	5	1	3	2	1	0
EABT16486	6	0	0	0	0	6	1
EABT16487	10	12	4	4	3	7	11
EABT16488	30	71	1273.75	120	29.86	14.14	9
EABT16489	96.6	210.89	68.13	354.1	53	52.03	65
EABT1649	5256.67	6152.86	3915.38	5013.07	2877.03	2834.97	2044.23
EABT16490	1	5	4	2	8	0	5
EABT16491	45	13	6	7	2	1	1
EABT16492	41	134	2756.03	14856.9	185.1	43.13	57
EABT16493	8	14	10	9	4	2	1
EABT16494	2	1	2	2	0	0	0
EABT16495	473	854	862.99	57	1565.91	610	6731.08
EABT16496	0	1	4	0	0	0	0
EABT16497	1	1	9	2	2	0	0
EABT16498	1	0	5	3	0	0	0
EABT16499	0	2	25	1	2	6	0
EABT165	4540.25	7540.33	7825.2	12922.3	8463.61	4073.22	9172.4
EABT1650	1	0	8	3	0	0	0
EABT16500	18	41	56	14	12	10	6
EABT16501	0	0	9	0	0	0	0
EABT16502	1	2	17	1	0	0	0
EABT16503	5	7	1	36	18	2	13
EABT16504	2	1	0	0	2	0	0
EABT16505	12	20	13	30	7	3	3
EABT16506	2	0	0	1	1	1	0
EABT16507	1	3	0	0	0	1	2
EABT16508	12	15	0	1	0	3	1
EABT16509	1	4	31.25	2	0	0	1
EABT1651	0	0	0	5	0	0	0
EABT16510	0	3	2	4	0	2	1
EABT16511	0	0	7	0	0	1	0
EABT16512	10	38.06	15	252	38	0	20
EABT16513	4	4	15	2	2	1	1

EABT16514	5	6	33	5.45	1	0	2
EABT16515	0	0	1	0	0	0	0
EABT16516	0	1	1	2	0	0	0
EABT16517	0	0	9	0	17	0	38
EABT16518	2	2	1	0	0	0	0
EABT16519	1134	1558	1263	2891.47	767	724	617
EABT1652	2	1	24	3	0	0	0
EABT16520	8108.03	14280	16576.7	9586.14	8297.41	7893.49	7727.4
EABT16521	0	0	2	3	1	0	0
EABT16522	0	0	7	17	26	0	0
EABT16523	0	1	3	0	0	0	0
EABT16524	0	3	6	0	0	1	0
EABT16525	13694.8	23149.5	32074	41417.1	18060.4	9618.5	12718.4
EABT16526	2	4	76	4	2561.79	4	16
EABT16527	0	0	4	1	0	0	0
EABT16528	1	0	8	0	0	1	0
EABT16529	634	381	106	255	236	279	137
EABT1653	0	2	3	24	2	0	1
EABT16530	0	0	0	1	3	0	1
EABT16531	6	3	6	8	2	4	2
EABT16532	25	98	338	121.64	90	17	23
EABT16533	1	2	3	2	0	1	0
EABT16534	18	113	177	433	87	1	28
EABT16535	3	27	18	0	0	7	1
EABT16536	2151.22	3719.05	2198.5	3666.52	2495.84	1225.52	1519.41
EABT16537	0	0	4	0	2	0	0
EABT16538	2	1	0	0	2	0	0
EABT16539	1	6	2	30	1	1	1
EABT1654	0	0	0	0	0	0	0
EABT16540	0	0	4	1	0	0	16
EABT16541	3	3	2	4	0	2	6
EABT16542	3	2	68	6	0	1	1
EABT16543	0	0	5	0	1	0	0
EABT16544	13	24	8	3	26	0	10
EABT16545	3	2	6	0	0	1	0
EABT16546	0	0	39	1	0	0	0
EABT16547	1502.27	2538.43	3594.37	3284.51	1856.73	2467.15	1534.24
EABT16548	15	15	9	49	12	0	1
EABT16549	1	0	1	0	2	0	1
EABT1655	250	20	2	16	51	9	33
EABT16550	0	1	17	1	0	0	0
EABT16551	0	0	0	5	0	0	0
EABT16552	1	2	5	5	0	0	0
EABT16553	2	5	2	8	5	1	0
EABT16554	0	1	3	0	1	0	1
EABT16555	4	4	0	1	0	10	1
EABT16556	0	0	0	0	0	0	8
EABT16557	0	1	6	0	0	0	0
EABT16558	0	0	12	5	0	0	0
EABT16559	0	0	6	0	0	0	0
EABT1656	3	5	103	4	1	2	3

EABT16560	2	1	3	4	1	2	1
EABT16561	92	183	368	778.01	1092.61	15	183.6
EABT16562	1	3	4	0	0	3	0
EABT16563	0	0	4	0	0	0	0
EABT16564	0	0	8	0	0	0	0
EABT16565	2	7	27	18	7	3	4
EABT16566	3	6	17	2	1	1	2
EABT16567	15	31	36	31	4	25	8
EABT16568	0	0	1	0	0	0	0
EABT16569	0	0	6	0	1	0	0
EABT1657	0	0	2	0	1	1	3
EABT16570	0	1	8	5	5	1	4
EABT16571	1	2	0	0	0	0	3
EABT16572	0	1	5	0	0	0	0
EABT16573	0	0	8	1	1	0	0
EABT16574	0	7	2	5	1	0	0
EABT16575	199.98	248.81	46	53	24	186	35
EABT16576	7	6	4	3	6	0	16
EABT16577	0	2	3	8	0	1	0
EABT16578	2079.12	5621.6	3067.15	1743.11	1850.36	2546.97	2312.82
EABT16579	1479.65	2264.28	2418.16	5233.75	1557.02	1224.91	1314.88
EABT1658	0	0	1	2	2	0	1
EABT16580	2442.22	7612.3	19300.1	4021.5	3977.06	1117.11	1277.31
EABT16581	1	1	15	0	2	0	0
EABT16582	921	1115.29	780	1669	1026.25	695	648
EABT16583	1	3	10	6	0	0	1
EABT16584	3	10	27	0	1	0	0
EABT16585	0	0	10	0	0	5	0
EABT16586	926.19	1367.31	1524.62	1523.59	850.87	1137.69	1303.45
EABT16587	1	6	16	4	0	0	1
EABT16588	0	0	9	4	1	0	1
EABT16589	8	6	0	11	10	0	3
EABT1659	0	1	8	0	4	0	0
EABT16590	3	7	7	7	0	0	1
EABT16591	1	0	8	1	0	0	0
EABT16592	3	8	47	4	10	0	5
EABT16593	1759.49	3169.36	2093	3948.98	4489.95	1327.99	1928.99
EABT16594	0	3	5	5	1	0	1
EABT16595	1082.39	1172.03	363.36	1662.83	1116.18	247.5	417.03
EABT16596	5	10	13	45	1	0	0
EABT16597	1	1	3	0	1	0	0
EABT16598	9	11	28	10	4	7	20.64
EABT16599	3	7	110	27	4	5	3
EABT166	0	0	1	3	1	0	0
EABT1660	0	1	6	0	0	0	1
EABT16600	734	994.55	1171.01	1836.12	755.31	483.97	520
EABT16601	0	1	8	2	0	0	0
EABT16602	0	0	0	0	3	1	20
EABT16603	3832.52	5542.37	6156.01	6700.61	4902.92	3504.01	3296
EABT16604	0	2	6	1	1	0	0
EABT16605	0	0	5	0	0	0	0

EABT16606	0	4	5	3	0	0	1
EABT16607	20727.9	2653.03	3422.5	3595.69	2633.87	94	247
EABT16608	0	1	7	5	1	0	0
EABT16609	1	6	16	5	1	0	1
EABT1661	2	0	9	3	0	0	1
EABT16610	0	1	3	0	0	1	0
EABT16611	0	6	3	0	0	1	1
EABT16612	1325.92	2003.36	1544.9	3045.03	1597.97	1399.11	1363.92
EABT16613	3	2	13	0	0	0	0
EABT16614	11	16.92	72	327	14	7	3
EABT16615	0	0	0	0	1	2	4
EABT16616	0	1	1	0	2	3	10
EABT16617	9	2	2	0	2	0	0
EABT16618	0	2	10	12	0	0	0
EABT16619	624	737	409	1164.03	802.13	668	635.72
EABT1662	5276.79	6589.2	5064.68	7393.09	4491.38	4768.08	4693.7
EABT16620	10457.8	8613.27	13257.4	5762.15	2510.47	3730.56	2863.2
EABT16621	0	1	6	1	0	0	0
EABT16622	1	2	1	15	1	0	0
EABT16623	11208.6	21493.8	32190.4	32699.7	7607.4	11177.4	8100.55
EABT16624	70	62.01	10	40	8	0	2
EABT16625	1	1	0	17	1	0	18
EABT16626	3	11	52.94	21	3	2	0
EABT16627	0	2	1	5	0	0	0
EABT16628	0	5	3	2	0	0	0
EABT16629	2	18	2	0	0	40	0
EABT1663	1	3	3	1	1	0	0
EABT16630	2062.31	3197.27	2203.6	3968.23	1952.01	1566.56	1733.84
EABT16631	0	0	3	0	2	0	1
EABT16632	5	22	41	26	2	11	8
EABT16633	3	2	0	0	0	4	0
EABT16634	0	9	16	17	0	0	0
EABT16635	5	7	8	2	1	2	0
EABT16636	1	1	16	1	0	0	1
EABT16637	318.56	1730.3	3505.33	1285.94	671.32	92	195.14
EABT16638	2	0	7	3	1	0	0
EABT16639	1	0	1	0	0	0	0
EABT1664	137.99	13951.8	14411.3	11956.9	457.52	325.84	2277.64
EABT16640	2	1	6	0	0	0	0
EABT16641	8	16	22	160	59	1	8
EABT16642	5	4	10	85	3	2	22
EABT16643	0	1	1	5	7	0	3
EABT16644	220.97	430	1478.28	2429	382	112	278
EABT16645	677.47	856.11	529.56	805.34	1008.56	458.38	625.73
EABT16646	0	12	0	2	6	0	0
EABT16647	0	0	1	2	4	0	0
EABT16648	3	1	4	8	0	38	0
EABT16649	0	0	15	1	1	0	0
EABT1665	1	1	10	0	0	0	0
EABT16650	1	16	30	15	3	0	2
EABT16651	0	0	0	0	0	0	0

EABT16652	340	495	339	687.45	191.87	44	142
EABT16653	0	0	7	8	1	0	1
EABT16654	0	0	1	0	1	0	2
EABT16655	0	1	3	0	0	0	0
EABT16656	5	2	0	0	0	1	1
EABT16657	817.03	1228.4	1080.71	1368.08	805.94	673	878.01
EABT16658	0	0	1	2	1	0	0
EABT16659	0	8	16	12	0	0	0
EABT1666	0	2	7	2	1	0	0
EABT16660	0	0	4	6	0	0	0
EABT16661	0	4	0	8	0	0	0
EABT16662	8	25	7	3	2	8	29
EABT16663	4112.34	4902.34	3800.27	5808.51	3365.92	3216.76	3045.06
EABT16664	1	8	21	1	0	1	0
EABT16665	2	1	4	0	0	0	0
EABT16666	8	28	57.98	37	14	0	0
EABT16667	2	6	0	0	1	1	0
EABT16668	4374.16	7551.77	8614.62	14572.8	6339.69	3839	3602.4
EABT16669	8	14	1	0	0	0	0
EABT1667	27	145	132	114	26	8	33.01
EABT16670	850.31	1067.27	854.37	1222.31	848.57	657.32	771.5
EABT16671	0	0	3	1	0	0	0
EABT16672	11	14	9	31	15	4	6
EABT16673	31	46	28	60	73	2	39
EABT16674	44	18	2	0	0	0	0
EABT16675	14	54	36	47	32	18	19
EABT16676	0	1	1	5	1	0	0
EABT16677	0	3	15	3	3	2	1
EABT16678	0	0	8	0	0	0	0
EABT16679	2	1	7	2	0	0	0
EABT1668	1	0	1	3	0	0	1
EABT16680	694.54	1450.5	1690.22	4002.98	842.03	643.67	577.16
EABT16681	3	26	4	3	3	3	1
EABT16682	0	0	13	0	0	0	0
EABT16683	5738.01	138	6	3	12379.2	892	100314
EABT16684	0	0	9	1	0	0	0
EABT16685	3	1	1	2	2	2	0
EABT16686	0	3	3	0	1	0	0
EABT16687	0	8	5	3	4	0	0
EABT16688	0	1	5	1	0	0	1
EABT16689	3	6	3	0	0	0	0
EABT1669	18	30	26	91	33	8	16
EABT16690	1169	1854.53	1020.66	3351.77	1307.41	379	803.97
EABT16691	88	140	86	118	50	24	52
EABT16692	584.01	502.94	140.01	567.21	414.06	478.57	525.34
EABT16693	1	0	4	0	0	0	0
EABT16694	285	565.77	831.66	790.34	1065.57	341.46	496
EABT16695	0	5	0	0	0	10	1
EABT16696	0	2	2	6	0	1	1
EABT16697	1	8	1	2	1	3	2
EABT16698	0	3	2	3	2	0	0

EABT16699	270.88	1143.08	1765.77	134	32	31	54.19
EABT167	4	2	13	3	1	5	1
EABT1670	1	0	0	0	0	2	0
EABT16700	313.04	472.31	292.86	333.98	283.47	187.82	273.77
EABT16701	909	1237	954.67	1577.14	937.87	724.89	691.22
EABT16702	3	52	201	0	14	1	20
EABT16703	0	4	7	1	0	0	0
EABT16704	723	1107.85	536	861	578.98	699	871.01
EABT16705	1049.26	1446.77	4092.78	3505.09	148	93.04	59
EABT16706	0	5	0	15	1	0	0
EABT16707	936	1403.39	1272.78	3106.14	1104	498.1	400
EABT16708	3	3	0	0	0	1	1
EABT16709	0	7	10	2	3	0	1
EABT1671	0	0	7	1	0	0	0
EABT16710	0	2	10	8	0	0	0
EABT16711	342	557	324	655	417	329	310
EABT16712	0	0	2	1	1	1	1
EABT16713	2	1	10	2	0	0	0
EABT16714	4	16	36	20	11	5	6
EABT16715	0	0	19	0	0	0	1
EABT16716	0	3	1	1	0	0	0
EABT16717	0	5	1	1	0	0	3
EABT16718	0	1	0	6	0	0	1
EABT16719	0	1	8	0	0	0	0
EABT1672	1	6	4	0	2	1	2
EABT16720	9	1	0	0	2	1	2
EABT16721	3	1	3	1	0	0	0
EABT16722	2	8	110	5	3	0	0
EABT16723	0	0	6	0	0	0	0
EABT16724	0	2	1	0	0	3	3
EABT16725	0	1	4	4	0	2	1
EABT16726	0	0	7	0	0	0	0
EABT16727	0	1	3	2	1	1	0
EABT16728	5	10	55	17	2	0	0
EABT16729	2	3	1	0	0	0	1
EABT1673	1	4	21	5	2	2	0
EABT16730	1	6	1	0	2	33	4
EABT16731	1	3	2	19	19	0	10
EABT16732	0	0	14	0	0	0	0
EABT16733	2	3	13	40	1	0	3
EABT16734	0	1	6	1	0	1	1
EABT16735	7	6	13	4	3	1	2
EABT16736	2	0	7	1	0	0	0
EABT16737	630.71	653.08	615.23	678.64	465.05	612.32	551.83
EABT16738	0	3	2	4	2	0	1
EABT16739	0	4	4	3	0	0	0
EABT1674	0	0	17	1	0	0	1
EABT16740	12	23	581.74	204.96	3	1	0
EABT16741	4	5	3	1	0	0	8
EABT16742	1	0	3	1.97	0	0	0
EABT16743	1	1	5	1	2	0	0

EABT16744	2	3	11	1	0	0	2
EABT16745	0	2	33	2	0	0	0
EABT16746	1	2	5	1	2	1	3
EABT16747	2	4	6	5	1	1	3
EABT16748	0	1	139.38	0	0	0	0
EABT16749	1	1	3	3	0	0	0
EABT1675	976.24	1607.96	1237	3482	1148.93	734.91	1016.01
EABT16750	17.42	20	39	36.13	21	6	28
EABT16751	2	2	52	5	0	0	0
EABT16752	1	5	12	3	1	0	2
EABT16753	0	1	17	5	1	0	2
EABT16754	4038.33	5463.33	6911.64	9175.77	4483.21	3721.9	3393.33
EABT16755	0	0	0	0	0	0	0
EABT16756	1	3	9	10	0	1	1
EABT16757	298.52	300	140	355.9	272	256	192.03
EABT16758	3	3	23	1	0	1	2
EABT16759	11	17	23	30	3	8	11
EABT1676	35	40	39	30	54	48	190
EABT16760	0	1	7	1	0	0	0
EABT16761	2	1	0	0	1	14	0
EABT16762	0	2	4	7	4	1	0
EABT16763	912	1568	1459.91	1324	1062	599	1044.98
EABT16764	24	82	60	220	51	22	35.12
EABT16765	2	9	11	18	3	3	1
EABT16766	0	1	6	1	0	0	0
EABT16767	0	0	2	5	0	0	0
EABT16768	1	3	15	6	1	0	2
EABT16769	56781.6	16673.1	4827.54	8730.25	11343.8	2404.9	2646.96
EABT1677	0	1	9	15	30	0	0
EABT16770	15	0	2	0	0	126	0
EABT16771	17	17	9	7	2	6	4
EABT16772	1	3	9	2	3	0	2
EABT16773	39.31	44	0	0	1	852.6	2
EABT16774	0	2	57	11	2	0	2
EABT16775	7	10	6	6	1	4	1
EABT16776	5	3	15	4.07	1	1	1
EABT16777	0	1	0	8	0	0	2
EABT16778	8	9	14	34	1	1	1
EABT16779	494.32	517.19	344.34	333.83	136.68	311.61	222.41
EABT1678	10991.1	23713.9	15069.4	27076.6	4986	4	288
EABT16780	7977	7208.19	1818	3501.02	17019	7568.58	6232.74
EABT16781	0	1	2	1	1	0	3
EABT16782	98	183	217.99	637.13	328.6	39	123
EABT16783	0	0	0	8	0	0	0
EABT16784	0	2	3	3	0	0	0
EABT16785	1	2	6	2	1	4	1
EABT16786	5631.6	22835	18769.4	3147.27	4231.17	7121.63	3234.24
EABT16787	1	3	30	0	0	0	0
EABT16788	439.43	566.53	339.8	582.96	458.22	447.09	335.6
EABT16789	0	2	6	3	0	0	0
EABT1679	1	0	4	1	0	1	0

EABT16790	4	1	18	2	1	3	0
EABT16791	0	1	9	7	12	0	0
EABT16792	1	1	9	1	0	1	0
EABT16793	5	1	15	1	1	0	4
EABT16794	4	5	1	7	4	0	2
EABT16795	10.04	13	33	8.27	4	43.99	3
EABT16796	1	2	5	10	1	1	2
EABT16797	4	4	6	24	47	14	0
EABT16798	3	10	13	2	2	1	1
EABT16799	4	4	10	2	0	1	1
EABT168	0	2	4	2	3	1	6
EABT1680	234	365	150	106	166	120	136.21
EABT16800	4	6	33.35	37	4	1	2
EABT16801	0	0	0	0	0	0	0
EABT16802	6	12	28	235.95	85	3	19
EABT16803	1	2	1	2	1	0	0
EABT16804	1	4	29	2	0	0	0
EABT16805	1	0	15	5	1	0	0
EABT16806	22	25	113	52	31	8	14
EABT16807	1641.3	2391.12	1820.96	5068.44	1190.46	1330	1121.64
EABT16808	0	0	0	18	0	0	0
EABT16809	0	1	9	0	0	0	1
EABT1681	2	0	9	0	0	0	0
EABT16810	0	2	2	0	1	0	0
EABT16811	1	0	21	0	2	0	0
EABT16812	4	9	6	13	0	1	1
EABT16813	3	2	13	4	0	3	1
EABT16814	727.2	1823.23	2910.11	1091.44	345.14	0	16
EABT16815	1	5	0	3	0	0	0
EABT16816	0	1	24	7	1	2	0
EABT16817	12	43	88.01	53	29	0	0
EABT16818	2	3	8	8	2	0	2
EABT16819	0	0	4	0	0	0	0
EABT1682	0	0	13	2	0	0	0
EABT16820	2	1	4	1	1	1	1
EABT16821	2	0	6	0	0	0	0
EABT16822	0	2	6	5	0	0	1
EABT16823	5886.77	5341.37	3075.57	2257.52	4706.06	1361.09	2747.77
EABT16824	2	7	2	17	2	0	2
EABT16825	6	9.03	0	0	0	8	5
EABT16826	13	31	146	69	4	18	2
EABT16827	0	0	3	0	0	0	0
EABT16828	2	2	8.59	4	0	4	4
EABT16829	0	1	6	0	0	0	0
EABT1683	0	2	3	1	0	0	0
EABT16830	0	6	1	1	0	0	0
EABT16831	0	2	1	0	1	24	10
EABT16832	174	480	661.07	918.01	17	40	3
EABT16833	0	5	6	7	3	3	0
EABT16834	4	6	21	1	1	0	0
EABT16835	125	133	102	45	136	2	18

EABT16836	0	1	16	2	0	1	0
EABT16837	2	2	0	0	0	1	2
EABT16838	0	0	1	4	2	0	0
EABT16839	2427.82	2500.08	656.93	2188.25	1842.06	3105.7	8861.25
EABT1684	5	1	4	4	3	1	1
EABT16840	22	29	40.96	22	8	29	24
EABT16841	8	3	15	5	0	0	0
EABT16842	3	4	53	4	3	0	1
EABT16843	4	8	5	1	3	2	0
EABT16844	0	1	4	0	0	0	0
EABT16845	43	102	87.7	142	1	107.96	33
EABT16846	4	4	28	8	1	7	7
EABT16847	165	308	1012	3253	144	13	45
EABT16848	1	5	1	9	3	0	0
EABT16849	1	2	4	5	0	1	0
EABT1685	0	1	1	0	0	0	0
EABT16850	16	28	81	75	68	5	16
EABT16851	13	12	4.15	1	0	41	28
EABT16852	0	1	3	1	0	1	0
EABT16853	33	2	0	1	4	1	4
EABT16854	4	10.98	150	2	3	17	2
EABT16855	0	1	6	3	1	0	0
EABT16856	0	0	0	3	1	0	0
EABT16857	36	5	5	0	0	44	0
EABT16858	0	0	5	1	0	0	0
EABT16859	0	1	0	0	0	7	0
EABT1686	16	36	48	108	24	14	16
EABT16860	0	0	6	2	2	1	1
EABT16861	0	2	0	0	1	6	2
EABT16862	0	0	2	2	0	2	1
EABT16863	4	4	5	1	0	0	0
EABT16864	4	5	106	3	1	5	1
EABT16865	9111.75	8670.67	3442	7599.07	24637.5	6942.02	6082.11
EABT16866	8	6	0	0	0	4	1
EABT16867	15	10	0	6	4	0	0
EABT16868	9884.97	9857.13	1051.97	6177.24	1886.31	5826.94	3417.59
EABT16869	94	119	282	965.67	514.01	313	1084.93
EABT1687	0	0	3	0	0	0	1
EABT16870	0	0	3	5	1	0	0
EABT16871	1	0	10	0	0	0	0
EABT16872	0	0	1	0	2	0	0
EABT16873	0	1	6	0	0	0	0
EABT16874	0	1	10	2	0	2	0
EABT16875	2	1	2	0	0	1	0
EABT16876	0	1	12	1	1	1	0
EABT16877	0	2	16	1	0	1	0
EABT16878	18	6	2	0	0	14	0
EABT16879	933.67	1375.05	989.8	1873.59	910.41	790.19	504.59
EABT1688	1	3	9	3	1	0	0
EABT16880	2	4	33	5	0	0	0
EABT16881	3	11	6	37	1	0	1

EABT16882	0	2	0	5	0	0	0
EABT16883	1783.62	1449.35	415	172	2990.98	5705.26	2830.16
EABT16884	0	0	0	1	0	0	2
EABT16885	0	0	6	1	0	0	1
EABT16886	4184.1	4859.77	3847.76	6748.69	3460.43	4504.07	3703.65
EABT16887	0	1	9	1	0	0	0
EABT16888	1	5	9	25	8	0	0
EABT16889	0	0	2	0	0	0	0
EABT1689	1	4	6	6	0	0	0
EABT16890	3	17	38	151	3287.17	2	71
EABT16891	1771.17	1990.31	1808.81	3105.93	1394.6	1513.6	1457.74
EABT16892	1	2	1	1	0	3.02	0
EABT16893	3	14	21	3	2	0	2
EABT16894	1	8	2	7	3	3	0
EABT16895	197	36	183	20	535.97	148.41	30
EABT16896	11	6	3	5	15	4	15
EABT16897	0	7	72	13	3	2	2
EABT16898	1	0	0	2	2	0	1
EABT16899	2	1	14	1	0	0	0
EABT169	50	62	17	78	24	9	6
EABT1690	0	2	14	6	2	0	0
EABT16900	311	414.95	177	844.01	481	209.91	220
EABT16901	0	2	4	36	8	0	10
EABT16902	2234.95	3167.26	3135.82	3750.31	1931.14	1928.52	1276.2
EABT16903	1	6	10	1	3	47.02	7
EABT16904	7	13	0	0	1	47	23
EABT16905	2	9	2	6.99	5	0	2
EABT16906	0	2	2	18	2	0	0
EABT16907	2	0	4	1	2	0	2
EABT16908	509	529.38	685	995.84	489.74	1	9
EABT16909	0	4	1	3	3	1	1
EABT1691	6	15	13	5	2	0	0
EABT16910	0	3	4	3	0	3	8
EABT16911	64	72	0	0	0	3	3
EABT16912	32702.2	19990.7	3579.68	10382.6	5713.3	7035.8	8531.57
EABT16913	851.43	1293.09	761.23	1549.52	561.16	499.25	590.05
EABT16914	2	17	2	3	5	1	4
EABT16915	1	1	9	4	0	0	1
EABT16916	3	14	1	5	1	1	2
EABT16917	1	7	9	5	1	2	3
EABT16918	0	0	7	6	0	0	0
EABT16919	1	0	4	4	2	0	1
EABT1692	2	2	17	1	0	3	0
EABT16920	0	0	0	0	0	15	2
EABT16921	1724.04	2294.89	978.07	3228.76	1018.85	388	657.17
EABT16922	1	3	11	2	0	0	0
EABT16923	2	1	3	1	0	0	0
EABT16924	0	0	9	2	3	0	0
EABT16925	9	6	6	11	1	1	5
EABT16926	0	0	2	0	0	1	0
EABT16927	10	11	1	0	0	0	0

EABT16928	83	220.62	324	506.97	707.98	8	84
EABT16929	3	8	1	8	3	1	6
EABT1693	3275.63	1994.98	2985.26	2552.26	581.61	3	29
EABT16930	0	3	5	10	3	3	7
EABT16931	160.39	115	34	16.01	6	2	0
EABT16932	0	0	0	0	0	0	0
EABT16933	2	0	9	0	0	2	0
EABT16934	573	901	2081.21	934.18	443	334	361
EABT16935	7	34	19	14	1	0	1
EABT16936	2	3	0	3	0	0	0
EABT16937	3	7	6	1	1	0	2
EABT16938	0	0	12	1	0	0	0
EABT16939	797.78	1088.75	1228.14	2195.02	1052.84	977.71	1110.17
EABT1694	1	5	12	3	3	0	0
EABT16940	1	1	2	0	1	0	1
EABT16941	0	1	4	1	0	1	0
EABT16942	1943.75	3764.45	4893.64	10724.9	2002.95	1882.22	1758.12
EABT16943	2	1	54	18	1	2	0
EABT16944	4	2	10	4	2	6	0
EABT16945	0	2	5	0	0	1	0
EABT16946	0	2	15	0	0	0	0
EABT16947	2	1	0	1	3	2	13
EABT16948	9	10	12	4	3	1	1
EABT16949	5133.73	5742.84	6560.88	5982.75	2462.74	3385.92	3667.6
EABT1695	0	0	6	2	0	0	0
EABT16950	0	0	0	0	0	0	18
EABT16951	1	2	4	3	0	0	0
EABT16952	1438.78	2172.02	1816.1	4452.2	1103.44	1615.54	967.03
EABT16953	3	0	15	0	0	0	0
EABT16954	1	0	10	0	1	2	0
EABT16955	0	1	10	1	1	0	0
EABT16956	1	1	2	3	0	2	0
EABT16957	0	0	5	0	0	0	0
EABT16958	0	0	1	3	0	0	0
EABT16959	0	0	1	4	0	0	0
EABT1696	1010.09	1838.15	1253.87	2420.04	1040.52	878.29	863.98
EABT16960	1	2	87	12	55	0	7
EABT16961	4	18	13	13	13	5	4
EABT16962	481	73	19.05	1	51	868.5	312.95
EABT16963	0	5	3	4	0	9	3
EABT16964	16623.6	14684.6	8654	6559.47	16850	10965.1	10131.1
EABT16965	0	0	0	0	0	15	2
EABT16966	36	100	133	215	16.01	43	30
EABT16967	0	0	1	0	0	0	0
EABT16968	0	0	5	0	0	0	0
EABT16969	1082	1710.07	1798.98	3789.26	950.12	749.03	547
EABT1697	2168.13	2717.41	1531.69	2910.87	1905.03	2227	2050.16
EABT16970	64	120.19	490	121	119	18	42
EABT16971	0	1	2	2	3	0	0
EABT16972	25	5	18	19	2	7	1
EABT16973	1	3	6	2	1	0	0

EABT16974	0	0	3	1	1	0	0
EABT16975	0	0	2	6	1	0	2
EABT16976	0	0	14	0	0	0	0
EABT16977	5065.91	7365.87	6810.3	11718.3	4599.91	4743.42	3866.01
EABT16978	7	17	26	24	3	10	5
EABT16979	632.77	244.99	0	0	0	424	0
EABT1698	0	0	31	6	12	2	20
EABT16980	0	0	3	0	1	0	1
EABT16981	6	4	18	3.05	1	15	0
EABT16982	17407.7	44415.2	33480.5	20564.3	12948.5	12	275
EABT16983	4	15	90.01	4	4	3	1
EABT16984	0	2	5	6	3	0	3
EABT16985	0	2	8	4	1	0	2
EABT16986	9	12	83	21	12	9	19
EABT16987	22	36	13	23	5	5.04	5
EABT16988	118	446.91	469	533	87.11	13	5
EABT16989	1	2	20	9	0	0	2
EABT1699	0	1	9	1	0	0	1
EABT16990	1131.41	1783.93	2026.07	3244.15	1026.92	792.13	808.42
EABT16991	2	6	0	0	0	5	5
EABT16992	5	2	6	1	1	1	0
EABT16993	17	55	78	416.78	415	2	7
EABT16994	15	18	18	13	23	1	31
EABT16995	144.64	223	400.77	824.01	333.97	85	162
EABT16996	0	2	57	0	0	0	0
EABT16997	5	3	0	0	0	5	1
EABT16998	1	20	3	0	3	0	4
EABT16999	5	7	71	5	6	1	3
EABT17	9	0	1	3	0	0	0
EABT170	0	1	8	0	1	0	1
EABT1700	3	3	3.99	2	0	0	1
EABT17000	5	3	0	0	0	0	0
EABT17001	0	0	1	1	1	0	4
EABT17002	3	0	1	16	2	0	6
EABT17003	13	8	0	0	0	29	0
EABT17004	0	4	0	1	1	0	2
EABT17005	9	0	1	0	2	3	18
EABT17006	0	1	2	0	1	3	1
EABT17007	0	0	7	0	0	0	0
EABT17008	2	8	5	1	0	2	2
EABT17009	2	1	5	0	0	0	0
EABT1701	1	7	30	22	1	0	0
EABT17010	18	25	18	42	5	2	10
EABT17011	0	1	5	1	0	0	0
EABT17012	6	24	199	9	0	4	10
EABT17013	8	7	3	2	5	1	6
EABT17014	4	0	4	1	3	0	0
EABT17015	1	6	10	56	15	0	0
EABT17016	0	3	0	7	3	3	1
EABT17017	491.99	761.95	628.63	639.03	493	224	469.01
EABT17018	90	94	62	79	66.01	139	274.04

EABT17019	5	12	34	24	741.41	3	13
EABT1702	0	1	9	0	0	0	0
EABT17020	0	1	2	1	0	16	0
EABT17021	0	0	0	3	2	0	0
EABT17022	22	27	12	80	34	0	0
EABT17023	0	1	5	1	0	1	0
EABT17024	1	1	1	5	1	2	0
EABT17025	4	15	2	3	4	9	3
EABT17026	3	0	0	1	2	3	8
EABT17027	1	14	4	38	3	1	0
EABT17028	0	1	2	3	0	1	0
EABT17029	6	20	61	875.28	86	0	1
EABT1703	0	4	1	4	0	2	0
EABT17030	0	0	0	4	1	0	0
EABT17031	0	0	7	0	0	0	0
EABT17032	880.44	767.3	106	233.19	299.05	179.99	389
EABT17033	1	0	6	0	1	0	0
EABT17034	14	18	135	2	6	5	8
EABT17035	0	2	0	2	0	0	0
EABT17036	0	0	2	4	0	0	0
EABT17037	563.6	841.54	570.12	1557.71	863.44	451	403.99
EABT17038	0	0	0	0	0	0	0
EABT17039	788.27	822.72	241.23	345.1	217	1107.9	764.05
EABT1704	15	15	85	11	2	11	8
EABT17040	9	9	16	10	8	3	3
EABT17041	1	2	30	2	2	1	1
EABT17042	606	741.78	619.89	1198	533	229	280
EABT17043	31	84	31	166	18	6	25
EABT17044	2	13	3	8	4	5	0
EABT17045	4	14	10	22	17	1	2
EABT17046	1	2	9	3	1	2	2
EABT17047	1	2	13	1	0	0	0
EABT17048	0	4	5	0	0	1	6
EABT17049	0	4	10	1	0	21	2
EABT1705	0	1	0	3	0	0	3
EABT17050	0	2	4	1	0	0	0
EABT17051	0	2	1	0	0	1	0
EABT17052	0	5	67	5	25	0	5
EABT17053	1	2	3	1	0	0	0
EABT17054	4	0	0	61	69.03	3	6
EABT17055	0	0	9	0	0	0	0
EABT17056	2	0	1	15	1	0	0
EABT17057	1	2	1	0	0	0	0
EABT17058	468	562.36	295.98	464	409	686	398
EABT17059	0	0	0	0	0	0	0
EABT1706	3	19	20	71	38.19	1	2
EABT17060	1	1	2	2	0	0	1
EABT17061	7	6	0	0	0	1	1
EABT17062	0	2	0	4	2	0	0
EABT17063	4	19	22	7	1	4	17
EABT17064	0	0	1	0	0	0	0

EABT17065	1	7	1	2	0	0	0
EABT17066	0	0	5	0	0	0	0
EABT17067	1	1	5	3	1	2	1
EABT17068	0	0	3	1	1	0	0
EABT17069	1	3	2	0	0	0	0
EABT1707	24926	27284	11825	9962	0	0	0
EABT17070	1	3	18	5	12	0	4
EABT17071	0	0	15	0	0	0	0
EABT17072	0	1	7	0	2	0	2
EABT17073	75.98	47	36	60	45	34	29
EABT17074	0	0	4	0	2	3	4
EABT17075	10	17	0	0	0	0	6
EABT17076	0	0	7	2	0	0	0
EABT17077	0	0	0	1	0	0	0
EABT17078	0	1	3	0	1	2	2
EABT17079	20	5	3	4	8	0	1
EABT1708	3	0	4	3	0	0	0
EABT17080	18	0	1	0	0	5	0
EABT17081	2	4	0	0	1	0	2
EABT17082	0	0	15	0	0	0	0
EABT17083	6	15	38	36	4	10	2
EABT17084	0	1	7	1	0	0	0
EABT17085	25	55	504	166	5	5	9
EABT17086	0	5	2	5	1	0	1
EABT17087	53	126.74	88	110	60	24	54
EABT17088	6	7	4	34	16	4	4
EABT17089	103	85	39	43	89	298	474
EABT1709	5	8	32	25	10	5	7
EABT17090	48	87	90	13	16	17	30
EABT17091	4	4	16	4	2	0	1
EABT17092	19	41	18	32	60	3	25.54
EABT17093	3	15	10	52	2	4	3
EABT17094	1	1	0	0	0	1	0
EABT17095	0	5	21	11	12	6	4
EABT17096	6	0	17	2	0	3	1
EABT17097	145	384	199	1532.61	155	19	55
EABT17098	0	0	16	0	0	0	0
EABT17099	0	0	19	0	0	0	0
EABT171	404.43	476.82	397	418	316	742	866.91
EABT1710	11	12	24	14	2	8	10
EABT17100	21	11	5	6	2	6	10
EABT17101	1	4	15	0	0	0	1
EABT17102	45	44	108	93	16	7	18
EABT17103	884.08	1101.6	2459	1479.02	651	349	547.98
EABT17104	1775.1	2414.08	2238.49	3872.42	1579.57	1377.88	1165.55
EABT17105	1	0	5	0	0	1	0
EABT17106	1	1	4	4	0	0	0
EABT17107	0	4	4	8	1	0	0
EABT17108	1	4	2	0	0	0	0
EABT17109	0	0	0	16	1	0	0
EABT1711	0	1	1	0	0	1	0

EABT17110	9615.86	12005.6	4124.04	7429.31	4443	374	1454
EABT17111	1	0	3	8	0	0	0
EABT17112	0	0	0	0	2	0	0
EABT17113	0	0	4	0	0	0	2
EABT17114	0	3	1	1	0	0	0
EABT17115	23	46	119.08	46.76	16.46	23	49
EABT17116	117.13	72.02	39	156	331	141	38.17
EABT17117	2	10.98	11	11	4	1	7
EABT17118	6	4	0	0	0	0	0
EABT17119	0	3	8	1	0	0	0
EABT1712	9	8	0	0	1	27	11
EABT17120	0	0	7	1	0	0	0
EABT17121	1	0	17	1	0	1	0
EABT17122	0	1	9	0	0	0	0
EABT17123	2	0	2	0	1	4	0
EABT17124	0	2	4	0	4	1	2
EABT17125	0	0	4	2.98	2	1	0
EABT17126	4	8	45	6	3	5	2
EABT17127	2	1	0	1	2	0	1
EABT17128	140.8	204.33	231.38	374.78	186.13	163.44	162.06
EABT17129	0	2	19	1	1	0	0
EABT1713	1	1	5	51	5070.06	4	11
EABT17130	773.01	1315.39	2023.83	1326.2	916.87	336	594.91
EABT17131	1124.72	1809.87	3565.85	2630.17	1602.65	1464.31	1099.43
EABT17132	1	5	44	5	1	0	3
EABT17133	2	0	3	0	0	4	0
EABT17134	2	1	28	1	1	1	0
EABT17135	0	0	0	0	0	0	0
EABT17136	0	1	3	1	2	0	1
EABT17137	1	5	2	3	1	1	2
EABT17138	3	18	5	0	3	14	10
EABT17139	326.83	554	225.2	711.56	156.01	94	103.03
EABT1714	0	2	1	6	2	0	1
EABT17140	893.81	1330.05	2026.34	2150.88	1313.22	924.72	1260.6
EABT17141	1	0	55	1	1	0	1
EABT17142	0	0	13	0	0	0	0
EABT17143	2	0	8	0	0	1	0
EABT17144	6	23	8	24	4	3	3
EABT17145	2	2	1	1	2	0	0
EABT17146	1896.05	2336.74	2089.74	1444.04	2221.11	2197.75	1430.87
EABT17147	14699.8	28853	9713.32	33578.1	5988.33	544	1362.05
EABT17148	0	7	7	0	5	34	24
EABT17149	19198.4	23899.3	36634.3	41234.6	13791.7	16246.6	11156.5
EABT1715	0	2	1	10	2	0	2
EABT17150	0	0	1	0	0	2	3
EABT17151	142.52	94	1	0	16	0	1
EABT17152	22	36	32	10	31	99.02	69
EABT17153	1	2	123	2	0	0	0
EABT17154	1	6	12	59	12	1	0
EABT17155	0	1	3	0	0	0	0
EABT17156	548.35	901.04	183	15	501	70	494

EABT17157	1	0	1	1	0	1	18
EABT17158	6957.65	6561.28	4817.2	6322.83	4774.2	7634.96	5013.84
EABT17159	6	13	57	50	4	1	7
EABT1716	857.75	1061.88	957.3	2450.73	1124.16	806.67	863.52
EABT17160	5	1	0	0	0	0	3
EABT17161	234	185	13	32	5	11	9
EABT17162	3	6	3	3	3	0	0
EABT17163	0	0	0	7	0	0	0
EABT17164	0	1	3	0	0	0	0
EABT17165	1	0	3	0	0	1	0
EABT17166	32	59	70.04	6	4	26	6.94
EABT17167	2552.87	2571.48	848.03	1281.65	1814.19	1230.4	1644.18
EABT17168	1	3	12	0	0	0	1
EABT17169	28	102	250.07	201.04	456.5	56	133.02
EABT1717	2061.86	3185.07	2082.59	3079	1516.05	1641.04	1619
EABT17170	0	2	5	1	1	0	0
EABT17171	2	1	1	0	1	15	16
EABT17172	1	1	2	1	2	1	1
EABT17173	1	0	2	0	0	0	0
EABT17174	0	0	4	2	0	0	1
EABT17175	13	14	16	3	38	2	6
EABT17176	192.24	309.93	224.19	558.28	249.85	177.9	260.45
EABT17177	1	0	3	0	0	1	0
EABT17178	0	0	6	0	0	0	0
EABT17179	0	0	2	1	0	3	0
EABT1718	2	1	19	7.15	11	3	0
EABT17180	784.01	1854.35	4675.69	4392.44	2827.61	2538.9	4729.77
EABT17181	901.06	1325.32	1458.38	1843.64	1066.9	1336.93	1019.98
EABT17182	0	2	7	3	2	0	0
EABT17183	0	4	10	0	0	0	0
EABT17184	1	4	0	1	1	0	1
EABT17185	0	1	30	2	0	0	1
EABT17186	2	4	5	2	1	1	0
EABT17187	19	12	2	1	1	0	3
EABT17188	173	746	1696	10371.6	852	49	83
EABT17189	2	13	14	6	7	8	5
EABT1719	0	0	8	1	0	0	0
EABT17190	5671.04	156	21	23	1016.41	3208.91	6975.59
EABT17191	9	10	15	2	5	2	0
EABT17192	0	0	13	0	0	0	0
EABT17193	0	1	4	0	0	0	0
EABT17194	0	6	1	5	0	0	0
EABT17195	4.03	10	27	15	4	11	8
EABT17196	2	0	1	1	1	0	0
EABT17197	1	1	1	0	0	0	3
EABT17198	0	0	5	0	1	1	1
EABT17199	1	0	6	4	0	0	0
EABT172	2	6	49	8	7	1	29
EABT1720	7	12	5	33	5	4	8
EABT17200	4	9	1	1	1	14	0
EABT17201	599.87	772	562	828.01	800	298	468.98

EABT17202	1133	1413.06	2796.62	6193.86	1365.89	1086.04	591.67
EABT17203	1	1	0	0	0	1	0
EABT17204	0	1	1	1	0	0	1
EABT17205	7	29	4	0	0	8	2
EABT17206	13	43	70	77	8	11	7
EABT17207	0	0	5	0	0	0	0
EABT17208	663.01	882	886	1812.78	1212	737	786
EABT17209	0	0	10	2	0	1	0
EABT1721	77	205	76	365	235	2	19
EABT17210	0	1	2	4	0	0	0
EABT17211	0	2	0	0	0	0	0
EABT17212	2597.64	16324.2	525.38	139.45	916.72	83.01	2324.29
EABT17213	0	1	1	3	0	0	0
EABT17214	3342.42	4629.18	3405.51	5149.07	3287.08	3398.32	3581.88
EABT17215	0	0	6	0	0	0	0
EABT17216	13	5	1	2	228.89	16	6304.54
EABT17217	0	3	8	18	2	0	0
EABT17218	534.63	736	909.98	2560.19	474.48	672.23	574.03
EABT17219	2	11	32	11	3	3	3
EABT1722	3	0	10	15.99	4	0	1
EABT17220	136	207	192	427.99	302.97	118	144
EABT17221	0	12	1	0	3	0	0
EABT17222	4319.84	31182.1	14766.8	26790.9	14159.1	6945.54	4021.41
EABT17223	0	0	14	1	1	0	0
EABT17224	0	0	1	2	0	1	0
EABT17225	1	1	21	3	1	1	0
EABT17226	1008.99	1164.99	914.99	1007	726	854	1909
EABT17227	0	4	14	5	1	0	1
EABT17228	0	0	11	1	0	0	1
EABT17229	2096.55	4001.8	11620.1	12588.9	2407.94	275.14	698.33
EABT1723	6240.49	6627.02	4934.57	4955.44	6972.54	3665.04	3437.97
EABT17230	130	281.64	413	321	41	0	10
EABT17231	6	4	11	75	76.38	0	5
EABT17232	4	5	32	20	8	2	1
EABT17233	192	125.7	5	1	1	107	4
EABT17234	9	13	6	62	46	1	3
EABT17235	0	1	12	3	0	0	0
EABT17236	2	3	1	0	0	0	0
EABT17237	0	0	10	0	0	0	0
EABT17238	0	3	1	2	0	0	0
EABT17239	0	1	8	1	1	0	1
EABT1724	2	2	5	4	1	4	2
EABT17240	17	16.95	2	16	5	2	2
EABT17241	346	1025	929	1462	1295.3	60	168
EABT17242	7	12	0	0	0	8	5
EABT17243	2	6	36	3	1	6	1
EABT17244	6858.63	3207.66	555.21	369	2760.16	3846.35	9092.85
EABT17245	5	7	18	5	8	35	32.42
EABT17246	1	0	6	4	0	0	0
EABT17247	1	3	5	2	2	0	1
EABT17248	1	5	2	24	5	0	0

EABT17249	1	4	193	0	2	1	4
EABT1725	0	5	15	2	4	1	0
EABT17250	0	2	9	3	0	0	0
EABT17251	5446.76	7231.9	5395.38	7925.4	5558.29	6583.34	6989.92
EABT17252	868.01	1122.45	540.85	1884.34	791.73	575.84	516.91
EABT17253	0	1	4	1	0	0	0
EABT17254	3	1	19	5	25	1	35
EABT17255	0	1	22	0	0	0	0
EABT17256	1	1	1	1	0	1	0
EABT17257	1	1	70	10	1	0	1
EABT17258	1	2	7	1	0	0	0
EABT17259	1503	1521.07	2050.02	2252.21	1118.98	1628	1162.99
EABT1726	145.91	175.41	120.12	324.07	123	46.06	107.38
EABT17260	12	15	2	2	0	0	0
EABT17261	147	175	339.37	174	43	124	45
EABT17262	1	4	37	3	4	0	5
EABT17263	1	2	11	1	1	0	0
EABT17264	10	6	9	2	2	17	20
EABT17265	1	1	21	2	2	0	0
EABT17266	8	5	13	10	11	1	16
EABT17267	0	2	1	30	2	0	1
EABT17268	20	58	3	37	43	8	67
EABT17269	3	10	18	1	0	2	1
EABT1727	6	9	33	10	0	1	0
EABT17270	33	152	124	294	38	3	42.18
EABT17271	0	2	18	7	1	0	0
EABT17272	24	32	5.59	42	13	14	15
EABT17273	28	21	7	148	83	0	7
EABT17274	1	0	2	2	0	0	0
EABT17275	0	0	6	5	0	2	1
EABT17276	0	0	5	6	0	2	0
EABT17277	6	1	12	3	3	2	3
EABT17278	0	1	2	1	2	0	2
EABT17279	4	1	7	1	0	0	0
EABT1728	2	1	1	0	5	0	5
EABT17280	31973	33534.6	14875.3	16537.9	10796.6	6286.93	10897.3
EABT17281	16	19	0	0	0	26	31
EABT17282	2	3	5	4	6	0	1
EABT17283	4	14	13	6	2	1	1
EABT17284	4396	4145.45	3328.15	5113.7	2388.35	7286.06	2633.05
EABT17285	2	18	6	3.04	12	3	9
EABT17286	0	2	31	5	0	0	0
EABT17287	0	2	0	1	0	0	1
EABT17288	2	2	3	0	0	1	2
EABT17289	28	76	88	35	7	5	4
EABT1729	0	2	9.44	2	0	0	0
EABT17290	0	5	1	21	6	1	2
EABT17291	2	4	38	16	0	0	0
EABT17292	3	3	3	6	0	6	5
EABT17293	5	4	5	4	0	0	0
EABT17294	0	0	11	2	1	0	0

EABT17295	0	1	14	18	0	0	0
EABT17296	2383.79	3806.35	2819.87	5329.73	2844.62	1991.42	2188.35
EABT17297	2	3	21	5	3	1	5
EABT17298	2	4	16	0	9	0	0
EABT17299	0	0	0	0	14	7	13
EABT173	2	3	1	6	0	0	1
EABT1730	1	1	0	0	4	0	0
EABT17300	4	2	4	4	0	0	0
EABT17301	17	16	58.61	284	3	2	0
EABT17302	12	29	9	107	6	0	0
EABT17303	0	0	10	0	1	1	0
EABT17304	3	6	2	3	0	0	0
EABT17305	1	6	4	6	0	0	1
EABT17306	0	0	4	0	0	2	0
EABT17307	0	2	7	3	0	15	9
EABT17308	1	1	6	2	3	0	0
EABT17309	0	0	0	0	6	2	7
EABT1731	0	0	9	0	0	0	0
EABT17310	0	1	1	2	1	0	2
EABT17311	1681.73	3218.53	2268.85	1958.88	1268.01	397.64	1245.01
EABT17312	0	2	12	0	0	0	0
EABT17313	13	9	17	4	5	0	1
EABT17314	0	0	0	5	4	0	5
EABT17315	1	7	3	4	5	1	1
EABT17316	10	13	55	21	8	0	0
EABT17317	1103.38	1316.81	869.72	1311	758.96	497.18	664.42
EABT17318	1	0	3	19	1	0	0
EABT17319	84	144	464	811	418	73	47
EABT1732	7617.17	8496.99	1539.01	7340.04	1294	7571	798
EABT17320	0	1	46	0	0	0	0
EABT17321	3	7	5	3	0	1	2
EABT17322	598.37	919.93	2650.43	4560.85	969.97	572.02	618.97
EABT17323	1	1	2	1	1	0	3
EABT17324	8	5	18	11	6	3	4
EABT17325	0	1	11	0	1	0	0
EABT17326	154.01	315.99	369.36	353.92	286.56	24	433.01
EABT17327	7	22	6	17	4	1	17
EABT17328	2	5	0	0	0	0	0
EABT17329	0	1	3	1	0	0	0
EABT1733	0	3	3	2	3	1	0
EABT17330	1	0	1	56	0	1	1
EABT17331	0	0	8	0	0	0	0
EABT17332	124.86	245.01	70	12.03	11	2	4
EABT17333	2	3	11	0	6	1	1
EABT17334	13	33	22.29	8	2	0	1
EABT17335	49	67	20	10	48	7	39
EABT17336	0	0	2	8	0	0	1
EABT17337	2	2	11	3	0	1	1
EABT17338	1	1	4	8	1	0	0
EABT17339	6	13	2	0	0	39	5
EABT1734	1853.03	3306.54	4036.59	6539.94	4976.62	1759.58	2529.78

EABT17340	3606.05	4242.72	1881	4046.42	2038.98	2621	2563.62
EABT17341	1	1	2	1	0	1	0
EABT17342	0	1	1	0	2	0	0
EABT17343	0	2	0	2	1	0	0
EABT17344	0	5	6	3	0	0	2
EABT17345	2	5	0	3	0	3	0
EABT17346	2	6	112	22	1	1	1
EABT17347	2	5	37	19	11	1	3
EABT17348	2001	3810.92	4903.62	3221.72	2419.35	1544.04	1663.75
EABT17349	1	5	9	2	0	1	1
EABT1735	0	0	3	1	1	0	0
EABT17350	1	1	8	3	0	0	0
EABT17351	0	0	9	1	0	0	0
EABT17352	4	0	6	0	1	1	1
EABT17353	1	0	0	1	0	0	0
EABT17354	2	0	5	1	1	1	0
EABT17355	1	0	1	2	0	1	0
EABT17356	1	0	5	0	0	3	0
EABT17357	1618.45	1956.99	2708.32	1028.48	4125.28	6578.15	5895.96
EABT17358	1	4	2.94	6	3	2	0
EABT17359	2	5	5	1	0	3	1
EABT1736	4	7	0	0	0	27	16
EABT17360	252.11	551.77	107	510.18	323.08	99	369.99
EABT17361	0	6	23	16	6	0	5
EABT17362	179	247.99	77	314.8	200	97	89
EABT17363	0	5	4	10	0	0	3
EABT17364	2	8	8	9	0	2	1
EABT17365	0	2	14	0	0	0	1
EABT17366	5	3	23	7	0	3	2
EABT17367	70.41	187.48	304	616.06	99.98	58	107.34
EABT17368	3	6	6	29	1	0	3
EABT17369	29752.3	31414.4	17492.2	17271.8	62034.1	29657	25021.3
EABT1737	0	3	10	1	0	0	0
EABT17370	2	0	0	9	1	0	1
EABT17371	1	9	70	24	25	0	10
EABT17372	487.86	784.08	1173.13	1536.51	681.93	537.36	533.13
EABT17373	0	3	3	0	0	3	1
EABT17374	5	4	0	0	0	1	1
EABT17375	2	25.24	17.99	55	19	3	0
EABT17376	7	5	5	1	2	1	1
EABT17377	12	49	70	16	5	3	14.99
EABT17378	2	1	3	2	2	3	2
EABT17379	0	0	0	5	1	0	0
EABT1738	0	0	4	1	0	1	0
EABT17380	1	3	8	2	0	0	2
EABT17381	8	20	93	64	1	1	9
EABT17382	25	27	115	93	11	12	5
EABT17383	2	3	0	1	2	1	11
EABT17384	2	0	2	3	1	0	1
EABT17385	1	14	20	30	25	0	0
EABT17386	1148	1503.78	1315	1642	920	926	789

EABT17387	2211.72	3021.48	929.92	1055	1449.13	2418.48	2436.06
EABT17388	0	4	3	3	6	1	6
EABT17389	0	3	1	1	1	0	0
EABT1739	0	0	0	0	8	0	0
EABT17390	1	1	24	3	2	2	1
EABT17391	376	428.94	2043.64	1422.02	245	25.98	85
EABT17392	2	38	36	97	180	3	151
EABT17393	121	227.4	340	471	280	164.52	224
EABT17394	0	1	0	0	1	0	6.85
EABT17395	1	0	6	3	2	0	1
EABT17396	0	2	12	2	1	0	0
EABT17397	1302.2	1672.83	2498.57	4243.61	986.08	1455.31	1292.35
EABT17398	190.97	305.95	184.77	158.02	142.15	26.21	106.78
EABT17399	1	0	6	1	0	0	0
EABT174	2	5	30	3	2	1	2
EABT1740	20900.3	30830.6	16488.1	3198.03	9984.75	37614.7	35615
EABT17400	0	2	4	0	0	0	0
EABT17401	2	2	2	3	1	0	0
EABT17402	0	2	5	0	0	0	0
EABT17403	0	0	9	0	1	0	0
EABT17404	5069.01	7808.52	6343.25	6101.34	4172.73	4557.64	4023.38
EABT17405	0	3	15	0	0	0	0
EABT17406	0	1	10	1	0	0	0
EABT17407	3	6	42	20	5	8	2
EABT17408	0	1	0	2	0	2	0
EABT17409	3	1	1	0	0	4	0
EABT1741	2	0	2	1	0	0	0
EABT17410	742	1248.29	1059.14	1137.99	863.08	766.01	836
EABT17411	1	0	3	3	0	0	0
EABT17412	10	11	6	27	19	6	1138
EABT17413	3	16	30	12	0	3	3
EABT17414	0	0	8.03	2	0	1	0
EABT17415	2	71.19	0	8	42	0	1
EABT17416	1	3	37	9	3	1	1
EABT17417	0	4	8	0	3	0	0
EABT17418	2794.93	5263.74	6271.87	9758.7	2830.06	3580.3	3399.55
EABT17419	2045	812.98	58.06	3	2	3	10
EABT1742	2.21	7	7	0	0	0	0
EABT17420	3	1	3	2	0	0	0
EABT17421	0	0	1	15	0	0	0
EABT17422	0	1	2	4	1	0	1
EABT17423	10	1	1	1	0	56	1
EABT17424	0	14	67	0	0	0	1
EABT17425	2953.1	6172.84	7292.92	3756.89	2801.54	861.93	1048.61
EABT17426	1008.59	3057.25	1644.5	2179.16	2616.7	557	792.04
EABT17427	2333.85	2226.06	1331.04	2178.86	3251.28	1787.76	1235.19
EABT17428	2	0	3	0	0	0	0
EABT17429	15	28	27	16	2	7	2
EABT1743	0	2	26	0	0	0	0
EABT17430	818.89	1523.99	1584	2094.5	1013	434	548.29
EABT17431	2	1	2	0	1	0	0

EABT17432	44	94	69.13	74.76	65	58	20
EABT17433	1	6	4	8	0	1	5
EABT17434	17	47	92	128	79	9	22
EABT17435	0	0	5	0	0	1	0
EABT17436	3	1	11	0	0	1	0
EABT17437	0	0	20	2	0	0	6
EABT17438	0	4	27	1	0	0	4
EABT17439	2	1	1	4	2	0	0
EABT1744	0	0	2	1	0	3	2
EABT17440	0	0	8	1	0	0	0
EABT17441	0	1	2	4	1	0	0
EABT17442	8	16	34	46	6	25	39
EABT17443	0	2	4	3	0	2	1
EABT17444	0	0	18	0	0	0	0
EABT17445	0	0	31	1	0	0	2
EABT17446	2363.3	4328.5	3025.01	3189.99	1742.84	2575.82	1769.33
EABT17447	255	1331.55	4763.03	776	795.12	1	6
EABT17448	3	0	7	7	0	0	0
EABT17449	0	2	6	0	0	3	1
EABT1745	4	3	1	51	0	0	1
EABT17450	6873.13	9033.67	4329.95	4378	2833.36	6497.94	3051.24
EABT17451	629.66	1063.05	706.98	1466.11	557.12	500	731.21
EABT17452	0	1	0	10	1	0	0
EABT17453	30	77	360.7	131.03	33	61	30
EABT17454	0	5	6	0	1	0	1
EABT17455	2	4	2	4	4	3	1
EABT17456	0	0	9	2	0	0	0
EABT17457	2	2	1	11	4	1	0
EABT17458	0	2	9	1	0	0	1
EABT17459	7	4	1	0	14	73.89	141.48
EABT1746	2	13.46	4	2	1	3	2
EABT17460	3	2	2	0	0	0	0
EABT17461	0	1	3	0	0	0	0
EABT17462	3	8	29	8	4	0	1
EABT17463	1	7	18	6	1	3	2
EABT17464	2	1	0	2	2	2	0
EABT17465	164	107	11	41	4	6	12
EABT17466	1	3	3	1	2	0	7
EABT17467	2	2	2	0	10	6	41
EABT17468	405	1417.02	478	447	1085.01	234.13	381
EABT17469	9	8	10	1	0	2	1
EABT1747	1	2	10	3	0	0	2
EABT17470	0	0	11	0	0	0	1
EABT17471	0	6	8	1	0	0	1
EABT17472	1167.1	2313.17	1615.56	4683.64	1444	1051	1279.23
EABT17473	188	292	616.98	516.41	290	189.85	147.18
EABT17474	2	4	9	5	0	0	1
EABT17475	0	4	5	4	0	0	2
EABT17476	1	2	6	2	0	0	0
EABT17477	2457.38	2682.19	2024.01	1356.28	4629.85	980.23	1914.39
EABT17478	0	6	12	2	1	0	0

EABT17479	0	2	4	2	0	0	1
EABT1748	0	1.01	55.01	0	0	2	1
EABT17480	0	1	4	0	2	1	2
EABT17481	0	2	2	5	0	0	2
EABT17482	15	8	49	0	5	38	22
EABT17483	4726.68	8734.57	7262.85	12605.3	3473.59	3144.12	3569.07
EABT17484	1	1	57	10	1	0	3
EABT17485	445.02	494.99	188	542.78	245	84	201
EABT17486	320	183	44	7	50	161	126
EABT17487	0	1	7	8	8	1	4
EABT17488	401.41	633.13	583.13	1582.24	544.39	472.29	452.25
EABT17489	357.92	716	14	381	132	0	0
EABT1749	4070.57	5956.53	5550.73	14007.4	2687.07	2723.85	2406.95
EABT17490	26	17	1	0	0	0	0
EABT17491	457.01	773	153	554.86	105102	32	217.08
EABT17492	0	1	7	0	1	0	0
EABT17493	0	0	9	0	0	1	0
EABT17494	1	0	2	0	0	0	1
EABT17495	8	11	28	6	2	15	4
EABT17496	0	1	8	2	0	1	0
EABT17497	2433.32	4998.94	5681.31	5244.61	4123.61	2941.12	4867.59
EABT17498	1	3	12	2	1	1	0
EABT17499	0	2	2	0	0	1	0
EABT175	1	3	14	3	0	0	2
EABT1750	8836.29	24838.4	44078.2	3374.93	10872.5	785.62	4420.94
EABT17500	1	1	8	2	0	3	1
EABT17501	25111.7	6171.56	1249.9	2958	8584.76	12971.3	2718.12
EABT17502	5	17	96	14	7	5	8
EABT17503	0	3	1	0	0	0	1
EABT17504	29	6	19	80	87293	220	701.44
EABT17505	0	0	1	7	0	0	0
EABT17506	5	17	11	50	6	19	3
EABT17507	1	1	15	5	3	0	0
EABT17508	0	1	11	2	0	0	0
EABT17509	3	6	25	6	3	3	1
EABT1751	0	3	0	0	49.99	0	0
EABT17510	27389.2	16994.9	10	1	27	2246.07	0
EABT17511	0	4	8	2	0	0	1
EABT17512	0	0	11	0	0	0	0
EABT17513	17	26	68	84	11	12	14
EABT17514	0	0	3	0	0	1	1
EABT17515	9	49	104.34	42.76	14	19	22
EABT17516	6637.26	5550	2806.11	2221.11	16188.3	4900.65	3775.42
EABT17517	8118.63	10749	1833.97	3663.4	2247.02	6722.19	15924.7
EABT17518	17	25	358.98	50	4	25	11
EABT17519	277	656	167	48	159.14	5	1
EABT1752	0	0	3	1	0	0	1
EABT17520	0	0	0	0	0	0	0
EABT17521	2	6	4	5	11	3	2
EABT17522	1	1	2	11	3	0	1
EABT17523	0	0	4	0	0	0	0

EABT17524	0	0	6	10	0	0	0
EABT17525	0	0	0	0	1	3	1
EABT17526	0	1	5	1	1	0	0
EABT17527	1	10	9	17	8	2	6
EABT17528	2	0	0	4	0	0	0
EABT17529	0	3	10	1	0	1	0
EABT1753	0	0	3	7	5	0	0
EABT17530	1441.57	2375.19	2430.67	2431.34	1563.13	1273.33	1719.9
EABT17531	1	2	5	2	1	0	0
EABT17532	5	15	21	17	5	16	7
EABT17533	1	1	5	1	2	0	1
EABT17534	0	2	4	0	0	0	0
EABT17535	1	0	3	0	0	0	0
EABT17536	1532.18	2649.63	2509.12	3181.55	1206.66	832.67	938.86
EABT17537	2	23.01	53	239.18	103.94	8	10
EABT17538	9	26	14	32	11	12	9
EABT17539	3	8	10	67	8	0	3
EABT1754	0	6	7	3	2	0	0
EABT17540	2	3	0	1	0	0	0
EABT17541	0	4	9	0	1	0	0
EABT17542	4	13	19	94	45	4	17
EABT17543	2	6.84	13	0	0	0	0
EABT17544	1	0	10	0	0	0	0
EABT17545	0	0	10	0	1	0	0
EABT17546	1	3	9	6	1	0	5
EABT17547	8	10	0	0	3	1	1
EABT17548	2	1	0	0	0	3	2
EABT17549	0	1	1	0	0	0	0
EABT1755	40	42	73	47	43	22	45
EABT17550	1	1	0	1	0	2	1
EABT17551	3	4	9	6	4	0	1
EABT17552	1	2	5	12	4	2	1
EABT17553	0	3	12	0	4	1	2
EABT17554	0	1	1	4	21	1	2
EABT17555	34	72	156.99	185	69	33.97	6
EABT17556	0	1	0	3	11	0	3
EABT17557	4	5	0	0	2	0	3
EABT17558	3	0	0	3	0	2	0
EABT17559	37.7	10	13	12	13	38	73
EABT1756	22.99	72	43.03	45.99	5	22	23
EABT17560	1	7	13	1	3	1	5
EABT17561	4	19	33	13	5	0	0
EABT17562	1579.02	3872.77	3936.19	7420.68	2621.46	1608.58	2354.67
EABT17563	27	14	2	0	0	0	0
EABT17564	1	1	0	0	0	0	1
EABT17565	0	2	3	0	0	0	0
EABT17566	0	0	4	7	1	0	1
EABT17567	2	5	5	3	2	2	4
EABT17568	0	0	9	0	1	1	1
EABT17569	11	19	24	5	1	5	4
EABT1757	3	4	27	5	2	2	1

EABT17570	4	12	151	7	6	13	5
EABT17571	2	1	3	1	2	9	2
EABT17572	0	1	5	1	1	4	1
EABT17573	1	8	3	5	1	1	4
EABT17574	1	0	3	2	0	0	0
EABT17575	1	7	7	7	2	3	1
EABT17576	4251.41	4066.21	3666.96	4149.43	2458.11	6048.73	5639.34
EABT17577	0	0	0	7	2	0	0
EABT17578	0	0	55	19	0	1	5
EABT17579	3	4	1	2	0	0	0
EABT1758	20298.4	19509.9	11194	25394.2	11824.8	20784.6	15373.4
EABT17580	0	10	0	0	3913.09	0	0
EABT17581	1220.61	1748.97	2740.21	2106.63	1513.36	1806.73	1609.61
EABT17582	0	0	1	0	0	0	0
EABT17583	0	1	1	2	0	0	0
EABT17584	0	0	0	0	1	0	3
EABT17585	92026.7	32379.5	19	4	40.5	47409.8	189.14
EABT17586	0	2	1	14	0	0	0
EABT17587	9	2	0	1	0	9	0
EABT17588	0	2	11	3	0	0	0
EABT17589	0	0	2	3	0	0	0
EABT1759	4	0	0	0	0	3	0
EABT17590	4	6	13	0	4	1	1
EABT17591	0	0	3	3	0	0	0
EABT17592	1	4	4	0	0	1	1
EABT17593	0	1	2	0	0	0	0
EABT17594	0	6	8	15	10	0	0
EABT17595	2	1	11	0	2	2	4
EABT17596	5	1	0	3	5	3	9
EABT17597	0	0	1	1	1	0	0
EABT17598	0	4	8	5	1	2	0
EABT17599	3	1	5	2	2	0	1
EABT176	1	2	3	2	0	0	0
EABT1760	1	2	1	0	2	5	2
EABT17600	1	2	3	15	0	0	1
EABT17601	0	2	7	0	1	0	1
EABT17602	0	0	2	0	2	0	2
EABT17603	0	4	3	4	0	0	1
EABT17604	6	17	9	27	27	0	17
EABT17605	13	3	22	0	0	13	10
EABT17606	0	0	34	1	0	0	0
EABT17607	0	0	1	3	0	0	0
EABT17608	14128.2	6901.2	4986.45	3255.42	11234	6673.3	3993.59
EABT17609	0	3	1	3	6	6	18
EABT1761	1	2	1	1	1	2	2
EABT17610	10	16	27	8	3	4	7
EABT17611	99209.6	3424.66	91	464	3755.66	10465.3	63387.3
EABT17612	0	1	4	1	0	0	1
EABT17613	1	8	31	35	8	9	7
EABT17614	1	2	1	3	4	0	0
EABT17615	2	3	33	2	3	0	0

EABT17616	3	6	4	1	0	2	2
EABT17617	7	46	50	40	14	18	25
EABT17618	722.48	1114.1	1388.79	1444.41	886.6	615.81	598.59
EABT17619	0	2	5	1	2	0	0
EABT1762	705.7	1044	888.51	3514.13	516	416	399.43
EABT17620	7	25	56	21	85	4	0
EABT17621	1	1	4	9	0	0	1
EABT17622	1	1	8	4	0	0	0
EABT17623	0	0	1	4	0	0	0
EABT17624	4.8	27.13	6	1	1	16	9
EABT17625	0	25	0	0	0	20	0
EABT17626	0	1	9	3	0	1	2
EABT17627	0	1	7	2	0	0	0
EABT17628	2	1	9	5	2	1	3
EABT17629	0	0	4	1	0	2	0
EABT1763	0	0	3	0	0	0	0
EABT17630	1	1	22	0	0	2	0
EABT17631	7	26	46	3	1	2	0
EABT17632	1	0	3	3	2	0	0
EABT17633	7	5	10	2	1	0	0
EABT17634	0	0	3	1	1	0	0
EABT17635	0	1	9	1	0	0	0
EABT17636	1	0	23	0	0	1	1
EABT17637	4.01	1	0	0	0	1	0
EABT17638	1	0	9	2	1	1	2
EABT17639	6	2	39	1	0	0	1
EABT1764	6	40	88	221	109	8.01	21
EABT17640	0	0	11	0	0	0	0
EABT17641	0	0	1	4	0	0	0
EABT17642	47	120	370	1204.59	7	2	13
EABT17643	187	272	138	458	238	111	100
EABT17644	2323.06	2520.04	988.98	1768.28	1682.71	1070.91	907.9
EABT17645	0	3	0	1	4	2	3
EABT17646	1490.55	1590.05	1095.02	1354.09	1293.82	1704.27	1302.77
EABT17647	19	7	32	33	5	2	2
EABT17648	0	0	0	1	0	0	0
EABT17649	0	1	0	8	0	0	0
EABT1765	12	25.01	40	27	1	14.97	17
EABT17650	3149.61	5941.48	4918.67	6666.6	3787.82	1964.88	3635.97
EABT17651	5	5	29	14	1	2	1
EABT17652	0	2	10	0	0	0	0
EABT17653	21	41	65	101	36	8	18
EABT17654	1	1	6	0	0	2	0
EABT17655	0	1	49.85	0	0	0	1
EABT17656	1	4	3	8	3	0	1
EABT17657	0	0	6	0	0	0	0
EABT17658	0	0	13	1	0	2	0
EABT17659	1909.93	2088.02	1866.8	3698.92	1147.54	2136.29	1632.5
EABT1766	7	1	6	2	3	0	0
EABT17660	1	2	20	7	1	0	0
EABT17661	37	2	80	11206.2	3	3	0

EABT17662	613.61	904.89	639	931	674	406	662
EABT17663	0	0	4	0	0	0	0
EABT17664	1493.86	1883.49	2390.52	3394.24	1343.46	1669.15	1367.17
EABT17665	2087.38	2533.38	1086.03	3260.22	2624.03	1556.02	1633.56
EABT17666	3	7	44	28	4	20	4
EABT17667	2889.7	4072.13	2797.03	25374	4422.13	1477.64	3297.7
EABT17668	0	0	1	0	0	0	0
EABT17669	0	0	11	0	0	0	0
EABT1767	0	3	8	0	0	0	0
EABT17670	6047.9	12952	10265.3	17172.4	7982.08	5245.99	6805.1
EABT17671	2	1	6	1	0	2	0
EABT17672	0	2	8	0	0	0	0
EABT17673	3	4	0	0	0	6	1
EABT17674	3506.78	2933.96	2793.3	3437.94	2744.82	13	207.63
EABT17675	1	3	1	12	3	0	0
EABT17676	23502.8	26876.7	2620.81	589.3	7636.59	9	297.01
EABT17677	1162.21	1363.18	446.17	1607.99	731.95	1208.16	1405.49
EABT17678	795.15	1247.67	1273.27	2662.59	1104.32	914.99	786.24
EABT17679	32	52.92	30	221	16	24	38
EABT1768	0	5	2	50	0	1	2
EABT17680	5	7	2	2	2	2	18
EABT17681	10	42	166	19985.7	34	2	3
EABT17682	347.24	532.05	669.44	1209.52	577	374	561
EABT17683	0	0	9	0	0	0	0
EABT17684	2	6	11	6	1	1	3
EABT17685	2	305	2	0	1	24	1
EABT17686	7	4	7	9	6	2	5
EABT17687	0	3	9	5	2	3	1
EABT17688	3	3	92	4	3	3	1
EABT17689	0	0	1	0	0	0	1
EABT1769	2	2	0	0	0	0	2
EABT17690	0	2	0	1	2	0	2
EABT17691	1	5	4	3	11	0	3
EABT17692	131.03	313	599.99	316.04	162	0	1
EABT17693	0	5	6	7	3	7	8
EABT17694	1	4	33	8	0	0	2
EABT17695	10	13	14	2	1	24	9
EABT17696	1	0	4	0	0	0	1
EABT17697	6	14	0	0	1	47	25
EABT17698	0	0	9	3	0	0	0
EABT17699	0	2	4	0	0	0	0
EABT177	2	5	0	0	1	1	8
EABT1770	1	3	2	0	0	0	2
EABT17700	0	0	5	0	5	1	2
EABT17701	0	0	0	0	0	0	0
EABT17702	0	0	5	1	0	0	0
EABT17703	0	0	3	2	0	0	0
EABT17704	33	63	55	5	66	12	8
EABT17705	2942.18	4763.08	3215.51	6785.87	3576.17	2192.61	2836.5
EABT17706	1	0	4	3	2	1	0
EABT17707	0	0	36	1	6	1	0

EABT17708	2	1	1	4	1	0	3
EABT17709	3	0	0	2	0	0	2
EABT1771	0	2	4	2269.97	26	0	1
EABT17710	1	1	9	8	0	0	0
EABT17711	0	0	1	0	1	1	0
EABT17712	1	1	0	3	0	0	0
EABT17713	2	8	0	6	1	0	1
EABT17714	5	11	4	4	3	2	4
EABT17715	1	2	8	1	1	0	0
EABT17716	22	26.98	81	88	32	1	9
EABT17717	11	1	3	0	1	1	6
EABT17718	0	0	0	0	0	1	0
EABT17719	81	197	17	6	37	0	9
EABT1772	0	2	4	2	0	0	0
EABT17720	0	2	16	0	0	1	0
EABT17721	13	24	16	26	3	10	5
EABT17722	1	3	2	7	0	0	0
EABT17723	0	0	5	0	0	1	0
EABT17724	11	3	2	1	7	44	58
EABT17725	1	2	3.12	5	2	2	3
EABT17726	1	3	0	0	0	0	2
EABT17727	6	12	38	89	1	3	4
EABT17728	6707.66	5186.48	1994.12	7088.36	1955.07	8827.18	4561.97
EABT17729	0	1	16	2	20	1	2
EABT1773	240	351.34	261.87	304.15	335.01	154.66	217.71
EABT17730	0	2	1	5	3	0	0
EABT17731	0	2	12	11	10	0	1
EABT17732	3	3	2	0	0	1	2
EABT17733	3	0	6	1	0	2	1
EABT17734	0	0	0	6	0	0	0
EABT17735	1	6	6	6	0	2	1
EABT17736	2	6	3	31	4	2	4
EABT17737	1	1	6	0	0	0	0
EABT17738	1	0	7	3	0	0	0
EABT17739	0	0	3	1	1	0	1
EABT1774	0	0	9	0	1	0	1
EABT17740	0	0	4	1	0	1	0
EABT17741	1	4	94	1	0	6	1
EABT17742	1	0	1	2	4	0	0
EABT17743	2	2	0	0	0	0	0
EABT17744	0	0	7	1	1	0	0
EABT17745	0	0	0	3	0	0	0
EABT17746	0	1	8	1	0	0	0
EABT17747	0	0	16	0	0	0	0
EABT17748	2	4	15	2	0	2	4
EABT17749	6	5	0	1	1	5	0
EABT1775	0	0	5	1	0	0	0
EABT17750	59	78.79	49	5	8.65	51.03	32
EABT17751	2	0	6	4	0	1	0
EABT17752	4	4	0	0	0	0	0
EABT17753	2	0	4	0	0	0	0

EABT17754	0	2	5	2	2	0	0
EABT17755	0	2	3	1	1	2	3
EABT17756	0	1	2	1	1	0	0
EABT17757	1872	1969.8	1759	3914.56	1158.91	1474.87	1143
EABT17758	0	2	14	3	0	0	0
EABT17759	490.99	1372.44	3025.23	2268.49	689.9	646.01	1040.02
EABT1776	0	1	7	1	0	0	0
EABT17760	12	13	3	10	2	9	0
EABT17761	30	35	10	5	1	0	0
EABT17762	29	3	0	0	0	2	0
EABT17763	1	8	16	0	1	4	1
EABT17764	275	967.12	2274.2	619	697	190	517.15
EABT17765	1315.78	2486	1777.48	5038.62	1439.99	1069.89	1294.01
EABT17766	4	6	6	9	4	5	6
EABT17767	8	35	740.96	25	1	9	13
EABT17768	83	203.99	362.26	673.91	380	87.69	90
EABT17769	0	0	6	0	0	0	0
EABT1777	0	0	0	0	1	0	6
EABT17770	3	9	22	17	1	2	8
EABT17771	10986.2	4716.6	90	802	5880.92	5	25.99
EABT17772	2	0	7	4	1	0	0
EABT17773	0	1	4	0	0	0	0
EABT17774	2	3	5	1	0	0	0
EABT17775	27	9	64	1.06	5	84	7
EABT17776	0	0	1	6	5	0	1
EABT17777	0	1	0	29	0	0	0
EABT17778	22	40	99	54.02	25	29	28
EABT17779	829.55	2148.3	5846.28	226	700.98	32	208
EABT1778	30	66	21.86	170.09	355.99	32	1324.87
EABT17780	0	0	6	2	0	2	0
EABT17781	0	0	1	2	0	0	0
EABT17782	2	22	1	0	0	0	0
EABT17783	2	2	39	0	1	2	1
EABT17784	0	4	5	13	11	2	1
EABT17785	8	11	21	8	0	0	1
EABT17786	0	1	1	2	0	0	0
EABT17787	6	14	21	48	11	5	5
EABT17788	0	1	1	3	3	4	0
EABT17789	0	0	2	2	0	0	0
EABT1779	688.98	991.04	1169.2	1303.04	1036.29	615.56	687.36
EABT17790	0	2	5	0	0	0	0
EABT17791	5951.87	4403.54	3524.87	7512.73	3377.44	6302.32	5421.92
EABT17792	1435	2590.02	3260.96	4728.98	1855.02	901	1084.3
EABT17793	2	4	8	44	9	1	12
EABT17794	0	0	6	0	0	0	0
EABT17795	0	0	3	17	9	0	0
EABT17796	0	1	4	0	0	0	0
EABT17797	2	0	7	0	0	5	0
EABT17798	1	1	20	0	0	1	0
EABT17799	1	1	32	0	0	0	0
EABT178	1	1	10	0	0	0	0

EABT1780	5	13	21	109	40	3	10
EABT17800	0	3	14	1	0	0	0
EABT17801	1	0	6	1	1	2	1
EABT17802	15	24	16	176	29	8	3
EABT17803	10	8	0	0	0	2	1
EABT17804	1415.84	2208.98	5176.77	4991.44	1357.34	1378	1022
EABT17805	0	0	0	0	1	0	0
EABT17806	0	0	5	1	0	0	0
EABT17807	5096.02	11852.4	5044.25	6108.42	9722.42	3537.01	5502.83
EABT17808	2	3	1	0	1	3	7
EABT17809	226.02	595.61	61	109	597.03	1955.54	2317.7
EABT1781	1	0	9	0	3	1	2
EABT17810	1	3	29	0	0	1	2
EABT17811	7	15	10	17	9	4	6
EABT17812	5	3	49.26	3	1	0	1
EABT17813	1	11	3	1	4	3	3
EABT17814	1	0	3	2	0	1	0
EABT17815	0	1	2	0	0	0	1
EABT17816	10	57	4	6	0	31	7
EABT17817	1	0	3	4	0	1	1
EABT17818	6	19	95.14	24	6	8	6
EABT17819	0	0	58	6	0	0	0
EABT1782	6	13	4	1	0	2	1
EABT17820	2	1	21	12	12	4	33
EABT17821	3	8	43	8	4	2	7
EABT17822	2	0	1	0	10	3	0
EABT17823	1085.93	1690	842.21	1425	1009.38	1182	1553.97
EABT17824	1	5	59	12	11	2	6
EABT17825	0	0	0	1	2	0	0
EABT17826	2	5	52.92	10	0	0	0
EABT17827	3	12	2	5	6	15	10.98
EABT17828	3254.32	3895.32	2726.76	3496.11	2486.13	2204.73	2605.82
EABT17829	505.04	584	232	648.75	380	328.68	356
EABT1783	10	40	12	0	2	7	10
EABT17830	15	26	1	1	3	13	8
EABT17831	2	4	1	10	2	0	6
EABT17832	507.35	771.99	969.77	1569.63	557.98	360	324
EABT17833	0	2	1	1	4	2	2
EABT17834	7	7	13	12	7	2	3
EABT17835	1003.28	1507.1	2007.16	2734.12	1308.92	1105.67	801.12
EABT17836	2	1	11	1	4	0	0
EABT17837	3	6	2	0	2	2.9	2
EABT17838	0	0	1	6	0	0	0
EABT17839	1	0	4	1	0	0	0
EABT1784	6	1	2	7	2	0	2
EABT17840	2	1	2	4	0	1	1
EABT17841	7	3	239	1	1	2	1
EABT17842	1	3	1	4	1	1	0
EABT17843	1	1	4	0	1	1	2
EABT17844	300	480	277	1063	297	210	186
EABT17845	0	2	0	0	0	0	1

EABT17846	4	1	15	13	0	2	1
EABT17847	1	7	11	3	1	0	0
EABT17848	0	0	13	0	0	1	1
EABT17849	0	0	24	1	0	0	0
EABT1785	0	2	3	1	0	0	0
EABT17850	0	0	1	2	0	0	0
EABT17851	0	0	0	0	0	0	0
EABT17852	592.11	887.08	390	954	907.04	626	962.01
EABT17853	2	6	142.84	5	0	0	0
EABT17854	2	0	4	2	1	0	0
EABT17855	0	0	8	3	0	0	1
EABT17856	0	1	7	5	0	1	0
EABT17857	0	4	7	6	3	2	0
EABT17858	4936.94	18508	8266	18921	5426.95	48	58
EABT17859	0	0	0	6	1	0	0
EABT1786	1	3	4	0	0	0	0
EABT17860	2	0	4	0	3	2	0
EABT17861	3	21	30	5	3	9	8
EABT17862	0	6	0	0	0	0	0
EABT17863	4	3	44	1	2	1	3
EABT17864	0	2	59	8	1	0	0
EABT17865	1	5	26.56	13	1	1	1
EABT17866	1	3	10	4	0	3	1
EABT17867	2	0	8	3	0	0	2
EABT17868	0	2	3	4.05	2	0	0
EABT17869	847.45	1131.82	823.92	2612.02	697	384.4	652.3
EABT1787	0	3	1	2	0	0	0
EABT17870	0	2	2	3	0	1	0
EABT17871	0	1	1	1	0	1	0
EABT17872	0	1	19	8	0	0	0
EABT17873	1	2	11	0	1	0	2
EABT17874	1	3	19	7	0	1	2
EABT17875	2	3	0	5	3	1	0
EABT17876	2	10	11	0	665.86	1	7
EABT17877	0	0	4	0	0	0	0
EABT17878	1	5	0	2	1	0	6
EABT17879	13	8	6	0	4	17	8
EABT1788	2379.11	3958.87	3374.65	6043.2	3261.1	3015.53	3076.95
EABT17880	63	199	1198.91	56	142.51	1	4
EABT17881	0	0	5	0	0	0	0
EABT17882	2	3	10	9	3	0	1
EABT17883	0	0	0	0	0	6	7
EABT17884	3	1	1	5	2	0	4
EABT17885	1	0	2	4	0	0	0
EABT17886	1	1	4	5	5	0	0
EABT17887	9	15	9	3	0	0	0
EABT17888	0	0	16	2	1	0	0
EABT17889	1	1	3	28	3	1	0
EABT1789	13	13	5	0	1	0	1
EABT17890	4	5	8	11	2	2	0
EABT17891	863.79	889	286	843.03	684	644.11	476

EABT17892	2	32	37	67	10	1	3
EABT17893	22416.5	5782.42	18699.9	138	16	36	0
EABT17894	0	2	4	0	1	0	0
EABT17895	781.3	5033.56	5134.37	1490.69	14971.2	238.97	1794.21
EABT17896	0	1	7	4	0	0	1
EABT17897	0	0	0	0	9	0	0
EABT17898	2	2	0	0	1	3	2
EABT17899	0	1	0	6	3	0	2
EABT179	4	4	41	1	1	5	0
EABT1790	0	0	7	0	0	1	0
EABT17900	80	90	86	174	199	18	53
EABT17901	0	1	14	13	3	0	0
EABT17902	129	157	64	90	136	111	87.76
EABT17903	5	19	94.09	12	18	11	16
EABT17904	1	7	25	8	0	4	0
EABT17905	0	0	6	0	0	0	0
EABT17906	0	0	1	1	0	3	3
EABT17907	12	20	0	0	0	3	3
EABT17908	6	8	16	31	3	1	0
EABT17909	26	35	2	4	10	12	21
EABT1791	18454.5	20751.8	13467.3	15827	16261.9	20294.3	19305.3
EABT17910	1	2	6	2	0	1	0
EABT17911	0	0	12	0	0	0	0
EABT17912	2	1	12	2	0	0	0
EABT17913	0	11	1	1	0	4	2
EABT17914	86	84	13	2	27	15	19
EABT17915	0	1	0	5	0	0	0
EABT17916	0	1	20	1	0	0	1
EABT17917	212.76	291	297	2259.88	464.35	117	659.71
EABT17918	0	0	2	8	1	0	0
EABT17919	2	2	25	0	0	1	2
EABT1792	0	0	1	1	1	0	1
EABT17920	0	2	0	4	0	0	0
EABT17921	266.32	324.59	446.03	60	163.28	394.7	691.06
EABT17922	4220.27	7708.94	4068.17	5826.79	2882.53	5801.43	6421.5
EABT17923	0	0	0	0	11	156.46	70.02
EABT17924	11	0	3	7	1	0	2
EABT17925	2	3	2	0	0	0	0
EABT17926	2	0	4	4	2	0	0
EABT17927	58	152.95	249	281.02	389.28	27	77.16
EABT17928	30	122	88	224.05	66	16	19
EABT17929	21	54	204.39	111.01	25	19	17
EABT1793	0	1	1	4	2	0	0
EABT17930	0	0	2	5	0	0	0
EABT17931	2192.78	2223.06	486	276	728	2375.79	2878.57
EABT17932	3335.06	7941.08	3382.79	1052.35	9323.62	324.03	2484.26
EABT17933	1	1	27	2	0	0	0
EABT17934	2	12	7	0	1	0	3
EABT17935	0	3	34	0	0	0	1
EABT17936	0	3	53	5	3	3	0
EABT17937	2	1	12	0	0	0	0

EABT17938	5	6	17	2	0	0	2
EABT17939	0	1	1	5	0	0	1
EABT1794	0	5	0	2	4	0	0
EABT17940	2	0	5	2	1	2	2
EABT17941	0	1	8	1	0	1	1
EABT17942	1	0	3	3	1	0	2
EABT17943	4	7	27	0	4	0	5
EABT17944	1	3	46	1	1	1	0
EABT17945	7	23	43	19	15	7	14
EABT17946	0	7.87	4	31	4	2	1
EABT17947	1	0	2	1	2	0	1
EABT17948	0	4	0	0	0	19	0
EABT17949	855.87	1270.99	1924.12	3117.08	1105.05	699.47	1580
EABT1795	14	35	10.95	20	7	4	3
EABT17950	0	0	6	0	0	0	0
EABT17951	9	5	7	0	0	1	0
EABT17952	0	2	7	0	0	0	0
EABT17953	11	9	36	10	2	4	7
EABT17954	1	2	9	1	0	1	1
EABT17955	11	3	9	2	3	48	1
EABT17956	2907.1	3491.1	4221.08	4669.99	2572.13	3096	2472.19
EABT17957	0	5	3	0	0	0	0
EABT17958	0	0	4	0	0	0	0
EABT17959	1	2	0	0	0	19	13
EABT1796	0	1	17	0	0	0	0
EABT17960	1	7	29	16	0	0	0
EABT17961	4	3	123	7	6	2	8
EABT17962	10	4	11	2	1	2	0
EABT17963	0	0	29	1	0	0	0
EABT17964	21	4	3	0	2	0	1
EABT17965	0	0	2	6	0	0	0
EABT17966	11	55	299.31	1397	610.24	2	29
EABT17967	2	3	0	0	0	2	0
EABT17968	4002.67	3087.98	14005.8	122915	1971.9	53	125.89
EABT17969	2	2	50	4	1	2	1
EABT1797	2	3	5	0	0	0	0
EABT17970	0	1	6	0	0	0	0
EABT17971	4	0	0	6	1	2	1
EABT17972	7155.91	1781.98	141	1135.16	219.02	1064	286
EABT17973	424	735.12	744.18	1417.08	355	418.11	223
EABT17974	0	0	0	0	0	0	0
EABT17975	0	1	3	7	0	1	1
EABT17976	0	0	5	3	0	7	5
EABT17977	2	4	2	1	1	0	0
EABT17978	0	0	1	1	0	0	0
EABT17979	0	0	0	0	0	15	0
EABT1798	2	3	2	1	5	3	4
EABT17980	0	1	85	0	0	1	0
EABT17981	1	2	27	1	0	0	0
EABT17982	0	1	8	1	0	0	0
EABT17983	2	0	2	1	0	0	0

EABT17984	1	0	2	0	0	6	2
EABT17985	19	30.01	0	0	9	3	80.03
EABT17986	32	60	31	12	1	6	4
EABT17987	0	1	16	0	0	0	0
EABT17988	90	373.16	295	180	267.89	54	71
EABT17989	3	7	0	0	0	6	0
EABT1799	2	3	20	1	1	0	0
EABT17990	0	1	0	3	0	0	0
EABT17991	0	0	5	0	1	0	1
EABT17992	1	145	47	7	0	2	2
EABT17993	21	34	20	80	31.67	6	18
EABT17994	0	2	6	15	0	0	0
EABT17995	0	0	7	1	4	0	0
EABT17996	30	54	72	346.18	137.37	3	33.12
EABT17997	0	0	1	12	0	0	0
EABT17998	0	0	4	2	0	0	0
EABT17999	2	4	17	7	2.91	3	7
EABT18	0	1	5	4	1	0	0
EABT180	15	37	30	271.76	16	2	12
EABT1800	39	63.18	85	22	96.05	26	61
EABT18000	3	8	114	16	5	0	0
EABT18001	14.47	6.95	1	0	8.56	26	92.63
EABT18002	1	2	10	0	0	0	0
EABT18003	237.06	334.13	477.41	564.82	476.67	454.28	374.86
EABT18004	1	0	3	1	0	0	0
EABT18005	0	0	0	0	0	0	0
EABT18006	9	30	15	39	21	11	50
EABT18007	1059.81	1968.69	3393.55	11142.7	968.05	983.83	554.04
EABT18008	813.57	951.03	467.93	1336.03	698.03	814.95	648.94
EABT18009	467	161	4	1	1345	47	4672.59
EABT1801	3	7	10	0	0	0	0
EABT18010	0	3	8	10	2	0	1
EABT18011	0	4	15	2	0	1	0
EABT18012	2	4	13	2	0	1	3
EABT18013	0	0	2	3	2	0	0
EABT18014	44	100	141	569.46	75	264	208
EABT18015	0	2	1	2	0	0	1
EABT18016	3663.23	4035.83	4543.65	5245.02	2574.99	3634.05	3145.13
EABT18017	23.01	36	33	187.98	68	11	38
EABT18018	3	1	18	21	0	0	0
EABT18019	0	1	4	0	0	0	1
EABT1802	0	0	6	0	0	0	0
EABT18020	1	0	8	6	1	1	2
EABT18021	0	1	1	0	0	0	0
EABT18022	3	4	2	0	0	0	0
EABT18023	351	498	125	466	343	129	196
EABT18024	1	4	2	5	4	1	6
EABT18025	0	0	2	1	0	0	0
EABT18026	0	1	15	263.45	1	6	1
EABT18027	1	1	3	11	1	0	1
EABT18028	1628.17	2525.94	2855.49	5102.5	1851.65	1731.58	1367.54

EABT18029	0	1	14	2	2	1	0
EABT1803	1	1	1	3	0	1	0
EABT18030	0	3	18	4	1	0	0
EABT18031	9	2	7	5	2	0	22
EABT18032	0	0	0	2	0	0	1
EABT18033	3	2	0	0	0	0	0
EABT18034	0	1	13	5	2	0	1
EABT18035	76	274.75	307.15	276.2	241.63	39.01	375
EABT18036	1	1	4	0	0	0	0
EABT18037	0	0	1	1	0	0	0
EABT18038	0	0	0	0	0	0	0
EABT18039	4	2	2	0	0	0	1
EABT1804	1	4	3	2	0	2	0
EABT18040	83.03	125	536	233.05	54.81	6	26
EABT18041	6113.19	5263.52	1851	6648.69	2765.63	4995.32	4317.01
EABT18042	3	5	15	6	1	0	3
EABT18043	4464.74	7257.35	8598.5	15100.6	5203.89	3194.66	3414.24
EABT18044	2	7	11	115	9	1	19
EABT18045	1127.01	1107.91	397	1267.57	721	863	807
EABT18046	1537.21	1803.23	607.12	1412.08	1160.86	808.53	3876.34
EABT18047	1	2	8	2	1	1	2
EABT18048	241	1023.3	1402	25	10634.2	4	858.53
EABT18049	0	4	7	15	2	0	1
EABT1805	12166.3	742.02	30	65	13	7557.76	3.39
EABT18050	7	3	23	15	0	1	3
EABT18051	4	24	8	31	2	0	0
EABT18052	10	70	703	1854	23	2	21
EABT18053	1	1	0	1	3	0	0
EABT18054	808.78	1242.99	988.58	2509.46	1005.95	490	790.99
EABT18055	5	0	0	0	1	4	0
EABT18056	0	0	1	0	0	0	1
EABT18057	5930.29	5259.42	6119.82	3544.37	13307.4	3784.88	2849.59
EABT18058	0	3	37	7	0	2	0
EABT18059	1	4	16	0	0	1	0
EABT1806	0	3	0	1	3	0	0
EABT18060	1	2	49	2	0	3	0
EABT18061	1124.65	2260.5	1566	7190.41	2207.92	2068.37	1745.96
EABT18062	34	26.99	41	44	45	15	36
EABT18063	0	3	15.79	9	1	1	2
EABT18064	0	2	11	0	0	0	0
EABT18065	0	4	23	122	3	0	1
EABT18066	51	68	119	259.58	183.64	125.99	180
EABT18067	1291.14	2148.03	2371.15	4630.64	1335.05	1109.75	1070.03
EABT18068	0	0	5	1	0	0	0
EABT18069	1107.36	883.16	963.02	1019.47	746.2	1179.87	848.36
EABT1807	45	50	414	20026.5	238	1	1
EABT18070	0	2	15	3	1	0	2
EABT18071	0	3	1	3	3	2	2
EABT18072	1	4	8	10	1	1	0
EABT18073	0	1	3	2	2	0	0
EABT18074	5699.89	1906.25	166	0	57	1769.96	870.01

EABT18075	8	13	14	277	28	9	9
EABT18076	634	849.11	775	1710.27	730.97	560	541.99
EABT18077	0	0	5	0	0	0	0
EABT18078	1	10	20	0	0	2	0
EABT18079	0	1	9	1	0	0	1
EABT1808	4	10	0	0	5	6	2
EABT18080	1	14	20	8	0	1	0
EABT18081	2	2	0	0	0	10	1
EABT18082	2	2	23	4	0	0	3
EABT18083	5	16	44	8	2	0	2
EABT18084	1	9	13	1	16	0	93
EABT18085	1	0	1	4	0	0	0
EABT18086	1	4	6	6	1	0	1
EABT18087	0	0	5	0	0	1	0
EABT18088	906.02	1118.72	629.07	55.02	211.99	1012.1	1000.86
EABT18089	0	3	4	2	0	1	0
EABT1809	3420.23	3926.1	1553.58	647.99	250.39	20	37
EABT18090	7	2	54	2	11	2	17
EABT18091	12	18	9	17	3	3	11
EABT18092	9	18.93	28	8	22	9	48.95
EABT18093	0	3	64	0	0	1	6
EABT18094	14	24	35	9	2	23	20
EABT18095	412	384.28	176.01	324	156.99	118.01	441.91
EABT18096	2	10	17	40	1	2	3
EABT18097	3	7	3	12	2	7	12
EABT18098	19	7	0	0	0	7	3
EABT18099	4156.95	3850.79	4707.57	2397.11	1962	4965.97	3143.07
EABT181	0	0	0	0	3	0	0
EABT1810	1	1	20	4	1	0	0
EABT18100	245.71	395.23	468.41	552.12	387.8	331.28	252.85
EABT18101	10	11	60	4	3	5.23	15
EABT18102	0	10	221	43.92	26	1	3
EABT18103	1	1	4	0	0	0	2
EABT18104	4	21	54	10	38	6	18
EABT18105	1148.65	5561.57	6639.99	4857.95	8724.99	335.05	1990.73
EABT18106	6	34	36	30	15	10	22
EABT18107	851.83	1344.48	4114.94	1937.73	1171.19	248.04	521.7
EABT18108	0	0	2	2	0	0	0
EABT18109	3516.11	4901.8	5446.32	9081.38	3943.95	2576.7	2818.29
EABT1811	1	2	1	0	0	0	0
EABT18110	0	0	3	0	1	0	0
EABT18111	4	6	25	0	1	1	2
EABT18112	51	77.01	429.52	188.14	123.36	26.3	93
EABT18113	666	1312.95	1316	2414.65	738.01	970	782
EABT18114	8	12.68	11	31	8	9	5
EABT18115	0	1	16	0	0	0	0
EABT18116	8	0	0	0	0	4	5
EABT18117	0	2	21	3	0	2	0
EABT18118	0	0	6	1	0	0	0
EABT18119	0	0	9	0	0	0	0
EABT1812	1	5	1	7	1	0	2

EABT18120	11	69	44	62	8946.1	45	71
EABT18121	2	6	37	14	4	2	0
EABT18122	2	5	4	1	6	0	15
EABT18123	0	1	3	1	0	0	0
EABT18124	2	3	4	5	0	0	0
EABT18125	490	1045	923	1508.8	955.37	456.99	454.98
EABT18126	1	9	3	0	0	2	2
EABT18127	1	2	23	4	2	2	0
EABT18128	0	0	5	1	3	3	2
EABT18129	0	0	9	1	2	0	0
EABT1813	0	3	0	0	2	0	1
EABT18130	1	2	34	2	2	1	1
EABT18131	1	1	18	4	0	0	0
EABT18132	0	0	3	3	7	0	0
EABT18133	0	1	8	1	2	1	0
EABT18134	0	1	4	0	0	2	0
EABT18135	0	2	3	0	0	1	0
EABT18136	19	13	68	19	12	19	6
EABT18137	2	6	21	2	4	0	6
EABT18138	3	0	30	1	2	0	1
EABT18139	0	0	4	1	0	1	0
EABT1814	197	262	255	189.02	127	211.62	204.47
EABT18140	0	0	1	32	1	0	0
EABT18141	4	20	8	1	1	1	4
EABT18142	3728.11	10049.4	9613.32	24688.7	6296.04	5471.08	4898.36
EABT18143	0	1	4	8	2	1	1
EABT18144	8	6	10	15	27	1	3
EABT18145	12	10	47	5	3	40	11
EABT18146	419	874.9	457	334	76	202	64
EABT18147	0	0	49	1	0	0	1
EABT18148	1	6	1	3	1	2	1
EABT18149	0	0	2	4	1	1	0
EABT1815	1	7	2	8	4	1	2
EABT18150	0	2	59	1	2	1	0
EABT18151	1	4	106	1	1	0	2
EABT18152	0	2	1	1	0	2	0
EABT18153	2	4	6	0	3	0	13
EABT18154	982	2742.01	3977.69	120	1069	97	266
EABT18155	15	21	36	27	12	12	15
EABT18156	2	3	7	3	2	1	0
EABT18157	1	4	5	0	0	0	1
EABT18158	1	3	8	7	1	0	1
EABT18159	1	0	6	0	0	0	1
EABT1816	569.99	899.04	641.72	1273.75	814.72	543.71	515.03
EABT18160	2	2	0	0	0	6	5
EABT18161	1	1	3	3	0	0	0
EABT18162	0	4	37	4	0	0	0
EABT18163	16	5	2	6	0	21	3
EABT18164	1	3	7	14	9	0	3
EABT18165	57	36	38	27.55	207	112.6	146.63
EABT18166	3	2	10	0	4	0	2

EABT18167	25	33	92	50	4	39	18
EABT18168	30	46	14	28	12	11	17
EABT18169	7	3	1	0	0	0	0
EABT1817	4	4	10	7	2	1	3
EABT18170	7	35	12	94	38	7	24
EABT18171	0	2	12	5	0	0	1
EABT18172	0	0	2	0	7	1	0
EABT18173	2	0	3	1	3	4	11
EABT18174	2	11	0	0	2	0	1
EABT18175	2	7	12	2	1	4	1
EABT18176	1	0	0	4	1	0	0
EABT18177	10	6	14	20	8	5	10
EABT18178	909.12	1104.28	1358	2371.66	1214.54	681.86	706
EABT18179	3	3	4	6	3	0	2
EABT1818	7	3	15	1	1	4	0
EABT18180	3	8	22	6	0	2	4
EABT18181	0	0	0	0	5	0	7
EABT18182	1	2	3	6	1	4	0
EABT18183	13	26	63	97	23	20	87
EABT18184	112	109	9	3	17	6	29
EABT18185	4.05	23.26	33.37	135.51	147.65	3.54	7.58
EABT18186	0	1	0	5	1	0	0
EABT18187	13	30	29	63	36	8	13
EABT18188	17	14	23	65	6	10	8
EABT18189	0	18	3	0	1	1	0
EABT1819	0	1	7	0	0	0	0
EABT18190	1377	2058	4894.95	2195.35	1410.15	1373.31	1344.49
EABT18191	0	0	2	3	0	0	0
EABT18192	1	0	12	1	0	0	0
EABT18193	6	4	0	0	1	0	1
EABT18194	3	6	25	0	2	0	1
EABT18195	3318	4522.57	5963.9	5934.34	3355.63	4332.74	2530.09
EABT18196	39	54	152.95	90.01	112	40	38
EABT18197	15664.4	14339.4	7328.41	14558.5	11029.1	15689.3	12495.9
EABT18198	2	5	25	2	0	1	0
EABT18199	2446.14	3906.02	4422.5	5631.19	3417.83	4053.45	3614.55
EABT182	7	51	21	0	9	1	3
EABT1820	0	0	25	0	0	0	0
EABT18200	0	4	11	3	2	1	1
EABT18201	1	5	4	5	1	1	2
EABT18202	0	0	20	1	0	0	0
EABT18203	24	0	3	5	0	3	8
EABT18204	3	6	44	11	3	1	1
EABT18205	5	2	3	2	6	0	0
EABT18206	1	4	6	1	1	4	0
EABT18207	17206.4	7216	1519	63	4509.84	34619.4	17643.3
EABT18208	16	6	3	0	0	0	0
EABT18209	4972.37	16054.9	1949.26	58	1750.19	11202	14
EABT1821	1	5	4	0	2	2	0
EABT18210	0	0	5	0	0	0	0
EABT18211	2	7	27	8	1	8	3.99

EABT18212	3	6	0	0	0	1	3
EABT18213	1	0	0	0	1	0	0
EABT18214	2	2	3	7	0	0	0
EABT18215	5352.71	6128	3334.02	10490.2	3603.84	5247.1	4263.94
EABT18216	4	14	36	7	4.97	1	3
EABT18217	1.99	6	103.02	4.66	15.98	2	15
EABT18218	7	6	6	1	2	9	24
EABT18219	0	2	10	1	1	0	0
EABT1822	2	0	15	1	1	0	0
EABT18220	0	0	2	2	2	0	0
EABT18221	1	0	36	2	0	2	4
EABT18222	0	0	7	1	0	0	0
EABT18223	0	1	7	1	1	0	0
EABT18224	4	4	30	1	2	4	2
EABT18225	9	6	4	1	0	1	0
EABT18226	0	0	3	1	3	2	1
EABT18227	8836.06	8227.1	5002.06	6932.01	6352.47	9697.21	7578.54
EABT18228	0	2	10	0	0	0	0
EABT18229	507.04	631.66	471.8	1252.96	681.02	349.3	658.95
EABT1823	0	0	4	9	1	0	1
EABT18230	2344.73	3304.75	1028.07	2254.51	2113.1	1551.09	2022.89
EABT18231	12	8	4	0	0	9	1
EABT18232	6	4	29	4	1	1	1
EABT18233	1	1	0	0	0	2	1
EABT18234	2	1	3	1	0	1	1
EABT18235	0	1	7	9	0	0	3
EABT18236	3	2	3	1	739	1	0
EABT18237	2	1	13	2	1	1	1
EABT18238	2	1	1	1	0	1	0
EABT18239	0	0	14	1	0	0	0
EABT1824	16	33	39	6	2	6	0
EABT18240	1	1	14	1	0	0	1
EABT18241	0	0	25.09	4	0	0	0
EABT18242	3	4	10	388.03	2	9	9
EABT18243	265	414	539.78	1235.07	528.99	116.02	216
EABT18244	0	0	20	2	0	1	0
EABT18245	3	2	11	5	0	0	0
EABT18246	1503.13	2108.21	3782.97	5710.95	2349.8	1691.06	1374.25
EABT18247	29	60	135	101	64	13	16
EABT18248	1	3	1	1	2	0	0
EABT18249	0	0	6	0	1	0	0
EABT1825	4	1	8	8	5	1	0
EABT18250	2	5	11	12	0	1	3
EABT18251	1	5	0	0	0	1	1
EABT18252	3433.27	4754.95	5649.82	5067.04	3134.29	2655.37	2985.71
EABT18253	4373.43	6123.23	5513.99	4362.28	5079.3	5443.47	5133.67
EABT18254	8	4	0	0	0	0	2
EABT18255	0	5	2	7	1	1	0
EABT18256	14	12	0	0	2	43	18
EABT18257	7	5	10	10	6	2	0
EABT18258	14	3	0	0	0	0	0

EABT18259	464.01	1018.35	1422.84	5961.92	2415.13	97.02	160
EABT1826	7	13	31	26	4	0	3
EABT18260	0	1	1	0	439.97	3	0
EABT18261	0	0	5	2	1	0	1
EABT18262	30	30	97.11	33	35	12	40
EABT18263	7	7	24	24	6	7	4
EABT18264	0	0	19	1	0	0	0
EABT18265	2	7	10	12	5	5	12
EABT18266	4	1	9	0	3	2	0
EABT18267	129	229	600.02	89	766.88	33	82
EABT18268	0	0	7	0	0	0	0
EABT18269	4118.31	9399.75	2979.7	5170.72	4089.84	2220.04	1530
EABT1827	0	0	0	0	92.58	0	0
EABT18270	0	0	6	0	1	1	0
EABT18271	0	0	5	2	0	0	1
EABT18272	20	14	6	4	15	2	4
EABT18273	0	5	2	8	7	2	0
EABT18274	148.01	240.35	329.07	992.99	396.02	81.15	96
EABT18275	1	3	0	1	2	0	0
EABT18276	0	2	8	0	0	0	0
EABT18277	0	3	2	13	0	0	0
EABT18278	1024.51	1987	2465	4897.05	1673.93	1184	1096.91
EABT18279	0	1	18	3	0	1	0
EABT1828	1	1	3	22.66	2	0	0
EABT18280	2	9	16	19	3	0	4
EABT18281	0	7	2	2	0	1	0
EABT18282	2	0	4	0	2	5	4
EABT18283	1	3	2	3	0	0	0
EABT18284	17	9	12	3956.66	2	0	0
EABT18285	1	5	5	8	2	0	1
EABT18286	0	0	2	0	0	0	0
EABT18287	3	13	28	10	11	2	8
EABT18288	1	1	42	1	7	0	3
EABT18289	2915.93	3388	1126.34	2709.78	1389	2828.1	1616.46
EABT1829	0	0	3	3	1	0	0
EABT18290	0	2	8	12	2	1	5
EABT18291	85.8	287	1500.09	621	765.98	28	207
EABT18292	5	3	0	0	0	2	0
EABT18293	3	14	13	15	2	0	0
EABT18294	0	2	2	3	0	0	2
EABT18295	668.9	1126.9	1384.78	2624.97	719.2	960.38	615.06
EABT18296	9	38.01	42	20	0	12	1
EABT18297	0	1	4	3	1	1	0
EABT18298	0	0	2	3	0	0	0
EABT18299	1	6	8	42	65	0	17
EABT183	10	20	0	0	0	0	0
EABT1830	1	5	10	4	586	3	0
EABT18300	2	4	10	9	1	6	1
EABT18301	30	110.85	244.87	181.95	20	26	19
EABT18302	4	5	1	2	0	0	0
EABT18303	29	1	0	0	1	40	3

EABT18304	0	1	6	0	4	0	0
EABT18305	0	2	24	2	3026.06	2	3
EABT18306	11	6	12	12	4	8	4
EABT18307	1	0	0	1	7	0	0
EABT18308	0	0	4	4	0	0	0
EABT18309	1	0	1	2	4	1	8
EABT1831	0	0	4	0	0	3	1
EABT18310	1	1	22	4	0	2	1
EABT18311	4151.95	5298.87	4235.05	15016.7	2662.78	5355.14	2531.02
EABT18312	3	6	30	2	9	0	1
EABT18313	4	9	3	5	5	0	0
EABT18314	247.02	364.2	549.4	673.13	376.37	193.92	234.67
EABT18315	54	2434.66	2170.84	1454.01	89281.3	41	249
EABT18316	28	41	25	47	15	6	15
EABT18317	2	3	16	4	0	1	0
EABT18318	6289.78	10606.5	12995.7	13730	5617.72	2002.52	5038.8
EABT18319	0	0	0	3	1	0	0
EABT1832	1	1	7	3	0	0	0
EABT18320	2	1	36	2	0	0	1
EABT18321	1	2	31	4	0	1	2
EABT18322	3	6	7	3	13	1	2
EABT18323	5	26	44	61	6	9	10
EABT18324	0	3	0	1	0	5	0
EABT18325	29	43	2	7	8	9	15
EABT18326	1	1	16	6	1	4	4
EABT18327	890.43	1289.18	429.7	1779.7	827.57	549.92	686.39
EABT18328	2	3	7	1	0	0	1
EABT18329	2	8	14	7	2	1	0
EABT1833	0	1	2	0	0	0	0
EABT18330	3	17	104	8	3	4	0
EABT18331	820.11	1441.36	1353.99	1106.11	1004	577	688
EABT18332	13	1	4	0	0	4	0
EABT18333	0	0	8	10	0	0	0
EABT18334	0	2	5	1	1	1	1
EABT18335	0	1	11	0	0	0	0
EABT18336	0	2	3	4	0	0	1
EABT18337	4	2	0	16	1	2	0
EABT18338	1	3	7.97	0	4	0	0
EABT18339	1	1	4	0	6	0	1
EABT1834	0	4	20	0	0	0	1
EABT18340	388	932	882	2414.91	592	366	241.44
EABT18341	4	3	0	0	0	0	0
EABT18342	16667.8	46522.3	356.22	0	152.22	7733.06	744.8
EABT18343	8	25	183	9	17	12	5
EABT18344	0	0	5	3	0	0	0
EABT18345	0	2	2	3	2	0	0
EABT18346	7469.77	32009.9	25165.6	5678.58	67984.6	723	5309.95
EABT18347	22	31.21	54	32	15	1	3
EABT18348	0	3	10	15	7	1	1
EABT18349	920	1192.32	759.84	33	1005.86	614.01	4673.76
EABT1835	3	9	20	2	2	23	12

EABT18350	1453.28	1376.51	5243.03	9918.82	380	786	400
EABT18351	578.84	435	228.99	403	540	445	529
EABT18352	78	111	484	0	0	0	1
EABT18353	0	0	0	6	0	0	0
EABT18354	0	2	6	3	3	1	0
EABT18355	711.02	834	186	134	203.93	769	938.32
EABT18356	3	4	2	2	4	9	5
EABT18357	1	7	2	12	6	0	5
EABT18358	4	5	0	0	0	1	1
EABT18359	14	3	3	49	29	3	1
EABT1836	10	6	21	1	0	2	2
EABT18360	9	4	0	4	4	0	1
EABT18361	1	2	10	4	0	2	0
EABT18362	547.96	688.8	1208	1472	573.01	547	504
EABT18363	0	1	1	1	2	1	3
EABT18364	0	1	2	5	0	0	0
EABT18365	0	2	17	0	5	1	8
EABT18366	0	0	6	0	0	0	1
EABT18367	0	0	0	0	8	0	0
EABT18368	7	11	11.99	1	0	0	2
EABT18369	1	2	30	0	0	3	1
EABT1837	2	0	3	0	5	1	10
EABT18370	1	1	2	0	0	0	0
EABT18371	54	59	29.59	62	35.99	4	19
EABT18372	0	2	0	1	0	0	1
EABT18373	13	0	0	0	0	2	0
EABT18374	1	11	39	1	2	0	8
EABT18375	0	6	9	0	1	1	1
EABT18376	19	18	0	4	7	0	10
EABT18377	2	0	5	0	0	0	0
EABT18378	0	9	22	2	0	2	4
EABT18379	69	125.43	655.06	172	15	24	18
EABT1838	2	0	30	1	3	2	1
EABT18380	1	2	0	0	3	0	5
EABT18381	21.98	44	13	120	8	24	7
EABT18382	2	2	1	2	0	0	0
EABT18383	0	0	2	10	0	1	1
EABT18384	0	0	1	0	0	0	0
EABT18385	0	2	1	7	0	1	3
EABT18386	3634.78	3678.02	1730.16	2149.42	1180.4	3066.69	2582.32
EABT18387	0	2	23	0	0	0	0
EABT18388	0	1	6	1	0	0	0
EABT18389	1	0	7	1	0	0	1
EABT1839	0	0	13	0	0	0	0
EABT18390	5	5	7	2	1	0	9
EABT18391	1	9	37	1	0	0	0
EABT18392	1	3	13	0	1	23	3
EABT18393	0	0	1	0	0	0	2
EABT18394	22654.3	15421.9	1678.61	273.14	2841.64	52940.9	11368.9
EABT18395	0	0	3	1	1	0	0
EABT18396	0	0	3	1	4	1	2

EABT18397	256	235	58	175	107	40	125
EABT18398	0	0	18	0	0	0	0
EABT18399	179.06	233	186	419	284	120	95
EABT184	1	1	6	0	0	1	1
EABT1840	0	0	21	0	1	1	0
EABT18400	13	28	16	54	36.99	10	13
EABT18401	1	1	71	9	2	0	0
EABT18402	74722.6	62791.8	45481.7	75875.6	2256.25	2458	2031.72
EABT18403	0	1	3	3	0	3	0
EABT18404	589	805	392.36	870	583	418	500.34
EABT18405	9	13	21	8	1	10	4
EABT18406	3403.64	4596.94	2235.58	3392.06	2396.7	5728.68	8963.07
EABT18407	5	8	74	2	3	9	13
EABT18408	0	0	1	2	0	0	0
EABT18409	1	7	57	12	0	0	2
EABT1841	1	2	19	2	0	0	0
EABT18410	2	8	47	3	0	0	1
EABT18411	1	5	3	2	0	0	1
EABT18412	15	18	0	0	0	6	4
EABT18413	6	21	10	176	15	1	2
EABT18414	1	1	12	1	1	3	7
EABT18415	1252.37	1641.56	1185.21	1169.89	961.94	1080.33	833.36
EABT18416	2	6	3	3	1	2	2
EABT18417	6.98	8	28	3	2	2	3
EABT18418	1	0	1	4	0	1	0
EABT18419	1562.61	1187.91	936.81	811.46	810.82	1653.05	1316.99
EABT1842	1	2	1	3	1	0	0
EABT18420	0	0	8	0	0	0	0
EABT18421	0	0	4	0	0	0	0
EABT18422	0	1	0	0	1	1	11
EABT18423	1	18	0	0	1	15	2
EABT18424	0	5	5	5	0	0	0
EABT18425	0	1	0	25	0	0	0
EABT18426	0	1	6	0	0	0	0
EABT18427	1	1	14	0	0	0	0
EABT18428	91	106.46	51.15	419.81	79.82	15	24
EABT18429	1262	1705.8	3865.74	3582.55	1562.06	1355.15	1320
EABT1843	0	0	11	0	0	0	0
EABT18430	6	32	25	4	18	2	1
EABT18431	10	73	26	87	3	3	0
EABT18432	1	0	1	1	4	5	31
EABT18433	16	16	12	2	1	1	0
EABT18434	1	4	9	0	4	3.83	5
EABT18435	5	4	9	10	0	2	0
EABT18436	0	0	0	0	0	0	0
EABT18437	5	6	0	8	0	2	0
EABT18438	2863.24	3351.17	3337.16	5241.58	2600.95	1742.3	2035.05
EABT18439	1	1	2	2	0	1	0
EABT1844	0	0	6	1	2	0	0
EABT18440	0	2	21.03	5	1	1	0
EABT18441	2	2	0	0	0	0	1

EABT18442	1473.18	1770.68	579.57	677.72	1774.88	22	603.64
EABT18443	5	4	0	0	0	1	1
EABT18444	35	66	76	13	24	4	13
EABT18445	2	1	7	4	0	0	0
EABT18446	3	6	35	4	2	1	1
EABT18447	281.35	607.84	777.03	1083.43	827.31	294.18	374.96
EABT18448	12	24	29	49	11	6	8
EABT18449	0	0	2	0	0	0	0
EABT1845	5	2	0	0	2	33	16
EABT18450	0	1	2	0	1	0	14
EABT18451	3	2	0	3	53	0	1
EABT18452	18	37	87	69.94	15	16	26
EABT18453	0	0	7	0	1	0	0
EABT18454	10	16	42.98	37	6	16	5
EABT18455	1	0	0	6	1	0	2
EABT18456	319.94	547.76	611.24	1353.57	121.16	279.24	259.47
EABT18457	1	7	16	2	3	1	0
EABT18458	0	4	1	5	2	1	0
EABT18459	0	0	12	0	0	0	0
EABT1846	0	0	1	0	1	1	1
EABT18460	2	0	3	0	0	0	2
EABT18461	1	3	10	2	3	0	0
EABT18462	190.68	226	133	351.59	56	44	74
EABT18463	997.05	1736.8	3277.44	2522.57	1569.98	797.01	854
EABT18464	76	147	524	1678.17	421.96	17	36
EABT18465	0	2	25	32	1	2	1
EABT18466	3	16	23	36	2	0	1
EABT18467	0	0	2	3	0	0	1
EABT18468	19	60	33	149	18	5	6
EABT18469	0	7	28	1	0	0	0
EABT1847	0	3	0	0	1	0	0
EABT18470	5641.63	6758.22	5808.04	2437.02	4706.03	11596.5	9230.18
EABT18471	1	0	28	4	0	0	0
EABT18472	6	10	5	13	1	0	2
EABT18473	2	3	34	0	0	0	0
EABT18474	1	1	4	0	0	0	0
EABT18475	0	1	11	2	0	0	0
EABT18476	0	3	8	1	0	0	0
EABT18477	0	1	7	0	11	2	10.3
EABT18478	1	2	0	8	0	0	0
EABT18479	5568.28	6013.08	440.02	142	725.4	6281.82	1169.02
EABT1848	24	81	24	0	28	37	31
EABT18480	842	1921.98	2845.25	4415.75	4939.25	211	1088.48
EABT18481	0	3	2	1	0	1	0
EABT18482	1	1	0	1	0	2	0
EABT18483	8	22	43	35	27	6	5
EABT18484	2	10	73.76	16	0	0	0
EABT18485	0	0	5	0	0	1	0
EABT18486	0	0	9	1	0	0	0
EABT18487	11	11	8	29	8	1	8
EABT18488	0	0	12	0	0	0	1

EABT18489	0	0	3	1	6	0	5
EABT1849	0	0	3	0	1	0	8
EABT18490	207.11	375.97	268.25	399.81	262.1	73	182.2
EABT18491	1	0	2	0	0	1	2
EABT18492	8	7	5	38	12	8	29
EABT18493	0	1	3	2	0	0	0
EABT18494	24.16	23	100	106	134.01	34	33
EABT18495	7	1	4	0	0	0	0
EABT18496	10	20	22	38	7	10	11
EABT18497	6	28	8	9	23	0	1
EABT18498	1	0	7	0	0	0	1
EABT18499	4152.19	5173.91	1673.4	2143.06	3224.12	2643.6	3111.89
EABT185	0	4	11	0	1	1	0
EABT1850	369.35	661.17	1339.54	2090.66	908.35	186	480.66
EABT18500	1	2	30	1	6	2	3
EABT18501	0	0	0	0	10	0	0
EABT18502	0	0	1	5	1	0	0
EABT18503	2	6	1	2	1	2	11
EABT18504	0	0	8	0	1	0	1
EABT18505	2	0	0	20	0	2	0
EABT18506	0	7	10	2	0	1	1
EABT18507	28.01	70	148	618.59	92	7	87
EABT18508	3597.52	4179.91	371.67	22	205.99	6089.57	1127.05
EABT18509	48	114	82	177	24	22	47
EABT1851	1	0	6	0	1	1	0
EABT18510	0	0	14	0	0	1	2
EABT18511	372.72	691.99	936.06	623.02	892.23	227.32	490.61
EABT18512	19	20	58.9	37	13	12	13
EABT18513	1	1	4	0	0	0	0
EABT18514	34	30.97	38	94.32	19.91	7	36
EABT18515	4	1	3	0	0	3	0
EABT18516	7	32	25	93	3	3	10
EABT18517	427	774	686.62	1117.75	923	282.82	448.91
EABT18518	1	2	6	1	0	1	0
EABT18519	41	171	120	80	177	9	9
EABT1852	2	3	2	3	0	0	0
EABT18520	0	0	49.11	0	1	0	0
EABT18521	0	0	5	21	2	0	4
EABT18522	2	1	38	3	0	3	0
EABT18523	0	0	4	4	0	0	0
EABT18524	3	7	1	0	0	7	0
EABT18525	0	0	1	0	3	0	2
EABT18526	23	45	13	17	106	0	272
EABT18527	26	5	0	5	0	0	1
EABT18528	0	3	4	4	0	0	0
EABT18529	0	1	1	0	3	0	0
EABT1853	0	6	40	18	3	0	1
EABT18530	5	7	6	1	1	5	1
EABT18531	0	0	1	1	1	1	0
EABT18532	1874.69	4603.94	9129.99	7771.91	5947.15	3329.34	4002.31
EABT18533	2	1	50	0	0	0	0

EABT18534	4	5	21	1	1	0	0
EABT18535	0	0	6	1	1	1	0
EABT18536	377.67	321.99	253	312	363	174	170.04
EABT18537	4	5	11	5	3	0	0
EABT18538	2	3	59	10	2	0	1
EABT18539	0	0	1	6	0	0	0
EABT1854	1	0	0	2	0	1	0
EABT18540	0	3	11	0	0	0	0
EABT18541	8249.75	12964.7	5305.99	7537.96	620	36	71
EABT18542	2	9	4	1	8	0	1
EABT18543	0	0	13	0	0	0	0
EABT18544	2	2	3	4	1	0	0
EABT18545	2	7	18	9	1	2	1
EABT18546	0	2	1	12	0	0	0
EABT18547	11	9	35	36	4	12	8.55
EABT18548	143.85	675.1	2540.44	4518.99	954	84	285
EABT18549	0	0	0	9	0	0	0
EABT1855	1	4	6	1	0	0	0
EABT18550	3	9	3	0	2	0	0
EABT18551	0	1	17	1	1	1	0
EABT18552	0	0	0	0	0	0	0
EABT18553	1	1	2	1	0	0	0
EABT18554	0	11	29	18	0	1	0
EABT18555	2	4	9	0	0	0	0
EABT18556	27	118	89	144	43	15	32
EABT18557	10	19	39.03	25	5	16.96	6
EABT18558	0	0	7	2	1	0	0
EABT18559	1	0	2	1	0	0	0
EABT1856	4	6	1	0	5	0	1
EABT18560	3	4	5	12	2	0	1
EABT18561	1	4	11	12	0	7	2
EABT18562	3	1	5	1	7	3	10
EABT18563	1	6	12	62	12	1	1
EABT18564	406.35	245.96	303.81	285.48	172.38	454.95	116.09
EABT18565	0	0	10	2	0	0	0
EABT18566	6	8	20	7	0	5	5
EABT18567	0	0	6	9	1	0	0
EABT18568	35	100.12	236.04	184.59	31	37	37
EABT18569	0	2	6	0	3	0	1
EABT1857	1	1	2	1	1	0	0
EABT18570	1	2	36	8	6	0	0
EABT18571	133.59	62	20.69	55	50	150	117.37
EABT18572	0	6	18	8	4	1	9
EABT18573	87.02	101.71	117	285.27	90	61	75.98
EABT18574	0	2	19	13	2	0	0
EABT18575	1485.36	1996.32	753.09	3442.23	982.52	800.04	670.81
EABT18576	3	4	15	2	0	2	1
EABT18577	1	1	2	2	0	0	1
EABT18578	0	0	1	16	0	0	0
EABT18579	3	14.97	154.53	96	1	4	9
EABT1858	36.72	70	108.07	60.26	30	17	16

EABT18580	597.19	941.25	490.86	895.23	691.74	503.56	752.08
EABT18581	0	1	4	1	0	0	0
EABT18582	0	0	10	0	0	0	1
EABT18583	0	6	16	6	3	0	3
EABT18584	1	2	13.04	11	1	0	4
EABT18585	1	1	5	1	2	1	1
EABT18586	27	426	77	64	48	3	1
EABT18587	0	1	0	1	0	0	0
EABT18588	0	3	3	4	4	0	2
EABT18589	0	0	8	0	0	1	1
EABT1859	1067.01	2531	3996	883.97	4667.01	711.39	2076.05
EABT18590	0	3	2	12	1	0	1
EABT18591	370.26	1683.23	2817.52	2989.02	4157.08	48	485
EABT18592	0	1	2	7	0	0	0
EABT18593	0	5	1	0	0	4	0
EABT18594	0	0	6	6	0	0	0
EABT18595	3	0	48	3	3	0	0
EABT18596	0	1	3	1	0	0	0
EABT18597	0	2	14	0	0	0	0
EABT18598	197.92	461	45	54	68	207	255.99
EABT18599	0	0	3	3	0	0	0
EABT186	8	16	9	6	3	3	0
EABT1860	0	1	4	2	0	0	0
EABT18600	17	9	46	47	12	10	22
EABT18601	2826.38	3724.17	1690.85	2137.04	2076.41	2533.77	2755.9
EABT18602	0	0	4	3	0	0	0
EABT18603	1	13	0	0	7	0	43
EABT18604	1	0	35	0	0	1	0
EABT18605	0	0	3	1	0	0	0
EABT18606	0	0	9	0	0	0	0
EABT18607	9	8	0	0	0	0	0
EABT18608	0	0	4	4	1	0	1
EABT18609	0	0	8	0	0	0	0
EABT1861	0	1	4	0	0	1	0
EABT18610	0	1	7	0	0	0	0
EABT18611	3	8	21	15	5	3	6
EABT18612	52	84	205	102	8	3.94	12
EABT18613	1	3	3	0	1	0	1
EABT18614	4	2	0	8.94	2.08	22	13
EABT18615	737.02	217.31	57	30	204	558	913.41
EABT18616	0	0	7	1	0	0	1
EABT18617	2	2	9	4	1	0	0
EABT18618	2	1	11	0	1	0	0
EABT18619	0	8	40	147	38	0	2
EABT1862	0	0	13	346	15	0	1
EABT18620	0	1	5	2	0	0	0
EABT18621	2571.05	3082.25	8465.01	3673.5	2211.95	667	1232.07
EABT18622	0	0	3	6	0	0	0
EABT18623	133.25	224.02	491.25	254.96	144.35	107.04	93.78
EABT18624	3	9	3	11	2	4	8
EABT18625	1	2	2	4	1	1	1

EABT18626	7348.05	14465.1	28930.4	14502	5816.56	11931.7	13530.2
EABT18627	0	0	5	35	580.01	0	3
EABT18628	2	6	5	41.51	10	3	15
EABT18629	440	978	2523	1187	291	218	370
EABT1863	0	1	2	0	0	0	0
EABT18630	0	0	0	4	0	0	0
EABT18631	3	4	3	0	1	0	1
EABT18632	0	2	0	0	0	0	0
EABT18633	0	0	2	0	0	3	0
EABT18634	0	1	19	0	0	0	2
EABT18635	8	1	3	0	0	0	0
EABT18636	0	2	21	6	0	0	0
EABT18637	15	1	0	0	1	63	0
EABT18638	4	35	23	31	2	7	11
EABT18639	0	17	4	1	0	0	0
EABT1864	2	4	8	31	4	0	3
EABT18640	880	1491	179.25	99	258	168	381.54
EABT18641	1	0	3	1	1	1	0
EABT18642	0	2	1	5	0	0	0
EABT18643	2	5	0	0	0	3	0
EABT18644	364.83	403.35	375.26	53	26	240.02	74
EABT18645	1	0	12	0	0	0	0
EABT18646	1	5	3	0	0	1	1
EABT18647	4	3	0	0	0	3	4
EABT18648	4	6	6	20	2	6	6
EABT18649	442.7	650.72	580.41	19.13	112.82	3	1305.42
EABT1865	1	5	13	1	2	2	1
EABT18650	3	2	13	0	0	2	2
EABT18651	0	4	0	23	0	1	0
EABT18652	0	3	2	1	0	1	0
EABT18653	53	103	31	231	131	33	47
EABT18654	0	5	3	14	0	0	2
EABT18655	0	6	3	7	0	0	0
EABT18656	5	0	6	2	7	0	0
EABT18657	4031.26	6400.81	8773.73	9503.44	3360.65	4426.44	3673.5
EABT18658	14	15	32	3	5	9	16
EABT18659	18	43	16	136	2.99	9	14
EABT1866	0	8	8	1	1	0	0
EABT18660	2	6	11	5	3	0	12
EABT18661	80	11	123	1400.41	134	1	2
EABT18662	3	6	56	7	3	4	3
EABT18663	0	0	11	8	0	0	0
EABT18664	0	1	9	4	3	3	2
EABT18665	1011.73	1808.77	2333.27	3287.7	1125	773.76	733.06
EABT18666	2	1	7	1	8	2	7
EABT18667	39	84	34	22	10	57	2
EABT18668	1	0	13	0	1	0	0
EABT18669	87	216.01	218.34	951.73	33	5	11
EABT1867	1	6	7	0	0	2	2
EABT18670	5	5	2	7	9	0	7
EABT18671	0	1	2	0	0	0	0

EABT18672	9	10	0	0	2	15	9
EABT18673	0	0	7	0	0	0	0
EABT18674	3962.48	6545.54	5461	10312.8	3146.99	4528.31	3052.85
EABT18675	0	2	17	0	0	0	0
EABT18676	1	6	52.93	20	10	0	1
EABT18677	2649	4390.63	25516.2	2608.7	2590.98	1569.64	1066
EABT18678	1	0	7	1	2	5	7
EABT18679	4	24	53	11	10	12	11
EABT1868	2	1	5	2	0	0	0
EABT18680	4	13	59	18	6	4	44
EABT18681	1	1	0	0	0	3	0
EABT18682	161.99	210.97	244.9	326.01	256	160.99	171.89
EABT18683	0	0	1	0	1	0	0
EABT18684	11	23	63	37	8	2	2
EABT18685	3	5	8	4	0	1	1
EABT18686	77	139	633	382	292	6	9
EABT18687	1	1	6	1	0	0	0
EABT18688	10	8.41	4	16.94	15	0	1
EABT18689	4160.62	7141.43	1756.29	241	1849.33	6568.47	8384.4
EABT1869	0	4	11	7	0	0	1
EABT18690	3	6	45	3	0	0	0
EABT18691	0	4	2	0	1	0	0
EABT18692	0	2	8	0	0	0	1
EABT18693	0	1	5	2	0	0	0
EABT18694	4	5	0	2	3	0	0
EABT18695	10	32	2	0	1	1	1
EABT18696	7	10	4	11	2	5	9
EABT18697	2597	2679	2667	2912.94	2900.12	3453.08	2406.39
EABT18698	4	4	9	12	5	0	0
EABT18699	1	2	1	0	0	1	2
EABT187	1039	1036	591	1961	559	792.04	744.19
EABT1870	1	0	15	0	0	2	0
EABT18700	4	0	1	30	0	1	0
EABT18701	449	679	508	33	427	31	1022
EABT18702	0	2	7.04	0	0	0	0
EABT18703	5	9	30	2	2	12	5
EABT18704	2	17	17	3	1	1	2
EABT18705	0	3	3	0	0	0	0
EABT18706	16	26	15	1	0	0	0
EABT18707	5	5	2	4	11	0	0
EABT18708	0	0	0	0	1	0	5
EABT18709	0	1	2	1	0	2	0
EABT1871	31	34.84	10	11	4	4	9
EABT18710	5	18	11	13	2	3	4
EABT18711	931	940.77	401.98	148	2729	10	2654.54
EABT18712	0	4	7	2	12	0	0
EABT18713	2	3	8	1	0	0	1
EABT18714	1	0	3	0	1	0	0
EABT18715	2	7	0	0	0	1	0
EABT18716	2	0	0	0	0	6	0
EABT18717	0	1	10	2	0	0	0

EABT18718	0	0	0	0	0	0	0
EABT18719	16	46	15	22	2	8	8
EABT1872	4	2	0	0	0	0	2
EABT18720	391.21	365.63	481.56	246.06	329.34	393.86	774.5
EABT18721	878.32	1890.46	1052.61	261	33	1	2
EABT18722	843.71	1652	1678.84	6324.53	3085.05	88	436.9
EABT18723	0	1	0	5	1	0	0
EABT18724	355.31	534.15	911.28	1036.1	441.19	397.83	315.51
EABT18725	307123	852598	106369	429045	144695	97	4660.66
EABT18726	1	4	3	2	3.94	1	1
EABT18727	0	0	0	3	12	1	0
EABT18728	0	0	1	0	5	0	0
EABT18729	0	2	7	0	0	0	1
EABT1873	732.73	1202.43	1504.13	2661.3	1285.98	1010.02	919.98
EABT18730	0	4	5	5	0	0	0
EABT18731	0	2.98	3	114	4	1	10
EABT18732	0	0	0	3	1	0	0
EABT18733	1	2	12	0	0	0	0
EABT18734	0	0	9	0	0	0	0
EABT18735	0	0	0	2	0	1	2
EABT18736	286.5	427.66	182.11	671.74	335.94	208.03	206.09
EABT18737	0	0	0	1	9	0	0
EABT18738	3	2	5	11	1	2	1
EABT18739	0	0	2	7	0	0	0
EABT1874	1	13.2	14	1	1	1	0
EABT18740	0	1	6	3	0	1	0
EABT18741	1765.53	3040.95	2865.67	6143.51	3776.65	1418.53	2149
EABT18742	1	1	0	11	6	0	0
EABT18743	0	0	0	0	3	0	7
EABT18744	43.01	119	128	227	103	40	93.99
EABT18745	10	25	55	5	11	6	3
EABT18746	0	2	4	2	0	0	0
EABT18747	0	0	2	3	3	0	1
EABT18748	1	1	18	8	1	0	9
EABT18749	3	2	10	0	0	2	1
EABT1875	0	0	1	0	4	0	12
EABT18750	9	9	0	0	0	7	0
EABT18751	1465.58	2059.23	3433.26	5395	1191.91	1024.98	1109.32
EABT18752	0	2	4	0	4	1	0
EABT18753	0	0	13	1	0	0	0
EABT18754	8	39	162	122	21	21	52
EABT18755	10	23	17.02	18	11	6	25
EABT18756	0	0	10	1	0	0	0
EABT18757	4	3	0	0	0	10	0
EABT18758	2131.54	3627.72	6086.5	8706.55	3301.72	2370.29	2290.65
EABT18759	2	3	10	10	8	1	0
EABT1876	13	13	20	4	5	32	35
EABT18760	3	6	12	5	4	5	6
EABT18761	0	2	3	2	1	0	0
EABT18762	650.08	617	746.99	1641.02	893.26	300.03	494.21
EABT18763	0	0	10	1	2	0	0

EABT18764	1601.93	3221.63	4767.28	5138.05	2416.73	1308.44	1514.88
EABT18765	2	5	2	5	9	0	4
EABT18766	1	0	13	3	0	0	0
EABT18767	3	1	4	5	2	1	1
EABT18768	80	86	0	0	5	7	1
EABT18769	16	61	91.44	72.77	13.82	30	31
EABT1877	0	0	3	1	0	0	1
EABT18770	2	14	9	211	4	0	1
EABT18771	0	0	4	3	0	0	0
EABT18772	2	8.95	7	4	2	1	3
EABT18773	0	9	17	14	3	0	1
EABT18774	0	2	70	0	0	1	1
EABT18775	0	1	3	29	2	0	1
EABT18776	0	1	3	19	0	0	0
EABT18777	1	1	2	2	0	0	0
EABT18778	0	0	1	4	2	0	0
EABT18779	2739.12	3885.13	2089	1840.04	1578	2965.87	2602.47
EABT1878	0	0	4	0	2	3	0
EABT18780	0	1	7	0	0	0	0
EABT18781	0	5	1	0	0	0	0
EABT18782	3913.96	9557.85	1132.52	83	869.06	7696.9	4913
EABT18783	2467.77	3156.38	6790.55	6110.14	2167.85	2544.86	3280.39
EABT18784	1	3	11	6	2	2	2
EABT18785	1	1	6	3	0	3	2
EABT18786	377.41	505.06	165.16	682.04	275.56	233.84	264.25
EABT18787	397	422	113	390	354.01	261	218
EABT18788	0	0	16	0	0	0	0
EABT18789	0	0	4	0	0	0	0
EABT1879	3	2	22	3	0	0	1
EABT18790	875.77	929.05	573	1044	757.01	578.01	494.02
EABT18791	5	18	15	17	14	1	9
EABT18792	0	1	10	0	0	0	1
EABT18793	0	0	5	0	2	0	3
EABT18794	1248.46	2252.76	2874.98	4861.05	2172.38	1499.04	1635
EABT18795	314	413.11	298	702	245	218	220
EABT18796	6	6	9	2	1	0	2
EABT18797	1	0	1	2	27	0	4
EABT18798	0	0	0	32	0	0	0
EABT18799	0	2	15	6	2	2	1
EABT188	4	6	11	50.24	1	5	3
EABT1880	0	0	4	0	1	0	0
EABT18800	8	18	0	0	3	1	16
EABT18801	2	10	17	12	2	0	2
EABT18802	0	0	2	0	0	0	0
EABT18803	4	2	6	5	41	6	41
EABT18804	2165.92	1027.48	79	3	64.55	1843.66	314.34
EABT18805	406.37	504.14	400.77	1290.2	516.94	304.4	224.15
EABT18806	3	14	5	2	0	3	22
EABT18807	0	1	1	2	0	2	1
EABT18808	0	1	2	8	0	1	1
EABT18809	1096.9	2178.82	2758.02	2149.23	1240.56	1182.53	1116.86

EABT1881	3	4	1	0	4	8	34
EABT18810	124	200	246	317.87	141	168	196
EABT18811	0	2	1	8	1	0	0
EABT18812	3930.6	5626.51	4364.14	4310.52	3929.95	1593.88	2853.23
EABT18813	4	7	0	4	0	2	0
EABT18814	1	0	43.16	4	0	2	0
EABT18815	1	1	1	2	0	0	0
EABT18816	0	0	0	0	15	0	0
EABT18817	0	5	2	0	0	0	0
EABT18818	1	9	35	25	67	4	10
EABT18819	0	0	5	1	0	0	0
EABT1882	0	0	15	0	0	0	0
EABT18820	0	1	0	2	0	1	1
EABT18821	0	0	7	2	1	1	0
EABT18822	0	0	0	0	2	0	0
EABT18823	17	13	10	1	0	2	1
EABT18824	0	0	6	3	0	0	0
EABT18825	1	0	59	1	1	0	0
EABT18826	13	20	38	15	9	6	10
EABT18827	0	16	8	0	0	1	0
EABT18828	0	2	5	4	1	0	0
EABT18829	1	0	8	6	2	0	0
EABT1883	0	0	0	0	1	0	3
EABT18830	38.49	99.47	74.04	218.23	89.49	30	42
EABT18831	900.37	1163.55	782.41	2464.5	1440.92	896.08	1010
EABT18832	4	4	9	1	0	1	0
EABT18833	4	3	37.09	1	0	2	1
EABT18834	8	3	17	2	0	12	0
EABT18835	79.3	85.94	278.8	163.29	26	15.03	9.99
EABT18836	5	6	30	7	3	1	1
EABT18837	3529.09	3814.22	10359.8	2902.78	513.13	348	411.28
EABT18838	1	2	13	5	1	0	0
EABT18839	0	5	5	1	10	0	0
EABT1884	0	4	34	2	5	0	0
EABT18840	1	2	10	33	2	1	8
EABT18841	0	5	42	3	1	0	0
EABT18842	25	13	3	3	13	4	77.03
EABT18843	0	0	13	0	0	0	0
EABT18844	315	236	466	1830.85	3350.98	24.92	57
EABT18845	0	1	4	1	1	0	0
EABT18846	0	1	1	2	0	1	0
EABT18847	4	3	0	0	0	0	0
EABT18848	6	29	116.98	76	69	15	43
EABT18849	7	10	15	8	5	5	8
EABT1885	9	15	11	21	3	5	3
EABT18850	0	2	2	0	0	0	0
EABT18851	938.23	1508.93	1354.98	2315.75	931.19	1263.65	958.57
EABT18852	4	3	57	10	1	0	1
EABT18853	322.99	474	658.99	816.36	726.87	308	355.19
EABT18854	1	8	49	8	3	0	0
EABT18855	7	3	2	1	4	2	1

EABT18856	8	19	61	18	6	11	5
EABT18857	2	0	4	5	0	2	1
EABT18858	0	4	10	0	0	0	0
EABT18859	1272.38	2243.84	24701.4	4017.4	3163.31	1611	1118.95
EABT1886	12	6	34	1	0	26	1
EABT18860	0	1	5	1	0	0	0
EABT18861	0	1	2	1	2	0	0
EABT18862	2	5	5	3	1	1	0
EABT18863	0	0	87	35	16	0	2
EABT18864	2	4	13	2	1	0	1
EABT18865	2	4	5	0	0	0	0
EABT18866	0	0	4	3	1	0	0
EABT18867	1	0	2	0	0	0	2
EABT18868	0	1	38	1	1	0	0
EABT18869	0	1	13	0	2	0	0
EABT1887	0	0	2	0	0	1	0
EABT18870	0	2	1	5	1	0	0
EABT18871	12	9	51	15	3	8	6
EABT18872	2	1	22	85	7	0	1
EABT18873	0	0	13	20	0	0	0
EABT18874	1	2	11	0	1	0	0
EABT18875	11	20	160	120.91	1	5	2
EABT18876	0	4	6	4	1	0	0
EABT18877	0	1	6	0	0	0	1
EABT18878	6	16	23	5	7	0	25
EABT18879	98323.7	41494.4	27	4	3	9064.12	3
EABT1888	10	49	14	53	258	2	8.98
EABT18880	0	0	2	0	4	0	3
EABT18881	2	1	11	2	0	2	1
EABT18882	3	16	15	9	0	1	2
EABT18883	4	9	4	0	4	0	2
EABT18884	0	1	1	1	0	17	0
EABT18885	0	0	20	4	1	0	1
EABT18886	0	5	10	9	6	1	5
EABT18887	986	1707	1441	2736.7	973	937	1013
EABT18888	8640.01	18606.7	14895.4	42659.2	11884.9	4778.13	6184.9
EABT18889	2	5	6	8	1	0	0
EABT1889	2158.37	1947.41	403.97	130	602.11	2866.15	1751.05
EABT18890	2	0	10	1	0	0	0
EABT18891	3	3	21	1	1	0	0
EABT18892	0	0	7	15	3	0	3
EABT18893	1659.99	31	296.01	425	1808.36	44	3358.96
EABT18894	1225	279	9	0	49	1248.48	265
EABT18895	1	1	14	2	5	1	4
EABT18896	6449.41	8150.22	4667.1	9194.06	2711.19	3796.57	3102.1
EABT18897	913.06	1428.63	1848.44	3123.66	1363.47	852.05	834.22
EABT18898	8	3	4	34	3	8	6
EABT18899	2	19.02	37	3	4	0	0
EABT189	1	24	21	16	2	2	5
EABT1890	0	1	14	4	1	0	0
EABT18900	2	29	65	12	1	0	0

EABT18901	6	7	11	9	2	1	3
EABT18902	0	2	7	2	1	0	0
EABT18903	4	13	37	4	7	5	4
EABT18904	0	2	21	0	0	0	3
EABT18905	1080.32	1379.8	1083.43	744.97	2225.02	1192.73	1271.1
EABT18906	1	0	5	0	1	2	0
EABT18907	0	0	10	0	0	0	0
EABT18908	0	1	2	1	0	0	0
EABT18909	2	3	7	1	0	0	0
EABT1891	2777.99	3659.11	3698.71	7347.75	2211.07	2232.28	2551.9
EABT18910	0	0	1	5	0	0	0
EABT18911	23998.8	27634.4	23050.5	17325.5	21616.7	25506.8	27817.5
EABT18912	53	65	239	329.94	577.38	3	11
EABT18913	466.11	1692.09	17	6	345.13	1382.71	2740.58
EABT18914	4	9.04	3	8	15.52	0	0
EABT18915	0	2	7	0	0	0	0
EABT18916	0	0	5	0	0	0	0
EABT18917	0	0	9	0	0	0	1
EABT18918	0	1	3	1	2	0	0
EABT18919	0	1	15	2	1	0	0
EABT1892	2	1	10	5	0	0	0
EABT18920	0	2	0	1	0	2	0
EABT18921	6	12	6	4	1	3	2
EABT18922	0	0	5.3	1	0	0	0
EABT18923	0	0	10	0	0	0	0
EABT18924	3879	11318.4	632.14	33	1725	3930.92	4550.08
EABT18925	3	3	20	1	0	1	4
EABT18926	322	434	461	630.99	320	190	315
EABT18927	1	6	15.02	13	6	3	4
EABT18928	2	0	4	5	62	0	3
EABT18929	0	2	19	0	0	0	0
EABT1893	9	28	294	7	4	16	4
EABT18930	2	0	0	2	2	0	6.66
EABT18931	0	1	8	2	0	1	0
EABT18932	1	0	1	2	0	0	0
EABT18933	0	9	51.94	5.86	2	1	5
EABT18934	0	1	3	0	0	0	0
EABT18935	3	2	0	0	1	7	2
EABT18936	1	2	6	1	1	0	1
EABT18937	0	0	5	0	0	0	0
EABT18938	3219.97	6541.48	8355.75	11023.8	2563.84	2536.92	2316.3
EABT18939	15778.6	25198.1	3645.14	15245.3	13900.7	298	873
EABT1894	0	2	16	0	0	0	0
EABT18940	2180.58	3996.44	3046.26	9217.73	2025.01	1112.86	1444.53
EABT18941	0	1	7	0	0	0	0
EABT18942	6	1	2	0	1	0	1
EABT18943	0	1	0	0	5	0	0
EABT18944	0	1	3	1	0	0	0
EABT18945	0	0	6	0	0	2	1
EABT18946	4247.26	1787.47	23	1	14	1404.31	109
EABT18947	127	251.6	254.02	353	309	94	125.2

EABT18948	877.01	1390.97	940.5	3349	1367.06	1002.52	935.76
EABT18949	3	0	5	5	0	1	2
EABT1895	8	2	11	1	7	3	11
EABT18950	171	214	0	0	8	14	71
EABT18951	0	1	3	1	0	0	1
EABT18952	5243.75	10770.8	14591	1578	5752.89	506	2299.86
EABT18953	7	16	9	14	3	13	5
EABT18954	5	11	6	20	4	4	2
EABT18955	1	3	1	0	0	0	0
EABT18956	0	0	1	0	2	3	13
EABT18957	150	112	103	49	10	305	44
EABT18958	1	0	4	0	0	0	0
EABT18959	6	4	80	7	4	23	4
EABT1896	0	1	11	0	0	0	0
EABT18960	1	1	20	1	0	1	0
EABT18961	1857.46	3231.85	7022.99	7612.93	2347.39	1777.71	1464.23
EABT18962	2	6	30	15	5	4	8
EABT18963	1	3	29	3	0	0	1
EABT18964	3	7	4	17	5	0	6
EABT18965	14	40	8	108.44	34.02	0	2
EABT18966	1	11	14	14	4	10	6
EABT18967	66	53	28	19	53.67	0	7
EABT18968	0	4	6	1	1	0	4
EABT18969	0	0	26	0	0	0	0
EABT1897	0	0	11	0	0	0	1
EABT18970	141	401.07	684.97	4779.69	600.55	489	554.06
EABT18971	0	0	9	3	4	0	1
EABT18972	4	2	4	14	2	0	0
EABT18973	1	2	9	3	2	0	0
EABT18974	0	0	7	0	0	0	0
EABT18975	1	0	3	0	1	1	0
EABT18976	604.32	927.4	1231.94	1764.56	1036.21	826.49	802.43
EABT18977	172	286.84	281.09	548.63	395.02	192	111
EABT18978	0	3	3	157	2	0	0
EABT18979	8	12	103	23	7	0	0
EABT1898	0	0	0	4	3	0	1
EABT18980	1	1	2	6	1	0	0
EABT18981	26	41	9	2	5	122	135
EABT18982	0	1	6	3	0	0	0
EABT18983	6	14	95	81.97	1	1	1
EABT18984	13	27	20	13	10	9	10
EABT18985	2	4	25	6	0	1	2
EABT18986	0	0	0	0	0	15	4
EABT18987	0	6	1	8	0	0	0
EABT18988	683.99	791.93	1028.07	1081.89	804.15	990.28	275.41
EABT18989	3	18	7	22	7	2	7
EABT1899	1	3	6	2	12	1	3
EABT18990	6600.92	7020.74	6518.96	19540.7	6453.39	6125.19	5927.83
EABT18991	0	0	5	0	0	0	0
EABT18992	0	2	2	0	2	0	0
EABT18993	1	1	23	14	12	0	0

EABT18994	0	1	15	4	3	2	2
EABT18995	0	0	11	6	1	0	0
EABT18996	1	10	11	0	0	0	0
EABT18997	0	1	7	0	0	0	0
EABT18998	3	8	11	3	1	1	2
EABT18999	514.33	1066	954.74	1825.94	1534.08	417.34	934
EABT19	0	0	2	0	0	1	0
EABT190	0	5	8	3	1	1	0
EABT1900	3	1	2	4	0	0	0
EABT19000	0	0	13	3	0	0	0
EABT19001	2	1	6	1	1	0	0
EABT19002	0	3	6	1	0	1	0
EABT19003	1	8	5	35	7	0	0
EABT19004	3614.73	5529.58	5593.78	7771.8	4136.04	2727.1	4330.21
EABT19005	1130.21	1651.87	2255.04	2505.01	1210.67	815.95	969.99
EABT19006	2305.92	2923.2	3731.49	4022.9	2094.04	2068.18	1568.47
EABT19007	3	7	9	11	18	4	11
EABT19008	13	41	26	57	73	8	48
EABT19009	2096.06	2463	1426	2600.07	1628.57	1534.01	1834.09
EABT1901	596.85	984.33	829.85	4074.67	907.94	630.25	626.96
EABT19010	4	12	37	8	0	0	0
EABT19011	12	8	134	4	3	9	1
EABT19012	0	0	1	3	1	0	0
EABT19013	7215.99	3293	2	1	5744.26	2693.33	20639.1
EABT19014	92	231	897.28	252	88	81.93	111
EABT19015	0	0	6	0	0	3	0
EABT19016	42	47.01	12	172	77	15.95	49
EABT19017	6	23	30	5	7.01	39	16.01
EABT19018	2495.99	5012.2	2764.32	2769.49	849	742	324.55
EABT19019	0	2	1	4	0	0	1
EABT1902	25	35	9	7	4	1	4.05
EABT19020	13	13	13	3	1	7	1
EABT19021	35	36	46.97	14	7	30	13
EABT19022	0	3	0	9	0	0	0
EABT19023	0	2	15	2	0	1	1
EABT19024	1342.35	1601.97	1120	1888.98	1065.91	1167.23	1070
EABT19025	1	3	1	0	0	2	0
EABT19026	2	8	53	0	0	0	0
EABT19027	3	2	11	1	1	0	0
EABT19028	0	1	20	0	1	1	1
EABT19029	0	1	0	6	0	0	1
EABT1903	3	1	2	0	1	0	0
EABT19030	1	5	142	2	3	0	0
EABT19031	2	4	8	12	2	1	0
EABT19032	23937.6	26848.6	39021.8	4538.05	11431.2	19579.1	18562.9
EABT19033	51	95	25	12	38	18	139
EABT19034	0	1	4	2	0	0	6
EABT19035	0	0	13	1	0	0	0
EABT19036	27	33	15	129.47	1	6	9
EABT19037	1	4	2	1	4	0	2
EABT19038	0	0	10	0	0	0	0

EABT19039	0	0	0	6	1	0	0
EABT1904	1	5	9	0	2	2	1
EABT19040	0	0	4	0	0	0	0
EABT19041	2	7	42	17	2	0	0
EABT19042	7	6	43	24	15	15	1
EABT19043	0	0	14	1	0	0	0
EABT19044	0	0	3	6	2	1	0
EABT19045	375	0	2	0	0	523	0
EABT19046	0	5	2	0	0	0	0
EABT19047	0	0	7	4	6	0	2
EABT19048	0	0	1	0	6	0	0
EABT19049	2979.66	3254.86	5023.01	3364.2	1657.1	3038.42	2119.84
EABT1905	0	3	1	1	0	0	0
EABT19050	3	4	2	4	0	7	2
EABT19051	2	1	4	0	1	4	5
EABT19052	933.92	2793.89	336.14	764.05	369.57	573.37	477.39
EABT19053	2494.82	3238.29	3458.27	3319.07	1870.22	1343.09	1270.81
EABT19054	38	132.63	224.96	345	36	45	35
EABT19055	0	0	4	0	0	1	0
EABT19056	2	6	5	14	11	0	3
EABT19057	1027.45	1509.13	849.13	1380.69	1030.8	410.68	793.33
EABT19058	0	0	0	2	0	2	0
EABT19059	12	52.01	67	127	114	7	4
EABT1906	1	0	14	0	0	0	0
EABT19060	7	7	17	4	0	2	1
EABT19061	0	2	71	0	0	1	1
EABT19062	0	0	0	0	1	2	2
EABT19063	3705.61	6662.2	9799.93	7430.14	3793.11	3728.06	2905.5
EABT19064	10	21	2	8.32	4	4	8
EABT19065	0	0	5	0	0	0	3
EABT19066	29	345.9	43	186	74	8	41
EABT19067	3	6	3	0	0	1	0
EABT19068	0	0	8	0	0	0	0
EABT19069	0	0	26	4	1	0	0
EABT1907	11	29	19	26	7	7	6
EABT19070	33	152	584.01	3171.2	36	104	18
EABT19071	1276.02	1919.64	3299.38	3490.86	1998.07	1150	1030
EABT19072	0	10	3	1	2	4	3
EABT19073	1	0	44	0	0	0	1
EABT19074	384.1	397.49	572.45	579.46	334.23	275.63	279
EABT19075	1	7	7	3	3	0	2
EABT19076	1	0	1	2	0	1	3
EABT19077	0	0	0	1	2	3	3
EABT19078	2	2	2	1	0	0	1
EABT19079	0	0	4	0	0	0	0
EABT1908	70	108	105	215	181	103	268
EABT19080	708	1338	802.28	2916.96	722.57	482.19	755.49
EABT19081	20325.5	27446.7	12372.8	22691.3	11180.7	17877.3	14621.9
EABT19082	2	4	4	1	0	1	4
EABT19083	1	4	5	13	1	5	7
EABT19084	0	0	1	1	0	0	0

EABT19085	1	0	1	6	2	0	0
EABT19086	5	4	17	8	4	2	3
EABT19087	4836.1	8140.14	13252.1	2126.1	4628.9	3481.83	5641
EABT19088	1	4	8	5	1	0	0
EABT19089	0	0	0	1	1	0	0
EABT1909	237	294	252.97	596.88	434.01	229.01	262.57
EABT19090	0	0	15	254.01	1	0	0
EABT19091	0	2	7	1	0	3	0
EABT19092	3	6	12	26	1	0	0
EABT19093	1	0	9	0	0	0	0
EABT19094	0	0	4	11	1	0	0
EABT19095	127	444.8	451	624.98	778.52	48	320.82
EABT19096	2459.13	2211.03	1096.5	3113.21	1748.09	697.82	2440.9
EABT19097	1	0	0	4	2	0	2
EABT19098	0	4	3	1	0	0	1
EABT19099	1826.02	2329.2	1402.1	1516.27	1076.78	1894.29	1843.69
EABT191	4093.07	9645.88	4685	16646.8	5066.5	3902.54	6937.77
EABT1910	1	2	3	0	0	1	0
EABT19100	28780.7	20505.9	19303.6	22575.9	12017.3	15977.6	6854.55
EABT19101	0	0	7	4	0	0	0
EABT19102	0	3	25	1	0	2	1
EABT19103	1746.71	1581.91	923.46	1633.7	1407.22	836.59	867.36
EABT19104	0	0	1	1	4	0	0
EABT19105	1	9	3	4	2	0	0
EABT19106	8	1	1	0	0	11	0
EABT19107	1	1	2	2	2	1	1
EABT19108	20	29	152	47.06	15	19	24
EABT19109	592.55	862.02	457.02	2301.21	402	445	313
EABT1911	3944.95	5141.47	3097.16	3495.39	2442.15	2405	2705
EABT19110	9	7	7	106	4	7	26
EABT19111	2	7	0	0	0	42	0
EABT19112	1	2	9	3	0	3	0
EABT19113	106.71	88	186.98	446.09	167	15	27
EABT19114	4	14	8.97	15	10	9	6
EABT19115	16	0	1	0	0	8	1
EABT19116	1	0	2	0	0	3	2
EABT19117	0	3	2	4	6	0	0
EABT19118	3	1	7	3	0	1	4
EABT19119	1	0	7	1	0	0	1
EABT1912	9	26	21	13	9	1	4
EABT19120	3	5	10	7	3	1	1
EABT19121	0	3	31.1	2	1	6	1
EABT19122	8	11	74	7	2	3	3
EABT19123	0	0	0	0	2	0	1
EABT19124	3	4	2	1	0	1	0
EABT19125	0	0	0	0	4	1	16
EABT19126	6.99	7	13	23.12	1	2	4
EABT19127	1	0	9	0	0	0	0
EABT19128	0	0	8	3	1	0	1
EABT19129	332.02	1144	659.59	1462.03	3292	40	376
EABT1913	0	1	9	0	0	0	0

EABT19130	8	42	62	105	119	2	1
EABT19131	1061.88	1820.94	2630.36	3052.39	2116.48	1326.1	1223.31
EABT19132	1	2	1	1	0	1	5
EABT19133	4	8	8	9	1	5	1
EABT19134	2	8	6	9.19	78	1	2
EABT19135	29001.6	22047.8	181	5881.93	2713.93	15	345
EABT19136	0	2	2	0	0	0	2
EABT19137	0	2	1	1	0	0	1
EABT19138	0	38	17	15	2682.01	3	12
EABT19139	188	252	223	509	117	66	96
EABT1914	4	20	0	0	0	3	2
EABT19140	3	1	3	10	7	3	16
EABT19141	13526.6	16419.5	10580.8	17935.7	8664.11	8559	10057.6
EABT19142	0	0	7	0	0	1	0
EABT19143	1	1	5	2	0	0	0
EABT19144	9	8	20	3	3	0	2
EABT19145	0	0	6	0	0	0	0
EABT19146	0	1	3	0	0	1	5
EABT19147	0	2	5	0	0	0	0
EABT19148	2	3	9	12	2	3	1
EABT19149	2	6	31	3	2	4	4
EABT1915	142	267	275	461	334	120	151.36
EABT19150	88294.1	242204	114398	71407	138657	6293.44	34010
EABT19151	1	6	65	15	0	2	0
EABT19152	3	3	9	3	4	1	1
EABT19153	0	0	15	0	0	3	1
EABT19154	3	4	1	12	1	1	0
EABT19155	6	12	4	14	1	2	2
EABT19156	1795.94	2816	3606.39	5289.21	2284.04	1511.65	1515.96
EABT19157	272.97	371.88	429.51	593	404	207	227
EABT19158	2	4	1	0	5	0	1
EABT19159	0	5	4	0	0	0	0
EABT1916	10	7	29.37	14	4	6	1
EABT19160	2	6	11	15	5	3	2
EABT19161	1	2	12	5	7	0	0
EABT19162	1076.52	2710.85	1193.03	1274.83	1049	575.89	365
EABT19163	1	3	20	1	3	1	0
EABT19164	1373.77	1885.55	2588.56	2685.18	976.99	1242.33	1260.45
EABT19165	1128.75	2537	1055.34	348	437	901.99	233.31
EABT19166	128600	23028.8	61	8	39341.9	90015	210787
EABT19167	1	5	20	1	1	1	1
EABT19168	8	7	0	0	1	3	7
EABT19169	0	0	7	1	0	0	0
EABT1917	1	4	0	9	7	0	5
EABT19170	2	3	5	5	1	1	0
EABT19171	401	670	396.05	259	199	2	9
EABT19172	6	20	3	6	10	1	1
EABT19173	22	27	187	302	5	0	1
EABT19174	5	6	30	15	24	0	11
EABT19175	1	1	2	2	0	1	0
EABT19176	0	0	0	1	0	0	0

EABT19177	0	2	23	1	0	0	0
EABT19178	2	3	0	0	3	1	7
EABT19179	0	2	11	4	0	0	0
EABT1918	13	8	1	6	4	0	0
EABT19180	0	1	0	0	0	0	0
EABT19181	0	3	9	0	0	0	0
EABT19182	0	0	8	1	0	0	0
EABT19183	4	3	51	3	0	1	2
EABT19184	5	9	13	8	4	1	4
EABT19185	3	3	1	1	0	0	0
EABT19186	2	9	47	27	3	10	7
EABT19187	2	1	6	1	0	0	0
EABT19188	115	54	0	1	5	25	30
EABT19189	1	1	5	8	2	1	1
EABT1919	5	3	0	0	0	1	1
EABT19190	125704	30524.9	1503.93	2662.13	3420.5	88132.4	8511.32
EABT19191	0	0	0	1	3	0	3
EABT19192	0	0	9	2	0	0	0
EABT19193	0	0	7	0	0	0	0
EABT19194	11	7	0	0	1	1	1
EABT19195	0	0	20	0	0	0	1
EABT19196	8	24	67	79	4	0	1
EABT19197	71.98	100.13	7	3	6	36	37
EABT19198	2	4	65	0	1	0	0
EABT19199	12	30	40	33	7	4	8
EABT192	0	3	4	6	0	0	1
EABT1920	4	19	15.98	328.06	56.97	3	8.99
EABT19200	607	845	682	1412.66	734.01	379	498
EABT19201	0	0	9	1	0	0	0
EABT19202	1	3	3	3	1	0	1
EABT19203	4	6	29	2	2	1	5
EABT19204	7	10	0	0	0	0	0
EABT19205	1527.93	1986.3	1415.82	3285.47	1427.7	1195.84	770.85
EABT19206	6919.47	8367.87	8991.56	13731.6	6153.13	5373.64	5521.42
EABT19207	0	0	22	0	0	0	0
EABT19208	1	0	23	27	2	1	2
EABT19209	948.78	1657.87	2239.47	4097.99	673.19	553.96	631.2
EABT1921	2	3	1	0	2	0	0
EABT19210	0	1	5	2	2	0	0
EABT19211	3	0	10	5	0	0	0
EABT19212	0	4	19	2	3	1	2
EABT19213	0	0	12	1	0	0	0
EABT19214	6437.47	8602.34	6270.44	12622.7	762	2638.36	996.71
EABT19215	1	1	8	0	0	0	0
EABT19216	1	1	3	0	1	0	1
EABT19217	1048.47	1871.08	1294.63	5555.14	2060.15	618.12	604.08
EABT19218	0	0	5	0	0	0	0
EABT19219	15	63	43	0	19	0	4
EABT1922	1	4	2	0	0	0	0
EABT19220	1	0	18	0	0	0	1
EABT19221	0	1	5	0	0	0	0

EABT19222	1	1	4	6	0	0	0
EABT19223	37468.3	12397.1	13	5	7723.2	58126	45251.3
EABT19224	0	0	4.13	1	0	0	0
EABT19225	15	38	8	0	14	2	23
EABT19226	1	2	2	1	0	0	0
EABT19227	14	23	54	2	4	15	10
EABT19228	2	0	1	1	0	1.96	2
EABT19229	1175	2492.06	1601.2	5319.52	2450.03	1072.1	2214.59
EABT1923	28378.2	29801.7	6370.6	14218.6	2833.16	3243.77	1687.94
EABT19230	2	4.98	61	18	17	3	6
EABT19231	0	1	1	2	0	0	0
EABT19232	0	3	0	8	0	0	0
EABT19233	4	3	7	6	1	2	4
EABT19234	309.91	500.84	545.26	871.06	282.99	155.97	334
EABT19235	0	1	8	1	0	0	1
EABT19236	609	896.48	586	1860.47	843.97	524	580
EABT19237	23	20	280.47	14	12.99	14	4
EABT19238	459.43	1206.99	371.79	739.72	203.1	905.24	590.67
EABT19239	1	4	1	0	1	0	0
EABT1924	3	3	6	0	0	2	0
EABT19240	1	4	18	1	0	3	1
EABT19241	1	6	2	4	1	1	2
EABT19242	2	7	9	16	2	18	1
EABT19243	0	2	4	0	1	2	2
EABT19244	1	5	0	0	0	0	1
EABT19245	0	3	2	3	0	0	0
EABT19246	16	32	22	71	38	13.92	28
EABT19247	2264.23	4265.37	2458.59	1613.35	752.69	858.56	685.56
EABT19248	7	4	3	3	3	1	1
EABT19249	5841.94	5495.84	3519.69	4858.54	3307	2451.16	2699.03
EABT1925	0	1	5	0	0	0	0
EABT19250	10	21	70	70	5	8	9
EABT19251	2	1	7	1	0	0	0
EABT19252	2	16	3	0	4	4	6
EABT19253	1	0	8	0	0	1	0
EABT19254	0	0	2	4	0	0	1
EABT19255	1	1	1	3	1	0	3
EABT19256	5919.67	5732.1	3081.06	3898.96	6449.21	3729.99	10017
EABT19257	1	0	19	0	0	0	0
EABT19258	1	7	14	0	5	2	1
EABT19259	21	157	70	175.43	2471.62	17	265.19
EABT1926	1885.7	2860.07	1998.97	4913.45	1488.65	1068.05	1243.43
EABT19260	2	0	12	1	0	5	1
EABT19261	227.07	358.99	181	625.28	104	74	130
EABT19262	13	5	1	0	1	17	7
EABT19263	3	2	2	1	0	0	0
EABT19264	2	1	0	0	0	0	0
EABT19265	0	0	1	1	8	1	15
EABT19266	1	16	7	11	2	1	9
EABT19267	3	14	30	5	6	5	8
EABT19268	1	8	9	7	1	0	0

EABT19269	0	0	6	0	4	0	2
EABT1927	0	0	2	1	0	0	0
EABT19270	0	1	4	0	0	0	0
EABT19271	3	0	7	1	3	0	2
EABT19272	0	5	3	0	0	0	0
EABT19273	0	1	5	0	0	0	0
EABT19274	0	0	0	3	11	0	3
EABT19275	1367	2619.25	1502.55	222	2528.96	789.57	2770.21
EABT19276	0	2	39	3	2	0	0
EABT19277	1	1	0	0	0	1	0
EABT19278	0	0	2	1	1	0	1
EABT19279	1	0	2	0	1	0	0
EABT1928	14	17	14	1	2	0	0
EABT19280	461	759	537	1239.22	429	468	537
EABT19281	1	2	3	3	6	0	2
EABT19282	43.04	10.77	0	0	2046	55.1	7462.93
EABT19283	4	6	4	0	1	4	4
EABT19284	0	1	1	1	0	5	2
EABT19285	5	14.99	0	9	0	0	0
EABT19286	1	1	10	1	0	0	0
EABT19287	0	2	0	253	0	33	40
EABT19288	0	0	0	0	0	0	0
EABT19289	1	2	11	0	1	0	0
EABT1929	19	32	43	1	60	15	5
EABT19290	2	1	6	1	0	0	0
EABT19291	6	12	9	3	0	23	22
EABT19292	0	2	78	1	0	0	0
EABT19293	0	6	9	7	1	0	0
EABT19294	3	4	3	1	3	0	0
EABT19295	2	6	11	2	0	1	0
EABT19296	6	14	28	15	2	3	3
EABT19297	2	7	26	23	6	0	6
EABT19298	67	119	195	684.05	517.58	40	45.01
EABT19299	1	2	0	4	0	1	4
EABT193	6	5	41	265.35	18950.4	6	111
EABT1930	0	2	5	3	1	0	2
EABT19300	839.61	1020.6	678.51	1209.99	769.81	741.29	778.54
EABT19301	73	23	1	3	0	137.03	0
EABT19302	0	0	6	0	0	0	0
EABT19303	1458.2	2128.21	744.33	2009.5	1925.56	1171.47	1381.62
EABT19304	1	3	4	0	0	0	0
EABT19305	0	1	7	0	0	0	0
EABT19306	1	2	8	1	1	1	2
EABT19307	0	0	13	0	0	0	0
EABT19308	0	0	15	1	0	0	0
EABT19309	0	0	1	8	0	0	0
EABT1931	2	0	16	1	0	0	0
EABT19310	1013.08	1602.89	1719.01	3631.93	1361.6	381.63	736.9
EABT19311	1	5	18	5	0	2	3
EABT19312	10	33	38	104	37	10	12
EABT19313	0	1	4	0	1	0	0

EABT19314	0	3	9	1	0	0	0
EABT19315	5	2	4	1	2	4	15
EABT19316	5	3	0	0	0	17	6
EABT19317	54	50	51	113	49	37	71
EABT19318	0	0	0	0	0	0	28
EABT19319	78	45	47	176	17	131	93.25
EABT1932	17	29	554	124	46	69	176
EABT19320	1	2	11	3	1	3	0
EABT19321	240	463	469.66	1534.18	1705.71	27	168.15
EABT19322	1650.99	2998.74	1227.72	4855.06	950.96	691	815.05
EABT19323	21	45	248.73	271	58	1	17
EABT19324	8.5	14	24	29	9	1	5
EABT19325	0	1	2	9	1	0	1
EABT19326	746.68	756	462.99	405.06	746.01	1131	616
EABT19327	116	179	58	23	51	187	61
EABT19328	3	3	6	15	0	0	1
EABT19329	0	2	4	2	1	0	0
EABT1933	0	1	6	2	0	33	0
EABT19330	0	1	19	26	0	0	0
EABT19331	193.07	143	137.91	108	46	5	12
EABT19332	2	4	12	7	0	0	1
EABT19333	37	0	1	0	0	6	0
EABT19334	0	0	1	0	50	0	0
EABT19335	0	1	1	0	0	0	0
EABT19336	3	8	3	5	1	13	3
EABT19337	0	1	16	1	0	0	0
EABT19338	6	2	32	6	1	2	2
EABT19339	638.61	583.38	89	635.99	101	0	261.99
EABT1934	2	3	4	2	0	1	1
EABT19340	0	2	19	1	1	0	1
EABT19341	0	0	13	3	3	0	0
EABT19342	208	3	7	2	0	471.98	0
EABT19343	1	0	3	1	0	0	0
EABT19344	1	0	5	1	0	1	0
EABT19345	3936.58	6196.81	6842.09	6772.58	4388.17	3799.95	3762.17
EABT19346	10	11	99	13	5	6	4
EABT19347	938	949.57	708.83	1241.07	655	1136	798.76
EABT19348	892.16	653.52	3151.28	2105.17	2961.46	1129.07	2241.62
EABT19349	1	0	10	4	1	0	0
EABT1935	2	2	36	6	1	1	1
EABT19350	0	0	7	3	0	0	0
EABT19351	0	0	3	0	0	1	0
EABT19352	49	20.08	131	4	54	124	61
EABT19353	344	557.29	641.67	336.83	439.07	260.98	462.81
EABT19354	2	7	7	25	52	1	0
EABT19355	2	1	6	0	2	0	0
EABT19356	0	6	3	8	0	2	1
EABT19357	0	1	54	2	2	0	0
EABT19358	2	4	0	0	0	2	2
EABT19359	1	1	2	7	1	1	6
EABT1936	3	11.99	0	0	0	6	10

EABT19360	0	1	2	0	0	0	1
EABT19361	0	0	6	0	0	0	1
EABT19362	36	36	53	45	28	6	20
EABT19363	0	0	0	0	0	0	0
EABT19364	0	2	5	1	0	0	0
EABT19365	1	0	36	1	3	0	2
EABT19366	5	10	9	7	3	10	3
EABT19367	0	0	18	0	0	0	0
EABT19368	1	24	21	106	104	2	11
EABT19369	2	3	4	2	2	1	0
EABT1937	888.56	1258.6	1456.84	1390.42	1039.97	682.85	724.92
EABT19370	0	1	4	0	0	0	0
EABT19371	1	0	7	2	0	0	0
EABT19372	0	1	9	0	1	2	0
EABT19373	677.04	964.74	513.91	1024	747	540	509.02
EABT19374	0	1	2	1	0	0	0
EABT19375	2	4	18	3	1	2	1
EABT19376	12	33	19	116	41	11.98	26
EABT19377	6	4	13	5	4	3	1
EABT19378	5	2	6.01	9	4	0	1
EABT19379	1	1	57	1	1	0	0
EABT1938	41.99	39	19.6	37.35	16.99	315.26	189.8
EABT19380	1926.15	2566.13	1635.81	4961.63	1714.64	1266.27	1140.51
EABT19381	1670.41	6979	987.08	808.59	2627.2	7345.9	1291.18
EABT19382	0	1	2	8	0	0	1
EABT19383	0	0	3	11	2	0	0
EABT19384	2	10	2	5	6	0	0
EABT19385	10275.8	10039.3	6658.28	8376.01	7161.28	10110.9	7443.7
EABT19386	11	41	10	5	7.55	6	10
EABT19387	5	4	10	0	0	0	1
EABT19388	2	7	1	1	0	0	0
EABT19389	2	2	3	0	1	9	0
EABT1939	1	3	13	1	1	1	0
EABT19390	0	1	10	4	2	2	0
EABT19391	1613.61	3585.09	200.28	182.35	870.53	9257.22	6528.32
EABT19392	0	2	13	0	0	0	0
EABT19393	5	9	17	36	8	4	8
EABT19394	9	20	29	32	3	16	10
EABT19395	5	8	11	34	11	1	12
EABT19396	1	1	1	8	0	1	0
EABT19397	7	66	23	61	8	0	2
EABT19398	1	3	1	0	0	0	3
EABT19399	0	0	3	0	0	0	0
EABT194	0	0	8	0	0	0	1
EABT1940	0	0	7	0	1	0	0
EABT19400	0	0	7	0	0	0	1
EABT19401	0	0	7	0	1	1	2
EABT19402	15	4	0	0	1	3	57
EABT19403	83.99	174	18	29	3964.81	21	107.99
EABT19404	2	2	7	1	0	5	2
EABT19405	4	3	2	7	3	3	4

EABT19406	0	0	6	0	0	0	0
EABT19407	0	0	6	1	1	0	0
EABT19408	9	3	0	0	0	0	0
EABT19409	0	1	33	3	1	0	0
EABT1941	3	34	54	61	10	2	4
EABT19410	0	0	0	0	0	0	0
EABT19411	0	1	3	5	0	0	1
EABT19412	0	0	4	3	0	0	0
EABT19413	71.8	1639.05	805.39	996	631.68	14	95
EABT19414	0	0	3	2	0	0	0
EABT19415	12	14	22	2	3	0	5
EABT19416	1	1	10	0	0	0	0
EABT19417	2	10	5	15	2	3	0
EABT19418	1	27	21	4	0	2	2
EABT19419	0	0	23	3	1	0	0
EABT1942	1985.17	2143.18	621.99	1222	1206.39	10	224.14
EABT19420	0	0	15	4	1	1	0
EABT19421	0	0	8	1	0	0	1
EABT19422	0	0	38	1	0	0	0
EABT19423	1734.2	2905.72	3664.99	7042	1675.71	1922.43	1619.65
EABT19424	85	109.95	95.02	525	157	61	69.03
EABT19425	3304.88	1236.86	80.64	40	543	108	150
EABT19426	1	2	7	1	1	0	1
EABT19427	0	0	9	1	0	0	0
EABT19428	1445.32	2488.32	1408.13	2916.16	1796.86	1313.34	1882
EABT19429	2758.65	4563.91	1448.9	1917.96	2077.6	3082.39	3958.45
EABT1943	13	7	9	12.98	3	1	1
EABT19430	0	6	8	3	4	0	0
EABT19431	1827.6	2039.6	2019.73	3519.48	1307.88	1175	862
EABT19432	10	13	25	23	2	6	6
EABT19433	11428	6268.05	76	83	2066.35	893	5486.73
EABT19434	0	4	2	8	0	0	0
EABT19435	559	2685	3847	3118.02	7726.81	120	784
EABT19436	2835.11	3489.43	1239.85	62	1040.48	1343	1532
EABT19437	227	216.01	82.95	341	63	232	194
EABT19438	1	35	3	0	0	2	0
EABT19439	0	1	72	0	0	1	0
EABT1944	221.78	296.08	444.41	61	151.18	290.01	905.08
EABT19440	11	21	20	34	6	4	5
EABT19441	62	1522.4	41	2678.13	17	21	14
EABT19442	1	6	2	9	2	0	0
EABT19443	2	2	3	2	0	0	1
EABT19444	1	0	6	0	0	0	0
EABT19445	1	3	1	10	0	0	1
EABT19446	0	8	8	0	0	0	0
EABT19447	0	1	3	0	0	0	0
EABT19448	3	4	5	0	0	2	3
EABT19449	0	0	11	2	1	0	3
EABT1945	4	12	21	6	8	16	1.4
EABT19450	25474.3	11917.9	93.32	38	732.64	13922.1	4989.76
EABT19451	0	1	5	2	2	1	0

EABT19452	2	1	32	1	0	0	0
EABT19453	5130.95	7524.03	8422.82	9317.03	3197.83	4723.41	4416.32
EABT19454	2	1	76	0	2	0	0
EABT19455	0	0	1	4	2	0	0
EABT19456	69	82	0	0	4	16	25
EABT19457	4	22	14	10	2	0	0
EABT19458	2	0	4	0	0	0	0
EABT19459	3	6	0	0	1	38	7
EABT1946	0	5	20	4	0	6	0
EABT19460	1	4	20	11	0	0	0
EABT19461	0	6	6	0	1	0	0
EABT19462	8	10	71	19	3	11	10
EABT19463	3227.01	8504.66	11717	4278.01	5658.95	1365	3000.75
EABT19464	0	0	6	0	3	0	0
EABT19465	5	2	17	3	0	0	1
EABT19466	4	3	1	3	0	0	0
EABT19467	693.64	1099	1693.78	2369.97	1385.13	851	860.65
EABT19468	103.01	172.01	203	325.17	145	75	131.99
EABT19469	2	9	5	0	0	0	1
EABT1947	0	0	1	5	1	0	0
EABT19470	0	1	0	0	0	0	0
EABT19471	2	10	29	253	490	1	8
EABT19472	3	3	3	0	1	0	0
EABT19473	2	2	1	2	1	2	0
EABT19474	3	10	2	24	1	3	6
EABT19475	1	1	4	0	0	0	0
EABT19476	8137.35	8791	4827.13	5731.89	23813.2	6022.98	5494.13
EABT19477	186	325.06	985.18	1132.3	258	110	147
EABT19478	6	4	2	1	7	1	1
EABT19479	0	4	8	0	2	0	0
EABT1948	5	9	38	4	4	4	2
EABT19480	2980.53	4230.75	4162.88	9278.74	2957.11	3238.56	3595.94
EABT19481	0	0	11	1	0	0	1
EABT19482	312	1048.39	197	36	3072.78	4	52
EABT19483	0	4	1	1	2	3	2
EABT19484	6866.84	2817.29	28	55	7293.66	1925.73	39909.3
EABT19485	0	0	0	0	0	0	0
EABT19486	0	0	13	7	0	0	0
EABT19487	1	3	11	3	1	4	2
EABT19488	1	0	6	0	2	0	1
EABT19489	0	4	2	7	2	0	0
EABT1949	104.98	171	130.35	133	457	13	86.07
EABT19490	0	1	3	0	0	0	0
EABT19491	15	36	45	5	1	3	1
EABT19492	1964.53	2124.01	1898	1183.48	1229.58	1898	805.82
EABT19493	4152.72	7189.48	6409.39	11843.3	4474.02	5287.22	4362.41
EABT19494	687.91	824.27	694.01	1111.46	620.98	474.56	505.38
EABT19495	6.02	40	146	154	4	0	1
EABT19496	0	0	5	0	0	0	0
EABT19497	0	0	5	0	0	0	0
EABT19498	0	0	5	1	0	0	0

EABT19499	1	3	8	4	0	1	1
EABT195	5	12	5	9	1	3	2
EABT1950	0	0	16	8	5	0	1
EABT19500	1	3	45	19	2	4	4
EABT19501	1	1	2	20	1	0	0
EABT19502	11	23	122	31	2	0	0
EABT19503	0	0	2	0	0	1	0
EABT19504	0	8	0	0	2	3	4
EABT19505	3	2	2	0	0	4	5.13
EABT19506	1	0	10	0	0	1	0
EABT19507	4	3	1	2	2	5	0
EABT19508	0	3	13	0	0	1	0
EABT19509	0	0	0	0	8	0	3
EABT1951	1	2	5	1	1	1	2
EABT19510	0	2	0	13	0	0	0
EABT19511	1	6	3	0	0	1	0
EABT19512	0	3	7	2	0	2	2
EABT19513	1	0	10	3	15	0	0
EABT19514	12	11	61.49	40	20	9	3
EABT19515	9	28	133	33	9	8	9
EABT19516	1	10	4	14	1	1	5
EABT19517	0	3	8	3	1	0	0
EABT19518	0	2	3	18	8	0	0
EABT19519	3	1	3	2	1	1	4
EABT1952	0	1	6	1	1	2	2
EABT19520	4	10	23.86	23.05	4	0	0
EABT19521	1958.01	2527.84	2846.62	2824.27	1748.77	1064.02	1192
EABT19522	1	10	13	4	1	0	3
EABT19523	11	17	27	4	3	6	0
EABT19524	1496.04	2281	1239.97	1872.05	1198.96	1786	1834.01
EABT19525	253.54	370.72	67.98	4	80	3	33
EABT19526	4262.71	9517.02	6792.74	32283.1	3832.34	650.57	1358.64
EABT19527	0	2	5	2	3	0	1
EABT19528	1192.02	1642.45	2262.12	3270.14	1422.41	848.01	1039.21
EABT19529	4	4	15	4	4	1	0
EABT1953	7	29	1	11	1	1	0
EABT19530	412.99	408	271	692.26	369	242	255.01
EABT19531	0	0	4	5	1	0	0
EABT19532	12	29	11	1	5	23	4
EABT19533	0	1	6	5	0	0	0
EABT19534	45	85	68.73	240	200.02	13	147.57
EABT19535	1	8	1	5	6	0	2
EABT19536	0	0	8	0	0	0	0
EABT19537	0	5	5	3	1	0	0
EABT19538	8	14	13	8	1	3	3
EABT19539	4	1	5	1	0	3	1
EABT1954	9	11	0	1	8	33	83
EABT19540	1	8	29	16	0	5	5
EABT19541	6	21	25	59	34	10	14
EABT19542	174.14	448.97	78	1242.33	2375.74	32	64
EABT19543	0	1	10	7	8	0	1

EABT19544	10442.4	11472	9034.02	8519.56	9654.56	11762.5	11149.3
EABT19545	2	4	26	4	0	2	0
EABT19546	3	0	0	0	0	3	0
EABT19547	2	5	1	0	0	0	0
EABT19548	6	6	7	10	7.98	1	5
EABT19549	12	33.01	41	167	33	11	16
EABT1955	9309.15	12788.4	18307.6	10757.2	6145.96	10722.5	8072.91
EABT19550	1	1	5	1	1	0	0
EABT19551	20	14.05	7	115	10	0	3
EABT19552	0	0	6	1	1	0	1
EABT19553	1	1	11	4	0	2	0
EABT19554	17	21	283.59	43	15	9	8
EABT19555	0	0	2	0	0	0	2
EABT19556	698	931.98	758	1288.48	712	513	426
EABT19557	4	1	35	9	4	1	0
EABT19558	4	3	54	8	1	5	0
EABT19559	1	2	6	0	0	1	0
EABT1956	1	6	52	2	1	6	0
EABT19560	1548.86	2117.45	289.27	898.89	1023.15	2003.23	2745.65
EABT19561	4	6	0	0	0	5	7
EABT19562	2	5	10	1	1	3	2
EABT19563	0	0	11	1	1	0	0
EABT19564	834	4717.97	313	979	18685.2	500	6730.11
EABT19565	1	2	2	25	2	1	7
EABT19566	0	1	1	39	20	0	5
EABT19567	0	5	10	7	0	0	2
EABT19568	0	1	227	3	1	1	0
EABT19569	0	1	10	1	0	0	0
EABT1957	2	1	8	3	17	0	0
EABT19570	15	8	1	0	2	2	12
EABT19571	4047.36	6251.25	5344.43	5636.59	4532.12	2636.26	2936.07
EABT19572	2	5	3	0	0	2	0
EABT19573	0	3	8	222	406	0	1
EABT19574	2181.72	3578.24	2061.57	6899.54	3186.94	3335.77	3637.05
EABT19575	11	17	64.06	19.69	10	11	4
EABT19576	5641.52	4483.01	2101.38	3477.43	3332.36	1471	1797.89
EABT19577	0	3	7	2	0	0	0
EABT19578	1	4	4	38	2	0	2
EABT19579	1	2	18	5	1	0	0
EABT1958	0	1	6	1	0	0	0
EABT19580	0	0	3	5	0	0	0
EABT19581	1410.24	2183.52	1589.17	3473.83	1201.25	1279.92	1200.31
EABT19582	1189.02	1753.54	1895	2157.1	1343.05	948.97	1024
EABT19583	111214	38753.7	8225.45	4	22	23	0
EABT19584	36.3	45.73	45	5	14	5	27
EABT19585	224	213	285.84	383	157	31.12	24
EABT19586	115	214	18	11	327	222	111
EABT19587	11	44	36	119	10	1	4
EABT19588	1	4	6	1	0	0	0
EABT19589	16	36	52	64	15	46	32
EABT1959	0	0	11	1	1	0	2

EABT19590	1	0	27	0	1	0	0
EABT19591	0	1	1	0	1	0	0
EABT19592	1	2	43	6	0	0	1
EABT19593	0	0	0	0	0	0	4
EABT19594	13	23	30	35	9	5	15
EABT19595	0	11	2	1	4	0	0
EABT19596	1	3	3	2	0	0	0
EABT19597	4	7	5	7	3	0	2
EABT19598	0	1	4	1	0	0	0
EABT19599	3	1	33	0	0	1	0
EABT196	4	13	71	3	4	2	2
EABT1960	3	3	23.01	1	0	1	1
EABT19600	3	0	33	16	1	2	1
EABT19601	0	0	3	0	0	0	0
EABT19602	0	3	2	0	1	1	0
EABT19603	5689.62	10357.5	5776.81	17059.7	4616.11	2874.23	2840.03
EABT19604	0	0	2	1	0	0	0
EABT19605	0	6	4	1	0	2	1
EABT19606	1	3	2	1	0	2	2
EABT19607	580.11	1142.84	1233.28	1317.12	510.04	524.82	392.95
EABT19608	4	1	38	1	2	0	0
EABT19609	2898.05	11685.1	7041.32	8624.87	9572.6	3339.17	6964.17
EABT1961	0	1	3	0	1	0	0
EABT19610	802.61	1323.27	2061.63	3949.69	1259.95	802.92	891.97
EABT19611	0	0	19	0	2	1	2
EABT19612	2	2	7	0	1	1	5
EABT19613	126	144	0	0	7	75	71.9
EABT19614	12	0	1	0	0	19	0
EABT19615	1954	4882.38	2686.04	4481.25	1076.82	4748.56	1216.35
EABT19616	3	17	3.24	5	6.98	2	10
EABT19617	166	215	71	119	63	79	52
EABT19618	0	2	13	0	1	0	0
EABT19619	3730.33	5191.57	4307.63	3192.74	2180.87	5174.49	4099.52
EABT1962	1	0	6	0	0	0	0
EABT19620	14	34	38	657.99	11	3	11
EABT19621	0	2	11	4	0	2	1
EABT19622	6	2	0	0	0	6	0
EABT19623	0	0	14	1	1	0	0
EABT19624	1	7	24	25	2	0	1
EABT19625	0	0	4	1	0	0	0
EABT19626	0	2	13	2	6	1	0
EABT19627	0	3	6	1	2	2	0
EABT19628	0	2	0	1	1	0	0
EABT19629	1	8	11	0	0	0	0
EABT1963	0	4	28	3	0	3	3
EABT19630	0	0	31	0	0	0	0
EABT19631	633.12	1020.18	716.76	827.7	820.26	1166.17	1252.95
EABT19632	488.94	719.85	483.76	1245.79	727.24	524.17	818.38
EABT19633	103	136	88	55	27	182	83
EABT19634	0	0	1	2	0	0	0
EABT19635	98876.4	41578.7	453.16	77	92.05	34376.3	741

EABT19636	0	0	1	1	0	0	0
EABT19637	17563.8	24049.2	7317.15	6741.02	9140.91	3979.63	16142.7
EABT19638	18	33	41	9	12	1	2
EABT19639	1964.97	2456.18	1865.42	2312.02	1708.47	1905.79	1640.08
EABT1964	9	13	142	4	9	14	12
EABT19640	0	0	0	0	4	0	0
EABT19641	0	0	5	6	1	0	0
EABT19642	1	3	5	2	0	1	1
EABT19643	0	4	1	1	0	2	0
EABT19644	0	2	0	2	2	0	0
EABT19645	1	2	11	8	0	0	1
EABT19646	18	15.02	8	13	3	1	2
EABT19647	1	0	19	1	0	0	0
EABT19648	123	254	800.22	322	183	80	171
EABT19649	0	1	4	1	0	0	1
EABT1965	27	8	5	0	0	44	0
EABT19650	1	1	8	2	0	0	1
EABT19651	1	0	2	2	2	1	2
EABT19652	0	0	20	0	0	0	0
EABT19653	0	5	2	3	10	0	1
EABT19654	0	6	5	1	4	1	0
EABT19655	1	4	5	0	2	5	1
EABT19656	66	44	10	110	25	63	33.97
EABT19657	593.33	265.08	138	2	131.85	738	577.8
EABT19658	144	219	55	185.51	137	161.05	111
EABT19659	1	1	3	1	3	0	0
EABT1966	0	2	16	8	4	0	1
EABT19660	2	0	2	0	1	0	0
EABT19661	1	2	5	7	1	1	0
EABT19662	6	2	6	0	0	0	1
EABT19663	0	1	35	1	1	0	0
EABT19664	4	11	3	18	6	6	4
EABT19665	0	0	6	0	0	0	0
EABT19666	1	1	38	0	0	1	0
EABT19667	0	0	0	14	0	0	0
EABT19668	59	102	67	157.95	47	24	63
EABT19669	90.08	41	80.83	34	17	95.96	28.94
EABT1967	3154.12	5979.95	8398.36	12366.3	3489.52	4070.74	3150.07
EABT19670	3	3	4	0	0	0	0
EABT19671	994.65	919.94	503	978.34	558	450.89	446.49
EABT19672	936.55	3112.56	9703.2	986.58	1142	3	227
EABT19673	116	30	14	502	22	38	31
EABT19674	23	8	65	1	0	1	0
EABT19675	0	1	1	0	2	1	2
EABT19676	0	2	1	2	0	0	0
EABT19677	0	0	2	0	0	0	0
EABT19678	0	1	4	0	0	0	0
EABT19679	0	0	5	0	2	0	1
EABT1968	0	0	18	0	0	0	0
EABT19680	1	5	37	9	1	0	1
EABT19681	0	0	5	3	0	0	0

EABT19682	0	1	4	0	1	7	2
EABT19683	1653.65	2956.98	6670.41	9568.02	1595.19	848.43	820.98
EABT19684	497.05	19	0	0	0	32	0
EABT19685	0	1	5	0	0	0	0
EABT19686	1	7	31	61.23	8	0	6
EABT19687	1	3	1	1	0	1	1
EABT19688	17	47	419.93	101.04	42	11	9
EABT19689	0	0	7	2	0	0	0
EABT1969	3	0	0	1	1	3	0
EABT19690	1	1	27	2	0	0	1
EABT19691	0	1	2	10	0	0	0
EABT19692	0	1	1	3	0	0	0
EABT19693	27	139	239	166	806.48	72	40.3
EABT19694	3811.08	2033	612.01	3	4	407	1
EABT19695	0	2	77	3	1	0	0
EABT19696	2	3	3	1	3	0	1
EABT19697	2	2	8	0	0	0	1
EABT19698	1253.98	2417.05	3655.34	3905.32	2341.42	533	859.29
EABT19699	3	4	0	0	0	0	2
EABT197	0	0	7	0	0	0	0
EABT1970	1	1	8	10	1	2	3
EABT19700	0	0	6	0	0	0	0
EABT19701	0	0	7	1	0	0	0
EABT19702	2	1	3	1	0	4	0
EABT19703	0	0	1	2	4	0	1
EABT19704	0	1	7	0	0	1	2
EABT19705	2	0	3.74	0	0	2	1
EABT19706	57	180	393.01	87	33	84.99	49
EABT19707	0	0	0	0	0	0	0
EABT19708	17	22	0	0	3	24	15
EABT19709	3474.62	4911.89	4119.79	9769.72	4667.47	1977.12	2921.5
EABT1971	0	0	5	0	0	0	0
EABT19710	6	25	37	41	7	0	4
EABT19711	9	18	0	0	0	0	0
EABT19712	40	394.67	1745.85	204.12	2124.54	19	145
EABT19713	0	2	1	13	3	0	0
EABT19714	0	0	1	8	1	0	0
EABT19715	2	2	0	0	0	3	0
EABT19716	2	23	1	7	9	0	0
EABT19717	2	6	16.14	5	6	17	5
EABT19718	7.97	5	6	2	2	26	22
EABT19719	66	408	581	935.95	633.9	76	156.01
EABT1972	1	0	14	0	0	0	0
EABT19720	4	9	30	9	14	4	3
EABT19721	2	4	0	8	0	2	6
EABT19722	1	6	11	5	5	1	5
EABT19723	3	2	1	13	2	0	0
EABT19724	3	8	1	10	1	3	0
EABT19725	150.06	214.01	81	200.91	116.73	96.05	79.66
EABT19726	11	9	38	29	8	2	1
EABT19727	0	0	1	3	0	0	0

EABT19728	0	1	8	0	1	0	1
EABT19729	0	0	4	1	0	0	0
EABT1973	1	2	9	2	1	0	1
EABT19730	2	1	2	0	1	1	6
EABT19731	0	1	3	0	1	0	1
EABT19732	1	7	11	0	1	0	0
EABT19733	0	0	2	1	0	0	0
EABT19734	0	2	11	2	0	3	0
EABT19735	1	1	26	2	0	4	4
EABT19736	0	2	6	3	0	0	0
EABT19737	2357.83	2743.57	4773.66	5611.65	2028.42	2131.02	1806.96
EABT19738	3803.7	6932.5	5563.21	1849.84	2797.35	6015.32	7155
EABT19739	3	5	10	0	0	4	1
EABT1974	6499.49	3612.78	1851.72	5974.11	2050	7006.93	5628.12
EABT19740	1	6	4	4	2	1	3
EABT19741	0	0	0	1	0	0	0
EABT19742	0	0	5	6	0	0	0
EABT19743	0	1	11	0	78	8	166
EABT19744	2	1	6	0	0	1	0
EABT19745	7	8	7	1	0	2	0
EABT19746	0	0	1	1	520	0	0
EABT19747	3	6	8	79	1	0	1
EABT19748	0	0	14	0	0	0	0
EABT19749	1	5	45.01	14	0	6	0
EABT1975	2178.18	3263.5	2436.42	3177.25	1887.21	2597.63	2176.63
EABT19750	0	2	0	9	1	0	7
EABT19751	7	21	35	71	18	31	82
EABT19752	2778.91	1823	4	0	106	515	366
EABT19753	2	2	3	2	0	0	8
EABT19754	40.32	39	16	27.98	47.99	0	10
EABT19755	0	2	18	0	0	2	0
EABT19756	311.97	442.99	531.1	1305.21	355	230.98	286
EABT19757	9	4	5	0	1	8	11
EABT19758	0	0	5	1	0	0	0
EABT19759	0	2	7	6	0	0	0
EABT1976	1	0	7	1	0	0	1
EABT19760	3837.12	6764.37	4776.95	4962.01	1601.85	7088.07	2014.17
EABT19761	6	8	52	3	4	1	1
EABT19762	494	627	635.16	1226.05	514.96	683.01	340
EABT19763	2053.51	2980.14	3605.77	10843.3	4172.36	1310	1197.56
EABT19764	24	25	195.99	12	10	3	0
EABT19765	2	8	9	3	0	1	0
EABT19766	25	16	4	242	111	59	67
EABT19767	502.96	803.39	1540.87	1044.33	420.55	163	405.21
EABT19768	0	0	3	0	0	1	0
EABT19769	0	1	1	0	0	3	1
EABT1977	262.23	398.01	162.69	255.89	176.97	187	132.68
EABT19770	26	107	109	167	32	65	35
EABT19771	6918.6	14065.8	16332.5	22439.4	9063.26	9770.44	9729.13
EABT19772	0	2	4	6	2	0	3
EABT19773	0	2	3	0	0	0	0

EABT19774	12.01	9	21	22	6	3	8
EABT19775	1085.65	1631.62	1155.97	4891.09	1563	837.2	874.01
EABT19776	0	1	9	0	0	0	0
EABT19777	1	6	0	11	0	0	0
EABT19778	18.99	31.97	39.55	42	0	2	2
EABT19779	0	0	0	5	1	0	0
EABT1978	976.48	1535.31	1879.8	2280.84	1334.88	1448.25	1713.43
EABT19780	2	0	5	1	0	1	0
EABT19781	2	3	3	3	0	1	1
EABT19782	1	0	5	2	0	1	1
EABT19783	3	1	4	11	1	0	0
EABT19784	1	2	19	0	1	0	0
EABT19785	34	61	132	565.6	85	10	26
EABT19786	0	0	5	0	0	0	0
EABT19787	11	21	58	159	19	9	9
EABT19788	9	16	1	21	0	7	0
EABT19789	1	1	145	1014.74	4	4	0
EABT1979	2	6	0	0	0	15	9
EABT19790	1255.18	2327.57	481	4826.97	1664.58	1	87
EABT19791	51	64	6	0	60	188	349
EABT19792	0	0	0	2	1	0	0
EABT19793	4	2	18	1	1	1	2
EABT19794	0	3	2	0	0	0	1
EABT19795	20484.1	26823.8	18192.2	1656.84	5482.99	242	1915.76
EABT19796	4	3	2	2	2	2	0
EABT19797	0	0	0	0	0	0	0
EABT19798	6211.97	8001.86	9018.17	10528.4	4945.37	4067.82	4147.93
EABT19799	4	6	15	16	1	9	2
EABT198	3	10	70	8	1	4	3
EABT1980	0	0	1	0	0	0	0
EABT19800	3	1	4	70	152	0	0
EABT19801	15	34	81	41	17	32	22
EABT19802	22	48	153	22	3	19	27
EABT19803	13845.9	7230.4	574.89	622.14	938.01	1551	1732.73
EABT19804	2	11	15	6	3	2	1
EABT19805	0	3	1	9	0	0	0
EABT19806	0	2	4	1	0	0	0
EABT19807	1	18	75.46	6	2	0	0
EABT19808	0	1	11	1	4	4	4
EABT19809	414.99	1156	1153.96	16481.7	171	34	17
EABT1981	0	5	18	2	3	2	0
EABT19810	0	0	22	0	0	1	1
EABT19811	2	5	18	8	2	3	2
EABT19812	2	3	5	5	1	4	4
EABT19813	182	230	332.11	1155	177	227.01	265.09
EABT19814	79	27	1	3	9	52	138
EABT19815	1.01	5	8	0	0	3	3
EABT19816	1	2	42	0	1	0	1
EABT19817	0	3	3	2	0	0	0
EABT19818	1	2	17	3	0	0	1
EABT19819	0	1	1	3	1	1	0

EABT1982	9	4	0	0	0	14	23
EABT19820	0	3	1	9	2	0	1
EABT19821	0	2	2	3	2	1	0
EABT19822	0	2	5	0	0	0	0
EABT19823	566.93	819	757.67	1647.82	530.35	554.67	486
EABT19824	6894	11504.4	6873.77	2306.63	1571.77	2126	2069.12
EABT19825	3670.54	5387.16	9460.7	5954.48	2335.98	4598.73	2600.91
EABT19826	4137.73	5532.23	3325.87	10751.7	3937.11	5952.13	3971.73
EABT19827	1164.19	1469	1171	3262.22	1143.01	1275	1095
EABT19828	0	1	3	0	0	0	1
EABT19829	3	3	0	0	0	18	2
EABT1983	0	0	5	0	0	0	0
EABT19830	1	0	1	0	0	0	1
EABT19831	36	9	47	10	19	47	6
EABT19832	273.96	650.83	106.08	43	144	176	221.1
EABT19833	1	1	16	0	0	0	0
EABT19834	0	2	20	7	0	0	0
EABT19835	14848.8	16888	11758.1	17601.3	7972.27	13639.8	13047.6
EABT19836	2840.14	3985.98	3151.81	10465	3024.96	2344.89	3347.02
EABT19837	0	2	4	2	0	0	0
EABT19838	1	0	4	0	0	1	1
EABT19839	0	4	0	3	0	0	0
EABT1984	1	5	12	5	0	0	0
EABT19840	0	1	17	1	0	0	0
EABT19841	6.97	15	0	0	1	7	6
EABT19842	1	1	5	3	0	0	0
EABT19843	4	9	7	14	0	1	0
EABT19844	0	2	11	3	0	0	0
EABT19845	0	0	19	3	0	0	0
EABT19846	1	2	2	1	0	0	1
EABT19847	1062	3324.01	99	180	289	108	696
EABT19848	3	10	5	11	16	5	0
EABT19849	0	0	0	0	0	0	0
EABT1985	1	1	0	2	0	5	2
EABT19850	0	4	16	0	1	1	3
EABT19851	1	0	11.92	2	1	2	1
EABT19852	6	4	21	16	1	6	3
EABT19853	2	5	25	7	127	2	0
EABT19854	0	0	14	3	1	0	0
EABT19855	0	0	25	0	0	0	0
EABT19856	4	1	6	0	1	7	0
EABT19857	0	0	14	1	0	0	2
EABT19858	0	0	0	0	0	0	0
EABT19859	10	3	13	22	10	16	8
EABT1986	2	5	19.04	5	7	6	2
EABT19860	1	1	0	2	0	0	5
EABT19861	0	1	0	0	6	0	21
EABT19862	2247.52	2840.77	2151.03	3969.64	1882	1799	1448.12
EABT19863	2	0	10	2	0	2	2
EABT19864	20077.7	16085.1	7817.56	7862.82	4175.38	7321.96	8423.02
EABT19865	1	0	9	5	2	0	1

EABT19866	0	1	9	0	0	0	0
EABT19867	2	3	0	0	0	9	4
EABT19868	0	4	3	15	1	1	0
EABT19869	5	18	17	6	9	34	69
EABT1987	2	7	15	1	4	0	0
EABT19870	0	2	9	2	0	0	0
EABT19871	1	2	6	4	0	0	0
EABT19872	1	2	10	172.76	3	2	4
EABT19873	5	9	3	1	0	1	0
EABT19874	1	0	4	0	0	0	0
EABT19875	0	0	2	1	1	1	0
EABT19876	1	1	4	16	3	0	0
EABT19877	680.94	975	849.06	1597.56	776	551.6	658.79
EABT19878	465.72	631.11	269	192	212.11	299.12	476.97
EABT19879	2504.08	6815.87	5649.58	7234.32	26348.2	566.97	1611.19
EABT1988	1048.93	2136.99	2000.18	1352	866.97	1032.1	1047.93
EABT19880	0	0	19	1	0	1	0
EABT19881	1763.03	3597.19	6628.41	7681.3	2825.39	1620.59	2620.94
EABT19882	5	3	1	2	0	0	0
EABT19883	7	10	10	2	2	0	0
EABT19884	0	8	12	14	0	4	5
EABT19885	0	3	7	1	3	0	3
EABT19886	0	1	0	1	2	14	1
EABT19887	0	0	0	13	0	0	1
EABT19888	1	0	6	3	0	0	0
EABT19889	1009.6	1297.81	1194.46	3860.38	1199.54	791.62	845.98
EABT1989	4	6	9	7	4	0	0
EABT19890	0	3	9	5	1	0	1
EABT19891	3	4	2	11	1	0	1
EABT19892	448.99	993.88	1234.36	1574.54	808.22	605.17	580.79
EABT19893	12	23.03	25	1	1	0	1
EABT19894	1	5	6	6	2	2	1
EABT19895	2253	4382.84	2765.91	8284.15	3988.68	1536.73	2459.29
EABT19896	56	44	1	0	9	25	25
EABT19897	1	1	1	4	4	0	3
EABT19898	1	2	4	8	2	0	0
EABT19899	0	1	5	2	0	0	0
EABT199	4	9	21	14	2	1	4
EABT1990	4	3	3	0	0	0	0
EABT19900	0	1	4	1	1	3	0
EABT19901	1	0	4	1	1	0	0
EABT19902	0	0	0	14	5	1	3
EABT19903	3	12	21	11	1	3	1
EABT19904	3	0	5	3	1	0	6
EABT19905	2	3	5	0	0	1	0
EABT19906	6	0	4	0	0	18	1
EABT19907	7	12	53	23	13	4	5
EABT19908	303.36	434.78	588.63	649.26	849.38	290.56	287.06
EABT19909	1	0	2	0	2	0	0
EABT1991	1	2	11	22	0	0	0
EABT19910	3	1	1	0	0	1	0

EABT19911	0	2	2	1	0	1	0
EABT19912	52	120	440.96	789.96	130.99	50.73	113.99
EABT19913	22	41	3	86	83	7	28
EABT19914	0	0	12	2	0	0	0
EABT19915	0	0	0	2	12	0	0
EABT19916	81	112	36	15	94	31	416.33
EABT19917	4	10	64	3	0	3	5
EABT19918	4	0	0	0	0	0	6
EABT19919	3	0	1	2	0	0	0
EABT1992	1	0	5	3	0	0	0
EABT19920	0	0	0	17	1	0	1
EABT19921	113	130	949	179	1224	24	40
EABT19922	0	0	5	0	0	0	1
EABT19923	1	0	5	0	2	0	6
EABT19924	0	1	3	1	0	2	1
EABT19925	0	0	4	5	0	1	0
EABT19926	0	0	5	1	5	0	1
EABT19927	0	3	69	2	2	4	0
EABT19928	3	9	15.02	3	359	6	4
EABT19929	0	0	8	0	0	0	0
EABT1993	2	3	37	3	1	5	2
EABT19930	10	67	47	33	451.32	19.93	9
EABT19931	0	0	10	0	0	0	1
EABT19932	0	4	49	3	1	2	4
EABT19933	0	0	6	0	0	0	6
EABT19934	0	0	1	9	0	0	0
EABT19935	5	19	198	15	2	5	8.58
EABT19936	0	5	0	0	1	3	22
EABT19937	0	0	13	4	0	0	1
EABT19938	0	0	0	0	0	0	19
EABT19939	19	26	56	118	11	4	13
EABT1994	806.36	1022.43	1116.13	1879.16	682.91	533.97	803.84
EABT19940	277.21	334.04	495.16	1683.73	418	188	89
EABT19941	1492.51	2306.96	2893.72	2871.66	1965.03	1359.18	1929.44
EABT19942	0	0	8	0	0	0	0
EABT19943	15	45	52	82	42	12	27
EABT19944	0	0	2	4	0	1	1
EABT19945	2	4	2	5	8	0	0
EABT19946	4	6	12	2	13	1	9
EABT19947	0	1	3	0	0	0	0
EABT19948	17	68	61	46	8	24	11
EABT19949	318.96	533.95	695.47	1220.07	666.94	333	252
EABT1995	0	8	4	3	5	0	0
EABT19950	0	0	8	0	0	0	0
EABT19951	3	2	2	53	0	0	0
EABT19952	0	3	4	2	1	15	4
EABT19953	0	0	8	0	1	0	0
EABT19954	2831.82	3911.59	2690.06	5532.05	2052.93	1643.49	1505.26
EABT19955	1	0	3	2	0	0	0
EABT19956	3	3	4	0	0	0	1
EABT19957	0	1	3	0	2	1	1

EABT19958	0	0	16	0	0	0	0
EABT19959	1	1	11	0	0	0	1
EABT1996	1	5	22	4	4	2	10
EABT19960	0	3	1	1	1	0	0
EABT19961	0	1	34	2	0	1	1
EABT19962	281	325.61	241	461.99	437.18	237	282.05
EABT19963	0	1	2	1	3	3	4
EABT19964	0	4	11	8	2	1	0
EABT19965	1151.68	1854.83	1664.55	2318.23	1118.15	819.01	790.43
EABT19966	0	0	5	1	0	0	1
EABT19967	128.91	86	45	11	8	109	42
EABT19968	2	2	0	1	2	3	0
EABT19969	1003.06	1281.1	815.06	1147.34	769.82	754.25	570.03
EABT1997	0	0	9	0	0	0	0
EABT19970	0	0	3	3	1	0	0
EABT19971	0	0	18	3	1	3	1
EABT19972	2	3	1	3	1	1	3
EABT19973	80	295.92	27	7	82	424.17	336
EABT19974	1	0	6	3	0	0	0
EABT19975	0	0	1	7	12	0	0
EABT19976	0	0	2	0	0	0	0
EABT19977	852.41	1005	592.35	1295.91	975.47	689	724.34
EABT19978	2	0	5	1	2	0	0
EABT19979	0	2	8	1	0	0	0
EABT1998	2	0	13	9	2	3	2
EABT19980	0	1	21	2	0	1	0
EABT19981	500	753	420	1120.55	695.99	330	469
EABT19982	4	19	166	39	9	0	0
EABT19983	0	0	15	2	3	4	1
EABT19984	233	463	330	778	180	248.2	220
EABT19985	762.62	1158.83	789.98	2048.28	1121.56	743.8	1007.8
EABT19986	22778.5	6554.22	59	0	6492.77	15541.4	31073
EABT19987	3	8	6	0	1	0	0
EABT19988	0	2	3	2	1	0	0
EABT19989	84	276	324	234.17	994.69	28	149.01
EABT1999	0	3	2	4	0	0	0
EABT19990	2	0	0	8	2	0	0
EABT19991	1	0	23	1	0	0	0
EABT19992	0	0	8	0	0	0	1
EABT19993	538.85	866.99	1087.49	1313.96	795	365.12	491
EABT19994	533.01	1505.05	566.95	1948.01	525	810	428
EABT19995	0	2	0	0	0	1	2
EABT19996	16	46	196	5	16	25	3
EABT19997	0	3	0	7	2	0	0
EABT19998	745	837.55	752.32	677.04	217	561	204
EABT19999	0	0	15	0	1	0	1
EABT2	0	1	2	0	0	0	2
EABT20	0	0	1	4	0	1	0
EABT200	0	0	28	8	3	0	1
EABT2000	1	2	152	0	1	0	0
EABT20000	2	1	6	0	1	0	0

EABT20001	0	0	2	1	0	0	0
EABT20002	1	1	3	7	3	1	4
EABT20003	1193	697.36	479	913.01	153	413	97
EABT20004	5	55	53	53	6	0	2
EABT20005	1	1	2	9	2	2	1
EABT20006	1	1	18	0	0	1	0
EABT20007	0	3	4	2	17	1	8
EABT20008	0	0	0	2	3	0	0
EABT20009	2	3	6	9	9	0	0
EABT2001	0	0	0	0	0	0	0
EABT20010	0	0	1	0	0	0	1
EABT20011	2485.34	6236.76	5126.03	5209.74	2823.23	2897.42	2550.6
EABT20012	603	829.39	477	1598.23	553	327	259
EABT20013	945	1134.86	1096	2706	1046.52	885.02	904.1
EABT20014	0	1	17	3	0	0	0
EABT20015	3598.28	4720.72	6660.61	8483.8	3864.64	3321.57	2291
EABT20016	0	1	5	1	1	1	0
EABT20017	5	6	0	0	1	36	17
EABT20018	1120.04	1578.51	1132.92	2314.87	916.93	1044.11	781.7
EABT20019	0	1	2	0	0	0	1
EABT2002	4	2	5	18	5	1	6
EABT20020	0	0	2	2	0	1	0
EABT20021	0	0	15	1	0	2	0
EABT20022	0	1	2	1	2	0	1
EABT20023	0	0	0	1	0	0	0
EABT20024	2	3	1	2	1	1	4
EABT20025	0	0	2	5	0	0	0
EABT20026	2048.14	2848.71	2592.21	4377.55	1577.5	2361.12	1888.06
EABT20027	0	0	7	1	0	0	0
EABT20028	8	28	45	37	29	3	83
EABT20029	1	5	7	0	0	1	0
EABT2003	21	4	6	1	15	35	43
EABT20030	99	223	94	108	110	49	112
EABT20031	1	0	5	3	0	0	0
EABT20032	0	6	2	1	0	4	0
EABT20033	27	7	5	4	3	0	5
EABT20034	1	3	2	1	1	0	3
EABT20035	0	1	11	0	0	0	0
EABT20036	0	3	0	3	0	1	1
EABT20037	0	0	8	2	0	1	0
EABT20038	0	1	48	1	0	3	0
EABT20039	1	2	8	5	2	1	0
EABT2004	0	8	4	5	25	0	5
EABT20040	0	2	26	2	0	0	0
EABT20041	1	3	6	1	1	1	0
EABT20042	769	1146	7516.76	4920.62	1935	981.72	1172.77
EABT20043	1	4	25	0	0	1	2
EABT20044	2	4	1	6	0	2	1
EABT20045	0	1	14	5	0	13	0
EABT20046	1698.57	3092.8	36	237.21	3142.87	2762.96	16827.6
EABT20047	0	4	15	1	3	0	1

EABT20048	168	600.73	586	1129.07	280	388	354
EABT20049	605.01	824.01	734.08	1879.13	1171	652.79	785.96
EABT2005	11124	22018.3	1133.91	345.4	15003.2	11560.5	71276.7
EABT20050	2	4	5	1	1	2	1
EABT20051	1618.66	1933.75	1863.1	4640.42	1662.01	1524.18	1555.03
EABT20052	1	4	2	3	0	3	1
EABT20053	549	1227.58	1083.01	1786.1	897.88	292	432
EABT20054	1	6	10	84	0	2	6
EABT20055	0	2	7	0	0	5	3
EABT20056	4	7	96	30	2	6	5
EABT20057	12	52.85	22	13	22	1	38
EABT20058	1	2	1	1	1	1	1
EABT20059	0	1	11	11	0	0	1
EABT2006	9	11	3	6	5	2	3
EABT20060	30	33	27	69	2	7	10
EABT20061	1	0	5	1	1	1	3
EABT20062	1415.58	1578.23	1073.71	3694.26	1561.28	18.01	224.91
EABT20063	16	23	6	44	13	0	9
EABT20064	34497.4	58351.1	64612.7	76592.4	25826.5	26466.6	21709.6
EABT20065	29	43	45	15	39	7	24
EABT20066	6	19	22	34	3	1	5
EABT20067	2	3	10	1	0	0	0
EABT20068	9	25	33	0	11	14	68
EABT20069	3	18	89	804	9	4	6
EABT2007	0	12	1	0	2	2	4
EABT20070	1427.99	2974.02	3872.51	4347.22	1455.18	1145.55	1155.98
EABT20071	0	1	2	6	0	0	0
EABT20072	0	1	1	4	0	0	0
EABT20073	0	0	18	0	0	0	0
EABT20074	7	3	1	0	1	6	5
EABT20075	2	0	2	0	0	0	13
EABT20076	208	173.51	94.94	15	51.56	468.79	353.82
EABT20077	0	1	5	1	1	0	0
EABT20078	11	24	8	67	35	2	8
EABT20079	2	2	0	1	0	0	0
EABT2008	92.84	180.99	86.38	10	61.15	91	209.55
EABT20080	233.84	778.99	1621.19	1632.69	1717.27	207	610.6
EABT20081	0	0	2	0	0	0	0
EABT20082	2	3	4	2	1	0	1
EABT20083	0	0	10	0	0	0	0
EABT20084	0	1	3	0	0	0	0
EABT20085	0	0	4	0	0	0	2
EABT20086	0	0	5	0	0	0	0
EABT20087	1	0	25	4	0	0	0
EABT20088	0	0	5	0	0	0	0
EABT20089	7	16	78	8	7	1	2
EABT2009	4	2	11	1	28	2	1
EABT20090	0	4	515	1	5	3	6
EABT20091	2	7	6	10	5	0	3
EABT20092	0	0	1	1	0	1	0
EABT20093	0	5	3	6	0	2	4

EABT20094	0	0	0	0	0	1	3
EABT20095	1	0	3	2	3	0	0
EABT20096	1	0	4	2	3	1	0
EABT20097	20	18	6	2	1	0	13
EABT20098	5	9	5	5	0	0	2
EABT20099	1	3	3	5	0	0	0
EABT201	1	0	0	7	0	0	0
EABT2010	4	22	39	138	10	3	2
EABT20100	1	1	0	10	21	1	20
EABT20101	1	1	8	14	49.98	3	3
EABT20102	3	17	9	2	1	0	2
EABT20103	1	6	12	8	1	1	1
EABT20104	0	2	0	5	1	1	1
EABT20105	0	0	4	1	1	0	0
EABT20106	0	1	10	11	0	0	0
EABT20107	2	0	4	3	0	0	1
EABT20108	0	50	14	1	0	1	0
EABT20109	1	1	28	1	0	0	0
EABT2011	14	31	26	178	34	33	57
EABT20110	0	0	5	33	1	0	1
EABT20111	2	1	5	2	1	7	3
EABT20112	9	13	12	5	0	1	3
EABT20113	1105.58	1355.83	936.18	1179.92	1138.4	1040.05	600.08
EABT20114	1	0	4	3	0	0	0
EABT20115	1866	2086.11	1925	3416.88	1491.99	1876.92	1248
EABT20116	1347.82	1772.61	1787.21	3263.73	1754.26	2270	1506.18
EABT20117	0	0	1	7	1	0	0
EABT20118	5	0	0	3	0	0	0
EABT20119	1699.95	1944.13	206.98	18	1334.01	2168.73	839
EABT2012	4	17	0	1	0	4	3
EABT20120	0	4	1	0	0	0	0
EABT20121	4	18	59	13	7	0	3
EABT20122	1	1	8	0	1	0	0
EABT20123	1	0	0	0	0	2	0
EABT20124	2	3	1	4	2	0	0
EABT20125	0	1	4	1	2	2	1
EABT20126	793.55	1620.74	2320.86	2401.52	739.2	563.91	816.01
EABT20127	0	3	12	0	0	0	0
EABT20128	0	0	1	3	0	1	0
EABT20129	16	1	0	0	0	16	0
EABT2013	0	0	13	0	0	0	0
EABT20130	1	2	1	0	6	1	8
EABT20131	0	1	12	0	1	0	0
EABT20132	1	0	2	1	0	0	4
EABT20133	0	0	12	0	0	0	0
EABT20134	9	9	30	26	4	3	0
EABT20135	554	804.01	603.98	291.61	308	1046.72	852.45
EABT20136	23	36	381	16	16	21	6
EABT20137	2	1	7	1	0	1	0
EABT20138	1028.36	1180.95	998.66	748.21	868.99	728.59	729.67
EABT20139	4	2	0	0	0	0	0

EABT2014	5	9	0	0	2	34	16
EABT20140	1	5	16	1	1	4	1
EABT20141	11	17.99	14.03	85	8	4	18.92
EABT20142	0	0	7	0	0	0	0
EABT20143	106.4	260	157.79	407.16	98	56	21
EABT20144	3	1	0	0	0	0	1
EABT20145	102	111	13	1004.97	116	23	2
EABT20146	0	2	5	0	0	0	0
EABT20147	0	2	10	0	0	0	0
EABT20148	0	0	7	3	1	1	1
EABT20149	0	0	8	1	0	0	0
EABT2015	0	29	17	1	2	0	0
EABT20150	0	0	3	3	2	0	0
EABT20151	2	2	28	4	3	0	1
EABT20152	8	7	0	0	0	0	0
EABT20153	115	192	185	643	167	105	123
EABT20154	2	1	1	1	0	1	0
EABT20155	3181.58	6129.45	8652.4	7278.67	4147.98	2383.03	2532.69
EABT20156	0	0	1	0	1	0	4
EABT20157	0	1	22	5	1	0	0
EABT20158	11	4	13	31	10	1	6
EABT20159	1	1	0	0	0	4	0
EABT2016	1	6	0	1	0	1	0
EABT20160	0	0	5	0	0	0	0
EABT20161	156	58	455	5598.22	162	6	33
EABT20162	0	3	7	4	0	1	0
EABT20163	5	3	2	5	1	0	0
EABT20164	0	2	5	7	2	0	0
EABT20165	0	0	3	1	1	0	0
EABT20166	0	0	12	1	0	0	0
EABT20167	380.87	889	526	1348.17	559.01	3	46.11
EABT20168	0	0	9	0	1	1	0
EABT20169	639	2243	2558.52	3891.86	1990.89	19	134
EABT2017	0	1	5	0	1	4	0
EABT20170	2	5	19	3	0	1	2
EABT20171	912	1287.04	1204.67	1248.14	846.23	490	756
EABT20172	3	3	12	1	1	0	0
EABT20173	0	0	0	6	1	0	0
EABT20174	585.96	1096.14	1213.27	2263.24	715	379	405.24
EABT20175	215.25	84.02	192.27	1197.27	74.14	67.91	62.27
EABT20176	0	2	1	5	1	1	4
EABT20177	3	10	36	90	8	1	0
EABT20178	5	11	6	8	1	10	3
EABT20179	0	0	1	0	0	0	0
EABT2018	21	862	200	99	45	19	38
EABT20180	886	1240.57	1197.05	2751.05	1157.06	645.99	595
EABT20181	0	1	0	0	1	12	12
EABT20182	57	119.95	158.78	208.76	34	27.25	33
EABT20183	11	28	66	300	24	3	14
EABT20184	0	4	7	1	0	2	0
EABT20185	5	5	1	32	2	0	0

EABT20186	1388.36	1307.01	260.01	1723.83	849.39	705.1	684.16
EABT20187	10698.2	68953.8	9878.72	14410.2	3685.34	1165.95	1951.8
EABT20188	0	0	0	2	1	0	1
EABT20189	2	1	7	13	6	2	2
EABT2019	906.08	1256.7	706.89	3465.22	912.72	774	678.99
EABT20190	44	271.97	202	469.82	117	17.17	41
EABT20191	3	1	0	2	0	1	0
EABT20192	1273.05	6194.82	8538.71	8977.28	7012.57	510	1260.93
EABT20193	2	6	19	9	0	2	2
EABT20194	1057	1407.99	540.19	1540	1229.94	664	637
EABT20195	6	5	15	1	1	1	0
EABT20196	4	10	1	8	3	2	0
EABT20197	2	1	9	16	5	0	11
EABT20198	0	0	0	1	0	2	0
EABT20199	2	6	11	10	2	3	0
EABT202	0	0	12	0	0	0	0
EABT2020	2.88	6	4	15	4	0	13
EABT20200	8	7	6	2	3	3	10
EABT20201	5	10	8	7	12	4	3
EABT20202	0	9	8	0	0	1	1
EABT20203	16	46	29	5	11	1	2
EABT20204	2	8	1	0	3	1	1
EABT20205	414.01	957.35	841.99	756	1097.99	50	210
EABT20206	1	0	14	0	0	0	0
EABT20207	17	10	5	2	3	12	2
EABT20208	2	0	55	0	3	0	2
EABT20209	0	1	1	0	0	1	5
EABT2021	0	8	4	1	0	1	2
EABT20210	71	60	56	185.49	74	9	13
EABT20211	4	9	47	25	9	3	4
EABT20212	0	1	0	3	2	0	0
EABT20213	0	7	10	9	0	0	2
EABT20214	2	27	27	42	7	0	1
EABT20215	0	6	2	1	1	3	0
EABT20216	3	6	141	8	9	0	2
EABT20217	2159.17	3924.73	12257.6	1833.25	4394	184.93	4630.25
EABT20218	737.12	1000.95	849.08	1260.92	732.04	597.18	495.1
EABT20219	2128.01	2486.36	2219.87	4758.02	1963.28	2129.95	1506.56
EABT2022	22	5	7	1	0	12	0
EABT20220	13	21	0	0	0	14	18
EABT20221	1	1	15	1	0	2	0
EABT20222	0	8	4	0	1	0	0
EABT20223	9	11	41	26	3	3	10
EABT20224	12	40	48	25	7	7	6
EABT20225	1	0	7	0	0	0	0
EABT20226	0	3	31	4	11	0	0
EABT20227	1973.99	2298.98	1732	6295.21	974	2087.67	813.34
EABT20228	1	1	4	3	0	0	1
EABT20229	38650.7	12318.2	13907.6	33674.3	9619.2	65526.2	55835.8
EABT2023	0	0	0	1	1	0	0
EABT20230	4759.46	3810.11	2017.39	343	1284.95	5945.63	5872.9

EABT20231	0	1	4	1	1	0	0
EABT20232	0	0	1	0	0	0	0
EABT20233	0	0	12	0	0	0	0
EABT20234	13	13	8	0	12244.8	14	44
EABT20235	144	223	173	636.05	148	42	89
EABT20236	0	0	4	0	6	2	8
EABT20237	0	1	1	5	2	0	0
EABT20238	3	2	3	10	4	3	4
EABT20239	2	5	13	6	1	2	0
EABT2024	3389.02	6208.94	7656.99	18277.6	3087.45	2589.83	2722.09
EABT20240	0	1	2	3	0	0	0
EABT20241	4	3	31	2	0	1	0
EABT20242	1	0	0	0	1	0	0
EABT20243	2	12	59	16	27	1	3
EABT20244	2	4	5	6	3	0	0
EABT20245	314.09	966.13	583.17	204.98	59	529	282
EABT20246	3557.41	4525.54	3286.37	4416.51	3369.3	3089.56	3283.95
EABT20247	5	15	6	11	32	0	2
EABT20248	2	3	5	1	1	0	1
EABT20249	1	4	1	0	0	0	0
EABT2025	0	0	10	0	0	0	0
EABT20250	1	4	13	1	5	5	7
EABT20251	0	1	3	2	1	3	0
EABT20252	1	1	38	0	0	0	0
EABT20253	2	6	21	1	2	0	1
EABT20254	1	2	11	4	0	0	0
EABT20255	24	1	3	0	1	21	1
EABT20256	706.39	1948.16	1284.48	372.54	385.1	1541.66	1721.46
EABT20257	2	5	3	6	1	0	0
EABT20258	0	1	2	2	0	0	1
EABT20259	1498.27	2095.56	1970.81	3216.77	1448.76	1394.78	1466.3
EABT2026	11	35	29	30	0	0	1
EABT20260	4	6	19	18	4	1	3
EABT20261	0	0	1	9	2	0	0
EABT20262	38	20	0	9	2	0	8
EABT20263	0	0	5	8	1	0	0
EABT20264	1	3	3	1	0	1	1
EABT20265	0	1	1	11	0	0	0
EABT20266	1	1	2	2	0	0	0
EABT20267	4	0	0	0	0	6	0
EABT20268	0	1	11	2	1	0	1
EABT20269	4	5	7	2	3	0	1
EABT2027	1	4	3	0	3	0	6
EABT20270	90	31	34	160	159	7	8
EABT20271	0	1	56	0	0	0	0
EABT20272	1	0	5	2	1	0	0
EABT20273	358.46	535.43	2396.35	107.99	392.7	268.54	134.01
EABT20274	1	1	7	11	2	1	3
EABT20275	24	19	21	90	2586.93	11	46
EABT20276	0	0	35	1	0	0	0
EABT20277	8	13	0	0	0	2	1

EABT20278	545	826	660.37	1239.09	742.57	294	327
EABT20279	4	0	0	0	0	8	2
EABT2028	0	0	5	1	1	0	1
EABT20280	0	0	19	0	0	0	0
EABT20281	671	969.41	1490.75	2216.19	782	961.74	847.71
EABT20282	17	41	50.01	39	49	1	12
EABT20283	1	3	5	5	0	0	0
EABT20284	0	0	0	0	1	1	0
EABT20285	1	0	8	0	0	0	0
EABT20286	3761.76	10594.5	1618.05	174.98	1107.18	347.95	1076.06
EABT20287	118	246	89	5888.01	8302.3	8	14
EABT20288	10	11	2	2	0	8	2
EABT20289	1	7	6	12	1	0	0
EABT2029	14	9	324.91	23	1	10	1
EABT20290	1	2	20	3	3	1	1
EABT20291	0	1	5	1	0	0	0
EABT20292	694.61	1195.12	787.91	3121.55	595.95	469.93	585
EABT20293	0	0	1	2	0	0	0
EABT20294	4	16	18	16	2	0	3
EABT20295	2	1	0	9	3	0	1
EABT20296	0	2	7	0	1	0	0
EABT20297	1	0	7	1	0	0	0
EABT20298	0	1	7	1	0	1	1
EABT20299	1	2	8	0	4	1	4
EABT203	4	25	16	57	3	1	2
EABT2030	2	3	18	1	3	7	3
EABT20300	14	89	98	186	44.01	3	10
EABT20301	0	1	1	0	0	1	0
EABT20302	115	84	208	235.35	254	12	41
EABT20303	2303.99	3405.26	5050.2	6393.82	2188.77	670.9	1151.92
EABT20304	7	8	16	6	5	8	2
EABT20305	12	3	0	0	4	3	19
EABT20306	2	0	17	0	0	1	0
EABT20307	1	2	4	1	1	0	0
EABT20308	2	7	9	11	2	0	2
EABT20309	5	10	13	1	4	3	2
EABT2031	12	14	37	1	4	6	7
EABT20310	0	6	12	568	0	0	2
EABT20311	1	4	24	2	1	0	0
EABT20312	1	2	15	2	1	1	0
EABT20313	1	2	28	3	0	0	0
EABT20314	0	0	13	3	3	0	1
EABT20315	3	6	9	3	0	6	0
EABT20316	0	2	4	2	0	2	1
EABT20317	4	8	0	0	4	0	0
EABT20318	3	8	19	5	4	0	6
EABT20319	0	0	6	0	1	0	0
EABT2032	1	0	3	0	0	0	0
EABT20320	0	2	16	2	0	2	0
EABT20321	1	0	4	4	0	0	0
EABT20322	2	5	2	13	2	4	6

EABT20323	0	0	11	1	0	1	0
EABT20324	8	3	1	0	1	5	10
EABT20325	1911.9	1730	753	976	6539.07	1263	1184.1
EABT20326	1191.33	1542.53	1768.84	2100.15	715.98	1019.01	897.34
EABT20327	723.02	959.82	904.88	1524	734.99	606	427.23
EABT20328	0	2	4	1	0	0	0
EABT20329	0	3	2	0	0	1	1
EABT2033	1	4	2	6	13	0	0
EABT20330	19	3	10	6	76.4	4	1
EABT20331	76.88	398	785.96	687	699	95	619
EABT20332	0	0	8	1	1	0	0
EABT20333	0	2	11	0	0	1	0
EABT20334	10	10	3	1	1	25.65	9
EABT20335	3	1	3	9	2	1	0
EABT20336	0	1	4	1	1	0	0
EABT20337	5081.03	6057.89	3077.44	4756.9	3499.66	5691.79	4473.06
EABT20338	0	5	4	0	0	0	0
EABT20339	0	5	12	49	0	0	0
EABT2034	0	0	3	0	1	0	0
EABT20340	0	3	3	1	0	2	0
EABT20341	1	1	13	3	1	1	2
EABT20342	0	2	1	1	3	0	0
EABT20343	4472.25	7572.36	8068.5	16654.7	4548	3044.52	3878.17
EABT20344	0	3	10	9	5	0	0
EABT20345	0	8	10	0	0	0	0
EABT20346	1816.99	1450	1074	813	1849	196.98	899.01
EABT20347	0	0	6	0	0	0	0
EABT20348	1	0	1	0	0	0	10
EABT20349	1	10	16	4	1	0	2
EABT2035	6	25.07	261.85	62	50	6	3
EABT20350	6	7	43	23	8	10	6
EABT20351	97	146	94	832.02	151	7	13
EABT20352	18	69	8	69	231.66	1	4
EABT20353	0	2	0	2	3	0	0
EABT20354	2	5	11	6	0	1	1
EABT20355	3112.22	3957.11	2816.23	5256.74	2690.23	2523.02	1893.62
EABT20356	0	0	3	1	0	0	0
EABT20357	0	1	4	0	0	4	1
EABT20358	2	0	9	0	0	2	4
EABT20359	0	1	1	1	0	0	0
EABT2036	1	4	11	3	0	0	0
EABT20360	0	0	0	19	0	0	0
EABT20361	4268.35	7343.07	10814.7	18107.2	4260.81	3725.18	4107.76
EABT20362	0	2	0	2	1	1	1
EABT20363	128	121	18	20	3	9	13
EABT20364	1	0	9	1	1	0	0
EABT20365	8	21	25	56	3	18	2
EABT20366	4	2	30	2	1	2	0
EABT20367	2	5	4	6	1	0	1
EABT20368	3	3	0	10	0	2	0
EABT20369	2362.09	3243.97	1836	2747.34	1941.63	2174.47	2012.3

EABT2037	1	13	16	1	2	1	4
EABT20370	588.54	662	129	357	1582	736.99	545
EABT20371	0	2	5	16	3	1	0
EABT20372	1	7	25	2	1	0	0
EABT20373	1	0	6	1	2	0	1
EABT20374	0	4	2	0	0	2	0
EABT20375	0	6	39	2	0	1	1
EABT20376	797.62	880.21	444.87	875.96	744.7	451.4	523.43
EABT20377	0	0	9	2	1	1	0
EABT20378	0	2	12	109	15	0	1
EABT20379	10	431	1042	225	16836	15	108.66
EABT2038	1	3	0	1	1	1	31
EABT20380	0	0	11	1	0	0	0
EABT20381	0	0	2	0	1	0	0
EABT20382	0	0	0	1	12	0	4
EABT20383	889.01	1149.82	452.46	1063.01	958	107.35	1359
EABT20384	114	220.71	85	263.11	32	30	24
EABT20385	8	11	6	3	3	7	2
EABT20386	749.56	646.64	1339.27	3473.41	837.96	7	47
EABT20387	1	11	6	1	2	0	1
EABT20388	245	454	67	882.01	216	7	102
EABT20389	1	8	36	61.56	7	0	2
EABT2039	0	1	8	0	0	0	0
EABT20390	0	0	1	0	0	0	1
EABT20391	7	15	18	20	2	10	6
EABT20392	1	3	3	1	0	0	0
EABT20393	67	197.64	576.97	5812.85	509	11	105.83
EABT20394	5763.95	11725	9171.12	23676.2	4572.88	5795.3	4070.02
EABT20395	1172.3	1399	606	990	533	1327	903
EABT20396	1	0	15	0	0	2	0
EABT20397	0	0	17	2	0	0	0
EABT20398	2	1	6	12	0	0	0
EABT20399	0	0	8	9	1	1	4
EABT204	538.97	823.1	500.06	572.94	351.55	431.39	439.83
EABT2040	0	4	5	9	3	0	0
EABT20400	0	1	9	1	1	0	1
EABT20401	0	2	8	0	0	1	0
EABT20402	0	6	1	0	1	0	0
EABT20403	7	0	4	0	0	4	0
EABT20404	111.78	235.59	63.21	56	206.4	2	8.87
EABT20405	3928.28	4474	5501.01	10137.6	3721.13	2681.98	2398.18
EABT20406	1	0	5	0	2	1	2
EABT20407	2178.99	2962.73	3739.32	5210.93	2406.98	1777.88	1989.36
EABT20408	1474.97	522	126	718.24	563.91	12	79
EABT20409	13	30.01	20.34	25	24	0	1
EABT2041	2	7	30	208.02	319.16	10	4
EABT20410	0	0	0	0	2	38	16
EABT20411	8694.95	2428.36	386	720.38	2147.19	1	116
EABT20412	105.54	295	724.55	4455.13	2024.46	95	27
EABT20413	3	3	1	0	0	14	0
EABT20414	4	6	9	2	0	0	1

EABT20415	0	6	7	12	1	1	0
EABT20416	0	1	5	0	0	2	0
EABT20417	2	1	9	0	1	1	0
EABT20418	2	5	0	1	1	0	1
EABT20419	3	4	14	0	1	0	0
EABT2042	0	0	7	0	0	0	0
EABT20420	4	10	1	1	4	0	0
EABT20421	3	10	7	0	0	3	2
EABT20422	2	1	1	1	0	7	1
EABT20423	19071.2	41088.7	28284.8	18532.1	8229.43	4802.99	4275.98
EABT20424	0	0	0	5	3	0	1
EABT20425	645.72	1118.5	520.07	755.75	611.85	409.53	576.08
EABT20426	2	4	0	1	1	0	0
EABT20427	0	0	0	0	0	0	0
EABT20428	1	2	46	1	2	1	0
EABT20429	26	45	236	33	21	32	38
EABT2043	0	2	11	1	0	0	0
EABT20430	1	1	4	14	7	0	0
EABT20431	4	10	5	3	1	2	2
EABT20432	0	0	5	0	0	0	0
EABT20433	124	238.24	23	0	1835.77	358	3917.41
EABT20434	170	222.86	105	353	274	54	134
EABT20435	0	0	1	2	1	0	1
EABT20436	3	4	3	1	0	1	0
EABT20437	3	12	27	6	2	5	1
EABT20438	2761.3	4228.41	4833.23	5392.61	2574.32	2151.24	2409.44
EABT20439	0	1	1	2	0	2	2
EABT2044	48	19	2	1	4	48	26
EABT20440	1780.07	2085.09	893.81	1452.92	2138.19	635.61	3199.84
EABT20441	0	0	25	8	1	0	0
EABT20442	0	1	5	0	0	0	0
EABT20443	15	7	4	0	172	5	85
EABT20444	9	19	4	39	6	0	1
EABT20445	4278.85	9266.69	5208.86	10801	2981.79	6224.29	3609.34
EABT20446	4	3	0	0	0	1	22
EABT20447	0	0	3	0	1	0	0
EABT20448	0	0	18	3	0	0	0
EABT20449	0	1	4	1	0	0	0
EABT2045	9	5	22.1	38	14.56	3	11
EABT20450	0	0	24	0	0	0	0
EABT20451	1	14	63.22	6	0	0	0
EABT20452	2	0	2	0	0	1	0
EABT20453	2	1	16	9	0	0	4
EABT20454	1095.03	1684.01	1814.08	2982.86	1311.78	1484.17	1035.99
EABT20455	2	4	0	1	0	1	3
EABT20456	2	1	0	0	0	3	0
EABT20457	6	2	0	0	0	0	0
EABT20458	623.99	1130.29	994.91	1626.33	601.2	472.11	640.72
EABT20459	1	6	5	0	0	0	0
EABT2046	83	151	127	779	103	42.72	63
EABT20460	0	2	4	4	0	1	1

EABT20461	494	534.63	387	724	536.11	459	298
EABT20462	0	1	6	7	1	0	0
EABT20463	2	6	1	4	1	1	2
EABT20464	1934.54	3094.66	2991.99	5877.54	2672.89	1753.14	2354.81
EABT20465	20	75	33	5	2	48	20
EABT20466	2	5	19	6	3	1	1
EABT20467	2	5	12	8	4	1	4
EABT20468	22	31	29	178	26	6	14
EABT20469	74	122	30	264	9	7	21
EABT2047	1	3	4	4	8	0	1
EABT20470	938	1314	1096.68	1930	941.02	1024	782.69
EABT20471	127	238.97	1635.85	140	566	118	1031
EABT20472	6	23.11	22	29	0	2	0
EABT20473	2	3	15	2	0	1	0
EABT20474	1	1	0	0	1	14	0
EABT20475	0	3	9	9	3	0	0
EABT20476	4	12	11	5	7	0	0
EABT20477	3	1	2	3	5	2	14
EABT20478	430	1206.87	1998.64	4816.55	1482	818	1749.06
EABT20479	3	4	23	2	1	0	2
EABT2048	1519.55	2192.77	1968.95	3420.89	2137.77	1508.76	1640.74
EABT20480	0	1	6	4.84	0	0	0
EABT20481	0	1	2	3	0	0	0
EABT20482	26	37	11	1	1	0	1
EABT20483	24	30	5	54	9	0	3
EABT20484	73	160	92	180	108	29	49
EABT20485	2	2	15	7	3	0	3
EABT20486	457.57	1178.93	3124.38	1088.43	1061.7	42	194
EABT20487	5	7	15	21	5	0	4
EABT20488	1	4	3	16	6	1	2
EABT20489	0	4	33	1	3	2	0
EABT2049	4	9	7	1	0	24	0
EABT20490	2	3	8	1	0	1	3
EABT20491	0	1	2	3	0	0	0
EABT20492	4	13.17	29	67	1	3	1
EABT20493	2988.73	5410.77	11345.4	4500.02	1584.99	2591	2403.23
EABT20494	0	2	17	3	0	0	0
EABT20495	1581.46	2047.59	936.05	4386.79	704.06	950	1134.25
EABT20496	1	0	32	0	0	0	0
EABT20497	2	10	2	16	20	1	4
EABT20498	1	1	0	0	3	5	9
EABT20499	0	1	2	2	0	0	0
EABT205	1	10	32	6	3	0	0
EABT2050	0	0	4	4	1	0	0
EABT20500	0	0	2	3	7	1	14
EABT20501	0	2	2	0	0	0	0
EABT20502	0	1	5	2	0	0	1
EABT20503	1	5	7	3	2	0	1
EABT20504	1	0	8	0	0	0	0
EABT20505	1	3	1	0	0	0	2
EABT20506	0	2	2	0	1	0	0

EABT20507	3	3	12	3	3	1	1
EABT20508	1	0	10	4	2	0	4
EABT20509	2	10	18	29	1	0	1
EABT2051	11	7	12	6	3	14	5
EABT20510	4	9	2	5	6	1	2
EABT20511	0	1	0	0	7	0	1
EABT20512	122.62	96.33	0	1	712	200.05	6
EABT20513	0	6	42	2	3	3	0
EABT20514	1525.91	2492.64	1769.99	3180.17	1420.32	897.29	1344
EABT20515	1151.66	1529.86	774.49	2345.23	891.74	559.12	1160.73
EABT20516	491	782	869.02	1455	936.93	491	431
EABT20517	5	9	17	3	8	2	4
EABT20518	0	0	4	5	0	0	0
EABT20519	0	5	16	19	1	1	1
EABT2052	1	3	2	7	2	1	0
EABT20520	1	7	0	0	3	0	2
EABT20521	0	0	19	0	1	0	0
EABT20522	0	2	1	0	2	0	0
EABT20523	3	0	23	2	0	3	2
EABT20524	2	4	21	1	0	4	2
EABT20525	49	180.11	398.88	1035	239	11	535
EABT20526	927.52	1067.09	1170.18	1308.21	1016.04	1367.61	590.58
EABT20527	944	1672.65	1288.53	4333.89	1074.53	494	715
EABT20528	0	0	2	1	9	0	0
EABT20529	1	0	10	0	0	0	1
EABT2053	4363.63	5181.42	2224	6231.32	2786.79	2012.75	2621.02
EABT20530	0	10	8	3	3	1	1
EABT20531	2395	4902.42	3842.68	3892.32	2088.86	2019	1970.74
EABT20532	24610.9	46672.9	42197	24073.6	81361.5	1370	7166.39
EABT20533	2	0	11	5	1	0	0
EABT20534	0	0	11	1	0	0	0
EABT20535	5	12	23	12	51	1	5
EABT20536	0	4	24	0	6	1	18
EABT20537	0	0	1	0	1	0	0
EABT20538	0	0	5	0	0	0	0
EABT20539	0	0	1	5	0	1	2
EABT2054	0	1	5	1	0	0	0
EABT20540	0	3	18.53	5	0	0	0
EABT20541	2	6	8	4	0	6	5
EABT20542	6	17	91	44	3	5	1
EABT20543	0	0	6	0	0	0	0
EABT20544	1	0	2	1	0	1	0
EABT20545	0	0	7	0	0	0	0
EABT20546	0	3	0	4	0	0	0
EABT20547	284	456.08	342	523	200	215	236.95
EABT20548	1	1	11	1	0	1	0
EABT20549	3	4	2	0	0	0	0
EABT2055	0	2	0	2	2	0	0
EABT20550	1	6	17	198	31	0	3
EABT20551	18	85	428.79	237	187.99	9	17
EABT20552	1	0	4	4	1	1	0

EABT20553	3329.9	4218.81	4624.97	10535	3186.01	2912.95	2317.01
EABT20554	2	0	2	2	2	1	11
EABT20555	2	11	15	3	1	4	1
EABT20556	2877.87	5640.45	6202.86	13131.9	2949.73	3285.32	2346.74
EABT20557	0	1	8	2	0	0	0
EABT20558	0	2	22	3	2	0	0
EABT20559	16	12	0	0	0	1	1
EABT2056	1	3	0	0	0	4	0
EABT20560	73	112	121	819.14	63.01	35	29
EABT20561	0	1	9	0	0	0	0
EABT20562	0	0	21	4	0	0	1
EABT20563	1307.14	1848.27	2431.75	3900.48	1726.32	569	1000.38
EABT20564	73	239	424	79	17.02	5	1
EABT20565	2	7	0	0	0	0	2
EABT20566	1	0	7	0	0	0	0
EABT20567	0	0	0	6	0	0	0
EABT20568	5	5	0	0	0	25	7
EABT20569	2	1	1	2	1	0	0
EABT2057	1	4	6	3	2	1	0
EABT20570	1321.94	562	160	291	103	277	144
EABT20571	1782	2255	1303	2344	1148.05	1365.05	1212
EABT20572	1	7	6	1	1	3	0
EABT20573	1378.68	1679.35	1289.43	2378.59	1383.09	919.16	870.94
EABT20574	0	0	15	1	0	0	1
EABT20575	2	11	3	1	0	0	0
EABT20576	983.92	1464.05	2917.58	1178.22	874.58	280.43	410
EABT20577	6	3	5	4	1	14	0
EABT20578	0	6	1	1	1	0	0
EABT20579	1	6	4	0	0	1	1
EABT2058	10	32	37	56	4	15	11
EABT20580	1	1	10	2	1	2	1
EABT20581	6	22	163	7	4	0	4
EABT20582	2	2	9	1	1	0	2
EABT20583	0	1	2	0	0	0	1
EABT20584	75.01	211	535	235	569.06	276.01	123
EABT20585	0	0	9	1	0	0	0
EABT20586	2	7	10	72	16	0	0
EABT20587	54	252	442	462.99	507.19	0	22
EABT20588	2096.23	3114.63	5673.03	2890.3	2276.22	2542.89	2314.82
EABT20589	2	1	4	2	0	0	0
EABT2059	0	5	11	83	7	1	2
EABT20590	1	10	4	15	33	0	4
EABT20591	1	1	28	1	0	1	0
EABT20592	0	0	19	0	0	0	0
EABT20593	0	1	6	0	0	0	2
EABT20594	0	0	10	0	0	0	0
EABT20595	0	0	0	0	1	0	0
EABT20596	0	11	9	203	98	1	596.02
EABT20597	1	0	7	0	0	0	1
EABT20598	7952.84	9396.39	3347.41	7030.8	3369.29	11962.5	3718.85
EABT20599	461.38	628.59	247.95	552.45	382	210.53	352.18

EABT206	1	0	4	0	1	0	0
EABT2060	0	0	8	0	0	2	0
EABT20600	1	1	10	2	1	0	0
EABT20601	18	16	13	7	8	7	9
EABT20602	0	0	6	0	0	0	0
EABT20603	1	36	41.29	55	26	1	12
EABT20604	14	24	271	157	4	6	1
EABT20605	0	1	6	0	0	1	0
EABT20606	1	3	7	3	1	1	3
EABT20607	2	0	11	0	0	0	0
EABT20608	0	0	3	0	0	1	0
EABT20609	0	0	7	2	2	2	2
EABT2061	1	0	15	0	0	0	0
EABT20610	29	65	4	38	9	31	24
EABT20611	6	5	10	5	2	2	1
EABT20612	163.88	458.86	1253.9	1329.77	774.9	196	164
EABT20613	0	10	0	0	0	1	0
EABT20614	1154.23	1441.47	1298.28	682.26	1006.02	1532.92	2617.86
EABT20615	1824.07	1911.17	916.96	1040	807.86	3155.89	1484.13
EABT20616	3	5	0	6	1	3	2
EABT20617	19	31.22	19	45	50	5	16.06
EABT20618	4	17	158	30	11	3	2
EABT20619	2	3	22	0	0	0	0
EABT2062	3	14	12	6	7	2	3
EABT20620	105	40	128.53	172.33	150	65	118
EABT20621	8	13	37	69.7	33	5	7
EABT20622	50	38	41	21	29	29	43
EABT20623	18	37.86	20	7	7	13	17
EABT20624	1	8	54	7	0	0	1
EABT20625	3	7	0	0	0	3	2
EABT20626	1062.99	1494.87	1545.37	2391.18	1087.52	816	943.01
EABT20627	0	0	4	0	0	2	0
EABT20628	2	0	9	0	0	0	0
EABT20629	435.86	371.19	233.03	678.8	180.23	228.99	113
EABT2063	37	146.99	148	116	7	47	11
EABT20630	2	12	5	17	1	0	2
EABT20631	378.02	533.97	222	214.13	117.94	18	20
EABT20632	0	2	3	1	1	0	0
EABT20633	39	24	11	13	592	32	0
EABT20634	30	22	58	60	106	7	49
EABT20635	474.77	624.44	1566.55	6463.77	12	1	4
EABT20636	0	0	12.37	1	0	0	0
EABT20637	0	0	0	0	1	0	0
EABT20638	0	1	8	1	0	0	0
EABT20639	0	0	0	1	3	0	0
EABT2064	2	1	0	9	0	1	0
EABT20640	2821.24	4435.58	5254.43	6602.02	2501.81	2737.1	2850.1
EABT20641	0	0	6	0	0	1	0
EABT20642	2	4	32	1	0	0	1
EABT20643	0	0	3	0	0	0	2
EABT20644	2	10	56	4	2	1	1

EABT20645	15	13	32.67	0	2	5	0
EABT20646	2694.95	4478.44	5513.31	6249.86	2236.98	1903.58	2152.93
EABT20647	1	0	11	1	1	1	0
EABT20648	42.02	69.62	391.56	1	6	1	4
EABT20649	5	13	0	4	4	6	27
EABT2065	1	4	1	0	1	0	1
EABT20650	0	0	7	0	0	0	0
EABT20651	11	17	0	6	0	8	0
EABT20652	0	0	3	0	0	0	1
EABT20653	1	5	0	4	3	0	0
EABT20654	0	0	26	1	0	0	0
EABT20655	3	3	27	12	1	0	0
EABT20656	0	2	10	0	0	0	0
EABT20657	109.95	168.99	67.01	197.94	102.97	25	141.6
EABT20658	10	5	4	1	1	0	2
EABT20659	0	0	1	0	1	0	0
EABT2066	4	19	0	0	0	224	2
EABT20660	0	3	3	13	8	1	0
EABT20661	1125.37	734.22	1777.15	3336.78	1936.25	213.46	300.81
EABT20662	6	9	0	0	0	3	1
EABT20663	2	4	16	2	3	4	4
EABT20664	5	3	8	2	0	0	0
EABT20665	65	53	0	0	3	20	21
EABT20666	0	1	3	0	0	0	0
EABT20667	10	14	22	42	11	0	4
EABT20668	0	0	4	1	0	0	2
EABT20669	13	54	98	475	39	13	14
EABT2067	0	1	0	0	0	0	0
EABT20670	0	0	8	0	0	0	0
EABT20671	1658.69	1683.9	1262.29	2512.63	1097.14	1654.35	1042.79
EABT20672	0	0	1	0	8	0	16
EABT20673	402.33	448.88	221.44	436.36	329.96	229.82	188
EABT20674	0	2	3	5	1	0	1
EABT20675	0	2	1	2	0	1	3
EABT20676	8	6	11	6	7	4	7
EABT20677	1654.54	3358.82	9082.45	5138.21	1920.41	1836.98	1304.85
EABT20678	0	2	8	8	0	1	3
EABT20679	2	0	84	2	0	0	0
EABT2068	2	1	2	5	0	0	0
EABT20680	0	3	4	8	1	1	0
EABT20681	1	1	12	0	0	2	1
EABT20682	2	3	7	0	0	0	0
EABT20683	511.55	678.57	819.86	867.16	372.34	134.33	425.31
EABT20684	9561.61	7251.69	631.79	449.07	2652.89	9092.92	16067.2
EABT20685	0	0	3	1	1	0	0
EABT20686	11	41	72.04	62	10	15	17
EABT20687	0	0	1	2	0	0	0
EABT20688	12	13	17	251.97	140	1	2
EABT20689	0	2	6	7	0	0	0
EABT2069	0	2	7	3	0	0	0
EABT20690	9	45	47	68	4	23	23

EABT20691	0	0	10	1	11	4	13
EABT20692	2	1	8	4	0	2	0
EABT20693	0	2	16	2	0	0	0
EABT20694	1	1	1	0	0	0	0
EABT20695	0	0	3	0	1	0	0
EABT20696	830	1219.49	1259	1958.79	905	851	981.32
EABT20697	4081.9	8083.48	7639	15640.5	2654.04	2755.07	3174.16
EABT20698	1	1	0	0	0	0	0
EABT20699	2	3	29	5	3	3	0
EABT207	45	20	2	0	0	46	0
EABT2070	0	4	25	2	0	0	1
EABT20700	8	7	26.93	7	26	0	3
EABT20701	621.48	4490.45	5407.33	43361.1	1120.84	211	198
EABT20702	2566.53	4183.83	3466.16	7205.93	3209.15	3385.57	3226.73
EABT20703	2	1	34	2	1	9	8
EABT20704	0	1	0	2	4	0	0
EABT20705	7	18	7	20.02	14	0	7
EABT20706	106	172	216	190.96	68	202	97
EABT20707	1	2	53	4	3	4	0
EABT20708	1822.74	3416.94	9943.45	8389.2	1916.8	2117	1600
EABT20709	2	31	8	45	9	3	1
EABT2071	0	2	1	16	1	0	0
EABT20710	5	5	27	21	10	6	10
EABT20711	0	4	2	4	0	0	2
EABT20712	1	1	10	5	0	1	0
EABT20713	0	0	7	0	0	0	0
EABT20714	6	23	15	35	2	18	5
EABT20715	0	5	1	0	0	0	0
EABT20716	0	0	0	17	0	0	0
EABT20717	2	2	4	1	0	4	2
EABT20718	0	0	3	13	3	0	0
EABT20719	0	0	3	8	0	1	0
EABT2072	0	1	0	4	5	0	2
EABT20720	2	1	16	0	0	0	0
EABT20721	0	0	6	2	0	0	0
EABT20722	993.95	1826.08	2573.93	3316.86	1410.32	1003	1000.11
EABT20723	1	6	6	0	0	0	1
EABT20724	18	86	113	66	1	2	1
EABT20725	0	0	3	3	0	0	0
EABT20726	1	0	6	1	0	0	0
EABT20727	0	0	0	7	0	1	1
EABT20728	1	3	43	1	6	0	0
EABT20729	0	1	0	1	0	1	0
EABT2073	0	1	3	0	0	0	1
EABT20730	4	4	6	1	63	2	1
EABT20731	1	9	4	1	4	1	0
EABT20732	5	2	2	0	7	11	26
EABT20733	0	0	4	3	0	0	0
EABT20734	707	871	1151.98	1320.48	392.03	319.74	400
EABT20735	0	2	4	1	2	2	2
EABT20736	120.99	146.01	612.41	170.94	636.04	23	19

EABT20737	1099.99	1996.87	1952.87	2888.25	1012.64	702.79	613.02
EABT20738	0	1	11	0	1	0	0
EABT20739	1	2	10	6	0	0	0
EABT2074	4	2	47.83	0	1	0	1
EABT20740	0	2	18	2	1	1	1
EABT20741	4	13	43	9	3	0	5
EABT20742	0	8	10	12	1	1	0
EABT20743	11	49	26	54	4	46	11
EABT20744	0	2	3	5	0	0	0
EABT20745	0	4	13	2	0	0	3
EABT20746	906.99	1076.96	871.15	2477.02	777.25	999	734.82
EABT20747	0	5	0	0	0	0	0
EABT20748	0	1	0	0	0	2	2
EABT20749	2	2	7	4	0	0	0
EABT2075	4	1	8	11	1	1	0
EABT20750	0	0	10	1	0	0	0
EABT20751	3395.05	5459.57	2530.92	14395.9	2509.34	1919.62	2935.68
EABT20752	35	115.53	205	69	7	4	9
EABT20753	14	8	0	0	0	1	0
EABT20754	1	1	6	4	0	0	1
EABT20755	0	1	13	1	0	0	0
EABT20756	691.85	1312.14	1247.97	2379.09	848.07	558.99	505
EABT20757	1	0	5	1	1	0	0
EABT20758	1451.4	1850.78	2845.66	2746.07	1022.8	1442.57	1189.04
EABT20759	4	5	0	5	4	0	2
EABT2076	0	0	4	2	0	0	0
EABT20760	0	1	7	0	0	1	1
EABT20761	1	1	0	0	0	8	0
EABT20762	91	163	793	185	68	67	66.94
EABT20763	5	18	106	46	5	21.3	9
EABT20764	1	2	5	1	0	4	1
EABT20765	4987.25	7355.97	5460.07	9908.65	3971.14	3246	3841.94
EABT20766	0	2	1	1	3	1	2
EABT20767	26	57	18	95	57	38	44
EABT20768	3	10	15	4	4	3	103
EABT20769	0	1	2	4	1	0	4
EABT2077	6	8	24	61	6	1	13
EABT20770	0	4	18	19	135	1	1
EABT20771	0	0	4	0	0	0	0
EABT20772	3.95	8	117	3	11	16	4
EABT20773	1	1	7	84	1	0	0
EABT20774	0	1	6	1	0	0	0
EABT20775	0	0	3	3	1	0	1
EABT20776	12	27	72	18	42	2	12
EABT20777	1	0	0	0	1	0	2
EABT20778	0	1	10	1	0	0	0
EABT20779	19	13	30	1.99	1	11	1
EABT2078	893.28	1216.98	1389.97	2032.68	962.59	769	692
EABT20780	14	24	0	0	1	0	0
EABT20781	0	0	5	0	0	0	0
EABT20782	0	1	1	1	0	0	0

EABT20783	1	8	12	2	0	9	1
EABT20784	0	0	4	0	0	0	0
EABT20785	288.01	423.95	309.71	708.03	381.1	242	194
EABT20786	1594.77	1905.41	2392.19	2632.46	970.04	2278.96	1223.24
EABT20787	0	0	6	1	3	0	1
EABT20788	0	3	20	3	2	0	0
EABT20789	3	1	2	1	0	0	0
EABT2079	0	3	2	0	1	1	0
EABT20790	6331.13	26416.5	35583.9	8919.71	12166.1	52	661
EABT20791	85.04	251.55	143	288	228	34	81
EABT20792	2	0	4	0	0	0	0
EABT20793	0	2	4	1	2	0	0
EABT20794	2664.49	2821.61	3117.77	4494.83	2178.38	1414.01	1192.09
EABT20795	634.08	2974.54	77.98	5306.06	390.16	704.32	655.06
EABT20796	1	1	16.37	10	0	3	2
EABT20797	0	1	3	1	0	1	4
EABT20798	0	0	2	11	4	1	0
EABT20799	0	2	2	13	0	0	0
EABT208	0	0	6	0	0	0	0
EABT2080	5	5.02	11	3	0	1	1
EABT20800	6	26	24	23	24	3	13
EABT20801	2216.49	2914.68	4973.6	3453.1	1828.98	2609.04	1889.86
EABT20802	1	0	2	1	1	0	0
EABT20803	3	2	3	0	1	1	1
EABT20804	7	0	0	0	258	3	11
EABT20805	10	3	1	0	0	0	0
EABT20806	546.85	816.72	372.48	232	382.05	192.21	294.02
EABT20807	1	1	12	3	0	0	0
EABT20808	7	5	55	9	6	16	14
EABT20809	0	3	9	4	0	1	2
EABT2081	11	19	0	4	0	7	1
EABT20810	1	3	6	0	2	2	7
EABT20811	0	1	1	0	0	82	3
EABT20812	3076.99	3948.91	2684.93	6634.6	1918.67	2387.56	1909
EABT20813	1	13	10	1	2	4	2
EABT20814	0	2	10	2	3	0	0
EABT20815	2	0	0	0	0	10	0
EABT20816	0	0	4	0	0	1	0
EABT20817	0	4	8	1	0	1	0
EABT20818	9	7	5	0	10	1	10
EABT20819	24.01	117.05	167.44	388.08	239.2	39.39	60.4
EABT2082	0	0	1	36	3	2	1
EABT20820	988.98	1322.96	1078.05	2522.25	1152.1	682.96	968.29
EABT20821	0	0	2	0	0	0	1
EABT20822	2	4	0	0	0	1	0
EABT20823	1430.98	3214.02	2700.5	6941.14	1284.95	1465.02	1311.97
EABT20824	429.28	1482.76	545.65	565.94	143.28	214	203.72
EABT20825	1	4	30	7	55	5	1
EABT20826	175	213.6	480.01	527.79	114.57	73.96	86
EABT20827	4	1	0	0	0	1	0
EABT20828	8063.13	15731.6	20872.1	16564	9665.05	7637.15	11279.6

EABT20829	5	11	9	7	3	0	0
EABT2083	622.85	934.15	442.02	2320.46	1383.01	169	439.95
EABT20830	2	2	0	0	1	1	0
EABT20831	6	14	3	2	4	0	4
EABT20832	3	9	116	3	1	0	0
EABT20833	282	332	156.1	532.93	196	86	159
EABT20834	0	5	2	1	0	0	0
EABT20835	5	14	51	17	6	13	12.92
EABT20836	0	5	5	31.98	8	0	0
EABT20837	0	0	11	0	0	0	0
EABT20838	0	3	11	1	0	2	2
EABT20839	8312.62	2700.2	9631.98	20533	3668.82	124	1051.74
EABT2084	2.98	1	0	0	0	0	0
EABT20840	4	10	3	0	1	0	0
EABT20841	0	0	3	4	0	0	0
EABT20842	0	1	5	1	0	0	0
EABT20843	9	20	16	29	9	0	11
EABT20844	0	5	13	0	0	0	0
EABT20845	0	3	24	1	0	0	0
EABT20846	1	0	0	2	7	0	1
EABT20847	1	4	0	9	7	0	3
EABT20848	4480.95	6119.96	10394.6	9464.99	2090.91	3354.92	1965.36
EABT20849	0	0	19	1	0	0	0
EABT2085	0	4	2	1	0	0	0
EABT20850	1223.27	1103.51	287.76	2	425	2573.75	2155.47
EABT20851	837.01	1567.14	1251.4	2958	1219.68	824.64	881.19
EABT20852	1343.19	1032.19	592	333	338	2434.34	2059.98
EABT20853	2954.07	6460.32	6906.77	2275.72	4505.91	1256.53	2654.76
EABT20854	14	11	23	11	5	11	4
EABT20855	1	0	4	1	0	0	0
EABT20856	1	1	4	1	0	0	0
EABT20857	5723.57	5348.84	511.15	401	5489.86	17005	16144.3
EABT20858	1	13	24	6	0	3	3
EABT20859	3	9	39	15	5	9	8
EABT2086	0	0	10	0	2	0	0
EABT20860	2	1	3	0	0	1	0
EABT20861	2	2	7.98	2	0	0	0
EABT20862	1	2	2	8	1	2	8
EABT20863	3	13	38	17	2	3	5
EABT20864	0	0	7	1	0	1	0
EABT20865	0	1	3	1	0	0	1
EABT20866	1	0	2	0	0	3	3
EABT20867	1089.26	1308.09	600.99	178.88	421.64	2683.29	887.9
EABT20868	0	0	3	0	0	0	0
EABT20869	0	2	3	0	0	0	0
EABT2087	153.44	211.23	502.29	503.27	289.81	90	108
EABT20870	1	0	10	1	0	1	1
EABT20871	11	48	403.75	96	5	25	1
EABT20872	1578.02	2548.47	2615.22	2812.29	1528.98	1257.99	1434.93
EABT20873	44.97	108.94	100	626.94	71	3	32.99
EABT20874	236	650	1311.95	842	331	327	327

EABT20875	1584	1492.89	416	1168	2186.6	1502	1014.8
EABT20876	0	3	1	2	0	0	1
EABT20877	1	1	3	17	0	0	1
EABT20878	1	0	0	0	1	0	1
EABT20879	0	0	9	0	0	0	0
EABT2088	0	3	52	16	29	1	2
EABT20880	0	1	10	0	0	1	0
EABT20881	1	0	7	0	1	0	0
EABT20882	10	19	39	523.95	37	2	9
EABT20883	0	5	22	1	1284.93	3	4440.79
EABT20884	0	3	1	2	1	0	0
EABT20885	0	0	0	5	1	0	0
EABT20886	17	31	29	70	11	7	26
EABT20887	539	612.08	453	1218	565.24	315	280
EABT20888	1	4	2	0	1	0	0
EABT20889	0	2	12	1	0	0	0
EABT2089	1	0	1	2	1	2	0
EABT20890	79.33	46.95	62.68	18	30	32	32.99
EABT20891	3	3	3	34	1	1	2
EABT20892	1	1	11	4	0	0	0
EABT20893	7	8	0	0	1	1	1
EABT20894	5	2	25	6	0	5	5
EABT20895	1755.21	2277.03	2054	3339.99	1250	1307	1543.06
EABT20896	1	1	0	0	1	3	4
EABT20897	1	12	6	4	1	2	0
EABT20898	89128.5	23239.8	21	1	38209.2	87201.6	203565
EABT20899	18129.1	18353.9	5797.29	10993.3	4759.72	11664.3	10177.3
EABT209	1	2	38	0	2	0	0
EABT2090	4280.92	2037	860	2331	1143	4252.71	2494
EABT20900	0	0	6	10	1	0	0
EABT20901	0	3	9	0	0	0	1
EABT20902	0	0	7	2	0	0	1
EABT20903	3	4	8	7	0	1	1
EABT20904	0	0	5	0	0	0	0
EABT20905	1	0	19	4	0	1	1
EABT20906	1	0	0	20	0	0	0
EABT20907	0	1	38	1	1	1	2
EABT20908	0	6	1	69	1	0	0
EABT20909	1	1	7	5	4	0	0
EABT2091	2	0	7	1	0	0	2
EABT20910	635	826	949	1568	720.01	746.39	619
EABT20911	0	9	3	0	0	0	0
EABT20912	34	78	83	157	20	22	27
EABT20913	0	2	7	4	0	0	0
EABT20914	5	1	1	0	1	0	0
EABT20915	1	3	4	0	0	0	0
EABT20916	0	0	5	2	0	1	0
EABT20917	0	1	1	0	2	2	0
EABT20918	6455.7	5224.58	832.97	255.94	740.66	6182.09	4176.28
EABT20919	4	2	7	2	2	0	4
EABT2092	15364.2	27676.6	15920.8	6279.97	7042.16	24220.7	33252.5

EABT20920	8	2	0	0	0	0	0
EABT20921	0	0	4	1	0	0	0
EABT20922	5	3	5	10	1	6	1
EABT20923	1	4	136	0	0	0	1
EABT20924	0	1	0	9	0	0	0
EABT20925	0	2	10	0	0	0	0
EABT20926	1	0	23	1	0	1	0
EABT20927	1	5	0	3	0	0	4
EABT20928	0	1	6	0	0	0	0
EABT20929	1	2	11	1	0	0	0
EABT2093	9	12	0	0	0	0	0
EABT20930	13	10	10	62	18	2	13
EABT20931	0	0	11	0	0	0	0
EABT20932	6	12	0	0	1	10	9
EABT20933	0	0	4	0	0	0	0
EABT20934	34	172	362	228.01	69	50.08	146.15
EABT20935	0	0	5	4	3	0	1
EABT20936	2	3	36	1	0	2	1
EABT20937	12	23	7	20	8	5	14
EABT20938	1	7	35.36	5	1	0	0
EABT20939	1	4	5	0	0	2	0
EABT2094	2	1	6	0	0	0	3
EABT20940	13	23	0	0	0	1	11
EABT20941	0	0	5	1	0	1	1
EABT20942	0	3	0	1	0	0	0
EABT20943	0	1	6	0	1	0	0
EABT20944	4	3	2	3	8	0	1
EABT20945	1	0	13	0	0	0	1
EABT20946	172	35	2	4	1	13	0
EABT20947	26	11	2	1	3	0	14
EABT20948	1	4	0	0	0	1	2
EABT20949	213.99	482.1	426.93	182.08	843.54	86	305.54
EABT2095	0	5	4	1	0	0	2
EABT20950	1	0	15	1	1	0	0
EABT20951	670	1326	281	484	497	1133.01	1496.31
EABT20952	2862.08	3379.99	3735.78	3207	2276.02	2767	2853.75
EABT20953	4	10	37	76	8	5	4
EABT20954	7198.54	9400.62	10090	5402.37	7612.97	4249.08	6176.04
EABT20955	10528	5717.61	2755.25	4648.7	2808.22	11614.4	8545.23
EABT20956	611	886	432	1222	515.09	513	602
EABT20957	0	0	0	0	0	0	0
EABT20958	0	0	6	8	0	0	1
EABT20959	4249.1	6680.4	6331.43	20722	3823.59	3841.61	4395.51
EABT2096	2637.48	3824	4717.98	5580.23	2114.22	2163	1930
EABT20960	1	1	8	9	2	0	1
EABT20961	2	9	11	6	2	0	1
EABT20962	0	3	8	0	1	1	1
EABT20963	0	2	2	6	0	2	1
EABT20964	5	1	2	2	0	0	0
EABT20965	0	0	4	8	0	0	1
EABT20966	563.37	1395.02	2317	3323.7	1107.36	656.01	675.8

EABT20967	4	7	20	9	10	2	7
EABT20968	0	2	2	2	0	0	1
EABT20969	8085.17	18549.6	10452.4	10033.9	6390.48	6495.67	8668.22
EABT2097	0	1	2	0	0	0	0
EABT20970	0	2	8	4	1	0	2
EABT20971	1	0	1	0	2	3	1
EABT20972	6243.67	9696.44	7272.12	6470.62	6216.29	2844.62	3963.21
EABT20973	0	0	4	0	0	0	0
EABT20974	1	2	22	2	1	1	0
EABT20975	0	0	9	7	1	0	0
EABT20976	0	2	7	7	2	2	0
EABT20977	4	1	8	1	0	4	1
EABT20978	356	1003.99	1673	15889.4	768.99	276	564
EABT20979	1	0	2	0	0	0	1
EABT2098	1	1	35	6	1	0	1
EABT20980	0	0	6	0	0	0	0
EABT20981	0	0	6	0	0	0	0
EABT20982	0	0	0	2	4	0	27
EABT20983	0	0	1	1	0	0	0
EABT20984	0	0	5	4	3	0	3
EABT20985	0	0	0	3	6	0	1
EABT20986	3	5	8	0	0	0	1
EABT20987	0	2	30	0	0	0	1
EABT20988	5	17	84	4	9	2	13
EABT20989	1	4	7	4	2	0	2
EABT2099	583	1489.03	2752.4	3118.28	1386.75	91	566.66
EABT20990	3	3	8	8	0	3	8
EABT20991	2	1	25	0	7694	0	4
EABT20992	1018.99	1344	834.25	1521.98	1095.24	765	777.67
EABT20993	4	1	4	1	0	6	0
EABT20994	1	6	2	1	2	0	0
EABT20995	2	2	6	2	0	0	0
EABT20996	1477.41	2316.55	242	1380.79	650.64	1004.09	1571.87
EABT20997	10	9	36	2	0	1	1
EABT20998	2788.88	4128.28	2930.22	5477.68	3600.04	3004.12	4589.79
EABT20999	0	1	6	0	5	3	3
EABT21	4	5	17	29	14	2	6
EABT210	0	1	1	4	0	0	2
EABT2100	1	5	9	6	1	2	2
EABT21000	0	0	3	0	0	0	0
EABT21001	0	2	11	1	1	1	0
EABT21002	1	0	1	2	2	1	0
EABT21003	4	2	2	1	3	1	3
EABT21004	608	1224	560.05	2417.24	648	364	498.78
EABT21005	1	0	16	0	0	0	0
EABT21006	0	0	76	0	0	0	0
EABT21007	6042.72	10164.3	8054.89	9753.32	6472.08	5554.76	4803.54
EABT21008	1	9	14	8	5	0	3
EABT21009	6301.25	12493.1	13252.8	26885.8	9031.98	9320.2	9155.43
EABT2101	1	3.15	15	7	5	0	1
EABT21010	16	13	8	12	2	27	25

EABT21011	1	0	6	2	0	0	0
EABT21012	2	0	4	3	1	5	1
EABT21013	0	0	4	1	2	0	2
EABT21014	5	10	22	5	1	6	4
EABT21015	8	42	26	3	6	2	7
EABT21016	0	1	0	0	0	0	0
EABT21017	0	0	1	0	1	0	0
EABT21018	0	0	7	0	0	0	0
EABT21019	0	1	39	0	0	0	0
EABT2102	42	38	34	14	13	0	0
EABT21020	2	2	6	0	0	0	0
EABT21021	0	1	21	0	0	1	0
EABT21022	1	11	66	18	3	1	0
EABT21023	0	0	11	1	1	1	2
EABT21024	1	1	4	2	1	0	0
EABT21025	1	4	1	2	0	0	0
EABT21026	1	1	3	0	0	0	0
EABT21027	4377.29	7035.24	6901.33	13389.3	3721.06	4665.16	2399.12
EABT21028	1	1	1	5	6	1	0
EABT21029	15	20	0	0	0	1	0
EABT2103	0	0	5	1	0	3	0
EABT21030	4	4	4	5	6	1	10
EABT21031	9	13	12	15	1	7	6
EABT21032	2704.07	4874.8	6152.43	10124.2	2212.72	2513.03	2510.01
EABT21033	2	1	5	0	1	1	0
EABT21034	0	1	5	0	0	0	0
EABT21035	0	0	11	0	0	0	0
EABT21036	0	6	0	2	3	1	4
EABT21037	0	5	10	4	1	0	0
EABT21038	0	1	5	0	0	3	0
EABT21039	237	488.03	678.37	2011.13	576.68	159.13	161.92
EABT2104	1478.96	1915.9	954.28	1380.5	1341.92	1092.96	1218.44
EABT21040	144	43	450.67	358.01	524.04	138.27	106
EABT21041	24.11	32	172	92.01	42	1	9.99
EABT21042	151.03	207	195.46	378.42	162.08	96.67	150.49
EABT21043	14	15	1	2	0	0	4
EABT21044	0	0	0	0	0	0	0
EABT21045	877.01	1085.62	1230.73	1623.15	1112.01	895.38	849.93
EABT21046	1	0	8	0	0	0	0
EABT21047	2	3	0	1	0	1	0
EABT21048	2	1	12	4	5	0	0
EABT21049	1215.64	841.36	155.31	52	335.66	1332.12	1724.76
EABT2105	0	1	7	0	0	0	0
EABT21050	0	0	5	2	0	0	0
EABT21051	0	0	5	0	0	0	1
EABT21052	6	6	3	17	3	0	6
EABT21053	31	20	472	26	12	30	10
EABT21054	2	3	13	0	1	0	1
EABT21055	2	2	5	0	1	0	0
EABT21056	7	2	97	8	7	5	9
EABT21057	509.11	906.16	601.69	1399.59	790.48	428.42	602.72

EABT21058	1	2	13	0	1	0	0
EABT21059	0	0	5	3	0	0	0
EABT2106	1	0	21	5.02	25	0	0
EABT21060	0	0	7	12	0	0	0
EABT21061	0	0	5	1	0	1	0
EABT21062	23	13	3	0	3	15	12
EABT21063	1	3	3	1	3	2	3
EABT21064	5663.58	5757.04	2559.22	3433.87	15371.1	4374.21	3463.32
EABT21065	2	0	4	1	0	0	0
EABT21066	3328.45	5467.74	8386.1	9952.36	3453.74	2819.63	3084.36
EABT21067	2	3	2	1	1	1	1
EABT21068	409.86	1875.39	3050.72	1051.39	898.82	84	319.26
EABT21069	2	9	5	14	29	2	24
EABT2107	99	234	258.08	773.62	301	56	26
EABT21070	188	224	121	60	174	144	244
EABT21071	98	184	456.58	431.49	76	59	123
EABT21072	1	0	4	0	0	1	0
EABT21073	46	67	9	6.97	7	7	8
EABT21074	4	22	30.93	54	3	2	6
EABT21075	0	0	3	0	4	0	1
EABT21076	1	0	1	1	0	2	0
EABT21077	0	0	1	1	2	0	0
EABT21078	2	2	5	1	5	3	4
EABT21079	1	1.01	13	2	0	0	0
EABT2108	1432.47	1990.86	1332.82	2358.49	1251.61	838.02	931.03
EABT21080	12	11	26	29	20	8	15
EABT21081	4	11	0	0	3	5	4
EABT21082	0	0	4	2	0	4	0
EABT21083	1	2	3	0	0	0	0
EABT21084	33.1	55	71.21	47	111.31	10	17
EABT21085	1	0	6	0	0	0	0
EABT21086	0	2	3	2	0	0	0
EABT21087	0	0	5	3	0	0	0
EABT21088	3	2	31	5	2	0	1
EABT21089	1	20	11	3	7	12	3
EABT2109	0	0	6	0	0	0	0
EABT21090	20	81	188	3736.98	112	2	2
EABT21091	0	4	7	2	1	0	0
EABT21092	1	19	25	3	3	19	4
EABT21093	0	0	9	6	0	2	0
EABT21094	1	4	16	1	2	0	4
EABT21095	0	2	1	2	1	0	1
EABT21096	0	4	27	2	11	1	1
EABT21097	1	1	1	2	1	1	1
EABT21098	3	2	10	10	1	1	2
EABT21099	63.95	7	6	3	2	181.02	35.99
EABT211	1575.95	2689.82	5974.69	2754.63	1029.07	1509.3	626.93
EABT2110	17	61	50	143	43	17	36
EABT21100	36	34	75.03	61	39	31	39
EABT21101	146	227	210	422	258	25	48
EABT21102	0	4	1	0	3	0	0

EABT21103	1	0	0	0	1	34	0
EABT21104	0	1	15	2	3	0	4
EABT21105	6.98	10	52.21	9	3	11	15
EABT21106	1	0	9	1	0	0	0
EABT21107	1213.01	1170	546.94	955.99	1124	1076	807
EABT21108	0	0	3	2	0	0	0
EABT21109	0	1	0	0	1	3	2
EABT2111	70.88	152.05	201.09	1349.44	705.5	46	67
EABT21110	689	1495.52	835.12	4396.54	950.02	792	569
EABT21111	0	0	0	0	0	0	0
EABT21112	3	12	0	0	1	16	13
EABT21113	1	4	21	1	3	1	1
EABT21114	60	74.99	132	229.06	159	46.21	151
EABT21115	1929.9	3050.47	2504.4	6704.46	2698.59	2732.63	2376.23
EABT21116	2	2	21	10	4	2	1
EABT21117	9	16	1	1	0	0	0
EABT21118	0	0	3	8	0	0	0
EABT21119	0	1	7	2	1	3	0
EABT2112	1	1	4	5	0	0	0
EABT21120	2	4	0	0	0	0	0
EABT21121	0	0	8	0	0	0	0
EABT21122	10087.2	8502.72	1403	1531.96	4635	1794.01	3557.01
EABT21123	97	192.25	129.99	269.83	50	91	51
EABT21124	9611.45	11995.3	7696.86	36738.6	4071.5	2421.13	2845.58
EABT21125	376.02	793.39	468.91	52	535	10	254
EABT21126	0	0	4	3	1	0	0
EABT21127	0	1	12	2	1	0	0
EABT21128	40	75	70	159	78	46	67
EABT21129	0	0	0	1	0	4	2
EABT2113	634.02	707.85	398.98	898.1	598.93	480	376.24
EABT21130	3	1	24	2	1	4	1
EABT21131	69.24	214	407	207	328	37.03	179
EABT21132	1481.73	3962.97	3415.96	6043.93	1604	908	1196.98
EABT21133	0	2	7	0	0	0	1
EABT21134	0	2	2	2	2	0	1
EABT21135	0	2	9	2	2	0	4
EABT21136	1662.34	1848	1474.83	1790.8	1175	1474.99	839.91
EABT21137	3	2	3	1	1	1	0
EABT21138	5341.55	7966.94	3841.81	1558.96	1401.11	2913.88	4532.42
EABT21139	2	5	26	0	2	0	0
EABT2114	2	2	15	2	0	1	0
EABT21140	4	12	55	13	2	1	1
EABT21141	1	2	6	1	0	0	0
EABT21142	2	10	13	3	2	8	4
EABT21143	3128.79	3063.45	1154.99	836.99	1761.98	725.99	1410.96
EABT21144	2	1	4	0	4664.75	1	1
EABT21145	0	3	6	1	1	0	0
EABT21146	10	12	17	6782.22	33	0	32
EABT21147	3292.47	7410.81	1153	4487.82	1901.13	2126.68	3292.59
EABT21148	602.48	851.99	1598.88	1392.84	697.01	449	454
EABT21149	349.91	467.99	586.31	1146.84	553.34	310.42	271.99

EABT2115	0	3	2	3	0	0	0
EABT21150	393	714	613.94	2094.55	502	222.95	200.05
EABT21151	3	5	15	0	0	0	0
EABT21152	823.04	1349.17	1604.77	1924.45	933	879	1104.7
EABT21153	1	3	28	4	12	6	6
EABT21154	5	7	19	9	2	6	5
EABT21155	1	1	11	13	1	0	0
EABT21156	0	0	0	5	0	0	0
EABT21157	662.79	2266.15	3446.7	1566.01	2087	1908	1292
EABT21158	516	470	83	26	24	1	3
EABT21159	2	5	0	0	0	0	0
EABT2116	0	13	12	32	17	6	19.98
EABT21160	297	716.59	522	1275.47	472	161	248.95
EABT21161	0	1	2	1	0	0	0
EABT21162	8	14	2	1	1	1	0
EABT21163	0	4	14	0	0	0	0
EABT21164	4	4	13	5	2	26	16
EABT21165	218.02	345.98	266.96	752.96	492.67	116.11	488.39
EABT21166	1484.02	1817.3	1662.14	1558.02	1218.73	1450	761.99
EABT21167	0	0	13	7	0	1	0
EABT21168	1	0	1	3	2	1	0
EABT21169	0	0	3	1	0	0	0
EABT2117	37	51	47	104	44	15	13
EABT21170	0	0	2	0	2	0	0
EABT21171	0	0	10	1	1	0	0
EABT21172	2	1	35	1	0	0	2
EABT21173	0	3	0	0	1	1	0
EABT21174	0	0	0	0	0	0	0
EABT21175	3	6	13	46	15	2	12
EABT21176	0	0	5	0	1	0	0
EABT21177	0	2	2	5	2	2	3
EABT21178	0	2	14	0	0	1	0
EABT21179	0	0	1	0	0	0	0
EABT2118	0	2	8	6	1	2	1
EABT21180	31	1	1	0	1	46.3	11
EABT21181	2424.62	5680.55	5332.84	2118	1504.35	640.52	1288.84
EABT21182	0	0	0	0	0	4	0
EABT21183	0	3	2	4	2	0	0
EABT21184	1	2	5	4	0	2	0
EABT21185	0	1	46	2	0	1	1
EABT21186	1	0	9	0	0	0	0
EABT21187	20	10	3	0	1	17	6
EABT21188	0	0	6	0	0	0	0
EABT21189	3	2	6	1	0	0	0
EABT2119	4	3	14.42	8	2	1	2
EABT21190	50	39	5	45	0	35	14
EABT21191	28	60	29	131	18	17	30.94
EABT21192	0	7	1	2	1	0	0
EABT21193	0	1	7	3	0	0	0
EABT21194	0	0	23	1	0	2	3
EABT21195	3	4	12	9	0	0	0

EABT21196	0	0	0	0	1	1	3
EABT21197	7	13	35	8	4	3	5
EABT21198	63.99	25	0	0	1	2	10
EABT21199	0	1	1	2	0	0	0
EABT212	9	2	51	2	1	0	2
EABT2120	1	0	12	2	0	0	0
EABT21200	0	0	13	0	0	0	0
EABT21201	0	0	16	0	0	0	0
EABT21202	786.02	1318.75	1055.01	2105.7	1791.99	724.16	830.03
EABT21203	7	9	11	26	1	8	10
EABT21204	10	6	7	1	4	2	17
EABT21205	5	7	4	3	1	2	1
EABT21206	0	0	8	0	0	0	0
EABT21207	0	3	4	9	10	0	0
EABT21208	0	0	0	0	0	0	0
EABT21209	0	2	1	3	1	1	1
EABT2121	0	0	2	4	0	0	0
EABT21210	0	1	3	3	0	0	0
EABT21211	1	1	2	4	0	0	0
EABT21212	1	0	3	0	1	2	0
EABT21213	2	5	29	2	4	2	1
EABT21214	0	0	6	2	3	0	0
EABT21215	0	1	4	2	2	1	1
EABT21216	70	56.19	5	0	1	70	0
EABT21217	0	3	4	1	0	0	0
EABT21218	0	0	0	13	1	0	0
EABT21219	0	0	4	0	2	0	1
EABT2122	1663.7	5502.58	3844	5232.92	1713.33	1285.74	2688.18
EABT21220	18	39	58	26	11	56	17
EABT21221	13	15	1	0	4	17	9
EABT21222	0	3	26	2	0	0	0
EABT21223	0	0	8	0	0	0	0
EABT21224	3	21	32	9	5	3.93	6.97
EABT21225	1	1	21	0	0	0	1
EABT21226	1	4	19.16	1	1	0	1
EABT21227	162	272	85	827.95	102	206	94
EABT21228	2	0	4	0	0	0	0
EABT21229	0	1	3	3	0	1	1
EABT2123	0	3	5	0	0	0	0
EABT21230	6	0	0	1	0	2	0
EABT21231	3	4	48	22	2	1	3
EABT21232	164332	163062	29073.6	7443.22	43618.1	16004.3	64784.7
EABT21233	0	0	26	0	0	0	0
EABT21234	2	2	1	0	0	0	3
EABT21235	0	1	9	0	0	0	0
EABT21236	0	2	6	0	0	0	0
EABT21237	2	6	35	1	0	2	0
EABT21238	7	4	1	2	0	0	0
EABT21239	5690.62	7922.93	9074.44	14794	6699.02	6266.89	6922.34
EABT2124	3	3	14	3	0	1	0
EABT21240	0	1	8	3	0	0	0

EABT21241	1275.29	2216.16	4147.98	2329.56	2109.53	733.9	798.07
EABT21242	52.3	86.01	21	118.34	71.02	8.96	56.97
EABT21243	0	0	5	0	0	3	1
EABT21244	1	3	14	1	0	2	3
EABT21245	1	1	8	4	1	0	1
EABT21246	819.29	1479.84	1928.4	1463.06	1696.31	592.86	930.23
EABT21247	10	11	48	18	12	4	9
EABT21248	1	6	10	4	0	3	6
EABT21249	95	67	28	115	3311	33	16
EABT2125	0	0	1	4	0	0	1
EABT21250	1	4	0	0	0	1	0
EABT21251	780.93	2306.18	4737.19	5154.45	8599.89	36	682.97
EABT21252	14	92	651	4	3	1	1
EABT21253	10360.6	10774.2	11836	7053.93	12559.3	3684.99	4584.76
EABT21254	16	0	0	0	0	1	1
EABT21255	2	6	0	1	0	0	0
EABT21256	1	0	4	105	0	0	1
EABT21257	117.98	147.01	145.05	280.76	78	45	51
EABT21258	8	4	79	25	2	6	7
EABT21259	0	4	12	3	11	0	5
EABT2126	0	2	6	5.97	0	3	0
EABT21260	2	3	0	1	2	0	0
EABT21261	0	0	10	0	0	0	0
EABT21262	119.01	116	153.41	1164	213	9	80
EABT21263	0	2	1	0	0	0	0
EABT21264	6790.09	4552.66	976.06	1448.75	1864.9	0	88.92
EABT21265	0	1	12	0	0	0	0
EABT21266	3	4	13	5	1	4	1
EABT21267	0	1	0	1	2	0	3
EABT21268	0	5	10	7	0	0	0
EABT21269	16	25	20	29	7	6	5
EABT2127	3131	2438.87	845	1458.68	1084	1842	1592.06
EABT21270	36	165.01	279	1778.99	69	2	21
EABT21271	0	0	5	0	0	0	0
EABT21272	1	2	7	1	3	0	0
EABT21273	30	101	28	47	7	1	2
EABT21274	2	3	5	0	0	0	0
EABT21275	1	0	4	3	0	2	1
EABT21276	0	1	17	0	0	0	0
EABT21277	2	17	9	2	4	1	3
EABT21278	0	0	6	1	0	0	0
EABT21279	0	1	5	0	0	0	0
EABT2128	0	0	3	1	0	0	0
EABT21280	100	166	58.3	287.04	160	197	180
EABT21281	454	887	1129.01	1164.67	687	415.04	497.97
EABT21282	0	0	7	46	7	0	0
EABT21283	0	3	11	0	1	0	0
EABT21284	3	1	2	0	2	0	5
EABT21285	2	2	3	2	2	0	0
EABT21286	2	3	14	7	1	2	5
EABT21287	52	114	98	135	32	49	48

EABT21288	1	2	27	0	0	0	0
EABT21289	0	0	17	0	0	0	1
EABT2129	0	0	16	3	0	0	1
EABT21290	14	22	69	24	15	18	2
EABT21291	1361.1	2067.4	4502.84	3495.91	2043.04	1649.05	1597.7
EABT21292	0	3	1	2	0	0	0
EABT21293	0	0	3	0	0	0	1
EABT21294	970.23	2363.28	2030.92	6328.61	1047.98	747.03	1034
EABT21295	4	7	10	6	3	6	5
EABT21296	0	0	2	0	20	0	0
EABT21297	3	4	4	5	1	0	9
EABT21298	1	2	1	0	0	7	5
EABT21299	96	240	607	1618.07	186	10	34
EABT213	0	4	2	4	3.92	0	1
EABT2130	4718.29	8414.33	8352.94	16671.8	7619.39	6200.05	4542.89
EABT21300	0	1	64	0	0	0	0
EABT21301	1183.63	806.94	88	68	647.16	0	19
EABT21302	0	1	1	0	2	1	0
EABT21303	1	3	5	0	0	1	0
EABT21304	0	1	6	3	0	0	0
EABT21305	1	0	6	1	0	1	0
EABT21306	1	6	80.9	5	0	1	2
EABT21307	22	18	0	0	2	0	4
EABT21308	0	2	0	7	0	0	0
EABT21309	0	1	24	0	1	0	0
EABT2131	0	0	18	0	1	0	2
EABT21310	4	6	15	17	4	7	4
EABT21311	2	1	5	1	0	0	0
EABT21312	0	7	1	1	1	0	1
EABT21313	0	5	12	1	0	0	0
EABT21314	1	1	2	0	2	0	0
EABT21315	3460.75	4083.26	10743.8	6957.95	3584.28	3595.97	3014.83
EABT21316	1	5	0	0	1	30	4
EABT21317	0	6	2	8	1	0	1
EABT21318	1637.71	19168.5	2169.97	25.58	815.67	3691.18	2023.54
EABT21319	6	13	9	42	3	0	0
EABT2132	0	1	0	2	0	2	0
EABT21320	0	0	1	1	0	0	3
EABT21321	1233.3	2108.55	2365.4	5114.09	1429.14	709.94	796.1
EABT21322	124.02	88	8	11	94	189	571
EABT21323	0	5	1	5	0	0	0
EABT21324	0	2	0	0	57	0	0
EABT21325	23.32	25.05	0	0	1	7	3
EABT21326	18	38	103	17	11	3	0
EABT21327	2380.16	3026.84	3077.23	5074.63	1852.95	1543	1237
EABT21328	4	1	0	68	0	2	0
EABT21329	0	0	0	1	0	2	0
EABT2133	15	9	8	15	6	10	9
EABT21330	3	3	14	15.01	7	1	0
EABT21331	2	5	4	8	0	2	1
EABT21332	1	2	7	0	0	0	0

EABT21333	19	15	60	10	2	12	7
EABT21334	2	0	3	0	0	0	1
EABT21335	0	0	1	1	1	0	0
EABT21336	1	1	26	3	4	0	0
EABT21337	1	2	40	2	1	1	1
EABT21338	0	0	7	4	0	0	0
EABT21339	0	1	0	5	1	0	0
EABT2134	2	1	0	1	2	3	0
EABT21340	0	1	1	2	4	0	4
EABT21341	0	0	1	10	0	0	0
EABT21342	77.88	74	14	31	0	57	68.7
EABT21343	1	0	6	1	0	0	0
EABT21344	0	0	3	1	6	0	0
EABT21345	0	0	2	1	1	0	2
EABT21346	0	0	3	2	0	1	1
EABT21347	0	0	8	0	0	0	0
EABT21348	3	7	13	5	1	1	0
EABT21349	35	104.03	200	106	35	2	8
EABT2135	1	3	5	1	0	0	1
EABT21350	1	4	10.12	6	0	1	0
EABT21351	0	1	0	0	0	0	0
EABT21352	0	1	12	0	0	0	0
EABT21353	0	5	8	1	2	0	0
EABT21354	0	0	10	1	0	0	0
EABT21355	666.75	828.33	1029.54	899.9	989.55	503.67	467.92
EABT21356	3	4	6	26	2	1	0
EABT21357	420	938	415.88	3201.21	538.13	251	252.03
EABT21358	0	2	7	0	1	0	2
EABT21359	1	2	3	3	0	1	1
EABT2136	5037.62	4141.6	1431.91	2601.69	8616.6	4361.84	3649.36
EABT21360	0	15	2	1	12	21	66
EABT21361	0	2	9	8	0	0	0
EABT21362	2	8	21	13	0	12	19
EABT21363	2	3	8	2	1	0	0
EABT21364	1	2	10	7	1	0	1
EABT21365	8	8	3	1	3	24	1
EABT21366	0	0	0	0	0	0	0
EABT21367	3481.59	3387.63	4081	5367.81	2124.51	3436.47	3171.68
EABT21368	4	2	15	0	11	5	24
EABT21369	767	1350.49	1554.01	1334.09	3354.31	455	965
EABT2137	2	0	55.85	3	0	0	0
EABT21370	0	3	31.36	3	0	0	0
EABT21371	45	47	45	5	11	73	57
EABT21372	0	2	3	1	0	0	1
EABT21373	0	2	2	1	0	0	0
EABT21374	1	1	9	3	2	0	0
EABT21375	0	1	3	7	1	0	0
EABT21376	0	8	10	1	2	0	0
EABT21377	0	0	9	1	0	0	0
EABT21378	48.95	337.07	53	1173.89	267	33	32
EABT21379	3	3	5	8	1	0	0

EABT2138	58	106	77.02	22	142.63	4	10
EABT21380	2	1	6	1	1	1	0
EABT21381	25	2	0	0	1	38	1
EABT21382	0	0	0	4	5	0	0
EABT21383	0	2	8	1	0	1	0
EABT21384	0	2	10	7	4	1	1
EABT21385	11	11	29	1	2	0	0
EABT21386	0	1	2	2	1	0	3.28
EABT21387	0	0	1	0	0	0	0
EABT21388	1	2	0	9	3	0	0
EABT21389	4	0	0	0	0	11	23
EABT2139	14	7	17	26	1	9	1
EABT21390	1	1	16	1	1	1	1
EABT21391	2	1	2	0	0	0	0
EABT21392	1	1	2	1	0	0	1
EABT21393	0	0	8	0	1	0	0
EABT21394	2	2	5	22	2	0	0
EABT21395	0	3	10	1	1	1	4
EABT21396	414.03	596.58	1046.71	870.9	545.04	227.06	408.74
EABT21397	2	4	11	0	1	2	2
EABT21398	0	28	51	120	0	0	0
EABT21399	2	5	24	13	5	0	1
EABT214	2.01	5	0	0	2	68	19
EABT2140	2	3	1.42	1	0	1	0
EABT21400	12	22	0	0	1	0	1
EABT21401	2	1	3	0	5	1	1
EABT21402	1	1	5	0	0	0	0
EABT21403	948.02	1555.03	1147.2	2707.01	1139	515.01	727.68
EABT21404	0	0	17	2	0	0	0
EABT21405	0	1	7	1	0	1	1
EABT21406	0	2	2	4	1	0	1
EABT21407	0	0	21	0	1	0	0
EABT21408	14	30	69	6	2	8	14
EABT21409	1	1	8	1	1	0	1
EABT2141	1	4	50	9	4	0	1
EABT21410	2	2	0	0	0	0	0
EABT21411	1	0	3	0	0	0	0
EABT21412	2	2	1	0	2	0	5
EABT21413	0	0	2	1	0	0	0
EABT21414	3	24	10	1	152	4	8
EABT21415	18	72.48	173.97	229	200	6	76.02
EABT21416	0	1	5	1	0	1	1
EABT21417	0	0	9	19	0	0	1
EABT21418	0	0	3	0	0	0	0
EABT21419	1	2	10	0	1	0	1
EABT2142	0	0	1	22	1	0	0
EABT21420	0	5	7	13	0	0	0
EABT21421	19091	27918.7	8548.21	33768.2	11539.6	5849.56	5110.05
EABT21422	2	0	9	11	1	1	2
EABT21423	10253.1	10580.8	7377.85	10014.4	9676.51	15870.4	10753.1
EABT21424	2	1	1	1	2	0	0

EABT21425	2837.26	7892.74	11695.4	11174.6	6529.38	1902.11	3442.3
EABT21426	0	5	5	3	1	1	11
EABT21427	7	53.98	13	5	2	5	4
EABT21428	0	0	0	0	0	0	8
EABT21429	154	737	1139.96	457	115.25	8	26
EABT2143	297	371	178.99	442	342	150	198
EABT21430	5	5	0	0	4	40	19
EABT21431	0	4	2	0	1	0	0
EABT21432	83	72	58	29	31.99	141	169.07
EABT21433	920	1511.7	696.56	1465.88	506.99	102	301.11
EABT21434	5	2	2	2	0	3	4
EABT21435	11	24	14	32	5	17	5
EABT21436	5	0	3	2	3	0	0
EABT21437	4453.53	5685.95	4198.26	6370.26	3749.79	4015.67	4318.6
EABT21438	8	11	3	0	0	0	1
EABT21439	0	1	2	1	0	0	1
EABT2144	12839.5	11271.4	6253.88	13130	10943.5	19299.8	15592.8
EABT21440	6	36	119.03	24	169	3	27
EABT21441	0	4	28	5	9	0	2
EABT21442	807.96	784.3	1138.89	981.99	814.08	1085.94	517
EABT21443	0	2	0	3	1	2	1
EABT21444	36	84	37	201	27	18	26
EABT21445	4	36	84	25	2	1	5
EABT21446	1	1	1	0	0	0	0
EABT21447	21	27	118	73	8	27	17
EABT21448	2	1	2	36	0	3	2
EABT21449	270.1	467	253	887.95	322	90	134
EABT2145	0	1	14	6	2	3	1
EABT21450	0	1	6	1	0	0	0
EABT21451	3	7	4	16	16	0	2
EABT21452	1	2	5	4	0	1	1
EABT21453	0	3	6	4	3	3	0
EABT21454	2	5	8	0	0	1	0
EABT21455	33	121	2	1	0	24	0
EABT21456	13	14	119	11	18	6	10
EABT21457	8	0	0	2	0	29	0
EABT21458	2	1	1	2	2	0	0
EABT21459	0	1	3	2	0	0	0
EABT2146	0	1	5	1	0	0	0
EABT21460	0	6	4	0	4	1	0
EABT21461	0	2	4	0	0	2	6
EABT21462	1	0	1	0	85	2	0
EABT21463	0	1	8	5	0	0	0
EABT21464	1	0	16	0	0	1	0
EABT21465	901.22	1291.47	1211.27	4723.67	1760.96	876.03	907.89
EABT21466	19458.5	45151.4	80628.6	46187	18019.9	5002.6	4770.24
EABT21467	1	2	9	0	0	0	0
EABT21468	0	0	9	0	0	0	0
EABT21469	0	1	5	0	0	0	0
EABT2147	0	0	8	4	0	0	0
EABT21470	15	33	28	10	4	1	7

EABT21471	2	1	42	1	2	0	0
EABT21472	3	11	17	23	7	4	0
EABT21473	28	21	20	6	42	5	52.58
EABT21474	12	15	42	56	5	8	8
EABT21475	130	162	324	331.01	56	56	51
EABT21476	3557.27	6129.8	8591.38	8115.99	4324.67	2630.8	3382.82
EABT21477	0	1	2	17	2	1	0
EABT21478	1	6	98	0	0	0	2
EABT21479	1	2	2	3	0	2	1
EABT2148	2251.65	2951.72	4779.24	3980.99	2891.55	2723.65	3027.23
EABT21480	0	16	11	19	3	5	15
EABT21481	0	0	2	4	1	0	0
EABT21482	0	1	17	18	2	2	1
EABT21483	1	3	10	38	10	0	1
EABT21484	46	61	15	1	4	22	5
EABT21485	0	1	8	1	0	0	0
EABT21486	43	60	59	82	48	22	39
EABT21487	0	3	9	8	5	0	0
EABT21488	15173.7	13608.1	17674.2	5160.52	5549.37	10609.2	2583.92
EABT21489	1	4	6	1	1	0	1
EABT2149	20762.8	3184.55	150	7	438.96	31816.5	421
EABT21490	1	2	7	0	0	1	0
EABT21491	1	1	4	0	1	14	2
EABT21492	27	46	32	75	42	8	11.13
EABT21493	191	612.82	488.04	523.3	6294.43	30	243
EABT21494	0	2	0	3	0	4	0
EABT21495	10	8	27	28	8	6	7
EABT21496	1	8	12	12	12	0	0
EABT21497	2	4	1	5	2	1	6
EABT21498	0	0	4	0	1	0	0
EABT21499	1.99	1	1	0	0	0	0
EABT215	0	0	2	0	0	2	0
EABT2150	1	2	77	4	0	1	0
EABT21500	1	2	5	53	13	0	0
EABT21501	0	1	6	0	0	0	1
EABT21502	0	1	8	0	0	0	1
EABT21503	1	1	2	1	0	0	0
EABT21504	0	0	10	0	1	0	0
EABT21505	0	0	5	1	0	0	0
EABT21506	0	1	3	4	0	0	0
EABT21507	160.2	92.99	185	11	6	79	35.9
EABT21508	1	3	4	5	9	0	0
EABT21509	0	0	10	18	2	1	0
EABT2151	1074.04	1652.85	1698.49	3049.18	1568.54	1196	1261.81
EABT21510	3	1	4	0	1	3	0
EABT21511	0	0	4	0	0	0	1
EABT21512	3	2	12.01	0	0	1	2
EABT21513	0	0	0	0	7	1	10
EABT21514	1833.88	3387.23	2745.89	4769.63	1619.28	1215.92	1624.34
EABT21515	6	14	1	2	2	57	1
EABT21516	7	14	16	16	6	7	3

EABT21517	380.15	1075	935.57	1572.19	1117	114	335.98
EABT21518	0	1	8	1	3	0	1
EABT21519	28183.2	36598.3	31707.6	31212.6	20136.9	14097.9	21467.6
EABT2152	0	2	0	3	0	0	0
EABT21520	1	0	11	1	0	0	0
EABT21521	1	10	2	5	1	2	0
EABT21522	3	0	5	0	1	0	0
EABT21523	2	2	3	2	0	0	1
EABT21524	33	19	18	35	3	27	4
EABT21525	16	44	1	0	0	0	1
EABT21526	30	40	17	12	6	32	7
EABT21527	1	2	40	2	1	0	2
EABT21528	12	15	3	6	2	12	31
EABT21529	0	3	8	0	0	0	0
EABT2153	0	2	0	25	4	0	0
EABT21530	244.99	278	305.86	399.98	295.92	159	93
EABT21531	0	0	26	0	0	0	1
EABT21532	0	1	3	7	0	0	2
EABT21533	1262	599	29.95	8	57	1078.18	354
EABT21534	0	0	22	0	0	0	0
EABT21535	3	22	10	65	8	0	0
EABT21536	0	3	9	0	0	0	0
EABT21537	846.98	1248.92	1463.25	4007	1233.63	886.03	980
EABT21538	57	35	297	39	21	0	6
EABT21539	139	200.05	322	275	648.2	37	193.97
EABT2154	3	5	4	9	5	0	0
EABT21540	0	1	4	4	0	0	0
EABT21541	1	0	7	0	0	0	0
EABT21542	0	1	1	1	0	0	0
EABT21543	0	2	44	1	2	0	3
EABT21544	0	1	2	1	0	1	0
EABT21545	4	3	15	8	6	0	2
EABT21546	0	1	2	31	4	0	1
EABT21547	1	4	28	0	1	0	0
EABT21548	6	2	1	1	5	0	0
EABT21549	3861.6	5074.67	4907.09	10652.8	3767	4244.62	2777.35
EABT2155	1	1	13	8	1	0	2
EABT21550	565	561.99	586	1180	448	394	226
EABT21551	1	9	4	6	4	0	2
EABT21552	17	33	42	48	43	10	21
EABT21553	0	4	2	2	1	0	1
EABT21554	0	0	7	8	1	0	0
EABT21555	0	0	14	0	0	0	0
EABT21556	3580.87	4791.77	2092.69	1581.87	3248.99	3194.57	4117.47
EABT21557	0	0	5	0	1	0	1
EABT21558	0	2	24	1	1	0	1
EABT21559	2906.73	4072.39	2526.87	1805	2009.4	1789	2665
EABT2156	0	3	7	0	1	0	0
EABT21560	200.72	606.07	149	190.99	227	101.97	747.32
EABT21561	4427.14	7195.03	1656.18	1198.87	1898	6087.93	8155.51
EABT21562	0	0	6	2	0	1	8

EABT21563	1	3	25	13	3	8	2
EABT21564	1	0	3	3	2	0	0
EABT21565	17	80	36	168	262	0	1
EABT21566	0	2	1	17	7	0	1
EABT21567	2	1	29	2	2	5	1
EABT21568	0	1	7	0	0	0	0
EABT21569	0	0	11	1	0	0	0
EABT2157	3	11	1	1	30	0	0
EABT21570	0	0	5	1	0	0	0
EABT21571	924.84	1561	893	4105.02	1053.62	822.09	545
EABT21572	2007	3003	4990.78	2900.75	1209	5	86
EABT21573	0	3	71.94	2	1	3	3
EABT21574	0	0	7	0	1	0	1
EABT21575	3	8	4	1	4	0	0
EABT21576	3307	7786.39	6227.05	7758.17	4358.27	2622.99	2898.95
EABT21577	1	2	25	8	0	0	0
EABT21578	3	6	17	0	2	18	8
EABT21579	0	2	10	9	13	0	1
EABT2158	1	0	2	0	0	0	0
EABT21580	2	7	11	13	7	0	5
EABT21581	4	10	0	0	1	4	2
EABT21582	0	0	2	6	0	1	0
EABT21583	4	4	22	2	4	19	16
EABT21584	0	0	0	9	1	0	0
EABT21585	1	3	11	0	0	0	0
EABT21586	4	7	6	5	5	2	6
EABT21587	0	1	16	0	1	1	1
EABT21588	2	0	1	1	0	11.88	0
EABT21589	0	0	12	0	0	0	0
EABT2159	0	1	0	5	0	0	0
EABT21590	0	1	17	1	0	0	2
EABT21591	0	0	6	2	0	0	0
EABT21592	1033.46	1638.76	2144.01	1878.57	1907.15	736.79	1193.93
EABT21593	8	13	7	0	1	8	0
EABT21594	0	1	10	0	0	0	0
EABT21595	0	0	0	5	1	0	0
EABT21596	6055.6	6721.95	3237.58	5043.51	3143.27	4125.02	4597.32
EABT21597	2	3	15	0	0	0	0
EABT21598	0	0	5	1	1	0	0
EABT21599	7	3	1	4	5	0	2
EABT216	9899.27	21327.1	10701.9	15426.8	5443.19	14511.6	13427.3
EABT2160	56.01	65	47	29	23	31	30.25
EABT21600	3	2	28	2	2	7	3
EABT21601	6	35	17	50	2	5	4
EABT21602	0	1	3	0	0	0	0
EABT21603	3	0	4	0	0	0	1
EABT21604	753.03	3282	3619.62	976	4243.27	183	1219.67
EABT21605	2	2	10	1	1	0	0
EABT21606	0	11	3	0	0	0	0
EABT21607	1	3	0	0	0	0	0
EABT21608	1	0	1	0	1	0	0

EABT21609	0	0	1	1	0	0	0
EABT2161	1	3	6	1	1	3	2
EABT21610	12.05	4	2	0	2	4	0
EABT21611	1344.45	1967.6	4614	4433.31	1829.03	1553	1293.98
EABT21612	0	3	4	16	3	1	0
EABT21613	0	1	1	3	0	1	0
EABT21614	3	10	1	0	4	17	13
EABT21615	22	18	43	5	26	27	11
EABT21616	0	0	6	1	0	0	0
EABT21617	1	1	1	8	0	0	0
EABT21618	0	1	1	1	1	0	0
EABT21619	0	0	1	0	0	0	1
EABT2162	0	0	6	0	0	0	0
EABT21620	0	2	6	2	1	0	0
EABT21621	0	0	34	1	0	0	0
EABT21622	3	4	41	4	0	2	0
EABT21623	0	1	4	0	0	1	0
EABT21624	0	7	1	1	1	0	1
EABT21625	4	9	6	2	0	1	1
EABT21626	0	1	1	0	37	1	2
EABT21627	3	5	9	4	2	0	5
EABT21628	1	0	5	0	2	0	0
EABT21629	0	3	2	0	4	0	2
EABT2163	1	2	3	0	0	0	0
EABT21630	971.8	1304.17	1281.95	2099.6	1384.39	751	670
EABT21631	0	1	4	8	0	0	1
EABT21632	14	11	20	0	4	24	52
EABT21633	0	0	6	0	1	0	0
EABT21634	3	2.44	25.39	14	1	0	1
EABT21635	9473.73	5139.21	1831.94	8195.84	2621.85	1919.15	2761
EABT21636	563.54	1027.01	1441.01	4596.26	435.93	683	615
EABT21637	0	12	9	36	3	1	4
EABT21638	109	98	1	0	36	0	1
EABT21639	5265.86	4588.21	4621.88	50.88	6894.64	36	33369.4
EABT2164	0	3	2	2	0	0	0
EABT21640	2	3	5	0	1	2	3
EABT21641	0	1	0	0	1	0	0
EABT21642	1	4	0	1	0	1	1
EABT21643	16	47	62.03	27	13	46	42
EABT21644	3	1	11	0	0	1	0
EABT21645	0	0	9	22	0	0	0
EABT21646	5	9	13	5	4	2	4
EABT21647	2	7	8	12	12	0	0
EABT21648	1207.98	1801.07	1764.68	1772.92	1082.19	1130.7	749.95
EABT21649	27	19	0	0	1	28	27.41
EABT2165	0	0	4	0	0	0	0
EABT21650	41	70	1	0	5	13	48
EABT21651	8	19	0	0	1	13	7
EABT21652	0	0	17	1	0	0	2
EABT21653	1	1	5	3	0	0	0
EABT21654	17	27	73	17	10	3	12

EABT21655	1	2	1	0	1	0	0
EABT21656	0	0	1	2	6	0	1
EABT21657	1	0	1	1	0	0	1
EABT21658	15.99	12	45	42	74	7	32.92
EABT21659	0	0	1	1	9	1	0
EABT2166	0	2	12	2	0	0	1
EABT21660	0	0	0	1	1	0	3
EABT21661	6	5	15	1	1	0	2
EABT21662	2313.53	3817.5	3979.36	4280.02	2187.17	950.9	1733
EABT21663	616.06	4489.07	3503.48	5008.54	982.64	35	88
EABT21664	1	5	3	16	2	0	0
EABT21665	3	5	0	0	0	5	6
EABT21666	287.54	566.03	1127.48	2033.68	551.16	237.05	189.32
EABT21667	3	5	7	1	0	7	1
EABT21668	8	37	7	8	2	1	5
EABT21669	0	0	73	14	1	2	4
EABT2167	2029.37	3021.15	9882.5	14284.9	2351.86	1372.44	1804.72
EABT21670	0	0	3	2	0	1	0
EABT21671	946	2079.68	1490.68	1583.85	2265	227.84	1378
EABT21672	2	12	19	15	5	3	4
EABT21673	0	3	34	0	0	0	0
EABT21674	3	2	0	0	0	0	0
EABT21675	0	0	5	3	0	0	0
EABT21676	2	2	0	0	0	1	1
EABT21677	5	1	0	2	3	0	0
EABT21678	7	11	58.05	28.1	3	8	10
EABT21679	0	6	47	4	1	0	0
EABT2168	1	7	2	0	0	1	0
EABT21680	0	1	19	0	0	0	0
EABT21681	0	0	6	0	0	0	0
EABT21682	1005.21	1359.91	139.02	59	1194.59	1922.13	5588.34
EABT21683	7	14	116	1	4	15	5
EABT21684	2096.46	2035.15	898.74	1319.09	1984.3	2919.99	2040.88
EABT21685	3	8	1	0	1	6	0
EABT21686	45	59	92	45	33	1	1
EABT21687	0	2	51	1	1	10	3
EABT21688	1	3	3	1	2	0	4
EABT21689	0	1	2	1	11	0	0
EABT2169	2	1	12	1	1	0	1
EABT21690	0	0	11	0	0	0	0
EABT21691	0	3	37	9	1	4	0
EABT21692	0	0	1	3	0	0	0
EABT21693	5	9	106	20	4	6	7
EABT21694	434.05	776	1859.05	1154	823.99	138	492
EABT21695	0	1	4	0	0	0	1
EABT21696	7	17	9	5	6	25.94	8
EABT21697	1	4	9	1	0	0	0
EABT21698	244	283	316.35	203.99	196	158.06	152
EABT21699	2	2	4	2	0	0	0
EABT217	0	4	70	3	1	1	1
EABT2170	0	2	1	21	5	0	0

EABT21700	1	3	2	0	1	0	1
EABT21701	8	16	42	43	52	21	62
EABT21702	0	0	18	3	0	2	0
EABT21703	0	1	2	4	0	0	0
EABT21704	5	3	3	10	1	0	3
EABT21705	996.89	2179.52	1671.98	1382.01	3557.24	490	2061.25
EABT21706	6	7	1	19	9	3	5
EABT21707	31	73	46	15	213	21	76
EABT21708	974.8	1194	700.97	1628.93	945.06	488.01	683.33
EABT21709	1	6	57	0	8	1	5
EABT2171	898	862.02	596	109	523	1827.99	1673.01
EABT21710	10	10	22	6	6	10	8
EABT21711	0	5	44	4	1	3	0
EABT21712	11179.3	1736	11	2	1414	3115.84	4785.96
EABT21713	0	0	0	0	0	0	0
EABT21714	0	0	15	0	0	0	0
EABT21715	0	3	5	1	0	1	1
EABT21716	0	0	0	0	14	0	0
EABT21717	0	3	0	0	1	2	1
EABT21718	1	3	8	25	9.86	0	2
EABT21719	0	1	23	1	2	0	0
EABT2172	0	0	2	4	0	0	0
EABT21720	0	0	8	0	1	0	0
EABT21721	11	46	38	121.06	331	8	12
EABT21722	3	5	0	0	7	88	47
EABT21723	1	0	2	1	0	0	0
EABT21724	73	61.99	17	9	0	1	1
EABT21725	2887.56	4556.51	4958.83	7446.37	2209.29	2761.11	2419
EABT21726	2	4	11	8	2	4	6
EABT21727	1	0	4	0	1	0	0
EABT21728	1	2	11	21	5	4	4
EABT21729	1	1	7	5	0	1	0
EABT2173	0	0	13	0	0	1	0
EABT21730	0	0	9	0	8	0	0
EABT21731	3	8	7	5	2	0	1
EABT21732	1137.24	2070.07	3620.36	5923.68	3115.52	2115.11	1049.88
EABT21733	3	4	1	7	2	3	1
EABT21734	1	6	14	0	0	1	0
EABT21735	174	113	57.46	48.91	20	11	28
EABT21736	0	0	0	2	1	0	0
EABT21737	150.99	205.99	8.99	33	377.57	4	200.03
EABT21738	2	1	15	9	1	0	1
EABT21739	2	2	0	2	0	0	0
EABT2174	443	598.99	471	813	512.55	328	445
EABT21740	28	69	110.01	20	21	9	43.94
EABT21741	0	0	9	0	1	1	0
EABT21742	3	18	1	0	3	3	2
EABT21743	464.98	575.49	737.65	944.72	593.04	415	388.03
EABT21744	2625.09	3142.06	965.96	1883.24	2277	1405.85	1166
EABT21745	5	12	0	0	1	13	8
EABT21746	0	0	4	7	3	2	0

EABT21747	1899.67	2599.59	1611.61	4012.2	1427.95	1698.47	1717.41
EABT21748	1	3	13	1	2	0	3
EABT21749	1	1	1	3	0	0	0
EABT2175	0	0	3	46	1	0	0
EABT21750	2	2	2	2	5	3	1
EABT21751	878.74	1312.56	1425.62	1156.14	750	779.61	663.81
EABT21752	42	76	74.01	175.01	91	34	55
EABT21753	0	1	2	1	0	0	1
EABT21754	0	0	10	2	0	0	0
EABT21755	303.55	435.99	190	1007.5	626	74	212.29
EABT21756	19	0	1	0	0	23	0
EABT21757	0	0	6	0	0	0	0
EABT21758	3	0	1	0	2	0	0
EABT21759	1	2	3	2	0	1	0
EABT2176	3	3	3	2	0	5	1
EABT21760	0	0	1	1	1	0	2
EABT21761	3451.15	6402.16	4576.26	7458.19	4646.71	2179.36	3711.47
EABT21762	50	559	43.04	10	12	10	5
EABT21763	0	1	3	1	1	0	0
EABT21764	1	1	2	4	4	1	1
EABT21765	196	388.05	2198	4064.07	794.99	192	195.22
EABT21766	916.17	1102.04	1015	1603.03	908.98	525	521.09
EABT21767	0	1	3	0	0	2	0
EABT21768	394	1074.12	2061.11	13385.2	1000	264	243.99
EABT21769	0	0	1	0	0	3	0
EABT2177	24	66	50	2462.01	41	3	32
EABT21770	0	1	2	5	1	0	1
EABT21771	0	0	0	0	0	0	7
EABT21772	0	0	7	0	0	0	0
EABT21773	4	12	38	37	2	0	0
EABT21774	41	102	112	100.54	102.95	7	25.99
EABT21775	222.78	544.91	538.96	233.47	304.28	81	98.09
EABT21776	3	0	1	0	0	0	0
EABT21777	112.07	39	30	5	10	78.69	1
EABT21778	227	73	40	0	0	791.73	16
EABT21779	0	0	1	0	0	0	0
EABT2178	0	2	0	24	0	1	0
EABT21780	5	3	5	4	1	2	2
EABT21781	12	13	9	3	7	24	34
EABT21782	2655.65	4264.85	4572.33	2098.06	5784.1	2673.93	4111.57
EABT21783	3825.2	2132.19	463.83	902.38	1306.08	5657.31	5142.24
EABT21784	1	1	18	2	0	0	0
EABT21785	445	815	718	876.02	520	135	194
EABT21786	0	0	10	1	0	0	0
EABT21787	0	0	6	0	0	0	0
EABT21788	0	10	6	20	1	0	0
EABT21789	4429.06	5173.21	3828.38	6742.46	4294.37	2872.67	2965.19
EABT2179	1	2	2	1	0	0	0
EABT21790	2	0	0	0	1	1	0
EABT21791	797.04	1327.03	1135.84	1879.28	1081.1	896.4	1028.53
EABT21792	4	7	29	13	2	1	1

EABT21793	1	1	8	1	0	0	1
EABT21794	2	5	0	0	0	0	0
EABT21795	5450.8	6518.75	5329.08	7939.43	16673.5	4421.16	3773.02
EABT21796	6	13	4	1	2	14.5	8
EABT21797	1288.48	2408.86	2215.28	4471.62	2180.07	1534.98	1801.02
EABT21798	0	1	9	8	82	0	4
EABT21799	1	1	1	1	1	5	2
EABT218	7	22	38	154.04	26	2	5
EABT2180	7	7	9	0	5	3	1
EABT21800	0	5	7	1	1	1	3
EABT21801	1753.58	2256.38	2662.9	3382.64	1382.69	1517.42	1372.61
EABT21802	1	3	2	0	0	0	0
EABT21803	1	0	6	1	1	1	0
EABT21804	1	0	0	0	2	1	6
EABT21805	0	4	7	1	1	2	0
EABT21806	608.83	1288.56	1810.85	2483.16	1501.93	318	708
EABT21807	0	0	4	1	0	0	1
EABT21808	16	6	7	16	5	17	13
EABT21809	0	1	8	0	0	0	0
EABT2181	2135.43	3398.95	2701.86	3781.21	1942.17	1693.77	2518.69
EABT21810	1	6	0	2	0	0	0
EABT21811	2647.17	3502.14	1216.17	2763.07	1381.97	1973.76	2627.68
EABT21812	0	1	6	0	0	0	1
EABT21813	2309.62	3506.1	3658.16	5025.53	2707.04	1929.52	1909.91
EABT21814	0	0	0	0	2	0	1
EABT21815	252	235	105	242.64	318	423.66	272.88
EABT21816	1	11	0	7	0	0	1
EABT21817	281	844.1	464	1293.65	278	238.99	231
EABT21818	3	23	0	1	0	2	0
EABT21819	0	0	16	0	0	1	0
EABT2182	0	8	10	1	0	0	2
EABT21820	0	0	8	0	0	0	0
EABT21821	3	4	9	8	6	0	0
EABT21822	10	14	8	782.49	2	14	22
EABT21823	0	0	7	2	1	0	0
EABT21824	3886.91	5045.84	8242.01	8865.09	3350.17	5464.57	7704.84
EABT21825	9663.56	21472	9039.35	6164.09	5506.54	7381.01	9896.06
EABT21826	3	15	53	12	4	6	5
EABT21827	0	0	0	0	9	0	0
EABT21828	7	22	19	36	12	6	17
EABT21829	5	2	6	3	0	0	0
EABT2183	0	0	6	0	1	0	0
EABT21830	0	0	13	1	0	0	0
EABT21831	0	0	0	0	6	0	1
EABT21832	0	1	3	4	28	0	3
EABT21833	1	0	0	0	9	0	0
EABT21834	11	50	103	440.9	31	15	45
EABT21835	1	0	3	1	0	0	0
EABT21836	0	0	2	15	15	0	0
EABT21837	5	3	8	5	3	1	2
EABT21838	0	0	10	0	0	1	0

EABT21839	1	0	5	0	0	0	0
EABT2184	0	14	0	0	0	20	0
EABT21840	2	13	18	57	4	5	2
EABT21841	0	1	14	1	0	1	0
EABT21842	1	6	40	2	0	0	0
EABT21843	7	7	54	5	7	1	2
EABT21844	517	492	411	735	437	392.01	285
EABT21845	2	5	1	0	3	0	0
EABT21846	8	3	3	7	1	0	0
EABT21847	1	1	24	1	0	1	0
EABT21848	4	5	9	0	0	0	1
EABT21849	3	16	51	20	3.86	2	3
EABT2185	3	4	16	2	4	0	2
EABT21850	187	244	136	416.96	174	119	190
EABT21851	0	2	2	7	0	1	0
EABT21852	246	327	222.01	263	220	221	161
EABT21853	4442.14	2822.82	1669.63	5658.5	1464.01	4697.98	5305.8
EABT21854	3	2	6	0	3	2	1
EABT21855	0	2	18	0	0	1	1
EABT21856	0	0	8	1	0	0	0
EABT21857	3	2	3	3	0	48	11
EABT21858	0	1	0	0	7	0	0
EABT21859	482	848	344.59	106	402	402.77	346
EABT2186	199.04	259.98	87.08	213.7	83	53	150
EABT21860	0	1	4	7	4	0	2
EABT21861	2711.01	4322.03	3075.11	4623	1862.34	2081	2685.17
EABT21862	0	0	2	0	0	0	0
EABT21863	721.12	1251.19	511.99	1697.41	879.83	863.46	666
EABT21864	628.66	1561.02	886.65	2419.55	1094.6	1203.24	1531.15
EABT21865	0	1	0	0	1	1	0
EABT21866	1	7	3	2	1	0	0
EABT21867	12	11	27	5	14	2	22
EABT21868	0	1	12	0	0	0	1
EABT21869	0	0	8	0	0	0	0
EABT2187	1	0	7	0	5	0	825.37
EABT21870	6	18	31	2	1	0	0
EABT21871	2106.59	2935.27	1096.87	2688.41	2140.8	1294.43	1450.63
EABT21872	1	0	0	9	1	0	0
EABT21873	6061.67	4462.71	1127.54	2138.93	2131.02	4929.94	5034.33
EABT21874	4	14	41	4	3	3	4
EABT21875	0	0	8	0	0	2	0
EABT21876	2	1	2	0	3	1	0
EABT21877	0	2	14	2	0	1	1
EABT21878	0	0	9	0	0	1	0
EABT21879	0	5	1	0	1	0	0
EABT2188	0	1	10	5	0	0	0
EABT21880	3	3	26	14	1	3	2
EABT21881	3	3	11	7	3	0	0
EABT21882	76.76	119.12	106.99	174.65	78	54	59.79
EABT21883	0	14	17	3	7	1	1
EABT21884	5	8	0	0	0	0	0

EABT21885	3	62.86	1	1	2	5	10
EABT21886	3	8	8	491	6	0	2
EABT21887	0	0	14	2	0	0	2
EABT21888	2	3	1	0	1	3	0
EABT21889	1	14	2	0	2	3	2
EABT2189	13	21	49	40	9	16	8
EABT21890	2	8	3	3	0	0	0
EABT21891	2	6	16	3	1	2	4
EABT21892	5	10	7	16	13	2	7
EABT21893	22	84	152.98	282	4	31	9
EABT21894	1	0	15	2	0	1	0
EABT21895	0	1	0	6	0	0	0
EABT21896	0	3	0	2	1	0	1
EABT21897	1	0	0	0	1	5	1
EABT21898	5722.27	1118.98	17.94	0	234.97	9679.3	1753.31
EABT21899	1208	767.04	328	1048.44	275	2077.05	947
EABT219	121	235	117	4493.2	224	17	98
EABT2190	4	2	3	0	1	3	1
EABT21900	0	4	4	18	3	5	10
EABT21901	1998.37	5940.19	9530.06	6467.84	8512.58	897.97	4615.86
EABT21902	1275.26	2645.4	1210.03	6042	1011.99	766.26	1720.37
EABT21903	0	0	3	4	0	0	0
EABT21904	0	2	8	5	1	1	0
EABT21905	1312.19	2452	1740.79	6117.67	1838.98	504	1079
EABT21906	2	12	13	47	5	2	2
EABT21907	0	0	12	1	0	0	0
EABT21908	11	30	13	11	5	1	5
EABT21909	4	6	51	4	4	3	3
EABT2191	1	1	0	0	0	1	2
EABT21910	0	1	12	77.86	4	2	0
EABT21911	1	3	9	0	2	1	1
EABT21912	1	4	8	4	0	0	0
EABT21913	0	1	5	1	0	0	0
EABT21914	0	1	1	2	1	0	0
EABT21915	4225.62	5017.38	7709.31	6686.61	3479.59	2152.48	2987.81
EABT21916	0	0	2	1	0	0	0
EABT21917	1	0	54	1	0	0	0
EABT21918	7	2	1	0	0	6	9
EABT21919	6	13	26	80	3	2	2
EABT2192	0	3	2	1	0	0	0
EABT21920	2712.03	2813.98	1396.51	1766	8729.67	2897.06	1880.9
EABT21921	1	0	0	2	0	0	0
EABT21922	10	16	0	0	0	0	0
EABT21923	1	3	40	0	0	0	0
EABT21924	1	0	4	1	1	1	0
EABT21925	1	0	3	0	0	1	0
EABT21926	8	21	43	9	0	2	2
EABT21927	0	0	0	0	2	2	19
EABT21928	9999.88	760	334	384	1666	2562	1011
EABT21929	1106	1382.97	728	1975	1192	1000.84	1109.91
EABT2193	2	4	40	4	823	1	32

EABT21930	2	4	11	0	0	15	0
EABT21931	0	0	18	0	0	0	0
EABT21932	12	13	86	22	2	0	0
EABT21933	58.4	81	120	153	74	25	62
EABT21934	2	4	96.99	30	0	8	3
EABT21935	14	16	119	18	6	13	14
EABT21936	2	17	10	3	3	5	7
EABT21937	0	0	7	0	0	0	0
EABT21938	1	0	1	0	18	0	0
EABT21939	1	0	11	0	1	0	0
EABT2194	0	0	14	0	0	0	0
EABT21940	2	10	3	2	2	3	2
EABT21941	0	0	3	4	1	0	0
EABT21942	0	0	18	2	0	0	0
EABT21943	3	12	12	3	18.26	17	47
EABT21944	2	3	72.5	3	0	0	0
EABT21945	0	0	6	0	0	0	0
EABT21946	0	3	19	2	2	0	1
EABT21947	0	0	5	5	0	0	0
EABT21948	15338.3	16674.5	3936.48	740.02	2684.63	18252	18427.1
EABT21949	52	104	52	23	13	28	58
EABT2195	1	1	0	0	0	6	2
EABT21950	0	0	4	0	1	0	0
EABT21951	0	0	10	0	0	0	0
EABT21952	3	0	26	2	0	0	0
EABT21953	28	39	36	5	13	0	64
EABT21954	0	0	2	1	8	0	1
EABT21955	709.14	1065.27	1280.14	2146.72	986.12	678.66	789.99
EABT21956	0	0	15	0	16	0	0
EABT21957	0	0	13	0	0	0	0
EABT21958	0	4	6	5	0	0	1
EABT21959	21	20	76.01	76213.1	7	0	1
EABT2196	2	0	0	0	0	3	0
EABT21960	3	1	7	6	2	1	0
EABT21961	690	1138.26	1212.45	2179.31	1245.48	784.89	886.11
EABT21962	0	6	17	2	3	0	2
EABT21963	4096	5949	5578.53	9920.36	3370.07	6383.07	4599.89
EABT21964	5	12	33	0	0	1	2
EABT21965	1	2	5	2	1	4	3
EABT21966	818	1054.73	2481.71	2171.1	1382.83	1259.63	751.97
EABT21967	4	3	17	5.11	0	2	3
EABT21968	11	6	4	0	0	4	0
EABT21969	3	3	4	4	2	0	2
EABT2197	0	2	24	3	1	3	2
EABT21970	92	70.13	16	239	180	7	24
EABT21971	1	1	11	0	1	0	1
EABT21972	1	0	50	0	0	6	0
EABT21973	1	2	43	1	0	0	0
EABT21974	170	272	144	234	120	49	78
EABT21975	10	8	11	0	1	1	1
EABT21976	1291.57	2762.29	3010.34	4679.64	1723.08	1106.21	1499.38

EABT21977	285	668	427	1530.99	912	287	157
EABT21978	1	5.77	29	3	8	3.92	0
EABT21979	10	34	111	59	40	21	25
EABT2198	1166.72	2075.06	1764.87	2778.43	908.78	902.6	1242.64
EABT21980	0	0	10	1	0	1	0
EABT21981	7	23	13	28	15	8	4
EABT21982	1	0	8	10.99	0	0	2
EABT21983	0	1	2	0	1	0	0
EABT21984	10878.3	6697.74	8786.49	4964	18905.9	4130.72	3991.55
EABT21985	0	0	3	0	8	3	13
EABT21986	7	5	5	1	4	0	2
EABT21987	12	3	0	0	0	10	3
EABT21988	24	29	47	301	118	53	21
EABT21989	1308.84	1552	951.41	4650.2	1225.93	771.88	1127
EABT2199	2	2	7	1	0	4	2
EABT21990	0	2	2	4	0	0	1
EABT21991	8	1	0	2	1	0	0
EABT21992	7	5	7	12	5	0	0
EABT21993	1	6	18	10	1	2	1
EABT21994	0	2	1	0	0	3	0
EABT21995	1	0	2	3	14	1	0
EABT21996	2	8	3	0	0	30	0
EABT21997	0	0	4	0	0	0	0
EABT21998	0	0	13	0	0	1	0
EABT21999	33	57	124	211	202	62	146
EABT22	2	3	2	4	0	1	0
EABT220	3	9	14	10	0	1	0
EABT2200	5545.87	4334.71	2035.56	17663.6	3362.45	8627.11	4481.6
EABT22000	180	313	1715.99	1590.74	383	121	85
EABT22001	2	2	28	7	0	1	1
EABT22002	6	19	273	5	47	10	32
EABT22003	4452.77	6686.82	6252.67	7473.2	5124.33	3609.14	4303.45
EABT22004	0	1	24	2	0	0	1
EABT22005	5	17	42	11	7	5	3
EABT22006	505	1247.94	2159.51	1880.41	1776.12	72.07	439.99
EABT22007	938.65	1549.26	945.01	2295.77	1100.19	667.15	1188
EABT22008	1	0	0	0	1	0	0
EABT22009	2	5	4	8	2	1	0
EABT2201	1	0	0	0	2	1	18
EABT22010	425.47	575.86	9	508.42	35	451.09	306.43
EABT22011	3	5	11	7	2	2	4
EABT22012	13	26	164	39	10	9	3
EABT22013	254	127	17	15	4	917.58	17.88
EABT22014	3.1	3.06	3.06	0	4.61	0	4
EABT22015	1023.03	1599.92	983.91	1755.87	881.22	758.42	813.85
EABT22016	2	2	1	9	1	0	1
EABT22017	4	8	4	1	0	0	0
EABT22018	23	36	18	33	9	3	5
EABT22019	0	0	0	2	0	0	1
EABT2202	2	0	2	2	2	1	2
EABT22020	0	3	10	0	5	0	1

EABT22021	274	374.97	609.38	1265.97	374.91	307	252
EABT22022	0	0	9	1	0	0	0
EABT22023	1	2	5	1	5	2	6
EABT22024	1548.44	2507.66	2947.08	2956.26	1712.1	1292.69	1389.84
EABT22025	1	2	76	5	0	0	0
EABT22026	479.99	756.9	1251.51	1753.98	547.2	415.05	339
EABT22027	0	1	2	2	0	1	1
EABT22028	7	4	2	4	1	7	1
EABT22029	0	0	2	4	0	0	1
EABT2203	9	43	20	44	29	9	74
EABT22030	80.05	110.06	49	126	46	46	34
EABT22031	3	3	28	2	5	0	2
EABT22032	0	2	1	4	0	0	1
EABT22033	13	0	3	0	0	1	0
EABT22034	4	5	33	17	4	0	0
EABT22035	0	0	10	4	0	0	0
EABT22036	0	6	1	1	0	4	1
EABT22037	0	2	2	3	2	1	0
EABT22038	2	0	0	0	1	5	5
EABT22039	10	8	42	24	13	1	3
EABT2204	82.09	32	45	30	84	4	14
EABT22040	0	0	10	2	0	0	0
EABT22041	1	2	19	0	1	0	1
EABT22042	4	9	53	95	41	0	7
EABT22043	3960.36	6463.25	4437.55	9150.12	3486.14	3745.22	3622.79
EABT22044	1	0	3	0	0	1	4
EABT22045	422.23	692.27	1304.39	713.21	1606.84	218	332.9
EABT22046	2	19	3	25	12	0	0
EABT22047	3	11	8	0	4	3	2
EABT22048	32	59	337	106	54	8	27
EABT22049	8	4	27	14	7	0	0
EABT2205	41	39.04	11	7	11	6	1
EABT22050	670	670	82	163	986	312	10224.6
EABT22051	34	35	0	0	0	1	1
EABT22052	1	1	22	7	0	0	1
EABT22053	0	0	6	0	0	2	0
EABT22054	665.8	1113.45	698.05	2313	1033.7	527.44	791.47
EABT22055	35	151.35	188.69	139	62	9.01	42.04
EABT22056	1696.37	2637.97	2253.23	4925.97	1545.54	1801	1729.02
EABT22057	1	3	16	935.93	42169.8	15	105.94
EABT22058	0	0	11	0	0	0	0
EABT22059	2	2	3	2	1	0	2
EABT2206	1	3	37	44	0	0	0
EABT22060	10738.6	12134	10882.3	9071.74	8131.05	9811.01	8603.41
EABT22061	2	1	2	0	0	1	1
EABT22062	1	1	0	1	0	1	0
EABT22063	0	1	9	0	0	0	1
EABT22064	1	0	15.03	2	0	0	0
EABT22065	808.98	752.26	455.02	463	1363	468	359
EABT22066	4	10	20	47	43	3	1
EABT22067	0	0	11	0	0	0	0

EABT22068	0	0	7	0	16	1	0
EABT22069	3	5	10	4	0	1	3
EABT2207	0	0	6	0	0	1	0
EABT22070	2	5	0	0	0	0	0
EABT22071	2	3	6	62.9	1	4	0
EABT22072	0	5	7	53	34	0	1
EABT22073	8	4	23	1	0	2	2
EABT22074	3	0	6	7	1	0	0
EABT22075	112	226	306	1324.05	900.55	36	77
EABT22076	1	0	0	1	8	0	1
EABT22077	1147.46	3461.16	458.7	1949	1252.01	334.99	256
EABT22078	9	29.98	25.17	10	3	5	11
EABT22079	4363.59	321	8	2	5	8462.05	77
EABT2208	0	1	6	0	0	0	0
EABT22080	0	0	5	1	0	2	0
EABT22081	229.01	147.98	24	7	70.51	373.2	479.37
EABT22082	3	3	2	0	2	0	0
EABT22083	11	18	36	24	271	15	53
EABT22084	0	0	21.03	0	0	0	0
EABT22085	1480.18	1656.95	1547.24	2090.97	1206.2	1631	1894.49
EABT22086	237.98	371	483	2299.05	805	204	261
EABT22087	108	421	1	1	66	111.02	236.88
EABT22088	0	1	8	2	0	0	0
EABT22089	0	1	12	1	1	0	1
EABT2209	1159.6	1734.2	916.47	3132.73	1183.63	759.86	990.18
EABT22090	21116.5	14108.3	11609.1	9626.34	3538	1486.15	1166
EABT22091	95.79	92	349.41	2936.95	1395.46	11	5
EABT22092	18	22	4	20	32	0	47
EABT22093	18	13	51	2	1	1	1
EABT22094	0	0	4	0	0	0	0
EABT22095	1123.5	2456.02	3879.58	4059.15	3164.77	1747.83	1527.01
EABT22096	2	1	19	2	0	0	2
EABT22097	6	24.01	269.98	84	12	2	34
EABT22098	109	345.77	1082.33	2343.96	321	79.98	119.97
EABT22099	410.98	540	305.02	361	540	161	314.09
EABT221	4	5	26	0	0	2	0
EABT2210	8	9	10	1.01	5	27	41
EABT22100	0	2	0	2	0	0	0
EABT22101	0	0	1	0	8	0	0
EABT22102	0	1	0	4	0	0	3
EABT22103	85.91	92	355.97	896.82	63	29	39.88
EABT22104	1	3	14	1	0	1	0
EABT22105	2371.94	4049.64	5927.21	7757.86	3830.97	2324.87	2164.81
EABT22106	37	34	49	83	20	14	21.94
EABT22107	0	4	11	0	2	3	2
EABT22108	886.84	1634.35	1409.99	3191.91	1097.12	898.56	1069.3
EABT22109	2214	1020	299	42	242.96	5572.79	2912.29
EABT2211	502	966.24	888	1761.85	417	328	374.04
EABT22110	0	1	10	0	0	0	0
EABT22111	1	0	6.41	0	0	0	0
EABT22112	0	1	3	0	0	1	0

EABT22113	1866.03	1698	711	9	415	3610	2307.57
EABT22114	0	2	3	3	1	0	1
EABT22115	41	9	23	4	7	1	4
EABT22116	31	12	14	15	41	16	54
EABT22117	1	1	6	3	1	1	0
EABT22118	0	1	10	3	1	0	1
EABT22119	0	2	24.99	2	2	3	0
EABT2212	49	93	225	1007.32	222	6	12
EABT22120	11	29	0	0	0	0	0
EABT22121	787.78	1016.85	571.51	1214.96	565.16	634.94	898.2
EABT22122	3	12	37.98	28	12	3	6
EABT22123	0	3	3	2	0	0	0
EABT22124	1492.48	2674.39	4962.74	1799.96	1368.06	765	1198.47
EABT22125	0	1	7	5	0	2	0
EABT22126	5873.92	13047.4	14797.6	32262.9	32609.3	311.55	994
EABT22127	1632	3087.18	505	740	520	515	884.05
EABT22128	2	2	46	16	2	2	0
EABT22129	0	5	7	5	1	0	2
EABT2213	16	26	208.83	29	11	9	15
EABT22130	1287.23	2049.87	15694.2	3462.31	2587.45	680	1421.14
EABT22131	0	1	1	5	0	0	0
EABT22132	5	3	8	2	3	0	0
EABT22133	0	3	5	0	0	0	0
EABT22134	2	2	8	1	1	0	0
EABT22135	9	7	63	20	14	15	15
EABT22136	582.01	959.96	263.2	110	362	1223.95	1557.1
EABT22137	0	1	4	1	1	0	0
EABT22138	0	1	13.47	2	1	0	0
EABT22139	7	19	16	2	0	0	0
EABT2214	2	7	5	1	6	0	1
EABT22140	1	1	16	0	1	0	0
EABT22141	4	11	36.44	11	5	0	1
EABT22142	0	0	4	1	0	0	0
EABT22143	0	1	0	3	0	0	0
EABT22144	0	1	12	1	1	0	1
EABT22145	62	75.99	89.78	35	9	80	28
EABT22146	3	4	8	0	1	6	5
EABT22147	0	1	19	1	0	0	0
EABT22148	5422.85	7200.05	8143.95	9543.92	4068	4290.61	3561.6
EABT22149	11	22	135	16	9	7	11
EABT2215	3097.32	4249.88	5841.34	6892.97	3792.21	2878.67	2856.61
EABT22150	0	4	8	5	2	0	4
EABT22151	0	0	9	0	0	0	0
EABT22152	0	1	1	0	1	0	3
EABT22153	0	1	9	4	2	2	0
EABT22154	139	529.01	525.94	651.22	101.19	92	163
EABT22155	0	4	72	14	0	2	0
EABT22156	608	631.31	748	884	829	314	244
EABT22157	2	4	9	1	3	2	3
EABT22158	9	9	0	0	5	82	10
EABT22159	5801.66	5671.85	6539.85	8186.06	3013.76	3619.36	3570.63

EABT2216	1	4	2	2	3	0	10.68
EABT22160	1	2	3	3	1	0	0
EABT22161	1	3	57	3	0	1	0
EABT22162	309.91	150	25	43	60	1403	96
EABT22163	2	8	10	57	13	1	0
EABT22164	2	1	1	0	0	1	0
EABT22165	4	0	2	0	1	2	1
EABT22166	6	21	110	19	0	3	1
EABT22167	137	307.94	41	1643.76	386	131	489.95
EABT22168	1	2	22	1	0	0	0
EABT22169	757.66	964.77	1265.32	1583.3	1252.71	726.14	769.79
EABT2217	0	0	0	0	0	0	0
EABT22170	3	4	0	8	0	1	1
EABT22171	670	895	573.97	1205.94	690	470.78	491.99
EABT22172	0	1	0	6.99	1	0	5
EABT22173	14	78	104	162	46	140	185.01
EABT22174	1	2	1	2	1	0	0
EABT22175	0	0	2	1	3	0	0
EABT22176	3	2	12	2	0	1	0
EABT22177	0	0	5	1	8	1	1
EABT22178	0	4	22	2	2	4	1
EABT22179	0	0	8	0	0	0	0
EABT2218	670	1033	937.4	1533	738	1052	700.93
EABT22180	1870.14	3045.37	1854.23	4654.32	1773.41	1181.14	1987.3
EABT22181	8224.98	17392.2	30776.7	29435.9	15987.2	11923.3	10861.1
EABT22182	7	1	6	2	0	1	0
EABT22183	1	0	5	0	1	0	0
EABT22184	0	0	11	2	0	0	0
EABT22185	0	0	3	1	1	0	0
EABT22186	1	3	6	4	0	3	2
EABT22187	2	53	3	113	3389.88	0	8
EABT22188	0	0	34	1	1	1	0
EABT22189	1	2	0	0	0	1	0
EABT2219	0	2	5	1	3	2	0
EABT22190	1	9	20	13	3	0	1
EABT22191	1270.42	2287.2	3190.5	5137.67	932.26	1006.34	991.02
EABT22192	0	1	7	3	3	0	0
EABT22193	11	10	0	0	2	3	3
EABT22194	2	1	8	3	0	1	0
EABT22195	1155	2182.15	1950.33	4204.86	2760.31	1334.33	2022.86
EABT22196	46	59	56.99	40	10	99.13	49
EABT22197	0	7	0	0	0	10	1
EABT22198	3092.95	3454.11	1527.47	1998.41	850.98	820.48	1281.08
EABT22199	1	1	2	0	2	0	6
EABT222	2	2	16	3	0	6	1
EABT2220	0	0	1	8	1	0	0
EABT22200	1	2	12	1	1	0	0
EABT22201	3	20	50	9	6	2	4
EABT22202	198	1260.28	2153.51	4224.53	486	70	123
EABT22203	20	20	3	1	3	6	6
EABT22204	3	3	0	0	0	0	0

EABT22205	1	4	22	4	0	4	1
EABT22206	0	0	0	5	0	1	0
EABT22207	0	0	8	0	0	0	0
EABT22208	0	0	2	1	0	0	6
EABT22209	3	1	15	0	0	0	0
EABT2221	7	18	7	4	0	9	3
EABT22210	15568.1	2269.99	16	0	0	13654.6	9.97
EABT22211	1	5	13	8	2	2	2
EABT22212	75	52	10	68.11	25	28	24.04
EABT22213	1605.43	4953.92	13012.9	10140.9	4642.08	2080.72	4525.76
EABT22214	2	2	3	1	3	2	25
EABT22215	0	1	1	16	1	0	2
EABT22216	3	9	1	2	0	0	0
EABT22217	1	3	1	0	0	0	0
EABT22218	1	2	23	3	1	1	3
EABT22219	1886.96	2399.65	1671.27	2951.3	1999.78	1209.6	1503.3
EABT2222	9	1	0	0	0	0	0
EABT22220	1	5	111	12	0	0	1
EABT22221	0	1	3	3	3	0	0
EABT22222	0	1	1	0	0	0	0
EABT22223	0	0	7	1	0	0	0
EABT22224	3	1	0	0	0	3	0
EABT22225	2	4	22	0	1	1	0
EABT22226	645.79	817	670	1043.08	708.94	649	451
EABT22227	27452.1	16067.6	26	369	14496.5	38464	40626.4
EABT22228	1960.03	3572.77	4332.03	10530.3	3087.03	1768.19	2017.87
EABT22229	3	1	0	0	0	18	0
EABT2223	707	621.46	436	1651.33	180.95	228.78	244.51
EABT22230	0	0	14	0	0	0	0
EABT22231	1	0	4	0	0	0	0
EABT22232	0	3	4	1	0	0	0
EABT22233	3018.95	5047.4	2277.31	3605.91	1966.92	1917.06	2172.07
EABT22234	5	1	0	0	0	0	1
EABT22235	180	207.36	346	375.02	162.11	158.98	163.3
EABT22236	11	98	63	128	0	2	1
EABT22237	0	0	3	0	1	3	0
EABT22238	1	9	11	3	1	1	7
EABT22239	2	10	6	7	8	0	0
EABT2224	7	21	34	1	1	2	0
EABT22240	18	10	9	11	2	7	3
EABT22241	0	1	19	1	0	0	0
EABT22242	1044	1434.82	1667.93	2204.27	946.04	690	603
EABT22243	4218.54	5615.38	9414.57	5024.5	2344.21	3928.63	4994.83
EABT22244	0	1	7	0	0	0	0
EABT22245	6	5	39	11	0	1	1
EABT22246	4406	5511.13	2979	10424.4	2433.56	3603.83	2234.7
EABT22247	5	10	24	9	4	5	2
EABT22248	97.69	388	491.51	427.44	80.82	58	60
EABT22249	698.99	1503.31	732.01	1202.99	540	29	215
EABT2225	0	0	19	2	1	1	0
EABT22250	17	95	32	14	29	11	7

EABT22251	0	0	4	1	0	2	0
EABT22252	430.21	573.33	296.02	630.85	351.56	289	234.88
EABT22253	0	0	0	0	0	0	1
EABT22254	2	5	7	4	2	1	3
EABT22255	784.28	581	16	2	806	1281.44	3902.18
EABT22256	2	2	1	1	0	0	1
EABT22257	1402.85	1996.91	1305.13	6236.27	9734.58	25	163
EABT22258	1	1	16	0	0	0	0
EABT22259	3	15	59	20	3	9	10
EABT2226	717.01	870.92	663.74	1051.76	627.55	448.94	485.78
EABT22260	991	212	104	2	139	88	101
EABT22261	0	0	12	0	0	0	0
EABT22262	0	1	29	1	15	0	1
EABT22263	0	0	6	1	0	0	0
EABT22264	2	0	1	0	1	2	4
EABT22265	1	2	10	1	0	0	1
EABT22266	2	3	4	3	0	0	0
EABT22267	1	3	5	3	0	0	0
EABT22268	0	0	1	2	0	0	0
EABT22269	0	3	4	0	2	0	1
EABT2227	2	6	18	10	4	0	0
EABT22270	40	85	82.88	121	20	9	18.05
EABT22271	3	13.1	143	2	1	2	0
EABT22272	46	23	284.9	72	70	2	9
EABT22273	2	0	1	0	0	0	0
EABT22274	0	1	2	4	0	0	1
EABT22275	2	3	4	0	0	1	3
EABT22276	0	3	9	2	0	1	0
EABT22277	1	8	5	34	10	0	1
EABT22278	3	0	15	6	0	0	0
EABT22279	59	116	17.96	17.96	6	0	0
EABT2228	4	5	26	3	7	0	0
EABT22280	0	0	2	2	0	0	0
EABT22281	12323.6	12714.9	3308	325	2394.36	107	132
EABT22282	775	1322.34	1670.99	1862.38	968.18	1040.06	670
EABT22283	1795.2	2956.08	2750.43	7664.69	1417.29	1774	1597.37
EABT22284	3	1	15	3	0	2	0
EABT22285	149	147	242.06	152	94	53	98
EABT22286	1	0	4	1	0	1	0
EABT22287	1	0	7	2	0	1	0
EABT22288	1	3	22	2	0	0	0
EABT22289	1	19	25	27	2	4	1
EABT2229	1	1	1	4	2	0	0
EABT22290	0	7	1	5	0	0	0
EABT22291	0	0	1	0	0	0	0
EABT22292	0	1	13	0	0	0	0
EABT22293	2	8.99	31	9	3	1	6
EABT22294	1	12	16	7	2	0	4
EABT22295	0	0	1	0	7	0	1
EABT22296	2	4	12	4	0	4	2
EABT22297	0	3	6	13	6	0	1

EABT22298	2	8	3	1	0	3	1
EABT22299	3	6	18	2	1	3	3
EABT223	1	0	3	0	0	0	0
EABT2230	1	0	4	0	0	0	0
EABT22300	3	1	0	1	1	0	0
EABT22301	7	11	0	0	0	0	0
EABT22302	5	1	9	4	2	0	0
EABT22303	1164.37	1898.85	1883.86	4846.57	1480.87	1161.36	792.2
EABT22304	3854.35	5703.48	9407.75	7440.32	4564.83	4231.56	4519.28
EABT22305	0	1	8	1	0	1	0
EABT22306	6	10	44	1	1	0	1
EABT22307	0	3	7	4	0	2	1
EABT22308	0	0	0	2	5	0	0
EABT22309	2227.02	3151.99	2742.71	6476.03	1674.94	3395.17	2469.64
EABT2231	0	3	4	2	0	0	0
EABT22310	6866.39	22439.6	55200.2	18838.5	20770	750.41	4618.11
EABT22311	2	5	21	7	1	2	6
EABT22312	1	0	2	0	0	0	0
EABT22313	0	0	7	0	1	0	0
EABT22314	0	0	7	0	0	0	0
EABT22315	1	3	45	1	0	2	1
EABT22316	3	4	12	2	1	1	1
EABT22317	12	8	8.47	13.36	3	2	6
EABT22318	790.66	984.44	856.38	1615.82	954	1212.68	737.99
EABT22319	3	3	32	1	0	0	1
EABT2232	14785.2	15427.2	12846.8	24317.5	7441.46	13640.6	11623.2
EABT22320	0	0	0	5	0	0	0
EABT22321	1514.97	1697.98	1080.56	2087	1081.01	949.03	728
EABT22322	1	1	9	1	1	0	0
EABT22323	0	0	0	0	1263.23	0	0
EABT22324	182	574.09	295	585.06	492.1	20	133
EABT22325	38	77	170	182	80	5	31
EABT22326	1	10	7	54	6	5	0
EABT22327	0	0	7	2	0	0	0
EABT22328	0	0	4	3	2	0	0
EABT22329	0	0	1	2	1	0	0
EABT2233	697.36	945.18	920.24	1414.58	826	539.01	629
EABT22330	2	0	3	0	0	0	0
EABT22331	5	11	28	15	3	3	9
EABT22332	12	25	10	11	9	0	3
EABT22333	1	1	0	5	0	0	0
EABT22334	2	1	14	5	1	0	0
EABT22335	27.91	23	28	1	7	55	21
EABT22336	0	2	3	0	1	0	0
EABT22337	5	2	3	1	1	0	4
EABT22338	1011.73	3751	3429.05	9845.28	2801	292	884
EABT22339	3	0	4	0	1	0	0
EABT2234	3	4	12	5	0	5	1
EABT22340	1774.3	3279	2801.36	5709.29	2036.05	1552.26	1746.83
EABT22341	0	1	2	1	1	0	0
EABT22342	0	9	66	48	1	2	2

EABT22343	158	460	1115.03	2027	306	60	168
EABT22344	1	8	25	45.72	79	0	0
EABT22345	3703	10240.7	12536.7	3413.49	1678.59	207.02	739.03
EABT22346	6	6	12	0	0	2	2
EABT22347	104.23	508.79	724.21	50000	1987.61	48.02	76.04
EABT22348	2	5	13	1	2	0	0
EABT22349	1	2	0	1	1	2	0
EABT2235	11	5	8	1	5	17	20
EABT22350	713.95	1180.57	955.65	1664.85	930.69	842.98	837
EABT22351	1677.83	2508.2	2463.79	3352.29	2745.01	2142.12	1916.9
EABT22352	3	5	0	0	1	13	11
EABT22353	2	12	9	13	11	5	11
EABT22354	4	9	12	23	1	5	9
EABT22355	0	3	35	2	1	0	0
EABT22356	3	19	1	2	0	65	1
EABT22357	0	0	16	1	0	1	0
EABT22358	0	3	3	11	3	0	3
EABT22359	14	21	0	0	0	0	0
EABT2236	1	0	7	2	0	0	0
EABT22360	0	3	20	0	0	0	0
EABT22361	0	3	6	14	1	0	0
EABT22362	1	1	0	0	0	1	0
EABT22363	55	153.06	200.18	294.65	103	26	59.96
EABT22364	9	24	39	41	6	5	5
EABT22365	6	26	0	0	0	27	0
EABT22366	1608.12	1383	562	1243	1145	1434	1108
EABT22367	0	1	5	4	1	1	0
EABT22368	3	10	21	17	1	0	3
EABT22369	1	33	18	1	0	0	0
EABT2237	1547	2146.88	3343	4336.39	1595	1735.73	1500
EABT22370	46.28	114.76	101.95	364.81	38	152.34	188.11
EABT22371	0	1	10	0	1	1	0
EABT22372	1050.27	1286.07	1261.29	2192.99	1067.85	836.96	1223.59
EABT22373	1	4	2	10.3	1	0	1
EABT22374	1	2	6	0	0	0	0
EABT22375	0	1	10	15	0	1	1
EABT22376	0	1	0	0	2	0	0
EABT22377	0	0	1	0	0	0	0
EABT22378	0	0	1	2	8	3	2
EABT22379	0	0	8	0	1	0	0
EABT2238	1	2	10	5	3	1	0
EABT22380	0	2	4	3	2	0	1
EABT22381	1	7	0	1	0	0	1
EABT22382	2	1	6	6	12	0	4
EABT22383	4415.63	5920.38	4323.1	8280.35	3464.11	3536.85	2810.86
EABT22384	0	0	12	0	1	0	0
EABT22385	47212.3	41765.1	2986	3606.04	56	10	2
EABT22386	1	2	3	14	2	2	0
EABT22387	3	2	2	5	1	0	0
EABT22388	0	0	4	0	1	0	2
EABT22389	0	0	13	0	0	0	0

EABT2239	0	1	0	4	3	1	1
EABT22390	375	575	831.98	640.89	630.96	349	379
EABT22391	6	15	13	26	14	5	9
EABT22392	0	1	5	0	0	0	0
EABT22393	1	1	14	27.85	2	2	1
EABT22394	844.02	1113.81	749.81	1586.96	789.01	484	511.11
EABT22395	95	147	249	786.12	392.17	490.26	42
EABT22396	35	18	52	31	10	95	13
EABT22397	58.98	92.86	98.39	67.37	49	68.34	73
EABT22398	1	1.33	11	2	1	2	1
EABT22399	598.11	942.52	1699.68	1968.94	1120.82	683.09	748.07
EABT224	83	259	360	7320.96	369	1	8
EABT2240	4	3	8	4	0	3	0
EABT22400	0	0	1	3	0	0	0
EABT22401	65	85	68	154.3	44	35	34
EABT22402	9	15	99.96	9	1	10	16
EABT22403	248.91	239.52	233.12	201	249	236.01	202.94
EABT22404	0	0	4	1	0	0	0
EABT22405	5	5	10	3	0	6	5
EABT22406	0	8	37	1	1	4	2
EABT22407	13	9	3	0	489.38	2	2
EABT22408	2	0	3	2	0	0	0
EABT22409	1	1	3	1	0	0	0
EABT2241	1	4	26.28	13	15	0	6
EABT22410	1304.15	1244.95	4819.35	4500.53	484.49	421.89	632.09
EABT22411	0	0	17	1	0	0	0
EABT22412	2	5	8	14	2	5	4
EABT22413	4	1	2	0	0	9	2
EABT22414	1	1	1	0	1	0	0
EABT22415	30	52	220	90	14	23	31.02
EABT22416	0	0	7	1	0	0	1
EABT22417	2	1	26	3	0	0	0
EABT22418	0	1	0	0	0	18	0
EABT22419	0	0	3	1	0	1	0
EABT2242	0	3	22	12	1	0	0
EABT22420	0	0	6	1	0	0	0
EABT22421	1	4	7	81	0	4	0
EABT22422	1	2	12	3	0	0	0
EABT22423	2	3	14	0	0	0	0
EABT22424	4	3	3	3	3	1	1
EABT22425	3	4	198	2	1	4	1
EABT22426	5	4	1	0	1	2	8
EABT22427	77	156	261	158	152.96	94	144
EABT22428	0	0	8	0	0	0	0
EABT22429	3	6	8	0	3	4	10
EABT2243	11	37	5	21	16	20	37
EABT22430	3	11	35	1	1	0	0
EABT22431	4	2	0	5	2	0	2
EABT22432	57.99	30	15	2	26533.2	26	84
EABT22433	3	0	4	1	0	0	1
EABT22434	1	3	7	1	0	1	3

EABT22435	0	0	5	4	1	0	1
EABT22436	0	0	7	0	0	0	0
EABT22437	2	0	1	1	0	2	1
EABT22438	1	5	26	5	3	0	1
EABT22439	0	37	5	5	0	0	0
EABT2244	0	1	3	7	1	0	0
EABT22440	347	71	175	7678.61	12	0	0
EABT22441	1940.94	2935.3	2180.59	3597.18	1895	1166	1687.42
EABT22442	0	13	22	0	1	3	6
EABT22443	359.22	650.83	557.65	968.96	598.94	455.03	515.02
EABT22444	2	17	6	13	2	1	9
EABT22445	0	3	67	4	0	1	1
EABT22446	0	7	3	2	1	0	1
EABT22447	0	0	2	18	0	4	3
EABT22448	23	37.79	142	1	8	0	6
EABT22449	0	2	3	1	1	0	4
EABT2245	0	1	14.85	3	0	0	1
EABT22450	3	4	71	4	503.83	4	1478.43
EABT22451	20	43	40	306.97	148	23	22
EABT22452	5	10	23	0	0	0	0
EABT22453	1	1	3	18	2	2	2
EABT22454	1	3	0	0	0	2	0
EABT22455	0	0	10	6	1	0	2
EABT22456	12	2	8	2	3	3	7
EABT22457	1	0	2	2	2	0	0
EABT22458	10	1	5	5	2	5	3
EABT22459	5	7	7	8	2	1	3
EABT2246	10	13	169	1	0	2	0
EABT22460	0	7	0	0	0	0	0
EABT22461	2	1	0	0	0	3	13
EABT22462	397.36	2206.6	1002.01	8347.21	4773.83	80.19	1178.9
EABT22463	0	0	10	11	1	0	0
EABT22464	0	1	8	0	0	0	4
EABT22465	1284.18	1898.42	2055.13	3923.76	1472.79	957.29	1336.43
EABT22466	2	3	34	4	2	3	2
EABT22467	0	1	1	0	1	0	0
EABT22468	0	5	1	1	0	0	0
EABT22469	1	6	4	6	1	0	0
EABT2247	0	1	1	0	4	0	0
EABT22470	813	962	115	23	1	0	0
EABT22471	0	1	6	2	1	0	1
EABT22472	21832.4	52	16	19	21	29310.3	19
EABT22473	5	5	23	9	3	2	8
EABT22474	27	134.99	177	251	622	18	74
EABT22475	0	3	13	1	0	0	0
EABT22476	2	2	5	0	0	1	1
EABT22477	1	2	8	1	0	0	0
EABT22478	1	45	75	14	4	0	1
EABT22479	0	0	9	2	15	0	0
EABT2248	7	31	3	42	5	0	0
EABT22480	0	0	7	0	1	0	0

EABT22481	1036.3	1880.22	2212.05	4002.39	1482.04	1053.02	1253.26
EABT22482	0	2	6	0	0	0	1
EABT22483	2	1	6	0	1	4	10
EABT22484	1143.03	1635.41	1397.83	3188.9	1022.97	830.91	719.95
EABT22485	2323.28	3719.43	2690.08	4248.07	2482.61	2029	1926.32
EABT22486	0	2	7	3	1	3	1
EABT22487	7	68	8	0	1	17	13
EABT22488	1	0	0	0	0	4	1
EABT22489	1	3	0	6	3	0	1
EABT2249	2	1	7	4	2	0	0
EABT22490	0	1	1	0	2	3	31
EABT22491	3	8	31.98	9	0	4	0
EABT22492	17	58	9	2	564.97	13	662.97
EABT22493	1	0	1	15	0	0	0
EABT22494	5891.04	13999.1	10506.3	37936.3	15772.8	5705.77	11791.2
EABT22495	1	0	4	0	0	2	0
EABT22496	108	506	107	48	32	90	80
EABT22497	93	107	67	187	120	84	75
EABT22498	0	2	5	4	0	0	0
EABT22499	0	0	0	0	0	0	3
EABT225	2	1	22	0	1	3	1
EABT2250	1	2	1	2	1	0	0
EABT22500	7	1	2	15	0	0	5
EABT22501	0	0	6	0	0	1	0
EABT22502	4	3	8	4	2	2	1
EABT22503	0	0	5	0	0	0	0
EABT22504	5	44	4	12	19	12	27
EABT22505	20.22	14	4	5	5	41.98	21
EABT22506	1	2	10	1	0	1	1
EABT22507	2	2	0	1	17	0	0
EABT22508	5	13	13	6	3	0	5
EABT22509	2	5	4	6	1	2	0
EABT2251	0	4	11	2	0	0	0
EABT22510	0	3	6	2	1	0	0
EABT22511	9344.64	14116	5134.52	5795.18	12559.9	3854.28	7252.74
EABT22512	2	2.99	4	1	0	1	1
EABT22513	0	1	6	1	0	0	0
EABT22514	0	4	7	20	1	0	0
EABT22515	0	3	9	0	0	0	0
EABT22516	2	0	4	6	1	0	0
EABT22517	2	2	7	0	0	0	1
EABT22518	8	28	68	17	161.92	11	1
EABT22519	5	8	0	0	0	1	3
EABT2252	2	12	23	7	2	6	1
EABT22520	12	24	53	1527.74	318	3	2
EABT22521	6	5	0	0	1	2	2
EABT22522	3	0	5	2	0	1	0
EABT22523	168	289	272	503	348	213	237.89
EABT22524	182.82	168	796.2	6	50	2	2
EABT22525	6	2	26	35	2	2	9
EABT22526	1	0	2	6	0	0	0

EABT22527	9	11	36	7	4	0	3
EABT22528	0	1	12	1	0	1	0
EABT22529	20	31	62	9	52.87	27	41
EABT2253	3	1	7	1	1	2	2
EABT22530	0	13	12	11	3	0	0
EABT22531	3	2	7	2	1	1	1
EABT22532	28	44.18	36	61	21	39.95	18
EABT22533	0	1	10	0	0	0	1
EABT22534	58	69	72	21	2	5	1
EABT22535	0	0	14	2	2	0	0
EABT22536	1	6	3	2	0	1	0
EABT22537	4	4	3	13	1	4	3
EABT22538	53.89	164.93	203.44	131.22	73.02	105.03	43
EABT22539	12	19	15	1	11	0	3
EABT2254	3	14	6	28	0	4	1
EABT22540	4	4	17	4	0	4	2
EABT22541	6	4	13	1	0	1	1
EABT22542	0	5	0	0	0	0	0
EABT22543	0	5	20	1	0	0	1
EABT22544	721.42	1535.99	1646.06	2398.98	868.72	323	593.54
EABT22545	25202.3	75042.7	22790.3	13773.2	38184	7983.49	12607.6
EABT22546	0	0	18	0	0	0	0
EABT22547	0	2	1	4	0	0	0
EABT22548	2	2	11	14	2	0	1
EABT22549	1	3	6	7	2	1	0
EABT2255	26	3	7	0	0	23	0
EABT22550	294	383	226.98	601	186	160	174
EABT22551	0	1	0	12	0	0	0
EABT22552	2	0	0	0	0	4	2
EABT22553	281	44	16	284	31	1098.36	857.49
EABT22554	2	6	2	0	0	0	0
EABT22555	2	6	2	0	0	0	0
EABT22556	0	1	0	2	0	0	1
EABT22557	4644.49	5389.03	3614.24	7661.6	2255.75	4697.39	4163.09
EABT22558	0	0	4	2	0	1	0
EABT22559	25	41	62	73	18	12	18
EABT2256	1534	2148	838	342	436	311	635
EABT22560	0	5	5	23	3	1	0
EABT22561	1	2	15	1	0	0	1
EABT22562	41	164	256	177	39	67	49
EABT22563	0	2	4	0	0	0	0
EABT22564	538.84	966.84	1185.63	2894.17	601	459	465.1
EABT22565	4	19	22	8	5	6	8
EABT22566	0	0	9	0	0	0	0
EABT22567	1	0	2	0	0	0	0
EABT22568	0	3	5.95	1	2	1	2
EABT22569	916.97	1274.98	1966.4	2718.61	1135.51	566.97	608
EABT2257	17.09	52	73	90.57	194.2	16	69
EABT22570	0	1	4	9	5.02	2	14
EABT22571	0	0	18	1	0	0	0
EABT22572	5	5	15	13	1	6	9

EABT22573	1	3	19	1	0	0	2
EABT22574	4	26	24	4	5	1	4
EABT22575	0	0	45.01	0	0	0	0
EABT22576	1	2	12	12	0	0	0
EABT22577	7	22	19	102	43	1	7
EABT22578	3	4	3	0	0	1	0
EABT22579	0	0	8	1	0	0	1
EABT2258	0	0	26	40	1	2	1
EABT22580	4	6	22	12	3	3	2
EABT22581	8020.99	10830.7	1259.1	3769.86	1312.2	15339	4685.1
EABT22582	0	0	4	2	1	0	0
EABT22583	5786.07	8158.8	4902.76	8277.99	4711.43	5782.5	4601.55
EABT22584	0	0	5	6	0	0	0
EABT22585	1	1	0	0	1	0	1
EABT22586	3040.53	4436.15	4456	9285.52	3666.3	2674.61	2065.31
EABT22587	3	0	3	1	0	1	5
EABT22588	0	0	3	2	0	1	1
EABT22589	3	29	6	18	3	0	1
EABT2259	6	3	2	0	0	5	0
EABT22590	18	28	38	63	24	2	8
EABT22591	32	52	141.67	124.89	55	19	39
EABT22592	0	2	9	0	0	1	4
EABT22593	0	2	8	8	2	2	6
EABT22594	0	239	86	13	5	17	3
EABT22595	0	1	5	2	0	0	0
EABT22596	7	17	39	1	1	1	1
EABT22597	1301.89	4003.11	4081.77	3613.37	2500.09	822.07	1053.32
EABT22598	1	4	3	6	1	0	0
EABT22599	3	14	6	1	6	3	10
EABT226	19	5	1	0	6	12	37
EABT2260	1330	2142.96	4272.2	3979	2373.88	1366	1605
EABT22600	24	6	40	81	17	5	2
EABT22601	1	4	3	0	0	0	0
EABT22602	0	0	6	1	2	0	1
EABT22603	2435	3557	818	2144	808	2202	1199
EABT22604	0	2	8	0	1	4	0
EABT22605	179.86	339.23	21	209	169.01	419.42	1145.59
EABT22606	4	12	29	6	0	1	3
EABT22607	2	2	11	2	0	0	1
EABT22608	0	0	5	1	0	0	0
EABT22609	0	10	7	11	1	3	2
EABT2261	3	3	0	1	0	1	1
EABT22610	1871.96	3733.79	4985.06	5512.3	1940.01	1074	1604.03
EABT22611	24	28	60	88	57	52	34
EABT22612	3	1	0	0	3	0	2
EABT22613	0	0	9	0	0	0	0
EABT22614	1	0	24	2	1	0	1
EABT22615	1	8	21	18	3	1	4
EABT22616	1	4	6	6	4	0	7
EABT22617	3	0	4	1	0	0	0
EABT22618	1	0	3	0	0	0	0

EABT22619	1349.99	1660.32	1307.08	1703.63	1067.47	1165	1096.95
EABT2262	8	13	22	6	1	0	4
EABT22620	2	1	11	0	0	0	0
EABT22621	1	0	14	6	2	0	7
EABT22622	2	6	1	0	0	2	0
EABT22623	1533.05	2712.96	2744.88	10152.2	3692.52	1205.89	1082.16
EABT22624	0	0	2	3	0	0	0
EABT22625	1	1	8	1	0	2	2
EABT22626	1	14	12	16	2	0	4
EABT22627	5	2	3	0	0	76	0
EABT22628	0	2	2	2	1	1	0
EABT22629	363.96	615.55	390.47	1117.48	408.06	185.97	317.2
EABT2263	0	0	78	0	0	0	0
EABT22630	0	0	4	1	0	2	0
EABT22631	19	30.99	329.68	5	8	36	35
EABT22632	287	321	26	50	18	201	33
EABT22633	0	0	7	0	0	0	0
EABT22634	2	5	3	20.01	23	1	19
EABT22635	0	0	26	3	3	6	0
EABT22636	166	329.02	1320.36	639.07	1061.56	57	154
EABT22637	4	2	3	21	8	6	10
EABT22638	0	0	0	0	1	0	5
EABT22639	118	82	22.04	185.97	12	14	33
EABT2264	0	0	3	1	0	0	0
EABT22640	0	0	5	0	1	0	0
EABT22641	2	2	6	8	15	0	6
EABT22642	41	143.61	0	0	3	31	9
EABT22643	27	18	38	2	1	14	15
EABT22644	146	128	422	1979	540.1	41	190
EABT22645	0	1	18	1	1	0	0
EABT22646	40	48.07	104	25	6	31	5
EABT22647	0	0	11	0	0	0	0
EABT22648	0	0	1	8	0	0	0
EABT22649	2	7	181	4	2	11	6
EABT2265	0	0	4	1	0	1	0
EABT22650	0	1	13	0	0	1	0
EABT22651	2221.83	3241.82	1856.25	3813.69	1078.79	1166.48	1421.38
EABT22652	29	52	35	38	16	29	24
EABT22653	7	63	8	31	30	2	14
EABT22654	3	6	13.87	1	1	2	0
EABT22655	0	2	8	0	0	0	1
EABT22656	68129.7	53506.9	17119.2	44204.9	69611.7	63956.1	39833.6
EABT22657	1	11	20	10	0	2	8
EABT22658	0	1	1	3	0	0	0
EABT22659	0	1	0	7	13	0	0
EABT2266	0	0	6	0	0	0	0
EABT22660	0	0	12	0	0	0	2
EABT22661	0	2	35	10	1	3	1
EABT22662	1	11	294	2	3	0	1
EABT22663	0	1	1	5	1	0	0
EABT22664	0	0	7	0	1	2	0

EABT22665	0	2	0	0	0	0	0
EABT22666	1	5	15	34	2	0	0
EABT22667	0	0	6	0	0	0	1
EABT22668	1619	1180	186	350	346.99	220.99	1417.88
EABT22669	0	2	2	2	0	0	1
EABT2267	1	4	53	1	1	2	1
EABT22670	4625.25	10402.3	16175.1	17012.3	9480.76	4283.46	5013.38
EABT22671	0	0	10	0	0	0	0
EABT22672	3	2	11	0	0	0	0
EABT22673	7	4.94	4	7	0	6	4
EABT22674	1	4	12	1	1	0	0
EABT22675	2	3	11	8	1	0	1
EABT22676	5664.8	5261.91	6961.22	1088.63	9983.05	699.87	1298.67
EABT22677	0	0	9	0	0	0	0
EABT22678	2	5	12	2	34	0	0
EABT22679	0	0	6	6	0	0	0
EABT2268	0	1	0	0	0	0	3
EABT22680	1	0	1	2	6	0	0
EABT22681	0	1	0	0	0	0	1
EABT22682	0	3	16	2	1	1	1
EABT22683	232	197	67	72	314.05	546	273
EABT22684	2	1	7	0	0	0	0
EABT22685	75	83	55	333	131	29	37
EABT22686	1308.85	1436.3	792	1735.01	753.99	1053	708.71
EABT22687	3	2	16	2	0	0	0
EABT22688	0	2	1	7	1	0	0
EABT22689	0	1	4	3	1	0	0
EABT2269	3	13	6	47	19	3	3
EABT22690	0	1	10	0	0	0	0
EABT22691	1	2	34	0	1	0	1
EABT22692	0	1	0	7	0	0	0
EABT22693	0	0	13	12	0	0	1
EABT22694	0	2	4	3	2	0	0
EABT22695	1	34	17	73	54	7	2
EABT22696	12846	12223.1	9144.18	10142.2	11484.4	14216.2	13247.3
EABT22697	1	2	96	2	0	0	0
EABT22698	603.11	1206.3	503.44	1818.78	935.67	13	84.84
EABT22699	2	5	6	9	4	1	1
EABT227	0	1	1	2	2	0	0
EABT2270	1	4	2	0	0	1	3
EABT22700	0	0	24	0	0	2	2
EABT22701	0	0	2	3	0	1	0
EABT22702	0	0	0	4	0	0	0
EABT22703	0	1	4	1	8	0	0
EABT22704	2	9	5	4	1	2	1
EABT22705	1795	2249.01	2442.04	3818.56	1780	892.85	1015
EABT22706	6	4	3	16	0	0	5
EABT22707	0	0	0	4	2	0	1
EABT22708	8	1	0	0	0	13	1
EABT22709	1	1	0	9	0	0	0
EABT2271	0	5	29	5	1	1	1

EABT22710	2	2	13	8	3	0	2
EABT22711	0	0	5	3	1	0	0
EABT22712	63	34	45	16	275	45	441.15
EABT22713	0	0	1	0	5	0	0
EABT22714	704.32	1248	1083	4527	1169.11	208	360
EABT22715	1	3	0	1	0	0	2
EABT22716	4	0	0	0	0	8	0
EABT22717	0	2	8	2	1	0	0
EABT22718	0	2	10	14	3	1	2
EABT22719	3545.58	5413.7	3703.58	2978.3	1006.36	1187.02	50
EABT2272	1	0	0	0	0	6	1
EABT22720	2	9	20	9	0	4	2
EABT22721	5	17	19	26	10	2	11
EABT22722	27	7	7	6	12	3	0
EABT22723	0	1	7	0	0	0	0
EABT22724	795.99	1101.95	1307.11	2199.29	1249.53	726	602.03
EABT22725	40895.5	67940.2	78173.9	75331.9	32175.4	2979.4	6880.65
EABT22726	0	0	0	1	0	0	0
EABT22727	1	0	8	3	0	0	1
EABT22728	1	4	0	0	0	0	0
EABT22729	10	11	34	14	2	13	4
EABT2273	9	27	5	1	1	7	3
EABT22730	4	6	23	1	0	1	0
EABT22731	1	8	20	52	3	5	7
EABT22732	2	0	5	2	3	1	0
EABT22733	2	4	15	11	2	0	2
EABT22734	0	1	3	2	0	1	0
EABT22735	1	1	2	16	1	1	1
EABT22736	1	3	11	0	0	0	0
EABT22737	0	3	0	5	2	0	1
EABT22738	1	1	13	0	3	4	2
EABT22739	1	1	3	0	1	1	1
EABT2274	2	4	0	1	1	0	0
EABT22740	1933.29	6765.74	6	5	19	2775.42	278
EABT22741	89	242.78	173.66	596.9	78	83	118.95
EABT22742	1	4	2	5	1	1	1
EABT22743	4	8	1	4	0	1	2
EABT22744	0	0	60	3	1	0	0
EABT22745	0	0	3	0	0	0	1
EABT22746	5087.98	4519.96	2290	2194	23867.4	4312.69	3533.88
EABT22747	0	1	2	0	0	0	0
EABT22748	0	0	6	1	1	0	0
EABT22749	1	7	3	47	10	0	0
EABT2275	0	2	2	0	0	1	0
EABT22750	1056.99	2106.2	2883.15	6106.14	1181.95	1283.99	837.02
EABT22751	0	1	0	0	0	0	0
EABT22752	2	3	21	312	71	13	16
EABT22753	0	2	45	0	0	1	0
EABT22754	0	1	12	0	0	0	0
EABT22755	1	1	3	2	1	0	0
EABT22756	0	2	10	3	1	2	5

EABT22757	8	40	111	195	98	2	4
EABT22758	0	1	2	1	3	0	0
EABT22759	1	9	6	45	8	3	12
EABT2276	1	1	3	0	1	0	0
EABT22760	0	0	13	0	0	0	0
EABT22761	7	2	1	0	3	2	12.01
EABT22762	3	3	1	0	0	0	0
EABT22763	67	100	20	1	3	0	0
EABT22764	137	188.01	138.77	502.47	145.47	95.03	85.79
EABT22765	6	3	11	3	2	4	0
EABT22766	1	4	0	9	11	0	1
EABT22767	3	3	2	4	4	0	1
EABT22768	2	4	3	0	0	1	0
EABT22769	0	6	21	4	3	3	0
EABT2277	0	1	7	0	0	1	0
EABT22770	2	3	16	0	0	0	1
EABT22771	2	3	0	0	1	4	1
EABT22772	1	2	3	0	0	0	0
EABT22773	0	0	2	0	2	0	0
EABT22774	0	1	0	6	0	0	1
EABT22775	0	2	5	0	0	0	1
EABT22776	1	0	14	0	0	0	0
EABT22777	0	0	1	4	0	3	1
EABT22778	2646.98	2152	1453.06	724	1879	797.99	980
EABT22779	575.78	1044.99	652.66	2128.99	961.1	459	529.48
EABT2278	0	0	3	5	0	0	0
EABT22780	9	19	16	78	14	5	14
EABT22781	3	5	7.08	8	2	0	5
EABT22782	2	3	9	6	2	1	11
EABT22783	0	2	3	1	0	0	0
EABT22784	0	2	10	3	0	2	0
EABT22785	0	0	7	0	0	0	1
EABT22786	1807.27	3752.43	2548.82	3382.65	2999.56	1341.59	2019.25
EABT22787	7	11	14	1968.17	29	1	5
EABT22788	0	1	4	1	0	0	0
EABT22789	6	4	9	5	2	0	0
EABT2279	0	0	3	3	0	0	0
EABT22790	2	5	14	4	4	0	2
EABT22791	0	0	3	1	2	0	0
EABT22792	2178.62	3315.27	4919.29	4225.84	1356.13	2200.04	1379.93
EABT22793	0	1	3	0	0	0	1
EABT22794	0	1	15	2	0	0	0
EABT22795	0	0	0	9	2	0	0
EABT22796	0	1	3	1	0	0	0
EABT22797	2	14	6	49	30	0	17
EABT22798	0	0	6.16	6	0	0	0
EABT22799	0	0	0	0	12	1	4
EABT228	2	15	24	8	5	5	5
EABT2280	115	13	1	6	5	87	30
EABT22800	0	4	18.16	2	2	1	2
EABT22801	0	1	3	40	0	1	0

EABT22802	52	42	269	5796.42	7	0	0
EABT22803	1	0	1	2	0	1	0
EABT22804	8	6	28	32	7	3	11
EABT22805	0	0	11	2	0	0	1
EABT22806	1	0	4	2	0	0	0
EABT22807	0	0	0	8	0	0	0
EABT22808	0	1	8	1	1	0	0
EABT22809	0	0	0	17	0	0	0
EABT2281	1	1	2	4	0	0	2
EABT22810	2	4	3	1	0	1	3
EABT22811	0	0	1	9	0	0	0
EABT22812	2	2	7	4	4	1	2
EABT22813	0	1	3	1	1	1	2
EABT22814	0	0	2	20	3	0	1
EABT22815	3	5	18	1	2	1	0
EABT22816	0	1	13	0	0	1	0
EABT22817	371	651.14	324.66	1356.07	391	253.03	349.12
EABT22818	374.89	1241.92	1567.89	528.99	1191.54	141	245
EABT22819	2	3	0	4	0	0	0
EABT2282	79.97	119	121	678.26	150.01	45	50
EABT22820	7	41	48	51	4	3	9
EABT22821	0	5	11	1	0	0	0
EABT22822	6	13	16	12	3	6	9
EABT22823	0	0	5	0	0	0	0
EABT22824	2	4	11	44	1	0	3
EABT22825	4	0	6	11.98	0	1	2
EABT22826	578.47	150	30	0	1	245.99	1
EABT22827	66	141	256.88	401	166	114.66	108
EABT22828	1736.75	2304.01	3666.51	4911.69	1342.94	783.11	831.7
EABT22829	0	0	8	5	1	0	0
EABT2283	2	2	15	3	2	1	0
EABT22830	0	1	0	1	4	0	1
EABT22831	0	1	3	0	0	0	1
EABT22832	1119.62	1310.93	755.35	973.55	781.52	1380.99	1198.62
EABT22833	5	1	11	0	0	4	0
EABT22834	1	0	2	2	1	0	0
EABT22835	15	28	86	40	22	68	16.2
EABT22836	2	0	23	3	2	1	0
EABT22837	2.01	2	4	19	5	2	2
EABT22838	5	0	0	0	0	27	0
EABT22839	1	1	13	1	1	0	1
EABT2284	0	1	3	0	0	2	0
EABT22840	107	175	246.01	355	249.95	92	124
EABT22841	4	64	6	18	1	1	3
EABT22842	1636.66	2546.23	3187.22	4601.64	1634.21	1616.47	1380.81
EABT22843	0	1	11	2	1	0	2
EABT22844	71.18	78.09	65.97	214.84	92.76	71.23	104.98
EABT22845	0	2	11	2	1	7	1
EABT22846	4	19	26	13	3	2	2
EABT22847	0	1	4	0	2	0	0
EABT22848	18.98	13	0	0	0	32	1

EABT22849	2	1	1	0	0	0	0
EABT2285	3	2	0	1	0	0	0
EABT22850	5037.63	4498.45	3752.37	7714.73	1700.18	4	156.21
EABT22851	3	0	0	0	1	3	0
EABT22852	0	3	1	0	9	0	0
EABT22853	496	1548.21	461.65	286	29	0	6
EABT22854	6	14	10	20	7	1	7
EABT22855	0	2	9	7	0	0	0
EABT22856	0	3	3	5	0	0	1
EABT22857	0	1.89	10.3	35	0	0	0
EABT22858	0	0	1	4	1	0	0
EABT22859	78	62	484.49	922.94	809.23	4	51.27
EABT2286	1	3	2	0	1	0	1
EABT22860	2	5	10	1	0	0	1
EABT22861	1	1	3	1	1	0	0
EABT22862	0	1	7	5	0	0	0
EABT22863	0	1	2	0	1	1	1
EABT22864	1077.59	1498	328.93	566	498.72	500.15	990
EABT22865	9	10	22	3	4	1	1
EABT22866	11	25	12	1	1	30	11
EABT22867	0	0	5	0	0	0	0
EABT22868	0	0	1	5	0	0	0
EABT22869	5	7	0	0	0	2	0
EABT2287	0	3	20	13.06	0	4	0
EABT22870	546	563.88	965.81	1154	449.96	577	1481.1
EABT22871	4	0	2	0	1	3	2
EABT22872	0	0	10	0	0	0	0
EABT22873	0	5	3	3	0	0	0
EABT22874	0	1	5	0	1	0	0
EABT22875	0	1	3	0	0	0	1
EABT22876	1	3	1	3	0	0	0
EABT22877	0	0	1	0	0	0	35.02
EABT22878	1885.23	2725.64	1997	2751.69	1279.69	1742.21	1335.04
EABT22879	0	2	14	12	0	0	1
EABT2288	0	1	3	0	0	0	0
EABT22880	6	4	24	7	4.03	1	1
EABT22881	0	1	3	4	2	0	0
EABT22882	69	111	187	123.1	240	66	83.99
EABT22883	69	171.19	223.79	146	64	26	50
EABT22884	0	2	16	1	0	0	0
EABT22885	4	6	3	6	3	1	1
EABT22886	1	0	3	2	1	1	0
EABT22887	343.02	629.93	374.46	1126.14	403	274	296.72
EABT22888	401.04	533.59	1056	2248.75	565.01	86	268.99
EABT22889	383	3019.94	1472.11	8466.21	851.88	602	1644.1
EABT2289	2	1	38	5	4	0	0
EABT22890	0	0	12	0	2	1	3
EABT22891	2806.79	4130.41	4502.4	13073.9	2227	1838	2132.85
EABT22892	1710.34	2922.89	2011.59	3790.03	2151.35	1344.51	1322.83
EABT22893	6	6	12	16	0	2	6
EABT22894	0	0	3	0	11	0	0

EABT22895	1	11	0	21	0	8	3
EABT22896	1150.96	1016.04	609.07	934.01	710	1167	820
EABT22897	2	2	3	0	0	0	0
EABT22898	1	0	3	0	0	1	0
EABT22899	3	3	20	4	0	2	0
EABT229	0	0	1	9	1	1	0
EABT2290	5	2	0	0	8	10	18
EABT22900	43	158	202	576.84	203	33	49
EABT22901	4	10	14	1	3	1	14.98
EABT22902	0	2	3	1	0	0	1
EABT22903	0	3	2	0	0	3	0
EABT22904	237	78	302.21	214.91	339.15	249.49	92
EABT22905	0	1	12	1	3	1	0
EABT22906	0	0	3	1	0	0	0
EABT22907	21	49	6	1	9	13	16.47
EABT22908	0	3	2	0	1	2	0
EABT22909	0	1	4	2	1	1	0
EABT2291	0	1	4	3	0	2	1
EABT22910	1	7	2	4	3	1	0
EABT22911	1562	2056.02	1208.81	2727.89	1113.99	891	1242.35
EABT22912	2230.98	6997.94	20474.9	6608.56	9683.2	530.74	2873.88
EABT22913	22	56	18	298	88.3	14	37
EABT22914	0	4	7	2	1375.99	0	3026.53
EABT22915	0	0	18	1	0	0	0
EABT22916	6	13	39	9	2	3	6
EABT22917	0	2	13	1	0	0	0
EABT22918	0	10	1	1	7	0	3
EABT22919	0	3	1	1	0	0	0
EABT2292	1918.68	3967.17	1366.43	991.47	726.02	1285.39	708.1
EABT22920	5	7	536.92	5	2	7	3
EABT22921	0	0	11	1	0	0	0
EABT22922	6	21	22	3	0	3	0
EABT22923	10	15	0	0	1	8	0
EABT22924	0	0	0	9	3	0	0
EABT22925	1	0	0	2	0	0	6
EABT22926	0	0	2	0	1	1	0
EABT22927	417	546.67	747.15	660.96	628	296.95	382
EABT22928	0	1	5	0	0	0	1
EABT22929	0	1	2	1	0	0	0
EABT2293	1	4	1	0	1	0	0
EABT22930	2740.55	2046.05	516	1703.41	1097.31	3397.21	1568.91
EABT22931	7	21	8	12	3	1	7
EABT22932	10193	12107	29	2	740.51	1	520.24
EABT22933	0	2	8	9	0	2	2
EABT22934	0	3	3	0	0	0	1
EABT22935	0	1	2	15	0	0	0
EABT22936	9	17	19	9	3	22	4
EABT22937	0	1	4	1	1	0	1
EABT22938	8	1	1	2	10	3	22
EABT22939	104.01	190.29	138.97	283.49	90	57.05	69.98
EABT2294	0	4	6	1	0	0	0

EABT22940	498	602.45	656	1116	386	437	294
EABT22941	2	2	8	0	0	0	0
EABT22942	3	1	0	2	2	0	1
EABT22943	5	2	2	0	2	7	5
EABT22944	1418.89	1741.98	1227.97	2238.08	1568.88	1372.92	1190.6
EABT22945	0	0	8	18	8	0	0
EABT22946	0	0	2	7	1	0	3
EABT22947	2438.39	5187.65	3051.27	5395.17	1868.57	1959.96	1609.57
EABT22948	1	3	23	3	1	0	0
EABT22949	1	3	14	0	1	0	0
EABT2295	0	2	13	4	1	2	3
EABT22950	1	4	3	1	1	0	0
EABT22951	3	2	2	5	1	8	2
EABT22952	256.52	371.98	277.55	707.55	298.84	149.01	309
EABT22953	0	0	7	2	1	0	1
EABT22954	5518.96	5498.93	2661.67	7560.5	2242.53	7446.07	3604.9
EABT22955	27	48	281	89	530.08	8	34
EABT22956	0	3	7	0	0	2	1
EABT22957	0	1	6	1	0	1	0
EABT22958	712.96	1033.73	957.08	1175.99	953.88	807.66	762.14
EABT22959	4	1	3	1	0	3	0
EABT2296	1	0	2	3	1	0	0
EABT22960	0	0	3	19	3	0	0
EABT22961	0	1	6	0	3	1	2
EABT22962	1	9	8	14	1	1	1
EABT22963	402.98	674.14	748.57	1941.52	804.53	567.38	663.31
EABT22964	0	0	1	0	5	0	1
EABT22965	0	0	16	5	0	0	0
EABT22966	3111.66	41578.1	15086	18929.8	6297.53	3713.27	3122.3
EABT22967	1	2	5	8	17	0	0
EABT22968	0	0	7	0	0	0	0
EABT22969	0	1	3	2	0	0	2
EABT2297	1	7	10	2	0	1	1
EABT22970	16	26	28	8	4	0	4
EABT22971	0	2	4	6	0	1	0
EABT22972	2	5	13	10	1	4	1
EABT22973	1	2	8	6	1	0	0
EABT22974	0	1	3	2	0	0	0
EABT22975	1	11	2	32	13	0	0
EABT22976	1	2	1	8	0	0	0
EABT22977	91	3	37	1523	31	6	1
EABT22978	1	0	0	4	0	0	2
EABT22979	7	20	10	90	51.08	6	4
EABT2298	0	3	11	4	0	0	0
EABT22980	3.01	4	0	0	0	5	1
EABT22981	0	1	2	1	1	0	0
EABT22982	5	3	5	16	2	1	1
EABT22983	0	1	5	1	0	0	0
EABT22984	1	2	3	3	1	2	0
EABT22985	0	3	3	0	1	2	1
EABT22986	0	8	15	0	1	1	4

EABT22987	1	6	8	3	0	1	1
EABT22988	19	44	1.33	8	2	10	12
EABT22989	3	1	0	0	0	3	1
EABT2299	2	3	3	4	0	0	0
EABT22990	6513.46	7820.84	3722.85	4737.22	8499.04	6034.41	5553.04
EABT22991	1	3	10	0	0	0	5
EABT22992	0	0	9	1	0	0	0
EABT22993	1801.12	2725.59	3285.47	7826.27	4663.22	1875.34	4431.49
EABT22994	55	88.24	37	181.82	91	27	66
EABT22995	0	0	2	0	0	0	1
EABT22996	48	77.91	103.8	11	24	67	35
EABT22997	131.97	417	761.82	510	775.05	13	22
EABT22998	918.61	1633	1110.49	1909	1423.04	1014.02	1109
EABT22999	0	0	0	10	1	0	0
EABT23	0	0	6	3	2	0	0
EABT230	106.23	269	82	48	73	161	73
EABT2300	1	8	48	194	7	1	3
EABT23000	902	1406.96	1423.22	1874	819	871	952
EABT23001	0	1	5	8	2	0	0
EABT23002	1	0	11	0	0	0	0
EABT23003	485	439	216	2748	1255.19	25	52
EABT23004	0	0	3	3	35	1	0
EABT23005	0	0	0	0	14	1	10
EABT23006	1	1	0	3	0	0	0
EABT23007	0	1	31	424	2	0	0
EABT23008	2604.42	1607.03	32	132	1736.52	4123.27	1908
EABT23009	7	2	0	2	1	1	0
EABT2301	3	10	15	3	4	9	4
EABT23010	1	1	5	0	0	0	0
EABT23011	2	1	4	2	0	1	1
EABT23012	6	5	21	5	4	1	0
EABT23013	2	0	0	0	1	0	0
EABT23014	155	247	234.04	559	163	146	123
EABT23015	2.09	5	32.04	1	5	0	3
EABT23016	0	2	2	5	0	0	0
EABT23017	1	7	113	2	0	0	0
EABT23018	7	12	39	19	516.36	7.01	5
EABT23019	3432	5682.15	5004.2	6864.59	2296.46	2423.99	2561.46
EABT2302	0	0	1	0	8	0	0
EABT23020	1	1	1	0	2	0	0
EABT23021	9410.89	14134.1	10904	19644.2	9361.3	9326.68	8098.1
EABT23022	1	0	40	2	1	2	3
EABT23023	0	15	54	4	1	4	0
EABT23024	5	0	15	0	2	1	0
EABT23025	4	5	18	6	4	0	3
EABT23026	1	1	10	0	2	0	1
EABT23027	0	0	14	0	0	0	0
EABT23028	0	0	0	9	2	1	0
EABT23029	0	4	15	0	2	7	3
EABT2303	0	5	1	0	1	0	0
EABT23030	1	1	2	0	1	4	0

EABT23031	1	0	15	2	1	1	0
EABT23032	1	0	2	0	0	1	3
EABT23033	1	7	6	0	1	5	9
EABT23034	2	3	1	2	3	3	21
EABT23035	108	476	1539.84	1602.41	930.96	255	364
EABT23036	1	3	7	7	0	0	0
EABT23037	4	2	1	39	0	0	1
EABT23038	0	3	11	2	0	0	1
EABT23039	7	57	34	31	72	9	6.87
EABT2304	10926.2	37901.2	19722.2	83135	9712.94	7806.49	3202.2
EABT23040	3724.08	6791.69	1434.09	1005.13	642.73	3843.08	1363.09
EABT23041	349.88	408	67.02	1429.16	301.76	54.83	230.78
EABT23042	0	0	1	3	0	0	0
EABT23043	837.91	1277.14	1909.54	2541.41	1054.87	1125.25	1294.06
EABT23044	1	3	4	0	1	1	0
EABT23045	2	5	11.02	7	12	0	2
EABT23046	745.88	1058.01	1220.58	5202.06	860.29	436.07	461
EABT23047	2219.66	4212	4650.33	5058.84	3405.11	1751.48	2867.6
EABT23048	1	1	18	4	0	0	2
EABT23049	22	3	1	0	1	9	2
EABT2305	0	1	3	1	0	0	0
EABT23050	1	14	1	2	1	2	0
EABT23051	25	30.99	60	24	33.82	7	78
EABT23052	3273.02	8622.39	6121.75	8847.81	3357.99	2185	3130.55
EABT23053	652.72	778.75	559.59	838.61	640.04	386.6	384.96
EABT23054	3	24	0	19	3	2	2
EABT23055	1	2	2	1	0	0	0
EABT23056	2	7	17	5	3	0	1
EABT23057	12	10	0	0	0	26	13
EABT23058	2235.33	2840.11	3164.44	2454.12	2324	2433	1611
EABT23059	0	5	0	5	0	0	0
EABT2306	4	7	1	5	0	2	2
EABT23060	1	4	12	0	0	0	3
EABT23061	0	0	0	8	0	1	0
EABT23062	1	7	35	1	0	1	0
EABT23063	2	2	3	2	0	0	0
EABT23064	37	63	102	124	17	46	48
EABT23065	0	1	1	4	0	0	0
EABT23066	1	3	5	2	3	1	4
EABT23067	0	1	4	0	0	1	0
EABT23068	0	0	0	0	2	0	0
EABT23069	148	36	2	0	1	77	0
EABT2307	1	0	4	2	1	5	2
EABT23070	1	5	4	0	2	2	2
EABT23071	0	2	5	0	0	1	0
EABT23072	2	3	20	1	1	0	1
EABT23073	7	3	7	1	1	4	3
EABT23074	5018.5	4475.48	3418.48	4407.62	2711.73	4758.46	4755.69
EABT23075	0	3	4	8	1	3	4
EABT23076	0	1	5	6	0	1	2
EABT23077	0	2	0	1	1	0	0

EABT23078	12809.7	3981.16	2414	168.82	6860.92	24927.8	25421.9
EABT23079	11	36	15	46	6	2	6
EABT2308	0	0	0	0	1	0	5
EABT23080	1	0	6	1	3	0	1
EABT23081	0	1	7	3	1	2	0
EABT23082	1	0	0	5	1	0	0
EABT23083	1	4	3	48	3	1	2
EABT23084	0	1	17	31	1	3	0
EABT23085	41	73	309	2644.82	272.12	78	162
EABT23086	1	0	5	7	0	0	0
EABT23087	0	0	2	0	0	0	15
EABT23088	1	2	16	2	0	0	0
EABT23089	24	7	4.01	1	0	37	0
EABT2309	0	1	22	1	1	5	0
EABT23090	1	2	45.99	1	0	0	0
EABT23091	0	3	1	1	5	11	15
EABT23092	0	0	38	6	0	3	0
EABT23093	0	3	3	1	4	0	13
EABT23094	0	4	3	8	0	2	2
EABT23095	27	18	9	5	11	15	34
EABT23096	0	0	6	0	2	0	0
EABT23097	1210.73	1913.26	3883.24	418.29	403.75	1297.86	541.85
EABT23098	0	4	4	5	0	2	0
EABT23099	1	0	2	1	1	0	2
EABT231	4	7	0	2	2	2	1
EABT2310	2	3	14	5	0	2	1
EABT23100	99	83	100	56.02	39	474.03	70
EABT23101	6984.11	8831.97	7725.41	14509.7	5027.39	1729.78	3292.63
EABT23102	2	0	4	1	1	1	3
EABT23103	17.99	35	57.25	14	3	25	6
EABT23104	0	20	7	1	0	1	0
EABT23105	1	2	3	4	0	0	0
EABT23106	0	1	2	1	2.98	0	0
EABT23107	1	0	0	14	1	2	0
EABT23108	1566	1737	1443	2254.99	969.95	1637.01	1352
EABT23109	1109.01	1469.17	1019.83	13625.9	4115.41	95	340
EABT2311	5	30	33	95	4	20	12
EABT23110	0	4	0	0	0	3	1
EABT23111	2	1	2	1	0	2	2
EABT23112	2	7	2	0	0	1	4
EABT23113	32	58	9	7	6	162	15
EABT23114	0	1	0	0	0	0	4
EABT23115	6	18	30	10	3	2	4
EABT23116	0	1	19	0	1	0	1
EABT23117	11	6	18	16	4	3	6
EABT23118	1	13	5	3	3	1	1
EABT23119	2	2	19	0	4	14	8
EABT2312	0	0	1	5	4	1	0
EABT23120	20	9	7	0	22	19	15
EABT23121	2	7	27	2	1	0	3
EABT23122	0	0	11	1	0	0	0

EABT23123	0	1	21	1	0	1	0
EABT23124	3	1	0	0	0	0	1
EABT23125	0	3	6	1	0	0	0
EABT23126	269.96	481	542.68	626	378	188.95	202
EABT23127	0	0	4	2	5	0	1
EABT23128	2	5	0	0	0	1	0
EABT23129	6	2	2	19	1	2	0
EABT2313	0	1	2	12	0	0	1
EABT23130	0	2	0	3	0	0	1
EABT23131	1	1	46	6	0	0	1
EABT23132	0	1	5	0	0	0	0
EABT23133	10	9	14.98	4	1.39	4	1
EABT23134	46	109	50.45	269	118	45.58	85
EABT23135	3	5	0	0	0	1	0
EABT23136	0	4	1	1	8	0	9
EABT23137	0	2	17	4	0	0	0
EABT23138	163	425.01	264	226.9	167	103	89
EABT23139	3	8	5	8	2	0	2
EABT2314	1	0	13	0	1	1	0
EABT23140	2	4	9	3	1	5	2
EABT23141	0	0	4	0	2	0	0
EABT23142	0	0	6	0	0	1	0
EABT23143	145.03	290	57	18	372.5	97	89
EABT23144	173.2	249.01	6	1	2	160.01	3
EABT23145	3	1	20	0	3	1	1
EABT23146	3	5	8	2	1	0	0
EABT23147	0	3	12	1	2	0	1
EABT23148	0	6	5	0	0	0	2
EABT23149	2	9	2	13	11	0	3
EABT2315	0	1	18	1	0	1	0
EABT23150	5	4	0	0	1	10	6
EABT23151	0	1	2	1	0	0	1
EABT23152	8	1	9	7	5	6	5
EABT23153	722.77	1074.72	2167.69	2097.01	1269	22	223
EABT23154	522.89	709.5	304	1019.91	384.84	199	229.63
EABT23155	5	6	110	21	5	4	4
EABT23156	3	5	2	15	0	0	2
EABT23157	0	5	4	1	0	0	0
EABT23158	1	1	31	14	81	0	0
EABT23159	2	0	23	2	0	1	1
EABT2316	17	14	29	0	6	0	0
EABT23160	0	1	0	9	0	0	1
EABT23161	2	3	0	1	0	1	0
EABT23162	2	9	7	0	1	1	2
EABT23163	0	0	2	3	1	0	0
EABT23164	6	18	9	11	1	3	4
EABT23165	0	2	14	4	1	2	1
EABT23166	0	3	1	15	1	0	3
EABT23167	21	31.23	26	589.02	109	21	48
EABT23168	4	2	23	6	6	10	0
EABT23169	0	3	5	20	1	0	1

EABT2317	28	80	12	692.94	94	152	320
EABT23170	6	9	30	1	1	0	2
EABT23171	18	21	22	34	13	3	13
EABT23172	40	89	70	95	31	42.98	43
EABT23173	0	4	3	9.99	27	0	7
EABT23174	0	6	16	5	2	0	0
EABT23175	2	4	3	7	8	4	4
EABT23176	1	5	0	12	2	0	2
EABT23177	0	1	5	0	2	0	0
EABT23178	0	0	73	0	0	1	0
EABT23179	0	0	6	0	2	1	2
EABT2318	0	0	2	6	2	0	0
EABT23180	1	2	3	1	0	1	2
EABT23181	0	1	9	2	0	2	0
EABT23182	2	0	1	2	0	0	3
EABT23183	3	2	0	0	0	2	0
EABT23184	1	2	14	2	0	0	0
EABT23185	192	229	146.74	452.42	214	71.77	94.95
EABT23186	0	1	6	0	3	3	25
EABT23187	1	4	29	60	3	4	1
EABT23188	1057.69	1494.37	931.78	2394.22	1123.62	623.04	919.87
EABT23189	4316.31	6104.32	9315.3	9	14	1253.42	32
EABT2319	0	1	2	5	1	0	0
EABT23190	0	2	2	2	1	0	0
EABT23191	0	1	0	9	0	0	0
EABT23192	1	1	1	3	1	0	0
EABT23193	2	4	13	13	2	1	0
EABT23194	0	1	5	2	0	0	0
EABT23195	9	14	3	2	2	0	2
EABT23196	2	5	3	5	3	1	4
EABT23197	0	2	6	0	0	0	0
EABT23198	494.56	860.77	4263.91	2322.16	469.94	901.27	271.17
EABT23199	4547.64	6404.85	7.01	4	4500.89	3788.49	16169.7
EABT232	3	2	4	17	3	1	2
EABT2320	0	0	12	2	0	0	0
EABT23200	1	0	6.3	0	0	3	0
EABT23201	1	0	4	5	0	0	0
EABT23202	0	0	2	0	1	0	0
EABT23203	0	0	6	0	1	0	0
EABT23204	0	0	13	0	0	0	0
EABT23205	2794.86	3033.07	2886.17	3460.04	1778.91	2975	2664.85
EABT23206	1	0	0	0	0	4	1
EABT23207	2428.8	1663.05	7	5	16	58.45	23
EABT23208	1	0	0	0	1	0	0
EABT23209	3	2	4	1	0	1	0
EABT2321	1	0	9	1	0	0	0
EABT23210	1179.38	1217.18	1061.85	454.87	87	2144.2	112.92
EABT23211	1	5	1	0	0	0	0
EABT23212	69	102	126	71	260.03	3	8
EABT23213	0	0	0	0	2	0	13
EABT23214	1141.11	2928.98	5719.14	7637	1788.94	1545	1252

EABT23215	1	0	2	10	12	0	0
EABT23216	3	8	5	0	3	4	5
EABT23217	49	204.02	300	362.77	711.01	6	304.01
EABT23218	0	1	1	2.41	2	0	2
EABT23219	2	4	22	2	8	3	16
EABT2322	0	1	9	0	0	0	0
EABT23220	3236.72	6527.52	3712.99	1885.93	1928.35	3533.66	3792.22
EABT23221	2	5	11	9	0	2	2
EABT23222	1	1	13	0	1	2	0
EABT23223	5	7	0	0	0	0	2
EABT23224	1	1	13	2	0	3	0
EABT23225	0	0	36	0	0	0	0
EABT23226	3	3	6	3	0	0	1
EABT23227	0	4	6	8	2	1	1
EABT23228	5	14	14	3	3	1	0
EABT23229	1	5	1	2	0	1	1
EABT2323	0	3	0	2	1	2	7
EABT23230	0	1	2	7	3	0	0
EABT23231	0	1	15	0	0	0	0
EABT23232	0	0	3	2	0	2	0
EABT23233	2	1	4	8	1	1	3
EABT23234	0	0	3	0	2	0	0
EABT23235	2	0	9	2	0	1	0
EABT23236	0	2	18	2	1	8	0
EABT23237	0	2	1	2	0	0	1
EABT23238	0	2	4	2	0	0	1
EABT23239	1	0	1	0	3	0	5
EABT2324	1	13	1	4	1	1	0
EABT23240	1	0	27.04	0	0	0	0
EABT23241	1433.14	1930	854.12	1016.74	1531	1251.99	1867.02
EABT23242	1	4	15	3	0	1	2
EABT23243	1	2	1	6	10	0	0
EABT23244	2	7	1	5	1	2	8
EABT23245	2	2	40.54	2	0	2	0
EABT23246	2	4	10	4	7	3	0
EABT23247	5	7	0	0	0	21	16
EABT23248	0	8	0	3	0	0	0
EABT23249	8	34	57	41	3	19	12
EABT2325	2	6	15.99	9	2	3	4
EABT23250	9	12	3	1	0	13	9
EABT23251	289.73	1111.09	3640.94	1874.93	1170	224.89	352
EABT23252	2643.97	5012.78	4644.72	10097.9	2234.84	3023.85	1916
EABT23253	0	1	8	0	1	0	0
EABT23254	0	0	9	0	0	0	0
EABT23255	5	4	24	23	0	6	2
EABT23256	3	2	0	7	7	6	3
EABT23257	0	2	27	0	2	1	0
EABT23258	2	14	17	8	1	4	3
EABT23259	3262.03	5319.15	2946.96	6859.24	3737.97	2187.11	2555.86
EABT2326	2	1	11	0	0	0	0
EABT23260	0	1	4	3	4	1	4

EABT23261	0	4	76	3	0	0	0
EABT23262	2028.78	3726.82	3069.45	3799.49	3405.96	2689.83	2137.1
EABT23263	0	1	2	1	7	0	0
EABT23264	0	0	1	0	0	0	1
EABT23265	2506.44	3662.85	4144.59	6476.16	3890.96	1951.81	2793.08
EABT23266	1	1	6	1	0	0	0
EABT23267	0	0	21	0	0	0	0
EABT23268	1	1	8	0	0	0	0
EABT23269	847	1461	248	158	4346	815.12	341
EABT2327	2	0	82.99	1	1	1	0
EABT23270	2	4	7	4	11	0	1
EABT23271	0	1	8	2	0	0	0
EABT23272	2	5	4	33	2	1	0
EABT23273	1	1	3	4	0	1	6
EABT23274	3498.53	7279.73	10705	720	9	1	0
EABT23275	0	0	2	4	0	0	0
EABT23276	4	13	7	2	0	16	8
EABT23277	5	1	41	7	14	1	10
EABT23278	5	3	20	0	5	0	26
EABT23279	3	1	3	4	0	0	3
EABT2328	1	3	3	0	0	8	1
EABT23280	0	2	4	43	3	0	1
EABT23281	57	7.98	16	1	0	129	0
EABT23282	3	1	0	4	6	1	2
EABT23283	1782	1951.99	1126	2209	1220.92	1504.97	1194
EABT23284	146	50	26	447	166	29	813
EABT23285	17	13	14	4	3	13	4
EABT23286	189	486	186	1539	689	0	11
EABT23287	1	0	7	1	0	1	0
EABT23288	0	5	0	1	0	0	0
EABT23289	1518.91	2071.72	1985	2804.7	1739.2	1671.84	1191
EABT2329	0	4	1	13	0	0	0
EABT23290	3	1	13	3	0	0	3
EABT23291	0	5	14	8	1	4	0
EABT23292	5	54	45	40	26	7	12
EABT23293	647.95	959.34	651.92	1784.83	955	293	465.15
EABT23294	0	0	3	0	0	1	0
EABT23295	0	1	17	2	0	0	0
EABT23296	3472.8	4739.51	3770.07	8762.87	2385.98	3377	2911
EABT23297	0	4	4	0	0	0	0
EABT23298	16378.4	26647.2	26562.7	24572.2	11200.9	2359.36	2359.14
EABT23299	1820.05	2647.02	2316.82	2750.03	1676	1063	1105.96
EABT233	0	2	7	0	0	1	1
EABT2330	0	0	20	0	1	0	0
EABT23300	0	5	7	6	11.02	0	1
EABT23301	66	200	76	52	14	37	56
EABT23302	0	0	1	0	0	0	0
EABT23303	0	1	3	9	0	0	0
EABT23304	0	4	1	1	2	0	2
EABT23305	1	6	3	2	1	0	0
EABT23306	0	1	9	4	0	0	0

EABT23307	0	0	6	0	1	0	0
EABT23308	2	1	7.7	7	1	0	0
EABT23309	0	1	4	6	0	0	0
EABT2331	8751.77	8765.36	1528.38	1446.11	4422.51	7276.79	6878.91
EABT23310	3	2	7	2	1	0	3
EABT23311	5275.46	8132.05	10495.4	1968.43	5938.4	3479.19	3067.7
EABT23312	0	1	3	0	0	0	0
EABT23313	2611.93	4392.2	3385.73	5033.43	2932.41	2448.14	3514
EABT23314	2	7	24	16	2	2	4
EABT23315	1	7	3	0	1	8	3
EABT23316	0	0	5	3	0	0	0
EABT23317	1	2	1	15	3	0	0
EABT23318	30	44	41	107	8	0	14
EABT23319	7	14	245	45	4	18	6
EABT2332	0	1	4	0	0	0	0
EABT23320	0	0	0	0	0	0	0
EABT23321	0	0	0	0	0	0	0
EABT23322	1	1	0	0	0	5	0
EABT23323	0	1	6	1	0	0	0
EABT23324	110	264.01	408.9	143	336	101	236
EABT23325	0	6	4	11	10	1	4
EABT23326	1	0	2	0	0	5	0
EABT23327	2	2	3	0	3	0	0
EABT23328	1	5	1	4	0	0	0
EABT23329	9	10	23	6	4	2	16
EABT2333	8	8	45	6	1	2	2
EABT23330	3	2	36	1	0	0	1
EABT23331	0	0	9	14	0	0	0
EABT23332	2	4	3	18	2	0	4
EABT23333	300.61	135.01	38	16	2164.11	33	37
EABT23334	1	1	1	0	1	3	0
EABT23335	1	0	3	0	0	3	0
EABT23336	9	4	10	3	10	0	2
EABT23337	0	1	2	0	2	1	2
EABT23338	1	7	48	9	1	2	1
EABT23339	1	0	61	0	1	0	0
EABT2334	1648.67	2230.12	2348.79	4277.41	1460.78	2515.7	1821.22
EABT23340	10	43	9	27	14	23	4
EABT23341	4	2	10	1	0	3	3
EABT23342	1	5	2	2	1	0	0
EABT23343	0	0	3	7	0	0	0
EABT23344	0	1	10	0	0	0	0
EABT23345	434	789.99	2126.32	1646.2	1699.37	348.03	429.86
EABT23346	2809.19	3047.91	3521.56	2914.92	2070.05	3817.53	3281.55
EABT23347	0	0	10	1	0	0	0
EABT23348	4	2	4	3	0	0	2
EABT23349	0	0	4	0	0	0	0
EABT2335	8	11	77.5	95.69	249.33	9	50
EABT23350	308	5269.76	15984.7	154	217	39	71
EABT23351	0	1	8	0	0	0	0
EABT23352	206	619.02	601	1861	209.32	275	316.04

EABT23353	2	2	10	5	4	0	1
EABT23354	11.01	20	146	6	8	4	22
EABT23355	4	4	9	7	1	0	0
EABT23356	1	22	17	2	1	6	2
EABT23357	0	2	19	1	0	1	0
EABT23358	1	3	0	4	0	0	0
EABT23359	3	2	39	6	0	0	0
EABT2336	0	0	3	0	0	0	1
EABT23360	0	0	0	0	0	0	0
EABT23361	1	0	10	0	0	0	0
EABT23362	0	0	2	9	2	0	2
EABT23363	0	2	4	0	1	0	2
EABT23364	0	1	0	3	5	0	0
EABT23365	0	0	1	0	1	0	1
EABT23366	0	8	7	13	0	0	1
EABT23367	1	4	22	2	0	3	1
EABT23368	0	2	11	5	1	0	0
EABT23369	0	2	25	0	0	0	0
EABT2337	4419.68	3182.36	1090.99	3423.42	1789.41	3402.02	2273.95
EABT23370	263	353.2	157	553	295	144	140.93
EABT23371	1	4	0	0	0	1	1
EABT23372	8	21	37	25	3	5	3
EABT23373	0	1	3	5	5	0	3
EABT23374	215.11	96	106	155.75	16	22	7
EABT23375	0	0	0	3	0	2	0
EABT23376	1131.21	1510.83	1067.1	2011.53	1722.44	1419.39	1066.33
EABT23377	15	15	7	22	13	3	15
EABT23378	3	3	62	7	2	4	2
EABT23379	0	3	1	1	2	0	0
EABT2338	0	0	4	7	0	0	1
EABT23380	39	57	137	9	0	1	1
EABT23381	0	1	12	1	0	1	0
EABT23382	0	0	6	0	0	1	1
EABT23383	0	2	3	6	0	1	0
EABT23384	61	6	3	0	5	4	30.02
EABT23385	8	13	12	75	10	0	11
EABT23386	0	0	3	0	0	0	1
EABT23387	0	2	7	2	1	0	0
EABT23388	1	3	12	7	1	1	3
EABT23389	17	24	1	0	0	16	1
EABT2339	2	6	9	5	7	0	1
EABT23390	23	31	0	4	10	29.13	56
EABT23391	29	69	91	92	4	8	6
EABT23392	1	0	5	0	1	0	0
EABT23393	0	0	2	0	0	0	1
EABT23394	191	323	377.04	328.81	128	144	196.19
EABT23395	1	9	8	10	1	0	3
EABT23396	2408.34	3396.42	3432.35	4668.35	2944.98	3120.39	2214.27
EABT23397	0	2	2	0	0	1	0
EABT23398	5	29	12	44	11	0	1
EABT23399	201.25	513.59	982	33221.6	47	11	3

EABT234	179	527.99	1311.27	14330.1	508.01	144.83	107
EABT2340	2	2	5	4	0	0	0
EABT23400	513.1	993.48	575.92	1768.91	179	218.98	309
EABT23401	56	26	28	35	7	60	19
EABT23402	1	2	33	2	0	0	0
EABT23403	0	7	18	4	1	1	1
EABT23404	75	108	159	176.96	155	118	136
EABT23405	9354.07	10364.1	13577.9	1323	5256.85	672	938.83
EABT23406	0	0	10	3	0	0	0
EABT23407	13	27	28	5	14	32	172.81
EABT23408	6021.45	6812.75	3479.94	4746.55	2024.23	6439.61	4607.68
EABT23409	612.58	838	844	1775.81	831	68	341.83
EABT2341	6	6	19	6	1	5	1
EABT23410	10	6	6	12.99	13	0	0
EABT23411	0	1	3	4	12	1	0
EABT23412	4	2	4	4	0	2	1
EABT23413	1582	2300.88	4254.93	13169.2	1108	2386.88	2798.94
EABT23414	0	1	5	1	2	1	2
EABT23415	4	11	18	27	11	0	1
EABT23416	0	1	9	0	0	0	0
EABT23417	0	0	1	0	2	0	0
EABT23418	0	0	4	0	0	0	1
EABT23419	5	7	6	3	2	3	4
EABT2342	1	12	6	3	2	1	12
EABT23420	0	1	7	1	3	1	2
EABT23421	3	11	13	15	1	0	1
EABT23422	2358.72	1606.03	31	0	477	6866.99	2401.97
EABT23423	0	0	9	1	1	1	0
EABT23424	5	4	5	1	2	11	1
EABT23425	0	0	4	2	1	1	1
EABT23426	0	2	8	0	0	0	0
EABT23427	0	1	5	2	0	0	2
EABT23428	0	0	13	0	0	1	0
EABT23429	0	1	10	4	0	5	0
EABT2343	0	0	2	0	0	0	0
EABT23430	0	0	12	0	0	0	0
EABT23431	1	0	5	2	3	0	0
EABT23432	6	13	8	19	0	11	11
EABT23433	783	1054.42	957	1497.01	889	674	859
EABT23434	0	2	13	1	1	0	2
EABT23435	2	1	1	1	15	0	0
EABT23436	69	231	453	5241.2	10917.3	2	61
EABT23437	30	5	1	1	0	29	0
EABT23438	1	0	10	7	4	1	1
EABT23439	0	1	6	0	1	0	0
EABT2344	993.64	1091.43	1354.55	1427.9	677.94	662.37	750
EABT23440	3	5	4	1	2	8	9
EABT23441	0	4	19	5	1	0	3
EABT23442	6	13	0	0	0	5	1
EABT23443	0	0	0	0	1	0	0
EABT23444	2701.59	3614.88	2812.07	2367.58	1890.8	1610.47	1678.26

EABT23445	0	1	2	2	1	0	0
EABT23446	0	0	7	0	0	0	0
EABT23447	3	62	525.02	41014.3	46	0	1
EABT23448	0	2	0	10	2	0	0
EABT23449	15	61.99	16	13	5	81	9
EABT2345	28	48	36	111	42	3	1
EABT23450	0	1	4	0	0	0	0
EABT23451	0	3	17	5	0	0	0
EABT23452	1	4	13	8	0	0	0
EABT23453	1	1	12	6	13	2.18	20
EABT23454	0	5	3	1	0	0	1
EABT23455	839.94	1133.97	782.37	2296.69	1301.96	534	933.21
EABT23456	1160.49	1468.49	1198.73	1882.18	913.6	1245.99	1178.01
EABT23457	0	1	6	1	0	0	0
EABT23458	1	1	22	1	0	0	0
EABT23459	1	1	0	1	0	1	1
EABT2346	0	4	5	1	0	0	1
EABT23460	5	3	13	8	2	2	0
EABT23461	34	48	58	206.81	181	125	280
EABT23462	713.1	1280.79	691.32	1555.02	921.94	605	583.04
EABT23463	0	0	0	0	0	0	0
EABT23464	0	4	1	3	0	1	0
EABT23465	31	375	944	1820	554	12	65
EABT23466	3	4	35	3	4	0	3
EABT23467	10	22	22	37	10	6	14
EABT23468	2	5	5	5	2	0	0
EABT23469	0	0	4	3	0	0	0
EABT2347	341.12	497.01	478.69	1362.22	743.32	328.67	361
EABT23470	1	2	3	6	2	0	1
EABT23471	0	2	5	4	1	1	0
EABT23472	0	2	10	2	1	1	5
EABT23473	74691	147285	336139	679.27	24369.6	715.66	5047.51
EABT23474	1	7	0	0	0	2	0
EABT23475	3	9	84	1	0	0	1
EABT23476	0	0	6	0	0	0	0
EABT23477	1	0	1	0	0	0	2
EABT23478	5	7	10	0	1	0	4
EABT23479	4	3	13	1	0	0	0
EABT2348	0	0	0	24	2	0	0
EABT23480	4	1	5	3	0	2	1
EABT23481	2668.35	3912.96	3975.86	5323.18	3421.26	1967.88	2174.4
EABT23482	2	2	0	0	0	4	2
EABT23483	10	28	28	32	14	9	7
EABT23484	0	1	4	0	1	0	1
EABT23485	35.09	38	15	51	7	1	1
EABT23486	2	3	2	15	2	0	1
EABT23487	0	4	1	5	4	3	5
EABT23488	2698.28	3169.34	1766.7	2998.88	2092.26	1574.55	1897.87
EABT23489	9303.22	5746.36	2391.6	1716.96	2836.67	4764.83	2491.98
EABT2349	1	0	5	2	0	1	0
EABT23490	33	33.18	0	0	1	5	5

EABT23491	940.01	2516.79	1655.35	4504.63	1126.98	638.26	453.7
EABT23492	0	0	4	0	0	0	0
EABT23493	9	9	4	3	7	4	29
EABT23494	6	0	8	1	0	0	0
EABT23495	0	0	8	2	0	1	1
EABT23496	0	0	6	1	1	0	1
EABT23497	5082.32	6051.29	3712.73	4950.37	2527.41	3671.12	2720.27
EABT23498	0	0	0	1	0	3	0
EABT23499	8	3	5	2	0	9	0
EABT235	0	1	3	1	1	0	0
EABT2350	1	2	13	6	0	2	0
EABT23500	3	13	5	12	2	2	0
EABT23501	15	5	0	6	0	5	5
EABT23502	0	1	0	0	1	2	5
EABT23503	35	93	35	125	76	114	218
EABT23504	0	1	2	0	1	0	1
EABT23505	0	0	19	1	0	0	1
EABT23506	0	0	6.82	0	0	0	0
EABT23507	1	1	29	1	0	0	4
EABT23508	0	0	0	0	0	7	1
EABT23509	1	1	10	0	0	0	0
EABT2351	64	145	130	1371.15	1341.19	67	58
EABT23510	7	13	23	13	2	8	15
EABT23511	1	2	8	1	1	1	1
EABT23512	4	5	12	8	1	0	0
EABT23513	0	0	13	0	0	0	0
EABT23514	0	0	0	6	2	0	0
EABT23515	1	2	2	6	1	0	0
EABT23516	1	0	4	4	1	1	1
EABT23517	0	0	3	0	0	1	4
EABT23518	0	2	16	0	0	0	0
EABT23519	1	1	6	0	0	0	0
EABT2352	0	1	1	1	0	0	0
EABT23520	9.01	14	35	16	4	17	5
EABT23521	0	1	1	0	15	4	112.59
EABT23522	1	1	3	13	0	2	0
EABT23523	0	0	22	0	0	0	1
EABT23524	1	1	9	0	1	0	0
EABT23525	1	0	5	0	0	0	1
EABT23526	659	1099	1202.35	2291.42	865.91	456.71	569.61
EABT23527	78	215	85	530	35	10	30
EABT23528	0	1	0	0	2	0	2
EABT23529	105	67.98	32	7	19	90	29
EABT2353	2	0	0	1	0	0	24
EABT23530	42	1608.62	14	0	16	990	444
EABT23531	0	1	12	1	0	1	2
EABT23532	24	34	93	3	18	2	33
EABT23533	15	57	46	414	705	1	12
EABT23534	10	14	2	26	6	4	5
EABT23535	18039	19868.5	15210.4	5231	6618.33	1858	2743
EABT23536	1628.01	1026	61	232	1149	1765.07	4440.34

EABT23537	1	2	1	0	4	0	0
EABT23538	0	2	1	0	9	0	0
EABT23539	0	0	3	20	0	0	0
EABT2354	7	0	17	9	1	1	0
EABT23540	1	1	7	0	0	0	0
EABT23541	0	0	2	0	0	2	0
EABT23542	0	1	4	6	0	1	0
EABT23543	1	4	27	4	0	2	2
EABT23544	7	6	27	14	3	3	0
EABT23545	0	0	11	0	0	0	0
EABT23546	1	0	0	0	0	7	1
EABT23547	2	0	22	19	3	1	3
EABT23548	4	21	1	5	9	0	2
EABT23549	15	5	1	0	0	0	0
EABT2355	0	0	0	0	0	0	0
EABT23550	0	0	0	0	0	0	0
EABT23551	1	7	3	3	2	1	1
EABT23552	1	1	16	1	0	2	0
EABT23553	7	10	21	2	0	1	1
EABT23554	701.92	949.05	666.88	926.77	734.12	315.58	574.22
EABT23555	765.19	1049.72	555.03	1031.84	632.59	366.02	356.95
EABT23556	0	2	1	7	0	0	1
EABT23557	0	0	0	11	1	0	0
EABT23558	7094.12	12247.9	6542.28	10325.3	5958.76	4626.54	5713.03
EABT23559	0	1	9	2	0	0	0
EABT2356	0	2	0	3	0	0	0
EABT23560	0	0	2	2	2	0	0
EABT23561	15932.4	16850.9	11816.2	14406.7	11818	12950.6	13097.7
EABT23562	1	5	17	0	0	0	1
EABT23563	0	5	0	1	0	0	1
EABT23564	0	0	0	4	1	2	0
EABT23565	417	466.08	787.9	802.09	527	377	332
EABT23566	631	1297.96	20072.1	7974.73	1245.07	1112.27	953.88
EABT23567	2	5	8	2	0	0	0
EABT23568	723.43	1531.11	1686.76	2162.03	836.51	819.64	915.05
EABT23569	1	1	4	2	0	0	0
EABT2357	0	0	32	2	1	2	0
EABT23570	13	41	60	69	60	26	55
EABT23571	166.15	323.31	235.29	233.16	420.93	0	6.26
EABT23572	2659.07	758.99	67	26	653	21	657.99
EABT23573	18	35	87	77	15	22	21
EABT23574	3881.39	6073.94	7313.59	26599.5	4017	4181	3539.95
EABT23575	1	1	5	3	0	0	2
EABT23576	2	3	7	24	25	1	0
EABT23577	0	0	2	1	0	1	1
EABT23578	2	3	4	9	1	0	0
EABT23579	0	2	7	7	1	0	1
EABT2358	0	0	4	0	0	0	0
EABT23580	0	0	2	0	0	0	1
EABT23581	3	2	5	16	3	1	9
EABT23582	0	0	1	2	1	0	1

EABT23583	0	5	3	1	0	7	2
EABT23584	1	0	6	0	1	0	1
EABT23585	0	5	17	1	1	0	0
EABT23586	0	3	1	13	0	1	1
EABT23587	1	3	1	2	0	0	1
EABT23588	0	3	14	3	0	1	0
EABT23589	2889.08	4578.78	3324.68	3005.83	1639.08	4672.11	3005.11
EABT2359	0	4	3	1	0	1	1
EABT23590	4	134.49	1	0	15	49	20
EABT23591	7	25	36	23	13	4	1
EABT23592	0	0	5	1	4	2	9
EABT23593	3	3	0	0	2	0	2
EABT23594	743.73	1370.79	824.95	1737.97	851.3	1123.65	1258.06
EABT23595	853.86	1173.34	1098.81	1750.96	979.23	725.33	695.34
EABT23596	0	0	10	0	0	0	0
EABT23597	2	2	1	0	0	6	2
EABT23598	1431.55	3846.02	4641.03	5384.85	3866.96	1814.35	2427.34
EABT23599	0	0	0	8	2	0	0
EABT236	164	570.3	2254.84	347	496.62	125	132
EABT2360	1	0	7	2	0	0	0
EABT23600	5	7	45	27	5	2	8
EABT23601	12	26	31	15	16	21	11
EABT23602	1	4	61.01	1	1	0	0
EABT23603	0	1	5	0	0	1	2
EABT23604	6	9	9	35	5	7	8
EABT23605	8	28	6	157	9	1	5
EABT23606	0	1	15	9	10	0	0
EABT23607	8	30	1	5	1	6	1
EABT23608	3	1	4	0	0	0	0
EABT23609	2	3	2	12	0	0	1
EABT2361	0	0	10	1	0	0	0
EABT23610	2	14	10	7	19	1	8
EABT23611	18	18	0	0	1	58	29
EABT23612	0	0	0	3	6	0	0
EABT23613	11	10	0	0	1	0	6
EABT23614	1	3	0	0	0	0	0
EABT23615	3	4	10	0	6	2	0
EABT23616	6759.01	6247.72	1641	2336.14	2748.17	16	180
EABT23617	0	1	0	8	2	1	0
EABT23618	2	1	4	1	0	1	0
EABT23619	0	3	13	3	0	0	1
EABT2362	0	5	11	2	1	0	0
EABT23620	0	0	0	0	0	0	0
EABT23621	2	2	7	3	1	6	1
EABT23622	4870.6	5332.24	2743.97	7936.36	3248.49	3915.21	3377.5
EABT23623	0	2	4	1	0	0	0
EABT23624	0	0	1	0	1	0	0
EABT23625	374	555.72	672.52	860.13	755.99	293.81	325.09
EABT23626	1	0	5	0	0	0	0
EABT23627	1	2	6	4	0	0	0
EABT23628	264	379.33	286	919	285	177	158

EABT23629	886	1381.7	1263.36	2835.64	1089.52	422.53	665.25
EABT2363	0	0	27	4	1	0	0
EABT23630	0	1	4	2.07	1	1	0
EABT23631	2	0	9	0	0	0	0
EABT23632	0	1	0	1	9	3	32
EABT23633	9	2	49	19	1	5	0
EABT23634	0	1	23	1	1	1	0
EABT23635	2	0	2	6	0	0	3
EABT23636	2	5	28	19	0	0	0
EABT23637	1	5	2	3	9	0	4
EABT23638	0	0	4	1	0	0	0
EABT23639	1	3	3	2	0	1	3
EABT2364	3	2	2	4	1	0	20.97
EABT23640	1	2	18	1	0	0	1
EABT23641	0	0	2	0	5	0	0
EABT23642	0	0	2	1	0	1	3
EABT23643	0	0	3	2	1	0	0
EABT23644	2745.46	5014.88	2730.94	2896.71	3704.66	1712.08	3418.35
EABT23645	3	4	12	7	0	5	2
EABT23646	1	0	88.98	0	0	0	0
EABT23647	459.01	1090.68	538	1114.68	432.7	224.12	276.04
EABT23648	0	0	5	0	0	1	0
EABT23649	0	0	16	3	0	0	0
EABT2365	0	0	8	0	1	0	1
EABT23650	1463.42	1926.95	2422.25	2013.9	1558.1	1793	1318.76
EABT23651	362.92	473.43	555.97	811.72	393.07	388.54	269.63
EABT23652	1	1	5	0	0	0	0
EABT23653	4	4	0	0	0	0	0
EABT23654	0	11	476	2	1	0	0
EABT23655	0	0	2	13	0	0	0
EABT23656	1	0	7	3	1	0	2
EABT23657	0	3	21	3	3	0	0
EABT23658	0	3	20	93	1	3	1
EABT23659	0	0	8	0	0	1	0
EABT2366	0	2	24	3	1	0	0
EABT23660	3	6	4	83	6	2	0
EABT23661	7	2	0	0	0	0	0
EABT23662	1	7	2	0	0	0	0
EABT23663	5	0	0	0	1	14	10
EABT23664	0	2	2	9	1	0	0
EABT23665	1	1	3	1	0	0	0
EABT23666	0	0	12	2	0	0	1
EABT23667	569	823	1540.77	1446	749	492.01	534.01
EABT23668	1	2	5	1	1	0	0
EABT23669	8	22	25	54	1	0	4
EABT2367	0	0	20	0	0	0	0
EABT23670	1	3	1	7	3	1	3
EABT23671	0	2	8	0	0	0	0
EABT23672	0	4	4	1	0	0	0
EABT23673	904.95	1335.18	2166.71	1994.39	818.9	421.19	687.84
EABT23674	20	37	48	94	24	18	19.95

EABT23675	1531.98	1027.06	956	1414.43	756	438.54	479
EABT23676	0	0	0	1	1	0	1
EABT23677	76	161	164.98	5	102.98	2	6
EABT23678	2	1	4	23	1	0	0
EABT23679	2342.3	1899.9	1336.42	1395.1	3574.03	750	690
EABT2368	113	293	720.99	5466.62	881	38	162
EABT23680	0	0	4	1	1	6	0
EABT23681	2	7	11	5	7	2	0
EABT23682	23	44	112	16	40	32	60
EABT23683	3	1	7.01	3	1	2	2
EABT23684	113	235	231	29811.8	9226.33	32	53
EABT23685	40	35	4	0	25	46	0
EABT23686	7	17	198	18.09	0	0	0
EABT23687	0	0	0	7	2	0	0
EABT23688	5	2	0	7	1	0	2
EABT23689	0	1	0	7	1	0	0
EABT2369	0	0	0	15	0	0	0
EABT23690	1541.93	2185.18	1774.23	4067.63	2080.25	1247.99	1740.23
EABT23691	0	4	2	2	0	2	4
EABT23692	4	12	1	0	3	0	0
EABT23693	0	3	3	3	0	0	1
EABT23694	0	3	6	2	8	0	2
EABT23695	4	8	8	22	4	5	12
EABT23696	0	1	10	1	0	0	0
EABT23697	1	0	2	4	2.25	0	1
EABT23698	0	0	1	0	2	0	1
EABT23699	1	6	15	2	0	0	4
EABT237	1	0	0	0	5	0	1
EABT2370	6	5	10	6	1	0	1
EABT23700	1	1	0	1	0	0	0
EABT23701	8	35.98	7	3	28	40	404
EABT23702	1	2	7	4	0	0	1
EABT23703	0	0	6	0	0	1	1
EABT23704	4	4	19	2	1532	2	3
EABT23705	9	11	21	87	494	1	2
EABT23706	8	9	1	0	0	13	0
EABT23707	7377.07	7899.23	1376.28	2057.5	1400.98	13994.4	5661.29
EABT23708	32	15	96	6	1	108	39
EABT23709	0	1	2	3	3	0	0
EABT2371	0	0	21	2	1	3	1
EABT23710	1	4	6	6.1	5	1	7
EABT23711	0	3	5	3	0	2	0
EABT23712	454	435	147	677.08	401	26	25
EABT23713	0	1	6	25	1	0	0
EABT23714	892.62	1651.44	1863.1	3229.59	1141.99	778.3	1236.14
EABT23715	103	87	52	17	265.01	113	363
EABT23716	11404.5	5634.91	1560.04	111	2600.2	9283.66	15627.8
EABT23717	72.01	164	841.83	1921.4	183.27	9	343.58
EABT23718	5517.63	6807.5	6299.32	7431.38	3385.27	6208.33	4456.21
EABT23719	62	224	152	334.19	4	87	26
EABT2372	3	3	4	0	2	0	0

EABT23720	2	0	24	0	1	34	28
EABT23721	0	0	8	1	0	1	0
EABT23722	1	2	5	0	0	0	0
EABT23723	5	0	1	0	0	1	0
EABT23724	0	0	21	0	0	0	0
EABT23725	1	1	20	5	1	3	1
EABT23726	3	1	53	13	2	3	6
EABT23727	10	7	30	5.43	7	81.35	7
EABT23728	1976.28	2443.22	2871.19	3375.95	1380.11	584.71	820.89
EABT23729	5	14	0	0	0	9	8
EABT2373	39	17	6	0	3	6	3
EABT23730	0	0	3	1	1	0	0
EABT23731	0	1	2	0	0	1	1
EABT23732	3	14	73	17	4	5	1
EABT23733	0	0	3	1	1	1	1
EABT23734	1	7	0	8	0	0	0
EABT23735	46.25	48	23	10	35	117	237
EABT23736	4	8	0	0	2	5	2
EABT23737	0	1	21	0	0	0	0
EABT23738	14	28	10	13	23	58	1
EABT23739	0	3	2	1.83	0	0	0
EABT2374	0	1	12	0	1	0	0
EABT23740	0	0	15	1	0	0	0
EABT23741	0	0	1	2	1	0	0
EABT23742	1	4	16	6	0	0	0
EABT23743	1	4	10	3	2	1	0
EABT23744	1	2	39	0	1	4	1
EABT23745	3	2	0	0	2	0	0
EABT23746	4	2	9	2	0	1	4
EABT23747	4	1	6	2	0	0	0
EABT23748	4809.88	6302.14	2601.98	3824.98	2437.35	5484.82	2699.31
EABT23749	6	1	3	0	0	1	2
EABT2375	1498.1	1832.96	566.42	1030.27	1357.75	1504.36	1382.75
EABT23750	1	4	3	1	0	0	2
EABT23751	0	2	1	4	1	0	1
EABT23752	206.25	391.2	635.21	560.5	340.25	188	340.04
EABT23753	3	18.99	16	3	8	4	2
EABT23754	0	0	1	0	0	1	0
EABT23755	397.98	779.58	443.99	816.02	426.38	425.67	536.1
EABT23756	0	1	13	0	0	1	1
EABT23757	0	1	10	0	0	0	2
EABT23758	0	0	16	2	0	0	0
EABT23759	0	0	14	4	0	0	0
EABT2376	3	6	7	2	1	1	0
EABT23760	0	1	10	0	0	0	0
EABT23761	1	1	14	1	0	0	0
EABT23762	1	0	0	0	0	9	0
EABT23763	0	0	1	0	1	1	1
EABT23764	0	7	11	44	63	5	5
EABT23765	0	1	2	2	0	0	0
EABT23766	0	2	1	1	2	2	2

EABT23767	28.05	47	1177.93	16	6	29	22
EABT23768	0	1	1	7	1	3	1
EABT23769	9	20	29	13	19	3	20
EABT2377	615.02	1166.96	1205.39	1139.7	681.25	506.51	479.02
EABT23770	5	2	1	1	5	2	27
EABT23771	1	1	0	9	27	0	25
EABT23772	2	2	11	0	0	1	0
EABT23773	3	1	0	0	1	3	1
EABT23774	500.28	703.7	382	1185.09	1023.02	388.06	911.92
EABT23775	0	1	4	5	0	0	2
EABT23776	1	4	9	1	2	0	4
EABT23777	196.02	276.99	120	514.96	315	130	164
EABT23778	0	4	37	3	11	0	15
EABT23779	1	1	7	2	1	0	0
EABT2378	0	2	5	1	0	0	1
EABT23780	3	6	9	9	0	3	2
EABT23781	0	0	2	1	0	0	0
EABT23782	445.99	818.89	663.15	2630.71	955.68	274.09	363
EABT23783	2	2	9	0	0	0	2
EABT23784	1	0	4	0	0	0	0
EABT23785	0	0	9	1	0	0	0
EABT23786	11	18	2	8	16	25	10
EABT23787	3	3	10	7	1	0	0
EABT23788	0	4	3	2	3	1	0
EABT23789	6.04	0	13	18	4	0	0
EABT2379	0	9	4	30	3	0	1
EABT23790	0	0	7	0	0	0	0
EABT23791	0	2	13	0	0	0	0
EABT23792	999.29	1517	2693.41	3962.94	1255	960	951.39
EABT23793	0	3	0	3	0	0	0
EABT23794	314	533	249	1648.97	901.6	122	269
EABT23795	1	0	2	1	3	1	0
EABT23796	0	5	8	9	17	0	0
EABT23797	1	2	14	1	5	5	18
EABT23798	0	2	6	3	1	1	0
EABT23799	0	1	33	3	2	0	0
EABT238	2	3	0	0	0	0	0
EABT2380	6	1	0	0	6	0	48
EABT23800	5	13	1	2	2	0	1
EABT23801	0	0	3	0	3	0	0
EABT23802	0	0	6	2	0	0	0
EABT23803	2707.74	3977.28	3745.76	6864.72	1566.15	2415.28	2448.6
EABT23804	597.94	1061.83	1346.75	37371.1	1259.64	431.09	1194.99
EABT23805	1269	5418	8030.94	17	1386	81	61
EABT23806	0	0	4	2	0	0	0
EABT23807	0	1	10	4	0	0	0
EABT23808	134.7	318	206.41	1591.3	1580.9	93.64	508.5
EABT23809	0	5	15	0	0	0	1
EABT2381	0	0	4	0	0	0	0
EABT23810	0	0	6	0	1	0	5
EABT23811	10668.4	13664.8	21173	4424.13	3948.85	2331.24	2395.53

EABT23812	1671	1043.02	437.83	4468.18	582.99	286	382
EABT23813	10	27	182.92	133.06	108	8	21
EABT23814	0	2	1	2	1	0	0
EABT23815	610149	828692	2234796	781833	186273	115617	146623
EABT23816	0	1	9	0	0	0	0
EABT23817	24	66	23	88	8	8	8
EABT23818	2966.94	4077.28	2418	4576.91	2137.33	2284.04	2054.13
EABT23819	0	1	9	1	0	0	0
EABT2382	0	0	2	0	0	1	1
EABT23820	2305.6	4711.83	3726.6	9613.72	1820.16	2131.98	1628.97
EABT23821	2	4	11	12	0	3	4
EABT23822	122	151	155	1248.01	450.93	96	60
EABT23823	0	4	0	2	3	0	0
EABT23824	2	2	2	7.01	9.02	0	0
EABT23825	18769	11016.6	41.66	8	470.07	20836.3	4804.26
EABT23826	0	3	0	1	0	1	0
EABT23827	0	0	0	0	1	5	10
EABT23828	0	0	3	3	0	0	0
EABT23829	1599.27	2221.51	1483.42	2487.52	1527.62	266.97	665.03
EABT2383	6	13	31	51	8	5	13
EABT23830	15	10	0	5	1	2	1
EABT23831	3	3	4	0	0	0	0
EABT23832	0	2	4	3	0	0	0
EABT23833	1683.25	2006.23	1494.53	2442.82	1330.17	1197.4	1620.24
EABT23834	0	1	1	7	0	0	0
EABT23835	4	2	26	3	0	0	1
EABT23836	5	73	226	1312	33	6	6
EABT23837	10	14	0	0	0	4	1
EABT23838	370.83	763.57	8	0	0	115.88	0
EABT23839	0	2	0	0	0	0	0
EABT2384	1	4	3	1	3	2	0
EABT23840	7	6	0	0	1	4	3
EABT23841	2	5	10	7	3	0	3
EABT23842	1960.83	2873.09	1087	2668.95	1606.62	790.03	1763.99
EABT23843	0	5	25	6	4	1	2
EABT23844	769	1109.03	952	1881	885.78	935	760
EABT23845	188	270	167	316	199	90	147
EABT23846	4160.18	6441.95	7415.52	10131.1	3196.94	3308.31	3381.51
EABT23847	6	14	18	16	18.06	6	14
EABT23848	12	11	24	1	1	4	2
EABT23849	18	34	6	1	2	5	2
EABT2385	0	0	0	7	0	0	0
EABT23850	0	0	2	1	0	0	0
EABT23851	0	0	4	0	0	0	0
EABT23852	1	0	0	3	3	2	1
EABT23853	0	1	7	0	0	0	0
EABT23854	304.47	526.06	486.61	558.38	301.59	213.97	312.96
EABT23855	3	7	1	3	1	1	0
EABT23856	7	3	12	0	0	2	3
EABT23857	121	239.12	385.99	5788.43	484	77	138
EABT23858	3539.27	5411.2	4903.83	6550.98	4272.1	3754.29	3242.81

EABT23859	1	2	0	0	1	0	0
EABT2386	8	8	0	0	0	1	0
EABT23860	5	5	4	3	1	0	1
EABT23861	0	0	10	3	0	0	0
EABT23862	0	0	4	0	0	3	0
EABT23863	1	2	3	0	0	1	0
EABT23864	3	2	0	0	0	0	0
EABT23865	0	0	4	0	0	0	0
EABT23866	1	3	6	3	0	3	3
EABT23867	0	0	2	0	0	0	1
EABT23868	1	1	1	0	0	0	0
EABT23869	0	4	3	0	1	2	0
EABT2387	1	0	2	1	2	0	0
EABT23870	146.95	248	155.02	741.87	372	84	173
EABT23871	14	21	117	20	7	10	21
EABT23872	2785.3	3636.72	8215.36	9642.7	3349.65	2681.54	1803
EABT23873	915	1741.15	2925.91	2847.99	1599.12	579	855
EABT23874	0	2	0	2	2	0	0
EABT23875	1	3	0	1	0	1	0
EABT23876	0	6	0	2	0	0	0
EABT23877	1502.01	1480.76	751.84	1524.81	998.39	1281.36	706.93
EABT23878	1	6	2	3	0	1	6
EABT23879	0	0	4	0	0	0	1
EABT2388	0	0	9	1	4	0	0
EABT23880	551	650	397.07	554	651.13	439.83	412
EABT23881	6074.8	12815	13414.9	27724.2	6251.45	6697.67	5956.87
EABT23882	10	11	21	43	7	3	2
EABT23883	2	2	10	2	0	1	1
EABT23884	2	2	54	39	0	0	1
EABT23885	1	5	1	14	5	0	0
EABT23886	0	0	8	0	0	0	0
EABT23887	0	0	5	1	0	0	1
EABT23888	2	5	13	15.07	1	3	5
EABT23889	2564.98	5588.66	3553.64	10867.6	4372.54	2410.55	3430.12
EABT2389	1	2	3	3	0	2	0
EABT23890	0	0	0	10	0	0	1
EABT23891	2828.03	5028.48	8963.83	3731.56	5476.16	1057	1503.05
EABT23892	0	0	0	0	0	1	5
EABT23893	0	0	15	2	0	1	2
EABT23894	1457.45	2569.44	3023.99	4555.77	1585.99	1894.29	1856.19
EABT23895	380	334	334.07	561.52	276.99	294.01	273
EABT23896	0	1	0	1	0	0	0
EABT23897	2	7	0	3	0	1	1
EABT23898	2	2	28	1	1	2	1
EABT23899	0	3	4	4	2	2	3
EABT239	1	0	7	2	0	0	1
EABT2390	5	4	4	1	3	1	3
EABT23900	0	1	7	1	1	1	2
EABT23901	249.99	311.94	180	1097.78	123	1	66
EABT23902	0	1	24	2	0	1	0
EABT23903	0	1	2	10	0	0	0

EABT23904	2	3	0	2	1	0	0
EABT23905	11	18	65	11	4	6	2
EABT23906	1	4	9	5	0	0	2
EABT23907	10	18	84.1	17	2	6	0
EABT23908	3	3	20	4	0	0	0
EABT23909	16	23	53	56	8	11	9
EABT2391	65	127	304	23	2312.24	7	29
EABT23910	6	0	1	0	0	7	0
EABT23911	18	19	71	26	15	3	16
EABT23912	5	18	86	10	0	3	4.63
EABT23913	0	5	7	36	3	1	1
EABT23914	0	1	15	1	0	1	1
EABT23915	0	0	5	0	0	0	0
EABT23916	13613.4	2149.56	310.47	3539.67	1014.28	11955	1025.61
EABT23917	665	785	605	899.59	572.82	347.01	366
EABT23918	92	33	1	4	2	79	0
EABT23919	26	45	7	4	19	0	40
EABT2392	0	0	0	0	0	0	1
EABT23920	2	23	2	8	0	1	0
EABT23921	30	30	2	1	3	3	5
EABT23922	390.62	411.78	398.61	1052.34	320.07	246	305
EABT23923	0	0	9	0	0	1	0
EABT23924	1	2	2	3	1	1	2
EABT23925	0	0	0	0	0	1	0
EABT23926	0	2	23	0	0	0	0
EABT23927	0	1	6	3	0	0	2
EABT23928	12	10	5	0	3	11	12
EABT23929	0	0	3	0	0	0	1
EABT2393	683.05	1690.72	890.96	1889.04	918	127	1563.55
EABT23930	6	3	1	0	0	8	0
EABT23931	0	4	3	6	1	1	1
EABT23932	1	0	2	6	0	0	1
EABT23933	0	0	9	0	0	1	0
EABT23934	1	0	0	3	2	1	1
EABT23935	340.74	265	843	427	1046	83	753.29
EABT23936	2	4	13	5	0	0	0
EABT23937	28	50	0	0	2	34	23
EABT23938	0	0	30	1	0	0	0
EABT23939	2	6	3	5	6	0	3
EABT2394	4	8	18	24	11	5	2
EABT23940	0	1	1	1	0	0	0
EABT23941	1	3	0	0	2	0	1
EABT23942	0	4	9	1	0	2	0
EABT23943	3074.75	2220.89	1165.37	1725.9	1686.64	1933.94	2020.59
EABT23944	1	0	2	1	0	0	0
EABT23945	1	4	10	1	1	1	2
EABT23946	0	2	3	2	4	0	0
EABT23947	0	2	4	1	0	0	0
EABT23948	0	0	2	1	1	0	0
EABT23949	1594.45	1711.15	348.93	1971.31	5902.64	870.32	994.38
EABT2395	1	1	8	4	6	1	2

EABT23950	0	0	7	0	0	0	0
EABT23951	32	0	0	0	0	17	0
EABT23952	2	2	4	3	1	0	1
EABT23953	3	4	1	0	0	8	4
EABT23954	1	10	1	1	3	1	0
EABT23955	0	0	15	14	17	2	0
EABT23956	13.01	27	85	6	9	8	16
EABT23957	5	12	94	19	2	6	3
EABT23958	0	0	14	0	0	1	0
EABT23959	0	1	20	1	1	0	1
EABT2396	3	4	9	9	1	0	2
EABT23960	1	3	8	0	0	0	0
EABT23961	48	136	104	23	117	0	0
EABT23962	898.75	1296.98	978.02	1567.62	948	942.29	757.24
EABT23963	15	20	19	11	7	0	0
EABT23964	0	1	2	2	0	3	0
EABT23965	0	6	0	0	0	0	1
EABT23966	2	3	6	5	0	0	0
EABT23967	0	0	3	8	0	0	0
EABT23968	1577	2589.89	2501.36	6403.12	3259.95	1959.87	1538.03
EABT23969	18971.7	4312.88	57.88	7	1116.11	10941.3	3950.04
EABT2397	160638	239821	224784	168052	145178	100236	169274
EABT23970	5	7	5	24	1	2	1
EABT23971	45	73	210.19	31	26	129	119.01
EABT23972	1	0	2	0	0	1	1
EABT23973	0	1	9	0	0	2	1
EABT23974	3	4	0	0	2	18	3
EABT23975	5	2	6	8	1	0	0
EABT23976	29.57	42.16	63.43	45.9	21	20.37	18
EABT23977	0	1	2	3	3	0	0
EABT23978	2	1	4	0	0	0	1
EABT23979	1	7	14	13	5	12	11
EABT2398	91.42	166.29	248.66	285.54	210.33	65	99.01
EABT23980	14	82	22	204	1	5	0
EABT23981	0	2	1	7	3	0	8
EABT23982	0	0	11	0	1	0	0
EABT23983	1	0	2	1	0	0	1
EABT23984	2212.17	2613.41	1828.09	1953.4	1944	2304.81	2121.73
EABT23985	2	15	2	7	0	5	1
EABT23986	6	1	17	0	3	38	12
EABT23987	0	2	3	0	1	0	0
EABT23988	1	13	39	20	42	6	11
EABT23989	1	6	1	9	5	2	3
EABT2399	2	2	37	3	1	4	0
EABT23990	13	0	19	1	13	23	2243
EABT23991	0	0	5	3	0	0	0
EABT23992	7	11	0	8	4	0	6
EABT23993	1672.15	2922.71	2356.37	5965.16	3718.56	964.3	1628.09
EABT23994	1	14	9	42.84	26	1	0
EABT23995	9	11	0	2	3	0	0
EABT23996	17	109	120	478	164	2	4

EABT23997	1	12	3	25	2	0	2
EABT23998	2573.71	4965.91	3520.62	8949.47	2447.9	2401.87	2974.1
EABT23999	0	1	6	0	1	0	2
EABT24	81	5	61	53.81	20	5	21
EABT240	0	3	6	0	0	1	0
EABT2400	2	1	1	28.72	0	0	7
EABT24000	9656.8	7355.64	6373.85	7067.17	2121.15	4236.5	3418.72
EABT24001	0	4	1	2	0	1	0
EABT24002	1	0	33	0	0	0	0
EABT24003	0	1	11	0	0	0	0
EABT24004	1785.1	2276.33	1885.34	4032.18	1685.27	1466.96	1655.58
EABT24005	1	4	9	19	0	1	1
EABT24006	0	1	18	2	0	0	0
EABT24007	0	1	3	3	0	0	0
EABT24008	1687.29	2810.74	2907.78	7030.08	1884.99	1491	1440.01
EABT24009	15	65	17	9	9	1	0
EABT2401	3	3	1	0	0	0	0
EABT24010	0	0	5	0	0	0	1
EABT24011	4	7	21	46	19	5	9
EABT24012	0	2	4	6	0	1	0
EABT24013	1	2	2	3	2	0	0
EABT24014	4	22	0	5	0	5	0
EABT24015	2679.49	4961.03	3514.11	8938.29	3389.7	2716.34	4024.83
EABT24016	5	2	9	0	0	1	0
EABT24017	3	5	0	7	1	1	6
EABT24018	1	0	3	0	0	0	0
EABT24019	965.66	1814.62	2716.81	4407.35	1189.96	919.66	1172.51
EABT2402	4	6	32	8	4	2	1
EABT24020	1	1	3	3	0	1	7
EABT24021	0	2	3	1	10	0	0
EABT24022	43	87.02	25	66	24	0	3
EABT24023	1	1	0	1	0	0	1
EABT24024	0	1	0	655.01	4	0	12
EABT24025	0	0	6	0	0	3	0
EABT24026	0	0	13	0	0	0	0
EABT24027	2	2	10	4	1	2	2
EABT24028	3251.43	7210.72	4347.23	6119.05	3497.95	2016.98	3389.6
EABT24029	1	0	7	2	0	0	0
EABT2403	1	6	5	12	4	0	2
EABT24030	71	125.01	91	213	240.01	79	88
EABT24031	0	2	5	3	1	0	0
EABT24032	3005	2401	1114	2668	1186	2758	1791.79
EABT24033	1	3	35	1	1	0	0
EABT24034	1	7	53	6	2	2	2
EABT24035	0	0	7	1	0	0	0
EABT24036	2	0	1	1	76	0	0
EABT24037	0	0	11	1	1	0	0
EABT24038	0	0	6	0	0	0	0
EABT24039	1	7	227	18	1	7	3
EABT2404	1257.31	2063.85	3036.79	4276.36	1532	1342.18	1421.18
EABT24040	1	4	0	0	0	0	0

EABT24041	2	2	11	0	1	0	1
EABT24042	0	3	2	15	7	0	0
EABT24043	2016.67	7732.62	11333.6	7978.98	1679	2178.02	3162.64
EABT24044	2	1	26	2	2	2	1
EABT24045	0	0	2	1	0	0	0
EABT24046	0	2	1	2	2	1	0
EABT24047	0	0	3	3	3	1	1
EABT24048	0	1	2	3	1	0	0
EABT24049	1	1	1	3	0	0	4
EABT2405	10132.5	6218.18	9.06	0	3293.07	18984.3	21077.4
EABT24050	2	7	6	1	1	8	1
EABT24051	0	0	3	2	0	0	0
EABT24052	1	2	21	1	0	1	1
EABT24053	534	1033.77	1408.69	1765.01	598	318	425.01
EABT24054	1	1	2	4	7	0	0
EABT24055	20	63	355.02	1137.35	85	54	91
EABT24056	47	147.27	108	764.53	2	6	1
EABT24057	23	85.76	51	158	19	5	1
EABT24058	630.86	973.91	1008.98	1704.87	835.99	781.97	666.2
EABT24059	25.75	44	34	65.06	9	22	15
EABT2406	0	0	0	0	2	0	27
EABT24060	1	4	1	5	0	1	1
EABT24061	1	6	29	3	7	0	0
EABT24062	0	4	19	1	0	1	0
EABT24063	0	1	2	2	0	0	0
EABT24064	0	0	6	0	0	0	1
EABT24065	0	1	1	4	1	0	0
EABT24066	0	1	2	0	0	0	0
EABT24067	8	3	28	20	4	5	3
EABT24068	0	1	2	0	0	0	0
EABT24069	254.01	420	139	579.97	365	351	229
EABT2407	1	0	4	1	3	1	0
EABT24070	1	0	11	7	0	3	0
EABT24071	0	0	5	1	0	0	0
EABT24072	0	3	4	4	2	0	2
EABT24073	0	0	7	0	0	0	0
EABT24074	18	78	172	167	103	0	1
EABT24075	313.7	426.35	172.01	398.89	408.04	157	324
EABT24076	6	1	2	10	0	8	0
EABT24077	4	2	0	2	2	7	4
EABT24078	4	4	5	1	10	2	14
EABT24079	0	2	4	2	0	0	2
EABT2408	54	88	147	46	39	6	17
EABT24080	2	0	4	0	0	3	0
EABT24081	1	2	4	1	0	2	0
EABT24082	1	6	4	3	0	1	3
EABT24083	1644	1869	434	164	762	2838.97	4343.84
EABT24084	204	438	238.03	1170.09	348	116	162.75
EABT24085	2	0	0	7	1	0	0
EABT24086	2	1	6	17	30	0	1
EABT24087	898.79	1066.91	471.7	1092.44	697.44	654.97	632.51

EABT24088	16	28	84	16.06	5	2	0
EABT24089	0	0	1	6	0	1	0
EABT2409	2	4	4	0	0	1	0
EABT24090	0	3	5	0	0	4	0
EABT24091	4	5	21	7	4	2	2
EABT24092	3	9	12	3	1	1	8
EABT24093	4	3	21	3	2	2	6
EABT24094	1	1	4	5	2	0	1
EABT24095	0	7	0	11	0	3	3
EABT24096	0	2	8	12	0	2	1
EABT24097	6791.42	4833.46	4222.44	7446.89	2099.74	563.09	705.86
EABT24098	0	0	13	1	0	0	0
EABT24099	1	4	4	6	0	3	3
EABT241	2	10	22	2	0	0	0
EABT2410	12	10	5	6	57	7	102
EABT24100	1	3	1	0	0	2	1
EABT24101	0	6	6	7	0	0	0
EABT24102	1380.69	2047.44	770	3002.47	985.83	759.83	780.29
EABT24103	16	3	0	4	1	0	0
EABT24104	5	11	11	0	0	3	0
EABT24105	1269.41	2383.59	3260.77	5379.15	1504.34	2083.47	2006.41
EABT24106	5	8	10	2	1	1	1
EABT24107	966.15	1518.67	1798.7	3610.01	1886.34	1033.38	1227.34
EABT24108	0	1	6	0	0	0	0
EABT24109	5	10	9	28	1	3	1
EABT2411	102.87	134	58	11	32	576.94	18
EABT24110	0	3	12	7	1	1	0
EABT24111	0	0	2	4	0	0	1
EABT24112	0	1	18	3	0	0	0
EABT24113	319.38	638.67	231	714.13	346.1	196.01	225.34
EABT24114	0	0	9	0	0	0	0
EABT24115	0	1	5	0	0	0	0
EABT24116	5	4	4	6	4	2	2
EABT24117	0	3	0	1	0	0	1
EABT24118	0	0	30	44	19.98	9	3
EABT24119	2	12	32	40	32	2	11
EABT2412	7773.66	15290	4039.81	2917.7	4610.21	32	574.74
EABT24120	3715.92	5458.77	4830.81	4909.6	2216.41	2142.86	2148
EABT24121	1	0	3	0	1	0	2
EABT24122	1	3	5	0	0	0	0
EABT24123	1	2	2	8	3	0	1
EABT24124	3533.07	6624.62	14856.4	5884.1	3290.74	3107.31	3894.36
EABT24125	40	160	161.01	155	734	21	76
EABT24126	0	5	2	1	1	0	4
EABT24127	0	2	3	2	0	0	0
EABT24128	0	0	0	0	1400.86	1	6
EABT24129	45	55	234.11	122	27	39	40
EABT2413	1	4	2	0	1	0	0
EABT24130	5	11	28	29	4	7	5
EABT24131	0	0	5	1	0	0	0
EABT24132	2013.63	3639.88	3023.46	1004	7489.2	3918.26	13135

EABT24133	0	1	6	9	0	1	0
EABT24134	0	0	4	1	0	0	0
EABT24135	1251.66	2158.18	1120.18	1618.31	905.72	2514.23	2003.5
EABT24136	1	0	7	0	0	0	2
EABT24137	17	18	187	13	12	42	9
EABT24138	0	5	11	12	0	0	0
EABT24139	0	2	6	2	2	1	0
EABT2414	0	0	7	1	0	0	1
EABT24140	69	82	125	696.01	462.38	65	16
EABT24141	15	14	35	45	19	6	18
EABT24142	22	49	0	2	2	43	9
EABT24143	16	26	35.27	81.2	25	27	18
EABT24144	0	0	13	0	0	0	0
EABT24145	1	1	1	0	0	0	2
EABT24146	0	3	1	4	0	1	1
EABT24147	0	4	1	0	0	3	1
EABT24148	3	3	3	1	0	7	2
EABT24149	0	1	3	0	1	0	0
EABT2415	0	0	3	1	0	0	0
EABT24150	774	1098.64	884.2	1998.61	928.61	647.87	869.54
EABT24151	1	1	29	4	0	0	0
EABT24152	857	895.74	767	846.73	582	761	660
EABT24153	0	4	30	6	2	0	1
EABT24154	3	0	2	7	0	0	0
EABT24155	0	3	7	0	2	0	1
EABT24156	1	2	1	3	0	0	0
EABT24157	214	417.75	414.95	1196.01	252	99.42	139
EABT24158	1	6	6	10	4	1	0
EABT24159	656.46	1901.14	679.05	1743.47	325.38	482.88	324.45
EABT2416	1	1	1	0	0	0	9
EABT24160	3	0	15	2	0	4	0
EABT24161	0	0	15	0	0	0	0
EABT24162	0	1	13	0	0	0	0
EABT24163	2	2	0	0	1	0	0
EABT24164	0	0	0	0	2	0	2
EABT24165	4	4	2	4	1	0	2
EABT24166	3	0	0	0	24	1	0
EABT24167	0	0	1	0	0	0	0
EABT24168	51	92	37.98	28	50	11	16
EABT24169	5	7	0	0	0	1.78	2
EABT2417	2	5	2	0	1	1	2
EABT24170	3	7	0	0	1	0	0
EABT24171	31	24	70	26	4	12	2
EABT24172	0	2	4	3	0	0	0
EABT24173	0	1	10	0	0	0	0
EABT24174	0	0	5	0	0	0	0
EABT24175	7	35	21	323.25	10.98	3	13
EABT24176	2	4	13	17	16	8	25
EABT24177	3	8	64.03	37.03	2	0	1
EABT24178	12	69	18	102	3155.53	0	49
EABT24179	4	7	0	1	1	2	2

EABT2418	0	2	2	0	11	1	10
EABT24180	0	1	9	8	0	0	0
EABT24181	0	1	0	2	0	2	0
EABT24182	648.92	1294.47	604.03	2451.71	655	505.72	459
EABT24183	2	4	1	0	2	2	2
EABT24184	2	7	81	11	4	8	6
EABT24185	0	0	8	2	0	0	0
EABT24186	0	0	1	1	1	1	0
EABT24187	0	0	4	0	0	0	0
EABT24188	0	2	1	1	2	0	1
EABT24189	0	5	7	13	5	1	1
EABT2419	0	5	8	2	3	1	1
EABT24190	5060.82	1604.04	101	10.32	1217.05	2911.85	5208.1
EABT24191	198.99	738.04	1895.96	925	501.9	137.99	131
EABT24192	0	0	7	0	0	1	0
EABT24193	0	2	0	4	0	0	0
EABT24194	3	19	36	105	64	7	12
EABT24195	865	1246	883	731	559.52	520	627.14
EABT24196	0	1	2	3	0	1	0
EABT24197	2848.01	5403.88	5974	2201.27	2721	2088.99	1573.12
EABT24198	3	4	9	8	0	0	0
EABT24199	0	3	34	0	2	0	0
EABT242	0	0	2	19	0	2	0
EABT2420	0	1	9	0	0	0	0
EABT24200	19	21	56	39	19	4	3
EABT24201	10	1	1	1	9	3	116
EABT24202	0	4	0	2	0	0	0
EABT24203	142.55	282.18	275	963.96	181	65	33
EABT24204	1	6	5	6	0	1	0
EABT24205	0	0	3	11	0	0	0
EABT24206	1581.4	3259.82	3866.87	4635.51	3826.83	1053.36	2617.75
EABT24207	0	0	6	1	0	0	0
EABT24208	0	1	2	1	3	0	0
EABT24209	0	2	4	0	1	0	0
EABT2421	23	41	293	122	23	22	66.9
EABT24210	17	21.93	48	4	0	4	1
EABT24211	372.92	991	1869.6	57253.9	87	44	2
EABT24212	1	2	17	4	1	0	0
EABT24213	142	276	348	2699.64	441.21	44	29
EABT24214	1	1	0	1	0	0	0
EABT24215	0	0	5	5	1	0	0
EABT24216	111	126	518	278	28	12	28
EABT24217	0	1	6	0	0	1	0
EABT24218	7.99	9	12	57	34	2	15
EABT24219	15	3	31	1	2	0	0
EABT2422	169	385	8	5	601	1	7
EABT24220	5670.23	7930.1	4637.3	12663.2	1799.23	12	218
EABT24221	9	20	34	29	8	9	21
EABT24222	0	2	6	5	0	0	0
EABT24223	1	4	11	4	1	2	0
EABT24224	3	11.99	4	4	3	5	33

EABT24225	3579.07	4976.56	4036.33	1911.15	773.04	909.04	1330.36
EABT24226	2	4	6	16	13	0	1.97
EABT24227	1	0	3	3	0	0	0
EABT24228	1	0	4	0	0	0	0
EABT24229	1	0	20	0	0	0	0
EABT2423	11	16	0	0	1	18	14
EABT24230	27	21	8	7	15	0	4
EABT24231	0	1	8	7	0	1	0
EABT24232	0	0	9	0	0	0	0
EABT24233	0	5	9	6	3	0	0
EABT24234	1	6	0	0	0	0	0
EABT24235	15	38.09	29	176	43	3	5
EABT24236	0	1	1	0	1	0	0
EABT24237	0	5	1	0	0	0	0
EABT24238	0	3	0	3	4	0	2
EABT24239	4	20	10	6	0	3	0
EABT2424	2215.8	2426	1691.26	4876.98	1242	1301.01	1291
EABT24240	2	3	8	0	0	0	0
EABT24241	0	0	2	5	5	0	0
EABT24242	0	0	5	3	0	0	0
EABT24243	132	463	33	39	20	359	6
EABT24244	0	1	0	0	18	1	54
EABT24245	2	7	13	26	48.03	0	0
EABT24246	0	0	52	2	0	0	0
EABT24247	1	3	251	3	2	0	1
EABT24248	523	909.75	6595.45	168	892.8	3	55
EABT24249	243	2635.21	3939.44	350	1667.36	75	1447.45
EABT2425	10	0	2	0	5	1	12
EABT24250	0	2	14	3	0	0	0
EABT24251	2680	3219	2631.83	3792.46	2082	2294	1393
EABT24252	0	1	1	0	5	0	0
EABT24253	359.99	1015.92	636.51	1898	2388.6	394	721
EABT24254	0	2	4	2	1	0	1
EABT24255	8	15	0	0	4	0	0
EABT24256	1656	2589.6	2312.21	3097.14	1709.78	1230.51	1281.42
EABT24257	3	3	12	0	0	1	0
EABT24258	158.2	249.7	499.45	327.47	257	150.11	841.79
EABT24259	0	1	7	2	0	0	0
EABT2426	4	1	13	11	4	0	5
EABT24260	0	1	2	3	0	0	1
EABT24261	0	0	36	0	1	0	0
EABT24262	1	5	4	19	1	0	0
EABT24263	3	5	33	4	0	2	1
EABT24264	4833.7	7247.09	4094.27	3679.26	7987.28	4958.4	4862.93
EABT24265	3048	3737.32	2261	4548.9	2288.88	3658.8	2164
EABT24266	2850.73	1683.94	51	67	267	267	574
EABT24267	0	2	18	1	0	0	0
EABT24268	1	0	9	0	1	0	0
EABT24269	678	1156	628	615	745	650	1177
EABT2427	2562.44	4363.11	3044.23	6370.36	2611.24	2322.2	2815.93
EABT24270	0	4	0	2	0	0	0

EABT24271	10	14	108	14	3	4	6
EABT24272	0	1	7	6	0	0	1
EABT24273	23	12	9	9	6	1	6
EABT24274	0	0	6	0	0	0	0
EABT24275	0	0	0	1	3	1	4
EABT24276	3	8	17	5	4	5	3
EABT24277	285	319	157	372	272	244	148
EABT24278	0	2	8	0	0	0	0
EABT24279	0	0	3	8	1	0	0
EABT2428	0	1	2	0	0	0	0
EABT24280	0	3	2	3	0	0	0
EABT24281	0	0	1	5	14	12	69
EABT24282	270	427.29	682.97	740.03	316.27	268.98	203.73
EABT24283	0	1	3	0	0	0	0
EABT24284	3	3	5	1	0	0	0
EABT24285	10	21	29	41	8	7	17
EABT24286	0	0	8	0	1	0	0
EABT24287	2	6	7	18	3	0	4
EABT24288	3	4	0	0	0	0	0
EABT24289	559	756.76	762.94	5340.11	1526.24	43	126
EABT2429	3189.05	3816.74	4693	4560.83	2423.8	2705	2069
EABT24290	3	3	14	0	0	0	0
EABT24291	0	0	1	0	0	0	0
EABT24292	0	0	0	10	2	0	0
EABT24293	0	4	31	3	0	1	0
EABT24294	2	9	42	4	0	3	0
EABT24295	8	3	4	1	1	4	5
EABT24296	1338.87	2551	56	31	1805	2032.03	9405.59
EABT24297	0	0	8	0	0	0	0
EABT24298	0	0	18	6	0	0	0
EABT24299	1	0	5	0	0	0	0
EABT243	1	9	28	23	8	1	1
EABT2430	2	2	10	11	2	2	1
EABT24300	1	2	11	7	0	2	0
EABT24301	6567.58	12876.2	8308.19	1288.07	11623.6	346.44	2855.46
EABT24302	13	2	0	1	0	0	0
EABT24303	386	793	1596.99	1373	520	360	287
EABT24304	0	5	11	8	1	0	0
EABT24305	1	1	2	1	0	1	1
EABT24306	4	49	45	220	101	0	0
EABT24307	59	110	44	198	57	22	30
EABT24308	0	0	2	5	2	1	1
EABT24309	0	1	3	0	2	1	2
EABT2431	0	0	4	6	3	0	1
EABT24310	207	810	1052.75	2440	565	230	166
EABT24311	1	10	3	6	1	0	1
EABT24312	0	0	2	5	1	1	0
EABT24313	0	1	4	0	1	0	0
EABT24314	0	2	3	5	0	5	0
EABT24315	13375.2	13104.1	17678.5	15651.9	10390.6	13211.7	15084.9
EABT24316	0	1	3	5	0	0	0

EABT24317	4	9	5	4	4	1	3
EABT24318	2	9	1	0	0	10	1
EABT24319	0	2	1	0	0	4	2
EABT2432	303.05	47	1	0	0	104.01	1
EABT24320	1	0	4	0	0	1	0
EABT24321	0	0	2	0	8	0	14
EABT24322	4	8	1	1	3	6	13
EABT24323	2809.86	4156.42	5660.57	6296.7	2379.22	2176.64	2102.73
EABT24324	1	0	34.3	0	0	1	0
EABT24325	11542.3	29847.1	9444.07	3815.03	13752.7	13113.5	3747.44
EABT24326	5705.84	10764.2	9159.69	12046.2	4389.61	6334.56	5852.93
EABT24327	7988.24	8148.59	7028.99	1161.34	5082.46	7824.1	6424.06
EABT24328	0	0	7.9	0	0	0	1
EABT24329	3	12	386	3	0	8	2
EABT2433	3	1	16	3	0	0	0
EABT24330	0	6	21	3	0	1	1
EABT24331	125.03	328	1351	1000	501.99	115	304
EABT24332	705.4	587.61	589	741.62	211	449.13	883.62
EABT24333	19	11	30	0	5	1	1
EABT24334	7	5	9	4	5	0	4
EABT24335	495.98	1027.04	1007.97	2571.29	1561.86	345	400.99
EABT24336	11	40	127.96	63	25	28	40
EABT24337	30	61.99	19	28	175.35	53	101.96
EABT24338	0	1	2	1	1	0	0
EABT24339	0	3	67	5	0	2	1
EABT2434	0	0	4	0	0	0	0
EABT24340	47991.3	31596.9	7963.37	9161.25	24631	62556.7	63665.1
EABT24341	15	22	70	12	6	10	22
EABT24342	0	4	5	0	0	1	0
EABT24343	0	0	6	18	0	2	3
EABT24344	5	11	45	3	1	0	1
EABT24345	207.97	360	341	1408	369	132	200
EABT24346	4	1	0	0	0	2	0
EABT24347	0	1	1	17	1	0	0
EABT24348	663.85	968.39	1022.49	1676.97	555	542.58	594.07
EABT24349	5740.13	7366.98	5794.26	14183.1	6139.55	5395.63	3644
EABT2435	0	1	6	1	1	0	0
EABT24350	4	3	0	19	6	4	1
EABT24351	4	2	0	1	2	1	0
EABT24352	0	4	1	0	2	7	5
EABT24353	1	0	68	4	0	0	0
EABT24354	0	0	6	2	0	0	0
EABT24355	829.35	1396.31	1316.01	3009.22	916.98	736.93	862.7
EABT24356	0	0	4	0	1	0	0
EABT24357	0	0	6	3	2	0	1
EABT24358	10	17	9	49	79	15	37
EABT24359	7	31	84	36	28	1	8
EABT2436	14	12	0	0	1	40	21
EABT24360	1	2	11	3	1	0	0
EABT24361	2	2	18	3	5	3	1
EABT24362	7	13	20	13	1	0	2

EABT24363	0	2	0	1	0	1	1
EABT24364	0	1	6	0	0	0	0
EABT24365	2	8	12	9	1	0	0
EABT24366	0	0	1	0	0	1	0
EABT24367	1	0	12	0	2	0	0
EABT24368	0	2	2	1	0	0	0
EABT24369	3	13	38	42	4	2	4
EABT2437	0	2	1	2	1	0	0
EABT24370	2	14	22	51	509.6	2	7
EABT24371	7	3	0	17	0	0	0
EABT24372	637.76	907.18	428.8	2587.53	2128.2	426.31	1111.63
EABT24373	3	0	0	0	0	2	4
EABT24374	20	21	0	0	2	14	13
EABT24375	3	6	0	0	2	14	16
EABT24376	396.52	755.04	338	405.54	778.59	80	201.99
EABT24377	316	508	568	798.01	345.99	262	348
EABT24378	0	0	0	16	0	0	0
EABT24379	3	1	3	0	1	0	0
EABT2438	0	7	59	9	2	0	0
EABT24380	4	5	35	6	1	0	0
EABT24381	3	1	29	35	7	2	0
EABT24382	0	2	1	1	2	0	0
EABT24383	1	1	4	1	0	4	2
EABT24384	0	1	1	3	0	0	0
EABT24385	2	1	13	0	0	0	0
EABT24386	2475	6811	22707.4	3281.99	9288.94	1745.1	2133.06
EABT24387	62	113.03	266	6348.3	167	2	58
EABT24388	0	0	1	0	0	2	3
EABT24389	5012.22	6465.84	11091.8	12775.9	4953.98	4542.31	2824.88
EABT2439	0	1	3	1	0	0	1
EABT24390	0	1	0	0	0	0	0
EABT24391	1	1	3	2	0	1	0
EABT24392	0	0	0	0	4	0	7
EABT24393	0	4	2	5	0	0	1
EABT24394	698.14	923.19	188	668	928.1	555	544.04
EABT24395	0	1	5	0	0	0	0
EABT24396	2	3	5	7	0	0	0
EABT24397	1	11	3	109.63	3	1	0
EABT24398	0	1	8	0	1	0	0
EABT24399	0	1	13	1	1	0	0
EABT244	0	0	63	1	0	0	2
EABT2440	0	0	5	0	1	0	0
EABT24400	1	0	5	0	0	0	0
EABT24401	5	4	11	9	2	10	6
EABT24402	0	1	7	2	0	0	0
EABT24403	31	132	33	22	4	185	19
EABT24404	0	3	4	1	3	0	0
EABT24405	7	1	0	0	0	2	0
EABT24406	580	632.2	908.34	824.03	432.69	585.92	411.3
EABT24407	0	1	6	1	0	0	0
EABT24408	0	1	6	1	0	0	0

EABT24409	0	0	1	1	0	0	1
EABT2441	2682.53	3399.66	781.06	1707.11	1354	2194.06	1662.03
EABT24410	5379.79	7792.71	15312	3526.12	5194.04	2898.17	3541.46
EABT24411	494.17	736.06	631.61	1709.23	466.26	176.91	365.03
EABT24412	0	2	7	1	0	0	1
EABT24413	0	0	17	0	0	1	0
EABT24414	0	0	26	0	0	1	0
EABT24415	1	1	5	1	2	0	0
EABT24416	0	0	7	10	0	0	0
EABT24417	39	22	19	9	16	72	67
EABT24418	8	4	13	5	18	2	25
EABT24419	0	9	8	4	5	0	3
EABT2442	0	0	7	2	0	1	0
EABT24420	1269.25	2207.72	1484.78	3494.51	1072.8	1345.56	1141.08
EABT24421	71763.7	72845.9	47814.4	48779.1	34732.4	1240.91	2979.72
EABT24422	11	20	0	0	0	19	0
EABT24423	5	12	0	0	0	21	15
EABT24424	5	3	6	0	1	0	1
EABT24425	641.01	1095	1598.87	1009.65	1151.93	285	1509.95
EABT24426	2	4	8	16	2	3	3
EABT24427	0	1	5	1	0	0	0
EABT24428	0	1	1	17	0	0	0
EABT24429	0	3	3	2	1	0	1
EABT2443	1	3	8	2.99	0	1	1
EABT24430	1	4.01	14	8	3	0	0
EABT24431	4	7	11	7	2	0	5
EABT24432	5	3	0	0	1	0	0
EABT24433	9	2	5	12	1	2	1
EABT24434	4	3.07	6	7	0	2	6
EABT24435	1658.99	2338	1320	2955	1810	1923.01	1824.99
EABT24436	0	0	7	1	0	0	0
EABT24437	0	1	0	3	0	0	0
EABT24438	108.03	234	172.95	1612.01	287.15	55	72
EABT24439	0	2	2	2	0	1	0
EABT2444	0	1	66	2	9	0	1
EABT24440	3	2	25	4	2	1	3
EABT24441	2	7	1	13	2	7	4
EABT24442	0	0	1	0	7	0	23
EABT24443	3	2	0	0	0	2	3
EABT24444	1616	2704	2380.1	6129.2	2149.48	1327	1867.94
EABT24445	2	0	28	0	0	2	0
EABT24446	1	0	28	26	3	1	4
EABT24447	4	10	85	6	2	2	3
EABT24448	143	472	697.01	1384.9	192	76	71
EABT24449	1	3	27	39	1	1	0
EABT2445	4	1	2	8	23	0	0
EABT24450	1455.87	2247.98	1645.78	2045.99	1275.33	1111.1	1072
EABT24451	2	1	17	5	1	0	0
EABT24452	0	4	4	0	10	5	35
EABT24453	8	33	24.03	39	26	6	15
EABT24454	1	0	5	0	0	3	0

EABT24455	1	5.99	13	12	6	1	2
EABT24456	941.14	1183.02	1006	2198.28	660	802.02	712.59
EABT24457	1708	1313	433	1344.4	863	635.81	1248
EABT24458	2931.32	3377.95	3502.66	4025.17	2227.99	2589.82	1629.8
EABT24459	5425.4	9892.93	7572.49	11023.3	8859.94	3837.6	7696.99
EABT2446	0	0	6	1	0	0	0
EABT24460	11	0	0	0	0	3	0
EABT24461	0	0	5	3	9	0	0
EABT24462	4.03	8	3069.3	110	522.68	5	104
EABT24463	0	0	11	1	0	0	0
EABT24464	0	1	4	0	0	0	0
EABT24465	0	0	2	0	7	0	0
EABT24466	1	5	5	6	0	0	1
EABT24467	3	13	107	27	119.97	0	0
EABT24468	0	2	7	9	2	1	0
EABT24469	3	5	5	30	6	1	5
EABT2447	6	21	35	39	13	27	18
EABT24470	0	0	11	8	3	1	0
EABT24471	0	0	9	1	0	0	0
EABT24472	0	1	1	23	45	2	0
EABT24473	13	24	62	15	9	7	5
EABT24474	0	1	5	1	0	1	0
EABT24475	1	7	5	9	1	0	1
EABT24476	0	0	6	1	0	0	0
EABT24477	0	0	0	0	0	1	6
EABT24478	17	9	25	1	8	46	21
EABT24479	1	1	1	1	1	1	3
EABT2448	29	10	6	4	5	0	0
EABT24480	1	2	16	1	14	2	13
EABT24481	1	4	8	3	3	7	1
EABT24482	0	0	12	0	0	1	0
EABT24483	699	794	1178	1498	518	398	378
EABT24484	0	1	57	1	0	0	0
EABT24485	0	0	7	1	0	0	0
EABT24486	11	11	47	17	11	7	12
EABT24487	3	1	54	16	0	5	2
EABT24488	1	0	10	0	0	0	0
EABT24489	187	272	61	1	96	266.01	658
EABT2449	0	0	10	4	0	1	1
EABT24490	0	1	5	1	1	0	0
EABT24491	13	50	441	50	22	35	24
EABT24492	1	6	10	17	3	0	16
EABT24493	1	4	13	3	1	3	0
EABT24494	0	0	9	0	0	0	0
EABT24495	12	8	27	4	8	0	0
EABT24496	0	1	4	0	2	0	0
EABT24497	6506.95	11377	5285.14	7041.94	5458.09	38	472
EABT24498	1	3	6	2	0	0	0
EABT24499	0	2	8	2	0	0	1
EABT245	2575.57	4068.63	3047.74	4787.02	3309.38	2405.83	2438.39
EABT2450	2	2	20	11	0	0	0

EABT24500	0	1	6	1	7	0	2
EABT24501	1	1	0	6	0	0	0
EABT24502	836.69	1256.39	1994.41	1649	1327.91	805.85	1019.38
EABT24503	0	1	3	1	7	0	5
EABT24504	0	2	5	1	2	1	0
EABT24505	0	0	5	0	0	0	0
EABT24506	35	27	4	1	0	14	1
EABT24507	331.58	511.64	647.58	1227.04	413.72	245.06	340.13
EABT24508	1	1	2	47	3	1	2
EABT24509	0	4	1	0	1	1	2
EABT2451	6	10	16	16	2	0	2
EABT24510	2157.23	1452	4054.01	716	1908	2143.99	1212
EABT24511	2	3	10	10	1	0	4
EABT24512	0	0	14	0	0	0	0
EABT24513	0	1	13	0	1	0	0
EABT24514	310	134	56	22	10	141	19
EABT24515	1	1	5	7	0	2	0
EABT24516	0	1	6	0	0	0	0
EABT24517	0	1	23	1	0	1	1
EABT24518	0	0	32	0	1	0	0
EABT24519	1	8	93	24	9	1	71
EABT2452	0	2	3	6	10	0	0
EABT24520	0	0	17	1	0	0	1
EABT24521	0	0	1	0	5	0	3
EABT24522	1	1	1	0	1	1	0
EABT24523	0	0	0	2	1	0	4
EABT24524	5	17	95.46	51	5	29	7
EABT24525	0	1	19	1	0	0	0
EABT24526	7	14	16	23	883.53	7	5
EABT24527	4	3	31	1	0	4	1
EABT24528	6	13	40	4	4	9	6
EABT24529	4	2	29	8	2	0	0
EABT2453	5	8	4	29	4	0	3
EABT24530	15	49	111	303	50	25	27
EABT24531	1	2	7	3	3	0	1
EABT24532	1	4	0	1	0	0	2
EABT24533	0	0	4	0	0	0	2
EABT24534	5	8	25	2	2	1	6
EABT24535	3	3	17	5	1	1	2
EABT24536	3287.46	4088.51	3301.96	1711	1084	2471.14	2221.25
EABT24537	1	1	0	0	0	0	0
EABT24538	601.79	761.19	387.71	546.63	735.56	514.45	418.65
EABT24539	3	3	10	8	3	0	0
EABT2454	9.58	41	16.01	100	61	0	41.95
EABT24540	0	0	1	0	0	0	0
EABT24541	0	0	9	0	0	0	0
EABT24542	1	0	1	27	2	1	4
EABT24543	510.34	784.6	689.7	1698.81	762.24	460.1	587.41
EABT24544	0	0	15	0	0	0	0
EABT24545	0	0	5	0	0	0	0
EABT24546	0	1	0	3	1	0	0

EABT24547	0	2	1	2	1	0	0
EABT24548	7	23	8	2	5	11	29
EABT24549	6	59.08	365.36	89.01	14	20	17.75
EABT2455	2113.87	1697.6	2234	408.94	3185.12	57	379.07
EABT24550	2	0	15	0	0	0	0
EABT24551	0	3.94	2	10	4	0	4
EABT24552	1	2	7	2	0	1	2
EABT24553	7690.51	15019.8	9797.2	10972.7	3659.06	4509.53	4443.03
EABT24554	11	6	7	10	3	3	2
EABT24555	4839.55	7039.67	2292.96	200	1941.12	9962.11	7303.46
EABT24556	0	0	0	0	4	0	14
EABT24557	1	6	1	1	6	0	0
EABT24558	1497.8	2615.51	1090.6	4129.09	1031.29	904.76	879.95
EABT24559	6	11	6	9	1	3	2
EABT2456	16	31	30.99	8	22	2	0
EABT24560	88	169	163.6	1368.41	110.07	65	58
EABT24561	1	1	2	4	2	2	0
EABT24562	0	2	5	1	0	0	0
EABT24563	3	9	17	17	6	6	8
EABT24564	961.04	1960.9	3819.94	6630.37	2253.03	1280.13	1295.27
EABT24565	0	0	2	0	6	0	0
EABT24566	12	7	3	3	0	10	4
EABT24567	0	0	4	0	0	1	1
EABT24568	1	0	1	3	0	2	0
EABT24569	44	108	78	1408	377	1	19
EABT2457	1	4	6	0	2	2	2
EABT24570	0	2	13	4	0	0	2
EABT24571	0	0	20	1	1	0	0
EABT24572	0	0	5	0	0	0	0
EABT24573	0	1	16	0	5	0	0
EABT24574	3	5	0	10	3	1	0
EABT24575	3	2	2	3	0	0	0
EABT24576	30	23	107	32	11818.3	12	69
EABT24577	34	46.17	9	1	31	0	68
EABT24578	0	0	3	0	1	0	0
EABT24579	16	38	17	28	9	11	7
EABT2458	1	10	9	3	2	1	2
EABT24580	0	7	22	1	2	0	1
EABT24581	99	153	8	27	50	45	132.99
EABT24582	13	10	15	64	3	0	1
EABT24583	2	9	18	86	11	1	0
EABT24584	1901.43	3351.44	2762.82	9244.04	2511.81	1701.45	2135.17
EABT24585	0	1	8	0	0	0	0
EABT24586	0	2	1	7	4	0	0
EABT24587	1395.28	2469.4	5533.86	3292.91	1497.07	1160	805
EABT24588	1	0	4	0	0	0	0
EABT24589	0	2	4	13	1	0	2
EABT2459	3	1	1	0	0	0	0
EABT24590	1131.97	1428.31	1925.15	1441	1272	640.92	889
EABT24591	503	693	240	155	350.02	1211.58	1463.96
EABT24592	773.03	861	816	1280	639	580	486

EABT24593	0	0	3	0	57	0	0
EABT24594	0	0	7	0	0	2	0
EABT24595	5	8	6	28	15	1	1
EABT24596	0	0	7	1	0	0	0
EABT24597	6	1	4	0	0	1	0
EABT24598	1	3	6	0	0	0	0
EABT24599	129	174.01	98	424.03	152	92	101
EABT246	3	17.83	14	19	5	15	7
EABT2460	0	0	18	1	0	0	0
EABT24600	0	4	12	2	1	0	0
EABT24601	0	5	12	3	0	0	0
EABT24602	1	0	18	5	0	0	0
EABT24603	0	0	2	3	0	0	0
EABT24604	1	5	2	4	1	4	1
EABT24605	0	2	5	0	0	0	0
EABT24606	14	2	1	2	0	0	0
EABT24607	2	1	0	0	0	14	0
EABT24608	3	2	41	0	0	0	1
EABT24609	0	2	5	0	0	0	0
EABT2461	10	2	19	7	2	3	1
EABT24610	0	1	22	9	2	0	0
EABT24611	1	6	21	14	11	0	5
EABT24612	0	2	4	4	0	0	0
EABT24613	0	1	10	0	0	1	0
EABT24614	0	0	0	0	1	1	3
EABT24615	2	2	10	6	0	3	2
EABT24616	13	79	175	30	24	52.02	85.97
EABT24617	1	0	3	0	0	0	0
EABT24618	1	1	1	0	0	0	0
EABT24619	6537.16	12881.9	5332.33	20583.5	7617.58	3802.87	5474.99
EABT2462	1	35	12	32	16	11	2
EABT24620	4	22	13	1	0	2	1
EABT24621	2	9	7	18	10	1	0
EABT24622	0	1	9	9	0	2	3
EABT24623	1	2	3	0	1	0	0
EABT24624	1	0	0	3	0	0	3
EABT24625	1	3	6	0	2	0	3
EABT24626	11	34	43	724	42	2	19
EABT24627	0	1	1	2	2	1	1
EABT24628	1	3	7	10	1	2	0
EABT24629	2	1	10	3	0	0	1
EABT2463	16	16	11	52	7	4	4
EABT24630	5	6	2	5	5	0	4
EABT24631	3	6	2	0	2	0	0
EABT24632	61	34	16	54	14	129	111
EABT24633	0	2	0	1	0	11	9
EABT24634	2	2	4	0	1	0	2
EABT24635	0	1	1	3	0	0	0
EABT24636	5	3	67	45	12	1	62
EABT24637	4	2	12	2	1	1	4
EABT24638	0	1	0	7	1	0	0

EABT24639	26	83.98	49	30	3	0	3
EABT2464	0	0	9	1	0	0	0
EABT24640	2	8	1	7	2	4	2
EABT24641	0	4	3	1	2	1	4
EABT24642	388	738	289.62	442	304	121	300
EABT24643	0	3	5	2	0	0	0
EABT24644	0	0	0	0	0	0	0
EABT24645	0	0	0	0	0	0	0
EABT24646	1	6	1	3	0	0	0
EABT24647	15037.2	27853.8	13848.6	7485.33	4728.36	7100.01	5737.92
EABT24648	601.09	68.01	252.64	852.65	117.6	1553.52	61.52
EABT24649	0	0	15	1	0	0	1
EABT2465	2	0	23	0	0	0	1
EABT24650	0	1	8	0	0	0	0
EABT24651	0	2	5	1	0	0	0
EABT24652	544	679	188	212	247	651.94	327
EABT24653	2311.07	3622	2270	2525	2861.99	3562.03	3401.01
EABT24654	0	1	1	8	0	0	0
EABT24655	5	5	10	4	1	0	1
EABT24656	1	5	18	0	0	0	1
EABT24657	3	8	38	4	2	0	0
EABT24658	78	149.31	243	254.93	116.41	63	106.55
EABT24659	0	1	2	2	0	0	0
EABT2466	0	5	1	1	1	1	0
EABT24660	0	0	8	0	0	0	0
EABT24661	0	3	47	2	0	0	7
EABT24662	1	5	18	2	2	2	2
EABT24663	0	2	1	1	0	0	0
EABT24664	16	40.99	96.03	299.54	6	1	0
EABT24665	0	1	21	0	0	1	0
EABT24666	13	12	20	17	4	3	7
EABT24667	0	0	3	1	3	0	0
EABT24668	0	1	36	2	1	1	2
EABT24669	1	1	1	3	0	6	2
EABT2467	1	2	16	2	1	0	1
EABT24670	12	3	48	30	11	4	1
EABT24671	0	0	9	1	0	0	0
EABT24672	0	0	0	0	0	0	1
EABT24673	4	3	1	1	0	2	0
EABT24674	4	12	11	7	12	3	10
EABT24675	8	40	8	72	32	0	7
EABT24676	8	8	30	5	1	0	2
EABT24677	9	54	1	3	16	1	3
EABT24678	2	9	7	2	3.01	1	3
EABT24679	195.49	296.83	230	557.9	195	135.19	206.96
EABT2468	2	0	0	0	13	1	74
EABT24680	1271.68	1831.71	1262.96	8290.74	1247.23	3594.19	4735.29
EABT24681	0	0	9	1	0	0	0
EABT24682	6	14	43	5	2	11	4
EABT24683	1	10	15	1	3	0	1
EABT24684	0	5	14	4	2	0	0

EABT24685	0	0	11	0	0	0	0
EABT24686	5023.7	7835.26	9527.88	6962.82	5234.29	3353.53	4306.03
EABT24687	9	17	19	0	8	1	1
EABT24688	7	8	23	5.99	0	0	0
EABT24689	0	1	3	1	0	0	0
EABT2469	939.06	1340.85	1005.04	2393.74	1027.3	687.88	699
EABT24690	3	2	9	0	0	0	0
EABT24691	14992.3	72247	20180.7	4835.19	24507.1	106	1009.25
EABT24692	1	5	17	4	1	1	0
EABT24693	951.97	201	2	6	410.64	58	0
EABT24694	0	2	4	0	0	0	2
EABT24695	63	67	146	140.05	102	58	99
EABT24696	2	0	8	0	0	0	1
EABT24697	2	2	2	1	1	0	1
EABT24698	1	0	3	0	0	0	0
EABT24699	3	1	1	0	0	2	0
EABT247	0	1	1	5	0	0	2
EABT2470	3663.9	5413.87	5317.37	5189.64	4144.59	3647.19	4242.45
EABT24700	7	20	25	22	19	9.01	17
EABT24701	2	1	27	2	1	1	1
EABT24702	10	3	0	1	0	16	5
EABT24703	2	0	7	2	0	2	0
EABT24704	8	31	25	8	0	0	0
EABT24705	0	0	6	0	0	0	0
EABT24706	3	6	53	2	0	1	0
EABT24707	14	5	2	16	1	0	1
EABT24708	2	8	0	0	0	0	0
EABT24709	0	3	3	1	0	0	0
EABT2471	0	1	1	6	0	0	0
EABT24710	0	2	0	1	17	0	0
EABT24711	598.12	964.72	1058.51	1796.84	754	460.83	566.71
EABT24712	920.51	1401.52	1555.62	3510.16	1031.39	1171.64	1017
EABT24713	1131.01	1402.23	2217.33	2587.53	838.46	854.01	624.99
EABT24714	1	0	3	0	1	0	0
EABT24715	0	3	3	1	0	2	1
EABT24716	0	0	0	0	1	0	34
EABT24717	2	4	14	4	0	0	0
EABT24718	0	4	14	14	1	1	1
EABT24719	0.75	0.47	31.6	0	0	0	0
EABT2472	0	1	0	0	1	0	0
EABT24720	488.41	675.09	56.01	64	1091.84	828.31	6137.05
EABT24721	1	2	0	1	0	0	0
EABT24722	5770.97	2054.3	83	9	584.95	708.33	1363.55
EABT24723	0	2	5	1	0	0	0
EABT24724	0	1	18	3	1	0	0
EABT24725	1	3	12	3	0	1	2
EABT24726	1	14	16	2	0	0	0
EABT24727	4	5	30	23	6	0	0
EABT24728	3342.26	4649.62	2049.82	2861.53	2411.22	3802.22	4608.47
EABT24729	2	1	16.04	32	0	0	2
EABT2473	1	4	3	1	0	0	0

EABT24730	0	0	1	0	0	0	0
EABT24731	0	3	5	0	0	0	0
EABT24732	15.99	22	57	19	4	5	3
EABT24733	0	3	1	0	5	1	5
EABT24734	0	0	5	0	0	0	0
EABT24735	0	0	4	0	5	0	2
EABT24736	1	0	20	3	1	0	0
EABT24737	0	0	13	1	0	1	0
EABT24738	0	0	5	2	0	1	0
EABT24739	4	7	18	8	3	6	5
EABT2474	1	1	19	1	0	0	0
EABT24740	64	148	111	670.09	186	12	28
EABT24741	2	3	6	3	3	8	1
EABT24742	7	29	33	49	61	14	23
EABT24743	7885.22	18533.2	11381.1	9754.57	1860.95	1675.17	1674.89
EABT24744	0	1	6	0	0	1	0
EABT24745	16	21	20	48	46.99	8	15
EABT24746	0	1	25	0	0	0	0
EABT24747	2	0	30	0	0	1	15
EABT24748	4	5	4	3	6	2	0
EABT24749	8	10	8	4	1	2	2
EABT2475	2	4	9.07	8	0	0	3
EABT24750	1300.24	1871.48	2059.65	3478.33	1545.9	885.99	1227.45
EABT24751	0	1	12	2	1	0	1
EABT24752	6	7	5	8.57	6	7	8
EABT24753	3	5	16	0	0	2	6
EABT24754	381.24	505.96	382.83	1480.86	520.03	101.8	297
EABT24755	106	183	190	347	96	65	102
EABT24756	1	13	2	14	0	5	4
EABT24757	87	122	147	699.28	35	4	17.91
EABT24758	2	6	2	3	5	1	2
EABT24759	52	151	191.25	358.34	192	17	76.04
EABT2476	2	1	3	7	1	0	3
EABT24760	0	1	4	3	31	0	1
EABT24761	7	1	8	15	1	0	1
EABT24762	0	0	16	0	0	0	0
EABT24763	0	2	4	0	1	0	0
EABT24764	2	3	7	2	5	6	3
EABT24765	4	14	120	23	1	5	2
EABT24766	71	111	280.99	119	1454.01	130	118
EABT24767	7	0	1	0	0	3	0
EABT24768	709	1436	3139.02	2794	1676.78	483	683
EABT24769	1	1	12	0	0	2	1
EABT2477	3	0	9	1	0	1	0
EABT24770	0	1	1	4	0	0	0
EABT24771	3	4	2	7	2	0	1
EABT24772	1	1	4	1	0	0	0
EABT24773	1	0	2	1	0	3	0
EABT24774	0	4	2	1	0	0	0
EABT24775	2	0	15	5	1	5	4
EABT24776	2	1	15	0	0	0	1

EABT24777	6	7	15	48.97	13	0	2
EABT24778	2	15	58	7	0	8	5
EABT24779	0	0	13	1	0	0	1
EABT2478	506.99	491.99	226.99	414.23	537.11	55	407
EABT24780	1	3	9	3	1	0	0
EABT24781	4	18	47	194	18	1	1
EABT24782	1	0	10	1	2	0	2
EABT24783	403	564	708.67	895.13	532	220	335.92
EABT24784	4	5	11	29	8	0	0
EABT24785	3	3	4	2	1	7	0
EABT24786	20	81	57	73	193	1	5
EABT24787	12	68	45	79	18	4	13
EABT24788	1	2	4	4	3	0	1
EABT24789	1	7	2	7	3	1	2
EABT2479	1	1	3	1	0	0	0
EABT24790	0	0	0	0	6	0	0
EABT24791	0	0	4	1	0	0	0
EABT24792	0	1	4	4	0	1	1
EABT24793	15	40	6	4	13	26	37
EABT24794	1399.07	2238.83	3048.52	1828.97	1343.72	1111	1407.59
EABT24795	0	1	12	1	0	0	0
EABT24796	1	5	0	0	0	1	1
EABT24797	3	2	20	8	0	0	0
EABT24798	0	1	8	1	0	0	0
EABT24799	3	2	4	17	10	1	0
EABT248	1	4	1	0	0	10	0
EABT2480	1	0	6	0	0	1	0
EABT24800	0	0	3	0	1	0	0
EABT24801	1	1	25	3	1	3	2
EABT24802	3	0	6	0	0	0	0
EABT24803	3	11	21	14	6	3	7
EABT24804	1	2	12	12	2	0	0
EABT24805	1	2	8	4	0	0	1
EABT24806	1	3	1	4	0	0	1
EABT24807	6	6	7	4	0	0	1
EABT24808	1	5	1	3	1	2	2
EABT24809	0	1	1	5.44	0	1	1
EABT2481	1	6	5	3	0	0	1
EABT24810	0	2	16	1	0	0	0
EABT24811	1	0	3	1	0	0	0
EABT24812	1	0	3	1	1	0	0
EABT24813	0	0	1	0	0	0	0
EABT24814	4	0	3	4	4	2	2
EABT24815	1	1	2	1	4	3	13.82
EABT24816	11	46	9	1	2	4	9
EABT24817	0	5	5	4	3	2	2
EABT24818	2	5	1	0	1	0	0
EABT24819	0	0	16	0	0	0	0
EABT2482	0	0	11.02	1	0	0	0
EABT24820	4	0	10	1	0	0	1
EABT24821	0	2	3	0	1	0	0

EABT24822	399.6	440.27	428.1	1135	459.12	350.33	430.52
EABT24823	0	0	5	0	1	0	0
EABT24824	0	1	4	0	0	0	0
EABT24825	0	2	8	1	0	2	0
EABT24826	2	1	2	8	1	0	10
EABT24827	2	4	8	1	0	10	0
EABT24828	130.03	260	145.68	807.52	260.61	102	165.54
EABT24829	0	0	5	2	2	0	0
EABT2483	7	4	8	7	12	5	12
EABT24830	1	15	2	3	0	2	1
EABT24831	4	6	15	0	2	2	0
EABT24832	277	618.02	654	4605.4	557	204	304
EABT24833	1	7	6	9	1	0	1
EABT24834	2	1	0	3	0	1	0
EABT24835	0	5	7	2	0	1	2
EABT24836	0	0	9	2	6	1	4
EABT24837	2	3	9	0	2	0	0
EABT24838	1091.56	1650.79	1509.05	970	817.02	677	607
EABT24839	125	147	75.92	86	112	15	7
EABT2484	572	698	725.01	653.95	590.01	533	496.99
EABT24840	0	2	2	4	0	0	0
EABT24841	0	0	1	13	0	0	0
EABT24842	23	80	73	906.49	45	3	32
EABT24843	413	867.04	2097.09	871.59	345.95	1172.87	805.91
EABT24844	3	1	7	0	0	1	1
EABT24845	10258	47653.6	66703.8	19786.4	10575.1	272.86	3387.24
EABT24846	8	2	1	1	3	0	0
EABT24847	1374	1920	2601	930	282	32	306
EABT24848	3	2	3	0	1	1	1
EABT24849	0	2	25	3	0	0	0
EABT2485	0	0	8	2	1	0	0
EABT24850	62	68	8	6	6	24	72
EABT24851	1	1	6	7	100	0	1
EABT24852	3	3	6	3	2	0	0
EABT24853	1	2	32	5	1	0	0
EABT24854	3	4	0	1	0	0	0
EABT24855	0	3	0	4	1	1	1
EABT24856	4	6	0	0	0	2	0
EABT24857	68.19	122.06	203	225.48	88	74.95	79.01
EABT24858	3	12	42	3	1	1	0
EABT24859	4	2	10	4	0	2	0
EABT2486	4	16	48.95	10	4	5	6
EABT24860	0	2	10	6	2	0	1
EABT24861	0	3	13	24	1	0	0
EABT24862	1297.99	1755.79	1962.29	2397.73	1335.86	1087.2	1066.15
EABT24863	4125.55	2495.83	6898.14	254.07	463.59	44	386
EABT24864	0	6	9	3	4	0	1
EABT24865	30	5	0	12	3	4	4
EABT24866	0	4	0	4	1	0	0
EABT24867	976.47	2112.84	606.73	2144.98	1414.25	809	1441.19
EABT24868	1	1	1	2	0	0	0

EABT24869	26	39	0	0	0	0	0
EABT2487	0	2	0	7	1	0	0
EABT24870	172	419.69	413.29	527.73	177.01	185	142
EABT24871	0	6	1	8	1	1	4
EABT24872	0	0	8	0	0	0	1
EABT24873	0	4	11	1	1	0	0
EABT24874	0.52	0	4	1	11	4	26
EABT24875	0	2	19	3	0	0	26
EABT24876	0	3	7	0	2	0	0
EABT24877	1	0	3	1	0	2	0
EABT24878	6	2	3	1	0	1	4
EABT24879	0	4	1	6	3	1	0
EABT2488	0	0	11	0	0	0	0
EABT24880	1282.61	2517.99	3057.73	8029.04	1928.08	1321.99	1125.66
EABT24881	1	2	13	0	0	0	0
EABT24882	0	0	0	1	0	0	0
EABT24883	5	8	9	25	5	2	2
EABT24884	8	7	19	4	3	0	3
EABT24885	2	2	4	11	1	1	0
EABT24886	1	3	8	1	0	0	4
EABT24887	178.02	987.85	3602.83	2563.3	2339.38	18	123.91
EABT24888	0	2	2	7	5	0	0
EABT24889	2	1	13	1	1	2	1
EABT2489	771.01	1360	1506.87	2238.3	936.99	695.19	1199
EABT24890	2	2	10	4	14	0	1
EABT24891	0	3	8	25	20	0	1
EABT24892	8	17	62	33	5	7	9.94
EABT24893	1795.35	2390.83	1935.9	2746.04	1432.35	687.66	815.89
EABT24894	0	0	14	1	1	1	1
EABT24895	0	3	9	2	0	0	0
EABT24896	2	0	94	1	1	0	1
EABT24897	3	10	10	521	23	0	1
EABT24898	2	11	171.98	11	2	4	0
EABT24899	0	2	17	120	6	0	1
EABT249	0	0	0	1	0	0	3
EABT2490	52	60	70	3	12	3	15
EABT24900	56	104	187	592	91	0	3.01
EABT24901	526	1116.33	1460.1	1520.32	1209.9	417.85	511.95
EABT24902	22	22	5	2	6	1	5
EABT24903	8	13	84.25	3	20	2	17
EABT24904	0	3	4	0	1	0	1
EABT24905	0	0	8	1	2	0	0
EABT24906	1	3	0	4	1	1	1
EABT24907	482	671.01	1292	1101	944	507	573.04
EABT24908	0	0	1	0	2	0	5
EABT24909	0	2	1	2	0	3	2
EABT2491	4600.16	6747.61	5938.26	12869	3976.03	3197.65	3367.52
EABT24910	0	3	1	0	0	0	0
EABT24911	4	4	4	1	0	6	0
EABT24912	505	744.11	418	1164	640.92	405	394
EABT24913	0	2	14	1	1	0	6

EABT24914	3	14	0	1	0	6	0
EABT24915	1	0	38	7	2	0	0
EABT24916	79	125.57	80	601.11	231	61	32
EABT24917	1	0	9	5	0	1	1
EABT24918	1	4	39	2	0	0	1
EABT24919	0	4	1	4	2	0	0
EABT2492	4	2	28	0	0	1	0
EABT24920	1	1	3	1	179.99	1	2
EABT24921	0	0	7	0	0	0	0
EABT24922	1	0	9	2	0	0	0
EABT24923	4	10	35	42	0	1	2
EABT24924	0	0	2	3	0	0	0
EABT24925	2	4	17	2	1	0	0
EABT24926	0	1	4	5	2	0	0
EABT24927	1407.66	2107.92	2217.77	5349.8	1918.94	1672.27	1936.9
EABT24928	2	8	0	0	0	0	1
EABT24929	14	38	115	58	67	15	27
EABT2493	2174.57	5028.57	2872.09	8569.67	3688.16	1945.29	3202.48
EABT24930	0	2	4	1	0	5	7
EABT24931	2116.45	2207.96	1123	1592.35	4443.89	1517	1361
EABT24932	1	2	5	5	1	1	0
EABT24933	0	1	0	6	4	0	0
EABT24934	498.08	1142.25	2061.71	1211.95	416.4	300.98	376.27
EABT24935	2	7	2	1	0	0	0
EABT24936	4	18	16	31	6	2	11
EABT24937	16	17	62	60	30	34	19
EABT24938	1	1	15	3	1	0	0
EABT24939	1	4	23	0	0	0	0
EABT2494	2	4	26	11	8	13	9
EABT24940	437.24	502.99	1190.91	696.68	423	1338.93	617.91
EABT24941	5	4	21	0	3	3	5
EABT24942	237.16	239	594	886	296.99	74	102
EABT24943	1	0	5	0	1	0	0
EABT24944	4	6	0	0	0	1	3
EABT24945	1	0	4	0	0	0	0
EABT24946	0	0	0	0	0	0	0
EABT24947	999.21	3293.89	3043.69	5441.61	1715.96	1246.17	1217.94
EABT24948	0	1	38	0	0	0	2
EABT24949	7	9	7	6	2	3	3
EABT2495	2	2	26	4	0	2	0
EABT24950	3436.02	5008.9	3493.84	5027.92	2818.73	3673.58	3232.23
EABT24951	16795.6	16155.6	208	1	3	0	0
EABT24952	0	1	3	1	0	1	0
EABT24953	899.93	1094.6	507.12	1133.83	427.54	693.85	545.94
EABT24954	0	0	16	0	0	0	0
EABT24955	0	0	12	0	0	1	0
EABT24956	2	3	11	2	1	1	2
EABT24957	0	0	0	1	0	0	1
EABT24958	1	2	49	8	2	1	1
EABT24959	7	17	16	3	1	17	4
EABT2496	0	0	11	0	0	0	0

EABT24960	0	3	1	15	1	5	2
EABT24961	18	8	8	2	378.91	0	3
EABT24962	0	2	0	1	1	0	3
EABT24963	1	1	4	1	5	0	1
EABT24964	861	971.66	444.11	1215	699.97	706	697.92
EABT24965	0	8	8	19	1	4	2
EABT24966	0	2	10	1	0	0	0
EABT24967	0	4	11	0	0	0	0
EABT24968	274.98	712.98	623.99	422.94	188.14	360.16	271.5
EABT24969	0	1	4	3	15	0	0
EABT2497	1	0	20	0	0	0	0
EABT24970	1392.32	1601.66	1296.3	2865.19	1171.48	831.94	746.29
EABT24971	9	14	10	9	1	1	1
EABT24972	38	57	73	134	58	26	31
EABT24973	24	30	0	0	0	2	2
EABT24974	3	12	5	18	1	2	1
EABT24975	7	1	25	3	1	1	1
EABT24976	2	0	4	0	0	0	1
EABT24977	0	2	37	3	0	1	0
EABT24978	0	2	0	3	0	0	0
EABT24979	3	13	1	3	2	20	24
EABT2498	0	0	20	4.28	0	0	0
EABT24980	49	55	271.9	143	405.79	145.05	244.1
EABT24981	7	16	8	37.99	1	0	2
EABT24982	2075.07	3558.87	4520.34	6364.65	3640.88	1489.88	2250.11
EABT24983	1120	1466.96	1377.52	36	386.74	1386	2638
EABT24984	0	0	0	10	2	1	2
EABT24985	0	0	9	0	0	0	0
EABT24986	4	14	7	0	0	10	3
EABT24987	2	7	25	29	4	3	2
EABT24988	0	3	0	9	0	0	0
EABT24989	0	0	2	0	6	0	0
EABT2499	3	2	34.03	24	1	3	3
EABT24990	1	0	0	0	0	13	0
EABT24991	0	0	0	2	2	0	0
EABT24992	0	2	52	1	0	1	1
EABT24993	1	5	7	0	1	0	2
EABT24994	30	61	62	14	19	1	8
EABT24995	1388.02	2507	2441.01	4955.76	2145.99	1332.35	1779.76
EABT24996	0	0	5	0	0	0	0
EABT24997	1	0	2	1	0	1	3
EABT24998	0	6	22.99	4	1	0	2
EABT24999	0	2	0	0	0	9	6
EABT25	2	1	4	0	0	3	0
EABT250	0	0	8	1	1	0	0
EABT2500	14	4	2	4	5	15	20
EABT25000	1	0	1	1	2	0	0
EABT25001	0	0	0	4	0	0	0
EABT25002	4	3	2	2	22	2	3
EABT25003	6	16	46	4	12	1	1
EABT25004	0	2	4	1	0	0	1

EABT25005	13000.7	9543.52	2488.09	3079.65	5814.32	7527.05	9095.19
EABT25006	0	2	2	21	3	0	0
EABT25007	552.81	1203.67	1885.01	1869.99	934.3	450	485.53
EABT25008	1	4	22	4	2	3	1
EABT25009	3347.38	4469.06	3262.65	8409.51	3178.53	5361.29	5386.76
EABT2501	0	0	29	2	0	0	0
EABT25010	16	59.87	4	0	53	4	46
EABT25011	2063.68	2225.95	563.94	1490.36	1045.23	3482.48	1739.31
EABT25012	1	2	1	6	7	0	3
EABT25013	1	0	6	1	0	0	1
EABT25014	1	3	3	4	0	0	0
EABT25015	3	4	12	0	2	1	0
EABT25016	0	2	0	9	2	0	0
EABT25017	4	4	45	0	1	4	2
EABT25018	0	0	11	0	4	1	0
EABT25019	0	0	3	0	0	0	0
EABT2502	3566.35	5290.61	4670.28	5772.33	5048.59	5462.56	3720.75
EABT25020	2	2	63	1	0	0	1
EABT25021	3	13	4	7	0	6	2
EABT25022	0	0	2	0	1	0	0
EABT25023	0	0	0	0	0	16	6
EABT25024	98.24	232	136.24	194.64	115	36.97	64
EABT25025	4	17	16	25	3	6	3
EABT25026	0	4	3	3	0	0	0
EABT25027	2	4	46	4	1	1	0
EABT25028	4	22	13	13	8.05	3	5
EABT25029	1	3	21	8	8	0	1
EABT2503	6	3	2	3	0	1	4
EABT25030	3515.04	7631.05	2266.3	962.69	2047.81	129	400
EABT25031	408	757.24	688.88	937.91	442.34	263	400
EABT25032	2	8	36	24	14	0	0
EABT25033	0	0	0	5	4	0	0
EABT25034	1	1	0	0	3	0	0
EABT25035	0	2	4	0	0	0	0
EABT25036	985	1314.92	742	165	191	38	64.06
EABT25037	0	2	4	0	0	0	0
EABT25038	0	1	4	0	0	0	0
EABT25039	0	1	1	1	0	1	1
EABT2504	0	2	4	2	0	0	0
EABT25040	14	72	11	0	5	31	33
EABT25041	3167.99	3437.54	211	369	283.79	4016.51	348.36
EABT25042	0	1	9	1	0	0	0
EABT25043	945.17	1131.01	513	20	1028	12	52
EABT25044	0	1	1	9	1	1	0
EABT25045	6	14	62	2	4	2	5
EABT25046	8	16	15	30	8	10	11
EABT25047	3	5	0	5	1	4	2
EABT25048	0	0	0	0	0	0	0
EABT25049	14	20	0	0	0	36	27
EABT2505	27	77	262.99	50	44	36	35
EABT25050	2480.95	3145.77	4534.29	8033.92	1149.87	1086.8	969.21

EABT25051	0	0	2	0	0	0	1
EABT25052	6	11	20	13	8	12	6
EABT25053	0	0	6	1	0	0	0
EABT25054	1	2	7	5	1	0	1
EABT25055	473	532	432	491	592.51	367.49	304
EABT25056	719.19	1014.32	652.09	967.98	789.95	592.99	672.44
EABT25057	11	14	45	104	210.69	10	9
EABT25058	18	40	40	176	90	29	61.91
EABT25059	3	1	1	0	0	0	0
EABT2506	290.88	515.16	402.86	709.16	66	227.84	112.91
EABT25060	1	0	25	0	3	0	0
EABT25061	4	1	2	0	2	0	0
EABT25062	0	2	10	0	0	0	0
EABT25063	1	0	1	10	0	3	0
EABT25064	1096.4	1284	1544.13	2802.35	993.2	984.46	1403.35
EABT25065	1	7	1	0	0	2	1
EABT25066	5	10	18	5	4	3	2
EABT25067	0	13	32	37	16	2	8
EABT25068	4	3	5	5	3	1	1
EABT25069	0	0	2	1	2	0	0
EABT2507	5	8	28	3	2	1	1
EABT25070	2	3	7	6	1	0	0
EABT25071	609.55	1113.29	875.97	2028	584.03	343	427
EABT25072	0	1	5	0	0	0	0
EABT25073	6	13	48	10	4	7	11
EABT25074	1	3	18	0	0	0	0
EABT25075	0	3	9	1	0	1	0
EABT25076	3	5	0	0	1	0	1
EABT25077	0	1	30	0	0	1	0
EABT25078	0	1	7	1	0	0	0
EABT25079	0	1	6	2	0	0	0
EABT2508	2	4	7	3	13	7	9
EABT25080	1	30	16.02	77	133	1	0
EABT25081	0	2	2	2	3	0	1
EABT25082	1	4	6	0	0	0	0
EABT25083	3	16	48	3	0	1	0
EABT25084	1.9	3	6	1	1	0	1
EABT25085	0	0	7	0	0	0	0
EABT25086	1	4	4	0	1	2	0
EABT25087	1	3	4	0	2	0	1
EABT25088	0	1	4	0	1	0	1
EABT25089	2	2	14	2	0	0	0
EABT2509	326	629	386.74	808.01	711.32	261	428.99
EABT25090	40	26	21	0	0	0	0
EABT25091	56	132	292	403	193	44	294
EABT25092	0	0	1	0	0	0	1
EABT25093	4	10	36	9	41	10	72
EABT25094	14	28	76	5	5	1	2
EABT25095	131	17	14	2	2	114.81	0
EABT25096	0	0	19	1	0	1	0
EABT25097	19	44	28	30	7	21	21

EABT25098	7	9	26	6	9	4	11
EABT25099	3739.09	7992.18	6767.51	17254.5	3569.05	3567.71	3746.95
EABT251	0	0	18	18	0	0	0
EABT2510	7	5	2	3	3	1	0
EABT25100	0	1	6	0	2	1	4
EABT25101	0	1	3	1	0	0	0
EABT25102	0	1	26	0	0	0	0
EABT25103	6	3	125	0	0	0	0
EABT25104	0	3	1	1	1	1	0
EABT25105	1	2	2	4	8	3	3
EABT25106	605.75	103.31	8.66	38.01	91.44	205.65	256.71
EABT25107	0	2	3	0	0	0	1
EABT25108	0	0	9	0	1	1	0
EABT25109	9	15	9.95	10	4	4	4
EABT2511	7501.54	9711.66	12723.3	22812.3	6014.95	10297.2	8329.74
EABT25110	11519.4	11510.3	9634.92	5696.14	7330.12	6984	7275.07
EABT25111	1488.04	2362	3374.64	5099.8	2292.63	1239	1218.08
EABT25112	5	3	0	0	0	0	0
EABT25113	3	1	0	0	15	1	10
EABT25114	0	2	1	0	2	0	0
EABT25115	3086.08	2575.46	1551.31	2120.78	6768.26	2581.6	1877.81
EABT25116	0	1	3	3	1	0	0
EABT25117	1	7	1	0	0	0	0
EABT25118	0	0	2	0	0	0	0
EABT25119	3	6	5	12.37	0	0	0
EABT2512	3	4	3	2	1	0	0
EABT25120	2	3	14	44	2	3	0
EABT25121	0	1	2	0	2	0	3
EABT25122	1	0	6	9600	176051	55	475
EABT25123	4879.88	5159.89	4620.01	5186.73	3383.06	5366.74	3436.01
EABT25124	0	0	11	0	1	0	0
EABT25125	3	10	10	24	2	0	5
EABT25126	2	4	15	78	6	0	1
EABT25127	1	1	5	1	1	0	0
EABT25128	2	2	8	1	0	1	4
EABT25129	0	1	6	1	0	0	0
EABT2513	24.99	33	17	207	8	10	30
EABT25130	2025	4215.15	4000.35	5647.93	2918	1406.09	2082.92
EABT25131	2	1	0	0	1	136	5
EABT25132	910.72	1551.55	1098.62	2230.35	1128.54	1258.69	784.47
EABT25133	10	24	19	69	9	6	4
EABT25134	0	4	4	0	0	0	0
EABT25135	4	48.06	72.3	45.71	15	1	3
EABT25136	8	15	0	0	0	0	0
EABT25137	3	3	1	2	366	0	1
EABT25138	468	484	4601.12	1522.07	206	51	6
EABT25139	1	1	14	88	31	0	5
EABT2514	0	1	8	0	2	0	0
EABT25140	4	3	14	6	3	2	1
EABT25141	0	1	4	4	0	1	0
EABT25142	9	3	64.12	9	3	1	0

EABT25143	78484.8	105052	60191.9	60169.5	65370.2	70101.1	89622.3
EABT25144	924.7	1151.73	777.67	1836.48	1104.24	614.27	821.12
EABT25145	1	0	150	5	1	2	1
EABT25146	0	0	8	2	0	0	1
EABT25147	13	107.71	80	64.99	124.12	3	5
EABT25148	22699.9	16622	19552.4	1487	16471.6	15.63	875.99
EABT25149	4	8	0	0	3	2	0
EABT2515	0	1	0	5	0	0	0
EABT25150	0	0	2	5	2	1	2
EABT25151	0	0	17	0	0	0	0
EABT25152	3773.95	4811.62	5388.87	15167.4	6120.23	1889.53	3087.1
EABT25153	1	2	0	13	0	0	0
EABT25154	3	3	11	2	0	5	1
EABT25155	0	1	19	1	0	0	0
EABT25156	1	1	1	2	0	0	0
EABT25157	3	7	5	1	0	0	0
EABT25158	9868.92	14305.2	12341.6	25751.1	9755.78	6705.35	7247.21
EABT25159	1	1	4	8	0	2	2
EABT2516	862.01	1291.95	1227.96	1436.67	1592.16	739.48	1113.95
EABT25160	1	1	3	0	0	2	2
EABT25161	6	18	27	223	8	2	14
EABT25162	80	28	85	40.17	16	18	25.03
EABT25163	0	0	19	2	0	0	0
EABT25164	0	0	4	6	0	0	0
EABT25165	0	0	1	0	1	0	0
EABT25166	1	0	10	1	0	0	0
EABT25167	0	4	1	3	2	1	24
EABT25168	11	18	47	32	3	0	2
EABT25169	2816	4166.27	700	158	1232.31	6350	1667.84
EABT2517	1181.36	2260.25	3648.98	4687.33	1972.03	848	1187.01
EABT25170	0	1	1	1	1	0	1
EABT25171	0	0	15	1	0	0	0
EABT25172	0	0	2	2	1	0	0
EABT25173	4544.32	7496.14	436.04	300	1907.18	4699.63	10090
EABT25174	0	1	4	2	0	1	0
EABT25175	2.97	10	92	4	2	1	4
EABT25176	14	1	1	0	4	0	1
EABT25177	2021.81	3890.61	2302.71	7495.23	2013.87	2091.01	1704.38
EABT25178	4	6	0	0	0	0	0
EABT25179	1	0	2	3	2	0	0
EABT2518	3646.83	3258.75	4322.85	4834.37	2058.83	1199.06	1918.27
EABT25180	67	36	7	1	19	28	120
EABT25181	7	1	24	3	0	0	8
EABT25182	1406.84	2057.12	1573.16	6144.38	1328.79	1232.98	1578.94
EABT25183	13	40.99	134.76	3322.76	81	13	21
EABT25184	1	0	7	0	1	10	3
EABT25185	1	1	1	2	2	0	0
EABT25186	2	0	3	3	3	0	0
EABT25187	1	0	0	13	1	0	2
EABT25188	0	2	11	2	0	1	0
EABT25189	0	0	7	0	0	0	0

EABT2519	0	0	5	1	0	0	1
EABT25190	0	0	21	0	0	0	0
EABT25191	9	6	31	5	1	6	4
EABT25192	204	347	1313.84	268	145	327	239.03
EABT25193	5543.18	9401.41	23590.6	5733.12	7212.19	4133.26	4341.58
EABT25194	0	1	2	1	0	1	2
EABT25195	0	0	9	2	4	3	0
EABT25196	0	2	0	3	1	0	1
EABT25197	0	0	3	0	0	0	0
EABT25198	0	0	3	1	4	0	0
EABT25199	3	12	12	3	0	6	3
EABT252	0	1	12	2	0	2	2
EABT2520	0	3	3	1	0	0	0
EABT25200	0	0	6	0	0	0	0
EABT25201	0	0	37	2	0	0	0
EABT25202	12	9	11	13	10	0	2
EABT25203	15	53	34	28	2	4	6
EABT25204	0	1	6	0	0	9	1
EABT25205	0	1	6	0	0	0	0
EABT25206	0	0	4	1	1	0	2
EABT25207	380.98	226	539.75	1929.48	368	55	107
EABT25208	0	3	12	3	1	0	0
EABT25209	0	1	2	6	0	0	0
EABT2521	1	7	4	21	2	0	0
EABT25210	0	5	2	5	0	0	0
EABT25211	379.02	556.5	533.28	763.59	363.16	413.7	422.86
EABT25212	5626.92	15510.5	21341.5	35360.6	7908.8	5701.86	6031.35
EABT25213	6	6	3	0	0	30	2
EABT25214	0	0	0	0	3	106.49	32.14
EABT25215	3	9	5	0	1	20	1
EABT25216	0	0	0	0	0	0	25
EABT25217	0	3	9	1	3	0	2
EABT25218	3201.07	7434.84	10626.4	29784.3	5541.62	2528.45	3396.71
EABT25219	2	0	21	5	0	0	1
EABT2522	39	54	24	12	7	27	32
EABT25220	1	1	3	3	0	0	0
EABT25221	5	5	9	1	0	4	2
EABT25222	20999	13215.8	3162.93	3894.09	3498.87	26047.6	6144.4
EABT25223	0	2	5	10	1	0	0
EABT25224	44758.1	66561.1	74321.7	14462	3336.81	8871.64	1646.04
EABT25225	11	46	51	31	6	0	0
EABT25226	0	0	4	1	0	0	1
EABT25227	6	17	19	1	11	7	27
EABT25228	0	1	3	3	0	3	6
EABT25229	1	5	29	6	2	6	0
EABT2523	3	7	0	0	0	16	2
EABT25230	0	1	2	0	0	0	0
EABT25231	1	2	5	2	4	0	0
EABT25232	7	33	66	1	7	0	1
EABT25233	0	1	8	0	0	0	0
EABT25234	5	0	1	0	0	0	1

EABT25235	9	23	185	32	9	42	19
EABT25236	2165.33	4259.66	3209.46	4647.84	1975.33	1705.04	1651.33
EABT25237	1117.11	1609.07	2855.98	2530.04	1222.39	1374.23	1145
EABT25238	26	80	691.99	112	456	29	78
EABT25239	111	274.36	89	214	63	25	70.02
EABT2524	1054.99	2057.91	5030.93	4333.99	1219.66	1432.23	1218.07
EABT25240	26.06	87	32.8	6	2	21	24
EABT25241	0	0	7	1	0	0	0
EABT25242	0	5	0	16	20	0	7
EABT25243	2	1	5	4	0	4	3
EABT25244	0	1	0	0	5	0	1
EABT25245	2864.77	3700.27	1644.96	4088.12	2015.15	2048.65	2438.04
EABT25246	4	13	13	13	5	8	9
EABT25247	1	1	10	1	3	0	0
EABT25248	10470	17848.8	1094	83	1467	5788.99	5526
EABT25249	4	9	9	3	7	6	18
EABT2525	0	0	20	2	0	0	0
EABT25250	0	1	4	2	1	1	0
EABT25251	0	8	106	6	13	0	2
EABT25252	1953.97	2577.33	2607.12	4083.38	1981.37	2621.98	1351.4
EABT25253	0	0	1	1	2	0	0
EABT25254	17	36	24	31	39.97	4	34
EABT25255	0	0	1	0	0	0	0
EABT25256	0	4	5	7	1	1	1
EABT25257	0	1	19	3	1	0	0
EABT25258	3	3	2	0	0	1	1
EABT25259	44	74	21	566	92	18	34
EABT2526	23	77	12	11	15	1	2
EABT25260	0	0	5	1	0	0	0
EABT25261	4505.28	8057.94	8154.62	11808.3	6091.42	5068.29	6959.82
EABT25262	625	1872.2	4547.66	2198.93	3246.49	235	690
EABT25263	13	35	27	59.68	9	0	0
EABT25264	2587.39	4270.65	7080.64	5277.54	2575.66	2203.33	2205.46
EABT25265	5	0	0	0	0	6	0
EABT25266	480.99	841.83	1028	2257.4	628.57	437.11	497.6
EABT25267	0	0	2	2	1	0	0
EABT25268	7	12	2	52	24	1	5
EABT25269	5346.02	9249.65	5426.76	7117.48	3615.83	3466.61	2890.6
EABT2527	1	1	12	1	1	2	0
EABT25270	1	1	9	3	1	4	2
EABT25271	0	1	2	3	0	0	0
EABT25272	2	1	0	0	0	1	2
EABT25273	321	576	941.39	764	506.7	260	291
EABT25274	1	9	4	23	6	2	1
EABT25275	108	808.98	31	2	89	1	722
EABT25276	9	9	0	0	0	0	0
EABT25277	499.92	759.44	704.01	1382.89	903	593.74	397.07
EABT25278	0	3	4	1	0	0	0
EABT25279	3	3	12	0	1	0	1
EABT2528	1	1	13	4	0	0	0
EABT25280	0	0	0	0	11	0	0

EABT25281	10	49	62	27	31	5	2
EABT25282	14	23	118	41	17	66.01	52
EABT25283	31	113	194	180.02	175.99	11	13
EABT25284	397.56	462	267.1	485.1	287	445	272
EABT25285	45	109	75	302	180	25	113
EABT25286	3	5	15	2	0	1	1
EABT25287	1	0	7	2	0	1	1
EABT25288	0	1	0	0	5	0	0
EABT25289	2	5	2	5	0	7	1
EABT2529	0	0	8	0	0	0	2
EABT25290	3	0	3	1	3	8	0
EABT25291	0	1	0	4	0	0	0
EABT25292	0	0	3	3	0	0	2
EABT25293	59	43	70	61	47	42	27
EABT25294	0	1	15	21	1	0	0
EABT25295	0	1	3	0	2	1	1
EABT25296	0	2	5	1	0	1	0
EABT25297	23	11	2	0	1	25	0
EABT25298	0	3	6	0	0	0	0
EABT25299	1	2	2	5	0	1	0
EABT253	0	0	0	1	2	0	0
EABT2530	0	2	35	8	0	1	0
EABT25300	0	0	3	1	4	0	0
EABT25301	2	40	93	8	21	0	6
EABT25302	1	1	7	5	2	0	0
EABT25303	506	877	529	1323.63	601	272	341
EABT25304	1	1	6	0	0	10	1
EABT25305	2823.86	3578.05	2300.18	4769.81	3701.87	3949.57	4223.78
EABT25306	0	0	1	0	2	0	0
EABT25307	6	4	0	2	0	16	2
EABT25308	2	1	32	6	0	0	1
EABT25309	0	2	165	5	0	0	1
EABT2531	0	2	8	1	0	0	0
EABT25310	0	0	33	0	0	0	0
EABT25311	2	0	10	4	6	1	1
EABT25312	518.66	909.44	574.33	2598.87	944.44	14.95	205
EABT25313	1	0	1	0	1	0	2
EABT25314	2	4	24	6	2	0	0
EABT25315	2	2	6	18	10	0	0
EABT25316	6	9	8	16	6	2	5
EABT25317	0	0	1	3	1	0	0
EABT25318	3	11	49	14	2	8	2
EABT25319	0	1	9	0	1	0	0
EABT2532	1	1	2	2	0	1	0
EABT25320	1	2	20	10	2	2	1
EABT25321	2	4	3	0	0	7	1
EABT25322	0	2	7	0	1	2	0
EABT25323	70	65	878	60	72	417.82	52
EABT25324	14	13	13	8	4	0	1
EABT25325	3	17	18	22	5	1	1
EABT25326	0	2	7	0	0	0	0

EABT25327	0	5	1	0	2	13	74
EABT25328	25	11	17	2	3	44	35
EABT25329	0	2	0	4	0	0	2
EABT2533	0	0	22	0	0	0	1
EABT25330	557	1088.15	2329	2139.17	951	118	287.01
EABT25331	1	2	1	4	2	1	2
EABT25332	0	0	0	0	0	0	0
EABT25333	3273.26	3944.11	4257.24	4488.23	2001.37	2710.94	2583.97
EABT25334	0	0	0	0	2	0	0
EABT25335	0	2	1	0	0	0	0
EABT25336	0	1	3	1	0	1	1
EABT25337	2	0	5	1	0	0	0
EABT25338	769.66	1098.09	387.92	17	532.24	97	424.01
EABT25339	0	0	5	0	1	0	2
EABT2534	5	4	1	2	1	1	0
EABT25340	0	0	9	1	0	0	0
EABT25341	2	1	1	5	0	2	0
EABT25342	1	7	35	4	1	0	0
EABT25343	1009.16	2050.49	79	15	308	5	4197.96
EABT25344	1	0	3	1	1	1	1
EABT25345	2	8	1	1	1	2	5
EABT25346	93	126.94	80	77.84	34.01	38	5
EABT25347	1	2	11	0	1	1	1
EABT25348	1877.05	4791.41	3584.5	6601.23	1705.59	2342.99	1546.16
EABT25349	0	118	1	1	1	1	0
EABT2535	531.39	731.71	592.23	962.35	563.63	299.79	555.54
EABT25350	2	2	9	3	0	2	0
EABT25351	17	10	0	0	2	0	1
EABT25352	2	3	28	3	0	0	0
EABT25353	0	0	4	7	0	0	0
EABT25354	3	1	4	1	0	11	3
EABT25355	0	0	0	0	0	0	4
EABT25356	0	1	1	3	0	0	2
EABT25357	12	41.01	10	121	242.99	0	21.98
EABT25358	3	2	2	14	0	2	0
EABT25359	0	2	7	1	0	0	0
EABT2536	5	2	5	24	2	0	1
EABT25360	944.67	1358.86	1615.86	2192.49	1307	1659.06	1003.83
EABT25361	0	1	2	7	1	0	0
EABT25362	1972.23	3148.24	4738.74	3500.21	2318.07	1305.17	2172.47
EABT25363	0	2	6	1	0	0	0
EABT25364	0	0	0	10	1	1	0
EABT25365	0	1	8	0	0	0	0
EABT25366	0	0	0	1	0	0	0
EABT25367	0	2	4	0	2	0	1
EABT25368	11.66	16	9	15.68	1	28	5
EABT25369	0	1	3	0	0	0	1
EABT2537	0	0	10	3923.42	0	0	0
EABT25370	134	191	531	639	273.98	21	167
EABT25371	0	5	11	5	1	1	1
EABT25372	0	1	3	0	0	0	0

EABT25373	269.28	490	749.47	1066.99	457	32	326
EABT25374	0	1	15	0	0	3	1
EABT25375	0	0	2	3	0	0	1
EABT25376	5	11	0	1	2	12	27
EABT25377	38	1123.34	2051.49	3	367	5	3
EABT25378	2	0	0	0	0	0	0
EABT25379	1320.7	1990.23	1964.49	2097.93	1417.01	648.95	1070.86
EABT2538	0	0	0	0	4	0	1
EABT25380	0	0	0	0	0	0	0
EABT25381	274	386	345	245	235	305	225.37
EABT25382	1	0	5	0	1	0	0
EABT25383	770.35	1100.01	883.87	2058.83	940.49	568.78	777.78
EABT25384	6	5	0	0	5	0	6
EABT25385	8	16	1	0	17	44	42
EABT25386	0	0	9	0	0	0	0
EABT25387	0	0	2	0	1	0	1
EABT25388	63033.6	89507.3	21781.4	21397.5	118695	2455	17202.9
EABT25389	36	37	176.66	1049.31	254.9	7	41
EABT2539	1	2	15	1	2	0	2
EABT25390	6	12	0	0	0	0	0
EABT25391	1	3	5	2	0	0	0
EABT25392	0	0	11	0	0	0	1
EABT25393	0	3	0	2	1	0	0
EABT25394	0	3	28	0	0	0	0
EABT25395	4	6	46	24	7	0	7
EABT25396	225.96	182.21	910.14	478	674.01	233	103.46
EABT25397	12	34	203	31	16	13	16
EABT25398	0	0	1	4	4	1	0
EABT25399	1	1	9	1	1	0	0
EABT254	20	43	141	40	46	26	26
EABT2540	1	3	36	2	3	4	3
EABT25400	0	1	7	0	0	0	0
EABT25401	11	21	0	0	0	0	0
EABT25402	2	1	4	1	1	0	1
EABT25403	1	12	6	27	0	0	0
EABT25404	0	4	0	6	0	0	0
EABT25405	0	1	17	1	0	0	0
EABT25406	1	0	4	36	0	1	0
EABT25407	0	0	7	0	1	0	0
EABT25408	2	2	22	30	18	0	10
EABT25409	2	2	4	5	0	1	0
EABT2541	0	0	18	2	0	2	0
EABT25410	0	0	4	3	0	0	1
EABT25411	0	2	2	1	1	0	0
EABT25412	1635.2	2773.96	3595.92	3016.37	2953.09	1356.95	2026.08
EABT25413	0	2	3	13	1	0	0
EABT25414	3	1	16	6	0	0	0
EABT25415	0	0	4	1	4	0	0
EABT25416	0	0	9	1	1	0	0
EABT25417	7	18	73	36	12	4	13
EABT25418	36	91	128	87	110.73	42	44

EABT25419	0	7	2	8	0	0	0
EABT2542	11	2	1	2	3	2	0
EABT25420	0	3	2	1	1	0	0
EABT25421	1177.91	2192.98	2634.69	5806.45	982	753.99	872.01
EABT25422	0	0	1	0	11	0	0
EABT25423	4	1	2	0	0	1	0
EABT25424	8	20	96.27	343.97	6	0	1
EABT25425	20	8	62.26	38	198.99	6	3
EABT25426	10866.1	1194.86	83.96	11	54.16	9699.98	269.5
EABT25427	1	19	4	2	9	0	2
EABT25428	4	20	100	8	5	4	12
EABT25429	0	2	26	2	5	1	21
EABT2543	6	34.03	211	42	7	2	4
EABT25430	476.63	471	69	132	2	244	0
EABT25431	19	25	49	13	12	6	4
EABT25432	0	3	14	0	0	0	1
EABT25433	45	81	410.81	239.06	42	80	53
EABT25434	0	0	13	0	0	0	0
EABT25435	0	0	9	0	0	0	0
EABT25436	1	1	7	0	0	0	0
EABT25437	4	5	25	1	1	2	0
EABT25438	1491.03	2376.27	5093.69	2179.74	1463.72	1754.72	1307.77
EABT25439	0	11	9	2	19	1	2
EABT2544	5723.58	7077.26	6501.69	9389.79	3791.34	3755.39	3102.36
EABT25440	1	2	2	1	1	0	1
EABT25441	0	3	9	0	3	1	0
EABT25442	1078.71	1334.91	5677.59	1596.57	1150.89	9	113
EABT25443	0	1	17	2	0	0	0
EABT25444	2	8	15	34	10	2	115.28
EABT25445	0	3	4	1	1	1	2
EABT25446	0	1	11	1	2	1	6
EABT25447	0	3	12	0	1	1	0
EABT25448	997.5	877.8	107.97	47.97	379.34	1233.31	1372.12
EABT25449	0	2	2	0	2	0	0
EABT2545	0	0	2	3	1	2	0
EABT25450	0	0	0	0	0	0	0
EABT25451	5	11	9	1	2	0	0
EABT25452	0	0	6	0	0	0	0
EABT25453	0	4	18	0	1	0	2
EABT25454	0	5	4	1	2	6	0
EABT25455	11	7	0	0	2	11	11
EABT25456	7	5417.03	369	134	33	1	244
EABT25457	217.83	369.09	647.64	440.97	280.97	240.76	133
EABT25458	79	46	0	0	4	46	37
EABT25459	2	0	10	2	0	0	0
EABT2546	8	4	3	26	7	1	3
EABT25460	780	870.68	349	1367.23	732	756.66	573.83
EABT25461	179.71	290.26	442	563	72	7	96
EABT25462	0	1	2	1	1	4	0
EABT25463	2461.31	2157.46	1334.74	689.72	2078.17	5870.48	4435.59
EABT25464	2	1	9	0	3	0	1

EABT25465	3	27	36	5	106	0	0
EABT25466	4	19	94	27	16	0	6
EABT25467	1	2	8	4	0	0	0
EABT25468	37	100	36	0	1559.18	16	117
EABT25469	0	2	3	1	0	0	0
EABT2547	29486.7	20856.1	36246.4	7795.95	4765	30	189.01
EABT25470	3	3	5	4	1	2	1
EABT25471	3632.63	2851.22	2513.91	3102.04	1511.99	3361	1149
EABT25472	7455.72	6930.3	5370.42	4991.74	4281	7216.57	4973.87
EABT25473	1	12	2	0	0	0	0
EABT25474	0	11	1	0	0	1	1
EABT25475	0	0	8	1	0	2	2
EABT25476	1	8	8	0	0	3	0
EABT25477	66	205.68	142	343	35	87	93
EABT25478	5303.85	5761.35	209.83	221.99	2685.36	8545.68	17497.3
EABT25479	7	12	0	0	0	15	7.27
EABT2548	202	316.63	982.24	801.07	324.48	171.1	195.01
EABT25480	2	2	54	2	0	9	1
EABT25481	1	0	40	3	0	0	0
EABT25482	714.01	1197.25	1193.97	1164.96	917.5	549	911
EABT25483	1	1	2	1	2	0	0
EABT25484	1	2	1	2	1	0	0
EABT25485	10	30	52	55	30	2	6
EABT25486	0	0	2	6	0	1	0
EABT25487	0	1	22	0	0	0	0
EABT25488	0	3	4	0	0	2	0
EABT25489	18	8	26	2	0	4	1
EABT2549	65	46	201	10	23	118	40
EABT25490	8	4	29	15	4	2	1
EABT25491	0	0	7	0	0	0	0
EABT25492	2982.1	5787.68	4663.01	3759.95	3521.03	1903	6768.89
EABT25493	1	1	23	0	0	2	0
EABT25494	925.49	1044.65	669.67	1336.38	720.54	362.54	450.97
EABT25495	2	2	11	3	1	0	1
EABT25496	0	0	0	0	5	0	0
EABT25497	16	22	31	42	12	17	14
EABT25498	523.63	817.47	430.01	935.83	615	361	466.02
EABT25499	1829.68	4551.24	4959.91	2037.97	2201.2	2630.21	2716.31
EABT255	0	1	1	0	3	0	0
EABT2550	4	3	0	0	0	1	0
EABT25500	6	25	33	35	6	1	3
EABT25501	1057.78	1634	1992	3136.32	1101	911.78	969.02
EABT25502	2	2	2	1	2	0	1
EABT25503	3	3	8	1	0	0	0
EABT25504	35	68	24.72	6	18	1	1
EABT25505	0	1	10	1	0	0	0
EABT25506	0	1	14	0	0	0	0
EABT25507	8	22	50	80	49	16	50
EABT25508	1419.01	1285.32	1331.52	1620.78	844.01	1015.91	1072.74
EABT25509	0	2	5	4	6	0	0
EABT2551	5398.78	4258.63	3435.9	1945.25	2148.1	6733.68	5735.16

EABT25510	0	1	7	1	0	1	0
EABT25511	0	0	4	0	0	0	0
EABT25512	2346.68	3665.36	3193.09	7222.81	3424.29	3060.2	2501.2
EABT25513	0	4	57	2	0	1	0
EABT25514	3084.57	4784.89	5419.39	8398.06	3723.08	4645.19	3493.07
EABT25515	0	1	1	1	1	0	0
EABT25516	915.81	1275.43	1238.58	1927.43	996.58	752.06	820.55
EABT25517	1	5	139	17	0	2	0
EABT25518	1	2	11	0	0	0	1
EABT25519	1	4	8	5	2	0	0
EABT2552	0	1	0	2	1	0	0
EABT25520	0	3	7	2	0	3	0
EABT25521	1	2	18	2	0	1	0
EABT25522	0	0	0	0	16	0	0
EABT25523	906.11	2104.85	571	474	937	6	28
EABT25524	5	7	5	1	0	0	0
EABT25525	267.11	415.02	379	1265.5	191	141.97	142.5
EABT25526	18	33	2	33	33	0	0
EABT25527	2	1	0	0	1	1	0
EABT25528	1	7	4	1	0	0	0
EABT25529	3933.24	4956.42	7355.44	6713.2	2724.57	3419.3	2747.72
EABT2553	0	0	14	1	0	0	0
EABT25530	0	0	1	1	1	0	1
EABT25531	5	5	0	2	0	5	0
EABT25532	0	1	3	0	0	1	0
EABT25533	11	40	9	2	2	120	32
EABT25534	26	15	87	105	66	35	17.98
EABT25535	8	14	59	87	132	2	42
EABT25536	1	3	3	0	0	0	0
EABT25537	405	586	641	1124	328	226	153
EABT25538	3125	4587.28	850.3	822.71	886.03	5134.41	3184.18
EABT25539	0	2	23	2	0	2	0
EABT2554	0	0	9	2	0	0	0
EABT25540	68	55.95	44.96	142	91	31.07	61.91
EABT25541	49	193	89	10.04	31	0	25
EABT25542	85	100.76	17	0	3	21	12
EABT25543	0	0	9	0	0	0	1
EABT25544	0	2	4	0	3	3	4
EABT25545	7	8	5	17	2	4	9
EABT25546	0	1	5	2	0	0	0
EABT25547	0	2	2	1	4	4	19
EABT25548	660.89	1249.07	678.99	1855.01	700.96	1197.4	1351.95
EABT25549	1051.93	1916.62	1100.97	9137.18	2799.69	289.81	941.36
EABT2555	5	6	12	5	0	4	5
EABT25550	0	2	16	2	0	0	0
EABT25551	7	7	45	13	1	7	7
EABT25552	0	0	19	16	0	0	0
EABT25553	20	19	8	26	4	6	2
EABT25554	6328.1	6687.37	4059.93	5193.93	4332.52	6193.88	4544.09
EABT25555	1681	5372.79	197	2134.45	1217.19	425	892.99
EABT25556	21	59	66.01	144	31	4	25

EABT25557	0	0	10	0	0	0	0
EABT25558	2	1	1	1	0	0	0
EABT25559	121	144	183.99	207	126	90	52
EABT2556	0	0	10	2	0	0	0
EABT25560	2	2	1	1	0	1	0
EABT25561	0	0	30	0	0	0	0
EABT25562	0	0	8	1	0	1	0
EABT25563	0	0	6	0	0	1	0
EABT25564	1	1	5	1	1	0	0
EABT25565	0	1	11	1	0	0	0
EABT25566	2	2	21	5	0	4	4
EABT25567	0	0	6	0	0	0	0
EABT25568	1	1	7	0	0	1	0
EABT25569	0	7	2	0	1	1	2
EABT2557	7	8	0	0	0	12	8
EABT25570	0	4	44	3	1	0	1
EABT25571	1	0	3	0	0	0	0
EABT25572	1	0	0	0	0	2	1
EABT25573	0	0	11	1	0	0	0
EABT25574	0	4	12	0	1	0	0
EABT25575	1	0	0	4	2	1	0
EABT25576	1799.03	1973.84	1041.49	967.16	796	1344	921.78
EABT25577	0	1	1	5	4	1	1
EABT25578	5	19	14	11	0	0	2
EABT25579	3	6	10	7	3	1	3
EABT2558	1	0	10	1	0	0	0
EABT25580	8	10	22	12	0	2	6
EABT25581	5	24	29	5	242.01	1	23
EABT25582	2	1	7	1	1	0	2
EABT25583	8	15	2	2	0	9	1
EABT25584	0	1	9	1	2	0	0
EABT25585	3	1	3	8	0	5	0
EABT25586	930.49	1213.99	2244.1	1370.41	580.16	1491.35	1589.1
EABT25587	422	684.03	741.83	797.41	671.02	344.44	478
EABT25588	1	1	13	0	0	0	0
EABT25589	0	0	1	1	0	6	0
EABT2559	0	0	23	0	0	0	0
EABT25590	1	0	18	0	1	0	0
EABT25591	4	5	16	1	0	4	11
EABT25592	1	0	0	0	1	0	0
EABT25593	584	710.89	1932.19	8749.61	1220.48	123	303.39
EABT25594	0	3	0	0	3	0	0
EABT25595	4	2	11	1	0	0	3
EABT25596	7	23	105	64	105.33	4	7
EABT25597	1	1	7	1	0	0	1
EABT25598	2	3	1	3	1	1	2
EABT25599	1	5	12	3	2	0	0
EABT256	0	0	16	1	0	0	0
EABT2560	1	0	30	1	0	0	0
EABT25600	7	9	20	2	6	1	1
EABT25601	1942.92	3684.88	3366.75	5261.48	6906.94	3	146.53

EABT25602	3257.4	4915.49	3089.6	9228.32	3296.84	2349.08	2878.2
EABT25603	309.31	369	236	61	331.85	1248.09	1612.3
EABT25604	0	1	6	2	0	0	0
EABT25605	0	0	0	1	0	4	1
EABT25606	0	1	5	1	0	1	0
EABT25607	0	3	0	0	0	0	1
EABT25608	467	551	217	651	395	242	262
EABT25609	2	0	14	2	0	0	0
EABT2561	0	2	0	1	0	0	1
EABT25610	7	13	0	0	0	14	12
EABT25611	2	0	9	0	0	0	0
EABT25612	4	11	10	6	3	0	3
EABT25613	33	11	6	6	8	19	37
EABT25614	0	0	16	2	0	0	0
EABT25615	0	4	16	7	2	6	4
EABT25616	2	2	3	6	0	1	1
EABT25617	1019.08	119.11	53.17	51.87	151.46	1006.34	547.05
EABT25618	8	26	30	42	9	8	2
EABT25619	64	28	33	13	76	1	12
EABT2562	4	8	9	5	10	3	2
EABT25620	0	2	6	2	1	0	0
EABT25621	5	1	0	1	0	2	0
EABT25622	0	0	19	1	0	1	1
EABT25623	7	6	17	4	6	4	5
EABT25624	395	777.15	408.8	730	314.01	74	108
EABT25625	74	116	84	144	86	20	52
EABT25626	306.36	539.45	568.64	1698.52	498.57	169.38	302.52
EABT25627	17	5	0	0	1	9	1
EABT25628	3	2	16	4	0	0	0
EABT25629	0	2	1	2	0	0	0
EABT2563	2304.55	2778.48	851.93	1209.57	1702.04	1936.72	2605.96
EABT25630	1	1	16	3	0	3	2
EABT25631	1	9	48	4	11	0	9
EABT25632	4	3	54	6	0	1	0
EABT25633	8	19	0	0	1	0	1
EABT25634	23	9	25	15	10	16	9
EABT25635	5	4	11	4	2	1	0
EABT25636	686	2196.03	22546.9	3210.61	1319.97	921.3	771.95
EABT25637	1	6	2	91	3	0	0
EABT25638	231.28	3050.76	4963.93	3191.98	1519.46	104	292.99
EABT25639	1	4	19	5	1	0	0
EABT2564	1	3	2	4	1	0	0
EABT25640	3	2	0	0	2	0	1
EABT25641	1	1	0	0	1	19	3
EABT25642	2	2	0	4	0	0	0
EABT25643	765.03	1250.96	2260.71	1800.03	1116.62	929.73	777.97
EABT25644	210.03	1026.12	1193.15	866.21	3733.62	22	260.05
EABT25645	2821.19	3996.88	2987.83	5239.88	2094.77	2923.06	2929.99
EABT25646	13	35	62.46	76	1	0	0
EABT25647	4	4	1	0	0	3	0
EABT25648	20	19	40	30	7	16	14

EABT25649	0	2	5	25	2	0	0
EABT2565	427	555	579	792	450	365	293
EABT25650	1	1	1	3	4	0	0
EABT25651	2	1	5	2	0	0	1
EABT25652	588.96	745.06	801.02	1190.54	638.76	442.43	521.32
EABT25653	0	7	4	7	1	0	0
EABT25654	5340.39	8928.31	6364.68	25796.6	5398.93	4034.13	5542.89
EABT25655	8	1	0	0	2	2	4
EABT25656	2	1	7	3	1	2	1
EABT25657	3	2	0	0	0	2	3
EABT25658	3	8	3	0	3	3	0
EABT25659	1	3	0	1	0	8	2
EABT2566	68	240	6	1	8	3	5
EABT25660	1	0	2	0	5	0	1
EABT25661	0	7	1	0	0	0	0
EABT25662	4768.55	12071	12425.7	9649.04	13291.5	2811.12	11320.9
EABT25663	2839.21	4281.5	2932.79	6906.42	3052.56	4614.01	3750.05
EABT25664	0	2	6	2	0	0	0
EABT25665	0	2	1	1	10	0	4
EABT25666	3	4	2	1	2	0	4
EABT25667	608	971.09	1094.27	2153.87	779.09	554	533.98
EABT25668	49.06	31	54	231	11	20	44
EABT25669	0	9	9	5	2	0	0
EABT2567	0	0	11	0	0	0	0
EABT25670	7	3	0	0	0	0	0
EABT25671	186.09	329.1	252.98	690.39	370	189	367.99
EABT25672	0	0	9	0	0	0	0
EABT25673	758	718	273.31	429.21	690	612	567
EABT25674	4091.72	4365.22	1096.08	662.81	772.78	3492.32	2257.1
EABT25675	14	13	42	0	0	4	2
EABT25676	0	0	1	3	0	0	0
EABT25677	27	6	0	1	0	4	0
EABT25678	469.12	640.09	480.95	1534.45	631.91	508.96	467.33
EABT25679	0	0	0	4	2	0	0
EABT2568	0	0	10	7	2108	0	15
EABT25680	11	20	19	6	0	2	3
EABT25681	1	10	3	2	8	4	8
EABT25682	0	1	2	0	2	0	0
EABT25683	0	0	0	30	12	1	81
EABT25684	1	2	1	0	1	0	3
EABT25685	999.68	1955.82	1899.2	4524.98	1242.87	988.87	1008.3
EABT25686	17	38	25	102	20	9	9
EABT25687	4130.81	4709.37	1691.48	6098.8	4265.18	1855.9	2495.58
EABT25688	128.48	283	401.85	1688.94	237	78	52
EABT25689	0	1	2	3	0	0	0
EABT2569	0	0	0	0	2	66	31
EABT25690	0	0	12	0	0	1	0
EABT25691	9724.9	6138.04	2873	5227.26	2624.05	1132.8	1806.52
EABT25692	0	4	2	1	0	3	0
EABT25693	2	5	1	170	68	0	16
EABT25694	0	0	2	1	2	1	0

EABT25695	4	7	19	13	2	12	4
EABT25696	3	1	19	74.99	2	2	6
EABT25697	23	54	18.96	51	15.92	6.85	9.01
EABT25698	11	12	0	2	4	1	17
EABT25699	0	1	3	0	0	0	0
EABT257	721.48	1139.08	750.49	1523.35	756.91	374.7	488.99
EABT2570	10	15	4	0	4	1	39
EABT25700	1	1	14	6	1	1	0
EABT25701	367	352	249.56	584.57	331	121	159
EABT25702	1721	2649.16	4545.42	5183.57	2071.23	1711.71	1594.24
EABT25703	1	1	42	0	0	0	0
EABT25704	0	16	5	1	0	2	4
EABT25705	4	4	120	0	0	2	1
EABT25706	933	10810.5	2131	391.08	6231.22	3	318
EABT25707	158	195	716	9	78	13	51
EABT25708	7902.26	9840.11	5909.87	5576.98	5719.05	1511	1337.57
EABT25709	514	900	1284.85	4308.39	802.01	402.98	402
EABT2571	1152	1396.99	1064.01	1346.18	1146.33	1165	1490.96
EABT25710	3	1	2	1	0	2	2
EABT25711	300.88	387	1059.74	285.94	360.13	660.89	370.99
EABT25712	0	1	0	3	0	1	0
EABT25713	1	9	6	5	1	1	3
EABT25714	0	2	7	0	0	0	0
EABT25715	0	1	14	1	1	1	0
EABT25716	2002.82	4483.37	4622.97	8296.74	5138.21	1567.3	3916.04
EABT25717	4632.14	6829.31	3464	902.64	1361.85	6617.55	5696.06
EABT25718	0	3	0	0	0	0	0
EABT25719	439	339	30	211	69	49	64
EABT2572	4580.98	6638.88	4294	9096.56	5390	85	498
EABT25720	76260.1	100707	184.61	1	26	139560	60
EABT25721	1	13	4	0	4	0	0
EABT25722	3	5	13	9	1	25	5
EABT25723	2.3	5	20	10	5	1	13
EABT25724	0	0	6	0	0	1	0
EABT25725	0	7	3	0	0	0	0
EABT25726	158	271	107	435	200	193.99	114
EABT25727	1	5	3	4	2	0	1
EABT25728	0	2	2	5	2	0	0
EABT25729	0	2	7	3	0	1	0
EABT2573	9	2	2	1	0	3	6
EABT25730	4	6	36	32	6	7	3
EABT25731	0	0	6	0	0	0	0
EABT25732	0	0	7	3	1	0	1
EABT25733	0	0	0	0	3	0	2
EABT25734	1	0	0	2	1	0	1
EABT25735	3	1	1	0	4	2	6.96
EABT25736	0	3	3	3	2	0	0
EABT25737	17	0	0	0	0	18	0
EABT25738	266.94	856.68	10279.6	718.96	531.71	42	39
EABT25739	0	1	13	0	0	0	0
EABT2574	4	1	10.78	2	2	1	3

EABT25740	0	0	14	0	0	0	0
EABT25741	1125.98	1624.99	2551.36	1770.31	1029.67	1062.93	907.3
EABT25742	0	6	0	7	1	0	0
EABT25743	0	1	18	0	0	1	2
EABT25744	20	28	105	24	15	4	14
EABT25745	212	1009.92	2282	26957.7	376	3024.98	978
EABT25746	1564.73	3990.47	3168.37	4805.45	2813.47	1059.3	2429.36
EABT25747	2	2	0	0	1	0	0
EABT25748	1	0	3	0	0	0	0
EABT25749	98	84	26	12	12	210	157
EABT2575	0	2	7	0	0	0	3
EABT25750	1	3	19	0	0	3	0
EABT25751	0	0	6	1	1	1	0
EABT25752	1	0	23	1	0	0	0
EABT25753	0	0	4	0	0	0	0
EABT25754	1	1	4	2	0	0	0
EABT25755	1	1	1	1	1	2	0
EABT25756	1	3	18	0	0	0	0
EABT25757	0	0	0	7.98	1	0	0
EABT25758	0	0	0	0	0	0	0
EABT25759	4	5	5	0	0	4	2
EABT2576	104	0	0	2	1	8	3
EABT25760	2175.18	5463.98	2867.75	514.97	997.68	1026.86	992.96
EABT25761	0	2	6	7	2	1	0
EABT25762	0	2	0	0	1	1	1
EABT25763	1	7	29	0	0	0	0
EABT25764	0	0	3	0	0	0	1
EABT25765	8588.55	6802.7	4444.02	7939.57	5950.02	10418.1	6830.89
EABT25766	1	0	4	0	0	0	0
EABT25767	0	5	12	11	20	0	26
EABT25768	2	3	4	0	0	0	0
EABT25769	2929.15	6056.19	1808.44	538.91	4547.03	118.99	1101.71
EABT2577	1102.99	1605.99	6305.93	21667.9	3645.09	217	970
EABT25770	32	16	6	3	3	34	8
EABT25771	32	79.37	53	73	91	6	38
EABT25772	8	4	0	0	2	34	21
EABT25773	0	1	6	0	0	0	0
EABT25774	0	7	3	5	10	2	2
EABT25775	0.9	2.94	1.94	1	9.39	1	0
EABT25776	0	2	1	0	1	1	0
EABT25777	7	3	3	5	4	16	4
EABT25778	0	4	2	1	2	0	1
EABT25779	0	3	3	1	0	0	0
EABT2578	0	0	1	8	1	0	0
EABT25780	0	0	23	4	0	1	0
EABT25781	0	1	3	0	0	2	2
EABT25782	3	0	0	1	0	0	0
EABT25783	187.81	366.03	2479.54	325.62	360.02	36	144
EABT25784	228	304.06	178	575.3	254.99	143.79	199.91
EABT25785	1	2	11	2	9	0	0
EABT25786	22	45	34	78	14	36	54.07

EABT25787	0	1	46	1	1	1	0
EABT25788	0	1	5	0	0	0	0
EABT25789	21	11	8	30	44	4	47
EABT2579	700	1860.11	1701	14691.6	1388.98	89	318.94
EABT25790	4469.8	7410.01	21031.4	4481.57	2173.44	42	315
EABT25791	2	0	24	1	1	1	4
EABT25792	0	0	9	1	0	0	0
EABT25793	0	1	1	4	0	0	0
EABT25794	3	9.11	5	0	0	12	1
EABT25795	0	7	23	21	3	1	0
EABT25796	0	0	1	3	0	0	0
EABT25797	0	8	25	10	1	8	2
EABT25798	2	16	362	2	25649.8	50	103589
EABT25799	2	5	11.01	22	2	0	0
EABT258	6	22	9	42.01	6	2	3
EABT2580	6613.37	6379.61	4177.81	3117.19	15578.2	5690.4	4276.95
EABT25800	0	0	6	0	2	0	0
EABT25801	370.95	820.32	2637.64	10145.1	679.93	44	141
EABT25802	3936.24	10648.3	7025.76	8308.4	4185.76	5343	7685.87
EABT25803	1	4	10	253	1	0	4
EABT25804	3	3	1	2	0	3	1
EABT25805	5	10	119.72	6	1	7	2
EABT25806	124	347.08	556.06	398.32	115.46	3	27
EABT25807	10	6	112	93	46	4	4
EABT25808	0	0	6	0	0	1	0
EABT25809	16	7	1	4	2	0	0
EABT2581	1448.09	1701.22	953.43	729.67	1535.83	1209.84	519.67
EABT25810	3	3	41	17	11	5	9
EABT25811	2	2	20	2	0	2	4
EABT25812	3	10	0	0	4	3	30
EABT25813	1	2	5	0	0	1	0
EABT25814	0	0	11	6	1	2	2
EABT25815	2	5	34	7	5	1	1
EABT25816	0	0	7	2	3	0	0
EABT25817	51.18	114	215.99	238	39	59.03	25
EABT25818	451.59	970.2	2466.21	1680.85	1122	254.18	401.21
EABT25819	0	0	14	3	0	0	0
EABT2582	507	782.07	1455.65	322	646	336	238
EABT25820	5	7	0	2	2	0	4
EABT25821	1	1	0	8	3	0	2
EABT25822	0	0	3	2	1	0	0
EABT25823	2	5	0	4	0	0	2
EABT25824	0	0	6	0	0	0	0
EABT25825	2	2	0	18	0	0	0
EABT25826	37	9	12	23	1	4	1
EABT25827	1	1	3	1	0	0	0
EABT25828	2	0	3	0	0	0	0
EABT25829	862.11	742.01	2954.89	912.23	354.97	205	888
EABT2583	0	1	0	0	5	0	0
EABT25830	0	0	14	0	0	0	0
EABT25831	529.93	803.08	199.83	485	96	182.94	328.49

EABT25832	0	0	12	4	8	0	7
EABT25833	1	2	20	3	0	3	0
EABT25834	1	7	13	1	1	0	1
EABT25835	2096.15	2019.44	869.31	3092.75	343.28	1	48
EABT25836	1	1	0	9	1	0	2
EABT25837	1	1	3	1	1	2	1
EABT25838	0	7	48	22	5	3	4
EABT25839	1	6	1	2	1	0	1
EABT2584	0	0	3	16.97	0	0	0
EABT25840	4	3	2	2	0	0	1
EABT25841	0	2	4	2	0	1	0
EABT25842	0	4	37	17	0	3	2
EABT25843	0	3	0	8	11	0	2
EABT25844	7.82	27	65	119	96	9	17
EABT25845	5	13	90	19	5	5	11
EABT25846	1	4	15	3	2	0	2
EABT25847	2	0	8	15	103	3	2
EABT25848	241	289.98	392.07	647.9	451.64	117	166.2
EABT25849	15	21	13.43	104	144.99	8	7
EABT2585	12	42	36	32	12	2	9
EABT25850	1	4	5	0	0	17.64	0
EABT25851	1	2	14	6	1	1	2
EABT25852	1	2	9	0	2	2	2
EABT25853	0	1	3	4	0	1	1
EABT25854	0	4	10	0	0	2	1
EABT25855	0	2	24	7	66	0	0
EABT25856	37	85	158	37.05	53	19	23
EABT25857	7	19	87	11	6	7	7
EABT25858	18	14	6	102	15	3	45
EABT25859	4	15	6	19	1	4	5
EABT2586	1	2	2	17	2	0	2
EABT25860	1224	1764	2405.81	3426.99	993	1250	708
EABT25861	0	0	1	1	1	0	0
EABT25862	0	0	4	0	0	7	0
EABT25863	14	20	29	31	37	11	11
EABT25864	3	0	28.99	2	5	0	1
EABT25865	1	0	6	22	1	0	0
EABT25866	5	9	19	6	7	9	9
EABT25867	1	5	4	4	3	0	5
EABT25868	1	2	5	8	3	0	29
EABT25869	0	3	0	2	0	0	0
EABT2587	1	2	7	1	0	1	0
EABT25870	0	0	1	6	1	0	0
EABT25871	5	5	2	0	0	9	0
EABT25872	0	4	23	22	5	1	10
EABT25873	129.2	170	213	449	247	148.76	111
EABT25874	1	1	20	4	0	2	1
EABT25875	0	1	11	1	1	3	1
EABT25876	1	2	0	0	0	0	13
EABT25877	11	21	336.92	79	12	14	13
EABT25878	4	10	6	1	0	0	1

EABT25879	5329.86	4003.08	194	3	8620.44	16184.4	46844.6
EABT2588	0	2	8	0	0	0	0
EABT25880	0	0	1	1	5	0	0
EABT25881	0	4	5	3	0	0	0
EABT25882	2	10	5	7	21	2	0
EABT25883	576.08	630.62	598.87	1299.5	637.99	576	370.03
EABT25884	6	5	1	22	4	1	6
EABT25885	3839.33	2728.26	1870.76	3424	1892.93	4107.71	5016.91
EABT25886	3	11	44	29	4	3	0
EABT25887	3562.74	3313.88	1185.8	5084.15	828.89	2725.01	2135.63
EABT25888	1	0	5	0	0	0	0
EABT25889	3	4	16	9	0	0	1
EABT2589	1830.56	2767.37	3977.52	5423.89	1779.9	1884.31	2054.46
EABT25890	56.55	55.44	51.25	90.74	47.2	33.04	7.96
EABT25891	0	0	0	0	0	0	1
EABT25892	0	1	0	1	2	0	1
EABT25893	0	1	5	1	1	1	0
EABT25894	2099.32	3920.61	1059.99	246.04	617	2074.38	2253.36
EABT25895	0	3	1	0	5	0	19
EABT25896	2	1	9	4	2	0	4
EABT25897	480.84	806.16	1062.46	983.19	858.87	654.02	392.99
EABT25898	2491.05	3385.71	4185.91	2806.7	1742.48	1816.61	1872.48
EABT25899	0	2	3	1	0	2	1
EABT259	0	2	3	2	0	4	0
EABT2590	0	0	15	22	7	3	6
EABT25900	0	0	3	1	1	0	0
EABT25901	3	1	9	169	4	3	0
EABT25902	8	15	7	18	1	4	5
EABT25903	446	1205	2297.24	2919.98	2966.21	65	337
EABT25904	2	5	2	9	5	0	1
EABT25905	10	15	8	9	1	27	4
EABT25906	0	3	149	1	0	1	2
EABT25907	0	6	21	10	6	0	4
EABT25908	321	51388.7	186	14	5	1359	28
EABT25909	11	9	0	3	0	6	2
EABT2591	0	0	2	17	0	0	0
EABT25910	0	0	7	3	0	0	0
EABT25911	0	11	4	10	0	2	4
EABT25912	0	1	13	2	1	0	0
EABT25913	1	7	3	0	0	0	0
EABT25914	415.83	636.58	280.12	1773.91	700.53	311.18	408.75
EABT25915	57	30	58	113	11	21	11
EABT25916	0	1	3	1	1	0	0
EABT25917	4228.78	5949.82	6272.46	8410.42	3275.84	2758.36	3637.43
EABT25918	2	0	5	4	1	0	1
EABT25919	601	2055.84	9758.37	4899.92	2007.25	1009.08	2011.47
EABT2592	0	3	7	5	1	0	0
EABT25920	0	3	0	2	0	0	0
EABT25921	3	4	1	4	0	2	1
EABT25922	7	5	307	1	2	9	2
EABT25923	0	1	1	0	1	2	2

EABT25924	0	0	4	0	1	0	1
EABT25925	1	1	5	0	0	0	0
EABT25926	0	2	3	6	0	1	0
EABT25927	468.98	724.15	1123.7	982.27	597.02	506.91	411
EABT25928	2	5	8	6	0	0	2
EABT25929	0	0	1	1	1	2	0
EABT2593	1	4	1	1	0	0	0
EABT25930	2	2	36	1	2	0	0
EABT25931	596.47	980.85	1076	1051.77	783.94	514	593.45
EABT25932	1104	1088	576.22	1157.93	741.81	1115.03	867
EABT25933	80.98	104.33	69.97	263.72	32	24	42
EABT25934	27250.7	43335.1	42027	12394.3	9394.09	6186.22	9313.09
EABT25935	2	7	31	14	2	0	2
EABT25936	2	0	3	0	0	0	0
EABT25937	1	0	6	1	0	3	2
EABT25938	8	4	11	2	6	8	30
EABT25939	1	3	26	1	0	0	0
EABT2594	0	0	0	0	0	0	0
EABT25940	0	2	19	1	0	0	0
EABT25941	6	2	8	2	0	8	3
EABT25942	0	0	3	0	0	0	1
EABT25943	1	2	8	0	0	0	0
EABT25944	0	3	35	21	2	2	0
EABT25945	2400.39	5308.4	4687.26	8055.71	2314.22	2206.67	2561.26
EABT25946	0	1	2	9	0	0	0
EABT25947	2056.4	3817.79	4879.15	3596.23	4060.73	2003.21	4967.64
EABT25948	3	5	0	0	1	5	6
EABT25949	603.52	916.01	567	925	601	346	527
EABT2595	0	0	9	1	0	0	2
EABT25950	0	0	13	3	0	0	0
EABT25951	0	0	4	3	0	0	1
EABT25952	3	0	1	1	0	0	0
EABT25953	0	1	9.13	5	1	0	0
EABT25954	0.96	0	1	5	0	2	0
EABT25955	0	1	1	2	0	2	5
EABT25956	5822.69	10383	13636.4	7631.76	15346.2	4848.37	4734.19
EABT25957	2	2	7	6	1	0	0
EABT25958	6	4	0	0	1	0	3
EABT25959	55	163	332	1591.86	114	29	23
EABT2596	0	3	5	0	0	0	0
EABT25960	0	2	17	0	0	2	1
EABT25961	2	4	5	2	0	3	1
EABT25962	4605.62	30817.4	31723.8	472	0	22	2
EABT25963	3	5	7	7	1	1	4
EABT25964	0	0	11	0	0	0	0
EABT25965	0	1	32	7	2	2	0
EABT25966	2	1	3	4	0	4	1
EABT25967	1	0	12	9	0	0	0
EABT25968	514.09	375.65	78	12	148.93	1590	1729.14
EABT25969	2640.9	4350.13	5683.96	18465.3	2578.37	4202.72	2768.03
EABT2597	0	4	13.97	1	0	0	0

EABT25970	2	3	56	2	3	1	3
EABT25971	0	2	1	4	0	0	0
EABT25972	0	0	18	0	0	0	0
EABT25973	28	71	126	79	11	13	13
EABT25974	0	0	6	4	0	0	1
EABT25975	1	1	17	0	0	0	1
EABT25976	91.83	430	117	287	461	348	759.97
EABT25977	0	1	34	1	0	0	0
EABT25978	2	17	27	49	10	6	19
EABT25979	996.02	1215.94	18	50	435	1195.15	2531.24
EABT2598	5	39	15	14	3	1	0
EABT25980	0	0	1	0	0	0	1
EABT25981	0	0	1	3	0	0	0
EABT25982	30	59	119	43	27	100.79	48
EABT25983	0	1	10	14	1	1	2
EABT25984	2	7	1	4	1	0	6
EABT25985	2	0	7	0	0	0	0
EABT25986	1	1	3	2	2	0	0
EABT25987	4	2	20	0	3	4	1
EABT25988	2	28	4	35	166	0	4
EABT25989	1	0	6	0	0	0	0
EABT2599	1682.42	2751.12	2579.66	4117.91	1414.03	1909.11	1521.99
EABT25990	0	1	4	0	0	0	0
EABT25991	1382	1786.07	1181	2269.84	1439.87	1336	1028.81
EABT25992	1	5	3	0	0	0	0
EABT25993	2	1	9	1	0	1	2
EABT25994	4	3	73	6	2	1	5
EABT25995	9	76	224	85	10	13	10
EABT25996	5754.88	6998.92	7602.29	8922.22	5187.6	4984.57	4654.27
EABT25997	0	1	1	0	0	0	0
EABT25998	5	6	44	0	2	8	1
EABT25999	12	3	3	1	11	23	9
EABT26	2	3	13	0	1	0	0
EABT260	20	8	170	2	3	6	4
EABT2600	20	42	75	28	10	12	4
EABT26000	1	1	1	0	0	0	0
EABT26001	1720	1562	102	53	703	1	91
EABT26002	0	1	0	5	0	0	0
EABT26003	1	0	1	0	0	1	0
EABT26004	16	18	9	10	7	14	25
EABT26005	2	3	1	2	1	1	0
EABT26006	0	0	3	9	0	0	3
EABT26007	0	1	18	0	1	0	1
EABT26008	211.98	359.69	476.98	663.99	400.73	229	264.99
EABT26009	1	3	6	2	227	0	0
EABT2601	0	1	16	1	0	2	0
EABT26010	20.97	21	0	0	0	1	1
EABT26011	1	3	17	2	1	0	1
EABT26012	0	0	5	0	0	0	1
EABT26013	161	224	205	413	275	172	161
EABT26014	2	3	1	6	2	0	2

EABT26015	4	6	9	12	0	2	4
EABT26016	1	1	11	0	1	0	1
EABT26017	3	10	13	6	4	1	1
EABT26018	4442.93	5124.55	1978.69	1566.15	2113.7	5077.37	4871.73
EABT26019	2	2	0	0	1	3	1
EABT2602	1	3	11	2	1	1	1
EABT26020	3510.62	4164.48	2560.03	3293.22	2322.88	4595.04	4694.38
EABT26021	2	3	15	16	1	0	5
EABT26022	1	1	2	0	3	0	0
EABT26023	6	1	0	0	0	4	0
EABT26024	1	1	8	0	0	0	0
EABT26025	1	2	2	10	2	1	1
EABT26026	2	2	6	8	1	0	1
EABT26027	14	17.1	22	1	1	4	2
EABT26028	11	24	1	8	6	0	23.16
EABT26029	0	0	0	1	2	0	1
EABT2603	0	1	9	0	0	0	0
EABT26030	40	34	0	0	0	1	0
EABT26031	2	2	1	8	0	0	0
EABT26032	1	0	6	4	0	0	0
EABT26033	0	1	7	0	0	0	0
EABT26034	0	3	5	1	0	1	0
EABT26035	0	0	1	0	2.02	0	3
EABT26036	0	0	0	6	0	0	0
EABT26037	2	1	12	6	0	0	0
EABT26038	10	14	19	41	1	2	3
EABT26039	1	1	35	2	0	0	0
EABT2604	2	0	1	0	0	3	2
EABT26040	243.06	381.84	182.88	469.97	636.01	247.88	340.96
EABT26041	0	4	1	1	3	0	0
EABT26042	0	3	33	2	1	0	0
EABT26043	0	0	3	0	0	0	0
EABT26044	2	1	3	0	0	0	0
EABT26045	1	2	11	10	0	0	2
EABT26046	2	0	6	2	0	0	0
EABT26047	0	0	16	1	0	0	1
EABT26048	1	2	1	3	1	0	0
EABT26049	1	3	16	4	1	0	0
EABT2605	1930.51	3001.52	1694.2	2541.96	2515.05	1175.22	3467.61
EABT26050	4	0	0	0	0	3	0
EABT26051	5	51	24	72	12	48	19
EABT26052	1	0	4	0	0	0	0
EABT26053	2	5	7	6	1	0	3
EABT26054	2	9	40	8	2	3	1
EABT26055	331.99	93.14	350.19	74	169.94	58	273.06
EABT26056	0	2	12	3	2	0	0
EABT26057	1	4	2	13	759.22	3	0
EABT26058	4	5	4	16	0	3	4
EABT26059	4	17	33	26	0	3	4
EABT2606	3	8	21	83	10	3	9
EABT26060	1	3	3	1	0	1	0

EABT26061	1	1	2	2	1	0	4
EABT26062	33	48	0	0	1	11	9
EABT26063	621	759.5	663.77	827.84	457.97	452.01	400
EABT26064	10	56	19	256	26	5	19
EABT26065	0	0	3	13	5	0	0
EABT26066	1	0	2	0	0	0	0
EABT26067	5	6	9	6	2	0	0
EABT26068	5	7	6	0	11	5	25
EABT26069	1489.64	1611.12	296.08	29	150.05	1626.01	514
EABT2607	5	7	7	3	3	0	0
EABT26070	0	0	5	1	1	0	2
EABT26071	3	0	7	0	0	0	0
EABT26072	1	2	1	1	7121.19	25	88745.9
EABT26073	0	2	4	1	1	0	0
EABT26074	0	0	1	7	0	0	0
EABT26075	3872.68	5990.05	3966.38	7454.06	3209.52	1883.23	2119.48
EABT26076	0	0	7	1	0	0	0
EABT26077	45	76.33	0	0	0	0	0
EABT26078	0	3	2	5	1	3	1
EABT26079	2	12	4	12	4	6	3
EABT2608	11	21	43	23.03	3	16	3
EABT26080	0	3	3	0	0	0	0
EABT26081	7	3	3	0	2	0	12
EABT26082	0	0	0	14	3	0	0
EABT26083	1	2	3	2	0	0	0
EABT26084	0	0	9	0	0	0	0
EABT26085	4816.71	6664.29	10703.2	3588.69	3726.82	3249.25	3080
EABT26086	0	0	61	1	0	1	3
EABT26087	3	0	0	0	0	0	4
EABT26088	0	0	5	11	1	0	1
EABT26089	15	21	0	0	0	50	0
EABT2609	0	0	11	0	0	0	0
EABT26090	3	10	12	22	14	7	6
EABT26091	0	4	11	2	1	0	0
EABT26092	0	10	9	12	0	0	0
EABT26093	1830.75	2245.3	1702.28	2551.81	1492.88	2167.26	1303.96
EABT26094	1833	2740.41	2570.92	5333.51	2346.24	2175	2570.68
EABT26095	0	0	1	3	1	0	0
EABT26096	9	9	17	1	5	21	28
EABT26097	4519.49	8080.77	15331.6	11680.3	5815.43	5433.22	5542.8
EABT26098	0	0	0	0	1	0	1
EABT26099	2663.17	20717	22	117	4	839.34	6
EABT261	0	0	9	0	0	0	0
EABT2610	0	3	4	9	1	2	1
EABT26100	1475.67	1888.54	2249.98	2216.57	1338.93	1165.85	820
EABT26101	0	5	2	2	0	0	1
EABT26102	4	9	39	64	29	1	7
EABT26103	0	1	24	0	3	3	3
EABT26104	2	6	1	2	2	1	0
EABT26105	8	18	17	70	835	20	58
EABT26106	2	8	3	4	12	5	3

EABT26107	0	0	5	0	2	0	0
EABT26108	1	3	5	0	0	3	1
EABT26109	0	5	26	4	0	1	0
EABT2611	3	13	13	0	0	3	1
EABT26110	0	0	9	0	0	0	0
EABT26111	1	1	0	0	3	13	9
EABT26112	1	0	0	6	0	0	0
EABT26113	9	25	18.94	54	156	0	55
EABT26114	3	3	68	14	2	1	2
EABT26115	3343.34	2950.38	796.43	439	660	3377.44	1886.76
EABT26116	7.01	18	67	52	24	9	24.34
EABT26117	0	0	6	0	3	0	1
EABT26118	13	74	131.16	495	189.99	1	5
EABT26119	3162.22	4460.05	4173.86	5436.41	3159.63	2817.87	2772.72
EABT2612	55	48	0	0	2	43	39
EABT26120	1	0	13	1	1	0	0
EABT26121	1	1	2	1	0	2	7
EABT26122	0	1	13.01	0	0	1	0
EABT26123	1	1	1	6	0	0	0
EABT26124	0	0	7	0	0	0	0
EABT26125	45	234	732.92	1710.56	1138.17	7	56
EABT26126	0	1	15	1	1	0	4
EABT26127	0	0	24	1	0	3	1
EABT26128	35	11	41	3	4	34.95	5
EABT26129	8165.89	15997.9	12401.5	7552.23	52247.2	5879.35	9835.34
EABT2613	2	7.63	20	9	1	1	4
EABT26130	2	4	29	1	4	1	2
EABT26131	8	3	9	2	0	0	2
EABT26132	0	0	1	0	2	0	8
EABT26133	1	4	66	6	2	1	0
EABT26134	0	0	0	0	2	5	4
EABT26135	2	2	7	2	0	0	0
EABT26136	1	0	16	0	0	2	0
EABT26137	0	1	1	1	1	0	1
EABT26138	351.2	1068.86	4337.89	15176.8	2215	73	215
EABT26139	0	2	20	1	0	1	0
EABT2614	408	422	828	1288.01	455	70	176
EABT26140	5	4	13	4	5	1	1
EABT26141	3	4	3	5	0	6	1
EABT26142	0	1	3	1	1	0	0
EABT26143	1	1	2	7	0	0	4
EABT26144	1	4	1	3	0	0	0
EABT26145	2	0	2	4	1	1	0
EABT26146	61	189	623	1092.03	241	12	4
EABT26147	5	15	24	15	1	8	1
EABT26148	0	0	4	0	0	0	0
EABT26149	18976	1742	94	0	2631	498	3636.95
EABT2615	1	3	2	2	1	0	0
EABT26150	2	1	5	0	1	1	1
EABT26151	0	2	6	2	2	1	0
EABT26152	2	9	3	3	3	1	2

EABT26153	2	7	47	151	16.98	6	6
EABT26154	0	1	26	1	0	0	0
EABT26155	1	0	1	13	2	0	0
EABT26156	0	0	18	0	1	0	0
EABT26157	8	16	185	12	3	9	3
EABT26158	2203.39	2801.6	2715.31	4236.2	2254.75	1855.83	1588.41
EABT26159	0	1	3	3	0	0	0
EABT2616	1	0	5	2	0	0	1
EABT26160	13	58	76	48	29	8	26
EABT26161	1	0	2	0	1	1	0
EABT26162	0	0	2	11	6	0	0
EABT26163	0	4	5	0	0	1	1
EABT26164	1	5	2	23	0	0	0
EABT26165	3	4	40	20	20	0	1
EABT26166	3	8	2	5	14	0	1
EABT26167	13	1	2	0	0	0	2
EABT26168	2	1	11	0	1	0	1
EABT26169	1	0	7	3	1	0	1
EABT2617	1	1	0	0	1	0	2
EABT26170	1110.17	742	227	1081	434	608	555
EABT26171	0	2	5	0	1	0	0
EABT26172	0	0	7	1	3	0	0
EABT26173	1	0	8	0	0	0	0
EABT26174	5	3	1	4	3	14	3
EABT26175	0	1	13	5	1	1	1
EABT26176	7	3	8	6	0	2	5
EABT26177	1	0	5	6	0	1	1
EABT26178	8909.71	8758.98	6893.76	7839.49	6360.11	6414.59	7247.92
EABT26179	2	3	3	2	5	1	6
EABT2618	337	54	0	0	1	573.27	0
EABT26180	0	5	56	15	0	2	0
EABT26181	1	2	3	0	0	1	1
EABT26182	6	2	7	0	1	3	4
EABT26183	1636.51	12	0	0	0	268.35	0
EABT26184	58	159	233	92	61	68	106
EABT26185	30	48	40	87	10	76	51
EABT26186	5	8	16	10	2	2	0
EABT26187	3	22	34	21	3	1	4
EABT26188	0	2	19	0	1	0	0
EABT26189	5	16	267	261452	4	0	0
EABT2619	6	14	40.5	4	6.55	10	5
EABT26190	6	9	12	0	2	0	1
EABT26191	1	0	4	0	0	0	0
EABT26192	0	0	1	0	8	0	2
EABT26193	6	32	84	121	45	1	11
EABT26194	1	0	1	0	0	5	0
EABT26195	4	0	36	23	3	0	1
EABT26196	0	3	0	5	1	31	0
EABT26197	1	1	1	0	0	1	1
EABT26198	291	383	327	617	238	237	142
EABT26199	27561	5319.82	41	3	223.98	8590.11	1027.97

EABT262	0	0	5	0	0	0	1
EABT2620	0	0	1	1	2	0	1
EABT26200	0	0	8	5	1	0	0
EABT26201	0	0	14	4	1	0	0
EABT26202	4	1	4	0	0	1	1
EABT26203	9	21	17	36	55	28	72
EABT26204	1	1	4	4	1	0	2
EABT26205	0	1	7	0	0	0	0
EABT26206	3	2	18	2	1	4	0
EABT26207	0	0	2	0	1	0	0
EABT26208	3	0	0	4	1	0	0
EABT26209	0	0	3	0	0	0	0
EABT2621	1015.85	1973.99	561.85	1728.99	1649.68	2185.69	2756.88
EABT26210	0	0	4	0	0	0	0
EABT26211	1	0	2	5	0	0	1
EABT26212	2	0	0	2	0	0	0
EABT26213	2	6	14	1	7	0	5
EABT26214	1	2	4	1	0	1	0
EABT26215	1	4	1	1	0	5	0
EABT26216	6	19	127	21	9	1	3
EABT26217	8	1	15	2	0	0	1
EABT26218	64.11	19	12	5	4	1	2
EABT26219	631	998.98	1381	1499	668.12	589.04	610.58
EABT2622	10	8	1	1	0	0	0
EABT26220	4	3	8	1	0	0	0
EABT26221	1	1	4	3	0	0	0
EABT26222	3	3	7	2	1	0	1
EABT26223	20.99	33.69	27	38	45.12	2	21
EABT26224	0	0	3	2.31	0	0	3
EABT26225	0	0	36	5	2	0	0
EABT26226	6	3	31	1	3	15	10
EABT26227	6	10	15	27	7	6	4
EABT26228	0	0	2	0	1	0	0
EABT26229	412	617	384	839	513.88	89	280
EABT2623	0	0	6	1	0	0	0
EABT26230	3	6	2	12	3	0	7
EABT26231	615.04	1108.28	861.78	1391.29	1158.57	626	652.35
EABT26232	2	1	10	0	1	1	1
EABT26233	0	6	0	0	0	0	0
EABT26234	120	127.04	124.01	306.35	203	47	44
EABT26235	0	0	0	8	0	0	0
EABT26236	87	3	0	2	10	34	39
EABT26237	0	0	5	0	0	0	0
EABT26238	0	4	1	1	0	0	0
EABT26239	8	24	28	41	16	20	11
EABT2624	5	9	1	5	2	1	0
EABT26240	10	7	5	0	1	18	1
EABT26241	0	1	3	0	4	0	1
EABT26242	68	95	13	21	7	62	146.92
EABT26243	4	2	6	0	0	1	2
EABT26244	1	0	18	11	0	0	2

EABT26245	1981.35	2384.3	1110.11	2832.42	1398.68	1192.02	958
EABT26246	0	3	16	10	0	0	0
EABT26247	1	4	1	12	3	0	0
EABT26248	176	282.21	63.12	425	555	134	263.03
EABT26249	5	22	61	23.17	8	8	10
EABT2625	11	28	15	23	1	2	9
EABT26250	17	20	0	0	0	4	1
EABT26251	3	5	49	1	2	0	1
EABT26252	878	1453.01	1622.12	1690.13	1368	755.61	1208.01
EABT26253	0	0	0	0	0	0	12
EABT26254	3	1	0	0	1	5	0
EABT26255	0	3	161.99	1	0	1	0
EABT26256	3	2	2	1	27	1	35
EABT26257	0	1	8	5	2	2	0
EABT26258	2070.97	2666.3	3512.53	6718.08	2030.21	2152.69	1866.6
EABT26259	2608	6581.3	442	944	2392	41	286
EABT2626	3	0	7	3	2	0	2
EABT26260	18	80.95	97	186	91	19	26
EABT26261	6	32	13	4	0	9	3
EABT26262	1	1	3	0	0	0	0
EABT26263	2	17	26	143	4	2	8
EABT26264	0	1	9	1	0	0	0
EABT26265	0	4	24.01	3	1	3	1
EABT26266	1473.99	3146.24	2881.77	2653.48	1834.19	1561.32	2226.35
EABT26267	0	1	0	0	2	0	2
EABT26268	0	0	16	0	0	0	1
EABT26269	968.39	1302.2	1224.25	2960.85	971.79	785.05	657.55
EABT2627	23	28	14	5	24	0	0
EABT26270	0	0	10	3	1	1	1.5
EABT26271	15	30	12	22.92	5	2	3
EABT26272	0	2	15.91	0	0	0	0
EABT26273	2199.3	2822.62	3339.81	2611.38	1765.66	1253.81	1202.61
EABT26274	0	1	11	0	0	1	2
EABT26275	1	7	67	3206.47	8	6	3
EABT26276	0	1	12	1	0	1	0
EABT26277	0	1	6	0	0	0	1
EABT26278	8	12	5	9	0	4	6
EABT26279	2	0	2	2	1	0	1
EABT2628	0	1	5.01	0	0	2	0
EABT26280	1	0	33	2	0	0	1
EABT26281	2	10	7	8	27	6	0
EABT26282	0	0	8	0	0	0	0
EABT26283	3	7.01	6	8	1	5	1
EABT26284	2	4	6	0	1	2	0
EABT26285	1704.96	3431.28	2333.03	9715.85	2348.01	712	2379
EABT26286	0	1	1	1	0	0	0
EABT26287	0	0	4	0	1	1	0
EABT26288	0	3	2	3	0	0	0
EABT26289	14134.7	23722.4	22897.2	36550	9908.72	12225.9	11922.2
EABT2629	3	1	2	11	5	0	7
EABT26290	11	40	35	64	35	16	40

EABT26291	1	1.88	1	5	0	0	0
EABT26292	5031.17	9568.35	10727	16569.2	4449.77	5918.07	4498.82
EABT26293	1	1	0	5	0	0	0
EABT26294	44	26	78	201.99	2	0	1
EABT26295	0	2	7	8	10	1	11
EABT26296	155.04	258.8	4009.64	1023	255.14	35	194.99
EABT26297	4	3	0	0	0	0	0
EABT26298	802.03	1178.67	1169.82	1922.85	713.1	910.23	820.64
EABT26299	1	10	51	4	2	0	0
EABT263	0	4	3	2	1	4	4
EABT2630	1	1	0	0	4	4	22
EABT26300	0	2	2	0	0	2	2
EABT26301	1	5	2	1	2	0	1
EABT26302	1	1	10	0	0	0	0
EABT26303	25	29	23	19.96	5	16	7
EABT26304	1	2	3	0	0	0	0
EABT26305	0	0	24	0	0	0	0
EABT26306	8	72	52	147	153	16	12
EABT26307	2	8	0	0	3	1	9
EABT26308	1	0	1	2	0	0	1
EABT26309	14	23	16	67.54	20	12	20
EABT2631	0	0	18	1	0	1	0
EABT26310	1049.91	1157.22	2177.44	1228.93	649.88	1033.09	556.11
EABT26311	0	0	17	0	0	0	1
EABT26312	1	6	213	1	0	2	2
EABT26313	4	3	6	0	2	0	0
EABT26314	0	0	0	0	1	1	9
EABT26315	0	2	10	2	1	0	0
EABT26316	0	5	2	4	3	1	0
EABT26317	344.93	537.99	339	1368.96	693.5	450.51	505.73
EABT26318	678.41	681	198	800	549.01	308	348.48
EABT26319	4	7	4	1	0	7	9
EABT2632	3183.48	7229.42	8160.72	13941.5	3085.09	5398.03	4313.84
EABT26320	0	1	0	0	2	4	1
EABT26321	0	0	1	6	0	0	0
EABT26322	1	0	5	1	1	0	0
EABT26323	286.52	386.25	660.99	704.83	238.16	187.99	261
EABT26324	0	3	80	2	1	1	0
EABT26325	0	1	3	1	0	0	1
EABT26326	7	19	18	46	6	6	16
EABT26327	4	0	6	2	2	0	1
EABT26328	4	5	93	10	95	4	29
EABT26329	584	388	18	5	11	0	1
EABT2633	36	161	55	178	49	7	8
EABT26330	2	2	0	0	2	5.05	11.01
EABT26331	0	0	14.31	0	0	0	0
EABT26332	1	3	1	15	1	1	3.89
EABT26333	0	1	5	2	0	0	1
EABT26334	270.44	4279.58	2796.78	6412.46	3758.08	7	234
EABT26335	0	1	10	36	1	0	0
EABT26336	1	2	3	9	1	2	1

EABT26337	24	44	117.4	30	65	26	89.99
EABT26338	0	3	0	0	2	1	4
EABT26339	3	0	7	2	1	0	0
EABT2634	0	0	1	4	0	0	1
EABT26340	25	142	103	5	37	10	3
EABT26341	0	0	9	1	0	0	0
EABT26342	9	29	275	50	14	0	5
EABT26343	1	0	1	1	0	3	0
EABT26344	6	3	0	0	0	0	0
EABT26345	0	1	6	0	0	0	0
EABT26346	0	0	0	0	0	2	4
EABT26347	0	9	3	0	0	0	0
EABT26348	95	249.75	257.18	1057	626.99	10	120
EABT26349	6	5	99	5	2	7	2
EABT2635	1	1	4	0	0	1	1
EABT26350	2	4	1	1	0	0	0
EABT26351	0	2	2	0	0	0	0
EABT26352	0	0	3	1	9	0	1
EABT26353	1	1	6	2	1	0	2
EABT26354	19	22	11	67	8	2	1
EABT26355	21	42.07	65	159	79	13	137
EABT26356	0	0	0	0	0	0	0
EABT26357	0	0	8	1	0	1	1
EABT26358	1	1	1	0	2	1	5
EABT26359	307	405	652	714	318	199	226
EABT2636	1	0	3	0	0	1	1
EABT26360	1	0	5	0	0	0	0
EABT26361	3	8	15	3	2	0	1
EABT26362	0	0	9	0	0	0	0
EABT26363	5	9	1	0	1	4	6
EABT26364	116	168	267.84	30	29	22	28.06
EABT26365	1771.79	2939.86	3500.9	4915.51	1570.57	1819.44	1415.59
EABT26366	16	20	6	40	3	0	17
EABT26367	18	1	1	0	0	10	0
EABT26368	1	4	6	9	8.01	1	3
EABT26369	0	0	6	4	1	1	0
EABT2637	1	3	4	1	0	0	2
EABT26370	0	4	1	3	0	0	0
EABT26371	2	0	1	1	1	1	1
EABT26372	0	3	16	20	1	0	0
EABT26373	643	612.8	144	524.93	392.98	327.93	334
EABT26374	3	0	0	2	1	1	3
EABT26375	2	2	2	1	0	0	0
EABT26376	218	177	5	6	116	325.97	648
EABT26377	0	0	5	3	0	0	1
EABT26378	0	0	6	2	2	1	1
EABT26379	0	1	6	1	0	0	1
EABT2638	2	1	5	0	0	0	0
EABT26380	0	1	4	2	0	0	0
EABT26381	0	0	1	2	0	0	0
EABT26382	1	3	1	5	0	1	0

EABT26383	8	11	67	154	23	3	11
EABT26384	0	2	1	3	6	0	3
EABT26385	7	18	37	65	33	7	12
EABT26386	4	39	63.01	179	414	10	36
EABT26387	2	2	19	4	22	0	0
EABT26388	1	9	22	259	34	3	2
EABT26389	0	0	9	1	0	1	0
EABT2639	0	2	13	1	0	0	0
EABT26390	1	3	12	4	136	0	1
EABT26391	0	1	3	0	1	0	2
EABT26392	13	5	2	2	6	25	27
EABT26393	8	16	6	10	4	0	4
EABT26394	0	0	0	0	0	0	0
EABT26395	1	0	4	1	0	1	0
EABT26396	3	8	11	5	1	2	4
EABT26397	214.47	325.57	288.86	733.18	156.01	144.16	149
EABT26398	3	3	3	0	0	3	0
EABT26399	12	15	13	8	1	4	8
EABT264	2	3	2	0	3	0	0
EABT2640	0	0	6	0	0	0	0
EABT26400	0	1	30	15	2	1	2
EABT26401	3	3	17	3	0	0	2
EABT26402	0	1	25	4	0	0	0
EABT26403	9042.27	13200.3	8864.24	12510.1	6454.02	15804.6	8051.04
EABT26404	0	0	0	0	9	0	0
EABT26405	5393.37	9061.6	12240.5	15127.6	6715.58	4466.1	5536.38
EABT26406	1	1	9	4	2	2	2
EABT26407	7	15	14	11	9	8	11
EABT26408	2	5	6	1	1	1	1
EABT26409	7106	6683.95	3270	4772.02	3956.44	8752.63	8968.96
EABT2641	0	4	2	4	0	0	0
EABT26410	0	1	6	0	0	1	0
EABT26411	941.96	1103.81	1007	962.03	830	738	538
EABT26412	4	4	0	2	0	4	1
EABT26413	0	1	21	3	0	2	0
EABT26414	1	6	3	0	0	7	6
EABT26415	3	4	7	53	5	0	1
EABT26416	2	4	9	4	4	2	0
EABT26417	26611.2	34520.7	34597.3	24068.9	13648.5	35718.6	25178.4
EABT26418	0	1	87	3	0	2	0
EABT26419	0	0	5	0	0	1	1
EABT2642	0	4	1	0	0	0	0
EABT26420	2580.34	3464.05	4652.24	4341.68	4250.88	2820.72	2068.77
EABT26421	0	3	17	0	0	0	0
EABT26422	1	3	0	0	0	1	2
EABT26423	13	11	2	21	4	0	9
EABT26424	4	0	3	0	0	0	0
EABT26425	0	0	3	7	1	0	1
EABT26426	0	0	6	0	0	0	0
EABT26427	3	4	13	3	0	2	2
EABT26428	3	15	224	11	0	6	2

EABT26429	1.02	0	3	18	6	0	2
EABT2643	0	1	4	0	0	0	0
EABT26430	10	9	4	2	0	7	5
EABT26431	6	16	15	0	10	11	16
EABT26432	25	36	83	2417.02	4	16	0
EABT26433	0	0	36	16	2	0	0
EABT26434	9	76	5	3	18	0	8
EABT26435	0	0	11	31	8	0	0
EABT26436	1	3	1	0	3	2	9
EABT26437	0	1	15.87	1	0	0	0
EABT26438	0	0	5	0	1	0	0
EABT26439	0	2	7	0	1	1	1
EABT2644	2	5	64	4	3	0	0
EABT26440	23	107	111	147.92	8	6	5
EABT26441	0	1	13	1	0	0	0
EABT26442	0	2	5	5	1	0	0
EABT26443	1	0	2	22	0	0	0
EABT26444	0	0	12	0	0	0	0
EABT26445	5	7	8	1	0	0	1
EABT26446	0	0	17	0	1	0	1
EABT26447	0	0	10	1	0	0	0
EABT26448	0	0	9	5	1	0	0
EABT26449	0	0	1	0	0	1	0
EABT2645	0	9	34	10	7	4	13
EABT26450	1	0	2	1	0	0	1
EABT26451	2	0	13	0	0	0	0
EABT26452	0	0	12	0	0	1	1
EABT26453	11	4	10	11	6	1	1
EABT26454	0	1	11	3	0	0	0
EABT26455	1	5	6	3	0	0	0
EABT26456	51	64	24	9	9	59	33
EABT26457	0	3	5	7	0	0	1
EABT26458	1	1	7	4	0	1	0
EABT26459	0	0	1	2	37	0	0
EABT2646	0	0	0	0	9	0	0
EABT26460	0	0	0	0	0	0	0
EABT26461	0	0	2	4	2	0	0
EABT26462	6	17	127	5.49	4	4	3.16
EABT26463	2153.99	3536.78	3019.04	6447.78	2465.81	1762.97	1779.05
EABT26464	1	0	11	0	0	0	0
EABT26465	0	4	0	1	2	0	2
EABT26466	1	7	7.92	6	0	3	2
EABT26467	1	3	30	0	0	0	0
EABT26468	2	12	9	22	4	3	5
EABT26469	45	65	95	47.04	31	9	28
EABT2647	2	10	8	4	3	1	2
EABT26470	2	0	55	3	1	0	0
EABT26471	205.04	539.99	672.52	759.96	1344.49	299.51	232.65
EABT26472	6081.99	708	15	4	2856.04	4324.58	17945.9
EABT26473	0	4	0	5	0	1	1
EABT26474	1	0	9	0	0	0	2

EABT26475	2	4	1	0	1	1	0
EABT26476	1	4	29	9	4	0	0
EABT26477	8	24	118	30	121	2	32
EABT26478	0	0	15	1	0	0	0
EABT26479	1	1	3	0	0	0	0
EABT2648	2	6	23.01	1	3	0	5
EABT26480	7	11	2	2	1	1	1
EABT26481	0	0	2	4	5	0	2
EABT26482	1	2	15	1	2	4	0
EABT26483	0	0	1	5	2	0	0
EABT26484	0	0	5	9	0	0	0
EABT26485	1	4	1	0	0	0	0
EABT26486	2	25	187.45	63	14	3	6
EABT26487	0	0	0	0	0	0	0
EABT26488	1	2	10	1	0	0	0
EABT26489	1.35	0	2	3	0	2	1
EABT2649	1	1	4	0	0	0	1
EABT26490	0	7	2	4	1	0	3
EABT26491	0	0	7	0	0	0	0
EABT26492	484.02	900.1	1286.71	828.24	1050.48	548.1	438.28
EABT26493	0	2	32	4	0	1	0
EABT26494	3	0	0	0	0	9	1
EABT26495	2	1	18	0	0	0	1
EABT26496	1238.91	1042.73	731.95	8151.53	1487.1	2	29
EABT26497	0	2	6	0	0	0	0
EABT26498	1	6	7	10	1	2	2
EABT26499	43.27	113.98	345	105	22	26	41
EABT265	0	0	1	0	1	1	1
EABT2650	0	3	3	2	1	0	0
EABT26500	1018.16	1230	530.97	1004.67	897.97	649	545
EABT26501	18	19	41	23	14	13	11
EABT26502	0	0	4	0	0	0	0
EABT26503	3850.44	7436.36	452	101	335	1	23
EABT26504	13	19	182	61	45	5	124
EABT26505	1	2	19	0	1	0	0
EABT26506	508.06	532.74	604.14	2438.58	794	360	459.55
EABT26507	1	5	17	1	2	0	0
EABT26508	166	272	81	328	239	339	229.68
EABT26509	1629.95	2647.58	2500.88	2813.23	2123.23	1169.02	1985.23
EABT2651	0	0	2	3	1	0	0
EABT26510	1	4	0	0	0	0	0
EABT26511	1	3	9	3	0	0	0
EABT26512	2	1	0	0	0	6	0
EABT26513	2	2	15	2	0	0	1
EABT26514	0	1	15	2	1	1	0
EABT26515	103	116	301	62	7	533.68	45.91
EABT26516	1	11	29	9	2	1	5
EABT26517	0	0	0	0	2	0	0
EABT26518	27	12	5	7	0	27	0
EABT26519	2	9	24	7	3	2	2
EABT2652	0	1	18	2	2	0	0

EABT26520	970.78	2017.29	1531.42	5492.9	1504.62	745.56	949.2
EABT26521	232.24	359.31	349.6	723.38	206.98	253.8	193
EABT26522	0	2	3	1	0	1	2
EABT26523	3	7	1.57	0	0	2	1
EABT26524	1	2	3	0	0	0	0
EABT26525	0	2	1	0	0	3	0
EABT26526	10	0	0	0	0	2	0
EABT26527	2	4	0	0	0	0	2
EABT26528	2	6	24	20	2	4	7
EABT26529	0	2	5	13	0	0	0
EABT2653	9	1	3	2	1	3	1
EABT26530	7	16	35	28	2	1	7
EABT26531	4	2	1	0	2	7	3
EABT26532	37	51	57	32	47	26	30
EABT26533	915.6	1332.02	1108.37	1448.16	1269.39	1158.36	1166.13
EABT26534	1	1	10	1	1	1	1
EABT26535	6	17	9	0	2	0	2
EABT26536	0	1	4	0	3	1	0
EABT26537	4	1	2	0	0	3	0
EABT26538	15	17	16	24.96	7.87	4	10
EABT26539	11	51	57	175	190.84	11	8
EABT2654	4	2	2	76	0	4	4
EABT26540	2	2	11	7	0	0	0
EABT26541	9	29	53	128	65	29	113.01
EABT26542	1	3	10	4	2	1	2
EABT26543	26	15	4	0	0	36	0
EABT26544	0	0	5	0	0	0	0
EABT26545	0	0	0	5	1	0	0
EABT26546	0	2	10	4	1	1	0
EABT26547	2093.41	5226.07	9782.95	5961.34	4761.22	368.36	1476.95
EABT26548	2	51	420.73	60	0	0	1
EABT26549	0	0	8	0	0	0	0
EABT2655	54	63	58.01	12	15	1	14
EABT26550	0	0	0	1	2	0	0
EABT26551	1	2	4	0	0	1	1
EABT26552	1	5	7	8	4	1	5
EABT26553	6273.7	13944.1	8585.69	6028.66	13036.7	2906.41	4127.3
EABT26554	0	1	10	1	0	3	0
EABT26555	0	0	1	4	1	0	1
EABT26556	0	1	8	0	0	1	1
EABT26557	18	22	75	5	2	0	3
EABT26558	1	9	0	0	0	0	0
EABT26559	7	6	115	5	2	3	2
EABT2656	2	1	40	21	2	3	6
EABT26560	0	0	3	1	2	0	3
EABT26561	1	0	0	2	2	0	0
EABT26562	1814	2116.04	1501	426	782	4249.09	3626.13
EABT26563	315	423	151	651	321	138	133
EABT26564	2	4	2	20	101.35	0	2
EABT26565	1	7	9	0	0	1	0
EABT26566	4	1	7	7	2	3	1

EABT26567	3	5	2	2	1	1	1
EABT26568	0	0	6	0	0	0	0
EABT26569	11041.1	9553.65	2243.95	4729.77	3125.44	13706.1	10895.1
EABT2657	0	0	2	0	0	0	2
EABT26570	3	1	0	5	0	1	2.94
EABT26571	0	0	23	0	0	0	0
EABT26572	3	2	2	10	1	8	0
EABT26573	90	370	293	287.98	519	3	174
EABT26574	0	1	11	2	2	4	0
EABT26575	562	31	234	1618	260	2	4
EABT26576	1	16	18	40	3	0	0
EABT26577	180.39	282.47	510.75	357	300	130	128
EABT26578	4	8	3	5	3	0	0
EABT26579	3	2	4	1	1	0	0
EABT2658	1987.1	3170	3348.83	6310.58	2699.2	1804.12	1907.61
EABT26580	14	8	11	7	1	0	2
EABT26581	1	1	12	1	0	0	0
EABT26582	3	3	8	1	1	0	0
EABT26583	424.35	712.14	523.01	893.7	517.82	418.32	330.62
EABT26584	18	24.86	95	23.57	13	11.01	16
EABT26585	4	1	4	1	0	0	0
EABT26586	0	1	2	0	3	0	0
EABT26587	709.81	1029.6	710.96	1087.8	738.34	451.24	505.57
EABT26588	1819.68	2590.62	8461.72	5781.76	2912.55	3202.19	2609.43
EABT26589	5	3	0	0	0	22	5
EABT2659	4	8	2	5	5	2	1
EABT26590	0	15	0	1	1	1	0
EABT26591	1	0	8	5	0	1	0
EABT26592	0	0	3	3	0	0	0
EABT26593	792.9	1204.06	2076.52	1300.18	936.84	1047.12	1094.83
EABT26594	56	93	0	1	0	74	1
EABT26595	325	371.99	477	633.27	382.98	293	206
EABT26596	3	3	15	0	0	0	0
EABT26597	1265.31	1715.15	1132.7	5234.92	976.18	838.02	1302.91
EABT26598	5	4	1	0	0	1	0
EABT26599	6	32	20	6	2	1	0
EABT266	1941.15	2915.12	1463.85	779.14	946.57	1	21
EABT2660	3	14	48	19	2	9	3
EABT26600	3531.71	5192.99	4916.81	10571.6	3061.01	2577.01	3172.52
EABT26601	1	8	6	5	3	0	2
EABT26602	0	0	5	0	0	0	0
EABT26603	0	9	10	3	12	0	0
EABT26604	0	0	4	0	0	0	0
EABT26605	313	569.11	24	264	961.31	4	39
EABT26606	288	374.04	168	560.97	186	188.01	234
EABT26607	3	11	2	1	1	5	7
EABT26608	1	2	24	5	1	0	0
EABT26609	1	8	3	27	3	0	0
EABT2661	4456.86	6091.07	2768.83	9180.93	5879.51	5066.72	12020.1
EABT26610	0	0	2	2	0	0	10
EABT26611	2	0	1	4	1	1	0

EABT26612	7	10	57	8	1	8	13
EABT26613	2	1	5	1	0	0	1
EABT26614	0	3	6	0	2	0	0
EABT26615	0	2	4	0	0	0	0
EABT26616	3	7	15	1	2	8	7
EABT26617	287.99	699.22	23	22	102	336.9	503.38
EABT26618	559.99	2516.16	316.4	99.73	153.04	282.09	786.01
EABT26619	617.01	1150.9	717	1338	1041	932.37	765
EABT2662	877.55	1306.33	1267.14	1502.56	929.96	742.99	932.02
EABT26620	891	1257.4	1137.56	950	581	520	476.99
EABT26621	3	4	35	8	7	3	11
EABT26622	0	0	0	0	3	0	8
EABT26623	10	4	25	0	0	21	11
EABT26624	28.04	47	107	52	3	1	3
EABT26625	558.24	864.97	778.78	2489.46	546.81	572	420.03
EABT26626	1	1	3	2	0	0	0
EABT26627	1	0	2	0	0	0	1
EABT26628	1	1	15	1	0	0	0
EABT26629	229.25	502.88	828.87	140.55	2110.16	252.67	2408.42
EABT2663	0	9	1	22	0	1	0
EABT26630	3	4	4	7	3	0	1
EABT26631	3	4	3	0	1	0	2
EABT26632	47	125	146	209	222.15	39	16
EABT26633	1184	597.99	1398.12	1123.91	1593.6	153	160.08
EABT26634	7063.51	4534	1154	92	1048	614.92	1917.98
EABT26635	129	215	151	368	79	92	119
EABT26636	4	12	5	3	3	22	14
EABT26637	0	0	16	4	2	0	0
EABT26638	0	2	7	10	3	0	0
EABT26639	1	2	0	1	5	0	5
EABT2664	1	4	1	0	1	3	4
EABT26640	3	1	19	0	0	0	0
EABT26641	1	1	10	4	0	0	0
EABT26642	2	6	4	3	0	0	2
EABT26643	0	0	4	0	2	0	0
EABT26644	0	0	0	0	0	6	0
EABT26645	2	4	14	8	4	0	1
EABT26646	0	2	4	1	0	0	0
EABT26647	1	1	2	0	3	0	5
EABT26648	0	0	2	0	2	0	1
EABT26649	3	1	0	3	1	0	0
EABT2665	93	154.96	165	566.85	506.85	509.13	112.24
EABT26650	1	0	3	5	0	0	0
EABT26651	0	1	14	0	0	0	0
EABT26652	14613	11725.7	6017.71	11104.3	9595.03	17759	12082.5
EABT26653	0	0	1	3	4	0	0
EABT26654	0	0	2	3	0	2	0
EABT26655	0	0	12	10	0	0	0
EABT26656	3117.61	3417.61	4226.41	5330.03	2221.18	3093.43	2333.93
EABT26657	1	1	1	0	1	1	3
EABT26658	1216.3	2184	2650.23	3616.02	948	1565.99	1096

EABT26659	86	3	0	0	0	5	0
EABT2666	1	2	1	2	0	0	0
EABT26660	0	0	2	3	1	0	0
EABT26661	493.01	1221.04	1587.54	1468.6	463.72	506.9	841.72
EABT26662	0	0	11	6	2	1	0
EABT26663	0	3	0	0	2	0	3
EABT26664	0	0	2	6	0	1	0
EABT26665	1680.55	2369.46	2031.46	4381.29	1974.17	1274.03	1296.33
EABT26666	0	0	6	1	0	0	0
EABT26667	27	17	4	7	4	8	54
EABT26668	6837.6	11053.6	7877.95	7114.59	6066.43	4606.71	7445.41
EABT26669	0	10	10	6	0	2	1
EABT2667	0	2	8	129	32	2	0
EABT26670	2	8	14	22	5	3	8
EABT26671	10	42	193.01	392	24	25	23
EABT26672	7	2	1	0	0	6	3
EABT26673	8	6	13	11	1	11	1
EABT26674	1	1	5	8.01	0	0	0
EABT26675	3	6	1	0	0	0	0
EABT26676	17	17	9	15.98	6	4	12
EABT26677	0	1	10	2	0	0	0
EABT26678	1	10	12	16	2	3	1
EABT26679	0	0	4	2	2	0	0
EABT2668	1	0	27	1	0	0	0
EABT26680	5	6	0	0	0	1	2
EABT26681	0	1	14	0	0	0	0
EABT26682	0	1	0	1	2	0	0
EABT26683	0	0	9	1	0	0	0
EABT26684	518	695.96	169	5	1896.01	2	32
EABT26685	320.91	370	169	388	355	320	426.99
EABT26686	1	12	21	1	5	0	5
EABT26687	1607.4	1996.74	450	31	806	2164.98	5249.57
EABT26688	243.22	393.87	177.73	563.52	362	254.34	200.95
EABT26689	1	0	1	10	4	2	1
EABT2669	1	0	0	0	4	0	7
EABT26690	0	1	2	10	0	0	1
EABT26691	278	489.24	1511	2513.01	1361.99	28	29
EABT26692	0	1	2	0	12	0	0
EABT26693	0	3	8	3	0	0	0
EABT26694	1	0	11	2	2	1	0
EABT26695	0	0	11	2	3	0	2
EABT26696	0	1	4	1	2	0	3
EABT26697	0	0	0	0	1	0	0
EABT26698	1030.41	1435.69	865.02	2028.75	1289.94	845.3	900.82
EABT26699	1	0	8	2	5	0	1
EABT267	1	1	3	0	0	0	1
EABT2670	0	4	1	3	0	0	2
EABT26700	16	68	57	14	9	7	4
EABT26701	3	4	3	8	8	0	1
EABT26702	4	3	13	9	0	0	0
EABT26703	4	3	27	7	3	0	0

EABT26704	1	11	22	31	2	5	2
EABT26705	13	15	12	33	1	7	0
EABT26706	14	24	22	107.97	44.84	12	25
EABT26707	2406	3551.76	3007.99	6603.25	1933.47	1381	1700
EABT26708	16	0	185	16	10	1	2
EABT26709	1	0	4	3	3	0	0
EABT2671	0	4	15	0	0	7	2
EABT26710	24	1	0	0	0	9	0
EABT26711	49	80	91	228.42	85	29.99	52.12
EABT26712	1	2	7	2	0	1	0
EABT26713	54	28	159.99	13	1	1	1
EABT26714	0	7	4	6	0	6	4
EABT26715	154.68	340	803	42	91	253	371
EABT26716	0	1	14	1	0	0	0
EABT26717	335.2	474.09	423.02	836.97	585.47	474	470.1
EABT26718	4	28	22	73.49	20	3	4
EABT26719	7	10	22	49	21	2	6
EABT2672	0	0	0	0	0	2	5
EABT26720	0	0	0	0	0	4	2
EABT26721	0	0	1	1	1	0	0
EABT26722	1	2	1	3	0	0	0
EABT26723	0	1	13	0	0	0	0
EABT26724	0	0	11	0	0	0	0
EABT26725	7	18	21	34	1	10	5
EABT26726	175	164.26	32	20	117	324.07	597.53
EABT26727	0	0	3	0	0	0	0
EABT26728	4	10	9	6	26	28	53
EABT26729	0	0	1	1	0	1	0
EABT2673	96.11	211	274.89	277	1091	19	241
EABT26730	161	317.18	808	1137.23	353	140	149
EABT26731	4	30	32	27	53	1	4
EABT26732	0	2	0	0	4	0	0
EABT26733	15	17	1	4	2	29	13
EABT26734	0	2	3	0	0	7	3
EABT26735	0	3	7	27	22	0	2
EABT26736	7	83	278	3730.44	1334.99	17	87
EABT26737	0	0	3	26	0	0	0
EABT26738	3808.32	18976.8	4906.39	4422.55	8504.65	2352.33	1965.72
EABT26739	2	8	9	0	0	0	1
EABT2674	8247.42	13184.1	8854.35	12882.9	7187.17	6605.74	7095.23
EABT26740	461763	219637	3326.99	141	21352.3	148826	244334
EABT26741	0	2	4	1	0	0	0
EABT26742	3	0	2	1	0	0	1
EABT26743	2	1	3	6	0	3	0
EABT26744	54	10	0	0	3	1	12
EABT26745	0	0	6	0	0	0	0
EABT26746	0	2	6	0	1	0	0
EABT26747	7	0	25	6	4	3	6
EABT26748	15	28	9	6	3	4	0
EABT26749	2	4	3	7	0	1	4
EABT2675	680.98	843.12	1067.5	1696.81	576.97	877.07	482.45

EABT26750	0	1	1	3	0	0	0
EABT26751	1	1	0	6	2	0	0
EABT26752	1	1	3	1	1	0	2
EABT26753	14	32	7	7	6	4	8
EABT26754	0	0	6	0	0	0	0
EABT26755	0	6	19	2	0	0	0
EABT26756	0	1	0	1	2	1	0
EABT26757	3	2	2	6	12	0	0
EABT26758	0	1	17	1	1	0	9
EABT26759	8	6	14	7	1	5	5
EABT2676	6	10	46	4	79	2	10
EABT26760	1	1	6	0	0	3	0
EABT26761	4	10	9	0	0	2	0
EABT26762	6	6	115	2	1	0	1
EABT26763	2	5	13	13	3	2	2
EABT26764	4987.13	4623	2745	2557.65	1882	4052.22	2364.02
EABT26765	2018.21	4869.75	5593.24	5203.3	4597.88	2271.49	2933.05
EABT26766	0	2	8	6	0	0	7
EABT26767	0	1	3	2	1	0	1
EABT26768	1	1	14	4	0	0	0
EABT26769	0	2	0	4	0	0	0
EABT2677	0	1	4	0	0	0	0
EABT26770	0	0	3	0	0	1	1
EABT26771	7	15	0	0	1	36	30
EABT26772	2	13	9	0	4	0	0
EABT26773	5	5	19	2	3	0	0
EABT26774	0	0	3	8	2	0	0
EABT26775	3	2	32	4	3	0	0
EABT26776	0	0	1	3	0	0	1
EABT26777	0	1	30	5	25	3	22
EABT26778	2	4	16	0	0	1	1
EABT26779	0	3	3	1	0	0	0
EABT2678	2	0	0	2	5	0	1
EABT26780	0	5	7	1	0	0	0
EABT26781	70	51	16	2	11	76	39
EABT26782	0	0	8	0	0	0	0
EABT26783	13	16	4	10	2	1	2
EABT26784	0	5	1	1	0	0	0
EABT26785	0	0	6	0	0	0	0
EABT26786	0	0	15	1	0	0	0
EABT26787	0	1	8	3	0	0	0
EABT26788	0	0	6	0	0	0	0
EABT26789	2.02	8	38	6	3	12	0
EABT2679	0	2	1	9	0	0	2
EABT26790	8	5	85	33	9	3	1
EABT26791	0	1	1	9	2	1	0
EABT26792	4460.02	3161.68	506.68	688.33	851.76	2681.88	3712.74
EABT26793	0	2	6	2	0	0	0
EABT26794	1	2	42	1	1	0	0
EABT26795	0	0	4	1	0	0	0
EABT26796	2	2	18	4	0	0	0

EABT26797	1	6	10	9	7	0	0
EABT26798	50753.4	76509.4	36195.6	13526	60168.4	4705.19	16873.8
EABT26799	1	0	30	1	0	1	0
EABT268	0	15	13	2	3	0	0
EABT2680	2	19	7	7	0	1	4
EABT26800	2	7	27	13	0	1	5
EABT26801	0	0	8	0	2	0	0
EABT26802	0	4	1	4	1	0	1
EABT26803	5	12	10	51	20	0	3
EABT26804	0	0	2	4	0	0	0
EABT26805	0	0	0	45	0	0	0
EABT26806	10	13.95	13	10	1	1	4
EABT26807	1	3	3	1	1	0	1
EABT26808	0	1	3	0	1	1	0
EABT26809	159	228.51	132.18	40	17	69	68.65
EABT2681	2	2	10	0	1	0	0
EABT26810	2	4	1	5	0	0	1
EABT26811	4	2	21.07	6	1	0	0
EABT26812	3	5	31	2	0	0	0
EABT26813	3	1	2	3	0	1	0
EABT26814	4	9	10	3	0	0	1
EABT26815	2354.17	6373.81	2192.05	34125.2	7264.18	1271.54	5779.09
EABT26816	0	0	5	2	0	0	0
EABT26817	4461.96	4113.51	2328.01	4403.95	2201.73	1984.95	1843.05
EABT26818	5	12	3	5	5	0	53
EABT26819	2	2	7	2	0	1	0
EABT2682	0	2	13	0	1	4	2
EABT26820	0	0	2	2	0	0	1
EABT26821	7	13	9	10	3	1	5
EABT26822	0	12	0	0	0	0	0
EABT26823	0	3	10	4	2	0	1
EABT26824	0	0	4	0	825.7	0	1
EABT26825	1	6	3	1	2	7	1
EABT26826	10446.2	8450.87	7555.49	1959.54	3587.18	11253.6	4710.34
EABT26827	1	0	2	10	0	0	2
EABT26828	408.99	650	366	1233.84	506.02	202	281
EABT26829	5	2	1	0	0	0	4
EABT2683	1	0	6	0	0	0	1
EABT26830	5	16	2	0	4	4	25
EABT26831	1	0	13	3	1	0	1
EABT26832	497.88	1302.29	2907.81	4111.16	3018.73	70.86	545.29
EABT26833	2148.79	2481.69	2008.58	4713.19	1698.62	1771.09	1371.37
EABT26834	0	13	0	0	0	12	1
EABT26835	2	0	4	5	1	0	0
EABT26836	1	1	1	7	0	0	0
EABT26837	11	32	34	87	9	1	1
EABT26838	0	2	9	1	1	0	0
EABT26839	1	3	9	1	1	0	0
EABT2684	4	3	7	16	1	0	1
EABT26840	7	9	28	24	18	5	28
EABT26841	0	0	4	0	0	0	1

EABT26842	0	0	9	3	0	0	0
EABT26843	1	1	2	1	0	0	0
EABT26844	0	3	6.12	4	1	1	1
EABT26845	1	6	18	6	8	19	6
EABT26846	0	4	1	0	0	0	0
EABT26847	2	16	0	0	0	15	0
EABT26848	0	1	16	0	0	0	1
EABT26849	0	0	8	0	0	1	0
EABT2685	1	1	10	0	1	1	0
EABT26850	3	4	8	33	16	2	2
EABT26851	0	0	3	0	0	0	0
EABT26852	0	2	0	0	1	6	1
EABT26853	7464.38	13925.7	10131.4	12772.3	7772.68	1987.97	7640.12
EABT26854	26	16	7	12	2	11	5
EABT26855	1	1	18	9	3	1	2
EABT26856	1	1	7	2	0	0	0
EABT26857	0	1	0	0	2	0	0
EABT26858	1	3	10	1	0	1	4
EABT26859	0	3	2	2	0	0	1
EABT2686	12	11	0	0	0	5	10
EABT26860	1	3	49	6	1	0	3
EABT26861	0	0	7	0	1	0	1
EABT26862	0	1	2	3	0	0	0
EABT26863	0	0	7	0	0	0	0
EABT26864	8	7	32	46	24	1	7
EABT26865	0	4	9	34	0	0	3
EABT26866	268.99	372	232	142	334	321	55
EABT26867	16	24	15	14	6	16	13
EABT26868	2553.8	6892.3	23633.2	44521.1	15116.8	1993.76	2245.97
EABT26869	0	1	4	3	0	2	1
EABT2687	7	19	12	30	1	7	2.46
EABT26870	0	0	6	0	0	0	0
EABT26871	0	0	4	1	0	1	0
EABT26872	0	1	11	20	1	0	1
EABT26873	0	11	17	1	1	3	0
EABT26874	20	62	249	258	46	3	12
EABT26875	1	3	200	16	1	4	2
EABT26876	964.32	1387	940	1586.06	881.01	684.91	690.01
EABT26877	0	0	0	0	5	0	0
EABT26878	0	1	2	3	0	1	0
EABT26879	2	4	10	1	0	0	0
EABT2688	2	8	25	6	2	1	2
EABT26880	0	1	4	1	2	0	0
EABT26881	2	0	0	2	2	2	1
EABT26882	3264.98	4574.34	1629.07	2686.28	1625.41	2583.22	2116.07
EABT26883	4	3	2	0	0	0	0
EABT26884	2	3	15	2	2	0	1
EABT26885	9	1	0	0	1	16	0
EABT26886	64	159	511	176.17	53.22	64.37	91
EABT26887	2	2	2	1	0	0	0
EABT26888	0	6	40	5	1	0	0

EABT26889	0	0	2	0	2	0	0
EABT2689	1	2	0	0	1	5	6
EABT26890	6	7	37	11	1	6	6
EABT26891	1	2	26	1	0	0	0
EABT26892	0	1	12	2	0	0	0
EABT26893	176.26	350	194.02	696.6	231.09	85	170.77
EABT26894	0	1	22	0	0	0	1
EABT26895	108	242	198	882	90	78	66
EABT26896	1	0	15	0	1	5	4
EABT26897	9	5	15	8	2	1	0
EABT26898	450.91	673.87	965.84	1212.78	286	283.99	257.46
EABT26899	0	0	32	5	0	0	0
EABT269	12	15	16.15	3	0	1	1
EABT2690	0	2	6	0	0	0	0
EABT26900	0	15	6	0	0	1	0
EABT26901	255.79	410.33	579.77	982.9	591.46	209.78	216.66
EABT26902	0	0	0	0	10	3	11
EABT26903	0	1	5	21	0	0	0
EABT26904	1	2	11	6	3	1	2
EABT26905	8348	13685.5	12586.9	11973.6	11745.8	5664.96	8135.24
EABT26906	0	2	3	0	1	0	0
EABT26907	17	247.05	238	245	71	8	15
EABT26908	0	0	2	0	0	0	0
EABT26909	232	358	495	652.3	445	171	284
EABT2691	0	0	5	0	0	2	1
EABT26910	0	0	7	0	0	1	0
EABT26911	0	0	0	0	0	0	0
EABT26912	5	11	16	4	15	2	2
EABT26913	0	5	2	0	1	0	0
EABT26914	23	59	41	64	50	9	22
EABT26915	1	2	0	2	1	0	0
EABT26916	5	13	19	2	2	0	0
EABT26917	0	0	7	0	0	0	0
EABT26918	13	22	7	21	17	9	10
EABT26919	11	64	9	61	3	0	0
EABT2692	2111	4841.16	6641.53	14067.7	2209.75	2548.93	775.13
EABT26920	0	1	22	0	0	1	0
EABT26921	1	0	2	2	2	0	3
EABT26922	0	1	4	0	0	1	0
EABT26923	0	2	0	10	1	0	6
EABT26924	1	2	241	0	0	0	2
EABT26925	3038.52	3188.65	1266.68	3804.07	5911.69	1454.1	1476.98
EABT26926	0	0	5	0	0	0	0
EABT26927	2	5	8	15	9	3	4
EABT26928	3	5	5	4	1	1	1
EABT26929	56	27.22	20	152.35	152.02	1	0
EABT2693	43	12	0	0	0	7	1
EABT26930	47	39	42	53.06	101.68	63.04	7
EABT26931	0	0	5	0	2	0	3
EABT26932	52	92	191.95	178	157.34	54.77	116
EABT26933	2776.52	2932.73	1280.94	2911.83	2100.19	3061.96	2343.44

EABT26934	140.73	179.68	159.43	248.57	158.58	111.17	164.03
EABT26935	17	28	63	31	23	20	17
EABT26936	3	10	1	1	9	8	8
EABT26937	1596.3	2688.99	2459.16	6083.37	505.36	1908.83	1641
EABT26938	2	1	2	1	2	0	0
EABT26939	0	0	0	0	0	0	2
EABT2694	4	2	28	0	0	0	0
EABT26940	2	2	16	2	2	2	4
EABT26941	27	54	44	112	61	23	31.03
EABT26942	4	8	13	3	3	0	1
EABT26943	0	0	48	0	0	1	1
EABT26944	4	4	45.41	4	0	0	0
EABT26945	1	0	55	0	2	0	0
EABT26946	0	2	2	1	0	0	0
EABT26947	8	14	11	18	1	2	2
EABT26948	1	6	45	0	0	2	1
EABT26949	8	19	31	161	19	10	8
EABT2695	3	0	2	5	2	0	0
EABT26950	0	0	14	1	0	0	0
EABT26951	1	1	2	8	0	0	0
EABT26952	523	924	533	2496	637	140.93	157.72
EABT26953	2	5	11	52	18	1	0
EABT26954	6	10	0	0	0	2	1
EABT26955	4441.61	4940.36	3528.5	4061.91	4186.68	2303.02	2703.63
EABT26956	0	4	0	3	97	0	23
EABT26957	43.4	262	141	0	26	40	12
EABT26958	0	3	5	9	0	1	0
EABT26959	6	8	14	10	4	3	3
EABT2696	1	4	13	0	0	0	0
EABT26960	5	3	1	1	0	8	0
EABT26961	0	2	1	3	0	1	0
EABT26962	0	3	6	3	1	1	1
EABT26963	0	0	0	0	0	0	0
EABT26964	5	15	19	8	5	1	9
EABT26965	0	2	12	0	0	0	0
EABT26966	1	0	9	0	0	0	0
EABT26967	0	0	1	1	0	0	0
EABT26968	0	3	10	6	10	0	3
EABT26969	0	0	40	0	1	1	0
EABT2697	1	4	8	1	1	0	0
EABT26970	2	5	8	5	0	4	1
EABT26971	1	0	1	0	0	0	0
EABT26972	0	0	0	0	0	5	0
EABT26973	5	3	2	2	3	8	4
EABT26974	0	1	11	3	0	0	0
EABT26975	0	1	22	1	1	1	0
EABT26976	567	697	404	968	447	433	348.87
EABT26977	1	7	10	2	2	0	0
EABT26978	65	207	101	161	271.35	10	72
EABT26979	387	849	1630.24	850	383	225	286.3
EABT2698	24388.5	5518.66	578.62	5031.39	6800.39	328.76	588.32

EABT26980	2	9	8	1	1	0	0
EABT26981	13	9	2	0	1	5	1
EABT26982	25	45	76	83	80	12	28
EABT26983	1	1	4	0	2	0	0
EABT26984	1	5	4	3	4	5	2
EABT26985	3	15	0	0	0	26	1
EABT26986	0	0	62	3	0	0	0
EABT26987	0	3	6	0	0	0	1
EABT26988	0	3	6	0	0	0	0
EABT26989	3	0	0	0	0	19	0
EABT2699	0	0	5	2	0	0	1
EABT26990	0	0	0	0	0	0	1
EABT26991	16	1	0	0	0	15	0
EABT26992	0	2	1	1	0	1	0
EABT26993	23	24	31	45	31	20	29
EABT26994	0	0	11	0	1	0	0
EABT26995	356.76	530.29	491.43	1363.59	505.16	345	409.48
EABT26996	388	1045.02	1873.8	1153.99	521.99	458	414.01
EABT26997	0	4	1	3	1	0	0
EABT26998	21	50	69	3	3	30	27
EABT26999	9685.9	21556	13830.4	21398	10127.8	5587.93	10890.4
EABT27	19	23	10	11	5	2	4
EABT270	985.04	1097	680	2081.43	622	624	449
EABT2700	2	1	0	0	1	5	5
EABT27000	2	5	38	3	0	3	0
EABT27001	544.71	869.96	925.01	1771.33	685	231	384.94
EABT27002	0	0	2	1	0	1	0
EABT27003	28	36	47	5.8	4	50	8
EABT27004	3	5	6	2	2	5	9
EABT27005	1	1	9	2	1	0	0
EABT27006	56	35	9	14	0	103	3
EABT27007	4	11	41	35	2	2	3
EABT27008	10	29.08	240.24	97.93	20	182.75	188
EABT27009	0	3	13	1	0	1	1
EABT2701	770	1314.32	745.05	1663.36	1254.23	169	665.62
EABT27010	5	1	0	0	9	19	27.07
EABT27011	2	1	20	0	0	1	1
EABT27012	32	58	115.01	70	39	25	29
EABT27013	0	0	3	2	0	0	1
EABT27014	1	1	0	2	0	0	1
EABT27015	14	39	65	40	39	20	30
EABT27016	1	1	0	1	0	1	0
EABT27017	0	0	1	4	1	0	0
EABT27018	0	0	0	0	0	0	0
EABT27019	0	1	20	0	0	0	1
EABT2702	3121	3810.39	5609.12	9379.29	2586	2674.79	1563
EABT27020	3	0	6	1	0	2	0
EABT27021	1	2	5	12	0	1	0
EABT27022	0	0	0	8	0	0	0
EABT27023	0	1	1	0	0	0	0
EABT27024	0	0	10	0	0	0	0

EABT27025	734.52	912.21	986.89	974.41	682.73	710.19	521.28
EABT27026	1	0	5	2	0	0	0
EABT27027	349	476	1196.4	1679.5	725.99	356	330
EABT27028	0	0	1	5	1	0	0
EABT27029	2151.69	3059.13	3292.41	5626.63	2352.63	2549.02	2434.83
EABT2703	456	1150.15	1542.21	1967.98	1914.33	263	604.77
EABT27030	1	1	7	6	4	0	0
EABT27031	0	1	8	0	1	1	2
EABT27032	0	1	10	0	0	2	0
EABT27033	1	2	34	1	0	1	4
EABT27034	13253.8	50685.8	49687.8	88185.3	2758	12	17
EABT27035	26	56	73	93	11	12	25
EABT27036	0	0	2	1	5	1	1
EABT27037	9	32	5	8	6	8	7
EABT27038	0	1	6	5	0	0	0
EABT27039	2	1	25	0	0	0	2
EABT2704	0	0	13	0	0	0	0
EABT27040	0	0	3	1	0	0	0
EABT27041	0	1	17	1	0	0	1
EABT27042	0	0	28	0	1	0	1
EABT27043	191	274	261	433	234	128	165
EABT27044	0	0	11	3	0	0	0
EABT27045	459	708	1389.54	5427.49	798.71	10	5
EABT27046	1727	2198.82	5455	9636.12	3098.15	37	42
EABT27047	7	8	15	3	34	0	0
EABT27048	0	2	30	1	1	0	0
EABT27049	0	0	5	0	26	2	5011.91
EABT2705	0	0	8.81	0	0	0	1
EABT27050	3	4	8	12	8	1	0
EABT27051	0	1	8	1	1	2	1
EABT27052	6	29	10	66	3	1	1
EABT27053	7	5	13	14	11	10	28
EABT27054	12	13	8	20	2	10	8
EABT27055	0	0	2	3	2	0	0
EABT27056	2	1	84	330	4	2	1
EABT27057	0	1	0	2	0	2	0
EABT27058	23	58	98	70.35	88.88	11	69.97
EABT27059	44	157	113	42	1107	4	21
EABT2706	1	2	15	0	0	3	0
EABT27060	0	0	18	0	1	0	0
EABT27061	1	2	0	1	0	2	0
EABT27062	5	2	0	0	0	0	0
EABT27063	0	2	0	3	0	1	0
EABT27064	4	4	9	1	206.45	0	1
EABT27065	878	1530.92	1008.56	756	433	1360.01	1247
EABT27066	0	1	4	1	1	0	1
EABT27067	2	2	3	21	2	1	5
EABT27068	1	1	4	8	0	2	0
EABT27069	6	4	1	26	17	0	0
EABT2707	3944.3	6783.37	5987.18	5246.1	2619.21	3800.76	5541.86
EABT27070	0	0	0	0	1	0	0

EABT27071	1	3	9	6	1	2	0
EABT27072	10	12	1	4	1	8	15
EABT27073	0	2	6	1	0	0	0
EABT27074	0	2	9.97	1	2	1	1
EABT27075	3	43	4	2	11	2	10
EABT27076	4	2	17	2	1	2	1
EABT27077	4	0	6	4	0	0	1
EABT27078	0	0	3	1	2	0	0
EABT27079	3	5	12	3	1	0	2
EABT2708	20	1	15	4	133	3	2
EABT27080	0	3	7	15	0	1	1
EABT27081	14	7	11	7	13	1	19
EABT27082	1	3	5	14	5	2	0
EABT27083	1283.14	2455.12	307.04	832.95	3153.68	1262.51	2315.67
EABT27084	3	2	3	4	0	1	0
EABT27085	1	1	12	2	0	0	1
EABT27086	245	251.26	76	1625.02	108	188	115
EABT27087	5	7	12	9	4	4	2
EABT27088	5	6	28	5	2	14	5
EABT27089	0	0	0	3	1	0	0
EABT2709	0	1	3	0	0	1	0
EABT27090	0	2	0	0	0	2	15
EABT27091	7	3	0	0	1	6	4
EABT27092	3	5	13	6	1	3	5
EABT27093	0	0	8	1	0	0	0
EABT27094	1	4	72	0	1	0	4
EABT27095	0	0	8	0	0	0	0
EABT27096	2	5	1	0	0	0	3
EABT27097	9	29	5	9	8	22	5
EABT27098	5839.79	3899.54	31	14	26	1554.55	46
EABT27099	27	48	54	58.92	24	149	27.67
EABT271	5337.53	6624.92	5157.83	3562.11	2642.12	5872.07	4897.37
EABT2710	0	0	15	0	0	0	0
EABT27100	1	1	4	0	0	0	1
EABT27101	9	10	7	39	20	0	15
EABT27102	0	1	3	5	0	3	0
EABT27103	1	7	6	27	1	0	0
EABT27104	15	19	25	7	5	8	3
EABT27105	0	0	14	0	0	0	0
EABT27106	0	1	1	1	2	0	1
EABT27107	16686.8	25553.5	23016.7	51991.4	20412.8	13039.2	15327.3
EABT27108	0	1	13	0	0	0	0
EABT27109	3	4	8	5	3	1	3
EABT2711	591.07	1272.31	1998.82	1617	780.38	521	596.13
EABT27110	1	0	15	2	2	0	0
EABT27111	1473	2171	2787.44	4207.24	1948.42	1330	1255.97
EABT27112	0	0	15	0	0	0	0
EABT27113	5	0	0	0	0	6	10
EABT27114	2	4	5	10	1	1	1
EABT27115	0	0	0	9	1	0	0
EABT27116	6	9	0	0	0	1	0

EABT27117	14545.5	27739.6	30028.4	35783	12215.9	16909.4	16604.7
EABT27118	1	3	3	3	0	1	1
EABT27119	0	2	1	9	3	0	8
EABT2712	351	454	506	895.89	424	252	317
EABT27120	45.94	54.11	12.77	8.19	60.73	133.82	113.77
EABT27121	1673.86	1983.35	1184.76	1120.12	1042.93	986.57	723.85
EABT27122	0	0	3	2	3	0	0
EABT27123	0	8	4	1	0	0	1
EABT27124	3	1	9	7	3	2	10
EABT27125	4	15	14	4	0	0	1
EABT27126	0	4	1	26	5	0	2
EABT27127	2	3	12	4	1	6	1
EABT27128	3	6	6	44	1	0	0
EABT27129	2296	2477	1075	1457.54	6637.09	1438.09	1578.99
EABT2713	8	7	8	14	3	7	4
EABT27130	2	1	8	0	4	0	16
EABT27131	1818.05	2386.57	3149.46	2281.92	1302.29	1896.11	2344.43
EABT27132	0	0	2	0	2	2	0
EABT27133	2	2	2	6	2	0	0
EABT27134	5893.68	8962.53	177.78	85.76	1983.92	8412.45	19501.7
EABT27135	13	2.36	1	0	0	7	0
EABT27136	809.26	1914.11	3609.26	7852.76	720.24	614.02	559.25
EABT27137	1	3	6	5	1	1	0
EABT27138	1	0	7	0	0	0	0
EABT27139	3	1	42	0	0	1	0
EABT2714	7	6	5	8	2	16	11
EABT27140	0	1	8	2	0	0	0
EABT27141	1	2	44	1	1	1	0
EABT27142	2810	4142.84	2170.37	5619.47	2362.42	1407.61	2290.41
EABT27143	9541.54	20104.1	10286	35320	9630.99	234.75	1564.32
EABT27144	2	14	33	8	2	4	3
EABT27145	71	8	7	1	1	25	0
EABT27146	0	2	0	0	5	1	1
EABT27147	0	0	5	5	0	1	0
EABT27148	0	1	17	1	0	0	0
EABT27149	4244.74	16452.2	19	2	1066.5	2099	1312.99
EABT2715	4	0	13	4	0	2	1
EABT27150	16	22	22	49	26	19	13
EABT27151	651.66	868.12	387	1402.32	618.3	350	441
EABT27152	0	0	4	2	0	0	0
EABT27153	3	5	9	0	0	1	1
EABT27154	0	2	9	0	1	1	0
EABT27155	0	0	25	1	1	0	0
EABT27156	13	23	0	0	0	1	1
EABT27157	1	5	23	1	3	0	2
EABT27158	0	3	8	7	0	1	1
EABT27159	4	5	19	8	8	5	1
EABT2716	2	15	22	44	2	0	0
EABT27160	0	0	6	0	1	0	4
EABT27161	1	16	12	289	14	0	3
EABT27162	1	0	2	1	0	1	3

EABT27163	0	0	12	0	0	0	1
EABT27164	1	8	3	5	2	0	0
EABT27165	8	6	19	14	3	18	13
EABT27166	1129.32	2135.24	2210.49	3356.48	1693.68	913.49	1109.89
EABT27167	0	1	14	2	0	2	2
EABT27168	0	2	25	1	1	0	2
EABT27169	0	0	6	0	0	0	0
EABT2717	4	12	26	2	1	2	3
EABT27170	0	4	3	2	0	1	0
EABT27171	3	5	3	6	1	0	1
EABT27172	6	5	10	0	3	0	3
EABT27173	0	1	2	2	0	0	0
EABT27174	0	3	0	1	2	0	0
EABT27175	0	0	2	1	1	0	0
EABT27176	3	8	31	96	34	9	8
EABT27177	1529	1972	208	10	53	5922	82
EABT27178	3	0	8	2	0	10	1
EABT27179	0	4	3	1	0	0	0
EABT2718	1	2	39	3	1	0	0
EABT27180	831.48	1570.71	105	126	109.02	306.04	264.62
EABT27181	17	32	87	62	115	29	49
EABT27182	2	2	4	1	4	18	1
EABT27183	7	17	125	22	18	5	18
EABT27184	1199	1724	3030	3579	1052.97	1070.04	917
EABT27185	0	4	47	0	0	0	1
EABT27186	0	0	0	0	5	1	10
EABT27187	2	16	14	0	1	2	2.06
EABT27188	6	2	9	5	0	0	0
EABT27189	0	0	6	0	0	0	0
EABT2719	5	9	39	8	10	8	12
EABT27190	0	1	1	0	2	0	2
EABT27191	131	353.49	680.52	1814.85	558.46	98.97	191
EABT27192	33	64.08	41.72	65.97	33.61	22	17
EABT27193	555.35	594	631.25	661.42	589.96	553.14	299.13
EABT27194	0	1	5	4	2	0	3
EABT27195	17	19	1092.08	176.7	94.44	82	14
EABT27196	6509.79	11412.2	9685.63	17737.4	7563.73	4245.43	4783.3
EABT27197	3	2	37	28	0	0	0
EABT27198	135	614	1767.43	891	1147.24	155.84	637.99
EABT27199	1	1	16	1	0	0	0
EABT272	0	1	3	0	0	0	1
EABT2720	1	0	9	7	0	0	1
EABT27200	0	2	1	0	0	0	1
EABT27201	2	6	13	3	0	0	0
EABT27202	50	17	135.39	45	11	34	20
EABT27203	1184.14	1881.72	1821.36	784.55	1136	722.92	1605.27
EABT27204	1	2	17	19	2	5	4
EABT27205	1	0	0	7	3	0	0
EABT27206	0	0	16	6	0	1	0
EABT27207	3	7	76	0	0	0	1
EABT27208	1	0	0	0	0	10	3

EABT27209	13	44	213	133	145	14	6
EABT2721	0	0	11	2	2	1	0
EABT27210	0	0	9.93	6	0	4	1
EABT27211	312	425	409	597	485	269	275.85
EABT27212	0	1	3	3	0	0	0
EABT27213	2	1	17	6	2	0	1
EABT27214	16	42	30	74	34	12	15
EABT27215	3	0	9	1	1	2	1
EABT27216	0	0	2	1	0	0	0
EABT27217	5	5	6	8	0	0	1
EABT27218	0	1	15	5	2	1	1
EABT27219	0	0	1	0	1	4	0
EABT2722	1	1	2	0	0	0	0
EABT27220	1200	4287.36	2044.46	5814.24	3774.61	681.23	1108
EABT27221	0	1	4	1	0	0	0
EABT27222	0	0	9	2	1	0	1
EABT27223	0	0	1	1	0	1	1
EABT27224	0	1	2	6	0	0	0
EABT27225	91	145.01	65	41	95	49	64
EABT27226	0	3	0	1	0	0	0
EABT27227	0	0	1	0	0	0	0
EABT27228	0	0	2	0	0	1	1
EABT27229	0	0	6	0	0	0	0
EABT2723	1	2	11	4	2	1	1
EABT27230	7	12	30.18	27	19	1	0
EABT27231	0	0	15	1	0	0	0
EABT27232	0	1	3	1	0	0	0
EABT27233	41	269	455	965	408.97	25	37
EABT27234	0	3	9	3	0	1	0
EABT27235	2	2	3	0	0	2	0
EABT27236	8	2	2	1	1	0	0
EABT27237	0	3	13	6	8	0	1
EABT27238	0	2	7	7	4	3	1
EABT27239	10	10	15	27	7	8	7
EABT2724	4	35	215.08	5	2	3	4
EABT27240	0	0	1	5	1	0	0
EABT27241	2	1	6	0	0	0	0
EABT27242	2	3	46	12	1	0	1
EABT27243	2	2	1	0	0	0	2
EABT27244	0	0	1	0	1	1	0
EABT27245	0	3	3	7	37	0	4
EABT27246	1	13	63	3	1	0	2
EABT27247	0	1	8	2	2	0	1
EABT27248	0	1	2	6	5	0	0
EABT27249	44	40.19	65	120	188	30	40
EABT2725	0	0	14	1	0	1	1
EABT27250	0	13	4	1	0	0	0
EABT27251	1	7	5	14	4	1	0
EABT27252	17839.3	10685.4	1913.31	2082.7	2748.01	25803.3	13577.5
EABT27253	14922.9	38181.9	15274.2	3513.33	2894.88	47	131
EABT27254	632	786.27	1299	1145.98	904	830	1317.92

EABT27255	0	1	9	10	0	0	0
EABT27256	0	1	0	0	4	2	2
EABT27257	1	1	16	5	2	1	2
EABT27258	6	16	43	39	5	2	3
EABT27259	5	3	10	4	2	0	5
EABT2726	1	4	1	2	3	0	7
EABT27260	13	11	34	6	4	14	9
EABT27261	0	0	9	0	0	0	0
EABT27262	2	1	7	1	0	0	1
EABT27263	15401.1	5681.18	1	3	2	16454.7	7
EABT27264	0	0	6	0	0	0	0
EABT27265	5	3	1	0	0	0	0
EABT27266	8	6	0	0	0	0	1
EABT27267	4	2	17	0	1	2	2
EABT27268	12	16	17	0	1	0	3
EABT27269	0	2	31.58	2	0	1	2
EABT2727	1201.4	2337.85	920	66	297	162	553
EABT27270	8	11	63	12	2	8	2
EABT27271	1192.93	2826.69	3427.85	1058.42	1489.05	110	454.04
EABT27272	0	0	11	0	0	0	0
EABT27273	10	9	52	3	2	2	1
EABT27274	4	2	0	0	2	0	0
EABT27275	1	3	4	3	3	2	2
EABT27276	1	0	10	30.13	2	1	3
EABT27277	1	1	2	0	0	0	0
EABT27278	36	4	5	0	2	14	7
EABT27279	1766	1537	1871	678	896	12	50
EABT2728	0	3	7	3	1	0	1
EABT27280	10	3	0	2	1	14	13
EABT27281	0	3	6	2	0	0	1
EABT27282	1	2	4	0	0	1	0
EABT27283	1	11	5	12	7	1	5
EABT27284	385.99	641.94	731.82	799	369	367.61	430.98
EABT27285	0	2	14	2	3	0	0
EABT27286	1	0	204.99	1015	1274.1	4	5
EABT27287	787.37	1372.14	2517.31	2353.58	899.34	892.55	671.03
EABT27288	1	3	8	13	0	0	0
EABT27289	10234.8	21250.8	20385	16786.1	12346.6	6405	10079.8
EABT2729	2	0	0	2	2	1	0
EABT27290	5	10	43	1	11	2	5
EABT27291	0	3	4	0	0	5	0
EABT27292	0	0	1	3	0	0	0
EABT27293	0	1	3	3	0	1	0
EABT27294	2	19	11	106	64	12	15
EABT27295	0	0	3	1	1	0	0
EABT27296	0	0	9	0	0	0	0
EABT27297	0	0	38.12	0	3	1	0
EABT27298	1932.92	859.09	245	855	461	2537	1430
EABT27299	1	7	12	1	2	0	0
EABT273	194.93	315	352.29	860.95	621.97	232.86	293
EABT2730	0	0	7	1	3	3	1

EABT27300	7.76	4	36	2	1	7	1
EABT27301	2	0	10	0	0	1	0
EABT27302	4	3	15	9	2	0	7
EABT27303	1297.53	1882.05	1228.18	1467.55	923.18	857.83	884
EABT27304	32	78	65	8	194	34	1802.74
EABT27305	1	1	4	0	0	0	1
EABT27306	0	0	8	0	0	0	0
EABT27307	1134.25	5089.9	6308.15	422	6524.25	287	4479.08
EABT27308	0	1	3	1	1	0	0
EABT27309	2	3	10	1	1	0	0
EABT2731	217	507.19	785.8	1301.74	503.99	118.99	132.01
EABT27310	0	1	6	1	0	1	0
EABT27311	4803.97	4558.68	2925.18	4957.71	4177.06	4503.76	4802.18
EABT27312	1	3	18	0	0	1	0
EABT27313	145	1277.63	135	570.01	488.12	2	27.22
EABT27314	88.71	192	186.28	1503.9	176	54.85	219.78
EABT27315	0	0	0	8	0	0	0
EABT27316	0	0	3	0	2	0	3
EABT27317	14	16	0	0	0	46.99	23
EABT27318	63	215	1229.32	131	226	43	31
EABT27319	0	0	2	0	0	0	0
EABT2732	22	10	5	2	3	1	0
EABT27320	0	2	4	3	2	0	0
EABT27321	1	4	4	9	1	1	0
EABT27322	0	3	2	1	0	0	1
EABT27323	6003.51	3557.91	464.65	217.02	1753.84	15056.4	10449.1
EABT27324	3897.98	5058.73	4057.07	7239.8	3184.52	3385.72	2844.61
EABT27325	14	26	125	7	35	51.91	25
EABT27326	39.8	80	133.03	117.21	79.63	52.7	65.28
EABT27327	0	0	20	4	1	0	0
EABT27328	10	18	10	90	2	0	2
EABT27329	7	0	1	0	1	2	0
EABT2733	276.01	326	155.24	576.65	324.99	142	278.02
EABT27330	1	1	5	7	3	1	6
EABT27331	1377.99	1735.97	1398	3001.02	2013.92	1651.11	1761.29
EABT27332	0	1	1	5	0	0	0
EABT27333	0	1	2	2	0	0	0
EABT27334	0	1	10	0	0	0	0
EABT27335	1369.63	1970.24	1996.36	2033.18	1342.75	1356.32	1534.67
EABT27336	793.8	906.79	945.57	2190.75	1236.17	700.96	607.41
EABT27337	1	5	1	0	3	10	0
EABT27338	5	11	74	22	6	5	3
EABT27339	6	12	1	12	1	1	1
EABT2734	1	4	12	16	7	0	3
EABT27340	0	0	19	2	0	0	0
EABT27341	3	6	6	7	0	0	0
EABT27342	2235.84	2661.82	1596.14	3124.3	1655.13	1555.85	1480
EABT27343	2	3	7	12	2	1	16
EABT27344	4536.03	8412.98	449	110	6575.9	1265.35	5373.05
EABT27345	12	28	59	37	6	9	8
EABT27346	0	2	4	0	1	1	0

EABT27347	0	0	20	0	0	0	0
EABT27348	268	131.8	585.67	311.5	1065.99	1	9
EABT27349	8	17	2	36	24	0	0
EABT2735	1439.28	1653.24	1836.27	3484.72	1386.37	1388.23	1274.75
EABT27350	0	1	0	0	0	3	2
EABT27351	0	2	4	3	0	1	0
EABT27352	967.91	1680.21	1312.09	798.04	1156	394.08	408.96
EABT27353	0	1	0	0	5	0	8
EABT27354	3	1	7	3	2	1	1
EABT27355	0	3	8	0	0	0	0
EABT27356	1838	2826	1618.11	2916.01	2706	227	1069.01
EABT27357	0	1	3	0	0	0	1
EABT27358	0	1	8	9	3	0	0
EABT27359	8	5	118	63	2	2	0
EABT2736	0	0	9	2	0	1	0
EABT27360	0	0	2	4	0	0	0
EABT27361	2	2	10	1	1	1	1
EABT27362	0	1	1	0	1	0	3
EABT27363	0	0	4	10	1	0	0
EABT27364	2	1	0	0	0	2	5
EABT27365	0	7	24	0	1	0	1
EABT27366	1	1	8	12	1	16	1
EABT27367	29	82	1083.1	237	513	101	136
EABT27368	1	0	6	0	0	0	0
EABT27369	0	1	17	11	0	3	0
EABT2737	1	5	4	1	0	0	0
EABT27370	1	3	15	4	2	1	0
EABT27371	0	4	21	7	2	4	0
EABT27372	1	0	10	3	1	0	1
EABT27373	1	4	5	1	1145.68	0	4
EABT27374	2	0	11	11	0	2	3
EABT27375	3690.93	5587.12	6785.28	8420.77	5143.58	1710.07	2305.46
EABT27376	1	0	2	0	0	0	0
EABT27377	2	3	10	7	1	2	13
EABT27378	19	44	38	9	17	41	12
EABT27379	0	7	16	18	3	1	0
EABT2738	0	0	2	4	0	0	0
EABT27380	0	5	6	1	5	0	2
EABT27381	0	0	30	7	0	0	1
EABT27382	3207.38	3632.98	3536.04	3442.72	1812.01	2849.32	2359.13
EABT27383	5	7	0	0	0	0	0
EABT27384	10683.4	21018.2	35720.5	22644.9	14118.4	7446.43	10947.4
EABT27385	57	71	62.81	150	56	16	28.03
EABT27386	0	1	4	1	0	0	1
EABT27387	1	1	18	2	1	1	0
EABT27388	3	6	1	0	1	2	22
EABT27389	1	2	3	0	0	3	1
EABT2739	3	5	5	2	0	2	3
EABT27390	0	2	1	0	0	0	0
EABT27391	0	11	23	3	7	9	4
EABT27392	1002.71	1182.31	878.01	1820.99	554	679.93	596.14

EABT27393	0	0	4	4	0	0	0
EABT27394	0	7	4	1	4	0	1
EABT27395	0	1	7	1	0	0	0
EABT27396	0	0	6	0	0	0	0
EABT27397	2	3	11.29	3	6	2	1
EABT27398	1	1	8	1	6	2	1
EABT27399	484.71	1139	1527	4283.39	1072	698.61	790.88
EABT274	0	0	2	1	2	0	0
EABT2740	2	1	43	0	0	0	0
EABT27400	0	0	11	0	0	0	0
EABT27401	150732	137340	139540	157865	102141	96051.3	141063
EABT27402	0	0	0	5	1	0	2
EABT27403	876.17	1518.2	1127.11	1487.02	1292.17	823.64	818.95
EABT27404	1	4	4	9	1	0	3
EABT27405	10	3	2	0	7	2	27
EABT27406	5	16	9	16	0	3	5.15
EABT27407	1	2	2	3	0	0	0
EABT27408	0	0	4	0	1	0	1
EABT27409	0	0	8	1	0	0	0
EABT2741	0	0	7	1	0	0	1
EABT27410	0	0	4	1	0	2	2
EABT27411	0	3	3	1	0	0	0
EABT27412	2	2	2	9	2	4	4
EABT27413	0	0	6	0	0	1	0
EABT27414	0	4	3	5	2	1	3
EABT27415	1383.22	2216.45	2309.14	5546.68	4439.06	1144.77	1212.17
EABT27416	2057.6	2860.34	1187	5043.48	1793.71	2751.95	3626
EABT27417	0	1	4	0	0	1	0
EABT27418	0	0	4	1	0	0	0
EABT27419	1	9	58	15	3	1	2
EABT2742	2	4	14	0	0	1	2
EABT27420	8987.07	15111	6452.82	20728.3	5348.28	423	1282
EABT27421	1	1	3	3	0	0	0
EABT27422	4	3	77	2	1	3	0
EABT27423	8	45.02	17	50	4	2	3
EABT27424	822.84	1551.99	1984.03	7463.41	1111.3	524	710
EABT27425	0	4	35	4	0	1	0
EABT27426	1	3	30.2	6	4	3	1
EABT27427	2614.97	3714.29	213	414	1284.85	3906	298.97
EABT27428	1	3	11	11	0	2	1
EABT27429	1	3	14	1	1	0	0
EABT2743	0	0	1	1	1	0	1
EABT27430	3	0	7	4	0	1	0
EABT27431	1578.38	2333.09	1859.77	3456.24	1017	1115	1004.05
EABT27432	1	2	9	6	4	0	1
EABT27433	0	3	9	12	9	9	9
EABT27434	2	1	15	17	38	0	0
EABT27435	1	49	0	2	5	0	2
EABT27436	4	30	207.87	1	0	8	0
EABT27437	3	4	12	2	1	2	0
EABT27438	0	2	7	1	0	0	0

EABT27439	1	0	8	0	2	1	0
EABT2744	0	1	12	6	0	0	0
EABT27440	6	8	9	2	28	8	18
EABT27441	28	93.08	215	14438.1	42	1	8
EABT27442	0	0	10	1	0	0	0
EABT27443	0	2	22	3	0	1	1
EABT27444	5	19	38	3	1	1	0
EABT27445	0	0	6	2	1	8	6
EABT27446	97.36	173	231.2	438	275	43	131
EABT27447	7.95	1	0	0	0	7	0
EABT27448	0	1	9	0	0	0	0
EABT27449	263	354.01	186.95	653.29	367.55	140	284.93
EABT2745	2	4	29	2	1	0	0
EABT27450	1	35	13	0	2	12	7
EABT27451	0	1	5	1	0	1	0
EABT27452	7	4	15	1	1	2	1
EABT27453	0	0	1	1	0	0	0
EABT27454	3	0	2	18	0	0	0
EABT27455	1	4	1	1	3	0	3
EABT27456	3	10	2	7	0	3	0
EABT27457	1	1	4	1	0	0	0
EABT27458	0	0	1	0	0	0	0
EABT27459	0	2	5	2	0	0	0
EABT2746	1	0	0	0	4	0	3
EABT27460	555.02	961.89	1280.52	2001.87	684	892.18	704
EABT27461	1	3	13	0	0	0	0
EABT27462	11	5	31	4	14	5	15
EABT27463	1	2	7	0	1	1	2
EABT27464	0	4	1	1	0	0	1
EABT27465	716	1891	603.18	1037	1488.11	910	3608.44
EABT27466	4	25	9	361	116	1	7
EABT27467	0	2	0	0	2	0	0
EABT27468	6	9	5	0	0	1	0
EABT27469	3	12	32	11	8	0	1
EABT2747	0	0	6	0	0	0	0
EABT27470	0	0	15	0	0	1	0
EABT27471	0	2	6	2	0	0	0
EABT27472	758.99	1117.02	942.04	1427.14	768	518	522
EABT27473	1	0	8	2	0	0	1
EABT27474	0	4	5.99	3	0	0	0
EABT27475	20	8	2	2	0	24	3
EABT27476	0	5	8	1	2	1	1
EABT27477	5	4	8	6	3	4	4
EABT27478	9	31	162	42	15	4	8
EABT27479	0	0	13	0	0	0	0
EABT2748	2	2	2	1	1	1	0
EABT27480	0	0	3	5	0	0	0
EABT27481	1	1	1	5	0	0	0
EABT27482	0	2	21	2	2	0	0
EABT27483	2	9	8	12	0	0	0
EABT27484	1	7	10	2	0	0	2

EABT27485	0	2	0	0	0	1	0
EABT27486	11	7	4	23	24	1	25
EABT27487	2	1	16	4	1	0	1
EABT27488	2	0	5	2	0	0	2
EABT27489	1	2	7	7	2	1	0
EABT2749	0	2	23	13	3	1	0
EABT27490	1	4	3	19	6	1	0
EABT27491	5	8	27	0	0	0	0
EABT27492	14	11	11	5	1	5	2
EABT27493	1	1	81	7	22	0	2
EABT27494	3	4	23	6	0	0	0
EABT27495	375	474.53	385	694.9	383.27	305.09	237.12
EABT27496	1	0	3	0	0	0	0
EABT27497	0	1	6	0	0	0	0
EABT27498	72	87	56	135.97	73	19	52
EABT27499	0	1	8	2	0	1	0
EABT275	18	6	10	7	74	23	57078.6
EABT2750	4	1	31	6	0	1	0
EABT27500	520	703.7	19090.7	595.21	1237.3	4	11
EABT27501	1	1	8	2	2	0	2
EABT27502	0	1	0	0	0	1	1
EABT27503	1261.3	1771.74	2846.26	1845.03	1583.88	53	374.02
EABT27504	0	0	3	0	0	1	1
EABT27505	2	1	21.96	7	2	0	0
EABT27506	27	8	3	1	1	35	6
EABT27507	68	125.03	53	252.55	76	34	72.97
EABT27508	269.99	348.73	1008	1080.68	230.65	301.15	297.92
EABT27509	69	118.88	107.03	369.66	153.34	54	92
EABT2751	0	3	3	1	2	0	0
EABT27510	0	2	2	7	1	0	4
EABT27511	4	6	51	40635.1	107	0	8
EABT27512	122	135	0	0	0	1	1
EABT27513	1	3	2	2	0	1	2
EABT27514	1	6	9	7	9	0	1
EABT27515	1	0	7	0	0	0	0
EABT27516	714	2671.45	1916.01	3438.65	690.62	449.71	952.3
EABT27517	0	5	10	1	0	28	5
EABT27518	19	8	37	20	1	4	8
EABT27519	26	1	0	0	1	3	2
EABT2752	0	5	12	0	1	0	0
EABT27520	0	0	6	1	0	1	0
EABT27521	0	1	2	1	0	0	0
EABT27522	1	1	5	5	1	2	3
EABT27523	0	0	0	0	0	16	4
EABT27524	2	15	13	1	0	1	0
EABT27525	3	7	13	5	0	2	0
EABT27526	1	6	8	7	1	5	8
EABT27527	41	95.03	144	28.97	14	4	11
EABT27528	0	1	19	0	0	0	0
EABT27529	4	1	0	0	2	1	1
EABT2753	151.99	213.01	355.02	786.98	373	69	216.07

EABT27530	1	27	0	0	0	5	0
EABT27531	0	7	29	2	1	0	2
EABT27532	1	2	6	2	2	0	0
EABT27533	2	4	1	1	1	3	0
EABT27534	0	0	8	1	0	0	0
EABT27535	1	5	10	19	28	0	0
EABT27536	0	1	10	1	0	0	0
EABT27537	0	0	4	0	1	0	0
EABT27538	21	24.72	31	124.01	22	4	17
EABT27539	9	49.04	157.5	39	10	0	2
EABT2754	58	64	54	137.07	14.88	27	33
EABT27540	288.02	263.72	256.01	411.25	130	231.99	112
EABT27541	185.63	276.97	437.06	715.94	232	138	145
EABT27542	1	5	6	40	16	4	3
EABT27543	0	0	4	1	0	0	0
EABT27544	4	14	29	25	3	1	8
EABT27545	0	4	8	3	2	1	1
EABT27546	0	0	8	0	0	0	0
EABT27547	8	4	13	9	0	0	15
EABT27548	1	0	1	4	0	0	0
EABT27549	381.95	487.89	962.11	2730.59	1272.29	627.13	728.74
EABT2755	1	3	6	1	1	0	1
EABT27550	1	1	6	11	4	1	0
EABT27551	1	5	2	9	1	0	1
EABT27552	5832.81	4974.06	2390.18	6024.33	2760.84	4105.92	6169.01
EABT27553	3	7	1	5	4	0	0
EABT27554	5	0	8	1	0	11	4
EABT27555	0	0	1	8	1	0	0
EABT27556	4	5	11	1	1	3	9
EABT27557	0	0	8	1	0	0	0
EABT27558	1662.63	2364.8	2266.02	3918.72	1653.75	1253.02	1410.71
EABT27559	0	1	0	2	5	0	0
EABT2756	0	0	0	0	21	0	0
EABT27560	2	9	13	0	6	0	10
EABT27561	0	0	4	0	0	0	1
EABT27562	3	1	1	0	0	2	0
EABT27563	1507.04	1958.71	3642.82	1714.93	1353.88	1300.07	1287.08
EABT27564	1692.53	5058.87	1989.61	3894.17	1733.54	1208.22	2016.63
EABT27565	37	31	62	19	14	3	4
EABT27566	9	9	0	0	0	2	1
EABT27567	4889.71	2584.11	404.44	2088.62	2289.01	643.77	984.33
EABT27568	0	0	11	0	0	0	0
EABT27569	0	0	16	0	1	0	0
EABT2757	0	12.9	14.57	4	4	2	5
EABT27570	1	1	1	2	0	0	0
EABT27571	16	32	226.01	17	12	12	8
EABT27572	5059.5	5519.38	3412.35	2650.23	13864.6	4383.63	3692.35
EABT27573	4	2	15	19	4	3	3
EABT27574	0	0	0	0	1961.29	0	3
EABT27575	1	0	5	0	0	1	0
EABT27576	43	51	2	0	12	0	0

EABT27577	0	1	12	1	0	0	0
EABT27578	378	642	530.01	3848.85	854	158	133
EABT27579	0	1	4	2	0	3	0
EABT2758	1	4	48	7	1	2	0
EABT27580	14	36	25	50.86	15	4	29
EABT27581	2	7	37	0	7	3	24.11
EABT27582	5	5	0	0	0	2	2
EABT27583	0	3	7	3	5	1	1
EABT27584	7	9	0	0	0	0	0
EABT27585	98	110	13	0	2	71	5
EABT27586	0	0	2	4	0	0	0
EABT27587	0	0	0	14	0	0	0
EABT27588	1	1	3	6	1	0	0
EABT27589	1	0	0	0	4	0	0
EABT2759	0	2	4	0	0	0	0
EABT27590	1	2	0	0	0	4	4
EABT27591	2	1	12	9	2	8	3
EABT27592	76.02	256.01	74.01	104.3	30	60	72.94
EABT27593	0	0	7	3	1	2	0
EABT27594	0	1	5	0	6	2	0
EABT27595	1	0	0	4	0	0	1
EABT27596	0	2	11	2	4	0	0
EABT27597	0	5	8	0	1	0	0
EABT27598	1	2	4	1	0	0	1
EABT27599	0	0	2	23	1	0	0
EABT276	17	21	77.99	381	12	5	12
EABT2760	6	6	4	1	1	2	5
EABT27600	1165.02	959.48	39	9	232	2201.67	1266.12
EABT27601	2	4.27	11	2	2	0	2
EABT27602	0	2	0	2	0	1	0
EABT27603	0	2	3	2	0	0	0
EABT27604	0	3	2	2	2	0	0
EABT27605	322	375	233.62	656.68	505.01	298	333.21
EABT27606	0	1	9	2	0	1	2
EABT27607	0	1	1	1	0	0	2
EABT27608	4	10	2	2	6	0	1
EABT27609	2	3	15	7	5	0	1
EABT2761	5	0	0	0	0	15	0
EABT27610	12	16	8	28	2	3	5
EABT27611	1999.18	6802.59	19947.3	5143.49	5824.66	24	124
EABT27612	1	0	16	0	0	0	0
EABT27613	0	1	21	0	0	0	0
EABT27614	0	2	3	0	0	0	1
EABT27615	4249.89	345.96	2	0	68.97	576.3	513.93
EABT27616	4	17	20	8.05	2	0	6
EABT27617	0	0	0	0	2	0	5
EABT27618	1	7	0	0	0	2	3
EABT27619	51	45	18	5	22	19	20
EABT2762	1	0	5	1	1	0	0
EABT27620	3	7	72	3	5	6	2
EABT27621	18937.1	23972.3	6996.77	3687	5495.54	24287.2	17702.9

EABT27622	9	10	28	10	4.41	6	18
EABT27623	2059.19	2858.3	944.69	1226.04	1734.67	3813.1	4840.31
EABT27624	0	0	3	0	1	0	0
EABT27625	3	11	18	197	8	3	18
EABT27626	3	12	9	24	8	1	7
EABT27627	41	160	269	816.97	55.99	126	250
EABT27628	2	3	1	3	1	0	1
EABT27629	13	44	29	49.21	4	34	5
EABT2763	0	0	0	0	0	0	0
EABT27630	0	3	3	0	0	0	0
EABT27631	0	1	5	3	1	1	0
EABT27632	46	113.23	85.89	102	37.97	69	228
EABT27633	6	3	9	6	8	12	3
EABT27634	35	68.01	35	134.81	37	19	29
EABT27635	0	0	0	0	1	0	15
EABT27636	0	11	15	14	7	2	2
EABT27637	2492.39	3628.19	1871.79	19090.2	4439.36	887.95	1610.31
EABT27638	1	7	3	6	3	0	1
EABT27639	0	1	0	2	0	1	1
EABT2764	0	3	1	23	1	1	1
EABT27640	0	0	4	2	0	0	1
EABT27641	232.18	352	119	605	544.06	99	172
EABT27642	0	7	111.13	17	1	2	8
EABT27643	0	0	2	4	0	0	1
EABT27644	0	0	5	0	0	0	0
EABT27645	1	2	4	0	0	0	0
EABT27646	9416	10726.2	7826.02	8372.68	6493.32	9251.14	7743
EABT27647	1	3	97	18	2	0	0
EABT27648	14	40	12	38	4	19	9
EABT27649	0	0	0	6	3	0	0
EABT2765	0	1	0	0	1	0	8
EABT27650	8	20	15	2	0	0	0
EABT27651	0	1	2	1	6	0	0
EABT27652	2	4	1	1	0	1	0
EABT27653	3	6	0	0	0	0	0
EABT27654	0	1	10	3	0	1	1
EABT27655	18	28	6	49	15	1	12
EABT27656	0	3	12	3	0	0	0
EABT27657	4	11	0	0	2	32	20
EABT27658	1	2	5	4	0	1	1
EABT27659	0	6	10	0	0	0	0
EABT2766	215	284.9	213.74	466.95	286.03	159.48	218.58
EABT27660	1	1	13	2	0	0	0
EABT27661	0	0	15	0	0	0	0
EABT27662	66	182	31	118	274	15	526
EABT27663	10	23	54	12	4	0	0
EABT27664	8	3	6	16	0	4	0
EABT27665	1	1	0	3	0	0	0
EABT27666	0	5	7	1	3	1	0
EABT27667	0	0	2	3	1	0	0
EABT27668	0	0	8	0	0	0	0

EABT27669	1	1	5	2	1	1	0
EABT2767	1	0	0	3	0	1	3
EABT27670	2	1	3	18	8.97	0	1
EABT27671	0	4.78	50	3	0	1	0
EABT27672	1	3	4	6	1	11	0
EABT27673	915.94	982.01	672.02	1542.84	863	956	629
EABT27674	0	0	5	0	0	0	0
EABT27675	2	7	26	7	1	1	1
EABT27676	0	1	0	0	0	0	0
EABT27677	0	6	12	0	0	1	0
EABT27678	0	2	6	0	1	0	0
EABT27679	170	284	123.06	291.88	131	84.78	124
EABT2768	0	0	10	1	0	0	0
EABT27680	3	8	0	0	0	1	1
EABT27681	0	0	0	0	0	0	3
EABT27682	3	1	5	0	0	0	0
EABT27683	2	8	1	8	1	1	1
EABT27684	1	1	1	1	0	0	0
EABT27685	4	5	1	7	2	0	2
EABT27686	1	11	8	9	0	0	0
EABT27687	0	42	51	13	296.6	0	1
EABT27688	7	12	24	2	2	0	1
EABT27689	0	0	2	4	3	0	0
EABT2769	0	0	3	0	0	1	0
EABT27690	0	2	7	1	0	1	0
EABT27691	0	2	0	1	0	0	19
EABT27692	0	2	9	12	1	6	1
EABT27693	0	1	1	0	3	0	3
EABT27694	1	2	0	0	0	1	0
EABT27695	0	0	7	1	0	0	0
EABT27696	8737.17	7631.99	1838.92	741.33	2261.95	13684	7913.88
EABT27697	3	12	8	26	2	8	1
EABT27698	0	1	0	8	1	0	0
EABT27699	1	1	9	0	1	12	0
EABT277	68	84	115	175	40	38	72
EABT2770	1	1	23	0	2	0	3
EABT27700	176.12	457	447	282	830.07	120	138
EABT27701	3	10	32	9	3	0	1
EABT27702	2	10	6	3	0	1	1
EABT27703	405.34	714.49	808.83	1819.45	753	368	331.01
EABT27704	23	52	86	24	13	4	3
EABT27705	1	1	15	1	1	0	0
EABT27706	0	2	31	0	0	0	0
EABT27707	0	0	0	0	1	0	0
EABT27708	1948.09	2725.85	861.94	97	170.43	2504.93	915
EABT27709	2813.07	4633.6	8745.15	16818.6	4040.44	781	1162.89
EABT2771	4	7	9	14	8	4	8
EABT27710	0	3	5	3	1	0	0
EABT27711	573	991.92	1184.18	1418	1134.3	398	509
EABT27712	0	0	22	0	0	0	0
EABT27713	13	19	49	20	9	22	16

EABT27714	3	3	3	1	2	1	0
EABT27715	0	2.75	27	3	1	0	1
EABT27716	0	0	2	3	0	0	0
EABT27717	169	305.89	121	55.45	291	140	128
EABT27718	239	401	453	806.01	541	154	240
EABT27719	2	2	9	1	0	0	2
EABT2772	14	62	32	19	31	28	141
EABT27720	0	0	0	0	0	0	1
EABT27721	0	1	6	0	0	0	0
EABT27722	0	1	3	1	1	0	0
EABT27723	0	1	1	2	0	0	0
EABT27724	1846.76	3439.78	5423.4	18433.6	4390.98	2180.32	2410.86
EABT27725	0	7	20	0	1	1	0
EABT27726	1	1	0	1	0	0	2
EABT27727	1	1	13	6	0	3	1
EABT27728	2	8	19	7	7	0	0
EABT27729	82	117	60	97.62	79	21	49
EABT2773	0	2	1	1	0	2	3
EABT27730	0	3	22	9.01	1	0	0
EABT27731	13	13.42	11	11	2	2	6
EABT27732	147	179.04	95	499.99	196	120	135.18
EABT27733	0	0	1	0	35.89	0	0
EABT27734	4	6	15	3	0	2	1
EABT27735	0	2	4	0	4	1	0
EABT27736	3959.21	2602.72	1224.68	3592.61	2592.24	4147.52	2783.05
EABT27737	0	0	5	8	4	0	0
EABT27738	3	1	18	2	1	3	1
EABT27739	6	3	17	0	0	0	0
EABT2774	0	4	3	8	4	0	3
EABT27740	0	0	0	3	4	0	72
EABT27741	0	0	9	0	0	0	0
EABT27742	1	2	23	3	0	3	0
EABT27743	1	0	2	3	0	0	0
EABT27744	85	70	90	23.15	5	7	0
EABT27745	11	27	9	397.01	56	2	22
EABT27746	1	0	0	0	0	0	34
EABT27747	38	59	51	18	14	31	18
EABT27748	0	0	5	3	0	0	0
EABT27749	6	10	44	12	4	4	3
EABT2775	1587.99	2048.26	2008.91	2050.1	1330.1	1343.03	1028.5
EABT27750	5	15	93	22	4	3	5
EABT27751	173	326.47	168	809.88	426.93	138.96	137
EABT27752	0	0	13	3	0	3	0
EABT27753	7	9	9	1	10	0	0
EABT27754	24	79	39	159	114	15	35
EABT27755	6	10	0	0	0	7	0
EABT27756	3	4	6	3	1	1	11
EABT27757	566.2	2051.5	111	58	1914.04	1442.77	7799.82
EABT27758	17.02	23	35.21	6	4	24	14
EABT27759	0	0	8	2	0	0	2
EABT2776	1060.36	1620.55	2069.22	1862.61	1539.24	1527.02	1121.03

EABT27760	0	0	10	0	0	0	0
EABT27761	0	4	2	0	0	0	2
EABT27762	7	6	59	6	2	0	0
EABT27763	0	0	5	1	1	0	0
EABT27764	0	3	5	6	8	0	15
EABT27765	0	3	8	0	0	0	0
EABT27766	3537.08	4725.49	2661.4	5537.79	3128.87	3749.55	3392.74
EABT27767	3810.59	5780.27	3762.69	10263.8	3823.99	3402.82	4155.51
EABT27768	19	2	4	0	0	0	0
EABT27769	283.94	538.87	327.71	1332.94	811.74	161.99	530.93
EABT2777	2	0	12	3	1	0	2
EABT27770	0	1	2	0	1	0	0
EABT27771	0	4	13	4	0	0	0
EABT27772	1	0	3	13	2	1	1
EABT27773	1450.2	1993.24	2527.44	2561.33	1371.35	1245.09	1333.44
EABT27774	1	1	12	1	0	0	0
EABT27775	1	0	2	1	2	0	1
EABT27776	0	1	9	12	0	0	1
EABT27777	0	1	10	0	0	5	2
EABT27778	2	0	0	22	0	2	1
EABT27779	3	0	18	2	1	1	1
EABT2778	2	6	19	2	2	2	2
EABT27780	2	2	0	6	1	0	0
EABT27781	1955.97	2887.04	5180.07	10594.1	2124.43	694.19	696
EABT27782	211	1606.48	285	786.92	688.81	3	50
EABT27783	2	1	3	0	2	0	0
EABT27784	0	0	0	2	2	0	0
EABT27785	0	14	3	6	2	0	0
EABT27786	2583.97	4836.23	7354.03	12173.2	5315.95	3605.27	3534.42
EABT27787	0	0	17	0	0	0	0
EABT27788	1	1	16	3	0	0	0
EABT27789	289	720	3010	3117.8	1127	53	76
EABT2779	2	0	2	19	0	0	2
EABT27790	7	25	53	7	7	0	3
EABT27791	288	354	115	518	372	168	175.54
EABT27792	0	1	5	0	0	0	0
EABT27793	0	3	15	3	1	1	2
EABT27794	0	3	12	0	0	1	0
EABT27795	0	0	15	2	0	0	0
EABT27796	1	0	2	1	0	0	0
EABT27797	0	0	1	5	1	1	0
EABT27798	0	3	3	15	2	0	1
EABT27799	814.85	973.76	626.21	1082.95	513.27	763.32	367.2
EABT278	3	2	5	1	2	1	0
EABT2780	10	61	93.21	769	51	5	37
EABT27800	2	37	10	10	4	11	4
EABT27801	0	1	5	0	0	0	0
EABT27802	0	0	2	3	1	0	2
EABT27803	0	4	1	0	0	0	0
EABT27804	630.32	1080.79	926.88	1799.97	1058.89	392.04	619
EABT27805	126	98	383	668	327	14	27

EABT27806	5	4	24	10	2	0	1
EABT27807	142	352	393.01	399	61	36	54
EABT27808	1	0	2	34	1	0	5
EABT27809	57	75	0	0	5	12	15
EABT2781	4	1.98	2	0	3	21	14
EABT27810	1	1	46	2	2	1	0
EABT27811	217.96	332	305	914.92	421	39	229
EABT27812	0	3	2	3	0	2	0
EABT27813	1	0	4	1	0	0	0
EABT27814	4	32	27	88	20	1	1
EABT27815	1094	1944.28	1449.04	3165.81	661.96	157	451
EABT27816	1	1	2	15	0	0	0
EABT27817	13	30.89	40	21	6	10	19
EABT27818	0	0	0	3	0	0	0
EABT27819	1	5	11	1	3	0	1
EABT2782	0	2	3	0	1	0	0
EABT27820	22	44	41	182	136.58	1	62
EABT27821	0	0	4	0	0	1	0
EABT27822	0	1	2	3	1	0	0
EABT27823	0	2	15	1	8	5	23
EABT27824	4	4	1	8	7	2	12
EABT27825	1926.34	2180.92	609.98	1649	2014.19	964.59	2671.02
EABT27826	0	0	1	0	2	3	1
EABT27827	0	0	7	0	0	0	0
EABT27828	4	2	0	0	2	13	5
EABT27829	8	15	2	1	0	2	3
EABT2783	0	3	1	2	4	0	5
EABT27830	86.31	87.88	39.02	245.84	154.02	89	121
EABT27831	5	6	37.61	5	0	0	0
EABT27832	0	7	1	3	3	2	0
EABT27833	0	0	8	1	0	2	0
EABT27834	1	8	6	7	3	2	3
EABT27835	0	0	4	2	0	0	0
EABT27836	4939.7	5902.98	9747.15	11505.2	2429.14	4029.57	2905.54
EABT27837	3	2	20	2	0	1	4
EABT27838	1496.56	1500.5	1075	1238.09	1003.69	1594.77	1179.03
EABT27839	0	2	3	2	0	0	1
EABT2784	5	1	20	3	1	5	1
EABT27840	0	2	3	1	0	0	0
EABT27841	5	21	22	68	3	0	6
EABT27842	398.05	528.49	357.62	582.68	771.98	464.06	345.24
EABT27843	0	3	22	4	0	1	1
EABT27844	197	370	1851.81	446	584	23	84
EABT27845	13	16	139	37	22	3	18
EABT27846	7478.07	7527.41	815.53	65	50	11	4
EABT27847	0	3	7	2	1	2	1
EABT27848	3	2	1	0	0	3	0
EABT27849	0	1	2	0	0	1	0
EABT2785	0	0	11	0	0	0	0
EABT27850	5	11	10	0	1881.52	2	6
EABT27851	0	0	0	0	0	20	6

EABT27852	1	0	4	0	0	1	0
EABT27853	0	4	4	6	5	0	4
EABT27854	0	0	4	1	0	0	1
EABT27855	1121.8	2705.66	3771.42	3958.93	1081.97	1189.04	981.01
EABT27856	1	1	12	0	0	5	1
EABT27857	2629	1871.6	95.2	87	305	2067.01	1178.1
EABT27858	102.88	117.21	483.79	220.3	652.1	147.36	181.71
EABT27859	2	2	11	1	1	1	0
EABT2786	0	2	1	3	0	0	0
EABT27860	0	0	6	0	0	0	1
EABT27861	9	149	8	4	50	9	22
EABT27862	0	1	7	0	0	0	0
EABT27863	61.75	6	6	2	105	198.48	1496.48
EABT27864	8	17	12	1	0	6	0
EABT27865	0	2	7	4	22	0	0
EABT27866	1	1	6	0	1	0	0
EABT27867	2199.49	2684.12	1248.18	1339.75	1829.84	1158.41	1134.29
EABT27868	0	1	8	0	0	0	0
EABT27869	2	13	19	89	23	2	5
EABT2787	1634.67	860.5	7	1	1	7181.37	3
EABT27870	0	0	0	0	0	0	7
EABT27871	2	9	3	1	3	0	0
EABT27872	2	0	2	1	0	0	1
EABT27873	1	4	3	32	4	4	1
EABT27874	1	2	2	2	0	0	0
EABT27875	2.05	1	5	0	1	6	0
EABT27876	0	6	63	12	2	0	0
EABT27877	0	0	3	0	1	0	2
EABT27878	0	2	3	0	0	11	0
EABT27879	1	4	1	1	0	1	10
EABT2788	0	2	0	20	2	0	0
EABT27880	0	0	0	0	8	0	0
EABT27881	617	1187.54	1576.48	4441.45	841.98	395.99	328
EABT27882	11	37.01	822.87	31	29	13	13
EABT27883	1	1	2	7	2	2	6
EABT27884	418.03	519.99	327.94	850.88	523.28	362.23	414.98
EABT27885	5	18	3	8	2	8	16
EABT27886	0	1	2	0	0	0	0
EABT27887	1	2	2	0	2	17	2
EABT27888	6	12	0	0	0	0	0
EABT27889	2	9	12	4	0	0	9
EABT2789	2495.66	4878.24	4976.2	6840.46	3534.45	3701.63	4552.95
EABT27890	2	16	37	26	9	3	1
EABT27891	0	0	1	28	0	0	0
EABT27892	1	1	2	2	0	1	1
EABT27893	370	658.4	851.99	1097.76	536.62	326.94	397.81
EABT27894	7	13	10	11	7	1	4
EABT27895	0	0	3	1	0	2	0
EABT27896	0	0	7	1	1	0	0
EABT27897	3	5	1	0	0	3	1
EABT27898	2	2	3	12	7	0	2

EABT27899	5	15	201.9	32	36	8	214
EABT279	0	4	31	0	2	0	0
EABT2790	3	1	17	0	0	1	1
EABT27900	4	5	25	16	1	0	1
EABT27901	2	1	27.09	56	1	0	1
EABT27902	0	2	8	8	0	0	0
EABT27903	1501.65	1933.21	1297.84	3707.23	1769.33	980.42	1067.05
EABT27904	5	16	0	0	0	10	8
EABT27905	662.69	940.29	744.11	1866.92	1106.09	694.94	1087.2
EABT27906	3369.53	5806.35	2822.21	7593.58	2859.07	2398.14	3055.71
EABT27907	1344	1682.7	1289.99	2296.95	1011.11	844.13	842.03
EABT27908	0	1	2	1	0	0	1
EABT27909	3	8	9	0	13	0	16
EABT2791	0	5	18	7	1	2	0
EABT27910	1066.88	1458.1	1333.22	2344.19	1527.08	1065.28	1006.77
EABT27911	0	3	3	4	18	2	1
EABT27912	0	0	12	0	0	0	0
EABT27913	1	9	5	28	8	0	0
EABT27914	2	5	24	5	1	0	3
EABT27915	430.96	711	1040	2240.09	1135.25	565	820.95
EABT27916	0	0	1	3	0	0	0
EABT27917	47	57	50	648.99	53	43	69
EABT27918	12	5	7	4	0	0	0
EABT27919	0	0	3	2	0	0	0
EABT2792	10	29	28	8	3	0	1
EABT27920	3602.19	5298.35	7723.63	7411.56	5224.3	3087.39	3319.33
EABT27921	21	38	20	72	10.99	30	21
EABT27922	1	1	6	1	0	1	0
EABT27923	2	5	18	3	3	3	8
EABT27924	0	0	13	0	0	0	0
EABT27925	2	2	4	0	0	0	0
EABT27926	1	2.91	1	2	1	3	1
EABT27927	13040	16761.6	13906.4	19684.9	9749.42	11301	10452.9
EABT27928	0	1	5	1	1	0	0
EABT27929	0	1	9	0	1	0	1
EABT2793	0	0	0	4	0	1	0
EABT27930	0	0	10	2	0	0	0
EABT27931	1340.03	10524.8	28845.6	6550.56	13625.6	328	3581.97
EABT27932	579.68	610.53	449.14	456.6	397.29	706.56	473.23
EABT27933	5	10	52	17	2	0	0
EABT27934	408	679.07	641.23	1483.58	1352.98	161	2628.57
EABT27935	4	32	38	31.1	5	7	8
EABT27936	88	114.92	97	184	92	50	73
EABT27937	3	8	24	10	10	0	10
EABT27938	3	10	22	2	3	4	2
EABT27939	10	2	6	3	2	1	2
EABT2794	0	2	2	1	0	0	0
EABT27940	0	1	5	0	0	0	0
EABT27941	2	3	21	3	0	1	0
EABT27942	1	0	7	22.99	1	0	0
EABT27943	5	8	10	21	3	7	2

EABT27944	0	6	2	21	24	0	50
EABT27945	3	5	6	158	4	0	3
EABT27946	2	4	9	0	3	1	3
EABT27947	21	23	15	49	24	26	151
EABT27948	0	0	5	0	119	4	0
EABT27949	0	1	15	1	1	1	1
EABT2795	0	0	0	0	0	1	4
EABT27950	1	4	6	2	0	0	0
EABT27951	2568.84	4089.34	2682.74	4112.08	2162.99	74	403.15
EABT27952	1926.54	2447.98	2762.6	6907.13	3462.24	2421.43	2084.97
EABT27953	0	1	6	2.31	0	0	0
EABT27954	0	0	5	1	0	0	0
EABT27955	5	1	10	7	5	1	9
EABT27956	508.67	588.02	402	983.14	385	277	336.56
EABT27957	0	0	8	1	0	0	0
EABT27958	0	0	15	0	0	0	0
EABT27959	3	13	1	30	2	2	5
EABT2796	3733.34	13270.8	11735.5	10283.3	4000.81	2678.98	4506.06
EABT27960	1	1	12	0	2	0	1
EABT27961	855.91	1130.38	803.04	1361	954	722	738
EABT27962	1	0	5	0	1	1	0
EABT27963	0	0	1	1	0	0	0
EABT27964	0	1	6	0	0	0	3
EABT27965	3	1	46	5	0	0	0
EABT27966	0	0	9	0	0	0	0
EABT27967	1	1	7	1	0	0	0
EABT27968	0	0	2	0	1	1	0
EABT27969	1	4	0	7	0	0	0
EABT2797	5597.23	7118.51	2010.73	392.07	2346.32	11617.5	12650.1
EABT27970	4	11.01	8	0	0	0	0
EABT27971	0	4	12	13	1	0	3
EABT27972	5	3	3	4	0	1	0
EABT27973	1	1	9	0	0	0	1
EABT27974	1	5	4	20	1	1	0
EABT27975	6	5	5	4	0	10	12
EABT27976	10	14	0	0	4	6	17
EABT27977	1	12	0	0	0	0	0
EABT27978	0	0	14	2	0	1	0
EABT27979	34	19	70	17	35	3	15
EABT2798	3	1	1	2	0	2	2
EABT27980	0	0	3	7	0	0	0
EABT27981	0	1	3	0	0	3	0
EABT27982	7	12	67	71	2	7	4
EABT27983	0	0	8	0	0	0	0
EABT27984	150	208	236	630.01	247	140	78
EABT27985	1	0	3	0	0	1	0
EABT27986	4	2	6	0	1	0	0
EABT27987	1	2	0	0	1	0	0
EABT27988	0	4	4	4	1	1	0
EABT27989	1	1	0	1	0	4	0
EABT2799	0	1	9	1	2	10	2

EABT27990	0	1	4	0	1	0	0
EABT27991	3495.96	8725.39	7449.72	11415.3	4552.86	1486.92	3287.01
EABT27992	0	1	2	2	0	1	1
EABT27993	773	1236	726.59	1860.15	980.38	816	818
EABT27994	0	0	5	2	0	0	0
EABT27995	1556.55	1938.72	889.99	1959.6	425.02	1116.75	877.93
EABT27996	0	0	2	3	0	1	0
EABT27997	0	2	1	3	1	0	0
EABT27998	1	0	2	1	0	0	0
EABT27999	0	2	6	1	0	0	0
EABT28	2	3	4	0	3	0	1
EABT280	5	1	0	0	2	3	1
EABT2800	5	25	72	86	2	5	14
EABT28000	0	2	4	1	1	0	1
EABT28001	1	0	16	7	3	0	0
EABT28002	1	0	6	1	0	0	0
EABT28003	1	2	4	0	1	0	2
EABT28004	3	0	5	1	0	11	6
EABT28005	0	2	5	2	0	3	0
EABT28006	1	0	0	4	3	0	0
EABT28007	2	7	3	15	3	0	2
EABT28008	267	566	1634	2343.25	1443	47	147
EABT28009	15	16	16	26	34	6	7
EABT2801	87	184	81	267.26	125	3	53
EABT28010	1	0	5	6	1	2	1
EABT28011	0	0	9	0	0	2	2
EABT28012	4	6	2	1	6	2	2
EABT28013	1883	2475.79	2132	3761.99	1948.41	1317	1517.72
EABT28014	5	23	5	12.06	5	14	27
EABT28015	0	1	6	0	0	0	0
EABT28016	9	1	1	0	4	18	14
EABT28017	1	1	14	2	2	0	0
EABT28018	528.24	883.3	687.21	1193.26	600.17	376	434.8
EABT28019	5	2	45	11	2	0	0
EABT2802	0	5	23	41	4	1	3
EABT28020	1	0	6	2	0	0	0
EABT28021	1	0	18	0	0	0	0
EABT28022	0	2	0	3	1	0	0
EABT28023	4	2	0	0	0	0	1
EABT28024	5	13.75	30.01	8	12	5	2
EABT28025	42	82	34	50	118	13	254
EABT28026	1	2	22	2	1	0	1
EABT28027	1915.96	876	898	1117	857	979.53	1431.07
EABT28028	9026.15	15283.1	21073.2	16310.5	8506.8	6818.94	9414.68
EABT28029	0	0	11	0	0	0	0
EABT2803	2	7	21	8	1	2	0
EABT28030	1	3	19	1	1	0	1
EABT28031	1	4	31	7	1	0	1
EABT28032	0	2	3	1	0	0	2
EABT28033	13	3	0	0	0	19	3
EABT28034	1	2	5	3	0	1	0

EABT28035	1	2	7	0	0	2	0
EABT28036	0	0	3	0	0	0	1
EABT28037	40	43	101	148	5	1	0
EABT28038	0	1	17	1	2	1	0
EABT28039	0	0	7	49	0	0	0
EABT2804	8	12	21	32	1	7	4
EABT28040	12	12	0	0	4	31	23
EABT28041	1	1	18	0	0	0	0
EABT28042	0	2	2	1	0	0	0
EABT28043	368	480.69	1702.82	602.01	336	3561.84	281
EABT28044	3197.49	6250.84	2631.26	3085.08	4142.64	1834.12	7970.15
EABT28045	1	3	3	0	0	0	0
EABT28046	461.63	794.29	466.24	1512.8	416.5	218.95	400.41
EABT28047	27	34	0	0	0	0	7
EABT28048	14	42	85	55.95	5	5	1
EABT28049	2	1	3	8	34	1	7
EABT2805	18	38	82	13	6	1	2
EABT28050	515	940	351	738	643	244	409.63
EABT28051	2	4	1	1	3	0	3
EABT28052	0	0	0	0	1	0	7
EABT28053	1623	809	2405.96	26	1074	671	16922.9
EABT28054	871	1271.99	1344	2513.11	1642.05	414	371.02
EABT28055	3	1	6	4	0	0	0
EABT28056	12	18	51	31	3	12	3
EABT28057	0	0	6	3	2	0	2
EABT28058	925.18	1270.75	555.91	1271.08	1088.74	606.71	986.81
EABT28059	12	29	32	22	6	2	6
EABT2806	1	0	0	4	0	1	0
EABT28060	644.75	918.71	489.8	858.86	784.2	791.97	1234.98
EABT28061	2	1	26	1	2	5	5
EABT28062	885.07	2017.25	2791.73	2525.98	1435.98	1047.88	1080.92
EABT28063	0	0	4	3	0	0	0
EABT28064	3	2	4	0	6	1	0
EABT28065	19	14	21	25	17	2	19
EABT28066	0	1	5	1	0	0	0
EABT28067	1	1	5	6	0	1	0
EABT28068	1	0	11	2	1	2	0
EABT28069	3	1	72	0	2	1	2
EABT2807	0	0	4	1	2	0	4
EABT28070	0	4	9	13	7	0	1
EABT28071	0	0	18	0	0	0	0
EABT28072	1	1	0	3	1	0	0
EABT28073	1478.94	2282.95	5330.52	5492.3	2199.01	1869.58	2462.39
EABT28074	9235.61	5975.34	1872.31	6683.56	2523.02	10252.2	6356.6
EABT28075	0	103	82	43	49	0	0
EABT28076	126.41	342.3	206.02	122	72	52	123
EABT28077	0	0	0	0	0	0	0
EABT28078	5	1	35	2	0	15	2
EABT28079	1824.35	2585.45	1857	4482.03	1421.32	1700.85	1456.24
EABT2808	6	10	26	16.99	17	11	10
EABT28080	4	4	1	8	0	0	1

EABT28081	0	1	41	3	0	1	0
EABT28082	18	44	114	250.84	70	8	31
EABT28083	111.26	325	493.02	1196	322.97	75	155
EABT28084	0	0	4	0	0	0	0
EABT28085	0	0	4	2	1	1	0
EABT28086	0	0	8	0	1	0	0
EABT28087	0	1	0	8	0	0	0
EABT28088	6	9	12	5	0	1	1
EABT28089	4	1	1	1	1	2	2
EABT2809	832	2214.98	1432.93	582	800.52	505	645.01
EABT28090	6.56	7	22	22	8	7	6
EABT28091	8	6	16	3	16	0	1
EABT28092	4	6	90	5	2	3	4
EABT28093	2	1	5	4	1	0	0
EABT28094	0	0	4	1	1	0	0
EABT28095	0	4	9	6	2	0	4
EABT28096	0	3	58	2	1	0	0
EABT28097	3	3	1	4	1	1	1
EABT28098	0	1	6	5	0	0	2
EABT28099	1	3	4	2	1	0	0
EABT281	3	9	12	20	3	5	5
EABT2810	0	6	1	6	1	0	4
EABT28100	0	0	0	8	0	0	0
EABT28101	0	0	3	7	0	3	0
EABT28102	1	3	0	0	2	1	3
EABT28103	5	7	8	16	5	8	1
EABT28104	5	9	21	3	5	0	3
EABT28105	0	0	6	4	4	0	2
EABT28106	0	0	4.83	2	0	1	0
EABT28107	4	15	7	20	3	4	2
EABT28108	0	0	0	3	2	0	2
EABT28109	1	1	6	0	1	0	2
EABT2811	2	4	1	1	0	0	1
EABT28110	0	0	3	0	0	0	0
EABT28111	1	3	8	117	11	1	5
EABT28112	2	4	14	2	0	4	4
EABT28113	2	8	8	8	1	1	1
EABT28114	0	3	7	0	0	0	0
EABT28115	1755.97	2821.76	1602.26	6890.63	2081.53	1222.34	1608
EABT28116	1	1	1	14	0	0	0
EABT28117	1	1	1	2	3	0	0
EABT28118	0	0	13	1	0	0	2
EABT28119	1	10	0	0	0	0	2
EABT2812	414	757	33	0	0	80	1
EABT28120	6286.24	9004.58	12810.7	9110.31	4546.31	6446.81	4830.1
EABT28121	0	4	1	1	0	3	2
EABT28122	0	0	1	1	0	0	2
EABT28123	10	7	1	0	0	10	0
EABT28124	2	1	5	0	0	1	0
EABT28125	5	18	5	21	329.79	2	20
EABT28126	9	15	2	2	1	22	8

EABT28127	0	1	3	3	0	1	0
EABT28128	0	0	9	2	0	0	0
EABT28129	0	0	3	10	0	0	0
EABT2813	2	3	2	0	0	0	1
EABT28130	0	1	2	2	27	0	3
EABT28131	0	1	2	0	0	13	0
EABT28132	0	1	16	0	0	0	0
EABT28133	5427.38	5440.03	1922	2325.35	14812	3159.44	3070.72
EABT28134	167.95	158.25	170	2442.95	375.24	28	113
EABT28135	633	817	357	916	721	474	456
EABT28136	3	4	44	3	3	4	1
EABT28137	1	6	2	1	0	1	1
EABT28138	2	5	12	7	3	1	9
EABT28139	2382.76	3058	266	234	474.14	1534.78	1290.6
EABT2814	44	64	83	70	39	26	40
EABT28140	12	6	0	0	4	9	12
EABT28141	6	15	4	2	8	1	8
EABT28142	1	5	0	0	0	2	1
EABT28143	301.43	431.97	337.47	553	291	251.02	349
EABT28144	0	1	7	2	0	0	1
EABT28145	3	3	14	51	24	4	16
EABT28146	0	1	3	2	1	3	1
EABT28147	19	29	9	28	14	0	4
EABT28148	0	0	6	0	0	0	0
EABT28149	0	13	211	8	0	7	3
EABT2815	442	543	5	86	146.68	70	266
EABT28150	2	1	3	0	3	0	0
EABT28151	0	1	30	1	8	1	0
EABT28152	1	2	1	1	2	0	0
EABT28153	2	1	1	3	1	0	3
EABT28154	10	15	9	19	37	5	9
EABT28155	0	2	9	0	0	1	0
EABT28156	1603.86	3808.37	2411.99	7171.64	1937.94	1957.4	1890.59
EABT28157	3	17	0	0	0	9	0
EABT28158	6	5	11	28	3	0	1
EABT28159	5	3	3	1	1	0	1
EABT2816	1	2	10	0	0	0	1
EABT28160	1	2	1	0	2	0	3
EABT28161	1261.64	1358.34	2826.59	2516.67	1156.71	839.61	679.15
EABT28162	13	30.99	69	46	5	10	13.1
EABT28163	6427.05	18971.3	11473.1	13207.5	3747.33	3044.19	3596.36
EABT28164	1	0	12	2	0	2	0
EABT28165	6	7	8	27	20	1	2
EABT28166	0	0	5	5	0	0	0
EABT28167	970.58	4709.75	528.5	9246.01	686.1	271.65	601.95
EABT28168	2	5	20	1	1	1	1
EABT28169	0	0	2	1	0	1	1
EABT2817	2	1	24	7	1	1	0
EABT28170	4	18	264	1026.28	6	76	15
EABT28171	4	5	15	3	5	6	1
EABT28172	550.92	768.16	874.24	2081.84	796.88	430.31	480.77

EABT28173	0	4	6	7	2	2	1
EABT28174	21	20	17	629.22	45	10	19
EABT28175	0	2	6	3	1	0	0
EABT28176	1	16	12	1	1	0	0
EABT28177	6	9	36	29	12	4	15
EABT28178	1079.46	1611.56	1862.61	3390.82	1233.74	1024.32	961.16
EABT28179	2	2	3	4	2	1	1
EABT2818	22202.2	23549.9	12820.3	17905.8	19046.9	22860.4	23298.8
EABT28180	11	21	158	25	3	10	3.97
EABT28181	0	1	3	2	2	0	0
EABT28182	1	1	3	6	0	1	0
EABT28183	4	23	115	68	2	0	1
EABT28184	167	520.01	1083.63	3300.72	476.87	29.01	131.02
EABT28185	4	4	7	8	4	1	6
EABT28186	3	3	3	11	1	4	2
EABT28187	1	1	6	1	0	0	0
EABT28188	2	7	13	3	2	3	5
EABT28189	10	14	90	41	18	8	11
EABT2819	0	0	13	0	0	0	0
EABT28190	0	0	12	0	0	0	0
EABT28191	109	189	42	195	199	59	77
EABT28192	912.48	420.52	378.59	501.18	249.1	220.09	235.04
EABT28193	1528.33	2314.19	2639.8	5999.9	1143.53	1247.86	917.59
EABT28194	43	286	1654.02	3096.84	46	2	9
EABT28195	0	3	0	0	0	0	0
EABT28196	28	43	12	18	9	24	8
EABT28197	23	9	0	0	1	7	0
EABT28198	2854.37	3438.57	3854.93	5027.22	1869.05	4081.96	3026.81
EABT28199	1	0	19	0	0	0	0
EABT282	1	6	4	0	4	47	7
EABT2820	0	7	0	5	2	0	4
EABT28200	15	26	0	0	1	2	3
EABT28201	1455.11	2393.43	2991.7	5407.77	1301.61	1162.63	1098.06
EABT28202	0	0	1	1	1	0	0
EABT28203	364.07	422.05	429	703.05	320.84	304.13	290.64
EABT28204	1	0	6	6	0	1	0
EABT28205	7	7	9	6	5	11	3
EABT28206	2971.15	2871.86	1278.01	15	45	2	70
EABT28207	1	0	5	0	1	0	1
EABT28208	7	5	49	13	6	5	6
EABT28209	1	1	3	0	0	0	0
EABT2821	5	9	6	18	3	2	2
EABT28210	2	3	5	2	1	0	2
EABT28211	4	8	15	9	0	2	0
EABT28212	1	1	3	0	0	0	0
EABT28213	0	0	8	4	2	1	0
EABT28214	1	0	13	2	2	0	2
EABT28215	0	2	4	0	0	1	1
EABT28216	0	1	7	3	2	0	0
EABT28217	2	2	1	0	1	0	0
EABT28218	2	0	17	0	1	2	0

EABT28219	650.07	1091.43	1718.91	2884.14	572.64	594.7	445.13
EABT2822	263	1025	927	12	49	0	0
EABT28220	7	17	55	31	17	14	11
EABT28221	1	3	5	7	0	1	2
EABT28222	0	0	17	1	0	0	0
EABT28223	1249	1766.88	3220.95	3334	1407.01	1195	777.53
EABT28224	753.22	715.04	481.1	91	512.19	1648.67	1819.02
EABT28225	0	13	1	1	0	1	0
EABT28226	0	5	1	0	0	0	0
EABT28227	10	10	0	0	3	33	25
EABT28228	12	17	58	9	3	9	5
EABT28229	6089.92	12035	17953.2	11509.2	9194.49	6121.81	7783.95
EABT2823	0	2	16	2	0	3	0
EABT28230	0	1	7	1	0	1	0
EABT28231	3	10	5	1	0	1	0
EABT28232	675.01	1133.14	590.62	1195.96	459	626.37	688.42
EABT28233	1	1	3	0	0	0	0
EABT28234	4	11	0	1	0	0	0
EABT28235	1	2	1	0	4	1	22
EABT28236	459	615	294	480	505.01	268.37	336
EABT28237	0	2	1	1	0	0	1
EABT28238	4	9	9	15	9	4	3
EABT28239	10.99	40	5	8	6	0	0
EABT2824	994.72	1797.38	1951.98	2326.41	1198.61	641.29	831.27
EABT28240	1586.01	2704.58	3212.37	8721.33	2022	1226.88	1622
EABT28241	0	1	1	0	0	3	5
EABT28242	571.07	1048.31	856.02	2419.08	742.53	308	439
EABT28243	2	0	5	1	0	0	0
EABT28244	5	2	6	0	5	0	4
EABT28245	0	1	12	1	0	0	1
EABT28246	5	12	21	14	1	0	13
EABT28247	1	1	1	2	1	0	1
EABT28248	2	6	3	0	1	0	1
EABT28249	1	3	2	1	0	0	0
EABT2825	2	6	13	9	7	0	9
EABT28250	0	0	6	2	0	0	0
EABT28251	0	1	3	0	0	0	0
EABT28252	0	1	7	3	0	1	0
EABT28253	0	0	6	3	0	1	0
EABT28254	2923.23	3972	930	519.99	1235.86	5687.9	3472.97
EABT28255	0	1	1	1	2	0	1
EABT28256	0	1	5	2	0	1	2
EABT28257	69.01	132	144.89	154	70	83.18	77.99
EABT28258	2	7.63	1	5	0	0	0
EABT28259	5841.6	10923	8646.71	3706.94	4186.22	2364.62	2923.02
EABT2826	4922.87	6582.98	5179.26	7999.27	3190.1	4043.11	3392.34
EABT28260	2	2	12	4	2	8	1
EABT28261	2	0	1	2	1	0	1
EABT28262	0	2	3	1	1	0	0
EABT28263	0	0	5	1	0	0	0
EABT28264	0	0	2	1	0	1	0

EABT28265	0	1	4	0	0	0	0
EABT28266	1	1	3	11	0	0	0
EABT28267	1	0	2	3	0	0	3
EABT28268	4	11	33	19	5	11	6
EABT28269	0	3	13	8	2	0	0
EABT2827	0	0	2	4	1	0	0
EABT28270	13	42	38.45	276	11	7	6
EABT28271	0	1	53	134	0	1	0
EABT28272	0	1	2	3	0	0	0
EABT28273	3	3	31	3	2	0	0
EABT28274	633.64	2179	371	42	324	10	9
EABT28275	0	1	5	2	0	0	0
EABT28276	1	1	10	2	0	0	0
EABT28277	0	0	1	1	1	1	0
EABT28278	3	9	16	50	3	4	5
EABT28279	0	1	5	4	0	1	0
EABT2828	15	10	0	1	1	1	1
EABT28280	0	0	6	0	1	2	11
EABT28281	2	2	3.87	1	2	0	0
EABT28282	4170.98	5577.22	3389.07	1991	1652.82	2426.85	1638.01
EABT28283	5	1	1	0	2	2	2
EABT28284	1	0	22	2	1	1	0
EABT28285	1	3	7	6	3.43	4	20
EABT28286	0	1	0	11	0	0	0
EABT28287	2912.49	2776.58	1158.56	2259.05	3239.54	1316.04	1454.7
EABT28288	0	12	14	7	2	3	3
EABT28289	0	0	12	0	0	0	0
EABT2829	2216.7	3018.9	1089.82	1642.08	1144.11	2078.63	1916.4
EABT28290	0	0	5	0	0	0	0
EABT28291	0	1	24.98	0	4	0	0
EABT28292	0	0	1	4	0	0	0
EABT28293	13	2	0	0	0	16	0
EABT28294	8	24	33	118.29	15	8	6
EABT28295	0	3	8	3	0	0	0
EABT28296	3	0	0	0	0	6	1
EABT28297	1	0	0	10	1	1	2
EABT28298	2	1	104	83	12	0	5
EABT28299	1	0	3	0	2	0	0
EABT283	2	3	2	1	0	0	0
EABT2830	4	3	17	10	0	7	5
EABT28300	1035.91	2089.63	1542.92	539.98	978.14	58	214.85
EABT28301	1	2	3	2	0	1	0
EABT28302	3	3	9	2	0	0	0
EABT28303	0	3	5	7	1	0	1
EABT28304	1310.94	2245.52	2053.36	4129.23	1101.66	2116.85	2265.25
EABT28305	5	8	3	1	0	5	3
EABT28306	25	92	120	87	8	22.03	25
EABT28307	16	26	0	0	1	10	16
EABT28308	239	169	24	119	73	184	22.11
EABT28309	5	33	91.07	371.49	53	2	19
EABT2831	0	1	5	2	0	2	0

EABT28310	3887.5	7424.52	6845.66	11309.8	4389.23	1497.99	3341.22
EABT28311	14673.7	11897	7404	4776.15	6795.91	17779	14474.6
EABT28312	160.96	238.04	192.89	536.17	282.95	129.07	188.99
EABT28313	3	2	11	5	2	1	1
EABT28314	0	1	0	1	2	0	0
EABT28315	2	3	63	4	2	5	1
EABT28316	1	2	2	11	5	0	3
EABT28317	1	4	1	0	0	0	0
EABT28318	1	22	4	14	1	2	0
EABT28319	1	1	22	0	0	0	1
EABT2832	1	2	3	0	0	0	0
EABT28320	0	2	10	2	1	0	3
EABT28321	3224.49	3609.02	1154	2344	15601.4	2353	3454.28
EABT28322	2	2	12	1	1	0	0
EABT28323	2595.2	4248.58	2464.48	1044	1723.99	5256.9	9356.43
EABT28324	20	60	33	2	20	1	11
EABT28325	0	0	18	0	0	0	0
EABT28326	2	0	7	18	0	0	0
EABT28327	0	0	22	2	0	0	0
EABT28328	0	2	13	0	0	0	0
EABT28329	1	9	40	26	5	0	2
EABT2833	3	9.97	9	10	5	2	1
EABT28330	2	2	15	3	1	1	2
EABT28331	548.31	765.19	1052.06	5321.71	806.17	588.04	725
EABT28332	1196.19	1225.59	320	737	608.04	1369.78	1482.91
EABT28333	40	61	70	90	74	14	35
EABT28334	0	1	5	0	0	0	0
EABT28335	1	1	8	0	0	3	2
EABT28336	181	278	241	373	161	87	89
EABT28337	3	2	56.69	9	2	3	3
EABT28338	145.02	7470.04	259.69	19	242.25	1829.16	1378.56
EABT28339	0	0	3	5	0	0	0
EABT2834	0	0	1	0	0	0	0
EABT28340	1	2	8	1	0	0	0
EABT28341	0	2	8	3	0	0	0
EABT28342	11	16	4	0	0	0	0
EABT28343	1	1	2	0	0	0	1
EABT28344	892.94	1356.85	1502.9	3901.8	1129.34	338.12	754.11
EABT28345	0	2	1	6	0	0	0
EABT28346	1	6	2	1	2	2	2
EABT28347	835.02	966.01	878	1778.03	846.99	728.99	593.01
EABT28348	105.31	7	5	2	1	125	0
EABT28349	1	1	8	0	0	0	0
EABT2835	1	3	4	0	0	0	0
EABT28350	736.49	694.17	764.58	1642.92	1327.47	483.26	804.43
EABT28351	427	389	139	201	1135.12	22.97	134
EABT28352	0	0	4	0	0	0	0
EABT28353	222.65	401.13	245.18	479.7	276.65	105.15	391.95
EABT28354	2	1	6	9	2	0	0
EABT28355	0	0	12	0	2	0	0
EABT28356	0	0	10	4	1	0	3

EABT28357	0	0	0	0	0	0	0
EABT28358	1	3	2	2	0	0	1
EABT28359	240	345.84	547	1085	487.98	165	210
EABT2836	7	36.86	12	47	4	14	18
EABT28360	19	58	159.97	284	93.03	25	19
EABT28361	6061.27	10230.8	15145.8	8763.98	4657.74	7746.81	4284.02
EABT28362	0	6	11	0	0	0	0
EABT28363	3	2	4	7	3	10	7
EABT28364	457.07	652.41	1537	1458.6	802.17	425.85	510.27
EABT28365	0	1	1	2	0	0	0
EABT28366	2342.71	3041.97	1571.27	671.77	2394.66	2933.36	1674.06
EABT28367	24	33	121	20	39	42	8
EABT28368	4	10	1	1	2	2	0
EABT28369	1	1	2	0	0	2	0
EABT2837	1	0	12	1	0	0	0
EABT28370	0	0	3	1	0	0	3
EABT28371	0	1	0	3	1	1	0
EABT28372	1	1	10	0	5	0	1
EABT28373	0	0	2	0	1	39	0
EABT28374	0	0	17	0	0	0	0
EABT28375	0	1	3.24	2	0	0	1
EABT28376	0	0	11	0	0	0	1
EABT28377	0	0	6	4	0	0	1
EABT28378	1059	1940.54	3264.89	7169.83	2526.32	1008.76	1016.14
EABT28379	5	20	33.99	167.02	38	5	6
EABT2838	0	0	10	0	0	0	0
EABT28380	1	0	8	0	0	0	0
EABT28381	0	0	2	4	2	0	0
EABT28382	8641.89	11442.4	7011.5	5822.31	3918.27	23999.1	26916.1
EABT28383	8	2	19	1	1	8	0
EABT28384	3.95	22.74	32.63	132.49	144.35	3.46	7.42
EABT28385	6	7	15	167	37	1	4
EABT28386	0	21	127.65	34	6	0	0
EABT28387	0	0	5	8	0	1	1
EABT28388	13	29	20	21	4	4	4
EABT28389	0	0	6	0	0	0	0
EABT2839	2	10	478.72	28	925.81	1	1
EABT28390	1350	1556.18	1306.01	2048	849	1006	837
EABT28391	11	9	11	1	0	2	0
EABT28392	3	2	4	0	0	1	1
EABT28393	1	1	1	2	1	10	11
EABT28394	980.61	1041.02	453	1181.99	1053	743	580.97
EABT28395	0	2	8	0	0	1	1
EABT28396	1042.01	2168.14	3762.49	9305.57	1265.99	568	1143.91
EABT28397	2	0	11	52	1	7	0
EABT28398	0	1	2	1	0	0	1
EABT28399	0	0	8	2	0	0	0
EABT284	6657.19	15613.2	17984.9	16038.1	9515.73	4336.84	8586.52
EABT2840	1089	1901.33	1956.11	4659.28	1255	1167	1094.99
EABT28400	4	13	18	4	0	0	1
EABT28401	6	13	37	14	6	0	1

EABT28402	6	15	10	0	2	6	6
EABT28403	0	1	1	2	0	0	0
EABT28404	0	3	22	18	0	0	0
EABT28405	1	1	3	0	1	2	0
EABT28406	0	0	11	1	0	0	0
EABT28407	1	4	18	12	5	0	3
EABT28408	0	2	47	1	4	2	2
EABT28409	20	67.3	105	342	19	7	16
EABT2841	125	166	168.19	469	181	44	103
EABT28410	0	1	3	2	1	0	1
EABT28411	3	13	24	2	2	0	0
EABT28412	16	6	12	5	5	4	3
EABT28413	0	1	3	0	0	2	0
EABT28414	2050.83	4495.97	5940.67	17694.2	4514.58	307	860
EABT28415	0	6	2	3	1	0	0
EABT28416	0	2	8	1	0	0	0
EABT28417	2	1	14	2	0	2	4
EABT28418	2440.67	3938.12	4220.4	5326.99	3446.53	2859.13	3341.97
EABT28419	7	7	2	0	1	6	7
EABT2842	8	13	46	58	28	0	66
EABT28420	0	1	4	0	0	0	0
EABT28421	0	1	3	0	0	0	1
EABT28422	0	0	22	1	0	0	0
EABT28423	0	1	5	2	0	1	0
EABT28424	1224.46	1815.41	2584.13	3901.69	1733.44	2008.37	1897.04
EABT28425	10049	31205.8	23089.8	40717.8	6309.34	4	147
EABT28426	4	2	13	3	1	0	1
EABT28427	9921.57	13453.7	46163.8	41752.4	10121	12308.7	9758.85
EABT28428	1	1	2	9	5	0	1
EABT28429	0	0	1	0	2	0	0
EABT2843	255.81	424.85	287.23	754.22	379.13	329	536.01
EABT28430	3637.71	3124.3	1870.08	2627.55	1654.69	3886.76	2247.11
EABT28431	1	10	22	62	2	0	1
EABT28432	2	11	68	0	1	4	8
EABT28433	8645.29	8206.62	1771	2565.87	2315.03	8944.23	6352.93
EABT28434	0	1	7	0	0	1	0
EABT28435	1	4	4	1	0	0	0
EABT28436	605.98	829.41	480	1301.71	993.23	719.86	680.32
EABT28437	2	10.66	7	16	2	4	1
EABT28438	73	132	62	246	120	15	60
EABT28439	5006.45	4365.85	719	2263.13	2938.88	1070	1016.01
EABT2844	3	6	10	10	1	1	35
EABT28440	421.02	863.24	1342.07	1564	970.69	408	648
EABT28441	1295	1706.81	1504	2351.03	942.52	1232.8	1116.31
EABT28442	8	4	50	7	2	3	0
EABT28443	1	3	13	10	1	19	1
EABT28444	0	1	0	2	1	0	0
EABT28445	0	3	13	8	0	2	0
EABT28446	1	2	37	2	0	4	0
EABT28447	3129.87	5045.07	16742.2	22546	4037.55	1351.45	2044.58
EABT28448	1	0	3	0	0	0	0

EABT28449	2	1	1	0	0	6	0
EABT2845	621.96	1029.12	1046.62	2370.6	945.98	390.94	472.99
EABT28450	4	5	16	0	0	17	8
EABT28451	0	0	7	1	0	1	1
EABT28452	0	0	7	3	1	0	0
EABT28453	0	0	1	2	2	0	1
EABT28454	16	45	78	531	633	1	11
EABT28455	194	233	99	863.16	361	8	41
EABT28456	21	22	53	11	5	0	4
EABT28457	1	0	3	0	0	0	0
EABT28458	11	6	3	8	1	17	6
EABT28459	0	1	1	11	0	0	0
EABT2846	1	29	13	590.52	101	3	7
EABT28460	12006	21769.6	11465.5	4445.54	5528.93	6843.78	8507.82
EABT28461	520.8	608.9	924	1683.19	688.61	532.06	574.27
EABT28462	1	1	6	5	0	0	1
EABT28463	2	2	4	0	0	0	0
EABT28464	0	0	7	0	0	0	1
EABT28465	2561.05	5537.74	3592.92	9593.99	1962.88	1509.44	1923.01
EABT28466	14	14	3	0	0	0	0
EABT28467	10	4	1	2	3	14	12
EABT28468	1	7	165	3	0	1	1
EABT28469	0	1	7	2	3	0	0
EABT2847	3	4	13	0	1	2	0
EABT28470	1	0	20	1	1	0	0
EABT28471	6	11	0	0	0	0	0
EABT28472	2	21	0	1	0	0	0
EABT28473	449	465	164	544	587	324	279
EABT28474	290.91	876.73	504.12	2526.84	827	171	295.11
EABT28475	0	2	5	5	1	0	0
EABT28476	1	1	3	8	2	1	0
EABT28477	5	3	1	0	0	0	0
EABT28478	2	1	2	10	2	0	3
EABT28479	0	1	1	2	2	2	0
EABT2848	2308.12	3343.52	4424.05	2124.5	3176.07	1447.22	2004.5
EABT28480	0	2	2	0	0	0	1
EABT28481	0	1	2	2	0	0	0
EABT28482	124	330.98	1853.03	671.81	189	159	191.03
EABT28483	7	5	4	3	2	2	1
EABT28484	1	1	0	0	0	1	1
EABT28485	3	1	0	0	0	17	1
EABT28486	1	1	1	0	1	0	0
EABT28487	523.2	644.89	254	658.09	416.17	296	236
EABT28488	4	4	0	3	0	3	2
EABT28489	8	8	1	17	0	0	0
EABT2849	13	18	0	0	3	16	10
EABT28490	0	0	6	0	0	0	1
EABT28491	0	2	15	3	0	0	0
EABT28492	0	1	96	1	2	0	0
EABT28493	0	2	5	10	3	0	0
EABT28494	291.91	630.18	483.8	1837.18	425	203.03	262

EABT28495	0	1	15	1	0	0	0
EABT28496	0	0	2	0	1	1	0
EABT28497	605.28	859.63	1423.86	1575.45	1105.12	663.68	556.93
EABT28498	4	3	11	4	5	0	2
EABT28499	0	1	5	1	3	3	3
EABT285	2	0	2	1	5	4	7
EABT2850	869.98	920.62	475.01	1372	762	866	692.17
EABT28500	5	3	1	0	0	0	0
EABT28501	3	18	51	3	9	0	2
EABT28502	0	7	0	0	0	1	0
EABT28503	22	42	29	31	184	32	41
EABT28504	1	0	51	2	0	1	1
EABT28505	0	5	5	3	4	1	2
EABT28506	26	56.03	54	53	20	19	34
EABT28507	2	21	20	3	3	1	1
EABT28508	0	2	3	53	25	1	0
EABT28509	1	0	0	7	1	0	1
EABT2851	0	1	1	6	0	0	0
EABT28510	0	0	0	1	0	0	5
EABT28511	0	4	10	0	2	0	1
EABT28512	0	0	3	1	0	1	0
EABT28513	0	1	8	0	0	0	1
EABT28514	5	8	8	2	3	4	4
EABT28515	123	293.21	243.15	468.39	85.84	100	127
EABT28516	2556.2	3528.13	1826.41	3385.46	2726.21	1999.3	2623.11
EABT28517	1727.46	2367.33	2492.95	2666.03	2229.48	1661.61	1434.56
EABT28518	0	0	0	9	0	0	0
EABT28519	0	0	3	0	2	0	1
EABT2852	8	6	0	0	1	0	2
EABT28520	0	3	7	5	0	1	1
EABT28521	3169.94	4950.31	3537.01	7442.85	2723.99	3727.6	2632.52
EABT28522	587	955.12	48	30	182	367	1354.61
EABT28523	1265.07	1711.49	1421.71	2632.12	1089.71	395.99	584.05
EABT28524	6	7	8	7	4	16	11
EABT28525	2	2	0	0	0	1	1
EABT28526	4464.66	9977.77	8820.67	2184.32	2042.67	94.27	591.97
EABT28527	499	985	927.31	2497	882.95	464.97	379
EABT28528	0	1	6	0	0	0	0
EABT28529	3	13	62.98	14	4	5	4
EABT2853	1	3	4	0	1	1	0
EABT28530	0	0	7	1	0	0	0
EABT28531	195	180	88	364	182.99	59	55
EABT28532	1	14	17	133	11	0	0
EABT28533	0	0	0	2	0	0	6
EABT28534	5	17	5	0	8	3	38
EABT28535	1	0	3	0	0	1	0
EABT28536	0	0	16	0	1	1	0
EABT28537	720	1215.06	3264.18	1488	926	588	691
EABT28538	0	0	0	1	1	0	0
EABT28539	1	16	11	11	0	4	3
EABT2854	1	0	12	1	0	1	0

EABT28540	4	6	55.18	26.98	0	1	0
EABT28541	1	4	1	7	7	4	2
EABT28542	18	16	10	6	91.52	1	3
EABT28543	1001.4	1545.27	1487.04	2147.6	1254.04	1313.08	856.67
EABT28544	3	3	1	276	341260	119	909
EABT28545	3	1	12	1	1	0	3
EABT28546	0	1	7	3	1	0	3
EABT28547	2	2	1	2	0	0	0
EABT28548	3299.12	4219.18	637	200	1269	4029.15	5183.53
EABT28549	0	0	3	0	0	2	1
EABT2855	1	9	23	0	1	3	0
EABT28550	1	2	4	4	5	0	8
EABT28551	1	6	11	2	1	1	1
EABT28552	1	1	18	0	0	0	0
EABT28553	0	1	11	0	0	0	0
EABT28554	744.76	1216.2	1155.98	2539	922.67	747.03	742
EABT28555	0	0	0	0	0	0	0
EABT28556	0	1	5	0	17	0	0
EABT28557	2	4	4	0	0	0	0
EABT28558	1	2	0	0	1	1	2
EABT28559	7	6	24	2	1	3	4
EABT2856	5	8	10	2	0	0	0
EABT28560	335.83	30	4	455.79	314.72	46	487.29
EABT28561	0	0	2	0	2	0	0
EABT28562	0	1	9	0	0	0	0
EABT28563	0	6	6	1	3	0	0
EABT28564	0	4	8	2	0	1	3
EABT28565	3	8.97	58	14	5	0	1
EABT28566	1	0	0	16	0	0	0
EABT28567	0	1	0	5	0	0	0
EABT28568	60	159.01	265.88	113	267	102	73.02
EABT28569	1	3	3	0	0	1	0
EABT2857	0	0	11	1	0	1	0
EABT28570	37	44	10	3	0	186	17
EABT28571	55	574	697.62	3411.73	578	314	542.08
EABT28572	1	0	82.94	4	0	3	4
EABT28573	1	4	2	0	2	0	0
EABT28574	59.6	6	0	3	0	146	0
EABT28575	16	96	42	20	12	79	41
EABT28576	7	8	14	6	0	2	0
EABT28577	27	47	306.02	89	35	12	36.15
EABT28578	5	4	4	15	2	6	3
EABT28579	0	2	13	4	10	1	7
EABT2858	2	3	20	0	1	2	2
EABT28580	2	9	4	18	7	2	1
EABT28581	0	0	4	7	0	0	0
EABT28582	3	3	10	1	7	0	14
EABT28583	2	9	24	17	5	10	3
EABT28584	1	1	2	0	0	0	0
EABT28585	0	1	43	15	1	0	1
EABT28586	0	1	0	0	0	0	0

EABT28587	0	3	21	1	0	0	0
EABT28588	0	0	1	1	8	0	10
EABT28589	2	2	1	9	1	0	1
EABT2859	5	1	12	1	4	0	4
EABT28590	818.73	1393.8	1644.31	1727.27	724.9	661	605.3
EABT28591	14215.2	16168.2	959	2303	35	3717.93	38
EABT28592	1	0	0	0	1	0	10
EABT28593	0	0	6	2	0	0	0
EABT28594	5942.9	11731	17599.2	21418	21933.7	5062.26	8094.71
EABT28595	14	22	15	59	4471.38	2	46
EABT28596	722.01	1407.48	1487.04	4643.59	1245.49	870.43	1193.03
EABT28597	3	2	5	4	5	1	2
EABT28598	3	3	3	5	1	0	0
EABT28599	0	2	16	2	0	1	2
EABT286	4	6	6	2	1	1	3
EABT2860	0	3	45	95	8	0	0
EABT28600	3	3	27	0	0	0	0
EABT28601	8	4	27	7	1	0	0
EABT28602	1	14	104	18	0	1	0
EABT28603	5	10	14	8	1	8	8
EABT28604	1	2	2	9	2	0	1
EABT28605	0	0	0	7	0	1	0
EABT28606	4	3	0	0	2	0	0
EABT28607	3	0	10	0	2	0	0
EABT28608	0	0	4	0	0	0	0
EABT28609	0	0	0	22	0	0	1
EABT2861	2	3	30	5	1	1	6
EABT28610	4	3	2	1	1	5	0
EABT28611	0	0	1	14	1	0	0
EABT28612	2	3	2	4	1	0	2
EABT28613	0	8	12	11	0	1	2
EABT28614	21	24	1	3	1	21	5
EABT28615	0	2	5	6	2	7	0
EABT28616	0	3	0	0	0	1	0
EABT28617	1	4	8.75	0	1	1	1
EABT28618	9	27	46	16	22	18	16
EABT28619	965	1336.28	837	1855.97	919.96	631.45	759
EABT2862	4	2	11	2	2	2	1
EABT28620	1	0	0	1	2.16	0	9
EABT28621	4	3	3	0	0	14	5
EABT28622	0	2	6	6	1	0	0
EABT28623	2	3	9	0	2	1	3
EABT28624	0	1	0	1	0	0	1
EABT28625	0	0	20	0	0	0	0
EABT28626	0	0	7	0	1	0	1
EABT28627	0	0	0	7	0	0	1
EABT28628	367	598.98	982	1749.53	829	164	259.54
EABT28629	1	0	26	1	1	0	0
EABT2863	11	38	20	42	11	8	20
EABT28630	0	1	15	14	0	0	0
EABT28631	1	0	2	1	0	1	0

EABT28632	4	13	23.98	123.99	9	3	1
EABT28633	36	36.74	8	22	25.6	245.34	107.58
EABT28634	1	2	0	0	0	1	0
EABT28635	1428.41	1410.49	1892.95	774.06	778.63	79	209.84
EABT28636	1	0	1	6	7	0	0
EABT28637	3	7	4	1	1	10	13
EABT28638	1	2	2	5	0	1	1
EABT28639	0	1	9	0	0	0	0
EABT2864	2	6	0	4	0	1	1
EABT28640	1	0	15	0	0	0	0
EABT28641	0	0	5	0	0	0	0
EABT28642	1	1	7	8	1	0	0
EABT28643	1	2	1	2	4	0	8
EABT28644	0	1	4	7	0	0	0
EABT28645	30	57	20	30	28	34	0
EABT28646	8	8	5	18	20492.2	13	53
EABT28647	1	4	0	13	3	0	1
EABT28648	0	0	8	1	0	0	0
EABT28649	197	325	547	922.98	458	172	151.63
EABT2865	1	18	0	0	0	0	0
EABT28650	0	3	34	2	4	0	0
EABT28651	5	7	3	6	4	0	1
EABT28652	76	102	122	328.22	121	54.99	67
EABT28653	0	3	3	0	0	0	0
EABT28654	1	2	1	1	0	0	1
EABT28655	0	0	11	0	0	0	1
EABT28656	1	1	16	2	2	1	0
EABT28657	0	2	0	4	1	0	0
EABT28658	1	6	67	2	0	0	1
EABT28659	10	2	8	0	0	8	0
EABT2866	0	1	5	0	0	0	1
EABT28660	0	0	4	0	0	0	1
EABT28661	8	6	2	0	2	27	3
EABT28662	2	4	27	2	2	0	1
EABT28663	1	1	1	1	0	1	0
EABT28664	2	6	6	3	0	2	3
EABT28665	1	1	5	0	2	0	3
EABT28666	1	8	26	7	0	4	3
EABT28667	0	1	6	0	0	1	0
EABT28668	0	0	1	16	1	0	0
EABT28669	2	0	12	2	0	0	0
EABT2867	2260.53	770.84	508	133	371	8	102
EABT28670	702.41	1725	2009.97	1373	477	1722.99	1997.9
EABT28671	3	3	17	4	0	6	2
EABT28672	2	3	6	4	0	1	0
EABT28673	5	20	29	6	0	0	0
EABT28674	4015.32	5661.95	6281.43	10259.3	3374.38	2877.09	3079.29
EABT28675	13	9	0	0	1	1	1
EABT28676	135	192	238	101.43	117	64	93.09
EABT28677	1	0	2	0	2	0	3
EABT28678	4518.03	8915.15	8362.98	4584.15	3775.97	2205.9	1957.34

EABT28679	0	0	3	0	0	0	0
EABT2868	21	71.99	45	15	3	8	2
EABT28680	0	3	5	0	0	0	0
EABT28681	4788.46	5198.63	3782.64	4060	1896	3428.73	2526.79
EABT28682	1	0	359	2	0	0	0
EABT28683	910.01	1296.69	691.99	1521.79	1182.28	703.86	552.74
EABT28684	10	8	34	13	5	3	5
EABT28685	1	2	2	1	1	1	0
EABT28686	2	0	1	7	1	1	0
EABT28687	1	1	24	3	0	1	1
EABT28688	1	0	12	5	0	1	0
EABT28689	0	2	114	2	0	0	0
EABT2869	164.04	208.54	309.51	556.92	133	2	4
EABT28690	0	1	7	2	0	0	1
EABT28691	2	8	11	9	4	1	5
EABT28692	1735.41	1998.25	3048.83	3802.26	1761.43	1868.32	1566.42
EABT28693	0	5	3	1	3	0	1
EABT28694	14	28	22	41	8	9	36
EABT28695	0	0	1	0	2	0	0
EABT28696	0	2	5	0	1	0	0
EABT28697	1033	1115.73	824.43	901	927.99	1225	886.12
EABT28698	3	0	3	0	0	0	0
EABT28699	5	9	10	11	6	6	5
EABT287	20	15	54	33	1	0	2
EABT2870	0	0	1	0	0	1	0
EABT28700	2	3	12	2	1	1	1
EABT28701	247	241.03	277.98	961.99	292	92	90
EABT28702	1	2	4	0	0	0	0
EABT28703	1	1	53	1	0	0	0
EABT28704	2	14	210	9	9	0	7
EABT28705	0	17.78	0	6	9.62	0	0
EABT28706	0	5	72	21	1	1	0
EABT28707	0	1	2	2	0	0	3
EABT28708	0	2	8	0	0	2	4
EABT28709	0	0	3	1	6	0	1
EABT2871	0	3	2	0	0	1	0
EABT28710	0	1	5	0	0	0	0
EABT28711	0	1	4	3	1	1	3
EABT28712	2	0	12	3	0	1	0
EABT28713	1	3	0	0	2	0	0
EABT28714	323	1085.61	2708.34	885.96	802.55	111	313
EABT28715	0	1	3	0	0	0	0
EABT28716	0	5	38	3	0	2	0
EABT28717	0	1	36	0	0	0	0
EABT28718	180.99	407.06	193	2476.2	1081	90	145
EABT28719	14	0	1	0	0	16	0
EABT2872	0	0	3	50	0	1	2
EABT28720	20	29	0	0	3	59	38.22
EABT28721	0	0	0	3	0	0	0
EABT28722	7	9	14	16	2	5	1
EABT28723	0	0	0	11	0	0	1

EABT28724	1	1	13	1	1	5	0
EABT28725	79	74	17	13	24	49	258
EABT28726	0	0	2	0	0	37	4
EABT28727	0	2	23	0	2	3	2
EABT28728	0	0	3	6	0	0	0
EABT28729	3	4	45	4	0	1	1
EABT2873	8	30	92	53	2	1	1
EABT28730	0	0	152	1	0	0	0
EABT28731	497.48	628	310	1037.61	479	429	414
EABT28732	132	142	742	219	87	67	47.01
EABT28733	0	1	3	0	0	1	0
EABT28734	0	0	9	0	0	0	1
EABT28735	0	1	7	15	5	2	1
EABT28736	7	1	1	2	0	0	1
EABT28737	6	5	25	3	2	2	5
EABT28738	0	1	5	3	5	0	0
EABT28739	1	0	1	0	0	0	0
EABT2874	7	9	13	20	2	6	1
EABT28740	0	0	2	1	0	0	0
EABT28741	271.94	514	145.08	163	432	244	364
EABT28742	16	0	1	0	0	1	2
EABT28743	0	0	4	0	1	0	0
EABT28744	30	16	0	6	1	40	2
EABT28745	6	11	22	67	11	0	0
EABT28746	11	14	16	0	3	1	3
EABT28747	10	1	16	3	32	10	3
EABT28748	6	4	2	0	0	14	2
EABT28749	0	2	3	10	1	0	0
EABT2875	188	343.66	404	389.77	216.83	115	233
EABT28750	0	0	3	11	1	0	0
EABT28751	0	1	24	0	1	0	0
EABT28752	2	2	12	2	0	1	3
EABT28753	1	2	2	8	3	0	0
EABT28754	38	44	20	2	6	19	11
EABT28755	0	1	2	7	0	0	0
EABT28756	1044	801	509	625	180	21	92
EABT28757	575.51	797	298.13	1023.02	1109.29	332	438.01
EABT28758	5	2	4	7	17	1	2
EABT28759	791.74	983.87	696	1468.06	758.91	583.87	505
EABT2876	713.01	1457.04	659	1367.03	854.03	253	476
EABT28760	0	4	3	1	0	0	1
EABT28761	1	0	12	1	0	1	0
EABT28762	6	29	60	275	4	0	3
EABT28763	0	0	0	3	0	0	393
EABT28764	0	0	0	8	0	0	0
EABT28765	2	5	0	0	0	0	0
EABT28766	17	9	33	3.94	9	2	2
EABT28767	0	0	2	0	1	0	0
EABT28768	14	34	112	62	38	28	16
EABT28769	0	1	2	1	1	2	0
EABT2877	1056	1511.03	580.86	500	785.96	853.77	525.29

EABT28770	1	2	1	0	4	0	0
EABT28771	25433.9	50226.1	28241.8	50159.5	19095.9	29171	29498.5
EABT28772	960	1583.59	1756.96	3797.03	1020.1	635.63	614.05
EABT28773	0	1	0	2	0	2	1
EABT28774	97	170.34	2	1	0	1	0
EABT28775	0	1	3	0	0	0	0
EABT28776	0	3	2	3	0	0	1
EABT28777	0	0	3	0	0	0	1
EABT28778	6286.91	8747.27	2717.17	19144.6	9326.57	834.24	594.06
EABT28779	0	19	23	68	13	2	4
EABT2878	0	4	1	3	4	1	2
EABT28780	1787.44	2469.85	2704.4	3378.32	1593.33	1345.19	1293.64
EABT28781	0	2	2	2	2	0	0
EABT28782	1638.91	2356.8	1006.26	2288.58	2547.69	1367.91	1955.04
EABT28783	4711.17	7246.45	13628.6	9792.49	4752.69	4106.03	4453.15
EABT28784	0	0	1	0	1	4	1
EABT28785	9	19	46	24	4	10	1
EABT28786	67	166	4188.97	109	376.66	59	449.98
EABT28787	38	67.01	47.13	7	0	0	0
EABT28788	0	3	1	0	0	1	0
EABT28789	1	2	9	4	0	1	1
EABT2879	0	0	5	0	1	0	0
EABT28790	1	6	3	1	0	0	0
EABT28791	0	5	2	9	0	3	1
EABT28792	33	36	28	56	8	9	6
EABT28793	218	352.8	145	406	88	33	28
EABT28794	6.02	195	28	6	28	0	6
EABT28795	9	8	80.93	31	2	0	2
EABT28796	2	5	6	0	2	6	4
EABT28797	5	2	24	1	1	1	1
EABT28798	1	6	30	10	0	4	4
EABT28799	1	4	17	1	0	0	0
EABT288	0	0	7	0	0	0	0
EABT2880	0	5	0	1	1	0	1
EABT28800	7	9	81	2	0	7	6
EABT28801	1	4	24	3	0	1	1
EABT28802	0	0	3	1	0	5	0
EABT28803	0	0	0	5	0	0	1
EABT28804	0	1	2	0	0	0	0
EABT28805	0	0	11	26	0	1	0
EABT28806	3	1	1	0	3	0	0
EABT28807	6	19	13.98	33	10	9	13
EABT28808	0	1	6	0	0	3	1
EABT28809	0	2	0	1	12	0	0
EABT2881	31	26.05	103.8	48	12	9	2
EABT28810	1	1	1.03	4	2	1	2
EABT28811	92	168	273.94	267	221	76	57.26
EABT28812	0	0	0	0	5	0	0
EABT28813	6	16	39	6	1	8	2
EABT28814	2963.29	4202.45	3210.46	3852.23	3647.65	2363	2561.6
EABT28815	272	372.31	331	522	316.58	167	203

EABT28816	2536.03	4709.12	2036.98	4352.42	3078.78	1468.88	1850.28
EABT28817	2283	3214	2300.79	3235	2329.39	1423.3	1827.66
EABT28818	0	0	6	1	1	0	0
EABT28819	1	1	3	3	1	0	0
EABT2882	1148.04	3660.04	8037.4	2737.83	2371.67	657.95	1514.25
EABT28820	1	1	3	10	1	1	0
EABT28821	1	3	23	0	0	0	0
EABT28822	2963.24	7851.4	3846.63	3488.11	2897.5	2830.45	2043.66
EABT28823	0	1	4	0	0	0	1
EABT28824	1	2	20	1	0	1	0
EABT28825	1	2	7	0	0	0	0
EABT28826	2	6	9	7	1	2	2
EABT28827	0	3	5	0	0	0	0
EABT28828	5	6	7	24	5	2	6
EABT28829	1	0	6	0	2	0	1
EABT2883	72.01	119	19	76.95	35	260.99	143.99
EABT28830	0	2	1	3	2	0	0
EABT28831	1	0	19	0	1	0	0
EABT28832	0	0	7	0	0	0	0
EABT28833	1	0	0	2	0	0	0
EABT28834	1705.42	2557.37	3572.86	9683.12	1352.34	1534.96	2101.05
EABT28835	0	0	3	5	0	0	0
EABT28836	0	0	0	0	0	0	0
EABT28837	0	0	7	3	0	1	0
EABT28838	0	1	12	1	0	0	0
EABT28839	0	3	3	3	0	2	1
EABT2884	2	1	0	4	4	0	3
EABT28840	0	0	8	0	0	0	0
EABT28841	0	0	0	0	1	25	8
EABT28842	0	0	3	2	1	2	0
EABT28843	1	2	9	6	1	4	0
EABT28844	25	35	1	0	2	13	11
EABT28845	2882.79	3691.9	1396.56	2026.77	2980.1	1577.84	1330.55
EABT28846	2	1	9	5	4	0	1
EABT28847	993.95	1750.05	1239.69	711	6350.9	137	67.33
EABT28848	0	0	22	6	1	0	1
EABT28849	1	4	8	5	1	3	4
EABT2885	0	0	28	1	0	0	1
EABT28850	1	0	0	0	4	0	9
EABT28851	1	1	10	14	1	0	1
EABT28852	0	0	11	0	0	1	0
EABT28853	2	9	21	18	2	1	6
EABT28854	0	0	0	0	1	0	0
EABT28855	128939	51360.8	283.23	23.51	379.08	47332.4	1956.61
EABT28856	0	1	17	0	0	0	0
EABT28857	0	1	9	1	0	1	0
EABT28858	4137.83	2488	267	1485	783	1	28.02
EABT28859	3	17	25	13	12	13	15
EABT2886	12	21	120	113	0	3	1
EABT28860	164.91	342.24	357	1783.89	409.03	92.98	189.11
EABT28861	0	1	0	0	0	11	3

EABT28862	333	648	637	1121.01	2119.36	378	507.01
EABT28863	1	22	0	0	1	1	1
EABT28864	4	14	15	27	12	0	3
EABT28865	0	1	8	0	0	1	0
EABT28866	1	3	2	4	0	2	0
EABT28867	1	2	2	10	1	0	16
EABT28868	7	8	5	2	0	0	2
EABT28869	121.07	233	330	1271	272	81	42.01
EABT2887	0	0	3	0	0	0	0
EABT28870	1	2	39	1	0	4	1
EABT28871	0	0	9	1	0	0	0
EABT28872	2213.09	3193.96	1197.1	3293.88	2006.47	616.1	2327.13
EABT28873	7	14	27	2	2	4	2
EABT28874	0	16	1	1	0	6	0
EABT28875	6	11	12	30	3	7.91	4
EABT28876	125.23	750.01	70.61	1152.16	78.63	85.09	61.71
EABT28877	0	1	31	0	0	2	6
EABT28878	1	0	0	0	0	18	28
EABT28879	0	4	6	0	2	0	5
EABT2888	0	0	0	0	0	0	0
EABT28880	0	0	5	2	0	0	0
EABT28881	2	4	10	5	0	2	1
EABT28882	9	40	102.23	43	24	2	0
EABT28883	151	247	1509.78	277	212	279	97
EABT28884	0	0	0	7	0	0	0
EABT28885	1	2	168	4	9	0	0
EABT28886	3	4	31.75	0	0	9	1
EABT28887	723.02	952.32	244	127	427.76	46	270
EABT28888	2	1	0	0	0	2	0
EABT28889	0	1	10	0	0	0	0
EABT2889	0	0	1	1	1	0	1
EABT28890	1	4	21	1	2	2	1
EABT28891	115988	25019.5	146	19	22012.4	84425.1	139473
EABT28892	2	0	0	5	0	0	0
EABT28893	1186.96	2015.48	1501.05	1662.48	2657.33	224.95	2426.68
EABT28894	0	0	4	1	0	0	1
EABT28895	1	2	2	3	3	0	2
EABT28896	6	0	0	0	0	16	0
EABT28897	1	1	14	1	0	1	0
EABT28898	2	2	0	0	0	4	4
EABT28899	0	0	0	0	10	0	0
EABT289	2697.64	4352.61	3621.04	8572.58	4366.27	3244.3	3745.95
EABT2890	52	56	97	1171.02	105	4	1
EABT28900	4	24	26.08	12	2	2	0
EABT28901	4	28	1	9	13	12	8
EABT28902	4	5	14	6	15	6	6
EABT28903	4	5	1	0	0	6	0
EABT28904	0	0	6	0	0	1	0
EABT28905	0	0	14	0	0	0	1
EABT28906	0	0	12	0	1	1	0
EABT28907	13	1	10	14	3	1	1

EABT28908	2	6	4	0	5	14	26
EABT28909	1	0	19.01	2	0	0	2
EABT2891	1	0	8	0	1	0	0
EABT28910	19	88	48	134	50	29	37.99
EABT28911	129	190	24	4	42	2	13
EABT28912	0	0	13	0	0	0	0
EABT28913	0	1	35	3	2	0	1
EABT28914	14	20	0	0	4	95	72
EABT28915	383.01	507.99	934.35	1008.41	555.07	354.51	304
EABT28916	0	1	2	0	6	1	0
EABT28917	1	1	9	3	1	0	2
EABT28918	2052.48	3226.19	4717.69	12812.5	2291.15	1211.08	1691.38
EABT28919	0	3	12	0	1	0	8
EABT2892	2	2	39	7	2	2	1
EABT28920	0	0	12	0	1	0	0
EABT28921	0	2	65.01	14	1	1	0
EABT28922	0	2	40.01	5	0	1	2
EABT28923	948.61	1332.5	821.67	1248.7	789.96	566.8	605.92
EABT28924	6	9	10.86	11	10	0	1
EABT28925	38	73	93.01	106	49	10	24
EABT28926	3	12	5	4	0	0	1
EABT28927	0	3	4	2	0	0	0
EABT28928	0	0	1	6	0	0	0
EABT28929	1	4	11	4	0	0	0
EABT2893	1276.04	2215.04	3352.06	2418.7	2260.01	1621.02	1477.63
EABT28930	239.25	296.36	277	560.9	363	149	169
EABT28931	2	1	1	7	0	0	0
EABT28932	0	8	5	11	2	1	0
EABT28933	3	1	9	3	5	16	5
EABT28934	1	5	1	0	0	0	0
EABT28935	2	10	48	28	21	5	2
EABT28936	0	0	0	2	1	0	1
EABT28937	6702.04	4873.76	3013.74	4679.61	4078.94	8734.7	4180.48
EABT28938	1	7	1	13	2	0	0
EABT28939	2	1	0	0	0	0	0
EABT2894	0	0	0	0	0	0	0
EABT28940	3	8	24.72	8	10	1	4
EABT28941	1	31	16	4	0	2	6
EABT28942	10	9	0	8	0	0	0
EABT28943	8	12	13	1	0	1	0
EABT28944	0	0	18	2	0	1	0
EABT28945	2007.21	4097.72	5754.61	8765.66	2024.04	1459.33	1465.08
EABT28946	0	1	5	0	0	0	1
EABT28947	0	2	50	2	1	2	0
EABT28948	0	1	0	1	1	1	4
EABT28949	11049.3	8920.61	6781.13	16646.3	6303.29	9171.73	5590.38
EABT2895	1	3	4	1	0	0	0
EABT28950	324	444	637.9	1242.51	857.13	191.06	180.34
EABT28951	2472.98	5460.72	11351.6	2857.64	2319.59	188	334.26
EABT28952	0	2	11	1	7	0	0
EABT28953	10704.3	19241.2	25838.2	4290.22	6223.73	7067.25	8186.44

EABT28954	3993.2	6980.48	7290.18	4701.04	2313.01	1811.06	2108.47
EABT28955	2	3	30	1	0	0	0
EABT28956	10	27	29	7	17	8	7
EABT28957	1	7	27	7	0	8.16	2
EABT28958	0	0	14	0	0	0	0
EABT28959	2	2	1	12	0	1	0
EABT2896	0	1	8	0	1	0	0
EABT28960	306	313	1061.79	274.07	82	147.05	202
EABT28961	2	18	7	22	0	0	7
EABT28962	2732.51	3747.69	4257.14	6759.1	2608.18	3216.31	2582.23
EABT28963	0	2	12	0	2	1	0
EABT28964	15	10	8	3	1	3	4
EABT28965	0	0	2	0	1	0	2
EABT28966	5	5	14	2	1	1	0
EABT28967	0	0	14	0	0	1	0
EABT28968	0	1	16	0	0	2	0
EABT28969	1	2	3	1	1	0	0
EABT2897	0	2	14	0	2	0	0
EABT28970	77	134	176.05	642.17	540.72	11.09	35
EABT28971	0	2	3	5	0	0	0
EABT28972	0	0	18	0	0	1	1
EABT28973	9937.06	45788.8	13656.5	22126	11109.6	9	416
EABT28974	6268.3	6001.09	5458.96	5223.28	4806.2	7073.14	5123.98
EABT28975	2	8	9	0	0	2	0
EABT28976	3	0	5	4	15	0	3
EABT28977	0	1	1	2	1	0	0
EABT28978	780	1417.11	739	2269.05	1996.12	25	182
EABT28979	0	0	6	0	2	0	0
EABT2898	0	3	4	2	0	0	0
EABT28980	652.73	991.09	567.04	1243.06	498.78	344.01	425.71
EABT28981	0	0	8	1	1	0	1
EABT28982	0	0	18	0	0	0	0
EABT28983	2845.42	3993.82	6019.31	3791.12	1714.77	3486.72	2530.98
EABT28984	2	7	5	12	2	5	27
EABT28985	0	2	3	0	0	0	0
EABT28986	875.32	1028.82	161.92	784	543.4	326	720.01
EABT28987	0	1	2	10	0	0	0
EABT28988	1	0	6	21	12	2	0
EABT28989	0	0	6	0	0	0	0
EABT2899	2	3	5	8	2	1	0
EABT28990	1	1	13	2	1	0	0
EABT28991	2	0	3	1	1	0	2
EABT28992	0	0	3	4	0	0	0
EABT28993	5	3	4	11	0	2	3
EABT28994	3661.96	7588.72	3822.11	11950.4	2441.85	2486.03	2950.24
EABT28995	604	1000.85	1210.1	7744.01	1175.13	1116.74	1647.76
EABT28996	142.68	68	51	59	17	14	69
EABT28997	0	0	8	0	1	0	0
EABT28998	24	31	0	0	0	0	0
EABT28999	0	0	0	0	0	1	0
EABT29	0	1	7	0	0	0	0

EABT290	1	0	6	2	2	1	0
EABT2900	10	9	7	2	1	3	1
EABT29000	6	21	6	28	12	0	2
EABT29001	2	3	0	0	0	0	0
EABT29002	10	36	14	13.93	7	8	0
EABT29003	0	1	4	0	0	0	0
EABT29004	0	2	24	2	1	1	2
EABT29005	1	1	34	2	0	0	1
EABT29006	21	69	769.65	1189.66	139	72	128
EABT29007	1	4	9	3	2	0	1
EABT29008	21	27	3	14	2	16	3
EABT29009	0	1	4	2	1	0	0
EABT2901	0	0	8	0	0	0	0
EABT29010	630.17	830.8	758.04	1522.45	871.36	376.59	464.35
EABT29011	1	0	4	3	17	0	5
EABT29012	240	445	438.76	676.99	285	461	301
EABT29013	1	1	5	0	0	0	2
EABT29014	5	8	23	14	4	3	8
EABT29015	0	7	4	1	0	0	0
EABT29016	1	0	3	0	1	2	0
EABT29017	920.99	1076.05	1410	791	596	842	865
EABT29018	5	1	5	2	0	2	0
EABT29019	1	3	7	5	5	2	1
EABT2902	0	1	4	0	5	0	0
EABT29020	1	0	4	1	1	2	5
EABT29021	0	3	11	0	1	0	0
EABT29022	1112.05	998	410	394.1	431	140	800.77
EABT29023	1	1	1	3	2	0	1
EABT29024	2953.91	4284.89	5606.84	4951.79	3114.08	1917	1831
EABT29025	0	0	0	9	0	0	0
EABT29026	1	3	6	1	1	0	0
EABT29027	4	11	10	8	2	0	2
EABT29028	84	132	33	32	225	65	164
EABT29029	11	33	41	41	10	6	10
EABT2903	4	8	1	6	0	1	1
EABT29030	1	1	11	0	1	0	0
EABT29031	0	0	11	0	0	0	0
EABT29032	968.98	1317	1283.04	1802.01	1127.03	835	700
EABT29033	3	4	12	1	4	10	0
EABT29034	0	1	9	0	0	0	0
EABT29035	0	0	9	4	58	0	0
EABT29036	0	0	12	1	0	0	0
EABT29037	0	7	34	68	32	2	3
EABT29038	48653	15618	239.02	17	996.23	10308.7	2202.88
EABT29039	4	3	4	4	2	0	1
EABT2904	683	830	355	781.44	357.31	532	645
EABT29040	1916.2	2004.55	807.99	1755.75	1248.66	853.9	872.55
EABT29041	21718.5	4765.27	35	8	1188.34	29117	12713.4
EABT29042	1	1	5	1	1	1	3
EABT29043	40	36	83	58	30	35	146
EABT29044	1	1	0	3	3	0	0

EABT29045	656	927.05	806.11	2118.3	949	428	536.01
EABT29046	2	0	1	0	0	0	0
EABT29047	0	1	4	9	4	0	0
EABT29048	1	2	8	14	3	4	18
EABT29049	1	0	2	0	0	0	0
EABT2905	1	0	10	1	0	0	0
EABT29050	1	1	0	1	1	0	1
EABT29051	0	2	9.05	1	1	1	4
EABT29052	0	1	17	0	0	0	0
EABT29053	2	4	10	1	0	0	0
EABT29054	0	4	7	0	0	0	0
EABT29055	1	5	6	15	20	1	0
EABT29056	105	352.08	478.39	243	913.94	187	171.86
EABT29057	4	4	0	0	1	1	0
EABT29058	2	0	0	5	0	7	1
EABT29059	15	57	53	8	2	2	10
EABT2906	0	1	2	1	0	0	4
EABT29060	3230.19	4779.51	5506.04	8668.01	3736.07	3694.33	3101.86
EABT29061	807.07	2268.72	919.48	2762.09	893.5	402.99	983.81
EABT29062	0	0	1	0	0	0	1
EABT29063	1	1	8	3	0	0	0
EABT29064	2	6	0	0	0	5	4
EABT29065	0	3	4	0	0	1	0
EABT29066	0	2	33	8	0	0	0
EABT29067	2	2	4	4	1	2	1
EABT29068	0	0	5	0	0	0	0
EABT29069	1	6	14	5	2	2	2
EABT2907	0	1	5	1	1	0	0
EABT29070	4	6	76	3	3	0	2
EABT29071	6	30	253.99	46	99	7	41
EABT29072	0	2	33	1	1	0	0
EABT29073	1	1	5	8	10	0	0
EABT29074	9040.13	9559.89	4446.71	5020.01	19759.1	8673.24	5317.23
EABT29075	2	5	31	9	1	3	6
EABT29076	0	0	0	0	2	1	0
EABT29077	5	9	65	21	6	3	7
EABT29078	713.58	951.32	864.19	1175.59	808.99	450.73	547.41
EABT29079	8	25	55	28	29.97	4	1
EABT2908	0	2	10	0	9	0	0
EABT29080	2004.05	2999.29	5762.11	4872.65	1792.26	1199.02	1096.59
EABT29081	1	2	24	9	1	0	1
EABT29082	1	2	3	1	0	0	0
EABT29083	0	0	24	0	0	0	0
EABT29084	2	0	1	2	4	0	1
EABT29085	5	10	160	3	1	1	3
EABT29086	4	12	32	7	2	3	5
EABT29087	620.99	1190	1984.41	1477.9	1644.99	591.69	763.85
EABT29088	0	0	3	0	0	0	0
EABT29089	5291.88	8042.87	6100.34	11229.5	5496.52	4913.4	4674.15
EABT2909	1	3	1	5	0	1	1
EABT29090	1	1	40	6	0	0	0

EABT29091	0	7	2	2	1	2	1
EABT29092	0	0	8	1	0	0	1
EABT29093	3	11	12	17	16	1	5
EABT29094	0	2	1	1	0	0	0
EABT29095	1439	1963.23	1813	2658.86	1737.93	1427.25	1416.43
EABT29096	2	2	0	3	1	2	2
EABT29097	0	2	70	4	0	0	1
EABT29098	0	2	8	0	0	0	0
EABT29099	5671.09	2218.18	520.66	98.38	1296.61	6947.65	6078.45
EABT291	0	5	8	1	2	0	0
EABT2910	0	1	5	1	0	0	1
EABT29100	0	1	4	1	0	0	0
EABT29101	1	8	11	2	2	0	0
EABT29102	2	0	1	0	0	0	0
EABT29103	0	0	8	0	0	0	0
EABT29104	0	3	13	1	1	0	0
EABT29105	15	24	71	14	2	7	8
EABT29106	6	3	4	5	1	4	0
EABT29107	0	0	0	22	0	1	0
EABT29108	0	7	2	16	0	0	1
EABT29109	888.72	1832.1	949.1	4469.81	1091	674	807
EABT2911	0	1	11	0	0	0	0
EABT29110	1	7	43	0	0	5	2
EABT29111	0	2	11	0	0	1	0
EABT29112	304.97	337	503.33	46	44	0	0
EABT29113	1	0	5	2	1	0	0
EABT29114	1161.91	746.2	250	703.03	458	1	23
EABT29115	0	2	0	0	0	0	5
EABT29116	2	1	7	1	0	1	1
EABT29117	8	16	138.01	4	6	3	7
EABT29118	1	4	14	0	0	1	1
EABT29119	0	0	1	8	0	0	1
EABT2912	38	60	16	151	41	102	58.02
EABT29120	3	8	8	2	1	0	1
EABT29121	2	0	1	0	3	0	2
EABT29122	2267	2566.93	1815	3870.66	2080.18	2663.82	1806
EABT29123	1	0	7	0	1	0	0
EABT29124	0	0	3	0	3	0	0
EABT29125	0	7	8	8	1	0	0
EABT29126	0	0	8	0	0	2	1
EABT29127	0	0	0	2	9	1	6
EABT29128	0	1	0	1	1	1	0
EABT29129	488	661	424.95	1509	558	305	287
EABT2913	0	0	28	2	0	0	0
EABT29130	987.21	1070	389	2010.62	1216.61	618	630
EABT29131	5	4	17	0	0	1	1
EABT29132	665.36	932.64	740.1	1259.55	695.75	498.93	461.73
EABT29133	1078.55	2143.04	2860.37	2469.5	973.67	455	667.88
EABT29134	0	0	11	3	4	4	0
EABT29135	0	2	18	1	0	2	1
EABT29136	3	7	8	14	4	2	1

EABT29137	0	1	3	0	1	1	3
EABT29138	3	11	9	10	8	1	1
EABT29139	11	17	108	71	2	4	2
EABT2914	4	11	13	92	5	0	2
EABT29140	1	0	3	1	1	1	0
EABT29141	108	75	244	37	379	112	571.35
EABT29142	2	12	7	0	0	0	1
EABT29143	1	15	8	3	1	0	0
EABT29144	0	0	21	2	3	0	0
EABT29145	0	0	4	1	0	0	0
EABT29146	14	31.87	0	0	0	1	0
EABT29147	1	3	4	6	1	4.99	2
EABT29148	0	3	3	10	2	1	2
EABT29149	4	2	0	0	0	5	0
EABT2915	0	1	4	0	0	0	0
EABT29150	1	1	1	0	1	0	0
EABT29151	3	1	29	0	0	0	1
EABT29152	7	9	44	19	5	7	12
EABT29153	0	0	0	0	0	0	5
EABT29154	8	10	7	11	44	0	40
EABT29155	0	7	5	1	0	0	1
EABT29156	1361	1807	962.99	1925	1037.7	660	658.45
EABT29157	9	0	1.81	0	0	3	0
EABT29158	21	35	22	145	16	26	9
EABT29159	4	2	3	1	0	2	8
EABT2916	4	2	0	0	0	1	1
EABT29160	1	4	4	13	21	0	0
EABT29161	8	2	0	0	0	0	0
EABT29162	0	7	54	32	3	0	2
EABT29163	1	2	4	0	0	0	0
EABT29164	112	71	16	19	32	65	364
EABT29165	8	10	12	3	4	28	5
EABT29166	0	0	1	1	1	1	1
EABT29167	0	0	50	0	0	0	1
EABT29168	8	12	0	0	0	60	39
EABT29169	1404.17	1908	1865	3838.26	1388.5	1312.04	996
EABT2917	0	4	32	8	8	0	0
EABT29170	1906.92	3534.92	2108.31	7421.76	1283.52	1761.01	1522.96
EABT29171	1	1	9	2	1	0	0
EABT29172	22	33	261	19	11	18	9
EABT29173	1	0	3	0	8	1	19
EABT29174	0	0	11	1	2	2	0
EABT29175	0	0	3	0	0	0	1
EABT29176	1	1	6	0	0	0	0
EABT29177	1	2	8	8	0	0	1
EABT29178	3	3	2	0	2	5	6
EABT29179	0	1	4	1	0	1	0
EABT2918	1	0	10	1	1	0	0
EABT29180	10	27	27.01	8.96	62	5	4
EABT29181	0	2	10	1	0	0	0
EABT29182	3	6	6	13	4	0	1

EABT29183	26	362.41	182.91	33.91	321.81	8	7
EABT29184	1	0	11	0	2	0	3
EABT29185	34	41	0	0	0	0	1
EABT29186	10	19	23	76	10	4	7
EABT29187	7	29	7	7	2	6	20
EABT29188	9	11	39	4	5	4	3
EABT29189	222.01	701.92	1119	4445.27	587.04	103	210
EABT2919	1	0	6	1	0	0	0
EABT29190	4	7	16	11	8	4	8
EABT29191	0	0	2	3	1	0	0
EABT29192	6014.4	7901.38	7022.44	10900.1	5309.83	4990.33	3795.42
EABT29193	0	4	6	4	0	1	2
EABT29194	0	0	19	1	1	1	2
EABT29195	4	1	3	6	3	0	1
EABT29196	0	0	0	0	0	0	0
EABT29197	8	33	35	54	18	1	2
EABT29198	0	1	8	1	0	0	0
EABT29199	1	2	30	6	1	0	1
EABT292	1	0	12.13	3	1	1	0
EABT2920	1	0	0	22	0	1	0
EABT29200	681.12	2418.34	16.01	0	56	1118.59	66
EABT29201	0	0	0	0	1	0	0
EABT29202	2957.94	2820	1679.31	1712	7753	2135	1476.05
EABT29203	6	50	102	1	1	2	2
EABT29204	0	3	3	2	3	2	1
EABT29205	9591.87	7544.01	7510.97	7666.93	3808.64	3142.02	1918.61
EABT29206	16	39	9	4	2	6	10
EABT29207	1	2	24	6	0	8	0
EABT29208	10344.6	8864.42	3748.37	922.89	1081.31	1844.89	919.76
EABT29209	1	1	821.19	298591	0	0	0
EABT2921	49	298	401	1676.8	272.24	20	150
EABT29210	2	0	3	0	0	2	0
EABT29211	10	18	10	30	10	15	7
EABT29212	22.1	119	22	1	5	67	18
EABT29213	78.94	104.02	269.05	423.23	41.67	1	7
EABT29214	0	9	52	462	2	2	0
EABT29215	0	1	0	5	0	0	0
EABT29216	2	8	0	0	0	4	3
EABT29217	0	0	6	0	0	0	0
EABT29218	19	31.97	31	50	30	10	12
EABT29219	1	0	13	0	0	0	0
EABT2922	1.82	1	1	7	1	0	2
EABT29220	1	4	4	51	1	1	0
EABT29221	2270.42	5053.73	6130.42	8056.95	3884.55	3414.9	4080.7
EABT29222	2333.19	3349	11678.7	7611.46	3456.79	4804.91	4178.74
EABT29223	2	7	4	4	1	1	6
EABT29224	64	149	53.68	60	64	7	34
EABT29225	0	0	10	5	0	1	0
EABT29226	1	3	6	1	0	1	22
EABT29227	0	2	12	1	0	0	0
EABT29228	839	1287.2	1054.41	1073	587.02	424.84	704.68

EABT29229	0	0	2	14	0	0	1
EABT2923	3	4	3	4	0	0	3
EABT29230	5770.45	9386.38	5286.39	5300.33	8713.65	4614.78	8026.69
EABT29231	0	4	5	0	0	0	1
EABT29232	3	1	3	5	1	0	0
EABT29233	2640.13	5032.51	8357.68	7760.31	2237.73	2408.3	2830.25
EABT29234	0	0	0	0	4	0	0
EABT29235	0	6	1	2	0	1	0
EABT29236	2	9	19	7	3	1	0
EABT29237	0	4	13	5	10	3	22
EABT29238	5	4	0	2	0	3	1
EABT29239	65	41	32	0	27	0	2
EABT2924	0	1	2	1	3	0	0
EABT29240	3	12	17	11	7	6	7
EABT29241	5	20	14	3	0	0	0
EABT29242	2	3	0	2	0	0	0
EABT29243	0	0	2	2	0	0	0
EABT29244	2	2	3	0	0	1	1
EABT29245	89.68	150	303.82	633	377	10	460
EABT29246	0	0	6	1	1	0	0
EABT29247	2136	12	2	2	1	3181.22	3
EABT29248	0	3	12	2	0	0	0
EABT29249	4	0	11	0	2	2	0
EABT2925	0	0	18	0	0	4	0
EABT29250	4	11	14	3	2	9	7
EABT29251	0	3	43	1	1	0	0
EABT29252	316	399.99	208	455	358	270	270
EABT29253	6	591	1304.62	1682.99	85	2	4
EABT29254	0	0	19	0	0	0	0
EABT29255	1	1	1	24	0	0	0
EABT29256	0	1	4	0	0	0	0
EABT29257	0	0	9	0	0	0	0
EABT29258	4	1	2	1	2	0	1
EABT29259	0	0	3	3	264.92	0	0
EABT2926	1	0	3	0	2	2	0
EABT29260	0	0	0	0	4	2	17
EABT29261	1	0	1	1	0	0	0
EABT29262	0	2	8	0	0	0	0
EABT29263	7	12	14	1	1	19	9
EABT29264	0	1	2	0	0	0	1
EABT29265	1	2	6	2	0	0	0
EABT29266	541	847	199.01	409	472	373.09	817.04
EABT29267	1	1	11	2	3	0	2
EABT29268	7522.74	9816.79	7095.55	12121.3	5592.79	6423.21	5311.33
EABT29269	778.38	1166.57	445.67	687.51	977.9	470.21	648.89
EABT2927	1	0	0	0	0	32	0
EABT29270	0	3	9	0	0	2	1
EABT29271	2	14	1.18	40	5	1	1
EABT29272	2	10	15	6	0	0	0
EABT29273	1	5	3	3	0	5	1
EABT29274	981.26	1542.86	2528.21	2969.01	1300.35	1404.48	1157.96

EABT29275	615.05	824.7	504.71	1475.06	469.03	367	298.97
EABT29276	1	3	12	2	0	0	0
EABT29277	0	0	8	0	0	0	0
EABT29278	2	4	3	2	0	0	0
EABT29279	0	2	0	1	1	1	0
EABT2928	1	5	13	8	0	1	3
EABT29280	0	2	3	0	1	0	2
EABT29281	1574.09	2403.28	1792.87	4222.11	1882.35	1587.9	1810.2
EABT29282	0	6	19	0	0	5	1
EABT29283	4674.48	6717.89	8107.47	9526.52	19062.5	105	280
EABT29284	6	9.98	113.12	11	39	3	5
EABT29285	3	4	0	4	2	1	1
EABT29286	0	2	0	3	2	4	0
EABT29287	3	8	22	12	2	1	1
EABT29288	4969.06	7446.45	5972.94	8141.52	3078.99	5507.41	6793.2
EABT29289	1	1	0	3	5	0	4
EABT2929	108	184.99	196.97	806	229.32	75.95	84
EABT29290	0	1	0	1	4	0	3
EABT29291	312	427.07	298.05	532.96	301	143	208
EABT29292	0	1	10	0	0	0	0
EABT29293	4	18.13	7	45	2	3	3
EABT29294	1	1	8	1	0	0	0
EABT29295	0	1	0	1	1	2	5
EABT29296	0	0	11	0	0	2	0
EABT29297	0	0	9	0	0	0	0
EABT29298	418.99	937.05	1749	2610.27	1917	176.04	616
EABT29299	2	2	13	0	0	1	1
EABT293	0	1	3	6	0	0	0
EABT2930	4271.84	920.7	75.93	36	11	5200.31	22
EABT29300	0	0	8	0	1	2	0
EABT29301	0	4	2	5	1	0	2
EABT29302	1	2	3	0	0	0	1
EABT29303	2	9	0	0	0	0	0
EABT29304	1106.15	2596.53	3968.87	4605.14	648.67	652.86	773.27
EABT29305	2	0	0	5	0	2	0
EABT29306	0	0	15	6	0	0	0
EABT29307	0	0	5	0	0	0	0
EABT29308	0	4	2	0	0	4	0
EABT29309	1	1	4	2	0	1	0
EABT2931	12	0	3	7	2	10	5
EABT29310	1	7	1	18	0	2	1
EABT29311	282.18	398.05	264	543.79	503	323.53	251.15
EABT29312	150	242	172	685.96	257.58	54	105
EABT29313	1322.75	3062.05	7124.88	3950.87	1777.35	865.99	1771
EABT29314	1	6	5	13	2	2	3
EABT29315	2	0	2	2	0	1	0
EABT29316	0	1	9	0	2	0	0
EABT29317	19	36	10	1	49	0	7
EABT29318	544	581	1194.98	5620	2777	19	24
EABT29319	2	2	9	8	1	0	2
EABT2932	1	2	2	1	0	0	0

EABT29320	15	27	124.97	42	11	6	19
EABT29321	2	2	5	2.53	1	0	1
EABT29322	21	5	45	1	4	50	89
EABT29323	16	144	3	2	3	11	24
EABT29324	0	0	0	0	1	0	4
EABT29325	0	5	28	0	2	2	0
EABT29326	28	45	29	123	71	2	40
EABT29327	1	1	0	0	2	0	0
EABT29328	9	8	17	9	5	9	2
EABT29329	0	3	1	2	3	0	6
EABT2933	1	0	0	10	0	0	0
EABT29330	3	7	34	14	5	6	3
EABT29331	0	0	11	1	0	2	0
EABT29332	30	48	193.17	166	408.43	31	251.91
EABT29333	0	0	1	4	0	0	1
EABT29334	6	15	0	0	0	4	2
EABT29335	390	999.02	1349	1512	1586	531	896.44
EABT29336	1	3	7	0	0	0	0
EABT29337	0	1	2	20	5	0	0
EABT29338	0	3	1	0	0	0	0
EABT29339	438.27	516.01	398.96	2167	616.01	354	377
EABT2934	1	0	1	2	0	0	0
EABT29340	47	126	137	237	177.99	19	113
EABT29341	0	0	8	2	2	0	1
EABT29342	1	0	76	80833.9	1	0	0
EABT29343	1553.96	4110.03	2207.24	3263.67	2143.38	1648.91	2186
EABT29344	402	227	1	0	28	1137.68	125
EABT29345	0	0	0	0	2	0	0
EABT29346	1	2	3	3	0	0	0
EABT29347	551.89	639.26	709.89	1246.1	456.08	406.83	414.71
EABT29348	0	2.92	2	0	0	0	0
EABT29349	49.93	17	2	0	3	0	3
EABT2935	0	0	13	2	1	0	1
EABT29350	2322.74	1583.99	2736.58	470.25	2192.21	707.01	1416.64
EABT29351	0	2	2	0	0	0	0
EABT29352	338.93	567.76	669.98	1580.57	678.02	267.99	400
EABT29353	10	81	44	71	127	11	15
EABT29354	10	12	47	115	1	1	2
EABT29355	8	4	4	4	4	5	9
EABT29356	12	24	13	19	2	6	5
EABT29357	0	0	1	5	4	1	0
EABT29358	4	7	3	8	0	0	0
EABT29359	0	1	18	0	0	1	2
EABT2936	602.99	1153.07	731	2976.51	729	373.9	770.05
EABT29360	4	6	13	9	10	1	13
EABT29361	0	0	2	0	0	1	0
EABT29362	0	0	0	0	1	0	0
EABT29363	9	14	60	5	7	3	12
EABT29364	0	1	9	3	2	1	1
EABT29365	406.99	441.37	447	1821.64	845.26	488.28	543.44
EABT29366	0	0	0	0	0	0	0

EABT29367	1585.01	1884.92	1662	2840.96	1354.16	1060	770
EABT29368	11	7	1	0	8	30	28
EABT29369	3	7	12	0	0	2	0
EABT2937	4	1	6	0	0	1	1
EABT29370	0	2	5	1	2	0	0
EABT29371	1	1	2	0	0	1	0
EABT29372	2	5	24	21	0	0	1
EABT29373	1047.71	1071.32	534.3	1200.87	1237.64	1026.42	959.98
EABT29374	0	3	5	0	1	0	0
EABT29375	851.28	1144.74	537.31	2345.27	1142.82	859.66	908.46
EABT29376	233.27	238.99	252.04	275	259.81	168	199
EABT29377	40196.4	40905.1	20038	29713.6	32372.3	37043.9	39193.6
EABT29378	35	55	48.1	38	1604	28	36
EABT29379	0	2	5	0	1	3	2
EABT2938	28992.3	62110.8	87292.7	2175.91	19984.1	3159.68	9793.03
EABT29380	0	2	2	1	3	1	0
EABT29381	980.36	1612.31	803.17	1927.49	638.71	1052.79	995.54
EABT29382	2	7	22	12.54	9	12	4
EABT29383	2	9	21	47	4	2	4
EABT29384	11	32	403.88	178.15	28	14	12
EABT29385	2358.94	4125.9	5427.13	8280.47	2299.91	2322.07	2273.96
EABT29386	424.17	517.6	539.45	827.03	441.66	287	327.96
EABT29387	1075.45	2741.43	1303.76	4582.06	1783.88	683.91	1212.61
EABT29388	1	3	6	17	1	0	1
EABT29389	0	0	4	0	1	0	0
EABT2939	17	63	348	111	111	2	57
EABT29390	1	0	7	1	0	0	0
EABT29391	2	1	7	3	1	5	2
EABT29392	0	1	9	0	0	1	0
EABT29393	10	12	52	8	1	3	1
EABT29394	0	1	15	1	0	0	0
EABT29395	2869.54	4156.41	5163.01	4053.59	3693.99	2927.92	3997.18
EABT29396	40	78	97	34	6	27	11
EABT29397	246.81	514.47	4449.87	164.99	639.51	477.26	717.56
EABT29398	4	6	18	3	1	0	0
EABT29399	0	0	5	0	0	0	0
EABT294	1	1	0	2	0	0	2
EABT2940	1	0	4	1	1	0	3
EABT29400	9894.92	11791.8	10700.9	12089.6	4968.13	7052.15	5128.93
EABT29401	2714.18	4079.64	3131.4	5720.21	2308.16	2203.7	2601.38
EABT29402	1	3	4	0	0	1	0
EABT29403	2	1	36	1	1	4	3
EABT29404	0	0	1	6	0	0	0
EABT29405	0	0	0	7	0	0	0
EABT29406	12	30	112	43	12	0	8
EABT29407	0	0	6	0	0	0	0
EABT29408	2746.28	4406.15	4491.5	4420.51	2204.21	3926.65	3336.91
EABT29409	0	1	1	11	11	0	1
EABT2941	0	0	1	11	0	0	0
EABT29410	2	4	0	0	0	3	2
EABT29411	2	3	0	0	1	14	2

EABT29412	0	1	7	14	11	1	0
EABT29413	0	0	24	2	0	2	1
EABT29414	0	0	11	11	5	1	1
EABT29415	1577.2	2481.5	2183.56	3467.55	2278.85	1912.18	1200.02
EABT29416	2	5	3	4	0	2	3
EABT29417	1	1	2	2	1	1	0
EABT29418	9	13	10	3	15	3	21
EABT29419	20024.7	8218.36	234.91	5	4101.91	25964.7	26591.9
EABT2942	3	5	11	2	1	3	3
EABT29420	0	0	0	5	0	1	0
EABT29421	0	3	1	1	1	0	0
EABT29422	0	2	3	4	1	0	0
EABT29423	0	6	12	2	1.99	0	0
EABT29424	61	1	1	3	288.74	82	2464.89
EABT29425	3	3	2	5	0	1	2
EABT29426	6011.61	10342.1	8150.97	18057.9	4855.25	3895.86	3868.2
EABT29427	8	15	30	21	3	3	2
EABT29428	2	0	5	3	0	1	0
EABT29429	0	1	5	2	1	0	0
EABT2943	0	0	18	1	0	0	0
EABT29430	3030.88	10714.9	12219.2	3787.2	18560.1	18	112
EABT29431	3975.35	9764.33	3149.94	841.04	3433.46	3004.58	4511.88
EABT29432	5	0	1	0	3	13	7
EABT29433	2331.25	2239.53	1912.9	2306.39	1166.99	235	445.74
EABT29434	3	2	2	2	0	5	0
EABT29435	2829.24	3443.18	7642.93	5626.96	2878.36	1012.99	2274.78
EABT29436	2	9	7	11	2	0	2
EABT29437	10	10	7	9	1	2	1
EABT29438	0	1	13	2	0	0	0
EABT29439	0	0	4	0	12	0	1
EABT2944	0	1	4	1	1	1	2
EABT29440	6	14	5	5	0	2	3
EABT29441	2	0	10	1	0	0	1
EABT29442	1	0	1	1	3	2.81	4
EABT29443	0	3	1	8	2	1	1
EABT29444	6	12	8	37	35	5	2
EABT29445	0	0	3	1	0	0	0
EABT29446	1	1	0	2	2	2	0
EABT29447	1	3	176	160	0	0	2
EABT29448	3	7	12	12	0	1	0
EABT29449	1	1	5	0	0	3	1
EABT2945	0	1	3	3	2	0	1
EABT29450	86	345	814.58	422.61	131.2	98	105.02
EABT29451	2	1	4	0	0	0	0
EABT29452	0	0	26	0	1	0	0
EABT29453	0	1	4	2	0	0	0
EABT29454	0	0	4	0	1	0	0
EABT29455	7	9	17	17	8	6	5
EABT29456	0	0	0	0	0	0	0
EABT29457	10	10	6	2	0	0	2
EABT29458	1	8	1	4	0	2	2

EABT29459	1	3	9	0	0	0	0
EABT2946	0	1	3	4	0	0	0
EABT29460	1	1	9	1	0	0	0
EABT29461	0	1	0	0	0	0	3
EABT29462	0	0	5	0	0	0	0
EABT29463	0	3	15	0	0	0	2
EABT29464	1338.62	1623	1035.06	2291.95	727.15	1159.23	1077.03
EABT29465	0	5	4	12	0	2	0
EABT29466	2	0	1	4	1	0	2
EABT29467	455.24	598.2	235.07	1009.05	427.79	227.03	405.01
EABT29468	8	14	8	18	3	6	4
EABT29469	0	2	15	4	2	3	1
EABT2947	1	0	16	0	0	2	0
EABT29470	0	0	0	2	8	0	2
EABT29471	3	5	9	8	7	5	4
EABT29472	2	7	23	12	2	6	4
EABT29473	0	0	4	0	0	0	0
EABT29474	37	16	24	23	2	13	21
EABT29475	825	895	664.69	1044	742.06	1236.8	910.01
EABT29476	28	14	16	2	5	6.73	1
EABT29477	1	2	1	5	1	0	0
EABT29478	3	2	1	1	0	2	1
EABT29479	0	2	14	5	3	5	5
EABT2948	0	6	17	3	14	0	0
EABT29480	0	2	0	3	0	0	0
EABT29481	0	3	7	3	1	0	1
EABT29482	1	4	1	0	9	0	0
EABT29483	0	1	3	0	0	0	0
EABT29484	2	42	16	91	11	3	10
EABT29485	29	98	44	69.96	45.98	31	32
EABT29486	678.8	1188.52	853.93	2422	811.43	790.88	797.37
EABT29487	0	0	9	0	0	0	0
EABT29488	13	2	79	35	3	2	3
EABT29489	0	0	1	0	3	0	0
EABT2949	8557	3837	2365.05	1804.01	1690	10041.3	3195.84
EABT29490	5	5	11	3	2	14.68	8
EABT29491	2	1	0	0	0	0	5
EABT29492	0	2	5	1	0	0	3
EABT29493	1	3	5	2	0	0	1
EABT29494	4	8	46	0	2	0	2
EABT29495	1	0	6	0	0	0	0
EABT29496	14074.9	9426.69	64	4	1518.48	465	4322.32
EABT29497	2095.16	2888.21	2326	3590.99	1762.8	2383.22	2081.02
EABT29498	0	0	0	0	0	0	0
EABT29499	1	4	13	6	2	2	1
EABT295	11	13	6	4	15	10	18
EABT2950	1	0	7	3	0	0	0
EABT29500	0	0	1	2	3	1	1
EABT29501	0	0	9	8	0	1	0
EABT29502	0	1	2	0	1	0	2
EABT29503	648	592	1265	580	530	872	467.15

EABT29504	0	1	2	3	0	0	0
EABT29505	526	680	412	196	403	932	1580.83
EABT29506	0	2	8	2	0	0	0
EABT29507	0	1	7	1	0	0	1
EABT29508	0	0	5	4	0	0	0
EABT29509	477	741.54	812	1974.44	685	581	549.1
EABT2951	4050.07	7362.26	8079.04	9783.79	5706.27	4851.09	5880.53
EABT29510	0	1	30	0	1	0	1
EABT29511	3	26	3	3	3	1	3
EABT29512	26	50	24	126	68.15	33.02	36.05
EABT29513	1	0	5	0	0	0	0
EABT29514	3	3	3	2	0	1	4
EABT29515	10	2	0	2	0	3	0
EABT29516	21	6	2	2	4	9	1
EABT29517	1	1	6	1	1	0	1
EABT29518	8.02	15	3	10	0	4	0
EABT29519	1	3	9	2	0	1	1
EABT2952	608.99	858.95	669	838.84	594.25	438.13	409
EABT29520	2	3	1	2	0	0	0
EABT29521	325.13	964.29	3317.99	8626.89	1075	219	507.98
EABT29522	0	0	1	0	0	0	0
EABT29523	4	2	0	25	13	1	0
EABT29524	4	8	2	2	1	0	0
EABT29525	0	6	20	12	1	1	0
EABT29526	1	0	2	2	0	1	0
EABT29527	1	2	1	0	0	0	0
EABT29528	1	0	7	1	0	0	0
EABT29529	0	1	5	2	0	0	0
EABT2953	0	3	1	3	0	0	0
EABT29530	0	3	12	2	1	0	1
EABT29531	4	4	1	0	3	4	4
EABT29532	1	2	48	0	0	3	0
EABT29533	3	11	0	0	0	8	8
EABT29534	0	2	1	2	2	0	0
EABT29535	27	58	0	0	1	3	15
EABT29536	0	0	3	0	1	0	0
EABT29537	0	2	11	5	1	3	1
EABT29538	3	2	1	11	0	3	0
EABT29539	0	1	1	5	0	0	0
EABT2954	2	2	0	1	0	0	0
EABT29540	0	0	2	15	6	2	0
EABT29541	4	14	12	0	0	8	3
EABT29542	2846.37	4023.83	8174.54	2338.04	5443.05	1401.46	1952.76
EABT29543	1	1	2	3	2	0	1
EABT29544	0	0	14	0	0	0	0
EABT29545	606.97	887.91	1904.53	1790.47	760.15	654.9	774.46
EABT29546	171	206	303.36	609	281	104	145
EABT29547	2	6	14	7	3	0	1
EABT29548	1	0	31	2	0	1	0
EABT29549	1880.01	2386.17	1767.01	4039.71	1567.48	1472	1106.68
EABT2955	4	6	57	4	0	1	2

EABT29550	2090	129	0	0	9	71	54
EABT29551	6	1	10	8	0	1	1
EABT29552	11	1	2	0	0	32	5
EABT29553	0	0	0	0	0	0	0
EABT29554	0	0	2	3	1	0	0
EABT29555	0	1	6	1	0	0	0
EABT29556	0	0	8	0	0	0	0
EABT29557	0	0	6	0	0	0	0
EABT29558	113	173	249	449.1	154	71	106
EABT29559	0	2	2	9	3	1	0
EABT2956	0	4	1	2	1	0	5
EABT29560	0	2	1	1	0	0	0
EABT29561	0	1	3	1	5	0	0
EABT29562	3	6	3	1	0	14	10
EABT29563	0	1	1	3	8	0	0
EABT29564	915.85	1104.02	998.69	1774.38	725.41	1545.58	1234.51
EABT29565	2	8	7	0	3	2	2
EABT29566	1969.66	2019.1	203	1093.65	583	0	107.01
EABT29567	1070	1724.93	2488	1091	960	1395	1227
EABT29568	17	32	35	50	15	5	18
EABT29569	0	0	3	1	1	0	1
EABT2957	2	6	18	4	2	2	1
EABT29570	98	161	97	139	118	27	126
EABT29571	0	1	12	0	0	0	0
EABT29572	0	0	2	3	1	1	0
EABT29573	0	0	8	10	0	0	0
EABT29574	51	33	2	2	14	0	0
EABT29575	18	23	32.86	343	77.87	1	78
EABT29576	27	45	0	0	2	4	5
EABT29577	0	0	3	3	1	0	0
EABT29578	0	0	6	0	0	0	0
EABT29579	2	3	0	122	26	0	1
EABT2958	1157.77	2795.1	471.02	365.77	4022.47	1579.74	7834.55
EABT29580	1701.99	5152.04	7218.71	4380.65	4328.3	1317.28	5919.64
EABT29581	10	5	3	1	2	5	14
EABT29582	0	0	7	0	0	0	0
EABT29583	3809.87	4051.11	3501.98	4819.64	2569.38	3748.92	2581.03
EABT29584	0	7	29	3	1	12	4
EABT29585	0	4	3	4	3	0	2
EABT29586	0	0	49.46	0	1	1	0
EABT29587	238	425.11	329	732.01	330	212	183
EABT29588	1	0	5	1	0	0	0
EABT29589	0	1	15	0	0	0	0
EABT2959	1	0	2	7	0	0	0
EABT29590	0	0	3	0	1	0	2
EABT29591	0	0	10	0	0	0	0
EABT29592	7	7	23	5	4	1	4
EABT29593	2	3	12.66	9	2	1	2
EABT29594	1	1	7	0	1	0	0
EABT29595	2	2	1	0	2	3	1
EABT29596	0	1	38	3	0	1	0

EABT29597	1	0	3	0	1	0	1
EABT29598	1105.08	519	296	4	510	225.08	2174.68
EABT29599	144.03	225	194.96	571	163.17	91	159.87
EABT296	1	1	45	4	0	4	0
EABT2960	1207.64	2956.64	2387.13	6135.86	1448.98	349.39	386.89
EABT29600	1	3.26	52	3	1	4	0
EABT29601	0	1	0	0	0	0	0
EABT29602	2	0	0	0	1	1	0
EABT29603	0	5	10	7	0	0	1
EABT29604	3	6	10	2	1	1	10
EABT29605	0	3	10	7	0	0	0
EABT29606	1	5	0	8	0	0	0
EABT29607	2	5	59.01	6	2	6	3
EABT29608	0	2	45	1	0	1	1
EABT29609	0	0	5	0	0	0	0
EABT2961	1	2	5	4	0	0	0
EABT29610	3959.42	6568.65	5254.81	10325.9	4478.91	4233.6	3723.97
EABT29611	1958.99	2809.96	1856.75	2529	1150	1988.68	1691
EABT29612	1	1	8	9	10	0	1
EABT29613	1	1	55	2	9	2	10
EABT29614	10394.1	17749.9	2713.42	12453.4	14215	6657.15	21105
EABT29615	1	1	3	0	1	0	2
EABT29616	0	0	6	0	0	0	0
EABT29617	1	7	7	9	0	0	4
EABT29618	0	0	59	1	0	0	0
EABT29619	53.77	148.7	109.92	6	402.01	14	29.01
EABT2962	96.99	179.48	38.39	97.1	49.87	51	39.09
EABT29620	0	1	3	1	0	0	0
EABT29621	18	65	85	48	20	18	16
EABT29622	1	4	2	4	0	0	0
EABT29623	1	1	1	9	2	0	1
EABT29624	0	0	33	1	2	0	1
EABT29625	1	0	3	1	1	0	0
EABT29626	2	2	2	4	0	2	2
EABT29627	812.7	1177.83	643	2032.3	1029	644	615
EABT29628	53	120	98	85	38	27	13
EABT29629	0	0	8	0	0	0	0
EABT2963	0	0	6	1	0	0	0
EABT29630	25	108	1274.05	210	1277	0	15
EABT29631	0	1	1	1	1	0	0
EABT29632	100	87	23	213	285	59	174.49
EABT29633	1	0	4	0	0	1	1
EABT29634	10	7	37	35	2	9	4
EABT29635	52351.4	19607.5	52	1	252.3	16647	1147.84
EABT29636	0	0	0	0	0	0	0
EABT29637	0	0	11	0	0	0	0
EABT29638	0	1	21	0	0	0	0
EABT29639	5	11	9	0	7	3	15
EABT2964	0	3	2	0	0	0	0
EABT29640	3	4	2	4	0	3	3
EABT29641	7	11	15	61	4	0	5

EABT29642	0	0	1	0	0	0	2
EABT29643	0	1	5	13	6	1	0
EABT29644	0	1	15	0	2	3	3
EABT29645	1716.3	3361.77	1576.99	3482.11	2055.28	1662.35	2529.92
EABT29646	1	3	31	27	2	0	4
EABT29647	0	0	9	2	0	0	0
EABT29648	3	13	3	7	6	0	1
EABT29649	0	1	4	2	6	0	0
EABT2965	25	32	97	22	10	19	12
EABT29650	2	1	15	2	4	0	0
EABT29651	58	109	11	11	9	0	0
EABT29652	1	2	11	25	5	0	1
EABT29653	0	0	9	0	0	0	0
EABT29654	4	15	109	9	1	8	0
EABT29655	17612.7	10085.1	864.12	153.99	3957.08	28447.7	9676.51
EABT29656	1774.78	1190.24	704	1136.06	891.07	303.07	1465.45
EABT29657	2226	1965	1307	362	238.2	386	574
EABT29658	6	10	14	11	4	4	4
EABT29659	82	120	81	7	26	29	87
EABT2966	1	7	3	1	5	0	6
EABT29660	2	4	13	1	0	0	0
EABT29661	783.57	1168.24	805.96	1560.54	794.79	622.56	748.04
EABT29662	0	4	0	0	0	7	0
EABT29663	3794.1	4082.87	1315.02	2934.91	1473.46	3656.54	3013.41
EABT29664	1	0	2	3	5	1	2
EABT29665	1811.72	2709.45	217.11	40	1004.04	2396.94	6451.35
EABT29666	4.03	49	0	0	0	12.84	0
EABT29667	2.92	234.79	231.78	241.92	67.53	75.99	79.99
EABT29668	8	10	5	3	7	2	17
EABT29669	1	1	72	0	2	0	2
EABT2967	0	4	91	23	2	1	1
EABT29670	0	0	5	4	1	0	1
EABT29671	349.02	682	537.87	1638	558.04	190	305.01
EABT29672	0	4	2	4	0	0	0
EABT29673	4	4	5	0	0	9	0
EABT29674	0	2	3	0	1	0	0
EABT29675	1	2	12	2	3	5	2
EABT29676	1	3	19	5	0	1	1
EABT29677	0	0	0	0	0	2	35
EABT29678	1	10	202	2	6	5	3
EABT29679	0	5	0	7	19	0	0
EABT2968	2	5	16	1	0	0	1
EABT29680	27	56	54	7	7	0	0
EABT29681	0	0	8	0	0	0	0
EABT29682	337.96	466	180	271.36	193	132.14	207
EABT29683	16906.3	19926.8	11592.4	20959.7	9708.06	32	683
EABT29684	2399.47	3664.75	2652.9	3327.08	2006.63	2255.81	1667.73
EABT29685	57	12	1	2	0	90	0
EABT29686	132.99	342	935.55	408.99	108.94	74	104
EABT29687	6	20	14	0	0	0	3
EABT29688	4	22	6	8	10	0	0

EABT29689	0	1	2	0	0	0	0
EABT2969	94	121	104.41	178	88	37	43.99
EABT29690	890.84	1262.01	1798.09	1774.12	1360	915	952.94
EABT29691	1	0	2	2	0	0	0
EABT29692	5	1	2	0	0	13	0
EABT29693	3	8	7	7	3	0	2
EABT29694	1	3	5	0	2	3	10
EABT29695	1833.94	2425.42	4587.88	6345.71	2612.97	1810.98	1378
EABT29696	1	0	8	1	0	0	1
EABT29697	9	68	39.06	40	16	7	20
EABT29698	3557.26	7465.29	13076.6	9628.33	4118.37	3593.53	3956.1
EABT29699	3	5	18	36	27	0	0
EABT297	0	1	1	8	0	0	0
EABT2970	0	1	7	1	0	0	1
EABT29700	0	0	45	1	0	0	0
EABT29701	2	1	11	0	1	8	3
EABT29702	8.01	42	0	0	1	6	0
EABT29703	0	0	0	0	0	0	0
EABT29704	3	0	1	2	4	0	18
EABT29705	1	1	8	1	0	1	0
EABT29706	0	0	4	1	0	0	1
EABT29707	0	9	19	0	0	0	0
EABT29708	1	0	1	1	0	1	1
EABT29709	2	5	12	2	0	2	0
EABT2971	2	7	71	1	2	6	1
EABT29710	0	2	1	0	5	0	0
EABT29711	40	82	129.01	51.94	133.43	29	52
EABT29712	65.58	245.5	157	436.13	56.12	23	35
EABT29713	0	3	6	1	0	0	0
EABT29714	6	3	10	10	2	3	1
EABT29715	0	1	6	23	0	0	0
EABT29716	3	6	20	8	1	1	1
EABT29717	0	1	3	1	1	0	1
EABT29718	1126.95	886.4	475.16	200.68	436.42	1587.2	915.77
EABT29719	0	2	2	1	2	0	0
EABT2972	284	292.97	332.16	515.92	110	48.89	110.05
EABT29720	106	67	7	18	6	357.97	103.83
EABT29721	5	11	7	1	4	0	0
EABT29722	0	0	43	0	0	0	0
EABT29723	0	1	5	0	6	0	2
EABT29724	658	945	674	1218.67	825	972	492
EABT29725	4	0	23	11	3	2	6
EABT29726	1	1	10	2	1	0	0
EABT29727	666	842.48	1673.87	1704.45	540.99	292.99	237
EABT29728	501.75	990.16	1779.58	2949.06	1059	311	566
EABT29729	2	0	2	0	0	0	1
EABT2973	0	1	2	0	2	1	0
EABT29730	0	1	4	2	0	1	0
EABT29731	4455.99	5575.98	4246.15	7088.26	3151.72	4189.1	3528.61
EABT29732	17	68.91	17	8	3	6	1
EABT29733	1	5	14	3	0	0	1

EABT29734	1375	1766.43	244	317	964	725	1081.12
EABT29735	1	3	3	2	0	2	2
EABT29736	1	3	5	7	0	1	7
EABT29737	4	14	3	11	3	1	1
EABT29738	0	7	11	2	1	0	0
EABT29739	1824.44	1513.14	874.87	1308.46	1043.31	1073.72	796.96
EABT2974	71	292.52	263	717.02	450	63.01	37
EABT29740	52608.9	42110.9	604	3	19	2	0
EABT29741	0	0	8	0	0	0	0
EABT29742	0	1	11	0	0	0	0
EABT29743	0	0	1	0	5	0	7
EABT29744	1	9	28	4	3	3	0
EABT29745	0	0	9	0	1	2	1
EABT29746	0	0	5	0	0	0	0
EABT29747	0	1	12	1.04	0	0	1
EABT29748	1	2	30	4	4	1	1
EABT29749	0	2	39	3	0	0	1
EABT2975	3	2	6	13	4	1	0
EABT29750	0	0	2	1	0	0	1
EABT29751	17.04	28	137	30	4	26	7
EABT29752	2311.38	4128.78	4703.01	5257.66	1769.29	650.96	1300.58
EABT29753	0	0	6	0	1	0	0
EABT29754	1	2	2	0	0	1	2
EABT29755	6521.05	13446.8	2475.02	30	2224.18	839	4343
EABT29756	0	1	17	3	3	0	1
EABT29757	2	6	0	0	1	3	4
EABT29758	2128.79	4990.97	1559.01	641.98	408	2454.54	2018.62
EABT29759	2	8	2	9	1	0	1
EABT2976	1	2	2	3	0	1	0
EABT29760	0	1	8	0	0	0	0
EABT29761	4	13	4	2	5	2	6
EABT29762	0	1	5	1	5	0	2
EABT29763	0	3	1	5	1	0	1
EABT29764	0	0	2	1	1	0	0
EABT29765	52	2	6	2	3	7	1
EABT29766	1	2	7	0	1	0	0
EABT29767	995.7	1557.04	1321.45	3195.78	1261.36	852.64	1016.3
EABT29768	2	3	11	12	0	0	0
EABT29769	2	2	3	0	0	3	10
EABT2977	0	1	1	2	1	0	0
EABT29770	0	2	33	6	1	0	0
EABT29771	7	12	13	0	5	0	0
EABT29772	1	3	1	4	2	0	2
EABT29773	0	1	11	0	0	1	1
EABT29774	708	956.97	2430.69	3296.41	2274	409	643
EABT29775	1	4	4	0	0	3	1
EABT29776	2	4	4	1	0	3	2
EABT29777	0	0	6	0	0	0	0
EABT29778	0	1	3	0	0	0	1
EABT29779	14	17.94	3	10	2	0	4
EABT2978	8	7	28	18	6	1	2

EABT29780	786	972	1667	1818	1081.96	378	653
EABT29781	0	0	6	0	0	0	0
EABT29782	1	7	22	5	0	0	0
EABT29783	0	1	1	2	0	1	3
EABT29784	2	7.39	45	5	5	2	10.99
EABT29785	209	443	441.08	268.05	293	150.45	225
EABT29786	236	233	191	283	433	210	208
EABT29787	0	0	5	1	1	0	0
EABT29788	0	0	0	0	6	1	14
EABT29789	1	0	1	1	0	0	0
EABT2979	1	3	2	3	1	1	0
EABT29790	2	4	3	12	2	1	1
EABT29791	0	0	17	0	0	0	0
EABT29792	0	2	3	0	0	0	1
EABT29793	1	1	10	1	0	1	1
EABT29794	458	170	0	1	0	87	7
EABT29795	0	1	10	0	0	0	0
EABT29796	15	17	2	3	1	1	0
EABT29797	1	2	38	7.83	2	1	1
EABT29798	0	1	2	2	0	0	0
EABT29799	36	172	558.04	23	22	4	8
EABT298	0	4	1	9	2	0	0
EABT2980	0	0	1	0	2	0	1
EABT29800	0	1	8	2	0	1	1
EABT29801	4	0	27	7	3	0	0
EABT29802	1	1	7	8	0	2	4
EABT29803	423.36	1375.11	2428.1	1015.87	83	121	260
EABT29804	0	3	10	3	0	2	0
EABT29805	5	11	55.95	6	7	8	6
EABT29806	2	16	2	16	2	0	0
EABT29807	0	0	3	4	1	0	0
EABT29808	3	1	53	6	4	2	1
EABT29809	10	17	26	29	10	24	19
EABT2981	1	4	11	0	9	5	2
EABT29810	0	0	8	0	0	0	0
EABT29811	0	0	3	1	1	3	0
EABT29812	0	0	3	2	0	0	0
EABT29813	1	0	3	5	2	0	1
EABT29814	0	1	1	5	0	0	0
EABT29815	0	1	4	0	0	0	1
EABT29816	0	2	1	2	0	0	0
EABT29817	1	0	8	3	0	3	4
EABT29818	1	0	5	1	0	0	3
EABT29819	1	1	1	0	1	0	0
EABT2982	231.16	170.83	35	0	84	93.38	206.82
EABT29820	0	0	6	5	0	1	0
EABT29821	0	3	56	0	0	0	0
EABT29822	0	0	3	1	1	4	7
EABT29823	0	5	104.52	45.84	58.93	2	20
EABT29824	2953.5	4225.81	5398.69	9132.68	2866.9	3499.45	3221.94
EABT29825	11	13	68	20	18	6	41

EABT29826	6405.93	8766.84	7842.84	13750.6	5938.98	6770.63	4573.81
EABT29827	0	0	3	0	0	0	0
EABT29828	1451.85	3196.01	3658.24	7092.93	3510.27	1297	2239
EABT29829	2	9	0	7	4	1	5
EABT2983	0	1	10	7	0	1	0
EABT29830	2	6	18	4	3	6	1
EABT29831	27	81	52	76	45.99	1	61
EABT29832	2	5	3	5	0	0	3
EABT29833	4	5	1	1	0	7	0
EABT29834	1205.74	1229.81	2489.99	1827.51	1962.68	369.22	871.22
EABT29835	7	19	67	11	4	10	4
EABT29836	0	1	2	0	0	0	0
EABT29837	2	4	3	6	1	0	2
EABT29838	0	0	2	2	0	0	0
EABT29839	1	1	5	3	0	1	4
EABT2984	1	1	4	3	0	0	4
EABT29840	0	1	7	1	0	0	0
EABT29841	3	4	13	8	2	0	1
EABT29842	1	1	0	2	0	2	0
EABT29843	0	0	6	1	0	0	0
EABT29844	1800	2515.52	2311.93	3313.57	1830	1094	1432.75
EABT29845	0	0	24	0	0	0	0
EABT29846	2701.51	3094.37	1677.71	2724.83	1445.61	2848.89	2559.24
EABT29847	0	0	8	0	0	0	2
EABT29848	1	3	38	1	5	6	1
EABT29849	1	0	3	0	0	0	0
EABT2985	2	3	0	9	0	0	0
EABT29850	0	4	24	2	0	0	0
EABT29851	0	5	19	6	4	0	4
EABT29852	34	39	75.99	16	2	0	0
EABT29853	8	28	45	39	11	14	15
EABT29854	12	5	1	7	6	5	16
EABT29855	4	1	8.07	14	7	0	0
EABT29856	15	14	26	38	6	2	13
EABT29857	5	8.99	45	9	1	1	4
EABT29858	3	12	56	14	1	3	2
EABT29859	0	0	13	0	0	0	0
EABT2986	0	0	0	5	0	0	0
EABT29860	1	2	8	2	0	0	0
EABT29861	12	72	188	254	10	0	1
EABT29862	2	0	9.67	1	0	1	0
EABT29863	144	446.53	268.82	147.93	130.04	2	31
EABT29864	0	0	1	25	0	0	0
EABT29865	2	4	6	2	1	0	2
EABT29866	1	1	0	0	0	0	0
EABT29867	0	6	0	0	0	1	0
EABT29868	0	0	1	3	2	0	5
EABT29869	2	1	1	4	1	0	1
EABT2987	4	10	38	85	5	2	6
EABT29870	3	4	7	1	4	0	0
EABT29871	0	3	12	1	0	1	2

EABT29872	0	0	6	1	1	0	0
EABT29873	1	1	36	0	2.32	4	4
EABT29874	2	1	0	0	1	1	0
EABT29875	1	6	37.03	7	0	0	1
EABT29876	33	13	14	162.11	7	3	13.96
EABT29877	10	12	10	2	0	8	2
EABT29878	1143	2113	1839.12	3157.18	1269	1248.99	2759.96
EABT29879	26	28	1	0	0	0	0
EABT2988	1	0	1	5	0	0	0
EABT29880	1	1	3	1	1	0	1
EABT29881	0	0	0	2	6	0	1
EABT29882	0	0	0	0	0	0	0
EABT29883	0	1	5	0	0	0	0
EABT29884	0	2	40	2	0	2	1
EABT29885	1	3	1	0	1	0	3
EABT29886	63	71	89	9	11	23	24
EABT29887	37	0	1	0	1	0	0
EABT29888	0	0	0	0	0	9	0
EABT29889	1	6	4	11	2	3	0
EABT2989	1892	10326.5	412.98	18036.4	1293.08	1127.22	1290.58
EABT29890	0	0	7	0	0	0	2
EABT29891	0	2	4	0	0	0	0
EABT29892	2	1	1	0	3	0	5
EABT29893	0	2	4	3	2.71	0	0
EABT29894	3	4	0	9	3	0	2
EABT29895	9	18	34	304	22	1	1
EABT29896	2308.02	3357.95	2152.64	6168.48	1981.43	1391.01	2160.34
EABT29897	9	7	8	17	4	1	2
EABT29898	1	10	30	1	1	5	1
EABT29899	0	2	10	0	0	0	1
EABT299	0	2	8	1	0	0	0
EABT2990	0	0	2	0	1	0	0
EABT29900	1270.89	2138.53	2758.57	4120.2	2111.93	1535.85	1709.9
EABT29901	2	4	91	4	2	1	2
EABT29902	0	4	4	1	0	0	1
EABT29903	10	13	6	57.1	18	0	18
EABT29904	651.3	1205.95	2160.23	4133.78	3400.53	332.06	1331.81
EABT29905	13288.1	36387.7	53.34	5	3716.74	6690.27	4597.95
EABT29906	1152.94	2836.03	3125.87	6251.3	2228.67	1101	1318.97
EABT29907	0	1	0	0	0	2	3
EABT29908	2	1	6	5	3	1	1
EABT29909	66	91	73	21	33.49	122	169
EABT2991	0	0	1	0	0	0	0
EABT29910	83	158	153	435.82	644.13	82.04	106.68
EABT29911	1	2	3	0	0	1	0
EABT29912	1	0	6	2	0	0	0
EABT29913	2	7	10	6	2	13	1
EABT29914	1327.92	2077.8	2248.87	4295.66	1886.16	950.24	1064.06
EABT29915	30	9	180	19	151	151	36
EABT29916	38952.1	21015.6	7528.14	433	2813.34	52	4182.4
EABT29917	556.41	824.85	637.19	1837.58	677.84	276	445.13

EABT29918	381.76	1665.14	3871.96	1516.48	2081.42	580.99	1179.23
EABT29919	24	18	0	0	0	0	0
EABT2992	12	15	0	2	3	0	0
EABT29920	3	7	26	5	3	2	0
EABT29921	1	4	2	2	1	1	0
EABT29922	78	149	157	670.36	71	32	24
EABT29923	0	0	13	0	0	0	0
EABT29924	5	0	2	18	0	4	4
EABT29925	3	1	6	1	0	1	1
EABT29926	412.8	883	4875.05	596	1134	105	349
EABT29927	0	2	9	2	2	1	7
EABT29928	0	3	3	1	1	13	4
EABT29929	0	0	0	0	0	12	4
EABT2993	21	17	25	5	12	1	2
EABT29930	4045.87	5441.79	5581.19	2352.23	2645.09	2347.01	2566.51
EABT29931	19	62	53.01	132	269	10	266
EABT29932	5	6	5	9	2	2	0
EABT29933	1	0	3	3	0	0	0
EABT29934	2	0	7	0	0	7	7
EABT29935	1	2	1	3	0	0	4
EABT29936	4828.6	5404.06	12647.6	6410.14	5205.06	4398.77	5317.85
EABT29937	1	0	6	3	1	0	0
EABT29938	3	25	31	26	11	6	3
EABT29939	33	114	398.96	59	53	5	63
EABT2994	1	3	1	9	1	1	1
EABT29940	2	23	51	21	3	1	24
EABT29941	0	0	0	4	0	0	0
EABT29942	1	2	1	0	0	0	0
EABT29943	7	11	2	20	8	2	11
EABT29944	325.99	606.46	949.02	599.08	785.25	298.03	471.66
EABT29945	1	2	36	0	0	6	1
EABT29946	597.14	1327.14	3123.22	3793.72	1686.15	676	833.53
EABT29947	13	36	34	49	53	9	18
EABT29948	1	1	17	5	0	0	0
EABT29949	3	9	42	5	0	12	1
EABT2995	2	3	12	6	1	1	0
EABT29950	0	4	7	0	1	0	0
EABT29951	1	2	4	31	2	1	1
EABT29952	114.75	690.83	1704.1	179.83	254	551.99	374.85
EABT29953	98	202	236.02	2990.54	216.12	43	362
EABT29954	0	0	1	0	0	0	0
EABT29955	0	0	8	0	0	0	0
EABT29956	0	0	6	2	0	0	0
EABT29957	2	0	0	5	0	0	0
EABT29958	0	0	0	0	7	2	0
EABT29959	637.87	3492.54	747.06	1370.21	1128.28	2242.62	6457.54
EABT2996	897.07	1722.72	3156.55	1166.6	2349.64	407.92	555.41
EABT29960	5167.65	1943.29	79	93.99	347.19	6440.9	4444.41
EABT29961	0	0	7	0	0	0	0
EABT29962	0	0	6	4	4	0	2
EABT29963	10	6	36	3	1	4	1

EABT29964	4553.84	4391.9	2235.87	3896.1	1577.18	3828.54	1655.77
EABT29965	1	2	2	2	0	1	1
EABT29966	2	1	2	2	0	1	0
EABT29967	0	4	6	0	0	0	0
EABT29968	2	3	0	0	1	1	2
EABT29969	0	1	21	4	5	1	0
EABT2997	9	4	4	12	2	0	8
EABT29970	0	0	13	1	0	0	0
EABT29971	0	0	1	0	3	0	3
EABT29972	3	8	104.59	6	10	4	4
EABT29973	6	16	26	14	18	0	8
EABT29974	0	0	8	0	0	0	0
EABT29975	0	10	1	0	0	2	1
EABT29976	0	1	1	3	0	0	0
EABT29977	59	94	109	38	53	112	97
EABT29978	0	5	22	1	0	0	1
EABT29979	0	0	6	0	0	0	0
EABT2998	51	43	105.01	162.7	12	35	71
EABT29980	0	4	5	7	0	1	0
EABT29981	2243.57	2173.59	778.12	810.76	1090.38	1626.22	1698.6
EABT29982	2463.84	3839.78	1281.88	160	1478	4954.68	5528.03
EABT29983	166.03	266.01	41	7	10	15	15.02
EABT29984	0	0	5	2	1	0	0
EABT29985	1555.98	2918.01	5020.82	6301.69	2676.43	1631.1	1449.99
EABT29986	1	1	1	3	0	0	2
EABT29987	4	12	2	5	1	13	4
EABT29988	1	3	4	7	2	0	0
EABT29989	0	0	0	27	0	0	0
EABT2999	21	5	1	0	1	12	2
EABT29990	0	0	11	0	0	0	1
EABT29991	26793.5	19140.1	819	4	4	4787.9	7
EABT29992	0	0	14	0	0	0	0
EABT29993	1086.91	1628.67	1964.6	2080.38	1696.98	965.61	880.78
EABT29994	0	0	8	8	0	0	0
EABT29995	0	1	1	4	1	0	0
EABT29996	0	2	2	3	0	0	0
EABT29997	0	0	0	0	0	0	2
EABT29998	2	1	3	0	1	0	0
EABT29999	0	6	7	11	0	0	3
EABT3	1	4	1	15	0	0	1
EABT30	2	2	1	0	1	2	0
EABT300	6	8	7	5	1	17	7
EABT3000	0	0	3	0	0	0	0
EABT30000	1	2	59	6	2	5	2
EABT30001	2589.41	3477.71	9292.79	4906.06	2490.72	206	157.74
EABT30002	0	0	17	1	1	0	0
EABT30003	0	1	0	0	3	0	1
EABT30004	0	1	4	2	0	0	0
EABT30005	12.72	5	19	7	1	3	2
EABT30006	2	6	0	0	0	0	0
EABT30007	8007.15	3432.76	14.01	63.26	185	2782.74	1389

EABT30008	0	0	5	2	0	0	0
EABT30009	286.88	416.37	344.13	797.03	383.44	263.86	293.61
EABT3001	600	1473.01	104	2015.86	8904.1	112	113
EABT30010	0	1	2	42	0	0	0
EABT30011	243	5	54	411	90	0	0
EABT30012	158143	226714	42148.1	153226	91376	47.02	5088.54
EABT30013	0	0	0	1	3	0	4
EABT30014	17.99	83	22	2	4	79.31	25
EABT30015	0	0	4	1	0	0	0
EABT30016	0	3	202.54	129	45	1	17
EABT30017	1	0	8	0	0	0	0
EABT30018	0	7	3	7	57	0	0
EABT30019	9	4	0	0	2	3	0
EABT3002	1	0	0	1	2	0	1
EABT30020	76	67	1	28	19	0	2
EABT30021	578.18	745.75	929.3	1293.23	941.13	385.28	472.9
EABT30022	4	5	2	0	0	2	0
EABT30023	266.97	469.42	370.42	999.05	365	269	297.13
EABT30024	4	6	43	2	3	1	2
EABT30025	5	4	4	4	1	1	0
EABT30026	0	0	0	1	10	0	0
EABT30027	4	5	4	3	0	1	7
EABT30028	0	4	3	1	0	1	0
EABT30029	2	1	5	6	9	0	1
EABT3003	65.95	165	63	478.01	157.02	60	100
EABT30030	2	7	1	0	0	2	4
EABT30031	0	0	0	0	1	0	5
EABT30032	0	0	4	12	1	0	0
EABT30033	2	0	3	0	0	2	1
EABT30034	0	0	3	0	0	1	0
EABT30035	24	26	62	57	8	32	24
EABT30036	47	108	729.54	96.27	44	72	22
EABT30037	15	20	18	13	4	29	12
EABT30038	2	5	49	8	1	1	3
EABT30039	0	0	0	0	1	0	8
EABT3004	6	6	17.07	28	6	4	3
EABT30040	0	0	7	2	0	0	0
EABT30041	0	0	2	0	3	0	0
EABT30042	1188.01	1703.47	1475	2093.61	1301.07	1369.99	1123.86
EABT30043	133	260.03	333.89	1135.16	467	66.05	125
EABT30044	0	0	4	0	0	0	0
EABT30045	316.59	312	88	158	405	99	693
EABT30046	5	5	0	3	18	0	66
EABT30047	381.01	551.06	707.68	2067.05	561.15	178	106
EABT30048	1	0	6	3	0	0	0
EABT30049	0	0	0	0	11	93.03	65.01
EABT3005	0	1	7	0	0	0	0
EABT30050	0	0	50	0	2	0	0
EABT30051	1	2	0	44	8	0	0
EABT30052	138	244.04	590.63	427.2	118	85	60
EABT30053	2128.23	3262.33	1468.17	3527.65	1729.78	1865.8	2499.3

EABT30054	1	0	18	1	0	1	0
EABT30055	0	0	6	0	0	0	0
EABT30056	30	40	74	66	47	20	32.91
EABT30057	566.09	819.89	694.68	687.44	698.7	548.13	573.43
EABT30058	0	2	3	6	1	0	0
EABT30059	1	3	2	12	3	4	3
EABT3006	0	3	2	4	5	0	0
EABT30060	23	31	0	0	1	0	0
EABT30061	473	590	665	1130	747	562	529
EABT30062	17	27	25	13	29	0	4
EABT30063	1	0	13	0	0	0	0
EABT30064	0	0	15	4	0	1	0
EABT30065	3	25	25	27	11	8	9
EABT30066	5	5	4	11	1	2	3
EABT30067	340.77	481.95	346.06	753.61	546.97	392.18	309
EABT30068	2	3	11	5	1	0	1
EABT30069	0	0	7	2	0	2	0
EABT3007	2915.36	3856.35	3813.33	10061.5	2561.5	3106.25	2962.59
EABT30070	0	0	12	0	0	0	1
EABT30071	24	18	21	0	1	5	1
EABT30072	0	1	17	1	1	0	1
EABT30073	1	0	16	1	0	0	0
EABT30074	0	1	16	6	1	0	0
EABT30075	648	742.93	827	965	760.97	511	534
EABT30076	11	10	23	6	7	8	5
EABT30077	0	0	3	0	0	0	0
EABT30078	26	40	56	59	38	18	20
EABT30079	2	2	12	7	9	7	2
EABT3008	0	0	2	0	2	0	0
EABT30080	0	3	3	8	1	4	9
EABT30081	3	8	17	6	2	0	1
EABT30082	16	7	89	16	8	7	6
EABT30083	4	4	17	11	3	1	3
EABT30084	0	1	1	0	1	2	0
EABT30085	26	32	98	49	3	3	1
EABT30086	0	0	1	1	3	1	1
EABT30087	0	1	1	0	9	5	14
EABT30088	0	3	11	3	15	0	4
EABT30089	2	7	13	0	0	0	0
EABT3009	1	5	5	2	0	0	0
EABT30090	0	1	0	1	21	1	14
EABT30091	0	2	15	1	0	0	0
EABT30092	0	1	5	1	0	1	1
EABT30093	0	4	0	1	0	0	0
EABT30094	0	2	4	6	2	1	0
EABT30095	2827.32	4577.22	5048	9175.45	2674.32	2227.99	1979.74
EABT30096	0	0	0	0	2	0	3
EABT30097	0	1	6	4	0	2	0
EABT30098	2	3	14	25	3	1	13
EABT30099	0	0	6	0	0	0	0
EABT301	426.45	536.28	537.28	931.75	549.28	494.78	623.52

EABT3010	1	0	13	0	0	0	0
EABT30100	7	7	3	5	2	2	8
EABT30101	1	9	4	23	0	0	1
EABT30102	0	0	5	1	0	0	1
EABT30103	5	3	50	4	4	0	2
EABT30104	0	6	2	8	0	2	0
EABT30105	1	2	0	0	0	0	0
EABT30106	4	5	1	5	5	0	6
EABT30107	3001.03	2181.88	1395.25	2576.94	1124	2489.75	1608
EABT30108	0	1	5	5	0	1	0
EABT30109	1	1	95	15	10	0	0
EABT3011	0	0	1	1	1	0	1
EABT30110	0	2	1	1	1	0	0
EABT30111	3	4	5	5	0	0	0
EABT30112	20	0	1	0	0	21	0
EABT30113	2	4	24	11	1	3	9
EABT30114	38	7	32	48	2	0	1
EABT30115	2	4	29	2	1	23	19
EABT30116	0	7	0	2	2	0	0
EABT30117	4	30.98	25	3	0	3	1
EABT30118	725.01	736.24	312.01	1006.09	528	525	434
EABT30119	0	3	1	4	2	0	0
EABT3012	0	0	0	0	0	0	0
EABT30120	8	18	51	38	6	7	5
EABT30121	313.01	352.46	152	515.83	186	129	277.01
EABT30122	0	0	5	0	0	2	0
EABT30123	1	3	2	5	0	0	0
EABT30124	0	0	20	1	0	0	0
EABT30125	14	34	17	9	6	13	41
EABT30126	48130.6	206077	88026.5	168420	64599.4	65247.1	61491.6
EABT30127	1443.17	1541.39	1050.68	1729.72	1391.88	631.02	711.92
EABT30128	0	3	16	2	0	2	0
EABT30129	5	44	57	56	63	2	8
EABT3013	74	301	234	103	478.52	530	1032.01
EABT30130	0	3	3	4	0	0	0
EABT30131	427.98	814.36	562.98	2874.57	877.01	430.12	1077.9
EABT30132	6	6	1	0	0	16	8
EABT30133	0	1	36	13.93	1	1	3
EABT30134	322	202	179	71	78	51	1001
EABT30135	2	7	0	0	0	12	7
EABT30136	0	3	66	4	0	0	3
EABT30137	978.19	1033.55	632.04	24159.4	5692.34	169.01	212.1
EABT30138	2	2	17	4	2	0	1
EABT30139	3	6	5	0	1	1	5
EABT3014	6	10	6	0	5	1313	28
EABT30140	0	0	11	1	2	0	0
EABT30141	0	0	1	8	0	0	0
EABT30142	3	2	8	0	0	6	6
EABT30143	0	0	10	0	0	0	0
EABT30144	3054.9	4342.92	2457.2	2897.04	2817.28	1988.14	7017.15
EABT30145	34	96	56	162	21	35	43

EABT30146	1	0	29	2	0	0	1
EABT30147	570.02	957.14	1056.42	1213.03	815.99	1113.03	488.08
EABT30148	0	9	3	3	2	0	1
EABT30149	0	0	13	0	0	1	1
EABT3015	1	3	1	1	0	0	5
EABT30150	207.19	897.37	1018.67	3591.94	557.03	39.54	245
EABT30151	0	3	0	0	4	1	4
EABT30152	0	0	11	0	0	0	0
EABT30153	5	9	25	3	1	1	0
EABT30154	0	6	3	0	0	0	0
EABT30155	4	10	27	10	2	5	6
EABT30156	0	1	4	8	0	0	0
EABT30157	945.99	1224.14	1723.97	2140.7	1300.69	750.4	832.99
EABT30158	39	54	113	1349.52	421	0	91.02
EABT30159	1	1	21	1	0	4	0
EABT3016	0	0	7	0	0	0	0
EABT30160	1177.4	2281.77	3486.17	5299.62	1856.39	1153.59	1470.04
EABT30161	10	1	0	31	84	0	1
EABT30162	0	1	107	0	2	0	4
EABT30163	329.11	596	1184	727	503.99	241	298
EABT30164	0	0	3	1	0	3	0
EABT30165	4	2	2	6	0	0	8
EABT30166	0	2	43	1	0	0	0
EABT30167	0	2	6	5	0	0	1
EABT30168	2	4	17	6	20	1	0
EABT30169	8044.99	11817.4	12099.9	12867.3	8216.03	11044.3	7082.24
EABT3017	1137	1834	2235	1951.02	1016	1001	816
EABT30170	33	49	15	9	4	182	27
EABT30171	23	192	0	0	1	31	0
EABT30172	2	5	3	20	7	1	3
EABT30173	0	0	0	2	0	0	0
EABT30174	234	279	283	936.94	405.02	150	222
EABT30175	570.96	780	894.52	1009	747	601.01	655
EABT30176	1	1	7	2	0	1	0
EABT30177	4461.19	2385.09	1375.25	4094.25	2146.34	9	483.31
EABT30178	3	2	10	1	1	0	0
EABT30179	1	1	3	8	15	1	2
EABT3018	2427.9	3352.3	1983.33	3649.03	1813.01	2492.7	2438.51
EABT30180	1	0	6	0	0	1	0
EABT30181	538.02	1039	4232.47	2588	1211	6	55
EABT30182	0	2	8	0	0	0	0
EABT30183	0	1	5	4	1	0	1
EABT30184	189.34	491.54	193.99	97.02	78.68	35	39
EABT30185	0	3	0	2.01	5.89	0	1
EABT30186	0	0	8.01	0	0	0	1
EABT30187	26	85	4916.51	14	127	3.09	9
EABT30188	0	2	3	2	0	0	0
EABT30189	61895.9	5650.6	28835.1	8538.63	5462.68	116467	23882.5
EABT3019	1	1	11	0	0	0	0
EABT30190	3	4	6	7	0	0	2
EABT30191	3	1	7	1	6	1	12.06

EABT30192	0	1	5	3.67	0	0	0
EABT30193	6	6	14	3	1	4	1
EABT30194	0	1	0	1	0	0	0
EABT30195	1	1	1	0	0	1	0
EABT30196	1	7	24	1	401	1	0
EABT30197	1	1	9	0	0	0	0
EABT30198	6	2	16	0	0	1	0
EABT30199	3	11	15.99	62	6	0	0
EABT302	768	1236	1014.52	2046	653	368	549
EABT3020	0	0	1	2	7	0	3
EABT30200	0	2	18	46	0	0	0
EABT30201	0	2	2	3	2	4	0
EABT30202	0	3	10	1	0	1	0
EABT30203	2	6	9	27	10	2	2
EABT30204	1238	1754.03	1081	1119	2255.07	218	583
EABT30205	102.2	0	0	0	0	13.88	0
EABT30206	1	7	13	14	0	2	2
EABT30207	0	0	1	0	0	24	3
EABT30208	27	82	152	47	27	69	170
EABT30209	1	6	3	6	1	2	2
EABT3021	61.73	153	220.85	507	810.6	63	125.58
EABT30210	483.78	818.93	786	1315.11	785.85	242.91	478
EABT30211	0	1	9	0	0	0	0
EABT30212	2	5	6	1	2	3	1
EABT30213	1	8	0	0	0	0	0
EABT30214	150	233	483.99	754	410	272	374
EABT30215	1357	2037.62	2573.09	4066.68	1485.16	2226.06	1536.75
EABT30216	0	0	19	1	1	0	0
EABT30217	9500.35	12220.9	31238.7	4139.41	1545.72	9157.23	4129.86
EABT30218	1217.93	2053.99	1811	3816.98	1742.44	940.83	1115.2
EABT30219	2113.38	3145.31	2318.01	6498.89	2746.66	2141.73	3905.36
EABT3022	9	8	12	7	2	9	6
EABT30220	1	0	2	15	1	0	1
EABT30221	2	4	3	12	0	1	6
EABT30222	0	0	3	0	0	0	0
EABT30223	3	4	39	0	0	0	0
EABT30224	0	0	5	0	0	0	1
EABT30225	531.33	1068.06	5522.07	58449.4	1707.98	4	7
EABT30226	0	0	7	3	1	1	2
EABT30227	1	9	5	2	0	0	0
EABT30228	1704.79	2818.7	1656.19	1602.8	1003.11	1919.45	1146.09
EABT30229	2	2	0	0	0	2	1
EABT3023	1	8	19	5	15	1	3
EABT30230	1	0	11	6	0	1	0
EABT30231	127	285	254	963	464	29	29
EABT30232	0	2	3	0	1	0	0
EABT30233	2	0	0	0	0	2	1
EABT30234	0	0	10	3	1	0	1
EABT30235	0	0	13	0	0	0	0
EABT30236	9	41	39	32	13	3	6
EABT30237	0	0	35	1	0	0	0

EABT30238	0	1	2	4	2	0	3
EABT30239	0	2	15	0	1	0	0
EABT3024	8	13	0	0	7	37	10
EABT30240	2	0	4	2	1	2	0
EABT30241	261.51	268	3	0	51	3	130
EABT30242	1	0	9	2	0	0	0
EABT30243	580.76	1118.33	976.04	2197.17	834.66	655.04	788
EABT30244	1	3	3	1	0	0	0
EABT30245	3	3	1	4	0	4	2
EABT30246	0	0	5	0	0	0	0
EABT30247	1439.85	3462.02	8362.68	4943.27	2400.5	275	1434.27
EABT30248	4	6	6	15	8	3	4
EABT30249	0	10	0	0	1	1	1
EABT3025	0	2	7	1	0	1	0
EABT30250	0	1	1	1	1	1	1
EABT30251	0	1	0	6	1	0	1
EABT30252	69	139.98	96	394.03	50	38	72.99
EABT30253	2	3	2	11	41	1	3
EABT30254	2	7	12	3	16	1	15
EABT30255	0	0	0	2	3	0	1
EABT30256	0	0	0	2	1	0	0
EABT30257	0	1	3	5	1	0	0
EABT30258	4	9	1	2	2	0	0
EABT30259	0	2	1	3	0	0	3
EABT3026	2829.91	4377.83	4377.63	5700.67	3770.6	3776.55	2809.95
EABT30260	63	165.49	277.93	318	325	101.98	143
EABT30261	0	0	0	0	0	0	0
EABT30262	0	0	6	2	0	0	1
EABT30263	0	0	0	0	1	0	0
EABT30264	802.42	1122.1	1059.96	2380.95	714.91	552.57	601.69
EABT30265	3	14	4	19	0	3	0
EABT30266	6.87	30.88	15	9	50	0	13.43
EABT30267	2769.59	4490.05	5180.07	6209.03	2571.35	1898.01	2113.01
EABT30268	0	0	6	0	0	0	0
EABT30269	5	4	7	19	5	0	0
EABT3027	10	4	0	0	0	3	7
EABT30270	1	2	4	1	0	0	0
EABT30271	2	4	4	4	0	3	0
EABT30272	2479.24	3765.64	2259.4	6758.32	2475.81	1841.11	2308.95
EABT30273	1101.01	2861.1	2390.15	5028.26	1965	915.96	1512.02
EABT30274	0	1	6	6	0	0	0
EABT30275	48	178.06	92	122	17	62.84	27
EABT30276	0	0	2	9	0	0	0
EABT30277	0	0	29	1	0	0	2
EABT30278	0	1	6	0	1	0	0
EABT30279	0	2	0	0	0	10	0
EABT3028	4	3	2	5	0	3	0
EABT30280	1	2	2	0	0	3	4
EABT30281	0	0	3	4	2	0	0
EABT30282	2865.89	13136.8	13947.9	6379.55	4844.6	436	2139.22
EABT30283	67	48	0	0	0	0	1

EABT30284	0	0	3	0	0	1	1
EABT30285	0	0	1	0	0	0	0
EABT30286	0	0	4	8	0	0	1
EABT30287	2	1	0	1	0	1	0
EABT30288	25	52	37	49	22	101	140
EABT30289	3	2	2	257	612	0	4
EABT3029	1	0	32	2	0	0	0
EABT30290	693	1469.88	804.46	1848.99	773.12	307	556
EABT30291	1	0	11	4	0	0	0
EABT30292	86	101	176	125	103	16	25
EABT30293	0	3	1	0	2	0	0
EABT30294	0	0	3	1	0	0	0
EABT30295	0	0	12	0	0	1	0
EABT30296	10	10	62	12	1	6	7
EABT30297	1	1	13	6	0	0	0
EABT30298	1	0	7	0	0	1	0
EABT30299	3	7	4	7	3	0	2
EABT303	9	18	4	35	8	0	1
EABT3030	2	5	3	0	5	0	3
EABT30300	1	6	45	1	1	2	0
EABT30301	769.87	452.07	83	83.01	109.1	60.92	131.57
EABT30302	0	1	11	0	0	0	0
EABT30303	0	1	0	0	2	0	0
EABT30304	0	4	6	1	0	0	1
EABT30305	101	86	110	209	95	48	46
EABT30306	1	17	16	56	9	0	3
EABT30307	156	597.43	412.55	1128.77	96.42	114	111
EABT30308	0	1	19	0	0	0	0
EABT30309	1	2	3	0	1	0	0
EABT3031	0	0	5	12	2	0	0
EABT30310	3	3	0	2	1	3	1
EABT30311	0	1	3	0	1	0	0
EABT30312	21.97	26	261	60	346.09	62	53
EABT30313	0	0	2	2	1	0	0
EABT30314	1	0	9	0	0	0	1
EABT30315	1	0	1	1	0	0	0
EABT30316	3	0	1	2	0	0	1
EABT30317	1	5	5	0	0	1	0
EABT30318	0	3	8	2	0	0	0
EABT30319	0	0	0	0	3	0	5
EABT3032	2	2	1	1	1	0	7
EABT30320	1	3	16	3	1	3	1
EABT30321	3	11.01	8	0	0	2	0
EABT30322	0	0	5	0	1	0	0
EABT30323	0	0	5	1	0	0	4
EABT30324	1	1	2	1	1	0	0
EABT30325	2	11	30	8	3	2	1
EABT30326	0	3	5	4	2	0	2
EABT30327	0	1	2	2	0	0	0
EABT30328	0	1	4	0	0	0	0
EABT30329	7	14	1	9	1	8	0

EABT3033	1592.84	4554.66	3127.73	3754.73	2083.78	1247.59	1072.58
EABT30330	0	3	10	12	4	0	2
EABT30331	0	2	1	16	2	0	0
EABT30332	4	11	3	5	0	0	3
EABT30333	1014.17	1447.98	1276	2497.18	881.47	913.66	1011.35
EABT30334	0	1	1	2	0	2	0
EABT30335	0	1	1	6	2	0	0
EABT30336	5	13	6	50.01	9	0	3
EABT30337	1	0	1	0	2	0	3
EABT30338	345	517.38	397	726	504	242.75	355.01
EABT30339	0	1	24	0	1	1	0
EABT3034	0	0	5	1	1	0	0
EABT30340	2	6	14	11	0	0	1
EABT30341	3	2	1	0	2	5	7
EABT30342	3	10	15	8	3	3	14
EABT30343	0	0	8	0	0	0	0
EABT30344	0	0	1	1	2	0	1
EABT30345	1	1	2	1	1	0	0
EABT30346	0	0	2	2	0	0	0
EABT30347	61	91	0	0	0	1	3
EABT30348	0	1	4	0	1	4	1
EABT30349	0	0	6	0	1	0	0
EABT3035	0	0	2	1	0	0	0
EABT30350	13	5	17	10	2	0	0
EABT30351	1	3	2	2	0	0	0
EABT30352	4	34	47	153	15	0	0
EABT30353	1502.01	4056.44	6843.24	3881.02	1004.67	43	468
EABT30354	1	3	8	9	0	0	0
EABT30355	1	1	0	2	1	0	0
EABT30356	7	6	10	10	2	18	14
EABT30357	4079.26	3559.92	520.95	1617.49	662.05	3988.97	786.02
EABT30358	0	0	3	2	0	0	0
EABT30359	4122.09	6936.32	7576.78	1010.99	1407.86	3338.39	2637.97
EABT3036	0	2	0	0	0	4	4
EABT30360	0	1	8	0	0	0	0
EABT30361	1	2	5	1	0	0	1
EABT30362	0	1	1	0	0	0	0
EABT30363	0	1	7	4	0	1	0
EABT30364	18	50	92	154	487.02	7	11
EABT30365	1	3	7	4	4	0	3
EABT30366	18	78	283	324	147	23	43
EABT30367	0	0	1	30.96	0	0	0
EABT30368	5	7	9	0	0	0	0
EABT30369	28	52	57	4	18	6	5
EABT3037	1	1	20	11	2	2	1
EABT30370	0	0	1	13	11	0	6
EABT30371	4	1	0	0	0	3	0
EABT30372	15	24	306	12	3	2	1
EABT30373	6	7	19	40	1	5	4
EABT30374	0	2	4	1	1	2	1
EABT30375	1	4	3	2	0	0	2

EABT30376	0	2	10	0	0	0	0
EABT30377	0	0	15	4	1	19.97	1
EABT30378	14	9	4	1	6	14	83
EABT30379	0	1	3	3	0	1	0
EABT3038	0	0	10	0	0	0	0
EABT30380	5	19	23	7	86	0	850
EABT30381	2	7	3	31	1	1	0
EABT30382	1134.97	1361	990	2886.02	964.6	978.94	710
EABT30383	3	0	3	5	3	0	2
EABT30384	0	0	3	1	0	0	2
EABT30385	0	0	73	69	1	1	0
EABT30386	2285.94	3946.46	1407.92	153.51	181.44	560	335.03
EABT30387	6	9	6	0	5	0	3
EABT30388	7	18	42	3	6	3	2
EABT30389	0	2	6	0	0	0	0
EABT3039	116	362	568.84	1060.16	457.98	21	138
EABT30390	1432.38	1506.08	522.55	1097.33	1648.96	1020.31	962.42
EABT30391	15	24	2	35	14	0	0
EABT30392	58	115	135	151	31	49	33
EABT30393	1576.86	1797	1574.4	2309	1821.2	1075.28	1131.21
EABT30394	1	1	9	1	0	0	0
EABT30395	0	3	2	0	1	0	0
EABT30396	0	0	20	0	1	0	0
EABT30397	50.99	146.84	274.75	166.67	32.05	68.03	41.94
EABT30398	0	2	7	3	0	0	2
EABT30399	4	0	1	0	1	1	0
EABT304	10	28	23	55	10	22	24
EABT3040	49	157	134	230	21	50	58
EABT30400	12	18	10	11	6	30	121
EABT30401	2	2	7	1	0	2	0
EABT30402	4	5	6	1	1	1	2
EABT30403	6129.95	5331.56	974.98	1478.99	3385.67	2154.47	4803.2
EABT30404	1	2	15	8	0	0	0
EABT30405	1	1	24	13	11	1	2
EABT30406	3813.6	6198.08	907.18	389	950.83	36	65
EABT30407	8	11	34	2	1	16	3
EABT30408	3	1	6	12	3	6	17
EABT30409	3419.49	6165.91	5151.16	4643.39	4640.42	2361.16	2965.31
EABT3041	5291.9	2242.97	114.66	102	220	13061.4	973.46
EABT30410	26	75	102	469	442	61	238
EABT30411	6	6	27	6	0	0	0
EABT30412	19.03	5	0	0	0	1	2
EABT30413	0	0	8	2	1	0	0
EABT30414	1	1	6	5	0	0	3
EABT30415	5	3	8	1	3	1	3
EABT30416	0	3	4	3	1	0	0
EABT30417	3	2	17	1	0	2	2
EABT30418	192.85	281.97	400.88	497.49	250.63	58	133.04
EABT30419	6.04	7	11	27	8	1	5
EABT3042	315.69	414	450	495	349	167	314
EABT30420	152	193	111.01	424	284	213	187

EABT30421	8	0	0	0	0	2	0
EABT30422	2	2	1	1	0	2	0
EABT30423	5	13	61	18	20	7	3
EABT30424	27	69	44.8	585	29	12	50
EABT30425	190.98	430.97	268	1405.85	471.56	65	131
EABT30426	8	10	0	0	0	1	2
EABT30427	0	1	4	1	3	0	1
EABT30428	24	21	30	56	19	33	13
EABT30429	0	0	1	0	0	0	0
EABT3043	2	7	61.2	4	2	11	7.94
EABT30430	0	0	1	0	0	1	0
EABT30431	0	4	8	4	1	4	0
EABT30432	19	10	44	19	5	0	0
EABT30433	194.01	444	68	125	10	1	3
EABT30434	1	5	2	1	3	0	2
EABT30435	466.99	660.41	879.37	1459.21	176	658.04	438.01
EABT30436	105	116	15	35	8	116	10
EABT30437	0	4	11	14	1	0	2
EABT30438	0	0	7	2	0	0	0
EABT30439	0	2	1	0	4	0	0
EABT3044	3	2	2	2	0	1	1
EABT30440	3	5	15	12	3	0	0
EABT30441	1	1	9	0	9	2	7
EABT30442	0	0	2	0	0	0	0
EABT30443	1	0	29	0	0	0	0
EABT30444	215	53	2	0	0	58	0
EABT30445	0	5	0	0	0	0	0
EABT30446	1	1	7	0	0	0	0
EABT30447	4090.84	1591.12	59	0	346.96	7343.01	2058.52
EABT30448	1	0	0	0	0	0	9
EABT30449	4	1	2	0	0	1	0
EABT3045	7	7	6	2	2	5	2
EABT30450	0	0	4	23	57	0	0
EABT30451	24709.3	13930.8	1509.07	632.81	6088.48	597.9	4052.81
EABT30452	0	0	3	6	0	0	0
EABT30453	2	7	16	10	1	5	5
EABT30454	0	1	5	7	2	0	0
EABT30455	633	948.8	580.14	2030.3	553.46	676.17	634.59
EABT30456	2	1	6	1	1	0	1
EABT30457	3	13	12	15	3	2	4
EABT30458	0	0	5	2	2	0	0
EABT30459	0	1	0	2	0	0	0
EABT3046	37	27	7	47	4	5	23
EABT30460	5	22	9	50	59	1	13
EABT30461	2	7	11	6	1	2	1
EABT30462	0	5	1	8	33	0	3
EABT30463	4	9	27	5	12	10.01	3
EABT30464	0	1	3	6	0	0	0
EABT30465	691.01	1073.03	939.69	1567.87	905	302	431
EABT30466	0	0	0	0	0	0	2
EABT30467	1	4	0	0	0	0	0

EABT30468	0	0	3	4	4	0	0
EABT30469	0	0	6	0	0	0	0
EABT3047	2	1	7	2	0	0	2
EABT30470	0	0	14	0	0	0	0
EABT30471	1	0	26	1	0	0	0
EABT30472	639	920.5	754.61	2007.96	468.22	715.01	1005.42
EABT30473	204	125	9	0	1121	201	5490.65
EABT30474	0	0	9	0	0	0	0
EABT30475	4257.02	7112.15	2067.55	9389.62	3073.01	4192.04	4333.88
EABT30476	2	7	20	0	0	0	4
EABT30477	0	2	2	0	0	0	0
EABT30478	0	1	1	2	0	0	1
EABT30479	203	390.48	559.02	1279.13	373	193.97	224.99
EABT3048	4	2	0	0	3	7	2
EABT30480	1	3	4	0	3	0	3
EABT30481	27	29	44	50	6	3	4
EABT30482	1601	2551.82	5476.59	6194.59	2696.87	1808.78	1710.63
EABT30483	1086.51	1563.9	1601.68	1967.59	1477.1	483.35	2791.71
EABT30484	6	7	0	0	2	20	8
EABT30485	0	3	24	2	0	0	6
EABT30486	4	6	5	3	0	0	1
EABT30487	0	2	7	0	0	1	2
EABT30488	0	0	5	0	0	0	0
EABT30489	0	19	57	417	1328.62	1	9
EABT3049	10	18	67	40.07	1	1	3
EABT30490	0	3	1	1	2	0	2
EABT30491	2718.91	9200.63	5325.02	2659.95	3894.13	32	1033.05
EABT30492	2	4	5	0	0	0	0
EABT30493	310.27	1688.41	1271.66	1698.03	1440.97	870.81	972.81
EABT30494	703.51	1175	611.99	1863.33	788	257	524.93
EABT30495	1	6	16	3	1	2	0
EABT30496	0	0	2	0	0	0	0
EABT30497	1	3	7	3	1	1	0
EABT30498	0	2	2	2	1	1	1
EABT30499	142	254	123.97	102	20	553.91	31
EABT305	0	3	8	4	1	0	1
EABT3050	0	4	28	0	0	0	2
EABT30500	0	2	2	0	0	0	0
EABT30501	2	3	1	0	0	0	0
EABT30502	0	2	2	9	1	0	0
EABT30503	8	21	82.99	2	3	4	0
EABT30504	323.52	689.9	271	247	52	121	45
EABT30505	1	0	1	0	0	2	3
EABT30506	447	847	545.72	3728.88	732.37	7	29
EABT30507	27.99	78	59	69	1	24	7
EABT30508	0	0	20	4	1	0	0
EABT30509	4300.09	4589.19	2890.35	2171.11	1383.71	3291.94	2262.19
EABT3051	1	0	1	1	0	7	0
EABT30510	11	46	281.95	21	99	5.27	11
EABT30511	0	0	6	0	0	0	0
EABT30512	3	0	2	0	0	0	1

EABT30513	11940.8	2209.45	471	88	345	3124.95	982.07
EABT30514	30	9	84	9	4	85	27
EABT30515	3914.23	5742.75	8067.6	12379.6	5113.83	4633.35	3947.53
EABT30516	80	250	678	1117	391	1	8
EABT30517	0	2	10	0	0	0	0
EABT30518	17	17	8.83	0	0	62	0
EABT30519	3684.42	2431.14	178	17	446	3393.99	2509.91
EABT3052	1	0	4	2	1	0	0
EABT30520	1064.16	1443.28	1519.02	2244.24	1027.99	819.29	771.84
EABT30521	43	74	35.06	6	15	40	67
EABT30522	1	0	8	6	8	0	11
EABT30523	12	29	13	29	11	2	38
EABT30524	1	4	88	3	2	3	1
EABT30525	0	0	6	6	0	0	0
EABT30526	0	4	0	3	5	0	0
EABT30527	2	1	16	2	0	0	1
EABT30528	3	0	0	0	1	4	4
EABT30529	2633.63	1200.81	56	153	1249.55	159.36	796.85
EABT3053	4	6	25	4	2	4	12
EABT30530	0	2	16	5	0	0	0
EABT30531	0	0	1	9	0	0	0
EABT30532	0	0	8	0	0	0	0
EABT30533	0	1	4	0	1	1	0
EABT30534	1	2	20	4	4	1	3
EABT30535	6	20	19	201	11	3	8
EABT30536	0	0	12	1	1	0	0
EABT30537	1	4	29	12	0	0	1
EABT30538	7	10	12	70	17	2	0
EABT30539	72.91	200.88	242.9	216.92	332.38	76.08	308.11
EABT3054	1.01	6	200.27	8	1	1	1
EABT30540	0	2	8	3	0	0	3
EABT30541	0	0	5	5	0	0	0
EABT30542	7	7	8	0	20	0	0
EABT30543	15326	18517.8	16755.5	13202	11753.8	15025.9	13833.3
EABT30544	1	0	1	1	13	0	10
EABT30545	0	1	3	2	0	0	0
EABT30546	0	0	5	0	2	0	0
EABT30547	1	1	0	4	1	0	0
EABT30548	1765.07	2361.72	3773.91	4527.23	1428.43	1612.78	1148.37
EABT30549	11178.9	8890.88	7549.64	12776.7	3225.13	5230.8	3016.77
EABT3055	0	0	7	0	0	0	0
EABT30550	1	1	4	0	0	0	3
EABT30551	0	0	4	0	0	10	1
EABT30552	0	0	4	4	0	0	0
EABT30553	7	4	5	2	4	10	5
EABT30554	2035.91	2971.24	3197.99	7355	3003.64	2094.31	2110.81
EABT30555	850	1360	692	1864.04	1125.03	531	581.62
EABT30556	3	2	17	7	2	1	3
EABT30557	2	7	3	34	187	4	18
EABT30558	1	0	0	1	0	1	0
EABT30559	0	1	1	4	1	0	0

EABT3056	0	0	18	0	0	0	0
EABT30560	0	0	10	1	0	0	0
EABT30561	6	8	10	20	1	0	1
EABT30562	1	4	8	5	0	2	0
EABT30563	1	2	53	2	0	0	0
EABT30564	5	3	19.7	471	17	0	3
EABT30565	4	4	13	2	2	1	1
EABT30566	1	1	7	0	0	1	0
EABT30567	0	1	5	1	1	0	0
EABT30568	80	36	10	3	0	128	7
EABT30569	1	0	14	2	1	0	0
EABT3057	0	0	0	8	2	0	0
EABT30570	0	0	48	47698.6	2	0	0
EABT30571	0	0	3	2	1	0	0
EABT30572	0	0	6	1	1	0	0
EABT30573	2	2	3	3	1	1	0
EABT30574	0	1	2	18	6	0	10
EABT30575	757	1611.82	2126.98	5034.17	1420.99	1389.02	1393.59
EABT30576	8	17	16	6	8	10	3
EABT30577	1	2	4	4	0	4	2
EABT30578	0	5	5	4	0	0	1
EABT30579	2	0	12	0	1	0	1
EABT3058	3	6	8	3	2	3	0
EABT30580	2	13	13	15	0	0	0
EABT30581	0	0	1	0	1	0	1
EABT30582	226	545.05	2061.91	4427.53	348.18	85	116.71
EABT30583	0	2	3	4	0	0	0
EABT30584	0	4	6	15	1	2	2
EABT30585	2	10	7	39	41	5	37
EABT30586	1	0	9	1	1	1	0
EABT30587	0	0	6	0	0	0	0
EABT30588	0	1	4	5	0	0	0
EABT30589	3	2	0	0	0	1	0
EABT3059	0	0	18	5	1	0	0
EABT30590	443.1	1050.86	1050	2637.58	766.8	381.31	355.53
EABT30591	670	803	769.9	778	842	598	561.04
EABT30592	2	5	78	2	8	4	5
EABT30593	590.81	600.07	527.05	1205.53	528.12	770.79	456.29
EABT30594	258	467.16	512.99	1541	337	98	139
EABT30595	0	5	4	2	0	3	1
EABT30596	0	0	7	1	0	0	0
EABT30597	1	1	6	0	0	0	0
EABT30598	1	3	1	13	1	3	0
EABT30599	1	1	2	1	0	0	0
EABT306	21086	105211	65340.2	41079	49146.3	3153.23	16365.4
EABT3060	403	683.3	922.89	1484.93	656.98	291	311
EABT30600	0	3	18	0	0	0	0
EABT30601	0	0	16	0	1	0	0
EABT30602	721.78	1208	3035.84	2069.98	769.04	688.32	513
EABT30603	0	6	0	0	1	0	1
EABT30604	6	1	14	2	0	9	2

EABT30605	4	19	10	5	6	3	17
EABT30606	0	0	6	1	0	0	0
EABT30607	2	3	6	4	0	1	0
EABT30608	3	3	4.66	0	0	1	3
EABT30609	0	0	8	6	0	0	0
EABT3061	43	25	18	4	3	21	10
EABT30610	0	0	25	2	0	0	0
EABT30611	14	5	24	4	0	10	4
EABT30612	2	0	17	0	0	0	0
EABT30613	840.55	1355.01	953.41	1911.99	665.19	527.36	446.13
EABT30614	43.01	31	32	1	16	14	55
EABT30615	0	0	1	10	0	1	1
EABT30616	0	1	5	3	2	0	0
EABT30617	36.01	15	0	0	7	194	172
EABT30618	0	1	0	0	0	0	0
EABT30619	0	0	6	0	0	0	0
EABT3062	136	224	287.53	1055	656	102.86	212
EABT30620	0	0	0	19	2	0	0
EABT30621	6	6	4	4	3	0	6
EABT30622	0	0	0	3	0	0	0
EABT30623	1	4	11	5	3	0	10
EABT30624	12	15	18.28	24	2	6	0
EABT30625	651.74	777.93	967.31	1626	1481.23	193.59	621.7
EABT30626	0	0	7	5	5	1	0
EABT30627	0	1	11	0	0	0	1
EABT30628	0	5	10	4	3	5	4
EABT30629	1	10	27	8	1	1	0
EABT3063	0	0	26	2	0	0	0
EABT30630	780.78	1442.98	1256.88	2311.67	833.35	1059.71	809.63
EABT30631	571.12	750.11	766	1298.95	704.98	254.99	283.13
EABT30632	477.41	692.41	769.62	1071.44	273.19	429.99	369.46
EABT30633	3	3	5	12	0	4	2
EABT30634	0	6	4	2	0	1	0
EABT30635	9	27	137.13	74	48	0	7
EABT30636	0	4	4	2	0	0	0
EABT30637	437.03	771.7	1040.7	1414.5	623.36	153.72	214.06
EABT30638	99	168.58	173.07	996.21	187.69	83.16	126.74
EABT30639	1	2	6	2	1	0	3
EABT3064	1	4	2	0	0	0	0
EABT30640	5	5	73	6	33	8	36
EABT30641	0	0	0	4	0	0	0
EABT30642	0	2	3	3	0	0	0
EABT30643	45	37	8	8	16	13	29
EABT30644	0	3	8	0	0	0	0
EABT30645	0	1	11	0	0	0	0
EABT30646	2	2	1	2	0	0	0
EABT30647	0	3	4	0	0	0	1
EABT30648	1	0	17	0	0	1	0
EABT30649	3	4	18	12	4	0	2
EABT3065	0	0	1	77	1	0	0
EABT30650	507.01	760	983.1	1232	637.98	462	502

EABT30651	0	16	1	0	0	7	0
EABT30652	0	2	3	0	2	0	2
EABT30653	1	2	12	14	2	1	4
EABT30654	1	3.97	18	0	0	0	0
EABT30655	1	3	7	0	0	1	0
EABT30656	0	0	3	1	50	0	0
EABT30657	1	1	4	0	0	0	1
EABT30658	0	3	25	2	0	0	1
EABT30659	2	1	6	32	2	0	5
EABT3066	0	3	4	9	1	2	2
EABT30660	0	0	26	0	0	0	0
EABT30661	4	4	20	1	2	3	2
EABT30662	1	8	5	4	5	0	0
EABT30663	0	3	2	5	0	0	0
EABT30664	8	39	63	125	5	1	2
EABT30665	2577.2	3641.54	3888.69	11190.3	2699.54	2748.52	1474.97
EABT30666	4544.29	6548.7	4275.83	16931.8	2777.05	4177.55	1802.33
EABT30667	0	0	5	2	1	0	0
EABT30668	2	4	11	2	0	1	0
EABT30669	4	7	16	11	1	5	32
EABT3067	0	1	0	13	0	1	0
EABT30670	11	8	95	8	1	2	0
EABT30671	3	2	9	2	0	0	0
EABT30672	0	4	13	5	1	0	1
EABT30673	1118.18	1513.71	1671	2879.32	1063.73	756	1240
EABT30674	29	18	6	14	2	15	2
EABT30675	0	5	35	3	0	1	0
EABT30676	3	3	0	0	2	1	0
EABT30677	0	1	5	1	1	0	0
EABT30678	4	8	6	2	13	5	29
EABT30679	0	0	21	1	1	2	0
EABT3068	0	1	6	0	1	0	1
EABT30680	0	2	5	1	1	0	0
EABT30681	0	0	3	13	0	0	0
EABT30682	0	0	0	5	0	0	0
EABT30683	70	74	0	0	0	19	107.01
EABT30684	105	166.15	124.04	270.02	108	73	72
EABT30685	3	4	1	1	4	2	1
EABT30686	1	2	4	9	0	1	0
EABT30687	1	0	3	0	0	1	1
EABT30688	1	3	18	7	2	2	4
EABT30689	0	7	6	1	1	4	1
EABT3069	1	8	25	7	0	0	0
EABT30690	18	6	9	1	0	24	31
EABT30691	0	1	0	0	0	0	1
EABT30692	7	4	6	9	5	0	12
EABT30693	0	0	2	1	0	0	0
EABT30694	0	0	7	2	0	1	0
EABT30695	2402.94	3304.8	3297.23	4285.06	1259.09	1813.01	3701.1
EABT30696	42	1	0	0	0	3	0
EABT30697	0	2	3	1	0	0	0

EABT30698	26	113	367.54	116	40	33	19
EABT30699	63	117	42	98	120.21	5	60
EABT307	213	566	606.99	933.01	85	99	149
EABT3070	0	1	14	0	0	0	0
EABT30700	3	4	2	0	1	4	10
EABT30701	0	2	9	2	1	1	2
EABT30702	0	2	7	0	1	0	0
EABT30703	0	0	3	2	1	0	0
EABT30704	3	3	0	0	0	16	3
EABT30705	2188.09	5150.28	2896.83	3021.44	3929.96	1414.97	1515.97
EABT30706	3249.47	4861.62	3605.48	3850.08	2219.86	3394.96	2900.94
EABT30707	0	4	1	2	0	3	1
EABT30708	129	151	89	190	13	57	50
EABT30709	6	3	7	3	7	3	0
EABT3071	0	0	3	2	0	0	1
EABT30710	1	1	1	2	1	1	0
EABT30711	14	21	0	0	0	0	0
EABT30712	2	0	16	2	1	2	0
EABT30713	0	0	4	0	0	1	0
EABT30714	2918.37	4275.21	3382.93	3547.6	1800.45	1549.85	1479.45
EABT30715	4	6	31	11	2	7	1
EABT30716	0	0	10	1	0	0	0
EABT30717	0	0	2	0	0	0	2
EABT30718	0	2	6	0	4	1	0
EABT30719	5090.64	2920.32	2415.61	7233.59	1756.17	3566.44	1597.72
EABT3072	0	0	2	1	0	0	1
EABT30720	0	0	3	0	0	0	0
EABT30721	2	7	50	3	1	0	1
EABT30722	23	38.38	0	0	0	1	4
EABT30723	2	2	8	3	7	0	2
EABT30724	8	22	16	12	6	11	3
EABT30725	0	1	8	2	0	0	1
EABT30726	4	3	1	1	2	0	0
EABT30727	0	0	5	1	0	1	0
EABT30728	1	2	1	0	4	0	0
EABT30729	0	1	13	0	0	0	0
EABT3073	0	1	4	0	1	1	0
EABT30730	0	3	14	0	2	0	0
EABT30731	294	438.99	261.03	1468	797	310	302
EABT30732	0	1	9	0	0	4	0
EABT30733	7	15	40	34	2	5	10.02
EABT30734	6	3	0	0	0	0	1
EABT30735	14	8	2	0	5	37.82	45
EABT30736	79	56	90	29	2	29	2
EABT30737	2	4	2	4	0	1	0
EABT30738	244	311	395	802	457	177	187
EABT30739	0	1	5	1	0	0	0
EABT3074	2001.2	2670.12	1611.5	797	3777.02	209	639
EABT30740	2	5	4	3	0	0	0
EABT30741	703.42	951.27	445.93	2174.42	756.58	469.72	438.71
EABT30742	796.33	1465.53	1139	2730.47	1668.82	250	1805.81

EABT30743	1	1	5	1	1	1	0
EABT30744	1	0	16	7	4	0	3
EABT30745	0	0	2	3	3	0	0
EABT30746	2	8	14	17	7	1	3
EABT30747	0	1	5	1	2	1	1
EABT30748	37	45	45	59	28	9	10
EABT30749	0	0	1	4	1	0	0
EABT3075	798	1538.89	1956.96	2444	1182	414.97	533
EABT30750	11	7	6	12	2	0	0
EABT30751	125	139.03	24	289	31	187	40
EABT30752	3	5	0	0	1	2	1
EABT30753	1	5	3	0	2	0	1
EABT30754	0	1	3	0	0	1	1
EABT30755	9	15	15	9	2	4	1
EABT30756	0	1	6	0	2	0	0
EABT30757	2	4	3	5	2	0	0
EABT30758	209	272	136.97	502.84	385.99	104	225
EABT30759	1	0	2	0	5	0	0
EABT3076	13	42	4	14	1	4	1
EABT30760	0	0	5	0	0	0	0
EABT30761	4	6	15	12	2	2	1
EABT30762	5801.81	9149.34	2767.7	4184.83	9238.68	5186.81	7151.27
EABT30763	13	25	59	17	5	1	1
EABT30764	0	1	0	0	1	2	10
EABT30765	11	97.28	502	40.74	2935.85	51.26	6351.34
EABT30766	2	4	11	1	0	1	0
EABT30767	6639.16	9769.99	6394.04	11729.1	5547.31	7529.85	5578.7
EABT30768	0	0	17	11	0	0	0
EABT30769	1	2	9	1	3	1	0
EABT3077	326.68	369	204	208	87	115	151
EABT30770	0	0	8	0	0	0	0
EABT30771	2	1	26	1	1	3	1
EABT30772	2	2	5	4	0	1	1
EABT30773	0	0	5	0	0	0	1
EABT30774	2	7	0	2	1	0	5
EABT30775	210	90.11	51	131	4233.11	4	8
EABT30776	0	3	8	6	0	0	1
EABT30777	0	0	6	0	1	0	0
EABT30778	0	1	6	0	0	0	0
EABT30779	0	1	15	2	0	0	0
EABT3078	2	2	16	3	2	2	0
EABT30780	2	3	3	0	2	8	6
EABT30781	1	5	5	8	2	0	1
EABT30782	0	0	8	1	1	0	0
EABT30783	1	3	7	3	1	0	0
EABT30784	0	7	0	0	0	1	0
EABT30785	841	1106.54	1018.91	2779.83	1142	917.08	716.19
EABT30786	11	25	49	38	2	9	8
EABT30787	0	0	6	0	0	0	0
EABT30788	0	0	12	1	0	0	0
EABT30789	1	6	13	43	6	2	0

EABT3079	1	8	32	7	5	2	3
EABT30790	0	0	7	0	0	0	0
EABT30791	0	1	2	0	1	0	1
EABT30792	1	0	0	1	2	0	0
EABT30793	1537.57	2815.97	2664.73	2210.47	1253.91	1352.87	1457
EABT30794	3	5	5	1	4	0	0
EABT30795	2	3	1	0	0	5	0
EABT30796	17	20.76	35.91	10	1	6	4
EABT30797	0	0	2	4	0	3	1
EABT30798	1437.22	1854.93	859.56	1180.66	777.92	569.49	681.74
EABT30799	0	1	19	2	1	0	2
EABT308	0	2	1	0	0	0	0
EABT3080	0	2	1	4	0	1	0
EABT30800	7571.76	12637.3	2233.93	270.97	3422.04	20791.5	20272.2
EABT30801	2	1	13	3	0	8	2
EABT30802	0	2	9	0	0	2	4
EABT30803	0	0	4	0	0	1	1
EABT30804	46	74	127	107.95	38	16	10
EABT30805	13	33.09	373	100	86.92	2	7
EABT30806	0	0	10	0	0	0	0
EABT30807	2	15.32	25	6	1	6	6
EABT30808	0	2	9	2	0	0	2
EABT30809	0	0	4	0	5	0	0
EABT3081	851	1461.6	582.53	1300.85	419.17	475.96	443.32
EABT30810	4	0	4	0	0	0	2
EABT30811	745	1161	2619.25	1868.86	870.92	631.49	710.01
EABT30812	703.71	990.21	489	1091.7	1692.03	431	585.88
EABT30813	0	0	5	0	0	0	0
EABT30814	62	193	263	1642.53	681.56	26	44.99
EABT30815	3	5	12	73	9	0	8
EABT30816	1076.42	2688.39	1578.48	2566.35	1796.69	1621.34	1832.82
EABT30817	0	5	0	1	0	0	0
EABT30818	2490.09	1109.28	1541.65	127	506.47	1037.58	1703.22
EABT30819	1	3	8	3	0	2	7
EABT3082	0	3	0	5	0	1	0
EABT30820	0	0	3	2	0	0	0
EABT30821	1	2	6	4	0	0	0
EABT30822	4	8	22	14	3	3	5
EABT30823	0	0	4	0	1	0	0
EABT30824	0	0	0	0	8	1	1460
EABT30825	1	0	0	2	20377.1	60.3	320155
EABT30826	0	2	9	1	0	0	2
EABT30827	6289.11	7006.84	6716	10359.2	3352.14	6516.58	5092.59
EABT30828	357536	121527	269.01	2	28	67340.6	349.01
EABT30829	4	16	0	32	1	0	0
EABT3083	2	10.77	18	48	9	23	5
EABT30830	454.87	723	1406.7	991.14	940.58	205	183
EABT30831	1	2	4	1	1	2	0
EABT30832	10	9	24	14	11	5	10
EABT30833	2	48	2	11	34	0	1
EABT30834	0	1	5	0	0	0	0

EABT30835	3	6	8	23	7	4	7.85
EABT30836	2	1	2	0	0	1	1
EABT30837	8	5	0	0	9	0	0
EABT30838	0	0	0	0	1	0	0
EABT30839	215	322	169	428.79	264.01	216	131
EABT3084	0	0	19	4	0	0	0
EABT30840	2	11	4	0	1	0	0
EABT30841	0	4	25	18	0	0	1
EABT30842	810	980	406	208	240.92	1741.66	669.63
EABT30843	0	0	6	0	0	0	0
EABT30844	729	1167.02	1445.74	2510.49	543.2	441.03	408.11
EABT30845	4	5	1	3	0	0	1
EABT30846	0	1	2	0	0	1	0
EABT30847	6550.48	12701.9	9141.02	17949.6	6011.99	9010.88	8827.89
EABT30848	0	1	2	3	0	0	0
EABT30849	300	367	110	892.35	361	13	7
EABT3085	1	2	1	0	0	0	0
EABT30850	0	1	4	2	1	1	0
EABT30851	29	68	126.14	119	29	112	297.33
EABT30852	0	9	11	21	2	3	0
EABT30853	1	4	7	2	0	0	0
EABT30854	1	3	1	6	2	4	2
EABT30855	6	1	0	0	0	3	0
EABT30856	3219.3	4496.99	4321.69	11654.3	1771.12	2413	1602
EABT30857	1922.51	4539.98	5537.52	6819.7	2598.54	13	178
EABT30858	7	23	14	11	7	1	3
EABT30859	0	1	1	0	0	2	1
EABT3086	15	16	10	419	139	43	103
EABT30860	0	4	1	4	0	0	0
EABT30861	39	5	0	0	0	0	0
EABT30862	299	481	430	1077.02	406	180	256.98
EABT30863	6	38	29	29	10	0	13
EABT30864	17	21	53	200	160	5	50
EABT30865	120	167	351	650	179	0	97
EABT30866	74	77	13	28	50	74	121
EABT30867	6	5	42	3	4	4	1
EABT30868	0	0	8	0	0	0	2
EABT30869	0	2	3	0	1	0	2
EABT3087	1	3	1	0	1	0	3
EABT30870	698.65	860.68	1222.51	1129.14	491	582.48	684.94
EABT30871	1	1	0	1	1	0	1
EABT30872	5	7	11	5	4	16	17
EABT30873	0	0	5	0	0	0	0
EABT30874	1778.98	5339.22	4334.11	9929.78	2093.58	117.02	329
EABT30875	2	3	6	43	23	0	2
EABT30876	0	0	8	1	0	0	0
EABT30877	0	0	2	0	1	0	1
EABT30878	3	25	60	66	1	1	1
EABT30879	182.95	318.95	356.6	1045.23	414	145	174
EABT3088	1	0	13	2	0	0	0
EABT30880	6	12	27	25	21	2	8

EABT30881	4378.3	4385.36	3640.88	5340.71	2655.78	3615.04	1818.07
EABT30882	1428	1507	548	866.03	4009.95	1048.72	1096.01
EABT30883	5242.71	5556.96	3155.91	3809.64	3645.9	5937.95	4260.98
EABT30884	8	63	0	0	1	0	0
EABT30885	4	5	3	0	1	0	0
EABT30886	0	4	18	9	1	0	1
EABT30887	9	12	24.98	24	14	45	78
EABT30888	2	1	7	2	3	1	1
EABT30889	0	0	9	1	0	0	0
EABT3089	1	1	0	8	0	5	1
EABT30890	3	1	2	0	0	1	0
EABT30891	0	0	7	0	0	0	0
EABT30892	92	113.04	122	215.21	54	89.01	80
EABT30893	0	0	3	1	0	0	0
EABT30894	0	2	7	5	5	3	8
EABT30895	4	32	183.83	325	29	31	14
EABT30896	1	8	8	1	2	0	0
EABT30897	4	16	8	22	2	10	0
EABT30898	2	5	20	1	0	3	3
EABT30899	3	9	37	174	17	0	8
EABT309	0	0	3	0	0	1	0
EABT3090	107	153	128.03	224.07	64	48	65
EABT30900	0	2	13	0	0	0	0
EABT30901	3	3	38	6	8	1	5
EABT30902	3	16	6	18	9	2	3
EABT30903	1	3	9	8	2	0	1
EABT30904	2	4	6	2	0	0	3
EABT30905	18	23	8	13	2	50	15
EABT30906	1424.48	2340.51	2231.45	4390.67	1568.09	1449.53	1159.03
EABT30907	0	0	0	1	0	0	0
EABT30908	1	1	9	0	0	0	0
EABT30909	4	11	26	1	0	3	4
EABT3091	83529.6	35547.8	40.96	34.99	10	53624.3	24
EABT30910	25	35	11	16	18	2	6
EABT30911	1	0	2	1	2	1	0
EABT30912	2348.13	3260.34	1376.96	1820.19	892.79	976.99	766
EABT30913	60	156	261	1608.05	33	45	111
EABT30914	12	4	0	0	0	0	0
EABT30915	1	1	4	0	0	0	0
EABT30916	1	6	5	0	1	5	1
EABT30917	2	6	54	8	6	3	8
EABT30918	1245.95	1961.29	2970.09	2937.32	1416.07	1107.13	993.57
EABT30919	1	2	9	3	0	0	0
EABT3092	0	0	0	17	0	0	0
EABT30920	0	2	2	1	1	0	0
EABT30921	0	2	1	3	0	0	1
EABT30922	2	8	3	17	0	0	0
EABT30923	4	5	6	21	6	7	3
EABT30924	2	2	0	1	0	1	0
EABT30925	0	18	90	34	1	3	4
EABT30926	0	3	4	0	37	0	0

EABT30927	3	1	20	2	0	0	0
EABT30928	1	3	1	17	0	0	0
EABT30929	181	288	2	0	0	28	3
EABT3093	0	1	1	5	1	0	2
EABT30930	6	13	51	5	5	19	21
EABT30931	674.37	927	938	1290.21	989.04	662	511.3
EABT30932	0	2	5	0	0	0	0
EABT30933	10	16	0	0	0	3	6.96
EABT30934	4	19	41	44	7	25	8
EABT30935	31	17	3	11	4	0	1
EABT30936	152.63	562	1	0	4	212	26
EABT30937	0	2	19	0	1	1	0
EABT30938	1420.08	4862.26	1482.12	6353.8	3680.81	51	134
EABT30939	1	1	2	3	0	0	0
EABT3094	99.71	151.01	436.98	93	137	181.89	43
EABT30940	21	36	163.06	81	9	53	13
EABT30941	3	6	0	2.98	0	3	2
EABT30942	0	0	0	0	1	1	0
EABT30943	462.99	852.81	943.3	1903.67	397	389.89	258
EABT30944	0	0	4	0	0	2	0
EABT30945	2	5	3	2	9	0	0
EABT30946	11	18	254.99	14	11	20	16
EABT30947	0	0	3	1	0	0	0
EABT30948	936.14	1400.08	4612.78	3077.26	670	476.72	379
EABT30949	2	2	1	0	1	1	0
EABT3095	1	4	8	1	0	0	1
EABT30950	5	8	6	12	3	0	6
EABT30951	3	3	6	7	2	1	2
EABT30952	0	0	0	0	0	0	0
EABT30953	0	2	15	1	0	0	0
EABT30954	0	0	1	0	0	0	0
EABT30955	0	0	1	0	0	0	0
EABT30956	0	6	34	3	0	0	0
EABT30957	19631	18436.9	7966.26	26180.2	22097.8	35383.4	25628.7
EABT30958	303	1793.88	814.22	88	487	0	18
EABT30959	0	2	5	1	0	0	1
EABT3096	2	0	0	2	0	0	0
EABT30960	0	0	2	0	0	0	0
EABT30961	0	0	8	0	0	0	0
EABT30962	1	4	3	5	2	1	1
EABT30963	1	3	26	2	3	11	6
EABT30964	3	7	11	9	1	0	1
EABT30965	1	1	1	0	0	1	1
EABT30966	2	5	25	1	0	1	1.04
EABT30967	0	1	6	2	0	0	0
EABT30968	60	164.02	143.92	133.47	31.86	33	28.46
EABT30969	0	0	4	0	0	0	0
EABT3097	10	8	0	0	5	44	37
EABT30970	12	59	36	2	6	20	7
EABT30971	18	17	22	8	6	8	4
EABT30972	0	0	0	1	1	0	4

EABT30973	60.94	79.18	125.68	40	198.63	3.09	13.94
EABT30974	3	4	17	2	0	1	1
EABT30975	0	0	0	0	0	0	0
EABT30976	39	132.71	183.99	190	53	56.99	38
EABT30977	1	5	1	1	1	0	2
EABT30978	0	2	0	1	1	9	3
EABT30979	16	82	129	560	379	17	40
EABT3098	0	0	1	0	5	0	0
EABT30980	0	1	0	1	0	1	0
EABT30981	0	0	5	0	0	0	1
EABT30982	1	15	13	8	0	1	0
EABT30983	0	0	4	1	1	0	0
EABT30984	0	1	1	1	1	0	1
EABT30985	0	4	5	0	0	4	0
EABT30986	0	1	4	3	2	2	0
EABT30987	360	511	558	745.86	257.99	522.17	444.24
EABT30988	1	9	7	18	10	0	4
EABT30989	0	1	9	1	0	0	0
EABT3099	0	2	5	1	0	0	0
EABT30990	0	1	3	0	0	1	0
EABT30991	1	7	11	4	2	2	3
EABT30992	2	2	0	2	1	3	7
EABT30993	1	0	17	2	0	2	1
EABT30994	1641.66	1917.91	1204.01	1437.99	1165.85	994.04	892.28
EABT30995	30	60.97	50	82	15	45.81	54
EABT30996	1	1	6	1	0	1	0
EABT30997	1	2	5	9	0	1	0
EABT30998	5	2	23	1	0	8	10
EABT30999	0	0	3	2	0	1	0
EABT31	0	0	1	7	0	0	0
EABT310	93.09	149.74	167.64	264.03	99.42	85.89	129.63
EABT3100	6	7	16.97	10.18	3.79	23.97	9
EABT31000	2878.45	4244.22	4254.84	8073.22	4720.25	4575.23	2082.06
EABT31001	1	1	24	0	0	0	0
EABT31002	0	1	0	1	0	0	0
EABT31003	0	3	3	0	0	0	0
EABT31004	2667.95	4208.41	8161.79	8945.99	5649.81	2788.14	3357.97
EABT31005	1	0	5	2	0	0	0
EABT31006	0	4	0	4	0	0	0
EABT31007	1	5	5	8	1	0	1
EABT31008	0	0	11	0	0	0	0
EABT31009	0	2	6	12	0	0	1
EABT3101	4	4	0	6	0	1	0
EABT31010	0	1	8	7	0	0	0
EABT31011	16	19	30	21	4	10	12
EABT31012	5794.2	9172.1	8172.01	7408.05	9155.34	4919.13	8986.24
EABT31013	0	1	7	0	0	0	0
EABT31014	185	216.22	167	354.02	246	235.01	184
EABT31015	0	1	13	2	0	0	0
EABT31016	0	0	0	0	0	0	0
EABT31017	280	741	500.01	2840.54	239	37	52

EABT31018	9	9	7	46	3	1	0
EABT31019	81	78.01	9	7.12	3	17	8
EABT3102	0	0	6	0	0	0	0
EABT31020	19	20	3	7	2	5	0
EABT31021	0	4	14	1	0	0	0
EABT31022	0	1	7	1	0	0	0
EABT31023	2	0	9.98	0	0	0	0
EABT31024	4	1	0	0	0	0	6
EABT31025	2700.71	2451.09	500.97	722	606.61	1120.33	659.62
EABT31026	2	2	1	2	0	1	1
EABT31027	3	1	18	0	0	0	0
EABT31028	0	0	0	0	0	0	2
EABT31029	11	29	41	25	4	8	5
EABT3103	0	0	10	0	0	0	0
EABT31030	1111.97	1068.9	2080.59	4758.31	1381.03	957.59	959.01
EABT31031	0	0	6	1	0	0	0
EABT31032	1	9	16.88	1	1	0	0
EABT31033	0	2	2	2	1	0	0
EABT31034	51	58	41.04	220.99	91	17	96
EABT31035	1	9	61	0	2	0	3
EABT31036	0	0	6	2	0	0	1
EABT31037	1	3	5	0	1	0	0
EABT31038	1	0	17	2	0	2	1
EABT31039	7	25	59.56	10	11	39	16
EABT3104	4180.93	6209.79	4719.35	5212.58	3792.22	3328.46	3661.85
EABT31040	0	10	4	10	0	2	9
EABT31041	0	1	3	0	0	0	0
EABT31042	0	1	1	3	1	0	0
EABT31043	0	0	2	0	2	0	0
EABT31044	3509.31	3504.07	1060.92	1706.64	3496.17	2622.45	3076.01
EABT31045	2009.96	10700.7	15643.4	13224.8	20095.1	1377	3854.8
EABT31046	0	1	31	10	2	1	1
EABT31047	0	3	3	11	2	0	0
EABT31048	0	0	4	0	2	0	0
EABT31049	0	0	1	3	0	0	0
EABT3105	3	5	1	1	0	1	0
EABT31050	81	214	127	377	127	57	57
EABT31051	2	1	3	11	2	0	3
EABT31052	0	3.41	5	17	0	0	0
EABT31053	497.88	910	33.01	81.06	46	403	177.94
EABT31054	60	138	123	1139	232	6	93
EABT31055	405.11	474.99	139	833	328.2	286	327.12
EABT31056	9	13	28	5	17	0	4
EABT31057	5	14	42	10	1	1	0
EABT31058	4	12	68	4	1	0	3
EABT31059	0	2	0	1	1	0	0
EABT3106	3	8.24	61	41	23.29	4	1
EABT31060	285.02	577	183	1604.97	732	1	16
EABT31061	2469.59	3935.02	171.98	40	19803.6	6	66
EABT31062	2974.77	3974.63	2991.78	5254	3224.8	2103.98	2242.17
EABT31063	1	6	22	16	4	9	8

EABT31064	5	12	8	7	19	0	2
EABT31065	1	0	14	2	0	1	1
EABT31066	0	1	0	0	2	6	8
EABT31067	3	1	8	8	1	1	1
EABT31068	405.54	615.68	400.99	119	763.42	5	33
EABT31069	0	0	5	1	1	0	0
EABT3107	0	0	42	0	0	0	0
EABT31070	59	38	120.01	80	58	0	1
EABT31071	83	115.97	41	117	81	44	76
EABT31072	0	0	2	5	0	0	1
EABT31073	1	8	23	4	26.02	25	9
EABT31074	265	370	405	1014.99	188.01	211	102
EABT31075	4	2	1	2	0	1	1
EABT31076	0	0	0	1	0	0	3
EABT31077	0	0	17	0	0	0	0
EABT31078	41	143	444	267	264	126.01	44
EABT31079	0	3	9	1	1	0	0
EABT3108	6	26	172.99	48	1	6	1
EABT31080	0	0	0	0	0	0	2
EABT31081	0	1	8	2	0	0	0
EABT31082	2	10	77	1	2	0	2
EABT31083	1	2	1	5	1	0	0
EABT31084	3	0	6	0	2	0	0
EABT31085	0	2	2	0	0	0	0
EABT31086	0	0	0	3	2	0	0
EABT31087	0	1	0	0	0	5	0
EABT31088	1	9	10	7	1	2	3
EABT31089	0	1	8	4	1	0	0
EABT3109	3	0	15	3	0	0	0
EABT31090	2	0	12	0	2	0	1
EABT31091	124.66	178	14	0	13	21	78
EABT31092	12	11	13	49	7	10	6.02
EABT31093	6	1	2	0	0	0	0
EABT31094	0	3	14	11	0	1	0
EABT31095	0	1	9	1	3	0	2
EABT31096	1	1	1	3	2	0	0
EABT31097	147.95	394	1402.05	641.99	35	106.76	129
EABT31098	1	0	2	1	0	4	1
EABT31099	4	7	7	12	10	1	2
EABT311	6	11	0	0	2	0	1
EABT3110	1	5	17	2	1	0	1
EABT31100	0	0	30	0	0	0	0
EABT31101	2	3	3	0	0	0	8
EABT31102	1	2	5	0	0	0	0
EABT31103	0	0	4	2	2	0	0
EABT31104	2	3	4	1	0	2	1
EABT31105	20	8	1	15	12	8	10
EABT31106	2	1	0	1	0	0	0
EABT31107	1	6	11	1	1	3	2
EABT31108	457	2031.01	61	547.93	403	4	126
EABT31109	0	1	17	1	0	0	0

EABT3111	144.3	201	457.36	196.72	104	101	76
EABT31110	1	4	11	0	2	0	0
EABT31111	2	1	5	0	0	1	0
EABT31112	1	0	3	1	0	0	0
EABT31113	2	2	2	2	0	0	0
EABT31114	0	0	2	0	0	0	0
EABT31115	2579.85	2780.43	2126.3	2446.44	2261.23	2063.17	2677.45
EABT31116	0	4	6	1	1	0	2
EABT31117	0	0	2	4	0	0	0
EABT31118	4	12	27	34	4	4	4
EABT31119	48	91	76	513	78	23	38
EABT3112	2390.77	3271.75	3008.49	4181.89	1327.68	1429.11	1177.63
EABT31120	1	1	10	1	6	0	5
EABT31121	65380.8	44605.4	32333.8	19535	77913.2	28308	19827.4
EABT31122	153	77	22	9	7	106	33.04
EABT31123	443.18	634.54	772.98	1068.82	371.02	370.97	301
EABT31124	3	1	12	2	0	4	5
EABT31125	3	8	27	5	0	6	4
EABT31126	0	0	0	0	0	0	0
EABT31127	6	24	49.57	262.08	97.34	25	76
EABT31128	0	3	3	5	0	0	1
EABT31129	0	2	8	0	1	0	1
EABT3113	2	6	10	20	2	7	4
EABT31130	2	1	4	6	4	2	1
EABT31131	21	236.02	3	2	7	28	21.91
EABT31132	1388.3	2453.19	910.73	2345.89	1360.01	892.05	896.88
EABT31133	1164.47	1695.57	1875.11	1331.4	1275.31	1081.32	1122.07
EABT31134	9	19	36	17	1	2	0
EABT31135	0	1	9	0	0	0	0
EABT31136	0	8	1	3	2	0	0
EABT31137	1	0	6	0	0	0	0
EABT31138	0	4	2	9	1	1	0
EABT31139	0	1	6	0	0	0	0
EABT3114	0	16	12	5	1	0	0
EABT31140	1	0	6	0	0	0	0
EABT31141	787.99	1620.09	4550.05	1764.84	1228.18	675.29	744.28
EABT31142	0	1	10	10	0	0	0
EABT31143	0	0	3	6	4	0	0
EABT31144	0	1	0	18	0	0	0
EABT31145	1	4	1	3	2	0	0
EABT31146	3399.91	2430.27	1536.01	2551.85	2286.53	4169.53	2148.95
EABT31147	2	1	43	5	0	0	1
EABT31148	3	4	44.2	4	0	5	2
EABT31149	9	14	55	31	5	9	3
EABT3115	1	2	4	2	1	0	1
EABT31150	38	58	40	228	46	7	36
EABT31151	2	2	29	1	0	0	3
EABT31152	20	12	33.94	4	6	1	0
EABT31153	2	5	3	13.99	0	6	2
EABT31154	721.38	1461.99	900.16	1338.06	778.02	433	770.81
EABT31155	0	0	21	1	0	0	0

EABT31156	1587.99	1844.66	966	1278	4002.89	1354.43	1038.54
EABT31157	3	3	3	1	0	0	0
EABT31158	3	8	1	2	1	0	2
EABT31159	65266.4	155145	275420	5909.47	18658.7	3077.13	2123.85
EABT3116	4	14	44	94	31	56	144
EABT31160	0	0	0	3	0	0	0
EABT31161	3	5	18	2	3	0	0
EABT31162	5	4	0	2	0	6	0
EABT31163	5	12	17	59	3	4	26
EABT31164	4	4	48	5	0	1	0
EABT31165	192	266	214	461.97	349	132.14	150
EABT31166	0	8	1	0	14	0	48
EABT31167	2207.48	3304.96	6750.53	4250.2	1370.31	2062.56	1911.33
EABT31168	1	1	22	0	0	3	0
EABT31169	0	0	2	0	1	0	0
EABT3117	281722	374659	17010.4	22	18	122966	42
EABT31170	0	2	13	1	0	0	0
EABT31171	0	0	3	2	0	0	0
EABT31172	1	1	7	4	0	2	0
EABT31173	1	2	1	1	3	3	3
EABT31174	26712	105767	153486	147980	22234.5	1813.11	7699.54
EABT31175	0	1	16	0	0	0	2
EABT31176	3467.62	6458.21	6044.15	5434.8	3159.29	2212.55	3276.4
EABT31177	430.9	1016.02	1332.1	3622.33	950.01	266.74	395
EABT31178	123	390	3581.86	72	1364.85	72	574
EABT31179	4	4	6	1	0	0	0
EABT3118	0	0	1	5	2	0	1
EABT31180	1	0	14	1	0	0	0
EABT31181	0	2	1	4	0	2	0
EABT31182	0	0	5	0	0	0	0
EABT31183	272	292.96	121	394	294.79	179	186
EABT31184	0	0	1	1	2	0	3
EABT31185	783.98	837.85	431	1070.83	723.14	524.99	895.71
EABT31186	1208.87	1872.57	1528.15	3605.71	1049.95	1249.66	1271.44
EABT31187	0	1	14	1	0	0	2
EABT31188	19	6	1	0	0	14	1
EABT31189	0	0	10	5	0	4	1
EABT3119	0	0	16	3	0	0	0
EABT31190	0	0	7	0	0	0	0
EABT31191	11	35	57	77	12	0	0
EABT31192	2741.53	6580.19	3366.71	1920.05	1873.34	2241.23	2039.24
EABT31193	1494.01	3080.33	4067.2	5424.17	2920.04	1965.02	1387.1
EABT31194	0	0	3	2	0	0	0
EABT31195	2	5	2	11	0	2	3
EABT31196	2206.35	3023.63	3457.71	2352.61	3705.84	2245.74	2323.55
EABT31197	1538.1	3253.96	1538.58	3632.38	1708.72	911	1985.65
EABT31198	0	0	27	16	1	1	6
EABT31199	166	293	344	634	428	109	194
EABT312	2	3	1	0	0	1	0
EABT3120	0	1	9	0	1	0	0
EABT31200	4	14	36	49	12	3	2

EABT31201	0	0	3	2	0	0	0
EABT31202	5220.98	6314.78	455.13	2472.72	800.83	5435.5	1245.99
EABT31203	105	99	23	72	166.98	122	460.15
EABT31204	1889.81	2845.3	2296.77	2917.59	1296.52	1294.61	1796.93
EABT31205	25	86.96	44	60.02	10	8	14
EABT31206	289.97	505.02	642.97	1120.67	313.96	275.07	286.57
EABT31207	5	30	59.87	24	7	2	2
EABT31208	2	0	16	3	0	1	0
EABT31209	0	0	1	0	0	3	0
EABT3121	33	71	322.77	44	312.99	6	110
EABT31210	1054.43	1402.26	1215.25	3238.58	1273.84	747.9	846.72
EABT31211	1	0	0	0	0	15	1
EABT31212	1	3	10	4	1	2	4
EABT31213	45	134.26	394	52	14	23	55
EABT31214	961.01	1138.02	1366.58	1569.12	911.41	665	716.83
EABT31215	3	8	13	0	7	0	1
EABT31216	13	15	12	7	5	4	4
EABT31217	0	0	1	0	1	0	1
EABT31218	2707.02	2673.46	1407	1569	3475.59	1550	1242.74
EABT31219	5	26	25	28	1	2	1.46
EABT3122	703	1215	2727.14	2977.82	1809.65	857	1041
EABT31220	0	3	4	2	0	0	0
EABT31221	0	0	12	0	0	0	1
EABT31222	2	3	1	2	0	0	0
EABT31223	2	3	13	5	2	1	4
EABT31224	0	5	2	1	0	0	2
EABT31225	31	38	49	62.1	35	12	32
EABT31226	1	2.01	8	0	1	0	4
EABT31227	4834.73	8426.55	8281.48	21824.1	5538.18	5791.19	5512.64
EABT31228	0	3	3	1	0	0	0
EABT31229	0	0	3	5	0	1	0
EABT3123	2	1	12	0	0	0	0
EABT31230	1	17	87.95	8	2	4	12
EABT31231	8	9	0	0	3	5	57
EABT31232	0	2	16	1	2	2	0
EABT31233	0	3	6	2	2241.17	1	13
EABT31234	0	1	0	0	1	0	0
EABT31235	16	7	13	9	4	0	0
EABT31236	2	3	8	16	1	0	2
EABT31237	50159.1	62984	3445.93	180	5804.45	19499.5	19977.7
EABT31238	0	1	4	1	0	0	0
EABT31239	9	10	28	4	1	5	3
EABT3124	481	607	436	686	331.99	250	208
EABT31240	0	20	11	1	0	7	1
EABT31241	1	7	18	2	0	0	1
EABT31242	0	3	24	4	2	1	0
EABT31243	59	186	183	382	157	8	126
EABT31244	0	0	0	0	0	12	0
EABT31245	1100	2145.77	2070	4358.25	1103.01	1384.16	949.01
EABT31246	1	0	6	0	1	0	0
EABT31247	1	9	6	63	345	2	68.94

EABT31248	33	44	41	42	18	33	26
EABT31249	0	1	0	3	1	0	0
EABT3125	0	2	0	0	0	6	1
EABT31250	1210.65	1372.92	419.68	54	440.03	1550.78	1257.55
EABT31251	2	1	3	0	0	1	0
EABT31252	2	0	0	0	0	0	1
EABT31253	1740.69	2359.88	1259	1457	822.99	1639.52	2250.77
EABT31254	667	1042.84	1287	945	530	408	403
EABT31255	2	1	0	1	0	1	4
EABT31256	0	0	2	0	0	2	0
EABT31257	2232.94	5634.82	5167.8	7706.32	2017.58	1974.86	2636.42
EABT31258	0	2	8	1	0	1	2
EABT31259	0	0	22	11	3	1	2
EABT3126	0	0	3	2	1	0	0
EABT31260	10	33	35	15	17	0	5
EABT31261	0	3	15	2	0	0	0
EABT31262	2	11	15	8	3	0	2
EABT31263	933.47	1381.47	1326.18	1336.45	902.34	849.73	789.49
EABT31264	5	9	97	4	2	2	2
EABT31265	10	16	27	29.16	4	10	14
EABT31266	0	0	1	20	0	0	0
EABT31267	2	4	108	5	2	1	0
EABT31268	71	147	177	174	154	31	87
EABT31269	1	4	1	1	1	1	0
EABT3127	658	1048.08	1021.75	2128.7	856	506.01	458
EABT31270	1	1	16	3	0	0	0
EABT31271	5	7	16	11	2	3	1
EABT31272	353	1023	1030	265	1042.85	66	451
EABT31273	0	1	7	4	0	0	0
EABT31274	2	1	6	0	0	5	2
EABT31275	4	4	3	0	1	0	0
EABT31276	1	3	8.02	0	0	0	1
EABT31277	1	4	0	0	3	0	13
EABT31278	3	5	4	0	1	3	0
EABT31279	3	5	5	7	0	1	1
EABT3128	0	1	4	3	0	0	0
EABT31280	0	1	8	3	2	0	1
EABT31281	0	2	5	0	1	0	0
EABT31282	0	0	13	0	1	0	0
EABT31283	0	2	1	2	0	0	0
EABT31284	0	6	0	0	0	0	0
EABT31285	1	2	0	1	0	0	0
EABT31286	0	0	5	3	2	1	2
EABT31287	2	17	19	12	16	1	2
EABT31288	17	0	9	4	152.52	0	1
EABT31289	1108.76	2031	883	3780.59	416	209.99	599
EABT3129	131	185	174.01	285.01	248	112	165
EABT31290	0	0	7	0	0	0	0
EABT31291	23	38	6	123.71	15	7	36
EABT31292	66542.1	18606.9	9334.61	35211.3	13147	63743	41588.2
EABT31293	7	18	66	683.17	43	10	23

EABT31294	10376.1	10245.4	4430.01	8086.33	4548.11	8051.02	6375
EABT31295	0	0	11	0	0	1	1
EABT31296	120.99	156	252.15	319.96	67	71	69.01
EABT31297	1	0	11	0	0	1	0
EABT31298	5649.46	6685.37	13107.5	7985.68	4726.44	3657	4032.09
EABT31299	2	5	5	1	0	0	0
EABT313	0	2	13	0	0	2	0
EABT3130	2	5	2	39	5	7	13.68
EABT31300	0	0	12	1	0	0	0
EABT31301	2	4	1	1	1	1	4
EABT31302	0	0	10	1	0	0	1
EABT31303	1	2	1	5	11	5	3
EABT31304	0	4	20	2	1	0	1
EABT31305	0	0	4	3	0	0	0
EABT31306	18	48	144	378	1	0	1
EABT31307	1384.99	1833.95	783.21	2077.02	1106.9	17	129
EABT31308	1	0	3	2	0	0	0
EABT31309	0	3	11	5	0	0	5
EABT3131	3	11	45	11	2	2	3
EABT31310	0	1	1	6	3	0	0
EABT31311	1	0	0	0	1	0	0
EABT31312	684.92	381.94	249.24	54	156.53	627.82	227.59
EABT31313	8	7	42	6	2	6	10
EABT31314	0	5	28	0	0	0	1
EABT31315	4	3	0	0	0	0	0
EABT31316	3	22	7	28	4	2	1
EABT31317	1504	2083.05	2414	2906.97	1171	1276	1261.61
EABT31318	1	6	10	2	2	1	8
EABT31319	0	0	8	0	0	0	0
EABT3132	0	2	2	0	0	2	1
EABT31320	19	44	2	6	4	132	17
EABT31321	0	0	0	0	3	0	0
EABT31322	4	22	25	9	18	1	2
EABT31323	0	6	0	0	0	0	0
EABT31324	0	4	18	0	0	0	0
EABT31325	0	0	9	0	1	0	1
EABT31326	998.05	1442.64	877	1917.6	651	1027.27	746
EABT31327	9	25.72	2.01	0	2.28	42.88	23
EABT31328	4	7	8	1	1	1	0
EABT31329	3	2	2	5	3	0	0
EABT3133	3	2	19	1	0	0	0
EABT31330	1	0	1	0	1	4	2
EABT31331	8	37	265.45	43	7	11	21
EABT31332	2760.06	4752.53	6981.74	8052.72	4037.25	2030.31	3600.12
EABT31333	0	1	11	21	0	0	5
EABT31334	2330.1	3164.92	2942	3614.16	966.71	2144.69	2198.07
EABT31335	294207	76486.6	1807281	3835323	23	39	0
EABT31336	5267.7	5477.24	6280.62	8377.66	3763.61	5291.21	4676.15
EABT31337	1	2	21	1	1	0	0
EABT31338	963.03	1337	2462.99	2498.64	1322.05	831.01	1027.13
EABT31339	0	1	7	9	0	0	0

EABT3134	2	2	2	2	0	0	0
EABT31340	733.98	972	1813.21	1743.85	734	401	758
EABT31341	36	307	332.01	44	19	21	8
EABT31342	135	294	788.18	1051.4	339.99	87	87.03
EABT31343	4	10	12	20	3	5	2
EABT31344	0	4	8	5	1	1	1
EABT31345	0	0	10	0	0	0	0
EABT31346	0	1	9	1	0	0	0
EABT31347	8	1	0	0	0	3	2
EABT31348	54	123	59	212	134	20	98
EABT31349	846.77	2072.93	614.88	125	1868.99	364.28	2781.69
EABT3135	2	0	1	0	1	0	0
EABT31350	2	0	8	0	0	1	0
EABT31351	3	8	15	58	30	12	21
EABT31352	2	0	6	1	0	0	0
EABT31353	0	1	23	0	1	0	0
EABT31354	2977.13	3827.04	2940.09	7127.03	2683	2526.77	2060.01
EABT31355	830.46	1980.47	3675	3689.55	258.03	80	50
EABT31356	2	0	3	2	0	0	0
EABT31357	4	14.06	15.91	23	5	3.23	3
EABT31358	0	1	8	1	1	0	0
EABT31359	392	1176.97	1235.11	763.23	992.33	209	1481.53
EABT3136	0	1	3	0	1	0	0
EABT31360	2	2	5	2	0	0	0
EABT31361	0	0	11	0	0	0	0
EABT31362	17	23	2	3	3	25	39
EABT31363	1	1	69	2	2	11	2
EABT31364	762.49	1561.15	5933.61	3080.32	1031.87	1202.03	969.13
EABT31365	6	9	1	8	2	3	0
EABT31366	7	10	44.48	10	5	1	11
EABT31367	0	2	1	8	0	0	2
EABT31368	2	5	18	0	0	1	8
EABT31369	2	4	10	3	0	0	0
EABT3137	6	11	29	16	13	6	5.98
EABT31370	0	1	16	0	1	0	1
EABT31371	0	0	0	0	0	0	0
EABT31372	1	10	28	11	37	0	135
EABT31373	3	1	3	0	0	12	0
EABT31374	10	20	14	24	21	3	13
EABT31375	1	5	45	5	1	2	1
EABT31376	0	1	6	4	0	2	0
EABT31377	2	1	11	1	4	0	2
EABT31378	2548.16	3130.32	2443.84	4139.98	1435	2373.05	2001.99
EABT31379	9	17	61	75	6	0	0
EABT3138	3	23	41	2	2	13	7
EABT31380	0	1	2	710.75	9.02	0	0
EABT31381	1	0	0	0	2	6	0
EABT31382	3	5	15	6	1	3	5
EABT31383	2	4	8	4	1	0	1
EABT31384	0	2	5	20	4	0	0
EABT31385	4	2	3	1	0	2	0

EABT31386	1	4	8	1	0	2	0
EABT31387	0	0	4	0	0	0	0
EABT31388	0	0	2	4	0	0	2
EABT31389	0	1	3	2	0	0	0
EABT3139	33	29	61	214	63.82	6	0
EABT31390	0	2	13	0	0	0	0
EABT31391	0	0	0	0	0	1	79
EABT31392	1	2	2	1	0	0	2
EABT31393	0	1	2	4	0	0	2
EABT31394	1	3	20	0	0	0	0
EABT31395	4	0	1	0	0	11	0
EABT31396	6411.99	20160.9	604.3	979	3278.97	14010.1	14123.5
EABT31397	8	6	39	8	2	10	6
EABT31398	1	1	26	0	0	0	1
EABT31399	1176.52	1305.19	766.28	1333.46	1346.93	912	792.76
EABT314	0	6	33	1	0	1	0
EABT3140	0	2	3	0	0	0	0
EABT31400	3	12	18	16	2	0	1
EABT31401	5949.64	2580.83	133	169	1918.07	16388.9	8002.64
EABT31402	0	1	9	1	0	0	0
EABT31403	2	2	8	4	6	1	2
EABT31404	0	0	8	0	0	0	0
EABT31405	0	2	1	1	9	1	3
EABT31406	3	4	0	0	2	19	8
EABT31407	455	599	732	1008.03	824.03	319.67	345.01
EABT31408	1	6	18	5	3	2	2
EABT31409	0	5	2	0	0	7	10
EABT3141	0	1	0	0	0	8	0
EABT31410	0	0	0	2	4	0	0
EABT31411	25	7	4	6	6	7	17
EABT31412	0	1	1	1	0	0	0
EABT31413	4641.6	7476.7	4180.66	8023.55	1940.75	4347.03	5341.11
EABT31414	0	2	2	1	3	0	0
EABT31415	0	1	14	0	4	2	0
EABT31416	0	0	3	0	0	0	0
EABT31417	4	17	174.51	43	18	2	2
EABT31418	3	11	5	12	4	9	7
EABT31419	1	0	5	0	1	0	0
EABT3142	12449.1	9074.51	4579.42	8470.45	9198.67	13278.1	9433.26
EABT31420	0	0	15	5	1	0	0
EABT31421	1	3	4	0	0	7	0
EABT31422	0	8	6	5	0	1	3
EABT31423	2	4	0	5	4	0	3
EABT31424	8	11	17	0	15	0	2
EABT31425	5	9	29	5	2	2	5
EABT31426	496.83	700	282	921.34	561	429.89	472.05
EABT31427	1	3	4	1	1	2	1
EABT31428	0	0	5	0	0	0	0
EABT31429	13	53	44	4	2	12	7
EABT3143	6	8	0	2	4	9	0
EABT31430	0	0	2	2	0	0	0

EABT31431	0	0	7	4	1	0	0
EABT31432	77	114	52	94	78	28	240
EABT31433	2	2	4	2	1	0	0
EABT31434	0	4	0	1	1	0	0
EABT31435	1	2	4	17	0	0	0
EABT31436	0	0	8	0	0	0	0
EABT31437	0	0	8	0	0	0	0
EABT31438	1	1	99	2	1	0	0
EABT31439	2	11	21	21	9	7	4
EABT3144	3	0	60	4	1	2	1
EABT31440	1813	3578.68	113	19	1947.26	3654	97
EABT31441	32	196	1164	170	1084	16	230
EABT31442	7	12	0	0	1	2	6
EABT31443	0	0	10	0	0	1	0
EABT31444	6809.32	1726.51	606.41	1138	1037.01	1841.99	2075.86
EABT31445	4	4	1	4	3	2	1
EABT31446	2	3	23	1	0	0	0
EABT31447	2	2	36	15	0	0	0
EABT31448	0	2	14	5	1	2	0
EABT31449	1	0	4	6	1	0	2
EABT3145	0	1	12	1	0	0	0
EABT31450	2	9	9	3	1	3	5
EABT31451	9	13	62	27	5	6	3
EABT31452	4	2	1	0	1	0	0
EABT31453	1	2	7	4	0	0	0
EABT31454	18	49	34	52	19	35	23
EABT31455	0	0	11	0	0	0	1
EABT31456	0	0	5	5	0	0	0
EABT31457	0	0	0	0	0	0	0
EABT31458	1	0	4	0	0	0	0
EABT31459	107.93	275	380.76	1270.71	478.98	64	69
EABT3146	0	0	16	15	0	0	0
EABT31460	49	237.01	187.43	592.3	82	60.01	34
EABT31461	1	1	23	1	1	0	0
EABT31462	0	0	9	0	1	1	0
EABT31463	9	6	13	0	1	8	2
EABT31464	0	9	2	0	0	10	11
EABT31465	6	7	5	10	5	1	0
EABT31466	536.98	644.66	579	1186	530.01	353	363
EABT31467	2195.11	2574.94	1482.99	3492.29	1763.33	2092.98	1695.21
EABT31468	6	8	10	10	8	40	2
EABT31469	0	0	25	0	3	0	0
EABT3147	11553.8	33669.4	36879.9	15851	15447.6	7232.99	9349.5
EABT31470	1	2	1	2	3	0	15
EABT31471	2	1	8	2	2	1	2
EABT31472	5620.93	6816.92	5115.37	10423.9	3945.1	5636.84	4852.91
EABT31473	1538.97	1256.99	361	445.13	582	7	88
EABT31474	865.07	920.04	475	1032.98	628.4	570	581
EABT31475	46.05	12	4	7	1	5	0
EABT31476	2	0	2	1	0	0	0
EABT31477	1	3	52	5	0	2	0

EABT31478	509	936	147	21	83	352	97
EABT31479	0	1	0	1	57.38	0	0
EABT3148	5	7	17	26	5	1	2
EABT31480	3	5	12	8	4	1	0
EABT31481	1	0	13	0	2	5	1
EABT31482	0	0	3	0	0	0	1
EABT31483	3	13	22	15	10	2	5
EABT31484	25	31	20	20	7	1	2
EABT31485	0	0	3	0	0	1	0
EABT31486	0	0	0	4	1	0	5
EABT31487	1049	3103.01	5367	2701.81	1991	1606	1569.91
EABT31488	2	1	5	4	0	1	2
EABT31489	2	2	11	0	0	1	0
EABT3149	4659.56	4291.5	2073.51	5172.59	3623.66	6037.43	3085.11
EABT31490	57.05	15	0	0	0	28	0
EABT31491	0	0	10	1	0	0	0
EABT31492	0	0	1	4	1	4	1
EABT31493	0	0	7	3	0	0	0
EABT31494	4	3	2	5	3	0	4
EABT31495	1	2	7	1	3	0	0
EABT31496	0	0	15	1	0	0	0
EABT31497	0	1	2	0	1	0	0
EABT31498	0	1	5	0	0	0	0
EABT31499	1	0	4	4	0	0	1
EABT315	1083.07	1617.31	1660.47	1257.13	750.12	586.71	549
EABT3150	0	2	7	2	1	0	1
EABT31500	50	0	0	0	0	28	0
EABT31501	2	1	16	0	1	1	3
EABT31502	0	1	60	1	1	0	2
EABT31503	18	12	0	0	2	33	11
EABT31504	0	2	22	1	0	0	0
EABT31505	606.22	476	1315.99	3135.23	1406	184	383
EABT31506	0	0	7	0	0	0	0
EABT31507	0	0	17	0	1	0	0
EABT31508	1	3	3	1	0	0	1
EABT31509	0	0	4	3	0	1	0
EABT3151	2323.79	11060.4	5958.23	463	5103.54	1223.02	4177.33
EABT31510	35	29	92.89	41	3	4	4
EABT31511	0	4	3	8	0	0	1
EABT31512	0	0	16	0	0	0	0
EABT31513	0	5	5	0	0	1	10
EABT31514	3	2	1	0	0	6	5
EABT31515	0	0	4	0	0	0	2
EABT31516	8	8	5	16	2	2	2
EABT31517	1	10	10	13	2	0	0
EABT31518	1101.94	2285.01	867.46	1377.71	1386.9	824	1851
EABT31519	8	16	15	30	9	2	8
EABT3152	1926.08	2721.86	1853.83	3643.44	2128.37	1367.1	3410.07
EABT31520	2	3	68	3	0	2	1
EABT31521	0	2	15.89	0	0	0	0
EABT31522	0	0	0	1	1	0	1

EABT31523	12.36	5	0	4	10.83	0	2.99
EABT31524	0	1	2	1	0	0	0
EABT31525	10	18	15	40	24	2	6
EABT31526	0	0	5	0	1	4	0
EABT31527	2	4	13	48	3	0	1
EABT31528	2	10	33	13	68	2	6
EABT31529	210.8	229.94	288.27	354.43	196	181.05	185.05
EABT3153	0	2	8	1	0	0	1
EABT31530	2	2	5	1	0	0	0
EABT31531	1	0	1	2	1	2	0
EABT31532	1	2	0	0	0	4	0
EABT31533	815	303	79.99	50	256.02	372	854
EABT31534	36	32	17	76	11	19	13
EABT31535	391	244.52	192	16	12375.1	6	66
EABT31536	53	61	0	0	0	0	1
EABT31537	0	0	0	0	1	2	1
EABT31538	2352.74	4599.46	6553.4	3836.18	2115.7	2085.12	1980.8
EABT31539	3	5	5	15	9	0	2
EABT3154	1	1	5	0	3	0	0
EABT31540	1924.99	2889.19	1296.36	2846.33	1397.48	2035.86	1459.58
EABT31541	1	1	1	0	4	0	0
EABT31542	0	4	5	1	0	0	2
EABT31543	1	0	6	1	0	0	0
EABT31544	0	0	3	0	0	0	10
EABT31545	0	0	11	0	1	0	2
EABT31546	0	4	17.99	55	13	4	13
EABT31547	2	1	12	1	0	3	1
EABT31548	0	1	3	0	0	1	0
EABT31549	0	4	3	0	3	6	1
EABT3155	0	2	2	0	3	0	0
EABT31550	2	4	0	0	69.92	0	27
EABT31551	350.22	586.99	162	322	688.83	160.65	600.93
EABT31552	1	0	2	3	1	2	0
EABT31553	0	0	9	1	0	0	0
EABT31554	5	6	11	3	13	2	6
EABT31555	100	12	0	3	8	5	9
EABT31556	1	3	12	0	1	5	1
EABT31557	4602.33	5588.78	849.18	1467.14	2855.51	6315.1	8516.58
EABT31558	0	0	1	1	2	0	0
EABT31559	1590.28	3055.14	3468.73	2425.84	1865.59	1131.37	1900.95
EABT3156	6	5	3	1	0	5	0
EABT31560	19	23	5	27	25	20	6
EABT31561	1	0	4	0	10	1	0
EABT31562	0	1	1	13	0	0	0
EABT31563	0	0	8	1	0	0	0
EABT31564	1	2	3	7	0	2	1
EABT31565	1	0	12	0	0	0	1
EABT31566	33	56	15	8	1	32	6
EABT31567	11	12	5	1	0	0	0
EABT31568	2	7	2	0	7	0	0
EABT31569	7	18	11	24	2	2	5

EABT3157	0	0	0	5	1	0	0
EABT31570	23	48	75	97	18	13	5
EABT31571	1	1	3	3	1	0	0
EABT31572	1194.76	317.01	150.83	2	276.51	2495.8	2653.05
EABT31573	3	9	60	29	2	5	2
EABT31574	0	0	9	1	0	0	0
EABT31575	2126.84	1727	254	626	733	1825.16	1481.16
EABT31576	8	11	65	37	14	11	12
EABT31577	0	0	4	3	0	0	0
EABT31578	7	7	17	21	6	2	1
EABT31579	79	146	367	165	545	3	289
EABT3158	0	0	0	7	0	0	0
EABT31580	1	6	9	10	0	2	0
EABT31581	4	2	0	0	3	0	0
EABT31582	0	2	7	4	0	0	0
EABT31583	1	2	8	1	3	0	0
EABT31584	24	17	10	15	17	1	8
EABT31585	1	3	2	4	0	2	0
EABT31586	0	1	17	6	0	0	0
EABT31587	16	130	40	45	346	8	56
EABT31588	2	6	13	0	1	1	0
EABT31589	0	0	1	1	2	0	0
EABT3159	0	0	1	3	0	1	0
EABT31590	1	0	9	0	0	2	0
EABT31591	315.99	121	101.94	854.24	38	227.92	101
EABT31592	2	0	6	1	0	0	1
EABT31593	1	0	2	3.17	0	0	1
EABT31594	1	2	0	0	0	10	2
EABT31595	0	0	15	2	0	0	1
EABT31596	7	6	5	21	32	0	0
EABT31597	0	0	0	6	0	0	2
EABT31598	0	6	7	1	3	1	2
EABT31599	0	0	6	0	1	1	0
EABT316	22.07	50.11	37.65	87.89	28.1	10	50.97
EABT3160	3	2	5	7	0	0	3
EABT31600	5	18.69	33	5	3	4	1
EABT31601	0	1	3	2	0	0	0
EABT31602	0	0	2	0	1	0	1
EABT31603	2	0	0	3	0	3	0
EABT31604	1287.21	1747.79	503	2135.02	1422	1110.58	2004.69
EABT31605	5	16	56	11	8	1	12
EABT31606	1	0	5	1	0	0	0
EABT31607	0	23	6	4	0	2	1
EABT31608	3	3	3	9	4	0	0
EABT31609	0	1	5	7	0	1	0
EABT3161	1579	3246.13	2265.81	5616.04	3046.32	894.64	1704.42
EABT31610	8	7	53	23	2	1	2
EABT31611	1	9	30	17162.3	2	4	3
EABT31612	2	4	2	0	0	1	0
EABT31613	4	3	5	13	0	0	0
EABT31614	4	7	25	13	0	0	1

EABT31615	2	0	9	6	1	0	1
EABT31616	0	2	7	6	1	0	1
EABT31617	5	2	1	0	0	4	2
EABT31618	1909.4	2284.1	1801.25	2293.7	1279	1416.03	1086.2
EABT31619	527.99	996.89	1958.39	1343.27	795.9	51	293
EABT3162	2	0	1	0	0	2	0
EABT31620	7	6	1	4	0	0	0
EABT31621	1	1	14	1	0	1	0
EABT31622	2	16	35	6	3	0	0
EABT31623	13	30	23	68	26.97	4	18
EABT31624	9	29	74	81	2	1	0
EABT31625	0	2	3	0	2	0	4
EABT31626	0	2	7	4	1	0	0
EABT31627	8	14	0	0	0	5	4
EABT31628	3	1	5	8	1	3	0
EABT31629	1	6	1	8	8	0	0
EABT3163	185	284	527	616	462	127	126
EABT31630	0	0	7	0	0	0	0
EABT31631	1	0	0	1	2	0	1
EABT31632	23	37	32	16.01	9	1	1
EABT31633	959.87	1448.99	1916.05	4091.07	766.83	933.02	695.89
EABT31634	1	2	6	0	0	0	0
EABT31635	1	2	30	6	8	1	0
EABT31636	2	2	17	0	0	0	0
EABT31637	5	3	1	0	0	0	0
EABT31638	0	2	5	5	0	1	1
EABT31639	1	3	17	1	1	0	0
EABT3164	0	6	2	0	0	0	0
EABT31640	8	44	64	170	90	0	0
EABT31641	3	5	21	0	0	2	1
EABT31642	24	88	52	30	35	35	63.01
EABT31643	4	6	10	36	72	3	32.03
EABT31644	0	2	14	1	0	0	1
EABT31645	0	0	3	2	0	5	0
EABT31646	287	551.45	1607	1173.03	344	18	404.27
EABT31647	0	1	3	1	0	1	0
EABT31648	0	1	24	4	0	1	0
EABT31649	5.93	19	29	17	3	6	5
EABT3165	1945.02	2733.93	1989.05	2576.14	3277.54	901.18	1180
EABT31650	6.99	18	28	43	8	8	12
EABT31651	1	1	18	2	0	2	1
EABT31652	0	0	12	0	0	0	0
EABT31653	1327.92	1857.99	2463.34	3116.88	1622.09	1147.98	1046.05
EABT31654	0	1	6	0	0	1	1
EABT31655	1127.99	2163.04	2062.12	4551.09	1281.09	1060.96	1393.28
EABT31656	0	0	27	4	0	0	0
EABT31657	1	5	36	0	1	1	0
EABT31658	33	29	52	20	14	28	70
EABT31659	6	18	8	31	13	2	5
EABT3166	1790	7138.24	8551.05	2019.63	22599.1	334	2081.98
EABT31660	3	0	0	9	2	0	0

EABT31661	609	848	1148.16	1629.04	598	442	523
EABT31662	1	5	0	0	0	1	2
EABT31663	3	21	69	9	4	9	1
EABT31664	1	6	4	0	7	0	1
EABT31665	0	2	5	5	1	1	0
EABT31666	4	11	4	16.93	1	4	0
EABT31667	0	2	22	15	8	0	1
EABT31668	2	3	6	8	3	2	8
EABT31669	15	9	30	26.99	2	0	0
EABT3167	3	1	2	3	2	0	0
EABT31670	0	1	18	0	1	0	1
EABT31671	15	45	66.25	98	13	14	14
EABT31672	9	31	141.99	9	6	3	1
EABT31673	2	5	20	37	7	1	2
EABT31674	253.32	293.92	467.02	716.06	334.97	176	222
EABT31675	3	5	7	14	6	2	4
EABT31676	2	4	13	3	2	2	1
EABT31677	2	2	87	0	1	0	0
EABT31678	1	0	13	1	0	0	0
EABT31679	30	30	0	0	0	1	2
EABT3168	1	1	8	0	1	0	1
EABT31680	0	0	13	2	0	0	0
EABT31681	222.32	539	916	820.02	664	49	340
EABT31682	1	2	0	0	0	0	0
EABT31683	1	1	0	0	0	3	2
EABT31684	4	7	0	0	0	8	5
EABT31685	2	2	37	2	2	1	1
EABT31686	0	1	7	1	0	0	1
EABT31687	1641.3	2888.61	4776.05	3733.31	2640.44	1875.9	2196.33
EABT31688	0	3	4	11	2	1	0
EABT31689	2	1	5	5	0	0	1
EABT3169	1616.94	2732.81	2151.05	2252.45	1442.72	909.49	1550.56
EABT31690	0	0	7	1	0	1	0
EABT31691	0	0	12	5	2	2	0
EABT31692	1	5	22	1	0	0	0
EABT31693	0	0	5	0	0	0	1
EABT31694	2	2	15	8	1	0	1
EABT31695	1	0	7	0	0	0	0
EABT31696	6	4	5	5	1	6	0
EABT31697	122	246	886.95	1526	255.08	120	197
EABT31698	9	33	75	103	54	0	120
EABT31699	1223.85	2795.92	2517.91	3988.79	4325.25	331	764.01
EABT317	2	5	29	8	0	1	1
EABT3170	45	167	1248.02	735	190	68	108
EABT31700	1	4	32	13	7	1	9
EABT31701	0	1	18	5	0	0	0
EABT31702	0	5	0	3	0	0	2
EABT31703	7683	10468.6	5758.06	3621.88	24803.4	7585.41	7622.35
EABT31704	0	2	17	6	3	0	3
EABT31705	2	2	0	2	1	1	0
EABT31706	1	3	38	7	1	1	0

EABT31707	172.99	3200.25	2523.19	2795.08	6408.22	74.96	2105
EABT31708	2	3	27	7	0	4	3
EABT31709	1	0	7	0	0	0	0
EABT3171	1	5	47.92	2	0	1	2
EABT31710	1	2	0	1	0	1	1
EABT31711	0	1	6	2	0	1	0
EABT31712	3	2	1	4	2	1	2
EABT31713	1	0	2	6	1	0	2
EABT31714	275	510.89	339	963	518.93	300	454.84
EABT31715	380.96	418.86	227	523.91	342.99	310	327.25
EABT31716	431.1	266.25	900.3	373.04	192.8	316	233.96
EABT31717	0	0	8	0	1	0	0
EABT31718	0	4	4	0	0	0	1
EABT31719	0	2	3	2	0	0	0
EABT3172	3	12	0	0	0	8	0
EABT31720	1761.15	2482.73	1862	2784.84	1336.03	1204.31	1414.99
EABT31721	23	24	15	14.02	11	0	0
EABT31722	8	6	6	62	7	5	13
EABT31723	675	277	29	14	73	643.27	703.21
EABT31724	18	5	1	0	0	11.84	0
EABT31725	4	1	5	3	0	0	0
EABT31726	0	4	43	14	0	0	1
EABT31727	2	19.99	12	57	5	4	3
EABT31728	1	6	17	8	3	2	2
EABT31729	1103.05	1993.93	1486.72	4969.27	1367.02	1122.89	1228.07
EABT3173	15	27	63	54	63	22	19
EABT31730	0	3	0	4	1	1	0
EABT31731	3	3	25	5	10	0	6
EABT31732	0	0	28	0	1	3	0
EABT31733	0	0	19	1	1	0	1
EABT31734	0	3	5	0	0	0	0
EABT31735	970	1170.74	1275.31	1310.03	1028.97	1151.2	752.18
EABT31736	0	3	4	4	0	0	0
EABT31737	0	0	8	0	0	0	0
EABT31738	0	0	3	3	0	0	0
EABT31739	2	2	0	0	0	0	0
EABT3174	3	16	3	2	23	5	137
EABT31740	7	11	33	3	1	3	2
EABT31741	1	25	229	1	7	1	1
EABT31742	1	8	9	1	2	9	0
EABT31743	2	8	6	0	0	0	0
EABT31744	0	1	1	1	0	2	1
EABT31745	0	2	3	0	1	2	0
EABT31746	773	931	855	1959.01	878.95	505	495.44
EABT31747	1	6	33	15	4	7	0
EABT31748	0	0	12	0	1	0	0
EABT31749	2	1	37	1	3	0	0
EABT3175	2	5	50	6	1	2	2
EABT31750	395	5371.08	21533.4	7196.24	9358.58	208	1040.01
EABT31751	0	0	0	4	0	1	1
EABT31752	37	47	69	59	0	0	3

EABT31753	0	2	1	3	1	1	0
EABT31754	5	21	1	0	0	0	0
EABT31755	2	1	9	0	0	0	0
EABT31756	1	2	1	4	1	0	0
EABT31757	8	16	27	17	3	0	0
EABT31758	0	2	3	1	0	0	2
EABT31759	1	0	6	0	0	0	2
EABT3176	0	4	5	1	1	0	0
EABT31760	1	1	10	0	0	0	0
EABT31761	499	619	802.96	948	402	323.03	382
EABT31762	2562.02	3366.82	981.97	4172.63	2703.42	2387.11	2543.92
EABT31763	0	4	8	0	2	0	0
EABT31764	0	3	2	2	0	0	0
EABT31765	1	3	3	19	4	1	1
EABT31766	0	0	0	3	0	0	0
EABT31767	0	4	4	1	1	0	0
EABT31768	2	1	3	15	0	0	0
EABT31769	8	46	105	293	200	4	54
EABT3177	9	14	15	5	2	3	4
EABT31770	1	3	0	1	1	0	18
EABT31771	0	2	1	0	3	0	8
EABT31772	6	0	4	30	3	1	1
EABT31773	404	773.31	824.02	1347.94	818	220	444
EABT31774	5	12	12	53	7	2	1
EABT31775	0	1	7	4	0	0	0
EABT31776	0	3	11	2	0	0	0
EABT31777	2	16	28	45	2	0	4
EABT31778	0	0	6	1	0	0	0
EABT31779	19	11	7	15	1	7	4
EABT3178	0	0	2	11	0	0	0
EABT31780	1395.96	1674.2	1302.62	1447.82	508.6	2747.83	1977.41
EABT31781	0	2	0	5	0	0	1
EABT31782	0	0	4	1	0	0	1
EABT31783	193	277	568.97	949.06	572	14	204.01
EABT31784	2	7	3	2	0	7	0
EABT31785	4	1	3	1	0	2	0
EABT31786	1	0	32	2	0	0	0
EABT31787	0	0	11	0	0	0	0
EABT31788	1	1	45	0	0	0	2
EABT31789	0	1	10	2	0	0	0
EABT3179	943.18	1618.06	158	342	795.12	1407.02	2229.33
EABT31790	0	1	5	2	0	0	0
EABT31791	0	0	4	0	1	1	1
EABT31792	59	108.14	88	165	110	20	47
EABT31793	562.94	331	193	708	1046.74	16	14
EABT31794	1	6	22	15	8	1	2
EABT31795	1	1	5	3	1	0	0
EABT31796	5	17	4	1	0	1	4
EABT31797	2	2	10	0	0	0	0
EABT31798	2359.13	1825.43	171.04	312	576	1947.36	2948.7
EABT31799	2	0	10	1	2	1	1

EABT318	6	19	51	41	99	2	38
EABT3180	0	0	3	0	0	0	2
EABT31800	0	0	0	4	0	0	1
EABT31801	2	3	5	12	1	2	1
EABT31802	8	34	47	278	87.06	8	31
EABT31803	3	13	14	10	5	10	6
EABT31804	2	1	2	0	0	0	0
EABT31805	1360.8	1740.3	2552.56	3282.51	1494.7	1071.49	1014.31
EABT31806	0	1	4	0	1	0	0
EABT31807	1	0	10	1	0	1	0
EABT31808	3	1	3	1	2	0	0
EABT31809	1	4	5	2	1	1	0
EABT3181	1255	2054.06	3917.24	420	1115.02	87	521.97
EABT31810	1	5	10	21	7	0	0
EABT31811	40.5	186	451.46	231	221	32	266.01
EABT31812	0	0	0	0	0	0	0
EABT31813	0	2	6	0	2	1	2
EABT31814	3037.87	3396.14	1175.44	2834.49	1244	1163.52	2428.61
EABT31815	1	1	10	2	0	0	0
EABT31816	0	0	6	0	0	0	0
EABT31817	2818.09	3281.14	2221.72	3885.91	7860.08	2326.4	2102.29
EABT31818	2	3	8	0	0	0	0
EABT31819	0	7	23	15	0	1	0
EABT3182	0	0	7	1	0	0	0
EABT31820	889.65	1680.73	2321.97	13710.9	1387.2	198	477.05
EABT31821	133	49	4	30	220	7	1500.99
EABT31822	1	2	5	22	7	1	0
EABT31823	0	0	29	0	0	0	0
EABT31824	8.99	4	2	0	0	16.94	8.02
EABT31825	0	2	5	8	2	3	3
EABT31826	0	0	2	7	0	0	0
EABT31827	3	10	31	16	3	0	6
EABT31828	5	10	40	42	3	1	5
EABT31829	0	1	10	0	0	0	0
EABT3183	1	1	4	0	0	0	0
EABT31830	1	2	6	3	4	0	1
EABT31831	1	0	14.08	1	0	0	0
EABT31832	2	3	10	5	3	3	2
EABT31833	7	3	0	0	0	1	12
EABT31834	3	1	7	5	0	1	0
EABT31835	0	0	1	0	3	0	0
EABT31836	5	12	5	1	0	0	0
EABT31837	4	7	21	7.02	4	1	4
EABT31838	11	26	37	26	9	6	6
EABT31839	1	5	5	0	0	0	0
EABT3184	1	1	3	5	1	1	0
EABT31840	0	1	4	1	0	0	1
EABT31841	4	5	20	3	1	0	0
EABT31842	0	2	3	3	0	1	1
EABT31843	11	22.01	11	32	99	11	11.01
EABT31844	1	0	1	0	1	0	1

EABT31845	2	2	9	1	0	1	0
EABT31846	0	0	14	4	0	0	0
EABT31847	0	0	24	0	1	0	0
EABT31848	9	39	58	101	14	17	29
EABT31849	1	1	2	1	0	0	0
EABT3185	1	2	5	0	0	2	0
EABT31850	68	110	0	0	0	4	6
EABT31851	10	23.89	29	1260.74	81	16	29
EABT31852	0	3	10	1	0	0	1
EABT31853	0	0	6	0	0	0	0
EABT31854	0	1	4	0	0	0	0
EABT31855	1	1	1	0	0	13	2
EABT31856	1	10	17	0	1	2	2
EABT31857	3	2	17	7	0	0	1
EABT31858	0	2	3	0	0	0	0
EABT31859	1384	1994.39	961	2428	650.89	706.16	647
EABT3186	11	27	37.76	63	10	14	9
EABT31860	0	2	4	1	0	0	0
EABT31861	1	5	2	1	0	2	2
EABT31862	452.09	874.59	701	1684.35	438.99	369.49	399
EABT31863	2	0	3	0	0	0	2
EABT31864	0	4	9	5	0	0	0
EABT31865	49	29	54	7	6	2	2
EABT31866	0	0	6	4	1	0	0
EABT31867	2	0	0	0	0	2	0
EABT31868	1	5	1	0	1	1	0
EABT31869	3	3	0	0	0	0	0
EABT3187	0	0	0	0	11	0	0
EABT31870	14	2	18	0	2	0	0
EABT31871	0	0	4	0	3	0	0
EABT31872	0	2	1	3	4	0	0
EABT31873	2	0	5	3	12	0	0
EABT31874	6	7	5	12	7	4	13
EABT31875	1	6	4	4	1	0	0
EABT31876	0	2	1	0	1	12	4
EABT31877	0	1	5	0	0	0	0
EABT31878	4	5	0	0	0	0	0
EABT31879	3388.95	4209.28	1380.89	2610.68	2072.2	2191.42	1959.5
EABT3188	4	4	5	0	0	8	4
EABT31880	3	1	1	5	0	5	1
EABT31881	2	0	39	5	3	0	0
EABT31882	1	8	4	8	2	2	2
EABT31883	0	1	5	0	0	0	0
EABT31884	1	1.29	10	1	0	0	0
EABT31885	0	3	52	0	0	1	1
EABT31886	0	0	13	3	1	0	0
EABT31887	103.19	87	263.98	54	56	78	75.24
EABT31888	783.27	1191.12	1055.87	2313.02	1136.02	732.81	676.55
EABT31889	518.58	985.81	1023.73	2617.01	831	533	797.4
EABT3189	980	3684.45	1527.05	4391.9	1248.53	1017.37	1463.67
EABT31890	3272.14	3362.83	529.03	536	1852	2709.54	5418.07

EABT31891	1	0	26	0	0	1	0
EABT31892	151.01	725	2652.99	1997.18	2453.7	15	346.01
EABT31893	1	3	10	0	5	0	0
EABT31894	1	3	13	1	0	0	0
EABT31895	32	50	10	3	11	17	77
EABT31896	0	0	7	0	0	0	1
EABT31897	0	8	8	5	6	0	0
EABT31898	2	9	46	7	0	1	1
EABT31899	705.72	1340.4	1548.52	1352.88	761.56	394	403.08
EABT319	0	0	1	0	4	0	0
EABT3190	2	5	4	14	9	0	0
EABT31900	1	0	2	26	0	0	0
EABT31901	1	0	2	2	1	0	0
EABT31902	0	0	2	15	0	0	0
EABT31903	0	1	6	0	0	0	0
EABT31904	0	0	13	0	0	0	0
EABT31905	185	294.06	471.98	2114.99	273	121.27	147
EABT31906	4	10	12	8	8	12	0
EABT31907	5	12	91	23	5	4	8
EABT31908	2	3	34	2	0	1	0
EABT31909	13	21	29	17.73	3	34	28
EABT3191	1	1	36	1	0	0	2
EABT31910	2	2	1	0	0	0	0
EABT31911	2	2	1	0	3	0	2
EABT31912	0	2	5	0	0	1	0
EABT31913	2	5	1	0	1	1	0
EABT31914	3	2	41	2	2	2	0
EABT31915	0	0	4	0	3	0	4
EABT31916	18	40	45	10	2	0	2
EABT31917	1	2	7	2	0	0	0
EABT31918	7	10	5	6	8	21.91	86
EABT31919	626.01	949	2718.53	9615.69	578	78	189
EABT3192	0	0	0	0	0	1	47
EABT31920	261.97	749.53	1214.12	434.9	165.99	0	14
EABT31921	1120	3543.98	8400.46	7586.57	17324.1	418	6576.44
EABT31922	0	3	8	4	1	0	3
EABT31923	21	43	22	62	41	12	37
EABT31924	0	2	5	2	0	2	0
EABT31925	1	4	17.8	5	1	2	0
EABT31926	3	2	42	11	1	6	2
EABT31927	1	4	71	0	0	0	0
EABT31928	2	4	15	11	3	0	1
EABT31929	0	1	8	0	1	0	0
EABT3193	0	2	3	0	0	0	0
EABT31930	1	9	7	22	2	1	0
EABT31931	0	0	0	0	2	1	1
EABT31932	0	0	43	1	0	0	1
EABT31933	0	2	32	0	0	0	2
EABT31934	0	3	5	1	0	0	0
EABT31935	0	2	2	2	1	0	2
EABT31936	3	2	17	1	2	0	0

EABT31937	9	14	498.43	480.01	10	0	0
EABT31938	8	21	6	16	3	0	0
EABT31939	0	0	0	6	0	0	0
EABT3194	23	34	238	17	26	2	2
EABT31940	3	0	11	2	3	0	2
EABT31941	1	2	5	3	0	0	0
EABT31942	1	0	5	9	0	0	0
EABT31943	0	0	1	2	0	2	0
EABT31944	328	424	446.38	653.9	263	445	229
EABT31945	0	4	4.69	1	0	0	0
EABT31946	0	0	2	1	2	0	0
EABT31947	2	4	7	0	0	0	1
EABT31948	0	0	5	0	0	0	0
EABT31949	0	0	0	0	0	0	0
EABT3195	9503.56	15871.3	9582.39	7645.43	6800.51	5562.65	6320.08
EABT31950	4	5	9	15	0	5	3
EABT31951	2	7	3	0	1	1	0
EABT31952	0	0	1	2	2	0	0
EABT31953	315.1	519.83	267.65	846	434.11	194	297.17
EABT31954	0	3	3	19	1	0	0
EABT31955	0	0	3	0	0	0	1
EABT31956	0	4	8	2	1	1	3
EABT31957	0	1	3	2	0	0	0
EABT31958	30	37	11	23	7	10	9
EABT31959	1	0	6	1	1	0	0
EABT3196	14892.7	5136.22	166.72	80	2677.86	6033.08	4987.7
EABT31960	3	2	5	2	1	2	5
EABT31961	2	4	29	0	2	1	1
EABT31962	0	1	12	9	1	0	1
EABT31963	11	20	1	12	4	4.88	18.99
EABT31964	2302.94	4089.04	2869.23	5888.37	2196.66	1710.39	2404.85
EABT31965	0	0	33	5	2	0	1
EABT31966	10	11	19	5	1	22	4
EABT31967	4	4	6	2	1	0	1
EABT31968	0	8	6	4	13	0	1
EABT31969	2	0	25	0	0	0	0
EABT3197	12	17	21	16	8	21	15
EABT31970	2	4	10	3	0	2	3
EABT31971	6926	6823.51	4874	6324.04	6232.22	9031.07	7518.14
EABT31972	591.25	534.55	0	26	13	170	27
EABT31973	0	4	1	3	0	0	0
EABT31974	1422.88	2242.66	1873.43	3821.54	3055.5	275.03	555.96
EABT31975	6	4	14	0	0	5	4
EABT31976	0	0	5	10	0	0	2
EABT31977	1	2	13	2	0	0	1
EABT31978	0	0	3	1	1	1	0
EABT31979	213	419.35	315.66	350.05	233	326.32	132.27
EABT3198	29	47	150.07	70	42	21	26
EABT31980	3	4	0	3	0	0	0
EABT31981	0	2	1	3	0	0	0
EABT31982	1	1	5	2	2	0	1

EABT31983	0	0	0	0	0	0	0
EABT31984	10	16	6	23	3	4	6
EABT31985	0	2	0	2	0	0	5
EABT31986	1	0	0	0	61	0	1
EABT31987	2	3	1	2	2	0	0
EABT31988	52	158	340	494	35	62	63
EABT31989	42931.2	64089.6	42452	21744.4	134323	35446.3	71940.5
EABT3199	0	0	3	0	1	4	0
EABT31990	89	51	517	181	75	3	14
EABT31991	1	5	0	2	1	3	6
EABT31992	357.1	486.01	586.14	444	311	268.98	279.11
EABT31993	0	0	46	0	4	0	0
EABT31994	0	0	7	0	0	0	0
EABT31995	7567.66	9518.23	8590.62	3833.19	8280.33	2641.13	3168.48
EABT31996	482	826	407.04	640	428	267	405
EABT31997	14	17	65	29	7	5	29
EABT31998	4	13	112	2	1	1	1
EABT31999	1	0	3	0	0	0	2
EABT32	6	5	0	0	0	0	0
EABT320	8484.49	8821.57	3746.68	6581.04	8995.82	7251.38	9245.04
EABT3200	1	3	1	2	0	0	0
EABT32000	0	0	0	0	0	0	0
EABT32001	3	6	19	24	3	6	5
EABT32002	386	513	134	864	386	404	243
EABT32003	0	2	0	5	0	0	1
EABT32004	23	66	95	74	15	3	8
EABT32005	1	1	9	3	0	0	0
EABT32006	2	0	0	6	2	0	0
EABT32007	1	0	6	3	0	0	0
EABT32008	12219.1	20005.1	20014.1	33229.3	17550.5	10834.7	14557.5
EABT32009	733.87	1033	659	1465	861	527	484.8
EABT3201	3	3	2	0	2	1	2
EABT32010	0	0	8	0	0	0	0
EABT32011	763.92	874.72	692	1066.46	737	517.87	523.95
EABT32012	0	0	59	12	0	1	3
EABT32013	0	5	0	1	0	0	0
EABT32014	0	0	1	2	0	2	1
EABT32015	2597.41	7433.16	98	96	287	5431.96	327
EABT32016	734.91	897.43	1914.81	1952.39	991.93	832.3	903.33
EABT32017	0	0	12	0	0	0	0
EABT32018	12	40	11.9	58	85.33	27	16.7
EABT32019	0	0	0	0	1	0	8
EABT3202	0	1	0	2	2	1	0
EABT32020	2	6	11	10	1	7	0
EABT32021	9	7	0	0	0	0	2
EABT32022	1	0	1	0	0	0	2
EABT32023	18	22.04	11	37	37	12	33
EABT32024	2	3	3	1	273	0	3
EABT32025	7157.55	8044.04	5334.87	10983.8	4355.31	8993.11	6823.78
EABT32026	2	7	15	7	0	0	0
EABT32027	0	2	8	0	0	0	0

EABT32028	0	1	8	0	1	0	0
EABT32029	753.01	1195.59	1433.08	4029.61	704.99	555	417.41
EABT3203	1	4	7	6	0	1	0
EABT32030	9	6	10	1	7	5	2
EABT32031	0	2	1	1	1	0	0
EABT32032	0	0	1	9	0	0	0
EABT32033	2	12	41	47	0	0	0
EABT32034	1	1	6	4	1	0	0
EABT32035	1	4	11	12	0	2	1
EABT32036	5	5	3	13	1	0	0
EABT32037	0	0	6	2	0	0	0
EABT32038	1	6	6	10	0	4	1
EABT32039	7	2	1	1	0	7	1
EABT3204	25240.4	15164.2	5582.92	22389.3	6132.61	17902.2	11681
EABT32040	1	0	29	0	0	0	0
EABT32041	0	0	3	6	0	0	0
EABT32042	0	3	7	3	3	0	0
EABT32043	0	2	5	1	0	0	0
EABT32044	7	30	8	7	2	7	5
EABT32045	3	3	5	1	1	0	0
EABT32046	0	0	4	0	1	0	1
EABT32047	63	177	899	607	624	0	32
EABT32048	2	1	3	7	2	1	0
EABT32049	1	3	28	3	2	0	4
EABT3205	2	2	0	0	0	0	2
EABT32050	0	1	8	1	2	0	0
EABT32051	4	12	15	8	2	1	0
EABT32052	0	0	0	4	0	0	0
EABT32053	0	0	5	2	0	1	2
EABT32054	0	0	6	0	0	2	0
EABT32055	0	3	17	0	0	0	0
EABT32056	0	2	7	3	1	2	2
EABT32057	303.27	386.84	361.87	460.05	211.95	380.54	335.54
EABT32058	0	0	2	7	2	0	0
EABT32059	1	1	1	10	0	2	9
EABT3206	1	0	2	0	0	0	3
EABT32060	1	2	2	2	4	1	5
EABT32061	0	1	8	0	0	0	0
EABT32062	0	1	10	1	0	0	0
EABT32063	52	92.83	431.06	74	22	56	51
EABT32064	0	0	7	0	0	1	0
EABT32065	1	2	3	11	0	2	2
EABT32066	968	2030.99	1580.85	1904	1760.64	657	987
EABT32067	634	999.59	979	1395.29	989.25	480.69	487
EABT32068	0	2	6	1	0	0	0
EABT32069	0	0	11	1	1	0	0
EABT3207	1	0	15	8	1	3	4
EABT32070	0	2	1	1	0	1	3
EABT32071	14508.6	20368.3	60038.2	23474.2	17831.2	7688.33	9013.85
EABT32072	11	8	10	1	2	1	2
EABT32073	11	19	31	14	13	0	0

EABT32074	0	0	27	1	0	0	0
EABT32075	3678.27	8876.47	5198.25	3236.72	3220.34	2501.74	3477.13
EABT32076	0	6	21	2	1	0	0
EABT32077	26728.4	35391.7	17952.2	45358.7	21646.2	21230.8	21330.1
EABT32078	0	0	4	4	1	0	0
EABT32079	0	2	1	2	0	0	1
EABT3208	1	0	0	0	2	2	0
EABT32080	2	5	24	13	5	1	1
EABT32081	1	3	7	0	1	0	0
EABT32082	0	2	2	4	2	0	1
EABT32083	19	27	26	14	16	29	17
EABT32084	10	11	54	9	4	0	0
EABT32085	1	8	27	3	2	0	1
EABT32086	0	2	10	3	1	0	0
EABT32087	0	0	8	0	0	1	13
EABT32088	664	981.06	731.04	1672.19	1772.07	433.02	558
EABT32089	0	3	54.32	1	1	0	1
EABT3209	13	55	18	17	65.97	15	84
EABT32090	4556.97	8089.54	5805.11	13749	5694.88	53	378.01
EABT32091	9	23	87	65	38	2	4
EABT32092	0	2	2	8	1	0	1
EABT32093	0	1	7	2	1	0	1
EABT32094	0	3	13	2	1	0	0
EABT32095	0	1	2	8	3	0	0
EABT32096	4	7	0	0	0	0	0
EABT32097	1	7	18	2	69	0	0
EABT32098	1	1	7	4	0	1	0
EABT32099	4	12	39	10	2	5	5
EABT321	7	4	8	0	0	0	0
EABT3210	1093.01	2126.95	3101.06	4125.2	1021.49	434.6	930.29
EABT32100	19	25	13	6	7	16	18
EABT32101	0	11	0	0	0	9	1
EABT32102	0	0	5	0	0	0	0
EABT32103	0	0	7	1	0	2	1
EABT32104	776.39	72	3.54	3	4	2516.22	0
EABT32105	4	1	4	1	0	0	1
EABT32106	0	1	5	0	1	0	0
EABT32107	19.96	29	3	0	6	27	12
EABT32108	11	10.55	2	0	1	33	24
EABT32109	1533.03	3147.03	11958.7	1360.22	2868.93	2607.3	2928.83
EABT3211	3283.48	5038.47	11537.8	1614	2954.02	4717.73	4456.72
EABT32110	0	2	7	6	2	0	0
EABT32111	0	0	1	2	2	0	1
EABT32112	1	1	0	14	1	0	0
EABT32113	2329.1	4350.71	3226.36	6196.67	3348.9	2519.09	3633.77
EABT32114	1	0	19	3	2	0	0
EABT32115	1	0	4	4	2	0	0
EABT32116	0	0	4	2	2	0	0
EABT32117	0	1	1	12	2	0	3
EABT32118	0	2	15	4	1	0	0
EABT32119	0	0	0	0	1	0	0

EABT3212	0	3	7	1	6	1	13
EABT32120	0	4	0	1	1	1	0
EABT32121	0	0	1	5	0	0	0
EABT32122	0	1	15	9	0	0	1
EABT32123	40	75	203.86	50	40	41	28
EABT32124	2	2	2	14	14	1	2
EABT32125	0	0	6	4	0	0	0
EABT32126	42514.7	25676.9	7153	5998.99	9892.26	13013.4	11261.3
EABT32127	17	96	118	484	423	11	47
EABT32128	0	0	6	9	20	0	1
EABT32129	4	18	8	0	0	1	0
EABT3213	1767.9	1932	509	1171	3856.03	948	941.87
EABT32130	0	0	10	1	0	1	0
EABT32131	0	1	1	1	3	0	1
EABT32132	0	1	88	0	1	0	0
EABT32133	0	14	2	1	11	0	1
EABT32134	0	0	1	3	0	0	0
EABT32135	3	2	4	3	1	3	0
EABT32136	2	0	20	2	0	0	1
EABT32137	2	0	6	0	0	0	3
EABT32138	1	0	1	0	0	1	0
EABT32139	0	1	23	0	0	0	0
EABT3214	16	44	154	1051.71	49	6	90
EABT32140	0	0	11	0	0	0	0
EABT32141	0	0	2	3	0	0	0
EABT32142	1	0	9	0	1	0	0
EABT32143	54	5	6	0	0	33	0
EABT32144	0	0	0	0	0	0	0
EABT32145	1	5	57	9	39	5	1
EABT32146	1	5	7	3	0	4	2
EABT32147	1	0	18	3	0	2	0
EABT32148	0	1	10	0	0	0	0
EABT32149	2	1	5	6	1	0	2
EABT3215	0	2	8	1	0	0	0
EABT32150	3	3	150	1	3	1	2
EABT32151	0	3	0	0	0	0	2
EABT32152	720	300	21	49	315	1497	1813
EABT32153	1	2	1	3	2	0	2
EABT32154	0	4	1	0	0	6	3
EABT32155	19	14	6	1	1	4	1
EABT32156	6	12	25	25	6	1	1
EABT32157	3	6	22	1	3096.34	5	456.9
EABT32158	1944.15	2450.14	1752.14	1996.06	1785.48	1316.64	1657.92
EABT32159	8	11	34	4	4	7	76
EABT3216	0	1	18	1	0	0	0
EABT32160	0	0	0	0	1	27.86	4
EABT32161	0	1	6	1	0	0	0
EABT32162	0	2	3	1	0	0	0
EABT32163	0	4	4	4	2	0	1
EABT32164	19	103	321	987	62	5	2
EABT32165	3	6	77	19	1	3	0

EABT32166	4012.27	3599.89	2905.19	1606.91	891.48	279	403.98
EABT32167	0	0	14	3	0	0	1
EABT32168	409.41	833.8	666.24	1095.11	618.22	273.13	620.19
EABT32169	0	0	11	0	3	0	0
EABT3217	2	7	17.46	36	29	6	2
EABT32170	4	13	10	7	10	4	0
EABT32171	1	7	5	5	1	13.23	0
EABT32172	0	1	20	1	1	1	1
EABT32173	0	0	13	2	0	0	1
EABT32174	0	0	0	0	0	0	1
EABT32175	5	3	11	0	0	0	2
EABT32176	0	0	13	0	0	1	1
EABT32177	3118.4	5183.04	231	98	970.97	915.4	2955.35
EABT32178	0	1	0	0	0	0	4
EABT32179	74	155	40	14	29.89	5	5
EABT3218	0	0	23	1	0	1	0
EABT32180	0	2	31	2	2	1	0
EABT32181	2	2	4	12	5	1	5
EABT32182	4	4	78	10	3	14	12
EABT32183	0	2	1	3	0	0	1
EABT32184	0	3	2	5	0	3	0
EABT32185	0	2	32	0	0	1	0
EABT32186	2	0	2	2	5	0	1
EABT32187	146	104	136	1083	15	55	0
EABT32188	0	0	6	0	0	0	0
EABT32189	4	1	5	8	18.59	0	0
EABT3219	5	5	24	5	0	0	0
EABT32190	482.68	736.08	1932.11	2146.8	2022.31	259	628.08
EABT32191	0	2	3	1	0	0	0
EABT32192	0	0	3	2	1	0	0
EABT32193	0	1	9	1	1	0	0
EABT32194	0	0	17	3	0	0	0
EABT32195	2	2	1	3	12	0	0
EABT32196	1	1	28	3	2	0	0
EABT32197	0	28	11	36	9	1	4
EABT32198	0	0	1	1	1	0	0
EABT32199	7	8	0	0	0	0	0
EABT322	0	3	4	4	0	0	0
EABT3220	0	7	6	10	1	0	1
EABT32200	3	1	2	0	1	14	2
EABT32201	0	1	88.99	0	0	0	0
EABT32202	6	3	29	2	12	0	3
EABT32203	17	42	31	28	16	6	29
EABT32204	3	5	14	4	2	0	0
EABT32205	0	0	7	10	2	0	2
EABT32206	89856.4	338074	40965	20769.2	52599.2	151	2792.25
EABT32207	2	7	18	16	5	1	10
EABT32208	3376.19	2467.1	2607.13	4615.35	2174.25	9	117
EABT32209	0	1	8	0	0	0	0
EABT3221	1	1	2	0	0	1	1
EABT32210	15	9	19	17	4	2	173

EABT32211	694	879.83	546	1719	622.12	351	394
EABT32212	2	11	77	1	853	1	4
EABT32213	0	0	1	5	0	0	1
EABT32214	0	0	3	0	1	0	0
EABT32215	0	0	5	0	0	0	0
EABT32216	2	7	39	12	18	1	2
EABT32217	0	1	0	7	0	0	0
EABT32218	1	0	4	0	0	0	0
EABT32219	1	1	7	10	1	0	0
EABT3222	1	0	0	0	0	0	1
EABT32220	4	9	7	0	1	1	3
EABT32221	0	3	9	1	3	0	1
EABT32222	118.13	246.9	364	266.88	204.62	11.86	215
EABT32223	0	0	3	0	0	0	1
EABT32224	0	1	13	0	0	0	0
EABT32225	4	6	4	1	0	4	0
EABT32226	4	1	5	0	0	0	0
EABT32227	0	0	0	0	0	0	0
EABT32228	796.02	2411.58	1683.13	4641.96	2141.11	1712.56	2338.82
EABT32229	1	0	2	4	0	4	2
EABT3223	144.05	135	58.87	221.6	70	29	74
EABT32230	1	2	9	2	1	2	0
EABT32231	1	1	4	9	5	2	0
EABT32232	853.44	1136.7	1004.35	1730.91	1023.36	577.79	681.04
EABT32233	0	0	17	0	0	0	0
EABT32234	0	0	5	0	0	0	0
EABT32235	687	885.09	997.53	3226.3	965.06	470.08	718.04
EABT32236	0	0	0	2	6	1	2
EABT32237	0	3	2	10	6	5	5
EABT32238	4	4	95	16	4	0	1
EABT32239	198	189	35	463.99	120	35	0
EABT3224	12940.3	6565.94	3978.78	6513.46	2853.46	6941	4269.4
EABT32240	3	4	16	6	4	1	1
EABT32241	0	0	22	6	1	0	1
EABT32242	64	112	147	121	79	39	47
EABT32243	1	2	5	8	2	1	0
EABT32244	0	0	2	15	0	0	1
EABT32245	2	2	5	1	0	1	0
EABT32246	2	2	16	0	0	0	0
EABT32247	2	17	20	103	22	0	0
EABT32248	0	0	4	1	0	0	0
EABT32249	1	0	4	0	0	0	0
EABT3225	0	27.97	92	0	2	10	0
EABT32250	1	7	3	0	0	1	0
EABT32251	8	41	16	15	19	11	4
EABT32252	1	1	2	6	0	1	0
EABT32253	0	1	3	2	1	0	0
EABT32254	2	3	10	1	1	3	1
EABT32255	0	6	6	7	26	0	0
EABT32256	0	0	5	0	0	0	0
EABT32257	634.92	1351.15	392.04	601.45	1012.36	514.02	591.4

EABT32258	0	1	3	5.07	2	0	0
EABT32259	22	41	125	54	17	0	5
EABT3226	9	14	5	9	2	0	2
EABT32260	0	1	11	0	0	0	0
EABT32261	9	13	44	62.68	30	2	22
EABT32262	0	2	1	0	0	0	0
EABT32263	1	8	58	6	0	7	0
EABT32264	55	5	1	0	0	36	0
EABT32265	4	10	26	2	2	0	0
EABT32266	1	24	9	18	1	10	1
EABT32267	0	0	24	0	0	0	0
EABT32268	1	2	11	0	1	13	3
EABT32269	0	3	5	2	0	0	0
EABT3227	16	40	16	21	10	0	0
EABT32270	700.87	760.69	1501.03	1545.14	673.76	444.02	484.87
EABT32271	8	17	28	8	1	1	5
EABT32272	2	7	0	0	0	7	2
EABT32273	1	15	2	1	0	14	2
EABT32274	2349.05	3814.56	3382.12	1821.87	1413.78	1140	1468
EABT32275	4	17	1	0	0	0	1
EABT32276	0	0	2	3	1	0	1
EABT32277	0	7	4	13	0	0	0
EABT32278	1964.02	2099	2478.73	4009	2132.51	40	201
EABT32279	0	5	19	3	0	0	0
EABT3228	0	0	5	10	1	2	0
EABT32280	0	0	6	0	1	0	0
EABT32281	351	561.56	548.26	1111.47	575.51	243.54	268.03
EABT32282	16	29	81	60	9	23	17
EABT32283	47	84.97	69	25	10	2	9
EABT32284	2	3	5	1	0	0	0
EABT32285	8	3	465	3	1	4	2
EABT32286	961.92	1889.27	6316.19	2541.49	1181.98	71	85.77
EABT32287	105.86	84	21	2	13	137	39
EABT32288	68	259.92	2087.86	11573.8	3354.59	10	98
EABT32289	2	3	1	0	1	0	1
EABT3229	7	9	0	0	2	5	1
EABT32290	2	1	22	7	2	1	0
EABT32291	0	2	2	5	2	0	0
EABT32292	1	4	15	1	1	0	2
EABT32293	0	0	7	0	0	0	0
EABT32294	77	98	38	26.99	11	39	130
EABT32295	2	4	13	14	1	1	1
EABT32296	61	103.08	291	199.86	49.96	17	30
EABT32297	2	6	38	1	0	0	0
EABT32298	792	1141.7	1113.08	1787.37	777.1	536.89	505
EABT32299	10	18	16	118	16	8	8
EABT323	0	1	6	0	0	0	0
EABT3230	0	1	6	0	0	0	0
EABT32300	3326.97	4094.02	5066.54	3987.96	3542.69	2636.14	2226.86
EABT32301	0	1	0	1	6	0	2
EABT32302	2	1	3	2	0	0	0

EABT32303	0	0	13	0	0	0	0
EABT32304	0	0	19	0	10	0	0
EABT32305	0	1	1	2	2	0	1
EABT32306	0	2	10	0	0	0	0
EABT32307	2	3	8	2	0	1	0
EABT32308	1	0	1	2	1	0	0
EABT32309	659.08	777	322	456.12	860.32	614.05	423
EABT3231	321.1	392.01	361	863.65	378.69	328	276
EABT32310	1	1	15	1	1	1	0
EABT32311	0	1	6	6	3	0	0
EABT32312	0	1	3	82	2	0	0
EABT32313	26	49	196.03	1776	103	44	84
EABT32314	1	5	41	2	0	0	0
EABT32315	3101.53	5158.57	1402.99	318	2417.45	5160.37	6955.29
EABT32316	3	7	11	10	3	4	1
EABT32317	1	3	2	8	0	2	0
EABT32318	1	5	3	92	1	0	0
EABT32319	10	13	24	3	19	4	34
EABT3232	5	0	0	1	0	0	0
EABT32320	4	8	3	10	2	6	10
EABT32321	2	0	2	0	1	0	0
EABT32322	3	5	4	3	2	0	6
EABT32323	118	173.02	115.25	352.58	153	85	81.88
EABT32324	1	3	15	107.05	10	3	9.47
EABT32325	1	1	28	0	1	1	0
EABT32326	0	1	5	4	0	0	0
EABT32327	907.18	986.04	787.87	1585.68	663.29	654.8	658.01
EABT32328	0	0	2	2	6	0	0
EABT32329	1	5	36	20	5	1	0
EABT3233	1	1	8	3	2	0	0
EABT32330	0	0	1	26	1	0	0
EABT32331	4	147.01	6	6	3	40	3
EABT32332	3	3	13	6	4	0	4
EABT32333	0	2	4	6.99	0	4	1
EABT32334	4	2	0	0	0	1	0
EABT32335	0	1	2	0	0	0	3
EABT32336	2	3	2	0	1	0	0
EABT32337	1	4	3	10	3	0	2
EABT32338	1	9	1	8	7	0	0
EABT32339	7	13	92	13	4	2	7
EABT3234	10	29	25	203	85	1	29
EABT32340	5	8	0	0	0	0	0
EABT32341	0	0	9	0	0	0	0
EABT32342	4	2	1	1	2	0	2
EABT32343	1	2	14	2	2	0	1
EABT32344	0	0	0	0	8229.07	1	1
EABT32345	88	151	310	17	25	6	36
EABT32346	2	9	11	5	1	4	11
EABT32347	0	0	2	3	0	1	3
EABT32348	8	6	4	13	2	0	2
EABT32349	2	7	15	15	3	1	3

EABT3235	4	2	0	0	0	1	4
EABT32350	132.88	248.8	237.87	1077	269.01	43	116
EABT32351	214.86	247.01	175	218	213.89	244.02	153.42
EABT32352	49	64	207.04	299.01	152.83	34	62
EABT32353	1	1	5	0	1	0	1
EABT32354	2	2	12	11	19	1	10
EABT32355	1	6	47	1	1	0	0
EABT32356	0	1	7	0	1	0	5
EABT32357	0	1	4	0	3	1	6
EABT32358	6	14	80	0	0	2	1
EABT32359	77	126.47	325	197.98	219.46	15	28
EABT3236	0	2	7	2	0	1	0
EABT32360	0	0	19	9	1	0	0
EABT32361	0	6	11	2	3	0	7
EABT32362	9	1	1	0	2	43	25
EABT32363	0	1	28	0	0	0	0
EABT32364	1238.55	1427.71	1068.05	3907.58	1396.01	1147	770.02
EABT32365	175	483	443.72	1183	735.11	88	333.13
EABT32366	3	8	3	5	2	2	3
EABT32367	22	0	0	1	1	24	0
EABT32368	2	6	0	12	3	10	3
EABT32369	0	4	4	0	0	0	0
EABT3237	26313.2	24537.5	8350.21	14107.3	52932.9	20357.6	20139.5
EABT32370	0	5	4	1	0	0	1
EABT32371	3247	11350.7	10573	16105.1	5968.68	291	2854.72
EABT32372	0	1	8	1	3	0	0
EABT32373	0	1	5	2	0	2	0
EABT32374	0	3	6	1	0	0	0
EABT32375	98	89.01	98.76	21	42.87	3	22.02
EABT32376	30	64	41	52	25	4	30
EABT32377	51	35	109	6	1	2	4
EABT32378	582.15	1010.32	1630.53	4983.07	1085.7	514.34	763.81
EABT32379	1	5	17	12	1	0	0
EABT3238	0	0	3	0	0	0	0
EABT32380	0	0	0	0	1	1	0
EABT32381	0	4	13	27	7	1	1
EABT32382	541.04	1018.94	1408.28	1260.31	943.02	698.05	809.41
EABT32383	1	0	0	1	2	0	2
EABT32384	0	3	4	1	0	1	1
EABT32385	2	2	1	0	0	2	1
EABT32386	679.81	1071.89	559	1169	713.85	543	508
EABT32387	3	10	5	11	1	0	0
EABT32388	1	1	10	0	0	1	1
EABT32389	0	0	1	0	1	0	1
EABT3239	0	0	14	0	0	0	0
EABT32390	0	2	8	5	1	0	1
EABT32391	0	5	13	2	0	0	0
EABT32392	2	3	3	0	2	2	1
EABT32393	19	28	75	41	28	5	26
EABT32394	0	0	2	3	0	0	0
EABT32395	0	6	13	4	3	0	4

EABT32396	2	0	5	6	6	1	27
EABT32397	1	1	2	1	2	2	2
EABT32398	2	1	0	0	0	6	0
EABT32399	1	0	2	17	1	0	0
EABT324	3	4	41	1	1	0	0
EABT3240	0	1	0	4	0	0	0
EABT32400	6	4	1	0	1	0	2
EABT32401	0	0	8	2	0	0	0
EABT32402	0	2	2	9	0	0	1
EABT32403	6	3	7	2	6	13	2
EABT32404	0	1	0	0	0	0	4
EABT32405	4	4	58.07	14	2	1	0
EABT32406	5	2	13	1	2	2	2
EABT32407	0	1	7	0	0	0	0
EABT32408	1	0	4	0	0	0	0
EABT32409	6	21	17	0	2	35	18
EABT3241	1	2	21	5	1	3	1
EABT32410	0	0	7	0	0	0	0
EABT32411	774.01	1537.48	762.96	1945.96	2907.61	942	1859.04
EABT32412	1	5	28	3	0	0	4
EABT32413	12	6	18	8	1	2	11
EABT32414	2	14	17	27	13	4	1
EABT32415	0	0	2	0	0	1	0
EABT32416	1	0	4	3	2	0	0
EABT32417	3	8	10	3	7	0	5
EABT32418	1	2	2	0	0	0	0
EABT32419	22	51.01	152	306.72	102	10	13
EABT3242	84	1550.1	815.12	825.88	386	1	37
EABT32420	0	0	10	1	1	0	0
EABT32421	2647.18	405.76	10	6	1322	134	735.41
EABT32422	0	0	9	0	0	0	0
EABT32423	16	13	5	11	0	21	0
EABT32424	0	1	17	0	0	0	0
EABT32425	0	0	4	5	0	0	0
EABT32426	1	0	3	9	9	1	1
EABT32427	2	2	4	5.98	2	1	0
EABT32428	2	15	85	19	5	1	0
EABT32429	13.11	25	22	38	9	4	8
EABT3243	0	2	11	4	2	0	5
EABT32430	0	1	25	0	1	0	1
EABT32431	1	2	0	0	1	0	2
EABT32432	33	29.92	6	45	9	11	27
EABT32433	917.05	1717.97	1158.93	7135.62	886.06	1090.76	987.98
EABT32434	1846.12	4330.67	9076.17	6376.76	3926.22	1756.17	2696.27
EABT32435	1716	2047.07	3071.51	3763.24	1221.3	1400.97	1019.57
EABT32436	290	347	130	437	274	169	205
EABT32437	3	5	4	5	9	2	1
EABT32438	1	7	2	5	9	0	3
EABT32439	2	0	4	2	0	1	2
EABT3244	1	2	1	0	0	4	0
EABT32440	3	4	39	5	2	4	2

EABT32441	302	271	103	119	349	811	2332.91
EABT32442	231.07	434.93	376.01	861.91	645.48	310.87	340.1
EABT32443	56.84	280.04	199	540.18	25.25	10	12
EABT32444	0	3	3	25	7	3	3
EABT32445	542.71	1195.38	1188.2	193.32	469.68	116.74	302.91
EABT32446	0	0	0	5	2	0	3
EABT32447	116.45	37	0	0	1	176.01	1
EABT32448	0	3	1	2	4	0	0
EABT32449	4	5	1	28	0	0	1
EABT3245	1	0	9	3	0	0	0
EABT32450	8	10.23	10	15	1	1	1
EABT32451	360.03	594	181.76	336.71	485.99	102.01	523.32
EABT32452	2	6	9	10	5	0	0
EABT32453	1	1	20.99	0	0	0	0
EABT32454	2	1	40	1	2	0	1
EABT32455	0	0	8	1	0	0	0
EABT32456	1	0	28	0	0	0	1
EABT32457	0	0	2	2	0	0	0
EABT32458	2	2	2	2	2	1	4
EABT32459	1	1	14	0	0	1	0
EABT3246	1754.92	2046.16	201	121	415	3501.89	2352.1
EABT32460	7	16	0	0	3	13	33
EABT32461	0	5	17	1	0	1	0
EABT32462	3	5	2	2	1	4	3
EABT32463	0	10	2	0	1	0	2
EABT32464	0	5	9	58	3	1	7
EABT32465	266.99	392	462.84	1682	230	21	79
EABT32466	0	0	0	0	0	0	0
EABT32467	0	0	1	1	0	1	1
EABT32468	11519.7	32382.2	27513.9	11663.1	100276	9063.43	18448.3
EABT32469	892.4	1432.32	1204.02	3402.41	1133.61	732	715.31
EABT3247	0	0	21	1	0	0	0
EABT32470	675.51	1718.44	1708.14	1030.84	1067.77	466.97	735.86
EABT32471	0	0	0	1	0	0	0
EABT32472	1	4	18	4	0	2	1
EABT32473	15	11	14.28	11	14	5	2
EABT32474	197	223.12	215	297	419.95	233.01	350
EABT32475	3	9	4	23	2	3	2
EABT32476	0	0	2	0	0	1	0
EABT32477	3	3	9	0	1	0	1
EABT32478	0	3	7	1	1	1	2
EABT32479	6259.99	7242.71	6466.65	16707.9	3858.14	4488.93	2634.48
EABT3248	30	82	30	488.06	100	34	15
EABT32480	0	4	6	66	3	1	1
EABT32481	0	5	3	1	2	0	7
EABT32482	13	11	8	15	133	6	6
EABT32483	2	7	3	10	33	0	3
EABT32484	13	17	48	5	0	9	0
EABT32485	2	5	14	0	0	15	6
EABT32486	2	4	2	9	1	2	1
EABT32487	26	61	138	4	26	4	55.98

EABT32488	0	1	1	0	1	0	1
EABT32489	0	0	4	1	0	0	0
EABT3249	1	0	2	1	0	0	0
EABT32490	3	11.89	10	2	1	2	3
EABT32491	0	1	10	3	0	0	1
EABT32492	2	3	5	2	0	0	0
EABT32493	1	1	7	2	1	1	0
EABT32494	1	0	3	0	0	2	1
EABT32495	57.16	24	4	3	7	49.42	5
EABT32496	0	1	5	4	0	0	0
EABT32497	386	1273.42	1556.97	3652.17	982.74	268.98	574
EABT32498	0	0	14	0	1	0	0
EABT32499	10137.3	8175.3	7344.39	3738.81	5902.31	7219.67	10513.8
EABT325	8	5	12	1	1	3	2
EABT3250	0	0	3	13	3	0	0
EABT32500	0	0	15	0	0	1	0
EABT32501	0	3	0	0	0	0	0
EABT32502	0	0	7	0	1	0	0
EABT32503	0	0	8	1	0	0	0
EABT32504	0	0	0	0	7	0	0
EABT32505	207.28	326.93	56	734.82	595.99	360.21	333.01
EABT32506	324	437	0	0	188	1499.38	393
EABT32507	44	164.15	136.97	153.94	117.84	50.13	129.31
EABT32508	1	1	8	0	0	0	0
EABT32509	16	23	52	10	4	5	0
EABT3251	0	0	2	1	0	0	0
EABT32510	372.72	455.26	539	859	413.8	404.95	409.02
EABT32511	2	2	0	0	0	6	4
EABT32512	21	23	19	33	13	4	10
EABT32513	10	3	16	3	2	2	2
EABT32514	764	1366.86	1510.54	3689.01	843.92	916.38	668
EABT32515	197	325	703	663.73	12552.4	209	135
EABT32516	1509.23	2258.01	2778.67	3164	1297	1690.11	1447
EABT32517	0	2	0	0	0	1	0
EABT32518	3	8	6	0	0	0	0
EABT32519	0	0	3	1	0	0	0
EABT3252	1	0	0	94.29	4	0	0
EABT32520	0	1	2	0	10	0	9
EABT32521	1	1	2	3	5	0	1
EABT32522	135	346	179.87	474	149.99	354.03	121
EABT32523	15	2	2	0	0	0	0
EABT32524	3	17.43	22	27	7	4	11
EABT32525	0	2	4	8	9	1	0
EABT32526	0	4	49	2	0	5	4
EABT32527	2	32	1	0	0	33	0
EABT32528	0	0	3	1	2	0	1
EABT32529	0	0	3	0	0	1	0
EABT3253	3	0	3	6	3	0	0
EABT32530	0	0	9	0	0	0	0
EABT32531	3	6	16	1	2	2	2
EABT32532	6	10	10	9	1	5	0

EABT32533	0	0	2	0	7	1	1313.07
EABT32534	4	9	390	4	9	3	1
EABT32535	1	0	2	2	1	1	2
EABT32536	0	1	7	0	0	0	0
EABT32537	7117.09	10484.9	5720.4	4810.73	2823.95	6188.99	10718.8
EABT32538	3602.13	3105.89	48	94	191	883	578.15
EABT32539	1	2	5	0	2	1	2
EABT3254	305.8	475.48	435.83	1041.51	441.86	275.01	240.92
EABT32540	6	10	7	13	4	2	2
EABT32541	4	7	0	0	0	2	2
EABT32542	0	2	2	2	0	0	0
EABT32543	5	3	33.02	18	4	3	8
EABT32544	131	266	481	3179.07	359	21	136
EABT32545	0	0	9	0	0	0	0
EABT32546	1	5	1	3	0	4	0
EABT32547	133	183	206	743.04	269	102.42	106
EABT32548	43.09	49	30.8	90.01	16	35	11
EABT32549	287.49	308.31	239	255.02	248.25	227	237
EABT3255	57	149.01	104	539	60	96	86.43
EABT32550	1	2	49	1	1	0	0
EABT32551	0	7	2	8	1	0	1
EABT32552	4	9	25	24	2	0	1
EABT32553	1	1	6	6	3	9	0
EABT32554	1	3	2	0	4	0	0
EABT32555	0	1	5	1	18	0	0
EABT32556	10949.1	4795.48	117	6	52	3276.11	700
EABT32557	1	0	6.81	1	0	0	0
EABT32558	1467.12	3178	6554.9	14956.3	4350.96	92	699.05
EABT32559	1	4	5	0	0	0	1
EABT3256	2	0	7	0	0	1	2
EABT32560	728.7	3727.78	21165.1	1402.18	971.84	780.67	32
EABT32561	0	1	16	1	0	0	0
EABT32562	1	2	4	4	0	0	0
EABT32563	122.21	164.48	42.92	18.56	143.21	10	47.13
EABT32564	0	1	1	18	0	0	0
EABT32565	472.28	613.33	496.91	1157.88	703.7	324.01	470.01
EABT32566	396.38	690.39	526.92	1769.68	798.86	406.52	568.78
EABT32567	2	4	23	15	2	0	1
EABT32568	1	15	5	4	1	3	0
EABT32569	3	0	3	3	1	2	1
EABT3257	1	0	9	0	0	0	0
EABT32570	5	11	3	23	8	0	1
EABT32571	2	0	25	3	0	0	0
EABT32572	7	22	22	41	74	10	7
EABT32573	2	4	0	3	0	0	0
EABT32574	25	43	31	33	26	18	23
EABT32575	1	0	4	0	0	1	1
EABT32576	2	0	4	2	3	0	0
EABT32577	5	0	35	1	0	1	0
EABT32578	0	2	10	0	0	0	0
EABT32579	3	5	9	2	8	4	46

EABT3258	0	1	8	5	0	4	0
EABT32580	4541.92	10887.6	5985.82	8353.05	3345.41	2763.03	2870.98
EABT32581	0	0	7	3	0	0	0
EABT32582	2	6	9	2	1.75	3	1
EABT32583	500.62	745.91	641.01	928	819	246	403
EABT32584	26	19	65.85	47	25	17	22
EABT32585	4	1	11	1	2	0	0
EABT32586	0	1	8	1	0	0	0
EABT32587	0	0	2	0	3	0	0
EABT32588	5	4	5	1	0	0	0
EABT32589	0	3	3	5	0	1	0
EABT3259	12	6	39	1	0	1	2
EABT32590	1	3	6	3	1	0	4
EABT32591	0	0	10	3	1	0	0
EABT32592	0	9	15	4	0	2	0
EABT32593	1	6	4	5	1	1	1
EABT32594	0	0	3	5	0	0	5
EABT32595	0	2	5	0	0	0	0
EABT32596	438	632	436	5065.3	1171.33	37	177
EABT32597	3255	3385	2685	3840.99	2277.46	4744	2629.02
EABT32598	6	6	11	1	0	1	2
EABT32599	3	5	19.74	10.01	6	3	14
EABT326	0	0	4	0	0	0	0
EABT3260	6402.77	7630.05	2100.96	2339.38	2477.22	3393.71	2624.47
EABT32600	5	0	0	1	0	33	0
EABT32601	1407.64	2147.42	1946.93	3080.32	1149.13	1185.25	1286.05
EABT32602	1	0	2	0	0	4	0
EABT32603	2	2	12	5	1	0	1
EABT32604	805.38	1214.95	985.35	1994.16	1196.93	701.91	770.19
EABT32605	802.11	2150.59	2283.98	3028.61	1849.33	395.12	623.01
EABT32606	0	0	5	2	0	0	0
EABT32607	344.32	198	163	31	14	56	11
EABT32608	1	0	2	2	0	0	0
EABT32609	1	0	15	0	0	0	0
EABT3261	0	1	10	2	1	0	0
EABT32610	5	14	16	7	2	0	2
EABT32611	0	0	5	1	0	2	0
EABT32612	5	8	20	27.84	2	0	5
EABT32613	0	0	9	0	0	0	0
EABT32614	3	2	1	2	0	4	3
EABT32615	0	2	0	3	0	0	0
EABT32616	179	455	535	304	315	12	90
EABT32617	0	1	3	1	3	0	4
EABT32618	0	1	14	2	1	0	1
EABT32619	744	878	315	670	441	194	309.04
EABT3262	6	10	15	75	40	1	2
EABT32620	381	636.99	913	1485.19	431	237	219
EABT32621	0	0	6	0	1	0	0
EABT32622	4305.48	8750.73	2913.75	6078.8	4162.11	2398.17	2343.98
EABT32623	0	0	7	1	0	0	0
EABT32624	1	80	6	10	5	0	0

EABT32625	0	0	4	0	1	1	0
EABT32626	0	3	4	2	0	0	0
EABT32627	3	5	0	0	1	1	0
EABT32628	6	1	0	0	0	65	0
EABT32629	630.91	2984.76	10764.3	247.6	1455.87	1754.96	280.74
EABT3263	0	0	4	3	1	1	1
EABT32630	0	0	2	1	0	0	0
EABT32631	0	1	6	13	0	0	0
EABT32632	0	0	1	0	4	0	0
EABT32633	4861.97	6194.27	5409.81	5562.78	2781.8	6669.85	9372.99
EABT32634	0	1	7	0	0	0	1
EABT32635	1	1	2	1	0	0	0
EABT32636	1	2	13	13	2	1	1
EABT32637	16	20	0	0	1	4	8
EABT32638	1	0	3	0	5	1	2
EABT32639	0	0	0	7	0	0	0
EABT3264	957.1	2787.43	4439.3	8287.4	4148.18	118	834.2
EABT32640	1	0	8	1	2	0	0
EABT32641	1	5	4	23	0	0	2
EABT32642	0	9	16	0	1	0	0
EABT32643	6	21	173	5	5	4	2
EABT32644	0	0	1	0	0	0	0
EABT32645	2	1	10	1	0	0	0
EABT32646	1804.23	2670.4	2797.07	7073.36	1414.2	1365.35	1132.85
EABT32647	0	1	6	0	3	0	3
EABT32648	1899.69	2776.99	2915.92	2537.96	2303.41	2482.97	3143.03
EABT32649	1	3	3	11	2	0	1
EABT3265	0	1	34	0	0	0	0
EABT32650	3	4	5	0	0	0	0
EABT32651	2	7	21	2	0	0	0
EABT32652	0	0	0	0	33	0	29
EABT32653	0	1	5	1	0	0	0
EABT32654	1057.98	937	421	1310.78	1127.95	117	385
EABT32655	1	0	11	0	0	0	0
EABT32656	937.74	1786.7	1757.47	3712.89	1367	920.16	769.02
EABT32657	1	3	3	10	0	2	0
EABT32658	0	2	2	2	0	0	1
EABT32659	3	15	21	2	1	5	16
EABT3266	1	1	0	0	0	1	3
EABT32660	1	0	2	7	0	0	0
EABT32661	0	0	8	0	4	1	0
EABT32662	4	2	4	6	2	1	2
EABT32663	0	0	9	0	1	0	0
EABT32664	1	3	13	1	0	30	8
EABT32665	0	0	8	1	1	0	0
EABT32666	0	0	2	1	3	0	0
EABT32667	1	0	3	1	0	0	0
EABT32668	0	0	25	9	1	0	0
EABT32669	74	234	720.9	390.6	154	95	157.12
EABT3267	3449.98	5924.37	3606.55	8288.67	2400.04	2472.99	3167.84
EABT32670	0	0	2	0	1	0	0

EABT32671	0	0	1	3	0	0	0
EABT32672	2	1	12	0	0	1	0
EABT32673	1	1	2	0	0	0	0
EABT32674	0	0	2	0	0	0	0
EABT32675	2	3	2	1	1	2	8
EABT32676	410.95	470.45	228	730.99	289.96	93	339
EABT32677	9	7	20	5	3	1	0
EABT32678	2146	3705.39	2301.22	6920.49	2073	2250.28	1935.97
EABT32679	7	5	2	1	0	0	0
EABT3268	1567.32	2006.16	1427.91	2586.51	1501.68	1395.14	1731.99
EABT32680	0	2	1	0	1	1	6
EABT32681	0	3	20	1	0	0	0
EABT32682	1	1	2	1	0	1	1
EABT32683	0	1	3	0	1	0	0
EABT32684	3	9	41	12	0	0	3
EABT32685	0	0	2	0	2	0	3
EABT32686	119	142.49	135	213	139	141	114
EABT32687	6138	1375	20	2	45	18573.5	312
EABT32688	5132.65	19899.2	14413.1	7579.87	4988.82	1992.03	2571.16
EABT32689	1	4	8	17	1	1	2
EABT3269	337.95	553.56	529.27	1375.17	559.7	218	306.02
EABT32690	1	2	31	0	0	0	2
EABT32691	0	2	37	0	0	0	0
EABT32692	0	2	5	14	1	0	1
EABT32693	46	144	537	2613.97	102	50	106
EABT32694	0	0	3	0	38	1	0
EABT32695	1	2	3	3	0	3	2
EABT32696	0	4	40	2	0	4	2
EABT32697	0	1	1	3	0	0	1
EABT32698	0	0	4	1	2	0	0
EABT32699	1	1	7	9	0	0	0
EABT327	1	1	0	0	0	5	2
EABT3270	0	0	5	0	1	0	0
EABT32700	0	1.99	5	1	0	0	0
EABT32701	2	3	8	0	0	1	0
EABT32702	2	1	64	15	3	1	0
EABT32703	0	1	3	0	0	0	0
EABT32704	0	0	0	2	2	0	1
EABT32705	1	7	4	1	0	0	1
EABT32706	0	1	4	0	2	0	1
EABT32707	8	14	61	20	4	7	4
EABT32708	5	1	2	0	0	0	1
EABT32709	210	406	949.85	255.25	80	419	203
EABT3271	0	1	2	1	3	3	6
EABT32710	1	11	0	0	0	0	2
EABT32711	2	2	3	0	1	0	4
EABT32712	7	11	7	0	3	0	27
EABT32713	1	0	24	0	0	0	1
EABT32714	296.07	379.98	0	0	9	16	23
EABT32715	153.69	332.47	375.93	267.29	212.64	27	48.98
EABT32716	0	1	6	7	0	0	0

EABT32717	3	4	17	4	14	2	1
EABT32718	1014.47	1822.78	523.86	237.38	397.28	25	222.22
EABT32719	3691.54	337	38	8	468	2	4
EABT3272	4	8	11	714	1	3	2
EABT32720	20	51	37	17	41	7	45
EABT32721	1	5	11	11	0	0	0
EABT32722	0	1	2	1	0	0	0
EABT32723	2	0	2	0	0	0	1
EABT32724	0	5.02	14	4	4	2	0
EABT32725	5	1	0	0	0	8	0
EABT32726	741.61	1128.93	802.82	2311.64	663.05	481.66	759.27
EABT32727	2	0	3	1	0	0	0
EABT32728	1	0	8	0	1	1	2
EABT32729	0	1	6	0	0	0	0
EABT3273	3	0	1	13	0	0	0
EABT32730	2	2	3	0	0	1	0
EABT32731	1026.01	1471.43	2559.6	2154.24	1190.16	1081.97	888.78
EABT32732	6	1	0	0	0	2	2
EABT32733	3056.96	4862.31	7321.84	8251.64	4910.22	3511.03	4767.74
EABT32734	3119.07	4264.95	3912.16	6484.27	2955.31	2599.4	2469.26
EABT32735	1	2	69.02	0	2	1	1
EABT32736	7	15	86.04	40	3	9	3
EABT32737	0	0	4	3	2	1	0
EABT32738	1	2	45	1	1	9	2
EABT32739	1	3	1	0	2	0	3
EABT3274	3	5	13.13	21	17	0	6
EABT32740	4597.95	4409.02	2592.77	4309.41	2357.88	4544.66	4985.23
EABT32741	1	1	4	0	3	0	0
EABT32742	4	2	6	49	1	0	19
EABT32743	1848.41	1969.98	1071.09	3153.94	1416.81	1538.1	1653.3
EABT32744	0	0	13	1	0	0	0
EABT32745	3	0	0	0	2	0	13
EABT32746	1	0	14	7	0	0	0
EABT32747	2	0	0	0	0	3	5
EABT32748	3	15	1	1	0	7	4
EABT32749	0	0	3	2	4	0	2
EABT3275	2520.52	3910.54	7520.21	5489.75	2367.64	2743	2343.04
EABT32750	0	1	13	0	0	0	1
EABT32751	34	50	8	2	3	12	20
EABT32752	1	0	10	1	0	0	0
EABT32753	792.98	1204.78	1480.28	2115.06	1389.1	842	758.03
EABT32754	0	1	0	1	0	0	0
EABT32755	0	1	7	2	2	0	2
EABT32756	0	0	1	0	5	0	5
EABT32757	0	3	6	0	0	0	0
EABT32758	0	3	97	1	4	3	2
EABT32759	0	4	12	2	0	5	2
EABT3276	0	0	12	1	0	0	0
EABT32760	0	0	1	0	0	0	0
EABT32761	3	5	3	3	0	0	1
EABT32762	0	4	8	1	0	0	0

EABT32763	0	1	2	1	1	0	0
EABT32764	0	0	13	3	0	0	0
EABT32765	0	7	43	89	1	1	1
EABT32766	0	0	6	16	1	1	2
EABT32767	64	186	151.92	163	940	9	170
EABT32768	0	1	0	1	0	1	3
EABT32769	0	3	174	2	0	0	1
EABT3277	4	2	11	0	0	1	2
EABT32770	822	2571	1145	731	594	690	2815.07
EABT32771	0	0	3	0	0	0	0
EABT32772	0	0	0	0	0	0	0
EABT32773	670.22	822	405	1267.89	752.04	553.99	533
EABT32774	3308.7	5085.98	5931.44	10611.4	2216.28	4825.58	4778.56
EABT32775	103	103	174	275.17	1748.08	41	1593.98
EABT32776	77	113.99	137.32	51.24	105.99	232.05	154.02
EABT32777	2	3	21	6	2	0	0
EABT32778	2160.7	2568.14	836.54	663.22	14272.1	4732.54	3304.79
EABT32779	34	30	25	16	8	2	3
EABT3278	1	0	7	1	1	1	0
EABT32780	2	1	3	1	1	1	0
EABT32781	7	24	13	31	14	0	12
EABT32782	4	10	14	0	0	0	5
EABT32783	13	5	0	0	1	79	7
EABT32784	0	6	40	0	17	0	2
EABT32785	0	3	27	4	2	0	0
EABT32786	1	2	17	2	0	1	2
EABT32787	22	7	48	0	4	40	20.7
EABT32788	1	8	2	19	18.43	1	0
EABT32789	0	0	5	0	0	1	2
EABT3279	0	1	8	1	2	0	0
EABT32790	1	0	7	0	1	0	1
EABT32791	0	2	0	4	0	0	0
EABT32792	0	1	4	1	0	1	1
EABT32793	0	2	0	2	0	2	0
EABT32794	25	187.99	0	1	4	27	15
EABT32795	3	1	14	1	0	2	0
EABT32796	4674.05	4808	3335.28	2254.99	815.71	4101.26	2102.79
EABT32797	0	1	7	1	0	0	0
EABT32798	14	31	9	2	3	22	4
EABT32799	0	0	5	2	0	0	0
EABT328	972.23	2052.05	2743.57	4107.9	1140.45	697.46	670.2
EABT3280	0	2	11	0	1	0	0
EABT32800	0	0	0	0	0	0	0
EABT32801	0	0	0	0	1	0	1
EABT32802	0	6	8	1	0	0	1
EABT32803	0	1	17	4	16	1	0
EABT32804	2	4	18.99	21	3	1	1
EABT32805	37	47	286.94	35	13	16	43
EABT32806	47771.5	12567.3	3309.06	9736.19	4705.34	16146	3009.42
EABT32807	2	1	7	0	0	0	1
EABT32808	0	3	6	1	0	0	0

EABT32809	1	3	2	17	4	1	3
EABT3281	1498.49	2811.42	3504.76	5759.11	1446.51	1442.52	1519.36
EABT32810	412.99	846.53	192	1263	739.37	334.99	492
EABT32811	0	0	3	3	0	0	1
EABT32812	0	3	2	0	0	0	0
EABT32813	1	3	5	20	0	0	0
EABT32814	0	2	3	15	0	0	0
EABT32815	0	3	40	7	3	0	0
EABT32816	0	4	24	13	3	2	13
EABT32817	0	3	3	5.98	0	0	1
EABT32818	0	2	3	0	1	0	0
EABT32819	3879.91	8307.99	10906.6	3482.66	3993.84	3209	3281.04
EABT3282	1680.99	2193.78	548	2292.03	756	1201.02	2479.19
EABT32820	3	8	12	5	0	1	0
EABT32821	627.83	1085.94	2676.25	2392.97	903.97	562	443
EABT32822	291.62	541.58	304.11	1142.28	477.68	229.94	209.02
EABT32823	1	1	14	2	1	0	1
EABT32824	6	5	41	1	0	1	2
EABT32825	0	2	8	0	1	1	0
EABT32826	6	7	19	7	11	13	2
EABT32827	0	0	5	1	0	0	0
EABT32828	259	440.24	466.01	651.22	478	195	271.95
EABT32829	6	24	12	2	2	0	2
EABT3283	2	2	11	1	0	0	0
EABT32830	1	6	9	2	1	0	0
EABT32831	10	3	14	4	1	1	0
EABT32832	0	1	1	2	1	4	1
EABT32833	0	3	8	3	4	0	1
EABT32834	1	1	8	5	1	3	1
EABT32835	12	32	18	37	21	7	15
EABT32836	8	3	1	0	12	27	86
EABT32837	0	2	3	4	0	0	0
EABT32838	729.08	1954.24	981.94	232	809	145	1064.56
EABT32839	6.93	5	3	1	1	3	2
EABT3284	0	0	4.69	1	1	0	0
EABT32840	0	0	0	0	114.61	0	0
EABT32841	0	0	4	0	0	0	1
EABT32842	0	4	10	0	0	0	0
EABT32843	0	0	0	0	0	0	0
EABT32844	0	2	43	0	0	1	0
EABT32845	5	13	26	21	7.01	5	13
EABT32846	0	1	1	1	1	0	0
EABT32847	0	1	88	32	31	0	1
EABT32848	395	582.03	368	1191	576	166	210.53
EABT32849	2	2	19	3	1	0	3
EABT3285	2	3	17	2	1	0	2
EABT32850	2	2	1	5	10	0	2
EABT32851	0	1	6	3	0	0	0
EABT32852	3	1	6	0	1	2	0
EABT32853	1	4	2	5	1	0	0
EABT32854	134	79.02	16	33.22	175.13	147.7	161.7

EABT32855	1	1	3	5	1	0	0
EABT32856	92	7	0	0	0	274.4	0
EABT32857	1	0	3	0	0	1	3
EABT32858	1	1	28	0	0	0	0
EABT32859	2650.02	4322.2	6762.07	7348.26	2390.3	2616.6	3111.84
EABT3286	1	2	16	9	3	2	8
EABT32860	2404.53	1855.65	832	1365.07	1302.52	1858.93	1908
EABT32861	0	0	0	0	0	0	0
EABT32862	622	1016	1247.93	744	682	826.95	839
EABT32863	1	0	5	0	0	0	0
EABT32864	0	1	23	0	0	0	0
EABT32865	0	0	15	2	0	0	0
EABT32866	7	5	48	4	0	3	1
EABT32867	1	0	6	0	0	0	0
EABT32868	1040.28	1326.83	1225.79	1509.37	873.98	1002.17	905.67
EABT32869	5	10	36	9	4	4	3
EABT3287	0	1	10	3	0	0	0
EABT32870	897.56	4554	4122	3324	6749.11	61	1923.83
EABT32871	1	2	4	1	0	1	0
EABT32872	15	7	4	3	10	17	34
EABT32873	1	7	0	3	0	0	0
EABT32874	1	10	29	5	2	1	1
EABT32875	4	17	120.27	83	5	12	10
EABT32876	0	0	7	3	0	0	2
EABT32877	54	88	0	0	8	9	72
EABT32878	4	4	14	9	1	1	2
EABT32879	0	1	2	1	2	1	1
EABT3288	1	1	8	4	0	0	0
EABT32880	0	1	12	0	0	0	0
EABT32881	0	0	8	0	0	0	1
EABT32882	0	0	9	0	0	0	0
EABT32883	427	582	721.28	1316	271	469	224.1
EABT32884	2	5	7	19	3	1	1
EABT32885	0	1	12	2	1	0	0
EABT32886	0	0	6	1	1	0	1
EABT32887	6	8	5	2	12	4	17
EABT32888	0	1	10	0	0	0	0
EABT32889	717	950	838	1527.46	820.77	760	724
EABT3289	0	1	0	6	8	16	12
EABT32890	3	3	1	1	3	0	0
EABT32891	2	5	0	0	0	5	2
EABT32892	0	2	11	0	0	0	0
EABT32893	145	15.02	0	0	0	54	13
EABT32894	4758.65	3645.82	3188.08	3104.66	3394.96	2654	1279.47
EABT32895	5	7	2	2	2	0	2
EABT32896	51.99	158	126.22	145	57.72	113	242
EABT32897	2	4	0	0	1	0	1
EABT32898	3	5	7	4	1	0	2
EABT32899	0	0	7	1	0	0	2
EABT329	6	18.04	15	6	8	2	2
EABT3290	2	8	12	24	1	1	0

EABT32900	1	5	16	4	1	1	3
EABT32901	4	5	6	2	5	2	27
EABT32902	0	1	5	1	0	0	0
EABT32903	7	9	30	12	1	5	2
EABT32904	1	3	12	2	0	0	0
EABT32905	0	1	2	0	0	1	1
EABT32906	0	0	5	0	0	0	0
EABT32907	1499.06	2152.89	8360.58	1098.93	1255.95	1089.64	2073.39
EABT32908	918	1150.98	721	2657.15	1316.58	493	620.05
EABT32909	0	2	1	7	4	0	0
EABT3291	2	1	4	6	2	1	1
EABT32910	0	1	9	0	0	0	0
EABT32911	0	2	3	3	0	0	0
EABT32912	1	6	15	9	2	3	4
EABT32913	11	14	247	24	5	2	6
EABT32914	40.33	5	7	0	0	2	7
EABT32915	2	4	42	0	0	1	1
EABT32916	1	4	9	3	3	3	0
EABT32917	1	0	8	0	0	0	0
EABT32918	4	17	29	4	8	2	0
EABT32919	4	9	3	8	1	2	0
EABT3292	1	6	1	1	3	0	0
EABT32920	2	3	72	1	0	0	0
EABT32921	0	0	4	2	0	0	0
EABT32922	0	0	8	2	1	0	0
EABT32923	0	4	0	6	1	0	0
EABT32924	5	1	2	3	1	0	0
EABT32925	0	1	12	0	0	0	1
EABT32926	584	1473.26	707.01	1893.37	1153.01	667.69	713
EABT32927	5	15	84	24	6	7	10
EABT32928	3892.57	5582.62	3802.73	11313.9	3812.93	2052.55	2177.69
EABT32929	0	4	0	2	0	0	1
EABT3293	2	5	24	2	0	0	1
EABT32930	3452.44	4174.08	6038.52	5214.9	2699.48	2894.14	2539.86
EABT32931	0	1	1	7	0	0	1
EABT32932	0	0	2	1	3	0	0
EABT32933	1	1	3	0	0	0	0
EABT32934	1	8	81	87	6	1	1
EABT32935	0	1	14	1	0	0	1
EABT32936	0	0	6	2	0	0	0
EABT32937	16	14	151	20	5	1	3
EABT32938	9	15	21	18	6	7	8
EABT32939	7790.25	12877.9	14954.4	25952.8	1815.99	1628.98	3113.89
EABT3294	0	0	9	1	0	0	0
EABT32940	2	6	5	27	4	5	2
EABT32941	2	3	9	1	2	1	0
EABT32942	4	3	10	2	2	4	1
EABT32943	0	0	11	1	0	1	1
EABT32944	6	5	0	0	1	9	5
EABT32945	3	3	14	1	3	0	1
EABT32946	0	1	0	1	2	3	0

EABT32947	103.01	205.71	416.99	335.3	91	71	101.24
EABT32948	1	2	31	3	2	0	0
EABT32949	1	0	11	0	0	0	0
EABT3295	10	44	65	32	234.99	5	16
EABT32950	0	0	4	7	1	0	0
EABT32951	1	4	13	3	0	1	1
EABT32952	1	3	19	1	1.04	3	1
EABT32953	0	0	0	0	0	0	1
EABT32954	444	481	197	610	313	119	416
EABT32955	198	505.95	210.27	320.07	841.08	35	139
EABT32956	1186.71	1615.85	807	2006.69	1006.13	820.85	776.55
EABT32957	263.15	655.16	801.75	471.23	506.45	402.15	270.73
EABT32958	444	809.73	941.03	1689.03	957.09	293.96	423.44
EABT32959	3	3	0	0	0	0	0
EABT3296	0	1	2	0	0	0	0
EABT32960	0	0	1	1	0	0	0
EABT32961	1	2	1	1	1	2	2
EABT32962	0	0	1	5	1	0	0
EABT32963	0	1	7	0	13	0	3
EABT32964	0	7	9	0	0	0	0
EABT32965	3960.09	3243.8	1652.1	1170.21	6670.56	674.73	5418.38
EABT32966	0	0	0	0	0	7	4
EABT32967	134.04	77.71	208.99	34	29	28.01	42
EABT32968	3	2.88	7	14	1	0	5
EABT32969	1	0	39	0	1	0	0
EABT3297	0	2	5	0	2	0	0
EABT32970	0	0	9	19	1	0	4
EABT32971	4	5	7	0	0	1	3
EABT32972	1	1	2	5	2	0	2
EABT32973	8	188.54	5	0	201	133	111.24
EABT32974	6	4	4	5	1	1	0
EABT32975	0	4	4	1	1	0	1
EABT32976	1	0	28	1	0	0	0
EABT32977	1	2	10	0	0	1	0
EABT32978	0	1	5	0	0	0	0
EABT32979	1	4	4	0	1	0	0
EABT3298	4	4	1	2	0	0	1
EABT32980	1	14	104	1487.87	83	0	116
EABT32981	115	156	175.99	25	111	152.07	225
EABT32982	4	23	1	16	4	0	3
EABT32983	5213.11	6199.69	5393.4	2859	4085.63	7963.37	7482.11
EABT32984	0	1	0	0	1	2	2
EABT32985	0	0	10	0	0	0	0
EABT32986	3	3	1	4	0	0	0
EABT32987	2	6	15	1	1	2	1
EABT32988	2	3	56	7	1	0	2
EABT32989	1	6	9	7	0	3	1
EABT3299	2	4	11	8	0	1	1
EABT32990	10	18	4	37	9	4	10
EABT32991	7	3	7	1	1	1	0
EABT32992	2670.72	4541.72	5302.03	6758.37	3229.93	2503.25	2421.05

EABT32993	0	3	44	1	1	1	0
EABT32994	2	1	0	0	1	20	8
EABT32995	0	2	1	0	1	0	2
EABT32996	12	39	59	26	68	16	47
EABT32997	2	13	68	9	2	3	4
EABT32998	1	4	16	2	2	3	3
EABT32999	2	1	7	1	2	18	10.21
EABT33	8	84.2	87	230	570.02	0	1
EABT330	1	3	1	2	1	0	1
EABT3300	3	28	23	23	1	1	2
EABT33000	1120.99	1304.99	762	840.19	1113	1380	992.01
EABT33001	1151.03	2405.97	1656.01	765	2518.55	132	1179.01
EABT33002	5601.2	9591.52	13888.2	14417.3	5354.82	7622.17	6012.76
EABT33003	1	3	16	4	1	1	4
EABT33004	14	9	0	0	0	0	0
EABT33005	1	5	10	0	6	2	1
EABT33006	1	1	2	1	0	3	0
EABT33007	0	0	0	2	0	0	1
EABT33008	9416.53	19989.3	17657.4	14106	5053.84	3585.62	4454.89
EABT33009	7	12	43	46	17	2	0
EABT3301	2	2	2	4	1	2	0
EABT33010	0	10	21	2	2	1	2
EABT33011	1	3	6	1.26	2	1	6
EABT33012	0	1	6	17	1	0	0
EABT33013	2	1	2	155	15	0	0
EABT33014	3	3	8	4	0	4	2
EABT33015	1	1.71	7	0	0	0	1
EABT33016	8	93	554.99	102690	5	2	0
EABT33017	0	2	4	0	0	0	0
EABT33018	784.85	1268.03	877.81	3467.29	1158.18	622.62	858.13
EABT33019	6	9	4	4	1	15	1
EABT3302	0	0	0	0	0	0	1
EABT33020	12	6	1	6	1	0	0
EABT33021	2	4	5	22	0	0	0
EABT33022	0	0	2	1	0	1	0
EABT33023	0	0	0	2	1	2	0
EABT33024	1	1	5	2	0	3	1
EABT33025	6	17	11	13	6	0	1
EABT33026	4	11	153	4	6	2	0
EABT33027	0	0	14	0	0	4	1
EABT33028	0	0	7	2	0	0	0
EABT33029	1	0	2	4	2	0	0
EABT3303	0	14	7	1	0	1	1
EABT33030	2850.44	3634.36	3522.85	7900.17	4041.42	3908.87	4028.65
EABT33031	20	35	60	53	8	10	8
EABT33032	2	4	3	21	11	0	0
EABT33033	4	3	3	0	1	4	3
EABT33034	1	5	58	9	4	1	2
EABT33035	6140.19	9594.56	6216.93	18729.2	4604.55	6493.56	4445.9
EABT33036	1	0	5	2	0	1	0
EABT33037	300	622	606.97	904	363	205	166

EABT33038	5	0	4	0	0	1	7
EABT33039	1272.01	3839.33	7534.64	2135	1311.82	1074.35	1129.27
EABT3304	1	0	5	0	0	2	0
EABT33040	3	7	1	1	4	0	0
EABT33041	15	101	76	86	21	17	33
EABT33042	827.79	1212.57	1583.29	2311.86	1070.61	721.98	816.27
EABT33043	0	0	0	0	0	0	0
EABT33044	0	0	0	0	0	0	0
EABT33045	0	1	4	0	1	0	0
EABT33046	1	2	2	1	0	0	1
EABT33047	0	40	34	18	205	4	4
EABT33048	0	0	13.01	1	0	0	0
EABT33049	413	525.98	275	780.28	421.98	333.06	302.06
EABT3305	0	0	5	0	0	0	0
EABT33050	0	0	8	4	0	2	2
EABT33051	1	3	2	0	1	0	0
EABT33052	6	9	16	26	17	5	6
EABT33053	15	7	4	1	2	4	7
EABT33054	2	4	16	6	1	2	0
EABT33055	1	1	7	2	0	1	0
EABT33056	49887	317171	178387	47854.1	36844.5	34027.8	14447.4
EABT33057	0	3	122	1	2	0	0
EABT33058	0	0	6	2	0	0	0
EABT33059	0	2	7	7	0	0	1
EABT3306	19	72	35	14	481	0	16
EABT33060	1	3	4	0	0	0	1
EABT33061	0	2	23	5	0	1	1
EABT33062	690.99	1356.51	2862.23	2863.66	854.01	512.03	749
EABT33063	26	0	0	3	0	0	0
EABT33064	174	361	366	941.02	815.99	145	186
EABT33065	238	794.28	5865.24	1658.87	1094.34	183	1748.75
EABT33066	408.79	679	1224.04	1306	1761.26	60	342
EABT33067	0	1	19	3	0	0	1
EABT33068	0	2	1	4	0	0	1
EABT33069	0	1	3	1	1	0	0
EABT3307	747.42	922	704.98	1351.96	1011.71	829.67	679.91
EABT33070	4305.43	7530.58	1008	1944	440	1236.12	303
EABT33071	99	54	83	877.07	258.76	1	0
EABT33072	2	2	19	7	1	0	1
EABT33073	1	1	10	7	3	1	0
EABT33074	3	4	0	0	0	3	0
EABT33075	2	2	0	0	1	1	4
EABT33076	8	4	33	10	3	11	3
EABT33077	1	1	9.79	3	5	3	3
EABT33078	0	0	5	0	0	3	2
EABT33079	11	22	112	35	0	0	1
EABT3308	3	4	29	7	2	6	1
EABT33080	6	4	25	8	3	1	3
EABT33081	0	2	7	2	0	0	0
EABT33082	0	2.64	1	1	1	0	1
EABT33083	0	0	13	1	0	0	1

EABT33084	4	9	1	1	1	1	2
EABT33085	3	2	6	7	0	0	0
EABT33086	22681.7	82645.5	36228.6	53892.5	16289.3	22254.9	30272.4
EABT33087	0	2	1	1	5	0	0
EABT33088	3	3	5	2	3	0	0
EABT33089	0	0	1	0	0	1	0
EABT3309	1062	205	807	5842.18	1140.13	0	17
EABT33090	15	32	45	226.96	3	42	19
EABT33091	0	3	4	29	3	0	0
EABT33092	6	3	0	4	1	0	0
EABT33093	3	6	30	2	3	4	2
EABT33094	1	0	1	0	1	1	1
EABT33095	0	1	7	0	0	1	0
EABT33096	9	17	14	4	8	0	0
EABT33097	1	0	34	0	13	2	0
EABT33098	2	0	2	3	0	0	1
EABT33099	5	20	6	34	2	0	13
EABT331	2	1	6	3	4	1	10
EABT3310	5	2	0	0	0	2	1
EABT33100	15	39	22	171.95	1591	5	7
EABT33101	3	57	70	52	17	0	3
EABT33102	1	1	4	0	0	0	0
EABT33103	1992.8	3065.3	5100.44	2522.67	2084.01	2293.01	2740.09
EABT33104	551.94	591.94	4101.72	1175.98	527.41	465.04	324
EABT33105	1488	1952.66	1010.99	2465.01	966	1780	1389.03
EABT33106	1	0	0	2	0	2	0
EABT33107	3	9	155	5	3	4	2
EABT33108	11	27	2	0	0	55	18
EABT33109	1	4	1	0	1	0	0
EABT3311	0	1	3	8	0	0	0
EABT33110	0	0	0	5	0	0	1
EABT33111	569.24	1067.41	1903.78	728.94	859	83	219
EABT33112	1386.09	2127.55	1561.26	1346.98	2221.14	2019	1785.17
EABT33113	356	419.02	434	1380.73	809.7	202	336
EABT33114	1	0	2	1	0	1	0
EABT33115	469.9	605.63	1782.84	1466.89	518.67	327.01	313
EABT33116	0	4	0	0	0	11	9
EABT33117	0	2	2	2	0	0	1
EABT33118	5714.04	9748.53	11690.1	9978.73	2462.16	3983.17	2965.75
EABT33119	0	0	2	4	1	0	0
EABT3312	348.94	949.59	487.19	940.39	797.11	346	633.72
EABT33120	2	0	1	1	0	1	0
EABT33121	11	5	47	17	7	4	9
EABT33122	2	2	13	2	1	0	2
EABT33123	589.95	1026.72	1105.02	1417.76	1323.34	462	720.45
EABT33124	1	1	7	2	1	1	0
EABT33125	3	3	13	7	5	2	13
EABT33126	17	2	10	34	16	0	2
EABT33127	1177.16	1495.09	818.36	1500.76	1428.81	674.49	1018
EABT33128	0	3	4	0	3	3	6
EABT33129	598.24	130	415	78	1021.11	836.6	94

EABT3313	0	1	5	0	0	0	0
EABT33130	1	3	5	3	1	0	0
EABT33131	0	2	4	2	0	0	2
EABT33132	0	0	1	1	2	0	1
EABT33133	2	0	3	7	0	0	4
EABT33134	0	1	11	2	0	0	0
EABT33135	0	1	3	0	1	0	1
EABT33136	0	0	0	0	0	0	0
EABT33137	2	2	0	0	0	0	1
EABT33138	1362.03	1885.13	1716.21	2913.74	898	973.28	913.99
EABT33139	307.05	316.23	72.57	205	469	383.79	665.99
EABT3314	4	2	58	2	1	2	0
EABT33140	0	1	7	1	6	0	3
EABT33141	2	0	2	2	0	0	0
EABT33142	13	7	27	3	3	14	9
EABT33143	1	2	10	3	1	0	0
EABT33144	3	5	4	6	2	1	3
EABT33145	15	6	1	0	0	1	0
EABT33146	808	1535	793.01	694.79	383	1965.97	1227.47
EABT33147	2015	3095.02	5117.89	2430.95	2374	1828.46	2522.25
EABT33148	333	549.7	232	287	389.96	5	34
EABT33149	0	0	1	0	3	3	2
EABT3315	0	3	0	0	6	9	34
EABT33150	21	171	199	185	67	151	1
EABT33151	1	2	1	1	3	9	10
EABT33152	0	0	4	0	0	0	0
EABT33153	1	0	3	2	1	0	0
EABT33154	0	0	3	5	1	0	0
EABT33155	13	16	19	28	2	47	22
EABT33156	2	0	4	3	1	1	0
EABT33157	1	3	20	13	3	7	10
EABT33158	2	4	3	1	1	2	5
EABT33159	0	0	0	0	1	0	0
EABT3316	29661.9	10865.8	4821.11	30671.1	9213.91	37656	36414.4
EABT33160	1659.08	2167.77	1725.03	3171.28	2219.26	1212.07	1389.82
EABT33161	0	2	11	5	1	5	3
EABT33162	0	0	5	1	0	0	0
EABT33163	18	10	0	0	0	7	12
EABT33164	0	0	10	4	1	0	0
EABT33165	1	3	16	0	1	1	2
EABT33166	1	5	6	13	1	1	1
EABT33167	0	1	8	0	0	1	1
EABT33168	0	0	13	2	0	0	0
EABT33169	0	0	3	0	0	0	0
EABT3317	4	7	6	1	5	7	11
EABT33170	1001.47	1419	911.52	1251.98	1092.83	961.82	1159.97
EABT33171	7199.2	12341.7	5930.94	11149.8	7304.81	6857.69	7594.91
EABT33172	6	15	17	29	43	6	27
EABT33173	0	0	0	0	9.5	67.01	42.49
EABT33174	742942	694823	428047	364267	703253	232743	324698
EABT33175	0	0	5	2	1	1	0

EABT33176	1	1	4	7	2	0	2
EABT33177	0	3	1	3	0	0	0
EABT33178	160.01	259	347	548	341.01	98.95	151
EABT33179	4	3	8	8	2	6	2
EABT3318	0	1	12	0	0	0	0
EABT33180	1	1	4	30	72	0	30
EABT33181	2	3	3	1	0	1	5
EABT33182	7	4	2	4	0	0	0
EABT33183	0	1	85	939.03	6	0	10
EABT33184	0	6	26	3	1	1	2
EABT33185	2	1	9	10	6	0	1
EABT33186	5	16	29	30	6	3	10
EABT33187	1771.65	3213.51	4406.4	4510.32	2238.42	2720.91	2047
EABT33188	297	3	58.01	2916.78	47	6	2
EABT33189	0	1	2	0	1	0	0
EABT3319	27	35	0	0	4	54	37
EABT33190	4	3	4	0	0	0	0
EABT33191	0	0	15	1	0	1	0
EABT33192	0	1	5	0	0	0	1
EABT33193	800.98	998.02	411.42	1057.94	627	254	554.88
EABT33194	0	3	9	4	0	0	2
EABT33195	5	33	17	26	4	1	1
EABT33196	0	1	14	0	0	0	0
EABT33197	0	1	20	0	1	0	0
EABT33198	4	4	10	2	1	0	5
EABT33199	0	0	1	5	0	0	0
EABT332	19	21	12.99	205	12	8	32
EABT3320	0	2	13	0	0	0	0
EABT33200	0	2	25	0	0	1	1
EABT33201	1	4	1	0	4	0	10
EABT33202	4	6	6	0	2	2	1
EABT33203	0	9	30	16	0	0	0
EABT33204	1	0	40	12	1	0	0
EABT33205	1	1	3	5	0	1	0
EABT33206	40	56	30	81	79	14	45
EABT33207	3	2	7	2	2	1	1
EABT33208	36	81	45	33	50	29	19
EABT33209	0	0	6	0	0	0	0
EABT3321	4	12	0	0	1	26	14
EABT33210	1	11	0	5	6	4	7
EABT33211	28	36	36	85	41	15	24
EABT33212	3	8	3	0	0	0	4
EABT33213	5507.1	5131.34	1210.03	423	2186.44	4945.5	4531.43
EABT33214	0	0	1	2	0	0	1
EABT33215	8	24	47	13	9	16	7
EABT33216	0	2	0	1	2	0	0
EABT33217	0	0	8	0	0	1	0
EABT33218	0	1	4	2	1	0	1
EABT33219	0	1	0	1	5	0	3
EABT3322	3	2	1	5	0	1	0
EABT33220	3	12	8	1	0	2	1

EABT33221	0	1	60.06	3	1	0	0
EABT33222	0	2	1	1	3	0	0
EABT33223	645.4	1149.36	1015.24	4094.59	1942.82	137	864.31
EABT33224	1794.3	6305.1	3486.19	4578.64	1082.9	117	577.41
EABT33225	85	58	201	24	33	18	39
EABT33226	3	29	242	31	4	8	2
EABT33227	1	0	3	1	0	0	0
EABT33228	350	320	20	32	44	48	53
EABT33229	1039.97	1705.02	1557.35	2375.24	1913.93	848.97	1340.74
EABT3323	0	6	4	13	2	1	2
EABT33230	1	1	8	8	3	0	7
EABT33231	30	36	0	0	0	9	4
EABT33232	0	0	6	0	0	0	1
EABT33233	0	3	19	1	1	0	2
EABT33234	10929.7	15404.1	12708.5	4369.7	5221.58	13421.3	7369.12
EABT33235	2	1	13	1	0	0	1
EABT33236	0	1	4	3	0	3	0
EABT33237	9	6	3	567.34	132	22	340
EABT33238	0	4	15	4	1	2	1
EABT33239	1629	2430.88	3774.23	3979.45	2252.05	1501.18	1626.82
EABT3324	2	3	30	4	2	3	2
EABT33240	0	1	10	2	0	4	0
EABT33241	5	0	0	2	1	0	0
EABT33242	0	1	6	1	0	0	0
EABT33243	1	0	0	0	1	0	0
EABT33244	48	72.6	38	116	78.02	24	39
EABT33245	5	5	11	3	2	3.73	0
EABT33246	7730.01	15338.9	7425.05	36	2353	7584.08	2667.86
EABT33247	4	0	2	1	3	1	2
EABT33248	824.99	1623.76	2073.38	3015.25	970.63	394	324.99
EABT33249	8	5	1	0	5	12	5
EABT3325	0	3	3	1	0	0	0
EABT33250	1	5	4	2	0	1	0
EABT33251	409	724	393	1199.54	509	284	290
EABT33252	2313.14	3543.55	2546.13	7907.26	3655.52	2974.24	3610.15
EABT33253	1	1	1	12	0	0	0
EABT33254	3	1	6	1	0	1	0
EABT33255	1	0	3	0	0	6	0
EABT33256	0	1	0	5	0	0	0
EABT33257	2	2	46	1	0	4	1
EABT33258	2465.37	4723.98	3796.15	8902.39	3351.31	2549.3	2625.05
EABT33259	2	5	6	3	1	1	6
EABT3326	4405.09	7564.91	12356.6	6343	7096.16	317	1243.96
EABT33260	1	2	22	0	1	2	0
EABT33261	0	0	0	20	2	0	0
EABT33262	588.68	928.03	859.02	2728.86	4383.21	742.02	589.27
EABT33263	3	4	8	16	5	0	1
EABT33264	1	2	3	2	4	1	4
EABT33265	0	1	1	10	1	0	0
EABT33266	344	505.94	580	872.55	372.62	141	206.88
EABT33267	186	318.94	297	615	335	121	276

EABT33268	1141.79	2080.29	2163.84	2472.92	950	1554.76	919.58
EABT33269	0	1	14	2	0	0	0
EABT3327	1	3	2	4	1	3	2
EABT33270	24289.6	4570.9	513	11126.5	5372.98	806	831
EABT33271	66	155	89.26	749.7	84	17.58	15
EABT33272	0	0	5	0	0	1	0
EABT33273	555	909.71	734	1538.04	889.11	468.99	596
EABT33274	0	10	5	4	1	0	0
EABT33275	0	2	8	4	0	4	0
EABT33276	42	11	5	1	6	40	16
EABT33277	1	0	9	2	2	0	0
EABT33278	0	2	2	37	1	2	1
EABT33279	0	1	5	0	0	0	1
EABT3328	1	1	4	1	0	4	3
EABT33280	0	0	7	0	0	0	0
EABT33281	1	8	21	1	2	0	0
EABT33282	2	0	21	1	1	0	2
EABT33283	0	1	0	12	0	0	0
EABT33284	6	2	7	1	0	0	1
EABT33285	0	2	55.61	3	2	0	0
EABT33286	41	36	134	10	0	2	1
EABT33287	0	1	3	0	1	0	0
EABT33288	630.66	915.02	1213.92	1522.84	667.55	605.99	478.02
EABT33289	11	0	1	0	7	10	5
EABT3329	0	1	0	0	2	0	12
EABT33290	21	13	0	0	0	197	0
EABT33291	5	16	30	25	20	9	13
EABT33292	0	3	4	4	1	0	1
EABT33293	0	0	10	0	0	0	0
EABT33294	0	4	3	0	0	0	2
EABT33295	1	3	2	4	1	0	0
EABT33296	4	3	4	2	6	0	6
EABT33297	1422.8	218.04	15	2	176.56	1076.77	687.68
EABT33298	8	2	3	4	1	0	0
EABT33299	0	0	9	0	3	0	4
EABT333	11	11	13	74	7	9	9
EABT3330	22	38	63	65	68	22	21
EABT33300	169	89.15	115	81	55.84	13	348.49
EABT33301	1	2	4	1	0	3	0
EABT33302	1740.46	3839.01	4744.55	7447.01	3128.65	1486.11	2332.33
EABT33303	1	6	0	0	1	11	0
EABT33304	0	0	2	1	0	0	0
EABT33305	1	3	13	9	2	0	5
EABT33306	0	3	6	0	0	0	0
EABT33307	0	2.13	19	2	1	0	2
EABT33308	7	11	8	15	13	3	16
EABT33309	2	3	61	6	2	0	0
EABT3331	290	338	101	320	209	171	126
EABT33310	0	13	35	36	1	1	0
EABT33311	13	5	2	1	0	0	2
EABT33312	0	2	2	16	0	0	0

EABT33313	2	0	6	5	7	1	449.2
EABT33314	0	0	4	0	1	0	0
EABT33315	0	0	4	6	0	0	1
EABT33316	0	1	2	2	0	0	0
EABT33317	3	0	0	0	2	0	4
EABT33318	4	9	18	6	5	24	23
EABT33319	0	2	24	12	1	0	0
EABT3332	0	5	43	6	0	0	1
EABT33320	0	4	7	0	0	0	0
EABT33321	1240.95	2375.34	5882.07	2456.98	865.34	961.03	1039
EABT33322	0	2	0	1	0	0	1
EABT33323	1574.3	1125.4	379.23	582.98	421.05	3348.22	2638.19
EABT33324	13	15	0	0	3	50	32
EABT33325	3	14	36	971.32	16	42	76
EABT33326	1375	2266	1341	3344.46	1013.96	610	692
EABT33327	6	13	121	3	0	3	1.83
EABT33328	1	3	1	1	2	1	1
EABT33329	2727.09	4038	4646.95	5539.28	2832.79	2985.42	2709.32
EABT3333	0	1	12	2	0	0	0
EABT33330	15	95	410	299.84	91	29	30
EABT33331	1	3	20	1	0	0	2
EABT33332	0	1	4	0	0	0	1
EABT33333	8200.81	14063.9	7294.64	12881.2	5219.29	8787.69	9640.71
EABT33334	2	4	4	11	2	0	0
EABT33335	0	0	3	0	0	0	3
EABT33336	1	1	6	0	0	0	0
EABT33337	1	1	23	2	0	0	1
EABT33338	0	0	4	17	0	0	0
EABT33339	85691.9	3631.68	36	58.65	4868.28	110387	26357.8
EABT3334	0	0	0	0	0	0	0
EABT33340	0	1	5	3	0	1	0
EABT33341	1	0	0	0	0	0	0
EABT33342	29	6	5	1	0	8	0
EABT33343	0	6	7	0	1	0	0
EABT33344	5	8	61	4	35	2	17
EABT33345	0	1	1	6	1	0	0
EABT33346	1632.32	2468.95	2098.45	3356.43	2163.06	1796.99	1325.73
EABT33347	506.49	741.94	246.99	812.95	509.34	239.01	443
EABT33348	0	0	6	0	0	0	0
EABT33349	0	1	6	1	0	0	0
EABT3335	32	94.04	101	333	150	40	58
EABT33350	3	13	23	36	36	0	11
EABT33351	0	1	1	4	0	0	0
EABT33352	0	0	8	1	1	0	0
EABT33353	1	2	3	0	1	0	0
EABT33354	0	0	3	0	0	0	0
EABT33355	0	27	39	113.04	3	0	0
EABT33356	0	3	9	1	0	0	0
EABT33357	10971.1	8464.24	5047.57	16905	5527.28	9675.41	7909.46
EABT33358	18	4	2	1	0	21	0
EABT33359	0	1	20	2	1	5	3

EABT3336	0	0	2	8	2	0	0
EABT33360	213.34	614.13	505.28	20188.4	1139.01	207.07	193
EABT33361	3	1	6	1	0	0	0
EABT33362	434	2069.14	3244.3	1035	5612.86	63	940
EABT33363	1	0	7	0	0	0	0
EABT33364	9	34	16	1	1	13	0
EABT33365	0	1	5	1	0	0	0
EABT33366	1	3	5	5	2	0	0
EABT33367	7	7	24	1	4	4	2
EABT33368	3	2	2	1	1	4	0
EABT33369	40	0	0	0	1	7	12
EABT3337	0	1	19	1	1	1	2
EABT33370	217.91	311	703.72	9324.97	657.98	122	153
EABT33371	6630.45	15252.5	12467.3	7053.8	6347.04	4803.44	3460.67
EABT33372	0	2	3	9	0	1	1
EABT33373	56	112.46	80	120	129	109.53	73
EABT33374	1286	1785	745.01	1203.21	1699.37	797	631
EABT33375	0	0	6	0	0	0	0
EABT33376	1	0	0	2	0	0	0
EABT33377	2	0	12	0	0	2	0
EABT33378	2	15	36	32	2	2	2
EABT33379	2096.74	1536	1300	9652.91	993	1463	1241.99
EABT3338	0	1	5	62.37	6	0	1
EABT33380	7	12	3	10	1	0	2
EABT33381	0	3	13	1	0	0	1
EABT33382	1	3	13	0	0	2	0
EABT33383	0	3	3	1	0	0	0
EABT33384	16	81	29	15	60	4	105
EABT33385	6893.01	12646.8	3981.73	8143.89	2342.75	862	1163.92
EABT33386	64	56	196	59	84	41	40
EABT33387	1	0	15	0	0	5	3
EABT33388	1	8	19	3	0	1	4
EABT33389	7985.8	18844	19076.3	12536.1	13694.1	1612.86	4267.66
EABT3339	2	6	0	0	0	1	1.01
EABT33390	1	6	4	37	7	1	1
EABT33391	0	2	0	0	0	5	1
EABT33392	18	45	76	5	9	15	13
EABT33393	1	1	16	3	5	0	0
EABT33394	6	3	6	7	0	7	4
EABT33395	692	990.64	551.76	1671	877.13	454.04	618
EABT33396	1	3	7	0	1	2	0
EABT33397	0	0	10	0	1	0	0
EABT33398	1	0	11	3	2	0	2
EABT33399	1826.02	5356.6	1232.79	331	4968.94	3749.66	39797.9
EABT334	0	0	3	6	0	0	0
EABT3340	2	10	12	4	1	1	6
EABT33400	0	9	17	8	10	1	1
EABT33401	2	8	8	22	7	0	4
EABT33402	0	1	2	1	4	0	0
EABT33403	0	0	23	2	0	0	1
EABT33404	0	0	4	1	0	0	0

EABT33405	0	3	50	9	0	0	4
EABT33406	0	3	4	2	0	1	1
EABT33407	1	0	0	2	2	0	0
EABT33408	1	2	3	2	0	1	1
EABT33409	0	0	15	0	0	0	0
EABT3341	0	1	5	0	0	0	0
EABT33410	1	19	23	110	29	0	1
EABT33411	0	0	2	0	0	0	0
EABT33412	3	1	12	4	4	0	2
EABT33413	0	3	4	0	0	0	0
EABT33414	0	1	29	1	0	0	0
EABT33415	2	1	58	4	0	2	0
EABT33416	0	4	2	1	0	1	1
EABT33417	0	0	9	0	0	0	0
EABT33418	0	1	12	4	1	5	3
EABT33419	1	0	13	1	0	0	0
EABT3342	1480.99	1656	804.45	2998.65	437	2344.54	719.87
EABT33420	0	3	2	0	0	0	0
EABT33421	6889.43	9358.51	3393.56	264.52	2520.87	1305.25	658.02
EABT33422	411	217	8	8.43	54	35	116
EABT33423	0	1	1	1	0	0	0
EABT33424	0	0	0	0	0	7	0
EABT33425	1604	2558	193.01	1326	3867.72	33	1654
EABT33426	2	4	2	1	0	2	5
EABT33427	0	0	19	2	0	0	0
EABT33428	353.45	374.07	284.98	406.27	530.81	341.05	425.07
EABT33429	0	0	4	0	0	0	0
EABT3343	0	0	4	2	2	1	0
EABT33430	113	196	148	392.77	345	97	158
EABT33431	1	1	0	1	1	0	6
EABT33432	0	3	98.04	2	0	0	2
EABT33433	2	5	7	10	1	0	0
EABT33434	0	0	5	1	0	2	2
EABT33435	0	0	7	1	2	0	0
EABT33436	0	3	3	3	0	0	0
EABT33437	1	1	0	4	0	0	0
EABT33438	4	4	5	2	2	0	0
EABT33439	185	171	35	113	149	230.77	165
EABT3344	5	23	25	2	16	1	2
EABT33440	4	5	16	0	0	0	0
EABT33441	73	755.5	1486.58	577.97	894.62	28	129
EABT33442	431	98	15	0	7	12	24
EABT33443	0	5	2	2	1	0	0
EABT33444	1	1	1	4	0	0	0
EABT33445	0	6	2	1	0	0	0
EABT33446	0	0	16	0	0	1	0
EABT33447	0	5	18	4	1	0	0
EABT33448	1	1	12	3	0	1	1
EABT33449	911.25	1288.9	1208.59	1625.68	1124.06	812.5	1232.94
EABT3345	0	0	1	12	0	0	0
EABT33450	9	13	5	5	3	11	5

EABT33451	0	0	0	0	0	0	0
EABT33452	9	3	46	33.01	8	1	0
EABT33453	0	0	5	0	0	0	0
EABT33454	3	6	7	4	0	1	2
EABT33455	4	14	11	4	0	1	0
EABT33456	5	2	4	0	0	0	1
EABT33457	2	3	5	0	0	0	0
EABT33458	580	2119.13	5777.41	2760.7	2221.56	149	410
EABT33459	0	1	3	3	0	0	0
EABT3346	0	0	0	4	53	0	0
EABT33460	5	19	34	51	21	10	17
EABT33461	2	15	13	3	2	1	0
EABT33462	2	3	1	0	0	0	1
EABT33463	0	1	11	2	1	4	0
EABT33464	1	0	95	2	0	0	0
EABT33465	0	5	3	0	0	1	2
EABT33466	840	1287.08	792	1000	354.1	310	490.06
EABT33467	9	66	7	0	3	23	14
EABT33468	2	11	4	0	0	0	0
EABT33469	183	384	71	53	95	489	263.05
EABT3347	0	3	73	0	0	0	1
EABT33470	0	3	3	0	0	0	0
EABT33471	1	4	24	0	7	0	0
EABT33472	8	14	101	10	2	27	6
EABT33473	0	4	6	1	1	0	0
EABT33474	0	2	6	2	0	4	0
EABT33475	19	17	5	11	19	5	10
EABT33476	102.57	205.02	182	1504.9	316	28	125
EABT33477	1	1	3	0	0	1	0
EABT33478	0	0	3	0	1	1	0
EABT33479	0	0	1	4	0	0	0
EABT3348	11	18	10	2	25	23	0
EABT33480	901.89	1195.02	983.11	2310.89	1022.19	1060.53	640.98
EABT33481	1	2	3	2	0	8	0
EABT33482	0	0	6	0	0	0	0
EABT33483	1	2	4	2	0	0	0
EABT33484	0	1	1	0	4	0	0
EABT33485	0	0	20	2	0	2	0
EABT33486	0	0	12	1	2	0	0
EABT33487	0	0	1	0	0	0	6
EABT33488	0	1	17	1	2	0	0
EABT33489	0	1	5	0	0	0	1
EABT3349	157.42	401.89	579.22	145.98	227.37	74	198.76
EABT33490	0	0	5	2	0	0	0
EABT33491	2628	2796.31	1478.27	2637.63	1313	2524.73	2518.47
EABT33492	0	6	7	5	0	3	1
EABT33493	1	3	16	3	0	1	0
EABT33494	3	5	3	6	4	0	3
EABT33495	1	0	7	2	1	0	1
EABT33496	29	95	54	57	6	7	5
EABT33497	1	2	1	2	0	0	0

EABT33498	2	1	3	4	0	3	1
EABT33499	146	185	0	0	12	22	7
EABT335	0	3	5	1	1	0	1
EABT3350	0	3	18	2	0	0	0
EABT33500	5057.26	10902.5	7002.52	11389.9	4291.36	5652.48	7497.92
EABT33501	0	1	4	2	4	0	0
EABT33502	1281.03	3295.14	6638.05	6906.75	1204.22	46	189
EABT33503	1	7	6	22	4	3	4
EABT33504	1	2	0	0	1	14	4
EABT33505	2	8	12	8	0	1	3
EABT33506	4	5	4	1	1	0	2
EABT33507	2	1	1	1	0	0	2
EABT33508	1	1	1	42	1	1	0
EABT33509	0	2	0	0	0	0	0
EABT3351	11	56	515.23	86	41	5	7
EABT33510	3	6	14	4	2	8	4
EABT33511	0	1	6	0	0	0	0
EABT33512	0	0	7	0	0	0	0
EABT33513	43422.1	52541.1	21962.2	70724.7	27351.7	29926.8	28138.5
EABT33514	0	0	3	0	1	0	1
EABT33515	8397.99	704	22	3	8	3261.76	5
EABT33516	3	18	119	20	3	3	14
EABT33517	70.58	97.99	57	208.97	60	26	61.19
EABT33518	4	8	4	30	4	3	2
EABT33519	0	0	8	0	0	0	0
EABT3352	157	188.97	144.87	120.98	127	50.08	67
EABT33520	0	0	0	7	0	0	0
EABT33521	131.36	64	4	31	41	0	2
EABT33522	1	0	2	12	0	1	0
EABT33523	57.95	357	85	48	77	81	163.69
EABT33524	0	0	16	0	0	0	0
EABT33525	14.98	15	3	5	7	0	16
EABT33526	1230.83	2138.75	1580.67	2419.26	1342.11	710.28	1029.97
EABT33527	201.1	366	499	502	347	370	273
EABT33528	72	119	102.41	61	39	24	62
EABT33529	0	0	1	3	0	0	1
EABT3353	0	3	6	0	2	1	1
EABT33530	0	2	2	38	4	0	1
EABT33531	3	10	5	13	1	2	2
EABT33532	1	0	2	4	0	0	0
EABT33533	0	0	9	0	0	0	0
EABT33534	0	2	22	2	1	7	4
EABT33535	4605.91	5501.36	5013.44	9861.53	3039.05	4636.78	4315.28
EABT33536	1830.15	2774.02	2802.07	32503.4	501	90	41
EABT33537	63	120	1121.29	590.9	52	132.01	78.01
EABT33538	1	2	1	0	1	0	0
EABT33539	0	0	5	110	1	2	4
EABT3354	0	0	15	0	0	0	0
EABT33540	873.27	1579.47	658.73	1436	879.82	421	2764.14
EABT33541	0	0	2	1	0	0	0
EABT33542	0	0	1	0	6	0	4

EABT33543	1	0	67	1	1	1	0
EABT33544	3.11	6	18	11300.2	5475.11	2	116
EABT33545	0	2	42	2	0	0	1
EABT33546	3	40	27	360	12	9	17
EABT33547	0	1	5	1	0	3	0
EABT33548	1	1	3	1	0	0	1
EABT33549	0	2	3	0	0	0	0
EABT3355	7	21	11	27	27	4	11
EABT33550	1979.07	3552.32	3757.78	8863.55	1797.5	2046.02	1435.37
EABT33551	1	11	14	8	4	1	7
EABT33552	1	3	2	0	0	1	1
EABT33553	0	0	0	5	1	0	0
EABT33554	868	1993	3998	3478	1886	882	1659
EABT33555	0	1	34	1	0	1	0
EABT33556	3926.56	5693.97	3117.4	3087.28	2228.64	3891.06	2576.03
EABT33557	3	5	6	7	1	1	1
EABT33558	0	0	4	0	0	0	0
EABT33559	0	0	0	0	0	0	0
EABT3356	0	7	5	5	1	0	3
EABT33560	19	19	13	27	5	9	9
EABT33561	0	0	6	4	2	0	0
EABT33562	1	9	14	13	1	0	0
EABT33563	6	3	9	50.13	486.02	4	13
EABT33564	5	1	2	1	0	0	0
EABT33565	3936.4	4810.13	3216.53	5474.23	2365.16	3606.64	2341.24
EABT33566	0	4	8	2	0	4	1
EABT33567	0	0	9	2.97	0	0	0
EABT33568	0	0	9	0	0	0	0
EABT33569	2	0	8	1	0	0	1
EABT3357	0	0	6	0	2	0	0
EABT33570	1	7	17	8	0	1	0
EABT33571	20	36	3	26	13	2	21
EABT33572	0	1	4	3	0	0	0
EABT33573	533.5	1025.59	190.07	113.6	455.32	1220.88	834.22
EABT33574	407	1050.49	2627.55	8973.22	1119.37	254	391.01
EABT33575	4	4	22	7	5	5	4
EABT33576	2	0	1	0	0	0	0
EABT33577	0	0	33	0	0	0	0
EABT33578	3	18	21	51	17	2	24
EABT33579	8	9	2	13	12	9	3
EABT3358	796	1097.91	2690	2318.48	583	722.92	542.66
EABT33580	0	0	11	4	1	0	2
EABT33581	1	2	9	3	0	0	0
EABT33582	1	1	3	3	0	0	0
EABT33583	1	3	4	0	0	4	1
EABT33584	7	13	20	34	265	25	8
EABT33585	1	0	1	1	1	0	1
EABT33586	0	1	30	1	1	2	0
EABT33587	1	3	3	9	0	1	1
EABT33588	0	0	5	3	0	0	0
EABT33589	0	1	5	0	0	1	0

EABT3359	12	7	47	1	1	0	0
EABT33590	0	1	2	4	1	0	0
EABT33591	0	1	1	1	0	0	0
EABT33592	4067.21	8504.26	5912.15	10489.3	4177.39	4674.88	5292.97
EABT33593	0	0	3	3	0	0	0
EABT33594	0	0	0	22	0	0	0
EABT33595	0	2	6	4	2	0	1
EABT33596	0	1	13	1	0	0	0
EABT33597	0	0	8	1	2	0	0
EABT33598	1946.52	2491	2588.92	2910.1	873.05	1795.21	1824.98
EABT33599	5263.92	6890.84	11126.2	18603.9	4140.81	4571.23	2650.83
EABT336	0	4	79	1	6	1	2
EABT3360	7	14	61	49	5	6	17
EABT33600	0	1	1	2	2	0	0
EABT33601	21	89	93	66	6	6	3
EABT33602	0	1	3	0	0	0	0
EABT33603	0	1	2	3	0	1	1
EABT33604	17	50	9	1	3	24	48.01
EABT33605	0	0	5	0	5	0	0
EABT33606	0	2	41	2	1	2	1
EABT33607	2	2	2	0	0	1	0
EABT33608	0	2	3	2	0	1	1
EABT33609	1	0	13	1	0	0	0
EABT3361	0	0	0	1	3	0	4
EABT33610	2	5	129	14	5	0	2
EABT33611	285.08	467	558.37	728.65	405.69	251	272.63
EABT33612	454.39	730.97	738.19	894.76	747.76	457.93	442
EABT33613	0	1	51	3	0	0	0
EABT33614	1	2	7	0	0	0	0
EABT33615	2	11	12	23	2	3	6
EABT33616	0	6	10	85	8	0	2
EABT33617	0	3	2	13	0	0	0
EABT33618	785.14	1257.79	586.93	1524.99	1113	834	826.98
EABT33619	37	154	521.25	537	55.77	30	22
EABT3362	37	269.25	517.02	1185	709.01	65	219
EABT33620	3040.52	3842.54	12498.9	8259.16	1797.02	4705.27	4309.28
EABT33621	0	1	8	2	0	0	0
EABT33622	5	7	3	10	0	0	2
EABT33623	23	20	9.97	19	7	1	0
EABT33624	1	1	1	1	2	0	1
EABT33625	0	1	1	0	1	0	0
EABT33626	20173.8	3156.02	32	2	4	17269.3	26
EABT33627	1	0	6	2	1	0	0
EABT33628	0	0	6	1	1	0	0
EABT33629	2	3	67.01	11	6	5	4
EABT3363	0	0	5	0	0	0	2
EABT33630	2	2	25	8	1	1	1
EABT33631	11	29	16	16	18	38	18
EABT33632	0	1	2	0	1	2	0
EABT33633	22	36	51	63	26	7	8
EABT33634	4	8	51	7	4	4	1

EABT33635	0	0	1	2	0	0	0
EABT33636	54220.6	68310.2	180389	304.99	848.2	1328.56	1510.51
EABT33637	3	6	49	83	4	0	0
EABT33638	691.99	1033.96	1229.4	1515.85	1037.85	626.95	672.81
EABT33639	0	2	3	0	0	0	0
EABT3364	0	0	4	1	1	2	0
EABT33640	3	2	2	4	0	0	1
EABT33641	0	2	1	1	6	1	0
EABT33642	0	0	0	0	0	0	1
EABT33643	2171.01	7513.2	2579.73	1383.62	1698	1525	3718
EABT33644	406.77	809.68	4148.54	1848.21	848.14	361.27	852.27
EABT33645	581.65	836	1020.23	1624.09	1135.93	618	528
EABT33646	0	1	3	28	0	0	0
EABT33647	14217.1	6242	552.11	530.14	22444.1	32533.7	153248
EABT33648	0	1	21	0	2	0	0
EABT33649	2	6	226.95	0	2	0	2
EABT3365	0	0	5	2	1	0	0
EABT33650	0	0	15	1	0	0	1
EABT33651	0	0	6	1	0	0	0
EABT33652	113.01	133	101	47	23	6	2
EABT33653	1	6	6	1	1	0	0
EABT33654	1390	2424.36	1656.79	684	884.03	15	42
EABT33655	32	42	211.01	31	26	22	57
EABT33656	1	8	10	11	3	0	5
EABT33657	0	0	0	0	0	0	0
EABT33658	8	21	32	34	44	12	25
EABT33659	156.01	1062.72	3341.52	54117.8	3131.28	45	171
EABT3366	0	2	2	14	4	0	0
EABT33660	719.74	1206	1503.03	1670	141	1960	60
EABT33661	602.13	831.96	1143	2128.58	698.13	431.09	470.04
EABT33662	2	0	6	0	0	0	2
EABT33663	3662.21	4792.45	3392.96	6068.58	3605.29	2078.65	2397.51
EABT33664	560.28	1063.17	352.88	1185	589.03	131	225.18
EABT33665	0	2	2	3	1	0	0
EABT33666	0	3	25	1	0	0	1
EABT33667	3	1	16	5	0	10	11
EABT33668	0	0	6	4	0	0	1
EABT33669	1	2	0	0	0	2	1
EABT3367	3	0	8	1	1	0	1
EABT33670	1643.01	4277.04	1936	5031.94	2458	147	494
EABT33671	0	0	2	2	0	1	0
EABT33672	2	12	3	17	1	2	1
EABT33673	0	0	2	12	0	0	0
EABT33674	0	5	0	0	0	7	1
EABT33675	0	2	23	3	2	0	0
EABT33676	10	13	16	17	2	3	1
EABT33677	0	4	31.01	8	9	5	3
EABT33678	6820.27	8675.01	7155.7	3020.97	9916.32	4074	4148.71
EABT33679	4	6	1	0	0	0	0
EABT3368	7	9	16	9	6	3	9
EABT33680	1	9	10	6	15	0	22

EABT33681	3	9	8	20	1	11	8
EABT33682	7	9	0	0	0	0	0
EABT33683	0	0	5	0	0	0	0
EABT33684	128	144	177.03	502.96	278.92	86	50
EABT33685	1	1	0	3	5	3	4
EABT33686	0	1	39	0	0	0	0
EABT33687	0	0	3	27	0	1	0
EABT33688	5	2	11	1	2	1	0
EABT33689	2708.57	3293.82	4371.8	694.71	581	758.23	878
EABT3369	13	35	19	165	62	17	13
EABT33690	0	0	8	4	3	0	0
EABT33691	1066.02	1463.99	1022	1654.39	1455.04	1497	1023
EABT33692	2	2	4	2	0	1	1
EABT33693	0	0	1	0	4	0	1
EABT33694	45	97	40	62	67	2	11
EABT33695	156	127	0	0	0	57	73
EABT33696	5829.61	6522.28	3135.19	4504.92	2502.08	2657.86	1316.79
EABT33697	2	2	5	0	1	0	2
EABT33698	0	0	2	0	0	0	0
EABT33699	276	246.05	93.06	33	58	148	122
EABT337	288.72	428.99	490	705.43	240.01	245.05	159
EABT3370	1	2	6	1	0	0	1
EABT33700	208	387.22	530.67	672.04	250.81	174.05	230.89
EABT33701	1	4	31	14	0	9	3
EABT33702	2	2	39	8	0	0	1
EABT33703	11	29	6	15	5	0	0
EABT33704	0	1	6	0	1	0	1
EABT33705	0	0	11	0	1	0	0
EABT33706	0	7	16	3	0	1	1
EABT33707	0	1	3	1	0	2	0
EABT33708	2584.19	4354.58	1776	3683.21	1934.84	1587.79	1589.89
EABT33709	3546.22	3792.35	665	1635	5780.67	2164.93	2325.09
EABT3371	1573.43	3105	4045.08	5871.21	2798	819.05	1434.08
EABT33710	2	9	12	68	6	0	3
EABT33711	1	4	7	6	3	0	0
EABT33712	2	5	6	26	1	20	8
EABT33713	0	0	0	0	0	0	1
EABT33714	34	57	67	126	74	21	27
EABT33715	9	19	74	5	42	5	8
EABT33716	921.08	1121.37	1013.26	1896.41	1587.8	525.93	535.82
EABT33717	2653.85	7406.59	9374.89	11797.7	3057.85	3647.81	5696.94
EABT33718	1	3	0	1	0	2	0
EABT33719	0	0	0	4	1	0	1
EABT3372	31	162	453.37	99	55.67	51.5	19.44
EABT33720	0	2	18	0	2.95	1	1
EABT33721	3	0	11	0	0	1	0
EABT33722	0	5	7	1	0	2	2
EABT33723	6	15	7	0	0	0	0
EABT33724	1	0	10	4	1	0	1
EABT33725	4	13	24	194	19	1	4
EABT33726	1	5	4	0	1	0	0

EABT33727	13.75	13	1	1	1	6	23
EABT33728	0	8	13	11	15	2	2
EABT33729	429.46	739.86	626.35	1308	662.67	135.31	458.96
EABT3373	1	1	8	1	1	0	0
EABT33730	0	3	13	7	2	0	0
EABT33731	0	0	7	0	2	0	2
EABT33732	0	10	0	29	2	0	0
EABT33733	13	19	49.45	41.11	4.01	0	0
EABT33734	1	2	3	5	1	0	0
EABT33735	0	4	1	0	0	2	0
EABT33736	3	2	12	27	1	0	0
EABT33737	12	14	8	4	1	13	1
EABT33738	15	1	1	0	0	4	0
EABT33739	0	0	3	0	0	0	0
EABT3374	0	0	0	0	0	0	0
EABT33740	0	0	0	0	1	0	15
EABT33741	0	0	1	2	1	0	0
EABT33742	5	6	3	0	1668.99	0	5
EABT33743	0	0	1	3	1	0	1
EABT33744	4	2	6	0	1	5	1
EABT33745	1	2	1	0	0	1	0
EABT33746	1	5	15	20	2	1	3
EABT33747	127	444.4	622.41	4573.6	295.46	39	112
EABT33748	3347.35	8684.61	295.25	1126.18	56	7738.03	33
EABT33749	3	5	0	0	0	1	0
EABT3375	8	28	135	42	11.07	5	8
EABT33750	0	1	53	31	0	2	1
EABT33751	0	0	0	0	4	0	0
EABT33752	1	17	3	0	18	9	0
EABT33753	119	138	161	510	259	74	147
EABT33754	7	12	5	65.94	11	1	1
EABT33755	0	0	0	0	3	0	1
EABT33756	47	79	113	7	18	3	28
EABT33757	1522	2955.46	3524.51	2680.61	2494.95	1184.28	2782.86
EABT33758	3	9	5	1	0	0	0
EABT33759	1	1	14	2	0	0	1
EABT3376	0	6	3	0	1	0	1
EABT33760	1	3	13	4	3	0	3
EABT33761	6	7	0	0	0	1	2
EABT33762	1	7	1	3	2	0	5
EABT33763	0	0	9	0	0	0	0
EABT33764	1	1	3	0	3	0	2
EABT33765	136054	76359.1	21058	53622	0	2	0
EABT33766	0	0	0	4	3	0	3
EABT33767	2812.32	2778.64	549	112	855	4958.7	4718.8
EABT33768	0	0	12	1	0	0	0
EABT33769	23	26	34	445	46	7	56
EABT3377	1321.76	1105.35	339	436.89	533.32	1450.47	1176.43
EABT33770	72	9	23	5	0	105	0
EABT33771	1	0	1	2	1	0	0
EABT33772	0	0	8	0	1	0	0

EABT33773	0	0	0	0	3	1	0
EABT33774	8	2	1	0	0	1	0
EABT33775	0	1	8	1	1	0	0
EABT33776	11	22	26	29	12	12	24
EABT33777	0	1	4	1	0	0	0
EABT33778	0	0	1	3	0	0	0
EABT33779	3	2	23	22	1	0	2
EABT3378	1557.93	2108	1484	1726	1310	1282.01	1795
EABT33780	0	1	2	2	2	1	0
EABT33781	107.85	280.63	92.97	84.41	134.89	106.15	21.01
EABT33782	0	0	3	4	0	0	1
EABT33783	0	1	0	9	1	0	0
EABT33784	0	2	4	3	0	0	0
EABT33785	1.98	15.89	19	3	0	9	2
EABT33786	0	0	1	0	8	0	0
EABT33787	2	2	3	0	0	1	0
EABT33788	0	0	3	0	2	0	2
EABT33789	129	154.72	184.06	3591.3	946.02	21	156.89
EABT3379	1	2	7	0	0	0	0
EABT33790	17	60.92	16	73	22	1	3
EABT33791	0	1	12	6	1	1	0
EABT33792	3	1	7	0	0	0	1
EABT33793	1	3	2	3	1	1	2
EABT33794	3	3	8	1	0	0	3
EABT33795	3	4	20	30	9	1	1
EABT33796	0	1	9	2	0	0	1
EABT33797	0	1	7	2	2	0	0
EABT33798	0	2	5	0	0	1	1
EABT33799	2243.97	3222	3391.43	6625.93	2585	2049.92	2127
EABT338	38	1	0	0	0	2	0
EABT3380	3	8	5	13	2	4	4
EABT33800	0	9	0	1	0	0	1
EABT33801	0	0	8	0	0	0	0
EABT33802	2	10	18	26	4	2	6
EABT33803	22	1	5	3	3	2	91
EABT33804	0	0	8	0	0	0	0
EABT33805	1	0	4	0	0	1	0
EABT33806	5	5	3	0	0	2	0
EABT33807	2866.53	4658.82	2270.84	7259.36	2444.01	1679.65	4609.62
EABT33808	0	0	46	0	0	0	0
EABT33809	119.99	148.77	271.66	546.99	381.01	62	86
EABT3381	8	12	2	0	65	4	7
EABT33810	0	1	43	0	0	0	0
EABT33811	3	1	9	3	1	0	0
EABT33812	0	9	18	54	2	2	6
EABT33813	0	4	7	13	1	0	1
EABT33814	852	804.01	55.93	565	175.61	155	347
EABT33815	10	13	15	6	5	4	9
EABT33816	0	2	2	1	3	1	0
EABT33817	30	69.04	950.1	2911.97	185	28	176
EABT33818	3	1	0	0	0	1	0

EABT33819	0	1	6	1	1	0	1
EABT3382	0	2	5	1	1	0	0
EABT33820	1106.15	1259.31	1066.09	2206.67	1796.99	1120.93	1604.03
EABT33821	161.82	11	5	0	0	27.63	0
EABT33822	0	0	14	0	0	1	0
EABT33823	0	1	12	2	1	0	0
EABT33824	3	4	2	0	1	0	0
EABT33825	0	0	11	1	0	0	0
EABT33826	8	6	10	19	6	5	4
EABT33827	2	1	19	9	0	1	0
EABT33828	1	1	15	1	0	0	0
EABT33829	0	2	9	1	0	0	0
EABT3383	2	1	2	0	0	4	1
EABT33830	0	1	7	0	0	0	1
EABT33831	52	59	117	6	35	4	7
EABT33832	2	8	11	3	23	2	6
EABT33833	0	1	20	6	0	0	3
EABT33834	11	24	31	39.01	12	7	13
EABT33835	1	3	0	0	1	0	0
EABT33836	1	2	6	2	0	1	1
EABT33837	0	1	5	1	0	0	0
EABT33838	0	6	88	14	3	1	1
EABT33839	2	2	6	2	1	0	5
EABT3384	0	1	9	0	0	0	0
EABT33840	0	0	4	1	0	1	0
EABT33841	10	11	19	10	4	46	6
EABT33842	58	167	380.95	98	347.05	235.03	129.79
EABT33843	0	1	6	11	0	2	0
EABT33844	13	1	1	92	1	0	0
EABT33845	0	1	11	0	1	0	0
EABT33846	1178	671.01	92.51	53	2	0	9
EABT33847	6674.72	5918.66	3288.04	7582.81	10778.3	4230.04	4969.01
EABT33848	3613.14	5042.96	2263.49	7582.84	2088.71	1706.52	3079.12
EABT33849	0	3	19	2	0	1	7
EABT3385	0	1	6	6	0	1	0
EABT33850	0	0	4	1	0	0	0
EABT33851	1	9	6	14	0	1	0
EABT33852	27	35	183	6	1	8	4
EABT33853	566.01	875.16	588.97	1176	475	365	585.98
EABT33854	256287	104822	503.08	11	79	447498	223.38
EABT33855	835	1300.03	479.98	2184.86	829.12	494	686
EABT33856	204	366.97	757.06	496	475	251.99	387.98
EABT33857	13	9	0	0	1	49	31
EABT33858	267.24	658.68	443	474.05	537.36	183.98	206.4
EABT33859	2	2	7	8	7	0	29
EABT3386	1	4	1	0	2	5	2
EABT33860	2	0	6	2	0	4	1
EABT33861	978.95	1488.03	1212.23	1680	993.97	525	749.96
EABT33862	1056	1284	750.02	2191.08	992	1157	947
EABT33863	8	22	6	14	0	5	5
EABT33864	5	7	16	11	13	22	2

EABT33865	3	2	6	5	5	3	1
EABT33866	1	7	0	1	0	0	0
EABT33867	9	26	22	7	0	0	0
EABT33868	22038.1	8267.51	2286.77	3944.63	5662.02	485.56	625.08
EABT33869	1	2	12	4	2	2	0
EABT3387	2	18	13	34	9	3	8
EABT33870	23228	17358.8	15597.1	14440.4	6168.95	4788.41	4494.95
EABT33871	33	70	94.33	1	3.12	9	5
EABT33872	409	652	625	705.9	485	244	291
EABT33873	0	0	3	4	0	0	0
EABT33874	0	2	226	7	9	35	14
EABT33875	1444.98	2281.18	3297.02	4878.19	1912.67	1261.98	1307.18
EABT33876	0	0	0	0	0	0	16
EABT33877	1	0	2	0	1	0	1
EABT33878	11	2	0	0	0	5	0
EABT33879	29	13	3	0	5	29.96	17
EABT3388	6	12	28	27	4	3	9
EABT33880	702.16	1315.77	1644.31	1678.1	731	547.39	692.05
EABT33881	0	1	6	2	0	0	0
EABT33882	3708.83	4851.77	1542.97	11137.9	7799.63	257	1275
EABT33883	31	236	151	38	19	13	37
EABT33884	17	81	32	25	10	44	31
EABT33885	1	8	9	7	0	0	1
EABT33886	2264.01	600	4	0	1	1	1
EABT33887	2	2	5	7	0	0	0
EABT33888	2	12	7	23	5	4	3
EABT33889	3012943	840030	382968	403926	33	22	0
EABT3389	3288.1	3205.82	1455.39	3728.47	1783.65	2708.63	2003.87
EABT33890	7	12	15	14	6	0	3
EABT33891	2	3	2	2	0	0	0
EABT33892	10	4	12	0	5	1	5
EABT33893	0	3	1	0	0	0	1
EABT33894	10	7	336	12	6	7	5
EABT33895	2803.99	5657.17	5122.41	5141.81	1925.93	2949.82	2903.9
EABT33896	1	0	17	0	1	0	0
EABT33897	2	3	20	1	3	0	0
EABT33898	1	14	3	1	0	0	0
EABT33899	5	5	35	6	1	4	5
EABT339	2861.91	3770.35	7029.94	8866.19	2504	2082.54	1484
EABT3390	0	0	4	0	0	0	0
EABT33900	7	5	2	3	0	0	0
EABT33901	1	0	4	0	0	0	0
EABT33902	5	4	4	2	0	0	1
EABT33903	15	23	25	51	19	35	32
EABT33904	0	2	10	0	0	0	0
EABT33905	10	36	11	20	31	2	26
EABT33906	19	38	80	46	4	7	6
EABT33907	0	0	5	2	0	0	0
EABT33908	0	0	4	2	0	0	0
EABT33909	21	5	11	10	0	7	5
EABT3391	0	0	0	0	0	0	1

EABT33910	718.15	1385.26	1545.58	2168.18	1154.11	771.97	953.52
EABT33911	597.06	966.76	864.59	2301.21	1206.85	727.3	758.58
EABT33912	1	2	2	0	0	0	1
EABT33913	0	0	5	1	0	0	0
EABT33914	0	0	0	0	1	0	6
EABT33915	0	0	3	0	0	0	0
EABT33916	2	2	0	0	0	0	1
EABT33917	0	1	2	9	0	0	0
EABT33918	3	16	65	0	1	9	1
EABT33919	0	2	3	3	1	1	1
EABT3392	0	1	15	0	0	0	1
EABT33920	256.01	682.31	1258.97	651.36	1819	43	213.04
EABT33921	0	0	6	1	0	0	0
EABT33922	0	2	1	3	0	0	1
EABT33923	1034.71	1208.34	565.26	1418.55	1233.4	1054.41	819.4
EABT33924	0	3	9	0	0	0	0
EABT33925	0	0	34	1	0	0	0
EABT33926	4	7	10	12	2	12	8
EABT33927	0	1	10	0	0	0	1
EABT33928	1	4	2	5	1	0	0
EABT33929	2	3	3	5	3	0	3
EABT3393	4	7	19	0	11	0	2
EABT33930	0	0	15	0	1	1	1
EABT33931	2	13	5	15	1	2	1
EABT33932	502.11	644	205	627.57	410	494	493.02
EABT33933	18	5	0	0	0	0	0
EABT33934	0	1	2	0	0	9	0
EABT33935	477.82	229.41	166.05	65	363	212	1952.68
EABT33936	1	8	6	4	117	0	1
EABT33937	13	0	0	0	1	13	0
EABT33938	10	25	101	86.14	9	20	14
EABT33939	0	2	0	0	0	9	0
EABT3394	13	3	1	1	0	5	9
EABT33940	44	56.07	71.01	264	134	5.08	40.17
EABT33941	19	15	64	83.62	106	2	4
EABT33942	1	2	34	27	0	2	1
EABT33943	0	0	36	1	3	0	0
EABT33944	1939.46	2474.58	2914.34	1094.84	2662.14	826.36	1318.86
EABT33945	5	13	5	6	0	1	0
EABT33946	0	1	5	0	0	0	0
EABT33947	96	146.01	100.14	276.21	228.39	48	123
EABT33948	1	7	10	10	6	1	15
EABT33949	0	0	20	0	0	0	0
EABT3395	0	0	13	0	0	0	0
EABT33950	0	0	163.62	0	2	0	3
EABT33951	3	1	64	3	0	0	2
EABT33952	276	444.65	357.02	1982.81	257.14	134.58	134
EABT33953	1	13	24	22	6	3	1
EABT33954	1	3	0	0	3	1	14
EABT33955	1	5	11	8	0	0	1
EABT33956	6	15	30	14	7	9	6

EABT33957	1	3	17	8	34	1	12
EABT33958	5	6	42	22	4	8	3
EABT33959	2	2	5	1	0	0	1
EABT3396	2	0	44	2	0	1	0
EABT33960	0	1	8	2	3	0	2
EABT33961	0	1	17	0	6	0	7
EABT33962	25	41.01	20	55	14	33	56
EABT33963	2	1	2	0	0	2	0
EABT33964	186	291	180	511	219	147	176
EABT33965	1748.3	2835.85	3429.07	9702.51	1935.04	1676	1317.46
EABT33966	6	4	14	0	0	0	0
EABT33967	3.71	1	174	2	3	1	4.09
EABT33968	0	0	9	0	0	3	1
EABT33969	0	0	5	1	1	0	0
EABT3397	4	3	0	7	13	3	23
EABT33970	0	6	5	0	0	3	0
EABT33971	1	0	0	0	0	15	7
EABT33972	12	4	8	3	0	2	0
EABT33973	2	1	10	0	0	2	0
EABT33974	11	72	22	10	16	44	10
EABT33975	369.41	579.51	203.99	1181.98	637.67	307.46	2392.19
EABT33976	1	8	15	2	4	1	4
EABT33977	3	4	1	0	0	3	1
EABT33978	2	2	9.01	0	3	0	6
EABT33979	2	2	38	2	0	1	0
EABT3398	4	8	8	2	0	0	0
EABT33980	0	0	8	1	0	0	0
EABT33981	1	0	36.12	11	0	0	0
EABT33982	1201	1747	1955.95	3363.22	1166.76	1149	1007.26
EABT33983	1774	2060	1590	1631	10385.7	1625	1421.32
EABT33984	240.83	273.03	726.7	823.83	177	7	21
EABT33985	94.74	270.26	218.42	52.75	372.42	15	48
EABT33986	4550.29	18772.5	1789.14	34	1223	3668.62	1479.08
EABT33987	2	15	35	3	0	2	0
EABT33988	29	82	134	30	842	3	17
EABT33989	5	41	6	1	3	36	15
EABT3399	0	2	8	2	1	1	0
EABT33990	6687.7	20435.4	18873.3	6375.69	18664.3	2325.25	6874.64
EABT33991	0	2	4.99	4	2	0	2
EABT33992	0	1	2	0	0	0	2
EABT33993	2	2	2	0	0	0	0
EABT33994	1	12	3	0	0	1.03	0
EABT33995	0	1	8	1	1	0	0
EABT33996	0	0	6	0	0	0	0
EABT33997	41	84	415	122	55	137	9
EABT33998	0	0	5	0	0	1	0
EABT33999	0	9	0	0	0	4	3
EABT34	0	0	6	7	0	0	0
EABT340	6650.49	4476.46	2750.26	4180.57	1840.65	5050.05	4287.62
EABT3400	7	7	8	3	0	2	1
EABT34000	1	11	64	2	0	3	3

EABT34001	0	1	2	5	0	0	0
EABT34002	0	0	4	0	0	0	0
EABT34003	6	21	0	0	0	0	11
EABT34004	3	17	111	1	2	1	3
EABT34005	4	8	1	7	0	0	2
EABT34006	3	4	15	1	1	8	2
EABT34007	4	17	58	5	18	15	5
EABT34008	1750.99	1803.99	940.09	12792.7	661.21	1366	1814.97
EABT34009	5	5	14	11	0	2	0
EABT3401	2	3	7	19	2	0	1
EABT34010	0	0	0	1	0	0	0
EABT34011	1	4	15	8	0	0	3
EABT34012	1	5	11	3	4	0	2
EABT34013	0	0	8	7	0	0	0
EABT34014	10	12	15	20	6	2	3
EABT34015	2523	4247.74	3434.74	6853.89	1495.09	2013	1762.8
EABT34016	6	7	85	459	3	5	4
EABT34017	7095.57	17532	23623.8	13312.5	4476.26	7341.4	7010.99
EABT34018	8	26	63	40	75	0	13
EABT34019	476	1308	417.26	999.99	404	27	415.99
EABT3402	0	0	0	4	0	0	0
EABT34020	344.37	1867.56	1181.88	2919.51	354	414.88	543
EABT34021	62	135	216.02	1137.94	57	14	71
EABT34022	787.47	816.9	298.93	1358.79	525.03	415.94	468.24
EABT34023	1	2	45	6	0	1	1
EABT34024	4	4	5	1	0	2	0
EABT34025	9	2	10	2	11	0	0
EABT34026	0	0	6	0	0	1	0
EABT34027	1	1	4	3	0	2	1
EABT34028	0	2	17	1	0	0	0
EABT34029	0	1	4	0	1	0	0
EABT3403	892	1996	1510.99	1839	1499	465.38	495
EABT34030	0	3	4	0	0	0	0
EABT34031	2	2	2	1	0	0	0
EABT34032	2	1	0	0	0	1	0
EABT34033	0	2	3	2	0	0	0
EABT34034	400	256	318.02	634	244	4	13
EABT34035	2	1	2	1	0	1	0
EABT34036	1	2	1	1	1	0	1
EABT34037	390	1013.99	59	0	47.98	0	14
EABT34038	38	112	267	31.52	28.99	7	12
EABT34039	1	7	11	1	1	0	1
EABT3404	0	0	0	0	0	2	37
EABT34040	9	4	12	3	0	48	6
EABT34041	0	1	14	0	0	0	0
EABT34042	6	74	738	19	53	10.52	11
EABT34043	2062.67	6736.31	5146.83	3116.87	3111.17	15	55
EABT34044	1	0	35	1	0	0	0
EABT34045	789.1	1387.84	2881.79	2328.1	916.87	1013.02	904.01
EABT34046	3	1	6	3	0	0	0
EABT34047	1	2	3	5	0	0	0

EABT34048	3	1	65.99	10	49	2	6
EABT34049	0	0	8	3	0	0	0
EABT3405	1279	1634	2812.03	3140.56	1231.75	1071	1008.11
EABT34050	1	1	2	2	0	1	0
EABT34051	0	0	0	2	0	1	0
EABT34052	4	0	2	2	1	1	0
EABT34053	1796.01	2372.66	1810.8	5436.67	2131	1176	1105.04
EABT34054	1	2.01	6	12	1	2	5
EABT34055	5	10	5	9	4	5	6
EABT34056	2	1	9	0	0	0	1
EABT34057	0	0	2	5	1	0	2
EABT34058	132.46	204	367.01	1075	545.31	57	43
EABT34059	3	5	18	5	0	1	1
EABT3406	1	1	25	5	0	6	0
EABT34060	711.1	1196.99	1555.97	3144.28	1133.98	880.5	862.9
EABT34061	0	1	7	7	0	0	0
EABT34062	4	5	5	7	4	1	3
EABT34063	1606.25	2435.58	1969.43	1390.81	1227.5	1388.07	1327.03
EABT34064	5	0	9	1	2	0	0
EABT34065	9	7	3	4	1	4	2
EABT34066	4	7	47	87	39	2	0
EABT34067	1510.04	2390.21	6034.3	2178.41	2011.43	1327.99	1517.95
EABT34068	5	1	14	11	1	1	0
EABT34069	0	2	2	1	0	0	1
EABT3407	304	60	17	231.49	33	1	17
EABT34070	2	36	2	2	7	0	2
EABT34071	1	1	2	0	0	0	0
EABT34072	0	0	0	4	0	1	0
EABT34073	1	1	5	0	0	4	0
EABT34074	0	1	10	1	1	0	0
EABT34075	1	1	1	1	0	1	0
EABT34076	3	2	15	8	2	9	6
EABT34077	1	3	6	5	1	2	4
EABT34078	0	1	26	1	1	5	1
EABT34079	3	9	27.01	0	19	0	3
EABT3408	2	2	1	0	0	0	0
EABT34080	0	3	9	0	0	0	0
EABT34081	191	424	653	2676.15	449	116	151
EABT34082	4693.27	10706.3	1915.92	375	1659.5	10096.4	14183
EABT34083	228	174	48	674	327	23	33
EABT34084	0	0	17	1	2	1	0
EABT34085	3	1	3	2	13	3	9
EABT34086	1	4	4	1	1	4	3
EABT34087	759.06	1366.99	1121.25	2775.4	591.13	459.19	548.01
EABT34088	0	0	2	0	1	0	3
EABT34089	9586.62	14537.1	11714.3	2445.74	4268.26	257.18	2039.42
EABT3409	1	7	39	3	5	0	2
EABT34090	2	8	12	3	3	1	1
EABT34091	0	5	27	4	13	2	1
EABT34092	1	0	3	1	0	0	0
EABT34093	54	33	70	0	21	18	47

EABT34094	550.72	1358.91	873	22387.5	1092	99	394
EABT34095	2	6	17	9	2	0	1
EABT34096	6	4	0	0	0	0	0
EABT34097	62	111.18	81	35	69	90	68
EABT34098	13	28	3	2	8	0	8
EABT34099	0	0	0	1	0	0	3
EABT341	6	11	0	0	0	2	11
EABT3410	0	3	5	3	1	0	0
EABT34100	1	0	4	0	1	0	0
EABT34101	0	2	1	5	1	0	0
EABT34102	3	33	102	127	4	0	0
EABT34103	14	34	33	29	13	43	12
EABT34104	0	4	9	3	4	3	1
EABT34105	1851.75	3668.64	1920.13	4757.79	1751.34	1674.16	1796.41
EABT34106	1	3	1	2	0	7	0
EABT34107	9	28	36	2	20	18	30
EABT34108	4	16	0	13	12	2	8
EABT34109	1902.48	2689.01	4021	4355.04	1318.16	2735	1463.32
EABT3411	0	5	0	0	0	1	0
EABT34110	0	0	3	1	2	1	2
EABT34111	0	0	0	0	0	4	15
EABT34112	8	2	7	3	1	3	1
EABT34113	1	2	1	0	0	0	7
EABT34114	4	13	4	31	5.52	1.06	1
EABT34115	0	3	10	1	1	0	0
EABT34116	19	31	24	4	0	3	0
EABT34117	1	1	1	1	0	0	1
EABT34118	1	3	2	5	1	0	0
EABT34119	1	2	0	0	0	2	4
EABT3412	0	1	26	1	1	1	0
EABT34120	0	0	2	9	2	0	0
EABT34121	2	0	6	20	5	0	5
EABT34122	4	3	15	0	0	0	0
EABT34123	0	0	4	0	0	1	0
EABT34124	0	0	1	0	0	0	0
EABT34125	14.13	26	41	11.12	8	25	60
EABT34126	1	3	11	2	1	0	4
EABT34127	0	0	0	0	1	14	6
EABT34128	2	3	1	1	0	1	0
EABT34129	8	9	32	2	1	0	2
EABT3413	558.43	975.46	1032.05	2575.69	797.43	575	748.74
EABT34130	6	6.99	8.07	29	20	13	24
EABT34131	2	6	9	6	1	11	2
EABT34132	1635.27	3782.22	6858.67	7872.37	2558.6	2017.82	2133.13
EABT34133	1	0	4	1	0	6	0
EABT34134	338.01	633.1	680.06	1436	506	57	141
EABT34135	1	2	7	0	0	1	2
EABT34136	6	13	16	19	5	7	9
EABT34137	62	108	103	202.91	81	1	6
EABT34138	5	2	5	1	0	0	2
EABT34139	28	36	0	0	1	1	1

EABT3414	0	0	5	1	0	0	0
EABT34140	0	0	3	0	0	0	0
EABT34141	3	3	9.99	2	2	2	2
EABT34142	0	0	6	1	2	0	0
EABT34143	0	0	3	0	0	0	1
EABT34144	3	5	0	0	0	22	0
EABT34145	3	77	3	92	10	7	5
EABT34146	0	1	0	16	1	0	0
EABT34147	0	7	6	5	6	0	5.5
EABT34148	777.18	1048.4	879.7	1442.49	845.96	384.04	481.75
EABT34149	0	2	19	0	0	0	0
EABT3415	32	18	0	1	6	40	27
EABT34150	1	1	1	8	5	0	0
EABT34151	3	1	12	9	2	0	0
EABT34152	0	1	17	3	0	0	0
EABT34153	4	7	4	19	118	1	1
EABT34154	12	65	455	5594.55	144.02	2	13
EABT34155	0	1	3	2	1	0	0
EABT34156	0	2	15	1	1	0	1
EABT34157	2	0	3	8	0	2	2
EABT34158	4	5	4	5	1	4	2
EABT34159	2	2	1	2	0	0	0
EABT3416	0	0	3	0	0	0	3
EABT34160	0	2	0	0	0	9	1
EABT34161	1	2	6	1	0	0	0
EABT34162	4	2	11	1	1	0	0
EABT34163	675.92	1245.82	997.5	1824.76	1058	855.57	634.37
EABT34164	0	1	0	0	0	0	0
EABT34165	0	1	4	0	0	0	2
EABT34166	1	2	98.99	8	3	2	1
EABT34167	0	3	0	0	1	0	7
EABT34168	0	2	5	3	1	0	1
EABT34169	0	1	1	0	0	0	0
EABT3417	8482.71	28907.7	23316.1	34611.5	14836.9	11796.1	13919.2
EABT34170	0	0	4	5	0	0	0
EABT34171	289	520	257.98	771	248.68	88	183
EABT34172	1	2	3	1	0	0	1
EABT34173	1087.99	1568.83	851.95	2156.72	1031.57	427.18	550
EABT34174	4	4	38	1	0	1	1
EABT34175	1	1	8	0	0	0	1
EABT34176	4525.22	6547.19	5578.48	9003.1	4462.86	4125.94	3933.95
EABT34177	1	13	91	27	82	2	3
EABT34178	0	1	2	5	2	0	2
EABT34179	15	25	15	42	15	11	16
EABT3418	30	127	136.3	2066.92	211.13	46.98	120
EABT34180	3	3	0	1	1	0	0
EABT34181	0	0	0	41	0	0	0
EABT34182	5	6	5	916.52	4	5	2
EABT34183	0	0	17	0	0	1	0
EABT34184	37	52	0	0	1	9	15
EABT34185	55	15536.4	2041.26	2606.17	519.58	30	3717.37

EABT34186	3094.73	3308.15	1497.69	2034.73	823	3252.91	1585
EABT34187	0	0	0	2	1	0	3
EABT34188	1612.37	2125.01	1200.18	2257.4	1390.92	798.78	1116.08
EABT34189	8	0	0	0	0	0	1
EABT3419	984	1014	705	895	673.09	543.98	453.27
EABT34190	0	0	5	1	0	0	0
EABT34191	3	17	0	0	0	12	7
EABT34192	0	0	20	2	0	0	0
EABT34193	12	8	6	7	4	1	3
EABT34194	3	3	7	15	5	2	3
EABT34195	2	33	200.05	35	5	1	1
EABT34196	13	58	117	152	129	11	28
EABT34197	2	2	11	3	0	1	0
EABT34198	3	2	4	22	0	1	1
EABT34199	0	0	1	3	0	0	1
EABT342	0	0	14	3	0	0	0
EABT3420	0	1	2	8	8	0	0
EABT34200	2515.12	3719.21	1088.56	3440.01	2846.9	515.36	480.58
EABT34201	2	0	0	4	0	1	0
EABT34202	2696	2230.19	566	840	2872	1462	1072
EABT34203	1745.7	2005.69	1082.82	2478.08	1286.74	1680.52	1440.31
EABT34204	0	0	0	0	0	0	16
EABT34205	0	0	9	0	0	0	2
EABT34206	722	904	386	48	322.09	632	1261.98
EABT34207	5	3	5	8	1	3	1
EABT34208	0	1	3	1	0	0	0
EABT34209	0	4	3	1	1	0	4
EABT3421	4	3	1	1	4	0	3
EABT34210	0	0	8	0	0	0	2
EABT34211	572	634.03	546.99	930	756	352	435.15
EABT34212	0	0	38	1	0	0	0
EABT34213	2	10	5	19	24	2	5
EABT34214	12	14	5	13	1	4.02	10
EABT34215	16.99	15	3	2	3	50	7
EABT34216	0	1	2	0	2	0	1
EABT34217	1	1	4	0	0	0	0
EABT34218	2	0	29	0	2	0	1
EABT34219	2	4	25.01	5	1	2	2
EABT3422	0	1	4	1	0	1	1
EABT34220	7	7	13	3	4	0	1
EABT34221	1	1	21	1	1	0	0
EABT34222	0	1	1	9	1	0	0
EABT34223	31	56	72.65	110.39	48	9	45
EABT34224	0	0	0	0	2	0	0
EABT34225	1	1	7	1	0	0	0
EABT34226	157.79	257.14	178.84	411.76	395.14	165.13	195.34
EABT34227	7	14	23	0	17	8	112
EABT34228	0	2	0	5	0	0	1
EABT34229	567	663.02	477	1996	415	386.88	424.9
EABT3423	1	2	21	12	1	0	1
EABT34230	0	0	15	1	0	2	1

EABT34231	0	0	0	0	0	0	0
EABT34232	376.92	584.35	169	1396.53	855.28	88	134.99
EABT34233	0	0	4	0	0	0	0
EABT34234	0	0	5	0	0	0	0
EABT34235	22	30	0	0	1	1	0
EABT34236	0	1	5	1	3	0	3
EABT34237	0	0	0	1	0	0	1
EABT34238	2	3	1	2	0	0	0
EABT34239	2	4	29	15	0	1	0
EABT3424	83	92	177	2066	554	3	12
EABT34240	1	1	0	0	0	0	36
EABT34241	4	5	22	3	0	0	2
EABT34242	361.27	423.79	357.08	405.94	320.25	199.69	242.48
EABT34243	1	1	15	12	6	0	4
EABT34244	4	4	5	0	0	1	1
EABT34245	0	3	1	8	2	0	0
EABT34246	0	3	11	28	3	4	0
EABT34247	719.18	1091	717.01	1275	735.92	1046.88	754
EABT34248	1	1	18	2	1	0	0
EABT34249	0	2	12	1	4	1	1
EABT3425	7	7	25	0	0	15	7
EABT34250	0	0	1	5	8	1	0
EABT34251	23	49	32.97	78.99	82.11	19.4	28
EABT34252	27	56	35	167	78	19.76	34
EABT34253	27	24	187	5809.81	23	14	4
EABT34254	4	3	13	2	1	17	5
EABT34255	21	52	57.04	61	7	16	33
EABT34256	9075.37	24095.3	21978.7	30936.5	7839.7	1655	3438.28
EABT34257	1	9	1	1	0	0	1
EABT34258	1603343	218297	22958	968.07	0	12	0
EABT34259	1	3	7	4	0	0	4
EABT3426	11	19	25	4	5402.48	3	22
EABT34260	1	0	6	1	0	0	0
EABT34261	1	0	5	0	0	0	1
EABT34262	0	4	3	0	1	0	1
EABT34263	1075.03	2677.65	2249.34	2278.79	2748.73	941.18	1124.78
EABT34264	2	2	2	0	1	0	3
EABT34265	0	0	5	0	1	1	1
EABT34266	9	2	4	1	1	2	6
EABT34267	0	2	14	11	0	0	0
EABT34268	0	2	4	2	1	0	3
EABT34269	8	16	0	0	0	0	0
EABT3427	0	2	16	1	0	1	0
EABT34270	209	251.98	133	412	260	91	120
EABT34271	15	11.01	4	5	2	7	7
EABT34272	29	13	3	3	2	0	0
EABT34273	0	0	5	1	0	0	1
EABT34274	0	1	4	0	1	0	0
EABT34275	171649	458240	502358	79753.9	397189	11072.2	73871.5
EABT34276	13	10	2	1	0	21	0
EABT34277	0	0	1	5	3	0	0

EABT34278	34.99	11	11	310.73	366	1	23
EABT34279	0	0	12	0	1	0	0
EABT3428	1	4	35	4	4	2	2
EABT34280	1	1	3	0	2	0	0
EABT34281	10	3	43	10	4	19	16
EABT34282	2	3	21	1	0	2	0
EABT34283	0	6	7	1	1	2	0
EABT34284	2	1	1	2	0	0	1
EABT34285	0	4	21	0	0	1	5
EABT34286	2	13	17	24	0	0	2
EABT34287	1	6	5	14	4	0	3
EABT34288	107	233	219	286	111	214	33.01
EABT34289	0	0	0	47	0	0	0
EABT3429	0	1	19	1	0	0	0
EABT34290	2	4	8	1	0	0	0
EABT34291	114.37	200	75	366.83	165.83	74.53	97.99
EABT34292	5011.54	15204.6	4884.37	4679.04	3487.72	2991.68	1974.22
EABT34293	0	0	4	0	2	0	0
EABT34294	3	2	0	7	0	0	0
EABT34295	6817.43	14988.8	9990.21	5719.05	7462.12	5890.86	6719.6
EABT34296	0	0	6	0	0	0	1
EABT34297	7	31	40.96	52	15	6	39
EABT34298	0	1	10	0	0	0	0
EABT34299	17	182	74	208	139	0	13
EABT343	1	2	7	0	0	0	0
EABT3430	5	11	87	136	23	8	5
EABT34300	22	2	11	10	4	2	5
EABT34301	3	3	39	4	1	5	2
EABT34302	280.81	1113.04	2695.05	829	1787.1	42	423
EABT34303	0	2	4	3	73	0	0
EABT34304	0	2	1	1	0	0	0
EABT34305	14	50	32	140	27	6	13
EABT34306	3	6	5	3	50	6	21
EABT34307	16979	20990	270	10	37	0	4
EABT34308	2592.24	3156.7	2116.95	3033.71	1744.82	1793.45	1451.83
EABT34309	572.6	619.01	327	411.89	309.27	164	68
EABT3431	4447.96	4537	451	461.28	899.19	4710.98	4594.03
EABT34310	972.28	2121.29	1715	4119.38	742.6	449.76	616.57
EABT34311	0	1	10	4	0	0	0
EABT34312	6	10	4	5	2	2	7
EABT34313	0	1	2	4	0	0	1
EABT34314	1	0	1	8	208	0	5
EABT34315	1	1	0	0	0	1	4
EABT34316	0	4	5	6	4	5	0
EABT34317	0	1	7	1	1	0	0
EABT34318	0	0	0	0	1	0	0
EABT34319	1	1	2	3	2	0	0
EABT3432	1	4	3	1	3	0	0
EABT34320	617	347	23	16	1617.57	696	381
EABT34321	1112.1	2213.67	396.12	1339	1849.04	2809.17	2573.09
EABT34322	0	0	0	9	0	0	0

EABT34323	0	5	4	6	3	1	1
EABT34324	0	28	2	1	0	0	0
EABT34325	0	0	25	1	1	1	0
EABT34326	2	0	0	1	5	0	0
EABT34327	0	1	5	6	0	0	1
EABT34328	0	2	17	5	1	0	0
EABT34329	1	4	2	0	0	0	0
EABT3433	2	1	2	27	1	2	0
EABT34330	0	2	3	3	2	0	4
EABT34331	2	3	2	0	0	0	1
EABT34332	803	1268.99	1476.01	2594.75	1011	895	974.2
EABT34333	25	21	0	0	0	2	1
EABT34334	8	5	21	4	0	0	1
EABT34335	0	0	7	2	1	0	0
EABT34336	727.61	1290.27	1438.85	3433.79	972.94	546	792.91
EABT34337	0	3	11	0	0	0	0
EABT34338	6	10	22	7	1	5	4
EABT34339	510.87	625.98	397.02	806.73	463.37	387.04	369.96
EABT3434	0	0	12	1	0	1	0
EABT34340	3	15	17	24	3	4	3
EABT34341	0	0	9	1	0	0	0
EABT34342	4	29	48	75	8	8	3
EABT34343	7412.04	10156.2	5027.8	10465.5	7046.99	9870.13	6848.5
EABT34344	0	2	19	0	0	1	0
EABT34345	15	12	60	21	0	14	1
EABT34346	4011.67	3469.17	2492.91	5268.07	1849.04	570	331.99
EABT34347	5	12	25	6	5	2	2
EABT34348	0	1	0	4	1	0	0
EABT34349	0	4	0	6	0	0	0
EABT3435	525.99	630.47	286.08	917.18	514.98	325.31	392.18
EABT34350	3	4	0	0	0	0	0
EABT34351	131	168	151.98	275	240	73	110
EABT34352	9	13	6	12	3	3	8
EABT34353	0	1	5	0	3	0	0
EABT34354	52	78	191	52	116	47	69
EABT34355	12	4	40	54	28	10	61
EABT34356	1	1	2	30	6	0	1
EABT34357	0	4	18	0	1	0	1
EABT34358	1	3	6	2	0	0	1
EABT34359	8	16	20.01	20	6	2	13
EABT3436	11	27	11	21	2	45	19
EABT34360	3	3	6	8	3	0	1
EABT34361	5473.34	11306.2	13162	1877.62	16267.5	1081.2	3242.22
EABT34362	0	0	9	1	0	0	0
EABT34363	1	1	2	6	0	2	1
EABT34364	36	29	24	12	15	19	28
EABT34365	47	121	41	48	35	108	115
EABT34366	5795.3	9473.17	9229.73	17746.1	3441	4815.72	4395.38
EABT34367	15	19	27	29	5	13	19
EABT34368	0	11	6	29	3	0	0
EABT34369	3	1	1	0	0	1	0

EABT3437	731.04	1159.01	777.31	1397.2	895	621.72	764.12
EABT34370	67	519.06	607.52	526	905.33	9	129
EABT34371	2	3	10	18	2.97	0	3
EABT34372	0	2	2	0	1	0	0
EABT34373	0	2	4	2	0	0	0
EABT34374	7	2	8	7	0	0	0
EABT34375	9	12	0	0	0	0	1
EABT34376	428	524.3	227.01	955.01	533.33	319	374.96
EABT34377	46	85	52	79	39	35	39
EABT34378	22	44.03	78	45	16	11	25
EABT34379	0	1	7	0	0	0	0
EABT3438	0	2	0	11	14832.1	3	56
EABT34380	0	1	0	4	0	0	1
EABT34381	0	4	8	0	0	0	0
EABT34382	0	0	10	1	0	0	0
EABT34383	2120.72	3282.73	2179.29	7181.96	1924.43	1569.72	2224.72
EABT34384	0	1	3	0	0	2	0
EABT34385	1119.59	2293.4	3266.6	6392.59	1994.21	706.44	1205.7
EABT34386	3	1	2	2	1	1	3
EABT34387	435.45	747.18	743.23	2201.92	770	310	365
EABT34388	10	17	100	27	7	0	1
EABT34389	0	0	1	4	1	0	0
EABT3439	330.02	526.44	1298.52	1585.91	843.01	74.49	103
EABT34390	193	293	156	672.26	651.57	123	248
EABT34391	53	76	0	0	0	1	0
EABT34392	2	5	8	8	3	27	3
EABT34393	0	2	7	0	0	0	0
EABT34394	228.92	306	550.03	1256.68	367	181	153
EABT34395	955.06	1382	674	3108.1	1178	1000	1103.02
EABT34396	2	1	2	1	0	0	0
EABT34397	4318.15	11422.7	10676.7	41777.7	4113.34	6091	4570.14
EABT34398	219.96	587.63	967.69	1915.75	233.91	13	68
EABT34399	43	59	8	1	0	19	2
EABT344	9	6	19	95	24	5	3
EABT3440	3	0	71	0	0	0	1
EABT34400	0	0	9	3	0	3	2
EABT34401	2967.2	3877.25	3912.06	3454.83	2368.8	2479.37	2192.48
EABT34402	0	0	10	0	0	0	0
EABT34403	0	1	7	24	0	12	1
EABT34404	7	9	8	15	6	5	6
EABT34405	0	1	6	0	0	0	0
EABT34406	3	0	1	6	3	0	0
EABT34407	1	2	2	2	0	0	0
EABT34408	2	0	3	7	4	1	1
EABT34409	3	0	13	2	2	2	1
EABT3441	10	11	50	98	20	11	23
EABT34410	0	4	35	11	87.24	3.97	0
EABT34411	15	28	12	18	2	15	9
EABT34412	3	9	11	8	6	0	6
EABT34413	3	5	5	45	3	0	1
EABT34414	2	1	6	2	0	1	2

EABT34415	0	0	0	0	12	0	0
EABT34416	0	0	10	0	1	0	0
EABT34417	510	2515.51	1650	392	4552.43	1	17
EABT34418	1	2	1	8	2	0	0
EABT34419	9	6	55	25	0	3	0
EABT3442	7	8	42	36	1	3	2
EABT34420	3	8	3	17	1	3.03	4
EABT34421	32	17	21	17	6	3	20
EABT34422	0	0	3	1	3	1	0
EABT34423	2515.61	4247.6	7884.2	459.86	2361.74	1311.81	2918.31
EABT34424	0	1	10	4	1	0	0
EABT34425	174	369.1	518	2187.17	362.08	115	59
EABT34426	5	11	25	19	3	4.14	3
EABT34427	0	2	10	4	1	1	0
EABT34428	0	0	0	0	1	0	0
EABT34429	16	17	0	92	2	0	0
EABT3443	2650	4235.66	2608.11	1217	1330	4086.03	5647
EABT34430	0	0	20	2	0	1	1
EABT34431	0	1	1	0	3	0	0
EABT34432	0	0	15.89	0	0	0	0
EABT34433	781.56	1308.38	878.2	1375.36	900.24	744.27	643.75
EABT34434	0	1	2	6	1	0	1
EABT34435	0	0	4	1	0	0	0
EABT34436	16	10.06	0	0	1	8	13
EABT34437	2	2	26	4	0	1	1
EABT34438	570.49	83	87.42	1290.93	511.13	1	93.49
EABT34439	0	0	2	3	1	0	0
EABT3444	1	2	1	1	2	1	0
EABT34440	3	3	7	0	0	0	1
EABT34441	1	2	24.41	1	0	0	0
EABT34442	0	0	0	2	1	0	0
EABT34443	7	79	420	1609.07	4066.98	10	22
EABT34444	1926.94	690	9	2	372.98	290	743.9
EABT34445	1	1	27	2	0	0	1
EABT34446	1	0	2	1	0	0	3
EABT34447	0	0	8	0	0	0	0
EABT34448	0	4	8	3	0	0	0
EABT34449	0	2	21	8	0	3	1
EABT3445	2	0	0	0	1	1	1
EABT34450	1	2	41	3	0	0	0
EABT34451	0	4	18	1	0	5	0
EABT34452	0	1	10	0	0	2	0
EABT34453	1	1	8	0	0	0	1
EABT34454	5	13	16	27	3	1.52	0
EABT34455	0	11	20	10	1	0	0
EABT34456	2	3	10	24	7	0	1
EABT34457	6291.24	10054.7	7566.09	9674.96	7162.34	6109.05	7607.15
EABT34458	1660.08	4883.86	213	666	301.22	14	120
EABT34459	58	133	153	1135.67	889	25	8.85
EABT3446	0	0	0	0	0	0	0
EABT34460	0	5	15	5	3	1	1

EABT34461	2	1	8	2	0	1	0
EABT34462	2	0	8	2	0	0	0
EABT34463	0	1	3	2	0	1	0
EABT34464	27287.7	29101.5	17552.6	26897	23313.4	37537.1	26629.4
EABT34465	13	9	3	0	1	4	0
EABT34466	4	5	4	1	0	0	0
EABT34467	0	1	7	4	8	0	7
EABT34468	5	2	1	0	0	18	1
EABT34469	0	0	0	0	0	14	1
EABT3447	0	1	25	2	1	0	0
EABT34470	0	0	4	0	4	1	0
EABT34471	1	0	0	0	0	2	0
EABT34472	0	0	1	8	1	1	1
EABT34473	0	0	0	0	1	0	0
EABT34474	0	2	10	0	1	0	0
EABT34475	0	1	7	4	0	0	0
EABT34476	10	7	0	0	0	0	0
EABT34477	0	2	25	0	0	0	0
EABT34478	94	141	57	481	162	0	2
EABT34479	1672.66	1894.88	3065.61	2649.49	1393	1302.53	1484.47
EABT3448	0	0	5	0	0	0	0
EABT34480	1.41	6	3	4	0	0	1
EABT34481	1608.22	2172	1548	3079.01	1651.18	1184.31	1029.01
EABT34482	5	8	16	35	13	2	8
EABT34483	0	2	13	2	0	1	0
EABT34484	119	342	1635.07	3782	376	51	162
EABT34485	456.94	900.48	488.6	1440.22	883.83	377.99	914.42
EABT34486	1	3	9	10	2	1	2
EABT34487	11857.5	9857.3	5255.77	8912.4	8192.87	12924.2	9328.18
EABT34488	0	2	6	0	0	1	2
EABT34489	0	1	5	6	0	0	0
EABT3449	0	0	0	0	0	0	0
EABT34490	0	0	47.13	2	0	0	0
EABT34491	0	3	7	3	1	0	1
EABT34492	2	3	1	3	0	0	0
EABT34493	0	0	1	0	2	0	0
EABT34494	0	0	8	7	4	1	0
EABT34495	9	19	15	19	1	0	1
EABT34496	8	28	8	1	2	27	7
EABT34497	1	2	6	4	3	2	8
EABT34498	664.45	1344.69	1392.83	2068.58	726.68	799.64	608.95
EABT34499	0	3	9	0	2	3	3
EABT345	0	7	2	10	24	0	3
EABT3450	4	0	0	0	2	7	8
EABT34500	13	2	2	0	0	0	1
EABT34501	10	23	33	42	9	21	21.39
EABT34502	0	3	1	12	1	0	0
EABT34503	1	8	167.01	77.06	769.05	1	0
EABT34504	1	9	31	4	5	0	3
EABT34505	1	3	10	4	0	0	0
EABT34506	2	2	2	3	1	3	3.02

EABT34507	13	30	6	13	2	0	0
EABT34508	2	3	0	2	0	1	4
EABT34509	4	13	9	4	2	0	0
EABT3451	1	2	18	3	0	0	0
EABT34510	2531.24	4650.43	2924.85	3548.88	1945.09	1839.93	2100.81
EABT34511	0	1	19	0	0	0	0
EABT34512	1	3	10	6	15	0	0
EABT34513	1	0	1	2	3	0	2
EABT34514	3	1	6	6	1	6	2
EABT34515	11	14	16	517.28	9	36	31
EABT34516	2	8	8	12	90	4	0
EABT34517	41058.1	61211.1	38138.3	40221.9	20342.5	26540.9	26867.7
EABT34518	1	8	23	36	8	2	6
EABT34519	1988.13	3300.05	2068.87	3741.98	2058.99	3124.98	4009.27
EABT3452	1	1	2	4	4	1	0
EABT34520	1283.23	2237.85	1280.96	2034.57	1651.33	1003.99	1406.61
EABT34521	38	105	64	117	22	19	16
EABT34522	1226.52	1450.4	1403.92	939.87	933.99	865.01	766.9
EABT34523	5	18	423	4	1	0	4
EABT34524	7	11	34	2	2	18	10
EABT34525	1	1	3	3	0	0	2
EABT34526	1078	1977.42	4259.19	28115.6	1043.55	300	459.91
EABT34527	44	326.98	102.65	510.21	97	238	150
EABT34528	5	3	3	0	3	0	0
EABT34529	0	3	6	4	4	0	0
EABT3453	2	5	12	77	6	0	7.72
EABT34530	0	0	1	0	0	0	0
EABT34531	1494.23	2801.5	6291.91	10085	4781.87	159	154
EABT34532	0	0	6	0	0	0	0
EABT34533	787.75	1155.99	844	1697	441.86	718	814
EABT34534	98.01	38	0	0	0	15	0
EABT34535	1	2	3	0	0	0	0
EABT34536	0	1	4	0	0	2	0
EABT34537	1	5	23	19	8	0	1
EABT34538	1	0	5	1	3	0	2
EABT34539	0	9.1	25.01	4945.36	476	3	2024.75
EABT3454	8.03	7	45	51	3	5	2
EABT34540	216.84	760.2	403.13	719.24	220.96	257.29	255.01
EABT34541	0	3	25	0	3	1	0
EABT34542	2	1	25	7	0	0	1
EABT34543	1	0	67	1	0	1	0
EABT34544	5	7	18	14	4	4	1
EABT34545	2	2	5	6	3	1	2
EABT34546	99.53	359.72	357	536.98	474.04	83	151.24
EABT34547	0	0	8	3	0	0	0
EABT34548	1	9	45	34	0	0	1
EABT34549	81	174	4922.89	1341	1415	33	439
EABT3455	4	6	6	4.99	4	3	1
EABT34550	0	1	3	0	1	0	0
EABT34551	8	1	0	0	0	0	0
EABT34552	141	252	134	643.98	405	99	147

EABT34553	287	320	331.18	301	186	498.83	967.25
EABT34554	2	1	7	1	0	2	0
EABT34555	2	12	28	11	1	26	4
EABT34556	6	18	18	8	3	3	10
EABT34557	8	5	33.14	18.95	6	7	3
EABT34558	1	3	18	3	1	3	1
EABT34559	0	6	41	15	1	0	0
EABT3456	0	1	37	0	1	0	0
EABT34560	20	26	31	70.31	14	3	15
EABT34561	1	2	12	5	1	0	0
EABT34562	2	2	2	2	4	0	0
EABT34563	2	12	3	60	0	2	1
EABT34564	1	2	9	1	2	0	0
EABT34565	1	0	1	0	1	1	0
EABT34566	0	0	1	2	2	0	0
EABT34567	4	11	101.99	674.64	4	2	6
EABT34568	4	5	21	9	4	5	4
EABT34569	0	0	4	0	0	0	0
EABT3457	1	0	9	0	0	0	0
EABT34570	1	0	22	3	0	0	1
EABT34571	2	4	1	1	0	6	8
EABT34572	0	0	9	1	0	1	1
EABT34573	5	2	3	0	0	8	4
EABT34574	1492.8	2166.57	1363.31	7266.48	1399	1363.78	1780.06
EABT34575	1	1	1	1	1	0	0
EABT34576	0	2	18	15	0	0	1
EABT34577	0	8	17	39	4	0	4
EABT34578	4013.92	13640.6	18944.3	19598.3	6914.34	2839.06	2468.73
EABT34579	0	0	7	2	0	1	0
EABT3458	0	1	1	4	0	1	0
EABT34580	294.65	461.01	271.04	541	411.89	208.01	279
EABT34581	73	200	71	161	165	7	7
EABT34582	0	2	5	0	1	0	2
EABT34583	2157.34	5407.8	6522.17	2801.69	5495.83	721.85	514
EABT34584	4	13	1	1	6	1	3
EABT34585	0	0	6	0	0	0	1
EABT34586	0	0	0	0	0	0	0
EABT34587	8	10	8	18	5	3	9
EABT34588	0	0	2	6	0	0	0
EABT34589	1560.65	1277.02	412.99	201.06	651.1	1201.2	3479.99
EABT3459	4	8	0	0	0	0	0
EABT34590	29	39	0	0	2	20	24
EABT34591	1	0	8	0	0	0	0
EABT34592	742	1275.22	1962	1980.08	1007.01	1162.98	905.87
EABT34593	10302.9	13899.9	10603.8	16639.7	9236.8	8407.96	6954.07
EABT34594	4	3	49	2	1	9	1
EABT34595	1	1	2	0	2	1	2
EABT34596	0	0	3	4	0	1	0
EABT34597	1	0	14	8	6	0	2
EABT34598	0	0	7	2	0	0	2
EABT34599	0	0	1	5	0	0	0

EABT346	24	23	15	22	2	34	12
EABT3460	0	2	16	1	1	0	2
EABT34600	6	2	2	3	8	0	12
EABT34601	0	1	7	0	0	0	0
EABT34602	0	0	3	3	0	0	0
EABT34603	1	2	11	7	0	3	0
EABT34604	12309.1	22339.2	14101.6	15702.9	5575.68	10953	6673.52
EABT34605	461	737.99	739	823.89	478.93	304	317.53
EABT34606	0	0	5	0	0	0	2
EABT34607	3	5	3	2	2	0	2
EABT34608	0	2	10	1	8	4	4
EABT34609	1111.93	1153.73	548	1385	707	443	599
EABT3461	20	25	53	25	8	18	13
EABT34610	7	0	3	0	0	1	0
EABT34611	2	2	2	261	34	4	4
EABT34612	0	0	8	0	0	0	1
EABT34613	1	5	113	8	4	0	0
EABT34614	24	15	10	0	1	6	5
EABT34615	1	0	0	9	1	0	0
EABT34616	0	0	4	1	1	0	0
EABT34617	1	8	11	3	0	6	0
EABT34618	3	7	9	17	8	0	0
EABT34619	47	318.64	199.69	221	253.48	13	39.76
EABT3462	1	1	2	46	0	0	0
EABT34620	4	17	20	5	5	2	4.25
EABT34621	345.8	525.69	494.11	2260.82	509.01	318.07	263.98
EABT34622	844.3	1433.87	559.02	1484.53	850.54	699.58	580.37
EABT34623	2	2	0	0	1	1	2
EABT34624	1	1	2	5	1	1	2
EABT34625	0	2	9	3	1	0	0
EABT34626	29346	35718	34313.3	41831.2	15605.9	19063.7	17519
EABT34627	0	0	1	0	0	0	7
EABT34628	2	5	5	23	4	3	3
EABT34629	3713.95	8853.62	3061.97	9542.57	2055.08	2746.05	2609.96
EABT3463	4	14	22	84	4	2	1
EABT34630	11	16.14	130	56	2	2	2
EABT34631	3	2	16	2	0	0	1
EABT34632	1	9	8	10	3	3	1
EABT34633	1	0	14	1	0	0	0
EABT34634	0	0	3	6	0	1	0
EABT34635	3	5	3	7	1	2	3
EABT34636	1	10	12	5	2	2	0
EABT34637	0	13	17	43	169	1	9
EABT34638	3	5	2	0	0	0	1
EABT34639	0	0	1	0	1	1	0
EABT3464	0	1	25	1	0	0	0
EABT34640	2625.64	4816.59	2168.08	1552.75	1754.24	607.28	1146.65
EABT34641	26	5	0	0	0	0	0
EABT34642	0	0	6	0	0	0	1
EABT34643	4	1	18	15	1	7	6
EABT34644	3	9	10	29	0	3	5

EABT34645	704	948.12	847	2221	762	554	441
EABT34646	0	2	4	4	0	0	5
EABT34647	751.96	789.43	387.98	1108.45	625.02	696.25	750.5
EABT34648	0	0	0	5	0	0	0
EABT34649	0	4	16	13	1	3	0
EABT3465	950	1566	1507.34	1804.09	933	903.03	834.78
EABT34650	0	0	4	0	0	0	0
EABT34651	3	2	2	0	1	1	0
EABT34652	5	7	42	1	2	19	8.01
EABT34653	1961.86	3399.63	3018	2406	1848.05	2141	1334.01
EABT34654	0	0	7	1	0	1	0
EABT34655	0	1	24	1	0	2	0
EABT34656	1	1	1	13	0	0	0
EABT34657	3	3	18	7	0	1	0
EABT34658	118.95	19	276.11	64	2370.64	10	55
EABT34659	1	0	0	1	2	1	6
EABT3466	1811.53	2386.11	2473	4512.15	1077.77	1695.92	1671.97
EABT34660	0	5	12	1	0	0	1
EABT34661	6	13	15	13	8	4	11
EABT34662	7	13	7	2	1	20	6
EABT34663	0	3	6	3	0	1	0
EABT34664	0	1	4	1	0	0	0
EABT34665	0	0	0	3	4	0	3
EABT34666	4	1	13	27	2	0	1
EABT34667	6	12.05	34	3	5	2	3
EABT34668	2	1	2	1	1	3	1
EABT34669	1	0	18	0	0	0	1
EABT3467	1	14	24	40	28	0	5
EABT34670	0	0	12	1	0	0	0
EABT34671	1	1	1	0	0	2	2
EABT34672	271.06	480	620.21	4561.9	1227.95	180	111.03
EABT34673	433	693	329.01	2090	717.98	322	356
EABT34674	0	0	0	8	1	0	0
EABT34675	386	829	1160	957.45	408	463	364
EABT34676	1	5	5	3	0	0	0
EABT34677	0	3	29	2	0	0	0
EABT34678	1	5	12	8	2	0	0
EABT34679	44	60	6	21	2082	4	17
EABT3468	2	2	0	0	0	0	0
EABT34680	2197.13	2261.38	2885.08	2440	592	1193.15	764.35
EABT34681	6	19	13	16	0	0	0
EABT34682	1	4	7	1	1	1	0
EABT34683	0	1	24	0	0	0	4
EABT34684	5	0	13	4	1	3	0
EABT34685	0	5	6	0	0	0	0
EABT34686	0	2	2	3	0	0	0
EABT34687	3	3	3	1	1	1	1
EABT34688	870.32	1863.78	5423.5	3595.04	1194.84	1334.59	928.03
EABT34689	0	0	5	0	0	0	0
EABT3469	12	5	12	8	4	5	6
EABT34690	5	0	11	4	0	2	3

EABT34691	0	4	2	2	0	0	0
EABT34692	33	40	58	34	56	18	46
EABT34693	7712.32	7514.59	4845.63	5730.98	4458.29	8864.71	6112.36
EABT34694	1	0	2	0	0	0	2
EABT34695	10546.4	6322.61	4234.13	9442.55	3047.78	12935.2	10858.1
EABT34696	0	0	0	1	0	0	1
EABT34697	0	4	0	1	0	0	1
EABT34698	5	8	23	0	0	3	5
EABT34699	0	1	2	2	0	0	0
EABT347	0	0	2	15	0	0	0
EABT3470	3	2	5	0	0	6	4
EABT34700	1	3	2	0	1	1	0
EABT34701	2	14	56	79	1	1	0
EABT34702	11	5	0	0	0	0	0
EABT34703	1	1	7	0	1	0	0
EABT34704	3	2	7	0	1	0	0
EABT34705	41	46.16	0	1	22	1	1
EABT34706	4	27	2	3	4	8	0
EABT34707	0	0	6	0	2	0	2
EABT34708	4	5	20	2	1	2	6
EABT34709	0	0	0	0	0	0	0
EABT3471	0	0	5	0	1	1	2
EABT34710	0	1	3	4	1	0	0
EABT34711	787.42	1185.37	926.53	880.34	516.61	894.85	834.7
EABT34712	1	0	8	1	0	0	0
EABT34713	0	1	1	3	2	1	7
EABT34714	1	1	8	0	0	1	0
EABT34715	22	6	19	16	22	0	0
EABT34716	1	3	14	6	1	1	3
EABT34717	0	3	0	0	0	3	0
EABT34718	5	3	43	0	0	2	1
EABT34719	1562.49	2308	9955.18	3243.88	3364	1126.95	1454.03
EABT3472	246	188	172	182	56	137	68
EABT34720	1	2	11	2	1	0	0
EABT34721	0	0	0	0	1	0	0
EABT34722	1	4	3	14	2	0	0
EABT34723	1	5	0	3	0	0	0
EABT34724	1	9	3	0	3	0	0
EABT34725	0	0	16	0	0	0	0
EABT34726	0	1	13	0	0	0	0
EABT34727	1	1	22	0	1	6	2
EABT34728	0	0	8	546.01	2	0	0
EABT34729	925.5	1842.19	3647.75	5237.27	2315.6	666.25	961.36
EABT3473	3	17	6	50	0	3	3
EABT34730	1234.31	1443.51	207	156	382	2174.94	1833.8
EABT34731	2	3	10	18	5	2	7
EABT34732	2	19	109	6	3	2	9
EABT34733	1	3	7	2	0	0	0
EABT34734	1	9	34	0	2	0	2
EABT34735	0	0	6	0	0	0	0
EABT34736	0	5	9	0	0	0	0

EABT34737	1	5	0	1	0	0	0
EABT34738	5.01	6	11	13	3	2	0
EABT34739	5	2	10	4	4	6	8
EABT3474	0	0	13	0	0	0	0
EABT34740	0	5	6	4	3	0	0
EABT34741	0	2	21	0	0	0	0
EABT34742	0	1	0	14	1	0	0
EABT34743	2	5	16	2	3	0	3
EABT34744	3165.2	5096.96	4002.5	6510.92	2766.4	3341.26	4842.05
EABT34745	4	12	1	10	6	2	1
EABT34746	0	1	5	4	2	0	1
EABT34747	257	354	110	663	303.99	205	146
EABT34748	929	1137.85	1202	1357.89	860.62	1272.89	896.78
EABT34749	2	5	5	2	0	3	3
EABT3475	2	0	1	1	0	1	0
EABT34750	0	1	4	7	1	2	2
EABT34751	0	0	4	2	1	0	0
EABT34752	26	6	46	15.83	2993.5	14	37
EABT34753	2	2	0	0	0	3	2
EABT34754	463.97	642.06	602.62	933.28	655.06	582.74	499.08
EABT34755	1	0	1	0	2	0	0
EABT34756	0	0	12	1	21	0	0
EABT34757	4	4	50	7.83	5	1	6
EABT34758	20429	13461.3	274	212.23	3269.59	32873	44414.7
EABT34759	0	2	9	1	0	0	0
EABT3476	189.01	135	91	31	11	14	23
EABT34760	0	3	0	7	0	0	1
EABT34761	1	0	3	0	0	0	0
EABT34762	0	0	9	0	0	0	0
EABT34763	0	0	8	0	0	0	0
EABT34764	1	1	1	4	0	0	0
EABT34765	4	3	81	2	2	0	2
EABT34766	2924.12	8188.2	5991.82	513	1081.99	10105.9	4606.21
EABT34767	0	2	4	0	1	0	0
EABT34768	0	2	48	2	1	0	1
EABT34769	0	1	6	4	9	2	0
EABT3477	0	0	1	3	1	0	2
EABT34770	1	2	15	4	1	3	5
EABT34771	21	3	0	0	1	34	1
EABT34772	0	1	6	0	0	0	0
EABT34773	5	50	68	179	135	1	1
EABT34774	160.94	333	329	445.06	196.84	97	178.52
EABT34775	1440.66	1916.95	7493.84	4914.52	2155.38	954.5	1268.85
EABT34776	5	9	48	16	2	4	1
EABT34777	75.02	152.97	98	246.1	75	40.91	56.41
EABT34778	5	46	22	63	0	0	0
EABT34779	0	0	44	0	0	0	0
EABT3478	1	8	13	18	6	0	1
EABT34780	0	7	16	1	0	3	2
EABT34781	1	1	1	0	0	0	1
EABT34782	5	1	0	1	0	7	0

EABT34783	0	1	1	0	17.08	1	0
EABT34784	2	3	3	0	4	1	0
EABT34785	5	9	7	20	0	2	2
EABT34786	1295.24	2024.15	1338.37	1986.62	1620.98	715	927.37
EABT34787	0	0	9	0	0	0	0
EABT34788	18	36	8	3	9	2	0
EABT34789	3110.14	4794.21	10982.3	11712.8	4017.51	3509.95	4287.14
EABT3479	5	7	7	3	0	1	3
EABT34790	9173.05	11755.8	15881.7	32814.4	2416.05	2571.67	2379.4
EABT34791	2	5	6	8	0	0	2
EABT34792	2	1	11	2	0	1	3
EABT34793	0	0	6	0	0	0	0
EABT34794	1	1	8	4	1	2	0
EABT34795	1	5	26	0	2	3	1
EABT34796	10	61	94	120	27	21	13
EABT34797	1	0	5	1	0	0	0
EABT34798	3582.38	4415.93	3813.84	6157.95	2796.02	4595.65	3913.09
EABT34799	0	1	10	0	0	0	1
EABT348	2	10	0	0	0	5	0
EABT3480	0	1	8	0	0	1	0
EABT34800	3	13	20	3	1	3	2
EABT34801	2	1	1	3	1	0	2
EABT34802	1429.05	1689.19	2690.15	2949.04	1845.03	1167.31	1251
EABT34803	81.16	113.93	175.59	217.03	126.5	78.54	83.19
EABT34804	0	2	0	0	0	6	0
EABT34805	0	0	3	8	2	0	1
EABT34806	3743.59	5702.58	8327.67	8692.96	2639.35	2771.23	3064.23
EABT34807	0	1	2	0	2	0	0
EABT34808	1	6	26	11	1	2	0
EABT34809	0	0	14	0	0	0	0
EABT3481	11	10	27	12	96.73	6	10
EABT34810	2	1	2	1	1	0	1
EABT34811	0	0	2	3	0	3	0
EABT34812	1	2	1	1	3	2	2
EABT34813	0	0	10	0	0	0	0
EABT34814	0	1	1	2	3	0	0
EABT34815	2	0	3	0	0	0	0
EABT34816	274.73	534.13	702.75	547.16	149	13	20
EABT34817	2	7	3	20	0	0	1
EABT34818	0	0	5	1	0	0	0
EABT34819	3	3	0	0	1	1	1
EABT3482	1	2	10	0	0	0	0
EABT34820	0	0	5	0	1	0	0
EABT34821	3004.97	3789.01	4393.79	3727.39	2150.13	2048.24	2380.51
EABT34822	8298.73	9492.04	4207.38	4867.06	3542.02	8460.6	5062.94
EABT34823	0	2	12	2	1	0	3
EABT34824	1	10	67.88	25	8	1	3
EABT34825	0	0	11	0	1	0	1
EABT34826	0	0	1	0	0	0	0
EABT34827	1	1	19	0	0	0	0
EABT34828	2	5	1	12	1	0	1

EABT34829	0	0	1	0	0	0	0
EABT3483	61743.2	60599.7	48806.2	21450.6	43194.1	21016.3	24259.7
EABT34830	2179.07	3702.95	4220.8	4802.59	2317.37	1687.06	2339.17
EABT34831	2	5	38	11	4	11	4
EABT34832	2389.95	49.99	2	0	1	411.16	0
EABT34833	10	24	191	26	2	0	0
EABT34834	0	0	0	0	0	0	0
EABT34835	4	5	42	0	2	0	1
EABT34836	192	292	500.08	570.82	330	219	176
EABT34837	6745.94	10800.5	4523.26	4819.22	1674.77	4746.2	2759.06
EABT34838	9	7	8	2	4	8	2
EABT34839	0	0	2	0	0	0	0
EABT3484	31	25	6	27	12	5	6
EABT34840	9	24	117	243	165.87	4	34.9
EABT34841	0	0	0	0	0	0	3
EABT34842	1228.8	1960.79	1403.25	4174.38	1223.29	876.24	924
EABT34843	1	2	17	2	2	0	0
EABT34844	2	2	1	8	2	0	0
EABT34845	2	2	11	1	2	2	1
EABT34846	1	0	11	0	5	2	0
EABT34847	0	5	15	0	0	1	0
EABT34848	2	1	17	0	0	0	0
EABT34849	1	6	15	7	2	1	0
EABT3485	1	3	6.01	6	21	1	2
EABT34850	50	28	9	2	0	258	0
EABT34851	3	5	7	0	5	1	2
EABT34852	0	0	5	1	0	0	0
EABT34853	2	2	2	13	5	1	0
EABT34854	0	0	7	5	2	1	1
EABT34855	8	5	5	7	3	0	1
EABT34856	8	16	21	44	8	1	10
EABT34857	0	5	22	0	4	0	0
EABT34858	1	2	2	1	0	2	0
EABT34859	5986.77	6960.21	2716.31	9564.99	2719.37	3446.67	3869.69
EABT3486	4	2	0	0	0	14	1
EABT34860	20	40	11.92	241.12	13	8	10
EABT34861	0	1	3	0	0	0	0
EABT34862	6	65	52	194.63	337.3	5	33
EABT34863	0	0	0	0	0	0	0
EABT34864	1108.99	2596.67	930.8	2314.39	834.62	911.63	932.03
EABT34865	0	0	1	0	0	0	0
EABT34866	1	2	19	2	2	0	3
EABT34867	4407.48	4005.08	1978.89	1965.83	9106.18	2360	2253.7
EABT34868	9	44	130.93	63	11	3	5
EABT34869	0	0	0	0	0	0	0
EABT3487	0	1	4	2	0	0	0
EABT34870	0	0	2	2	5	4	3
EABT34871	2	1	14	0	0	5	0
EABT34872	34	30.71	22	17	30	132	28
EABT34873	618	971.98	804.96	1039	725	243	430.18
EABT34874	0	0	0	2	0	0	0

EABT34875	2	7	15	6	2	1	0
EABT34876	2	9	17	7	5	1	3
EABT34877	0	1	17	2	2	2	1
EABT34878	0	2	13	4	5	1	0
EABT34879	6	39	8	4	11	1	1
EABT3488	0	0	7	1	0	0	0
EABT34880	1	1	138	1	5	0	0
EABT34881	9	20	3	13	1	3	4
EABT34882	0	0	11	1	0	0	0
EABT34883	7	2	1	8	0	5	1
EABT34884	0	0	4	0	2	1	0
EABT34885	2499.83	4919.83	9618.4	8157.73	3423.35	2323.18	3071.74
EABT34886	101	27	4	10	25	589	865
EABT34887	18	132	1183.8	3021.57	75	13	6
EABT34888	0	0	3	5	0	2	0
EABT34889	0	5	5	0	1	0	0
EABT3489	3	14	50	7	3	0	5
EABT34890	0	0	0	0	3	0	0
EABT34891	4011.41	5253.19	4675.1	8022.6	5073.99	4863.89	5546.07
EABT34892	1922.9	3885.38	1495.87	3007	1680.22	124	464
EABT34893	11	17	81	37	6	24	16
EABT34894	3	1	3	0	0	0	0
EABT34895	75	140.01	221.25	346	330	42	58.08
EABT34896	0	7	0	0	0	2	0
EABT34897	559.21	685	215	850.55	362	351.01	277
EABT34898	0	3	6	8	20	0	0
EABT34899	2	7	16	0	0	0	0
EABT349	121	166	146	193	345	43	66
EABT3490	0	3	1	4	0	0	2
EABT34900	1	2	91	0	2	0	0
EABT34901	0	6	23.01	6	4	0	1
EABT34902	0	4	6	2	0	0	0
EABT34903	342	369	352	3791.01	218	100	169.83
EABT34904	14	36	11	54.21	0	46	1
EABT34905	9	12	0	0	0	0	0
EABT34906	0	2	9	1	0	0	0
EABT34907	393	740.37	1141.69	1339.02	875.88	419	611
EABT34908	6490.69	7801.03	7249.2	3857.5	18360.9	6347.64	7189.09
EABT34909	24	39	55	43	67	24	60
EABT3491	0	0	2	0	1	1	0
EABT34910	3126	3486	2928.42	4379.26	2282.97	2888	1558.97
EABT34911	1	1	3	0	4	0	1
EABT34912	0	0	10	2	0	2	1
EABT34913	685	1185	1107.88	4296.99	1031.05	378	456.34
EABT34914	910.8	1301.33	1631.33	22	675.58	193.02	2773.19
EABT34915	1	0	4	0	0	0	1
EABT34916	0	2	2	0	1	0	0
EABT34917	3	4	2	6	0	1	0
EABT34918	3.94	2	1	10	1	2	1
EABT34919	13	15	0	0	0	0	1
EABT3492	1631.12	1965.13	926.64	381.06	527.86	1962.4	1231.9

EABT34920	2415.08	3789.93	8231.94	6200.79	1662.83	5834.85	3664.99
EABT34921	720	1283.84	1496.22	2020.77	1139.28	315	477
EABT34922	7171.47	2944	93	5209.01	1616.54	3114.22	1782.04
EABT34923	0	0	0	3	2	0	1
EABT34924	0	1	2	3	1	0	0
EABT34925	0	0	0	0	0	0	0
EABT34926	0	0	0	10	0	0	0
EABT34927	2	16	2	0	3	1	2
EABT34928	0	5	3	5	0	1	2
EABT34929	3	7	1	14	0	0	8
EABT3493	2	0	7	3	1	0	0
EABT34930	0	0	2	2	0	0	0
EABT34931	0	2	6	0	0	0	0
EABT34932	1	4	8	2	2	0	0
EABT34933	558.91	1241.97	967.97	389.97	3065.05	14.82	138.55
EABT34934	0	0	3	8	0	0	0
EABT34935	0	5	6	30	1	1	0
EABT34936	0	4	42	1	1	0	0
EABT34937	0	1	1	3	0	0	0
EABT34938	26	46	5	16	7	7	17
EABT34939	10	27.05	21.01	26	7	3	16.4
EABT3494	0	2	9	2	0	4	0
EABT34940	2	2	10	2	3	1	2
EABT34941	3	5	1	3	6	3	3
EABT34942	0	3	10	14	1	2	2
EABT34943	1011.1	1395.31	1334.22	3808.01	642	1802.06	2095.51
EABT34944	3	1	8	3	0	0	0
EABT34945	8	4	13	11	1	2	1
EABT34946	0	3	8	9	1	1	0
EABT34947	9252.74	9794.15	10497.3	7769.13	7875.09	9285.34	8938.31
EABT34948	6	21	60	85	2	0	0
EABT34949	0	5	5	0	0	3	0
EABT3495	1	1	7	0	0	0	0
EABT34950	371.96	792.75	1357.21	876.02	502.46	68	212.04
EABT34951	2	13	53	32	6	1	3
EABT34952	1	0	7	1	0	0	0
EABT34953	1	6	11	6	3	6	4
EABT34954	0	2	0	2	4	0	2
EABT34955	0	0	1	18	0	0	0
EABT34956	1	8	17	14	16	4	6
EABT34957	0	0	5	2	2	0	0
EABT34958	8	18	30	20	35	13.12	17
EABT34959	0	3	2	1	0	0	0
EABT3496	2	8	0	1	1	0	0
EABT34960	3	13	38	27	3	2	4
EABT34961	7	5	3	12	0	4	9
EABT34962	4	16	6	10	27	3	46
EABT34963	5	11	0	1	3	1	16
EABT34964	2325.24	4443.96	3302.94	7812.72	4491.47	2286.75	3474.14
EABT34965	1190.54	2230.35	2907.66	2736.74	1126.11	996.94	1272.31
EABT34966	1	1	8	0	1	1	0

EABT34967	380.15	739.76	516.94	853	305	410.16	352.98
EABT34968	6	6	0	0	0	0	0
EABT34969	25	12	5	37	8.35	9	8
EABT3497	2373.06	3607.4	5974.71	7365.23	2888.25	2481.88	1539.58
EABT34970	1	0	1	3	2	0	0
EABT34971	1355	1425.71	981	1467	951.54	801	644.86
EABT34972	1	9	1	2	5	29	6
EABT34973	689.99	1449.39	1740.94	2952	1953.09	344.4	563
EABT34974	1	1	8	0	0	0	0
EABT34975	0	1	4	0	0	0	0
EABT34976	0	0	1	0	2	0	0
EABT34977	10	9	21	7	3	0	2
EABT34978	31	34	13	2	2	52.12	4
EABT34979	37	43	117	69	68	2	19
EABT3498	34.81	141.97	1244.2	159.01	240.02	95.42	186.91
EABT34980	1	10	11	6	6	4	5
EABT34981	2	6	0	0	1	7	3
EABT34982	0	0	11	1	0	0	0
EABT34983	0	1	4	4	3	0	2
EABT34984	1	2	25	2	1	1	0
EABT34985	0	0	19	0	0	0	0
EABT34986	28	61	0	333	60	1	3
EABT34987	0	7	0	0	0	12	4
EABT34988	0	4	234.98	19	8	0	14
EABT34989	6	12	91	45	5	4	2
EABT3499	950	1764.94	1274.89	1051.69	1289.03	1123.06	1036.99
EABT34990	2	2	1	0	0	5	0
EABT34991	0	0	6	0	0	0	0
EABT34992	2	1	16	2	0	0	2
EABT34993	8	23	31.98	23	4	4	6
EABT34994	3	4	13	14	3	4	1
EABT34995	32	110	195.99	143.33	2574.38	12	6962.91
EABT34996	0	1	2	5	0	0	0
EABT34997	0	5	4	1	1	0	0
EABT34998	0	2	1	4	3	0	1
EABT34999	0	0	3	3	1	0	0
EABT35	2882.58	5822.88	4371.76	4218.93	4014.3	2254.48	6388.33
EABT350	1	2	1	5	0	0	0
EABT3500	3	3	0	0	0	5	0
EABT35000	0	2	4	3	1	0	1
EABT35001	0	1	2	7	6	0	0
EABT35002	685	19	5	1	0	821.02	1
EABT35003	0	0	3	7	0	1	0
EABT35004	6	9	3	4	0	1	1
EABT35005	0	1	5	1	1	1	0
EABT35006	0	2	11	2	0	0	0
EABT35007	1	1	0	2	0	1	0
EABT35008	0	1	9	0	0	0	0
EABT35009	0	2	14	8	0	0	0
EABT3501	0	2	5	2	1	3	4
EABT35010	5761.07	2792.93	8052	35234.3	2471	33	86

EABT35011	136.81	286.45	930.98	1088.59	676	217.01	246.99
EABT35012	0	0	16	11	0	0	0
EABT35013	1448	754.8	12	14	38	1987.06	538
EABT35014	4	7	7	2	0	0	0
EABT35015	13	16	32	3	12	10	35
EABT35016	3	10	36	9	3	3	1
EABT35017	2	3	81	11	1	1	3
EABT35018	3	7	5	5	4	2	1
EABT35019	2553.95	3981.77	2113.94	3041.11	2639.47	1866	3027.99
EABT3502	532.68	1712.28	4936.94	1573.09	2079.2	75.85	670.7
EABT35020	33	0	1	0	0	6	0
EABT35021	38	89	374.83	43	21	51	55
EABT35022	0	0	13	2	0	0	0
EABT35023	0	0	25	0	0	0	0
EABT35024	0	0	0	0	9	0	0
EABT35025	1	2	9	8	1	0	0
EABT35026	15	22	295	29	17	6	9
EABT35027	0	1	5	1	2	2	1
EABT35028	1	2	1	3	0	0	0
EABT35029	0	1	1	3	0	0	0
EABT3503	0	0	1	0	0	0	0
EABT35030	4	5	9	11.26	4	1	4
EABT35031	0	0	4	2	0	0	0
EABT35032	171458	307264	190876	101611	129064	51	3436.44
EABT35033	0	0	3	0	2	0	0
EABT35034	19	42.98	34	69	33	6	12
EABT35035	375	761.1	370.64	1740.92	545.14	129	330.38
EABT35036	3969.88	5511.4	5926.9	6401.54	4379.22	5273.07	4476.65
EABT35037	4	11	14	10	0	0	1
EABT35038	2	3	84	2	0	1	0
EABT35039	172	354.06	248	266	254	37	142
EABT3504	1	0	62	0	0	1	1
EABT35040	2	3	3	1	0	1	1
EABT35041	3326.39	6476.99	4876.74	31243.5	6971.9	155	303.94
EABT35042	3772.94	4088.26	287.74	2	9378.41	3	45
EABT35043	0	1	23	0	3	0	0
EABT35044	1	8	17.95	6	1	1	3
EABT35045	1	4	24	3	4	0	0
EABT35046	0	3	8	1	1	0	0
EABT35047	316.01	406	397	842	228	124	111
EABT35048	0	1	5	0	0	0	0
EABT35049	137.07	209.78	332.84	645.29	363	169	148.97
EABT3505	5	23	6	2	0	8	0
EABT35050	428.98	391	886.78	1887.28	499	250	851.01
EABT35051	3	12	16	1	0	0	0
EABT35052	0	0	7	0	0	0	0
EABT35053	2	0	6	1	1	0	0
EABT35054	16	10	48	26	4	18.75	20
EABT35055	2	3	22	7	2	1	0
EABT35056	0	1	15	1	0	0	3
EABT35057	26854.3	21999.4	16967.6	17886	9705.84	20209.2	14473.1

EABT35058	11	122	138.67	15	56.28	1	1
EABT35059	1	7	4	5	2	0	0
EABT3506	13	27	54	4	8	6	16
EABT35060	266.83	302.91	175.08	543.99	290.97	127.99	92
EABT35061	0	0	2	2	1	0	3
EABT35062	4440.1	6755.63	6606.16	13980.5	5475.81	4631.89	4668.92
EABT35063	3	6	5	1	3	0	0
EABT35064	9	13	37	5	8	2	14
EABT35065	0	0	25	1	0	0	0
EABT35066	5	2	0	0	0	2	0
EABT35067	0	5	59	1	0	0	0
EABT35068	1	3	0	0	0	1	0
EABT35069	1063.01	966	503	117	938	1531	872
EABT3507	10	11	4	1	0	1	2
EABT35070	4045.58	6331.13	2555.34	2820	3039.46	4330.84	7009.97
EABT35071	0	0	2	0	0	0	1
EABT35072	27248.8	22360.4	247	130	1662.94	8732.4	1046.96
EABT35073	2	3	1	0	0	3	0
EABT35074	1	0	6	5	0	2	2
EABT35075	0	1	5	0	0	1	1
EABT35076	25	27	47.03	60.02	16.94	11	18.98
EABT35077	9755.28	26484.4	44654.7	3031.05	21065.4	5656.57	17335.8
EABT35078	7	10	11	17	1	2	8
EABT35079	0	1	2	3	0	0	0
EABT3508	12	18	15	32	10	5	5
EABT35080	0	1	0	0	1404	0	0
EABT35081	4	1	0	0	0	6	3
EABT35082	2	17	14	9	2	11	7
EABT35083	1	6	7	4	1	2	1
EABT35084	0	1	10	0	0	0	1
EABT35085	13	33	75	42	13	18	7
EABT35086	17	14	10	0	0	25	8
EABT35087	1	1	0	0	0	4	2
EABT35088	0	0	6	0	0	0	2
EABT35089	0	0	4	0	0	0	0
EABT3509	63	137	75	83	1002.98	0	20
EABT35090	0	1	8	2	0	0	1
EABT35091	1	0	7	2	1	0	2
EABT35092	0	3	9	2	0	0	0
EABT35093	0	2	4	2	1	0	0
EABT35094	29	77.02	23	55.14	63	11	48
EABT35095	0	1	3	0	1	0	0
EABT35096	1	4	2	6	1	1	1
EABT35097	1	0	2	0	0	0	0
EABT35098	1	2	11	0	5	0	0
EABT35099	3	1	0	0	0	4	1
EABT351	0	2	14	0	0	0	0
EABT3510	0	1	9	1	0	0	0
EABT35100	1	1	15	0	0	1	0
EABT35101	0	0	36	12	12	1	0
EABT35102	8	3	4	13	2	0	1

EABT35103	118	191.31	109	196	172	36.21	114.02
EABT35104	0	10	2	3	1	0	0
EABT35105	0	2	2	3	0	0	0
EABT35106	1	2	1	0	0	0	1
EABT35107	0	9	10	3	2	0	2
EABT35108	0	0	2	0	0	0	3
EABT35109	496	1396.13	3021.01	2807	757.94	61	342
EABT3511	1	2	2	1	1	0	0
EABT35110	0	2	1	0	0	1	0
EABT35111	1	3	10	7	0	0	0
EABT35112	2	3	10	2	0	5	1
EABT35113	6	15	32	10	1	0	0
EABT35114	1301.99	2591.43	4537.94	1885.85	2309.43	289.06	1157.25
EABT35115	3	6	22	4	7	0	1
EABT35116	33	30.94	8	61	20	3	82.98
EABT35117	0	0	2	4	0	0	0
EABT35118	1006.4	1240.6	1883	2198.12	1236.41	654.01	695.1
EABT35119	13904	1180	97	20	15	5611.54	52
EABT3512	0	1	2	4	1	0	0
EABT35120	0	0	0	3	1	0	5
EABT35121	0	6	12	1	1	2	0
EABT35122	2	5	53	2	2	0	0
EABT35123	0	0	0	0	0	0	0
EABT35124	14	16	1	0	0	0	0
EABT35125	388.11	785.04	445.08	897.83	715.2	228	184.07
EABT35126	392	784.83	757.12	2260.61	529	267	349
EABT35127	0	3	6	2	3	0	0
EABT35128	0	5	16	0	13	5	2
EABT35129	0	0	5	6	1	0	0
EABT3513	12	23	68	61	2	8	7
EABT35130	594.98	915.9	671.44	956.52	828.78	534.11	534.58
EABT35131	25	21	24	5	5	11	9
EABT35132	1662.43	1353.86	1157.23	200.99	17	107.14	55.03
EABT35133	0	3	0	1	0	0	1
EABT35134	2	6	4	36	429	41.01	97.02
EABT35135	1	1	10	0	0	0	0
EABT35136	2163.04	4222.87	6097.53	6147.15	3252.37	1626.21	1880.39
EABT35137	5	7	33	42	0	2	2
EABT35138	0	1	0	13	1	0	0
EABT35139	62.01	155.84	111	394.93	211	22	110
EABT3514	1	0	6	0	1	1	4
EABT35140	1	3	7	26	8	1	1
EABT35141	1	1	0	37	2	0	0
EABT35142	14	52	12	12	101	1	18
EABT35143	827.67	1123.02	953.28	3626.14	888.19	743.01	576
EABT35144	71	146	142	698	187	48	70
EABT35145	208.38	389.93	1018.11	262.33	628.01	30	222.33
EABT35146	8	5	31	1	1	1	0
EABT35147	0	0	7	1	0	0	0
EABT35148	40196	28483.1	53	82	191	9051.08	1396.93
EABT35149	1574	3206.99	2044.03	3542.62	3545	579	766

EABT3515	77	145.01	819.07	480.32	382.37	207.47	219.83
EABT35150	3	2	3	0	0	3	0
EABT35151	0	0	0	3	1	0	0
EABT35152	0	1	0	5	0	0	0
EABT35153	1	1	7	0	0	1	0
EABT35154	829	1098.01	1606.99	1647.99	796.51	741	634.71
EABT35155	2	1	3	4	0	0	0
EABT35156	0	1	10	1	0	0	0
EABT35157	2	5	7	2	0	0	0
EABT35158	1357.05	2219.81	2320.27	3096.65	2303.41	1415.32	2164.33
EABT35159	1	2	6	1	0	0	0
EABT3516	0	0	5	0	1	0	0
EABT35160	4148.24	11572.2	26431.8	4176.94	4691.18	1849	2085.97
EABT35161	1	1	2	0	0	0	0
EABT35162	4	2	12	10	1	9	1
EABT35163	0	3	0	3	2	0	2
EABT35164	4326.49	9440.96	4557.9	6005.08	4488.45	5402.66	5265.63
EABT35165	22	13	0	0	0	2	0
EABT35166	1040.86	1832.4	2120.56	2062	1313.96	1051.74	1020.79
EABT35167	0	0	4	0	1	0	0
EABT35168	0	0	0	7	0	0	0
EABT35169	573.81	653.65	357.46	777.94	517.93	371.2	451.98
EABT3517	0	3	9	3	2	0	1
EABT35170	0	1	7	0	0	0	0
EABT35171	0	0	3	4	0	0	0
EABT35172	1	0	17	1	3	2	0
EABT35173	15	25	0	0	2	49	14
EABT35174	10125.5	15804.6	14549.1	20122.7	10641	12043	10929.1
EABT35175	126	246	993.88	3194.17	545.01	60	144.02
EABT35176	0	2	7	0	0	0	0
EABT35177	1	0	10	1	1	0	1
EABT35178	2	3	22.9	0	0	0	1
EABT35179	1	0	22	1	0	4	0
EABT3518	0	1	0	3	0	0	0
EABT35180	702	955	1343	1286.87	820	572	573
EABT35181	0	0	8	0	0	0	0
EABT35182	1	8	7	0	0	21	8
EABT35183	0	0	4	0	0	0	1
EABT35184	7	1	0	0	3	10	2
EABT35185	3	2	9.97	4.99	1	3	3
EABT35186	803.86	1014.21	698.56	1264.58	1479.57	609.42	588.96
EABT35187	27.04	51	154	145	21	30	27
EABT35188	242	322	107	196	242	546.97	963
EABT35189	89	329.02	1721.13	6821.65	352.27	133	101
EABT3519	508.99	661.9	616.47	798.81	470	340	345
EABT35190	3011.09	3630.22	9769.7	896.44	4318.38	3788.63	4781.99
EABT35191	4064.21	8258.55	4473.62	6819.12	2368.41	4105.89	3017.08
EABT35192	0	5	9	0	0	0	0
EABT35193	0	2	7	1	5	0	5
EABT35194	0	5	0	0	0	5	1
EABT35195	0	0	9	6	0	0	0

EABT35196	12	18	179.81	44	23	0	68
EABT35197	3	4	6	22	6	0	0
EABT35198	134.02	262.09	219.86	684.46	231	127.99	109.02
EABT35199	0	3	21	3	2	1	0
EABT352	5	13	55	6	1	4	1
EABT3520	0	0	0	5	1	0	0
EABT35200	850.1	1441.99	1262.91	1443.57	1112.28	649.09	917.6
EABT35201	94	62.96	38	75	17	2	23
EABT35202	0	2	4	0	1	11	1
EABT35203	1	2	3	8	1	0	0
EABT35204	0	2	0	3	0	0	0
EABT35205	0	0	10	0	0	0	0
EABT35206	1	2	0	0	3	0	1
EABT35207	0	7.99	54	4	2	3	1
EABT35208	1	3	13	2	0	0	0
EABT35209	202.07	639.63	1353.44	44	323.27	5	196
EABT3521	0	2	1	1	301	0	1
EABT35210	0	0	0	2	0	0	0
EABT35211	8	9	26	0	1	0	1
EABT35212	0	2	11	1	1	1	2
EABT35213	742	829.96	195	19	325	908	1377.34
EABT35214	11	60	93	29	25	2	6
EABT35215	0	0	6	0	0	0	1
EABT35216	0	5	6	6	2	1	4
EABT35217	0	0	0	5	0	0	1
EABT35218	2	3	15	187	24	0	0
EABT35219	2	12	50	3	4	1	0
EABT3522	25281.1	30097.7	9026.43	13428.9	3037.01	1607.83	1005.9
EABT35220	3183.3	4346.1	2237.46	1656.99	6528.48	1109.05	1382.76
EABT35221	712.7	959.02	1032.41	1773.07	723.69	472.71	576.02
EABT35222	1	0	5	0	1	7	0
EABT35223	267	66	20	0	3	0	18
EABT35224	1	0	3	3	1	0	0
EABT35225	3	5	3	1	1	1	4
EABT35226	1	1	7	0	0	1	0
EABT35227	59218.8	35055.2	12833.6	191.9	11	6076.45	7
EABT35228	9	39	217	34	16	15	8
EABT35229	0	1	0	4	0	1	0
EABT3523	557	727	1242	1089	898.53	347	454
EABT35230	0	0	12	0	0	0	0
EABT35231	3	16	8	32	4	5	1
EABT35232	772.04	1150.85	1006.71	1446.17	1229.58	735.17	951.49
EABT35233	102.6	879.01	121	2	0	1	2
EABT35234	1	9	4	1	3	6	2
EABT35235	3	5	10	12	2	0	1
EABT35236	0	3	8	0	1	1	0
EABT35237	1	0	4	0	0	0	1
EABT35238	2	7	13	5	0	0	0
EABT35239	1	0	2	1	0	1	0
EABT3524	270.63	431.98	372.02	723.67	403.05	230.17	277.47
EABT35240	1	0	5	1	7	0	1

EABT35241	4867.91	9254.29	23030.9	14082.9	3864.77	2609.1	2669.34
EABT35242	8	8	9	0	16	20	32.01
EABT35243	67	138.07	125	202.05	156.97	45.75	227.66
EABT35244	0	0	4	0	1	6	1
EABT35245	5	7	14	64	73	10	11
EABT35246	1	0	2	3	0	0	1
EABT35247	0	0	0	0	3	12	2
EABT35248	5	1	2	0	0	0	0
EABT35249	0	5	1	0	0	0	0
EABT3525	0	0	4	6	0	0	0
EABT35250	0	0	0	0	0	0	1
EABT35251	0	1	8	9	3	1	0
EABT35252	0	2	6	3	0	0	0
EABT35253	1	5	4	3	1	1	1
EABT35254	2	10	24	21	0	0	3
EABT35255	7	23	35	12	0	2	2
EABT35256	615	1120.69	548	4167.74	1114.22	363.21	528.44
EABT35257	0	0	4	0	1	0	0
EABT35258	0	3	9	5	0	3	0
EABT35259	0	2	0	0	0	2	1
EABT3526	612	922	2675.83	2288	672	16	8
EABT35260	1	0	11	0	0	0	0
EABT35261	3	2	3	6	0	0	2
EABT35262	1622	2673.7	1619	1763.01	4178.5	639	539
EABT35263	1	3	9	2	3	2	3
EABT35264	2	1	12	3	4	1	4
EABT35265	3	6	37	4	0	0	1
EABT35266	50.48	36	2	0	0	4	5
EABT35267	0	0	2	5	2	1	0
EABT35268	0	1	10	1	0	2	1
EABT35269	1	1	2	1	1	0	2
EABT3527	0	0	4	3	0	1	0
EABT35270	510.01	721.3	648.95	2307.41	491.02	348.03	465.12
EABT35271	1	5	3	5	1	1	0
EABT35272	0	0	10	0	1	0	0
EABT35273	0	0	6	0	0	0	0
EABT35274	1	1	2	0	1	1	0
EABT35275	2775.66	4307.63	6758.71	10355.8	4577.73	4137.66	4060.02
EABT35276	1617.27	7207.98	6271.7	4038.58	4756.45	217	2093.93
EABT35277	0	1	3	1	0	0	1
EABT35278	1	2	3	3	5	2	5
EABT35279	2	7	41	9	7	1	6
EABT3528	2	1	25	5	1	0	0
EABT35280	0	1	1	1	1	0	0
EABT35281	0	0	5	8	1	0	0
EABT35282	0	0	9	1	0	0	0
EABT35283	114	251	210	230	210	136	182
EABT35284	0	1	6	1	1	0	6
EABT35285	0	8	25	9	2	0	4
EABT35286	1	6	3	11.99	1	0	1
EABT35287	54	175	161.99	78	45	21.98	80

EABT35288	143	170	119	224	62	104	83
EABT35289	1	0	7	4	1	0	0
EABT3529	162	297	3254.51	560	86	0	0
EABT35290	6	8	19	24	31	2	12
EABT35291	8	47	8	15	10.98	5	4
EABT35292	4	6	46	2	3	0	0
EABT35293	0	1	2	0	0	0	1
EABT35294	5	15	111	16	10	6	7
EABT35295	0	0	8	0	0	1	0
EABT35296	1	2	1	1	1	0	0
EABT35297	0	6	16	2	1	0	0
EABT35298	1	0	6	1	0	0	0
EABT35299	2	1	9	1	10	1	3
EABT353	0	1	16	0	0	1	0
EABT3530	13073.6	9999.91	12811.2	12549.7	7353.63	20233.2	8866.66
EABT35300	7192.82	6987.78	4206.4	5378.6	5548.82	9233.15	6806.77
EABT35301	3267.82	2163.3	1396	1481.16	482.94	54	206
EABT35302	3	1	35	1	0	8	1
EABT35303	2	22	18.99	24	4	17	9
EABT35304	0	0	2	0	0	0	1
EABT35305	4223.35	4907.85	1257.64	4251.14	2036.76	3569.84	3272.25
EABT35306	2	5	0	8	4	1	0
EABT35307	0	5	8	0	1	1	2
EABT35308	0	0	8	1	0	0	0
EABT35309	2	1	5	22	5	2	2
EABT3531	419.25	912.48	1134	3297.34	1200.52	522.25	886.21
EABT35310	1304.21	1905.11	1235.49	3480.96	941.75	219.84	387.63
EABT35311	1	2	8	1	2	4	0
EABT35312	2	4	6	4	1	2	5
EABT35313	1	4	7	1	1	3	2
EABT35314	490	579.13	970.06	965	569	291	365
EABT35315	0	3	98.91	4	1	3.99	0
EABT35316	2770.83	4106.32	4609.15	6356.72	2698.01	2139.37	1731.06
EABT35317	0	1	6	1	0	0	0
EABT35318	171.08	305.19	294.94	1079.12	555.97	133.94	153
EABT35319	0	5.02	6	6	0	0	0
EABT3532	2752.18	2789.97	487.99	10	127.13	8978.78	908.95
EABT35320	0	5	0	0	1	5	2
EABT35321	4	2	0	0	2	9	7
EABT35322	0	1	1	0	2	0	1
EABT35323	2	4	5	5	0	2	0
EABT35324	0	1	9	1	1	0	0
EABT35325	0	0	8	1	0	0	0
EABT35326	568.01	802.54	442	350	149	1429.15	579
EABT35327	0	0	5	0	0	0	0
EABT35328	22	29	0	0	1	0	0
EABT35329	0	1	35	0	0	1	0
EABT3533	1917.35	2907.17	2606.37	8006.4	2905.9	1796.23	1422.91
EABT35330	15	19	6	24	4	3	4
EABT35331	1	5	13	3	30	1	47
EABT35332	554	811.47	343	867.99	368	379	367.46

EABT35333	408.37	1039.37	954.1	8	91	20	26.64
EABT35334	1471.93	1663.2	5032.79	4758.94	2650.73	1848.06	1836
EABT35335	35	9	1	19	1	7	1
EABT35336	0	0	0	1	0	1	0
EABT35337	3	4	21	3	2	0	0
EABT35338	0	0	34	0	1	0	1
EABT35339	1	1.03	0	0	0	1	7
EABT3534	2	3	18	5	0	0	2
EABT35340	0	1	31	0	0	0	0
EABT35341	0	9	8	1	0	0	0
EABT35342	1	3	1	8	6	0	1
EABT35343	5	8	2	5	1	1	0
EABT35344	728.97	2891.55	3356.06	3442.87	577.89	24	34
EABT35345	0	0	1	1	5	0	0
EABT35346	304	395.99	349	731	291	216	181
EABT35347	0	0	5	2	1	0	2
EABT35348	0	4	192	12	30	1	1
EABT35349	0	1	0	0	1	1	0
EABT3535	0	1	17	2	2	1	4
EABT35350	0	3	1	2	0	0	0
EABT35351	391.01	569.61	301.44	920.57	651.11	448.69	496.24
EABT35352	1	0	1	5	1	1	2
EABT35353	0	0	12	0	0	0	0
EABT35354	5	5	38	18	3	0	0
EABT35355	0	0	4	0	0	0	0
EABT35356	2	13	10	2	4	2	0
EABT35357	1	2	16	0	0	1	0
EABT35358	735	958.54	470.93	1615	428	13	39
EABT35359	0	4	1	3	0	0	1
EABT3536	0	0	10	1	0	0	2
EABT35360	15	0	0	0	0	0	0
EABT35361	2	1	7	3	3	0	2
EABT35362	0	1	6	5	0	0	0
EABT35363	0	2	11	0	0	1	0
EABT35364	9	6	42	51	1	0	1
EABT35365	723.19	873.17	28	24.34	24	500	47
EABT35366	0	0	6	0	0	4	1
EABT35367	928.23	1096.29	959.8	1212.79	615.28	707.16	473.3
EABT35368	0	2	9	12	0	0	0
EABT35369	0	0	3	0	1	0	0
EABT3537	1	0	26	1	2	0	0
EABT35370	3	14	16	2	1	1	0
EABT35371	0	0	6	42	0	2	0
EABT35372	0	3	0	2	2	0	0
EABT35373	0	0	17	2	0	0	0
EABT35374	0	1	9	1	0	0	0
EABT35375	1	0	1	9	0	1	0
EABT35376	1	4	31	9	1	0	0
EABT35377	1450.07	2230.48	2663.17	6258.19	1775.37	1355.1	1521.6
EABT35378	3	8	16	7	1	2	1
EABT35379	0	0	16	2	0	0	0

EABT3538	1	4.98	11	15	3	3	3
EABT35380	2	1	10	9	3	4	3
EABT35381	1	7	2	13	1	0	1
EABT35382	14	31	38	8	25.89	19	14
EABT35383	1320.99	1906	2263.98	2932.23	1859.98	1795.16	1294.02
EABT35384	0	2	9	2	2	4	0
EABT35385	2865.66	3626.18	2806.84	6924.43	2730.79	3172.3	2878.04
EABT35386	10	7	75	3	4	8	5
EABT35387	3	52	13	0	1	14	1
EABT35388	0	1	48	1	1	0	1
EABT35389	1	0	2	1	0	0	0
EABT3539	0	0	2	0	1	0	0
EABT35390	740.2	1679.32	580.02	677.79	700.35	213	206.2
EABT35391	1	1	0	1	0	3	1
EABT35392	10	3	1	0	2	17	3
EABT35393	0	1	0	0	6	0	0
EABT35394	5	8	19	19	7	8	13
EABT35395	0	3	1	0	1	1	0
EABT35396	494	1340.3	868.99	1394	1113.72	289.98	915.77
EABT35397	9	14	17	27	3	1	2
EABT35398	1	7	98	0	0	2	4
EABT35399	1	3	5	3	0	0	1
EABT354	0	0	0	0	0	0	0
EABT3540	3	3	26	1	0	0	0
EABT35400	1	4	0	1	5	0	2
EABT35401	1137.87	2097.93	2921.41	3423.4	2088.64	1032.13	1026.72
EABT35402	10	29	17	87	21	2	6
EABT35403	10	23	27	2	2	15	17
EABT35404	0	0	1	1	0	0	2
EABT35405	92	183	349.19	325	75	42.05	93.49
EABT35406	26.43	2	0	0	0	9	0
EABT35407	0	0	6	6	0	0	0
EABT35408	4138.88	2694.75	2	0	520	7526.81	2133.02
EABT35409	0	0	6	11	0	0	2
EABT3541	1	4	3	631	15	2	0
EABT35410	39	43	22	141	539	2	19
EABT35411	0	0	0	0	0	0	6
EABT35412	0	0	2	0	2	1	0
EABT35413	4	9	8	18	7	7	2
EABT35414	0	1	5	2	1	0	0
EABT35415	0	4	6	1	0	0	0
EABT35416	54.86	85	389.06	254	25	65	26
EABT35417	3	17	48	12	3	0	2
EABT35418	1	1	3	2	1	0	0
EABT35419	0	0	3	1	0	0	1
EABT3542	3	2	5	0	1	0	0
EABT35420	0	0	0	0	1	0	2
EABT35421	4	3	2	5	4	4	12
EABT35422	2	11	235.89	2	0	0	0
EABT35423	3	3	4	3	0	1	2
EABT35424	11	3	8	3	5	2	3

EABT35425	1	2	2	2	0	1	1
EABT35426	2	3	1	0	0	0	0
EABT35427	13	51	903	2488.75	40	94	77
EABT35428	1	2	2	0	5	1	9
EABT35429	4	0	7	3	0	0	3
EABT3543	436	58	28	20	2	130	3
EABT35430	1538	2424.21	1301.98	4361	1217	1392.05	1696.99
EABT35431	0	3	14	2	0	0	0
EABT35432	8	21	0	0	1	3	1
EABT35433	2	5	7	10	8	0	2
EABT35434	536	1086	1288	2426.07	764	479	872.04
EABT35435	3119.16	4406.04	346	383.96	1759.37	8506.66	11432.1
EABT35436	2	9	22	97	12	3	12
EABT35437	3	9	18	0	1	1	0
EABT35438	0	1	3	7	0	0	0
EABT35439	0	0	5	0	0	1	0
EABT3544	4	19.58	25	26.96	3	2	4
EABT35440	6	1	0	0	2	0	0
EABT35441	0	0	1	0	3	0	2
EABT35442	2	0	5	0	0	2	6
EABT35443	1	8	2	7	1	0	1
EABT35444	10	61	20.13	13	4	7	7
EABT35445	1256.01	1770	1804.95	1927.77	1327.03	770	784.02
EABT35446	4	5	6	1	4	0	1
EABT35447	0	0	0	0	0	0	9
EABT35448	7	14	28.01	18	9	0	2
EABT35449	1	1	12	1	1	0	0
EABT3545	1	2	4	1	2	1	0
EABT35450	0	0	1	1	0	0	0
EABT35451	15	60	52	35	6	1	2
EABT35452	553.94	694.16	519.88	783.35	710.26	621.34	447.46
EABT35453	0	0	1	2	1	0	0
EABT35454	1	3	58	2	2	2	4
EABT35455	4	6	103	5	9	2	2
EABT35456	0	0	7	2	0	0	1
EABT35457	3	3	4	6	3	0	0
EABT35458	0	4	6	1	1	0	0
EABT35459	2	2	31	2	9	2	0
EABT3546	0	1	10	1	0	0	0
EABT35460	2	2	7	1	0	2	2
EABT35461	0	1	0	6	0	0	0
EABT35462	1	0	0	0	0	0	0
EABT35463	0	1	1	10	2	0	8
EABT35464	2	9	8	38	3	9	19
EABT35465	120629	92754.2	48610.1	42503.3	1100365	46667.2	187877
EABT35466	2	3	0	5	0	3	0
EABT35467	0	0	12	2	1	0	0
EABT35468	0	0	2	0	0	0	0
EABT35469	1	0	2	5	1	0	0
EABT3547	9	42	7	19	5	24	6
EABT35470	290	312	601	691	291	199	268

EABT35471	0	1	5	3	1	0	4
EABT35472	8	23	45.74	8	53	0	0
EABT35473	0	0	5	0	0	0	1
EABT35474	0	0	13	0	0	0	1
EABT35475	6604.32	5833.78	3415.46	1576.13	3381.11	6483.16	4214.79
EABT35476	0	0	5	0	0	0	3
EABT35477	74	49	15	54	58	3	10
EABT35478	2	1	3	1	0	1	0
EABT35479	2	1	9	12	3	6	1
EABT3548	0	1	4	0	0	0	1
EABT35480	0	0	8	0	1	1	0
EABT35481	4	27	27	40	7	5	3
EABT35482	13	2	13	2	2	0	0
EABT35483	2	0	1	8	0	0	0
EABT35484	0	2	5	7	0	0	0
EABT35485	0	0	2	0	27	0	0
EABT35486	639.21	723.97	1167.01	2322.8	611.99	572	680
EABT35487	1	3	8	2	0	0	0
EABT35488	1	1	8	1	0	0	0
EABT35489	472.18	996.45	765.47	1570.26	535.38	470.42	474.54
EABT3549	0	82	127	4583.65	55	0	0
EABT35490	578.08	448.99	46	19	14	1007.08	83
EABT35491	138	128	106	325	37	26	54.06
EABT35492	2	6	6	2	1	1	0
EABT35493	23	93	103.35	126.7	24	45	50
EABT35494	0	1	1	3	1	0	0
EABT35495	0	0	6	1	0	0	0
EABT35496	1	2	0	0	0	0	0
EABT35497	1	1	32	4	0	1	1
EABT35498	1	2	3	1	0	1	0
EABT35499	8	6	1	0	0	14	0
EABT355	0	1	1	0	1	0	0
EABT3550	5	6	20	6	1	0	1
EABT35500	1	9	12	9	0	2	1
EABT35501	0	1	6	2	4	0	1
EABT35502	0	1	3	1	0	0	0
EABT35503	2	0	18	4	0	1	1
EABT35504	0	0	2	0	1	0	0
EABT35505	0	1	3	1	3	0	0
EABT35506	0	6	0	0	2	0	0
EABT35507	2	1	14	1	1	2	0
EABT35508	593.2	787.17	639.26	3732.33	648.96	1558.43	2303.52
EABT35509	0	1	9	0	4	0	9
EABT3551	0	1	6	3	0	1	0
EABT35510	1	3	1	7	0	1	1
EABT35511	6	53	70	0	9	0	2
EABT35512	0.25	2.53	98.4	4	0	0	0
EABT35513	71.38	211	236	636	47	66	173
EABT35514	126	82	0	1	2	112	36
EABT35515	6690.29	10938.9	13627	19723	7300.72	1721.98	2799.38
EABT35516	4	1	27	8	3	0	0

EABT35517	701.56	1008.27	421.62	1512.93	688.21	714.61	1003.46
EABT35518	11	6	10.08	2	1	4	1
EABT35519	6	2	0	0	0	8	0
EABT3552	3	1	1	5	0	0	12
EABT35520	7.89	10	0	4	3	2	0
EABT35521	2	1	11	2	2	1	0
EABT35522	0	2	6	3	10	0	0
EABT35523	9	25	2	0	4	16	0
EABT35524	2114.09	1806.12	1299.23	616.97	824.97	1233.26	1148.84
EABT35525	392.48	631	817.34	842.84	546.93	478.59	543.18
EABT35526	0	0	5	0	0	0	0
EABT35527	1	0	3	0	0	1	0
EABT35528	0	0	3	4	0	0	0
EABT35529	2921.24	5323.9	4250.98	9204.46	7091.97	779	2241.79
EABT3553	0	1	7	0	0	0	0
EABT35530	0	1	4	5	1	0	0
EABT35531	4	6	6	0	1	0	0
EABT35532	1	1	2	3	0	0	0
EABT35533	2	11	78	17	6	13	13
EABT35534	0	0	0	0	0	0	0
EABT35535	4	12	18	16	9	2	3
EABT35536	644.79	1864.38	994.07	2643.73	1225.96	1284.99	1823.44
EABT35537	0	0	4	2	0	0	0
EABT35538	39154.7	62679.1	23744.7	23011	54747.3	1954.28	10200.1
EABT35539	0	2	4	7	2	0	1
EABT3554	5	23	4	1	1	0	2
EABT35540	1	1	0	2	0	0	0
EABT35541	0	0	7	0	1	0	0
EABT35542	159.17	156.11	66	147	10	97.71	45.42
EABT35543	488.5	107	20	32	64	0	144
EABT35544	0	1	7	1	0	0	0
EABT35545	88.88	10	538	20	455.01	71	88
EABT35546	0	0	8	0	0	0	0
EABT35547	2	23	2	2	3	0	0
EABT35548	4	4	0	0	1	2	1
EABT35549	0	1	6	1	0	0	1
EABT3555	2	1	12	5	2	2	2
EABT35550	0	0	10	0	0	0	0
EABT35551	0	1	0	2	10	0	0
EABT35552	3	27	14	8	3	0	3
EABT35553	0	5	2	8	19	2	0
EABT35554	0	3	11	11	1	0	1
EABT35555	0	1	5	1	0	0	1
EABT35556	3	7	8.02	2	0	0	4
EABT35557	21266.2	11989.5	15557.7	2543.13	4523.27	23563.7	22334.2
EABT35558	0	0	9	1	1	0	1
EABT35559	0	4	5	1	1	0	0
EABT3556	3	2	4	3	1	3	2
EABT35560	410.95	748.81	697.36	1280.69	589.93	286	272.07
EABT35561	0	7	20	1	0	1	1
EABT35562	3	11	16	15	4	5	7

EABT35563	1744	3209.02	1046.01	1595	1614	1122	987
EABT35564	564.17	598.59	1578.35	5624.74	3397.47	108	8337.01
EABT35565	1	6	4	3	3	10	13
EABT35566	1	1	7	2	0	2	0
EABT35567	1330.97	2111.35	2250.98	424.13	509	3043.21	2501.63
EABT35568	0	0	4	4	0	0	2
EABT35569	527.01	820.99	875.67	863	946.96	651	415.7
EABT3557	3	3	25	5	4	4	1
EABT35570	181	390.98	281	136	286.67	223	186.16
EABT35571	0	1	2	2	1	0	1
EABT35572	691	926.04	1040	940.87	1125.76	599.08	749
EABT35573	0	0	0	3	5	0	0
EABT35574	2	1	7	1	0	1	0
EABT35575	1	6	24	6	1	2	0
EABT35576	3	4	2	1	0	4	0
EABT35577	0	0	0	0	0	0	0
EABT35578	10	10	3	15	3	11	5
EABT35579	33.5	49	0	0	0	4	5
EABT3558	0	0	2	0	0	10	1
EABT35580	546.96	1294.88	1072	3837.17	1295.72	858.05	1001.64
EABT35581	0	0	4	1	0	0	1
EABT35582	5	0	1	1	1	1	3
EABT35583	2	3	11	1	0	0	0
EABT35584	0	1	3	1	0	0	0
EABT35585	2	7	7	2	0	20	2
EABT35586	21	30	14	6	20	0	89
EABT35587	0	4	6	6	4	0	0
EABT35588	4	7	1	0	1	5	4
EABT35589	0	0	6	1	1	0	1
EABT3559	2	2	7	4	0	2	2
EABT35590	0	6	2	3	6	0	4
EABT35591	0	0	0	5	1	0	0
EABT35592	6	6	4	0	3	0	0
EABT35593	8	20	65	21	1	6	1
EABT35594	7068.11	6434.38	8063.41	715.84	67.82	3	117
EABT35595	2491.23	3153	1816.64	3833.42	1988.37	1780.6	1611.26
EABT35596	11	12.99	23	16	3	7	9
EABT35597	0	3	1	0	0	0	0
EABT35598	1	1	23	1	1	0	0
EABT35599	1	2	11	1	0	0	0
EABT356	778.57	1225.09	346.99	403.52	693.36	1219.06	1164.71
EABT3560	4	5.96	18	17	5	3	2
EABT35600	0	1	3	1	0	0	1
EABT35601	0	4	14	0	0	0	0
EABT35602	0	4	7	5	2	0	3
EABT35603	1	2	27	0	0	0	0
EABT35604	0	0	4	0	1	0	1
EABT35605	6	5	16	3	2	1	4
EABT35606	0	0	0	0	2	8	0
EABT35607	13	392.53	3	4	1448.67	0	14
EABT35608	197.98	217	203	676.98	132	14	12

EABT35609	1	3	23	1	1	4	2
EABT3561	30731.1	14704.4	8589.38	74	38	0	2
EABT35610	2	4	0	8	1	0	0
EABT35611	44	66	104	88	161	36	31
EABT35612	1	1	4	6	1	1	0
EABT35613	980.81	2098.09	3693.27	5518.37	2261.43	564.98	820.21
EABT35614	6	1273.39	27277.7	50338.6	33	360.97	242.03
EABT35615	1	0	5	0	0	0	2
EABT35616	1	2	2	39	0	1	1
EABT35617	0	0	6	1	0	0	1
EABT35618	178	594	263	68	95	25	110
EABT35619	323.05	470	596	1695.96	657.99	209	122
EABT3562	3	11	3	9	0	2	0
EABT35620	120.85	310.89	720.1	3157.54	407.73	16.77	99.91
EABT35621	593.01	739	454	704	368	446	373
EABT35622	0	4	15	4	3	3	9
EABT35623	1001.77	1905.58	2369.08	3265.97	1294.5	1169.33	1182.61
EABT35624	26	74	103	99.06	14	24	13
EABT35625	1	2	3	1	2	2	1
EABT35626	3	6	1	4	99	1	0
EABT35627	0	2	5	0	1	0	0
EABT35628	1736.02	3565.01	2917.87	5503.31	2412.04	1885	2213.18
EABT35629	1	2	2	3	0	0	0
EABT3563	8	22	34	5	9	14	17.04
EABT35630	0	5	8	11	0	2	2
EABT35631	1	4	3	3	2	1	29
EABT35632	2	5	4	7	1	1	0
EABT35633	6	52	15	4	0	0	0
EABT35634	1	6	9	11	1	11	0
EABT35635	0	0	9	0	0	0	0
EABT35636	1	0	7	0	0	0	0
EABT35637	1	1	7	0	0	0	0
EABT35638	0	1	1	0	0	2	1
EABT35639	7711.5	8279	2627.63	8601.53	3320.89	4819.81	7881.33
EABT3564	0	1	2	0	1	0	0
EABT35640	0	0	18	5	0	0	0
EABT35641	0	0	4	0	1	1	0
EABT35642	384.03	431.3	303.45	881.98	457.36	294.15	354.09
EABT35643	3	5	10	11.06	0	0	2
EABT35644	2	2	4	3	0	0	0
EABT35645	1	0	8	0	0	1	0
EABT35646	2	5	6	2	0	3	0
EABT35647	3	3	32	98	7	2	14
EABT35648	2	17	40	19	11	11	6
EABT35649	2	11	3	2	0	10	0
EABT3565	0	0	4	0	0	0	0
EABT35650	0	5	2	51	0	0	0
EABT35651	0	1	10	1	2	0	5
EABT35652	10	12	4	4	2	6	11
EABT35653	2	3	2	1	0	1	0
EABT35654	0	1	10	0	0	0	0

EABT35655	11	1	1	3	1	9	5
EABT35656	274	650.8	1868.93	1774.93	787.08	206	463.05
EABT35657	2	6	7	15	3	0	5
EABT35658	435	604.56	236	440	347	20	135
EABT35659	0	0	12	16	3	0	0
EABT3566	166.77	253.27	235.69	439.91	342.35	173.48	197
EABT35660	8	16	32	28	7	13	7
EABT35661	0	0	5	3	0	0	0
EABT35662	3	0	19	1	0	0	0
EABT35663	1414.3	5076.53	4267.42	5149.26	4461.89	2480.5	2964.18
EABT35664	8	21	35	45	4	1	1
EABT35665	655.1	200.03	145.88	335	148.78	101.57	272.57
EABT35666	1	1	18	0	1	3	1
EABT35667	5	14	0	0	0	5	0
EABT35668	6857.91	1261.81	102	988	183.98	6587.87	1343.03
EABT35669	0	0	0	3	0	1	0
EABT3567	6	5	6	4	3	0	0
EABT35670	3	1	0	0	0	1	2
EABT35671	2	3	0	8	0	4	5
EABT35672	0	0	2	3	3	0	2
EABT35673	0	0	9	3	0	1	0
EABT35674	18	11	0	0	2	5	4
EABT35675	37.17	49	38.97	120.12	35	18	25
EABT35676	0	0	0	2	0	0	0
EABT35677	754	834.94	368	1104.26	526.03	727	459.09
EABT35678	1	0	19	2	0	0	0
EABT35679	0	1	2	0	0	0	0
EABT3568	1	0	4	1	0	0	0
EABT35680	348	610	682.38	1017	708	307	349
EABT35681	5	4	18	66	3	2	2
EABT35682	121.01	1982.01	5412.97	723.99	339	483.66	255
EABT35683	10	24	0	0	0	9	1
EABT35684	2	2	6	4	0	2	2
EABT35685	2	2	6	4	10	0	3
EABT35686	2	1	13	0	1	2	1
EABT35687	0	0	5	0	0	0	0
EABT35688	908.71	1431.88	1963.78	2795.78	866.99	1061.98	863.01
EABT35689	4	15	83	43981.7	1	0	0
EABT3569	1709.64	2088.11	1050.24	2613.4	1782.01	1773.77	1378
EABT35690	1	2	0	2	0	1	0
EABT35691	1	3	6	1	1	0	1
EABT35692	1	2	3	11	1	0	2
EABT35693	1	1	11	1	0	0	1
EABT35694	1	1	9	0	18	2	0
EABT35695	1	0	4	5	0	0	0
EABT35696	123.95	147	77.01	168.74	334.01	95.01	84.48
EABT35697	0	3	18	6	2	2	1
EABT35698	2	11	4	41	5	0	1
EABT35699	2	3	0	0	1	8	3
EABT357	105	84	28	21	44	7	7
EABT3570	0	0	24	1	0	0	1

EABT35700	246.5	326.48	384.15	511.27	362.43	200.3	289.85
EABT35701	27	895.56	183	610.11	329.09	6	4
EABT35702	10	16	237	4	10	0	8
EABT35703	1958.48	3397.04	5060.94	7124.38	3339.01	1596.7	2318.22
EABT35704	0	0	2	4	0	0	0
EABT35705	0	0	7	1	0	0	0
EABT35706	0	2	1	1	0	0	1
EABT35707	7	23	0	0	0	4	0
EABT35708	1387.09	2721.86	3974.13	6751.33	4159.33	1038	11920.9
EABT35709	163.89	241.01	232.66	461.03	237.19	164	171.37
EABT3571	4	7	12	5	2	0	0
EABT35710	8	15	89	11	1	4	4
EABT35711	1	0	20	6	0	1	3
EABT35712	3	21	27	9	0	0	3
EABT35713	6	1	13	0	1	0	1
EABT35714	0	0	7	3	1	0	1
EABT35715	737	1051.26	966	1732	831.37	666.96	574
EABT35716	12	0	0	0	0	24	0
EABT35717	2820.65	5891.86	5815.62	16926.3	2514.3	2451.85	2750.88
EABT35718	1731.42	2185.17	2217.2	4692.86	1491.7	1272.53	1525.47
EABT35719	0	0	2	0	1	0	0
EABT3572	1	6	0	0	1	3	1
EABT35720	0	7.76	3	1	2	1	0
EABT35721	1	0	8	2	0	0	0
EABT35722	1	0	8	1	0	0	0
EABT35723	0	1	2	1	0	0	0
EABT35724	2	1	0	0	0	5	0
EABT35725	0	0	1	50	0	1	0
EABT35726	0	1	3	11	0	0	1
EABT35727	0	0	2	0	0	0	5
EABT35728	8	10	0	0	1	10	9
EABT35729	2	0	3	0	0	1	0
EABT3573	0	2	6	3	0	0	0
EABT35730	828.91	506	36	26	182	2136	1995.05
EABT35731	2229.12	4505.34	4083.54	9412.88	2200.62	2020.8	2190.47
EABT35732	1394.31	2539.16	3108.76	3972.38	1398.65	1298.11	1457.52
EABT35733	0	1	2	1	0	0	1
EABT35734	1	2	5	0	6	0	0
EABT35735	0	3	17	11	1	0	1
EABT35736	3	2	7	6	1	0	0
EABT35737	0	2	1	1	3	0	6
EABT35738	0	2	12	0	0	0	0
EABT35739	0	0	21	0	1	0	0
EABT3574	352	476.98	561	1007	497	383	338
EABT35740	0	0	2	0	0	0	1
EABT35741	10	8	84	42	6	4	6
EABT35742	183	388	258	434	334	234	149
EABT35743	4	6	6	2	1	0	1
EABT35744	3	6	6	11	0	0	1
EABT35745	3	5	5	2	2	0	1
EABT35746	0	0	5	0	0	0	0

EABT35747	0	2	11	10	1	2	0
EABT35748	56	27	672.68	4536.81	2	107.29	42
EABT35749	13	16	22	11	5	10	15
EABT3575	0	1	61	0	0	0	0
EABT35750	35	41	106	32	20	5	12
EABT35751	0	0	4	6	0	1	0
EABT35752	2	2	2	3	6	0	6
EABT35753	3277.47	2680.65	1481.08	1361.77	1386.54	717.71	996.98
EABT35754	0	2	16	0	0	1	1
EABT35755	5	1	12	5	0	1	0
EABT35756	640.73	895.58	900.99	1392.27	610.54	546.95	431.73
EABT35757	1703.17	3653.07	7463.98	1076.47	1748.97	870	783.87
EABT35758	0	0	6	0	0	1	0
EABT35759	6	5	18	4	3	0	12
EABT3576	0	0	0	0	37	0	0
EABT35760	1	2	0	2	0	1	1
EABT35761	3	7	19	2	1	0	3
EABT35762	0	0	1	12	0	0	0
EABT35763	0	0	4	2	0	0	0
EABT35764	0	4	6	1	1	2	9
EABT35765	19	22	8	2	3	42	12
EABT35766	1	1	11	1	1	2	2
EABT35767	2	8	1	14	0	0	0
EABT35768	3	2	5	7	0	3	1
EABT35769	2	4	0	1	0	1	0
EABT3577	0	0	4	2	0	0	0
EABT35770	1	0	1	4	1	0	1
EABT35771	0	1	1	2	0	0	0
EABT35772	4	9	123	2	4	2	1
EABT35773	1410	2193.51	3028.24	4646.09	2188.44	1995.16	2030
EABT35774	2151.56	4936.3	3632.06	8317.43	2936.49	1366	2587.6
EABT35775	110	23	22	338	389	1	19
EABT35776	0	0	20	0	0	0	0
EABT35777	0	0	4	1	0	0	0
EABT35778	3	3	112	3	0	0	0
EABT35779	278.76	488.39	1454.68	582.02	306	206	252
EABT3578	0	1	0	4	1	0	0
EABT35780	2	5	10	3	1	3	0
EABT35781	1	2	26	4	8	0	2
EABT35782	1298.81	1955.55	1447.13	3783.98	1222.76	1621.11	1311.57
EABT35783	0	0	0	0	2	0	0
EABT35784	2	0	9	16	1	1	1
EABT35785	2	4	4	1	0	5	2
EABT35786	239.37	403.88	293.64	944.8	350	167.92	233.58
EABT35787	0	0	6	0	0	0	0
EABT35788	1147	2387.4	1129.63	1617.65	115	9	28
EABT35789	733.9	816.1	670.74	1245.07	530	434	768.99
EABT3579	1	0	0	1	0	0	0
EABT35790	0	0	4	0	0	1	1
EABT35791	7	1	23	2	0	4	2
EABT35792	1	1	8	7	4	0	10

EABT35793	1	4	8	6	0	9	0
EABT35794	0	1	2	6	1	1	0
EABT35795	4	16	22	21	8	9	3
EABT35796	1024	1384	579	3091.23	1387.89	1324.18	1103.78
EABT35797	0	0	0	179	0	0	0
EABT35798	0	8	6	0	0	1	2
EABT35799	18	25	60.05	86	22	9	15
EABT358	7	0	0	0	1	2	1
EABT3580	0	0	30	0	0	1	0
EABT35800	1	2	32	6	3	0	1
EABT35801	0	1	44	2	0	0	0
EABT35802	17	31	142	192	137	8	32
EABT35803	1	5	2	0	0	13	34
EABT35804	977	1164	1471	6270.18	2558	1448.99	2635.02
EABT35805	0	2	6	0	0	0	0
EABT35806	5	2	0	0	3	0	1
EABT35807	1	0	0	0	0	0	2
EABT35808	322	971.64	2407.92	2148.85	2282.99	180	446.01
EABT35809	0	1	3	2	0	0	0
EABT3581	1.18	6	24	8.95	431.97	1	2
EABT35810	0	1	17	2	1	0	0
EABT35811	387.98	666	443	709.32	664	331	416
EABT35812	0	1	12	0	0	0	0
EABT35813	0	0	5	1	0	0	0
EABT35814	1814.07	2546.66	1423	3079.88	1792.93	1314.62	1278.5
EABT35815	1	2	6	2	0	0	0
EABT35816	23	1	5	0	0	10	0
EABT35817	18	15.12	37	109	14	8	24
EABT35818	3	2	1	0	3	3	12
EABT35819	4	5	4	6	4	0	1
EABT3582	1	7	5	2	4	1	5
EABT35820	0	0	2	7	1	0	0
EABT35821	2	2	9	1	1	0	0
EABT35822	4	2	10	0	1	0	1
EABT35823	0	0	16	0	0	0	0
EABT35824	6	6	3	0	0	13	14
EABT35825	0	0	9	0	1	0	0
EABT35826	12	20	43	61	8	5	6
EABT35827	1	0	6	2	0	0	0
EABT35828	0	1	1	0	0	2	0
EABT35829	2	3	5	6	5	1	0
EABT3583	2	6	16	21	2	2	6
EABT35830	5	14	22	6	31	1	3
EABT35831	0	1	2	0	0	0	0
EABT35832	0	8	3	9	1	11	0
EABT35833	2	0	2	1	3	0	4
EABT35834	0	0	1	4	0	0	1
EABT35835	94	225.99	97	122	84	14	23
EABT35836	5	69	11	0	5	30	26
EABT35837	0	1	7	0	0	0	0
EABT35838	0	0	0	7	0	0	0

EABT35839	0	0	7	0	0	1	0
EABT3584	0	0	8	1	0	0	0
EABT35840	0	0	1	1	1	0	0
EABT35841	6	8	7	17	0	1	1
EABT35842	937.35	1507.67	1623.53	3886	1009.99	1079.51	1362.91
EABT35843	0	0	0	0	0	0	0
EABT35844	0	1	23	1	0	0	0
EABT35845	0	6	4	1	0	0	0
EABT35846	98489.7	145796	158.94	11	8918	22131.5	74055.3
EABT35847	0	0	0	0	0	0	0
EABT35848	0	0	15	0	0	0	0
EABT35849	0	0	12	0	0	0	0
EABT3585	0	0	0	22	1	0	0
EABT35850	1	1	8	1	1	1	0
EABT35851	1376.99	1585.93	1187.26	2523.31	1204	1228	835
EABT35852	24	2	2	1	1	26	0
EABT35853	7.2	3	29	154	30	0	5
EABT35854	1	1	11	2	0	0	0
EABT35855	7646.56	8709.22	11499.1	9102.61	4392.51	6608.46	6817.63
EABT35856	0	1	6	0	0	1	0
EABT35857	0	3	1	3	0	1	3
EABT35858	1	0	2	0	2	0	4
EABT35859	0	1	2.24	1	2	1	1
EABT3586	2538.02	3385.66	2110.05	2642.7	2695.96	3439	2873
EABT35860	0	1	3	4	12	0	1
EABT35861	6	11	45	4	1290	2	6
EABT35862	0	0	3	1	0	0	0
EABT35863	234.23	243	121	594	156	85	194
EABT35864	1470.22	1962.31	1649.68	2368.32	2081.97	1119.71	1052.76
EABT35865	0	3	16	0	2	2	1
EABT35866	0	1	1	0	0	0	0
EABT35867	0	2	13	2	1	1	0
EABT35868	0	0	7	1	0	0	0
EABT35869	5	4	11	10	3	6	7
EABT3587	19	9	5	1	0	5	17
EABT35870	2	5	17	18.23	188	0	1
EABT35871	1	0	3	1	0	0	0
EABT35872	0	1	7	0	0	0	1
EABT35873	1	1	0	0	1	0	1
EABT35874	3	4	9	1	2	1	2
EABT35875	1	0	0	0	0	0	0
EABT35876	623.58	1738.66	898	316	405	19	21
EABT35877	0	0	5	0	1	0	0
EABT35878	1	4	9	14	1	0	1
EABT35879	2	3	2	3	1	1	5
EABT3588	6	2	32	1	0	0	0
EABT35880	0	3	22	1	2	0	3
EABT35881	0	1	45	6	0	0	0
EABT35882	1021.23	1562.68	1867.44	4282.11	3913.17	571	462.01
EABT35883	0	0	10	0	0	0	0
EABT35884	0	4	3	0	0	1	1

EABT35885	2	2	9	0	0	0	1
EABT35886	0	0	9	0	1	0	0
EABT35887	5	1	0	0	0	4	0
EABT35888	1	0	5	2	0	0	0
EABT35889	1	4	1	14	1	0	1
EABT3589	4	1	50	5	0	3	0
EABT35890	311	674	704	1350	469	277	428.12
EABT35891	22	27	26	35	17.98	17	44
EABT35892	0	0	5	1	1	0	1
EABT35893	0	0	6	0	0	0	0
EABT35894	0	6	3	1	8	0	1
EABT35895	1	5	7	4	0	0	3
EABT35896	0	0	5	0	0	0	0
EABT35897	1	2	10	2	1	1	1
EABT35898	3	3	58	4	0	0	0
EABT35899	0	1	7	4	4	2	3
EABT359	0	0	21	1	0	0	0
EABT3590	843.86	1013.89	518.01	1443.38	918.02	683.78	654.22
EABT35900	11	31	49.98	11	7	23	3
EABT35901	3	16	75	19	4	2	8
EABT35902	2	8	8	19	3	0	2
EABT35903	0	0	3	4	0	0	0
EABT35904	1	0	4	0	0	0	0
EABT35905	2	0	2	0	0	0	0
EABT35906	4249.19	7124.19	2560.58	1108.84	4283.85	2250.57	5806.61
EABT35907	2	5	6	5	0	0	1
EABT35908	191	406.85	129	65	10	0	3
EABT35909	1	5	22	5	1	0	0
EABT3591	2	27	10	20	0	4	4
EABT35910	0	1	2	2	1	1	0
EABT35911	1	3	8	0	0	0	1
EABT35912	0	1	6	5	0	0	1
EABT35913	1	0	0	0	2	0	0
EABT35914	2327.58	4144.17	6186.43	6205.57	3526.51	1123.39	2364.25
EABT35915	0	0	14	1	0	0	0
EABT35916	57	97	124	1220.06	380.9	13	19
EABT35917	1	3	6	0	1	1	0
EABT35918	3	7	13	8	1	2	1
EABT35919	0	0	1	5	0	0	1
EABT3592	28	34	0	0	0	9	46
EABT35920	17816.8	9154.37	3037.24	4048.96	4745.87	17479.9	9804.99
EABT35921	17	13	8	55	44	6	33
EABT35922	1	0	6	3	0	0	0
EABT35923	1165	3270.54	4763.19	3350.83	1220.76	776.49	765.9
EABT35924	0	0	2	2	0	0	0
EABT35925	0	1	7	15	0	0	0
EABT35926	0	2	4	0	0	0	0
EABT35927	1465.85	2651.41	3256.48	3847.81	2365.81	1751.57	1593.65
EABT35928	2954.07	5083.64	3924.59	9554.73	2438.72	2971.2	2623.53
EABT35929	3	2	27	28	17	0	2
EABT3593	0	0	1	0	834.38	0	0

EABT35930	1	4	8	3	1	0	3
EABT35931	7143.22	10331.2	9056.32	16374.9	5728.82	5314.76	5262.65
EABT35932	0	0	6	0	0	0	0
EABT35933	0	0	26	0	0	1	0
EABT35934	986.91	1615.64	1108.38	3154.33	976.13	405.93	637.99
EABT35935	0	0	9	2	0	0	0
EABT35936	349.92	567	350.07	1389.42	613	197	262
EABT35937	1	0	8	0	0	0	0
EABT35938	0	1	3	1	0	1	0
EABT35939	0	4	7	7	0	4	6
EABT3594	3427.43	8287.68	4681.34	513.87	3576.13	4016.78	7011.38
EABT35940	2	2	8	0	0	0	0
EABT35941	29	5	4	7	7	1	2
EABT35942	3925.04	4364.71	1727	4711.88	2277	2519.57	2587.05
EABT35943	0	0	6	1	1	0	0
EABT35944	0	2	3	16	0	0	1
EABT35945	3	1	4	0	6	2	4
EABT35946	1526	2230	1416	2100.03	1150	1132.97	1105
EABT35947	0	3	3	0	0	1	0
EABT35948	1	2	12	0	1	0	1
EABT35949	1.99	0	6	4	1	0	0
EABT3595	0	2	1	2	1	0	0
EABT35950	0	0	2	1	1	0	0
EABT35951	3	5	4	1	0	19	0
EABT35952	3	0	81	4	0	4	2
EABT35953	0	1	10	0	1	0	3
EABT35954	2	10	27	3	2	2	1
EABT35955	37270.4	26503	13185.3	772.01	16813.9	13156.6	70293.6
EABT35956	27.99	41	71	106.64	38	11	12
EABT35957	0	0	2	3	0	0	0
EABT35958	15350.7	6426.01	883	1975.95	1314	322.06	2555.15
EABT35959	10	5	0	0	0	23	0
EABT3596	16	1	23	42	26	20	14
EABT35960	1	0	7	2	0	4	0
EABT35961	0	0	6	0	0	0	0
EABT35962	0	0	1	17	2	0	1
EABT35963	1	0	77	1	1	0	0
EABT35964	0	2	5	6	1	0	0
EABT35965	0	2	10	1	0	0	0
EABT35966	649.03	1155.87	2055.76	2859.76	869.34	115	406.63
EABT35967	7	7	29	3	1	0	0
EABT35968	0	2	11	2	0	0	1
EABT35969	0	1	3	0	0	0	2
EABT3597	3	8	21	1	4	2	4
EABT35970	0	1	1	0	0	0	0
EABT35971	2	6	5	4	2	0	4
EABT35972	5	1	0	0	0	1	1
EABT35973	5	10	6	7	7	1	12
EABT35974	2093.85	2818.99	4060.14	1882	1524.99	2126	1646.41
EABT35975	678.77	925.05	1323.06	2405.72	626.46	229.65	272.7
EABT35976	279.98	343.09	249.01	402.01	640.1	131.97	190.98

EABT35977	4	13	65	21	10	0	1
EABT35978	1	3	5	10	12	0	1
EABT35979	8	15	27	43	14	4	7
EABT3598	35	22	7	1	0	25	11
EABT35980	6507.76	7494	4373.93	3097.21	8274.86	1000.89	6521.71
EABT35981	53148.5	6386.14	23250.7	70977	89	4	9
EABT35982	0	1	1	3	5	3	1
EABT35983	0	4	9	0	1	0	1
EABT35984	2	7	7	0	1	1	0
EABT35985	897.04	1066	501	1617.44	815	966.2	564
EABT35986	470.99	48	60	110	63.01	35	65
EABT35987	72	113	441	98	55	173	156
EABT35988	0	1	2	7	1	0	1
EABT35989	508	1334.99	1836.74	1911.55	993.89	169	307.27
EABT3599	4	41	23	26	1	2	1
EABT35990	0	1	2	3	0	0	0
EABT35991	0	1	4	0	0	0	0
EABT35992	3958.56	5419.62	3300.18	9056.09	2588.93	3463.77	3243.42
EABT35993	0	0	7	0	0	0	0
EABT35994	229	1640	73	636.62	8	4	12.37
EABT35995	1301.11	2065.69	1434.6	3097.28	1507.94	437.02	864.26
EABT35996	3946.06	5396.72	5187.11	8048.48	4389.42	4326.5	3532.22
EABT35997	2	16	10	4	0	0	0
EABT35998	0	3	13	0	2	1	1
EABT35999	456	640	1112.03	890.99	607.28	196	200
EABT36	6	7	11	18	2	2	2
EABT360	11820.1	5806.99	100	55	6350.68	17679.1	31301.8
EABT3600	2	4	0	0	0	0	0
EABT36000	10	18	9	3	0	1	0
EABT36001	5	4	8	7	1	0	1
EABT36002	1	2	6	1	0	0	0
EABT36003	0	2	4	0	1	2	0
EABT36004	2	19	18	15	1	2	1
EABT36005	21	44	17	13	5	7	70
EABT36006	520.44	903.34	724.69	1423.94	454.07	346.81	363.99
EABT36007	0	0	0	0	0	0	0
EABT36008	1	3	5	43	4	0	1
EABT36009	1	1	24	0	0	0	1
EABT3601	1	2	12.4	1	1	0	2
EABT36010	9	28	14	12	18	2	12
EABT36011	0	1	2	0	2	0	0
EABT36012	1	2	4	42	1	4	0
EABT36013	0	0	3	0	0	0	0
EABT36014	2	2	2	2	0	0	0
EABT36015	0	0	3	0	1	0	1
EABT36016	4	7	4	16	12	1	2
EABT36017	1219.07	1459.46	1767.84	2887.96	888.58	687	801.02
EABT36018	1	4	8	4	2	0	1
EABT36019	0	1	0	0	0	1	0
EABT3602	0	9	3	29.06	18	1	1
EABT36020	0	3	1	2	0	0	0

EABT36021	299.03	511.95	422.57	704.34	335.31	127	239.98
EABT36022	0	2	4	2	0	1	1
EABT36023	0	0	11	0	0	0	0
EABT36024	240.98	410.06	82	62	22	0	1
EABT36025	1	1	12	1	0	0	1
EABT36026	1	7	16	1	2	0	1
EABT36027	3	9	12	30	3	5	3
EABT36028	1	0	2	11	0	1	0
EABT36029	0	2	7	3	0	1	0
EABT3603	7	52	68	64	5	2	1
EABT36030	0	1	13	0	0	0	0
EABT36031	1038.46	1265	1524.51	2512.41	791.37	1126.84	883.23
EABT36032	0	1	30	4	0	0	0
EABT36033	0	0	3	0	0	1	0
EABT36034	0	2	8	14	2	1	0
EABT36035	0	0	2	0	0	3	0
EABT36036	281	262	44.17	159	408	267	133
EABT36037	0	3	9	0	1	0	0
EABT36038	7	11	52	9	1	1	2
EABT36039	0	3	31	0	0	2	1
EABT3604	0	3	4	5	4	0	0
EABT36040	1	3	18	5	0	0	0
EABT36041	0	4	4	6	0	0	0
EABT36042	0	1	0	1	0	0	0
EABT36043	3006.86	1343	1984	1936.65	1502.06	0	51
EABT36044	1720.11	2788.88	2664.1	3209.52	2041.16	1266.43	1190
EABT36045	0	1	1	6	1	0	0
EABT36046	4	12	7	9	10	2	0
EABT36047	1	3	2	7	2	1	0
EABT36048	3	3	65	1	2	2	1
EABT36049	0	1	6	7	0	1	0
EABT3605	0	0	2	0	1	0	0
EABT36050	0	0	0	1	2	0	1
EABT36051	0	0	5	4	0	0	0
EABT36052	15	25	51	29	14	12	14
EABT36053	1	0	19	1	0	0	0
EABT36054	2	2	23	2	0	0	1
EABT36055	0	0	4	0	1	0	1
EABT36056	0	2	1	4	1	3	3
EABT36057	1	3	1	0	0	0	0
EABT36058	0	0	5	2	0	0	0
EABT36059	0	0	0	0	0	0	0
EABT3606	5206.27	8712.58	10951.2	7919.07	3396.17	4956.86	4165.28
EABT36060	0	1	8	0	2	0	0
EABT36061	0	1	6	3	1	1	4
EABT36062	101.94	261	274.99	462.45	124	56	52
EABT36063	0	1	2	0	0	0	0
EABT36064	2101.06	3327.1	3692.86	5089.51	3362.15	3389.47	2494.12
EABT36065	0	3	1	7	12	0	19
EABT36066	0	1	1	0	0	2	0
EABT36067	0	0	24	7	0	0	0

EABT36068	3339.98	4323.48	2784.21	6446.51	2379.01	2787.85	2206
EABT36069	0	1	43	0	0	0	0
EABT3607	5	51	46	4	3	5	1
EABT36070	0	1	18	2	3.01	3	3
EABT36071	2	4	8	0	0	1	0
EABT36072	1	4	6	3	2	0	0
EABT36073	8	11	13	10	6	3	11
EABT36074	2	0	3	0	1	0	0
EABT36075	1	2	5	9	0	1	1
EABT36076	2	5	4	3	1	0	1
EABT36077	0	1	2	0	0	1	1
EABT36078	7	10	18	33.97	9	2	1
EABT36079	7	21	9	20	7	1	5
EABT3608	378.91	617.93	666.72	1149.4	625.86	361.01	442.72
EABT36080	30	125	531.92	162	154	7	27
EABT36081	971	2171.13	194	143	82	1049	348.99
EABT36082	2	1	0	10	0	0	8
EABT36083	0	2	5	0	0	0	0
EABT36084	0	2	2	2	0	0	1
EABT36085	1	1	0	10	1	0	0
EABT36086	0	0	0	0	39	12	160.14
EABT36087	0	0	25	0	0	0	0
EABT36088	3	5	14	11	3	5	5
EABT36089	32	34	22	48	34	51	43.94
EABT3609	64	701	53	4	8	12	3
EABT36090	0	0	9	0	0	0	1
EABT36091	1024.99	15011.6	4070.35	148.85	4	0	0
EABT36092	5	1	2	0	3	8	8
EABT36093	0	0	9	1	0	1	0
EABT36094	0	0	44.01	3	0	0	0
EABT36095	0	0	0	0	0	0	1
EABT36096	0	0	4.87	1	0	0	0
EABT36097	2	6	11	9	172	1	1
EABT36098	0	1	5	4	0	1	0
EABT36099	224	369	560	1038.01	297.85	153.93	190
EABT361	8	10	11	6	0	8	4
EABT3610	1	17	16	9	2	1	2
EABT36100	1	2	0	0	0	1	0
EABT36101	119	310	2941.69	3903.41	827.59	24	51
EABT36102	4	13	2	11	2	0	0
EABT36103	0	0	7	1	1	0	0
EABT36104	1	5	17	6	7	5	2
EABT36105	11	9	31	16	3	3.2	0
EABT36106	7	8	0	0	0	4	0
EABT36107	0	7	6	2	0	2	0
EABT36108	31.35	43	38	48	15	12	20
EABT36109	7	1	52	21	9	0	0
EABT3611	565.14	701.01	978	1369.31	812	684	507.97
EABT36110	6	6	1	0	0	1	0
EABT36111	0	0	0	0	0	0	0
EABT36112	4	4	6	1	0	9	6

EABT36113	2	6	4	15	6	1	1
EABT36114	11	20	55	14	2	25	6
EABT36115	1540.38	3228.87	5577.55	5237.64	3297.72	1124.43	2052.64
EABT36116	2	23	15	25	101	0	1
EABT36117	0	0	4	2	1	0	1
EABT36118	2	2	53	4	0	1	0
EABT36119	16	13	1	1	0	0	0
EABT3612	6	19	12	88.06	15	6	6
EABT36120	6	20	38	0	14	0	6
EABT36121	1	2	3	0	1	0	0
EABT36122	44	81	46.12	138.05	84.54	49	74
EABT36123	2	1	8	2	1	0	0
EABT36124	2	9	82	2	1	0	0
EABT36125	1	3	0	6	0	0	1
EABT36126	1712.43	1077.32	177.67	1307.88	1597.17	35.08	75.94
EABT36127	0	0	0	0	0	0	0
EABT36128	0	2	5	0	1	1	0
EABT36129	0	0	11	0	0	0	0
EABT3613	1	4	2	1	2	0	1
EABT36130	0	0	7	1	1	0	0
EABT36131	0	1	11	7	1	0	0
EABT36132	0	0	5.92	2.88	0	0	2
EABT36133	1728.78	2577.53	2614.56	3682.57	1988.1	1386.72	1467.02
EABT36134	0	0	0	3	1	0	0
EABT36135	1	1	3	1	0	1	2
EABT36136	1	2	1	0	0	0	0
EABT36137	1	4	26	3	5	2	1
EABT36138	216	170	76	73	43	570.4	586
EABT36139	5	14	25	17	7	3	8
EABT3614	0	1	2	2	2	0	0
EABT36140	0	0	1	2	0	1	0
EABT36141	1	1	6	40	25	1	6
EABT36142	1	3	6	12	26	0	1
EABT36143	0	0	4	4	0	0	7
EABT36144	0	0	0	44	0	0	0
EABT36145	269.32	653	801	1662.64	409	197.04	177
EABT36146	0	0	6	0	0	0	0
EABT36147	3	0	0	0	0	3	0
EABT36148	0	0	3	3	0	0	0
EABT36149	810.51	1206.07	1551.76	2353.21	1003.08	1068.01	863.28
EABT3615	0	1	15	2	0	0	0
EABT36150	2	0	0	4	1	0	0
EABT36151	1	7	4	30	11	3	4
EABT36152	1	1	1	5	7	0	2
EABT36153	169	132	85	17	26	0	0
EABT36154	0	0	2	4	1	0	0
EABT36155	5	1	42	0	6	5	3
EABT36156	1187.99	1802.8	5375.48	6463.03	1683.96	1838.25	2005.81
EABT36157	1	3	33	0	2	3	4
EABT36158	0	2	5	4	0	0	1
EABT36159	19	15	73	79	19	2	9

EABT3616	4	0	0	0	0	8	0
EABT36160	13.03	40.01	0	1	0	0	0
EABT36161	1	0	10	2	0	0	0
EABT36162	1	0	23	0	0	2	0
EABT36163	508	887.88	598.12	286	680	866.64	1655.2
EABT36164	2	1	0	3	1	0	0
EABT36165	8008.98	11443	17257.5	42288.1	43593.1	269	582
EABT36166	2544.67	5109.19	1552	3197.08	1688.99	1901.05	1663.01
EABT36167	0	0	17	0	1	0	0
EABT36168	1	3	10	2	8	2	10
EABT36169	1	0	8	0	0	0	0
EABT3617	13	36	78	72.79	7	31	27
EABT36170	2	1	3	39	10	16	52.21
EABT36171	71	49.89	256.7	411	92	15	6
EABT36172	0	1	2	4	10	0	19
EABT36173	0	0	6	0	0	0	0
EABT36174	6024.84	7204.91	6263.93	5849.87	2883.34	1113.84	999.34
EABT36175	0	0	0	0	0	0	0
EABT36176	208.99	501.47	795.69	1251.47	310.13	53.51	111
EABT36177	46.01	24	8	16	51	50	2
EABT36178	95.18	245.31	2546.19	186.92	51.68	177.64	52.98
EABT36179	1	4	0	3	1	0	1
EABT3618	3	22	234	56	6	2	1
EABT36180	0	0	6	1	0	0	0
EABT36181	0	0	4	1	39	0	0
EABT36182	0	1	4	0	0	0	1
EABT36183	0	0	9	2	2	0	0
EABT36184	1	1	3	5	5	0	2
EABT36185	0	0	7	2	0	1	1
EABT36186	5	3	4	8	7	1	1
EABT36187	12	25	91	20	5	18	6
EABT36188	2	8	40	20	3	1	4
EABT36189	0	3	1	0	0	1	1
EABT3619	26	30	0	0	2	0	3
EABT36190	0	1	11	33	2	0	1
EABT36191	0	2	2	3	1	0	0
EABT36192	3	3	0	9	2	1	0
EABT36193	1	0	3	2	0	0	0
EABT36194	0	1	12	3	0	0	0
EABT36195	98.03	187	301.38	943.37	223	77.98	125
EABT36196	1	1	39	0	0	0	0
EABT36197	1	3	8	18	0	0	1
EABT36198	4	13	31	59	2	0	0
EABT36199	14281.3	13692.7	12189.7	7118.24	5577.65	8931.01	4132.46
EABT362	2	2	11	1	0	0	0
EABT3620	3	3	6	1	1	0	0
EABT36200	31	0	6	0	1	32	24.19
EABT36201	1	1	3	11	1	0	0
EABT36202	2	3	30	1	0	3	1
EABT36203	0	2	5	0	0	0	0
EABT36204	0	0	18	10	12	0	0

EABT36205	0	1	137	3	2	0	0
EABT36206	3	6	17	8	0	0	0
EABT36207	2	2	11	2	0	0	0
EABT36208	9547.13	15643.1	4471.52	7892.66	3098.23	5039.51	3328.89
EABT36209	1	2	1	2	0	0	1
EABT3621	0	0	4	6	1	0	0
EABT36210	353.99	654.01	535.99	2049.74	413	339	502
EABT36211	7	20	4	1	3	3	2
EABT36212	3	3	0	0	0	1	4
EABT36213	2	5	12	8	5	2	5
EABT36214	0	2	4	3	1	0	0
EABT36215	0	0	18	1	1	0	0
EABT36216	1	0	7	2	1	0	0
EABT36217	0	1	3	6	0	0	1
EABT36218	0	2	8	6	2	0	0
EABT36219	1	8	10	3	3	2	0
EABT3622	304	114	18	154.01	43	168.6	7
EABT36220	2	3	8	5	3	2	0
EABT36221	942	1369	948	1982	977	304.99	516
EABT36222	4957.55	7289	7648.07	10932.5	4057.83	3772.54	3379.95
EABT36223	0	0	6	0	0	0	1
EABT36224	2	3.05	4	2	4	1	7
EABT36225	0	1	3	2	2	0	2
EABT36226	2	8	0	2	3	1	11
EABT36227	4555.46	7650.02	6704.04	15230.9	4002.35	3217.96	2829.57
EABT36228	1	0	8	0	0	0	2
EABT36229	9	8	0	12	6	0	0
EABT3623	2	6	8	2	0	0	0
EABT36230	1	2	1	0	0	0	0
EABT36231	1	3	42	2	0	2	4
EABT36232	1238	310	85	48	348	465	1107.25
EABT36233	2	4	30	2	1	0	0
EABT36234	0	6	1	9	9.99	1	1
EABT36235	0	0	3	7	0	0	0
EABT36236	0	0	26	0	0	0	0
EABT36237	10	16	7	107	26	3	61
EABT36238	2129.99	3922.24	4919.12	2562.3	1214.01	900	1680.71
EABT36239	1	0	10	1	0	0	3
EABT3624	0	1	0	0	1	0	0
EABT36240	14	49	17	23	8.81	3	2
EABT36241	23	75	181	410	317	22	268.08
EABT36242	139.2	464.96	148.96	457.2	28	364	17
EABT36243	5114.69	8664.95	9248.46	4563.82	7302.12	1421	1871.66
EABT36244	10	21	128.08	28.55	24	7	14
EABT36245	1300	1954.99	1192	3643.12	1077	1525.5	1402.04
EABT36246	1	0	10.01	0	0	0	0
EABT36247	1	0	3	0	0	2	1
EABT36248	4	17	25	156	23	7	27
EABT36249	0	12	19	121	3	2	7
EABT3625	1	5	0	0	0	8	0
EABT36250	18487.1	18243.8	10947.2	15896.4	17476.5	20075	21700.2

EABT36251	10	6	1	2	1	2	1
EABT36252	1	1	6	2	3	0	0
EABT36253	0	0	2	0	1	9	1
EABT36254	2	6	1	4	0	0	0
EABT36255	1	2	47	4	1	2	0
EABT36256	0	1	6	0	0	0	0
EABT36257	5	8	14	41	41	2	8
EABT36258	7	7	6	4	3	0	3
EABT36259	57	275	10	1	119	160	430.01
EABT3626	2	2	6	6	1	5	3
EABT36260	6	7	66	7	0	2	2
EABT36261	0	1	10	0	1	0	0
EABT36262	0	1	7	0	0	0	0
EABT36263	0	0	6	0	0	0	0
EABT36264	1	6.96	4	6	9	0	2
EABT36265	2	5	5	0	2	1	1
EABT36266	23	2	4	1	0	14	1
EABT36267	3	3	101.58	6	2	1	0
EABT36268	0	1	9	1	0	1	1
EABT36269	4	6	14	22	2	0	2
EABT3627	1	0	3	0	0	0	0
EABT36270	0	3	5	48	9	1	10
EABT36271	198.99	206.1	153.02	255	255	204.41	333.87
EABT36272	57	38	17	5	6	36	48
EABT36273	23	42	36	60	15	4	15
EABT36274	0	1	85	0	1	0	0
EABT36275	0	2	3	7	0	0	0
EABT36276	0	2	4	0	0	0	0
EABT36277	0	1	0	0	0	0	0
EABT36278	1	8	9	4	0	0	2
EABT36279	0	1	3	0	0	1	1
EABT3628	754	745.13	875.83	1167	485.99	1060.12	634
EABT36280	2	1	19	9	0	0	1
EABT36281	1	0	14	1	0	0	0
EABT36282	103	58	5	4	4	68	34
EABT36283	1	2	45	1	0	4	0
EABT36284	0	1	12	0	0	0	0
EABT36285	2	0	20	1	1	0	1
EABT36286	0	1	2	8	1	0	0
EABT36287	0	0	8	3	0	0	0
EABT36288	1	1	1	2	0	0	0
EABT36289	4	6	0	10	17	1	1
EABT3629	2	5.54	15	3	1	11	2
EABT36290	2	1	5	1	0	0	0
EABT36291	1	1	5	4	3	1	0
EABT36292	2465.14	2701	819.88	1257.82	2755.53	2049.16	5459.64
EABT36293	11	2	2	0	0	0	1
EABT36294	104	200	713.26	1330.92	449	102	64
EABT36295	358	596.96	409.99	1350.16	499.01	262	303
EABT36296	2	1	1	0	1	0	4
EABT36297	1005.55	1476.14	2478.46	3836.51	1466.57	599.94	595.79

EABT36298	2	8	0	0	0	3	3
EABT36299	0	0	8	0	0	0	0
EABT363	0	1	6	1	0	0	0
EABT3630	112	299	212	2080.52	1201.13	56	49
EABT36300	0	0	4	0	1	1	0
EABT36301	0	12	56	5	1	0	0
EABT36302	0	0	2	2	0	1	0
EABT36303	6	11	5	2	1	1	0
EABT36304	1	1	16	0	2	0	0
EABT36305	1751.97	2343.03	2336.7	1904.96	1625.16	2352.03	2262.95
EABT36306	0	0	9	1	0	0	0
EABT36307	0	1	0	0	0	8	1
EABT36308	22	106	116.5	236.99	23.99	9	12
EABT36309	1	2	0	1	1	0	0
EABT3631	0	0	16	7	1	1	0
EABT36310	1	6	5	78	2	0	0
EABT36311	2	2	0	0	3	5	12
EABT36312	0	0	3	2	0	0	0
EABT36313	0	0	3	0	0	0	0
EABT36314	0	1	16	0	0	0	0
EABT36315	0	0	43	2	0	0	2
EABT36316	0	1	6	0	2	0	1
EABT36317	0	0	8	0	0	0	0
EABT36318	1245.8	1880.99	4554.95	1069.91	753.22	2505.7	1384.92
EABT36319	0	2	18	1	0	2	0
EABT3632	0	1	7	3	0	0	1
EABT36320	1	47	3	0	1	5	7
EABT36321	5	8	1	5	3	0	0
EABT36322	2	3	14	8	10	0	10
EABT36323	1	4	7	0	0	0	1
EABT36324	2	8	6	8	0	3	4
EABT36325	2	3	6	14	3	1	1
EABT36326	0	0	10	0	0	0	0
EABT36327	1	1	4	1	2	1	3
EABT36328	48	61	0	0	0	14	4
EABT36329	2	1	2	0	0	9	2
EABT3633	0	0	2	0	3	1	1
EABT36330	0	5	6	6	1	0	0
EABT36331	0	0	17	4	0	0	0
EABT36332	0	1	7	1	0	0	0
EABT36333	6	14	36	766	41.96	3	35
EABT36334	2	7	2	2	1	0	0
EABT36335	3159.92	5576.5	1452.04	9782.91	4475.86	4270.04	4178.59
EABT36336	3	4	5	12	16	0	9
EABT36337	0	0	1	12	0	0	0
EABT36338	1	8	4	4	0	1	1
EABT36339	1	0	11	4	3	0	1
EABT3634	0	1	0	5	3	0	0
EABT36340	975	1351.95	734.42	2288.1	686.95	808.89	677
EABT36341	14	29	35	21	20	8	14
EABT36342	1	4	5	8	7	0	1

EABT36343	0	3	5	2	0	2	0
EABT36344	0	0	1	23	3	0	0
EABT36345	0	0	5	1	0	0	0
EABT36346	1	7	2	1	1	3	1
EABT36347	5	19	55	15	0	6	4
EABT36348	1	2	85	2	0	0	2
EABT36349	0	0	10	1	0	0	0
EABT3635	5	7	8	30	9	1	11
EABT36350	0	0	6	0	0	0	0
EABT36351	1	2	0	2	0	1	0
EABT36352	0	1	9	1	0	0	0
EABT36353	2	3	7	7	1	1	0
EABT36354	3	11	42	37	7	0	3
EABT36355	427	1056.01	633.46	528	976	945.99	2095.35
EABT36356	20	1	4	14	10	0	1
EABT36357	1	7	10	8	2	0	0
EABT36358	909.17	1085.19	927.85	1276.7	1023.14	618.25	615.54
EABT36359	10	22.65	4	104	27.05	12	50
EABT3636	15	10	0	0	1	1	2
EABT36360	0	0	17	1	0	0	0
EABT36361	0	0	8	2	0	2	0
EABT36362	2	6	16	13	1	1	2
EABT36363	0	1	4	6	0	0	0
EABT36364	48	15	4	2	0	106	0
EABT36365	1	0	5	1	0	0	0
EABT36366	3	10	259	2	1	6	4
EABT36367	0	0	8	0	0	1	0
EABT36368	0	2	13	1	0	1	4
EABT36369	432.94	969.81	1149	4269.42	1793.31	150	1442.33
EABT3637	0	0	2	3	0	0	2
EABT36370	6	7	0	0	0	5	3
EABT36371	0	7	2	0	0	1	0
EABT36372	12	16	34	14	4	1	4
EABT36373	4	2	3	14	1	0	1
EABT36374	13	3	0	12	8	0	3
EABT36375	5	0	0	0	0	0	0
EABT36376	6	7	67	27.06	22	0	0
EABT36377	1	2	1	3	13	0	0
EABT36378	1	8	7	7	8	0	0
EABT36379	0	0	0	1	1	0	2
EABT3638	90	163	426.27	193.34	104	84	105
EABT36380	0	1	2	1	3	0	0
EABT36381	0	1	14	1	0	0	0
EABT36382	1	6	5	0	0	0	0
EABT36383	8	2	4	1	0	3	2
EABT36384	3	2	2	540	4232.57	0	130
EABT36385	1	1	3	0	0	0	0
EABT36386	0	1	1	0	0	1	0
EABT36387	1	0	2	1	0	1	0
EABT36388	13	31	38	227	18	0	1
EABT36389	1	1	3	0	1	1	0

EABT3639	92	151.14	299.85	316.97	253	93	105
EABT36390	0	4	84	1	2	1	2
EABT36391	5	5	9	5	0	1	3
EABT36392	1	2	45	1	1	0	0
EABT36393	1	0	2	0	15	1	27
EABT36394	1	0	7	1	0	0	0
EABT36395	6	10	25	19	8	3	5
EABT36396	0	0	11	0	0	0	0
EABT36397	4191.36	6445.27	3625.23	7411.08	3127.18	2374.42	2918.84
EABT36398	2	17	10	5	33	1	134
EABT36399	0	0	1	0	6	0	0
EABT364	2463.34	2232.21	677	1064.99	966.95	582	469.99
EABT3640	15	19	24	34	56	2	19
EABT36400	883.88	1868.72	800.35	2651.32	998.26	659.99	1169.04
EABT36401	1	0	2	0	0	0	2
EABT36402	1	5	8.73	4	0	1	0
EABT36403	0	1	3	1	0	1	0
EABT36404	0	2	10	15	7	0	6
EABT36405	586.52	868.58	641.02	1329.64	655.52	471.28	590
EABT36406	1062	1057.98	695.55	1354	482	655.37	582
EABT36407	0	3	24	0	1	6	2
EABT36408	1	7	10	13	1	1	1
EABT36409	202	307	126	2194	736	52	192
EABT3641	1	1	25	1	0	2	1
EABT36410	5	15	55	51	7	2	5
EABT36411	1	2	11	2	0	0	0
EABT36412	2	18	45	337.01	52	5	1
EABT36413	0	0	5	4	0	0	0
EABT36414	1	4	16	2	3	7	0
EABT36415	0	1	3	1	2	1	1
EABT36416	7	2	0	0	1	1	2
EABT36417	4	9	23	3	0	2	6
EABT36418	3159.12	6909.67	8986.77	4778.83	1721	1396	1424.89
EABT36419	2	6	12	3	9	3	1
EABT3642	30	27	0	0	2	30	16
EABT36420	0	0	5	1	1	0	0
EABT36421	1	0	15	5	2	10	0
EABT36422	0	0	8	0	0	0	0
EABT36423	0	3	0	0	0	0	0
EABT36424	2	2	3	2	0	1	0
EABT36425	12	25	85	53	6	2	0
EABT36426	3	4	7	22	2	3	0
EABT36427	1	0	8	1	0	1	0
EABT36428	3	9	39	25	0	3	1
EABT36429	3	10	58	8	2	4	3
EABT3643	0	0	3	3	0	2	0
EABT36430	0	0	1	2	0	0	1
EABT36431	0	4	1	4	0	0	1
EABT36432	2184.85	17767.6	4922.16	11737.8	11772.7	3999.68	2391.89
EABT36433	0	10	6	15	0	1	1
EABT36434	1	2	11	5	0	0	0

EABT36435	0	0	2	0	0	0	0
EABT36436	29	2	0	0	1	48	1
EABT36437	3628.14	5252.15	3851.33	4271.58	2294.82	5771.8	5026.72
EABT36438	198.01	303.08	240.01	559.74	217.01	126.5	134.1
EABT36439	1	9	5	5	4	1	1
EABT3644	742.15	894.92	1503	1147.04	735.61	513.01	493
EABT36440	0	2	1	0	0	0	0
EABT36441	53654.9	5649.1	13	2	3025.46	53331.7	25657.5
EABT36442	0	0	1	4	1	0	0
EABT36443	0	1	1	0	0	0	0
EABT36444	0	0	0	10	2	0	0
EABT36445	0	0	5	0	0	0	0
EABT36446	1	2	9	1	3	0	0
EABT36447	951.97	1601.01	850.01	1780.65	1057.97	528	1192.98
EABT36448	4	3	35	2	0	0	0
EABT36449	0	1	0	7	0	0	0
EABT3645	1	4	32	2	2	2	0
EABT36450	0	3	15	0	0	0	0
EABT36451	794.94	9045.25	12332.2	2556.44	2313.88	74.86	336.57
EABT36452	3	8	13	1	0	0	2
EABT36453	0	0	14	1	0	0	0
EABT36454	3	6	36	4	1	3	2
EABT36455	0	5	25	3	2	1	0
EABT36456	0	0	2	0	0	0	0
EABT36457	2	4	11	5	0	1	1
EABT36458	0	2	0	4	0	0	0
EABT36459	0	0	14	0	1	0	0
EABT3646	1007	1182.49	1165.92	1115.1	2370.63	615	731.28
EABT36460	0	0	0	0	1	0	0
EABT36461	0	0	5	0	0	12	1
EABT36462	2	24	0	0	0	2	0
EABT36463	7	10	58	66	4	7	15
EABT36464	126.09	185	199	492	219	28	123
EABT36465	1	2	53	2	1	0	2
EABT36466	0	0	4	0	0	0	0
EABT36467	0	2	23	10	3	0	11
EABT36468	0	0	9	0	1	0	0
EABT36469	7	8	0	1	2	1	1
EABT3647	0	4	32	0	0	1	0
EABT36470	2	1	0	0	0	0	2
EABT36471	0	7	24	16	1	2	3
EABT36472	4	4	4	1	2	5	11.6
EABT36473	3069.64	4558.94	2621.53	7989.16	1540.83	1924.99	2364.36
EABT36474	3785.88	6322.23	6249.5	16891.6	2269.84	4826.73	4465.61
EABT36475	1	3	1	0	0	0	0
EABT36476	2	6	14.95	26	1	4	0
EABT36477	4	17.04	28	7.97	0	1	4
EABT36478	1342.05	1391.99	809	1335.86	903.83	949	644
EABT36479	0	2	17	0	0	0	0
EABT3648	2	1	6	9	2	2	1
EABT36480	16	25	36	0	3	3	7

EABT36481	0	0	12	2	1	0	0
EABT36482	4	8	13	0	5	1	0
EABT36483	0	1	6	1	1	2	1
EABT36484	0	0	22	0	0	0	0
EABT36485	0	1	0	4	0	1	0
EABT36486	1	2	61	1	0	1	0
EABT36487	0	0	31	0	0	0	0
EABT36488	0	1	4	1	1	1	0
EABT36489	0	0	0	0	7	0	0
EABT3649	2012.19	1653.29	635.7	1717.78	1899.3	2095.7	2685.21
EABT36490	413.72	520.98	589.23	864.03	428.22	291.13	155.87
EABT36491	2	9	6	1	2	16	6
EABT36492	4	1	2	1	1	2	0
EABT36493	151	241	76	381.39	152	95	79
EABT36494	0	2	1	2	0	0	2
EABT36495	1202.26	1685.25	1767.9	2027.94	1540.7	777.95	1028
EABT36496	182	350.93	282	1045.98	226	142	209
EABT36497	0	1	3	2	7	0	0
EABT36498	348.03	550.43	758.85	898.73	555.98	324.82	320.98
EABT36499	92.03	203	205	352.23	137	124.43	132.03
EABT365	51	64	14	7	13	12	20
EABT3650	18	89	133	101.97	81	26	30
EABT36500	2832.2	1422.06	606.39	2553.94	1509.55	91	506.26
EABT36501	3759.51	2769.07	528.93	192.13	1627.41	1302.02	2258.75
EABT36502	755.39	946.57	1452.61	1859.33	715	629.73	576.94
EABT36503	2	6	15	21	2	0	2
EABT36504	0	0	5	1	0	2	0
EABT36505	1174	1835.78	1487.02	4694.7	1414	624	671
EABT36506	0	5	4	7	2	0	3
EABT36507	1	0	6	0	0	0	1
EABT36508	0	1	9	0	0	0	0
EABT36509	1	2	20	2	0	0	1
EABT3651	1	7	6	15	28	1	2
EABT36510	434.63	370.99	509.98	842.18	419	357	406.31
EABT36511	0	0	3	11	0	0	1
EABT36512	4	1	18	1	0	10	0
EABT36513	4	3	63	9	0	0	0
EABT36514	0	1	14	3	0	0	0
EABT36515	246	424.23	764	275	104	30	55
EABT36516	5	6	1	0	0	3	2
EABT36517	6239.73	7809.62	5865.01	3953.26	4695.29	7713.13	7528.87
EABT36518	1252	2249	132	41	203	3885.04	144
EABT36519	1	1	1	0	1	0	2
EABT3652	0	0	4	0	0	0	0
EABT36520	1	3	6	2	5	7.04	64
EABT36521	158	306.41	317.99	580.09	59	81	181
EABT36522	1215.85	1647.32	1684	336.98	2306.07	839.6	3357.57
EABT36523	5	1	6	2	0	0	1
EABT36524	1	7	21	1	5	0	3
EABT36525	68	138	580.01	941	429	26	56
EABT36526	2	0	27	2	1	0	0

EABT36527	5	3	1	8	4	3	2
EABT36528	3894.47	3804.66	2107	2286.65	5650.79	2593.07	2242.95
EABT36529	1	0	5	0	0	0	0
EABT3653	33.35	36	17.92	42.43	35	15	44
EABT36530	14	37	20	11	11	0	0
EABT36531	0	2	3	0	1	1	4
EABT36532	0	0	5	0	0	0	0
EABT36533	1	2	2	5	2	1	10
EABT36534	0	0	5	0	0	0	0
EABT36535	1	12	6	6	1	8	42
EABT36536	1	3	12	12	10	0	0
EABT36537	2	16	27	66	32	0	7
EABT36538	97	109	42	9	13	0	15
EABT36539	28	77.85	74.58	164	77.42	6	10
EABT3654	0	1	0	1	0	3	0
EABT36540	3	4	14	23	3	3	3
EABT36541	7	5	2	32	14	1	3
EABT36542	0	0	4	9	0	0	0
EABT36543	0	0	12	1	0	0	1
EABT36544	3	3	87	40	0	1	0
EABT36545	1	2	3	0	0	0	0
EABT36546	2	11	11	14	2	2	1
EABT36547	1	2	1	31	0	0	0
EABT36548	198.98	263.04	567.37	929.98	476.07	159	77.03
EABT36549	4598.68	8206.39	7296.89	6288.39	3136.22	4578.49	2451.88
EABT3655	763.12	1125.75	652.33	1154.91	1004.23	378.72	588
EABT36550	26	15	20	8.02	2	33	15
EABT36551	1	0	2	1	0	0	0
EABT36552	0	0	13	0	0	0	0
EABT36553	11	21	4	3	1	6	7
EABT36554	0	4	12	3	0	0	2
EABT36555	0	1	3	0	0	0	2
EABT36556	6	4	5	24	2	0	2
EABT36557	2	1	1	1	0	4	0
EABT36558	9	6	4	3	0	7	0
EABT36559	9	65	5	1	88	0	0
EABT3656	0	1	10	0	0	1	0
EABT36560	0	3	7	18	2	1	0
EABT36561	0	2	3	1	0	0	0
EABT36562	1471.04	3319.75	2995.79	6182.85	2861.85	538.74	1590.14
EABT36563	1	4	5	9	2	2	2
EABT36564	1	1	2	4	0	0	1
EABT36565	1	2	9	2	0	0	0
EABT36566	0	3	5	2	1	1	2
EABT36567	0	0	4	2	0	1	0
EABT36568	1	0	7	15	0	0	0
EABT36569	3	2	25	0	2	3	0
EABT3657	1	1	10	1	2	0	0
EABT36570	1	8	0	14	3	0	0
EABT36571	1391.54	2297.08	1686.92	3026.58	1200.89	1218.02	1250.1
EABT36572	0	0	3	0	0	0	0

EABT36573	0	0	5	2	1	0	1
EABT36574	1416.94	1861.92	987.65	2203.05	1167.99	1285.2	1035.36
EABT36575	274	916	2391.19	2473.74	652.24	120	136
EABT36576	0	0	15	0	0	2	2
EABT36577	0	0	6	0	0	0	0
EABT36578	3	5	13	2.07	1	0	1
EABT36579	1	1	16	0	0	1	0
EABT3658	813.02	1681.25	834.8	2389.18	577	671	765
EABT36580	3	5	3	3	1	0	0
EABT36581	0	4	4	1	0	0	0
EABT36582	1	0	0	2	3	2	3
EABT36583	272	392.58	340	598.62	493.13	222	212
EABT36584	0	0	13	2	0	0	0
EABT36585	0	0	7	0	0	0	1
EABT36586	0	1	2	1	2	1	1
EABT36587	1	0	0	27	0	0	0
EABT36588	2	5	6	2	1	2	0
EABT36589	0	4	16	0	5	0	6
EABT3659	1062.78	1553.51	2226.97	3092.23	1129.03	702.99	978.65
EABT36590	6191.01	11113.7	16393.2	65666.1	5599.64	2521.84	1366
EABT36591	30	25	0	0	0	4.87	6
EABT36592	105	193	91	278.89	103	48	124.12
EABT36593	21	8	0	0	0	3	2
EABT36594	0	2	4	2	0	0	1
EABT36595	1572.76	3528.6	3890.45	5679.11	2022.08	1597.92	1861.94
EABT36596	8	55	20	12	2	12	0
EABT36597	141	629.03	828.25	1021.69	258.51	117	295.36
EABT36598	3	17	8	2	1	5	2
EABT36599	4196.07	3669.47	960.77	1021.95	491	16	14
EABT366	2	1	5	1	1	0	2
EABT3660	4	10	155.98	9	5	1	4
EABT36600	6	8	0	7	0	5	2
EABT36601	0	0	1	5	0	2	1
EABT36602	56	122	4	7	26.04	0	0
EABT36603	3	8	5	8	0	1	5
EABT36604	0	0	2	0	3	1	4
EABT36605	1	1	20	6	5	0	1
EABT36606	0	1	0	4	39	0	4
EABT36607	2	1	3	0	0	0	0
EABT36608	22	62	113	716	25	11	33
EABT36609	229.9	245	147.99	288	285.97	85	82
EABT3661	183	845.53	27	66	107.67	16	314
EABT36610	0	0	1	1	2	0	6
EABT36611	2	6	15	9	2	1	0
EABT36612	3106.41	12498.1	31989.5	62466.1	16257.6	1940.64	3524.33
EABT36613	77	86	8	16	35	3	28
EABT36614	0	0	19	2	0	0	1
EABT36615	1072	1602.78	967.99	1504.79	1227.09	1072.43	1312.54
EABT36616	0	3	34	3	0	0	0
EABT36617	1	4	4	1	0	0	0
EABT36618	2	6	83	6	8	0	0

EABT36619	0	0	1	2	1	0	0
EABT3662	1	4	4	42	5	1	0
EABT36620	15	6	17	15	5	2	3
EABT36621	0	3	0	2	0	4	0
EABT36622	0	0	17	0	0	0	0
EABT36623	0	0	10	0	0	1	0
EABT36624	6	17	6	11	5	0	0
EABT36625	1	0	0	0	0	1	2
EABT36626	3	3	6	2	1	0	0
EABT36627	9	12	4	19	0	14	11
EABT36628	3	2	0	0	1	0	4
EABT36629	0	1	8	0	1	0	0
EABT3663	1361	6029.98	1521	563	1212	26	50
EABT36630	0	0	3	0	6	0	1
EABT36631	1	2	2	3	0	0	1
EABT36632	3	16	32	29	7	3	2
EABT36633	15	3	1	1	0	0	0
EABT36634	1	1	0	3	0	2	0
EABT36635	6	8	16	12	1	0	0
EABT36636	339	599	421	1389.07	571	222	272.01
EABT36637	1	0	12	2	0	1	0
EABT36638	2137.32	4288.78	7559.47	9182.78	2503	1783	1517
EABT36639	4	11	26.04	18	4	17	0
EABT3664	0	1	5	2	0	1	0
EABT36640	1	4	17	7	0	1	2
EABT36641	2	10	26	3	2	30	18
EABT36642	0	0	10	1	0	0	1
EABT36643	3	3	9	3	0	1	1
EABT36644	0	0	5	0	0	0	1
EABT36645	1	3	41	0	0	2	0
EABT36646	2	6	3	7	2	0	0
EABT36647	556.02	933.43	469.14	1185.49	771.96	145.02	359.23
EABT36648	12	13	3	10	4	0	1
EABT36649	0	0	18	1	0	1	0
EABT3665	0	0	7	1	0	1	0
EABT36650	0	3	2	2	1	0	0
EABT36651	18	74	36	50	189	1	13
EABT36652	0	2	4	2	0	1	1
EABT36653	8	24	23	60	3	4	8
EABT36654	2	14	8	5	13	9	37
EABT36655	1	1	9	5	0	0	0
EABT36656	123	152	186.95	829	297	114	106
EABT36657	11	9	5	16	3	3	7
EABT36658	1	1	2	1	0	1	0
EABT36659	115	615.09	999.07	882.61	871.44	62.09	162
EABT3666	0	2	3	4	0	0	0
EABT36660	4	22	81	17	2	2	0
EABT36661	10	38	41	21	6	2	7
EABT36662	2	1	0	0	0	3	3
EABT36663	0	0	2	1	0	0	0
EABT36664	0	1	2	1	0	2	2

EABT36665	0	0	5	9	0	1	1
EABT36666	0	3	10	3	1	2	1
EABT36667	13	2	1	0	0	0	0
EABT36668	2	3	1	0	0	2	1
EABT36669	19	26	68.01	5	34	0	6
EABT3667	0	0	0	0	3	0	0
EABT36670	1293.01	2887	3039.99	8384.31	2253.81	1793	1928.74
EABT36671	3146.91	3989	2908.83	4135.05	1940.98	2945	2655
EABT36672	716.36	1378.53	1421.78	2646.94	760	627.01	545.38
EABT36673	19	24	96	26	15	5	10
EABT36674	5	8	2	0	0	3	0
EABT36675	14.06	37	117	723.95	34.02	22	148
EABT36676	0	1	0	0	0	8	5
EABT36677	2	4	14	4	8	2	3
EABT36678	2	2	6	0	1	0	1
EABT36679	924	1931	2016.63	4363.03	1003	1230	952.23
EABT3668	158	189.96	171	362	126	56	83
EABT36680	2	1	21	4	2	0	0
EABT36681	9618.61	12651.7	7239.34	8296.08	1844.78	1	45
EABT36682	16	46	62	104	10	6	11
EABT36683	0	0	10	4	10	1	0
EABT36684	5	2	1	0	1	2	1
EABT36685	0	1	10	0	0	1	0
EABT36686	0	3	0	0	1	0	0
EABT36687	1	3	19	11	5	0	2
EABT36688	0	1	2	29	5	0	0
EABT36689	2	1	9	1	0	1	0
EABT3669	6	2	21	3	0	1	1
EABT36690	0	10	22	9	1	0	4
EABT36691	128	361.01	251	406	70	73	128
EABT36692	420.24	1767.38	2918	2234.46	1971.21	199	418
EABT36693	766.1	1029.86	1027.05	1637	782.4	821.99	875.22
EABT36694	0	0	0	8	0	0	0
EABT36695	565.77	1097.88	950.39	1751.67	701.72	394.65	584.86
EABT36696	11	0	0	0	0	2	4
EABT36697	0	0	1	17	0	0	0
EABT36698	2	4	145.07	0	0	0	0
EABT36699	0	1	11	4	0	0	0
EABT367	12747.2	20708.8	8179.72	17903.1	8976.21	7913.67	6521.98
EABT3670	1	1	7	0	0	0	0
EABT36700	1	1	2	0	1	0	4
EABT36701	3	7	27	10	1	0	1
EABT36702	2	4	6	0	0	1	3
EABT36703	0	0	5	1	0	0	0
EABT36704	0	1	2	0	0	3	0
EABT36705	0	0	7	0	0	0	0
EABT36706	2	6	6	39	5	3	2
EABT36707	1	12	45	1	0	2	0
EABT36708	35	50	0	2	1	7	9
EABT36709	182	316	396.03	514	205	285	236
EABT3671	28.85	10	5	1	3	27	9

EABT36710	2	1	1	9	1	0	0
EABT36711	0	4	0	0	3	14	18
EABT36712	0	3	2	0	0	0	0
EABT36713	502.13	481	193	656	165	270	219
EABT36714	37	0	6	0	0	526.24	0
EABT36715	754	996.01	1253.24	1993	888.03	1378	1361.79
EABT36716	2	6	40	6	11	2	1
EABT36717	387.11	392.01	222.02	357.87	373.6	746.15	354.07
EABT36718	2091.12	3335.99	4748	7334.8	1751	1793.5	1427
EABT36719	9842.42	10685.8	4315.51	9885.14	6052.98	90	503
EABT3672	0	5	34	12	0	0	0
EABT36720	0	6	2	4	0	5	0
EABT36721	0	0	5	6	0	0	0
EABT36722	6	30	16	2	2	0	0
EABT36723	1	7	33	3	7	5	0
EABT36724	0	0	92	2	0	0	0
EABT36725	28	24	54	564.04	19	53	56
EABT36726	0	1	5	0	0	0	1
EABT36727	0	0	3	4	3	0	2
EABT36728	0	4	19	6	1	0	1
EABT36729	1	1	29	13	0	1	0
EABT3673	0	0	5	0	0	0	0
EABT36730	12	8	0	0	0	7	3
EABT36731	0	1	9	1	0	0	1
EABT36732	0	2	2	2	0	0	0
EABT36733	1	1	4	1	2	0	2
EABT36734	37	49	111	56	11	18	20
EABT36735	9	28	26	46	25	7	12
EABT36736	1	1	70.18	1	0	1	0
EABT36737	0	1	3	4	0	0	2
EABT36738	0	0	13	1	0	0	0
EABT36739	2275.26	2796.21	772.64	1993.65	2194.61	3824	1879.32
EABT3674	634	812.07	708.67	1356.96	350	456.01	554
EABT36740	0	1	1	3	0	0	1
EABT36741	3	3	35	1	1	0	2
EABT36742	382.22	540.11	512.02	836	810.7	347.08	357.54
EABT36743	30950.3	5102.25	326	307	0	13727.4	5
EABT36744	3	0	4	0	0	0	0
EABT36745	1	6	35	13	0	1	3
EABT36746	0	1	0	0	4	0	0
EABT36747	1	2	10	1	0	1	1
EABT36748	8	7025.88	16831.1	18264.9	860.81	16.96	13061.2
EABT36749	0	1	4	0	0	1	0
EABT3675	0	5	11	1	4	0	1
EABT36750	1139	1321.54	867.87	675	740	989	970.62
EABT36751	0	9	156	3	0	0	0
EABT36752	0	0	14	1	2	2	0
EABT36753	553.19	1037.51	637.03	2525.51	299.74	202	258
EABT36754	21.89	7	6	2	2	6	0
EABT36755	0	0	3	3	0	0	1
EABT36756	0	0	0	0	0	0	0

EABT36757	0	0	4.84	1	0	0	0
EABT36758	34.26	33	53	53	54	14	25.98
EABT36759	22	43	19	3	4	13	10
EABT3676	1	8	1	6	1	0	1
EABT36760	0	0	3	1	0	0	0
EABT36761	8	8	34	30	1	1	5
EABT36762	3	3	40.97	4	0	4	0
EABT36763	1	6	11	6	1	2	5
EABT36764	5	7	29	7	4	2	3
EABT36765	7	9	28	37	12	0	5
EABT36766	0	0	15	1	0	0	0
EABT36767	479.4	527.37	827.47	671.46	464.86	474.59	511.26
EABT36768	0	0	8	0	0	1	0
EABT36769	4	26	19	59	86	0	0
EABT3677	0	0	4	1	1	0	2
EABT36770	0	4	2	3	0	0	0
EABT36771	1	0	4	3	3	1	1
EABT36772	0	0	26	2	0	0	2
EABT36773	1	1	12	1	0	0	0
EABT36774	3	5	20	23	30	1	2
EABT36775	955.58	1827.03	1701.96	4140.36	1795.58	1464.98	1348.77
EABT36776	30	55	10	177.07	71.03	5	11
EABT36777	1994.3	2864.52	3547.82	9261.64	2121.34	1141.11	1321.87
EABT36778	280	298.01	156.02	13	29	0	6
EABT36779	75	94	16	215.02	219	5	10
EABT3678	2	16	1	1	0	1	0
EABT36780	3	7	36	40	1	5	2
EABT36781	0	0	3	3	4	0	0
EABT36782	2	4	0	0	0	0	0
EABT36783	1	5	12	0	1	0	0
EABT36784	0	0	1	3	0	0	3
EABT36785	2106.75	1571.26	2975.7	694.79	1432	719.91	646.59
EABT36786	7	11.05	3	1	1	2	1
EABT36787	1	0	4	0	0	0	0
EABT36788	3	3	0	0	0	0	0
EABT36789	0	1	3	4	2	0	0
EABT3679	1	0	0	0	0	0	1
EABT36790	1	2	3	1	1	2	8
EABT36791	10	22	10	4	1	16	4
EABT36792	10	17	0	0	2	12	7
EABT36793	0	2	10	0	0	1	0
EABT36794	254.48	344.7	304.76	953.18	377.35	219.79	289.77
EABT36795	0	0	5	1	0	0	0
EABT36796	24	5	6	0	16	0	29
EABT36797	1	0	7	0	0	0	0
EABT36798	7	21	16.62	142	3	0	0
EABT36799	0	0	2	4	3	0	2
EABT368	497.27	558.92	688.22	857.02	339	408.57	411.39
EABT3680	7	9	505	4	1	1	0
EABT36800	1	2	4	1	0	0	1
EABT36801	0	0	2	1	0	0	0

EABT36802	1	11	2	0	1	7	6
EABT36803	0	0	1	5	1	0	0
EABT36804	1060.26	2129.44	1671.31	3990.45	2997.05	1449.6	1325.72
EABT36805	2	7	7	37	0	0	0
EABT36806	0	2	1	2	1	0	0
EABT36807	2	2	11	0	0	0	0
EABT36808	1052.86	1997.29	3092.55	4312.51	1199.16	1010.36	993.5
EABT36809	0	6	14	8	44	1	0
EABT3681	0	0	19	5	1	0	1
EABT36810	1222.51	2195	4709.95	5495.99	2059.56	1241.38	1539.95
EABT36811	319.81	406.38	407.4	494.08	364.93	257.45	273.11
EABT36812	562.01	447	82	440	184	188	394.76
EABT36813	0	7	6	44	0	1	4
EABT36814	2772.3	6682.97	14884.2	9223.58	2263.26	4513.15	2161.95
EABT36815	1	1	27	0	0	0	0
EABT36816	4090.01	5296.25	2375.43	4524.93	2697.37	3233.11	5374.42
EABT36817	0	1	38	1	0	0	2
EABT36818	1	0	7	0	0	0	0
EABT36819	17	12	47	19	4	5	11
EABT3682	12180	9778.69	6097.8	4138.26	4150.41	16353.1	7506.91
EABT36820	0	1	3	0	0	0	0
EABT36821	57	62.07	86.05	12	17	0	1
EABT36822	11	14.01	10	14	15	5	33
EABT36823	2	2	1	2	0	0	0
EABT36824	1401.28	1841.16	1404.16	1655.78	1196	520	990
EABT36825	0	4	1	3	1	0	2
EABT36826	512	714.93	50	394	344.06	1434	2010.18
EABT36827	0	4	18	4	1	2	0
EABT36828	0	2	3	4	1	3	0
EABT36829	0	0	0	0	1	0	0
EABT3683	0	0	4	4	1	0	0
EABT36830	0	1	5	1	0	0	0
EABT36831	2	5	3	3	1	0	0
EABT36832	0	3	3	3	0	0	0
EABT36833	959.78	1633.43	1499.38	4726.69	1537.78	815.38	1058.46
EABT36834	3244.69	7698.12	17649.6	15486	3528.95	4288.74	3893.6
EABT36835	2163.61	2839.07	1911.17	5156.58	1970.84	2006.96	1393.27
EABT36836	2647.85	3573.78	1716.77	6547.13	2579.53	2484.02	2026.28
EABT36837	0	0	16	2	0	0	1
EABT36838	0	0	8	0	0	0	0
EABT36839	1	7	19	11	1	3	3
EABT3684	839.66	1055.79	2916.83	2995.77	481.93	1016.37	400.97
EABT36840	9	2	4	0	0	4	0
EABT36841	0	2	11	2	2	1	1
EABT36842	4	1	6	3	1	0	1
EABT36843	2509.07	3749.94	3706.21	4648.65	1629.42	1756.54	2336.01
EABT36844	3	1	7	5	0	0	3
EABT36845	1561.46	1562	899	1352.11	4493.41	880	870.86
EABT36846	0	0	1	2	2	0	1
EABT36847	1004.99	817.98	426	2238.48	1713.01	674	7954.91
EABT36848	0	0	3	8	0	0	0

EABT36849	0	6	5	10	4	0	2
EABT3685	4	3	14	9	3	21	2
EABT36850	9	242	2	1	1	9	1
EABT36851	2	7	3	5	0	0	2
EABT36852	0	0	10	0	0	0	0
EABT36853	0	0	4	0	1	0	0
EABT36854	0	4	8	4	0	0	0
EABT36855	0	1	1	4	5	0	0
EABT36856	788.12	1138.96	616	1569.85	842	491	603
EABT36857	3	0	1	2	0	1	0
EABT36858	0	3	4	4	3	1	1
EABT36859	0	4	12	16	5	1	3
EABT3686	26343.9	20700.5	9011.13	8524.38	7131.94	30866	20729.1
EABT36860	1	1	8	4	0	1	1
EABT36861	23	47.99	2	4	13	0	3.12
EABT36862	1	4	7	0	0	0	0
EABT36863	9	25	23	30	16	13	11
EABT36864	0	1	0	4	2	0	0
EABT36865	2	6	0	0	1	9	5
EABT36866	8	8	2	0	0	1	0
EABT36867	4	9	111.99	6	0	10	2
EABT36868	12	35	169	6	5	11	6
EABT36869	2	4	24	0	0	0	0
EABT3687	1	0	2	0	2	1	4
EABT36870	1	1	3	1	0	0	0
EABT36871	3	1	7	1	8	12	9
EABT36872	0	0	0	1	2	0	0
EABT36873	0	2	13	14	0	3	2
EABT36874	1780.52	2306.18	1904.81	4741.39	1498.99	1903.74	1499
EABT36875	559.99	297	77	188.16	186	433	357
EABT36876	1	1	3	0	0	0	1
EABT36877	9	12.93	7	0	3	1	6
EABT36878	3	0	6	1	0	0	1
EABT36879	5	3	3	2	630.34	1	5
EABT3688	0	0	5	3	2	0	1
EABT36880	5	2	1	0	3	24	1
EABT36881	0	0	1	1	0	0	0
EABT36882	0	1	8	1	0	0	0
EABT36883	0	0	4	0	0	0	0
EABT36884	0	0	21	13303.5	2	0	0
EABT36885	1	0	17	1	2	0	0
EABT36886	1	2	0	0	2	4	3
EABT36887	0	0	6	6	29	0	1
EABT36888	2	15	7	6	0	1	0
EABT36889	3	0	6	5	0	2	1
EABT3689	1	1	3	6	2	0	0
EABT36890	0	0	0	0	2	2	7
EABT36891	0	0	7	1	0	0	0
EABT36892	36	84	173.01	273	85	32	88
EABT36893	8	12	0	0	0	8	7
EABT36894	6	2.21	0	4	5	2	1

EABT36895	54	174	101	13	10	83	33
EABT36896	366.96	456.35	690.45	802.13	360.87	425.68	400.01
EABT36897	1	3	0	0	0	0	2
EABT36898	1	4	9	15	13	0	0
EABT36899	7	26	22	29	5	16	10
EABT369	1	1	0	2	3	2	2
EABT3690	6	12	10	18	7	2	3
EABT36900	1	0	3	9	1	0	0
EABT36901	1	2	3	0	0	1	0
EABT36902	62	118	0	68	1	0	0
EABT36903	0	0	14	0	0	0	0
EABT36904	1	0	1	1	0	0	0
EABT36905	0	10	4	4	1	0	1
EABT36906	7	14	11	10	7	4	7
EABT36907	175	456	355	45	132	263	400
EABT36908	74.18	142.24	75	47.89	62	22	39.49
EABT36909	877.27	1476.38	1469.02	3411.19	2816.43	267.99	1156.86
EABT3691	1	1	5	0	0	0	0
EABT36910	45.09	152.98	36	5	4	0	0
EABT36911	300.02	510.99	633	839.18	503.61	136	276.14
EABT36912	1	16	31	5	1	5	4
EABT36913	2	1	25	11	1	0	2
EABT36914	2	4	2	0	1	0	0
EABT36915	13134	4588.16	828.11	159	1295	19355.3	5988.8
EABT36916	974.38	1588.17	2257.66	1791.48	1644.83	1081.91	1202.14
EABT36917	0	0	4	0	0	0	0
EABT36918	0	0	4	3	1	0	1
EABT36919	1124.96	2313.05	6171.92	4702.07	2262.03	1921.92	2277.83
EABT3692	1	0	0	0	0	1	2
EABT36920	0	1	8	4	1	0	0
EABT36921	0	3	6	5	1	2	2
EABT36922	102.63	194	125	265.18	247	49	85
EABT36923	13	21	99	43	100	48	59.09
EABT36924	18	34	21	32	28	3	11
EABT36925	3	1	4	0	1	0	0
EABT36926	4679.95	8576.99	7047.93	3887.87	10047.8	3859.99	3309.6
EABT36927	1	0	1	3	0	1	0
EABT36928	3171.7	5954.11	9401.22	9314.68	3822.46	2759.27	4212.58
EABT36929	377.15	735.79	2793.13	578.95	771.31	299.36	180
EABT3693	0	0	0	0	0	0	0
EABT36930	2	5	2	0	4	0	0
EABT36931	0	5	2	1	1	0	2
EABT36932	4	2	11	2	0	1	1
EABT36933	0	0	11	0	0	0	4
EABT36934	2	5	32	5	2	0	1
EABT36935	0	1	10	1	0	0	0
EABT36936	1	4	24	4	2	1	2
EABT36937	2719.61	7773.11	4342.16	2839.97	9188.13	1584.15	6647.42
EABT36938	0	0	15	1	6	0	0
EABT36939	0	0	0	10	1	0	0
EABT3694	5	4	0	0	0	0	0

EABT36940	0	3	2	5	0	1	1
EABT36941	2304.59	3266.75	2914.04	5807.27	2600.09	1923.6	1331.92
EABT36942	3	14	18	3	0	1	1
EABT36943	0	0	8	1	1	0	0
EABT36944	15	26	107	69.02	8	4	0
EABT36945	0	0	13	0	0	0	0
EABT36946	8391.1	11236	9054.06	12694.4	10769.1	6933.89	6112.26
EABT36947	1140.47	1486.11	531.61	3492.13	1673.58	1648.84	1524.08
EABT36948	3	6	5	6	0	2	0
EABT36949	2268.29	3292.6	2483.38	5492.96	2043.45	1042.41	1626.92
EABT3695	1	1	8	1	0	0	0
EABT36950	2	3	7	2	0	0	1
EABT36951	9	20	28	19	7	7	12
EABT36952	0	1	8	1	0	0	0
EABT36953	0	0	1	3	1	0	1
EABT36954	1	3	44	1	1	0	0
EABT36955	258	246.32	204	178.23	239	304	451
EABT36956	1	13	21	27	0	2	2
EABT36957	8	7	14	0	1	1	0
EABT36958	15	35.23	12	95.92	44.17	2	1
EABT36959	0	0	4	0	0	0	0
EABT3696	2	3	2	4	4	2	11
EABT36960	1	8	3	0	0	0	3
EABT36961	18	79	14	33	32	37	25
EABT36962	18	34	132	101	8	15	15
EABT36963	0	4	3	27	3	0	0
EABT36964	101	234.04	295.63	594.89	96	158.04	94.03
EABT36965	1	0	6	0	0	0	0
EABT36966	0	1	3	2	4	1	0
EABT36967	7	12	21	50	28	2	1
EABT36968	0	2	7	5	0	0	0
EABT36969	12	45	438	2198.31	48	8	4
EABT3697	0	0	0	16	0	0	0
EABT36970	0	0	4	1	0	0	0
EABT36971	2	7	25	17	8	7	4
EABT36972	236	249.17	338	537	293	163.12	168
EABT36973	0	0	5	1	0	0	0
EABT36974	25	7	0	0	5	5	21
EABT36975	0	0	0	22	0	0	0
EABT36976	1408.05	2200	2591.68	3122.9	1255.09	1187.11	1143.89
EABT36977	3	0	22	8	0	0	1
EABT36978	452135	467822	374658	208635	332410	146281	159411
EABT36979	1	4	16	2	0	1	1
EABT3698	429.25	851.71	1260.57	646.16	938.04	780.63	684.45
EABT36980	0	3	29	6	4	0	0
EABT36981	0	0	3	0	0	0	0
EABT36982	1	14	5	7	17	0	5
EABT36983	3	2	3	0	0	2	0
EABT36984	21	14	14	3	6	7	17
EABT36985	1	1	16	1	5	0	4
EABT36986	624	901	1164.62	2056.8	547	584	601

EABT36987	0	0	8	1	0	0	0
EABT36988	2179.92	1582.1	5507.28	4679.34	3326.84	713.09	748.6
EABT36989	0	1	5	3	0	0	0
EABT3699	2	6	4	4	2	6	0
EABT36990	13	24	84	28	10	14	12
EABT36991	1	2	5	9	3	0	0
EABT36992	2468.36	4493.88	6020.37	6283.06	4608.56	3512.12	3610.25
EABT36993	1491	2194.18	2244.01	3546.59	2062.03	1269.15	1671.19
EABT36994	2	2	0	3	1	0	0
EABT36995	4	2	0	1	0	7	2
EABT36996	0	2	21	1	0	0	0
EABT36997	586.12	1034	506	2965.17	720.1	294.99	394
EABT36998	0	1	7	0	1	0	0
EABT36999	0	0	4	2	25	2	1
EABT37	0	0	2	1	1	0	3
EABT370	0	0	11	0	1	0	2
EABT3700	0	0	11	8	0	0	1
EABT37000	0	1	4	2	1	1	0
EABT37001	9	4	0	3	1	3	4
EABT37002	1	1	1	2	3	0	1
EABT37003	3	0	0	8	0	1	0
EABT37004	1386.26	2282.41	1265.12	3314.04	1107.03	927.85	869.15
EABT37005	2	8	23	16	1	2	8
EABT37006	2	1	16	2	1	5	5
EABT37007	7	39	27	76	101	2	8
EABT37008	30	29	51	86	13	10	2
EABT37009	1	2	5	1	1	0	1
EABT3701	4	2	0	0	0	4	1
EABT37010	2	2	9	4	0	0	0
EABT37011	3169.79	4444.68	3695.92	4468.17	2552.56	1537	2398.73
EABT37012	0	0	0	81	27	0	1
EABT37013	0	0	10	2	0	1	1
EABT37014	1	1	12	3	2	5	4
EABT37015	0	0	12	0	1	0	0
EABT37016	1	2	6	0	0	0	0
EABT37017	4	11	2	8	4	4	11
EABT37018	8600.25	5406.8	3656.7	3659.57	3029.07	8080.34	5601.91
EABT37019	6	6	8	1	0	1	1
EABT3702	0	2	23	0	0	0	0
EABT37020	2	2	5	4	1	0	0
EABT37021	368	595	257	88	161	841	849.76
EABT37022	3	9	5	22	37	2	18
EABT37023	26	62	84.69	29	4	1	6
EABT37024	1	1	6	1	1	0	1
EABT37025	3	1	9	0	3	0	3
EABT37026	3	5	1	2	0	0	0
EABT37027	14	10	9	17	25	10	1
EABT37028	0	4	7	0	0	1	0
EABT37029	0	1	2	0	0	3	2
EABT3703	77	104	0	0	4	11	20
EABT37030	0	6	31	1	0	0	1

EABT37031	3	8	25	1	1	1	1
EABT37032	6	12	3	12	5	12	22
EABT37033	0	1	3	4	0	1	1
EABT37034	0	1	0	1	1	0	0
EABT37035	2	0	0	0	18	0	54
EABT37036	0	1	28	0	0	0	0
EABT37037	0	2	7	4	1	0	0
EABT37038	0	0	19	0	0	0	1
EABT37039	3	5	10	0	1	0	0
EABT3704	9	11	0	0	2	27	12
EABT37040	5	2	5	0	0	1	0
EABT37041	0	0	10	1	1	1	0
EABT37042	0	0	3	0	0	0	0
EABT37043	20	4	13	0	2	26	9
EABT37044	6	12	41	10	1	0	1
EABT37045	4	7	15	12	16	0	1
EABT37046	0	1	4	1	1	0	0
EABT37047	134.01	237.64	2781.51	165.97	265.18	96	227.15
EABT37048	0	5	6	4	2	1	0
EABT37049	1	0	3	27	2	0	0
EABT3705	1	1	0	0	1	0	2
EABT37050	3934.51	6844.2	6652.73	12052.1	4919.55	7867	4606.05
EABT37051	0	1	17	0	0	0	0
EABT37052	6027.99	6070.57	2286.1	6950.69	3860.81	6420.06	6443.59
EABT37053	0	0	0	0	2	0	21
EABT37054	0	3	2	9	0	0	0
EABT37055	9	5	0	0	0	18	6
EABT37056	2	0	10	1	0	0	0
EABT37057	13.98	27	118.06	75	25	9.01	16
EABT37058	8	7	0	0	0	0	1
EABT37059	2.62	12.97	10	75	2	2	9
EABT3706	0	5	6	2	2	0	4
EABT37060	0	1	2	17	3	0	0
EABT37061	0	0	10	0	0	0	0
EABT37062	2	8	18	2	2	0	1
EABT37063	6	20	11	35	8	3	4
EABT37064	2485.23	3190.01	3046.03	3597.29	1837.18	2369.04	1375.97
EABT37065	0	0	10	20	0	0	0
EABT37066	1	5	9	2	2	1	0
EABT37067	0	2	7	3	0	0	0
EABT37068	2	2	16	70	91	2	3
EABT37069	695.84	1102.25	1143.51	1487.15	1033.94	633.49	1409.24
EABT3707	34	76	155	120	32	23	38
EABT37070	2	1	28	10	3	4	4
EABT37071	1	5	3	0	6	0	3
EABT37072	1474.9	1608.42	2201.34	2326.75	1210.66	1775.24	1314.63
EABT37073	0	3	41	0	0	0	0
EABT37074	1	3	2	13	2	0	6
EABT37075	0	1	17	0	0	1	0
EABT37076	0	19	0	0	0	5	16
EABT37077	0	3	7	6	3	2	4

EABT37078	246.78	427.56	560.04	3468.78	513.59	47.01	139.89
EABT37079	19	38	13	22	14	22	14
EABT3708	1665.21	2441.46	3102.98	7436.99	1817.12	1162.46	1033.68
EABT37080	0	0	0	0	5	1	5
EABT37081	2	0	0	0	1	1	4
EABT37082	0	1	0	1	0	2	0
EABT37083	1	0	16	1	0	1	1
EABT37084	1123.18	3170.35	253	232.99	2190.85	655.98	2131.41
EABT37085	2	2	6	2	1	1	5
EABT37086	4	0	1	0	3	2	10
EABT37087	60	35	25	73	943	1.69	8
EABT37088	0	2	4	1	2	2	0
EABT37089	2392.41	5182.47	2044.94	2173.42	3348.08	1247.7	2995.45
EABT3709	0	0	0	0	0	0	1
EABT37090	49	171.89	5	2	30	59	191
EABT37091	0	0	1	0	0	0	0
EABT37092	0	2	6	2	7	12	2
EABT37093	3	10	8	9	30	1	2
EABT37094	0	0	3	0	1	0	0
EABT37095	592	991.93	855.02	928.98	483	178	251
EABT37096	4296.93	6108.23	3300.9	3499.41	3050.94	3262.35	823.02
EABT37097	0	6	10	203	4	1	1
EABT37098	0	0	10	2	12	1	0
EABT37099	7	3	8	52	71	2	1
EABT371	0	0	1	4	0	0	0
EABT3710	1	0	13	4	0	0	1
EABT37100	57	39.99	314	115	35	25	28
EABT37101	2	1	3	2	0	2	0
EABT37102	20	30	34	83	35	6	11
EABT37103	0	3	3	0	1	0	0
EABT37104	0	3	49	14	1	11	2
EABT37105	6	17	17	140	3	1	14
EABT37106	0	0	20	0	0	0	0
EABT37107	6	3	1	0	3	5	16
EABT37108	0	0	2	1	0	0	1
EABT37109	1099	1569	2055.02	2604.88	1821.74	927	1332
EABT3711	1	0	1	1	0	0	2
EABT37110	32	220.79	151	673.89	75	9	20
EABT37111	1	3	9	7	0	0	0
EABT37112	0	2	7	1	2	1	0
EABT37113	1	3	70	2	2	1	2
EABT37114	13604.3	12317.7	12613.1	12198.9	18372.3	7352.13	8940.81
EABT37115	711	996	1245.02	2191.18	630.09	534	538
EABT37116	663	1214.18	928.98	1504.11	747	695	497
EABT37117	970.01	1461.15	1030.02	1896.36	896	734	639
EABT37118	675.71	1439.61	1785.47	4545.89	1291.77	400.84	675
EABT37119	0	0	14	0	0	1	0
EABT3712	2	3	9	4	1	1	0
EABT37120	0	1	5	0	0	0	0
EABT37121	0	4	11	8	1	0	3
EABT37122	1	2	0	3	3	0	0

EABT37123	2	10	0	0	0	1	0
EABT37124	58	40	2	1	0	98	3
EABT37125	2	8	4	6	2	0	3
EABT37126	0	0	6	4	0	0	0
EABT37127	3	6	2	8	4	3	3
EABT37128	112	183	502.92	22	264	12	53.5
EABT37129	3	1	1	0	1	5	7
EABT3713	9	8	9	4	0	10	10
EABT37130	1796.64	1752.47	1360.68	2017.59	701	783.89	761.88
EABT37131	20	20	0	0	1	30	29
EABT37132	47	237.81	536.25	1113.97	257	29	87
EABT37133	1	4	8	1	0	0	0
EABT37134	187	483	247	1019	620	130	67
EABT37135	2	1	2	6	0	0	2
EABT37136	1	1	1	4	0	1	0
EABT37137	0	2	4	1	1	1	0
EABT37138	0	0	33	2	3	0	0
EABT37139	2	3	5	3	0	1	1
EABT3714	1	1	4	2	1	0	0
EABT37140	0	4	11	17	0	0	1
EABT37141	0	0	7	0	0	0	0
EABT37142	0	1	4	0	0	0	1
EABT37143	8766.57	6198.91	24	1	76	6628.96	1823.67
EABT37144	1	1	9	28	12	2	2
EABT37145	1	0	4	0	0	0	0
EABT37146	1	1	1	6	0	0	0
EABT37147	1	2	2	2	0	1	0
EABT37148	37	68	121.03	78.61	136	53	81
EABT37149	0	2	82	0	1	1	0
EABT3715	7	19	3	37	3	1	3
EABT37150	0	0	28	1	0	0	0
EABT37151	60	75	401	302.68	11	4	9
EABT37152	0	0	5	0	0	0	0
EABT37153	36	35	379.86	673.05	865.24	26	217.99
EABT37154	6	10	17	63.99	69.78	6	6.57
EABT37155	1961.84	3538.47	4381.6	4787.73	3839.98	1184.55	2258.76
EABT37156	1	2	10	5	1	2	0
EABT37157	13	11	13	40	53	2	37
EABT37158	2	1	14	1	0	0	0
EABT37159	1	2	0	5	0	0	0
EABT3716	23	71.79	48	262.72	8	0	10
EABT37160	0	2	60	2	0	2	1
EABT37161	3	9.98	7	0	1	1	0
EABT37162	26162.4	8042.62	65	33	2924.99	21780.1	12671.2
EABT37163	2	16	54	83	23	2	1
EABT37164	9	1	8	5	0	0	0
EABT37165	1	1	5	1	0	1	0
EABT37166	1	29	11	90	62	5	3
EABT37167	2	1	45	0	0	0	1
EABT37168	1465.43	1875.45	1452.3	2654.6	1255.55	1594.33	1680.46
EABT37169	1	7	20	1	0	1	1

EABT3717	0	2	3	4	0	0	0
EABT37170	557.08	1175.17	1161.87	3913.84	784.9	564.8	429
EABT37171	7	5	20	13	5	17	9
EABT37172	2	6	2	0	0	0	1
EABT37173	0	10	5	7	5	2	0
EABT37174	0	3	14	0	0	0	1
EABT37175	12310.1	8839.29	3281.01	4583.99	2729.02	10266.3	4756.26
EABT37176	16	40	14	12	16	15	13
EABT37177	1	1	6	6	2	0	3
EABT37178	2604.36	3547.47	8005.02	36014.3	3556.78	2182.11	3263.79
EABT37179	16	40	17	4	0	0	0
EABT3718	0	3	5	76	11	0	23
EABT37180	5	5	20	3	0	3	0
EABT37181	0	1	3	0	1	0	0
EABT37182	3	10.9	8	11	2	2	0
EABT37183	1	0	0	3	1	0	2
EABT37184	3	5	5	18	251.95	0	411.26
EABT37185	0	0	2	0	0	3	0
EABT37186	0	4	9	6	1	1	0
EABT37187	0	4	8	0	0	1	1
EABT37188	1	1	4	0	0	0	0
EABT37189	0	0	18	0	0	0	0
EABT3719	4222.19	2675.02	194	225.58	1957.11	10098	9659.48
EABT37190	2985.27	3329.07	4345.12	2993.61	1411.35	572	549.99
EABT37191	0	0	3	0	0	0	0
EABT37192	99	1	0	0	0	78.15	12.98
EABT37193	22.56	33.23	20	30	8	14	4
EABT37194	0	1	1	73	0	2	0
EABT37195	1	0	16	8	0	0	0
EABT37196	223	371.3	474.86	567.98	1185.03	171.13	240
EABT37197	1	3	44	5	2	0	0
EABT37198	0	2	5	0	1	0	0
EABT37199	15	17	20	20	4	1	5
EABT372	143	207	204	665.99	211	74	95
EABT3720	1	5	10.97	0	0	0	0
EABT37200	721.37	826.37	445.1	1393	638.43	700.38	704.5
EABT37201	3	8	31	28	1	5	9
EABT37202	0	1	20	1	0	0	1
EABT37203	2	4	14	39	42.98	8	5
EABT37204	0	0	8	0	0	0	0
EABT37205	1	8	5	13	4	1	2
EABT37206	2	0	4	1	1	0	0
EABT37207	549.7	842.77	444.86	1547.46	360.34	239.86	203.99
EABT37208	3237.99	8329.93	8013.76	20	756.7	226	251
EABT37209	3	5	43	5	2	0	0
EABT3721	24	71	66	159	102	63	97
EABT37210	0	0	0	0	0	0	0
EABT37211	0	1	6	0	1	1	0
EABT37212	1	2	2	0	0	0	0
EABT37213	1	1	9	0	0	0	0
EABT37214	0	6	10	4	2	0	1

EABT37215	3434.88	3590.79	6205.57	17087.5	2173.46	45	225
EABT37216	1	0	3	0	0	0	0
EABT37217	1203.72	2358.89	3321.35	5162.85	1331.21	1116.01	1090.71
EABT37218	3	0	5	0	3	0	0
EABT37219	971.83	1419.32	893.28	2848.29	911.54	565	1043
EABT3722	0	2	0	4	0	0	0
EABT37220	10	2	5	0	0	18	14
EABT37221	0	2	7	6	1	0	4
EABT37222	1	6	7	7	4	0	6
EABT37223	3	14	19	13.02	1	3	2
EABT37224	382	559.24	621	1066.5	207.98	198	217
EABT37225	6	1	7	2	0	0	0
EABT37226	1	1	9	0	1	1	0
EABT37227	1	4	1	0	1	1	0
EABT37228	204	609.12	667.37	231	203.26	199.77	191
EABT37229	2	0	4	1	0	0	1
EABT3723	7	20	14	36	2	5	2
EABT37230	0	1	15	2	0	1	0
EABT37231	0	0	2	3	0	0	0
EABT37232	17	55	6	27	3803.05	5	19
EABT37233	0	0	4	1	0	0	0
EABT37234	159.76	226	314.65	725.84	205.38	128.05	138
EABT37235	0	17	30	64	0	0	0
EABT37236	0	3	10	1	1	1	0
EABT37237	2194.98	3548.9	4310.06	3992.16	2572.97	1960.67	2008.71
EABT37238	1	4	0	3	1	0	1
EABT37239	0	2	3	2	2	0	0
EABT3724	0	1	1	0	0	3	5
EABT37240	0	1	5	2	0	1	3
EABT37241	1584.17	1883.47	384.04	330	842.74	5	55
EABT37242	378.03	567.33	492.98	82	522.96	208	505
EABT37243	1	1	10.99	0	0	0	0
EABT37244	1	3	3	8	3	0	0
EABT37245	5	6	35	38	32	1	1
EABT37246	256	291	485	623.22	262	125.93	145
EABT37247	1	1	9	0	1	4	4
EABT37248	90	60.23	15	48	10	4	11
EABT37249	0	10	12	7	1	0	2
EABT3725	11	15	59	37	12	5	7
EABT37250	967.81	1450.07	1180.19	1917.69	924.03	575.9	1102.29
EABT37251	24	12	6	11	16	1	1
EABT37252	1	3	11	2	12	1	1
EABT37253	16	35	214	1492.87	262.24	48	39
EABT37254	1	0	0	3	1	0	0
EABT37255	1	3	2	2	0	0	0
EABT37256	28	7	1	3	6	1	1
EABT37257	0	2	1	8	0	0	0
EABT37258	0	0	1	5	0	0	0
EABT37259	0	3.96	12	0	8	14	0
EABT3726	0	0	0	4	0	0	0
EABT37260	2	1	5	0	0	0	0

EABT37261	15	9	2	1	0	7	0
EABT37262	99	50	4	16	23	90	62
EABT37263	22	72	1	1	1	84	20
EABT37264	18	85.97	13	33	14	6	38
EABT37265	2537.29	3700.61	4641.16	4276.29	2080	1565.97	1990.11
EABT37266	1	2	7	2	0	0	0
EABT37267	0	0	7	0	0	1	0
EABT37268	4931.97	14877.9	6343.85	10074.3	682.29	33	86
EABT37269	38	44	12	14	23	19	12
EABT3727	2	3	5	2	1	0	2
EABT37270	0	1	1	1	4	0	1
EABT37271	0	0	8	0	0	0	0
EABT37272	1	1	3	1	0	0	0
EABT37273	10	6	0	0	0	5	2
EABT37274	6	12	145.07	135	16	3	18
EABT37275	1	1	9	3	0	6	0
EABT37276	2	7	2	4	7	1	3
EABT37277	3	6	0	15	26.89	0	2
EABT37278	0	0	21	16	11.01	0	5
EABT37279	15	15	43	105	38	5	18
EABT3728	0	0	2	1	0	0	0
EABT37280	1	0	4	1	0	0	1
EABT37281	960	1344	1429.07	2599.14	748.83	402.38	756
EABT37282	1	1	0	2	0	0	4
EABT37283	5	4	5	3	5	11	0
EABT37284	55	60	65.3	128.14	71	23	42
EABT37285	2	1	13	5	0	0	0
EABT37286	1	2	0	3	1	0	1
EABT37287	2367.49	5041	5341.01	11803.7	2577.06	1473	1583.72
EABT37288	438.72	825.64	254.58	314.92	556.5	348.8	251.32
EABT37289	12749.1	20147.4	15619	12058.4	9948.92	9569.48	14279.6
EABT3729	1	0	6	10	0	0	0
EABT37290	10134.5	10387.8	3619	6238.99	38933	13503.3	9778.15
EABT37291	55	107	64	153	60	33	45
EABT37292	0	2	2	1	0	0	0
EABT37293	0	3	9	1	0	0	0
EABT37294	9	13	49	41	8	15	7
EABT37295	0	0	20	0	0	0	0
EABT37296	1019.99	1575.96	1459.98	2230.38	1278.23	702	857.98
EABT37297	5	7	9	10	10	8	1
EABT37298	4	12	3	7	2	4	2
EABT37299	12	4	1	1	0	0	0
EABT373	0	22	8	2	1	8	1
EABT3730	0	0	17	1	1	0	3
EABT37300	0	0	5	1	6	0	3
EABT37301	0	1	11	0	0	0	0
EABT37302	0	4	69	1	2	0	0
EABT37303	1	0	2	0	2	0	0
EABT37304	0	1	8	8	0	3	1
EABT37305	0	0	0	0	0	0	2
EABT37306	19	56	41	38	77	3	2

EABT37307	0	3	17	0	2	0	0
EABT37308	2	2	1	0	0	6	0
EABT37309	5	1	40	2	1	5	5
EABT3731	0	2	3	11	4	1	4
EABT37310	0	7	47	24	8	2	1
EABT37311	45	27	3.01	35	2	0	3
EABT37312	1	4	5	3	3	0	0
EABT37313	8	2	6	1	2	2	4
EABT37314	1	2	4	3	0	0	0
EABT37315	698498	331319	90909.6	343140	12	11	0
EABT37316	0	0	11	0	0	0	0
EABT37317	0	0	6	1	0	0	0
EABT37318	0	1	10	0	0	0	1
EABT37319	1	2	12	2	1	0	3
EABT3732	1	3	0	0	0	2	0
EABT37320	258.84	666.22	439.44	1575.09	576.33	47.28	323.89
EABT37321	1	1	2	4	1	0	0
EABT37322	2	5	8	8	1	3	1
EABT37323	4	0	2	2	0	0	3
EABT37324	19	29	60	80	80	4	270.28
EABT37325	0	4	3	5	0	0	0
EABT37326	8	9	4	13	3	3	4
EABT37327	0	1	0	2	13	0	0
EABT37328	2	1	2	1	0	0	1
EABT37329	1	2	9	2	0	1	0
EABT3733	1	1	11	1	0	0	1
EABT37330	3	2	20	1	3	1	5
EABT37331	22	32	3	0	6	41	29
EABT37332	0	0	7	12	5	0	3
EABT37333	1	1	2	0	0	0	1
EABT37334	5	11	85	40	5	5	8
EABT37335	0	0	40	0	0	0	0
EABT37336	21	33	26	61	22	7	16
EABT37337	0	4	2	0	1	0	0
EABT37338	0	3	17	32	14	0	1
EABT37339	20	29	36	14	10	16	17
EABT3734	0	0	0	0	0	0	0
EABT37340	2	2	20	1	2	8	1
EABT37341	1074.2	1307.41	927.93	1120.48	965.71	760.46	884.28
EABT37342	42	31	9	8	3	10	20
EABT37343	1	9	5	38	1	1	0
EABT37344	5	5	7	20	6	0	3
EABT37345	1413.5	1397.47	419.99	1151.76	382	1135	1374.86
EABT37346	0	1	14	0	0	0	0
EABT37347	1712.35	13150.7	796.06	697.54	1315.82	822.21	3802.47
EABT37348	1	2	3	0	0	0	0
EABT37349	28	18	23	6	12	2	6
EABT3735	0	1	9	1	0	0	1
EABT37350	18	58	123	242.88	91	30	59
EABT37351	37	0	2	0	0	54	0
EABT37352	1303.25	1873.17	1551.49	1403	1322.01	983	999

EABT37353	7	5	0	0	0	11	3
EABT37354	0	2	2	2	2	1	0
EABT37355	0	0	6	1	0	1	0
EABT37356	450.47	688.99	682	1610.21	714	804.17	446
EABT37357	0	2	16	0	0	0	0
EABT37358	0	0	8	7	0	0	0
EABT37359	87	61.97	14	16	19	11	15
EABT3736	0	0	3	0	0	0	2
EABT37360	0	0	14	4	0	0	0
EABT37361	479.83	657.88	1389.52	800.12	621.37	606.15	606
EABT37362	0	2	16	8	2	4	1
EABT37363	0	0	10	0	0	0	0
EABT37364	2	1	7	4	0	1	0
EABT37365	0	3	5	0	1	0	0
EABT37366	4	8	5	6	1	1	3
EABT37367	0	1	1	1	0	0	0
EABT37368	4	3	7	2	1	0	0
EABT37369	0	12	112	10	0	17	5
EABT3737	0	0	5	1	0	1	0
EABT37370	3	2	0	0	104	5	0
EABT37371	24623	15140.7	2461.08	5757.15	3590.5	13619.6	12998.3
EABT37372	0	0	4	2	0	0	2
EABT37373	12	11	25	3	0	4	0
EABT37374	585	1047	1005	1465.99	1115.76	988	995.07
EABT37375	10	33	25	17	29	9	25
EABT37376	1866.96	2262.75	5039.52	1251.31	2596.2	1299.01	2133.71
EABT37377	0	0	7	0	0	0	0
EABT37378	0	2	34	1	0	0	0
EABT37379	0	0	15	2	0	0	1
EABT3738	283.06	334	412.72	737.9	489.25	278	327.67
EABT37380	277.21	143.04	3	1	1107.55	232.96	5675.33
EABT37381	2	4	18	16	4	3	3
EABT37382	0	0	22	11	0	1	0
EABT37383	0	3	2	2	0	0	0
EABT37384	6	9	5	0	0	0	0
EABT37385	3090.5	7526.14	9496.38	10284.1	4712.32	4962.04	4179.7
EABT37386	7	6	6	0	1	3	7
EABT37387	4	3	0	8	4	0	4
EABT37388	5	2	2	1	34	5	19
EABT37389	0	6	26	11	0	0	1
EABT3739	9	10	16	11	0	1	2
EABT37390	0	1	7	1	1	2	0
EABT37391	0	4	3	1	1	0	5
EABT37392	1	1	9	2	0	0	0
EABT37393	0	0	1	0	0	0	1
EABT37394	1	0	4	0	1	0	1
EABT37395	2	7	133	9	1	0	1
EABT37396	0	1	5	2	1	2	0
EABT37397	0	1	2	64.01	0	1	1
EABT37398	2	2	0	6	2	0	1
EABT37399	1	1	17	1	1	1	0

EABT374	10	130	122	39	1303.01	25	27
EABT3740	2	4	15	2	0	1	0
EABT37400	2	0	3	1	0	1	1
EABT37401	23	19	4	1	1	12	13
EABT37402	17	30	54	44	361.95	13	49
EABT37403	2	4	14	1	2	2	1
EABT37404	0	3	27	0	0	0	1
EABT37405	4	8	30	28	11	0	4
EABT37406	2	1	4	2	0	0	0
EABT37407	1	4	5	4	0	0	1
EABT37408	347.95	453.79	475.96	2090.47	507.97	275.97	330.14
EABT37409	0	0	2	14	6	0	0
EABT3741	0	0	5	2	1	1	0
EABT37410	0	0	2	1	1	0	0
EABT37411	0	1	1	0	3	0	3
EABT37412	9	6	64	15	261	0	3
EABT37413	297	383	522.06	1016.41	381	338	303.96
EABT37414	1	3	16	2	0	0	0
EABT37415	0	0	0	0	0	0	2
EABT37416	2	9	29	2	3	1	5
EABT37417	0	1	2	1	0	0	0
EABT37418	0	5	14	0	0	0	0
EABT37419	63	69	134.73	189.04	120	82	87
EABT3742	494.51	876.36	528.78	1004.9	366.94	315.95	293.43
EABT37420	1762.54	3683.72	2195.59	4293.94	2597.51	1487.61	2696.51
EABT37421	2248.32	3576.19	2453.03	1134.54	1567.21	2561.04	3285.11
EABT37422	2056.64	3627.05	4189.56	10715.1	2824.37	2254.96	2395.76
EABT37423	3	6	56	10	0	1	1
EABT37424	0	1	0	1	1	0	0
EABT37425	0	0	14	1	0	0	1
EABT37426	182.06	569.47	1263.85	4910.83	629.19	116.06	108
EABT37427	0	3	44	0	0	0	0
EABT37428	0	0	3	8	6	0	0
EABT37429	2	4	12	2	0	0	0
EABT3743	0	0	1	8	1	0	0
EABT37430	0	1	14	2	0	0	0
EABT37431	1	1	14	2	0	2	0
EABT37432	1	0	14	6	0	0	3
EABT37433	0	0	3	6	0	1	0
EABT37434	1	3	3	3	3	3	0
EABT37435	0	0	5	1	0	1	0
EABT37436	4	2	6	9	0	1	5
EABT37437	603.34	851.98	734.42	1165.43	655	402	425
EABT37438	0	7	0	13	1	3	1
EABT37439	0	0	0	0	0	0	0
EABT3744	828	1352.02	3080.87	2395.8	1054.01	727.03	728.97
EABT37440	24.93	151	21.08	7	5	91	4
EABT37441	964	1172	797	1077	734	656	592
EABT37442	2	0	9	16	39	1	4
EABT37443	3828.49	4421.89	2745.96	1545	5036.52	2673.44	1950.53
EABT37444	0	1	2	5	2	0	0

EABT37445	0	0	0	0	0	8	2
EABT37446	0	1	2	2	0	0	3
EABT37447	0	2	3	1	0	2	0
EABT37448	0	2	4	0	0	7	2
EABT37449	608.95	912.64	760.25	1681.37	506.56	504.49	521.05
EABT3745	0	1	5	1	1	0	1
EABT37450	0	0	4	1	0	0	0
EABT37451	1	1	6	2	1	0	0
EABT37452	1	0	1	1	1	0	6
EABT37453	0	0	1	0	1	0	3
EABT37454	1	4	3	7	0	0	1
EABT37455	18	9	80	60	24	1	3
EABT37456	1	2	13	3	4	3	4
EABT37457	53334.8	26366.1	3010.1	1686.17	5617.07	1557.94	7547.07
EABT37458	129.02	229	64	116	168	19	187
EABT37459	84.93	162	362.72	429.89	232.31	85.86	75
EABT3746	1	1	3	28	5	0	0
EABT37460	0	0	0	0	0	1	0
EABT37461	0	1	14	10	0	0	0
EABT37462	4	13	46	20	2	5	3
EABT37463	0	2	15	9	0	0	0
EABT37464	0	1	5	0	0	1	1
EABT37465	1	1	2	1	0	2	0
EABT37466	0	4	15	13	23	1	4
EABT37467	0	0	2	1	0	0	0
EABT37468	1	2	12	11	0	1	1
EABT37469	53	191	147	82.01	17	31	13
EABT3747	1	0	3	0	0	1	1
EABT37470	11	39	1	1	0	9	0
EABT37471	6	36	55.54	34.97	2	7	0
EABT37472	25	91	35	24	16	51	9
EABT37473	21	49	21	27	54	19	23
EABT37474	255	85	5	0	188	367	1623.99
EABT37475	323	535	430	1098.97	470	337	222
EABT37476	1	3.06	0	1	0	0	1
EABT37477	3	3	14	2	1	0	1
EABT37478	1	0	14	0	1	0	0
EABT37479	14	22	9	8	6	4	2
EABT3748	310.99	548.77	472	542.8	427	111.92	382.85
EABT37480	8	15.18	15	192.02	14	1	11
EABT37481	2	1	1	13	0	0	0
EABT37482	1	9	4	5	2	0	0
EABT37483	0	1	4	6	0	0	0
EABT37484	0	2	17	2	0	0	0
EABT37485	0	0	10	2	2	0	0
EABT37486	0	0	0	0	2	0	4
EABT37487	6	6	18	123.99	6	2	3
EABT37488	0	0	2	0	0	0	0
EABT37489	0	6	2	1	0	1	0
EABT3749	0	0	2	0	4	0	0
EABT37490	0	0	7	0	0	0	0

EABT37491	7	4	32	4	1	8	5
EABT37492	0	2	3	8	0	2	2
EABT37493	531.22	916.68	1386.56	2936.38	527.74	287.83	477.34
EABT37494	1	10	14	2	10	0	14
EABT37495	1	0	5	1	1	0	0
EABT37496	152	110	68	17	39	93.99	5
EABT37497	3	0	29	1	1	0	0
EABT37498	5	6	14	17	4	4	3
EABT37499	1	2	25	5	10	5	0
EABT375	2	2	0	0	0	0	0
EABT3750	0	1	5	1	0	0	0
EABT37500	3	1	1	0	0	9	1
EABT37501	0	14	0	0	0	1	0
EABT37502	2	1	2	1	1	10	0
EABT37503	0	2	8	8	0	0	0
EABT37504	0	0	1	4	6	0	0
EABT37505	2126.19	2498.01	3451.19	4486	1750	1724	935.01
EABT37506	0	2	3	3	2	1	1
EABT37507	2	0	3	3	1	0	0
EABT37508	0	0	2	0	0	19	2
EABT37509	4	12	40	1	1	0	0
EABT3751	3	6	2	16	5	0	1
EABT37510	353.99	861.98	852.64	1654.65	1182.11	354	637
EABT37511	1	3	10	16	6	0	5
EABT37512	5	6	0	0	0	1	0
EABT37513	0	0	4	0	0	2	0
EABT37514	1	0	6	8	0	3	2
EABT37515	0	3	9	1	0	3	0
EABT37516	264	1962.22	52	8029.3	34	1	7
EABT37517	0	5	20	2	4	0	0
EABT37518	0	3	4	8	4	0	2
EABT37519	0	1	2	9	0	1	0
EABT3752	0	1	1	1	3	0	8
EABT37520	1	4	23	0	0	0	2
EABT37521	1	1	5	0	0	0	1
EABT37522	0	3	3	8	1	1	0
EABT37523	1	2	16	2	1	1	10
EABT37524	0	1	4	0	1	0	0
EABT37525	113	183	241.99	201	898	28	90
EABT37526	1	0	4	0	0	1	0
EABT37527	0	0	5	1	7	1	0
EABT37528	0	0	12	0	0	0	0
EABT37529	1	1	5	3	1	0	2
EABT3753	90	127	65.3	282.02	623.02	36	139
EABT37530	16	49.01	16	6	2	1	4
EABT37531	0	11	9	16	10	0	2
EABT37532	5	1	2	25	0	0	7
EABT37533	1	8	11	38	2	0	0
EABT37534	0	0	16	1	0	1	1
EABT37535	28	29	0	0	0	0	0
EABT37536	1	1	6	6	9	0	1

EABT37537	0	1	10	0	0	0	0
EABT37538	2	4	8	0	2	0	0
EABT37539	1	3	1	3	0	3	1
EABT3754	0	0	3	2	1	0	0
EABT37540	25	72	115	1097	24	9	25
EABT37541	2	2	5	18	3	1	2
EABT37542	1	15	51.97	8	5	2	7
EABT37543	3	10	5	11	6	10	0
EABT37544	0	1	3	3	2	0	0
EABT37545	0	0	4	1	0	0	0
EABT37546	0	0	8	0	0	0	0
EABT37547	5242.79	4368.09	1476.89	2125	1748	564.81	1042.04
EABT37548	0	0	1	1	2	0	0
EABT37549	0	0	7	2	0	0	0
EABT3755	0	6	0	0	0	1	1
EABT37550	3	1	1	0	11	32	37
EABT37551	8	11	13	9	2	4	3
EABT37552	7886.86	15997.3	9598.07	1367.54	8881.02	5172.25	16129
EABT37553	1	2	20	4	0	0	0
EABT37554	0	0	5	0	0	0	0
EABT37555	1264	1233.68	2224.67	1883.04	2099.98	1497	7672.86
EABT37556	3	13	98	34	6	2	4
EABT37557	0	0	21	5	0	0	0
EABT37558	1	1	4	2	4	0	0
EABT37559	3	8	25	14	9	2	3
EABT3756	2	8	13	5	2	1	2
EABT37560	0	1	0	9	0	0	2
EABT37561	1	2	4	1	0	1	2
EABT37562	2	3	67	4	0	6	7
EABT37563	6	5	9	8	42	3.03	896.93
EABT37564	0	0	1	0	0	1	0
EABT37565	1	12	1	0	5	1	14
EABT37566	0	0	7	5	0	1	1
EABT37567	1	1	8	3	1	0	1
EABT37568	0	2	10	0	0	1	0
EABT37569	13	20	55.16	6	15	7	13
EABT3757	13	16	65	38	1	5	0
EABT37570	23	48	17	7	6	48	30
EABT37571	1	5	5	1	1	2	2
EABT37572	105	107	0	0	4	51	52
EABT37573	12	57	6	0	1	2	1
EABT37574	0	1	3	3	1	0	0
EABT37575	2	6	19	2	0	0	1
EABT37576	4	20	15	48	13	1	0
EABT37577	4166.02	3316.27	632.2	2364.97	955.93	3813.93	2536.16
EABT37578	0	0	35	0	0	3	1
EABT37579	14	32	3	0	5	14.82	29
EABT3758	0	5	2	0	0	0	0
EABT37580	2	25.9	29	4	1	0	0
EABT37581	1	4	12	4	1	1	1
EABT37582	1	7	30	6	1	0	1

EABT37583	0	2	26	0	0	1	0
EABT37584	0	2	10	3	0	0	0
EABT37585	5	5	0	1	10	1	3
EABT37586	0	1	6	1	0	0	0
EABT37587	0	1	0	5	1	2	2
EABT37588	0	0	19	0	0	2	1
EABT37589	938.8	1658.03	1578.34	1896.05	1482	893.1	1904.8
EABT3759	6	6	2	3	3	0	2
EABT37590	0	2	10	0	0	0	0
EABT37591	1	0	4	0	0	0	1
EABT37592	0	2	17	0	4	0	0
EABT37593	0	1	6	1	0	0	1
EABT37594	2	2	5	3	1	0	0
EABT37595	116.02	331.55	244	1369.1	303.27	92	49
EABT37596	0	2	3	4	0	0	0
EABT37597	0	0	1	0	1	1	0
EABT37598	16388.9	16986.1	10465.1	12122.9	12841.1	15599.5	16703.4
EABT37599	1474.09	2085.75	2288.17	2907.02	1689	1296.06	1173
EABT376	0	6	11	14	1	0	1
EABT3760	1	3	7	2	2	0	1
EABT37600	0	0	23	2	0	1	0
EABT37601	0	2	0	9	0	0	0
EABT37602	696.26	1119.85	1170.83	2968.08	852.07	816.01	640
EABT37603	0	0	16	0	0	0	0
EABT37604	0	2	7	0	0	0	0
EABT37605	1	4	16	5	3	0	2
EABT37606	2	1	22	1	1	12	2
EABT37607	2	3	2	1	0	1	0
EABT37608	88	52	19	4	24	134	130
EABT37609	0	0	8	0	0	0	1
EABT3761	0	1	2	0	1	0	0
EABT37610	0	0	12	0	0	1	0
EABT37611	0	2	3	2	0	1	0
EABT37612	0	0	0	0	0	0	0
EABT37613	1	1	9	7	1	0	0
EABT37614	52	30	2	18	8	21	14
EABT37615	1737.57	1893	1551.4	1406.68	3009.99	1123.98	839.29
EABT37616	0	16	8	33	1	1	0
EABT37617	1	4	7	7	8	5	9
EABT37618	1	1	1	0	0	0	8
EABT37619	8	7.03	25	2	1	1	1
EABT3762	0	1	6	0	0	0	0
EABT37620	191.99	229	163	550.04	313.02	146	153
EABT37621	0	2	18	0	0	0	0
EABT37622	2	3	1	1	0	0	1
EABT37623	0	0	14	0	0	0	0
EABT37624	21	2	24.75	27	11	0	2
EABT37625	0	0	18	1	0	1	0
EABT37626	0	1	0	0	0	0	1
EABT37627	9	4	175.01	18	1	13	0
EABT37628	0	0	2	4	0	0	0

EABT37629	2	4	1	10	1	0	0
EABT3763	5	10	5	23	3	2	4
EABT37630	2	4	70	14	1	2	2
EABT37631	1	0	1	2	1	0	1
EABT37632	0	0	0	0	0	0	0
EABT37633	1728124	489106	1649898	9799.06	3	26	0
EABT37634	1910.39	2560.52	4100.24	2986.89	2662.24	1685.53	1886.07
EABT37635	998.46	1861.54	1863.24	2936.69	2197.43	286.97	612.67
EABT37636	0	2	9	0	0	0	0
EABT37637	2	4	12	4	2	1	4
EABT37638	7	8	9	2	0	7	0
EABT37639	1	0	7	2	0	1	0
EABT3764	1	0	2	1	1	0	1
EABT37640	0	0	0	31	22	4	146
EABT37641	1191.08	1559.24	1464.98	2173.93	1391.69	774.28	980.66
EABT37642	1	1	1	3	0	0	0
EABT37643	974.1	1221.99	3046.11	1988.29	1173.03	829.76	716.36
EABT37644	23	25	12	22	47	52	56.02
EABT37645	2	10	0	0	0	0	0
EABT37646	1755	2596.01	4833.61	3826.01	1526.73	1114.44	1250
EABT37647	0	2	2	0	0	0	0
EABT37648	0	2	12	3	0	2	4
EABT37649	4	5	0	0	0	0	1
EABT3765	0	0	9	0	0	0	1
EABT37650	615	47	48	103	65	28	80
EABT37651	3261.24	3717.47	2023.4	2618.07	13022	2729.15	2971.32
EABT37652	0	1	2	2	3	0	0
EABT37653	91	153	372.88	338.76	104.85	70	82
EABT37654	0	5	3	4	9	1	18
EABT37655	1	4	11	0	0	1	2
EABT37656	1	6	10	6	0	2	1
EABT37657	0	0	6	0	0	0	0
EABT37658	1	4	0	0	0	1	0
EABT37659	0	1	8	0	0	0	1
EABT3766	1714.23	1815	953.79	1266.16	754	2225.38	1818.02
EABT37660	0	0	2	26	1	0	1
EABT37661	0	1	3	1	0	0	0
EABT37662	0	1	3	0	1	1	0
EABT37663	23	105	22	0	1	66	6
EABT37664	274	477.06	121	493	322	116	243
EABT37665	0	5	5	5	0	0	0
EABT37666	0	5	13	8	4	0	0
EABT37667	1	2	9	0	0	0	0
EABT37668	218	281	278	885.98	204	76	108
EABT37669	1	0	1	2	0	0	0
EABT3767	1	1	8	0	0	1	0
EABT37670	10	10	0	0	1	30	12
EABT37671	0	0	1	0	0	0	0
EABT37672	7	4	0	0	1	3	0
EABT37673	2391.44	3039.5	2462.24	4020.32	1669.03	2082.31	1655.48
EABT37674	2	0	4	0	1	0	0

EABT37675	1	11	10	4	50	6	1
EABT37676	0	1	1	3	2	0	0
EABT37677	0	0	3	0	0	0	0
EABT37678	742.49	907.56	331.31	600.5	1085.93	453.88	615.23
EABT37679	5	0	13	0	6	6	1
EABT3768	2	6	0	0	0	1	1
EABT37680	8	31	73	23	11	4	7
EABT37681	243.28	500.94	760.62	460	189.99	123	169
EABT37682	436.45	496.83	406.67	541.04	502.27	310.51	269.76
EABT37683	1	1	25	5	1	0	0
EABT37684	1	1	4	2	0	0	0
EABT37685	2	10	0	8	1	15	1
EABT37686	0	2	5	1	0	1	5
EABT37687	35	42	132.11	46.84	6	30	9
EABT37688	0	0	8	0	1	0	0
EABT37689	0	1	1	0	0	8	1
EABT3769	1	1	12	1	0	1	1
EABT37690	0	1	32	3	0	0	0
EABT37691	1	0	1	0	0	3	1
EABT37692	1	3	40	1	0	1	0
EABT37693	1	0	4	0	0	0	0
EABT37694	0	4	13	4	1	0	1
EABT37695	0	1	8	3	0	2	0
EABT37696	10	18	0	0	1	4	0
EABT37697	0	3	94.05	0	0	1	0
EABT37698	1	0	8	0	1	0	0
EABT37699	6070.01	7486.51	7140.67	13628.8	10048.2	6198.09	8099.07
EABT377	5	0	3	3	0	2	1
EABT3770	0	2	14	1	0	1	0
EABT37700	1	3	4	3	0	0	1
EABT37701	2176.71	3174.18	3590.45	7244.01	2578.23	1948.41	1763.69
EABT37702	0	1	8	0	0	0	0
EABT37703	0	1	12	0	0	2	1
EABT37704	0	1	5	0	0	0	0
EABT37705	16	11	76	6	20	2	0
EABT37706	5	14	12	29	3	7	6
EABT37707	0	0	12	2	0	0	0
EABT37708	0	0	32	6	0	0	2
EABT37709	14	14	59	1	3	1	2
EABT3771	6	25	40	52.93	7	14	2
EABT37710	0	0	7	0	0	0	0
EABT37711	76	69	63.75	14	16	39	45
EABT37712	1030.64	1926.32	1584.51	1646.46	680.91	515.93	833.11
EABT37713	0	4	4	0	0	2	1
EABT37714	1	1	15	0	1	0	0
EABT37715	2	11.02	52	1.85	4	1	3
EABT37716	2	1	14	2	1	1	1
EABT37717	4	654.93	3305.88	5800.17	7	10	208
EABT37718	0	0	1	0	73	0	0
EABT37719	12276	20250.8	2332.87	1316.21	9211.33	2722.22	11913.9
EABT3772	3454.43	4067.19	2288.32	3563.89	2304.88	2150.29	1882.04

EABT37720	1	3	34	10	0	1	5
EABT37721	258.58	723.23	108.03	247.29	77	241	74
EABT37722	0	1	1	2	2	0	4
EABT37723	3	8	9	11	7	1	0
EABT37724	1	0	1	0	1	0	0
EABT37725	15	44	85	24	24	15	15
EABT37726	202	240	289.21	570.76	611	438	35
EABT37727	2	4	2	1	0	6	1
EABT37728	1168.8	1590.3	1277.21	374	368.15	2423.28	2027.57
EABT37729	24269.8	5718.4	3	1	7	29009.2	8
EABT3773	0	1	3	3	0	0	0
EABT37730	0	2	1	12	0	0	0
EABT37731	0	3	0	1	2	0	0
EABT37732	673	861.6	1406.04	2807.87	663.93	715	754.57
EABT37733	13	19	10	24	8	6	8
EABT37734	4	4	1	2	1	1	3
EABT37735	0	0	0	0	0	1	8
EABT37736	0	3	2	2	0	2	0
EABT37737	1	1	5	8	0	0	0
EABT37738	1	7	15	49	0	1	1
EABT37739	1	0	8	1	0	0	0
EABT3774	2546.98	3834.1	3107.53	5280.8	2233.07	1506.98	1607.18
EABT37740	665	1282.03	2508.28	3507.01	4007.99	177	786
EABT37741	1	0	4	2	1	0	0
EABT37742	96	145	3	0	0	5	1
EABT37743	4	4	5	2	0	8	0
EABT37744	1	1	243	239823	2	0	0
EABT37745	2	1	5	0	0	4	0
EABT37746	4	93	6	8	4	6	18
EABT37747	0	4	18	0	0	1	0
EABT37748	5	7	2	1	0	0	0
EABT37749	636	1083	1009	1146.13	402	1448	624
EABT3775	0	0	0	47	0	0	0
EABT37750	25	17	5	12	0	7	1
EABT37751	2	1	0	0	0	0	8
EABT37752	3	4	1	4	1	1	0
EABT37753	1180.45	3966.12	8941.83	4711.89	2029.8	1978.37	1522.99
EABT37754	3	2	0	0	0	0	0
EABT37755	0	0	3	0	2	0	2
EABT37756	0	3	16	9	0	0	0
EABT37757	1	0	19	6	0	8	1
EABT37758	9	31	1	3	0	11	2
EABT37759	0	0	2	0	0	0	0
EABT3776	1	3	5	0	1	0	0
EABT37760	7	27	36	162	21	0	1
EABT37761	2	8	3	5	1	2	2
EABT37762	2	5	6	0	1	0	6
EABT37763	2	9	8	2	0	0	2
EABT37764	2	14	8	51	8	0	1.99
EABT37765	1	3	29	7	0	0	2
EABT37766	0	1	5	0	0	0	0

EABT37767	1	0	6	1	0	1	0
EABT37768	0	0	2	0	3	0	0
EABT37769	882.32	1385.49	1702.64	2280.89	1524.05	795.79	1227.81
EABT3777	16	13	15	3	0	0	0
EABT37770	387.04	836.46	293.63	227.72	819.18	279.04	673.52
EABT37771	1	3	0	0	0	0	1
EABT37772	365	555.86	752.17	1538.76	819.24	174	292.9
EABT37773	3	9	13	6	0	24	10
EABT37774	3	2	22	0	0	0	1
EABT37775	776	1242.53	1403.51	2406.01	517	939	693
EABT37776	2926.62	14502.6	18338.2	1253.13	6529.49	222.04	2194.74
EABT37777	32	86	72	156.98	98.51	26	25
EABT37778	3	0	5	1	0	0	0
EABT37779	0	3	10	37	25	0	9
EABT3778	7	37	104.01	5	23.98	12	27.86
EABT37780	710	1199	1541.78	2240.84	1284	739.68	750
EABT37781	5.94	65.1	53.89	25.99	29.91	2	0
EABT37782	0	1	6	0	0	0	0
EABT37783	0	0	4	3	2	0	0
EABT37784	7288.18	18906.9	21365.6	20073.5	10647	1312	3198.85
EABT37785	0	0	3	0	0	0	0
EABT37786	3	6	10	3	4	1	6
EABT37787	0	0	7	2	0	0	0
EABT37788	63	162	41	11	148	4	8
EABT37789	0	1	1	2	1	0	1
EABT3779	0	0	0	0	2	0	1
EABT37790	4	12	44	9	0	2	1
EABT37791	311.02	265	77	267	78	235.93	448.94
EABT37792	632	523	238	107	179	71	455
EABT37793	0	2	13	3	1	0	4
EABT37794	30	80	78	165	22	35	32
EABT37795	127	235	274	398.94	350	73	89
EABT37796	0	0	0	0	1	0	0
EABT37797	27	829	45	4	1583.93	16	90
EABT37798	1	0	10	0	1	0	0
EABT37799	0	0	15	1	0	0	0
EABT378	286	448.81	142.13	628.75	241.19	237.93	165.98
EABT3780	1	0	11	22	0	0	0
EABT37800	1	3.6	0	0	0	6	0
EABT37801	0	12	1	2	0	0	0
EABT37802	53	0	0	0	0	98	0
EABT37803	0	1	2	2	0	0	1
EABT37804	1	4	12	8	3	0	0
EABT37805	0	1	2	0	1	0	1
EABT37806	1	11	5	9	13	0	11
EABT37807	33.22	15	4	0	2	27	16
EABT37808	2	0	19	1	1	0	0
EABT37809	3	13	12	20	0	0	0
EABT3781	2	3	2	22	2	0	2
EABT37810	4	0	14	1	0	10	0
EABT37811	3	6	118.01	5	3	1	0

EABT37812	2	1	32	2	1	0	0
EABT37813	2	7	36	1	0	0	1
EABT37814	12	16	6	0	0	3	0
EABT37815	2	1	9	0	2	1	0
EABT37816	0	0	1	3	0	0	0
EABT37817	509.71	722.56	979.42	1591.34	776.07	523	540.39
EABT37818	0	0	1	1	0	1	0
EABT37819	150.88	83	220.86	53.78	123	375	288.76
EABT3782	1	12	1	1	0	0	0
EABT37820	1455.33	2740.28	2564.36	5453.14	1410.06	695.06	1207.97
EABT37821	1312.96	1344.75	746	1505.11	948.93	1160	1003.01
EABT37822	3	12	6	11	0	0	0
EABT37823	0	1	24	1	0	0	1
EABT37824	3	2	6	0	0	0	2
EABT37825	1	1	1	1	1	1	2.99
EABT37826	255.03	794.01	426.01	1992.88	173.03	88	215
EABT37827	1485.89	2845.36	1431.85	5082.23	1752.03	1028.6	1761.47
EABT37828	0	0	1	3	0	0	1
EABT37829	775	1104	1912.46	2020.94	972	624.22	483
EABT3783	478	759.03	277	960	427.99	371	324
EABT37830	2391.55	2023.34	310	270.99	553.54	3071.87	2395.85
EABT37831	1	0	3	8	0	1	0
EABT37832	73	83.41	460.08	33	12	50	39
EABT37833	525.99	713	755.01	1027.38	457	344	373
EABT37834	0	0	1	3	2	0	2
EABT37835	0	0	2	2	6	0	0
EABT37836	1	3	16	0	1	26	3
EABT37837	0	0	14	1	2	1	0
EABT37838	1	1	5	0	0	6	1
EABT37839	0	0	0	2	2	0	1
EABT3784	160.98	347.22	319.89	894.33	500.79	153.18	135.97
EABT37840	3	7	1	0	0	3	9
EABT37841	4	2	4.08	3	2	2	1
EABT37842	1	2	4	0	3	1	8
EABT37843	0	0	7	0	0	0	0
EABT37844	10883.9	14721.1	17072.8	13498.2	8299.93	7101.99	7011.14
EABT37845	0	7	5	9	0	2	2
EABT37846	1	1	2	1	0	1	0
EABT37847	1	0	18	1	0	0	0
EABT37848	0	1	4	0	0	0	0
EABT37849	2	4	47	7	3	4	2
EABT3785	0	0	4	0	0	1	0
EABT37850	21	53	17	0	29	10	107
EABT37851	2	13	3	5	2	0	0
EABT37852	36	10	1	278	7	0	0
EABT37853	0	1	6	0	0	0	0
EABT37854	5369.52	8381.52	13689.5	10968.6	4496.81	5746.07	6769.02
EABT37855	1	5	5	2	1	1	2
EABT37856	2	8	41	8	2	1	4
EABT37857	6	14	6	1	2	4	5
EABT37858	0	0	1	14	13	0	0

EABT37859	0	2	4	10	0	0	0
EABT3786	1	0	4	0	0	0	0
EABT37860	2	3	4	2	7	0	0
EABT37861	0	4	5	21	1	1	0
EABT37862	4273.34	5130.52	6881.11	6969.63	3273.48	1768.27	3102.48
EABT37863	2	3	0	1	0	6	8
EABT37864	505.99	599.01	541.08	702	570.41	354.35	302.07
EABT37865	733.31	538.82	250	336.15	967.04	38	111.62
EABT37866	0	2	3	1	0	0	0
EABT37867	1	0	6	3	0	0	0
EABT37868	0	0	10	0	0	0	0
EABT37869	30	62	51	445.01	488.04	6	17
EABT3787	4511.98	6221.78	2714	5883.35	3565.94	4260	4016.57
EABT37870	1	7	33	1	4	0	1
EABT37871	13	8	32	24	6	9	6
EABT37872	0	0	4	0	0	0	1
EABT37873	80	224	377	4788.44	381	32	108
EABT37874	0	0	12	0	0	0	0
EABT37875	1484.01	2131	2454.94	3106.72	1108	975	1814
EABT37876	425.05	570.22	576.76	1079.22	639.19	398.13	400.5
EABT37877	0	0	6	0	0	0	0
EABT37878	1	1	2	0	0	0	0
EABT37879	55.89	91	111	151.39	69	9	59
EABT3788	10	5	44	3	17	8	98
EABT37880	6105.12	8741.88	4926.97	5181.01	4241.08	6382.09	4670.06
EABT37881	0	7	3	1	1	5	3
EABT37882	0	1	1	0	2	0	2
EABT37883	1	23	33	4	1	0	0
EABT37884	34	74	24	152.02	12.99	35	42
EABT37885	3	7	4	5	2	2	2
EABT37886	5874.41	1588.13	391	183.19	490	67	148
EABT37887	0	4	2	2	1	0	0
EABT37888	0	10.82	5	19	3	3	6
EABT37889	0	2	5	1	2	0	2
EABT3789	0	0	0	10	0	1	0
EABT37890	2807.98	3143	3118.93	4570.34	3407.7	1914.29	1971.99
EABT37891	3	2	4	0	0	0	0
EABT37892	0	0	2	1	0	0	0
EABT37893	0	3	21	0	0	0	0
EABT37894	0	0	4	1	0	0	0
EABT37895	2443.76	2612.92	1310.03	3908.09	1534	3081.5	2391.58
EABT37896	0	1	0	0	0	39	0
EABT37897	18	34	10	27	13	21.7	16
EABT37898	448	669	811.98	1027.95	890	331	357
EABT37899	0	0	14	13	7	0	2
EABT379	599	749.63	10	7	41	723.94	500
EABT3790	91	130	287	2170	159	13	33
EABT37900	0	1	4	1	0	0	0
EABT37901	4	4	5	52	11	0	0
EABT37902	0	3	6	0	0	0	0
EABT37903	14019	10173.5	8363.9	2577.71	12395.4	4868.05	2293.03

EABT37904	0	0	4	0	1	1	0
EABT37905	1	2	69	12	3	1	2
EABT37906	0	3	3	0	0	0	0
EABT37907	87	989	48	33	272	1265	1350.89
EABT37908	10483	9606.61	92055.5	16424.6	4175.19	2823.57	1805.12
EABT37909	835.54	1120.02	1346.64	1408.4	884.29	373.51	638.84
EABT3791	0	0	7	0	0	0	1
EABT37910	0	3	7	3	1	0	0
EABT37911	23	16	0	0	0	0	0
EABT37912	402	488	256.89	627.98	451	284.18	233
EABT37913	11	5	0	0	0	0	0
EABT37914	0	1	22	1	1	0	0
EABT37915	0	5	24	1	0	0	0
EABT37916	0	0	6	0	0	3	1
EABT37917	0	3	6	3	1	0	1
EABT37918	1	0	0	1	1	1	5
EABT37919	12527.2	14574.3	11890.5	7071.32	8269.15	9465.24	8432.6
EABT3792	0	3	25	1	3	1	1
EABT37920	2	13	9	8	5	6	2
EABT37921	1443.27	3946.61	9879.33	4750.72	1944.89	1308.57	1522.32
EABT37922	0	0	2	0	0	1	1
EABT37923	24	17	22.22	6.66	0	13	20
EABT37924	0	0	5	0	0	0	0
EABT37925	155.11	219	114	408.49	313.3	94.38	180.73
EABT37926	3	4	32	5	2	0	2
EABT37927	154.97	130	442	109	85	45	149
EABT37928	15	49	311.98	204	7	11	1
EABT37929	0	0	2	0	2	0	0
EABT3793	2	1	3	2	1	2	3
EABT37930	0	0	7	0	0	0	0
EABT37931	3	2	15	1	0	1	1
EABT37932	1	0	11	0	0	0	1
EABT37933	2528.6	4186.9	1629.75	3320.51	1940.03	2726.95	2003.15
EABT37934	0	0	1	0	0	0	0
EABT37935	15	6	0	2	1	7	1
EABT37936	625.38	1058.5	1773.7	1841.24	846.39	1096.59	929.95
EABT37937	0	3	4	0	0	0	0
EABT37938	0	14	19	319.98	11	0	0
EABT37939	0	1	29	3	0	1	0
EABT3794	0	0	0	0	0	1	0
EABT37940	1	1	4	2	2	1	1
EABT37941	0	1	17	0	1	0	0
EABT37942	0	0	5	1	2	0	0
EABT37943	0	0	2	0	0	1	2
EABT37944	4063.33	2096.9	14	6.01	72	802.94	247
EABT37945	2	2	1	0	0	0	0
EABT37946	0	2	3	0	1	0	1
EABT37947	1	3	6	1	0	2	0
EABT37948	1	1	0	0	0	1	0
EABT37949	0	4	18	0	0	1	0
EABT3795	5	3	17	3	5	2	3

EABT37950	1	4	13	2	2	2	0
EABT37951	944.87	1227.69	1558.47	2208.77	676.98	930.07	906.64
EABT37952	50	198.08	1224.57	9351.29	828.99	9	35
EABT37953	0	0	18	1	0	0	0
EABT37954	4	7	31	2	0	2	3
EABT37955	4	4	5	1	0	1	1
EABT37956	11	20	2	4	7	11	25
EABT37957	0	3	5	0	0	0	0
EABT37958	0	1	3	1	1	0	0
EABT37959	104	215	164	295	908.53	203.93	552.95
EABT3796	0	3	7	4	0	0	0
EABT37960	0	1	6	0	0	0	1
EABT37961	3	4	86.77	32	3	0	0
EABT37962	0	0	0	0	0	0	0
EABT37963	2	1	1	456.02	6	5	1
EABT37964	4	1	8	1	5	2	5
EABT37965	883.32	952.15	609.99	1639.99	942.06	568.94	574
EABT37966	0	2	7	2	1	2	0
EABT37967	1	2	21	1	1	4	2
EABT37968	2	2	0	0	1	0	0
EABT37969	44	236	193	97	278	4	11
EABT3797	2	0	25	0	2	0	1
EABT37970	0	1	5	2	0	0	0
EABT37971	5	11	0	0	0	7	9
EABT37972	687	1199.03	801.67	1575	908	586	621
EABT37973	35	59	17	121	21	71	69
EABT37974	0	14	29.15	79	10	0	1
EABT37975	0	1	5	0	1	0	0
EABT37976	4	10	81.96	88	5	2	3
EABT37977	0	1	4	0	0	0	0
EABT37978	38	21	15	1	1	25	3
EABT37979	794.98	745	718	554	412.02	370	462
EABT3798	0	0	12	1	0	0	1
EABT37980	0	14	0.05	0	27	0	2
EABT37981	27	30	5	8	2	24	1
EABT37982	0	1	1	2	3	0	0
EABT37983	8	30	73	68	31	14	23
EABT37984	1	0	4	5	0	0	4
EABT37985	3	25	24	26	0	6	10
EABT37986	1302.24	2719	760	738.4	1789.61	237	4616.9
EABT37987	28	25	70	141	120	10	14
EABT37988	0	4	5	3	2	0	0
EABT37989	0	5	0	12	0	0	2
EABT3799	0	1	4	3	0	0	0
EABT37990	0	1	2	3	1	0	0
EABT37991	7	13	19	41	10	1	6
EABT37992	2715	1635.55	1320.66	1559.96	840.11	2539	1298
EABT37993	164	256	282	410	1950.03	72	106
EABT37994	1	2	5	10	0	0	0
EABT37995	2	2	1	0	0	4	0
EABT37996	641.81	2275.57	774.97	3329.38	1404.74	33	494.94

EABT37997	0	0	0	0	0	0	0
EABT37998	3	6	10	15	0	3	2
EABT37999	0	2	6	0	0	5	2
EABT38	2	1	14	11	0	0	2
EABT380	0	0	0	2	0	0	0
EABT3800	16	29	162.49	3	1	2	5
EABT38000	1	1	27	1	1	9	2
EABT38001	1	0	9	1	0	0	0
EABT38002	11	49	106	56	21	16	13
EABT38003	0	0	5	0	1	0	0
EABT38004	1999.48	3096.51	3339.46	3145.84	1280.52	455.61	600.22
EABT38005	4	0	0	4	1	4	5
EABT38006	7	14	14	36	3	3	7
EABT38007	0	3	6	5	1	0	3
EABT38008	0	0	10	0	0	2	1
EABT38009	7	36	82	30	14	12	13
EABT3801	3	0	2	7	0	0	1
EABT38010	1	3	5	1	0	2	0
EABT38011	2	11	34	3	2	4	2
EABT38012	24	19	48.98	2	1	0	0
EABT38013	0	0	15	1	10	0	0
EABT38014	0	2	7	2	0	0	2
EABT38015	0	5	16	2	1	1	0
EABT38016	721.18	1297.05	1251.99	2350.11	782	640	556.97
EABT38017	1	0	4	17	2	0	0
EABT38018	828.48	1275.41	4306.96	3133.02	556.99	731	387.01
EABT38019	76	172	126.98	122.15	68	81	124
EABT3802	1	2	5	1	0	0	0
EABT38020	3135.55	7343.79	694.82	1	1871.58	152.36	377.83
EABT38021	0	0	7	9	0	0	1
EABT38022	0	0	10	0	0	0	0
EABT38023	16	20	6	32	17	40	64
EABT38024	5	7	5	13	5	9	6.23
EABT38025	0	1	1	0	2	0	1
EABT38026	1	2	17	1	2	7	2
EABT38027	8	25	28	2	11	0	7
EABT38028	0	4	9	9	1	0	0
EABT38029	4	2	0	0	0	1	3
EABT3803	2110.18	3098.13	2692	3841.09	2227.02	3677.02	2092
EABT38030	0	0	4	0	0	0	0
EABT38031	0	1	3	1	0	0	1
EABT38032	2	2	6	2	2	2	2
EABT38033	10	35	69	66	127	12	14
EABT38034	151	2	1	367	60024.1	19	263
EABT38035	17	14	17	22	661.99	0	117
EABT38036	0	4	0	1	0	3	0
EABT38037	23	0	0	0	0	4	2
EABT38038	1344.12	1547.48	435	479	619	3121.46	3544.32
EABT38039	1	1	3	2	2	0	0
EABT3804	0	0	2	0	1	0	1
EABT38040	494	996.17	1146.17	2510.54	485.06	549.18	378

EABT38041	1	1	5	1	1	1	2
EABT38042	1598.8	2971.07	4312.75	3606.98	2701.14	270	1096
EABT38043	0	1	4	1	1	2	0
EABT38044	0	6	7	5	5	2	3
EABT38045	25	117.35	80	214	136	5	6
EABT38046	1	2	3	2	0	0	1
EABT38047	686	1020.37	1095.62	2223.01	1009	444.55	550.79
EABT38048	54	149.98	96	3	0	0	0
EABT38049	43	33	11	27	10	19	18
EABT3805	0	0	12	1	0	0	0
EABT38050	23	99.99	175	4489.01	15633.8	21	62
EABT38051	0	1	6	1	0	0	1
EABT38052	1	1	1	0	2	2	2
EABT38053	0	2	2	2	0	1	2
EABT38054	0	1	2	31	0	0	1
EABT38055	0	3	2	11	0	0	1
EABT38056	0	0	42	1	2	0	0
EABT38057	0	0	1	31	9	0	0
EABT38058	0	1	18	3	0	0	0
EABT38059	449.81	657	553.39	937	469	400	386.64
EABT3806	1	0	3	9	0	0	1
EABT38060	0	3	2	2	0	3	1
EABT38061	425.01	685	704	2012.94	363	402.01	221.2
EABT38062	0	4	3	0	2	2	4
EABT38063	0	0	4	0	1	0	0
EABT38064	0	0	5	1	0	0	2
EABT38065	17	3	5	5	29	68	22
EABT38066	2	4	12	4	0	0	0
EABT38067	0	1	4	5	1	1	2
EABT38068	0	1	8	0	0	0	0
EABT38069	223.97	467	288	670	261	276	229
EABT3807	1	0	5	1	1	0	11
EABT38070	0	1	4	1	0	2	1
EABT38071	3	6	277.43	8	5	0	345
EABT38072	2	4	3	8	1	6	40
EABT38073	0	3	9	0	0	0	0
EABT38074	1	1	14	3	1	0	0
EABT38075	1	2	11	1	0	0	0
EABT38076	0	0	0	3	3	0	2
EABT38077	268	251	144	377.93	329	205	200
EABT38078	1	0	2	1	0	0	0
EABT38079	1	6	6	6	1	1	1
EABT3808	2	4	27.02	163.01	13	2	10
EABT38080	62	18	4	2	12	36	68
EABT38081	0	0	5	0	0	0	0
EABT38082	11	17	3	10	18	2	9
EABT38083	18	18	0	0	5	50.1	37
EABT38084	0	0	23	0	22	0	0
EABT38085	2968.94	4370.58	5343.16	5929.47	4303.7	2227.17	3917.73
EABT38086	1	0	6	0	1	1	0
EABT38087	0	14	1	10	2	1	0

EABT38088	1	2	7	0	0	0	0
EABT38089	0	0	9	1	0	0	0
EABT3809	6	9	5	9	4	3	2
EABT38090	0	3	8	1	0	1	2
EABT38091	4	9	9	5	1	11	0
EABT38092	9	16	19	32	1	12	3
EABT38093	1	0	6	1	0	0	0
EABT38094	0	0	5	1	0	1	1
EABT38095	6	7	20	11	4	12	3
EABT38096	0	0	6	0	0	1	0
EABT38097	0	1	11	0	0	0	0
EABT38098	5	9	72	17	4	1	4
EABT38099	0	0	6	0	0	1	1
EABT381	102	1	1	0	3	12	122
EABT3810	3	4	3	1	1	6	2
EABT38100	4	5	0	0	2	0	1
EABT38101	5	32	17	92	24	1	13
EABT38102	0	2	14	6	0	1	1
EABT38103	8731.38	13938.9	13409	5079.88	7252.01	725.53	3072.64
EABT38104	2	1	0	0	0	1	0
EABT38105	0	0	7	1	1	0	0
EABT38106	2	4	6	5	2	3	2
EABT38107	0	0	8	1	0	0	0
EABT38108	0	4	5	3	0	0	0
EABT38109	46	59	67	162	58	19	43
EABT3811	264	417.02	588.12	903.59	495.31	344.99	343
EABT38110	14	16	223.5	567.38	18.03	17	19
EABT38111	0	5	17	6	0	0	1
EABT38112	16	61	354.68	298.21	44.16	14	35
EABT38113	0	1	9	3	1	0	3
EABT38114	3	5	4	10	4	1	0
EABT38115	12	19	10	64	20	9	9
EABT38116	4	6	2.66	5	6	0	6
EABT38117	7	11	5	8	12	0	8
EABT38118	1	0	8	0	2	0	2
EABT38119	23	85	284	462	99	20	26
EABT3812	2	4	2	13	2	1	2
EABT38120	0	2	2	0	0	0	0
EABT38121	1	0	7	2	0	1	0
EABT38122	1446.02	2471.85	3252.76	2866.36	1760.98	2126.09	1492.81
EABT38123	1	1	1	0	0	0	2
EABT38124	0	0	8	1	1	0	0
EABT38125	379.04	503	441.99	1242.37	440.99	13	19
EABT38126	1	3	3	1	1	0	0
EABT38127	2	6	11	0	0	0	0
EABT38128	0	0	2	5	8	0	0
EABT38129	3	5	10	2	1	2	6
EABT3813	2	1	19	1	3	0	2
EABT38130	7	5	16	5	1	0	0
EABT38131	1	5	13	25	6	0	1
EABT38132	2257.09	3196.87	4159.3	6059.54	1511.51	2384.86	2062.48

EABT38133	0	1	2	0	0	4	2
EABT38134	0	0	0	0	3	0	2
EABT38135	1025.84	1213.63	820.25	1579.22	824.49	1006.31	691.5
EABT38136	5	15	2	8	18	16.43	97
EABT38137	13	63	205	22	4	1	4
EABT38138	2	1	2	0	0	0	1
EABT38139	3	0	9	0	0	0	0
EABT3814	1636.86	3019.15	2001.47	2823.65	1300.1	1399.52	1397.44
EABT38140	4	10	10	7	7	1	6
EABT38141	1	0	26	2	0	1	0
EABT38142	0	2	4	9	0	0	1
EABT38143	6	16	2	30	7	2	0
EABT38144	3330.34	5647.12	6596.69	12934.9	5355.86	2373.38	3672.44
EABT38145	501.55	570.32	777.08	6015.22	185.16	19.84	42
EABT38146	0	1	11	1	0	0	0
EABT38147	1764.31	2678.48	2768.15	4628.78	2439.11	1308.92	1719.74
EABT38148	8.2	13	11	1	0	29	4
EABT38149	0	1	0	6	0	0	0
EABT3815	0	0	0	0	0	0	0
EABT38150	0	1	5	0	0	0	0
EABT38151	3	28	358.45	9	5	2	2
EABT38152	1	0	1	1	1	0	3
EABT38153	1	5	45	2	4	3	3
EABT38154	1	2	4	0	3	1	1
EABT38155	5	1	3	5	0	1	0
EABT38156	0	0	6	3	2	0	1
EABT38157	1	0	1	1	1	1	6
EABT38158	0	1	1	0	1	0	1
EABT38159	599	2604	2037.04	3723.77	1587	1698	1536.42
EABT3816	0	0	7	0	0	0	0
EABT38160	0	0	5	2	0	0	0
EABT3817	1	3	3	2	1	1	0
EABT3818	0	0	10	1	0	0	0
EABT3819	0	3	5	6	3	1	1
EABT382	0	1	1	2	0	0	0
EABT3820	379	786.99	1065	3692.97	1076	278	255.46
EABT3821	1	2	16	0	0	6	4
EABT3822	1937.92	8282.42	9247.63	67874.1	1647.87	1044.41	1167.38
EABT3823	76	21	5	0	1	0	0
EABT3824	1	1	6	3	0	0	0
EABT3825	2203.78	4022.8	5046.73	6878.77	3918.78	2788.12	2939.22
EABT3826	5	23	24	60	15	2	8
EABT3827	7	9	8	41	6	4	19
EABT3828	1	0	10	0	0	0	0
EABT3829	2	1	6	5	0	0	0
EABT383	2164.59	1772	852	2118	2657.99	1958	771
EABT3830	63	93.06	278	36	22	111	48
EABT3831	2	0	14	7	2	0	1
EABT3832	10	21	38	10	4	0	1
EABT3833	0	6	7	7	2	4	11
EABT3834	36	110	166	26	1164	3	19

EABT3835	1	17	22	23	22	1	2
EABT3836	1743.92	2913.96	5808.13	3095.99	1466.6	518.98	892.3
EABT3837	8	11	224	38	70	39	23
EABT3838	1	1	3	0	1	0	0
EABT3839	0	0	18	0	0	0	0
EABT384	2353.54	4009.37	4293.48	4249.27	2240.37	872.08	1270.71
EABT3840	2	4	14	2	0	1	0
EABT3841	2672.71	2767.46	1353.59	3014.91	1391.75	2398.54	2444.8
EABT3842	0	4	95	8	0	0	1
EABT3843	3	1	27	5	0	0	2
EABT3844	0	3	3	7	1	0	0
EABT3845	5	3	1	1	1	0	3
EABT3846	1548	1400	549	796	3204.42	1258.06	1127
EABT3847	3	1	0	0	0	5	1
EABT3848	5	16	10	1	0	6	4
EABT3849	2	5	15	2	3	3	1
EABT385	618.02	1016	652.74	1253.24	1002.59	599	663.73
EABT3850	0	0	12	1	0	0	0
EABT3851	1234459	911801	350906	350382	1437.87	31	110
EABT3852	0	0	2	0	1	2	7
EABT3853	0	3	10	4	1	2	2
EABT3854	0	0	2	2	0	0	2
EABT3855	210	428	697.02	2826.01	620	20	34
EABT3856	0	6	21	2	3	5	0
EABT3857	0	1	13	1	0	1	0
EABT3858	3	5	42	7	3	1	1
EABT3859	200	872.1	1069	330.36	104.11	62.03	80
EABT386	0	1	5	2	1	0	0
EABT3860	20	17	2	17	58	2	2
EABT3861	4	3	6	4	2	0	0
EABT3862	25.3	70.85	36	59.48	61	37.04	87
EABT3863	0	0	7	2	0	0	0
EABT3864	1	9	9	4	2	0	0
EABT3865	7	6	0	0	0	0	0
EABT3866	0	7	0	0	3	0	1
EABT3867	1	0	11	0	0	0	0
EABT3868	0	0	9	1	0	0	0
EABT3869	886.52	1254.81	1064.68	1980.52	1160.39	399.25	656.71
EABT387	5912.74	6487.05	3446.96	10589.3	3241.57	6573.76	5259.16
EABT3870	1	4	8	2	1102	0	1
EABT3871	18	27	10	22	444	3	27
EABT3872	0	0	3	0	0	0	0
EABT3873	6	2	11	6	2	2	2
EABT3874	636	1055.23	1422.86	873.18	1027	947	1296.98
EABT3875	3	4	2	2	1	1	0
EABT3876	4	10	10	0	2	1	1
EABT3877	2	3	2	2	1	0	1
EABT3878	1	3	46	4	0	0	0
EABT3879	0	0	3	0	0	0	0
EABT388	2	11	11	1	4	4	2
EABT3880	0	1	8	0	0	0	0

EABT3881	1	6	3	12	16	0	0
EABT3882	0	0	1	17	0	0	0
EABT3883	838	1181	917	1714	737.54	531	655
EABT3884	300	794.03	916.86	79	1012	151	159
EABT3885	38	82	24	26	55	0	18
EABT3886	0	2	1	4	3	0	1
EABT3887	0	1	0	0	1	1	0
EABT3888	556.87	827	749.96	734.43	592.49	493.16	470.01
EABT3889	0	3	8	0	0	0	0
EABT389	0	2	11	0	0	0	0
EABT3890	0	0	1	4	0	1	1
EABT3891	6	2	13	1	0	1	0
EABT3892	471	3174.82	1946.01	1385.79	2052	419	710.07
EABT3893	0	4	374	4	3	1	0
EABT3894	6	16	128	33	7	13	9
EABT3895	1	3	4	4	0	0	0
EABT3896	1	6	50	0	0	6	4
EABT3897	11	16	13.31	16.32	8	95	4
EABT3898	0	0	4	1	0	0	0
EABT3899	3	3	5	2	3	0	1
EABT39	2	6	2	4	0	0	0
EABT390	1	2	68	0	0	0	0
EABT3900	1923.21	3716.82	6285.42	3320.55	3157.01	1512	2385.68
EABT3901	1	1	0	2	0	0	5
EABT3902	1	3	5	12	1	4	3
EABT3903	2	12	19	0	0	4	11
EABT3904	6	5	12	11	3	14	2
EABT3905	1	2	4	0	180	3	1
EABT3906	3	8	61	16	1	1	5
EABT3907	1507.99	2375.13	1403.97	4309.96	2121.14	1703.78	1854.1
EABT3908	2	5	3	0	0	0	0
EABT3909	0	2	0	2	0	0	2
EABT391	0	0	12	2	0	1	1
EABT3910	355	447	441	806	331	284	229
EABT3911	17	36	37	59	30	11	19
EABT3912	655.81	959.43	1090.26	2017.11	774.88	377.88	575.89
EABT3913	2	26	6	2	3	0	0
EABT3914	0	9	5	0	0	1	0
EABT3915	3	4	4	20	5	1	0
EABT3916	21	58	105.05	59	25	26	45.01
EABT3917	0	0	9	2	5	0	0
EABT3918	0	0	7	2	0	0	0
EABT3919	1	3	7	1	1	0	2
EABT392	0	1	18	0	0	0	0
EABT3920	2	2	3	3	1	6	0
EABT3921	0	0	3	2	2	0	1
EABT3922	1091	2136.08	1717	4205.94	835.54	613.33	948.35
EABT3923	2	7	32	0	0	0	1
EABT3924	0	5	3	4	15	0	1
EABT3925	1	1	4	0	0	2	2
EABT3926	1	2	3	0	0	1	1

EABT3927	3	2	5	1	1	1	2
EABT3928	3	2	8	11	0	0	1
EABT3929	0	0	20	6	0	1	0
EABT393	585.07	694.85	595.75	818.04	260	145	314.47
EABT3930	768.27	831	499.86	1070.6	757.38	927	555.27
EABT3931	10	29	684.01	1303.9	37	15	22
EABT3932	0	3	4	2	2	1	2
EABT3933	2	4	25	12	0	0	0
EABT3934	2	2	8	1	0	0	0
EABT3935	1	3	2	1	0	0	0
EABT3936	623.95	923.61	388.81	768.32	986.51	360.85	288
EABT3937	60	71	0	0	2	4	13
EABT3938	0	0	0	1	0	0	0
EABT3939	0	0	32	0	1	0	1
EABT394	5	2	0	3	0	3	3
EABT3940	0	2	2	2	0	0	1
EABT3941	0	2	14	2	2	0	0
EABT3942	283.35	333.69	286.02	321.02	2794.23	241	282.35
EABT3943	0	4	3	3	0	1	4
EABT3944	0	0	3	1	0	0	0
EABT3945	17	15	11	6	1	6	2
EABT3946	0	1	6	3	1	3	0
EABT3947	0	0	53	9	1	0	0
EABT3948	12	84.99	4	6	25.94	0	3
EABT3949	0	5	3	1	0	0	1
EABT395	0	1	1	0	2	3	1
EABT3950	1	0	14	0	0	0	0
EABT3951	0	0	5	1	0	0	0
EABT3952	0	1	3	2	4	0	1
EABT3953	671	692.42	1009.38	1097	1096.31	637.56	571
EABT3954	0	1	5	4	4	6	2
EABT3955	0	1	15	0	0	0	0
EABT3956	3	10	29	2	1	1	0
EABT3957	0	0	6	0	0	0	0
EABT3958	1647.3	2632	1493.16	3512.54	3165.99	1540.01	1608.98
EABT3959	2	3	47	55	3	1	0
EABT396	0	0	4	1	0	1	0
EABT3960	0	4	10	2	0	5	0
EABT3961	3	7	37	13	0	0	0
EABT3962	0	11	4	7	2	1	3
EABT3963	6227.19	10680.8	4272.62	3787.73	3015.03	3698.34	958
EABT3964	0	0	1	2	1	0	0
EABT3965	0	5	7	19	0	0	0
EABT3966	404.97	572.07	577	1063.44	349.53	212	315.99
EABT3967	0	4	0	28	3	0	0
EABT3968	30	30	4	0	0	36.5	1
EABT3969	13	8.01	13.66	6	5	28	30
EABT397	1	0	12	4	0	0	0
EABT3970	1	1	11	2	14	3	12
EABT3971	1	5	7	2	2	0	0
EABT3972	0	1	4	3	0	0	0

EABT3973	96	51	7	4	2	98	3
EABT3974	3	6	8	8	2	1	1
EABT3975	2	3	5	0	0	23	4
EABT3976	0	0	9	0	0	0	1
EABT3977	8	32	40	109.03	25	8	14
EABT3978	0	1	7	0	0	0	0
EABT3979	0	0	21.02	0	0	1	0
EABT398	885.21	1297.31	1547.02	2447.3	396	506	472
EABT3980	6	9	21	11	1	2	2
EABT3981	3	0	3	2	1	1	1
EABT3982	822.49	1188.8	959.89	1675.78	864.41	1858.64	539.93
EABT3983	4	5	10	2	1	0	1
EABT3984	2	1	2	0	1	1	0
EABT3985	54.95	48.99	42	26	35	16	30.54
EABT3986	1	7	20	19	2	0	0
EABT3987	1087.98	2123.2	7717.11	1715.96	2269.96	486	1786.02
EABT3988	4	10	0	0	2	15	6
EABT3989	0	1	4	0	1	2	0
EABT399	0	2	43.97	3	0	0	0
EABT3990	169	228	626.99	3505.7	1020.02	7	17
EABT3991	10	24	22	23	12	7	17
EABT3992	457	624	868.2	551.96	508	448	486
EABT3993	3	3	2	6	8	0	1
EABT3994	0	2	5	1	0	0	0
EABT3995	0	0	2	18	0	0	0
EABT3996	1	5	11	4	4	5	6
EABT3997	0	0	21	1	1	0	0
EABT3998	6	16	71	23	1	14	2
EABT3999	0	0	2	0	1	0	2
EABT4	12	18	35	10	6	13	15
EABT40	6	4	21	7	4	2	3
EABT400	0	0	10	0	0	0	0
EABT4000	1	5	4	1	26	0	0
EABT4001	2	10	5	9	2	5	3
EABT4002	6899.62	6419.89	356	6919	3031.87	206	565.06
EABT4003	2	1	29	9	0	0	1
EABT4004	2	4	7	0	0	11	0
EABT4005	0	0	7	1	0	0	0
EABT4006	1	3	31	3	0	0	0
EABT4007	20	43	43	47	28	22	23
EABT4008	4	41	22.84	13	5	10	8
EABT4009	0	0	0	0	2	1	2
EABT401	57318	16901.8	844.09	403	422	461.99	330.86
EABT4010	2	1	4	1	0	0	0
EABT4011	718	1036.88	858.01	1589.85	797	590	556
EABT4012	0	0	5	0	0	0	0
EABT4013	0	0	0	0	1	0	1
EABT4014	0	0	11	1	0	1	0
EABT4015	4825.43	5350.39	2743.86	6577.29	1713.86	3331.35	2701.75
EABT4016	0	3	13	3	1	0	0
EABT4017	705.82	1271.28	900.69	1989.11	700.04	613	518.08

EABT4018	6	34	33	3	5	12	9
EABT4019	7	11	5	4	6	13	5
EABT402	17	36	38	18	214.97	29	96
EABT4020	0	0	13	0	0	0	0
EABT4021	1	4	31	2	4	2	2
EABT4022	1	2	10	0	0	0	0
EABT4023	1200.28	2297.67	2868.73	3618.02	1863.95	1317.03	1309.36
EABT4024	0	0	4	0	0	0	0
EABT4025	4	7	12	4	8	5	4
EABT4026	41	55	12	45	29	46	167
EABT4027	2	3	4	3	0	1	0
EABT4028	0	0	0	1	2	0	1
EABT4029	0	6	0	0	0	0	4
EABT403	0	0	9	4	1	0	0
EABT4030	3	4	0	1	5	0	0
EABT4031	1	4	74	13	0	3	0
EABT4032	1	0	4	0	0	0	0
EABT4033	1	2	0	1	4	0	2
EABT4034	4394.94	8612.45	21364.4	10247.6	4021.21	5397.57	4564.99
EABT4035	0	1	7	0	0	0	0
EABT4036	4	7	1	9	0	1	2
EABT4037	0	1	2	0	1	0	0
EABT4038	0	2	1	0	0	2	0
EABT4039	9	25.77	9	165.08	13	2	6
EABT404	6	10	0	0	0	0	0
EABT4040	2	5	45	4	2	0	2
EABT4041	1	1	8	2	199	3	11
EABT4042	6	2	0	0	0	0	0
EABT4043	253	455.54	395	1352	346	261	205
EABT4044	7929.67	19133.7	1526.27	3609.19	9520.45	10257.8	8925.24
EABT4045	2	5	21	5	1	1	1
EABT4046	0	2	3	8	0	0	0
EABT4047	2	1	16	0	0	0	0
EABT4048	4	10	2	19	22	7	31.67
EABT4049	3	2	25	2	0	0	0
EABT405	1	0	8	5	0	2	1
EABT4050	26	44	21	39.98	7	9	13
EABT4051	8	7	336	113	536	30	16
EABT4052	0	0	24	0	0	1	0
EABT4053	1	2	4	3	0	0	0
EABT4054	1	9	2	1	3	0	2
EABT4055	5.94	4	16	6	0	1	3
EABT4056	0	0	12	0	0	0	0
EABT4057	42	78	79	120.06	43.97	21	32.96
EABT4058	1	2	3	5	0	0	2
EABT4059	0	2	1	1	0	0	0
EABT406	3	70	7	22	1	0	1
EABT4060	18	24	38	69	4	20	4
EABT4061	11	2	0	0	3	0	6
EABT4062	0	0	16	1	1	0	0
EABT4063	2	1	5	6	0	0	1

EABT4064	3	3	14	4	0	0	1
EABT4065	377.24	523.99	759.03	885.11	601.09	384.02	588.88
EABT4066	0	2	3	9	5	0	1
EABT4067	0	0	3	0	0	0	0
EABT4068	4	2	6	2	2	0	1
EABT4069	2	2	2	4	2	0	0
EABT407	805.5	988.71	638.2	2302.63	822.83	434.1	726.85
EABT4070	3313.78	4021.83	5685.46	2204.65	2673.64	2027.75	2627.96
EABT4071	0	0	11	3	0	0	0
EABT4072	23.01	125	1624	55.01	68.7	172.26	73
EABT4073	2	0	15	3	0	1	2
EABT4074	29	64	85	98	75	16	29
EABT4075	0	0	2	1	0	0	2
EABT4076	1	0	19	0	0	0	0
EABT4077	19	29	92.01	381	113	26	85
EABT4078	2966.95	5705.4	6723.76	12002	12694.9	2627.93	7617.52
EABT4079	3	0	5	1	0	0	2
EABT408	0	3	3	5	0	8	0
EABT4080	0	0	3	4	9	0	6
EABT4081	8	15	10	16	4	2	3
EABT4082	19	32	20	0	437	4	0
EABT4083	1	3	12	9	1	0	0
EABT4084	0	0	7	0	0	0	1
EABT4085	4	2	4	7	0	0	1
EABT4086	0	1	9	0	3	0	2
EABT4087	3	6	7	20	0	0	0
EABT4088	1	6	11	1	0	1	4
EABT4089	8	9	0	0	0	0	0
EABT409	0	0	0	0	18	0	0
EABT4090	0	2	8	1	0	0	0
EABT4091	0	0	3	0	4	1	5
EABT4092	0	0	9	1	0	0	0
EABT4093	1644.65	3137.06	3712	4047.32	1535.84	1769	1052
EABT4094	0	1	1	0	1	0	0
EABT4095	0	2	17	2	0	3	0
EABT4096	0	0	2.06	2	0	1	0
EABT4097	5	0	0	0	0	4	0
EABT4098	3	8	29	2	2	0	1
EABT4099	3	2	0	1	2	0	28
EABT41	0	2	8	0	0	0	0
EABT410	0	1	17	0	0	0	0
EABT4100	887	1353.92	1517	2228.09	1282.05	639	650
EABT4101	0	0	19	1	0	0	0
EABT4102	9128	9180.1	300	5891	279	1	18
EABT4103	3	6	7.95	14.98	6	2	5
EABT4104	1	3	0	0	2	3	4
EABT4105	0	0	4	0	0	13	0
EABT4106	3	0	7	3	5	10	2
EABT4107	3	4	15	1	2	0	0
EABT4108	30	25	26	0	0	0	0
EABT4109	0	0	1	0	0	0	0

EABT411	0	0	8	0	1	0	0
EABT4110	747.78	1197	2718.39	8272.26	2167.15	45	319
EABT4111	1	0	11	0	0	0	0
EABT4112	1389	1678	1296.98	1178.1	1073.5	1247	1069.33
EABT4113	2	7	16	2	1	0	0
EABT4114	1	2	3	2	1	0	5
EABT4115	0	1	6	0	1	0	0
EABT4116	2242.12	2732.39	3613	6833.73	3316.96	2002.99	1833.09
EABT4117	795.11	1234.61	1234.04	2035.77	791.59	630	764.65
EABT4118	2465.89	4034.28	3285.45	9262.43	2639.63	1481.74	1694.04
EABT4119	1122.29	1185	1274.9	2877.3	326.05	993	516
EABT412	0	0	6	1	0	1	0
EABT4120	1	2	2	0	1	1	2
EABT4121	2	0	1	2	1	0	1
EABT4122	0	0	13	0	0	0	0
EABT4123	5994.86	5079.92	2461.69	1578.75	1338.38	7260.49	1430.32
EABT4124	1	0	9	6	4	1	0
EABT4125	0	10	41	3	2	1	1
EABT4126	0	3	2	1	2	0	0
EABT4127	0	5	5	1	1	0	0
EABT4128	0	1	1	0	0	11	0
EABT4129	0	2	7	0	2	0	0
EABT413	1	8	11	19	1	0	1
EABT4130	1	3	11	0	0	0	0
EABT4131	0	0	3	0	0	0	0
EABT4132	2	8	21	3	3	1	5
EABT4133	16796.8	21950.4	85487.8	18808.7	24118.9	534.68	1113.58
EABT4134	241	305.89	264.13	353.01	253.86	204	210.04
EABT4135	0	0	3	0	0	0	1
EABT4136	183	259	64	287.56	179	117	141
EABT4137	1	7	5	7	2	1	1
EABT4138	9	4	51.32	1	0	1	0
EABT4139	0	3	43	2	0	0	0
EABT414	0	0	6	0	0	0	0
EABT4140	124	206	551	134.01	24	141	37
EABT4141	1	0	63	0	0	0	0
EABT4142	0	0	7	7	0	0	0
EABT4143	0	2	43.49	2	0	2	0
EABT4144	0	1	2	2	1	0	0
EABT4145	1584.14	1932.31	1341.68	3824.14	1675.37	1066.85	1402.65
EABT4146	14	17	10	55	11	4	2
EABT4147	6596.38	8760.9	6103.17	6769.87	5512.3	6142.5	7354.16
EABT4148	2	8	26	13	1	5	6
EABT4149	42	46	37	17	10	13	14
EABT415	1	7	7	2	0	3	1
EABT4150	0	0	3	3	0	0	1
EABT4151	519.08	1047.66	1053.84	3392.61	654.59	345	521.87
EABT4152	5	7	0	0	0	0	0
EABT4153	13	24	65	10	16	3	11
EABT4154	0	0	0	1	2	0	1
EABT4155	658	49	72	145	28	4	36

EABT4156	0	2	2	2	1	0	0
EABT4157	499.93	789.03	568	1075.09	361.98	307	267
EABT4158	2	1	18	1	1	0	0
EABT4159	20	19	0	0	3	58	26
EABT416	3	12	6	31	5	4	1
EABT4160	1	1	7	3	3	0	6
EABT4161	2	14	8	52	17	0	1
EABT4162	0	1	6	0	3	0	0
EABT4163	0	1	9	0	1	0	0
EABT4164	1127.98	1296.54	1105	2150.75	1040	1930.09	1107
EABT4165	784.38	1073.03	688.87	1040.62	847.81	362.01	324.06
EABT4166	0	3	7	0	0	1	0
EABT4167	1	2	3	4.94	4	0	1
EABT4168	1	2	3	1	0	0	0
EABT4169	6	8	2	4	2	4	2
EABT417	1318.71	588.17	140.99	841.91	40	14	24
EABT4170	0	0	6	0	0	0	0
EABT4171	246	111	0	27	57	20	52
EABT4172	0	1	4	4	1	0	0
EABT4173	2	8	22	15	0	4	3
EABT4174	0	2	2	0	2	0	0
EABT4175	2	0	6	0	0	0	0
EABT4176	8	9	20	11	5	8	3
EABT4177	356	534	307	403	491	323	257
EABT4178	0	1	6	1	0	0	0
EABT4179	4	23	11	20	3	8	6
EABT418	0	0	6	0	0	0	0
EABT4180	2	1	3	1	0	0	0
EABT4181	16.01	39	26	40	11	3	11
EABT4182	1	4	10	4	0	3	3
EABT4183	4	1	0	1	1107	1	0
EABT4184	5	2	3	0	0	2	0
EABT4185	73	76	127	170	428	4	171.94
EABT4186	2	5	67	2	0	0	1
EABT4187	543.68	770.93	435.13	824.27	479.08	403.71	305.34
EABT4188	0	0	3	2	0	1	1
EABT4189	0	0	0	0	0	0	0
EABT419	1	5	245	1	1	0	1
EABT4190	4	1	3	2	0	0	3
EABT4191	0	2	38.1	0	0	4	2
EABT4192	1	1	13	2	0	0	1
EABT4193	8	14	0	0	0	0	4
EABT4194	0	0	11	1	0	0	0
EABT4195	144.79	130.02	86	1121	10	96.54	24
EABT4196	5	0	6	0	0	2	1
EABT4197	0	1	3	3	1	0	1
EABT4198	0	2	3	12	2	1	0
EABT4199	4	1	21	16	10	0	1
EABT42	0	3	14	4	2	0	2
EABT420	1	0	8	0	1	1	0
EABT4200	2242.31	3760.09	1860.73	7698.24	1540.35	1273.39	1603.39

EABT4201	307	325	1771.39	1184.77	391	6	31
EABT4202	0	3	5	0	0	2	0
EABT4203	1	0	7	1	0	1	0
EABT4204	0	1	8	2	0	0	0
EABT4205	131	215	389	748.94	191	18	46
EABT4206	0	0	0	1	0	0	2
EABT4207	0	1	1	2	0	2	0
EABT4208	0	0	1	0	0	0	0
EABT4209	382	607	856.85	1384.02	390	330	332.24
EABT421	0	3	25	1	0	0	1
EABT4210	0	0	1	5	0	0	0
EABT4211	0	0	0	5	3	0	0
EABT4212	787.94	1094.99	773	129	799.86	1620.01	819.22
EABT4213	4	7	14	9	2	2	0
EABT4214	12274.7	7066.1	2169.32	11380.5	2810.66	2508.08	1308.78
EABT4215	2	4	11	1	0	0	0
EABT4216	78	130	77	80	75.95	2	3
EABT4217	3	11	18	6	4	6	1
EABT4218	4716.03	6989.46	2327.44	6283.23	3626.54	4541.65	4084.44
EABT4219	13	23	36	271	34	4	13
EABT422	1	1	3	2	0	0	0
EABT4220	1	2	12	2	0	0	0
EABT4221	0	0	3	4	0	0	1
EABT4222	0	2	18	1	0	0	0
EABT4223	1	1	1	25	1	0	0
EABT4224	2	8	13	2	1	0	0
EABT4225	1575.55	3278.16	3491.18	6243.22	1406.87	1494.73	1636.99
EABT4226	0	2	28	6	1	0	1
EABT4227	2	0	5	0	0	0	0
EABT4228	2	3	0	1	1	23	6
EABT4229	0	0	11	1	0	0	0
EABT423	1	3	10	19	1	2	2
EABT4230	25	26	101	19	41.01	4	2
EABT4231	0	0	2	11	0	0	0
EABT4232	0	6	7	8	2	0	0
EABT4233	6	6	1	1	0	3	0
EABT4234	19	44.25	61	15.63	10	20	22
EABT4235	2318.72	4378.78	3779.2	3634.18	1795.4	1338.6	1423.78
EABT4236	1399.97	2367.69	3432.11	3820.24	3097.23	731.94	1390.9
EABT4237	0	0	2	5	0	0	0
EABT4238	0	0	1	2	0	0	1
EABT4239	3	82	138.77	6	239	5	33
EABT424	0	0	2	0	2	1	0
EABT4240	1823.97	2904.59	3689.78	4516.71	1739.22	1845.67	2015.21
EABT4241	5	2.01	8	7	0	8	3
EABT4242	3	3	2	4	0	1	3
EABT4243	9	28.2	31	29	3	1	3
EABT4244	9	6	0	0	0	0	0
EABT4245	0	2	11	1	0	1	0
EABT4246	1698.13	2743.67	1723.38	5688.42	1478.53	1662	1535.97
EABT4247	6271.46	12777.4	13319.9	25349.9	5029.59	3727	4556.28

EABT4248	0	1	1	2	1	1	0
EABT4249	1423.38	1736.58	1035.03	1411.83	1690.97	503	100
EABT425	1404.45	1866.01	639.39	4194.91	949.71	712.85	884
EABT4250	2	6	9	19	7	2	4
EABT4251	1698.98	2080.18	1341.02	1911.76	1041.83	1595	1789.6
EABT4252	1	4	2	0	2	0	1
EABT4253	0	0	6	2	0	0	0
EABT4254	1	3	3	6	1	0	1
EABT4255	5	0	0	3	0	0	0
EABT4256	0	1	12	3	2	0	2
EABT4257	0	1	21	1	0	0	0
EABT4258	967.89	1960.95	2381	2051.52	1344.69	1044.03	1072.8
EABT4259	747.42	981	832	1181.05	488	520	524.76
EABT426	0	0	3	508	1	1	0
EABT4260	1020	1458.87	1152	2257.26	1147	1356.45	1026.17
EABT4261	4	8	0	14	6	6	10
EABT4262	0	0	3	2	1	0	0
EABT4263	180	845.08	1488.24	488	1062.46	90	389
EABT4264	3556.87	4017.12	1554.05	1857.65	3569.91	1855	1745.99
EABT4265	32	4	8	4	5	17	17
EABT4266	0	1	28	9	5	0	8
EABT4267	1	5	16	3	0	0	0
EABT4268	0	0	10	0	0	0	2
EABT4269	534	740.98	1190.98	995.93	681.48	817	546
EABT427	10	14	9	14	6	0	2
EABT4270	1855.62	4020.35	4785.79	6872.77	3774.32	1481.93	1087.56
EABT4271	0	4	1	3	0	0	0
EABT4272	3962.14	4089.08	4302.94	26676.2	3182.38	660	996
EABT4273	0	0	8	0	0	1	0
EABT4274	0	1	1	0	0	0	3
EABT4275	14	59	78	76	4	6	10
EABT4276	0	0	8	0	0	0	0
EABT4277	8138.88	12070.4	10219.4	21086.7	8772.31	7136.69	7890.27
EABT4278	0	1	1	3	1	0	0
EABT4279	0	4	10	13	0	0	0
EABT428	0	0	2	2	0	0	0
EABT4280	4770.52	4570.09	1586.01	3076.92	13983.1	4632.66	3685.02
EABT4281	0	6	6	3	4	2	6
EABT4282	0	0	0	8	1	0	0
EABT4283	3684	6615.15	11108.9	22520.6	3508.87	4041.04	2829.02
EABT4284	0	1	11	6	1	0	2
EABT4285	0	3	4	0	2	2	2
EABT4286	90	173.05	107.06	387.14	255.96	89.96	236.13
EABT4287	0	1	9	0	1	0	1
EABT4288	0	0	1	1	1	0	3
EABT4289	1	0	19	3	1	2	1
EABT429	654.62	1213.6	1451.21	1493.19	2733.79	533.87	370.22
EABT4290	1	1	9	0	6	5	10
EABT4291	6	5	11	1	3	3	12
EABT4292	29	49	102	412.02	333	16	16
EABT4293	0	3	4	0	0	0	0

EABT4294	630.56	1232.7	1290.56	2515.68	914.09	539.44	719.14
EABT4295	918.58	1122.27	950.52	1739.78	1058	1069.24	696
EABT4296	7403.16	3572.14	3928.16	5667.11	822.35	2299.18	1269.25
EABT4297	305.02	432	482.92	685.37	396.92	347.56	343.15
EABT4298	910	1966.27	1427.01	1664.17	1434.34	551	907
EABT4299	15	200	36	101	49	11	18
EABT43	1	6	30	3	1	0	0
EABT430	456.99	715.79	729.25	1009.74	472.09	443.06	489.04
EABT4300	3	2	42	132.01	7	2	19
EABT4301	1	4.12	4	8	0	0	0
EABT4302	0	0	6	0	0	0	0
EABT4303	1	0	4	1	0	0	0
EABT4304	3	2	28	2	0	0	0
EABT4305	1	1	5	35.98	62	0	14
EABT4306	5486.6	6879.25	3581.07	6730.93	3693	6642.75	5531.18
EABT4307	0	1	6	22	0	0	0
EABT4308	3206	4566	3734.93	5768.34	1587	2401.37	2154.29
EABT4309	1	2	0	9	3	0	0
EABT431	0	1	6	1	0	0	0
EABT4310	1624.17	3378.92	3562.14	6014.05	1754.27	1409.21	1607.62
EABT4311	7	14	18	19	14	6	4.01
EABT4312	0	4	11	3	0	0	0
EABT4313	0	1	0	7	1	0	0
EABT4314	0	0	0	1	0	0	0
EABT4315	3213.08	10885.2	11655.6	4958.75	6063.43	210	6116.04
EABT4316	8101.48	8098.23	4951.15	6816.34	6959.81	9908.5	7907.12
EABT4317	19	52	26	96	13	4	6
EABT4318	1778.19	2653.97	1729.98	2484.36	1281.33	1584.85	1896.19
EABT4319	363.98	171.99	124.99	267	46	3	0
EABT432	5	0	2	0	0	3	2
EABT4320	2	2	13	4	0	1	0
EABT4321	23	21	4	49	17	14	28
EABT4322	3	4	6	13	15	0	3
EABT4323	0	0	8	0	0	0	0
EABT4324	3197.1	2873.02	1138.58	2733.12	1402.21	2757.06	1620.82
EABT4325	2	0	2	0	1	1	0
EABT4326	0	2	0	3	1	0	0
EABT4327	1548.32	3138.6	865.94	1917.35	2811.71	1118.91	1596.62
EABT4328	0	1	5.04	2	0	0	0
EABT4329	1203.07	2316.06	597.02	2866.93	959.47	659.2	1570.56
EABT433	1	9	3	69	2	0	2
EABT4330	3	1	2	1	0	3	6
EABT4331	4551.9	9786.21	12265	9311.28	8892.97	7865.66	5947.46
EABT4332	0	1	34	1	3	0	0
EABT4333	0	0	14	3	0	0	0
EABT4334	1	2	3	6	20	0	4
EABT4335	0	0	0	0	1	0	7
EABT4336	0	2	1	0	3	0	3
EABT4337	0	2	1	0	1	1	0
EABT4338	69	50.41	82.87	17.77	13	175	59
EABT4339	0	0	6	0	0	0	0

EABT434	29	108	560.99	230	91	67	70
EABT4340	0	0	15	8	0	0	0
EABT4341	0	2	5	3	0	2	0
EABT4342	3	9	0	0	0	6	0
EABT4343	1642.2	1654.99	546.04	2185.82	1037.89	989.35	1193.61
EABT4344	5	5	7	33	371.05	3	1
EABT4345	1	0	7	1	0	0	0
EABT4346	1	1	1	1	1	0	0
EABT4347	6915.68	19493.2	16828.4	36538.3	5125.71	816.3	1234
EABT4348	589	1176.28	1129.36	2063.44	683	590.19	543.99
EABT4349	6326.83	5943.37	6985	19602.2	2040.46	317	782.97
EABT435	2	4	6	2	1	0	2
EABT4350	58	86	70	53	119	8	17
EABT4351	3	1	5	1	1	0	0
EABT4352	50	66	62	92	26	9	13
EABT4353	0	1	9	0	0	0	0
EABT4354	18.96	152.55	1364.66	317.64	62	31.48	45.09
EABT4355	2	1	1	7	0	1	6
EABT4356	4	6	25	8	3	3	9
EABT4357	0	4	40	0	0	1	0
EABT4358	0	0	6	0	0	1	0
EABT4359	0	0	17	1	1	0	0
EABT436	2	7	7	0	0	2	1
EABT4360	0	1	7	1	1	0	0
EABT4361	18	25.71	30	34	2	16	6
EABT4362	0	0	5	1	1	0	1
EABT4363	0	4	3	36	3	0	0
EABT4364	0	0	2	2	8	0	0
EABT4365	0	1	2	1	0	1	0
EABT4366	1	4	7	1	1	0	0
EABT4367	157	591	242	177	782	13	481.67
EABT4368	0	2	23	1	0	0	0
EABT4369	18	28	7	52	12	9	7
EABT437	0	0	4	1	0	0	1
EABT4370	2135.63	2674.87	2736	2998.13	1557	1561	1484
EABT4371	0	0	15	0	0	1	0
EABT4372	3	6	12	9	2	2	3
EABT4373	0	0	0	0	10	2	8
EABT4374	0	2	7	72	0	0	0
EABT4375	27	20	16	17	2	9	0
EABT4376	0	0	3	0	1	0	0
EABT4377	0	0	0	0	0	3	5
EABT4378	0	1	7	3	3	0	1
EABT4379	10	18	4	28	21	2	15
EABT438	0	2	3	3	0	0	0
EABT4380	398	558	213	471	488	26	77
EABT4381	3	1	0	0	0	1	2
EABT4382	2	6	1	0	0	3	0
EABT4383	137.68	136.98	23	2	15	0	4
EABT4384	0	5	5	9	0	4	0
EABT4385	71	109.82	23	20	34.89	9	12.15

EABT4386	0	0	1	1	2	0	0
EABT4387	0	1	7	1	0	0	0
EABT4388	5	4	18	11	3	1	2
EABT4389	558.4	697.93	929.9	1278.33	742.16	1086.38	971.54
EABT439	1	9	14	19	0	3	2
EABT4390	1	5	16	1	0	0	0
EABT4391	2	5	16	4	3	2	3
EABT4392	0	2	3	0	1	0	0
EABT4393	0	0	41	6	1	0	0
EABT4394	19	24	2	5	2	1	0
EABT4395	0	1	7	6	2	0	0
EABT4396	0	0	0	1	3	0	1
EABT4397	0	2	4	0	0	0	0
EABT4398	1	10	43	8	5	0	1
EABT4399	0	0	1	0	1	0	1
EABT44	3	0	0	1	0	17	1
EABT440	6	12	10	2	1	8	5
EABT4400	8	2	0	0	0	9	3
EABT4401	0	0	3	3	2	1	1
EABT4402	0	6	1	41	9	1	3
EABT4403	13	6	16	5	5	3	11
EABT4404	12	5	8	11	5	0	3
EABT4405	0	0	1	0	0	0	0
EABT4406	0	0	0	0	5	1	5
EABT4407	11	20	29	34	7	14	24
EABT4408	0	1	2	5	1	0	0
EABT4409	28	42	24	32	30	13	17
EABT441	0	0	7	0	0	0	0
EABT4410	2026.44	1951.76	1848.34	2175.03	1533.36	3011.27	2459.54
EABT4411	75	259.28	764	1744.94	611	32.98	35
EABT4412	0	0	1	0	2	0	0
EABT4413	25	27	60	56	74.44	11	22
EABT4414	248	511.74	675.23	1584	1078.58	242	474.01
EABT4415	4	3	11	8	5	1	1
EABT4416	1	0	7	1	0	0	0
EABT4417	0	0	3	0	0	0	1
EABT4418	3	3	49	7	4	6	3
EABT4419	1	3	3	6	1	0	2
EABT442	1	6	3	10	2	0	0
EABT4420	0	0	13	0	0	0	0
EABT4421	2	4	8	2	3	5	0
EABT4422	2	18	31	7	10	1	3
EABT4423	10	30.99	30	164	445	5	14
EABT4424	1	2	4	2	0	0	1
EABT4425	0	0	20.98	0	0	0	0
EABT4426	0	2	1	1	1	1	0
EABT4427	2	7	18	1	4	0	0
EABT4428	2	7	10	17	2	2	2
EABT4429	0	0	4	0	3	0	0
EABT443	7	15	12	20	5	4	4
EABT4430	0	1	10	0	0	1	1

EABT4431	0	2	3	1	1	1	1
EABT4432	4441.31	4913.04	4562.56	3756.38	2557.45	4451.08	3861.77
EABT4433	2754.8	3168.98	2780.4	5906.91	1690	2512.04	1427.03
EABT4434	12	13	0	0	0	31	9
EABT4435	0	1	3	7	0	0	0
EABT4436	1	0	16	1	0	2	0
EABT4437	222	90	40	360	758.78	43	2977.98
EABT4438	0	0	8	0	0	0	0
EABT4439	0	0	20	0	0	0	0
EABT444	1	1	4	1	1	0	1
EABT4440	364	499	194	581	450.16	579.92	389.39
EABT4441	0	1	7	0	0	0	0
EABT4442	2	10	16	15	7	0	2
EABT4443	14553.6	15578.3	1305.65	2815.83	5621.91	3516.56	4167.08
EABT4444	1	5	0	0	0	6	0
EABT4445	416.35	659.41	794	1004	413	611	528.04
EABT4446	1	3	7	0	0	0	0
EABT4447	2	3	14	0	0	0	1
EABT4448	1153.67	1904.26	2238.34	3217.63	1315.71	901.36	1133.27
EABT4449	9	6	115	3	8	0	0
EABT445	0	0	2	1	0	0	0
EABT4450	0	0	2	0	0	1	10
EABT4451	2	0	23	2	1	0	0
EABT4452	1	0	1	4	1	5	1
EABT4453	1	0	2	0	0	2	0
EABT4454	0	1	6	1	0	0	1
EABT4455	8	11	18	0	37	0	8
EABT4456	0	0	0	2	0	1	2
EABT4457	3	3	16	1	0	1	1
EABT4458	1	0	5	6	0	2	0
EABT4459	1	0	4	1	0	0	6
EABT446	2	9	17	3	4	0	7
EABT4460	0	0	7	0	0	0	0
EABT4461	5	3	0	0	0	0	1
EABT4462	0	0	12	2	0	1	0
EABT4463	1	9	12	31	1	0	0
EABT4464	0	4	2	0	0	0	0
EABT4465	62	188	219.04	1112.54	29	8	11
EABT4466	0	0	1	2	1	0	0
EABT4467	0	2	0	1	0	2	0
EABT4468	1756.07	2423.27	1633.12	2032.87	1224.93	1505.57	1301.03
EABT4469	1	1	5	3	1	1	3
EABT447	3813	2121.95	134.16	1	639	2766.13	5066.09
EABT4470	0	0	91	0	0	0	0
EABT4471	1	1	23	1	1	0	1
EABT4472	10097.3	33867.6	8691.36	7426.1	11269	7157.94	12776.6
EABT4473	0	0	0	3	6	0	2
EABT4474	162	184.09	1040.88	1861.32	806.97	38	112
EABT4475	0	2	6	0	0	0	0
EABT4476	1	0	42	1	0	0	0
EABT4477	0	4	8	30	8	0	2

EABT4478	0	0	8	0	0	0	0
EABT4479	1893.87	3046.98	4686.89	6383.43	2757.84	3904.6	3230.9
EABT448	1	6	1	0	0	0	0
EABT4480	20	35	0	0	0	13	1
EABT4481	0	1	2	0	2	2	1
EABT4482	3	9	2	0	22	1	1
EABT4483	4	0	3	0	0	1	0
EABT4484	5	4	3	1	0	1	2
EABT4485	214.01	107	1	0	0	783.03	8
EABT4486	1675.41	2665.28	1965.47	3561.6	1576.95	1405.54	1443.04
EABT4487	457	1118.08	860	2667	1515	29	85
EABT4488	1	3	17	3	1	0	0
EABT4489	0	1	3	1	1	0	0
EABT449	1	9	2.04	9	2	1	0
EABT4490	0	1	0	1	2	0	0
EABT4491	1	0	8	0	0	1	0
EABT4492	6	2	17	5	11	5	84
EABT4493	9	11	23	5	15	0	2
EABT4494	7	5	17	6	0	0	3
EABT4495	3	2	16	3	2	4	2
EABT4496	1	2	27	0	1	2	0
EABT4497	2	5	23	20	4	6	2
EABT4498	1	0	31	0	0	2	0
EABT4499	0	6	4	4	2	0	1
EABT45	660.04	1666.95	2432.13	3838.78	1539.36	1459.95	1273.98
EABT450	0	0	11	0	0	1	0
EABT4500	0	1	13	1	1	1	0
EABT4501	188	14366.4	16	9	11	1321	14
EABT4502	6	8	15	18	6	1	2
EABT4503	1	1	6	6	7	2	1
EABT4504	3457.13	5857.38	3805.3	14294.7	2606.1	1577	2318.07
EABT4505	0	2	4	3	2	0	2
EABT4506	3	4	0	0	0	3	2
EABT4507	0	0	6	0	0	1	0
EABT4508	2	1	1	4	1	0	1
EABT4509	710.92	828.4	411.81	1534	514.01	534.34	1341.29
EABT451	2	4	3	5	1	2	2
EABT4510	2	17	12	36	4	2	5
EABT4511	109	88	38.99	384	104	63	55
EABT4512	0	6	6	0	0	0	1
EABT4513	0	0	13	1	0	1	0
EABT4514	0	1	31	3	2	0	2
EABT4515	0	0	3	3	0	1	0
EABT4516	0	0	7	0	0	0	0
EABT4517	0	1	3	6	2	2	0
EABT4518	3	1	2	1	0	0	0
EABT4519	928.01	1044.98	1218.38	272.07	987.96	866.18	492
EABT452	1	3	17	5	20	0	0
EABT4520	2	5	7	1	0	3	0
EABT4521	10281.3	14306.5	10216.4	11173.1	7743.65	6372	10822.6
EABT4522	490.01	528	207.64	400.6	721.65	245.01	132

EABT4523	0	1	0	1	0	0	0
EABT4524	0	0	5	0	0	0	0
EABT4525	0	1	1	0	0	0	3
EABT4526	0	0	5	1	2	0	1
EABT4527	0	4	41	1	0	0	0
EABT4528	11082.1	16226.1	23515.5	13394.1	11129.5	13037.9	8769.61
EABT4529	23597.2	687	1	0	1	2360.87	1
EABT453	11	11	8	33	12	1	1475.36
EABT4530	0	4	3	0	0	0	0
EABT4531	11.87	16	54	11	5	5	3
EABT4532	16	34	38	328	18	0	37
EABT4533	23	20	0	0	5	54	36
EABT4534	2	0	11	1	0	0	1
EABT4535	2	3	3	0	1	6	1
EABT4536	12	10	11.05	3	5	1	2
EABT4537	1790.4	2824	933.52	3526	1331.63	1457	1908
EABT4538	1	4	7	11	16	3	4
EABT4539	0	3	4	1	0	1	0
EABT454	0	2	7	0	0	0	1
EABT4540	8	1	0	0	3	6	3
EABT4541	0	5	2	0	0	0	0
EABT4542	1	1	2	1	0	0	0
EABT4543	0	0	5	0	0	0	0
EABT4544	1	2	15	1	1	0	1
EABT4545	3	0	1	0	4	7	14
EABT4546	2	3	19	7	0	0	1
EABT4547	0	0	2	3	0	0	0
EABT4548	1	9	15	7	0	3	6
EABT4549	8	99	148	755.95	29	3	3
EABT455	0	1	4	0	0	0	0
EABT4550	0	0	1	4	0	0	1
EABT4551	2	0	2	5	4	0	2
EABT4552	0	5	10	6	1	0	0
EABT4553	4	3	11	1	2	1	1
EABT4554	387	577	1041.62	1456	810	363.03	581
EABT4555	3	6	13	0	0	0	0
EABT4556	5772.52	5740.27	9105.6	2378.8	14087.8	4897.08	2375.59
EABT4557	1018.01	1368.29	1983.42	1478	869	957.08	566.96
EABT4558	1	1	0	3	1	0	1
EABT4559	5	10	54	24	0	3	1
EABT456	0	2	16	3	0	0	0
EABT4560	0	0	12	0	0	0	0
EABT4561	1	5	15	5	2	4	2
EABT4562	1	0	8	0	0	0	0
EABT4563	14	15	0	0	0	36	33
EABT4564	2	5	11	0	5	4	8
EABT4565	6	23	28	5	25	10	0
EABT4566	0	0	0	21	0	0	0
EABT4567	0	0	5	0	0	1	0
EABT4568	0	1	4	9	0	0	0
EABT4569	3126.69	1390.6	1118.22	1234.26	127	2707.01	479.06

EABT457	8	15	86	10	2	2	3
EABT4570	5	5	9	2	0	3	0
EABT4571	0	0	0	0	1	1	3
EABT4572	0	0	21	0	1	0	0
EABT4573	0	1	9	0	0	1	2
EABT4574	2	7	6	43	39	0	0
EABT4575	0	1	12	1	0	0	0
EABT4576	31	2	0	0	0	10	0
EABT4577	230.62	34	121	483.22	93	15	10
EABT4578	0	0	11	3	0	0	0
EABT4579	4	2	26	10	14	0	0
EABT458	1	0	2	0	72	9	215
EABT4580	1181.05	1834.15	1005.48	566.61	568.97	224.43	163.46
EABT4581	1	24	1	0	0	18	0
EABT4582	0	2	4	0	0	0	2
EABT4583	3	0	0	0	0	2	1
EABT4584	2	5	5	9	1	2	0
EABT4585	4945.14	4800.19	89	177	1296.25	9400.01	1256
EABT4586	4162.02	4322.07	2031.36	2685	2276.27	2804.34	1775
EABT4587	11	14	18	9	2	0	1
EABT4588	3	4	12	8	5	0	9
EABT4589	4	4	21	1	0	5	0
EABT459	3862.81	4132.59	1362.75	3038.27	3709.54	343.04	319.31
EABT4590	1	2	2	8	1	0	0
EABT4591	1	0	6	1	2	0	1
EABT4592	5371.43	3960.13	129	142	303.75	3536.69	1191.25
EABT4593	2939.42	4177.58	4650.51	9271.35	2958.33	3330.6	2649.68
EABT4594	0	0	5	5	2	0	0
EABT4595	0	3	6	0	0	0	0
EABT4596	3	5.95	191	64	0	4	13
EABT4597	8	35	36	54	24	0	3
EABT4598	0	0	4	2	0	0	0
EABT4599	0	0	7	5	1	2	0
EABT46	0	1	4	0	0	0	0
EABT460	8	6	9	8	11	0	1
EABT4600	361.51	1334.03	2097.39	312	1307.59	8	235
EABT4601	1	4	0	0	0	1	0
EABT4602	2	0	6	1	0	0	0
EABT4603	0	0	15	0	0	0	0
EABT4604	3	3	1	0	0	3	5
EABT4605	0	6	15	3	1	1	0
EABT4606	0	0	30	1	0	1	0
EABT4607	6	3	0	0	0	6	0
EABT4608	10	30	12	16	11	0	1
EABT4609	1	1	9	1	0	0	0
EABT461	3	2	2	4	0	3	0
EABT4610	201	590.68	32.2	18	25	397.66	96
EABT4611	0	1	2	4	1	0	1
EABT4612	3	2	12	4	1	2	0
EABT4613	3	2	5	0	0	1	0
EABT4614	1	2	16	1	0	0	0

EABT4615	1	0	1	0	0	1	0
EABT4616	0	0	7	1	0	0	0
EABT4617	0	1	6	3	1	1	4
EABT4618	1	3	0	2	0	0	0
EABT4619	0	0	4	0	0	0	0
EABT462	19	21	62	17	18	44	25
EABT4620	24	56	49	64	60	10	23
EABT4621	0	1	3	0	0	1	0
EABT4622	123	40	26	19	16	29	65.44
EABT4623	0	0	8	2	0	0	0
EABT4624	0	1	1	0	0	5	0
EABT4625	4	3	19	1	6	0	0
EABT4626	0	0	1	0	0	2	0
EABT4627	0	3	0	2	1	0	4
EABT4628	2	7	3	8	5	1	11
EABT4629	3	14	5	4	8	1	2
EABT463	0	1	7	6	1	0	0
EABT4630	2	7	4	11	4	5	5
EABT4631	0	4	88	5	3	0	0
EABT4632	0	1	0	2	4	11	3
EABT4633	0	7.72	2	3	2	0	0
EABT4634	1	0	13	0	1	0	0
EABT4635	0	1	5	4	0	0	1
EABT4636	0	0	2	2	0	2	0
EABT4637	8	14	2	2	0	0	0
EABT4638	5	5	6	2	0	2	1
EABT4639	0	0	11	0	0	0	0
EABT464	0	2	7	8	0	0	0
EABT4640	0	3	6	8	1	1	1
EABT4641	0	0	2	0	0	0	0
EABT4642	12	13	51	18	7	12	6
EABT4643	0	0	6	0	0	1	0
EABT4644	9	5	44	6	0	21	11
EABT4645	1	1	41	2	1	1	1
EABT4646	22477.8	25061.2	23559.4	17980.8	22167	19537.4	18058.3
EABT4647	1	1	4	0	0	1	0
EABT4648	5	4	3	1	0	1	1
EABT4649	2134.87	3638.63	1228.03	1923.31	1397.91	985.54	964.67
EABT465	1018.44	1706.9	1590.01	1717.01	1932	1011	1327.54
EABT4650	0	4	1	1	3	0	0
EABT4651	0	0	0	3	0	0	0
EABT4652	7	11	0	0	0	0	0
EABT4653	0	2	3	6	1	1	1
EABT4654	1	0	18	0	1	0	2
EABT4655	69	126	108	164	31	54	78
EABT4656	0	2	10	2	0	1	0
EABT4657	0	0	4	2	0	0	0
EABT4658	0	0	3	1	0	0	0
EABT4659	3	3	5	0	2	0	2
EABT466	2	5	7	6	0	0	0
EABT4660	76.97	157.91	219.25	443	78	74	79

EABT4661	1	4	11	2	2	0	1
EABT4662	2	0	3	1	0	0	5
EABT4663	1	5	2	7	2	2	1
EABT4664	1	2	43	3	0	0	0
EABT4665	1	0	7	0	0	0	0
EABT4666	402	713.12	602.63	2862.08	702.15	181	284.06
EABT4667	34805.8	12064.5	352	34	15194	66607.6	125700
EABT4668	2	5	5	4	1	1	0
EABT4669	4621.67	7721.53	5098.91	5563.99	3907.37	3870.59	4295.95
EABT467	2	0	0	0	0	0	4
EABT4670	5	5	0	0	9	5	12
EABT4671	0	0	10	4	0	0	1
EABT4672	5	11	10	20	2	5	9
EABT4673	2	1	0	0	0	10	0
EABT4674	1	0	11	3	0	0	0
EABT4675	0	1	7	2	1	1.58	1
EABT4676	2402.94	3824.89	4728.84	4327.37	4740.08	1509.55	1736.84
EABT4677	2	6	6	3	0	2	7
EABT4678	0	0	10	1	0	0	2
EABT4679	0	0	2	2	1	0	0
EABT468	0	1	7	0	0	0	1
EABT4680	5	5	56	4	0	3	0
EABT4681	0	1	0	17	0	0	0
EABT4682	0	0	7	0	0	0	0
EABT4683	23	26	0	0	1	9	11
EABT4684	0	1	2.29	0	0	0	2
EABT4685	2	2	0	0	0	3	1
EABT4686	493.31	1128.97	1722.09	697.1	468	16	105.03
EABT4687	0	1	3	2	2	0	0
EABT4688	356.54	700.78	286	769.94	576.41	204.02	419.05
EABT4689	7	5	14	2	1	2	1
EABT469	4	4	19	0	0	0	0
EABT4690	0	9	6	2	0	0	1
EABT4691	0	0	9	0	0	0	0
EABT4692	0	2	10	1	0	0	0
EABT4693	0	2	14	5	2	0	4
EABT4694	0	0	0	0	0	1	0
EABT4695	0	2	15	0	0	0	0
EABT4696	350.79	584.98	668.43	1334.2	502.57	388	492
EABT4697	0	4	1	0	0	0	0
EABT4698	0	6	4	3	2	1	0
EABT4699	1	11	3	31	0	3	2
EABT47	6514.74	4927.33	175.76	102	1059.38	6124.28	5425.99
EABT470	10	31	123	36	7	18	7
EABT4700	6	6	18	2	1	0	1
EABT4701	0	1	1	7	4	0	0
EABT4702	5	39	52	303	64	9	20
EABT4703	1	1	2	3	1	2	1
EABT4704	2	2	4	4	0	0	0
EABT4705	19	47	106	84	2	5	1
EABT4706	0	0	1	0	0	0	1

EABT4707	1	1	5	1	0	0	0
EABT4708	2	1	0	0	0	4	0
EABT4709	111197	38163.6	87	50	47.73	166569	250
EABT471	0	2	11	2	0	0	0
EABT4710	3848.01	1651.01	4353.9	715	1163	2813.43	1971.28
EABT4711	0	0	4	3	0	0	0
EABT4712	1	12	2	0	4	47	13
EABT4713	4	8	3	1	0	4	7
EABT4714	0	5	29	16	2	0	0
EABT4715	3	3	2	0	1	1	1
EABT4716	11	25	6	27	6	1	0
EABT4717	1	0	16	0	0	1	1
EABT4718	3	6	19.79	12	1	1	0
EABT4719	2	5	0	5	1	2	4
EABT472	0	2	13	0	1	1	1
EABT4720	1843.45	3093.52	3422.26	6941.8	1766.94	1574.78	1537.36
EABT4721	0	0	3	5	0	0	1
EABT4722	6	19	13	29	23	1	8
EABT4723	1	7	27	0	86	51	1
EABT4724	0	0	4	0	0	0	0
EABT4725	1	0	27	0	1	0	0
EABT4726	1	3	2	3	0	0	2
EABT4727	2	2	2	5	1	1	0
EABT4728	2275.91	3319.46	3721.06	4779.02	2947.21	2267.05	1872.23
EABT4729	6	2	0	0	0	6	4
EABT473	0	1	9	1	0	0	0
EABT4730	2	2	2	6	0	10	1
EABT4731	10	10	0	0	0	0	0
EABT4732	0	0	4	1	0	0	0
EABT4733	0	2	1	0	4	0	7
EABT4734	1	7	5	3	4	1	2
EABT4735	544	1015.61	812	2491.26	1096.99	473	533.92
EABT4736	5	9	1	6	2	1	0
EABT4737	1179.47	1525.06	1032.16	2438.06	1478.93	1337.28	1173.07
EABT4738	3191.01	3772.02	2809.19	4502.03	2690.88	3282.67	3640.04
EABT4739	4	3	10	3	0	0	0
EABT474	514	1296.93	2274.54	1083.18	676	254	272
EABT4740	0	2	6	2	2	0	1
EABT4741	0	0	11	0	0	0	0
EABT4742	18	41	304.51	28	19	31	32
EABT4743	0	1.99	1	2	0	0	0
EABT4744	10.02	7	6	6	2	26	18
EABT4745	0	0	5	8	0	0	0
EABT4746	0	1	4	8	2	0	1
EABT4747	39	44	77	26	15	42	83
EABT4748	0	0	0	0	3	2	4
EABT4749	0	0	2	2	2	0	0
EABT475	0	1	6	0	0	0	0
EABT4750	9114.28	8608.68	6758.47	8965	4216.88	2219.51	1408
EABT4751	2	0	8	0	0	0	0
EABT4752	1	10	38	9	5	2	3

EABT4753	13	15	25	2	1	0	0
EABT4754	0	0	0	0	0	0	2
EABT4755	0	0	3	4	0	0	0
EABT4756	7	21	36	267	48	0	3
EABT4757	0	2	6	3	2	1	0
EABT4758	3294.86	3285.08	370	31	543	3710.01	2569
EABT4759	1	1	9	1	0	0	0
EABT476	0	0	0	1	3	0	3
EABT4760	0	0	1	7	25	0	3
EABT4761	3	2	0	0	1	0	5
EABT4762	0	0	3	2	0	0	0
EABT4763	98	143.79	135	388	164	62	84
EABT4764	4364.74	5383.71	2336	6298.71	1999.08	3905	3673.03
EABT4765	2	7	1	1	0	1	1
EABT4766	5451.42	8445.9	7238.03	10611.5	3415.4	3108.99	3506.92
EABT4767	0	1	6	0	0	0	0
EABT4768	14	9	30	1	4	6	6
EABT4769	3	6	157	0	7	3	5
EABT477	442.99	768.46	726	2239.28	814.13	469.71	484.07
EABT4770	14	3	10	11	17	3	8
EABT4771	2577.18	3269.34	2282.36	3477.77	2414.94	3322.52	2606.03
EABT4772	3	11	7	19	3	1	0
EABT4773	1	0	1	0	0	0	0
EABT4774	1	0	19	0	1	0	0
EABT4775	0	0	3	0	1	0	19
EABT4776	0	3	0	9	0	2	0
EABT4777	9	25	44	17	3	10	5
EABT4778	0	1	15	1	0	0	1
EABT4779	3	5	3	11	4	3	1
EABT478	1	0	0	1	0	0	0
EABT4780	0	0	1	3	1	0	0
EABT4781	0	2	3	2	0	0	0
EABT4782	198.1	288	228	224	219	23	101
EABT4783	143.82	98	363.01	110.18	39	12	38
EABT4784	3	3	0	0	0	0	0
EABT4785	2	3	4	0	8	0	2
EABT4786	0	0	6	1	1	0	1
EABT4787	0	11	23	20	12	0	1
EABT4788	0	2	7	4	0	0	0
EABT4789	60687.8	80180.9	28838.1	69808	39261.5	15	488
EABT479	22	43	31	58	35	12	14
EABT4790	9	8	37	0	2	2	3
EABT4791	0	7	1	11	1	0	0
EABT4792	13	18	0	8	9	0	0
EABT4793	2	11	2	4	0	0	4
EABT4794	1	5	1	10	2	1	1
EABT4795	0	1	0	0	0	15	0
EABT4796	2	0	6	1	1	0	0
EABT4797	1	3	4	0	0	2	0
EABT4798	2465.61	2848.83	3570.17	7164.79	1481.75	1385.97	1276.53
EABT4799	1	2	1	1	0	0	0

EABT48	1014.05	1986.29	2639.99	4809.96	1438.42	888.19	963.02
EABT480	1	2	2	1	0	1	0
EABT4800	16	78	88	199.84	331.02	10	33
EABT4801	613	1636	692	1231.6	696	6	9
EABT4802	0	0	12	0	0	1	0
EABT4803	4	6	14	0	3	1	9
EABT4804	2	5	1	2	2	4	0
EABT4805	0	5	13	8	10	0	1
EABT4806	2386.14	3423.96	1488.76	7215.88	2260.01	1720.26	2615.29
EABT4807	1	0	1	10	3	0	0
EABT4808	5	0	7	2	0	0	0
EABT4809	0	0	6	0	0	2	0
EABT481	639.3	1093.91	840.55	2176.05	1026.99	463.08	529.03
EABT4810	0	3	9	0	0	0	0
EABT4811	591	1369.62	2314.44	598	927	114	1354.44
EABT4812	1043.37	7244.97	3844.32	174.3	1574.91	90	1049.51
EABT4813	1	3	10	0	0	0	2
EABT4814	0	2	4	3	0	0	0
EABT4815	1	0	8	0	0	0	0
EABT4816	10	7	33	13.89	9	7	7
EABT4817	1191.11	1846.22	2025.76	13	7485.94	15	407.85
EABT4818	0	0	0	0	5	0	0
EABT4819	311.17	346	391	713	475	244	258
EABT482	0	3	17	3	1	0	1
EABT4820	1	0	22	0	0	0	1
EABT4821	0	0	0	6	0	0	0
EABT4822	0	1	0	81	5	0	0
EABT4823	0	1	8	0	1	0	1
EABT4824	0	0	3	3	0	0	0
EABT4825	0	1	28	0	200.38	0	1
EABT4826	0	1	1	2	1	15	3
EABT4827	1	2	0	18	2	0	0
EABT4828	0	1	4	1	0	0	0
EABT4829	1	2	2	4	1	0	3
EABT483	1	0	17	2	0	0	0
EABT4830	7202.72	3031.68	66	0	52	7734.07	1260.06
EABT4831	0	1	5	2	0	0	0
EABT4832	140.91	3914.33	1905.8	451	409	337	281.97
EABT4833	0	1	11	2	4	0	1
EABT4834	0	5	26	12	1	0	2
EABT4835	0	0	0	5	0	1	0
EABT4836	35	69	275.23	46	33	12	13
EABT4837	0	2	4	6	0	1	0
EABT4838	680.73	1140.93	256.59	833	95.18	304	428.92
EABT4839	3	12	32	8	2	3	5
EABT484	0	2	5	4	1	0	0
EABT4840	85	156	94	205	95	71	106
EABT4841	1	0	5	3	6	0	1
EABT4842	2	1	3	2	3	0	0
EABT4843	5	9	0	0	0	0	0
EABT4844	6	5	5	6	0	0	0

EABT4845	2	7	2	0	2	4	0
EABT4846	2	0	6	0	1	0	0
EABT4847	0	3	10	1	10	0	0
EABT4848	39243.1	32432.7	27826.7	7996.24	12736.1	49187.4	33716.1
EABT4849	313.07	522.79	457.37	1429.52	693.01	59	162.87
EABT485	2	5	10	7	0	3	2
EABT4850	0	2	3	3	1	0	0
EABT4851	1612.22	2401.16	1624.31	4286.36	2209.11	1274.66	1693.31
EABT4852	2	4	2	1	3	1	4
EABT4853	5	2	4	0	0	0	0
EABT4854	2	8	25	3	0	5	6
EABT4855	0	0	19	19	1	0	1
EABT4856	2	2	3	2	0	0	2
EABT4857	19	51	53	87	8	4	9
EABT4858	15	9	33	2	1	0	1
EABT4859	6	11	5	1	2	6	1
EABT486	179	565.02	675.83	1504	622.01	25	41
EABT4860	3	8	1	0	4	29.69	21
EABT4861	2328.82	2970.74	3736.68	4680.82	2667.06	1405.88	1549.35
EABT4862	1	1	3	0	2	0	0
EABT4863	0	3	6	5	0	0	0
EABT4864	0	1	3	1	1	1	0
EABT4865	1	0	14	1	0	0	0
EABT4866	7	0	0	0	1	3	0
EABT4867	6016.38	6665.23	3309.37	6656.18	3037.81	3722.22	3473.27
EABT4868	0	0	8	3	0	0	0
EABT4869	0	3	2	1	1	0	2
EABT487	0	0	4	0	1	0	0
EABT4870	1084.01	1547.98	1003.98	2648.98	636	803	820.97
EABT4871	4874.21	5188.56	4941.4	8430.95	3406.32	4826.79	2843.98
EABT4872	0	6	6	14	10	1	1
EABT4873	10	8	0	0	0	8	2
EABT4874	1	0	8	8	1	1	0
EABT4875	0	2	5	0	1	0	1
EABT4876	7465.61	7322.18	5043	5999	6222	9207.98	6638.05
EABT4877	0	2	13.34	0	0	0	0
EABT4878	28	19	48	61	37	61	122
EABT4879	0	1	0	0	0	4	2
EABT488	5729.92	4528.78	1857.25	3642.47	8256.98	4126.57	3229.06
EABT4880	0	0	11	0	0	0	0
EABT4881	8.45	30	22	22	7	13	9
EABT4882	0	2	112	0	0	0	0
EABT4883	6615.75	6783.23	5324.97	7339.99	2998	6158.03	6046.19
EABT4884	1	3	1	27	2	0	2
EABT4885	43	25	2	0	2	11	6
EABT4886	0	0	21	2	1	1	1
EABT4887	1	4	0	1	0	0	0
EABT4888	0	2	4	5	3	0	3
EABT4889	0	0	11	0	0	0	0
EABT489	74	199.15	58.95	350.44	341	115	189
EABT4890	2	7	31	0	1	1	2

EABT4891	1	5	0	4	0	2	0
EABT4892	2	14.99	5	7	16	0	16
EABT4893	0	1	1	5	1	0	0
EABT4894	0	1	4	1	2	0	0
EABT4895	41	144	250	425	151	71	70
EABT4896	0	2	8	1	1	0	0
EABT4897	1	4	7	4	2	3	2
EABT4898	3	3	31	0	0	2	0
EABT4899	0	0	9	4	0	0	0
EABT49	0	0	2	0	0	0	1
EABT490	0	0	1	0	0	0	1
EABT4900	4	9	7	14	8	1	9
EABT4901	3	45	3	0	0	38	0
EABT4902	0	5	6	2	1	3	0
EABT4903	3	9	67	8	2	0	0
EABT4904	0	1	8	1	0	0	1
EABT4905	22	20.97	14	4	0	3	1
EABT4906	0	1	6	0	0	0	0
EABT4907	0	1	4	0	0	0	0
EABT4908	3	32.02	9	11	4	0	4
EABT4909	0	1	2	0	0	0	0
EABT491	0	1	5	0	0	0	0
EABT4910	87.02	67	23	2	7	22	17
EABT4911	0	0	2	0	1	0	0
EABT4912	458	640.01	1223.73	1270.94	820.33	417.57	325.02
EABT4913	0	1	2	2	0	1	1
EABT4914	6615.94	4198.09	2111.24	4755.23	4990.88	4924.29	4249.06
EABT4915	0	1	26	1	0	0	0
EABT4916	0	3	8	3	4	0	0
EABT4917	0	2	15	1	0	0	0
EABT4918	0	0	7	0	1	0	0
EABT4919	0	2	0	0	0	0	0
EABT492	0	2	25	0	0	2	0
EABT4920	1	1	3	1	19	10	10
EABT4921	0	1	1	5	0	0	0
EABT4922	2691.81	3599.79	660	138	432.07	2331.77	1227.7
EABT4923	120	143	171	288.04	141	128.23	126
EABT4924	1	9	45	10	2	0	1
EABT4925	0	5	0	0	0	1	2
EABT4926	5	18	40	31	7	6	4
EABT4927	1369	1944	1953	1421	1114	1224	941.15
EABT4928	2	6.98	8	0	1	1	1
EABT4929	808	1522.65	923	1301	1217	749.99	626.05
EABT493	2	12	16	15	3	2	9
EABT4930	0	6	4	6	0	2	5
EABT4931	1	3	2	3	1	0	1
EABT4932	1	5	0	21	2025.42	0	1
EABT4933	0	0	1	2	1	0	0
EABT4934	0	0	9	1	0	0	0
EABT4935	1	0	9	1	0	0	0
EABT4936	2	5	3	0	2	31	16

EABT4937	0	0	11	10	1	0	0
EABT4938	22	9	1	0	0	46	2
EABT4939	2	2	3	1	3	0	4
EABT494	14	16	11	79	10	5	24
EABT4940	1	2	21	34	1	0	1
EABT4941	0	4	3	2	1	0	0
EABT4942	5	4	2	2	0	10	4
EABT4943	0	12	1	0	0	7	0
EABT4944	0	0	2	3	5	0	0
EABT4945	0	0	2	0	3	0	0
EABT4946	0	1	5	1	0	1	0
EABT4947	8	12	13	27	5	3	5
EABT4948	0	1	11	0	0	0	0
EABT4949	2	2	4	10	2	1	0
EABT495	0	4	0	0	2	1	1
EABT4950	2	60	14	5	1	3	2
EABT4951	0	1	6	0	0	0	0
EABT4952	1	5	5	0	0	1	2
EABT4953	2	8	5	5	0	1	1
EABT4954	7	6	7	8	17	4	7
EABT4955	2	2	2	0	0	1	0
EABT4956	3	6	27	0	1	0	0
EABT4957	0	0	0	6	0	0	0
EABT4958	18217.1	13147	28	7	2084	25684.4	14567.3
EABT4959	2808.08	4383.82	3831.34	2333	203.99	1939.22	216
EABT496	0	5	4	9	3	0	1
EABT4960	0	2	6	0	0	0	0
EABT4961	4243.8	6312.85	6120.2	9864.11	3344.03	4802.68	3735.2
EABT4962	2	7	8	15	28	0	1
EABT4963	2	4	10	1	0	1	0
EABT4964	0	1	4	0	0	3	2
EABT4965	0	2	4	0	0	1	0
EABT4966	1	1	7	0	0	2	0
EABT4967	0	2	2	2	0	0	0
EABT4968	0	0	3	1	0	0	0
EABT4969	562.64	909.98	930.33	1091.68	694.71	585	688.96
EABT497	2	16	7	3	0	0	0
EABT4970	0	0	1	33	2	0	3
EABT4971	0	0	0	0	1.66	0	5
EABT4972	2	1	25	1	0	1	0
EABT4973	0	0	8	0	0	0	0
EABT4974	1	0	6	2	1	0	0
EABT4975	0	3	123	0	2	3	2.01
EABT4976	1	1	6	1	1	0	0
EABT4977	1	0	0	168	40	8	7
EABT4978	16	12.98	14	61.62	1	2	5
EABT4979	0	0	4	0	0	0	1
EABT498	8	24	13	18	4	3	4
EABT4980	0	0	1	18	2	1	0
EABT4981	1	1	1	4	1	0	0
EABT4982	0	1	2	4	0	0	0

EABT4983	8	18	25	16	0	0	0
EABT4984	1	0	8	2	0	0	0
EABT4985	1	4	2	9	1	0	0
EABT4986	1945.11	10299.8	9630.24	7213.5	10825.4	266	2239.21
EABT4987	2	1	0	0	0	0	5
EABT4988	5	12	21.99	3	3	0	0
EABT4989	33.03	69	73	31	145	10	345
EABT499	27	80	297.99	560	117.94	8	28
EABT4990	7	53	15	80	18	2	9
EABT4991	0	1	7	8	0	0	0
EABT4992	7227.39	9990.97	11256.5	9178.69	6028.57	8142.73	5203.39
EABT4993	786.04	1733.31	3406.72	4677.87	1267.69	510.95	642
EABT4994	0	7	29	19	5	2	1
EABT4995	0	1	43.03	8	0	2	0
EABT4996	0	2	14	4	0	0	0
EABT4997	0	0	0	0	0	0	2
EABT4998	0	1	3	6	9	0	0
EABT4999	0	0	7	0	0	0	0
EABT5	2	1	2	0	4	1	4
EABT50	3	2	0	0	0	1	1
EABT500	0	0	1	0	4	0	4
EABT5000	1	2	4	0	1	0	3
EABT5001	1	2	29	1	0	0	0
EABT5002	1373.36	996.02	468.83	57136.4	4	2	1
EABT5003	2153.03	3170.72	4123.75	6395.22	2314.56	1509.82	1470.97
EABT5004	1	2	14	6	1	1	0
EABT5005	0	5	12	2	0	0	0
EABT5006	0	2	1	0	0	0	0
EABT5007	0	1	5	1	0	1	0
EABT5008	3	1	47	67	0	5	1
EABT5009	1	3	1	1	2	1	1
EABT501	0	0	4	0	0	0	0
EABT5010	2	6	18	4	2	0	0
EABT5011	0	0	2	1	0	0	0
EABT5012	1	1	10	2	2	0	0
EABT5013	1	0	17	8	0	0	0
EABT5014	0	2	1	1	1	0	3
EABT5015	0	1	2	0	0	0	0
EABT5016	4	5	23	5	3	7	17
EABT5017	0	0	7	1	0	0	0
EABT5018	0	1	4	0	0	1	0
EABT5019	11	0	0	0	1	1	0
EABT502	0	1	2	1	0	1	0
EABT5020	102.18	309.87	376.58	7	31	69	9
EABT5021	0	7	4	3	1	1	6
EABT5022	2	1	6	1	2	0	3
EABT5023	0	0	18	0	0	1	0
EABT5024	17	8	6	2	3	12	0
EABT5025	2	4	4	2	0	0	0
EABT5026	1	5	2	0	1	2	2
EABT5027	0	0	14	9	1	0	0

EABT5028	4	3	1	0	1	0	0
EABT5029	3	3	3	1	6	0	0
EABT503	1	2	1	1	0	0	1
EABT5030	0	2	48	5	1	0	0
EABT5031	1	0	20	0	0	0	0
EABT5032	3	11	8	15	3	4	2
EABT5033	0	4	7	4	0	1	1
EABT5034	5	46	19	17	3	7	5
EABT5035	0	0	45	0	0	0	0
EABT5036	0	1	7	0	0	0	1
EABT5037	173.98	476.79	148.8	2327.16	26.03	98.85	84.8
EABT5038	227	526	615.21	169	292	2	19
EABT5039	1146	1543.55	928	1893.99	1064	1059	1122.01
EABT504	0	1	1	1	0	0	3
EABT5040	0	1	2	0	0	3	0
EABT5041	1	7	0	0	0	0	0
EABT5042	0	5	5	2	0	0	0
EABT5043	9	3	29.29	1	1	5	2
EABT5044	4	3	2	1	1	0	1
EABT5045	1	1	3	1	0	0	1
EABT5046	0	2	0	18	0	0	0
EABT5047	1	8	19	4	2	2	3
EABT5048	0	0	7	2	0	0	0
EABT5049	7	10	0	0	1	52	30
EABT505	1	4	4	10	2	0	4
EABT5050	44	0	1	0	0	4	14
EABT5051	1	2	4	2	0	0	0
EABT5052	1	1	0	0	1	1	0
EABT5053	1164.02	1583.86	2620.98	2120.45	1227	1569.81	1410.01
EABT5054	625.12	1071	1053	2225.73	724.24	636.05	608.51
EABT5055	3	2	1	0	0	1	2
EABT5056	4	3	2	0	0	3	0
EABT5057	2	0	1	16	3	1	6
EABT5058	3	2	15	7	4	2	0
EABT5059	0	11	2	14	3	0	4
EABT506	0	2	5	0	3	0	2
EABT5060	0	0	6	2	7	0	0
EABT5061	0	1	8	1	0	0	0
EABT5062	1	1	3	1	1	1	1
EABT5063	0	0	8.42	1	0	0	0
EABT5064	0	0	5	0	0	0	1
EABT5065	0	0	12	0	0	0	0
EABT5066	0	0	6	0	0	0	1
EABT5067	0	1	5	2	0	0	0
EABT5068	613	899	1274	1663	956	604	722
EABT5069	0	5	532	2	2	5	3
EABT507	3	10	16	5	3	0	3
EABT5070	20	54.27	83	149	54	8	18
EABT5071	3052.89	4802.12	3220.39	3747.37	1546.99	2693.09	3001.87
EABT5072	180	246.6	2191.33	386.95	127	417.01	137.99
EABT5073	1	1	14	2	1	6	2

EABT5074	1	0	7	0	2	1	2
EABT5075	0	4	3	4	1	5	1
EABT5076	0	1	2	6	1	0	1
EABT5077	9	42	37	24	5	3	6
EABT5078	1	1	11	0	1	0	1
EABT5079	315.14	513.29	200.87	947.35	460	277.35	199.01
EABT508	503.94	687.17	593.57	804.72	536.87	414.98	428.29
EABT5080	39	63	3	0	28	13	50
EABT5081	3	7	49	8	1	0	0
EABT5082	2	1	14	5	2	6	1
EABT5083	0	0	27	2	0	0	0
EABT5084	0	0	0	0	11	0	12
EABT5085	0	0	19	6	2	0	0
EABT5086	1	0	26	7	0	0	0
EABT5087	0	0	15	0	0	0	1
EABT5088	0	1	24	2	1	0	5
EABT5089	9	19	8	2	3	6	7
EABT509	6	40	457	1	5	0	1
EABT5090	0	1	10	0	0	1	0
EABT5091	1024	1867.34	562	531.98	2160.27	2291.13	2283.94
EABT5092	0	0	1	0	4	0	0
EABT5093	0	3	6	1	0	0	0
EABT5094	4	2	28	3	0	1	1
EABT5095	1	1	3	0	0	0	0
EABT5096	88.03	97	115	44	4	45	12
EABT5097	1	1	12	14	0	1	0
EABT5098	2	2	1.92	0	3	2	9
EABT5099	25472.2	35718.4	14840.9	9136.69	7392.45	7740.32	15498.7
EABT51	468.99	689.91	483	766	532	401	433
EABT510	464.62	683.52	1392.48	1253.18	678.96	435	397
EABT5100	0	1	4	1	1	1	1
EABT5101	2	6	0	1	0	21	5
EABT5102	3	12.45	21	13	3	7	2
EABT5103	5	6	0	0	0	7	4
EABT5104	1	6	21	4	1	0	3
EABT5105	0	1	1	5	0	1	0
EABT5106	938	1860.35	1237.2	3116.91	1342.39	498	1802.98
EABT5107	36	50	106	172	46	27	27
EABT5108	0	1	9	1	0	0	0
EABT5109	2	0	0	2	1	1	1
EABT511	0	1	5	4	1	0	0
EABT5110	1	1	15	0	1	0	0
EABT5111	1	6	6	2	0	0	2
EABT5112	0	4	4	3	0	2	0
EABT5113	0	0	3	5	0	0	0
EABT5114	0	3	1	8	0	2	0
EABT5115	1	2	7	2	0	1	0
EABT5116	0	0	4	1	0	1	0
EABT5117	282	381	623.02	1058.05	310	290	240.93
EABT5118	1	1	9	2	1	1	1
EABT5119	1	1	7	3	0	0	0

EABT512	39	37	121	150	59	3	42
EABT5120	0	1	2	0	0	1	1
EABT5121	1	0	16	15	1	0	0
EABT5122	12	3	4	2	3	1	1
EABT5123	1	5	15	9	2	3	1
EABT5124	1	0	0	7	0	0	0
EABT5125	0	1	20	4	0	0	0
EABT5126	1	2	11	13	1	0	0
EABT5127	0	0	3	3	1	0	0
EABT5128	1	0	0	0	2	0	4
EABT5129	1	2	4	0	1	2	1
EABT513	1	8	18	1	6	0	0
EABT5130	7	8	27	1	1	2	2
EABT5131	0	1	4	14	1	0	1
EABT5132	57	53	38	0	0	0	0
EABT5133	0	1	3	0	3	1	2
EABT5134	0	2	2	0	1	0	1
EABT5135	450.17	517.71	511	960.17	510.45	332.86	251.05
EABT5136	0	4	10	1	2	1	6
EABT5137	0	0	5	0	0	0	0
EABT5138	0	0	0	8	1	0	0
EABT5139	70.1	111.39	185.11	163.78	102	95.65	148.84
EABT514	2195.06	3149.68	2388	3309.74	2149.74	1758.66	2335.76
EABT5140	0	0	0	2	17	0	1
EABT5141	0	0	3	0	1	0	0
EABT5142	0	1	11	0	0	0	3
EABT5143	0	0	4	2	0	0	0
EABT5144	2	2	35	2	1	6	1
EABT5145	0	2	14	0	1	0	0
EABT5146	4125.71	3994.15	2641.61	5645.09	2646	5165.27	3365.49
EABT5147	0	2	0	3	0	0	0
EABT5148	0	1	5	0	0	1	1
EABT5149	2	17	49	20	5	8	15
EABT515	1	1	6	0	0	1	0
EABT5150	0	4	56	3	0	2	0
EABT5151	43	55	90	105.02	54	24	30
EABT5152	0	1	2	1	0	1	1
EABT5153	4	6	7	5	0	2	1
EABT5154	942.03	1669.9	1719.82	2676.89	1186	858	1024
EABT5155	16	13	0	0	2	6	14
EABT5156	1242.99	3473.32	1258.01	918.99	5867.68	57	94
EABT5157	14088.4	9237.52	10093.2	4632.39	25741.6	4244.92	3815.95
EABT5158	609	1286.88	3406.96	1185.83	8414.1	23	134
EABT5159	1	3	12	5	1	0	0
EABT516	3	11	19	9	7	1	5
EABT5160	0	0	5	2	0	1	0
EABT5161	0	1	3	0	3	0	1
EABT5162	8	4	31	8	4	0	2
EABT5163	3	9	39	6	3	0	10
EABT5164	1765.77	2281.79	2021.14	3175.78	1968.33	1578.51	1380.2
EABT5165	0	0	0	0	0	0	0

EABT5166	25	28	0	0	5	73	47
EABT5167	0	7	2	4	7	0	1
EABT5168	136	138.99	46.97	219	157	34	19
EABT5169	2	1	5	0	0	1	0
EABT517	0	1	1	2	0	0	0
EABT5170	1630.96	1480.67	338.42	753.27	4274.56	1272.85	1243.19
EABT5171	0	2	1	2	1	0	0
EABT5172	0	3	12	1	0	0	0
EABT5173	5	11	150	32	3	5	10
EABT5174	0	2	3	1	1	9	3
EABT5175	0	1	6	6	3	0	0
EABT5176	2	4	7	6	0	3	1
EABT5177	1	0	0	1	0	0	2
EABT5178	7	11	1	0	4	21	8
EABT5179	1016.91	2138.05	1784	4873.85	711.99	75	441.02
EABT518	5	4	5	1	1	4	2
EABT5180	0	0	3	3	0	0	0
EABT5181	1	6	30	1	0	0	1
EABT5182	12	9	0	0	3	4	80
EABT5183	22	10	1	12	4	0	0
EABT5184	0	18	0	0	0	4	0
EABT5185	1	0	7	0	0	0	0
EABT5186	15	24	16	188.94	22.99	36.04	38
EABT5187	0	2	2	3	1	0	0
EABT5188	342.13	1435.09	1908	1445	1180	279	676.81
EABT5189	4	0	5	0	1	0	10
EABT519	2369.99	3258.73	2638.77	4827.37	3024.11	2412.99	2554.91
EABT5190	1	0	0	9	2	8	5
EABT5191	0	1	16	13	25	2	1
EABT5192	2	1	4	1	0	0	1
EABT5193	10	15	6	1	0	27	6
EABT5194	0	0	0	1	9	0	0
EABT5195	1	2	4	43	2	0	1
EABT5196	1	4	42	25	1	0	0
EABT5197	3183	5053.57	4138.38	7445	9693.28	3045.8	3202.17
EABT5198	8	18	6	2	0	53	0
EABT5199	2175.4	2825.11	2210.67	2541.66	2364.34	2794.29	2562.14
EABT52	0	1	2	0	0	0	2
EABT520	9	10	83	7	1	2	0
EABT5200	0	0	10	0	1	0	0
EABT5201	3	2	3	1	2	1	0
EABT5202	0	0	16	0	0	0	1
EABT5203	1961.03	3392.99	3499.07	3589.01	2162.04	1610.92	2062.74
EABT5204	7100.93	12041.9	11507.7	13342.2	6426.15	7542.14	8519.37
EABT5205	2	0	0	1.86	2	4	15
EABT5206	2	2	1	0	0	2	2
EABT5207	1	2	3	5	1	1	0
EABT5208	663.05	1152.42	1094.94	2477.29	900.16	690.73	633.16
EABT5209	0	1	6	1	0	0	0
EABT521	0	0	7	0	0	0	0
EABT5210	21	18	27	41	9	6	5

EABT5211	0	2	13	0	0	0	0
EABT5212	1	0	0	0	0	0	18
EABT5213	0	1	1	1	1	1	1
EABT5214	3	1	2.98	1	0	4	2
EABT5215	55	103	179.01	1531	81	3	6
EABT5216	5699.95	9265.3	17825.5	18261.6	5785.76	7862.25	7048.39
EABT5217	9	2	1	1	3	0	3
EABT5218	321.02	338.69	407.93	926.36	336.23	229	274
EABT5219	2	2	5	6	1	1	1
EABT522	3	8	7	2	0	2	4
EABT5220	294	227	686	155	650	137	233
EABT5221	62	103	530	794	182	85	108
EABT5222	0	0	5	4	0	0	0
EABT5223	1	2	0	0	0	1	0
EABT5224	21	24	15	15	8	14	1
EABT5225	5	7	33	22	4	3	9
EABT5226	0	1	2	15	11	0	1
EABT5227	390.83	331	316.82	1100.01	114	352	41
EABT5228	5	6	9	17	3	11	29.6
EABT5229	1	0	5	0	0	0	0
EABT523	0	0	3	2	1	1	0
EABT5230	1	1	4	4	0	0	1
EABT5231	2	5	0	0	1	12	8
EABT5232	478	578	288	439	423.64	278	274
EABT5233	8	79	8	30	34.91	5	268.35
EABT5234	0	3	10	7	0	2	6
EABT5235	5	3	13	3	1	0	0
EABT5236	26	35	56.99	8	8	52	20
EABT5237	0	0	1	6	0	0	0
EABT5238	5457.92	3832.87	104	163	8743.82	3607.92	49750.5
EABT5239	0	2	3	3	0	0	0
EABT524	0	1	4	15	0	0	0
EABT5240	0	1	17	0	0	0	0
EABT5241	7	5	3	7	3	0	0
EABT5242	2	5	43	2	2	2	4
EABT5243	9	23	7	26	332	3	80
EABT5244	0	1	80	0	0	0	0
EABT5245	0	1	8	0	1	2	0
EABT5246	1	13	7	0	0	1	0
EABT5247	2	3	3	0	0	0	2
EABT5248	489.99	832.99	601	1560	454	54	133.86
EABT5249	0	3	3	0	0	0	0
EABT525	1272.89	1647.05	1441.82	3165.89	1572.99	1472.68	1039.53
EABT5250	0	0	0	0	1462.95	0	0
EABT5251	3	0	10	0	0	0	1
EABT5252	0	1	1	0	0	0	3
EABT5253	27	76	10	0	3	191	9
EABT5254	1305.75	1531.43	1366	974.05	1579.94	11	43
EABT5255	2	11	39	2	2	4	5
EABT5256	7	3	12	8	2	1	0
EABT5257	0	0	0	22	0	0	0

EABT5258	5	0	0	106	10	0	2
EABT5259	0	0	1	4	2	0	0
EABT526	2	1	10	5	1	1	2
EABT5260	44	246	0	1	5	1	8
EABT5261	11	10	4	2	2	6	4
EABT5262	0	0	3	2	0	0	0
EABT5263	590.51	900.01	866.43	2797.21	748.91	422.97	596.22
EABT5264	2	2	7	6	3	3	3
EABT5265	0	0	14	0	0	0	0
EABT5266	0	1	4	1	5	0	0
EABT5267	0	2	13	2	0	1	0
EABT5268	3	54	42	3	2	15	2
EABT5269	301	549	597.27	1889.64	318.02	200	239.79
EABT527	1187.55	1871.23	2374.78	4081.87	1074.98	1260.1	872.51
EABT5270	7779.64	10633	9041.8	14220.7	7565.7	7802.39	6175.8
EABT5271	0	0	9	2	1	0	0
EABT5272	0	1	1	1	0	0	0
EABT5273	2	1	7	1	0	0	0
EABT5274	0	3	7	0	0	2	0
EABT5275	0	0	11	1	0	0	0
EABT5276	22	65	318	395	11	16	16
EABT5277	0	0	6	0	0	0	0
EABT5278	535	38	7	0	11	164.64	39
EABT5279	0	1.01	1	3	1	0	1
EABT528	2	1	10	5	0	1	0
EABT5280	2	7	0	0	0	3	9
EABT5281	1	0	4	1	0	0	0
EABT5282	0	3	0	4	0	0	4
EABT5283	0	2	12	0	0	0	0
EABT5284	4	8	7	4	0	0	0
EABT5285	0	1	1	0	1	0	1
EABT5286	42	82	133	135	467.94	13	48
EABT5287	1	2	5	0	1	10	4
EABT5288	2720.71	4862.4	9825.85	7696.57	3111.9	2513.55	2222.95
EABT5289	13	20	81	36	7	12	5
EABT529	0	0	4	13	0	0	1
EABT5290	0	0	8	6	0	0	0
EABT5291	2	3	1	3	1	0	0
EABT5292	1	2	1	0	0	2	0
EABT5293	850.03	1448.7	1604.75	2945.87	652	403	444
EABT5294	0	2	5	1	0	1	0
EABT5295	0	0	3	1	0	0	0
EABT5296	7	2	2	0	0	1	0
EABT5297	0	1	5	0	1	0	0
EABT5298	572	1007	370	1062.96	633.98	432	467
EABT5299	611	791.57	653.75	1196.1	705.04	599.01	539
EABT53	30	39	0	0	0	1	0
EABT530	0	3	8	2	1	0	0
EABT5300	3270.3	6593.5	20620	8010.81	3337	2905.82	2963.95
EABT5301	1	5	1	0	0	0	0
EABT5302	12	23	16	4	41.83	34	376.97

EABT5303	1197.11	1024.31	311	115.03	322.09	1640.11	1951.55
EABT5304	0	0	7	0	0	0	0
EABT5305	5	9	1	4	1	4	1
EABT5306	1	1	0	0	1	0	5
EABT5307	376.11	593	718.44	2455.21	834.81	282.07	422.1
EABT5308	3	2	7	2	0	0	2
EABT5309	0	0	6	0	0	2	0
EABT531	6	15	0	0	1	47	13
EABT5310	0	1	1	2	1	0	0
EABT5311	3	1	8	0	0	1	1
EABT5312	2	2	9	9	22	1	14
EABT5313	639.04	707.03	72	144.81	256.88	469.01	705.99
EABT5314	0	2	3	1	2	0	0
EABT5315	0	0	1	1	4	1	14
EABT5316	5	8	38	9	0	9	2
EABT5317	1	1	8	5	0	0	0
EABT5318	0	5	5	0	1	0	1
EABT5319	734.93	5205.63	3243.37	21	538.61	423.99	2839.4
EABT532	0	2	1	3	2	0	0
EABT5320	1	2	16	3	1	0	0
EABT5321	1	2	14	0	0	1	0
EABT5322	3	4	3	11	6	0	2
EABT5323	1	1	83	1	0	1	0
EABT5324	4	6	24	1	0	0	0
EABT5325	0	6	3	1	1	1	0
EABT5326	5	12	8	3	0	1	4
EABT5327	0	0	6	2	0	0	0
EABT5328	362	627	1300.72	2130.34	569	366	305.02
EABT5329	2	0	4	2	1	0	0
EABT533	0	2	8	0	1	0	2
EABT5330	711	983	686	1227.08	993.25	747.98	756.76
EABT5331	141	40	5	2	3	33	6
EABT5332	0	0	3	0	3	0	2
EABT5333	0	0	6	1	0	0	0
EABT5334	6	5	0	0	0	0	2
EABT5335	4357.28	9574.92	20669.9	15746	5662.81	7483.66	6443.41
EABT5336	0	0	5	0	0	0	0
EABT5337	1	0	35	2	1	0	0
EABT5338	1	2	6	0	1	2	1
EABT5339	0	1	3	0	2	0	1
EABT534	1	0	28	1	0	0	0
EABT5340	0	2	1	9	0	0	1
EABT5341	1	0	2	1	0	0	0
EABT5342	1	1	1	6	0	0	0
EABT5343	51	52	41	337	22	251	124
EABT5344	9	29	33	73.99	44	0	5.01
EABT5345	0	20	2	1	5	2	6
EABT5346	130	261	404	816.37	288	87	116.1
EABT5347	4	8	2	21	5	5	26
EABT5348	2	14	0	3	0	14	0
EABT5349	1469	4893.29	5231.33	15332.4	3288.46	343	1695

EABT535	0	3	2	10	0	0	1
EABT5350	0	5	1	0	0	0	0
EABT5351	3	19	36	32	32	3	7
EABT5352	2	0	3	0	1	0	0
EABT5353	124.95	604	1809	103	198	8	142
EABT5354	53	34.99	24	25	5	29	33
EABT5355	497.64	658.4	839.04	1343.96	670.93	411	292.81
EABT5356	4046.85	4105.35	2560.17	3301.5	2887.92	2589.47	3095.24
EABT5357	2112.81	196	2	19	58	2266.97	209
EABT5358	0	3	2	6	2	0	1
EABT5359	0	0	1	0	0	0	0
EABT536	51	79	31	97	58.01	5	69
EABT5360	2	3	0	4	0	0	0
EABT5361	1103.99	1320.88	1698.02	3716.2	1744.07	1604.1	801.51
EABT5362	1	1	0	0	0	1	5
EABT5363	879.34	296.73	15	0	2526.01	1439.41	36442
EABT5364	1	1	11	8	0	1	8
EABT5365	5	9	41	12	1	19	4
EABT5366	8	11	43	33	4	13	7
EABT5367	12152.8	12636.9	12535	10774.8	10568.7	15875.8	12276.3
EABT5368	138.1	165	296	322.46	144.06	125.17	124.66
EABT5369	5	1	1	1	0	0	0
EABT537	5	12	7	10.16	2	1	5
EABT5370	3723.25	5388.48	11238.6	9611.2	2749.27	2381.16	3689.19
EABT5371	1	0	2	2	0	0	0
EABT5372	0	1	16	1	0	0	1
EABT5373	1	0	8	1	1	0	0
EABT5374	1	1	15	0	1	0	2
EABT5375	5	2	6	6	1	0	0
EABT5376	0	3	15	1	1	2	0
EABT5377	3	1	5	0	58	1	1
EABT5378	3896.48	3826.72	1013.27	2170.48	2000.59	1224.42	1299.91
EABT5379	23	42	52	74	5	31	11
EABT538	0	0	0	1	0	0	8
EABT5380	6	4	5	1	0	5	2
EABT5381	0	0	5	0	0	0	1
EABT5382	6	6	0	0	0	8	5
EABT5383	1	7	47	22	1	0	0
EABT5384	32	18	19	3	1	11	7
EABT5385	0	2	11	0	2	4	2
EABT5386	0	1	6	1	0	0	0
EABT5387	578	761.76	343	1134.22	650.33	302.79	595
EABT5388	0	0	13	0	0	0	0
EABT5389	77	252	137	249	283	4	26
EABT539	1059	1209	1024	1193.19	555	888	923
EABT5390	0	0	3	2	1	0	0
EABT5391	1	6	2	3	0	7	1
EABT5392	1	1	0	0	0	1	0
EABT5393	1161.62	2064.83	1856.64	2009.68	1160.48	692.6	1260.67
EABT5394	0	2	1	3	1	0	1
EABT5395	6	33	27	10	4	1	0

EABT5396	0	1	6	1	0	0	0
EABT5397	1	0	4	4	1	1	0
EABT5398	14	32	50	23	7	3	6
EABT5399	0	1	3	3	1	0	0
EABT54	0	0	0	5	0	0	0
EABT540	4	4	1	23	3	0	1
EABT5400	5	30	31	24	10	0	0
EABT5401	3	2	0	0	7	1	7
EABT5402	0	0	9	1	0	0	0
EABT5403	0	1	6	3	1	2	2
EABT5404	3	3	6	9	2	0	0
EABT5405	63	79.23	36	114.75	126	52	84
EABT5406	5	10	10	2	1	2	11
EABT5407	200	586	1867.02	10342.7	685	36	131
EABT5408	0	29.01	188	49	13	1	2
EABT5409	2	2	7	1	1	0	1
EABT541	0	1	3	1	1	0	3
EABT5410	5897.54	8807.05	8598.49	11896.2	4524.31	9297.09	9063.39
EABT5411	1	2	2	0	4	0	4
EABT5412	0	1	8	1	1	1	0
EABT5413	0	5	0	0	2	1	1
EABT5414	3	7	7	1	0	2	3
EABT5415	958.97	1086.89	386.07	1670	519.06	390.99	372
EABT5416	1	0	23	6	2	5	0
EABT5417	56	84	140	155	121.01	6	67
EABT5418	3	8	33.88	10	2	4	5
EABT5419	2	10	10	3	5	8	7
EABT542	1	1	5	0	0	0	0
EABT5420	0	0	34	2	0	0	0
EABT5421	461.12	676.05	770.3	1378.58	569.08	202.56	418.14
EABT5422	0	0	4	3	0	0	0
EABT5423	0	5	0	0	1	0	1
EABT5424	4	12	14	31	3	3	41
EABT5425	4	14	7	23	10	0	0
EABT5426	384	517.02	341	959	605.29	152	483.98
EABT5427	2	0	0	0	0	33	1
EABT5428	2	3	10	2	0	0	0
EABT5429	64	146.28	391	581.07	55	204	33
EABT543	12	18	0	0	1	4	4
EABT5430	2	1	4	0	0	0	0
EABT5431	5	5	0	6	5	1	7
EABT5432	0	2	1	4	3	0	0
EABT5433	1	0	26	7	0	3	0
EABT5434	0	3	0	4	0	1	0
EABT5435	4	0	0	1	9	2	21
EABT5436	6	29.99	105	48	3	11	9
EABT5437	18	89	19.21	5	1	0	0
EABT5438	0	0	7	3	1	0	0
EABT5439	21	70	83.1	125.23	77.99	0	7
EABT544	0	0	0	0	2	0	0
EABT5440	1	0	5	0	0	0	0

EABT5441	0	0	2	0	0	0	0
EABT5442	1	4	8	0	1	0	1
EABT5443	1	0	5	0	0	0	0
EABT5444	99.98	152.49	247.63	345.66	146.02	150.87	142.28
EABT5445	3	1	17	1	20	1	1066
EABT5446	1	0	1	2	0	0	1
EABT5447	156	250	35	130.07	86.62	13	17
EABT5448	2508.4	4434.35	7756.93	5465	5183.64	4237.7	2518.45
EABT5449	214.69	233.82	333.6	107	200.44	197	367.02
EABT545	265	297	126	181	99	590	82
EABT5450	4	7	4	3	2	13	5
EABT5451	53	195	209.35	50	131	7	30
EABT5452	0	5	0	0	0	0	4
EABT5453	1	2	4	14	19	4	4
EABT5454	8	13	7	13	5	4	6
EABT5455	1	2	13	1	0	0	0
EABT5456	2	2	0	0	0	1	2
EABT5457	874.53	1295.89	1366	2038.39	1011.93	945.46	971.04
EABT5458	0	2	4	2	2	0	1
EABT5459	5	3	0	0	0	1	0
EABT546	2.01	3	10	11	0	0	0
EABT5460	1837.16	2945.37	2723.26	6263.71	2012.31	1527.58	1361.55
EABT5461	0	0	11	0	0	0	0
EABT5462	562.04	474	24	545	105	199	191
EABT5463	1	18	4	2	0	6	0
EABT5464	5	5	0	0	0	0	0
EABT5465	0	1	6	0	0	1	2
EABT5466	0	1	4	3	1	0	0
EABT5467	1592.51	1934.69	1628.13	2614.35	1637.41	1238.11	1344.5
EABT5468	2857.13	595	185.76	779.99	274	6578.71	1075
EABT5469	21	77	121.01	234	388	35	41
EABT547	1	0	2	0	0	1	0
EABT5470	1	1	3	1	0	0	0
EABT5471	0	0	0	0	8	0	80
EABT5472	1693.43	2358.17	2447.1	5945.27	2511.61	1313.12	3098.6
EABT5473	1	3	1	0	4	0	3
EABT5474	4	1	2	5	0	2	3
EABT5475	1	0	7.02	2	3	0	4
EABT5476	0	0	2	0	1	0	0
EABT5477	1147	1662	1699	2817.91	1857.5	931	1055.83
EABT5478	0	0	5	0	0	0	0
EABT5479	0	1	4	0	0	0	0
EABT548	20	45	82	114	69	22	24
EABT5480	0	0	7	0	0	0	0
EABT5481	0	0	6	1	0	0	1
EABT5482	0	0	2	0	0	0	1
EABT5483	1	4	5	1	5	2	13
EABT5484	0	1	3	1	0	1	0
EABT5485	0	2	10	2	2	0	0
EABT5486	0	0	14	0	0	0	1
EABT5487	0	1	5	0	0	2	0

EABT5488	0	1	15	1	1	0	0
EABT5489	0	5	0	0	2	0	0
EABT549	0	0	2	0	0	0	0
EABT5490	1	2	5	3	0	0	0
EABT5491	1	7	0	7	2	0	0
EABT5492	0	3	10	3	0	0	1
EABT5493	17	17.85	138	2	4	9	2
EABT5494	0	0	0	0	4	0	0
EABT5495	13	1	0	0	0	11	0
EABT5496	3	6	72	7	5	10	3
EABT5497	8	8	23	8	1	2	0
EABT5498	0	1	9	1	0	0	0
EABT5499	0	0	5	1	0	0	0
EABT55	0	1	7	0	0	1	0
EABT550	8	14	82	58	114	4	11
EABT5500	4	5	6	0	1	0	0
EABT5501	10	13	2	2	3	4	2
EABT5502	3	9	3	0	0	0	1
EABT5503	1	1	28	1	0	0	0
EABT5504	1588.58	2049.56	1821.92	3031.14	1039.08	1502.1	1390.49
EABT5505	0	2	6	2	2	0	0
EABT5506	0	4	10	1	0	0	0
EABT5507	12	41	47	16	40	10	23
EABT5508	0	3	3	7	1	0	0
EABT5509	1	2	5	1	0	2	0
EABT551	0	0	1	1	1	0	0
EABT5510	527	601	296	1020	473	539	562
EABT5511	103	132	5	6.97	2	7	2
EABT5512	7	7	23	20	4	14	7
EABT5513	0	3	1	4	0	4	2
EABT5514	0	3	2	3	0	0	1
EABT5515	6	18	23	33	17	2	1
EABT5516	0	0	2	0	0	0	0
EABT5517	0	0	2	0	0	0	0
EABT5518	0	0	13	3	4	0	6
EABT5519	2	6	22	0	0	0	3
EABT552	1519.34	2127	3753.92	3344.54	1044	891.68	965
EABT5520	0	0	5	0	0	0	0
EABT5521	10	1	13	267	95	1	1
EABT5522	0	0	0	7	14	0	0
EABT5523	0	0	0	0	0	0	0
EABT5524	0	3	0	8	2	1	3
EABT5525	2	1	0	0	1	1	0
EABT5526	8	17	54	2159	20	2	3
EABT5527	0	1	1	3	1	0	1
EABT5528	0	2	0	0	1	1	0
EABT5529	2	3	5	0	1	0	0
EABT553	7	17	0	0	0	0	1
EABT5530	0	0	0	0	0	0	0
EABT5531	0	0	0	0	0	15	22
EABT5532	1	29	27	56.02	8	1	22

EABT5533	0	0	1	0	5	0	0
EABT5534	1022.97	1502	1049.97	1309.01	983.9	412	805.69
EABT5535	0	0	2	1	5	0	1
EABT5536	0	1	2	0	1	0	3
EABT5537	0	1	4	6	0	0	0
EABT5538	462	528	70	164	327	430.99	998.04
EABT5539	1	1	10	1	1	3	1
EABT554	1	1	6	4	2	3	1
EABT5540	0	4	2	15	1	0	14
EABT5541	0	2	12	0	0	0	0
EABT5542	1	2	8	0	2	0	0
EABT5543	1	2	23	14	1	0	0
EABT5544	0	5	9	7	0	0	1
EABT5545	369	493	391	477.01	340	181.88	214
EABT5546	0	0	21	0	0	0	0
EABT5547	7	35	42	24	11	1	4
EABT5548	0	2	70	0	0	0	0
EABT5549	0	0	3	2	0	0	0
EABT555	3	2	0	0	0	0	1
EABT5550	1	0	0	0	0	0	1
EABT5551	461	508	434.42	981.05	748.01	482	369.93
EABT5552	1	2	2	0	0	0	0
EABT5553	55	188	239.17	913	523.98	102	84
EABT5554	3	4	1	1	1	1	0
EABT5555	4	1	0	0	0	10	6
EABT5556	0	1	2	0	0	4	0
EABT5557	0	1	11	1	0	0	0
EABT5558	369	437.14	248	443.02	397.88	451.28	349.06
EABT5559	0	0	15	0	1	0	0
EABT556	0	0	8	2	0	1	0
EABT5560	1055.54	1812.09	1552.46	3265.82	1408.76	1004.98	1185.12
EABT5561	1	0	0	0	0	3	2
EABT5562	10539.7	16022.7	9564.56	7131.29	5430.58	13189.1	8435.5
EABT5563	7	6	74.96	6	1	10	3
EABT5564	4	38	49	18	4	0	1
EABT5565	0	1	1	0	2	0	9
EABT5566	3412.99	4672.88	2284.6	1986.84	7217.04	4873.82	5737.82
EABT5567	0	2	1	0	1	1	0
EABT5568	1	1	1	0	0	1	0
EABT5569	12	6	24	25	863	26	4
EABT557	2	9	5	0	0	6	1
EABT5570	2310.29	1242.97	994	820	414.94	3515.45	1347.48
EABT5571	2	1	1	0	0	14	6
EABT5572	0	3	1	0	1	0	0
EABT5573	1	0	11	3	0	0	0
EABT5574	639.07	1448.56	1501.58	8112.82	398.35	46	223
EABT5575	2780	3678.56	3290.77	4796.03	1748.02	3847.01	2481.35
EABT5576	0	1	9	1	0	1	0
EABT5577	1	8	6	5	2	34.98	10
EABT5578	0	0	6	5	1	0	0
EABT5579	1	1	7	0	1	1	0

EABT558	1	6	2	0	0	0	1
EABT5580	3	1	23	2	1	1	4
EABT5581	23	25	0	1	12	84.01	33
EABT5582	0	0	19	1	2	1	1
EABT5583	4	12	16.02	14	1	0	1
EABT5584	0	1	2	7	1	1	0
EABT5585	0	1	81	0	1	2	1
EABT5586	2	1	34	4	2	0	1
EABT5587	106.99	185.01	227.09	276	75	114.67	108.01
EABT5588	0	1	15	2	0	1	1
EABT5589	0	1	15	0	1	2	0
EABT559	0	0	0	0	2	0	1
EABT5590	2	4	3	3	1	1	0
EABT5591	1	1	63	1	0	0	0
EABT5592	1	4	1	1	1	0	0
EABT5593	0	0	3	0	0	0	0
EABT5594	0	0	12	0	0	0	0
EABT5595	2	5	20	4	0	1	2
EABT5596	278.97	335	168	41	89	145	179
EABT5597	1809.21	2374.61	1818.71	3602.71	1899.01	1747.03	1745.24
EABT5598	0	0	0	0	0	0	0
EABT5599	7	18	47	57	0	1	0
EABT56	1	6	12	8	3	1	0
EABT560	24784	25501.8	19123.3	25575.6	21643.2	31543.7	26603.4
EABT5600	108	69	142	2	9	55	21
EABT5601	0	5	3	1	0	0	0
EABT5602	1	0	1	0	0	0	0
EABT5603	1	5	29	8	4	1	1
EABT5604	1	7	26	35	33	1	3
EABT5605	0	2	5	1	1	0	59
EABT5606	0	1	1	5	0	0	0
EABT5607	0	0	8	0	0	2	0
EABT5608	3	6	0	0	0	0	0
EABT5609	155.31	245.02	89	213.28	185	68.94	90.01
EABT561	3	6	8	8	9	2	2
EABT5610	0	1	0	6	1	1	1
EABT5611	0	0	10	3	0	1	0
EABT5612	1	4	28	3	0	1	0
EABT5613	24	550.04	192.98	25	16	8	12
EABT5614	15742.6	23097.8	169	21	6690.53	3580.92	58858.2
EABT5615	35	94	483	56	18	22	19
EABT5616	2	6	16	7	4	1	4
EABT5617	12	28	0	0	0	0	0
EABT5618	0	2	1	0	1	1	0
EABT5619	1	0	2	0	1	0	0
EABT562	1	0	6	10	0	1	2
EABT5620	4	2.99	3	3	3	1	4
EABT5621	1309.88	1731.92	2726.11	2647.46	1474.53	1354.13	1132.12
EABT5622	5	2	1	8	0	0	1
EABT5623	1480.69	3162.65	3114.36	8415.89	2640.85	1761.74	2518.93
EABT5624	427	1438.22	1685.16	1278.11	166	91	61

EABT5625	1316.69	2130.87	2756.86	3462.16	1548.52	977	1005.03
EABT5626	0	0	12	0	1	0	0
EABT5627	0	2	14	29	10	0	0
EABT5628	2078.76	2874.15	3659.93	4482.36	2702.33	1552.88	1672.87
EABT5629	647	1048	1446.92	2665	1541.04	687	507.03
EABT563	0	1	0	0	3	1	7
EABT5630	139.4	13	1	1354	1260.3	1	5
EABT5631	1	1	3	1	0	0	7
EABT5632	0	0	9	0	0	0	0
EABT5633	0	1	1	0	2	0	2
EABT5634	581	1048.52	3991.26	4440.13	5608.02	710	827.71
EABT5635	7	19	15.98	2	4	8	21
EABT5636	0	1	1	6	8	0	1
EABT5637	0	0	16	2	0	0	0
EABT5638	3	0	6	1	36	0	1
EABT5639	1	0	1	4	1	0	2
EABT564	1	0	1	10	0	0	0
EABT5640	5767.15	9695.08	5567.91	11464	5301.61	6661.5	6917.14
EABT5641	0	0	1	2	2	0	0
EABT5642	0	2	2	2	0	2	4
EABT5643	0	2	4	2	1	0	2
EABT5644	3	3	7	14	5	0	1
EABT5645	2	2	11	5	0	0	1
EABT5646	8.03	19.99	29	29	6	3	5
EABT5647	2	14	8	36	6	1	6
EABT5648	560	752	984.33	2506.05	722.36	538	536
EABT5649	1	0	0	0	1	9.01	2
EABT565	592.49	1111.9	1488.99	2140.25	890.18	592.96	719.94
EABT5650	1	2	3	0	2	1	0
EABT5651	1	3	14	1	0	0	1
EABT5652	1	1	26	3	4	2	1
EABT5653	0	0	13	0	0	0	0
EABT5654	0	1	1	3	1	0	2
EABT5655	1101	1968.06	1366.42	1435.99	758.7	771	767
EABT5656	0	0	4	6	0	0	0
EABT5657	0	1	0	20	2	0	1
EABT5658	377.67	574.36	509.1	2420.3	660.2	203.04	475.1
EABT5659	0	3	0	0	1	2	1
EABT566	22	28	52	53	23	15.22	26
EABT5660	17660.9	23474.5	26930	12640	3200.64	722.46	348.79
EABT5661	19	40	32	46	64	14	26
EABT5662	0	0	12	0	0	0	0
EABT5663	0	0	8	2	2	0	0
EABT5664	621.01	517	362	455	269	760.4	315
EABT5665	0	2	5	6	0	0	1
EABT5666	1792.52	2230.95	3484.25	5461.03	1889	1616	1138.7
EABT5667	0	0	10	0	0	3	0
EABT5668	1	0	1	5	9	0	1
EABT5669	0	1	2	1	1	1	3
EABT567	1	1	0	0	2	1	2
EABT5670	99	99	62	15	3	68	20

EABT5671	1	1	3	1	0	0	0
EABT5672	695	1237.13	1681.88	3273.99	952	535.32	606
EABT5673	858.88	1273.98	848.63	1756.24	942.95	816.96	765.73
EABT5674	0	7	3	2	0	0	1
EABT5675	3107.7	4598.06	3065.81	7942.42	3761.78	2166.83	2592
EABT5676	1	7	30	2	3	0	5
EABT5677	0	3	8	1	3	4	0
EABT5678	3173.53	4801.75	6090.21	7468.46	3795.6	3951.88	3482.27
EABT5679	0	0	15	1	0	0	0
EABT568	1	12	6	8	1	9	1
EABT5680	1	4	7	8	1	1	0
EABT5681	4	0	9	8	4	0	2
EABT5682	0	6	37	4	3	0	1
EABT5683	1178.69	1514.73	881.48	1482.92	11479.3	1409.98	1434.49
EABT5684	1	5	9	2	0	0	0
EABT5685	0	0	0	0	0	0	0
EABT5686	4	5	4	0	1	0	0
EABT5687	0	1	10	0	0	0	0
EABT5688	0	1	6	7	1	0	0
EABT5689	3064.04	4624.59	3651.11	3107.72	1911.82	1829.1	2174.81
EABT569	1	1	2	6	1	1	0
EABT5690	0	0	8	0	0	0	0
EABT5691	1	2	4	3	0	1	0
EABT5692	1	5	7	1	1	0	0
EABT5693	1	3	4	2	0	1	0
EABT5694	0	1	8	0	0	0	0
EABT5695	82	104	162	174	22	48	81
EABT5696	1	2	15	2	0	2	1
EABT5697	0	0	6	3	0	0	0
EABT5698	19	2	0	0	0	102	0
EABT5699	204	337.36	533.02	535.69	305	87.97	292
EABT57	4	3	5	40	13	13	7
EABT570	20	30	7	3	33	23	71
EABT5700	1161.02	2145.85	2657.01	5949.65	1971.98	1444.84	878.13
EABT5701	1	0	8	5	1	0	2
EABT5702	3	3	19	9	2	5	1
EABT5703	0	3	7	5	1	0	6
EABT5704	0	0	3	1	0	0	0
EABT5705	1	0	40	5	1	0	1
EABT5706	1	0	0	0	3	0	7
EABT5707	4	3	6	4	2	8	6
EABT5708	38	151	92	235	108	58	35
EABT5709	5	3	62	16	3	1	3
EABT571	1429.69	1353.93	1010.59	1739.33	799.01	1054.8	867.7
EABT5710	9690.7	12663.6	4849.7	7792.34	4588.92	6614.28	8197.16
EABT5711	14	17	10	47.4	3	40	16.47
EABT5712	7	4	12	8	8	0	0
EABT5713	99	149	424	326	70	208.94	132.24
EABT5714	0	3	30	0	1	0	0
EABT5715	0	0	20	1	0	0	0
EABT5716	0	0	0	2	37	0	0

EABT5717	2	0	8	0	0	1	1
EABT5718	2	0	18	1	0	2	1
EABT5719	0	0	3	2	0	0	0
EABT572	2	1	12	1	1	4	1
EABT5720	1	0	48	0	0	0	1
EABT5721	0	0	13	1	0	0	0
EABT5722	1096.03	1949.58	1471.35	3602.63	898.4	1021.15	824.74
EABT5723	885.46	1093.06	1207.62	2551.7	1029.01	777.24	709.89
EABT5724	6859.33	6524.79	6319.13	5135.15	3595.92	5813.83	5317.86
EABT5725	3	1	17	0	0	0	0
EABT5726	2	6	6	4	2	2	1
EABT5727	0	0	10	5	0	1	0
EABT5728	6	15	44	16	8	1	4
EABT5729	12057	15994.1	16723.1	34696.6	8583.64	13381.5	7345.47
EABT573	1	0	3	3	1	0	1
EABT5730	0	2	4	2	1	0	1
EABT5731	1	0	1	3	0	0	1
EABT5732	0	1	46	1	0	0	0
EABT5733	1	0	5	1	0	0	0
EABT5734	0	9	5	40	9	0	0
EABT5735	861	1466.98	1475.96	1669.05	822.01	662	720.26
EABT5736	0	0	2	0	72	0	328.27
EABT5737	0	7	32	12	3	5	10
EABT5738	10	8	1	2	250	12	17
EABT5739	1128	1427	853	1825	710	1320	1247
EABT574	5345.3	6965.05	6277.06	10339.4	4408.66	3418.5	4120.9
EABT5740	5955.63	822.36	36	37	125	69	171.04
EABT5741	1	1	0	0	0	0	17
EABT5742	1	0	2	0	1	1	3
EABT5743	2	3	2	2	0	1	6
EABT5744	27102.8	5999.46	1877.94	2768.81	2251.28	905.23	1178.71
EABT5745	1	3	31	7	1	1	0
EABT5746	5479.03	5809.88	3970.97	3497.68	2826.31	7087.18	6925.29
EABT5747	3	0	10	2	4	1	0
EABT5748	3	8	3	30	10	0	5
EABT5749	170.1	273.81	148	653	316.82	4	140
EABT575	0	0	8	0	0	0	0
EABT5750	1	12	57	4	2	0	1
EABT5751	0	3	4	0	1	0	1
EABT5752	7	5	28	16	1	8	5
EABT5753	5	1	1	0	0	9	0
EABT5754	0	1	4	2	0	0	0
EABT5755	3	6	11	0	2	1	0
EABT5756	1	1	0	11	1	0	0
EABT5757	0	0	1	2	0	0	48
EABT5758	116.3	145.94	83.02	177.98	125.67	71.8	123.36
EABT5759	0	1	3	1	1	0	0
EABT576	1	13	7	0	1	9	5
EABT5760	0	0	1	1	0	0	1
EABT5761	0	1	15	2	1	0	0
EABT5762	0	2	18	7	0	0	0

EABT5763	0	0	30	3	1	0	0
EABT5764	18	19	15	4	12	0	1
EABT5765	33	11	0	1	2	11	14
EABT5766	1	6	7	0	0	1	0
EABT5767	0	8	8	14	0	0	0
EABT5768	1	1	23	2	0	0	0
EABT5769	0	0	4	1	0	0	0
EABT577	6	8	11	1	1	1	3
EABT5770	18	6	4	2	0	22	0
EABT5771	0	1	2	0	0	0	0
EABT5772	2	2	2	1	1	0	2
EABT5773	0	2	2	1	0	0	0
EABT5774	1	1	1	0	0	2	0
EABT5775	9	12	35	23	11	17	8
EABT5776	11278	10072.9	5669.45	8393.8	9140.63	14184.5	10822.1
EABT5777	2	12	127	18	6	0	0
EABT5778	1	0	0	7	1	1	0
EABT5779	3	3	15	6	1	0	0
EABT578	1	3	3	1	1	0	0
EABT5780	7	17	37.01	9	3	12	20
EABT5781	1	0	12	0	0	1	1
EABT5782	1	0	5	0	0	0	0
EABT5783	1	3	3	1	1	0	2
EABT5784	457.01	692.24	172	193.89	163.57	206.15	501.03
EABT5785	3	41	32	595	1330.38	3	21
EABT5786	0	0	4	0	3	0	1
EABT5787	4454.7	5882.45	2700.88	5739.26	3465.6	6032.81	7048.92
EABT5788	11943.9	18922	21916.6	11256.5	11279.5	3132.16	5584.34
EABT5789	0	0	0	0	0	0	0
EABT579	1	7.84	7	2	2	4	2
EABT5790	0	4	18	0	1	0	1
EABT5791	4	4	5	12	5	5	2
EABT5792	0	0	5	2	0	1	1
EABT5793	6	6	6	1	0	1	2
EABT5794	0	3	4	8.77	9	0	3
EABT5795	1	8	3	1	0	1	2
EABT5796	437.42	643.07	544.25	960.94	415.4	309.72	257.67
EABT5797	2	5	148	0	0	1	1
EABT5798	0	0	10	0	1	0	0
EABT5799	856.27	1296.24	886	2334.69	1296.14	1226.92	1545.35
EABT58	7	22	58	24	11	3	6
EABT580	115	340.39	681.49	398.81	114.7	211.62	140.14
EABT5800	59	102	66	67	88	84	132
EABT5801	1	1	4	1	0	0	1
EABT5802	9913.3	18009.1	17295.8	17084.6	20565.8	8997.86	16579.5
EABT5803	3	11	13	6	2	2	2
EABT5804	5	19	38	11	0	2	0
EABT5805	1	2	7	0	1	2	0
EABT5806	2	4	14	5	5	3	1
EABT5807	2	1	0	0	6	3	9
EABT5808	3	1	5	1	1	0	0

EABT5809	2	0	3	0	3	15	3
EABT581	0	0	0	6	0	0	0
EABT5810	0	0	0	3	5	0	1
EABT5811	3	5	33	4	5	2	5
EABT5812	42	6	3	28	10	9	12
EABT5813	3	5	11	8	1	2	5
EABT5814	59	20	6	2	28	208	327.96
EABT5815	0	6	1	0	6	5	0
EABT5816	1	4	6	4	0	0	0
EABT5817	0	2	5	0	1	1	0
EABT5818	15656	16288.7	11361.7	13842	10226.4	18103.9	13807.7
EABT5819	657.9	1099.52	476.49	1281.84	764.1	569.53	679.73
EABT582	0	1	6	1	0	2	1
EABT5820	2	0	16	4	0	0	4
EABT5821	0	0	10	2	0	0	0
EABT5822	2	2	22	7	5	2	1
EABT5823	0	4	7	4	1	0	0
EABT5824	0	1	4	1	0	0	0
EABT5825	4	3	1	0	0	0	1
EABT5826	15	36	29	4	17	15	8
EABT5827	1	3	9	0	0	0	0
EABT5828	0	1	3	0	0	0	1
EABT5829	9729.64	7049.96	3703.84	6380.76	1956.28	11367.8	5833.87
EABT583	3978.95	5678.81	2851.33	15417	3036.06	2496.88	5113.54
EABT5830	40	94	102	105.11	117.99	21	22
EABT5831	0	0	4	2	0	0	0
EABT5832	0	1	0	0	0	3	1
EABT5833	0	0	0	0	1	2	4
EABT5834	5	14	45	2	1	5	5
EABT5835	10	179	86	6	235.83	10	15
EABT5836	3	12	1	7	1	1	3
EABT5837	240	561.95	712.96	2535.04	619.01	125	315
EABT5838	1	1	6	1	0	0	0
EABT5839	0	0	31	0	0	0	0
EABT584	360	507.74	391	1187.25	314	283.94	318.98
EABT5840	0	3	7	2	1	0	0
EABT5841	0	0	33	2	0	0	0
EABT5842	0	0	12	0	0	0	0
EABT5843	1	10	2	14	1	1	3
EABT5844	401	612	650	1324.59	370.01	319	299
EABT5845	21	51	42.96	5.72	63	69	201.01
EABT5846	0	0	8	4	0	0	0
EABT5847	1	9	3	36	17	1	4
EABT5848	2	10	3	10	4	3	7
EABT5849	2	8	8	11	3	1	0
EABT585	0	0	11	0	2	0	4
EABT5850	1	0	20	2	2	0	1
EABT5851	0	2	1	9	4	0	0
EABT5852	142.42	118.94	80.09	53.29	25	56	67.01
EABT5853	420.06	708.02	408.95	916.92	585	264	354
EABT5854	0	0	13	1	2	1	0

EABT5855	1	0	4	7	0	0	0
EABT5856	329	22	41.98	5	1	72	1
EABT5857	1	5	4	3	21	0	1
EABT5858	0	2	0	1	0	5	8
EABT5859	3	23	59	51	14	1	2
EABT586	0	4	6	5	0	0	0
EABT5860	0	0	0	2	4	0	1
EABT5861	0	3	1	2	0	0	0
EABT5862	0	1	0	5	0	0	0
EABT5863	3869.29	5617.13	4649	5223.02	4457.56	4283.69	5339.02
EABT5864	3700.17	4286.08	4070.83	5737	3475.98	2980.47	2368.47
EABT5865	1427.99	2167.63	1957.73	4315.3	1997.68	1307.98	1396.98
EABT5866	0	2	4	2	0	0	0
EABT5867	0	0	0	6	0	0	0
EABT5868	0	0	7	0	1	0	0
EABT5869	4	16	17	2	2	0	1
EABT587	2	3	7	11	0	0	0
EABT5870	1552.77	2223.68	1033	1095.89	1035.4	1073	1130
EABT5871	4642.8	6895.49	1292.82	2914.53	2251.24	4494.43	5324.4
EABT5872	0	1	4	3	21	0	3
EABT5873	1	0	6	16	1	1	1
EABT5874	0	2	3	4	0	1	3
EABT5875	0	2	0	92	6	0	0
EABT5876	2	4	11	5	5	0	1
EABT5877	0	0	12	0	0	0	0
EABT5878	0	3	4	22	1	1	6
EABT5879	0	0	0	7	2	0	1
EABT588	2	3	0	0	0	0	2
EABT5880	7	11	64	8	8	1	6
EABT5881	0	4	0	1	0	0	0
EABT5882	0	1	2	20	0	0	0
EABT5883	6	13	6	43	3	5	6
EABT5884	5	15	37.01	11	4	8	6
EABT5885	0	0	3	3	0	0	0
EABT5886	2	0	2	0	2	17	3
EABT5887	3	11	33	1	0	0	1
EABT5888	0	1	9	27	0	0	0
EABT5889	3	7	20	72	15	0	7
EABT589	4	7	13	1	5	2	0
EABT5890	0	0	0	0	0	0	2
EABT5891	392	555.8	411.15	836.16	623.46	354.17	280.32
EABT5892	0	0	9	0	0	0	0
EABT5893	1002.07	1693.45	1112.85	3736.01	1235.04	681.08	609.71
EABT5894	1	2	10	4	2	1	0
EABT5895	4	5	2	0	1	1	0
EABT5896	0	0	5	1	0	0	0
EABT5897	2	6	23	1	0	0	3
EABT5898	0	0	10	0	0	0	0
EABT5899	5	3	0	0	3	20	6
EABT59	0	2	3	2	0	0	0
EABT590	0	2	3	1	0	0	2

EABT5900	13843.2	24282.2	43037.6	54371	6486.95	13310.8	7515.61
EABT5901	38	74	77	308	196.05	20	214
EABT5902	48	92	4	84	6	0	0
EABT5903	224	447	1707	1178.65	543.99	262	142
EABT5904	329.36	789.6	476.82	908.71	426.61	266.58	529.51
EABT5905	0	0	3	0	1	0	5
EABT5906	0	2	0	5	0	0	0
EABT5907	1	3	9	2	0	3	2
EABT5908	1311.65	2331.68	2214.53	4429.63	2010.44	1057.77	1361.76
EABT5909	1	1	1	8	0	0	0
EABT591	5	3	30	2	2	1	2
EABT5910	1	1	2	6	1	0	0
EABT5911	1	0	6	3	0	2	1
EABT5912	3106.59	4859.21	5218.44	4860.2	2333.23	1908.54	1935.96
EABT5913	2	6	7	14	1	1	3
EABT5914	14	6	36.19	12	25	7	25
EABT5915	12	3	12	10	4	2	3
EABT5916	1	3	2	2	0	0	0
EABT5917	2	5	10	3	3	0	3
EABT5918	59	89.54	45	428.19	72.88	13	22.04
EABT5919	52.72	81.96	203.45	147.29	23	48.09	57
EABT592	1	2	1	1	0	0	0
EABT5920	0	1	4	2	1	0	0
EABT5921	0	0	10	0	0	1	0
EABT5922	2	10	19	21	1	1	0
EABT5923	4	12	7	4	8	1	0
EABT5924	4	8	0	0	5	0	0
EABT5925	20	24	160.58	32	9	25.03	15
EABT5926	2379.15	5239.98	1948.17	1603.16	3597.8	86.27	462
EABT5927	7	9	15	12	3	1	6
EABT5928	0	1	0	5	0	0	0
EABT5929	0	1	2	1	0	2	0
EABT593	2	2	1	0	1	0	0
EABT5930	0	1	5	0	0	0	1
EABT5931	4	11	22	10	4	3	0
EABT5932	2	12	2	0	0	0	0
EABT5933	458	609	481.28	634.9	619.94	440.84	373.99
EABT5934	0	2	3	0	0	0	0
EABT5935	3	5	0	0	0	0	0
EABT5936	0	2	0	2	0	1	0
EABT5937	3	0	0	6	1	0	1
EABT5938	0	0	3	2	0	0	0
EABT5939	0	6	12	0	0	0	0
EABT594	0	0	14	0	2	1	0
EABT5940	5	12	211.98	104.9	183	17	4
EABT5941	0	0	6	0	0	1	0
EABT5942	1	4	4	1	0	0	0
EABT5943	104	142	13	1	95.93	2	490
EABT5944	1822.08	1785.65	556	1608	1251.99	1441	2939.07
EABT5945	1	0	7	0	0	0	0
EABT5946	3677	5646.66	11752	4979.98	7572.84	2107.97	1513

EABT5947	392	257	8	0	1	1	0
EABT5948	0	0	1	8	0	0	0
EABT5949	0	0	2	1	1	0	1
EABT595	0	0	15	0	1	0	0
EABT5950	577	819	6	0	88	221	331
EABT5951	4	1	0	4	0	0	0
EABT5952	2	1	4	2	0	7	3
EABT5953	546.93	633.11	308.34	304	300.95	518.88	331.02
EABT5954	0	8	0	21	2	0	0
EABT5955	1838.07	3042.77	3472.5	3320.73	2309.83	1457.15	1708.51
EABT5956	0	0	22	0	0	0	2
EABT5957	0	0	4	0	0	0	0
EABT5958	0	0	0	1	0	0	1
EABT5959	1	0	2	0	2	1	0
EABT596	383.98	934	1958.17	4438	1216.98	253	354
EABT5960	2	10	1053	3	2	1	1
EABT5961	2	0	3	0	0	2	2
EABT5962	7	16.7	12	43	12	2	14
EABT5963	0	0	5	0	0	0	0
EABT5964	3	5	17	2	2	1	4
EABT5965	0	10.55	832.07	31.03	27	6	43
EABT5966	0	0	4	0	0	0	0
EABT5967	7	12	38	19	4	2	8
EABT5968	2	1	7	2	1	1	0
EABT5969	2	4	14	2	4	1	0
EABT597	2	2	5	8	2	4	0
EABT5970	2	0	4	0	1	5	1
EABT5971	0	3	3	6	0	0	0
EABT5972	0	0	17	1	1	0	0
EABT5973	0	0	7	0	0	4	1
EABT5974	0	1	6	0	1	0	0
EABT5975	235	377	537	820	280	182	144
EABT5976	146	321.05	618.03	505.6	95.12	157	97
EABT5977	650.66	1360.73	790.06	564	1027.91	696.97	641.92
EABT5978	3	2	7	4	0	0	1
EABT5979	3	4	16	14	3	7	3
EABT598	49	73.01	185.04	538.86	142	24	58
EABT5980	1	0	5	0	0	0	0
EABT5981	3106.71	3707.39	3699	1313.24	1728.02	7262.55	7506.23
EABT5982	0	0	5	2	0	0	0
EABT5983	0	0	2	1	1	0	0
EABT5984	3	16	24	17	3	6	5
EABT5985	1	5	3	3	0	0	0
EABT5986	0	0	4	2	0	0	0
EABT5987	144	307	283.89	415.68	71	164.98	198
EABT5988	58	26	57	12	1	77	0
EABT5989	2	4	1	0	1	18	1
EABT599	1360	1653.74	1106.3	1457.43	1659.98	1293.52	1028
EABT5990	1	1	10	0	2	0	0
EABT5991	2	0	1	3	0	1	0
EABT5992	20.07	2	1	0	3	60	21

EABT5993	1	3	0	1	0	1	0
EABT5994	470.98	848.02	813.8	897.94	464.81	419.68	231.94
EABT5995	0	0	6	0	0	0	0
EABT5996	0	0	6	0	0	0	0
EABT5997	662.48	807.67	728.07	893.24	1206.15	534.12	474.01
EABT5998	0	2	8	1	1	0	0
EABT5999	4	18	13	17	3	10	6
EABT6	0	1	15	7	3	0	0
EABT60	1	6	89	5	0	5	2
EABT600	1781	3643.99	1285.12	1335.57	344	1086.93	584.93
EABT6000	1	2	15	4	0	1	1
EABT6001	1	2	3	1	0	1	0
EABT6002	9	42	53	29	44	6	68
EABT6003	20	12	40	1	5	21	18
EABT6004	21331.1	19139.5	12493.5	19511.4	15955.6	23634.2	20745.2
EABT6005	1	0	21	1	1	1	0
EABT6006	1	0	10	1	0	2	0
EABT6007	2	4	11	9	0	7.85	7
EABT6008	23	46	23	4	30	59	64
EABT6009	10814.1	1998.96	438	483	365	2224.13	497.44
EABT601	4	4	0	0	0	3	6
EABT6010	0	2	13	0	0	0	0
EABT6011	1	1	29	2	0	0	0
EABT6012	1	1	0	4	0	0	0
EABT6013	3	2	3	0	0	0	0
EABT6014	1	0	0	0	0	5	2
EABT6015	1123.05	1764.86	1463	2968.2	1123.09	843.02	921.68
EABT6016	5	7	12	5	4	3	3
EABT6017	332	592	744.21	2548.01	83	134	272
EABT6018	0	2	5	5	0	0	1
EABT6019	0	2	33	0	1	0	0
EABT602	0	1	4	7	0	2	2
EABT6020	2886.91	5337.85	4516.04	9823.71	2067.05	2855.02	2688.15
EABT6021	2	19	0	2	1	3	15
EABT6022	0	7	12	4	21	0	1
EABT6023	1	2	8	1	1	2	1
EABT6024	0	3	3	1	1	1	0
EABT6025	19	0	1	2	4	2	0
EABT6026	4	6	2	0	5	2	19
EABT6027	2	4	10	11	0	0	0
EABT6028	5	1	0	0	0	11	0
EABT6029	0	0	0	6	0	0	0
EABT603	1188	1576	973.43	2413.74	986	735.01	833
EABT6030	364.89	504.91	529.29	750.43	624	329	440
EABT6031	0	0	7	0	0	0	0
EABT6032	0	0	2	0	0	1	2
EABT6033	0	1	10	1	0	0	0
EABT6034	0	9	4	0	0	1	1
EABT6035	44	137	860.37	11584.8	3351	5	203
EABT6036	12	34	282	44	1	0	0
EABT6037	0	1	19	1	0	1	0

EABT6038	1	1	0	0	0	0	9
EABT6039	574	314	2	1	47	600	260
EABT604	11	39	133	6	1	5	1
EABT6040	0	1	1	1	0	0	1
EABT6041	64.91	100.98	135	283.28	78.19	37	44
EABT6042	0	4	5	0	6	0	8
EABT6043	1	1	1	2.93	1	1	0
EABT6044	0	1	5	0	0	0	0
EABT6045	2	2	4	4	1	0	0
EABT6046	0	0	0	7	0	0	0
EABT6047	0	1	2	0	15	0	2
EABT6048	0	1	7	0	0	0	0
EABT6049	1273.35	2424.08	6188.2	5215.58	2089.7	1630	1792.43
EABT605	4105.19	10060.6	8404.94	16878.1	6526.9	1990.58	2414.87
EABT6050	46	48	72	24	14	7	20
EABT6051	1	1	1	2	0	1	0
EABT6052	363.07	547.03	318	1103.98	253.13	325	212
EABT6053	1	9	50.73	8	1	0	1
EABT6054	1	2	27	0	3	2	1
EABT6055	1	5	19	13	13	0	5
EABT6056	2	5	4	21	4	0	1
EABT6057	3	5	23	5	3	8	3
EABT6058	1	0	2	3	4	1	4
EABT6059	5532.02	5926.49	2028	3834.97	20572.7	4791.4	4381.05
EABT606	0	2	18	26	1	0	1
EABT6060	0	0	9	3	0	0	0
EABT6061	3	8	2	22	2	0	0
EABT6062	1060.25	1720.22	2208.14	3065.97	2974.61	665	1686.21
EABT6063	1709.49	4085.52	3173.58	5870.86	1049.9	851	917.09
EABT6064	1	3	5	0	0	0	1
EABT6065	0	1	10	3	2	4	2
EABT6066	6	4	2	24.14	13	2	0
EABT6067	2	1	14	9	4	0	6
EABT6068	0	0	3	6	1	0	0
EABT6069	4	17	74	161	874	13	251.07
EABT607	7	8	31	6	1	1	1
EABT6070	1128.1	1427.97	802.03	2987.7	1001	543	679.99
EABT6071	4145.05	6001.32	3221.52	5966.87	2406.07	5093.95	4331.75
EABT6072	2	3	4	1	2	4	1
EABT6073	2	2	4	9	2	4	6
EABT6074	0	2	3	0	0	1	0
EABT6075	0	1	7	1	0	1	0
EABT6076	0	1	7	0	0	0	0
EABT6077	2	2	15	2	1	2	1
EABT6078	65	123	93	168	91	2	578
EABT6079	465.02	864.86	1394.65	2297.71	1000.57	464	459.11
EABT608	12	4	22	5	2	7	21
EABT6080	0	3	3	4	2	0	3
EABT6081	0	2	1	1	0	0	1
EABT6082	0	0	4	1	0	0	0
EABT6083	4665.4	7572.69	5890.35	10421	3520.01	2281	4817.03

EABT6084	0	0	11	0	2	0	0
EABT6085	0	0	5	0	0	0	0
EABT6086	0	3	1	6	0	0	0
EABT6087	0	1	1	7	0	0	1
EABT6088	0	0	0	0	0	0	0
EABT6089	0	2	1	5	2	0	0
EABT609	2124.19	150	21	4	19	1477	48
EABT6090	0	0	5	1	0	0	0
EABT6091	3	1	0	0	0	0	1
EABT6092	2	9	2	6	3	0	0
EABT6093	685.94	1097.24	579	2225.05	814.32	433.98	825.19
EABT6094	0	2	7	1	4	0	2
EABT6095	3269.53	4923.63	2721.3	6898.69	3524.25	2691.56	2088.88
EABT6096	1081.08	1788.38	1687.78	2716.92	648.99	1620	579
EABT6097	1	1	5	6	6	0	1
EABT6098	0	1	6	0	0	0	0
EABT6099	1	3	15	3	0	0	1
EABT61	0	0	0	0	0	0	0
EABT610	1	8	2	24	2	0	0
EABT6100	181	134	73	212	61	43	67
EABT6101	11	11	3	1	1	3	2
EABT6102	7	9	58	17	7.96	18	8
EABT6103	878	2677	6318.54	27056.6	3010.95	845.85	2797.96
EABT6104	0	3	41	5	0	0	0
EABT6105	0	3	2	9	0	0	1
EABT6106	1	5	27	9	0	1	1
EABT6107	96	237	149	305	169	38	105
EABT6108	1	0	0	0	0	6	0
EABT6109	3555.9	2604.24	1219.05	1532.17	4409.08	3789.11	4029.2
EABT611	2	0	21	2	1	0	1
EABT6110	0	1	10	1	1	1	0
EABT6111	1	0	0	0	0	3	0
EABT6112	0	2	4	2	2	0	6
EABT6113	3	2	1	0	0	5	0
EABT6114	0	3	3	0	1	0	0
EABT6115	668	1155.21	1390.86	2451.4	1507.8	925.93	958.64
EABT6116	1	6	11	3	2	1	10
EABT6117	0	0	13	0	0	0	1
EABT6118	0	1	0	1	2	0	0
EABT6119	1	3	3	7	0	0	0
EABT612	0	1	0	2	1	2	0
EABT6120	27	139	13	17	147	0	1
EABT6121	5	1	1	0	0	0	0
EABT6122	5	21	19	40	24.02	3	7
EABT6123	21	29	3	3	7	5	26
EABT6124	923.55	158.75	3	57	0	1076.88	1
EABT6125	1	4	3	8	0	2	0
EABT6126	0	1	3	1	1	0	0
EABT6127	14343.2	8281.05	4187.61	5498.31	7202.48	15574.5	13567.1
EABT6128	0	1	0	0	2	2	2
EABT6129	0	1	1	0	2	0	0

EABT613	0	4	21	2	0	0	0
EABT6130	115.22	1	1	0	0	13	0
EABT6131	7	11	2	14	41	2.63	49
EABT6132	0	1	5	0	0	0	0
EABT6133	1	4	5	1	1	0	0
EABT6134	7	3	0	0	0	0	0
EABT6135	3033.34	4500.99	2523.42	6368.43	3011.97	3155.46	2965.04
EABT6136	1	0	34	0	0	1	2
EABT6137	0	0	1	0	4	0	1
EABT6138	122	487	617.98	143	99	294.06	259.35
EABT6139	2	1	1	0	0	0	0
EABT614	8	8	10	3	0	16.57	2
EABT6140	2688.08	4033.24	2550.98	5634.99	2511.89	1617.53	2311.63
EABT6141	2	1	0	0	1	6	3
EABT6142	0	1	5	1	0	0	0
EABT6143	2	0	5	0	0	0	0
EABT6144	0	0	1	0	0	1	0
EABT6145	0	0	12	0	0	0	0
EABT6146	0	2	5	15	5	0	0
EABT6147	0	2	3	4	0	0	3
EABT6148	0	7	4	20	1	2	2
EABT6149	0	2	8	3	0	0	1
EABT615	22	11	16	31	9	17	11
EABT6150	3	2	65	2	0	0	1
EABT6151	10	9	16	33	64	26	125.04
EABT6152	4124.73	6734.3	6194.48	5717.91	2449.28	2154.1	1701.59
EABT6153	1	8	6	4	8	0	2
EABT6154	0	0	7	1	0	0	0
EABT6155	58	105	44	92	74	31	35
EABT6156	0	0	2	1	0	0	0
EABT6157	1	1	3	0	2	1	0
EABT6158	706.58	549.49	493	1257.53	525.45	94.83	286.05
EABT6159	38.01	90	109	273	49	21	52
EABT616	3.88	7	3	0	4	9	46
EABT6160	0	2	11	75	14	0	3
EABT6161	0	4	6	13	7	5	6
EABT6162	2	10	152	5	1.13	7	1
EABT6163	0	2	3	0	0	1	1
EABT6164	16545.1	14027	1077.59	572	1958.02	11375.9	16999.9
EABT6165	0	0	4	1	0	0	0
EABT6166	53	96	87	31	20	0	3
EABT6167	0	0	2	0	1	0	0
EABT6168	0	0	37	0	0	0	0
EABT6169	1	0	1	3	0	0	0
EABT617	6	17	70	58	39	1	29
EABT6170	0	1	21	3	1	0	1
EABT6171	0	5	2	2	0	0	0
EABT6172	0	3	0	0	0	0	0
EABT6173	1167.91	1936.97	1436.05	3085.25	2150	1139	1179.92
EABT6174	1	2	1	3	0	0	1
EABT6175	2	4	16	9	5	0	2

EABT6176	7	4	86	21	5	1	0
EABT6177	0	2	0	1	0	0	0
EABT6178	0	1	8	3	0	3	1
EABT6179	3684.05	5312.54	2723.03	10500.1	1522.3	20.59	22.98
EABT618	0	6	2	0	0	2	5
EABT6180	746.99	1637.99	4783.28	8404.2	1049	1164.02	680.7
EABT6181	0	0	1	0	1	0	0
EABT6182	1	1	14	1	1	0	1
EABT6183	1	0	37	0	0	0	1
EABT6184	880.03	1075.04	697	1687.87	638	576	388
EABT6185	1	1	5	0	0	0	0
EABT6186	0	0	1	13	0	0	0
EABT6187	2	1	7	6	1	0	1
EABT6188	0	0	4	1	0	0	0
EABT6189	2	3	7	8	2	3	2
EABT619	205.01	128.02	22.02	1	0	433.42	0
EABT6190	0	6	1	0	0	2	1
EABT6191	0	0	5	0	0	0	0
EABT6192	0	0	7	0	0	0	1
EABT6193	5	2	32	4	2	0	0
EABT6194	2	3	10	0	0	0	0
EABT6195	0	1	5	11	0	3	0
EABT6196	1	1	25	1	3	0	0
EABT6197	2	3	1	1	2	0	1
EABT6198	2	0	15	1	1	1	0
EABT6199	0	1	3	0	55	0	0
EABT62	1191824	1130123	998808	593559	1418583	323371	662340
EABT620	0	2	33	9	0	0	2
EABT6200	1	2	31	5	1	0	2
EABT6201	868.21	1149.02	1761.47	1196.13	595.15	415.12	536.5
EABT6202	24	18	19	4	14	0	7
EABT6203	0	0	5	0	0	0	0
EABT6204	0	12	4	0	0	0	1
EABT6205	7479.05	5414.97	1169.41	1631.98	2321.09	4343.67	4989.22
EABT6206	0	0	0	0	4	0	0
EABT6207	453.93	609	569.96	3734.59	561	531	407.42
EABT6208	1	0	25	13	0	1	0
EABT6209	6	10	34	17	8	4	15
EABT621	1	3	5	2	2	0	0
EABT6210	3	4	28	1	0	2	0
EABT6211	0	1	5	0	1	4	0
EABT6212	1	0	2	2	2	0	0
EABT6213	0	0	0	0	0	6	0
EABT6214	0	1	1	0	0	0	1
EABT6215	0	0	3	4	0	1	0
EABT6216	0	0	7	1	0	0	0
EABT6217	9507.4	15389	9179.33	13094.4	8708.74	8600.53	8438.25
EABT6218	0	3	2	2	0	1	0
EABT6219	3162.39	6168.37	319.6	135	571.89	2333.26	567.28
EABT622	1	7	13	2	1	1	0
EABT6220	1447.53	2325.41	2679.65	5934.94	1529.01	1566.01	1525.67

EABT6221	103	253	283	65	259	8	59
EABT6222	3	16	20	1	2	2	2
EABT6223	0	1	0	1	4	0	1
EABT6224	1	0	0	1	8	0	0
EABT6225	3	2	0	5	1	0	0
EABT6226	0	3	4	9	1	3	0
EABT6227	0	1	4	3	9	0	0
EABT6228	1	1	1	0	0	0	0
EABT6229	2144.17	2595.59	2868.89	4008.28	1673.6	3761.01	2447.25
EABT623	165	293	3	0	156	317.01	590
EABT6230	0	1	1	0	0	0	0
EABT6231	0	0	7	16	1	0	1
EABT6232	1	2	3	3	3	0	0
EABT6233	1	1	2	6	0	0	0
EABT6234	0	3	5	3	0	0	0
EABT6235	0	2	26	8	0	1	0
EABT6236	0	3	7	5	2	0	0
EABT6237	2	3	2	0	0	4	1
EABT6238	791.98	1160.36	956.68	1704.62	739.1	815.79	718.39
EABT6239	0	0	1	7	0	0	0
EABT624	1	4	8.71	7	0	4	2
EABT6240	0	0	2	0	1	0	1
EABT6241	0	1	0	1	3	0	4
EABT6242	1	1	17	4	0	0	0
EABT6243	6	1	2	2	3	0	1
EABT6244	0	0	0	9	0	0	0
EABT6245	0	2	22	0	1	1	0
EABT6246	454.87	464.78	300.07	490.37	567.98	274	255.03
EABT6247	2	0	1	1	3	0	1
EABT6248	5	9	2	1	1	8	9
EABT6249	2	1	17	9	24	4	0
EABT625	0	1	16	0	0	0	1
EABT6250	1	1	3	2	1	0	1
EABT6251	0	1	1	5	1	0	0
EABT6252	2	9	3	11	8	1	7
EABT6253	0	2	3	0	0	0	0
EABT6254	1995.17	2112.64	1343.12	1771.67	848.84	937.88	1121.98
EABT6255	1	0	5	1	0	0	0
EABT6256	0	1	1	2	0	0	0
EABT6257	0	1	29	1	0	1	0
EABT6258	761.55	1473.33	2751.27	3824.14	712	774.01	1294.94
EABT6259	40	3	0	0	0	3	0
EABT626	65.95	127.55	121.15	41	30.99	7.01	4
EABT6260	10254.2	12009.6	6963.94	20677.8	5932.59	11061.8	8244.03
EABT6261	8	27.97	2	29	26	6	35
EABT6262	2	8	5	0	0	0	1
EABT6263	0	2	1	2	0	0	0
EABT6264	23	15	32.99	69	9	1	8
EABT6265	3240.14	2494.05	741.38	2189.69	1000.95	2183.96	1461.9
EABT6266	1	3	6	0	0	0	1
EABT6267	7	16	104	12	4	5	1

EABT6268	2	0	3	0	0	1	2
EABT6269	0	1	2	0	0	2	0
EABT627	0	1	24	2	0	0	0
EABT6270	0	2	11	1	0	0	0
EABT6271	33	32	14	10	1	6	10
EABT6272	1	1	8	1	2	0	4
EABT6273	5.98	7	9	151.18	76.93	6	12
EABT6274	2	8	16	0	0	0	0
EABT6275	1	1	8	41	0	0	0
EABT6276	1	4	4	24	13	1	1
EABT6277	23	75	43	357.9	86	70	167
EABT6278	0	0	11	0	0	0	0
EABT6279	165	280.39	513	2024	404	136	161
EABT628	815	983.15	115	202	67	17	3
EABT6280	40	86.01	177.99	17	27	18.55	21
EABT6281	7	23	9	32	17	6	24
EABT6282	0	2	0	5	0	1	6
EABT6283	1	2	0	0	0	2	0
EABT6284	263	293	106	283	227	195	246
EABT6285	17	23.27	20	28	21	13	36
EABT6286	0	0	1	5	1	0	0
EABT6287	17	30	44	8	3	8	14
EABT6288	320	432	372	954.04	259	380.03	284.01
EABT6289	3	6	6	3	3	0	5
EABT629	2	3	4	4	95	0	0
EABT6290	14580.6	12721.2	14275	14889.2	10043.1	10359.6	9918.98
EABT6291	740.83	1535.99	2359.5	2988.92	763.04	684.65	764.97
EABT6292	6	49	15	62	8	19	8
EABT6293	23	32	52	40	20.22	11	20
EABT6294	0	3	2	1	0	1	0
EABT6295	9545.37	7530.34	9624.49	10053.5	10879	8080.08	14195
EABT6296	178	196	278	66	146	135	144
EABT6297	0	0	2	2	1	0	0
EABT6298	6	12	11	70.4	75	3	3
EABT6299	1	8	58	3	0	0	0
EABT63	0	1	14	1	0	1	2
EABT630	3	4	0	0	0	0	0
EABT6300	0	1	4	2	0	0	0
EABT6301	1355.54	1682.07	2457.65	3233.83	1666.15	987.86	1219.71
EABT6302	3116.1	6158.12	2700.29	687.29	1187.29	4170.86	5090.83
EABT6303	0	0	3	0	0	0	0
EABT6304	54165	65970.1	65860.3	36491.2	37615.7	56550.4	56850.1
EABT6305	3964.43	11854.6	2705	12080.2	3832.06	353	415
EABT6306	20	54.96	41.36	47	4	14	13
EABT6307	1824.06	2903.64	758.75	1912.92	1409.38	1127.03	1474.41
EABT6308	0	0	0	0	0	0	5
EABT6309	0	2	1	6	1	0	0
EABT631	5338.34	7106.35	7590.72	10488	4724.97	8515.93	5605.82
EABT6310	0	1	14	0	0	1	0
EABT6311	0	3	4	1	1	0	0
EABT6312	3	15	38	95	36	36	21

EABT6313	1	3	22	1	0	3	1
EABT6314	0	1	5	1	0	1	0
EABT6315	83.09	129.01	160.99	217.02	166.07	81.96	75.05
EABT6316	0	0	1	0	2	0	4.02
EABT6317	1842.32	2814.05	3591.31	5912.54	1832.24	1609.4	1539.02
EABT6318	1	0	1	2	1	0	3
EABT6319	22	161	6	6	44	38	181
EABT632	437	777	512.15	1768.45	403.38	381.67	379.39
EABT6320	2	3	4	0	0	0	0
EABT6321	5	11	24.01	16	29	1	4
EABT6322	0	2	1	2	0	0	0
EABT6323	0	0	1	0	0	0	1
EABT6324	15	1	2	1	1	41	8
EABT6325	3	10	5	6	5	1	4
EABT6326	0	1	3	0	0	7	0
EABT6327	116	401.48	166.97	1193	159	159.98	108.01
EABT6328	49	57.37	49.03	68.2	277.72	55	352.06
EABT6329	0	0	1	0	1	0	1
EABT633	0	4	9	7	0	0	1
EABT6330	2	4	1	0	3	15	17
EABT6331	5	28	4	15	3	0	11
EABT6332	0	3	5	1	0	0	0
EABT6333	6	2	3	0	0	13	0
EABT6334	8	10	8	14	7	11	19
EABT6335	0	5	7	4	0	3	4
EABT6336	1589.15	1626.8	199.81	192.07	378	3751.02	2875.16
EABT6337	5	9	5	3	13	0	0
EABT6338	0	1	3	2	11	0	0
EABT6339	0	1	2	1	1	0	3
EABT634	0	0	10	0	0	0	1
EABT6340	4	9	17	3.05	2	24	8
EABT6341	2	11	50.85	674.01	23	3	16
EABT6342	1	0	1	9	0	1	3
EABT6343	3	0	27	0	1	5	0
EABT6344	1	1	22	0	0	0	0
EABT6345	607.91	2427.92	5356.46	25.87	1944.72	121.23	711.79
EABT6346	234	879.03	1046.65	9120.97	1868.56	127.92	85
EABT6347	4	2	4	7	1	0	2
EABT6348	1	4	4	9	1	2	1
EABT6349	200.54	257.89	328.15	512.15	229.81	160.69	161
EABT635	3.96	12	19.65	30	8	4	7
EABT6350	0	0	1	0	0	0	0
EABT6351	570	869.82	495	687	450	200	648.01
EABT6352	65	12	0	1	7	163	34
EABT6353	194	328	371	57	35	1	75
EABT6354	1368.79	2321.95	1117.8	2642.09	1215.59	814.67	1363.3
EABT6355	7	103	5	3	27	41	58
EABT6356	0	3	1	3	5	1	17
EABT6357	1	4	7	10	0	0	0
EABT6358	4	14.01	15	6	7	9	24
EABT6359	831.54	1144.05	2939.33	1579.13	1427.97	116.36	143.27

EABT636	0	1	6	0	0	1	0
EABT6360	3	9	20	2	5	4	0
EABT6361	1177.06	1767.24	2085.78	3023.63	1308.67	725.99	849
EABT6362	8	16	3	9	3	12	4
EABT6363	1	2	11	1	0	0	0
EABT6364	3	2	26	14	0	0	0
EABT6365	14	31	10	56	35	7.01	8
EABT6366	0	0	28	151	0	0	16
EABT6367	1	1	15	0	0	0	1
EABT6368	4205.51	5564.04	6403.72	11331.2	5462.49	4558.25	5018.11
EABT6369	15	20	0	0	0	5	1
EABT637	2	2	27	3	0	1	2
EABT6370	15	43	6	6	3	4	16
EABT6371	0	0	5	1	0	0	0
EABT6372	3	1	0	0	0	0	1
EABT6373	2	9	8	3	0	1	3
EABT6374	5	29	135.05	16	0	15	20.8
EABT6375	0	3	26	1	0	0	0
EABT6376	0	3	6	1	0	0	0
EABT6377	2124.21	2536.04	2729.91	5439.54	964.33	2221	1632
EABT6378	0	0	0	0	0	0	0
EABT6379	0	3.04	8	5	1	0	0
EABT638	5	8	13	1	6	1	1
EABT6380	4	1	16	0	2	0	1
EABT6381	1608.38	2593.54	3779.77	5262.93	3299.48	1475.75	2153.14
EABT6382	0	0	6	0	2	0	1
EABT6383	1	1	4	1	0	0	0
EABT6384	10	2	13	4	2	2	4
EABT6385	0	2	19	0	0	0	0
EABT6386	0	0	4	1	1	0	0
EABT6387	269.17	8	1	4	0	1401	0
EABT6388	0	2	32	5	0	0	0
EABT6389	0	4	8	6	1	3	1
EABT639	0	2	3	0	0	0	0
EABT6390	1	0	9	0	0	0	1
EABT6391	1831.11	2653.6	2372.66	4954.45	2188.08	1645.45	1114.12
EABT6392	21	16	2	0	3	151	102
EABT6393	1	0	2	1	0	2	0
EABT6394	4	4	2	1	1	0	2
EABT6395	0	0	7	1	0	1	0
EABT6396	13	15	55	21	13	14	21
EABT6397	2	2	61	0	2	0	1.18
EABT6398	1	1	1	2	2	0	0
EABT6399	9217.54	17251.9	18717.3	19010.2	12990.7	10796	11245.2
EABT64	1	5	11	0	1	0	1
EABT640	0	0	2	1	1	0	0
EABT6400	7	16	13	2	1	0	1
EABT6401	1	1	10	13	1	1	0
EABT6402	7923.02	14416.5	12182.3	6834.98	12118.9	7415.51	6688.27
EABT6403	0	8	3	3	1	0	0
EABT6404	0	1	0	0	6	0	0

EABT6405	0	3	1	2	0	0	1
EABT6406	5	10	7	5	0	3	9
EABT6407	0	1	5	2	0	0	0
EABT6408	337	319.22	43	77	166	479	1391
EABT6409	0	0	0	0	0	0	20
EABT641	0	3	0	5	0	0	0
EABT6410	6	4	1	0	2	3	6
EABT6411	12	163.56	1377.01	251	350	64	118
EABT6412	0	2	25	1	3	0	0
EABT6413	0	0	8	0	0	0	3
EABT6414	3	1	25.44	1	0	0	0
EABT6415	60	45	34	10	39	28	13
EABT6416	2	3	17	6	1	1	2
EABT6417	9	7	49	21	3	3.52	4
EABT6418	3	6	57	12	0	7	0
EABT6419	194	231	202	424	217	107	130
EABT642	2	1	14	1	3	0	2
EABT6420	3	3	6	3	3	0	2
EABT6421	69047.8	68726.1	84364.9	244994	37453	68676	41823.3
EABT6422	0	0	1	10	0	0	0
EABT6423	1	3	12	2	1	0	0
EABT6424	1461.28	3276.04	2974.17	3975.97	1656.04	689	1216.85
EABT6425	2	6	4	4	0	5	0
EABT6426	3	2	2	0	0	9	0
EABT6427	0	0	2	1	1	0	0
EABT6428	1	1	2	0	2	0	6
EABT6429	0	9	1	8	4	0	0
EABT643	0	0	3	0	0	0	0
EABT6430	0	0	9	0	0	0	0
EABT6431	0	0	4	0	0	0	0
EABT6432	3	6	0	0	4	0	15.97
EABT6433	0	1	3	5	0	0	0
EABT6434	0	0	13	0	0	0	0
EABT6435	779.7	992.51	386.74	605.87	1279.44	2	11
EABT6436	1	1	2	0	1	0	1
EABT6437	0	0	3	0	0	0	1
EABT6438	269.64	1198.48	325	766.71	264	399.78	369.23
EABT6439	5	18	33	8	4	0	0
EABT644	52	227	96	1508	249	95	70
EABT6440	3	9	101	12	3	3	3
EABT6441	8	5	15	4	2	9	5
EABT6442	1	4	4	6	0	1	0
EABT6443	1	3	14	3	0	1	2
EABT6444	0	4	6	1	1	1	1
EABT6445	392.76	563.32	526.21	858.16	436.45	222.99	317.47
EABT6446	1	0	6	4	0	0	0
EABT6447	1729.2	2877.75	1785.44	3017.35	1166.05	1848.04	1384.97
EABT6448	26	5.66	0	0	1	7	1
EABT6449	1	1	1	5	0	3	0
EABT645	1819.99	3345.48	4928.74	7697.82	2528.25	1813.36	1637.74
EABT6450	28.32	59	31.76	232.03	94	23.28	44

EABT6451	0	0	14	1	0	1	0
EABT6452	1	3	8	2	1	0	2
EABT6453	14	12	27	15	3	0	4
EABT6454	0	2	6	1	0	0	0
EABT6455	1200	2025.18	2240.68	2936.84	1604	1052	1061.81
EABT6456	8	4	0	0	0	0	0
EABT6457	1	2	17	0	3	0	0
EABT6458	6	12	1	16	3	4	3
EABT6459	0	0	0	0	15	8	0
EABT646	2	4	0	0	0	0	7
EABT6460	1	4	11	2	0	0	0
EABT6461	9	40.08	29	28	1021	31	19
EABT6462	0	2	3	3	1	3	0
EABT6463	1	1	1	18	6	0	1
EABT6464	0	0	8	3	0	0	0
EABT6465	1	5	1	14	4	2	0
EABT6466	0	0	5	0	0	0	0
EABT6467	2386.19	2879.23	3184.91	3736.61	1837.48	1764	1110.99
EABT6468	1	1	1	1	2	0	1
EABT6469	0	1	2	2	1	0	2
EABT647	1	42	498	175	66	6	5
EABT6470	0	0	4	10	0	0	0
EABT6471	1	3	18	5	0	1	0
EABT6472	12	12	0	0	1	23	9
EABT6473	1	6	11	2	4	0	9
EABT6474	0	1	5	0	0	0	0
EABT6475	0	2	2	3	0	1	0
EABT6476	9	13	14	33.27	4	8	9
EABT6477	3	3	0	0	3	10	19.08
EABT6478	0	0	22	2	1	0	0
EABT6479	1	0	15	0	0	1	0
EABT648	6	1	1	2	4	0	0
EABT6480	0	2	1	2	1	0	7
EABT6481	257.79	431.01	366.54	506.29	2979.43	109	194.97
EABT6482	2374.67	44048.9	16169.8	13093.3	144	160035	464.15
EABT6483	0	0	17	3	2	0	0
EABT6484	873	1053.74	683	1706.08	791	952.01	692
EABT6485	0	3	11	1	1	0	2
EABT6486	1	0	18	0	0	0	0
EABT6487	0	0	4	0	0	0	1
EABT6488	0	1	9	3	0	0	0
EABT6489	2	4	1	0	1	0	7
EABT649	0	1	6	0	0	0	0
EABT6490	0	4	1	0	0	5	0
EABT6491	1	5	7	25	3	3	1
EABT6492	0	0	4	1	0	0	1
EABT6493	0	2	2	1	0	0	3
EABT6494	0	5	14	1	0	0	0
EABT6495	0	2	6	9	8	0	0
EABT6496	3	3	13	8	1	1	1
EABT6497	1798.98	4048.98	2127.73	1828.63	2177.8	59	70

EABT6498	33	16	4	1	4	26.65	2
EABT6499	1	2	0	0	3	1	1
EABT65	0	2	3	2	0	0	5
EABT650	0	0	2	5	3	0	0
EABT6500	1	1	13	3	0	0	0
EABT6501	1	0	3	5	0	0	1
EABT6502	0	0	3	0	0	0	0
EABT6503	0	2	13	0	0	0	0
EABT6504	7	26.08	44	8	0	9	0
EABT6505	1	1	8	0	2	1	0
EABT6506	0	4	3	0	0	1	0
EABT6507	0	1	6	1	0	0	0
EABT6508	3	2	25	5	4	0	0
EABT6509	0	1	1	0	1	1	2
EABT651	0	6	4	0	0	1	1
EABT6510	0	0	0	2	12	0	0
EABT6511	0	2	1	7	2	1	0
EABT6512	3	1	6	15	6	0	1
EABT6513	0	1	0	0	0	0	5
EABT6514	0	4	3	3	1	0	0
EABT6515	12	28	66	180	93	46	112
EABT6516	4	7	2	1	0	0	0
EABT6517	0	14	0	0	2	0	13
EABT6518	8199.6	7938.83	1855.47	787	3772.11	815	3814
EABT6519	0	2	1	0	1	2	5
EABT652	1	1	16	1	3	2	3
EABT6520	5	3	7	3	1	9	3
EABT6521	3	2	10	5	0	1	1
EABT6522	0	2	3	1	0	1	1
EABT6523	8	20	17	77	17	1	38
EABT6524	1	5	1	2	1	1	19
EABT6525	1	5	2	6	0	0	1
EABT6526	97	98.02	659	1417.85	386.32	32.91	155.78
EABT6527	1	6	9	6	1	12	1
EABT6528	53.35	92	76.05	266	1364.75	4	150.91
EABT6529	2	7	3	2	3	2	2
EABT653	2	2	11	19	0	0	0
EABT6530	2281	317	414	5	1391.88	97	2145.87
EABT6531	0	2	0	11	1	0	0
EABT6532	0	0	5	1	0	0	0
EABT6533	0	0	7	0	0	0	1
EABT6534	0	0	5	1	0	0	0
EABT6535	74	119	122.7	19	10	28	36.47
EABT6536	34	59.1	51	201.01	54	8.31	1
EABT6537	0	0	2	3	1	1	0
EABT6538	0	0	23	0	0	0	1
EABT6539	5	2	0	0	0	5	2
EABT654	0	0	3	4	0	0	0
EABT6540	646.64	1155.42	2545.39	2554.05	964.96	512.88	644.99
EABT6541	0	0	17	0	0	0	0
EABT6542	1	0	10	0	0	0	0

EABT6543	0	0	3	0	2	0	4
EABT6544	2	6	2	1	0	13	0
EABT6545	1	5	0	0	0	2	3
EABT6546	0	0	0	4	3	0	0
EABT6547	1	1	3	0	0	0	1
EABT6548	5	18	6	83.09	9	0	12
EABT6549	0	1	33	0	0	0	0
EABT655	751.37	739.05	221.85	595.7	943.35	527.66	577.18
EABT6550	0	0	0	0	1	8	6
EABT6551	0	1	13	1	0	0	0
EABT6552	4	1	22	3	1	1	1
EABT6553	2757.73	7626.42	7601.02	11913.7	5458.64	2648.11	5753.08
EABT6554	12	12	20	16.86	31	7	7
EABT6555	318	363	266.79	681	344	223	194
EABT6556	482	1047.08	1384.02	2270.65	454	292.95	361.13
EABT6557	1	12	55	9	1	0	0
EABT6558	0	1	6	0	0	0	0
EABT6559	1	3	4	0	1	0	0
EABT656	1	0	1	0	4	0	0
EABT6560	0	1	1	3	0	0	0
EABT6561	1	10	7	0	0	13	9
EABT6562	0	0	7	1	0	0	0
EABT6563	12	17	9	1	0	24	0
EABT6564	0	0	3	0	0	2	0
EABT6565	271	348.38	143	548	345	130	237
EABT6566	6	10	31	2	0	4	4
EABT6567	3	2	2	2	0	0	0
EABT6568	7	2	0	3	0	1	2
EABT6569	3885	2494.29	954	56	2630.6	391	913.31
EABT657	7386.26	16346.1	35428	10849.9	7732.1	8084.04	12886.4
EABT6570	1	12	20	1	2	0	2
EABT6571	0	1	1	1	0	0	0
EABT6572	1327.01	1659	3294.62	4591.98	1071	967	738.01
EABT6573	0	2	9	0	0	0	0
EABT6574	1	7	1	2	47	0	2
EABT6575	77	33	5	0	1	0	9
EABT6576	5	4	0	0	0	1	0
EABT6577	0	0	0	0	0	0	0
EABT6578	0	1	0	35	0	0	0
EABT6579	775.92	871.51	731.58	1451.49	623.07	850.89	401.34
EABT658	0	1	2	7	3	0	0
EABT6580	0	0	6	3	8	0	9
EABT6581	3	2	1	1	0	0	0
EABT6582	546.25	1775.83	6080.82	5488.9	164974	173	1889.87
EABT6583	115.02	119	141.17	136.17	64	87	43.07
EABT6584	1311.64	2282.26	2289.9	4908.48	2229.7	1230	1486.12
EABT6585	1	1	0	3	2	0	0
EABT6586	17	45	20	13	10	8	14
EABT6587	0	1	9	1	0	0	0
EABT6588	3	3	10	6	2	1	15
EABT6589	0	0	3	2	0	0	0

EABT659	0	0	0	0	0	0	0
EABT6590	0	5	9	0	0	8	1
EABT6591	3408.7	3253	2283.98	4496.28	1742.96	3174	2581.6
EABT6592	19	67	209	2663.16	1179.39	23	69.29
EABT6593	0	7	7	6	0	0	0
EABT6594	1868.86	4375.59	2833.64	2584.19	3125.69	524.99	2343.15
EABT6595	47	14	65	101	23	8	15
EABT6596	2	7	1	3	1	12	0
EABT6597	0	0	14	0	0	0	0
EABT6598	2	1	2	5	4	1	2
EABT6599	3	5	0	0	4	13	47
EABT66	40	95	89	964	643	25	13
EABT660	0	0	7	1	0	0	0
EABT6600	2	2	3	1	1	0	1
EABT6601	0	0	2	0	0	0	0
EABT6602	0	1	5	0	0	0	0
EABT6603	0	0	2.1	13	0	0	0
EABT6604	1	1	1	0	1	0	0
EABT6605	2	1	8	0	0	0	2
EABT6606	6	22	41	12	20	13	30
EABT6607	0	0	7	0	1	0	1
EABT6608	0	0	6	0	0	0	0
EABT6609	0	1	3	3	0	0	0
EABT661	22616.2	26959.6	16879.7	16403.2	16582.6	31160.7	57024.1
EABT6610	0	2	45	3	0	0	2
EABT6611	5449.58	7643	8141.43	10411.1	4303.97	5686.6	3120.39
EABT6612	3354.01	3372.5	2536.12	2894.08	2147.56	1365.02	1441.47
EABT6613	1021.17	1508.92	1655.35	1190.12	1463.49	1048.72	1519.66
EABT6614	0	0	6	2	0	0	0
EABT6615	0	3	2	0	3	0	0
EABT6616	2707.73	3230.01	1447.14	2612.44	1229.68	2286.9	1701.89
EABT6617	0	0	4	2	0	0	2
EABT6618	5	16	46.9	46	4	4	6
EABT6619	0	0	5	2	0	0	0
EABT662	1	3	240.04	4	3	0	0
EABT6620	5	8	104	10	3	1	4
EABT6621	0	0	2	1	0	0	0
EABT6622	1	27	467	35	5	0	0
EABT6623	8	11	6	10	7	3	9
EABT6624	1638	2191	2931.5	3585.05	1603.94	1726.03	2668.07
EABT6625	0	1	5	0	1	0	1
EABT6626	2	3	9	2	0	1	2
EABT6627	3	8	31	1	9	4	3
EABT6628	0	0	1	1	0	0	0
EABT6629	1	0	18	0	3	3	0
EABT663	0	0	1	1	5	1	1
EABT6630	1	2	4	0	3	0	0
EABT6631	16	36	24	64	19	12	17
EABT6632	42	102.95	35.02	1.1	39	4	1
EABT6633	11	10	47	34	136	5	1
EABT6634	5	1	23	5	0	0	1

EABT6635	356	543	550	842.57	349.79	293	428
EABT6636	5	4	4	6	2	2	4
EABT6637	0	0	8	2	1	2	1
EABT6638	1	1	1	1	0	1	0
EABT6639	0	0	13	0	0	0	0
EABT664	1	6	0	0	0	27	1
EABT6640	3593.61	6106.87	15931.4	9594.24	5082.32	2866.49	3020.55
EABT6641	6	4	7	16	1	0	2
EABT6642	3	1	12	0	4	0	0
EABT6643	0	0	48.2	0	0	0	0
EABT6644	1	3	11	1	0	0	0
EABT6645	1220	2400.89	987.99	3961	1449	50	128
EABT6646	0	1	2	1	0	1	0
EABT6647	0	0	0	2	0	1	0
EABT6648	0	0	19	0	0	1	0
EABT6649	0	0	4	0	1	3	2
EABT665	0	0	10	1	0	0	0
EABT6650	386.78	696.09	161	1290.91	486.01	314.81	365
EABT6651	1	1	11	0	0	0	0
EABT6652	0	0	0	0	1	0	0
EABT6653	0	0	11	2	0	0	2
EABT6654	123	167	87	242	119	72	69
EABT6655	0	9	6	71	199	1	19
EABT6656	0	1	0	0	0	0	0
EABT6657	0	0	5	0	0	0	0
EABT6658	1958.88	2358.06	1989.36	3124.36	1763.25	1534	1255.87
EABT6659	380.04	641.31	1268.35	803.64	327.82	611.15	466.37
EABT666	1	16	9	0	6	0	1
EABT6660	1	2	34	0	2	3	8
EABT6661	1	1	5	0	1	0	0
EABT6662	6	15	0	0	0	1	1
EABT6663	0	2	0	0	1	0	1
EABT6664	0	0	2	7	18	0	1
EABT6665	2	3	17	1	1	6	9
EABT6666	6	4	5	7	0	0	0
EABT6667	0	2	2	1	0	0	1
EABT6668	2	1	11	0	0	0	3
EABT6669	4	16	35	29	4	4	4
EABT667	1	0	8	0	0	2	0
EABT6670	1385	1050	1022	2299.98	223	31	114.93
EABT6671	135	133	1811.04	2996.62	68	185	62
EABT6672	236	452	705	769.06	789	462	479.71
EABT6673	1	1	30	1	2	4	1
EABT6674	2	0	0	0	0	14	3
EABT6675	135.56	76.43	357.13	1264.06	88	2	5
EABT6676	1	0	8	2	0	1	0
EABT6677	0	0	8	0	0	0	0
EABT6678	1	1	6	11	2	1	1
EABT6679	1	0	2	2	0	0	0
EABT668	4344.71	4019.96	3172.34	3240.84	5369.47	1120.98	1326.1
EABT6680	0	0	9	0	0	0	0

EABT6681	2	13	2	8	4	2	4
EABT6682	5.91	60.03	129	15	9	1	0
EABT6683	62.61	132.88	131	399.75	209.88	15	95.04
EABT6684	0	0	3	7	0	1	3
EABT6685	0	1	12	0	1	0	0
EABT6686	353	581.28	571	835	504.24	400	371
EABT6687	0	1	4	1	0	0	0
EABT6688	1	3	7	0	0	3	0
EABT6689	1	1	3	1	0	1	0
EABT669	1	1	0	0	1	1	0
EABT6690	3	8	20	14	2	2	2
EABT6691	0	2	11	0	0	0	1
EABT6692	2	5	10	4	10	0	4
EABT6693	0	2	5	2	1	0	0
EABT6694	896.16	1059.24	605.66	645.31	647.99	235.04	240
EABT6695	2	2	0	1	1	1	4
EABT6696	32	35	25	58	16	7	18
EABT6697	0	0	4	1	0	0	0
EABT6698	0	6	11	9	0	0	0
EABT6699	2	32	92	208	76	0	2
EABT67	6612	8340	11848	15776.4	7446.84	7835.92	5764.06
EABT670	0	0	4	0	1	0	0
EABT6700	1	0	4	6	1	0	5
EABT6701	0	2	1	3	2	1	0
EABT6702	0	0	14	0	1	0	0
EABT6703	1378.58	1825.44	2019.13	2671.94	1777.42	1209.17	934.25
EABT6704	334	1691.5	307	3736.43	276.01	72	293.85
EABT6705	2	0	12	5	0	3	2
EABT6706	4	2	19	0	0	4	0
EABT6707	0	0	6	4	0	0	0
EABT6708	37	109.27	91	48	112	30	105
EABT6709	0	1	2	2	1	0	0
EABT671	0	0	0	0	5	0	0
EABT6710	0	0	2	1	0	1	0
EABT6711	0	0	44	2	1	0	6
EABT6712	0	3	1	6	0	0	2
EABT6713	0	1	1	5	0	0	1
EABT6714	0	1	0	0	0	0	1
EABT6715	2652.36	2577.87	4615.83	4713.02	1985.52	1504.65	1199.85
EABT6716	0	6	2	0	0	3	3
EABT6717	0	0	9	2	1	0	0
EABT6718	3	5	14	1	0	2	1
EABT6719	1	2	3	4	0	0	0
EABT672	2	1	0	0	0	0	0
EABT6720	1	1	4	8	1	0	0
EABT6721	1	7	10	16	1	4	2
EABT6722	0	2	3	2	0	0	0
EABT6723	2.56	1	0	0	0	6	0
EABT6724	5	5	5	0	1	2	8
EABT6725	0	2	9	2	0	0	0
EABT6726	0	0	5	0	2	0	0

EABT6727	0	0	0	1	1	0	0
EABT6728	3	0	11	16	3	4	4
EABT6729	0	1	1	4	0	0	0
EABT673	0	0	26	1	0	2	2
EABT6730	11	40	55	59	4	11	21
EABT6731	347.88	556	18	61	199.99	3	20
EABT6732	2	3	43	2	0	1	0
EABT6733	0	3.59	19	0	0	0	0
EABT6734	1	2	7	5	1	1	19
EABT6735	205	299	156	551	243	71	174
EABT6736	0	0	8.97	2	0	0	0
EABT6737	1	4	2	0	1	4	1
EABT6738	0	3	3	6	0	4	6
EABT6739	2	97	35	147	102	8	4
EABT674	0	0	10	0	0	0	0
EABT6740	1	5	1	0	0	11	0
EABT6741	0	2	4	0	1	0	0
EABT6742	1	2	13	1	0	1	0
EABT6743	1	1	3	32	7	0	4
EABT6744	1176.28	2634.15	306	40	372	569.97	392.75
EABT6745	0	0	4	2	0	0	0
EABT6746	0	0	6	1	0	2	0
EABT6747	0	0	12	1	1	0	0
EABT6748	1	0	10	1	0	1	0
EABT6749	0	1	4	3	0	1	0
EABT675	1	5	3	10	2	0	0
EABT6750	1	1	1	4	0	0	0
EABT6751	20	18	2	16	1	0	0
EABT6752	4	3	2	8	7	0	9
EABT6753	6	0	8	4	4	1	1
EABT6754	5	23.12	21	41	7	5	5
EABT6755	10	17	5	14.89	10	5	9
EABT6756	693.45	1840.13	2162.07	3704.51	659.81	16	61
EABT6757	454.12	823	204.25	439	269	384	240
EABT6758	1	0	13	1	0	0	1
EABT6759	2	0	3	0	0	0	0
EABT676	1	4	89	3	0	0	0
EABT6760	21	128.97	54.07	54	13	69	107
EABT6761	0	5	3	1	0	0	0
EABT6762	2	1	4	5	0	0	0
EABT6763	2	3	0	1	1	1	0
EABT6764	0	0	4	0	0	0	0
EABT6765	0	0	4	0	0	0	0
EABT6766	1	0	0	3	2	0	7
EABT6767	1	0	48	3	1	0	1
EABT6768	0	0	0	0	0	0	0
EABT6769	0	7	2	0	0	1	4
EABT677	0	0	16	1	1	0	0
EABT6770	0	0	1	0	1	5	1
EABT6771	135	143.14	200.14	432.16	294.69	188.01	107
EABT6772	0	1	0	3	0	0	0

EABT6773	14	68.19	301.98	45	5	13	33
EABT6774	0	0	5	0	0	0	0
EABT6775	0	0	0	0	0	3	0
EABT6776	7	18	20	10.02	10	13	21
EABT6777	0	3	23	4	13	0	0
EABT6778	213	303.28	21	182.68	2	0	0
EABT6779	12702	6420.96	23860.4	25529.2	8465.2	6416.38	7616.5
EABT678	0	0	0	8	0	0	0
EABT6780	2	9	4	1	0	17	1
EABT6781	0	1	11	0	0	1	0
EABT6782	1	3	20	0	1	4	1
EABT6783	3	17	39	22	5	1	9
EABT6784	5101.91	16580.7	89419.1	6627.94	12384.3	3914.82	5269.97
EABT6785	2428.66	5933.8	5428.71	14313.1	4277.05	1047.02	2407.04
EABT6786	140.11	318	445.01	5454.12	98	64.99	95
EABT6787	1927.85	2760.59	2308.52	4105.1	1903.29	1252.73	1697.87
EABT6788	0	0	6	1	0	0	0
EABT6789	1376.66	2675.19	4035.79	6485.67	3608.72	622.56	1159.52
EABT679	0	3	2	11	0	0	0
EABT6790	1186.19	2102.04	1478.63	1925.29	1505.68	1330	1686.03
EABT6791	1	1	155.02	15	0	3	0
EABT6792	73.01	60	14	117.63	30	48	21
EABT6793	1	3	58	5	2	4	1
EABT6794	1	0	9	0	0	0	0
EABT6795	1	6	5	1	1	2	1
EABT6796	204	754.7	1386.89	2241	673.98	129	307.84
EABT6797	1	4	14	3	0	3	1
EABT6798	0	1	3	4	0	0	0
EABT6799	0	3	2	0	0	0	0
EABT68	1.03	9	29.46	7.71	2	4	5
EABT680	0	1	1	5	1	0	0
EABT6800	19624.5	30148.1	25002.5	51846.9	26139.9	24019.7	26954.3
EABT6801	2	6	0	0	0	2	1
EABT6802	1	0	6	0	0	2	0
EABT6803	1	7	3	3	0	0	2
EABT6804	2	6	3	9	0	0	2
EABT6805	1	0	25	3	0	0	0
EABT6806	6	28	7	123.39	4	0	1
EABT6807	0	0	7	0	0	0	0
EABT6808	3	3	9	8	3	0	0
EABT6809	5	9	19	6	3	4	5
EABT681	5	17	34.99	37	5	4	7
EABT6810	1	1	18	0	0	0	0
EABT6811	0	0	13	8	1	0	3
EABT6812	0	5	8	8	24	0	3
EABT6813	0	0	0	0	0	0	0
EABT6814	115	680	328	204	318	186	508.01
EABT6815	4	17	19	5	3	0	0
EABT6816	1871	2577.64	1843	2919.15	1324.99	1813	1988.1
EABT6817	2	1	10	6	2	1	3
EABT6818	1785	3783.08	3079.09	2303.94	3524.5	3370.97	7848.38

EABT6819	1	8	13	2	1	0	7
EABT682	1448.96	3121.66	26	7	172.78	3685.66	1145.03
EABT6820	0	2	32	6	12	4	6
EABT6821	10947.7	9809.34	3612.82	3776.41	8220.36	5319.46	6934.95
EABT6822	1	14	16	9	3	0	0
EABT6823	1	1	1	2	0	0	1
EABT6824	0	0	1	1	2	0	7
EABT6825	5	38	128	15	7	9	8
EABT6826	0	3	1	0	0	1	1
EABT6827	6	21	20	10	3	1	2
EABT6828	1	3	9	5	1	1	4
EABT6829	758	1392.56	468.69	219.23	527.9	32	119
EABT683	0	1	22	0	0	0	1
EABT6830	0	1	1	0	0	1	0
EABT6831	4.27	67	0	0	0	27	0
EABT6832	20	29	245	1	3	0	0
EABT6833	8	9	0	0	0	0	0
EABT6834	0	1	4	0	0	1	1
EABT6835	0	5	3	20	1	0	0
EABT6836	3240.96	5229.24	2481.5	2588.11	12156.2	2777.28	2982.87
EABT6837	0	0	3	2	1	2	0
EABT6838	1	5	24	3	2	0	2
EABT6839	11	2	5	0	0	26	0
EABT684	34666.6	33780.8	7615.72	9449.51	8131.27	25273.9	12197.4
EABT6840	3	23	62	50	39	0	3
EABT6841	0	9	10	8	0	1	0
EABT6842	0	0	18	0	0	0	0
EABT6843	0	0	0	0	0	0	1
EABT6844	1	2	4	3	1	0	0
EABT6845	100	359.62	1017	251	245	66	135
EABT6846	0	2	5	1	1	0	3
EABT6847	0	0	15	0	1	0	0
EABT6848	1	1	6	0	0	0	0
EABT6849	1	4	10	2	0	0	0
EABT685	3346	6312.88	5102.13	13664.3	4028.04	1993.98	2539.03
EABT6850	11141.1	12431.6	8708.14	8789.74	9438.16	10557.4	10824.6
EABT6851	2	0	5	3	2	0	0
EABT6852	4780.04	8577.76	11970.9	13342	5044.45	4944.21	5089.18
EABT6853	0	3.87	3	0	1	0	0
EABT6854	2	1	0	1	0	1	0
EABT6855	46	215	340	477	64	66	50
EABT6856	0	0	0	0	0	0	0
EABT6857	0	15	1	0	0	83.22	0
EABT6858	0	1	3	1	11	0	0
EABT6859	0	0	10	0	0	1	1
EABT686	0	1	11	6	0	2	2
EABT6860	0	2	4	0	0	0	1
EABT6861	0	0	3	1	2	2	0
EABT6862	3	0	10	8	1	2	0
EABT6863	1	6	2	1	2	0	1
EABT6864	0	2	4	4	1	0	1

EABT6865	3	4	9	13	0	2	4
EABT6866	5	0	1	0	1	1	2
EABT6867	0	1	8	1	0	0	0
EABT6868	2	6	0	1	3	0	1
EABT6869	1	4	4	27	2	0	0
EABT687	0	0	0	4	0	0	0
EABT6870	2	33	9	140.11	13	1	2
EABT6871	0	2	4	2	3	1	5
EABT6872	1	2	0	9	2	0	0
EABT6873	126	175	158.02	11	272	125	276
EABT6874	0	2	4	2	0	0	0
EABT6875	0	1	16	3	0	3	1
EABT6876	9	1	0	2	3	16	3
EABT6877	12	13	0	0	0	0	1
EABT6878	1	0	24.98	0	0	2	0
EABT6879	1	1	13	2	1	0	1
EABT688	0	2	19	0	0	6	1
EABT6880	2745.65	4156.35	2532.15	4263.3	10220.4	1872.86	2135.18
EABT6881	1	3	13	18	3	5	6
EABT6882	0	1	2	2	2	0	0
EABT6883	2	4	19	15.41	2.51	0	1
EABT6884	0	0	1	1	2	0	1
EABT6885	3	6	10	3	2	0	0
EABT6886	0	1	54	1	0	1	0
EABT6887	1	4	18	1	2	0	0
EABT6888	0	1	2	0	2	0	0
EABT6889	1	2	5	8	0	1	0
EABT689	1	0	52	4	1	0	3
EABT6890	0	1	2	3	0	1	0
EABT6891	1982.65	3341.07	2769.74	20759.9	3580.92	649.31	690.03
EABT6892	1040	1685.96	754	1679.85	678.92	928	943.97
EABT6893	0	1	1	5	1	0	1
EABT6894	2	8	18	1	0	1	3
EABT6895	2	3	0	2	1	0	0
EABT6896	3	0	2	6	0	0	0
EABT6897	1	1	1	21	0	0	0
EABT6898	41.03	62	121.67	28	13	4	10
EABT6899	2741.15	3918.9	1505	1744.99	1859.59	2456.14	4884.53
EABT69	0	0	0	5	0	0	0
EABT690	3	1	10	14	1	0	1
EABT6900	0	0	9	1	0	1	0
EABT6901	1	0	3	0	0	0	0
EABT6902	0	0	0	0	1	1	3
EABT6903	0	1	2	2	0	4.05	1
EABT6904	87	90	32	153.06	58	31	102
EABT6905	0	1	1	6	1	0	0
EABT6906	0	1	14	4	0	1	0
EABT6907	2	6	2	1	0	0	0
EABT6908	1221.53	2265.65	2341.45	3335.27	1278.99	1504.95	1181.56
EABT6909	0	2	9	2	0	0	0
EABT691	1	2	16	0	0	0	1

EABT6910	1037.07	1065.43	855.18	1496.06	1016.35	486.02	546
EABT6911	2	5	8	10	1	4	0
EABT6912	194.03	311.19	591	605.21	199	235.41	168
EABT6913	0	0	6	0	0	0	0
EABT6914	0	0	14	5	7	0	3
EABT6915	1	0	8	2	0	1	1
EABT6916	1	3	0	0	0	1	2
EABT6917	3	6	16	0	1	2	1
EABT6918	1	1	11	5	0	0	2
EABT6919	1	3	5	4	0	0	1
EABT692	1337.58	1894.72	1588.5	2232.77	2043.69	984.46	1385.48
EABT6920	1	3	32	6	0	1	2
EABT6921	2.9	4	14	2	1	0	3
EABT6922	2786.3	5193.48	1139.48	255.68	1504.95	7333.72	8201.66
EABT6923	327	501.07	814.14	771.79	441.97	503	444.67
EABT6924	4	13	1	21	2	0	0
EABT6925	0	1	17.63	9	2	0	0
EABT6926	0	2	7	14	0	0	0
EABT6927	0	0	1	0	0	0	0
EABT6928	0	4	0	1	0	1	2
EABT6929	4	5	3	1	0	0	4
EABT693	0	2	5	19	0	1	0
EABT6930	2	2	14	1	0	0	0
EABT6931	2	2	3	0	0	1	1
EABT6932	0	0	1	1	1	0	1
EABT6933	0	0	3	2	0	0	1
EABT6934	5	16	23	22	7	10	9
EABT6935	10	8	41	10	6	8	6
EABT6936	0	2	3.17	33	4	0	0
EABT6937	7	12	66	6	15	0	15
EABT6938	196	331	391.03	529	364	124	142
EABT6939	1	1	4	6	1	0	3
EABT694	22	54	197	6	22	2	2
EABT6940	0	0	2	2	0	0	0
EABT6941	1980.92	3637.09	2408	4079.06	2848.21	1570.53	2246.82
EABT6942	205.01	596	1651.01	63949.9	266.78	220.99	859.64
EABT6943	173	410	1295.97	44179.5	208	179	357.87
EABT6944	857	1887.01	2706.58	5463.67	1059	59.29	167
EABT6945	3	6	1	1	2	1	2
EABT6946	0	0	1	159	0	2	0
EABT6947	5651.07	4416.27	57	6	0	0	0
EABT6948	136	213.57	33.77	30.02	66	48	101
EABT6949	3	1	63	2	0	1	0
EABT695	266.42	494.89	1529.05	1827.84	369.89	27.99	61
EABT6950	4	2	7	0	0	0	0
EABT6951	1	1	10	0	0	0	0
EABT6952	7	26	0	6	0	2	0
EABT6953	10	3	0	0	0	8	0
EABT6954	1	3	11	7	0	0	0
EABT6955	0	1	0	0	1	1	0
EABT6956	0	1	4	0	0	0	0

EABT6957	2	1	1	0	1	1	0
EABT6958	2	0	4	0	0	0	0
EABT6959	1	7	2	21	1	0	1
EABT696	0	8	0	0	0	0	0
EABT6960	0	0	6	4	0	0	0
EABT6961	4	8	3	13	3	1	5
EABT6962	8	12	48	7	2	6	4
EABT6963	211.03	304.19	517	68.37	430.02	361.18	67
EABT6964	6	9	4	17	11	1	3
EABT6965	6336.11	16672.9	14840.4	3133.18	13381.5	344.99	3658.25
EABT6966	9130.86	9110.47	7486.2	7027.95	10970.1	10642	11909.9
EABT6967	1.02	0	5	1	0	9	3
EABT6968	4	34.97	97	13	23.04	15	29
EABT6969	1586.88	381.02	11	21	1269.07	114.93	2
EABT697	755.94	1022.84	1191.72	1207.7	1556.57	346.99	731.01
EABT6970	5	11	12	6	6	6	3
EABT6971	0	3	3	1	0	0	0
EABT6972	1120.61	2060.01	2216.43	608.49	1378.93	879.87	1029.08
EABT6973	2505.15	4614.86	5862.22	3597.92	2729.67	384.5	549.16
EABT6974	9	86	221	31	12	0	5
EABT6975	1	0	1	0	3	0	0
EABT6976	0	1	267.22	1	9	1	4
EABT6977	2024	1628.61	368.8	946.01	5481.97	1292	986.89
EABT6978	0	2	4	3	0	0	1
EABT6979	1	1	13	9	2	1	0
EABT698	5042.75	11532.6	2068.01	3972.63	3254.54	3870.02	3115.67
EABT6980	3245.94	5559.75	2117.86	2689.27	1844	2926.58	2164.52
EABT6981	0	1	15	2	0	1	0
EABT6982	8	19	21	5	9	10	22
EABT6983	5323	9807.55	7856.2	10613.9	3026.36	4584.71	3546.72
EABT6984	0	9	17	0	0	4	0
EABT6985	0	2	20	1	0	0	0
EABT6986	2729.33	3297.98	1968.1	4143.21	1471.96	1779.06	2182.18
EABT6987	2	5	0	1	3	0	0
EABT6988	10	12	0	0	0	9	12
EABT6989	2	5	9	9	4	1	4
EABT699	12	6	0	0	0	4	9
EABT6990	1	3	6	4	0	1	1
EABT6991	0	0	0	3	1	0	3
EABT6992	12353	30957.4	7127.53	3908.77	7730.25	16878.7	24357.2
EABT6993	1	3	5	4	21	0	0
EABT6994	2023	3848.76	5865.23	8729.91	2657.46	1958.49	2706.4
EABT6995	0	2	7	2	0	1	1
EABT6996	2	0	19	6	58	2	2
EABT6997	1081.01	1291.65	1440.23	1875.06	1042.69	695.06	599.04
EABT6998	1	5	9	1	0	0	0
EABT6999	3	2	0	0	1	0	2
EABT7	0	6	0	2	0	0	0
EABT70	0	2	9	3	4	1	4
EABT700	1	3	4	1	7	0	0
EABT7000	200.66	350	218	663.87	467.82	202	131

EABT7001	7	14	2	1	0	0	0
EABT7002	0	3	0	7	0	0	0
EABT7003	1	0	4	0	0	0	1
EABT7004	2	4.57	1	1	0	0	1
EABT7005	52	29	16	91	10	21	15
EABT7006	0	0	1	3	0	0	2
EABT7007	0	4	2	3	1	1	3
EABT7008	503	1853	490.58	376	103	208.18	424
EABT7009	0	15	4	0	0	0	0
EABT701	6	12	17	20	51	15	12
EABT7010	2416.9	1752.7	214	385	177	28	6
EABT7011	0	0	1	4	0	1	0
EABT7012	0	0	9	0	0	0	0
EABT7013	1	3	0	0	0	15	4
EABT7014	0	0	6	3	0	0	0
EABT7015	0	0	10	0	0	1	0
EABT7016	0	1	0	1	2	0	3
EABT7017	0	0	4	0	0	0	0
EABT7018	2470.47	2476.33	1504.68	5453.52	2780.34	2547.05	3896.04
EABT7019	0	4	4	16	10	1	1
EABT702	2	2	10	0	0	1	0
EABT7020	4316.99	10675.2	4224.4	3034.73	4336.56	2871.04	2901.7
EABT7021	0	4	2	1	0	3	1
EABT7022	1415.11	1882	2574.34	5550.47	1662.74	2470.33	1583.52
EABT7023	3	5	2	1	2	0	0
EABT7024	1	1	3	3	0	0	0
EABT7025	349.74	218.09	78	257.84	97.99	177.01	89
EABT7026	2	1	6	0	0	1	0
EABT7027	0	2	4.01	4	3	1	0
EABT7028	3998.96	6541.73	8973.74	9992.15	3801.58	3635.69	3643.93
EABT7029	0	2	1	6	0	0	0
EABT703	1	3	7	7	0	1	1
EABT7030	76	36	2	0	10	6	26.3
EABT7031	0	0	4.01	0	0	0	0
EABT7032	0	0	6	3	1	1	0
EABT7033	1	0	5	0	0	0	0
EABT7034	2	1	10	2	0	1	2
EABT7035	1	5	14	1	0	2	4
EABT7036	1	1	0	6	1	1	0
EABT7037	1997.92	1684.01	375	617.07	2669.02	1156	748.93
EABT7038	0	3	3	2	2	0	1
EABT7039	1	5	3	0	0	5	3
EABT704	2	10	11	30	5	0	5
EABT7040	0	3	25	0	1	2	0
EABT7041	0	0	14	2	0	0	0
EABT7042	2	1	6	0	0	0	0
EABT7043	1	1	16	1	0	0	0
EABT7044	2	4	23	20	49	6	18
EABT7045	5	13	13	3	1	2	4
EABT7046	5	0	9	2	0	1	0
EABT7047	0	0	2	1	5252.32	3	16

EABT7048	0	0	6	0	0	0	0
EABT7049	6165.31	5523.72	3546.57	3285.55	2929.66	6744.73	5987.54
EABT705	50	148	156.92	488	163	47	31.28
EABT7050	0	0	0	2	3	0	0
EABT7051	27	9	13	85	1	0	0
EABT7052	0	0	5	1	0	0	0
EABT7053	0	0	1	0	1	0	0
EABT7054	2715.04	5640	4977.83	3415.4	2889.41	878.98	1687.79
EABT7055	71	89	26	151.56	72	31	60
EABT7056	0	3	1	2	0	0	0
EABT7057	0	0	0	0	1	0	2
EABT7058	6	6	3	2	0	0	1
EABT7059	5	0	0	0	0	2	0
EABT706	216	278.07	1360.4	41.98	3	11	16
EABT7060	0	1	0	6	6	0	1
EABT7061	0	1	3	4	6	0	0
EABT7062	1932.25	4586.19	3004.81	1924.68	343.17	1944.11	278.82
EABT7063	35	52	41	71	30	13	23
EABT7064	13	33.04	61	4	1	8	9
EABT7065	1	6	0	7	1	1	1
EABT7066	2	2	13	5	1	3	3
EABT7067	13	5	5	1	4	1	44
EABT7068	1	2	24	0	2	1	2
EABT7069	7778.48	15221.8	7770.27	18523.1	10981.1	5276.79	10226.8
EABT707	12	9	29	5	43	5	60
EABT7070	0	1	1	0	1	0	0
EABT7071	1	1	3	1	0	0	0
EABT7072	0	12	42	1	0	0	0
EABT7073	20	44	2	5	0	28	7
EABT7074	61	0	0	0	0	39	0
EABT7075	0	0	13	2	0	0	0
EABT7076	0	3	11	4	0	3	0
EABT7077	374	918.01	739.58	4471.52	524	401	503
EABT7078	6	11	189	11	1	12	11
EABT7079	2	4	1	8	0	3	0
EABT708	10367.5	11136	478	237	1219.21	10875.3	1774.62
EABT7080	90	104.6	69	60	62.3	22	61
EABT7081	1	6	79	219.98	35.08	9	6
EABT7082	3	1	0	1	28	0	0
EABT7083	1	0	16	1	0	0	0
EABT7084	7096.43	8493.95	11142.5	43935.8	5448.98	1112.21	2165
EABT7085	0	0	1	0	0	0	1
EABT7086	6	0	8	0	0	0	3
EABT7087	1	2	4	2	0	7	6
EABT7088	5	24	0	4	5	0	0
EABT7089	11	18	11	31	65	8	21
EABT709	0	1	3	1	0	0	0
EABT7090	11	18	7	1	0	2	0
EABT7091	15	12	19	66	3	7	6
EABT7092	5	27	36	27	1	3	3
EABT7093	26	16	36	48	67	15	16.24

EABT7094	0	1	10	0	1	0	0
EABT7095	1	0	12	0	0	0	0
EABT7096	0	4	9	0	0	0	0
EABT7097	2	1	1	2	1	0	0
EABT7098	0	1	12.71	0	1	0	2
EABT7099	0	0	0	0	0	0	0
EABT71	1	2	2	0	0	2	1
EABT710	4	3	16	8	1	1	4
EABT7100	2	1	2	0	0	0	0
EABT7101	0	0	8	3	1	3	0
EABT7102	0	0	28	0	0	0	0
EABT7103	2	4	12	1	0	1	0
EABT7104	3033.66	5014.99	3775.81	9088.25	3392.68	2528.49	3247.83
EABT7105	7131.94	10391.5	11533.5	2832.92	2857.12	12979.2	5981.03
EABT7106	1659	1650.8	506	508	980.98	1491.97	766
EABT7107	0	13	3	14	0	1	0
EABT7108	0	0	2	0	4	0	0
EABT7109	0	3	6	15	1	1	1
EABT711	3	2	1	0	0	2	1
EABT7110	0	0	1	154	0	0	0
EABT7111	18	23	2	34	11	12	18
EABT7112	0	2	6	0	0	1	1
EABT7113	97	676	80	43	1248.46	28	92.01
EABT7114	1	3	0	2	0	0	0
EABT7115	175	442	1224.99	45	25	90	69
EABT7116	0	0	4	0	1	0	0
EABT7117	1	3	3	1	1	1	0
EABT7118	462.98	832.52	1282.49	292.76	557	322.13	551
EABT7119	37	40	105	96.96	22	8	24
EABT712	0	1	5	0	1	0	0
EABT7120	141	516.54	248	497.31	407.58	166.58	118.98
EABT7121	2	4	138.95	17	6	0	2
EABT7122	2	5	9	0	0	0	0
EABT7123	36	139	133.99	282	174	59	25
EABT7124	0	0	3	2	0	0	0
EABT7125	12	5	5	0	1	9	11
EABT7126	1	2	0	2.96	1	0	1
EABT7127	3	8	2	2	1	0	1
EABT7128	0	1	1	1	1	8	0
EABT7129	10	14	9	13	6	0	1
EABT713	2	3	17	0	1	7	0
EABT7130	15	15	0	0	3	13	19
EABT7131	1	1	8	0	0	0	0
EABT7132	56	16	0	0	0	69	12
EABT7133	10	15	8	0	2	3	9
EABT7134	0	3	12	1	0	0	0
EABT7135	0	4	27	1	0	0	1
EABT7136	0	0	10	0	0	0	0
EABT7137	10786.5	6483.76	220.97	241.03	5060.65	6067.41	15825
EABT7138	21	19	19	11	1	4	2
EABT7139	491	1523	35	66	62	561	468.99

EABT714	3	4	4	3	0	1	0
EABT7140	1	0	6	6	0	0	0
EABT7141	0	2	3	0	1	0	0
EABT7142	654	582	521.06	807.33	534.6	43	219
EABT7143	9081.28	10166.9	7824.32	6673.37	7666.16	6983.62	7557.49
EABT7144	3	0	11	3	0	0	0
EABT7145	0	0	0	8	7	0	0
EABT7146	2.03	5	18	0	18	4	19
EABT7147	1	3	12	2	0	1	0
EABT7148	0	1	3	1	0	1	0
EABT7149	0	0	23	6	1	1	0
EABT715	1	1	2	1	0	1	0
EABT7150	2	0	8	2	2	1	3
EABT7151	2	0	2	1	0	0	0
EABT7152	5	2	0	0	0	0	0
EABT7153	2	4	7	4	0	0	1
EABT7154	1	1	24	5	0	2	1
EABT7155	0	0	3	0	0	0	0
EABT7156	5	3	0	0	0	0	0
EABT7157	0	0	1	2	7	0	1
EABT7158	1	0	0	0	0	7	1
EABT7159	10	0	3.55	37	7	1	1
EABT716	0	0	0	0	2	0	18
EABT7160	1	0	9	0	0	0	0
EABT7161	1256.67	4103.23	2920.61	4942.78	4576.48	957.88	4547.85
EABT7162	4	2	10	14	3	3	4
EABT7163	4	3	1	5	1	1	4
EABT7164	0	2	0	3	0	0	0
EABT7165	0	0	8	0	0	0	0
EABT7166	5.01	12	7	1	2	0	7
EABT7167	2	6	0	0	2	0	0
EABT7168	0	1	10	8	0	0	1
EABT7169	0	1	8	1	1	0	0
EABT717	0	1	8	0	0	0	0
EABT7170	5	9	17	26	9	0	3
EABT7171	2324.76	4303.8	2273.61	4786.21	2098.12	1551.46	1692.88
EABT7172	634.39	2684.47	997.02	249	1528.32	214	910
EABT7173	1	0	5.16	0	0	0	0
EABT7174	0	2	13	1	2	0	0
EABT7175	995.99	1258.9	1129.6	3025.5	1143.29	415.23	418.08
EABT7176	231	396	464.88	765.74	601	134	245
EABT7177	0	0	5	0	0	1	0
EABT7178	3380.92	5017.02	6439.97	15152.9	3887.18	3052.09	3851.01
EABT7179	3	9	8	9	4	2	4
EABT718	1	1	3	3	0	0	1
EABT7180	0	0	10	0	0	0	0
EABT7181	1	11	281	4	0	2	0
EABT7182	0	6	3	0	130	0	2
EABT7183	0	5	0	1	0	0	0
EABT7184	613	584.98	228	541.91	343	416.05	280.41
EABT7185	0	1	10	2	0	0	0

EABT7186	0	0	12	1	0	0	0
EABT7187	597.97	777	706	1789	684	511	441
EABT7188	1	0	3	0	1	2	0
EABT7189	1	0	0	0	0	0	0
EABT719	1	14	1	0	3	5	11
EABT7190	0	0	5	0	0	0	0
EABT7191	0	1	9	0	1	2	0
EABT7192	196.99	262	311	230.1	43	151	84
EABT7193	0	0	5	0	0	0	0
EABT7194	4	14	75	5	0	2	0
EABT7195	3	2	2	11	2	0	0
EABT7196	28	59	15	123	14	39	25
EABT7197	41	316	135	173	107	25.87	53
EABT7198	75043.3	108445	59177.9	21125.2	75635.2	5078.52	24217.5
EABT7199	1	2	0	28	0	0	0
EABT72	4	2	1	2	3	0	1
EABT720	0	0	0	1	0	0	0
EABT7200	1	1	35	0	0	1	1
EABT7201	0	0	44.72	16	14	0	1
EABT7202	3	1	5	10	0	1	1
EABT7203	0	1	7	0	0	0	0
EABT7204	0	2	3	0	0	2	0
EABT7205	1	6	31	6	2	1	1
EABT7206	1	3	10	1	0	0	0
EABT7207	2	2	13	0	0	0	1
EABT7208	2	3	5	0	1	4	3
EABT7209	0	5	5	3	0	0	0
EABT721	2	8	58	0	2	16	0
EABT7210	1	1	5	1	0	0	1
EABT7211	794.98	2504.7	6761.68	10878.3	4409.69	333	2484.92
EABT7212	21	89	51	165	31	3	15
EABT7213	4783.78	8399.17	3901.25	7027.77	2047.05	3362.13	2085.74
EABT7214	18232.2	2421.14	14	0	1	15056.4	4
EABT7215	0	0	9	1	5	0	0
EABT7216	1388.96	1816.68	2030.71	4761.63	1529.72	803	1186.18
EABT7217	0	1	0	0	1	0	0
EABT7218	2	106	182	304.02	4	7	1
EABT7219	1	8	2	15	2	1	1
EABT722	4099.11	7064.27	6088.91	7179.37	4705.97	3234.37	4765.18
EABT7220	1	7	10	33	13	0	1
EABT7221	650.64	778.15	677.68	1330.43	760.99	352.04	353.27
EABT7222	0	1	6	0	3	0	3
EABT7223	0	3	2	2	0	0	1
EABT7224	0	8	44	6	0	1	1
EABT7225	1900.97	2464.82	3519	5980.36	1852.88	1776.08	1585.72
EABT7226	45	52	36	52	31	1	213.97
EABT7227	0	0	1	0	1	0	2
EABT7228	0	0	0	7	1	0	0
EABT7229	0	0	19	2	0	0	0
EABT723	11	11	19	10	9	16	5
EABT7230	0	1	0	0	3	0	0

EABT7231	0	0	11	2	1	1	0
EABT7232	3	18	34	60.9	130.11	5	1
EABT7233	1	5	1	2	3	0	0
EABT7234	0	2	11	7	2	2	2
EABT7235	735	791	464.06	951	246	258	138
EABT7236	3	0	6	1	3	0	0
EABT7237	0	0	21	0	0	0	0
EABT7238	159	742.69	133.86	491	639	222	1021.92
EABT7239	76	174	701.27	99.67	111	132.83	104
EABT724	5	3	1	0	3	0	1
EABT7240	1185.52	2320.53	1595.23	5084.94	2025	937	1426
EABT7241	2	2	11	6	1	2	2
EABT7242	1241.1	1350.48	864.2	640.05	403.29	686.21	1328.8
EABT7243	1	3	78	10	3	0	1
EABT7244	2	4	6	4	0	2	2
EABT7245	5	8	4	10	18	0	2
EABT7246	1	2	1	2	1	0	0
EABT7247	9	30.98	41	17	5	2	1
EABT7248	1	1	1	0	0	0	0
EABT7249	0	2	47	0	1	1	1
EABT725	2154.95	2411.07	2660.35	2820.21	1749	1600.16	1910.32
EABT7250	3	5	10	0	0	0	1
EABT7251	0	0	5	1	1	0	1
EABT7252	1	0	30	0	0	0	0
EABT7253	465.94	503	319	507.26	301	263.18	253
EABT7254	1	0	1	0	0	4	1
EABT7255	0	0	3	5	1	1	0
EABT7256	1	0	77	3	1	0	1
EABT7257	13.88	8	5	0	3	44	8
EABT7258	0	0	4	18	266.75	0	4
EABT7259	0	4	0	5	0	1	0
EABT726	296	487	1546.99	962.64	5578.39	12	24
EABT7260	0	0	0	1	1	0	2
EABT7261	12	0	0	0	0	8	0
EABT7262	1	2	9	11	3	1	1
EABT7263	0	1	0	2	2	0	0
EABT7264	1	1	4	3	0	1	3
EABT7265	1	2	2	3	0	2	1
EABT7266	7335.17	8051.69	8416.87	10131.1	4749.77	5367.73	5170.72
EABT7267	2342.62	3377.9	3175.38	4790.13	3915.55	1471	1681.98
EABT7268	6	7	3	0	4	0	2
EABT7269	0	8	22	5	27	1	6
EABT727	0	0	3	2	0	0	0
EABT7270	0	1	1	3	0	0	0
EABT7271	13	42	26	7	1	2	1
EABT7272	6963.33	17235.2	13877.5	36237	7321.75	5283.34	7859.42
EABT7273	73	111.01	4735.91	1337.07	568.88	128	1579.62
EABT7274	3	3	10	4	3	0	0
EABT7275	1	6.27	17	1	1	0	0
EABT7276	0	4	12	1	0	0	0
EABT7277	12	7	63	12	2	9	8

EABT7278	0	6	9	5	7	0	2
EABT7279	0	2	7	0	0	0	0
EABT728	13588.2	23455.8	12175.3	16986.2	7607.41	9267.64	9071.6
EABT7280	1	3	5	0	1	0	0
EABT7281	8	1	10	1	4	11	1
EABT7282	5	24	8.05	13	14	5	23
EABT7283	32	2	4	6	3	0	0
EABT7284	0	4	0	0	3	0	0
EABT7285	3	3	0	0	2	3	3
EABT7286	0	2	17	1	0	0	0
EABT7287	997.2	1436.57	2364.95	1085.6	1236.6	1199.11	1522.93
EABT7288	0	4	3	4	3	0	2
EABT7289	1	2	3	27	6	3	16
EABT729	18	14	0	0	1	0	2
EABT7290	0	2	30	1	0	1	1
EABT7291	4	16	0	0	2	31	24
EABT7292	0	1	17	1	0	0	0
EABT7293	5	5	1	4	0	1	3
EABT7294	0	1	3	0	0	0	0
EABT7295	3	2	4	9	0	1	0
EABT7296	0	1	8	0	0	0	0
EABT7297	3	1	4	8	8	1	2
EABT7298	0	1	11	2	0	1	0
EABT7299	1	1	2	1	2	0	1
EABT73	2	29	38	22	2	4	2
EABT730	1	3	2	3	0	1	0
EABT7300	0	0	20	1	2	0	0
EABT7301	2	1	35	5	1	2	2
EABT7302	7	33	14	17	3	4	5
EABT7303	7	12	9	1	5	0	37
EABT7304	0	1	16	2	0	0	0
EABT7305	95	112	192	490	117	36	51
EABT7306	0	3	11	0	0	0	0
EABT7307	0	5	1	1	2	1	0
EABT7308	0	4	2	0	8	0	0
EABT7309	1478.07	1751.51	44	68	332.89	164.24	975.37
EABT731	0	1	4	1	4	1	7
EABT7310	0	0	15	14	1	0	1
EABT7311	3	0	4	0	1	0	0
EABT7312	0	1	4	0	1	0	0
EABT7313	3	0	16	3	3	1	0
EABT7314	1	1	2	1	0	0	0
EABT7315	3286.4	3317.85	343	432	315.87	4066.92	2425
EABT7316	0	1	9	1	0	0	0
EABT7317	2	0	30	0	0	0	0
EABT7318	1	9	0	0	1	11	6
EABT7319	4	10	5	9	3	2	0
EABT732	570	568.01	1319.97	789.02	648.51	143	481.58
EABT7320	2231.64	3160.38	2295.83	3452.83	2085.27	1982.06	1925.29
EABT7321	10486	14415.5	10142.5	12759.4	7263.93	11339.1	8599.53
EABT7322	1	0	3	1	0	0	0

EABT7323	8965.18	9446.85	5342.9	7950.47	7713.67	12795	8156.97
EABT7324	1	0	10	0	0	3	0
EABT7325	0	0	0	3	1	0	0
EABT7326	2697.39	10564.9	10739.9	30756.6	3592.08	228	1890.35
EABT7327	2	2	5	3	0	2	1
EABT7328	926.6	2524.21	2550.87	3656.19	1795.43	136	1277.99
EABT7329	2	1	0	0	0	3	2
EABT733	386.65	351.95	277.53	607.86	300.74	393.3	283.15
EABT7330	7.95	6	4	103	6	2	1
EABT7331	15	17	10	3	4	1	1
EABT7332	0	0	4	2	0	0	0
EABT7333	706.2	1246.04	821.97	1867.89	683.63	296.61	816.04
EABT7334	29	44	66	102.01	52	10	23
EABT7335	6034.05	10501.1	7680.69	18901.2	6469.41	7405.15	7586.94
EABT7336	3	8	6	7	6	4	8
EABT7337	1	1	12	16.98	3	0	2
EABT7338	1	1	8	1	3	0	4
EABT7339	0	1	3	1	1	1	2
EABT734	87	371	94	4209	971	0	11
EABT7340	0	0	4	9	0	2	0
EABT7341	782.97	884.98	481.94	1414.64	801	455.12	491.64
EABT7342	4	6	6	6	0	4	1
EABT7343	1641.45	981.64	970.15	1054	1458.03	2188.58	3072.69
EABT7344	21977.6	14030.7	6910.62	5900.19	5022.28	10120	5726.67
EABT7345	0	0	3	1	0	0	0
EABT7346	3	4	20	13	1	4	3
EABT7347	0	0	0	0	11	0	11
EABT7348	1	0	3	1	0	0	0
EABT7349	8	17	77	1	2	3	0
EABT735	2415.27	12042	4479.1	8668.49	3392.73	2231.5	2422.35
EABT7350	737.67	1051.97	680.97	1323.23	804.15	731.5	500.07
EABT7351	0	2	10	1	1	0	0
EABT7352	1395.52	2351.68	1951.62	4196.07	2314.74	767.93	1177.18
EABT7353	1179.71	1371.15	888.51	1416.31	1138.74	848.27	885.07
EABT7354	7	13	18	16	1	10	12
EABT7355	0	0	2	0	3	0	0
EABT7356	6	15	13	28	14	3	12
EABT7357	0	2	3	3	1	0	0
EABT7358	1	6	5	222	12	1	21.95
EABT7359	0	0	11	1	0	1	0
EABT736	187	213	197.72	348.85	191	120.02	124.98
EABT7360	0	0	2	1	0	1	0
EABT7361	5476.26	7511.75	1321.26	3545.43	1196	5556.03	5264.34
EABT7362	0	2	6	2	0	0	0
EABT7363	1	2	10	1	0	11	3
EABT7364	7	6	14.99	10	0	4	4
EABT7365	0	0	5	0	0	1	1
EABT7366	763	763	620.65	7080.37	2835.88	58	741
EABT7367	0	0	0	4	0	0	0
EABT7368	580.01	912.46	673.31	1400.12	843.98	831.42	672.11
EABT7369	0	0	5	0	0	1	0

EABT737	7	23	16	338.87	22	1	14
EABT7370	0	2	9	3	2	4	1
EABT7371	5	14	28	22	4	3	2
EABT7372	15	16	0	0	0	45	30
EABT7373	2	1	7	1	0	0	0
EABT7374	0	1	5	8	1	2	1
EABT7375	1515.33	2178.2	1334.43	1665.09	1099.46	1238.06	1255.34
EABT7376	1	4	8	1	0	17	0
EABT7377	0	0	3	1	5	0	0
EABT7378	17	20.99	16.04	35.59	12	47	18
EABT7379	0	4	0	0	0	1	1
EABT738	25	2	11	1	0	10	0
EABT7380	0	2	8	4	1	1	0
EABT7381	7	2	5	0	1	12	1
EABT7382	2	5	29	2	4	0	0
EABT7383	1	1	2	1	0	0	0
EABT7384	1	1	3	0	5	1	7
EABT7385	5	7	12	0	0	3	1
EABT7386	2	8	3	3	1	0	0
EABT7387	0	3	0	0	2	0	0
EABT7388	2	2	9	1	0	0	0
EABT7389	3	4	33	33	8	3	9
EABT739	2	3.55	4	0	0	0	0
EABT7390	3	1	0	0	0	0	2
EABT7391	1	3	13	11	3	0	0
EABT7392	1	1	0	2	2	1	0
EABT7393	2	8	2	2	3	0	19
EABT7394	0	0	16	2	0	0	0
EABT7395	0	6	4	12	4	0	0
EABT7396	1	4	40.02	0	21	11	44.82
EABT7397	2118.54	4599.21	5417.59	8938.17	1914.93	2571.92	2435.65
EABT7398	5	4	2	0	1	1	1
EABT7399	3	8	0	0	1	4	8
EABT74	1	2	8	1	2	0	0
EABT740	0	8	31	4	2	0	1
EABT7400	1	3	12	5	0	0	0
EABT7401	3	3	3	4	3	1	0
EABT7402	12	2	10	12329.8	4	11	28
EABT7403	1	2	0	27	3	1	1
EABT7404	4	5	12	1	0	0	0
EABT7405	2	1	9	1	1	7	3.04
EABT7406	2845.87	4059.66	5795.94	7252.2	3006.15	4336.36	2585.78
EABT7407	8235.49	13586.7	10017.4	17718.7	6448.67	7979.51	6993.61
EABT7408	1	1	5	0	1	0	0
EABT7409	0	0	0	9	0	0	0
EABT741	0	0	0	12	0	0	0
EABT7410	6	6	39	5	0	2	2
EABT7411	0	3	10	0	0	0	0
EABT7412	0	1	2	5	0	0	1
EABT7413	0	1	1	3	0	0	0
EABT7414	3596.03	9950.03	4681.25	8550.58	2037.01	70	10

EABT7415	1286.74	1206.8	78	168	889	2718.01	5344.54
EABT7416	0	1	49	0	1	0	0
EABT7417	1	3	0	0	0	0	3
EABT7418	0	4	1	3	0	0	1
EABT7419	0	0	8	2	0	0	0
EABT742	5820.67	5765.13	1358.42	679.93	1561.59	15	35
EABT7420	0	19	0	0	1	0	0
EABT7421	1	1	1	0	0	0	2
EABT7422	0	0	0	0	1	4	6
EABT7423	0	1	5	1	1	0	0
EABT7424	1	11	8	4	0	8	1
EABT7425	1552.98	3687.93	7640.31	12769.6	2469.49	1386.89	1796.34
EABT7426	11	8	0	1	0	34.99	0
EABT7427	0	0	1	0	4	0	1
EABT7428	8	38.28	14.99	10	15	0	4
EABT7429	19489.9	58294.1	50690.3	6160.82	40996.4	1080.38	9725.78
EABT743	873.98	1227.29	1087	2426.86	1196.97	762.02	840.5
EABT7430	0	0	6	0	0	0	0
EABT7431	14060	9939.01	5673.88	10189.2	7562.9	17994.1	7998.86
EABT7432	924.07	1243.53	729	1532	785.02	626.99	769
EABT7433	2	2	0	5	0	0	0
EABT7434	0	2	2	0	0	0	5
EABT7435	2155.86	4259.06	14149.4	2554.09	3969.74	1378.03	1448.18
EABT7436	2	4	8	6	1	19	21
EABT7437	4	8	232	5	2	4	2
EABT7438	0	0	10	0	1	0	0
EABT7439	11773.5	21823.9	20557	41992.9	11190.7	7047.47	6446.2
EABT744	7	9	24	15	3	5	2
EABT7440	0	5	6	0	0	0	0
EABT7441	1	3	3	3	0	0	0
EABT7442	0	0	9	0	0	0	0
EABT7443	0	0	0	0	0	0	0
EABT7444	0	1	0	4	0	0	0
EABT7445	0	0	11	2	0	3	1
EABT7446	2	11	25	9	13	1	1
EABT7447	5	6	4	1	1	20	3
EABT7448	20	15	0	0	0	1	2
EABT7449	182.04	376	2207.1	3540.11	844	65	172
EABT745	0	4	1	2	0	0	2
EABT7450	3	3	4	1	1	1	1
EABT7451	3188.91	4733.31	6908.03	4640.7	3747.15	2902.01	1844.07
EABT7452	0	0	5	5	0	0	0
EABT7453	3	2	3	2	0	1	2
EABT7454	5	5	29	37.99	1	5	2
EABT7455	1	3	6	11	0	0	0
EABT7456	1518	1605	1150	2695.1	1416	1429.03	736.02
EABT7457	0	1	12	0	0	0	0
EABT7458	23	29.99	68	85	94	14	44.23
EABT7459	0	0	0	3	3	0	3
EABT746	3	12	19	3	6	1	39
EABT7460	0	1	8	0	7	0	2

EABT7461	0	1	9	0	0	0	0
EABT7462	2	3	13	10	0	0	0
EABT7463	0	4	2	0	5	0	9
EABT7464	1	5	1	0	1	0	1
EABT7465	715.48	919.96	1199.8	1788.21	632.9	567.32	515
EABT7466	44	85	99	183	93	6	13
EABT7467	3	8	28	26	3	2	1
EABT7468	21	39	19.81	2	3.01	19	12
EABT7469	2	7	9	1	1	0	2
EABT747	0	0	8	0	0	0	0
EABT7470	6	72.82	17.98	149.65	2	29.9	10.08
EABT7471	1	1	2	4	2	0	2
EABT7472	3	6	0	0	0	0	0
EABT7473	40	41	18	187	46	6	11
EABT7474	2	5	15	10	0	1	0
EABT7475	0	1	7	1	0	0	0
EABT7476	7	13	9	72.3	21	0	6
EABT7477	1009.44	1265.27	3039.7	1495.72	1063.47	340.15	413.99
EABT7478	0	0	5	0	0	0	0
EABT7479	0	1	0	0	1	21	11
EABT748	400	421	123	410.39	752	1	48
EABT7480	2	1	7	5	5	0	13
EABT7481	13	63	57	32	6	31	23
EABT7482	413	726	384	1752.1	1262	551	604
EABT7483	0	2	3	2	2	1	0
EABT7484	274	784.81	1940.55	4030.02	937	187	379
EABT7485	6547.19	38869.4	17842.3	38240.1	11492.6	6122.26	4308.94
EABT7486	2	7	0	0	1	0	4
EABT7487	4	17	39	44	8	6	8
EABT7488	0	0	4	0	0	0	2
EABT7489	0	4	0	0	0	0	0
EABT749	531.61	1415.72	2871.1	3526.92	1750.46	163	442.96
EABT7490	0	1	0	0	0	6	1
EABT7491	267	55	28	55	35	10	90
EABT7492	8	12	17	3	4	8	6
EABT7493	0	1	5	2	1	0	0
EABT7494	2	3	6	0	0	4	2
EABT7495	0	1	6	0	0	0	1
EABT7496	0	1	12	0	0	0	0
EABT7497	0	0	9	0	0	0	0
EABT7498	0	3	0	63	6	2	0
EABT7499	443	586.96	758	1267.95	181	601	388
EABT75	1	1	9	4	0	5	0
EABT750	29	53	32	148.01	32	17	38
EABT7500	1	1	1	1	0	0	0
EABT7501	0	2	4	4	1	0	0
EABT7502	2	3	3	1	0	0	0
EABT7503	13	0	0	0	1	64	0
EABT7504	0	2	149	6	2	5	4
EABT7505	771	893.72	839.62	1459.23	747	507.69	515
EABT7506	7972.67	11179	7007.87	14512.8	11089.6	5308.7	5915.35

EABT7507	13	26	15	29	19	7	4
EABT7508	3	4	10	10	6	3	5
EABT7509	0	0	10	0	0	0	0
EABT751	0	1	8	3	1	1	0
EABT7510	1051.14	1814.25	2823.39	6485.33	1448	679	750.91
EABT7511	3341.35	4883.41	3047.85	5591.9	2973.27	2070.74	2367.57
EABT7512	4	6	8	6	4	0	0
EABT7513	1	3	0	0	0	5	1
EABT7514	8	12	2	1	0	27	5
EABT7515	2647.52	3351.42	1684.59	2225.39	1566.1	2407.27	1786.85
EABT7516	698.92	955.29	719.21	849.72	1063.91	307.08	454.25
EABT7517	0	0	17	0	0	1	1
EABT7518	0	0	1	10	2	1	0
EABT7519	0	0	2	1	0	0	1
EABT752	0	2	27	0	0	0	1
EABT7520	0	2	9	0	1	0	0
EABT7521	1775.99	4988.98	6502.99	12475.1	4369.36	1272	2371.95
EABT7522	14	7	1	0	0	7	1
EABT7523	1	0	24	4	0	0	0
EABT7524	3773.03	14876.9	3844.99	531	5574	247	1623.7
EABT7525	5	11	15	2	1	1	2
EABT7526	1	0	0	2	1	0	0
EABT7527	1	1	11	0	0	1	0
EABT7528	3	4	12	4	1	23	8
EABT7529	0	0	15	1	0	1	0
EABT753	0	3	3	3	2	0	6
EABT7530	2583.99	3921	6927.73	5222.44	2654.14	1	103
EABT7531	1	2	0	0	0	2.03	3
EABT7532	0	0	2	6	0	0	0
EABT7533	2	2	62.1	4	0	0	0
EABT7534	175	408	744.84	97	63.94	337	118
EABT7535	0	4	1	0	0	0	5
EABT7536	8	21	84	20	4	4	4
EABT7537	0	0	0	0	2	8	2
EABT7538	1	1	2	0	0	0	1
EABT7539	1	4	3	0	0	0	1
EABT754	0	2	0	6	0	0	0
EABT7540	1	0	1	8	1	0	2
EABT7541	2	1	32	1	1	1	1
EABT7542	1	2	5	3	0	0	0
EABT7543	0	0	11	0	0	0	0
EABT7544	5	16	12	15	11	4	6
EABT7545	0	2	8	1	0	0	0
EABT7546	15	17	35	31	68	10	19
EABT7547	0	0	38	0	0	0	0
EABT7548	976.82	1542.73	2632.62	3493.35	1814.49	1166.3	1225.96
EABT7549	0	0	15	0	0	1	0
EABT755	51083.8	22	5	1	8	4066.07	2
EABT7550	2	3	11	2	2	0	0
EABT7551	536.01	658.92	419	867	438.93	706	411.88
EABT7552	3201.11	4318.33	14357.9	7092.9	2727.96	552	1629.05

EABT7553	511	836	1019.5	1100.57	390	297	379.97
EABT7554	0	0	2	14	0	0	6
EABT7555	18	0	14	3	0	16	0
EABT7556	196	367	400	635.35	802.75	143	273.5
EABT7557	0	1	1	2	0	0	0
EABT7558	271	643	759	1782.85	364	127	231
EABT7559	2	5	35	5	6	1	1
EABT756	2	0	5	2	3	1	3
EABT7560	10	3	0	0	1	24	3
EABT7561	0	2	0	1	2	0	2
EABT7562	5.58	9	11	2	4	1	3
EABT7563	0	4	10	0	2	0	0
EABT7564	0	1	6	0	0	0	1
EABT7565	21	56	227.02	136	31	28	63
EABT7566	6	3	3	150	2	0	1
EABT7567	0	1	10	0	0	0	0
EABT7568	1	4	2	11	4	0	1
EABT7569	0	1	2	1	0	0	0
EABT757	2964.67	3700.83	1694.01	5786.5	1814.97	2228	1933.08
EABT7570	69.37	133	152.92	75	96.47	39.18	673.75
EABT7571	107	430	580.04	6118.21	341	39	69
EABT7572	1.91	2	4	11	15	3	9.87
EABT7573	0	3	0	0	2	2	1
EABT7574	0	0	1	0	4	0	10
EABT7575	1	0	4	0	0	0	0
EABT7576	3	3	4	5	46	0	2
EABT7577	0	0	7	0	0	0	0
EABT7578	0	1	7	0	0	0	0
EABT7579	1	3	5	1	0	0	0
EABT758	0	0	10	2	1	0	0
EABT7580	15324	26722.8	8017.23	10830.2	13016.8	9752.78	8880.19
EABT7581	191.81	452.18	1303.76	686.27	1006.92	35	231
EABT7582	0	0	0	0	2	0	0
EABT7583	0	3	7	0	2	0	0
EABT7584	5	2	18	7	1	1	0
EABT7585	2	33	0	0	0	1.83	3
EABT7586	25	7	0	0	0	18	0
EABT7587	11	9	7	6	5	6	37
EABT7588	6399.49	7399.76	8521.33	7206.18	2511.95	2101.82	2466.72
EABT7589	0	1	3	0	0	0	0
EABT759	0	0	13	5	4	0	1
EABT7590	3704.27	6243.1	4292.3	8295.23	5175.92	3037.49	3545.49
EABT7591	1	6	31.44	0	0	0	0
EABT7592	91	91	233.28	188	87	125.66	64.97
EABT7593	2	7	10	9	0	1	0
EABT7594	0	0	0	0	1	0	7
EABT7595	2	0	1	3	1	0	0
EABT7596	6	2	27	1	0	2	0
EABT7597	578.75	642.49	464.07	645.94	704.79	570.04	519
EABT7598	0	0	3	0	1	1	0
EABT7599	2	5	88	0	3	0	0

EABT76	0	0	3	0	1	0	1
EABT760	1	9	2	8	10	0	0
EABT7600	3	3	3	3	1	2	1
EABT7601	1952.8	2815.85	3841.88	5808.68	2284.48	2621.55	2618.44
EABT7602	8	3	19	3	1	14	12
EABT7603	5	12	43	8	16	2	11
EABT7604	5	5	39	3	4	0	1
EABT7605	763.23	425	172.9	5	9	1	9.31
EABT7606	0	1	14	4	0	0	0
EABT7607	14	44	131	250.06	70	7	10
EABT7608	0	0	9.03	4	1	0	2
EABT7609	0	2	15	0	2	0	2
EABT761	2	9	0	0	0	15	0
EABT7610	3	3	21	12	1	2	2
EABT7611	0	1	13	2	2	0	1
EABT7612	1	5	4	0	16	0	0
EABT7613	969	1467	1112.01	2068	748	1427	1106.34
EABT7614	1	2	11	1	0	0	0
EABT7615	1230	1818	1944	3342.13	1264	21	492
EABT7616	2	3	22	3	0	1	1
EABT7617	897.52	1125.15	1648.96	2534.11	1054.88	870.65	697
EABT7618	1	1	9	2	1	1	0
EABT7619	0	2	3	3	0	0	0
EABT762	1	1	16	0	0	0	0
EABT7620	1047.06	1989.43	2879.38	3176.29	1746.01	639.98	1098.36
EABT7621	0	1	3	8	0	0	1
EABT7622	411	86	11	8	5	164	1
EABT7623	1	0	6	2	0	0	0
EABT7624	16	8.02	13	23	1	0	1
EABT7625	2	9	17	8	0	0	3
EABT7626	8	12	0	0	0	16	3
EABT7627	0	1	3	1	1	0	1
EABT7628	0	4	1	8	2	0	0
EABT7629	2	2	3	5	3	1	0
EABT763	1	0	0	1	0	2	2
EABT7630	0	0	2	2	1	0	0
EABT7631	0	0	2	22	0	0	0
EABT7632	27	91	278	106	21	1	1
EABT7633	0	0	1	3	0	0	0
EABT7634	2	6	54	6	6	4	5
EABT7635	1	7	10	1	0	0	2
EABT7636	33	33	15	39.86	11	5	12
EABT7637	4	6	24	2	0	0	1
EABT7638	1	2	2	1	0	1	0
EABT7639	0	1	0	13	2	0	0
EABT764	1	2	10	1	3	1	3
EABT7640	1	3	6	0	169	0	1
EABT7641	31	13	0	13	1	41	1
EABT7642	498	1222	56	13	927	146	72
EABT7643	1	1	7	56	3	0	0
EABT7644	1	2	23	1	0	0	0

EABT7645	2	8	3	242	1	1	2
EABT7646	0	0	1	0	11	0	6
EABT7647	0	2	4	3	0	0	0
EABT7648	416.99	640.91	658.94	1430.48	694.93	469.17	283
EABT7649	1	4	9	7	4	0	2
EABT765	4	5	1	7	3	5	2
EABT7650	3836.84	1265.98	5210.26	15019.4	2025.65	695.34	201.01
EABT7651	263.34	180.45	23	224	400.88	133.07	1479.08
EABT7652	3	2	4	1	0	0	0
EABT7653	0	0	3	0	0	0	0
EABT7654	0	0	9	3	0	0	0
EABT7655	2	1	0	0	0	2	0
EABT7656	0	0	8	0	0	1	0
EABT7657	1	0	7	3	0	0	0
EABT7658	4	9	8	9	2	0	3
EABT7659	1	0	28	0	0	0	1
EABT766	0	3	13	3	1	2	1
EABT7660	0	0	13	0	0	0	0
EABT7661	336.04	245.27	1752.17	449.53	250.3	134	155
EABT7662	3	4	8	1	4	16	28
EABT7663	0	1	5	7	1	0	0
EABT7664	24	16	0	0	0	24	1
EABT7665	284.59	705.82	611.26	814.73	336.3	54.96	149.11
EABT7666	0	4.87	23	1	5	1	3
EABT7667	2	63	1	0	0	8	0
EABT7668	3	6	0	1	1	0	0
EABT7669	2	2	6	4	1	0	0
EABT767	0	1	2	2	0	0	0
EABT7670	0	4	1	0	0	5	1
EABT7671	0	0	9	0	0	0	0
EABT7672	1	6	10	5	2	1	0
EABT7673	75	93	118.07	245.01	93	62	106
EABT7674	0	0	5	1	0	0	0
EABT7675	7	2	2	1	1	1	0
EABT7676	1	1	2	4	0	0	0
EABT7677	1	5	4	0	0	0	1
EABT7678	9	18	59	27	1	0	0
EABT7679	0	1	6	0	0	0	2
EABT768	0	0	6	0	0	0	0
EABT7680	0	0	5	0	0	0	0
EABT7681	0	2	9	4	2	0	1
EABT7682	1	0	32	0	1	2	1
EABT7683	0	2	7	0	4	1	1
EABT7684	3	6	12	19	1	2	3
EABT7685	1	2	3	3	1	3	9
EABT7686	153	283.62	341.6	262.01	99	169.01	112.81
EABT7687	4	5	0	0	1	0	0
EABT7688	0	0	2	7	0	0	0
EABT7689	0	2	5	3	2	0	0
EABT769	2	0	2	5	1	0	0
EABT7690	9	20	21	7	0	0	1

EABT7691	1	1	3	5	0	1	1
EABT7692	0	1	4	3	1	0	0
EABT7693	0	2	4	41	15	0	0
EABT7694	1	2	4	10	2	0	2
EABT7695	0	2	6	3	0	0	0
EABT7696	4	7	20	349	28	9	5
EABT7697	0	0	9	0	0	0	0
EABT7698	2	3	6	17	1	1	2
EABT7699	0	1	6	2	1	1	0
EABT77	0	2	12	1	0	1	0
EABT770	1	1	3	8	2	2	1
EABT7700	20	39	0	0	5	52	45
EABT7701	664.73	1144.98	370	888.6	296	891.26	700.24
EABT7702	2	5	13	2	0	0	0
EABT7703	25	23.05	31	3	2	18	8
EABT7704	0	0	4	4	0	0	0
EABT7705	27	83.86	95	190	184.31	22	39
EABT7706	0	4	4	5	1	3	2
EABT7707	0	0	5	2	6	0	1
EABT7708	0	6	10	14	10	0	0
EABT7709	1	0	37	2	1	0	0
EABT771	0	7	5	4	0	0	1
EABT7710	0	1	6	1	0	0	0
EABT7711	1369.76	2817.41	4813.7	6945.74	1866.18	1878.97	1442.97
EABT7712	0	3	2	0	0	1	0
EABT7713	108.64	126	73	288	93.01	97	159.17
EABT7714	688	1269	992.01	283.92	2760.99	91	169
EABT7715	386	3167.89	1273.63	976.54	377.12	1659.55	348.94
EABT7716	2	5	4	3	1	4	4
EABT7717	0	4	3	24	9	0	0
EABT7718	88	124	116.92	337.05	119	87	80.99
EABT7719	0	0	7	26	7	0	0
EABT772	0	6	22	6	7	3	3
EABT7720	2	2	7	0	2	0	2
EABT7721	898.84	1312.86	1483.9	2963.94	601.99	5	20
EABT7722	1	6	8	1	18	1	0
EABT7723	2	3	3	0	2	3	1
EABT7724	3	2	4	0	0	2	3
EABT7725	13.02	53	10	8	6	43	7
EABT7726	2	5	6	2	0	2	1
EABT7727	41	70	189.71	85.25	22	39	44
EABT7728	2	5	13	20	0	0	1
EABT7729	0	0	4	4	1	0	2
EABT773	38	53	0	0	1	8	10
EABT7730	0	0	0	6	1	0	0
EABT7731	0	1	10	0	3	1	0
EABT7732	44	61	115	70	690	9	414
EABT7733	0	4	18	4	0	0	1
EABT7734	414.15	428.66	410	371.08	524.02	290	257
EABT7735	7	9	6	1	2	9	1
EABT7736	3557.11	87198.2	27246.7	1500.28	29843.4	26	910.05

EABT7737	0	0	4	23	0	0	0
EABT7738	3	6	19	1	0	1	0
EABT7739	8	6	9	10	2	3	4
EABT774	0	1	3	0	0	0	0
EABT7740	0	4	8	2	0	1	0
EABT7741	1	3	8	8.53	3	0	8
EABT7742	20	107	19	57	6	1	7
EABT7743	1	2	2	4	0	0	3
EABT7744	231876	52073.6	69	15	52958.7	217774	387193
EABT7745	0	2	0	0	0	1	2
EABT7746	11	17	19	16	14	0	8
EABT7747	110	151.1	75	283.74	134	99	71
EABT7748	0	2	29	2	0	0	0
EABT7749	0	8	10	3	0	1	0
EABT775	0	1	1	3	0	0	0
EABT7750	1825.24	1894.76	2177.08	1370	1069.03	1993.19	1587.33
EABT7751	0	1	8	1	0	0	0
EABT7752	0	0	0	0	0	0	5
EABT7753	0	2	1	1	0	0	0
EABT7754	1	1	2	3	0	0	0
EABT7755	362	640.29	468.92	1933.08	315.26	206.47	361.86
EABT7756	9	6	4	29	5	3	4
EABT7757	18	61	53	210	81.84	24	42
EABT7758	0	1	64	88	9	0	3
EABT7759	0	0	6	0	1	0	1
EABT776	0	0	0	1	1	0	0
EABT7760	5	4	0	0	0	1	2
EABT7761	5	12	22	10	3	4	3
EABT7762	0	1	3	0	5	0	0
EABT7763	2	2	9	0	0	2	0
EABT7764	0	6	24	0	0	0	0
EABT7765	2	5	4	5	1	8	4
EABT7766	0	2	20	8	1	4	0
EABT7767	8	9	1	21	2	3	8
EABT7768	44.54	74	55	327.03	27	12	30
EABT7769	6	14	157	27	0	5	4
EABT777	0	0	0	0	1	0	2
EABT7770	0	5	8	5	0	2	0
EABT7771	2	1	9	2	0	2	0
EABT7772	0	0	0	0	0	0	0
EABT7773	28	23	1	0	0	1	2
EABT7774	2	2	3	3	0	0	0
EABT7775	38	76	203	157	340	6	69
EABT7776	0	2	0	6	0	0	1
EABT7777	0	3	10	0	0	0	0
EABT7778	131	155	36	366	219	26	99
EABT7779	0	0	25	0	0	1	0
EABT778	1	4	0	0	0	2	0
EABT7780	2190.6	1276	112	32	3249.08	2927.02	14576.6
EABT7781	0	1	4	3	0	1	0
EABT7782	2	1	2	0	0	0	0

EABT7783	9	19	3	4	10	12	28
EABT7784	3112.56	49	36.01	184.99	1716.2	835.44	15336.8
EABT7785	0	3	8	7	2	0	1
EABT7786	64	28	4	16	10	0	0
EABT7787	3	8	4	1	1	4	2
EABT7788	0	0	36	0	0	0	0
EABT7789	0	2	74	10	8	0	3
EABT779	3	2	1	5	3	0	0
EABT7790	0	0	7	1	0	1	0
EABT7791	0	4	8	14	3	0	0
EABT7792	3	5	6	11	4	0	1
EABT7793	353.51	334.23	484.74	707.23	230	295.21	269.76
EABT7794	2951.05	4204.57	1749.7	4008.12	2124.15	1665.92	2013.65
EABT7795	11	9	15	7	0	4	2
EABT7796	2	0	0	0	0	1	3
EABT7797	0	0	0	0	0	2	63
EABT7798	0	0	0	1	7	1	2845
EABT7799	1	4	2	3	1	0	0
EABT78	14	28	48	40	9	2	8
EABT780	0	0	11	3	2	0	1
EABT7800	0	1	2	13	1	0	0
EABT7801	0	0	4	0	0	0	0
EABT7802	1103.29	1745.07	1117.02	1777.02	1105.48	1067.25	904.03
EABT7803	22	33	3	0	2	34	20
EABT7804	1	1	11	3	0	0	0
EABT7805	1	3	0	1	0	0	0
EABT7806	477.6	299	564	2314.08	329.01	3	20
EABT7807	0	0	5	0	9	0	0
EABT7808	4	11	34	9	6	5	1
EABT7809	58	191.6	17	13	21	45	73
EABT781	9	13	0	0	0	6	13
EABT7810	0	0	6	0	1	0	0
EABT7811	3	0	1	0	0	2	7
EABT7812	5	4	34.2	2	1	1	0
EABT7813	0	1	4	0	0	0	0
EABT7814	0	2	1	1	2	0	0
EABT7815	6	5	11	49	11	9	13
EABT7816	3	9	18	7	2	0	2
EABT7817	1	3	2	1	1	0	0
EABT7818	1069.98	1522.99	1256.99	758.62	1001.94	647.43	626
EABT7819	0	0	16	0	0	0	0
EABT782	0	0	10	0	0	0	0
EABT7820	0	0	6	0	0	0	0
EABT7821	0	0	0	0	0	0	0
EABT7822	0	1	4	1	0	0	0
EABT7823	0	2	17	0	0	0	2
EABT7824	0	0	0	0	0	0	13
EABT7825	0	12	64	14935.6	0	0	0
EABT7826	832.05	2001.22	5809.48	10014.7	2266.02	1577.02	1656.11
EABT7827	1127	1578.04	1508	3129	1040.02	1218.26	925
EABT7828	1	6	7	4	0	0	0

EABT7829	802.73	1146.17	1265.55	2714.63	753.32	697.13	734.93
EABT783	2	1	3	2	0	0	0
EABT7830	82	99	24	0	6	3	39
EABT7831	1007.61	902.52	329.95	1174.87	461.61	1102.23	599.94
EABT7832	3	6	28	12	5	4	6
EABT7833	132	460	1912.02	6404.49	690	29	148
EABT7834	1	3	6	2	0	4	4
EABT7835	8	1	0	0	0	7	1
EABT7836	1	14	6.01	4	2	0	4
EABT7837	1	4	20	0	1	1	0
EABT7838	2	4	2	1	2	1	3
EABT7839	1	4	20	46	3	8	9
EABT784	6	1	4	1	1	0	0
EABT7840	0	0	1	0	0	1	0
EABT7841	971.14	1488.05	3297.19	5721.02	1953.1	1014.99	832
EABT7842	8	58	7	2	18	1	9
EABT7843	1	3	12	57	6	1	4
EABT7844	2	0	6	0	0	0	0
EABT7845	6695.66	3177.23	240	1594.66	998.3	1055.77	1135.11
EABT7846	6042.99	4081.59	97.31	2	2442.53	13514.3	4287.54
EABT7847	3	2	4	1	1	1	0
EABT7848	0	2	1	1	2	0	0
EABT7849	6	12	27	21	1	3	3
EABT785	19	9	15	47	127	22	6
EABT7850	0	14	89	5	1	1	0
EABT7851	0	0	7	1	0	0	0
EABT7852	1	3	17	0	0	0	0
EABT7853	1744.67	1971	151.94	487.88	695.98	448	815.09
EABT7854	1	0	3	1	0	0	0
EABT7855	1	0	4	1	0	4	0
EABT7856	0	0	18	0	0	0	0
EABT7857	1	2	91	9	0	0	1
EABT7858	5	6	16	14	1	4	2
EABT7859	0	0	4	0	0	0	0
EABT786	0	6	3	13	1	0	0
EABT7860	1866.15	3044	4436.76	4487.34	3025.13	2015.42	2037.59
EABT7861	4	11	3	1	3	0	0
EABT7862	4	22	48	57	3	0	0
EABT7863	0	2	3	1	2	0	1
EABT7864	712.02	1034.45	1016	1960.91	837.29	312	503.45
EABT7865	3	6	11	0	1	1	0
EABT7866	0	1	0	0	2	4	2
EABT7867	0	0	3	0	3	0	0
EABT7868	99	94.14	133.91	193	66	105	341
EABT7869	0	2	11	0	0	0	0
EABT787	1	3	4	3	0	0	0
EABT7870	2	7	17	1	0	0	0
EABT7871	0	1	0	1	8	0	0
EABT7872	1994.61	2699.86	2225.86	3084.88	812.24	1290.76	827.71
EABT7873	15	29	52	49	15	0	47
EABT7874	4	5	14	3	1	1	3

EABT7875	1	0	1	1	0	0	0
EABT7876	16	27	10	22	12	51	10
EABT7877	11	69	64	93	10	5	11
EABT7878	0	2	0	1	0	0	4
EABT7879	8	8	9	2	2	2	0
EABT788	0	3	12	0	4	0	4
EABT7880	0	0	12	0	0	0	0
EABT7881	0	0	3	0	1	1	0
EABT7882	1	5	4	0	0	0	0
EABT7883	230.98	407.3	438.92	627.3	190.63	192	210.95
EABT7884	0	0	1	4	1	0	0
EABT7885	0	1	3	1	0	0	0
EABT7886	2560.99	4329.01	8338.46	12825.8	3342.63	3674	5237.17
EABT7887	3	19	48	16	8	0	1
EABT7888	0	3	1	0	0	0	0
EABT7889	2947.6	698	33	25	62	162	345
EABT789	0	1	40	1	2	2	0
EABT7890	19	32	55	61	6	10	8
EABT7891	3	23	58	22	19	2	3
EABT7892	2637	7897.2	643.55	549	1557.23	236	3032.3
EABT7893	1	11	4	15	3	0	1
EABT7894	1	1	0	2	12	0	22
EABT7895	3	2	3	0	1	0	0
EABT7896	0	0	2	1	5	0	0
EABT7897	5	11	1	2	0	2	2
EABT7898	154	269	509.99	1675.75	1531.89	145.87	75
EABT7899	1027.01	1529.07	1110	2361.05	1040	937.97	828
EABT79	27	25	220	16	9	16	15
EABT790	4	6	6	25	32	0	9.02
EABT7900	476.99	715	1128.26	2427.4	373	405	281.85
EABT7901	0	0	1	2	6	0	0
EABT7902	2	1	6	0	0	2	0
EABT7903	1	5	22	5	0	0	1
EABT7904	0	1	16	0	0	0	0
EABT7905	1285.02	2548.65	5952.92	7471.12	2221.36	702	1220.03
EABT7906	64	181	22	79	13	5	15
EABT7907	3	1	3	1	18	0	0
EABT7908	2	0	12	0	1	0	0
EABT7909	0	2	2	2	1	1	0
EABT791	3	5	9	14	3	3	10
EABT7910	0	0	4	0	0	0	0
EABT7911	0	8	1	0	0	1	2
EABT7912	0	1	11	5	0	1	0
EABT7913	1	1	10	6	4	0	2
EABT7914	4	20	1	0	2	47	1
EABT7915	1	2	1	1	2	0	0
EABT7916	2	8	5	5	2	2	8
EABT7917	2	7	1	1	3	6	4
EABT7918	100	145	142	395	193.01	136	71
EABT7919	3	1	5	0	0	0	0
EABT792	5	4	2	0	0	0	0

EABT7920	1	1	3	1	0	0	0
EABT7921	0	2	0	0	0	8	0
EABT7922	0	1	10	0	0	0	0
EABT7923	0	1	10	0	0	0	0
EABT7924	366.02	591	793.25	1367.03	453.71	361	381
EABT7925	3	3	0	3	2	0	1
EABT7926	0	1	2	3	0	0	1
EABT7927	0	0	2	5	0	2	0
EABT7928	0	0	10	0	2	0	0
EABT7929	20	32.01	35	49	33	15	44
EABT793	1	2	0	1	0	1	3
EABT7930	0	0	3	0	0	2	0
EABT7931	0	1	0	1	0	1	2
EABT7932	2	0	1	0	4	8	1
EABT7933	0	1	3	0	0	0	0
EABT7934	0	1	3	3	17	1	1
EABT7935	24	33	36	52	36	5	20
EABT7936	61	78	187	1072	316	57	99
EABT7937	1	1	6	0	0	0	0
EABT7938	0	2	0	0	2	1	0
EABT7939	3	8	8	14	2	0	0
EABT794	13	1	5	6	7	1	7
EABT7940	70	37	26	6	3	10	3
EABT7941	0	1	4	2	1	0	0
EABT7942	4	19	58	10	4	4	7
EABT7943	9	14	0	0	0	0	2
EABT7944	2	3	0	0	0	11	2
EABT7945	6	11	9	4	4	3	8
EABT7946	0	3	13	0	1	0	0
EABT7947	0	1	6	1	0	1	0
EABT7948	0	0	5	5	0	0	1
EABT7949	33.92	49	60	110	167	33	84
EABT795	0	0	0	34	0	0	0
EABT7950	20	35	54.03	1	15	1	21
EABT7951	80.85	120.77	128.1	208	162.76	55.95	47.44
EABT7952	1	2	2	1	0	0	0
EABT7953	0	5	2	0	1	0	0
EABT7954	16	12	50	48	2	1	3
EABT7955	2	1	7	0	0	0	2
EABT7956	15339.5	10297.8	771.44	5458	4300.15	6476.44	6295.32
EABT7957	14	20.67	38	7	7	15	20
EABT7958	2613.25	2739.05	557	331	701.99	2312.56	2517.12
EABT7959	484	453.96	407	473	580	389.85	279.02
EABT796	3	6	27	10	5	0	6
EABT7960	1	1	9	9	1	1	1
EABT7961	6	20	57.99	12	12	7	11
EABT7962	0	0	1	1	1	0	1
EABT7963	6	15	66	8	4	3	8
EABT7964	1	1	5	3	1	0	0
EABT7965	0	0	10	0	0	0	0
EABT7966	1	3	6	1	0	0	2

EABT7967	0	2	10	1	0	0	0
EABT7968	2	11	2	12	6	0	0
EABT7969	1	0	7	1	0	0	0
EABT797	919.91	1261.16	515.14	1127.01	1133.52	1096.39	2294.02
EABT7970	0	0	9	5	1	0	1
EABT7971	0	1	2	11	2	0	0
EABT7972	1	2	1	2	0	0	0
EABT7973	0	7	3	21	11	0	0
EABT7974	1	0	4	1	0	3	0
EABT7975	9	35	42	37	24	15	16
EABT7976	4	12	9	11	4	2	3
EABT7977	1	8	17	0	2	1	1
EABT7978	0	4	3	2	1	0	0
EABT7979	12	22.12	32	2	0	0	0
EABT798	0	1	2	1	0	0	0
EABT7980	127	161.97	186	269	140	53	72
EABT7981	0	1	3	3	0	0	0
EABT7982	32	36	61	437	53	0	6
EABT7983	3	0	0	0	0	6	0
EABT7984	3	2	31	19	3	2	3
EABT7985	0	1	2	4	0	0	1
EABT7986	4	2	0	5	1	1	0
EABT7987	0	2	0	0	2959.23	3	17
EABT7988	1403.27	5869.21	116.98	95	44	3611.63	93
EABT7989	32	34	41	54.08	11	32	30
EABT799	2	1	3	93	67	0	2
EABT7990	61	89	97	48	64.9	42.01	27
EABT7991	880.58	1539.08	1129.77	912.91	528.46	592.12	452
EABT7992	5	19	39	37	1	13	13
EABT7993	2	3	9	1	2	1	1
EABT7994	1	3	1	0	0	0	2
EABT7995	17	5	2	4	1	2	3
EABT7996	0	0	4	0	0	3	0
EABT7997	17	16	0	0	0	1	3
EABT7998	5	6	0	1	0	3	2
EABT7999	3	5	4	52	10	1	0
EABT8	0	1	0	12	2	2	0
EABT80	0	0	5	0	0	0	1
EABT800	2	3	13	14	9	0	4
EABT8000	78	162	131	437.99	346	24	17
EABT8001	0	1	2	2	0	0	0
EABT8002	67	275	65	822	46	56	79.01
EABT8003	0	1	9	0	0	0	0
EABT8004	1	0	15	0	0	5	2
EABT8005	8	18	76	17	8	2	15
EABT8006	0	0	7	1	0	0	0
EABT8007	0	1	4	4	3	0	0
EABT8008	0	2	2	6	1	0	3
EABT8009	3	1	5	2	0	8	0
EABT801	13	10	12	0	4	2	35
EABT8010	1091.66	1711.57	2314.84	3802.71	1780.29	1068.9	1105.93

EABT8011	511	841.14	2035.3	2198.93	844	71	342
EABT8012	1	0	0	0	0	0	4
EABT8013	14	42	27	30	6	7	17
EABT8014	0	6	8	15	0	1	1
EABT8015	1622.96	1999.21	1014.03	2518.24	1050.96	1871.85	408
EABT8016	4	19	4	0	182.06	0	3
EABT8017	61	42	14	2	18	11	54
EABT8018	1	3	2	3	1	1	2
EABT8019	3	8	22	24	26	6	23
EABT802	2	0	7	2	0	2	2
EABT8020	200	345	247.9	725.2	420.99	83	135
EABT8021	2162.07	2153.01	1398.4	2325.25	2616.04	468.79	702.15
EABT8022	78	125	75	373	253.37	56	68
EABT8023	22	58	73	111	32	16	17
EABT8024	39	57	0	0	4	33	23
EABT8025	1	1	0	5	0	0	0
EABT8026	0	0	2	30	0	0	0
EABT8027	2	1	9	0	0	0	0
EABT8028	2	3	19	2	1	0	1
EABT8029	0	17	4	0	1	0	1
EABT803	5631	8791.96	7781.36	11967.1	5775	7306.04	5899.99
EABT8030	2	1	3	3	0	2	1
EABT8031	6826.1	8289	5724.8	11219	4460.56	3498.09	4359.39
EABT8032	0	2	2	0	0	3	3
EABT8033	2	1	11	1	0	0	0
EABT8034	78	663.9	682.01	528.03	227	188	283
EABT8035	471.1	725.57	340.94	707.12	549.02	234.42	321.73
EABT8036	2	0	3	0	1	1	2
EABT8037	3	13	25	2	1	1	1
EABT8038	0	1	0	0	0	3	0
EABT8039	7	17	6	15	3	2	8
EABT804	19	9	0	0	0	28	0
EABT8040	21	42	53	9	0	1	0
EABT8041	1255	1745.3	2289.63	2527	1048	1219.25	907
EABT8042	1	1	4	0	0	0	0
EABT8043	879.58	1183.06	1411.91	2628.19	1084.96	911.82	828.39
EABT8044	3	1	0	0	1	36	0
EABT8045	7909.07	8954.59	2587.4	3872.13	2447.65	16311.4	10820.3
EABT8046	2	3	4	2	1	3	3
EABT8047	9	44	14	0	3	2	0
EABT8048	0	2	8	1	0	1	0
EABT8049	0	4	11	0	1	0	0
EABT805	0	1	2	9	3	0	0
EABT8050	0	1	4	1	0	0	0
EABT8051	1	5	4	0	0	0	0
EABT8052	2	4	22	1	0	0	0
EABT8053	1388.01	1288	1067	1068	1055.67	1360	1557
EABT8054	1	1	14	0	1	1	0
EABT8055	0	1	6	1	0	3	0
EABT8056	26	33	62	64.91	33	21	21
EABT8057	7	42	62	14	19	32	49

EABT8058	2078.34	4726.43	5706.46	3230.04	1650.47	0	8
EABT8059	16	34	35	13	4	0	5
EABT806	1	3	10	11	4	0	0
EABT8060	1	2	6	2	1	0	0
EABT8061	0	2	12	1	0	0	0
EABT8062	2274.81	3034.11	2665.02	5754.29	2282.74	1950.91	1754.21
EABT8063	0	0	6	0	0	1	1
EABT8064	0	1	8	0	1	0	0
EABT8065	6	7	0	0	0	3	7
EABT8066	0	2	11	15	9	8	18
EABT8067	0	0	4	1	0	0	0
EABT8068	82	107	61	110	14	37	24
EABT8069	1	0	19	2	2	0	0
EABT807	2	1	3	1	0	0	0
EABT8070	0	3	6	4	2	1	0
EABT8071	0	4	2	5	1	0	0
EABT8072	0	4	1	3	0	3	1
EABT8073	6	2	11	8	16	2	7
EABT8074	0	1	22	0	1	0	0
EABT8075	13	22	14	1	0	9	0
EABT8076	392	244	100	60	102	807	1090.98
EABT8077	0	3	7	1	1	0	0
EABT8078	0	0	0	8	0	0	0
EABT8079	5	4	3	0	2	2	1
EABT808	2	3	9	3	7	1	28
EABT8080	4	6	8	6	0	0	1
EABT8081	90	41.66	2	69	16	4	11
EABT8082	13.94	26	7	12	10	2	14
EABT8083	243.77	364.1	363.97	765	447.36	103.08	179.64
EABT8084	0	0	2	1	1	0	0
EABT8085	3	7	9	8	0	1	2
EABT8086	0	0	0	0	32	255	133
EABT8087	1	1	0	0	1	0	1
EABT8088	4	2	2	7	9	5	8
EABT8089	14	152	2607.29	80	31	4	19
EABT809	0	0	4	1	0	0	0
EABT8090	21	14	1	0	0	20	2
EABT8091	12	38	16	51	11.01	8	6
EABT8092	10	3	0	0	0	1	0
EABT8093	0	0	8	5	1	0	0
EABT8094	70	158	370.94	682.04	85	16	25
EABT8095	163	238	106	261	154	91	97
EABT8096	12845	44469.6	27174.3	49070.9	39121.4	1963	24535.7
EABT8097	4	8	39	14	2	3	3
EABT8098	155	204	225	1045	332	13	45
EABT8099	0	1	1	4	0	0	0
EABT81	0	0	7	0	0	0	0
EABT810	1	2	1	2	2	0	0
EABT8100	166	319.55	224	133	309.48	107.22	155.91
EABT8101	501.94	795.98	1680.52	1408	608.21	642	523.11
EABT8102	0	7	7	2	4	0	2

EABT8103	2	2	1	0	0	1	0
EABT8104	4	1	0	0	0	0	0
EABT8105	0	1	4	1	0	0	0
EABT8106	1	0	2	3	0	0	0
EABT8107	1	1	4	0	0	2	1
EABT8108	0	1	7	3	0	0	0
EABT8109	16	18	1	6	8	16	22
EABT811	2	0	6	1	0	0	0
EABT8110	0	2	8	5	1	1	0
EABT8111	4927.98	4000.02	2141.05	4142.1	1738	4400.01	3376.29
EABT8112	3	0	7	2	0	0	0
EABT8113	5988.39	5254.83	716.1	3660.37	1076.42	5307.59	2958.83
EABT8114	1	4	27	2	0	0	0
EABT8115	1401.65	3769.75	862.24	507.71	1075.96	1682.67	1775.24
EABT8116	21	40	43	69	33	22	27
EABT8117	2081.26	1930	673.88	2340.02	1335	479	822.11
EABT8118	1	0	17	1	0	0	0
EABT8119	2	0	6	2	1	5	1
EABT812	0	4	138.99	1	0	1	1
EABT8120	13	22	24.05	7	5	4	1
EABT8121	0	0	5	2	1	1	1
EABT8122	1	2	7	0	0	1	0
EABT8123	61.29	145	465	1388.68	178.01	33	65
EABT8124	2448.17	3864.24	3545.95	4772.1	2637.01	2189.02	2816.08
EABT8125	6	17	22	9	0	21	8
EABT8126	686	1197.81	890	2111.86	912.15	475.48	590.38
EABT8127	0	2	2	2	0	0	0
EABT8128	2	0	0	13	0	0	0
EABT8129	0	6	11	0	0	0	0
EABT813	0	1	5	3	3	0	0
EABT8130	130	303	162	1043.94	86	45	273.21
EABT8131	9405.08	21202.1	11726.5	3511	10671.3	1496	4599.04
EABT8132	3792.5	9904.4	119	230.87	690	918.86	1858.77
EABT8133	0	6	3	3	0	0	1
EABT8134	29	59	100	49	12	13	5
EABT8135	3453.29	4737.27	3505.34	5647.48	2517.83	2354.97	2442
EABT8136	2	0	0	0	1	2	3
EABT8137	0	1	21	9	0	1	0
EABT8138	2	4	20	56	1	1	4
EABT8139	0	0	12	2	3	2	0
EABT814	2	2	10	0	0	1	1
EABT8140	0	18	5	1	0	1	0
EABT8141	4	12	7	2	2	1	4
EABT8142	27	42	7	89	81	3	279
EABT8143	1	2	14	30	10	0	3
EABT8144	0	2	30	2	1	0	2
EABT8145	0	1	3	5	0	1	2
EABT8146	350	502.97	337.82	1490.37	519	175	397.17
EABT8147	5	3	2	2	0	16	0
EABT8148	0	0	7	0	1	0	0
EABT8149	1	1	2	3	1	0	2

EABT815	1	1	19	0	0	0	0
EABT8150	5486.28	6172.12	847.33	1605.89	1413.95	6467.99	5814.97
EABT8151	12	7	7	8	8	0	0
EABT8152	1	1	6	0	0	1	2
EABT8153	0	1	5	2	0	3	4.01
EABT8154	1	3.38	73	1	4	3	3
EABT8155	0	1	7	5	1	0	1
EABT8156	3	7	40	3	0	2	1
EABT8157	0	5	0	0	0	0	0
EABT8158	132	121.04	32	195	276	7	248
EABT8159	3	2	5	1	1	0	0
EABT816	1	2	0	0	0	4	4
EABT8160	0	0	0	0	0	5	3
EABT8161	9	2	0	0	0	51	0
EABT8162	3	4	1	6	0	1	4
EABT8163	0	0	8	0	0	0	0
EABT8164	527.9	952.41	832.2	2289.32	1086.99	177.92	412.31
EABT8165	0	1	5	0	2	0	1
EABT8166	12	12	0	0	1	2	3
EABT8167	3	17	8	3	3	3	1
EABT8168	57	91	286.07	36.01	797.84	78.96	67
EABT8169	2	3	9	1	0	1	0
EABT817	0	4	0	11	2	0	1
EABT8170	4	5	43	4	2	1	2
EABT8171	709.41	958.68	903.94	2053.9	882	690.05	609.32
EABT8172	9	26	7	26	45	24	90
EABT8173	4	5	7	20	21	1	1
EABT8174	4	7	1	3	1	1	0
EABT8175	189	418.76	327	486	312	161	182
EABT8176	0	0	8	0	0	1	1
EABT8177	0	1	0	0	0	0	0
EABT8178	5	4	6	48	11	0	4
EABT8179	0	0	9	0	0	0	2
EABT818	1	2	9	2	1	0	0
EABT8180	3	4	0	0	0	7	0
EABT8181	0	2	2	11	0	1	3
EABT8182	469.01	1041.46	1553.76	4637.71	1571.11	956.38	918.49
EABT8183	2	3	0	0	0	2	1
EABT8184	0	0	6	0	0	0	0
EABT8185	0	6	9	11	3	3	0
EABT8186	0	4	4	6	0	2	0
EABT8187	7	9	0	0	0	0	3
EABT8188	0	2	5	0	0	0	0
EABT8189	1	1	8	6	0	0	0
EABT819	3	3	21	1	0	0	1
EABT8190	15	24	4	6	10	19	52
EABT8191	344.81	463	529.21	873.87	203	198	212
EABT8192	2	0	5	0	0	0	0
EABT8193	15	12	44	99	7	0	3
EABT8194	1	2	3	3	1	0	15
EABT8195	0	0	4	2	0	0	0

EABT8196	0	8	5	13	4	2	1
EABT8197	0	0	13	9	0	0	0
EABT8198	908.16	1179	810	4026.79	731	652.99	649.96
EABT8199	13	21	49	40	8	17	18
EABT82	33	58	99	2526	72	23	101
EABT820	52	52	8	14	18	61	19
EABT8200	0	0	0	3	1	0	0
EABT8201	1	4	3	3	2	4	0
EABT8202	1	4	7	5	1	6	2
EABT8203	3	1	51.99	4	15	1	1
EABT8204	0	3	6	9	5	0	1
EABT8205	22668	17126	871.03	11266.9	6158.96	451	503
EABT8206	2	1	2	2	0	0	0
EABT8207	0	13	16	30	0	0	0
EABT8208	0	2	15	6	0	3	0
EABT8209	1	2	3	0	0	0	1
EABT821	0	2	6	1	0	1	0
EABT8210	0	4	8	1	3	0	0
EABT8211	0	1	11	0	1	0	0
EABT8212	1212.04	1927.3	2913.48	4086.02	1502.94	1087.01	1182.91
EABT8213	8	5	0	3	0	0	0
EABT8214	1	2	23.01	11	2	0	0
EABT8215	2	2	4	6	5	2	2
EABT8216	2210.95	3911.45	4604.83	5846.25	2903.09	1684.61	2893.62
EABT8217	190	337.1	584.34	4145.9	443.3	24	86.55
EABT8218	4695	7460.25	24214.1	17230.5	6732.04	6259.28	3726
EABT8219	3	1.99	83.98	0	1	0	2
EABT822	17	44	41	25	4	8	2
EABT8220	31	33	158	39	18	4	5
EABT8221	2	0	15	21	2	0	0
EABT8222	318.41	392	395.96	437.62	388	352	278.65
EABT8223	0	0	0	0	0	0	0
EABT8224	0	1	17	6	0	8	6
EABT8225	2	7	51	4	5	0	12
EABT8226	22	54	35	13	14	2	1
EABT8227	0	0	1	2	1	0	0
EABT8228	0	0	24	0	0	0	0
EABT8229	439.99	497.88	677.86	752.32	345.01	191.66	294.35
EABT823	2	5	5	4	1	0	0
EABT8230	294.02	480.44	202.01	468.52	402.84	144	632.97
EABT8231	903.67	1126.65	1107.71	1937.37	760.52	695.22	737.19
EABT8232	0	0	1	1	1	0	1
EABT8233	0	3	1	0	5	0	4
EABT8234	0	0	11	0	0	0	1
EABT8235	0	2	2	1	0	2	0
EABT8236	0	1	0	0	2	0	0
EABT8237	4	1	121	2	0	0	0
EABT8238	12.02	7	9	41	24	11.62	14
EABT8239	110	193	49	190	113	70	79
EABT824	0	5	4	1	1	0	1
EABT8240	0	0	4	0	0	0	2

EABT8241	0	1	3	1	0	0	0
EABT8242	939.98	2604.93	3642.47	8273.24	1703.9	430	892.01
EABT8243	0	1	6	0	0	0	0
EABT8244	15	0	9	1	0	14	0
EABT8245	0	1	10.01	3	1	0	0
EABT8246	0	0	8	0	0	1	0
EABT8247	0	2	4	0	2	0	0
EABT8248	17	24	43	10	14	53	52
EABT8249	1	3	43	0	1	0	0
EABT825	2	4	4	16	1	0	1
EABT8250	3832.06	3290.3	1684.4	2254.48	2131.01	912.08	1675.51
EABT8251	0	0	41	0	0	0	0
EABT8252	20	45	28	96	11	11	21
EABT8253	0	0	18	2	1	0	0
EABT8254	3	13	1	0	0	0	1
EABT8255	2	0	3	5	2	3	1
EABT8256	2	0	1	1	1	0	1
EABT8257	0	0	14	0	0	0	0
EABT8258	9	3	10	0	0	5	2
EABT8259	809.98	1521.65	597.8	54	515.38	93.07	2384.8
EABT826	618	969	710	1056.92	577.69	416	422.89
EABT8260	1	0	1	13	4	1	18
EABT8261	14766.3	86482.5	96	5	2084.54	22925.3	11360.4
EABT8262	22	30.38	26	23	15	210	96
EABT8263	2	0	0	0	0	0	9
EABT8264	11	12	0	0	2	1	0
EABT8265	4	1	0	2	0	0	5
EABT8266	3	2	3	0	1	0	0
EABT8267	0	2	4	7	14	0	0
EABT8268	849.23	8	2	0	0	336	0
EABT8269	1	1	2	2	3	0	0
EABT827	0	0	0	0	0	0	13
EABT8270	0	1	6	0	0	0	0
EABT8271	1	0	5	2	0	0	0
EABT8272	7	11	1	0	11	11	31
EABT8273	1	0	5	0	0	0	0
EABT8274	0	1	6	0	0	3	0
EABT8275	0	1	1	2	0	0	0
EABT8276	11	382	139	223	367	1	30
EABT8277	2	2	15	32	5	0	1
EABT8278	1	4	187	4	1	0	1
EABT8279	2	0	0	1	0	0	0
EABT828	9	10	5	1	0	20	3
EABT8280	2	1.01	3	3	1	3	2
EABT8281	12	23	11	36	23	4	16
EABT8282	533.01	725	485	1163.69	527	573.02	683.98
EABT8283	2	12	7	39.96	0	3	1
EABT8284	1	5	0	1	1	0	0
EABT8285	160	213.1	95	257	140	234	126
EABT8286	212	525	955.29	163.84	300	31	88
EABT8287	2	2	2	1	0	4	0

EABT8288	2	3	1	0	0	10	0
EABT8289	0	3	2	8	2	0	1
EABT829	1357.11	2403.43	1721.38	4093.85	890.99	985	1086.05
EABT8290	1	1	0	0	0	2	1
EABT8291	0	1	6	0	0	0	0
EABT8292	272	466.72	629.82	843.54	526.91	274.96	261.08
EABT8293	0	0	5	3	0	0	0
EABT8294	254.25	682	1119	253	751	44	121.02
EABT8295	0	0	1	0	1	0	0
EABT8296	799.01	1139.69	600	1238.64	820.88	771.8	984
EABT8297	1	3	0	44	1	2	9
EABT8298	0	1	3	4	0	1	1
EABT8299	0	0	2	1	4	0	0
EABT83	5	6	30	199.01	88	2	10.99
EABT830	11	28	98	1482.55	9	5	7
EABT8300	0	0	9	4	1	0	0
EABT8301	703.19	1219.71	630.71	1536.51	925.34	302.18	545.66
EABT8302	2	0	3	0	7	0	0
EABT8303	3	4	59.08	26	2	0	1
EABT8304	0	0	7	6	0	0	0
EABT8305	1	2	4	3	1	0	0
EABT8306	0	2	3	4	2	0	1
EABT8307	5	7	107	36	1	15	2
EABT8308	1045	1640.32	3618.37	2445.82	1450.01	1001.77	1244.47
EABT8309	110	546.56	204	419.53	400.96	10	23
EABT831	0	0	8	0	1	0	0
EABT8310	0	0	0	2	3	0	3
EABT8311	1	3	6	6	0	2	2
EABT8312	1	3	79	0	1	2	0
EABT8313	0	2	4	2	0	1	0
EABT8314	0	0	4	1	0	0	0
EABT8315	955.16	1190.36	1280.86	2000.14	932.57	601.64	652.23
EABT8316	0	0	5	2	0	0	0
EABT8317	3339.54	4481.65	4696.06	7354.51	3670.32	2429.79	3755.97
EABT8318	0	1	4	1	0	0	0
EABT8319	0	0	5	0	0	0	0
EABT832	1	4	6	1	0	1	0
EABT8320	5	8	17	14	12	5	10
EABT8321	1	0	1	1	0	0	0
EABT8322	1	2	0	2	0	0	0
EABT8323	1151.96	1518	1588.99	2449.01	903.94	926	791.98
EABT8324	5845.96	7746.17	6945.62	4514.19	4002.4	5448	5045.28
EABT8325	8	13	3	1	5	0	5
EABT8326	4	5	34	23	168	0	0
EABT8327	2	1	9	1	0	1	0
EABT8328	2	3	2	0	0	0	0
EABT8329	24	48	74	63	6	15	31
EABT833	4	13	41	4	5	8	0
EABT8330	13	46	4	1	9	12	56
EABT8331	0	0	6	0	0	0	0
EABT8332	2	1	6	21	6	0	3

EABT8333	0	2	0	1	0	0	1
EABT8334	5	4	5	1	0	0	0
EABT8335	1	0	4	1	0	0	0
EABT8336	0	0	7	1	2	1	0
EABT8337	4	11	1	9	4	1	2
EABT8338	1	0	49	0	0	0	2
EABT8339	1	1	3	1	0	0	0
EABT834	2	3	11	5	1	2	0
EABT8340	2460.57	11132.1	3944.54	2333.53	3855.66	921.1	1414.81
EABT8341	0	1	4	1	0	0	0
EABT8342	30	13	3	0	3	0	0
EABT8343	2	1	0	0	0	10	0
EABT8344	3	11	24	13	8	0	20
EABT8345	0	1	12	0	0	0	0
EABT8346	15	0	0	0	2	3	13
EABT8347	2	2	4	2	0	1	0
EABT8348	0	1	2	0	0	0	0
EABT8349	23	75	54	87	84	2	79
EABT835	0	2	1	4	0	0	1
EABT8350	0	0	5	0	0	1	0
EABT8351	1	6	0	0	0	2	0
EABT8352	8	38	16	9	47	1	3
EABT8353	7794.22	19656.5	14700.1	30870	5939.15	5	284.93
EABT8354	1	1	17	4	0	2	1
EABT8355	0	0	1	2	1	0	0
EABT8356	2	2	5	2	1	1	1
EABT8357	0	2	8	0	0	2	3
EABT8358	1755.85	2304.02	1725.24	3991.64	1341.88	1396.9	1310.75
EABT8359	260	321	397	609	348	238	260
EABT836	89	171	95.06	293.02	151	46	67
EABT8360	3	1	12	1	0	2	1
EABT8361	0	1	13	0	0	2	0
EABT8362	0	3	17	1	0	0	0
EABT8363	0	1	11	0	0	0	1
EABT8364	1527	2167.57	1932.96	5590.23	3981.77	1501	1343.1
EABT8365	1	1	1	2	2	1	0
EABT8366	0	2	13	1	0	2	1
EABT8367	7	5	0	0	8	84	40
EABT8368	3825.66	4630.62	4939.83	4922.24	2942.92	3906.34	3255
EABT8369	1	3	5	0	1	0	0
EABT837	1	2	8	1	0	0	4
EABT8370	24	18	8	7	7	3	1
EABT8371	0	0	10	0	0	0	0
EABT8372	2	5	12	13	0	5	1
EABT8373	32	45	27	66	23	7	9
EABT8374	0	3	1	5	4	0	0
EABT8375	1	0	4	2	0	0	0
EABT8376	3	0	3	0	2	0	0
EABT8377	0	1	33	1	0	0	0
EABT8378	2	10	7	28	2	3	5
EABT8379	1	2	4	8	0	2	1

EABT838	2	3	29	4	0	0	0
EABT8380	1	0	11	1	0	0	0
EABT8381	1	2	22	2	1	0	0
EABT8382	4	12	24	40	34	4	1
EABT8383	0	2	0	46	0	0	4
EABT8384	0	1	12	0	0	0	0
EABT8385	1495	2016.21	1607	3780.92	1242	870	1156
EABT8386	0	3	0	8	0	2	0
EABT8387	696.23	1046.02	714.2	1207.58	820.77	523.71	589
EABT8388	16	25	30	16	2	1	1
EABT8389	316.13	542.38	296.45	861.61	330.85	251.16	225.46
EABT839	0	0	0	0	0	0	0
EABT8390	5	4	12	8	3	4	4
EABT8391	2	7	1	0	2	0	1
EABT8392	0	0	5	6	0	0	0
EABT8393	0	1	6	5	7	0	1
EABT8394	2	0	3	16	2	0	0
EABT8395	0	4	8	9	2	0	1
EABT8396	0	1	4	0	0	0	0
EABT8397	0	3	8	1	0	1	0
EABT8398	8	3	36	4	3	11	8
EABT8399	500	50	15	40	39	0	6
EABT84	0	1	2	2	1	0	0
EABT840	2	6	8	26	2	1	0
EABT8400	6	7	149	3	0	3	4
EABT8401	0	0	0	5	0	0	1
EABT8402	0	2	1	8	1	0	0
EABT8403	0	4	7	2	0	0	2
EABT8404	966.41	1519.37	823.94	1289.57	973.95	914.02	1120.09
EABT8405	4	9	0	2	5	0	1
EABT8406	27	39	155	2	4	17	9
EABT8407	9	6	4	2	3	3	6
EABT8408	3	12	14	1	1	1	1
EABT8409	664	23	293.83	1157	6689.23	65	161
EABT841	89	223	178.95	92	131	146.33	433.99
EABT8410	0	0	6	2	1	1	2
EABT8411	2	0	51	1	2	6	2
EABT8412	81	73	17	81	51	52	85
EABT8413	0	1	5	2	1	0	0
EABT8414	2	2	1	9	1	0	1
EABT8415	3	2	0	0	0	0	0
EABT8416	1	2	3	8	1	0	1
EABT8417	15	20	13	40	17	1	2
EABT8418	430	790	796.06	1607	801.47	320	335
EABT8419	14	10	5	1	0	0	1
EABT842	0	3	1	1	0	5	0
EABT8420	5	1	10	6	4	1	7
EABT8421	5	8	25	10	10	20	42
EABT8422	2	1	1	3	2	0	3
EABT8423	0	0	10	0	0	0	0
EABT8424	0	2	12	0	0	0	0

EABT8425	4	14	3	42	0	1	3
EABT8426	17	20	2	1	3	5	0
EABT8427	49	139.98	108.35	246.53	52	37	49
EABT8428	2	3	22	0	0	1	0
EABT8429	0	1	0	0	1	3	0
EABT843	4	7	45	17	4	3	2
EABT8430	3	2	4	21	1	0	0
EABT8431	1	0	17	1	0	0	0
EABT8432	274.08	678.99	557.37	952.15	841.1	220.18	457.97
EABT8433	0	0	7	0	0	0	1
EABT8434	34	82	174	54.06	86	1	29
EABT8435	0	1	1	5	1	0	0
EABT8436	0	2	1	6	1	0	2
EABT8437	0	0	3	0	1	0	3
EABT8438	6	11	4	0	0	20	2
EABT8439	0	1	9	0	0	0	1
EABT844	1	4	6	10	6	1	17
EABT8440	0	2	9	2	0	0	0
EABT8441	27.92	94.99	474.56	250	106	19	35
EABT8442	0	3	13	0	0	1	1
EABT8443	851.36	1150.25	829.02	1618.84	2607.14	1144.2	962.63
EABT8444	0	2	12	5	1	0	0
EABT8445	7	18.26	74	388.97	15	2	50
EABT8446	1	4	0	4	0	0	0
EABT8447	0	3	3	2	0	0	2
EABT8448	0	0	1	7	0	0	0
EABT8449	7	7	5	10	5	7	7
EABT845	0	0	3.97	3	1	0	2
EABT8450	4	29.99	23	68	52	4	28
EABT8451	3	7	13	9	0	1	1
EABT8452	2	4	0	0	0	0	1
EABT8453	1	3	32	3	0	3	1
EABT8454	0	0	0	3	3	0	1
EABT8455	1	3	19	2	2	0	2
EABT8456	6	14	5	10	6	2	4
EABT8457	3	0	227	13	3	3	7
EABT8458	1	0	3	9	0	0	1
EABT8459	3	2	22	6	2	6	5
EABT846	0	0	0	0	2	1	2
EABT8460	3031.09	4726.24	2451.06	5241.95	2184.03	2018.16	2376
EABT8461	3	13	40	2	0	1	1
EABT8462	0	2	2	1	4	0	0
EABT8463	8	7	9	64	8	5	2
EABT8464	182.01	9	0	0	4	217.93	2
EABT8465	10	16	14	3	2	2	3
EABT8466	1	0	4	4	0	0	0
EABT8467	1	1	17	0	1	1	1
EABT8468	0	2	67	5	0	1	1
EABT8469	30	9	15	2	48	3	2
EABT847	0	4	3	8	0	0	0
EABT8470	3	2	3	7	0	1	0

EABT8471	0	1	2	2	0	0	0
EABT8472	1526	595	17	0	126	127	317
EABT8473	0	0	7	1	0	0	0
EABT8474	0	2	16	0	0	0	0
EABT8475	0	0	0	0	4	0	0
EABT8476	1403.89	8814.86	15636.7	3155.3	8636.31	89.94	1241.6
EABT8477	3	6	10	1	1	0	0
EABT8478	2	6	13	0	1	2	3
EABT8479	0	2	4	0	1	2	1
EABT848	0	1	1	1	0	0	0
EABT8480	0	0	10	4	0	1	0
EABT8481	0	2	4	1	0	1	0
EABT8482	34	107	52	199	107	8	46
EABT8483	553.99	1022.58	1728.72	2674.14	648.95	354.02	307.02
EABT8484	1	6	4	0	0	0	0
EABT8485	7	6	23	13	4.99	8	3
EABT8486	0	3	7	0	0	4	0
EABT8487	970.99	1233.25	1028	1825.26	1495	760.6	929
EABT8488	3	2	1	0	1	3	0
EABT8489	0	0	0	0	9	0	4
EABT849	0	2	19	1	0	0	0
EABT8490	4006.76	4675.25	2100.01	1703.09	2236.66	2704.51	4290.18
EABT8491	2	5	7	0	0	0	4
EABT8492	2	8	8	5	0	2.99	7
EABT8493	2	33	25	0	2	14	1
EABT8494	1	2	2	3	1	0	0
EABT8495	358.08	867	397	188	2787.69	323.17	2375
EABT8496	1083.02	1385.98	837.61	2400.82	1142.45	488.55	842.21
EABT8497	1	18	1	1	14	3	12
EABT8498	0	1	13	0	0	1	1
EABT8499	0	6	0	4	4	0	3
EABT85	760	997.95	870.03	1797.49	1116.03	898	712.42
EABT850	0	1	20	1	1	0	0
EABT8500	0	3	11	2	1	0	0
EABT8501	0	8	27	0	0	0	2
EABT8502	1	2	6	0	2	0	0
EABT8503	0	1	1	14	1	0	1
EABT8504	5	4	0	0	0	0	0
EABT8505	1	1	5	0	1	0	0
EABT8506	1	0	2	0	0	0	0
EABT8507	4349.45	7384.36	5933.7	4942.38	2097.39	1370.27	2268.32
EABT8508	0	0	13	0	0	0	0
EABT8509	15	28	18	28	17	18	11
EABT851	3	6	10	0	6	1	0
EABT8510	0	0	0	2	2	0	0
EABT8511	214	632.43	1323.72	752	1275.88	23	50
EABT8512	3	1	1	1	0	5	0
EABT8513	1	2	4	2	1	0	0
EABT8514	708.99	992.14	1004.97	1472.54	842.84	438.72	409
EABT8515	0	2	13	1	1	1	0
EABT8516	13444.5	11414.7	5924.92	10566.7	8054.87	12244.1	9645.64

EABT8517	28.01	8	1	7	0	4	5
EABT8518	0	1	4	0	0	1	0
EABT8519	2	2	7	0	1	0	0
EABT852	238	387.08	393	505	211	170	135
EABT8520	3	4	26	7	2	2	2
EABT8521	0	2	1	0	0	0	2
EABT8522	2	1	3	0	0	0	1
EABT8523	125.79	42	1	0	0	79	0
EABT8524	45	54	54	15	0	0	0
EABT8525	0	4	13	1	0	0	0
EABT8526	0	1	0	1	1	0	1
EABT8527	4	15	3	51	2	4	5
EABT8528	3512.96	9783.88	9894.98	8881.13	4658.96	3274.99	5273.87
EABT8529	307.09	325	336	574	110	144	225
EABT853	1	0	15	2	5	0	1
EABT8530	9	26	9	22	18	8	6
EABT8531	5	4	8	10	3	1	5
EABT8532	2747.5	3412.05	20235.2	6183.99	3294.61	1236	847
EABT8533	27	15	4	7	4	1	1
EABT8534	11	3	3	0	1	0	1
EABT8535	1036.29	1478.65	28	13	1051.56	1446.5	4665.89
EABT8536	0	1	1	25	0	0	0
EABT8537	1	1	0	0	3	1	6
EABT8538	0	18	0	1	0	12	1
EABT8539	4	10	18	15	3	1	3
EABT854	7	4	13	0	1	2	2
EABT8540	0	1	3	2	1	0	0
EABT8541	1	5	9	12	2	11	2
EABT8542	1	1	7	2	0	0	1
EABT8543	1	1	9	2	0	0	1
EABT8544	398.23	407	285	967	411	313	390
EABT8545	376.18	498.32	503.73	1054.86	453.29	298.75	296.76
EABT8546	1	2	30	10	1	1	0
EABT8547	0	3	21	6	1	0	0
EABT8548	15124.7	16505.6	12178.8	9625.43	9330.95	11645.2	11668.2
EABT8549	0	0	9	3	0	0	0
EABT855	3782.71	5368.52	2515.62	5021.21	2078.76	3437.18	4344.53
EABT8550	6	6	7	2	1	2	1
EABT8551	0	0	4	0	0	0	0
EABT8552	1979	2483.12	3514.36	2870.89	1338	1807.69	1550.98
EABT8553	1	11	17	24	0	0	0
EABT8554	3	1	9	1	11	0	10
EABT8555	0	4	17	7	4	4	7
EABT8556	0	2	4	4	2	1	1
EABT8557	1	3	8	10	1	3	1
EABT8558	3	2	6	2	0	3	2
EABT8559	616.98	1390.02	1596.6	1982	756.97	181.05	745
EABT856	1	1	2	5	0	1	1
EABT8560	9	25	47	45	7	13	12
EABT8561	1	6	2	5	0	1	0
EABT8562	0	0	0	5	1	0	0

EABT8563	1	0	2	4	0	3	0
EABT8564	0	1	46	106	5	1	0
EABT8565	0	5	9	1	0	0	0
EABT8566	1677.83	2681.92	1368.8	3368.74	1958.74	1045.43	1107.81
EABT8567	0	0	5	1	0	0	0
EABT8568	1	4	14	6	2	0	1
EABT8569	418	1136.72	3648.95	8995.37	1753.87	18	231
EABT857	0	0	1	4	1	0	0
EABT8570	6	4	0	6	2	2	3
EABT8571	1	0	1	1	0	23	1
EABT8572	1	1	3	6	5	0	0
EABT8573	953.08	1513.51	3875.88	3558.57	1428.93	1047.01	1231.75
EABT8574	3	5	11	9	18	3	4
EABT8575	2	8	18	19	1	0	1
EABT8576	0	4	0	0	0	1	2
EABT8577	1	2	4	0	1	1	4
EABT8578	162.61	373.67	369.78	376.48	300	59	150.99
EABT8579	1	0	8	2	0	0	1
EABT858	1722.26	1304.81	1330.08	1874.04	1625.81	1071	712.96
EABT8580	0	2	19	0	0	0	0
EABT8581	0	0	16	2	0	0	0
EABT8582	4	9	9	6	1	1	1
EABT8583	539.94	751	848.16	977.03	641	355	418
EABT8584	1	10	1	0	0	2	0
EABT8585	2	2	4	2	0	11	5
EABT8586	892.87	1038.43	1041.59	1669.47	650.6	640.14	806.21
EABT8587	0	2	6	3	0	3	1
EABT8588	0	4	15	5	0	0	1
EABT8589	0	0	1	0	0	0	0
EABT859	0	4	1	0	0	0	0
EABT8590	0	0	6	0	0	0	0
EABT8591	4	14	0	0	0	0	0
EABT8592	0	2	9	1	0	0	0
EABT8593	0	3	30	11	1	0	1
EABT8594	0	5	2	9	5	0	1
EABT8595	113	168.62	172.89	1004.58	62	60	114
EABT8596	832.97	1282	1468.21	5706.83	922.14	529.38	600.55
EABT8597	0	0	6	1	0	0	0
EABT8598	0	1	6	0	2	2	0
EABT8599	0	0	9	1	4	1	1
EABT86	1	0	4	1	0	0	0
EABT860	44	70	100	105	17	18	20
EABT8600	3	7	54	7	5	2	3
EABT8601	0	3	19	5	0	0	1
EABT8602	1104.78	2067.05	2219.6	5413.09	2158.58	829.82	969.8
EABT8603	1	3	4	0	0	2	1
EABT8604	2	3	1	2	0	0	0
EABT8605	0	0	0	0	3	0	0
EABT8606	3	3	0	0	1	3	4
EABT8607	9	2	3	3	0	4	0
EABT8608	67880.2	85426	821.69	1771.92	188.26	1738.85	1954.43

EABT8609	1159	1108.9	749.45	1287.33	693	453.99	790
EABT861	32	19	57	1197.93	35	44	70
EABT8610	1	2	20	2	1	0	0
EABT8611	5	1	5	2	0	1	0
EABT8612	3	1	2	3	0	0	1
EABT8613	6	9	8	11	2	1	6
EABT8614	2050.88	3157.14	3437.59	3860.35	1888.64	2085.74	1757.6
EABT8615	7	20	25.01	29	10	3	23
EABT8616	127	214	202.92	575.95	543.8	137	150
EABT8617	0	0	0	1	2	0	0
EABT8618	0	1	7	0	0	0	0
EABT8619	0	0	6	3	0	0	0
EABT862	0	0	7	0	2	0	0
EABT8620	0	1	6	1	0	0	0
EABT8621	4	4	8	4	5	3	9
EABT8622	1	0	2	0	0	0	0
EABT8623	1	0	9	0	1	0	0
EABT8624	0	0	0	1	6	3	14
EABT8625	60	86.09	170.98	128	52.52	2	28
EABT8626	3	7	5.01	0	0	0	0
EABT8627	9	13	69	58	6	21	12
EABT8628	8	22	24	9	12	21	262
EABT8629	3	8	15	0	1	1	0
EABT863	0	0	3	1	0	0	0
EABT8630	822.03	1043	757	1410.95	830.99	807	676.38
EABT8631	0	5	0	10	0	0	4
EABT8632	1	2	6	0	0	0	0
EABT8633	0	0	8	1	0	0	0
EABT8634	1	1	10	0	0	0	1
EABT8635	2	2	6	0	0	1	0
EABT8636	0	1	15	2	0	0	0
EABT8637	0	1	1	0	11	0	1
EABT8638	0	2	8	0	0	0	1
EABT8639	906.01	1498.02	2359.61	3389.62	858	699	844.56
EABT864	0	0	10	0	0	0	0
EABT8640	0	2	0	6	2	1	0
EABT8641	1	9	7	2	0	1	1
EABT8642	0	0	14	3	0	2	1
EABT8643	2257.01	2995.15	1597	683	2080.7	445	304.03
EABT8644	5	34	78.4	27	0	1	1
EABT8645	0	0	1	13	3	0	0
EABT8646	62.97	267	264	1084.99	639	81	83.54
EABT8647	6482.98	6038.62	3800.24	4906.81	4050.18	4568.72	4567
EABT8648	0	0	2	4	0	0	0
EABT8649	1	4	4	3	0	1	1
EABT865	0	0	4.71	0	0	0	0
EABT8650	750.09	885.25	1501.82	1817.47	740	639.39	557
EABT8651	0	3	49	1	0	0	0
EABT8652	1497	2198.39	1729	1241	2390.83	1000	1458.28
EABT8653	162.17	165.09	401.04	427.81	227.01	164.01	155.96
EABT8654	2181.21	2335.7	2185.4	3526.91	2237.44	1749.86	1438.86

EABT8655	0	8	0	1	2.99	4	1
EABT8656	2	1	0	0	0	13	0
EABT8657	0	1	5	2	0	0	0
EABT8658	1	5	13	0	1	0	2
EABT8659	21	47	31	53	11	2	41
EABT866	15	17	25	21	6	12	8
EABT8660	1	0	6	0	0	0	1
EABT8661	2	2	2	1	0	0	0
EABT8662	3	12	23	2	4	1	0
EABT8663	2794.55	4247.44	3597.69	5011.52	3672.75	3477.9	2224.95
EABT8664	0	5	1	1	0	2	0
EABT8665	0	0	9	0	0	0	0
EABT8666	6104.91	9781.11	7022.44	3030.9	2422.24	8967.44	3637.8
EABT8667	0	0	4	1	0	1	0
EABT8668	6	7	52	4	156.43	3	2
EABT8669	0	2	21	18	12	1	26
EABT867	15	6	12	21	3	2	15
EABT8670	0	0	4	0	0	0	1
EABT8671	1084.05	914.92	1029.03	113.01	12	2086.24	10
EABT8672	1129	1971	1668.11	2457	1386.88	829	1079.55
EABT8673	4	2	1	0	0	5	1
EABT8674	2	3	15	0	0	4	2
EABT8675	2	5	7	3	5	0	1
EABT8676	0	4	0	1	1	1	1
EABT8677	0	0	30	1	1	0	0
EABT8678	1183.19	1914.34	1153.92	1728.08	918.36	808.03	832
EABT8679	13	1	5	12	0	0	2
EABT868	0	0	0	3	2	0	1
EABT8680	7917.2	13198.4	70172.6	15625.5	9363	7342.83	5233.97
EABT8681	7	8	5	20	47	0	1
EABT8682	0	9	9	15	0	0	2
EABT8683	4	14	4	9	0	0	0
EABT8684	2	16	1	0	0	0	0
EABT8685	121	16	23	160	36	6	6
EABT8686	1	0	15	1	1	0	2
EABT8687	6	8	0	0	0	1	0
EABT8688	2	17	30	71	30	0	0
EABT8689	1276.75	2138.19	2480.19	3109.11	1531.13	1071.77	1152.05
EABT869	0	0	1	7	0	0	0
EABT8690	374.07	495.58	609.88	886.54	349.41	292.63	295.88
EABT8691	1	1	1	0	1	0	11
EABT8692	1	0	10	8	1	3	3
EABT8693	0	0	0	2	0	0	1
EABT8694	12	22	12	7	4	12	2
EABT8695	1	2	6	20	4	0	0
EABT8696	19	13	44	15	9	17	79.19
EABT8697	151.02	317.15	405.89	718.19	600.12	93	212.14
EABT8698	200	348.65	472.4	410	318.38	177	287
EABT8699	0	4	0	1	0	2	2
EABT87	4	3	7	11	1	0	0
EABT870	0	1	10	0	0	0	1

EABT8700	1	8	4	20	0	4	0
EABT8701	5	4	7	12	2	10	3
EABT8702	0	2	3	21	0	0	0
EABT8703	1	4	0	6	2	2	7
EABT8704	0	0	3.99	1	0	1	8
EABT8705	1050.86	1520.66	1353.69	2972.25	1347.12	784.91	1049.16
EABT8706	1	10	17	0	0	0	4
EABT8707	3665.01	6240.07	1636.1	4308.99	5144.01	704	14854.2
EABT8708	1	0	0	0	0	3	1
EABT8709	0	0	5	1	0	0	0
EABT871	20	41	25	11	13	8	5
EABT8710	0	1	2	103.75	1	7	16
EABT8711	0	2	21	11	0	1	0
EABT8712	1	3	1	0	6	0	1
EABT8713	2	0	1	0	1	0	1
EABT8714	8	46	93	26	115	4	17
EABT8715	1	7	1	4	0	0	0
EABT8716	1	0	56	1	1	1	0
EABT8717	0	0	0	5	4	0	1
EABT8718	0	0	7	2	0	0	0
EABT8719	1	6	10	20	5	6	2
EABT872	0	0	52	0	1	1	3
EABT8720	1	4	43	3	0	0	0
EABT8721	6	7	14	39	36.6	6	5
EABT8722	0	1	4	1	7	0	2
EABT8723	0	1	2	2	2	0	1
EABT8724	26	83	103	261.98	198	6	11
EABT8725	0	0	1	1	0	1	2
EABT8726	2	5	17	8	2	1	1
EABT8727	1	4	17	7	0	1	4
EABT8728	0	1	7	0	0	0	0
EABT8729	4	0	0	1	1	0	13
EABT873	2	3	18	1	2	2	0
EABT8730	719.92	394.98	105.99	42	512.76	2613.61	231
EABT8731	232.85	290	319	432	394	183	146
EABT8732	0	1	5	24	3	0	0
EABT8733	0	1	4	3	11	0	2
EABT8734	4	2	1	0	1	1	6
EABT8735	4	3	12	4	3	2	1
EABT8736	3	5	4	1	0	3	0
EABT8737	53.23	95	63	36.21	90.94	43.79	58.88
EABT8738	1	2	5	0	5	0	5
EABT8739	4	13	29	15	3	2	8
EABT874	1	11	9	0	0	0	0
EABT8740	17	11	4	0	10	16	0
EABT8741	0	0	8	2	0	0	0
EABT8742	0	1	8	1	0	0	0
EABT8743	0	3	2	2	0	0	0
EABT8744	0	0	5	0	0	0	0
EABT8745	1697	2134.98	1314	2914.11	1622.06	1062.2	1316.68
EABT8746	55	42	34	36	66	3	15

EABT8747	3	17	38	30	10	4.01	31
EABT8748	0	0	1	0	6	0	1
EABT8749	0	0	19	2	1	1	2
EABT875	1	2	3	3	2	0	0
EABT8750	461.25	571.95	352.38	1011.11	434.98	150.1	176.07
EABT8751	208	261	184	1723.76	182	11	346
EABT8752	335.31	440	428.26	817.99	337	247.98	302
EABT8753	32	7	2	1	0	119	1
EABT8754	1	2	8	1	0	0	1
EABT8755	9	7	39	13	26	2	13
EABT8756	1	4	18.42	5	3	2	4
EABT8757	1	6	2	0	0	0	1
EABT8758	0	1	13	3	0	0	0
EABT8759	0	1	3	2	0	0	0
EABT876	1	4	30	9	0	0	1
EABT8760	31	12	7	0	0	0	0
EABT8761	0	0	5	1	0	0	0
EABT8762	0	0	5	6	0	0	0
EABT8763	478	1593.29	5109.03	3348.28	933.32	360.02	845.99
EABT8764	1	1	5	0	0	0	0
EABT8765	1	2	2	1	1	1	0
EABT8766	366	193	49	32	131	17	93
EABT8767	1	1	0	2	1	0	0
EABT8768	52	55	0	0	1	14.55	30.98
EABT8769	0	4	7	1	1	3	0
EABT877	3656.57	5420.1	599.3	664.01	1460.06	8289.77	4960.51
EABT8770	3	1	12.03	2	1	0	0
EABT8771	0	1	0	1	0	0	0
EABT8772	16	37	17	243	89	3	2
EABT8773	18	16	51	18	22	0	1
EABT8774	3	0	23	8	16	5	1
EABT8775	3	2	1	3	0	1	0
EABT8776	3	0	0	0	0	6	0
EABT8777	4	4	0	4	0	1	0
EABT8778	1	3	218	16	12	2	0
EABT8779	6	5	7	2	0	3	4
EABT878	0	0	5	1	0	0	0
EABT8780	163	226	101	374	52	290	110
EABT8781	1042.6	1192.61	738.97	1169.33	900.23	754.88	643.33
EABT8782	1380.51	1897.99	1339.05	1897.44	1402.36	1132.98	1486.13
EABT8783	1	4	4	2	4	0	2
EABT8784	0	0	5	0	0	0	0
EABT8785	2	3	3	0	1	2	3
EABT8786	2	3	24	4.77	1	3	2
EABT8787	1	2	4	1	0	0	0
EABT8788	1	2	1	0	0	1	0
EABT8789	3	3	2	31	0	0	0
EABT879	10	9	38	20	7	2	8
EABT8790	0	3	6	1	0	0	1
EABT8791	17	15	4	0	1	13	6
EABT8792	0	0	0	12	0	0	1

EABT8793	2	13	3	5	1	0	0
EABT8794	0	2	7	1	0	0	0
EABT8795	1	1	8	1	0	1	0
EABT8796	241.01	337.95	233	723.75	364.24	139.31	292.26
EABT8797	0	1	6	13	7	0	3
EABT8798	1	2	0	6	0	3	0
EABT8799	232.02	267.08	149.99	516.94	262	192	250.17
EABT88	0	0	2	2	9	0	0
EABT880	0	1	2	0	2	1	0
EABT8800	1	3	10	6	5	0	2
EABT8801	23	25	26	9	9	5	5
EABT8802	1	2	19.07	1	1	0	0
EABT8803	274.04	442	240	1194.89	575.16	213	338
EABT8804	610.59	823	1097.98	1697	890.08	287.1	412.06
EABT8805	0	2	1	0	2	0	0
EABT8806	0	2	5	0	0	0	1
EABT8807	3	14	10	27	2	5	6
EABT8808	4871.74	5378.25	4643.37	7955.07	3786.17	5117.32	4162.42
EABT8809	0	1	3	4	0	2	0
EABT881	0	0	4	10	0	0	0
EABT8810	6351.72	12249.1	24148.4	4317.1	11648.2	4064.78	8859.54
EABT8811	0	0	0	0	0	0	7
EABT8812	0	0	2	0	0	4	0
EABT8813	0	0	51	2	0	0	0
EABT8814	317	1219	1132	1256.98	1549.13	89.69	350.01
EABT8815	1819.01	2803.93	8696.9	2013.16	1599.55	1709.89	1169.31
EABT8816	2	0	33	1	3	0	1
EABT8817	0	0	3	1	1	1	1
EABT8818	795.4	1250.05	1318.54	5981.81	1336.98	540.29	893.63
EABT8819	1	0	4	0	0	0	0
EABT882	11	18	47	25	10	9	12
EABT8820	0	0	6	1	0	0	0
EABT8821	0	1	1	4	0	0	0
EABT8822	4	6	8	4	1	1	3
EABT8823	2327.54	2841.57	2961.13	4483.81	1825.96	2084.99	1306.93
EABT8824	1351.08	2022.84	1336.35	3163	1516	1109	1148.66
EABT8825	0	3	24	1	0	0	1
EABT8826	0	1	7	0	4	0	0
EABT8827	0	0	13	0	0	1	0
EABT8828	3221.85	5566.63	10759.7	12263.9	3500.18	2325.27	2787.12
EABT8829	0	2	1	45	0	0	0
EABT883	4	13	13	2	2	0	0
EABT8830	0	0	11	1	0	0	0
EABT8831	0	2	2	0	1	0	0
EABT8832	0	2	5	0	0	0	0
EABT8833	6	11	21	1	6	17	10
EABT8834	6	6	38	15	14	0	0
EABT8835	10	8	0	18	2	2	1
EABT8836	2	6	1	8	0	2	1
EABT8837	0	4	5	2	0	1	2
EABT8838	5	5	1	0	4	4	7

EABT8839	0	3	2	8	2	0	0
EABT884	0	0	0	1	1	0	0
EABT8840	5	2	6	4	1	3	2
EABT8841	0	4	7	4	2	2	1
EABT8842	1	1	3	7	1	2	1
EABT8843	3	1	5	1	0	0	0
EABT8844	517.09	536.01	938.36	1769.02	733	451.35	509.78
EABT8845	13	13	8	14	45	1	4
EABT8846	7838.32	6943.12	865.99	436	1849	9936.03	9044.39
EABT8847	871.11	1428.38	2262.72	2132.68	1496.01	1042.25	818.92
EABT8848	1058.04	3239.02	8000.02	5264.34	8628	678.33	3134.24
EABT8849	0	7	2	0	0	5	1
EABT885	21	76	17	9	14	0	1
EABT8850	1	0	4	21	0	1	2
EABT8851	5	9	0	0	1	30	18
EABT8852	10146.7	10134.1	7237.65	6802.58	16050.7	6987.03	6621.82
EABT8853	1	4	28	3	1	0	0
EABT8854	3	20	16	14	0	1	0
EABT8855	0	1	17	1	2	1	0
EABT8856	1	1	8	0	0	0	1
EABT8857	0	1	14	0	3	0	0
EABT8858	2	4	9	137	17	0	4
EABT8859	1036.97	1392.28	804.8	2058.43	1216.8	927.99	917.65
EABT886	0	4	0	0	1	0	2
EABT8860	2	1	10	0	0	0	0
EABT8861	0	0	6	1	1	0	0
EABT8862	0	1	15	2	0	0	2
EABT8863	9	29	35	22	15	19	18
EABT8864	0	0	0	0	0	18	1.06
EABT8865	15017.9	28344.7	9442.81	3916.83	13403	26442.9	19626.7
EABT8866	1	2	27	0	0	2	0
EABT8867	4	6	8	2	0	0	0
EABT8868	3	13	13	35	0	2	0
EABT8869	347	798.91	3085.63	1416.83	90.37	180.01	182
EABT887	4	6	35	14	0	5	1
EABT8870	3	12	8	6	7	76	8
EABT8871	0	1	4	0	0	0	0
EABT8872	1238.47	1964.6	1108.13	462	1225.85	76	1090.74
EABT8873	0	0	3	2	0	0	0
EABT8874	0	1	4	0	0	0	1
EABT8875	0	1	9	20	0	0	0
EABT8876	0	4	3	0	1	0	1
EABT8877	8	7	30	17	12	12.4	11
EABT8878	3	1	3	1	0	0	0
EABT8879	39	22	3	5	0	6	14
EABT888	1	0	6	0	0	0	0
EABT8880	0	0	12	1	1	0	0
EABT8881	0	3	9	11	1	1	0
EABT8882	1	0	6	0	0	0	0
EABT8883	440	679	347	1138.27	815.01	541.93	420.88
EABT8884	0	0	8	3	0	0	0

EABT8885	0	1	59	2	0	0	0
EABT8886	5	8	0	0	4	10	65
EABT8887	7	7	12	15	5	1	7
EABT8888	4	0	0	0	0	2	0
EABT8889	13.03	2	6	13	6	0	0
EABT889	2	2	2	1	0	0	0
EABT8890	0	0	3	1	0	1	0
EABT8891	0	0	6	0	0	0	1
EABT8892	0	2	2	1	0	2	0
EABT8893	0	0	19	0	0	0	0
EABT8894	1	2	2	0	0	0	0
EABT8895	3	6	46	14	0	9	5
EABT8896	2	4	19	12	2	0	0
EABT8897	0	3	9	3	17	0	0
EABT8898	1	0	16	0	0	0	2
EABT8899	0	0	0	3	1	0	0
EABT89	0	0	7	3	1	0	0
EABT890	13	3	3	0	0	9	0
EABT8900	7	12	48	17	1	0	4
EABT8901	1	4	14	11	1	2	3
EABT8902	14	42	48	56	1	13	2.85
EABT8903	1	3	4	2	0	1	0
EABT8904	1096.27	1913.8	2063.88	3427.18	1372.73	1000.79	896.64
EABT8905	2	0	3	0	0	0	0
EABT8906	0	0	4	3	0	0	0
EABT8907	44	87	596.66	110.96	11	28	20.72
EABT8908	0	0	10	0	0	0	0
EABT8909	1020.92	2851.51	1379.78	1540.73	2418.84	384.87	1223.7
EABT891	3	2	8	1	0	0	0
EABT8910	0	0	2	0	0	0	0
EABT8911	2	0	7	1	1	0	0
EABT8912	128	181	162.09	170	78	63	78
EABT8913	0	3	4	2	1	2	2
EABT8914	2	2	7	0	0	0	1
EABT8915	8135.08	11125.3	10690	6871.44	8017.9	6	428
EABT8916	378.03	518.37	355.18	920.1	521.91	462.25	368.01
EABT8917	0	2	9	1.14	0	0	1
EABT8918	5	3	5	1	5	3	8
EABT8919	398	419.14	183	643	436	398.13	274.17
EABT892	0	1	4	0	0	2	0
EABT8920	275	803.77	1029	5143.67	401	259	110.13
EABT8921	0	3	105	0	1	1	0
EABT8922	0	3	4	7	4	0	1
EABT8923	2	0	3	1	0	0	0
EABT8924	0	3	7	14	0	1	5
EABT8925	2	2	4	3	0	0	2
EABT8926	6	1	17	7	4	1	0
EABT8927	7	8	0	0	0	0	0
EABT8928	274	567.9	571	293	365.92	122	207
EABT8929	8	13	0	0	4	47	29
EABT893	0	1	1	3	0	0	0

EABT8930	1	0	1	0	1	0	0
EABT8931	2	1	0	0	0	4	0
EABT8932	5070.91	15639.4	2348.14	1411	3603	822.85	4067.01
EABT8933	0	0	0	13	0	0	0
EABT8934	0	1	3	1	0	0	0
EABT8935	8	19	1	17	1	2	3
EABT8936	0	0	32	0	0	0	0
EABT8937	2	18	1	2	0	1	3
EABT8938	1864.01	3226.64	2369.67	4637.96	2900.56	901.6	1375.75
EABT8939	0	3	5	3	4	0	0
EABT894	0	1	4	7	1	0	0
EABT8940	0	3	6	8	3	1	1
EABT8941	1382	1558	1056.65	907	3613.59	931	1002
EABT8942	0	2	3	0	1	1	0
EABT8943	1	0	3	1	0	0	0
EABT8944	4	4	2	2	3	0	0
EABT8945	2	2	5	0	0	0	1
EABT8946	1	1	3	1	0	0	0
EABT8947	1	1	1	0	1	0	0
EABT8948	1	3	4	1	1	0	0
EABT8949	2	4	19	1	2	0	1
EABT895	5	20.98	9	15	4	3	4
EABT8950	0	0	1	0	0	0	0
EABT8951	4	2	1	3	2	0	0
EABT8952	0	4	8	1	1	0	0
EABT8953	4	9	7	12	5	1	8
EABT8954	0	2	2	2	0	0	0
EABT8955	207	301	150	240	187	115	161
EABT8956	4	7	11	6	0	1	0
EABT8957	4	9	16	14	3	3	3
EABT8958	0	1	23	1	0	0	0
EABT8959	6	1	2	0	0	8	0
EABT896	127.72	704.43	59	1038.55	134.1	226.79	254.31
EABT8960	1	2	15	2	1	0	1
EABT8961	0	1	3	0	0	0	0
EABT8962	4	8	0	0	3	14	6
EABT8963	4637.64	5225.37	1677	3030.54	3293	4315.84	3336.26
EABT8964	0	0	6	3	1	0	0
EABT8965	2057.59	3448.27	1997.85	4966.6	2230.19	1595.1	1615.29
EABT8966	264.27	347.15	338.98	310	394.71	185.32	309
EABT8967	3265.12	4470.6	4179.39	6275.56	2551.91	3906.18	3855.52
EABT8968	2	3	1	1	0	1	0
EABT8969	1	0	2	7	49	1	11
EABT897	0	2	6	2	1	0	2
EABT8970	1461.69	1924.9	1869.06	5876.61	1083.42	1077.4	803.81
EABT8971	0	9	0	0	2	0	0
EABT8972	52	78	36	24	43	48	179
EABT8973	421.57	571.61	466.9	804.24	568.87	361.43	417.1
EABT8974	94	94	5	52	1	4	1
EABT8975	0	0	7	0	0	0	0
EABT8976	0	6	5	1	0	5	0

EABT8977	0	0	2	0	4	0	0
EABT8978	4	0	1	0	0	4	0
EABT8979	1	1	7	4	514.07	1	1
EABT898	518.65	216	1430.5	7959.8	836.01	220	307.79
EABT8980	1	3	31	12	7	0	0
EABT8981	0	11	1	0	0	14	1
EABT8982	52	78	39	202	73.82	19.98	55
EABT8983	1254.07	1600.42	4486.92	3311.14	2142.9	1236.69	2095.19
EABT8984	25305.8	23676.7	8175.31	5460.02	9567.06	37073.7	32287.4
EABT8985	3	7	1	2	1	0	1
EABT8986	0	0	11	0	0	0	0
EABT8987	0	2	2	0	2	0	0
EABT8988	1	1	2	9	0	0	0
EABT8989	3	1	5	2	1	0	0
EABT899	12	21	35	62	62	55	9
EABT8990	0	0	3	0	1	0	1
EABT8991	6	59	73	240.99	156	1	5
EABT8992	0	0	13	0	0	0	1
EABT8993	0	0	7	5	1	0	0
EABT8994	2	7	5	3	0	0	2
EABT8995	0	2	2	5	1	0	1
EABT8996	2	5	29	13	5	0	1
EABT8997	43	15	14	51	28	41	25
EABT8998	715.91	976	611	758	713.02	601	584
EABT8999	3197.87	5221.81	7617.98	9809.58	3869.75	2955.44	3027.89
EABT9	0	0	3	3	0	0	0
EABT90	3	4	1	8	4	0	11
EABT900	0	0	11	3	1	1	2
EABT9000	0	0	2	0	0	0	0
EABT9001	540	841	666	1232	613	749	481.99
EABT9002	0	0	5	0	0	0	0
EABT9003	7	12	15	12	4	7	4
EABT9004	5	12	15	10	0	2	0
EABT9005	682	740.72	438	732	474.05	419.92	407.15
EABT9006	1	1	9	0	0	0	0
EABT9007	1	1	49.01	4	0	0	2
EABT9008	1	2	5	2	3	1	0
EABT9009	2	3	14	2	3	0	1
EABT901	0	2	15	1	1	1	2
EABT9010	1	0	10	3	0	0	2
EABT9011	51	62	67	95	54	10	50
EABT9012	0	0	2	1	0	0	0
EABT9013	0	1	11	9	0	0	0
EABT9014	12599.1	13262.8	8069.04	11576.5	10060.5	14761.6	7857.02
EABT9015	1	0	13	1	1	0	0
EABT9016	46.23	34.04	11	10.33	2	90.88	3
EABT9017	9	4	0	0	0	0	0
EABT9018	0	3	0	9	1	0	4
EABT9019	1147.9	1709.31	1062.96	3365.72	1067.81	840.61	863.43
EABT902	0	1	1	1	0	1	1
EABT9020	1	2	4	4	2	1	0

EABT9021	0	0	5	0	0	0	0
EABT9022	1	4	23	0	2	0	0
EABT9023	0	3	9	14	3	0	3
EABT9024	0	3	22	0	0	2	7
EABT9025	0	0	7	0	0	0	0
EABT9026	0	1	2	0	0	0	0
EABT9027	2	2	3	2	3	0	0
EABT9028	2	2	15	1	0	1	1
EABT9029	21	22	331.01	7	34	30	17
EABT903	0	1	8	2	1	0	1
EABT9030	0	0	11	0	0	0	0
EABT9031	0	0	10	0	0	2	0
EABT9032	1	4	6	11	2	3	1
EABT9033	885.91	1310.83	1015	2195.97	724.39	886.95	587.99
EABT9034	2	2	3	10	2	0	0
EABT9035	557	206	2979.54	3079.98	640.01	7	14
EABT9036	0	0	5	1	0	1	0
EABT9037	1	1	7	1	0	1	1
EABT9038	420.03	458.46	430.73	762.03	354.93	380.78	238.84
EABT9039	2408.99	2417.73	1493.98	2522.02	1500.61	637.13	962.01
EABT904	5	8	57	10	2	8	10.01
EABT9040	347.99	472	521.09	1987.64	428	300.02	353.83
EABT9041	469.97	539	528.91	790	528.98	498	396
EABT9042	2523.56	2969	4898.2	3829.99	1857.98	1911	1172.03
EABT9043	19.98	56	5	1	12	3	24
EABT9044	112	196	1050.88	2498.06	1148.29	32	238
EABT9045	0	3	3	0	0	1	2
EABT9046	0	1	5	0	2	1	0
EABT9047	12668	8889.28	4720.79	4880.86	5943.02	13163.2	12089.1
EABT9048	0	0	0	5	8	0	0
EABT9049	0	1	6	5	0	0	0
EABT905	0	0	5	0	1	0	0
EABT9050	394	660.49	1161.2	2219.75	343.28	341	149
EABT9051	1	8	21	1	0	1	0
EABT9052	0	0	9	6	1	0	0
EABT9053	3	14	0	4	2	0	1
EABT9054	0	0	2	7	1	0	0
EABT9055	32.86	80.86	268.74	145	218.21	24	157.99
EABT9056	5684.98	5575.55	2402.79	5316.58	4872.17	8838.99	4512.85
EABT9057	0	2	5	0	0	0	1
EABT9058	0	2	17	0	0	0	0
EABT9059	0	0	4	0	1	1	0
EABT906	0	0	6	1	1	0	0
EABT9060	34	24	84	94	20	19	33
EABT9061	0	0	8	1	0	0	0
EABT9062	8	7	5	0	1	1	0
EABT9063	5	20	19	20.98	16	0	5
EABT9064	13	5	42	3	5	6	6
EABT9065	0	1	9	2	0	1	1
EABT9066	1	1	11	0	1	0	0
EABT9067	6	7	21	0	6	17	0

EABT9068	1	1	0	2	0	0	14
EABT9069	1134.11	2159.95	1647.69	5903.94	1171	651	1185.19
EABT907	0	0	6	6	1	0	0
EABT9070	1	0	10	0	1	2	0
EABT9071	3	4	5	6	3	2	12
EABT9072	1157.16	1901.59	1776.17	3507.84	1636.26	896.88	1235.92
EABT9073	0	3	12	11	0	1	2
EABT9074	2	0	3	7	0	2	0
EABT9075	1	4	5	0	0	1	0
EABT9076	1	0	7	0	1	0	0
EABT9077	0	1	3	1	0	0	0
EABT9078	1	4	5	1	3	1	2
EABT9079	0	0	3	1	4	0	6
EABT908	144	122	183	200	167	129	185
EABT9080	0	0	0	0	0	0	0
EABT9081	6133.31	8854.95	5088.45	7594.24	3927.31	5175.97	6438.43
EABT9082	0	1	0	18	1	1	4
EABT9083	1	6	18	17	5	1	0
EABT9084	1	4	31	2	0	0	0
EABT9085	0	2	4	0	0	20	2
EABT9086	1822.58	2706.85	2186.71	3964.23	1712.33	1386.35	1490
EABT9087	1	4	12	12	2	2	3
EABT9088	0	3	0	0	0	0	0
EABT9089	519	447	307	383	186	249	236
EABT909	1	3	6	0	0	1	3
EABT9090	0	0	0	3	1	0	0
EABT9091	0	3	2	3	0	0	0
EABT9092	0	2	10	2	2	0	0
EABT9093	0	0	4	2	0	0	0
EABT9094	2	2	9	3	0	1	1
EABT9095	188.47	338.28	509.03	489.39	155.17	156.01	163.04
EABT9096	0	3	9	9	2	1	1
EABT9097	8	21	23	14	2	2	1
EABT9098	25	38	48	11.01	2	4	0
EABT9099	0	0	3	0	0	2	0
EABT91	13	15	29	7	14	1	15
EABT910	7	3	3	0	1	0	0
EABT9100	2	5	15	2	0	1	1
EABT9101	0	0	3	0	0	2	0
EABT9102	0	3	3	5	0	0	0
EABT9103	0	0	7	0	1	1	0
EABT9104	87.16	116.88	70.68	157.85	85.03	69.15	60.3
EABT9105	0	0	16	0	0	0	0
EABT9106	609.26	1031.76	530.6	992.24	772.89	355.16	578.32
EABT9107	0	0	0	0	0	0	0
EABT9108	2	2	4	4	1	0	0
EABT9109	2	4	1	0	1	8	0
EABT911	1	0	12	0	2	1.02	0
EABT9110	223	341	254.66	624.96	233.97	88	170.96
EABT9111	3	7	16	1	0	0	3
EABT9112	8	15	3	7	4	0	3

EABT9113	2	5	12	5	2	4	2
EABT9114	0	0	2	0	1	2	2
EABT9115	2	2	1	1	1	0	3
EABT9116	54	231.03	430.62	30682.6	58	8	10
EABT9117	849.87	1062.82	523.12	2347.48	863.66	821.42	836.01
EABT9118	0	6	2	4	0	0	0
EABT9119	0	0	9	0	0	0	0
EABT912	0	0	2	0	0	0	0
EABT9120	1	0	3	1	1	0	1
EABT9121	3	14	30	2	23	1	3
EABT9122	307.73	370.31	503.41	657.93	267	247.01	222
EABT9123	0	1	0	0	1	0	0
EABT9124	2775.56	3321	2551.77	4308.01	2386	3037.34	2470.76
EABT9125	1624.03	2166.83	921	3564	628.53	1141.98	704
EABT9126	0	3	4	10	2	0	0
EABT9127	1	3	6.07	0	1	0	1
EABT9128	0	0	11	1	0	1	0
EABT9129	992.57	1757.39	782.67	2134.34	882.95	753.14	1011.98
EABT913	0	0	2	0	0	0	0
EABT9130	1292.59	1645.17	2352.2	1744.25	1229.62	1954.53	1736.27
EABT9131	1171	1702.97	1064.93	2127.15	1171	971.98	1289
EABT9132	5	15	69	1	1	1	0
EABT9133	0	3.99	2	2	0	0	0
EABT9134	17	26	0	0	2	32	19
EABT9135	7	35.21	46.02	224	8.6	0	1.29
EABT9136	5.98	24	10	91	0	0	0
EABT9137	0	0	2	0	0	0	0
EABT9138	0	5	20	15	9	2	2
EABT9139	9	4	6	0	0	12	0
EABT914	1	1.02	6	1	0	0	1
EABT9140	1	0	97	0	2	0	0
EABT9141	5396.61	5448.49	5075.14	4424.16	4731.04	5681.15	5504.64
EABT9142	171	253	383	1012.04	288.98	82	115.6
EABT9143	4	2	22	12	1	0	0
EABT9144	2	4	2	6	0	1	0
EABT9145	0	2	1	3	2	0	0
EABT9146	1	0	0	2	129	0	0
EABT9147	18	17	0	0	1	2	3
EABT9148	0	2	0	1	3	0	0
EABT9149	3064.82	455	98.66	1	19.97	11223.8	45.21
EABT915	1118.34	1754.1	1311.5	3279.87	1214	732.44	759.9
EABT9150	0	1	4	0	0	1	0
EABT9151	16	34	110	213.99	4146.99	22.03	103.93
EABT9152	3473.75	13786.2	8430.22	15258.7	12041	78	1248
EABT9153	1	4	4	2	0	10	5
EABT9154	8874.55	9214.95	5813.9	7301.43	6206	8380.33	7164.58
EABT9155	0	0	2	2	0	0	0
EABT9156	527	855.92	795.88	4076.38	1146.62	240	165
EABT9157	17	27	43	81.61	15	6	12
EABT9158	8	2	4	0	0	3	0
EABT9159	39	104	370	130	56	23	14

EABT916	238	265	378.79	157	308	236.66	317.34
EABT9160	2	8	5	12.07	1	2	2
EABT9161	1062.95	1757.38	1129.76	2369.35	997	968.99	1090
EABT9162	0	5	15	78	4	0	0
EABT9163	0	0	7	2	0	0	0
EABT9164	3	9	2	1	4	6	22
EABT9165	11	12	16	6	4	5	6
EABT9166	7	24.67	23	38.03	3	6	6
EABT9167	1	1	8	1	0	0	0
EABT9168	5	6	13	6	1	3	2
EABT9169	0	0	4	3	2	0	0
EABT917	2	4.04	15	7.97	4	0	8
EABT9170	0	2	1	1	1	0	0
EABT9171	12	41	147	32	5	19	8
EABT9172	0	1	10	0	0	0	0
EABT9173	1	1	32	20	4	0	2
EABT9174	3	6	4	1	0	1	2
EABT9175	1	2	3	5	0	1	1
EABT9176	1	9	19	31	0	0	0
EABT9177	830	974	663.06	1273	1391	703	638
EABT9178	9	29.15	17	34	14	4	9
EABT9179	1	0	5	1	0	0	1
EABT918	0	0	1	54	5	0	1
EABT9180	866.93	1768.02	2578.52	4006.25	1398.71	729.37	1028.69
EABT9181	1	1	4	0	0	0	0
EABT9182	0	5	9.06	2	0	2.11	1
EABT9183	22	28	49	27.91	3	19	11
EABT9184	0	0	6	0	0	0	0
EABT9185	3	2	36	2	0	0	1
EABT9186	0	3	4	7	21	0	7
EABT9187	11	8	4	3	1	6	20
EABT9188	8	14	75	43	7	30	12
EABT9189	0	1	2	0	0	0	0
EABT919	0	1	15	1	1	1	0
EABT9190	54	110	185	786	173	18	97
EABT9191	3	1	1	14	0	1	0
EABT9192	1	4	19	4	1	0	1
EABT9193	3	10	0	44	1	0	0
EABT9194	553	824	607	877	626.45	476	484.77
EABT9195	1979.42	3367.59	1180.81	4178.48	2343.33	2635.92	4358.23
EABT9196	6407.72	12303.4	5220.94	2843.36	2855.73	9859.64	12599.8
EABT9197	0	4	2	0	0	1	0
EABT9198	6	16	19	2	3	1	0
EABT9199	0	5	3	0	3	1	2
EABT92	0	0	7	1	1	1	0
EABT920	0	2	3	21	5	0	0
EABT9200	0	2	6	3	1	0	0
EABT9201	306.36	136.71	1175.77	268	84	1	7
EABT9202	1	4	1	1	0	0	1
EABT9203	0	4	0	1	0	2	6
EABT9204	0	2	1	2	0	0	0

EABT9205	2	0	9	0	0	0	0
EABT9206	4	7	4	2	1	0	0
EABT9207	3037.64	3012.47	1873.49	1421.23	9660.45	2167.06	1725.98
EABT9208	3	1	65.64	6.01	5	1	0
EABT9209	0	0	0	38.95	0	0	0
EABT921	1	12	2	5	2	0	0
EABT9210	0	0	12	0	3	8	1
EABT9211	0	1	3	2	1	0	2
EABT9212	981.17	1579.89	2848.01	995.1	1527	590.99	671.58
EABT9213	5	6	5	18	2	0	1
EABT9214	5	22	4	1	4	5	9
EABT9215	1	3	6	8	5	2	4
EABT9216	48	3	18	4	0	16	1
EABT9217	1788.56	2322.38	1872.21	3903.17	1424.05	1699.43	2214.1
EABT9218	0	0	7	0	0	0	0
EABT9219	24	38	53	4	23	5	26
EABT922	0	0	8	0	0	0	0
EABT9220	0	0	1	0	1	1	2
EABT9221	2	1	18	5	2	0	0
EABT9222	0	2	9	3	1	0	1
EABT9223	17	33	25	5	9	10	8
EABT9224	2	2	21	0	0	3	2
EABT9225	1	1	27	1	1	1	0
EABT9226	1	3	5	2	25.3	1	10
EABT9227	1	1	18	1	0	1	0
EABT9228	1	9	26	4	0	2	0
EABT9229	1	3	2	1	0	0	0
EABT923	53	26	85.98	72	1	0	2
EABT9230	2	4	13	9	0	11	1
EABT9231	709.76	804.8	300.99	620	716.05	617.59	1209.01
EABT9232	3	5	10	40	4	0	0
EABT9233	0	2	3	15	0	0	1
EABT9234	0	1	9	6	2	0	0
EABT9235	2	2	2	5	0	1	0
EABT9236	0	1	0	0	0	0	4
EABT9237	0	0	4	4	2	0	0
EABT9238	1	2	4	3	2	0	1
EABT9239	2	5	5	39	6	2	3
EABT924	0	1	1	0	0	0	0
EABT9240	1227.68	1816.87	1977.21	2181	1607.59	870.14	1091
EABT9241	23485	39224.8	26079.2	12628.5	11078.9	8626.32	12555
EABT9242	70	104	33	135	158	17	57.02
EABT9243	0	2	9	1	1	0	0
EABT9244	814.68	2031.46	3746.25	3674.87	2602.85	1073.24	3055.04
EABT9245	1632.2	2007.4	1650.86	4514.79	1397.97	1247.57	1097.01
EABT9246	1142.9	1582.12	2191.4	1649	1487.27	965.15	1211.66
EABT9247	3	1	9	0	0	0	0
EABT9248	1	8	3	13	3	0	0
EABT9249	2	0	2	0	3	0	6
EABT925	0	0	13	4	0	0	0
EABT9250	0	2	1	0	0	0	0

EABT9251	5	10	6	2	3	2	2
EABT9252	0	0	8	0	0	0	1
EABT9253	0	2	1	5	2	0	3
EABT9254	17	26	29	10	85	6	11
EABT9255	0	1	3	19	2	0	3
EABT9256	1	6	1	0	2	4	4
EABT9257	2	4	21	3	7	0	6
EABT9258	1	18	63	43	1	6	7
EABT9259	0	1	16	2	1	0	0
EABT926	0	0	2	2	0	0	0
EABT9260	7	39	11	4	2	3	7
EABT9261	0	1	8	0	0	0	0
EABT9262	1	0	6	0	0	0	0
EABT9263	0	0	7	0	0	0	0
EABT9264	0	0	7	0	0	0	0
EABT9265	2	6	25	4	0	3	1
EABT9266	0	0	5	0	0	0	0
EABT9267	65388.6	44139.5	172386	7717.86	1319.67	19589.4	718
EABT9268	0	1	5	4	0	1	1
EABT9269	106	199	47	150.86	106	468.14	119
EABT927	0	0	15	4	0	1	0
EABT9270	0	0	2	0	6	0	0
EABT9271	0	0	0	1	0	0	1
EABT9272	0	1	22	0	1	0	0
EABT9273	78	213.86	286	691.95	461.77	39	57
EABT9274	0	2	2	0	2	0	0
EABT9275	0	0	5	1	0	0	0
EABT9276	4	4	13	7	1	2	0
EABT9277	3523.21	5273.04	3821.33	10941.8	4192.51	4911.19	4077.93
EABT9278	0	1	25	10	0	0	0
EABT9279	6	5	13	5	1	3	0
EABT928	1643.99	8809.93	16372.6	431	5063.06	284	5686.01
EABT9280	9	12	24	8	3	6	1
EABT9281	1	2	31	5	1	0	0
EABT9282	8	3	10	10	11	1	3
EABT9283	0	0	4	0	0	0	0
EABT9284	1	4	10	5	0	1	0
EABT9285	0	0	0	0	0	0	0
EABT9286	12	24	9	2	5	6	24
EABT9287	0	2	11	0	1	0	0
EABT9288	3	2	3	2	0	0	3
EABT9289	0	1	1	4	0	1	0
EABT929	0	1	9	0	0	0	0
EABT9290	7	9	5.13	11	2	10	2
EABT9291	0	1	2	1	0	0	1
EABT9292	0	2	5	3	3	8	6
EABT9293	1	0	1	4	0	1	0
EABT9294	1	3	6	1	3	0	1
EABT9295	7	2	3	1	8	5	19
EABT9296	3	5	1	6	0	0	1
EABT9297	0	0	5	0	0	1	0

EABT9298	0	3	6	0	0	0	0
EABT9299	1040.16	1971.99	1418	1653.3	1226.81	763.92	996.27
EABT93	1	2	10	1	0	0	0
EABT930	2	2	48	4	1	0	0
EABT9300	0	0	3	9	3	0	5
EABT9301	0	6	10	14	2	1	1
EABT9302	1	2	0	1	0	0	1
EABT9303	922.45	1970.01	2125.19	3887.37	1418	856	1132.03
EABT9304	1098.44	1691.68	1249.32	2319.01	875.47	1098	884.78
EABT9305	0	0	0	0	0	10	0
EABT9306	2	0	1	4	12.03	0	0
EABT9307	6	2	6	6	6	0	2
EABT9308	0	2	7	1	0	0	0
EABT9309	3120.31	6054.46	12597.5	11188.9	3971.07	4724.63	4506.76
EABT931	0	1	3	7	1	0	1
EABT9310	3	4	13	6	1	1	1
EABT9311	2	4	64	2	2	3	0
EABT9312	7	3	2	1	1	1	5
EABT9313	0	0	0	4	0	0	0
EABT9314	256	370	329.55	777.02	215	86	91.22
EABT9315	8	16	8	9	50	1	13
EABT9316	2	2	6	4	0	0	1
EABT9317	0	0	5	0	0	0	0
EABT9318	1053.98	2706	407.6	2067.08	945	4	88
EABT9319	139	217	617.7	1083.06	734.26	61	53
EABT932	0	0	8	0	0	0	3
EABT9320	0	11	12	3	2	0	1
EABT9321	0	1	13	0	0	1	0
EABT9322	2	2	5	2	1	0	1
EABT9323	1	7	0	21	1	1	1
EABT9324	0	1	3	0	2	0	2
EABT9325	0	5	22	1	1	1	1
EABT9326	0	0	2	8	1	3	0
EABT9327	663.97	1422.98	395.38	406.73	687.18	174	133
EABT9328	0	0	0	5	2	0	0
EABT9329	246.06	516	510.17	1217.56	585.23	175.24	315
EABT933	624.98	1032.12	376	894	769.61	179	1292.19
EABT9330	1	2	1	4	0	2	1
EABT9331	18	13	54.15	10.2	11	39.34	38
EABT9332	0	0	4	8	0	0	1
EABT9333	3	3	0	0	1	2	1
EABT9334	1	3	7	4	4	1	2
EABT9335	0	0	1	3	2	0	0
EABT9336	0	2	7	0	0	0	1
EABT9337	2	4	19	0	0	0	0
EABT9338	24	121	197	168	74	13	19
EABT9339	454.09	698.42	249.41	484.94	360.08	356.88	469.92
EABT934	1	0	4	4	2	1	0
EABT9340	2	9	24	16	0	0	0
EABT9341	0	1	18	0	0	0	0
EABT9342	0	1	7	0	0	0	0

EABT9343	1655.47	3195.89	4942.07	5164.65	2952.9	3731.63	3438.72
EABT9344	185	268	151	42	917.42	11	66
EABT9345	1	0	2	2	1	0	1
EABT9346	6	9	21	74	22	7	5.75
EABT9347	2	11	35	31	6	5	3
EABT9348	0	0	3	0	0	0	0
EABT9349	2	1	6	10	9	0	3
EABT935	0	0	0	0	0	0	4
EABT9350	3418.17	6487.47	7759.5	11677.2	2642.71	881.36	911.93
EABT9351	401	306	193	260	232.07	410	133
EABT9352	0	0	4	5	4	3	0
EABT9353	5	4	0	0	0	1	2
EABT9354	6	59	4	2	45	0	71
EABT9355	7	5	18	104	9	6	59
EABT9356	0	0	6	0	0	0	0
EABT9357	1	6	1	1	4	0	1
EABT9358	104	13	8	4	2	114.03	1
EABT9359	0	0	13	0	0	0	1
EABT936	0	2	5	0	0	1	0
EABT9360	10	30	37	26	5	0	0
EABT9361	33	205	1	0	1	0	1
EABT9362	0	3	5	0	0	0	0
EABT9363	0	0	6	0	0	0	0
EABT9364	0	0	3	1	0	0	0
EABT9365	335	472	347	659	381	355	333.15
EABT9366	0	4	3	2	2	0	0
EABT9367	1	4	11	9	0	0	0
EABT9368	10	64.93	54	360	439.05	2	4
EABT9369	0	2.49	14	0	0	1	1
EABT937	24	23	11	6	0	16	12
EABT9370	0	0	3	2	0	0	0
EABT9371	9	18	31	13	0	1	1
EABT9372	10	61	38	10	4	10	1
EABT9373	2	8	18	1	5	0	1
EABT9374	0	0	1	1	0	0	0
EABT9375	0	0	3	9	0	2	0
EABT9376	0	1	6	0	0	9	0
EABT9377	4	11	30	2	0	0	0
EABT9378	55	51	247.39	10	115	6	32
EABT9379	1	0	0	1	0	0	0
EABT938	1	2	1	0	0	0	0
EABT9380	4	3	91.99	32	11	4	49
EABT9381	7	18	26	12	11	3	10
EABT9382	706	998.42	922.36	1976.53	629	523	421.17
EABT9383	0	0	3	1	0	1	0
EABT9384	0	0	5	1	0	0	0
EABT9385	1	2	0	0	0	0	0
EABT9386	0	1	0	1	0	0	5
EABT9387	0	1	0	2	2	0	2
EABT9388	45	119	126.57	55	5	13	3
EABT9389	895.99	1407.42	3708.18	7235.93	1443.54	625.99	866

EABT939	0	0	2	0	12	0	0
EABT9390	0	3	6	5	2	1	2
EABT9391	0	2	154	0	0	0	0
EABT9392	6	2	12	2	3	0	13
EABT9393	0	0	3	1	1	0	0
EABT9394	2	13	20	41	112	2	33
EABT9395	139.01	226.02	100	381	181.06	56	177.08
EABT9396	1	3	5	0	0	0	0
EABT9397	2	2	4	0	1	0	0
EABT9398	582.3	753.77	354.2	1499.15	540.95	447.97	448.16
EABT9399	8	19.02	15	46.94	9	3	7
EABT94	152	287	339	153.8	100	139	60
EABT940	2	10	1	10	0	4	0
EABT9400	0	1	14	0	0	0	0
EABT9401	1	0	4	0	1	0	1
EABT9402	7	9	15	23	475.99	8	7
EABT9403	0	0	28	2	0	0	0
EABT9404	21	45	69	22	3	20.99	3
EABT9405	1	8	1	54	4	1	0
EABT9406	0	0	8	0	0	0	0
EABT9407	0	3	2	4	0	0	0
EABT9408	292	315	280	199.87	2311	2	39
EABT9409	370.3	158	43	69.03	68.94	49.09	120
EABT941	52	70	197.95	22	183	21.35	58
EABT9410	34831.8	31065.2	2563.4	4862.74	5475.94	4039.28	9999.57
EABT9411	4	8	0	0	0	0	1
EABT9412	730.91	1219.38	1263.04	2244.1	1103.57	716.16	888.74
EABT9413	0	3	3	0	1	0	0
EABT9414	2	10	21	0	0	0	0
EABT9415	850.04	1350.76	1273.92	2604.12	765.01	671.44	694
EABT9416	2	13	48	19	4	0	5
EABT9417	13	12	1	0	0	2	0
EABT9418	2	1	0	0	0	2	1
EABT9419	0	7	4	15	1	3	2
EABT942	1	5	26	1	28	15	7
EABT9420	0	0	4	0	0	0	0
EABT9421	3	3	0	0	0	0	0
EABT9422	882.07	1160.56	687.69	1502.96	531.46	716.09	595.93
EABT9423	0	0	0	7	0	0	0
EABT9424	1	2	18	5	2	1	4
EABT9425	2	15	35	0	0	54	4
EABT9426	2046.67	3209.66	4756.3	6550.86	1841.89	1719.59	1865.66
EABT9427	5	0	3	0	0	4	1
EABT9428	1788	1866.99	571	272	815	1233	1093.06
EABT9429	0	2	11	3	6	0	2
EABT943	0	0	5	0	0	0	0
EABT9430	5	3	1	6	0	32	1
EABT9431	4	11	4	8	9	4	5
EABT9432	4	10	4	20	44	0	38
EABT9433	0	0	1	0	0	0	0
EABT9434	590.75	659	1983.02	1899.69	935.29	797.49	451.44

EABT9435	1	0	2	0	12	0	0
EABT9436	1	5	11	2	2	3	4
EABT9437	2	4	3	6	1	0	0
EABT9438	0	1	5	0	0	2	1
EABT9439	2	4	4	0	1	3	1
EABT944	0	1	2	1	0	0	0
EABT9440	3	5	9.54	5	49	0	40.85
EABT9441	481	324	106	678.12	82	900.26	466.68
EABT9442	9	19	171	171	32	5	30
EABT9443	1	2	12	7	5	9	3
EABT9444	0	0	7	0	0	0	0
EABT9445	0	2	12	0	0	0	0
EABT9446	1	2	2	2	0	0	0
EABT9447	1	0	4	2	1	3	2
EABT9448	1241.89	980.05	2003.86	316.54	116	2	5
EABT9449	0	3	5	11	0	2	3
EABT945	3	5	22	1	1	0	0
EABT9450	0	2	1	2	2	6	1
EABT9451	5	4	11	3	0	0	0
EABT9452	0	0	1	10	5	0	0
EABT9453	7	3	27	6	1	2	7
EABT9454	3	2	0	0	0	62	0
EABT9455	2	2	11	3	0	1	2
EABT9456	5	3	18	1	0	0	0
EABT9457	230	439.27	471	1286.95	621	282	318
EABT9458	1342.41	4661.82	4282.9	1475.67	3230.31	46	573.1
EABT9459	1809.99	2032.15	1363.01	1785.66	2413.06	1011.94	1261.04
EABT946	0	2	3	1	1	0	0
EABT9460	394.68	621.23	572	2396.98	569.45	299	229
EABT9461	5738.82	8525.71	10526.2	16384.4	3719.18	6325.58	5493.07
EABT9462	993.89	1509.34	1670.07	1545.93	1138.33	1056.26	1057.57
EABT9463	1083.67	1390.84	1678.9	1645.16	850.07	1007	900.99
EABT9464	2	0	3	0	4	0	4
EABT9465	1	7	19	13	4	2	5
EABT9466	1	0	0	0	0	0	0
EABT9467	0	6	26	14	2	1	0
EABT9468	0	0	7	0	0	0	0
EABT9469	4946.3	8874.28	5841.73	12730.1	4231.74	3814.02	3905.8
EABT947	0	0	2	3	2	0	0
EABT9470	1	1	8	4	0	0	0
EABT9471	0	3	5	6	2	9	0
EABT9472	7	0	8	1	0	2	0
EABT9473	986.94	1441.39	1544.76	1933.81	1763.64	1005.58	1352.79
EABT9474	89	392	86	89	210	14	74
EABT9475	1	2	7	1	2	4	2
EABT9476	0	0	14	0	0	0	0
EABT9477	0	0	1	7	2	0	0
EABT9478	198.43	282.08	126.35	88.45	352.55	190	141.99
EABT9479	6	14	1	1	1	2	6
EABT948	0	0	2	4	2	0	0
EABT9480	1	2	6	36	0	0	1

EABT9481	2	8	17	2	0	0	1
EABT9482	1	3	9	1	0	2	1
EABT9483	26396.7	17039.6	7519.22	6496.66	12157.7	19074.7	11487.9
EABT9484	1	1	0	0	1	0	2
EABT9485	5	13	14	29	17	33	37
EABT9486	0	1	14	10	0	1	2
EABT9487	86253.1	37986.5	165.99	75	679	42558.3	4941.79
EABT9488	570.88	869.46	585.1	1057.91	405.77	461.75	421.96
EABT9489	2	5	51	4	0	0	2
EABT949	4	14	17	18	1	1	0
EABT9490	21	33	155	187.1	38	13	21
EABT9491	0	0	0	0	10	0	2
EABT9492	0	0	22	1	0	0	0
EABT9493	0	0	6	0	0	0	0
EABT9494	0	0	7	0	0	0	1
EABT9495	0	3	15	3	0	0	0
EABT9496	0	0	3	4	0	0	0
EABT9497	1621.99	2651.25	1378.59	3550.78	1289.36	1313.07	2051.15
EABT9498	777	1192.75	1057.88	2631	676	620	587
EABT9499	4	3	5	17	0	1	0
EABT95	0	2	3	3	3	1	4
EABT950	1	0	2	0	0	0	1
EABT9500	299	337.99	145	346.24	242	212	196
EABT9501	5	0	62	11	62	0	3
EABT9502	1	2	6	2	3	0	0
EABT9503	0	1	6	1	2	0	0
EABT9504	0	0	15	0	0	0	0
EABT9505	1	2	34	1	1	7	0
EABT9506	4	2	5	3	16	3	4
EABT9507	4	13	120	5	2.66	0	1
EABT9508	0	5	6	0	0	1	0
EABT9509	82	107	0	0	0	2	2
EABT951	2	1	27	4	4598.75	1	2
EABT9510	54	70	28	125	47	32	2
EABT9511	2	7	35	0	1	3	1
EABT9512	250	407.42	462	1101.5	608	289	259
EABT9513	686	878.08	355.19	1156.34	817	672.7	658
EABT9514	0	0	3	2	0	1	0
EABT9515	0	2	1	3	1	0	1
EABT9516	0	0	10	0	0	0	0
EABT9517	2	3	0	0	1	1	13
EABT9518	0	1	16	0	0	0	1
EABT9519	0	1	1	2	0	0	1
EABT952	44	49	11	43	36	27	53
EABT9520	5	4	23	2	2	0	0
EABT9521	0	1	2	1	2	0	21
EABT9522	0	3	1	1	0	0	1
EABT9523	0	2	0	0	1	0	2
EABT9524	0	0	21	2	5	0	0
EABT9525	236.07	227.18	26	0	9	121.4	53.07
EABT9526	0	0	0	12	0	0	0

EABT9527	0	2	19	0	0	1	0
EABT9528	2	2	37	5	4	0	0
EABT9529	0	0	2	4	5	0	1
EABT953	0	0	1	1	0	0	1
EABT9530	2	3	0	0	0	0	1
EABT9531	0	2	3	6	2	0	0
EABT9532	6	8	65	35	0	0	1
EABT9533	0	4	0	3	0	1	2
EABT9534	14817	16831.5	14368.6	12189.4	12248	13356.3	14158.4
EABT9535	4	9	33	15	10	5	1
EABT9536	117	152.97	271.07	109	425.23	6	35
EABT9537	11	17	15	35	2	6	15
EABT9538	0	2	5	0	0	0	0
EABT9539	2	0	3	5	0	1	0
EABT954	0	0	1	3	3	0	0
EABT9540	6	12	15	6	6	0	2
EABT9541	0	0	1	0	5	0	0
EABT9542	0	8	0	0	0	1	0
EABT9543	1	5	16	21	2	0	2
EABT9544	0	2	6	3	0	0	0
EABT9545	0	1	0	4	2	1	0
EABT9546	4	1	1	1	0	0	0
EABT9547	6	5	44	23.11	8	12	5
EABT9548	1	2.05	5	19	0	3	2
EABT9549	0	1	2	0	0	3	1
EABT955	614.76	981.99	1283.67	1573.72	567	448	470
EABT9550	0	4	1	1	0	0	0
EABT9551	311.81	419.68	319.32	901.61	510.7	264.74	311.36
EABT9552	1	0	12	0	0	0	0
EABT9553	3508.7	5829.42	2626.52	5273.54	1436.3	531.93	849.81
EABT9554	2	6	7	41	5	0	1
EABT9555	2	1	82	7	2	4	0
EABT9556	0	3	13	0	2	0	1
EABT9557	0	0	5	1	0	0	0
EABT9558	97	297.01	781.28	260.17	81	160	129.12
EABT9559	151	240.97	84	180.05	29	97	100
EABT956	0	9	25	6	1	0	0
EABT9560	0	2	4	1	0	0	1
EABT9561	2	3	30	5	1	3	0
EABT9562	24	40	50.03	1020	18	22.64	46
EABT9563	11	19	132	3	6	0	2
EABT9564	13	10	11	1	3	18.99	4
EABT9565	0	0	7	0	0	0	0
EABT9566	2	2	0	0	3	5	1
EABT9567	0	0	16	3	0	7	0
EABT9568	718.99	1012.35	797.02	2118.74	731.02	513.01	661.98
EABT9569	0	1	15	1	0	1	1
EABT957	0	4	7	0	4	0	3
EABT9570	13168.2	24441.2	20446.8	13079.6	19906.2	1214.28	4301.74
EABT9571	481.94	698.92	272.88	1064.03	357.99	291.8	212.03
EABT9572	0	2	1	0	0	0	0

EABT9573	0	0	10	0	1	0	0
EABT9574	2435.65	5155.64	3646.25	7950.68	2427.94	1097	1615.22
EABT9575	1	3	8	0	0	1	0
EABT9576	4	2	29	6	1	5	1
EABT9577	0	0	12	0	0	0	0
EABT9578	0	0	9	0	0	0	0
EABT9579	3	6	12	1	0	0	1
EABT958	1	0	4	0	0	0	0
EABT9580	5	7	0	0	0	0	0
EABT9581	0	2	8	1	0	0	0
EABT9582	454.98	531.25	280.93	688.69	364.99	363.56	323.26
EABT9583	9	28	7	8	5	1	2
EABT9584	0	1	14	1	0	0	0
EABT9585	4	7	21	444.24	56	0	4
EABT9586	12	51.77	43	78	12	13	9
EABT9587	14	16	2	7	516	5	1
EABT9588	0	0	0	0	3	0	0
EABT9589	219	455.74	33	9.05	69	69.08	237
EABT959	287	472	337	623	443	258	199.95
EABT9590	942	1227	1459.02	1769	958	910.11	699
EABT9591	0	3	0	1	1	1	9
EABT9592	12	21	0	0	1	13	2
EABT9593	1	7	8	1	2	2	0
EABT9594	8	4	6	2	0	4	4
EABT9595	8	6	0	1	0	0	0
EABT9596	0	0	3	3	0	0	0
EABT9597	1184.81	1573	1381.38	2933.45	1056.52	680.01	532.1
EABT9598	5	3	0	3	0	0	0
EABT9599	0	3	24.01	3	0	1	1
EABT96	4	21	37	26	13	2	7
EABT960	0	0	0	0	4	1	5
EABT9600	0	4	9	5	3	1	4
EABT9601	0	0	0	9	1	0	0
EABT9602	8	73	52	70	97	2	4
EABT9603	0	0	0	2	0	0	1
EABT9604	53	60	56	20	10	5	4
EABT9605	0	2	9	0	1	0	0
EABT9606	0	1	12	0	0	0	0
EABT9607	0	0	14	1	0	3	1
EABT9608	1	3	8	1	0	0	2
EABT9609	2	7	2	2	5	1	0
EABT961	1	4	0	0	0	2	1
EABT9610	0	2	10	2	0	0	1
EABT9611	5	2	40	2	0	0	0
EABT9612	1	2	8	2	0	0	0
EABT9613	0	2	7	23	0	0	0
EABT9614	2	0	36	1	1	0	0
EABT9615	2	2	18	0	0	0	0
EABT9616	0	3	1	4	8.21	1	0
EABT9617	2	2	18	7	1	0	0
EABT9618	12	40	45	79	6	8	5

EABT9619	0	1	2	1	24	0	1
EABT962	1015.79	1590.73	1678.71	2912.43	2194.76	703.26	1346.92
EABT9620	6	20	5	5	2	18	6
EABT9621	8	18	8	11	4	1	2
EABT9622	11	36	3	50	0	10	0
EABT9623	0	0	0	0	0	3	2
EABT9624	0	1	0	8	3	0	4
EABT9625	2455.09	3119.96	892	2092.68	1276.88	854	1669.59
EABT9626	207	433.94	462	835	338	170	116
EABT9627	0	0	0	0	12	0	0
EABT9628	227.6	192.34	466.86	116.06	87.48	83.12	49.99
EABT9629	643	718.11	464	928.48	488	488.61	430.07
EABT963	3	15	8	32	5	4	3
EABT9630	0	3	12	0	1	0	1
EABT9631	0	2	8	0	0	0	1
EABT9632	3	8	44	19	2	0	1
EABT9633	1	7	2	1	1	1	0
EABT9634	0	1	3	1	1	0	0
EABT9635	0	2	0	3	3	0	0
EABT9636	0	2	18.87	2	4	0	0
EABT9637	3	7	4	6	1	0	2
EABT9638	1947.46	899.04	899.24	963.65	144.81	2374.94	214.93
EABT9639	0	0	0	0	1	0	6
EABT964	11319.5	12361.9	9616.12	13330.1	15784.5	12257.3	8914.3
EABT9640	40	18	1	0	0	34	10
EABT9641	0	1	0	3	0	1	1
EABT9642	2863.77	2771.28	1006.98	1786.98	3722.45	1426.82	1533.1
EABT9643	138	718	161	31325.6	1	0	0
EABT9644	4089.38	6043.2	9222.23	8183.47	4470.99	3273.99	2820.97
EABT9645	5	0	1	1	1	2	14
EABT9646	2	0	8	1	1	0	0
EABT9647	1	0	5	0	1	0	0
EABT9648	17	34	0	0	3	58	26
EABT9649	0	1	1	12	0	0	0
EABT965	522	576.89	240	536	365	484.17	336
EABT9650	0	1	0	1	5	0	0
EABT9651	1	3	18	5	1	0	0
EABT9652	0	14	1	8	15	1	3
EABT9653	3	0	0	0	1	0	1
EABT9654	1	2	1	0	0	0	0
EABT9655	0	2	2	0	0	0	3
EABT9656	12	12	0	0	0	0	0
EABT9657	1720.25	2992.3	286.4	163	593.25	2188.68	2937.26
EABT9658	4	1	6	2	0	1	1
EABT9659	0	2	8	9	22	0	6
EABT966	0	0	37	0	0	0	0
EABT9660	1443	2229.99	898.3	732	5652.71	87	310
EABT9661	0	5	17	0	1	0	0
EABT9662	1	2	11	3	0	3	1
EABT9663	145	238.96	388	951.01	597	13	48
EABT9664	1	0	0	0	4	0	7

EABT9665	3	8	7	14	5	1	1
EABT9666	0	1	2	0	1	0	1
EABT9667	2	5	14	81	1	0	0
EABT9668	11	6	7	39	5	2	3
EABT9669	0	4	3	1	5	0	1
EABT967	7	5	1	6	51716.9	193	589161
EABT9670	5	8	2	10	5	1	1
EABT9671	36	124	175.97	329	200	24	194
EABT9672	0	3	2	0	0	1	0
EABT9673	101.54	130	17	6	43	698.65	262.58
EABT9674	174.99	340.82	587.12	102	910.09	476.99	481.93
EABT9675	1	0	1	1	0	15	0
EABT9676	0	1	1	0	1	5	5
EABT9677	0	2	79	3	0	0	1
EABT9678	1	0	1	7	0	0	0
EABT9679	0	0	8	2	0	0	1
EABT968	3282.91	2142.17	1242.78	1818.04	1728.31	1911.63	6100.67
EABT9680	2	14	25	7	2	1	2
EABT9681	6	7	43	15	11	8	3
EABT9682	0	0	18	1	1	0	1
EABT9683	0	1	6	3	0	0	0
EABT9684	2	3	12	1	0	0	0
EABT9685	1	0	1	2	1	0	0
EABT9686	58	94.87	391.13	230	114	20	139
EABT9687	10	10	2	3	25.99	16.99	24
EABT9688	0	0	2	0	1	0	0
EABT9689	0	0	6	0	0	0	0
EABT969	1	0	12	0	15	1	8
EABT9690	0	0	42	0	1	0	0
EABT9691	0	0	1	2	0	1	1
EABT9692	13187.6	11625.3	2617.13	2280.18	297.43	4901	393.02
EABT9693	1277.07	1727.22	1931.89	5084.35	3064.59	763.49	1050.94
EABT9694	543	558	1662.38	705.7	942.98	265.76	322
EABT9695	211.93	373	456.87	882.01	523.61	198	239.99
EABT9696	2	6	6	6	1	0	0
EABT9697	13390.6	36652.8	34147	43753.3	16127.1	5758.63	7918.71
EABT9698	2	0	1	0	0	0	1
EABT9699	1	5	17	45	6	3	1
EABT97	2	3	69.15	6	0	2	2
EABT970	0	2	0	3	3	0	0
EABT9700	13	30	25	29	28	7	21
EABT9701	3	12	57	10	0	0	2
EABT9702	6	13	20	18	6	0	3
EABT9703	2	6	8	0	2	0	2
EABT9704	0	0	10	3	0	1	1
EABT9705	1	2	5	1	1	0	2
EABT9706	1	4	4	25	1	2	0
EABT9707	0	0	2	2	3	0	3
EABT9708	0	4	25	5	1	1	0
EABT9709	2	3	4	9	72	1	0
EABT971	1	7	4	3	2	3	0

EABT9710	0	2	0	35	1	0	1
EABT9711	1	2	6	2	0	0	0
EABT9712	0	0	5	0	1	0	0
EABT9713	0	0	0	1	1	0	1
EABT9714	1	1	0	0	1	0	2
EABT9715	1	0	6	0	1	0	0
EABT9716	10	14	12	4	5	3	1
EABT9717	0	3	6	1	1	1	0
EABT9718	3	2	32	1	3	2	8
EABT9719	0	0	18	1	1	0	0
EABT972	3	16	17	21	43	0	13
EABT9720	1	1	9	1	0	1	0
EABT9721	1	0	7	5	1	0	4
EABT9722	0	2	5	0	9	0	2
EABT9723	4	21	25	3	1	4	4
EABT9724	0	0	3	1	0	1	0
EABT9725	5	12	3	11	13	1	8
EABT9726	309	388	186	597.91	377	176	224
EABT9727	4	3	11	4	0	2	1
EABT9728	37.76	129	110.99	148	19	40	24
EABT9729	2	1	3	0	0	0	0
EABT973	2361.76	4486.56	2328.92	3866.17	2403.93	3208.85	3830.98
EABT9730	1	0	11	8	0	0	0
EABT9731	296.23	77.97	22	13	66	76	79
EABT9732	7651.48	9247.58	11706.5	8829.85	3181.05	2737.34	2043.1
EABT9733	22	40	593.17	12	16	26	21
EABT9734	0	0	10	4	0	0	0
EABT9735	0	1	8	0	0	0	0
EABT9736	169	279.94	185.74	600	473	87	112.55
EABT9737	5	21.01	37	323	89	6	2
EABT9738	3	0	1	1	0	0	0
EABT9739	0	8	10	2	1	1	1
EABT974	0	1	7.06	2	0	0	0
EABT9740	0	2	4	0	0	0	0
EABT9741	13	19	31	3	2	13	11
EABT9742	1	1	6	1	2	0	0
EABT9743	12	13	227	12	10	1	2
EABT9744	3442.22	3669.35	1842.51	2241.42	1656.14	1888.54	2044.69
EABT9745	1	6	47	2	3	3	0
EABT9746	9	12	21	33	16	11	8
EABT9747	1	2	3	11	0	1	0
EABT9748	7	7	8	1	0	1	0
EABT9749	2	14	23	24	215.58	4	12
EABT975	0	0	13	0	0	0	0
EABT9750	0	1	8	0	0	0	0
EABT9751	0	0	0	0	0	0	0
EABT9752	1808.98	1713.73	794.15	592.05	489.64	318.3	207
EABT9753	4	7	5	15	2	3	2
EABT9754	3	8	44	17	4	11	15
EABT9755	14	15	0	0	0	0	0
EABT9756	0	0	4	1	0	0	0

EABT9757	10	12	20.66	18	5.58	6	7
EABT9758	15	2	2	0	0	47	0
EABT9759	90	214	334.55	956.58	147	21	50
EABT976	7	10	17	11	4	13	9
EABT9760	0	0	0	0	1	1	3
EABT9761	0	0	0	7	0	0	0
EABT9762	2	5	10	7	12	1	4
EABT9763	238	705	742	1664.13	581.98	177	313.72
EABT9764	0	1	2	2	0	0	1
EABT9765	1	1	5	0	1	0	0
EABT9766	1	0	17	9	1	1	1
EABT9767	0	1	8	8	1	1	1
EABT9768	65	140	52	112	32	25	82
EABT9769	1544	2022.74	1062.99	1349.01	1748	1139	914
EABT977	1988.52	2424.18	2521.12	3112.07	1938.43	1686.88	1371.96
EABT9770	1	2	3	0	0	0	0
EABT9771	6	4	24	22	9	1	2
EABT9772	3	6	29	0	2	3	1
EABT9773	40	342	175	16	8	12	2
EABT9774	0	0	2	2	0	0	1
EABT9775	2	2	3	10	5	0	0
EABT9776	1	5	75	3	2	2	14
EABT9777	9	40.91	1	1	381	7	2245
EABT9778	0	0	8	1	0	0	0
EABT9779	4	7	0	0	0	1	1
EABT978	167	1621.57	200	1250.18	853.05	415	897
EABT9780	2	1	4	1	0	0	0
EABT9781	90	256	320.19	704.92	1143.68	11	4
EABT9782	1	2	10	2	0	0	0
EABT9783	1	1	6	5	0	0	1
EABT9784	0	0	23	0	0	0	0
EABT9785	299.06	373.61	204.75	568.33	301.09	198.79	235.52
EABT9786	3	11	14	11	13	3	0
EABT9787	179767	188208	125874	89331.6	165509	78185.2	93438.7
EABT9788	0	0	2	1	4	0	0
EABT9789	0	0	6	0	0	0	0
EABT979	0	0	5	1	0	0	0
EABT9790	1	3	7	14	5	0	8
EABT9791	648.94	4124.3	2663.76	4148.8	2641.19	1914.92	1869.08
EABT9792	11	19	19	86	134	11	8
EABT9793	0	3	17	1	0	0	0
EABT9794	29	12	29	18	4	5	7
EABT9795	2	1	15	1	1	0	0
EABT9796	2	4	0	3	0	0	0
EABT9797	0	0	5	0	0	1	0
EABT9798	100	180	103	742.06	45	7	17
EABT9799	4	3	5	4	0	0	0
EABT98	0	0	19	3	0	0	0
EABT980	2	1	8	13	4	2	4
EABT9800	600.38	1021.67	1063.53	2687.91	1012.76	536.97	464.01
EABT9801	1112.98	1751.07	3041.66	6303.22	2102.04	1437.46	1099.82

EABT9802	1	0	8	0	0	1	0
EABT9803	2	3	8	0	0	0	1
EABT9804	0	0	6	11	1	0	1
EABT9805	0	3	4	6	0	0	0
EABT9806	10675.7	18426.6	8798.57	10324.9	9601.94	7533.23	13088.1
EABT9807	0	2	0	0	0	9	1
EABT9808	0	0	5	4	6	0	0
EABT9809	84	138	184	753.42	159	61	38
EABT981	396.6	472.07	533.68	793.03	554.84	329.46	344.82
EABT9810	4	4	3	4	10	0	1
EABT9811	9173.8	12547.4	18898.1	3265.48	2591.2	669.36	104.8
EABT9812	28	147	698.85	174	200	14	27
EABT9813	47	18	117.4	3	2	22	21
EABT9814	1	2	4	6	3	3	0
EABT9815	1	0	2	0	0	0	0
EABT9816	2	11	24	170	16	0	4
EABT9817	0	1	7	2	0	3	1
EABT9818	28	0	0	0	0	1	0
EABT9819	1139.09	2139.79	3005.77	3241.37	1310.61	873.37	1171.47
EABT982	232	243	664	100	154.12	230	94
EABT9820	0	0	1	0	19	0	1
EABT9821	0	0	11	0	0	3	0
EABT9822	13	9	221	14	4	1	3
EABT9823	65	32	7	5	36	3	558
EABT9824	25	48	62	60	15	2	7
EABT9825	2	4	30	11	4	1	0
EABT9826	1	4	20	2	1	2	0
EABT9827	1	0	10	0	0	5	0
EABT9828	2	1	8	2	0	1	1
EABT9829	7	8	25	23	7	3	78
EABT983	0	0	1	1	0	2	0
EABT9830	1048.94	1268.91	989.78	2693.32	915.54	933.5	579
EABT9831	2	2	3	7	1	0	2
EABT9832	2	4	8	25	11	7	7
EABT9833	1	4	11	85	2	1	2
EABT9834	2	0	0	0	0	0	1
EABT9835	1	1	14	2	1	1	0
EABT9836	0	5	19	11	2	1	4
EABT9837	2	4	26	7	4	0	5
EABT9838	4	6	1	0	2	7	20
EABT9839	0	0	1	0	39	0	0
EABT984	1036	1730.08	1895	3277	2117	555.79	571
EABT9840	1	2	1	4	0	0	0
EABT9841	209	365.39	301.64	747.86	429.29	218.69	245.66
EABT9842	0	0	20	1	1	0	0
EABT9843	0	0	9	1	1	0	0
EABT9844	1580.01	1397.19	1466.48	1190.43	801.67	1397.35	1047.25
EABT9845	0	0	12	0	0	0	0
EABT9846	19	11	5	4	2	3	2
EABT9847	0	0	3	0	0	0	4
EABT9848	0	0	6	4	0	0	0

EABT9849	2	3	12	4	0	0	1
EABT985	9	0	0	0	0	18	0
EABT9850	0	0	0	2	0	0	1
EABT9851	0	3	1	0	0	0	2
EABT9852	1	1	7	1	0	0	0
EABT9853	6	0	9	3	1	1	0
EABT9854	437	659.09	717.36	919	438	326	406
EABT9855	19187.7	4514.55	1538.91	3177.3	2413	396.13	1307.01
EABT9856	4641.11	7261.61	6375.95	5158.04	5638.43	2315.66	2623.72
EABT9857	2	3	13	4	0	0	1
EABT9858	0	0	5	2	0	0	0
EABT9859	1	1	10	1	0	0	1
EABT986	0	2	0	0	0	0	0
EABT9860	0	4	33	1	1	1	3
EABT9861	1	5	11	3	0	2	3
EABT9862	0	0	5	0	0	1	0
EABT9863	1	2	17	3	0	3	1
EABT9864	0	0	0	2	0	0	0
EABT9865	3	4	17	0	0	25	0
EABT9866	39	96	40	58	8.9	16	6
EABT9867	0	0	1	0	1	4	0
EABT9868	19	22	30	47	21	6	11
EABT9869	0	0	3	0	0	0	16
EABT987	2030.85	4803.4	8707.98	7871.63	2917.86	1814	2165.14
EABT9870	1414.84	3953.24	5947.16	4941.31	2968.15	2891.94	2150.5
EABT9871	120.74	84.01	1	0	2	81	4
EABT9872	1863.01	2945.99	2508	2585.42	1462.41	1374.71	1480.26
EABT9873	19	39	32	44	4	2	7
EABT9874	727.01	839.94	533.16	1264.99	699.58	434	420
EABT9875	9	19	10	6	2	7	2
EABT9876	1	1	4	3	1	2	5
EABT9877	1	1	21	0	0	1	0
EABT9878	1507.76	2511.71	1648.62	2148.59	1084.07	1498.84	1099.62
EABT9879	7	1	1	0	1	2	1
EABT988	334	408.02	215	888	307	127	164
EABT9880	1	8	12	10	1	2	4
EABT9881	0	2	0	3	0	0	0
EABT9882	0	0	1	3	0	0	0
EABT9883	0	0	9	2	0	0	0
EABT9884	84	138	212	1140	141	89	35
EABT9885	1	0	13	1	0	2	0
EABT9886	7	8	12	45	2	3	3
EABT9887	2	5	48	1	0	0	2
EABT9888	2	1	0	1	1	0	3
EABT9889	1	0	0	0	2	0	0
EABT989	33	39	0	0	5	13	23
EABT9890	1	3	5	6	1	2	1
EABT9891	0	0	11	2	1	0	1
EABT9892	417.95	555.76	413	791.75	358	235	215
EABT9893	0	0	0	0	2	0	1
EABT9894	4	2	41	4	1	0	0

EABT9895	9633.4	10742.7	11619.8	7969.4	7649.85	7977.49	9214.73
EABT9896	1	10	32	2	0	2	0
EABT9897	0	3	7	1	1	0	1
EABT9898	0	0	22	2	0	0	0
EABT9899	6	7	11.06	42	5	5.98	6
EABT99	1	3	19	4	0	0	1
EABT990	6	14	26	9	4	3	1
EABT9900	3	1	9	2	0	4	0
EABT9901	1042.76	1851.51	3572.14	2047.92	2649.64	940.17	1916.22
EABT9902	164.02	386	2177	2114.71	5460.24	299	140
EABT9903	21	21	115	17	10	5	3
EABT9904	1	2	2	12	2	1	0
EABT9905	0	0	1	0	0	2	0
EABT9906	0	1	4	0	0	0	0
EABT9907	0	0	17	1	0	0	0
EABT9908	0	5	14	5	3	0	1
EABT9909	1	2	5	6	2	5	3
EABT991	0	0	40	2	0	9	0
EABT9910	1	2	4	2	0	0	2
EABT9911	1	7	2	1	1	0	1
EABT9912	0	0	6	1	0	0	0
EABT9913	0	28	3	0	0	1	0
EABT9914	1	0	1	29	1	0	4
EABT9915	11707.3	14022	3292.42	4367.13	2667.63	5787.37	3234.73
EABT9916	1	9	44	0	32.12	0	4.29
EABT9917	2	7	14	9	0	1	0
EABT9918	4	1	1	1	1	0	0
EABT9919	0	0	0	0	0	0	0
EABT992	0	2	26	0	1	0	0
EABT9920	0	0	3	5	0	0	0
EABT9921	0	3	6	2	4	2	10
EABT9922	0	4	1	7	0	0	0
EABT9923	1	3	0	1	0	7	1
EABT9924	1	0	0	5	0	0	0
EABT9925	1	1	1	2	0	1	0
EABT9926	0	0	17	2	0	0	0
EABT9927	0	0	5	1	0	0	0
EABT9928	1	6	0	0	0	4	0
EABT9929	1901.18	3173.03	5412.11	7724.32	1635.47	1423.92	1804.55
EABT993	4	4	3	4	5	1	2
EABT9930	0	1	2	2	0	1	0
EABT9931	2	3	21	5	0	1	0
EABT9932	1	0	3	0	4	0	0
EABT9933	1	0	0	9	2	0	0
EABT9934	2	3	14	1	0	0	0
EABT9935	0	2	4	0	1	0	0
EABT9936	2	3	23	31	9	1	0
EABT9937	17	23	14	47	18	1	1
EABT9938	33	108	76	15	11	47	31
EABT9939	3	3	5	7	1	0	5
EABT994	0	0	6	1	0	0	0

EABT9940	346	1019.98	1038.41	312.28	203.09	460.93	282.24
EABT9941	0	1	13	0	0	0	0
EABT9942	19	49	25.98	166.02	150.27	1	17
EABT9943	2	0	2	7	1	0	0
EABT9944	0	1	3	0	0	1	1
EABT9945	3	3	5	0	0	3	0
EABT9946	1	0	5	0	0	0	0
EABT9947	0	0	0	0	0	8	0
EABT9948	0	1	8	6	3	0	0
EABT9949	3880.97	6531.7	6485.91	35125.7	5746.16	375.33	1296.7
EABT995	0	0	13	0	1	0	0
EABT9950	9480.42	3672	2076.92	47	506	24070.2	4653.56
EABT9951	0	0	73	0	0	0	1
EABT9952	0	0	4	2	1	0	0
EABT9953	2	5	62	3	1	2	1
EABT9954	1	2	5	0	1	1	0
EABT9955	1	2	6	5	0	0	1
EABT9956	5	5	75	18	0	4	3
EABT9957	0	4	0	0	6	10	24
EABT9958	2	1	16	8	0	0	2
EABT9959	156	340.97	435.88	1283.97	120	22	108
EABT996	30	150	302.13	270	849.54	23	155.93
EABT9960	1	1	14	0	1	1	2
EABT9961	101	271	10	174	16	0	1
EABT9962	0	6	26	0	0	0	1
EABT9963	9	18	3	3	0	0	0
EABT9964	0	0	10	0	0	0	0
EABT9965	0	4	1	7	1	1	2
EABT9966	0	1	8	0	0	3	0
EABT9967	0	2	2	2	2	0	0
EABT9968	6547.91	6873.08	4715.39	11473.1	2892.91	4608.65	3542.03
EABT9969	1	1	4	4	2	1	3
EABT997	66709.9	9450.98	12	1	87.64	138050	815.88
EABT9970	509.96	749.45	853.18	880.54	683.95	481.85	394.03
EABT9971	8	6	14	205	12	6	1
EABT9972	0	0	8	0	1	0	0
EABT9973	3554.63	2654.83	1137	3521.2	1876.91	4517.31	3421.03
EABT9974	10	3	0	10	1	1	1
EABT9975	0	1	5	6	2	1	1
EABT9976	50	50.8	65	126.32	135.11	44	73.62
EABT9977	1	2	12	8	0	3	1
EABT9978	0	0	2	3	0	0	0
EABT9979	1117.26	2165.37	2543.42	2265.65	2361.96	2176.94	1448.88
EABT998	901.78	704	1673	5360.86	2632	190	243
EABT9980	0	0	65	0	0	2	3
EABT9981	1024.89	1249.32	1944.66	1421.43	1008.01	1656.04	2412.95
EABT9982	19563	12637.4	8185.95	9492.03	4008.24	22169	4795.15
EABT9983	135	231	95	61	49.95	108	49
EABT9984	0	1	3	6	0	0	0
EABT9985	1842.01	4282.36	4217.63	5418.07	1692.01	3662.96	3537
EABT9986	5	11.1	7	18.01	1	0	6

EABT9987	0	6	1	0	1	3	0
EABT9988	2007.6	7934.47	10373.9	3630.25	3301.88	37	192.02
EABT9989	35.98	55	135	71.77	24	59	59
EABT999	1	0	6	0	0	1	0
EABT9990	55	141	235	0	0	0	0
EABT9991	202.68	531.01	803.7	1633.62	334.73	249.27	213.97
EABT9992	894.3	1977	1347	331	2402	90	790.04
EABT9993	66	117	513	207	39	45	48
EABT9994	1	0	15	0	0	0	0
EABT9995	55	2	2	0	0	236	0
EABT9996	0	0	6	0	0	0	0
EABT9997	0	2	0	2	2	1	0
EABT9998	0	3	2	0	0	0	0
EABT9999	12	19	15	10	2	11	1
