# Supplementary material for: Transcriptome Analysis of the Emerald Ash Borer (EAB), Agrilus planipennis: De Novo Assembly, Functional Annotation and Comparative Analysis
Source: PLoS One. 2015 Aug 5;10(8):e0134824. doi: 10.1371/journal.pone.0134824 (PMC4526369; doi:10.1371/journal.pone.0134824)
Supplement: S2 Table — (PDF) [file pone.0134824.s005.pdf]

GeneID	logFC	logCPM	PValue	FDR
EABT755	11.74908	9.6579	9.74E-30	2.71E-25
EABT22472	9.287282	8.43323	5.63E-23	7.83E-19
EABT36748	-9.18284	6.2232	2.95E-21	2.74E-17
EABT25456	-8.99773	5.84919	1.96E-20	1.37E-16
EABT11324	8.820758	5.60496	4.87E-20	2.71E-16
EABT29247	8.035621	5.08092	3.78E-18	1.75E-14
EABT34185	-7.56165	7.37156	6.84E-18	2.72E-14
EABT14053	7.318908	10.431	1.64E-17	5.70E-14
EABT26183	7.651784	4.69809	5.21E-17	1.61E-13
EABT25908	-6.74459	9.10225	9.05E-16	2.52E-12
EABT19045	11.86871	2.55657	1.23E-15	3.12E-12
EABT8268	7.28075	3.75048	2.96E-15	6.86E-12
EABT12596	7.605751	3.40997	3.76E-15	8.04E-12
EABT7784	6.562731	5.63476	8.69E-15	1.73E-11
EABT35614	-7.1276	3.77914	1.46E-14	2.71E-11
EABT13825	6.539218	4.95262	1.71E-14	2.98E-11
EABT12292	-6.53725	4.49561	4.62E-14	7.57E-11
EABT15872	6.128019	8.23912	5.95E-14	9.20E-11
EABT18893	6.313614	4.72923	7.84E-14	1.15E-10
EABT1664	-6.08092	7.23007	9.67E-14	1.35E-10
EABT34832	6.152392	5.25801	1.14E-13	1.51E-10
EABT16001	5.946567	9.63315	1.75E-13	2.21E-10
EABT16683	5.953975	6.52561	2.37E-13	2.87E-10
EABT37717	-6.74224	2.83715	6.56E-13	7.60E-10
EABT33188	7.137298	2.22799	7.02E-13	7.81E-10
EABT17190	5.760333	6.51195	8.51E-13	9.11E-10
EABT4529	5.679547	8.571	1.13E-12	1.17E-09
EABT4501	-5.67732	7.27897	1.33E-12	1.32E-09
EABT22594	-10.6416	1.41428	3.75E-12	3.59E-09
EABT17611	5.433954	10.6481	5.37E-12	4.98E-09
EABT35002	5.738627	3.45755	8.04E-12	7.22E-09
EABT19342	6.623627	1.71451	2.06E-11	1.79E-08
EABT29253	-6.02079	2.69995	2.27E-11	1.91E-08
EABT33339	5.137972	10.4442	3.81E-11	3.12E-08
EABT8409	5.420042	3.41914	4.09E-11	3.25E-08
EABT12756	5.859285	2.14704	5.10E-11	3.95E-08
EABT28338	-5.10831	6.35001	6.33E-11	4.66E-08
EABT30011	6.138626	1.94688	6.37E-11	4.66E-08
EABT38034	6.712934	1.24744	7.44E-11	5.30E-08
EABT12947	5.115729	5.07208	7.62E-11	5.30E-08
EABT17685	-6.60507	1.76482	8.96E-11	6.08E-08
EABT6130	7.223198	0.84675	9.25E-11	6.13E-08
EABT10024	5.079896	4.46854	1.41E-10	9.09E-08
EABT19684	5.275849	3.00291	1.67E-10	1.06E-07
EABT6387	5.622959	2.10377	1.76E-10	1.07E-07
EABT15233	4.995578	4.88895	1.77E-10	1.07E-07
EABT2576	10.01941	0.69149	2.24E-10	1.33E-07
EABT30205	9.991422	0.6633	2.59E-10	1.47E-07
EABT381	7.050294	0.67378	2.59E-10	1.47E-07
EABT37192	7.007268	0.63081	3.47E-10	1.93E-07
EABT18315	-4.91452	4.74386	3.79E-10	2.07E-07
EABT1805	4.612718	7.64502	1.25E-09	6.69E-07

EABT29550	4.594162	5.1029	1.94E-09	1.02E-06
EABT23530	-4.67862	4.15804	2.03E-09	1.05E-06
EABT25349	-9.62434	0.45363	2.26E-09	1.15E-06
EABT26575	4.751243	3.1973	2.31E-09	1.15E-06
EABT2018	-4.7749	3.26375	2.37E-09	1.16E-06
EABT29667	-5.66732	1.4154	3.19E-09	1.53E-06
EABT11730	4.448855	5.85936	4.13E-09	1.95E-06
EABT609	4.400084	5.1345	6.25E-09	2.90E-06
EABT28075	-9.42847	0.27196	7.08E-09	3.23E-06
EABT22079	4.342072	6.17698	7.64E-09	3.43E-06
EABT20379	-4.83801	2.28093	8.79E-09	3.88E-06
EABT21928	4.295293	7.37599	9.49E-09	4.12E-06
EABT10723	4.378916	3.95111	1.03E-08	4.42E-06
EABT35701	-4.46965	3.33035	1.34E-08	5.65E-06
EABT8464	4.892571	1.55573	1.45E-08	6.01E-06
EABT5856	4.470874	2.43239	1.77E-08	7.24E-06
EABT17992	-6.46579	0.74381	1.87E-08	7.43E-06
EABT5278	4.387771	3.14108	1.87E-08	7.43E-06
EABT27615	4.195735	6.14601	1.93E-08	7.56E-06
EABT4832	-4.2162	5.45399	1.96E-08	7.56E-06
EABT4155	4.320759	3.44394	2.16E-08	8.24E-06
EABT7074	9.250686	-0.0786	2.29E-08	8.42E-06
EABT29424	6.309558	-0.0607	2.29E-08	8.42E-06
EABT33515	4.153815	7.13123	2.32E-08	8.42E-06
EABT25377	-4.3039	3.65961	2.33E-08	8.42E-06
EABT35119	4.136174	7.85974	2.44E-08	8.70E-06
EABT37650	4.283207	3.34795	2.65E-08	9.35E-06
EABT37797	-4.35755	3.22461	2.70E-08	9.39E-06
EABT22977	5.431876	0.53299	3.67E-08	1.26E-05
EABT8276	-4.52776	2.12236	4.17E-08	1.42E-05
EABT7736	-4.03782	9.93677	4.45E-08	1.49E-05
EABT3549	-9.10006	-0.0299	4.62E-08	1.52E-05
EABT30189	4.030938	10.0202	4.63E-08	1.52E-05
EABT26149	4.022949	8.31481	4.91E-08	1.59E-05
EABT32719	4.030686	5.95187	5.31E-08	1.70E-05
EABT26236	5.367098	0.46959	5.46E-08	1.73E-05
EABT26659	5.350438	0.4533	5.93E-08	1.85E-05
EABT5357	4.00704	5.14743	6.94E-08	2.14E-05
EABT37802	9.048227	-0.2792	7.60E-08	2.32E-05
EABT19441	-4.03766	4.10792	8.02E-08	2.43E-05
EABT33821	4.439516	1.40502	8.24E-08	2.47E-05
EABT32104	4.004883	3.6997	9.93E-08	2.94E-05
EABT35607	-4.3297	2.17042	1.20E-07	3.50E-05
EABT31500	8.964326	-0.362	1.28E-07	3.72E-05
EABT1655	4.211382	2.04663	1.35E-07	3.86E-05
EABT14277	-4.78872	0.98273	1.53E-07	4.33E-05
EABT19413	-3.92909	4.22031	1.56E-07	4.38E-05
EABT10656	5.453147	0.00212	1.63E-07	4.53E-05
EABT36441	3.825315	9.8267	1.64E-07	4.53E-05
EABT28560	4.056382	2.48431	1.73E-07	4.71E-05
EABT13943	3.828375	5.65992	1.85E-07	4.99E-05
EABT11705	8.875243	-0.4495	2.23E-07	5.96E-05
EABT11818	3.762308	7.90305	2.44E-07	6.39E-05
EABT25426	3.762277	7.5268	2.45E-07	6.39E-05

EABT8399	3.89561	3.07027	2.46E-07	6.39E-05
EABT28348	4.455446	0.7749	3.27E-07	8.43E-05
EABT35986	3.868141	2.98552	3.43E-07	8.72E-05
EABT5050	8.780295	-0.5424	3.45E-07	8.72E-05
EABT5613	-3.935	2.66431	3.79E-07	9.51E-05
EABT26472	3.680149	6.69515	4.23E-07	0.00011
EABT28794	-4.42185	1.19182	4.88E-07	0.00012
EABT32331	-4.5877	0.79902	4.90E-07	0.00012
EABT9995	5.25756	-0.1868	5.12E-07	0.00012
EABT35981	3.634705	9.82637	5.21E-07	0.00012
EABT6482	-3.63535	8.97817	5.22E-07	0.00012
EABT1442	3.657442	5.43976	5.34E-07	0.00013
EABT30696	5.772209	-0.5813	5.43E-07	0.00013
EABT11479	3.69453	3.86886	5.49E-07	0.00013
EABT24648	3.718532	3.34873	6.04E-07	0.00014
EABT25617	3.674154	4.11563	6.12E-07	0.00014
EABT31707	-3.63075	5.20052	6.66E-07	0.00015
EABT29765	5.176791	-0.2642	7.42E-07	0.00017
EABT33369	8.64312	-0.6758	7.45E-07	0.00017
EABT15076	-4.74582	0.56326	7.74E-07	0.00017
EABT36850	-4.15614	1.50172	8.02E-07	0.00018
EABT3242	-3.62645	4.16059	8.37E-07	0.00018
EABT16607	3.543516	8.47484	9.03E-07	0.00019
EABT7218	-5.08165	0.34326	9.55E-07	0.0002
EABT32856	4.264957	0.59333	1.00E-06	0.00021
EABT11872	-8.52515	-0.5489	1.03E-06	0.00022
EABT338	5.628181	-0.7181	1.03E-06	0.00022
EABT5630	3.98073	1.20811	1.07E-06	0.00022
EABT24	4.554853	0.39488	1.07E-06	0.00022
EABT23590	-4.45426	0.67805	1.08E-06	0.00022
EABT23350	-3.51867	5.92679	1.18E-06	0.00024
EABT7214	3.490422	8.29398	1.24E-06	0.00025
EABT10729	-3.75546	2.24367	1.37E-06	0.00028
EABT36714	8.530937	-0.7842	1.45E-06	0.00028
EABT29887	8.530937	-0.7842	1.45E-06	0.00028
EABT19333	8.530937	-0.7842	1.45E-06	0.00028
EABT37351	8.530937	-0.7842	1.45E-06	0.00028
EABT34258	3.454424	14.7556	1.52E-06	0.00029
EABT14165	-3.85801	1.83975	1.56E-06	0.0003
EABT5740	3.434601	6.68422	1.80E-06	0.00034
EABT6739	-4.95384	0.22783	1.87E-06	0.00035
EABT11139	3.408609	8.47501	2.00E-06	0.00037
EABT35682	-3.45509	4.52632	2.01E-06	0.00037
EABT20108	-8.38804	-0.6707	2.05E-06	0.00038
EABT6530	3.4242	5.29962	2.09E-06	0.00039
EABT997	3.397049	10.1734	2.13E-06	0.00039
EABT32973	-3.9677	1.1698	2.18E-06	0.0004
EABT32893	3.837395	1.27904	2.22E-06	0.0004
EABT26334	-3.40839	5.63645	2.31E-06	0.00042
EABT32624	-5.60902	-0.0412	2.43E-06	0.00043
EABT3543	3.484522	2.90237	2.70E-06	0.00048
EABT22210	3.355439	8.07759	2.73E-06	0.00048
EABT14577	3.375861	4.23184	3.17E-06	0.00055
EABT9149	3.329227	5.73455	3.49E-06	0.00061

EABT35020	8.366351	-0.9418	3.54E-06	0.00061
EABT13888	5.425223	-0.9083	3.54E-06	0.00061
EABT36091	-3.29459	7.45423	3.94E-06	0.00067
EABT2149	3.282294	8.49973	4.16E-06	0.00071
EABT23951	8.322093	-0.9839	4.28E-06	0.00072
EABT34438	3.355138	3.3016	4.39E-06	0.00074
EABT32421	3.282035	5.52723	4.64E-06	0.00077
EABT21712	3.264546	7.60806	4.64E-06	0.00077
EABT33626	3.253972	8.46088	4.90E-06	0.00081
EABT2280	3.707496	0.95267	4.97E-06	0.00081
EABT33297	3.283353	4.6308	5.07E-06	0.00083
EABT36200	8.276435	-1.0272	5.19E-06	0.00083
EABT21180	5.335307	-0.9916	5.19E-06	0.00083
EABT23916	3.240192	7.89461	5.33E-06	0.00085
EABT9216	4.510466	-0.3519	6.56E-06	0.00104
EABT16962	3.29512	3.06151	6.67E-06	0.00106
EABT36743	3.178473	9.08571	7.53E-06	0.00118
EABT27687	-8.13732	-0.8912	7.72E-06	0.0012
EABT18303	5.239413	-1.0796	7.72E-06	0.0012
EABT31750	-3.18723	5.98356	7.79E-06	0.0012
EABT18586	-3.39727	2.341	8.03E-06	0.00123
EABT25095	3.512122	1.15733	8.12E-06	0.00124
EABT22966	-3.16216	8.936	8.28E-06	0.00126
EABT27863	3.913718	0.04723	8.88E-06	0.00134
EABT31555	3.62016	0.75745	8.93E-06	0.00134
EABT12606	3.144525	8.70337	9.14E-06	0.00137
EABT4577	3.33628	1.9953	9.23E-06	0.00137
EABT11259	-3.51263	1.42682	9.34E-06	0.00138
EABT12828	-5.33206	-0.2887	9.79E-06	0.00144
EABT23384	3.890298	0.02563	9.85E-06	0.00144
EABT25638	-3.14506	5.17411	1.06E-05	0.00155
EABT2432	3.26218	2.39554	1.13E-05	0.00164
EABT17661	4.686931	-0.725	1.14E-05	0.00164
EABT9358	3.562578	0.81896	1.15E-05	0.00164
EABT22553	3.248315	2.28772	1.16E-05	0.00165
EABT9818	8.130092	-1.1648	1.17E-05	0.00165
EABT33047	-8.06719	-0.9524	1.17E-05	0.00165
EABT35545	3.711699	0.58182	1.18E-05	0.00166
EABT28574	3.86649	0.00372	1.21E-05	0.0017
EABT2618	3.215858	2.55417	1.36E-05	0.0019
EABT6124	3.115371	4.02355	1.43E-05	0.00198
EABT19138	-7.99347	-1.0163	1.45E-05	0.00199
EABT19166	3.059064	11.153	1.47E-05	0.00201
EABT25106	3.132523	3.4119	1.50E-05	0.00204
EABT5835	-3.57099	1.11916	1.51E-05	0.00204
EABT8685	3.484329	1.04479	1.51E-05	0.00204
EABT27145	3.702743	0.25723	1.71E-05	0.0023
EABT37746	-3.92804	0.20948	1.79E-05	0.00236
EABT21099	3.742083	0.10534	1.79E-05	0.00236
EABT33063	8.023573	-1.264	1.80E-05	0.00236
EABT22439	-7.95515	-1.0495	1.80E-05	0.00236
EABT27519	5.082445	-1.2219	1.80E-05	0.00236
EABT4239	-4.15007	0.03153	1.87E-05	0.00243
EABT35668	3.019632	6.92795	1.87E-05	0.00243

EABT29183	-3.21674	2.12747	1.91E-05	0.00247
EABT6009	3.013161	7.58584	1.92E-05	0.00248
EABT30513	3.012078	7.729	1.93E-05	0.00248
EABT17415	-4.50448	-0.172	1.94E-05	0.00248
EABT33270	2.987445	8.75629	2.20E-05	0.0028
EABT16853	4.522344	-0.8757	2.25E-05	0.00285
EABT26907	-3.27559	1.58755	2.29E-05	0.00289
EABT32264	3.997206	-0.1294	2.33E-05	0.00292
EABT1129	2.970013	11.618	2.41E-05	0.00301
EABT21318	-2.97091	7.83971	2.43E-05	0.00302
EABT32143	3.970783	-0.1538	2.61E-05	0.00323
EABT6259	4.248033	-0.5945	2.65E-05	0.00326
EABT25706	-2.9565	7.01564	2.66E-05	0.00326
EABT26199	2.9508	8.94272	2.69E-05	0.00328
EABT18203	7.908558	-1.3699	2.82E-05	0.00339
EABT20255	4.96743	-1.3245	2.82E-05	0.00339
EABT26710	4.96743	-1.3245	2.82E-05	0.00339
EABT15313	2.940837	12.964	2.83E-05	0.00339
EABT34145	-4.05947	-0.0477	2.93E-05	0.00349
EABT12114	-3.22811	1.54586	3.01E-05	0.00358
EABT21898	2.931847	6.67653	3.04E-05	0.0036
EABT12878	-5.12107	-0.4748	3.09E-05	0.00363
EABT36451	-2.93023	6.76176	3.09E-05	0.00363
EABT33770	3.555915	0.28953	3.19E-05	0.00373
EABT7283	4.478087	-0.9158	3.23E-05	0.00376
EABT3309	2.949877	4.24294	3.24E-05	0.00376
EABT1184	-3.29917	1.34034	3.44E-05	0.00397
EABT27861	-3.457	0.87566	3.77E-05	0.00433
EABT34658	3.214489	1.04673	3.87E-05	0.00443
EABT4576	4.432428	-0.9569	3.89E-05	0.00443
EABT37907	-2.92773	3.58242	4.48E-05	0.00509
EABT38037	7.847418	-1.4257	4.53E-05	0.00509
EABT12462	-7.74661	-1.2285	4.53E-05	0.00509
EABT35816	4.90629	-1.3785	4.53E-05	0.00509
EABT18661	3.422509	0.45305	4.85E-05	0.0054
EABT7667	-4.3324	-0.3225	4.85E-05	0.0054
EABT24249	-2.86056	4.99473	5.09E-05	0.00564
EABT16895	3.02458	1.79721	5.14E-05	0.00568
EABT5468	2.840933	5.68497	5.27E-05	0.00579
EABT23465	-3.01476	2.19673	5.59E-05	0.00612
EABT36436	4.336534	-1.0427	5.69E-05	0.0062
EABT32367	7.783573	-1.4836	5.79E-05	0.00629
EABT406	-3.92225	-0.1669	5.98E-05	0.00647
EABT19066	-2.99458	2.08563	6.03E-05	0.0065
EABT4299	-3.15072	1.30567	6.28E-05	0.00673
EABT10156	-3.1832	1.23909	6.29E-05	0.00673
EABT28891	2.790578	11.0353	6.40E-05	0.0068
EABT4950	-4.26218	-0.3834	6.41E-05	0.0068
EABT14420	-2.95188	2.14202	6.51E-05	0.00687
EABT3407	2.915623	2.43815	6.52E-05	0.00687
EABT6411	-3.18419	1.02901	6.64E-05	0.00693
EABT2930	2.791144	6.27185	6.64E-05	0.00693
EABT24693	2.82055	4.10056	6.65E-05	0.00693
EABT10896	2.808794	4.53757	6.79E-05	0.00704

EABT3609	-2.87361	3.09796	6.81E-05	0.00704
EABT21762	-2.90264	2.77307	6.83E-05	0.00704
EABT22260	2.801654	4.16095	7.26E-05	0.00746
EABT29041	2.766077	8.62143	7.33E-05	0.00749
EABT22440	2.864129	2.63593	7.82E-05	0.00795
EABT5744	2.753334	8.94259	7.83E-05	0.00795
EABT27435	-4.90351	-0.6644	8.03E-05	0.00812
EABT7622	2.832278	2.88481	8.18E-05	0.00824
EABT23281	3.386385	-0.0289	8.70E-05	0.0087
EABT7744	2.73242	12.0422	8.72E-05	0.0087
EABT32687	2.735909	6.80197	8.72E-05	0.0087
EABT12130	2.855831	2.15086	9.12E-05	0.00906
EABT2698	2.721365	8.79447	9.28E-05	0.00917
EABT33129	2.777921	3.43403	9.29E-05	0.00917
EABT23969	2.714771	8.43301	9.63E-05	0.00937
EABT30112	7.646724	-1.6063	9.64E-05	0.00937
EABT5408	-7.60528	-1.3483	9.64E-05	0.00937
EABT2015	-7.60528	-1.3483	9.64E-05	0.00937
EABT15187	-3.59893	-0.0758	9.81E-05	0.00948
EABT33441	-2.79302	3.2151	9.81E-05	0.00948
EABT7491	2.853662	2.25798	9.88E-05	0.00951
EABT14989	-2.70701	6.88107	0.0001	0.0098
