# Supplementary material for: Transcriptome Analysis of the Emerald Ash Borer (EAB), Agrilus planipennis: De Novo Assembly, Functional Annotation and Comparative Analysis
Source: PLoS One. 2015 Aug 5;10(8):e0134824. doi: 10.1371/journal.pone.0134824 (PMC4526369; doi:10.1371/journal.pone.0134824)
Supplement: S3 Table — (PDF) [file pone.0134824.s006.pdf]

GeneID	logFC	logCPM	PValue	FDR
EABT29342	-10.048	10.12876	1.98E-25	3.10E-21
EABT37744	-9.9419	11.69775	2.62E-25	3.10E-21
EABT26189	-9.9306	11.82234	2.78E-25	3.10E-21
EABT21959	-9.9633	10.04392	3.57E-25	3.10E-21
EABT19583	10.9656	6.836047	4.55E-25	3.15E-21
EABT30570	-9.9488	9.3679	5.70E-25	3.29E-21
EABT25122	-10.61	7.054984	7.98E-25	3.95E-21
EABT7402	-10.246	7.416184	1.51E-24	6.53E-21
EABT15109	-9.7965	8.278252	3.81E-24	1.47E-20
EABT27511	-9.6303	9.137045	4.87E-24	1.69E-20
EABT23189	9.99971	7.016196	1.20E-23	3.78E-20
EABT3117	9.5907	7.885146	2.04E-23	5.88E-20
EABT33636	9.2117	11.29207	4.03E-23	1.07E-19
EABT36884	-9.2945	7.526945	1.67E-22	4.13E-19
EABT35689	-9.0432	9.252138	1.95E-22	4.52E-19
EABT23473	8.95537	12.19048	2.12E-22	4.60E-19
EABT31611	-9.1499	7.894449	2.50E-22	4.86E-19
EABT33544	-9.2799	7.291507	2.52E-22	4.86E-19
EABT10343	9.48143	6.33142	6.49E-22	1.18E-18
EABT23805	8.87755	6.803969	4.72E-21	7.87E-18
EABT29209	-8.5021	12.01645	4.76E-21	7.87E-18
EABT1631	-9.4461	5.636057	5.45E-21	8.59E-18
EABT13844	9.11177	5.962614	8.28E-21	1.25E-17
EABT37208	8.64161	6.801457	1.70E-20	2.46E-17
EABT21146	-8.6253	6.55663	2.61E-20	3.62E-17
EABT25377	9.36247	4.835835	1.30E-19	1.72E-16
EABT2537	-8.5938	5.767566	1.34E-19	1.72E-16
EABT30187	8.44761	6.097662	1.45E-19	1.79E-16
EABT1771	-9.1	4.97825	1.70E-19	2.03E-16
EABT5630	-10.229	4.23305	3.21E-19	3.71E-16
EABT18284	-8.3461	5.7807	4.27E-19	4.77E-16
EABT7825	-7.8595	7.697735	8.11E-19	8.79E-16
EABT10097	-7.7926	7.186896	1.67E-18	1.76E-15
EABT13522	7.82026	6.465903	2.70E-18	2.75E-15
EABT9643	-7.5988	8.767327	3.05E-18	3.03E-15
EABT33016	-7.5271	10.48044	4.05E-18	3.90E-15
EABT33246	7.68747	6.694752	4.75E-18	4.45E-15
EABT6345	7.68381	6.223819	7.71E-18	7.03E-15
EABT37633	7.39972	14.49132	8.81E-18	7.83E-15
EABT34539	-7.6165	6.104881	1.27E-17	1.10E-14
EABT10206	-7.3943	8.187651	1.36E-17	1.15E-14
EABT12596	-7.5442	6.294585	1.57E-17	1.30E-14
EABT24024	-12.354	3.188918	3.76E-17	3.03E-14
EABT37516	-7.2629	6.805721	5.74E-17	4.52E-14
EABT5960	8.40073	3.878611	9.31E-17	7.17E-14
EABT17661	-7.1236	7.287447	1.08E-16	8.15E-14
EABT17893	7.08513	8.030487	1.12E-16	8.25E-14
EABT23684	-7.0069	8.69962	1.60E-16	1.16E-13
EABT5319	7.26648	5.502996	1.94E-16	1.37E-13
EABT38020	9.27518	3.279938	2.19E-16	1.52E-13
EABT5002	-6.9241	9.638693	2.57E-16	1.75E-13
EABT17681	-6.9064	8.123612	3.46E-16	2.31E-13

EABT4817	7.27445	4.825261	5.38E-16	3.52E-13
EABT18352	11.9213	2.75942	6.94E-16	4.43E-13
EABT3561	6.86061	6.910129	7.02E-16	4.43E-13
EABT31380	-8.3824	3.310614	1.06E-15	6.54E-13
EABT22787	-7.1182	4.780376	1.25E-15	7.62E-13
EABT23350	6.70069	7.807433	1.48E-15	8.86E-13
EABT15633	6.53054	10.19594	3.37E-15	1.98E-12
EABT34182	-7.4793	3.679637	3.75E-15	2.14E-12
EABT29991	7.63769	3.520475	3.76E-15	2.14E-12
EABT12248	8.19304	3.118725	3.89E-15	2.18E-12
EABT20090	8.84283	2.851053	4.04E-15	2.22E-12
EABT15582	8.16484	3.090799	4.70E-15	2.55E-12
EABT14989	-6.4532	7.509563	7.83E-15	4.18E-12
EABT21639	6.50259	6.020161	1.06E-14	5.59E-12
EABT10562	7.45585	3.340469	1.28E-14	6.53E-12
EABT509	8.67049	2.680414	1.28E-14	6.53E-12
EABT18342	11.4783	2.32048	1.34E-14	6.66E-12
EABT14354	8.66099	2.671015	1.36E-14	6.66E-12
EABT37963	-8.6594	2.673467	1.36E-14	6.66E-12
EABT3541	-7.6538	3.142219	1.42E-14	6.86E-12
EABT36384	-7.9856	2.917581	1.53E-14	7.29E-12
EABT5069	7.97224	2.900146	1.71E-14	8.02E-12
EABT19694	7.61795	3.102494	1.86E-14	8.59E-12
EABT23447	-6.2831	9.167024	1.98E-14	9.01E-12
EABT28053	6.52933	5.079075	2.00E-14	9.01E-12
EABT29740	7.59897	3.083725	2.11E-14	9.38E-12
EABT12790	11.412	2.254882	2.14E-14	9.39E-12
EABT34986	-11.378	2.221501	2.61E-14	1.13E-11
EABT35333	6.87984	3.744612	3.44E-14	1.47E-11
EABT21252	7.30654	3.192836	3.48E-14	1.47E-11
EABT10226	6.93802	3.613361	3.77E-14	1.56E-11
EABT22524	7.0263	3.483355	3.79E-14	1.56E-11
EABT33237	-7.4995	2.989729	4.02E-14	1.64E-11
EABT29755	6.36459	5.121669	4.92E-14	1.98E-11
EABT23654	7.81182	2.741554	4.99E-14	1.98E-11
EABT9116	-6.149	8.750174	5.03E-14	1.98E-11
EABT21318	6.3804	4.932056	5.48E-14	2.13E-11
EABT20648	8.44922	2.461632	5.59E-14	2.15E-11
EABT22347	-6.1053	9.455208	6.37E-14	2.43E-11
EABT25962	6.07447	8.803567	8.11E-14	3.06E-11
EABT27441	-6.0644	7.664021	9.78E-14	3.65E-11
EABT35227	6.066	7.498339	1.02E-13	3.78E-11
EABT14709	-8.3462	2.364045	1.11E-13	3.99E-11
EABT38034	-8.3462	2.364045	1.11E-13	3.99E-11
EABT426	-7.3411	2.833269	1.16E-13	4.15E-11
EABT20795	-6.0815	6.220263	1.30E-13	4.59E-11
EABT28206	6.40514	4.169704	1.34E-13	4.71E-11
EABT13755	-6.0173	7.164573	1.46E-13	5.07E-11
EABT20287	-6.0416	6.3709	1.57E-13	5.40E-11
EABT34914	6.20824	4.523107	2.31E-13	7.84E-11
EABT21822	-6.5847	3.457798	2.41E-13	8.11E-11
EABT32285	7.22174	2.711296	2.61E-13	8.70E-11
EABT19441	-6.0209	5.235728	3.30E-13	1.09E-10
EABT10044	6.80679	3.01107	3.52E-13	1.15E-10

EABT19287	-10.982	1.830587	3.58E-13	1.16E-10
EABT3680	6.94025	2.831475	4.00E-13	1.28E-10
EABT23767	6.19524	4.055141	4.72E-13	1.50E-10
EABT20145	-6.2547	3.821969	5.07E-13	1.60E-10
EABT26924	10.9157	1.765171	5.66E-13	1.77E-10
EABT25922	8.09673	2.113985	5.91E-13	1.83E-10
EABT2822	6.26089	3.709826	6.22E-13	1.91E-10
EABT11017	-7.4286	2.367928	6.32E-13	1.92E-10
EABT22920	6.71572	2.921421	6.44E-13	1.94E-10
EABT25798	7.41696	2.352173	6.83E-13	2.04E-10
EABT9990	10.8794	1.729393	7.18E-13	2.13E-10
EABT28682	7.40496	2.340361	7.40E-13	2.17E-10
EABT34922	-5.8015	6.198151	7.70E-13	2.24E-10
EABT12360	8.02942	2.04774	9.19E-13	2.66E-10
EABT33649	10.8294	1.680266	9.94E-13	2.85E-10
EABT28560	-6.7847	2.682849	1.10E-12	3.14E-10
EABT25707	6.29842	3.338463	1.13E-12	3.18E-10
EABT22113	6.28831	3.328533	1.21E-12	3.38E-10
EABT12065	-6.0767	3.752578	1.38E-12	3.83E-10
EABT37852	-7.9457	1.969609	1.51E-12	4.16E-10
EABT24329	6.95319	2.447001	1.54E-12	4.20E-10
EABT28544	-7.9353	1.959379	1.62E-12	4.38E-10
EABT11677	7.28349	2.220907	1.69E-12	4.54E-10
EABT1807	-5.5915	8.144201	2.13E-12	5.69E-10
EABT34523	6.68469	2.58017	2.16E-12	5.72E-10
EABT6976	7.89542	1.916011	2.21E-12	5.81E-10
EABT31159	5.54675	11.93069	2.53E-12	6.60E-10
EABT18048	5.80654	4.311532	2.71E-12	7.00E-10
EABT31178	5.63838	5.66559	2.73E-12	7.00E-10
EABT33188	-5.645	5.365375	2.95E-12	7.52E-10
EABT1079	-5.6411	5.38605	2.99E-12	7.57E-10
EABT16702	10.6541	1.50796	3.11E-12	7.81E-10
EABT33986	5.71648	4.663993	3.20E-12	7.99E-10
EABT11182	5.53803	7.009601	3.50E-12	8.67E-10
EABT12249	7.82344	1.845343	3.54E-12	8.70E-10
EABT1269	-5.5053	7.817685	3.89E-12	9.50E-10
EABT2177	-5.614	5.121686	4.11E-12	9.97E-10
EABT26275	-5.5736	5.502924	4.28E-12	1.03E-09
EABT3272	-6.0001	3.334607	4.33E-12	1.04E-09
EABT22057	-5.8551	3.725678	4.41E-12	1.05E-09
EABT17249	10.5955	1.450519	4.54E-12	1.07E-09
EABT32534	6.56754	2.465233	4.66E-12	1.09E-09
EABT6832	7.77142	1.794309	4.96E-12	1.15E-09
EABT419	7.77142	1.794309	4.96E-12	1.15E-09
EABT22662	7.11691	2.05738	5.03E-12	1.16E-09
EABT2989	-5.444	7.996318	5.70E-12	1.30E-09
EABT35042	7.08717	2.028228	6.11E-12	1.38E-09
EABT20850	7.08717	2.028228	6.11E-12	1.38E-09
EABT32629	5.44323	7.256068	6.23E-12	1.39E-09
EABT17841	7.73567	1.759255	6.26E-12	1.39E-09
EABT22440	-5.4503	6.764703	6.28E-12	1.39E-09
EABT734	-5.4786	5.897231	6.58E-12	1.44E-09
EABT3893	6.50712	2.406028	6.92E-12	1.51E-09
EABT34728	-6.0665	2.948984	7.53E-12	1.63E-09

EABT6530	6.34053	2.553127	7.62E-12	1.64E-09
EABT10550	-5.5396	4.692064	8.61E-12	1.84E-09
EABT35797	-10.483	1.340766	9.12E-12	1.94E-09
EABT31741	7.67404	1.69887	9.34E-12	1.97E-09
EABT2317	-5.8329	3.294484	1.03E-11	2.16E-09
EABT2938	5.33024	10.27792	1.07E-11	2.24E-09
EABT30828	6.98875	1.931825	1.16E-11	2.41E-09
EABT9950	5.46615	4.884007	1.19E-11	2.46E-09
EABT33360	-5.3165	8.161892	1.29E-11	2.65E-09
EABT34611	-6.9371	1.885676	1.54E-11	3.14E-09
EABT6942	-5.2712	9.826277	1.59E-11	3.22E-09
EABT4977	-10.391	1.251353	1.64E-11	3.30E-09
EABT36366	6.93412	1.878376	1.65E-11	3.30E-09
EABT10777	-5.3057	6.564759	1.66E-11	3.30E-09
EABT30289	-6.9148	1.863903	1.78E-11	3.52E-09
EABT24248	5.298	6.553041	1.79E-11	3.53E-09
EABT18074	10.3782	1.237808	1.83E-11	3.57E-09
EABT26312	7.5696	1.596667	1.83E-11	3.57E-09
EABT30137	-5.252	8.422524	1.94E-11	3.75E-09
EABT928	5.25129	7.865605	2.00E-11	3.85E-09
EABT33950	10.3608	1.220731	2.04E-11	3.92E-09
EABT21886	-5.9134	2.799298	2.06E-11	3.92E-09
EABT24951	7.53535	1.563185	2.28E-11	4.31E-09
EABT27436	7.53535	1.563185	2.28E-11	4.31E-09
EABT31851	-5.4321	4.162434	2.52E-11	4.72E-09
EABT28786	5.2668	5.899523	2.57E-11	4.78E-09
EABT219	-5.2574	5.996579	2.58E-11	4.78E-09
EABT5526	-5.3138	4.939186	2.80E-11	5.16E-09
EABT22010	-5.795	2.850023	3.04E-11	5.57E-09
EABT4769	10.2979	1.159315	3.05E-11	5.57E-09
EABT22977	-5.3543	4.435831	3.09E-11	5.61E-09
EABT22167	-5.317	4.546758	3.53E-11	6.37E-09
EABT9391	10.2701	1.132171	3.64E-11	6.54E-09
EABT35422	6.80003	1.747363	3.93E-11	7.02E-09
EABT2000	10.2512	1.113788	4.10E-11	7.29E-09
EABT16270	6.78775	1.735388	4.25E-11	7.52E-09
EABT9733	5.61646	3.078936	4.36E-11	7.67E-09
EABT3549	-5.1681	6.027825	4.50E-11	7.87E-09
EABT24897	-5.6815	2.88828	4.64E-11	8.08E-09
EABT5797	10.2128	1.076304	5.23E-11	9.07E-09
EABT6943	-5.0869	9.297718	5.37E-11	9.27E-09
EABT23399	-5.0759	8.886821	5.80E-11	9.96E-09
EABT24983	5.25776	4.298333	6.18E-11	1.06E-08
EABT36698	10.1833	1.047537	6.30E-11	1.07E-08
EABT29598	6.16981	2.076468	6.37E-11	1.08E-08
EABT22631	6.01347	2.2337	6.56E-11	1.10E-08
EABT25720	7.3664	1.398293	6.74E-11	1.13E-08
EABT20310	-5.546	3.014705	6.75E-11	1.13E-08
EABT2846	-5.4889	3.072806	7.98E-11	1.32E-08
EABT26957	10.1429	1.008267	8.12E-11	1.34E-08
EABT3532	5.59544	2.800153	8.25E-11	1.36E-08
EABT13830	5.96909	2.190494	8.74E-11	1.43E-08
EABT24247	6.33253	1.839733	8.96E-11	1.46E-08
EABT12123	5.11049	5.089624	9.15E-11	1.48E-08

EABT16748	10.1223	0.988224	9.25E-11	1.49E-08
EABT27500	5.00777	8.093902	9.67E-11	1.55E-08
EABT18154	5.05368	5.830826	9.99E-11	1.60E-08
EABT7181	6.09482	2.003451	1.03E-10	1.64E-08
EABT19135	-5.0171	6.391559	1.08E-10	1.71E-08
EABT5020	5.73018	2.428486	1.09E-10	1.71E-08
EABT20923	10.0909	0.957627	1.13E-10	1.77E-08
EABT33854	5.50322	2.845418	1.15E-10	1.79E-08
EABT11253	7.27803	1.312232	1.18E-10	1.84E-08
EABT7645	-6.2716	1.785017	1.26E-10	1.94E-08
EABT18209	5.07173	4.802391	1.33E-10	2.05E-08
EABT8089	5.02827	5.222628	1.43E-10	2.19E-08
EABT34042	5.27453	3.400395	1.44E-10	2.21E-08
EABT25693	-7.2366	1.276312	1.46E-10	2.22E-08
EABT24211	-4.932	9.67634	1.48E-10	2.24E-08
EABT2246	7.23599	1.27135	1.54E-10	2.32E-08
EABT11795	10.0369	0.905145	1.58E-10	2.37E-08
EABT34253	-4.9523	6.375784	1.64E-10	2.45E-08
EABT29678	6.57572	1.528891	1.65E-10	2.46E-08
EABT11139	5.7343	2.216561	1.91E-10	2.84E-08
EABT10407	-5.0819	4.091626	1.96E-10	2.89E-08
EABT22425	6.54689	1.50087	1.98E-10	2.91E-08
EABT11638	5.97725	1.889198	2.19E-10	3.21E-08
EABT19568	6.18761	1.698839	2.26E-10	3.29E-08
EABT26255	7.17501	1.212095	2.27E-10	3.29E-08
EABT25103	9.96933	0.839538	2.41E-10	3.48E-08
EABT706	5.01712	4.286053	2.51E-10	3.62E-08
EABT16337	9.95775	0.828307	2.59E-10	3.71E-08
EABT15350	7.14806	1.185936	2.68E-10	3.82E-08
EABT6946	-7.1401	1.182678	2.68E-10	3.82E-08
EABT4975	9.94608	0.816989	2.78E-10	3.94E-08
EABT28174	-5.195	3.169185	2.93E-10	4.13E-08
EABT29579	-9.9301	0.802489	2.99E-10	4.20E-08
EABT27745	-5.4394	2.504421	3.06E-10	4.28E-08
EABT10943	9.9105	0.78249	3.47E-10	4.81E-08
EABT25705	9.9105	0.78249	3.47E-10	4.81E-08
EABT3086	-5.3672	2.583159	3.56E-10	4.92E-08
EABT7110	-7.0941	1.13802	3.58E-10	4.93E-08
EABT9029	5.54251	2.246391	3.72E-10	5.10E-08
EABT23804	-4.7898	9.065666	3.75E-10	5.12E-08
EABT22002	5.74002	1.968057	3.80E-10	5.17E-08
EABT35209	4.94276	4.280907	3.89E-10	5.27E-08
EABT28730	7.08316	1.122988	4.03E-10	5.44E-08
EABT19766	-5.8711	1.790813	4.09E-10	5.50E-08
EABT26432	-4.8577	5.114844	4.14E-10	5.54E-08
EABT662	5.86739	1.782675	4.42E-10	5.89E-08
EABT32150	7.06407	1.104486	4.55E-10	6.04E-08
EABT4072	4.88501	4.54549	4.66E-10	6.17E-08
EABT25906	7.05442	1.095146	4.83E-10	6.37E-08
EABT35702	5.84925	1.765112	4.96E-10	6.51E-08
EABT24462	4.80482	5.464857	5.23E-10	6.85E-08
EABT24863	4.7668	6.633634	5.26E-10	6.85E-08
EABT36091	4.77468	5.872742	5.69E-10	7.39E-08
EABT19673	-5.1475	2.84729	6.08E-10	7.86E-08

EABT22520	-4.842	4.455113	6.15E-10	7.90E-08
EABT16222	4.80792	4.935892	6.15E-10	7.90E-08
EABT29397	4.75642	6.002054	6.22E-10	7.96E-08
EABT4882	9.81107	0.686246	6.41E-10	8.14E-08
EABT32769	6.3606	1.320252	6.43E-10	8.14E-08
EABT33967	6.3606	1.320252	6.43E-10	8.14E-08
EABT18242	-5.2563	2.476008	7.25E-10	9.14E-08
EABT27466	-5.3024	2.371861	7.38E-10	9.27E-08
EABT22448	6.98506	1.028013	7.44E-10	9.32E-08
EABT38151	5.29867	2.363816	7.57E-10	9.45E-08
EABT260	6.32707	1.287822	7.93E-10	9.86E-08
EABT34094	-4.6762	8.330686	8.13E-10	1.01E-07
EABT13267	6.96462	1.008244	8.45E-10	1.04E-07
EABT17779	4.69649	6.397594	8.46E-10	1.04E-07
EABT14620	6.31001	1.27133	8.83E-10	1.08E-07
EABT812	6.95428	0.998257	9.01E-10	1.10E-07
EABT10756	6.95428	0.998257	9.01E-10	1.10E-07
EABT25879	5.96111	1.479473	9.48E-10	1.15E-07
EABT30162	9.74525	0.622661	9.59E-10	1.16E-07
EABT34880	6.94388	0.988201	9.61E-10	1.16E-07
EABT38050	-4.6758	6.013416	1.00E-09	1.20E-07
EABT5258	-9.7275	0.606628	1.04E-09	1.25E-07
EABT1893	5.37156	2.08117	1.11E-09	1.32E-07
EABT17767	4.88672	3.417329	1.11E-09	1.32E-07
EABT8921	9.71806	0.596421	1.13E-09	1.34E-07
EABT34515	-4.999	2.893707	1.17E-09	1.38E-07
EABT7115	4.76706	4.143793	1.18E-09	1.39E-07
EABT447	6.90148	0.947259	1.25E-09	1.46E-07
EABT82	-4.6673	5.18514	1.29E-09	1.51E-07
EABT18649	4.92702	3.064604	1.35E-09	1.57E-07
EABT13750	4.71167	4.352209	1.44E-09	1.67E-07
EABT11818	9.67629	0.556145	1.46E-09	1.69E-07
EABT29419	5.52389	1.759196	1.49E-09	1.72E-07
EABT1126	6.86885	0.915771	1.53E-09	1.76E-07
EABT34258	4.57187	8.375217	1.54E-09	1.76E-07
EABT25183	-4.616	5.58223	1.59E-09	1.81E-07
EABT33325	-4.7444	3.806451	1.66E-09	1.89E-07
EABT7437	5.50536	1.741343	1.68E-09	1.90E-07
EABT24387	-4.5719	6.517013	1.78E-09	2.01E-07
EABT14592	4.88155	3.020875	1.80E-09	2.03E-07
EABT8406	6.19391	1.159273	1.82E-09	2.04E-07
EABT33013	-6.1857	1.156071	1.82E-09	2.04E-07
EABT10588	5.48659	1.723267	1.89E-09	2.11E-07
EABT5965	4.74485	3.588501	2.04E-09	2.27E-07
EABT21478	9.61865	0.500632	2.07E-09	2.29E-07
EABT35398	9.61865	0.500632	2.07E-09	2.29E-07
EABT11258	4.52132	8.702412	2.12E-09	2.34E-07
EABT10093	5.82889	1.351951	2.17E-09	2.38E-07
EABT26740	4.56344	5.590214	2.25E-09	2.46E-07
EABT27882	4.72916	3.573444	2.26E-09	2.46E-07
EABT9140	9.60387	0.486413	2.26E-09	2.46E-07
EABT31572	6.15622	1.122966	2.30E-09	2.49E-07
EABT16854	6.14664	1.113745	2.44E-09	2.63E-07
EABT7358	-5.4335	1.67682	2.50E-09	2.69E-07

EABT15595	6.137	1.104465	2.59E-09	2.78E-07
EABT9267	4.48546	11.28718	2.64E-09	2.82E-07
EABT18207	4.59309	4.459815	2.75E-09	2.93E-07
EABT15997	5.57538	1.500835	2.77E-09	2.95E-07
EABT33057	6.76626	0.816963	2.87E-09	3.04E-07
EABT37697	9.55861	0.442897	2.98E-09	3.13E-07
EABT26149	9.55861	0.442897	2.98E-09	3.13E-07
EABT3252	-9.5544	0.440032	2.98E-09	3.13E-07
EABT1379	9.5432	0.428095	3.26E-09	3.42E-07
EABT2724	5.39563	1.635783	3.34E-09	3.48E-07
EABT3225	9.52762	0.41314	3.58E-09	3.70E-07
EABT13892	9.52762	0.41314	3.58E-09	3.70E-07
EABT34429	-9.5234	0.410294	3.58E-09	3.70E-07
EABT5875	-9.5234	0.410294	3.58E-09	3.70E-07
EABT25259	-4.7399	3.031952	3.63E-09	3.74E-07
EABT14383	-4.483	5.44416	3.72E-09	3.81E-07
EABT22271	6.07775	1.047493	3.73E-09	3.81E-07
EABT4812	4.46867	5.802353	3.87E-09	3.95E-07
EABT35545	4.74507	2.962991	3.90E-09	3.97E-07
EABT34900	9.51188	0.398028	3.94E-09	3.98E-07
EABT4470	9.51188	0.398028	3.94E-09	3.98E-07
EABT19030	6.06764	1.037775	3.97E-09	3.99E-07
EABT5600	6.06764	1.037775	3.97E-09	3.99E-07
EABT33270	-4.4343	7.332019	4.01E-09	4.02E-07
EABT38071	5.0962	2.0034	4.02E-09	4.02E-07
EABT28468	5.72768	1.254627	4.06E-09	4.05E-07
EABT8278	5.50761	1.435723	4.23E-09	4.20E-07
EABT20273	4.47414	5.120965	4.40E-09	4.36E-07
EABT21683	6.69358	0.74712	4.47E-09	4.42E-07
EABT22802	-4.4245	6.392095	4.55E-09	4.48E-07
EABT23646	9.47986	0.367321	4.77E-09	4.67E-07
EABT32201	9.47986	0.367321	4.77E-09	4.67E-07
EABT3800	5.70123	1.229234	4.78E-09	4.67E-07
EABT14771	4.87239	2.359891	5.08E-09	4.95E-07
EABT19657	6.02645	0.998234	5.11E-09	4.95E-07
EABT5493	6.02645	0.998234	5.11E-09	4.95E-07
EABT13992	4.48935	4.383213	5.26E-09	5.05E-07
EABT32132	9.46358	0.351719	5.26E-09	5.05E-07
EABT7599	9.46358	0.351719	5.26E-09	5.05E-07
EABT29085	5.68332	1.212054	5.34E-09	5.12E-07
EABT22649	5.46059	1.390623	5.66E-09	5.40E-07
EABT11040	-4.7945	2.507982	5.74E-09	5.47E-07
EABT31677	9.44712	0.335946	5.80E-09	5.50E-07
EABT11880	9.44712	0.335946	5.80E-09	5.50E-07
EABT25043	4.67644	2.89733	6.01E-09	5.68E-07
EABT17034	5.99477	0.967852	6.20E-09	5.84E-07
EABT18978	-5.6478	1.182719	6.32E-09	5.94E-07
EABT34004	6.63009	0.686219	6.57E-09	6.16E-07
EABT10976	5.98405	0.95758	6.62E-09	6.19E-07
EABT36751	5.64682	1.177068	6.68E-09	6.23E-07
EABT14048	-4.895	2.12881	6.93E-09	6.44E-07
EABT21378	-4.4618	4.090515	6.99E-09	6.48E-07
EABT17980	9.41361	0.303874	7.08E-09	6.53E-07
EABT36274	9.41361	0.303874	7.08E-09	6.53E-07

EABT14102	6.61704	0.673724	7.11E-09	6.54E-07
EABT15874	4.99931	1.910601	7.36E-09	6.76E-07
EABT27086	-4.4118	4.560444	7.50E-09	6.86E-07
EABT12597	4.88893	2.118496	7.58E-09	6.90E-07
EABT224	-4.3413	6.732689	7.59E-09	6.90E-07
EABT17489	-4.7498	2.465241	7.60E-09	6.90E-07
EABT19996	5.26223	1.507875	7.64E-09	6.90E-07
EABT18349	4.52444	3.467018	7.64E-09	6.90E-07
EABT1175	9.39656	0.287567	7.83E-09	7.02E-07
EABT8219	9.39656	0.287567	7.83E-09	7.02E-07
EABT12949	9.39656	0.287567	7.83E-09	7.02E-07
EABT29799	4.59726	3.020848	7.86E-09	7.02E-07
EABT23041	-4.4079	4.375641	8.30E-09	7.40E-07
EABT33894	4.79713	2.287925	8.35E-09	7.42E-07
EABT1006	4.3132	7.160825	8.60E-09	7.63E-07
EABT17880	4.4214	4.126668	8.85E-09	7.83E-07
EABT15979	6.5772	0.635577	9.04E-09	7.98E-07
EABT15946	-4.2933	8.177385	9.10E-09	8.01E-07
EABT7566	-5.582	1.119806	9.45E-09	8.30E-07
EABT34278	-4.8014	2.173759	9.62E-09	8.40E-07
EABT37149	9.36185	0.254388	9.62E-09	8.40E-07
EABT18151	6.56367	0.622633	9.80E-09	8.54E-07
EABT14118	-5.9099	0.891321	9.89E-09	8.60E-07
EABT8400	5.58064	1.113724	1.00E-08	8.69E-07
EABT21090	-4.308	5.76482	1.02E-08	8.86E-07
EABT13792	-4.3101	5.663685	1.03E-08	8.88E-07
EABT10603	-4.2713	8.609035	1.04E-08	8.95E-07
EABT4102	-4.2907	6.421757	1.05E-08	8.97E-07
EABT861	-4.3863	4.12297	1.06E-08	9.07E-07
EABT5585	9.34418	0.237509	1.07E-08	9.07E-07
EABT37012	-9.34	0.234783	1.07E-08	9.07E-07
EABT4822	-9.34	0.234783	1.07E-08	9.07E-07
EABT1862	-4.7167	2.328842	1.08E-08	9.16E-07
EABT28885	5.35314	1.287783	1.10E-08	9.27E-07
EABT4002	-4.2759	6.654393	1.15E-08	9.68E-07
EABT5244	9.32628	0.220429	1.19E-08	9.94E-07
EABT32358	9.32628	0.220429	1.19E-08	9.94E-07
EABT33874	4.99226	1.717161	1.19E-08	9.94E-07
EABT33789	-4.2815	5.708667	1.22E-08	1.02E-06
EABT23167	-4.4909	3.098753	1.25E-08	1.04E-06
EABT8312	9.30816	0.203145	1.32E-08	1.09E-06
EABT10075	9.30816	0.203145	1.32E-08	1.09E-06
EABT30564	-4.5449	2.776266	1.35E-08	1.12E-06
EABT29203	6.50824	0.569667	1.37E-08	1.13E-06
EABT11594	6.50824	0.569667	1.37E-08	1.13E-06
EABT5462	-4.4938	2.987358	1.38E-08	1.13E-06
EABT17153	5.8606	0.839488	1.40E-08	1.15E-06
EABT35772	5.8606	0.839488	1.40E-08	1.15E-06
EABT31237	4.26211	5.65438	1.42E-08	1.16E-06
EABT37128	4.51136	2.875657	1.44E-08	1.17E-06
EABT36333	-4.4023	3.479576	1.45E-08	1.18E-06
EABT2263	9.28981	0.185651	1.47E-08	1.19E-06
EABT19436	4.32337	4.179535	1.52E-08	1.23E-06
EABT1386	4.20896	9.018966	1.53E-08	1.24E-06

EABT9562	-4.3429	3.893701	1.54E-08	1.24E-06
EABT3001	-4.271	4.877464	1.57E-08	1.26E-06
EABT8237	5.83697	0.816938	1.61E-08	1.29E-06
EABT35233	5.83697	0.816938	1.61E-08	1.29E-06
EABT7736	4.18716	8.63996	1.77E-08	1.41E-06
EABT9149	6.46522	0.528626	1.77E-08	1.41E-06
EABT694	5.01242	1.521868	1.79E-08	1.42E-06
EABT20136	4.56708	2.475954	1.80E-08	1.42E-06
EABT21006	9.2524	0.150015	1.83E-08	1.44E-06
EABT27207	9.2524	0.150015	1.83E-08	1.44E-06
EABT19454	9.2524	0.150015	1.83E-08	1.44E-06
EABT34307	4.74181	1.97819	1.89E-08	1.48E-06
EABT30372	4.66225	2.15938	1.93E-08	1.51E-06
EABT13621	5.25552	1.194647	1.99E-08	1.55E-06
EABT14334	5.80079	0.782439	2.01E-08	1.56E-06
EABT36205	5.45961	0.998212	2.09E-08	1.63E-06
EABT32758	6.43582	0.500602	2.10E-08	1.63E-06
EABT1315	9.21399	0.113476	2.29E-08	1.77E-06
EABT28492	6.42089	0.486383	2.30E-08	1.77E-06
EABT32643	5.08227	1.336141	2.30E-08	1.77E-06
EABT7605	5.08227	1.336141	2.30E-08	1.77E-06
EABT13676	4.70941	1.947376	2.30E-08	1.77E-06
EABT25338	4.50654	2.50445	2.32E-08	1.78E-06
EABT14956	5.43842	0.978027	2.38E-08	1.81E-06
EABT15919	4.20899	4.684245	2.46E-08	1.87E-06
EABT33026	5.21831	1.159231	2.49E-08	1.89E-06
EABT26762	5.76368	0.747094	2.51E-08	1.90E-06
EABT9951	9.19439	0.094854	2.56E-08	1.93E-06
EABT3347	9.19439	0.094854	2.56E-08	1.93E-06
EABT15007	9.19439	0.094854	2.56E-08	1.93E-06
EABT23178	9.19439	0.094854	2.56E-08	1.93E-06
EABT28689	5.75109	0.735118	2.70E-08	2.03E-06
EABT33871	6.39055	0.457519	2.75E-08	2.05E-06
EABT17741	6.39055	0.457519	2.75E-08	2.05E-06
EABT24896	6.39055	0.457519	2.75E-08	2.05E-06
EABT28167	-4.1257	7.080211	2.80E-08	2.08E-06
EABT26684	5.04855	1.304071	2.83E-08	2.10E-06
EABT29916	4.12364	6.788078	2.86E-08	2.10E-06
EABT13692	-4.7136	1.80809	2.86E-08	2.10E-06
EABT10285	-4.4235	2.661057	2.87E-08	2.10E-06
EABT28069	9.17453	0.075988	2.87E-08	2.10E-06
EABT19439	9.17453	0.075988	2.87E-08	2.10E-06
EABT27094	9.17453	0.075988	2.87E-08	2.10E-06
EABT29669	9.17453	0.075988	2.87E-08	2.10E-06
EABT9563	5.40602	0.947212	2.89E-08	2.11E-06
EABT19573	-4.7687	1.695477	2.90E-08	2.11E-06
EABT23017	5.73839	0.723041	2.92E-08	2.12E-06
EABT13563	5.18977	1.132087	2.96E-08	2.15E-06
EABT31998	5.72558	0.710862	3.15E-08	2.28E-06
EABT26715	4.25708	3.558176	3.16E-08	2.28E-06
EABT27161	-4.5715	2.077731	3.20E-08	2.31E-06
EABT19061	9.15439	0.056872	3.23E-08	2.31E-06
EABT31927	9.15439	0.056872	3.23E-08	2.31E-06
EABT3440	9.15439	0.056872	3.23E-08	2.31E-06

EABT35955	4.09814	7.597734	3.23E-08	2.31E-06
EABT34423	4.10306	6.855831	3.24E-08	2.31E-06
EABT9585	-4.3897	2.698451	3.25E-08	2.32E-06
EABT15343	-5.3758	0.923278	3.29E-08	2.34E-06
EABT33844	-6.3516	0.42527	3.30E-08	2.34E-06
EABT25803	-4.6397	1.885786	3.36E-08	2.38E-06
EABT33852	4.90614	1.42081	3.42E-08	2.41E-06
EABT24569	-4.1676	4.366023	3.42E-08	2.41E-06
EABT1621	4.07917	9.352152	3.45E-08	2.43E-06
EABT23677	5.01402	1.271271	3.48E-08	2.44E-06
EABT25309	5.01402	1.271271	3.48E-08	2.44E-06
EABT15126	-4.085	7.516743	3.51E-08	2.45E-06
EABT34093	9.13396	0.0375	3.63E-08	2.52E-06
EABT5548	9.13396	0.0375	3.63E-08	2.52E-06
EABT18774	9.13396	0.0375	3.63E-08	2.52E-06
EABT35511	9.13396	0.0375	3.63E-08	2.52E-06
EABT5353	4.13703	4.732471	3.71E-08	2.57E-06
EABT36409	-4.1165	5.007309	3.81E-08	2.63E-06
EABT18924	4.25842	3.214394	4.00E-08	2.76E-06
EABT12932	5.35037	0.894343	4.03E-08	2.77E-06
EABT32735	9.11324	0.017864	4.09E-08	2.81E-06
EABT9378	4.61343	1.856317	4.15E-08	2.84E-06
EABT12161	-4.4662	2.195747	4.29E-08	2.94E-06
EABT10294	-4.211	3.375718	4.50E-08	3.07E-06
EABT28149	4.70376	1.629278	4.53E-08	3.09E-06
EABT12942	-4.1919	3.500922	4.59E-08	3.12E-06
EABT390	9.09221	-0.002043	4.62E-08	3.12E-06
EABT28432	9.09221	-0.002043	4.62E-08	3.12E-06
EABT21328	-9.088	-0.004597	4.62E-08	3.12E-06
EABT36902	-9.088	-0.004597	4.62E-08	3.12E-06
EABT16450	4.23309	3.190471	4.68E-08	3.15E-06
EABT1964	5.11076	1.057101	4.76E-08	3.20E-06
EABT37047	4.07005	5.356071	4.81E-08	3.22E-06
EABT896	-4.1313	3.930858	5.10E-08	3.42E-06
EABT16158	4.68311	1.609733	5.14E-08	3.43E-06
EABT737	-4.3903	2.312892	5.19E-08	3.45E-06
EABT33659	-4.0131	9.635268	5.19E-08	3.45E-06
EABT15768	9.07088	-0.022229	5.22E-08	3.45E-06
EABT12030	9.07088	-0.022229	5.22E-08	3.45E-06
EABT17424	9.07088	-0.022229	5.22E-08	3.45E-06
EABT10690	6.27906	0.351687	5.30E-08	3.50E-06
EABT13624	5.63258	0.622605	5.46E-08	3.60E-06
EABT5785	-4.2072	3.126486	5.56E-08	3.66E-06
EABT14681	9.04922	-0.0427	5.90E-08	3.86E-06
EABT100	9.04922	-0.0427	5.90E-08	3.86E-06
EABT4830	9.04922	-0.0427	5.90E-08	3.86E-06
EABT22260	5.61879	0.609545	5.93E-08	3.86E-06
EABT8710	-5.6106	0.606684	5.93E-08	3.86E-06
EABT13245	4.02001	5.932866	5.97E-08	3.88E-06
EABT33107	4.92389	1.185853	5.99E-08	3.89E-06
EABT29117	5.06958	1.018095	6.09E-08	3.94E-06
EABT32487	5.06958	1.018095	6.09E-08	3.94E-06
EABT33327	5.28062	0.828232	6.11E-08	3.94E-06
EABT14617	4.71141	1.450395	6.58E-08	4.24E-06

EABT19198	9.02724	-0.063467	6.69E-08	4.29E-06
EABT33918	9.02724	-0.063467	6.69E-08	4.29E-06
EABT9980	9.02724	-0.063467	6.69E-08	4.29E-06
EABT36868	4.7914	1.312136	6.83E-08	4.37E-06
EABT6569	4.09166	3.814389	6.93E-08	4.42E-06
EABT1920	-4.3427	2.268007	6.93E-08	4.42E-06
EABT6162	4.89572	1.15921	7.10E-08	4.51E-06
EABT36390	6.22851	0.303841	7.11E-08	4.51E-06
EABT23475	6.22851	0.303841	7.11E-08	4.51E-06
EABT3054	4.62656	1.556278	7.23E-08	4.58E-06
EABT26815	-3.9562	8.973424	7.41E-08	4.68E-06
EABT18093	9.00492	-0.084536	7.60E-08	4.77E-06
EABT21300	9.00492	-0.084536	7.60E-08	4.77E-06
EABT11358	-5.5685	0.566805	7.60E-08	4.77E-06
EABT19011	5.02718	0.978004	7.84E-08	4.91E-06
EABT2327	6.21126	0.287534	7.86E-08	4.91E-06
EABT5323	6.21126	0.287534	7.86E-08	4.91E-06
EABT25145	4.87662	1.14117	7.95E-08	4.96E-06
EABT20772	5.23217	0.782413	8.14E-08	5.06E-06
EABT9813	5.23217	0.782413	8.14E-08	5.06E-06
EABT10202	-4.1969	2.764603	8.15E-08	5.06E-06
EABT19620	-4.1054	3.276204	8.61E-08	5.33E-06
EABT4141	8.98224	-0.105918	8.64E-08	5.33E-06
EABT7498	-8.978	-0.108395	8.64E-08	5.33E-06
EABT21053	4.17986	2.800029	8.69E-08	5.35E-06
EABT20832	5.2198	0.770727	8.76E-08	5.38E-06
EABT18770	-4.528	1.6325	8.84E-08	5.43E-06
EABT31438	5.54779	0.542407	9.00E-08	5.51E-06
EABT28704	4.52946	1.629262	9.21E-08	5.63E-06
EABT32560	3.92021	8.290776	9.30E-08	5.68E-06
EABT28876	-4.0136	4.08588	9.35E-08	5.70E-06
EABT4449	5.20732	0.758946	9.42E-08	5.73E-06
EABT5037	-3.9597	5.101061	9.51E-08	5.77E-06
EABT19352	4.99455	0.947188	9.52E-08	5.77E-06
EABT10970	6.17612	0.254355	9.63E-08	5.83E-06
EABT24626	-4.0654	3.415576	9.77E-08	5.90E-06
EABT33432	5.53317	0.528597	9.80E-08	5.90E-06
EABT12988	8.9592	-0.127621	9.84E-08	5.90E-06
EABT3504	8.9592	-0.127621	9.84E-08	5.90E-06
EABT13155	-8.955	-0.130082	9.84E-08	5.90E-06
EABT18731	-5.1865	0.744168	1.01E-07	6.08E-06
EABT19542	-3.9867	4.195647	1.05E-07	6.25E-06
EABT644	-3.9675	4.476004	1.06E-07	6.34E-06
EABT1248	6.15823	0.237475	1.07E-07	6.35E-06
EABT7846	5.51839	0.514653	1.07E-07	6.35E-06
EABT5521	-4.3429	1.974853	1.07E-07	6.35E-06
EABT22187	-5.1738	0.732199	1.09E-07	6.48E-06
EABT27307	3.90568	6.545831	1.10E-07	6.54E-06
EABT23274	3.89812	7.308998	1.12E-07	6.57E-06
EABT6397	8.93579	-0.149656	1.12E-07	6.57E-06
EABT3575	8.93579	-0.149656	1.12E-07	6.57E-06
EABT23339	8.93579	-0.149656	1.12E-07	6.57E-06
EABT31035	8.93579	-0.149656	1.12E-07	6.57E-06
EABT17054	-8.9316	-0.1521	1.12E-07	6.57E-06

EABT19237	4.31394	2.047579	1.14E-07	6.67E-06
EABT35077	3.88511	9.369988	1.14E-07	6.68E-06
EABT23857	-3.9018	6.417852	1.16E-07	6.77E-06
EABT22697	5.50346	0.500572	1.17E-07	6.78E-06
EABT35778	5.16923	0.723014	1.18E-07	6.85E-06
EABT336	6.14011	0.220395	1.18E-07	6.87E-06
EABT17853	4.80773	1.076194	1.20E-07	6.94E-06
EABT27945	-4.686	1.217535	1.22E-07	7.05E-06
EABT37776	3.87545	8.086783	1.23E-07	7.08E-06
EABT20596	-4.4722	1.580048	1.24E-07	7.12E-06
EABT14185	4.94985	0.905049	1.24E-07	7.13E-06
EABT3418	-3.9204	4.932818	1.25E-07	7.15E-06
EABT18075	-4.29	2.029675	1.25E-07	7.18E-06
EABT33464	5.48837	0.486354	1.27E-07	7.28E-06
EABT14242	8.91199	-0.172032	1.28E-07	7.33E-06
EABT23605	-4.6769	1.208925	1.29E-07	7.35E-06
EABT19292	6.12176	0.203111	1.32E-07	7.50E-06
EABT2839	4.09467	2.825656	1.32E-07	7.53E-06
EABT24397	-5.135	0.695686	1.37E-07	7.79E-06
EABT23284	-4.093	2.722887	1.45E-07	8.15E-06
EABT830	-3.9137	4.455229	1.46E-07	8.15E-06
EABT7349	6.10317	0.185617	1.46E-07	8.15E-06
EABT13435	6.10317	0.185617	1.46E-07	8.15E-06
EABT32212	6.10317	0.185617	1.46E-07	8.15E-06
EABT35963	6.10317	0.185617	1.46E-07	8.15E-06
EABT31082	6.10317	0.185617	1.46E-07	8.15E-06
EABT3065	-6.0952	0.182996	1.46E-07	8.15E-06
EABT16079	-6.0952	0.182996	1.46E-07	8.15E-06
EABT17012	4.45188	1.556261	1.47E-07	8.15E-06
EABT34037	8.8878	-0.194761	1.47E-07	8.15E-06
EABT19986	8.8878	-0.194761	1.47E-07	8.15E-06
EABT30447	8.8878	-0.194761	1.47E-07	8.15E-06
EABT1200	-8.8836	-0.19717	1.47E-07	8.15E-06
EABT11089	4.9154	0.872616	1.52E-07	8.42E-06
EABT34860	-4.3095	1.830758	1.57E-07	8.68E-06
EABT1145	-3.9639	3.564775	1.57E-07	8.68E-06
EABT25738	3.84168	7.254185	1.58E-07	8.70E-06
EABT19809	-3.8318	7.931494	1.61E-07	8.84E-06
EABT16178	6.08434	0.167908	1.63E-07	8.95E-06
EABT12829	6.08434	0.167908	1.63E-07	8.95E-06
EABT36724	5.44214	0.442836	1.66E-07	9.11E-06
EABT13188	8.86319	-0.217853	1.68E-07	9.20E-06
EABT721	8.86319	-0.217853	1.68E-07	9.20E-06
EABT13122	-3.8759	4.734853	1.70E-07	9.26E-06
EABT32345	4.18286	2.199103	1.71E-07	9.31E-06
EABT26428	4.33661	1.729227	1.73E-07	9.39E-06
EABT25637	-5.4182	0.425301	1.82E-07	9.89E-06
EABT34438	-3.8852	4.257516	1.84E-07	9.96E-06
EABT27177	4.36564	1.62276	1.85E-07	1.00E-05
EABT16864	5.08988	0.64835	1.87E-07	1.01E-05
EABT28134	-3.8399	5.178654	1.92E-07	1.03E-05
EABT7696	-4.1125	2.368111	1.92E-07	1.03E-05
EABT16495	3.92158	3.680263	1.93E-07	1.04E-05
EABT21709	8.83815	-0.241321	1.94E-07	1.04E-05

EABT16996	8.83815	-0.241321	1.94E-07	1.04E-05
EABT11667	8.83815	-0.241321	1.94E-07	1.04E-05
EABT3248	-4.014	2.852947	1.95E-07	1.04E-05
EABT23078	3.83954	5.165617	1.97E-07	1.05E-05
EABT7504	4.60983	1.141149	2.01E-07	1.07E-05
EABT20581	4.52111	1.271232	2.05E-07	1.09E-05
EABT18508	4.07623	2.46506	2.06E-07	1.09E-05
EABT12125	8.81267	-0.265177	2.23E-07	1.18E-05
EABT29821	8.81267	-0.265177	2.23E-07	1.18E-05
EABT20271	8.81267	-0.265177	2.23E-07	1.18E-05
EABT8154	6.02634	0.11344	2.27E-07	1.20E-05
EABT37194	-6.0184	0.110873	2.27E-07	1.20E-05
EABT37097	-4.3222	1.586726	2.28E-07	1.20E-05
EABT32980	-3.8329	4.46522	2.33E-07	1.23E-05
EABT13963	-3.8939	3.527181	2.37E-07	1.24E-05
EABT23354	4.58051	1.113659	2.39E-07	1.25E-05
EABT12701	-3.8075	4.968349	2.41E-07	1.26E-05
EABT22553	-4.135	2.073044	2.43E-07	1.27E-05
EABT34008	-3.7622	7.570724	2.47E-07	1.29E-05
EABT6784	3.75812	10.38008	2.47E-07	1.29E-05
EABT2549	4.31628	1.576523	2.48E-07	1.29E-05
EABT25323	3.87259	3.70809	2.51E-07	1.30E-05
EABT32920	6.00647	0.094818	2.54E-07	1.32E-05
EABT30946	4.17907	1.921132	2.56E-07	1.32E-05
EABT16192	8.78674	-0.289434	2.57E-07	1.32E-05
EABT16483	8.78674	-0.289434	2.57E-07	1.32E-05
EABT26945	8.78674	-0.289434	2.57E-07	1.32E-05
EABT20208	8.78674	-0.289434	2.57E-07	1.32E-05
EABT1748	8.78674	-0.289434	2.57E-07	1.32E-05
EABT23716	3.81561	4.538477	2.60E-07	1.33E-05
EABT9743	4.23161	1.753165	2.63E-07	1.35E-05
EABT12805	5.36163	0.367258	2.64E-07	1.35E-05
EABT18701	3.94339	2.918575	2.72E-07	1.39E-05
EABT6035	-3.7474	7.428691	2.76E-07	1.41E-05
EABT37938	-4.0609	2.247167	2.78E-07	1.41E-05
EABT13868	5.98633	0.075951	2.84E-07	1.44E-05
EABT2971	5.98633	0.075951	2.84E-07	1.44E-05
EABT12878	5.98633	0.075951	2.84E-07	1.44E-05
EABT23109	-3.7354	7.663563	2.90E-07	1.47E-05
EABT36178	3.77043	5.246921	2.92E-07	1.48E-05
EABT15935	4.09165	2.113794	2.95E-07	1.49E-05
EABT15503	-3.7982	4.332781	2.96E-07	1.49E-05
EABT18343	4.33104	1.443016	3.01E-07	1.51E-05
EABT17029	-3.8355	3.701525	3.01E-07	1.51E-05
EABT152	5.00592	0.56961	3.05E-07	1.53E-05
EABT18026	-4.1166	1.964686	3.07E-07	1.54E-05
EABT25148	3.72093	8.18961	3.14E-07	1.57E-05
EABT26736	-3.7412	5.795069	3.15E-07	1.57E-05
EABT36736	5.9659	0.056835	3.19E-07	1.59E-05
EABT10671	5.9659	0.056835	3.19E-07	1.59E-05
EABT36348	5.32813	0.335882	3.20E-07	1.59E-05
EABT33370	-3.723	7.11742	3.25E-07	1.61E-05
EABT26462	4.63671	0.915676	3.27E-07	1.62E-05
EABT1750	3.71126	9.362891	3.30E-07	1.63E-05

EABT16077	-3.8022	3.888399	3.35E-07	1.66E-05
EABT19026	8.73342	-0.339206	3.45E-07	1.70E-05
EABT13259	5.31108	0.319935	3.53E-07	1.74E-05
EABT20679	5.31108	0.319935	3.53E-07	1.74E-05
EABT35038	5.31108	0.319935	3.53E-07	1.74E-05
EABT22913	-4.0357	2.147047	3.54E-07	1.74E-05
EABT10870	5.94518	0.037463	3.58E-07	1.75E-05
EABT9132	5.94518	0.037463	3.58E-07	1.75E-05
EABT37302	5.94518	0.037463	3.58E-07	1.75E-05
EABT20908	-5.9372	0.034953	3.58E-07	1.75E-05
EABT2727	3.80276	3.779784	3.60E-07	1.76E-05
EABT4239	4.50969	1.047404	3.62E-07	1.76E-05
EABT18782	3.77308	4.081173	3.78E-07	1.84E-05
EABT30503	5.29383	0.303809	3.89E-07	1.89E-05
EABT17570	4.41088	1.168104	3.92E-07	1.90E-05
EABT8205	-3.6889	7.392678	3.93E-07	1.90E-05
EABT31885	8.706	-0.364751	4.00E-07	1.93E-05
EABT872	8.706	-0.364751	4.00E-07	1.93E-05
EABT3151	3.68959	6.478023	4.07E-07	1.96E-05
EABT15578	4.05433	1.993276	4.12E-07	1.99E-05
EABT19090	-4.0663	1.917906	4.14E-07	1.99E-05
EABT2149	4.4013	1.159168	4.14E-07	1.99E-05
EABT16699	3.72314	4.723338	4.24E-07	2.03E-05
EABT38048	4.9471	0.514623	4.27E-07	2.05E-05
EABT36124	5.27637	0.287501	4.30E-07	2.05E-05
EABT1296	3.8724	2.808581	4.30E-07	2.05E-05
EABT8457	4.11728	1.759077	4.32E-07	2.06E-05
EABT34083	-3.804	3.328862	4.33E-07	2.06E-05
EABT16512	-4.0549	1.907301	4.42E-07	2.10E-05
EABT14899	3.68695	5.786362	4.47E-07	2.12E-05
EABT13647	4.72958	0.698526	4.48E-07	2.12E-05
EABT34543	5.90282	-0.002081	4.54E-07	2.15E-05
EABT33543	5.90282	-0.002081	4.54E-07	2.15E-05
EABT16479	-4.0712	1.825163	4.62E-07	2.18E-05
EABT17273	-4.3736	1.138147	4.64E-07	2.19E-05
EABT33421	3.6825	5.667342	4.64E-07	2.19E-05
EABT13782	8.67806	-0.390757	4.66E-07	2.19E-05
EABT34765	5.25869	0.271007	4.75E-07	2.23E-05
EABT28800	5.25869	0.271007	4.75E-07	2.23E-05
EABT35775	-3.9296	2.332904	4.81E-07	2.25E-05
EABT26687	3.85838	2.750275	4.84E-07	2.26E-05
EABT11465	-3.8235	2.974807	4.87E-07	2.27E-05
EABT37873	-3.6621	6.160643	4.99E-07	2.33E-05
EABT28718	-3.6763	5.209229	4.99E-07	2.33E-05
EABT11757	-4.9085	0.483609	5.08E-07	2.37E-05
EABT25248	3.72255	4.03429	5.11E-07	2.37E-05
EABT24175	-3.9309	2.268061	5.12E-07	2.37E-05
EABT25232	5.88117	-0.022267	5.12E-07	2.37E-05
EABT6592	-3.6665	5.314833	5.20E-07	2.41E-05
EABT23003	-3.6643	5.36026	5.23E-07	2.42E-05
EABT604	4.44609	0.988086	5.24E-07	2.42E-05
EABT26324	5.2408	0.254322	5.25E-07	2.42E-05
EABT9507	4.55499	0.839413	5.25E-07	2.42E-05
EABT29953	-3.6589	5.482748	5.31E-07	2.44E-05

EABT30050	8.64956	-0.417239	5.43E-07	2.48E-05
EABT17655	8.64956	-0.417239	5.43E-07	2.48E-05
EABT3896	8.64956	-0.417239	5.43E-07	2.48E-05
EABT21972	8.64956	-0.417239	5.43E-07	2.48E-05
EABT15762	8.64956	-0.417239	5.43E-07	2.48E-05
EABT18533	8.64956	-0.417239	5.43E-07	2.48E-05
EABT29167	8.64956	-0.417239	5.43E-07	2.48E-05
EABT23532	4.90136	0.471963	5.55E-07	2.52E-05
EABT799	-4.8931	0.469259	5.55E-07	2.52E-05
EABT4195	-3.6982	4.066998	5.71E-07	2.59E-05
EABT10055	-3.668	4.665777	5.76E-07	2.61E-05
EABT19674	5.85918	-0.042739	5.79E-07	2.62E-05
EABT36048	5.85918	-0.042739	5.79E-07	2.62E-05
EABT25901	-4.2079	1.333033	5.89E-07	2.66E-05
EABT20388	-3.7119	3.721361	5.96E-07	2.69E-05
EABT37811	4.53077	0.816863	6.04E-07	2.72E-05
EABT34145	-4.8775	0.454765	6.06E-07	2.72E-05
EABT32318	-4.8775	0.454765	6.06E-07	2.72E-05
EABT21256	-4.6674	0.645545	6.10E-07	2.74E-05
EABT18520	8.62049	-0.444217	6.35E-07	2.83E-05
EABT29586	8.62049	-0.444217	6.35E-07	2.83E-05
EABT17482	8.62049	-0.444217	6.35E-07	2.83E-05
EABT8338	8.62049	-0.444217	6.35E-07	2.83E-05
EABT7416	8.62049	-0.444217	6.35E-07	2.83E-05
EABT37832	3.80023	2.785529	6.38E-07	2.84E-05
EABT20161	-3.6164	6.389368	6.40E-07	2.85E-05
EABT14646	-3.6451	4.807994	6.41E-07	2.85E-05
EABT34154	-3.6156	6.388654	6.43E-07	2.85E-05
EABT30592	5.20433	0.220361	6.45E-07	2.86E-05
EABT17775	5.83686	-0.063505	6.55E-07	2.90E-05
EABT36359	-4.6536	0.632726	6.60E-07	2.91E-05
EABT14277	4.87004	0.442806	6.63E-07	2.92E-05
EABT12997	3.63055	5.249528	6.63E-07	2.92E-05
EABT6786	-3.6109	6.352215	6.64E-07	2.92E-05
EABT10460	3.62717	5.287947	6.72E-07	2.96E-05
EABT22752	-3.8809	2.221736	6.88E-07	3.02E-05
EABT32505	-3.7071	3.459855	6.88E-07	3.02E-05
EABT6341	-3.7167	3.334826	6.95E-07	3.04E-05
EABT1656	4.648	0.62255	7.14E-07	3.12E-05
EABT7330	-4.6397	0.619792	7.14E-07	3.12E-05
EABT10741	5.18574	0.203077	7.16E-07	3.12E-05
EABT27422	5.18574	0.203077	7.16E-07	3.12E-05
EABT4039	-4.1734	1.300983	7.20E-07	3.13E-05
EABT6704	-3.6004	5.807627	7.23E-07	3.14E-05
EABT36776	-4.1246	1.402663	7.27E-07	3.16E-05
EABT7078	4.09164	1.500715	7.33E-07	3.18E-05
EABT244	5.81418	-0.084575	7.42E-07	3.19E-05
EABT25020	5.81418	-0.084575	7.42E-07	3.19E-05
EABT5591	5.81418	-0.084575	7.42E-07	3.19E-05
EABT1010	5.81418	-0.084575	7.42E-07	3.19E-05
EABT2074	8.59081	-0.471708	7.45E-07	3.19E-05
EABT5720	8.59081	-0.471708	7.45E-07	3.19E-05
EABT11913	8.59081	-0.471708	7.45E-07	3.19E-05
EABT32787	8.59081	-0.471708	7.45E-07	3.19E-05

EABT6643	8.59081	-0.471708	7.45E-07	3.19E-05
EABT26943	8.59081	-0.471708	7.45E-07	3.19E-05
EABT29532	8.59081	-0.471708	7.45E-07	3.19E-05
EABT26634	3.65127	4.116199	7.47E-07	3.19E-05
EABT26559	4.49366	0.782362	7.47E-07	3.19E-05
EABT13540	4.23092	1.185792	7.49E-07	3.19E-05
EABT20882	-3.7395	2.972279	7.61E-07	3.24E-05
EABT22227	-3.8164	2.465356	7.62E-07	3.24E-05
EABT18413	-4.1164	1.395092	7.63E-07	3.24E-05
EABT676	4.83802	0.413047	7.94E-07	3.35E-05
EABT12761	-4.8298	0.410387	7.94E-07	3.35E-05
EABT1129	3.9071	2.02311	7.94E-07	3.35E-05
EABT16049	5.16692	0.185583	7.95E-07	3.35E-05
EABT2654	-5.1588	0.183031	7.95E-07	3.35E-05
EABT27917	-3.6907	3.28244	8.23E-07	3.47E-05
EABT25745	-3.5581	8.660835	8.29E-07	3.49E-05
EABT23007	-3.7641	2.667449	8.36E-07	3.51E-05
EABT9961	-4.1	1.379831	8.39E-07	3.52E-05
EABT135	5.79115	-0.105957	8.42E-07	3.53E-05
EABT8002	-3.6539	3.624326	8.56E-07	3.59E-05
EABT9822	3.97273	1.729182	8.63E-07	3.61E-05
EABT6622	3.73744	2.811413	8.69E-07	3.61E-05
EABT1009	4.82174	0.397935	8.70E-07	3.61E-05
EABT30524	4.82174	0.397935	8.70E-07	3.61E-05
EABT30818	3.6048	4.536714	8.76E-07	3.61E-05
EABT10373	8.56052	-0.499734	8.76E-07	3.61E-05
EABT7249	8.56052	-0.499734	8.76E-07	3.61E-05
EABT15510	8.56052	-0.499734	8.76E-07	3.61E-05
EABT13336	8.56052	-0.499734	8.76E-07	3.61E-05
EABT27185	8.56052	-0.499734	8.76E-07	3.61E-05
EABT11515	8.56052	-0.499734	8.76E-07	3.61E-05
EABT34289	-8.5563	-0.501907	8.76E-07	3.61E-05
EABT3775	-8.5563	-0.501907	8.76E-07	3.61E-05
EABT21507	4.0608	1.472136	8.77E-07	3.61E-05
EABT19872	-4.0916	1.372139	8.80E-07	3.62E-05
EABT10165	-3.9161	1.869571	8.84E-07	3.63E-05
EABT30424	-3.6925	3.133409	8.85E-07	3.63E-05
EABT27846	3.65171	3.617996	8.93E-07	3.66E-05
EABT19764	4.0199	1.556211	9.11E-07	3.73E-05
EABT2029	3.81766	2.287807	9.16E-07	3.75E-05
EABT34766	3.54989	6.496735	9.32E-07	3.81E-05
EABT31291	-4.3368	0.891418	9.36E-07	3.82E-05
EABT24842	-3.6271	3.76609	9.39E-07	3.83E-05
EABT32239	-3.7197	2.799508	9.52E-07	3.87E-05
EABT26418	4.80528	0.382662	9.55E-07	3.87E-05
EABT13271	4.80528	0.382662	9.55E-07	3.87E-05
EABT26086	5.76773	-0.127661	9.57E-07	3.87E-05
EABT36486	5.76773	-0.127661	9.57E-07	3.87E-05
EABT24489	5.76773	-0.127661	9.57E-07	3.87E-05
EABT23602	5.76773	-0.127661	9.57E-07	3.87E-05
EABT33536	-3.5318	8.932736	9.68E-07	3.91E-05
EABT18407	5.12851	0.149945	9.85E-07	3.97E-05
EABT11395	3.63979	3.539262	9.85E-07	3.97E-05
EABT35315	4.59092	0.569581	9.87E-07	3.97E-05

EABT20688	-3.8758	1.917933	1.00E-06	4.03E-05
EABT332	-3.9619	1.619629	1.02E-06	4.10E-05
EABT10775	3.55889	5.10404	1.03E-06	4.11E-05
EABT33808	8.52958	-0.528314	1.03E-06	4.11E-05
EABT29494	8.52958	-0.528314	1.03E-06	4.11E-05
EABT31993	8.52958	-0.528314	1.03E-06	4.11E-05
EABT17320	8.52958	-0.528314	1.03E-06	4.11E-05
EABT8383	-8.5254	-0.530464	1.03E-06	4.11E-05
EABT2890	-3.5877	4.137916	1.04E-06	4.15E-05
EABT19913	-4.7804	0.364601	1.05E-06	4.17E-05
EABT21970	-3.8862	1.841994	1.05E-06	4.18E-05
EABT32596	-3.5336	6.251526	1.05E-06	4.18E-05
EABT30158	-3.5729	4.343681	1.07E-06	4.24E-05
EABT11007	4.5763	0.556029	1.07E-06	4.24E-05
EABT35512	4.5763	0.556029	1.07E-06	4.24E-05
EABT16722	4.4296	0.722962	1.08E-06	4.25E-05
EABT33539	-4.4213	0.720183	1.08E-06	4.25E-05
EABT35348	3.99017	1.528721	1.08E-06	4.26E-05
EABT23436	-3.5277	6.30124	1.09E-06	4.28E-05
EABT31502	5.74394	-0.149696	1.09E-06	4.28E-05
EABT1442	5.74394	-0.149696	1.09E-06	4.28E-05
EABT33769	-3.7011	2.740981	1.09E-06	4.30E-05
EABT10365	-3.5525	4.622685	1.13E-06	4.42E-05
EABT3424	-3.5399	4.958802	1.14E-06	4.48E-05
EABT3660	4.10091	1.229114	1.15E-06	4.49E-05
EABT31264	4.56152	0.542349	1.17E-06	4.55E-05
EABT20471	3.5497	4.626145	1.18E-06	4.60E-05
EABT7366	-3.5066	6.73658	1.19E-06	4.65E-05
EABT5035	8.49795	-0.557472	1.22E-06	4.70E-05
EABT25017	8.49795	-0.557472	1.22E-06	4.70E-05
EABT31788	8.49795	-0.557472	1.22E-06	4.70E-05
EABT13119	8.49795	-0.557472	1.22E-06	4.70E-05
EABT37167	8.49795	-0.557472	1.22E-06	4.70E-05
EABT22753	8.49795	-0.557472	1.22E-06	4.70E-05
EABT26948	8.49795	-0.557472	1.22E-06	4.70E-05
EABT22575	8.49795	-0.557472	1.22E-06	4.70E-05
EABT13098	8.49795	-0.557472	1.22E-06	4.70E-05
EABT26805	-8.4938	-0.559599	1.22E-06	4.70E-05
EABT21573	5.08904	0.113405	1.23E-06	4.71E-05
EABT13116	5.08904	0.113405	1.23E-06	4.71E-05
EABT25805	4.29784	0.850533	1.23E-06	4.71E-05
EABT33546	-3.7266	2.436155	1.23E-06	4.72E-05
EABT18825	5.71974	-0.172072	1.24E-06	4.74E-05
EABT18150	5.71974	-0.172072	1.24E-06	4.74E-05
EABT35067	5.71974	-0.172072	1.24E-06	4.74E-05
EABT37709	5.71974	-0.172072	1.24E-06	4.74E-05
EABT29618	5.71974	-0.172072	1.24E-06	4.74E-05
EABT23916	-3.5087	5.737151	1.24E-06	4.74E-05
EABT35594	3.49726	6.929005	1.25E-06	4.75E-05
EABT31267	4.40316	0.698499	1.25E-06	4.75E-05
EABT24555	3.52255	5.11447	1.27E-06	4.81E-05
EABT24903	4.75473	0.33585	1.27E-06	4.81E-05
EABT13125	4.28578	0.839388	1.31E-06	4.98E-05
EABT24249	3.49614	5.896268	1.32E-06	5.00E-05

EABT11990	3.48762	6.850004	1.33E-06	5.01E-05
EABT15357	-3.4859	6.983929	1.33E-06	5.03E-05
EABT15005	-3.6342	2.946629	1.35E-06	5.10E-05
EABT8772	-3.8233	1.869585	1.36E-06	5.12E-05
EABT11195	5.0689	0.094782	1.37E-06	5.15E-05
EABT20216	4.12263	1.085592	1.39E-06	5.25E-05
EABT1037	-4.2653	0.825353	1.41E-06	5.28E-05
EABT1456	5.69513	-0.194801	1.42E-06	5.33E-05
EABT12898	-5.6872	-0.19713	1.42E-06	5.33E-05
EABT26496	-3.4728	6.94265	1.44E-06	5.36E-05
EABT19073	8.46562	-0.587231	1.45E-06	5.36E-05
EABT9916	8.46562	-0.587231	1.45E-06	5.36E-05
EABT37427	8.46562	-0.587231	1.45E-06	5.36E-05
EABT14060	8.46562	-0.587231	1.45E-06	5.36E-05
EABT34779	8.46562	-0.587231	1.45E-06	5.36E-05
EABT25998	8.46562	-0.587231	1.45E-06	5.36E-05
EABT30051	-8.4614	-0.589335	1.45E-06	5.36E-05
EABT8297	-8.4614	-0.589335	1.45E-06	5.36E-05
EABT9193	-8.4614	-0.589335	1.45E-06	5.36E-05
EABT36144	-8.4614	-0.589335	1.45E-06	5.36E-05
EABT26157	3.93663	1.479317	1.47E-06	5.45E-05
EABT19795	3.46077	8.105573	1.48E-06	5.48E-05
EABT6273	-4.0456	1.182843	1.50E-06	5.55E-05
EABT32556	4.26136	0.816838	1.51E-06	5.56E-05
EABT33831	4.26136	0.816838	1.51E-06	5.56E-05
EABT37113	5.04847	0.075915	1.53E-06	5.63E-05
EABT13005	-5.0403	0.073449	1.53E-06	5.63E-05
EABT32312	-4.7118	0.3012	1.54E-06	5.65E-05
EABT30765	3.61416	2.923804	1.54E-06	5.67E-05
EABT3884	3.53912	3.794234	1.56E-06	5.71E-05
EABT13080	3.80778	1.850679	1.56E-06	5.71E-05
EABT29038	3.80778	1.850679	1.56E-06	5.71E-05
EABT29172	3.7754	1.978073	1.56E-06	5.71E-05
EABT18661	-3.5032	4.401525	1.57E-06	5.73E-05
EABT2391	3.72133	2.199035	1.61E-06	5.85E-05
EABT24898	3.95576	1.375141	1.61E-06	5.85E-05
EABT30510	3.7435	2.090432	1.61E-06	5.86E-05
EABT28338	3.76987	1.972983	1.61E-06	5.86E-05
EABT3194	3.80173	1.845118	1.62E-06	5.86E-05
EABT9971	-3.856	1.626127	1.62E-06	5.86E-05
EABT234	-3.446	7.758423	1.62E-06	5.88E-05
EABT19379	5.67009	-0.217894	1.63E-06	5.88E-05
EABT24484	5.67009	-0.217894	1.63E-06	5.88E-05
EABT35190	3.45081	7.20977	1.63E-06	5.88E-05
EABT12961	-3.529	3.69543	1.66E-06	5.99E-05
EABT3778	4.34877	0.648295	1.70E-06	6.11E-05
EABT31363	5.02775	0.056799	1.72E-06	6.11E-05
EABT19927	5.02775	0.056799	1.72E-06	6.11E-05
EABT8081	-5.0196	0.054348	1.72E-06	6.11E-05
EABT2740	8.43255	-0.617617	1.72E-06	6.11E-05
EABT29110	8.43255	-0.617617	1.72E-06	6.11E-05
EABT16044	8.43255	-0.617617	1.72E-06	6.11E-05
EABT29722	8.43255	-0.617617	1.72E-06	6.11E-05
EABT32844	8.43255	-0.617617	1.72E-06	6.11E-05

EABT19219	8.43255	-0.617617	1.72E-06	6.11E-05
EABT34718	8.43255	-0.617617	1.72E-06	6.11E-05
EABT8249	8.43255	-0.617617	1.72E-06	6.11E-05
EABT33810	8.43255	-0.617617	1.72E-06	6.11E-05
EABT36069	8.43255	-0.617617	1.72E-06	6.11E-05
EABT17688	4.48527	0.471933	1.79E-06	6.33E-05
EABT14951	4.48527	0.471933	1.79E-06	6.33E-05
EABT25175	4.48527	0.471933	1.79E-06	6.33E-05
EABT26064	-3.7391	1.949313	1.84E-06	6.49E-05
EABT26340	4.33485	0.635466	1.84E-06	6.49E-05
EABT18217	4.33485	0.635466	1.84E-06	6.49E-05
EABT35455	4.33485	0.635466	1.84E-06	6.49E-05
EABT8716	5.64462	-0.241363	1.87E-06	6.57E-05
EABT17330	-5.6367	-0.243654	1.87E-06	6.57E-05
EABT27325	4.1385	0.915628	1.87E-06	6.60E-05
EABT29901	4.46952	0.457428	1.95E-06	6.86E-05
EABT32915	8.3987	-0.648656	2.05E-06	7.12E-05
EABT19816	8.3987	-0.648656	2.05E-06	7.12E-05
EABT25703	8.3987	-0.648656	2.05E-06	7.12E-05
EABT126	8.3987	-0.648656	2.05E-06	7.12E-05
EABT27139	8.3987	-0.648656	2.05E-06	7.12E-05
EABT34835	8.3987	-0.648656	2.05E-06	7.12E-05
EABT3107	8.3987	-0.648656	2.05E-06	7.12E-05
EABT36155	8.3987	-0.648656	2.05E-06	7.12E-05
EABT15580	8.3987	-0.648656	2.05E-06	7.12E-05
EABT25675	8.3987	-0.648656	2.05E-06	7.12E-05
EABT11310	8.3987	-0.648656	2.05E-06	7.12E-05
EABT9690	8.3987	-0.648656	2.05E-06	7.12E-05
EABT10759	-3.5522	2.972314	2.05E-06	7.13E-05
EABT34903	-3.4243	5.84288	2.06E-06	7.15E-05
EABT18804	4.66632	0.254288	2.07E-06	7.16E-05
EABT9677	4.66632	0.254288	2.07E-06	7.16E-05
EABT3407	-3.7503	1.802493	2.07E-06	7.16E-05
EABT79	3.77514	1.735178	2.10E-06	7.24E-05
EABT30225	-3.3997	9.790236	2.10E-06	7.24E-05
EABT25555	-3.4323	5.014246	2.10E-06	7.24E-05
EABT13286	3.69673	2.047492	2.11E-06	7.26E-05
EABT16983	4.4536	0.442775	2.13E-06	7.33E-05
EABT17961	4.11526	0.894246	2.14E-06	7.33E-05
EABT6806	-4.1069	0.891442	2.14E-06	7.33E-05
EABT12365	3.51407	3.328264	2.14E-06	7.33E-05
EABT17141	5.61868	-0.265219	2.14E-06	7.33E-05
EABT10716	5.61868	-0.265219	2.14E-06	7.33E-05
EABT13568	5.61868	-0.265219	2.14E-06	7.33E-05
EABT36867	4.19842	0.758868	2.15E-06	7.34E-05
EABT12337	4.98539	0.017789	2.16E-06	7.37E-05
EABT28658	4.98539	0.017789	2.16E-06	7.37E-05
EABT4186	4.98539	0.017789	2.16E-06	7.37E-05
EABT11276	-3.6831	2.039507	2.17E-06	7.40E-05
EABT18088	3.51677	3.254678	2.18E-06	7.41E-05
EABT24137	3.8378	1.50068	2.19E-06	7.43E-05
EABT11996	3.78663	1.654824	2.21E-06	7.50E-05
EABT12820	-4.0301	1.005243	2.25E-06	7.62E-05
EABT11403	-3.8875	1.317136	2.26E-06	7.65E-05

EABT8778	3.76197	1.723101	2.26E-06	7.65E-05
EABT10453	4.64798	0.237408	2.29E-06	7.72E-05
EABT1020	4.64798	0.237408	2.29E-06	7.72E-05
EABT7571	-3.3944	6.535403	2.32E-06	7.82E-05
EABT33183	-3.4595	3.830698	2.33E-06	7.84E-05
EABT15518	4.4375	0.427973	2.33E-06	7.84E-05
EABT2444	4.96374	-0.002118	2.43E-06	8.18E-05
EABT36645	8.36404	-0.680378	2.45E-06	8.19E-05
EABT24608	8.36404	-0.680378	2.45E-06	8.19E-05
EABT15045	8.36404	-0.680378	2.45E-06	8.19E-05
EABT14518	8.36404	-0.680378	2.45E-06	8.19E-05
EABT8251	8.36404	-0.680378	2.45E-06	8.19E-05
EABT37073	8.36404	-0.680378	2.45E-06	8.19E-05
EABT34181	-8.3598	-0.682407	2.45E-06	8.19E-05
EABT7950	5.59227	-0.289476	2.47E-06	8.20E-05
EABT32089	5.59227	-0.289476	2.47E-06	8.20E-05
EABT21917	5.59227	-0.289476	2.47E-06	8.20E-05
EABT6886	5.59227	-0.289476	2.47E-06	8.20E-05
EABT9405	-5.5843	-0.291729	2.47E-06	8.20E-05
EABT918	-5.5843	-0.291729	2.47E-06	8.20E-05
EABT27571	3.72713	1.776654	2.48E-06	8.22E-05
EABT31672	3.96537	1.104314	2.48E-06	8.22E-05
EABT29619	4.17245	0.735013	2.48E-06	8.22E-05
EABT12856	4.17245	0.735013	2.48E-06	8.22E-05
EABT4758	3.57606	2.490135	2.51E-06	8.31E-05
EABT26349	4.27778	0.582978	2.52E-06	8.33E-05
EABT14052	4.27778	0.582978	2.52E-06	8.33E-05
EABT19695	4.62939	0.220327	2.53E-06	8.34E-05
EABT7256	4.62939	0.220327	2.53E-06	8.34E-05
EABT36779	-3.7336	1.70175	2.53E-06	8.34E-05
EABT10933	3.91344	1.203244	2.56E-06	8.41E-05
EABT23405	3.36347	7.691989	2.63E-06	8.64E-05
EABT32377	4.15929	0.722935	2.67E-06	8.77E-05
EABT34732	4.15929	0.722935	2.67E-06	8.77E-05
EABT19016	-3.8232	1.379868	2.68E-06	8.78E-05
EABT66	-3.4311	3.870837	2.69E-06	8.79E-05
EABT6150	4.94176	-0.022304	2.74E-06	8.95E-05
EABT16488	3.4111	4.277592	2.77E-06	9.04E-05
EABT6870	-3.9366	1.082715	2.79E-06	9.09E-05
EABT29070	4.61056	0.203042	2.81E-06	9.13E-05
EABT23261	4.61056	0.203042	2.81E-06	9.13E-05
EABT1045	-4.6023	0.200546	2.81E-06	9.13E-05
EABT2267	5.56536	-0.314148	2.85E-06	9.24E-05
EABT28703	5.56536	-0.314148	2.85E-06	9.24E-05
EABT2667	-3.9861	0.9649	2.88E-06	9.34E-05
EABT8259	3.47028	3.185951	2.89E-06	9.35E-05
EABT27637	-3.3459	8.180795	2.90E-06	9.38E-05
EABT2351	-3.3932	4.380613	2.93E-06	9.45E-05
EABT26969	8.32853	-0.712812	2.94E-06	9.45E-05
EABT37335	8.32853	-0.712812	2.94E-06	9.45E-05
EABT32784	8.32853	-0.712812	2.94E-06	9.45E-05
EABT4357	8.32853	-0.712812	2.94E-06	9.45E-05
EABT7396	8.32853	-0.712812	2.94E-06	9.45E-05
EABT21778	8.32853	-0.712812	2.94E-06	9.45E-05

EABT21923	8.32853	-0.712812	2.94E-06	9.45E-05
EABT11735	-3.7464	1.525702	3.02E-06	9.70E-05
EABT35846	3.84246	1.271153	3.06E-06	9.81E-05
EABT9311	4.91943	-0.042777	3.09E-06	9.87E-05
EABT11856	4.91943	-0.042777	3.09E-06	9.87E-05
EABT34000	4.91943	-0.042777	3.09E-06	9.87E-05
EABT37397	-4.9113	-0.045148	3.09E-06	9.87E-05
EABT35386	4.59148	0.185548	3.11E-06	9.94E-05
EABT9776	4.59148	0.185548	3.11E-06	9.94E-05
EABT23812	-3.346	6.08655	3.16E-06	0.0001
EABT22281	3.35115	5.656995	3.18E-06	0.0001
EABT6873	3.83337	1.262835	3.22E-06	0.0001
EABT8261	4.23344	0.54232	3.23E-06	0.0001
EABT30751	-3.5789	2.133533	3.28E-06	0.0001
EABT29635	5.53795	-0.33925	3.29E-06	0.0001
EABT24992	5.53795	-0.33925	3.29E-06	0.0001
EABT19935	3.71559	1.589804	3.31E-06	0.0001
EABT23380	3.9137	1.056991	3.32E-06	0.0001
EABT8858	-3.9053	1.054156	3.32E-06	0.0001
EABT25251	4.11907	0.686084	3.34E-06	0.00011
EABT20393	-3.3282	6.467546	3.41E-06	0.00011
EABT33071	-3.3952	3.737981	3.41E-06	0.00011
EABT15812	-3.4376	3.160508	3.41E-06	0.00011
EABT16480	4.03081	0.816812	3.43E-06	0.00011
EABT24039	3.65162	1.788278	3.47E-06	0.00011
EABT6949	4.89676	-0.063544	3.49E-06	0.00011
EABT36196	8.29212	-0.745993	3.54E-06	0.00011
EABT30223	8.29212	-0.745993	3.54E-06	0.00011
EABT23744	8.29212	-0.745993	3.54E-06	0.00011
EABT21019	8.29212	-0.745993	3.54E-06	0.00011
EABT32969	8.29212	-0.745993	3.54E-06	0.00011
EABT33686	8.29212	-0.745993	3.54E-06	0.00011
EABT17783	8.29212	-0.745993	3.54E-06	0.00011
EABT9209	-8.2879	-0.747969	3.54E-06	0.00011
EABT19790	-3.3225	6.200086	3.58E-06	0.00011
EABT25908	3.72414	1.500663	3.59E-06	0.00011
EABT29112	3.4515	2.939512	3.60E-06	0.00011
EABT29972	4.10541	0.673588	3.61E-06	0.00011
EABT19551	-4.01	0.802666	3.67E-06	0.00011
EABT20988	4.35421	0.351591	3.68E-06	0.00011
EABT5902	-4.3459	0.349042	3.68E-06	0.00011
EABT11721	-3.3237	5.767566	3.68E-06	0.00011
EABT34988	3.62409	1.839507	3.72E-06	0.00011
EABT19672	3.30135	7.212977	3.79E-06	0.00012
EABT8411	5.51	-0.364795	3.81E-06	0.00012
EABT21687	5.51	-0.364795	3.81E-06	0.00012
EABT4138	5.51	-0.364795	3.81E-06	0.00012
EABT1745	-5.5021	-0.366986	3.81E-06	0.00012
EABT28855	3.55703	2.109078	3.89E-06	0.00012
EABT18677	3.29398	8.608236	3.90E-06	0.00012
EABT25045	4.87372	-0.084614	3.95E-06	0.00012
EABT28572	4.33696	0.335818	4.05E-06	0.00012
EABT23660	-4.3287	0.333281	4.05E-06	0.00012
EABT37395	3.87099	1.017982	4.21E-06	0.00013

EABT33756	3.99305	0.782311	4.22E-06	0.00013
EABT37480	-3.6629	1.546371	4.25E-06	0.00013
EABT24948	8.25476	-0.779954	4.28E-06	0.00013
EABT20252	8.25476	-0.779954	4.28E-06	0.00013
EABT7547	8.25476	-0.779954	4.28E-06	0.00013
EABT36120	8.25476	-0.779954	4.28E-06	0.00013
EABT15006	8.25476	-0.779954	4.28E-06	0.00013
EABT209	8.25476	-0.779954	4.28E-06	0.00013
EABT4191	8.25476	-0.779954	4.28E-06	0.00013
EABT5132	8.25476	-0.779954	4.28E-06	0.00013
EABT19666	8.25476	-0.779954	4.28E-06	0.00013
EABT27297	8.25476	-0.779954	4.28E-06	0.00013
EABT10027	-8.2506	-0.781903	4.28E-06	0.00013
EABT21944	4.53269	0.131755	4.28E-06	0.00013
EABT27620	4.53269	0.131755	4.28E-06	0.00013
EABT12267	-4.5245	0.129316	4.28E-06	0.00013
EABT35725	-5.4736	-0.392971	4.42E-06	0.00013
EABT26388	-3.5457	1.980039	4.45E-06	0.00013
EABT36267	4.06364	0.635438	4.54E-06	0.00014
EABT25858	-4.0553	0.632781	4.54E-06	0.00014
EABT26875	3.63772	1.609603	4.57E-06	0.00014
EABT31293	-3.3647	3.381624	4.58E-06	0.00014
EABT11478	3.55824	1.921038	4.64E-06	0.00014
EABT14438	3.41857	2.77964	4.66E-06	0.00014
EABT3630	-3.2902	4.990608	4.71E-06	0.00014
EABT14236	4.51255	0.113369	4.77E-06	0.00014
EABT196	4.51255	0.113369	4.77E-06	0.00014
EABT36725	-3.3774	3.105905	4.82E-06	0.00014
EABT35952	4.30182	0.303744	4.90E-06	0.00015
EABT37160	4.82651	-0.1277	5.07E-06	0.00015
EABT2513	-3.5921	1.658154	5.08E-06	0.00015
EABT8651	5.45243	-0.417284	5.15E-06	0.00015
EABT32550	5.45243	-0.417284	5.15E-06	0.00015
EABT18147	5.45243	-0.417284	5.15E-06	0.00015
EABT26251	5.45243	-0.417284	5.15E-06	0.00015
EABT3456	8.21642	-0.814733	5.19E-06	0.00015
EABT966	8.21642	-0.814733	5.19E-06	0.00015
EABT6183	8.21642	-0.814733	5.19E-06	0.00015
EABT4790	8.21642	-0.814733	5.19E-06	0.00015
EABT14801	8.21642	-0.814733	5.19E-06	0.00015
EABT27581	8.21642	-0.814733	5.19E-06	0.00015
EABT15163	8.21642	-0.814733	5.19E-06	0.00015
EABT6168	8.21642	-0.814733	5.19E-06	0.00015
EABT1477	8.21642	-0.814733	5.19E-06	0.00015
EABT32691	8.21642	-0.814733	5.19E-06	0.00015
EABT35141	-8.2122	-0.816655	5.19E-06	0.00015
EABT20978	-3.2432	7.925555	5.25E-06	0.00015
EABT35218	-3.6248	1.511785	5.26E-06	0.00015
EABT35916	-3.293	4.221699	5.29E-06	0.00016
EABT217	4.49212	0.094746	5.33E-06	0.00016
EABT2490	4.49212	0.094746	5.33E-06	0.00016
EABT16420	3.65263	1.435544	5.37E-06	0.00016
EABT28092	4.14046	0.457398	5.37E-06	0.00016
EABT1133	-3.3665	2.957005	5.49E-06	0.00016

EABT10743	-4.0123	0.593626	5.75E-06	0.00017
EABT8885	4.80231	-0.149736	5.77E-06	0.00017
EABT14674	-3.2792	4.137983	5.82E-06	0.00017
EABT7850	4.12436	0.442745	5.87E-06	0.00017
EABT60	4.12436	0.442745	5.87E-06	0.00017
EABT26998	4.4714	0.075879	5.95E-06	0.00017
EABT1600	4.4714	0.075879	5.95E-06	0.00017
EABT433	-4.4632	0.073485	5.95E-06	0.00017
EABT35388	5.42276	-0.444262	6.00E-06	0.00017
EABT20038	5.42276	-0.444262	6.00E-06	0.00017
EABT11021	5.42276	-0.444262	6.00E-06	0.00017
EABT15634	5.42276	-0.444262	6.00E-06	0.00017
EABT9887	5.42276	-0.444262	6.00E-06	0.00017
EABT1357	3.92783	0.722909	6.05E-06	0.00017
EABT4742	3.44367	2.224905	6.12E-06	0.00018
EABT15770	3.68156	1.246033	6.22E-06	0.00018
EABT33814	-3.3277	3.112857	6.24E-06	0.00018
EABT18952	3.21301	7.809743	6.25E-06	0.00018
EABT3181	3.22511	5.912246	6.26E-06	0.00018
EABT17156	3.60201	1.486427	6.26E-06	0.00018
EABT28111	-3.8454	0.836625	6.29E-06	0.00018
EABT29945	8.17702	-0.850371	6.32E-06	0.00018
EABT36480	8.17702	-0.850371	6.32E-06	0.00018
EABT7788	8.17702	-0.850371	6.32E-06	0.00018
EABT23225	8.17702	-0.850371	6.32E-06	0.00018
EABT29873	8.17702	-0.850371	6.32E-06	0.00018
EABT28717	8.17702	-0.850371	6.32E-06	0.00018
EABT13967	8.17702	-0.850371	6.32E-06	0.00018
EABT14732	8.17702	-0.850371	6.32E-06	0.00018
EABT25468	8.17702	-0.850371	6.32E-06	0.00018
EABT24261	8.17702	-0.850371	6.32E-06	0.00018
EABT31657	8.17702	-0.850371	6.32E-06	0.00018
EABT14298	8.17702	-0.850371	6.32E-06	0.00018
EABT31535	3.57886	1.556144	6.36E-06	0.00018
EABT35668	-3.2702	3.920509	6.40E-06	0.00018
EABT4631	4.10808	0.427942	6.41E-06	0.00018
EABT29952	3.24617	4.711061	6.43E-06	0.00018
EABT14296	3.20586	9.177725	6.44E-06	0.00018
EABT23097	3.21942	5.90023	6.47E-06	0.00018
EABT36237	-3.9061	0.708067	6.51E-06	0.00018
EABT35454	4.7777	-0.172113	6.56E-06	0.00019
EABT3314	4.7777	-0.172113	6.56E-06	0.00019
EABT28096	4.7777	-0.172113	6.56E-06	0.00019
EABT21227	-3.278	3.6659	6.60E-06	0.00019
EABT31520	4.45038	0.056762	6.66E-06	0.00019
EABT16279	3.99122	0.569524	6.75E-06	0.00019
EABT15425	3.19701	8.807375	6.79E-06	0.00019
EABT26605	-3.4484	2.015059	6.85E-06	0.00019
EABT37540	-3.2483	4.073038	7.00E-06	0.0002
EABT19110	-3.8926	0.695793	7.01E-06	0.0002
EABT3359	5.39246	-0.471754	7.02E-06	0.0002
EABT28408	5.39246	-0.471754	7.02E-06	0.0002
EABT32355	5.39246	-0.471754	7.02E-06	0.0002
EABT13295	5.39246	-0.471754	7.02E-06	0.0002

EABT12252	-5.3845	-0.473858	7.02E-06	0.0002
EABT33286	3.73176	1.037596	7.05E-06	0.0002
EABT8751	-3.2229	4.725962	7.13E-06	0.0002
EABT15937	-3.8205	0.814092	7.21E-06	0.0002
EABT5186	-3.5478	1.532653	7.22E-06	0.0002
EABT2150	4.22887	0.237374	7.26E-06	0.0002
EABT20550	-3.528	1.600072	7.27E-06	0.0002
EABT23708	3.97629	0.555972	7.32E-06	0.0002
EABT20119	3.51864	1.667437	7.32E-06	0.0002
EABT4667	3.37139	2.435641	7.32E-06	0.0002
EABT29575	-3.3685	2.395058	7.48E-06	0.00021
EABT27318	3.23282	4.242028	7.49E-06	0.00021
EABT25513	4.75267	-0.194842	7.49E-06	0.00021
EABT3594	3.19087	6.172446	7.51E-06	0.00021
EABT24438	-3.2149	4.630038	7.54E-06	0.00021
EABT17667	-3.1771	8.6072	7.61E-06	0.00021
EABT2815	-4.0667	0.395348	7.66E-06	0.00021
EABT9511	8.13652	-0.886912	7.72E-06	0.00021
EABT37578	8.13652	-0.886912	7.72E-06	0.00021
EABT9425	8.13652	-0.886912	7.72E-06	0.00021
EABT13675	8.13652	-0.886912	7.72E-06	0.00021
EABT7200	8.13652	-0.886912	7.72E-06	0.00021
EABT2982	8.13652	-0.886912	7.72E-06	0.00021
EABT16002	8.13652	-0.886912	7.72E-06	0.00021
EABT35329	8.13652	-0.886912	7.72E-06	0.00021
EABT18604	8.13652	-0.886912	7.72E-06	0.00021
EABT9710	-8.1323	-0.888775	7.72E-06	0.00021
EABT6578	-8.1323	-0.888775	7.72E-06	0.00021
EABT14420	3.23264	4.091664	7.73E-06	0.00021
EABT26713	3.61299	1.295785	7.73E-06	0.00021
EABT20286	3.21207	4.639782	7.85E-06	0.00021
EABT16526	4.21004	0.220293	8.02E-06	0.00022
EABT17944	5.36152	-0.49978	8.23E-06	0.00022
EABT23090	5.36152	-0.49978	8.23E-06	0.00022
EABT11878	5.36152	-0.49978	8.23E-06	0.00022
EABT5732	5.36152	-0.49978	8.23E-06	0.00022
EABT25787	5.36152	-0.49978	8.23E-06	0.00022
EABT20428	5.36152	-0.49978	8.23E-06	0.00022
EABT33257	5.36152	-0.49978	8.23E-06	0.00022
EABT34613	3.80358	0.793878	8.27E-06	0.00022
EABT7778	-3.3371	2.490535	8.39E-06	0.00023
EABT16135	-3.2957	2.833603	8.45E-06	0.00023
EABT11453	-3.1802	5.265964	8.47E-06	0.00023
EABT25970	4.72719	-0.217936	8.55E-06	0.00023
EABT26708	3.52532	1.507686	8.56E-06	0.00023
EABT31054	-3.2055	4.13118	8.73E-06	0.00024
EABT30386	3.19581	4.441266	8.85E-06	0.00024
EABT14818	4.19097	0.203008	8.88E-06	0.00024
EABT14998	3.5175	1.500629	8.94E-06	0.00024
EABT32313	-3.1747	4.773474	9.25E-06	0.00025
EABT36778	3.5765	1.262795	9.45E-06	0.00025
EABT32109	3.14051	7.530055	9.46E-06	0.00025
EABT21673	8.09485	-0.924401	9.48E-06	0.00025
EABT33097	8.09485	-0.924401	9.48E-06	0.00025

EABT22691	8.09485	-0.924401	9.48E-06	0.00025
EABT34734	8.09485	-0.924401	9.48E-06	0.00025
EABT18473	8.09485	-0.924401	9.48E-06	0.00025
EABT24199	8.09485	-0.924401	9.48E-06	0.00025
EABT3265	8.09485	-0.924401	9.48E-06	0.00025
EABT15400	8.09485	-0.924401	9.48E-06	0.00025
EABT6136	8.09485	-0.924401	9.48E-06	0.00025
EABT6660	8.09485	-0.924401	9.48E-06	0.00025
EABT24324	8.09485	-0.924401	9.48E-06	0.00025
EABT17935	8.09485	-0.924401	9.48E-06	0.00025
EABT16209	8.09485	-0.924401	9.48E-06	0.00025
EABT35338	8.09485	-0.924401	9.48E-06	0.00025
EABT795	-8.0907	-0.926235	9.48E-06	0.00025
EABT36283	5.3299	-0.528361	9.66E-06	0.00025
EABT32738	5.3299	-0.528361	9.66E-06	0.00025
EABT29322	5.3299	-0.528361	9.66E-06	0.00025
EABT30300	5.3299	-0.528361	9.66E-06	0.00025
EABT36392	5.3299	-0.528361	9.66E-06	0.00025
EABT29700	5.3299	-0.528361	9.66E-06	0.00025
EABT22315	5.3299	-0.528361	9.66E-06	0.00025
EABT36707	5.3299	-0.528361	9.66E-06	0.00025
EABT29608	5.3299	-0.528361	9.66E-06	0.00025
EABT8829	-5.322	-0.530418	9.66E-06	0.00025
EABT30958	3.21183	3.651736	9.72E-06	0.00026
EABT29613	4.70125	-0.241404	9.79E-06	0.00026
EABT10831	4.70125	-0.241404	9.79E-06	0.00026
EABT15468	4.17163	0.185514	9.85E-06	0.00026
EABT17968	-3.1293	10.8881	9.91E-06	0.00026
EABT26069	3.35007	2.190221	9.94E-06	0.00026
EABT11551	3.56723	1.254429	9.94E-06	0.00026
EABT8671	3.18963	3.990771	1.01E-05	0.00026
EABT4511	-3.2912	2.563293	1.01E-05	0.00026
EABT11471	3.3762	2.003183	1.02E-05	0.00027
EABT35026	3.34519	2.185827	1.02E-05	0.00027
EABT23686	3.45455	1.609571	1.04E-05	0.00027
EABT33951	4.36308	-0.022342	1.06E-05	0.00028
EABT19917	4.36308	-0.022342	1.06E-05	0.00028
EABT6346	-3.1186	7.137408	1.08E-05	0.00028
EABT27625	-3.4389	1.600088	1.09E-05	0.00028
EABT29876	-3.5166	1.317193	1.09E-05	0.00028
EABT2200	-3.1127	8.091291	1.10E-05	0.00029
EABT1387	3.1435	4.920493	1.10E-05	0.00029
EABT19032	3.10833	9.239267	1.12E-05	0.00029
EABT19357	4.67484	-0.265261	1.12E-05	0.00029
EABT18090	4.67484	-0.265261	1.12E-05	0.00029
EABT19227	4.67484	-0.265261	1.12E-05	0.00029
EABT25480	4.67484	-0.265261	1.12E-05	0.00029
EABT15504	4.67484	-0.265261	1.12E-05	0.00029
EABT1295	-3.1072	8.732638	1.13E-05	0.00029
EABT32993	5.29757	-0.557519	1.14E-05	0.00029
EABT10784	5.29757	-0.557519	1.14E-05	0.00029
EABT17863	5.29757	-0.557519	1.14E-05	0.00029
EABT21543	5.29757	-0.557519	1.14E-05	0.00029
EABT27141	5.29757	-0.557519	1.14E-05	0.00029

EABT36954	5.29757	-0.557519	1.14E-05	0.00029
EABT22306	5.29757	-0.557519	1.14E-05	0.00029
EABT2579	-3.1063	7.826237	1.14E-05	0.00029
EABT2698	-3.1147	6.279912	1.15E-05	0.0003
EABT14866	3.68626	0.850459	1.16E-05	0.0003
EABT36157	8.05195	-0.962891	1.17E-05	0.0003
EABT14960	8.05195	-0.962891	1.17E-05	0.0003
EABT21964	8.05195	-0.962891	1.17E-05	0.0003
EABT20645	8.05195	-0.962891	1.17E-05	0.0003
EABT19599	8.05195	-0.962891	1.17E-05	0.0003
EABT6549	8.05195	-0.962891	1.17E-05	0.0003
EABT33577	8.05195	-0.962891	1.17E-05	0.0003
EABT13422	8.05195	-0.962891	1.17E-05	0.0003
EABT2812	8.05195	-0.962891	1.17E-05	0.0003
EABT25310	8.05195	-0.962891	1.17E-05	0.0003
EABT11377	8.05195	-0.962891	1.17E-05	0.0003
EABT20068	8.05195	-0.962891	1.17E-05	0.0003
EABT24002	8.05195	-0.962891	1.17E-05	0.0003
EABT6019	8.05195	-0.962891	1.17E-05	0.0003
EABT32913	3.36084	1.931532	1.17E-05	0.0003
EABT18965	-3.73	0.732331	1.18E-05	0.0003
EABT5918	-3.2418	2.723042	1.18E-05	0.0003
EABT27246	4.3404	-0.042815	1.19E-05	0.0003
EABT16396	3.40205	1.710861	1.19E-05	0.0003
EABT12415	-3.7941	0.606824	1.19E-05	0.0003
EABT20069	-3.1693	3.634114	1.19E-05	0.0003
EABT21306	3.98868	0.319838	1.22E-05	0.00031
EABT31060	-3.1276	4.632477	1.22E-05	0.00031
EABT11266	-3.1119	5.256088	1.24E-05	0.00031
EABT17802	-3.445	1.440039	1.27E-05	0.00032
EABT12641	-3.1746	3.334941	1.28E-05	0.00032
EABT36465	4.64793	-0.289519	1.29E-05	0.00032
EABT30563	4.64793	-0.289519	1.29E-05	0.00032
EABT24706	4.64793	-0.289519	1.29E-05	0.00032
EABT35122	4.64793	-0.289519	1.29E-05	0.00032
EABT19951	-4.6398	-0.291686	1.29E-05	0.00032
EABT13969	-3.7795	0.593654	1.29E-05	0.00032
EABT27708	3.15419	3.739751	1.29E-05	0.00032
EABT28385	-3.4617	1.364499	1.30E-05	0.00032
EABT30535	-3.3904	1.63266	1.31E-05	0.00033
EABT17771	-3.1496	3.63251	1.33E-05	0.00033
EABT9773	3.4452	1.435508	1.33E-05	0.00033
EABT9953	4.31736	-0.063582	1.34E-05	0.00033
EABT26986	4.31736	-0.063582	1.34E-05	0.00033
EABT21973	5.26449	-0.587279	1.34E-05	0.00033
EABT27290	5.26449	-0.587279	1.34E-05	0.00033
EABT20728	5.26449	-0.587279	1.34E-05	0.00033
EABT31932	5.26449	-0.587279	1.34E-05	0.00033
EABT1929	5.26449	-0.587279	1.34E-05	0.00033
EABT29251	5.26449	-0.587279	1.34E-05	0.00033
EABT30166	5.26449	-0.587279	1.34E-05	0.00033
EABT22450	4.11203	0.13172	1.35E-05	0.00033
EABT23085	-3.0929	5.355522	1.36E-05	0.00034
EABT20351	-3.1399	3.686242	1.38E-05	0.00034

EABT19712	3.10084	4.760596	1.42E-05	0.00035
EABT3923	8.00772	-1.002435	1.45E-05	0.00035
EABT3939	8.00772	-1.002435	1.45E-05	0.00035
EABT16413	8.00772	-1.002435	1.45E-05	0.00035
EABT29239	8.00772	-1.002435	1.45E-05	0.00035
EABT31933	8.00772	-1.002435	1.45E-05	0.00035
EABT24518	8.00772	-1.002435	1.45E-05	0.00035
EABT20496	8.00772	-1.002435	1.45E-05	0.00035
EABT24719	8.00772	-1.002435	1.45E-05	0.00035
EABT7682	8.00772	-1.002435	1.45E-05	0.00035
EABT3647	8.00772	-1.002435	1.45E-05	0.00035
EABT12692	8.00772	-1.002435	1.45E-05	0.00035
EABT14085	8.00772	-1.002435	1.45E-05	0.00035
EABT17799	8.00772	-1.002435	1.45E-05	0.00035
EABT28886	8.00772	-1.002435	1.45E-05	0.00035
EABT8936	8.00772	-1.002435	1.45E-05	0.00035
EABT32185	8.00772	-1.002435	1.45E-05	0.00035
EABT18798	-8.0035	-1.004206	1.45E-05	0.00035
EABT30829	-8.0035	-1.004206	1.45E-05	0.00035
EABT14611	4.62052	-0.314191	1.48E-05	0.00036
EABT37715	4.62052	-0.314191	1.48E-05	0.00036
EABT24246	4.62052	-0.314191	1.48E-05	0.00036
EABT1956	4.62052	-0.314191	1.48E-05	0.00036
EABT25268	-4.6124	-0.316338	1.48E-05	0.00036
EABT5215	-3.0913	4.568285	1.50E-05	0.00036
EABT29097	4.0916	0.113333	1.50E-05	0.00036
EABT19800	-4.0833	0.11098	1.50E-05	0.00036
EABT31108	-3.1605	3.08493	1.51E-05	0.00037
EABT28455	-3.118	3.741037	1.52E-05	0.00037
EABT22462	-3.054	7.016324	1.55E-05	0.00038
EABT25357	-3.5763	0.902236	1.57E-05	0.00038
EABT21125	3.17413	2.864481	1.57E-05	0.00038
EABT22091	-3.0684	5.508992	1.57E-05	0.00038
EABT18330	3.68398	0.68603	1.58E-05	0.00038
EABT18288	5.23065	-0.617665	1.59E-05	0.00038
EABT26794	5.23065	-0.617665	1.59E-05	0.00038
EABT10892	5.23065	-0.617665	1.59E-05	0.00038
EABT21471	5.23065	-0.617665	1.59E-05	0.00038
EABT13081	5.23065	-0.617665	1.59E-05	0.00038
EABT38056	5.23065	-0.617665	1.59E-05	0.00038
EABT34652	5.23065	-0.617665	1.59E-05	0.00038
EABT4476	5.23065	-0.617665	1.59E-05	0.00038
EABT34936	5.23065	-0.617665	1.59E-05	0.00038
EABT7072	5.23065	-0.617665	1.59E-05	0.00038
EABT33508	-5.2227	-0.619648	1.59E-05	0.00038
EABT25549	-3.0486	7.147321	1.59E-05	0.00038
EABT7429	3.04465	9.623401	1.60E-05	0.00038
EABT36310	-3.926	0.268489	1.62E-05	0.00039
EABT7473	-3.3638	1.532686	1.64E-05	0.00039
EABT35213	3.35506	1.596374	1.66E-05	0.0004
EABT6280	3.3829	1.464797	1.69E-05	0.0004
EABT30519	3.3829	1.464797	1.69E-05	0.0004
EABT5835	3.81781	0.412955	1.71E-05	0.00041
EABT12116	4.59257	-0.339293	1.71E-05	0.00041

EABT22093	4.59257	-0.339293	1.71E-05	0.00041
EABT212	4.59257	-0.339293	1.71E-05	0.00041
EABT28504	4.59257	-0.339293	1.71E-05	0.00041
EABT8813	4.59257	-0.339293	1.71E-05	0.00041
EABT35650	-4.5844	-0.341418	1.71E-05	0.00041
EABT22744	4.27016	-0.106036	1.71E-05	0.00041
EABT33221	4.27016	-0.106036	1.71E-05	0.00041
EABT34563	-4.2619	-0.108277	1.71E-05	0.00041
EABT35994	-3.1189	3.304962	1.73E-05	0.00041
EABT29926	3.03596	6.24703	1.78E-05	0.00042
EABT14220	3.29379	1.811206	1.78E-05	0.00042
EABT13905	3.91573	0.254221	1.79E-05	0.00042
EABT36487	7.9621	-1.043093	1.80E-05	0.00042
EABT28877	7.9621	-1.043093	1.80E-05	0.00042
EABT23422	7.9621	-1.043093	1.80E-05	0.00042
EABT12499	7.9621	-1.043093	1.80E-05	0.00042
EABT32690	7.9621	-1.043093	1.80E-05	0.00042
EABT16354	7.9621	-1.043093	1.80E-05	0.00042
EABT7591	7.9621	-1.043093	1.80E-05	0.00042
EABT4898	7.9621	-1.043093	1.80E-05	0.00042
EABT19630	7.9621	-1.043093	1.80E-05	0.00042
EABT5839	7.9621	-1.043093	1.80E-05	0.00042
EABT27706	7.9621	-1.043093	1.80E-05	0.00042
EABT4890	7.9621	-1.043093	1.80E-05	0.00042
EABT4498	7.9621	-1.043093	1.80E-05	0.00042
EABT36039	7.9621	-1.043093	1.80E-05	0.00042
EABT35340	7.9621	-1.043093	1.80E-05	0.00042
EABT279	7.9621	-1.043093	1.80E-05	0.00042
EABT30161	-7.9579	-1.044832	1.80E-05	0.00042
EABT37640	-7.9579	-1.044832	1.80E-05	0.00042
EABT23533	-3.1622	2.6832	1.81E-05	0.00042
EABT13050	-3.0742	3.990065	1.82E-05	0.00042
EABT22605	-3.3031	1.695676	1.82E-05	0.00043
EABT29214	-3.144	2.842018	1.83E-05	0.00043
EABT30800	3.04689	5.121272	1.83E-05	0.00043
EABT31058	4.04986	0.075843	1.87E-05	0.00043
EABT24353	4.04986	0.075843	1.87E-05	0.00043
EABT19525	4.04986	0.075843	1.87E-05	0.00043
EABT24447	3.80096	0.397841	1.87E-05	0.00043
EABT23956	3.80096	0.397841	1.87E-05	0.00043
EABT24560	-3.0551	4.410111	1.87E-05	0.00043
EABT4527	5.19599	-0.648705	1.89E-05	0.00044
EABT179	5.19599	-0.648705	1.89E-05	0.00044
EABT324	5.19599	-0.648705	1.89E-05	0.00044
EABT32824	5.19599	-0.648705	1.89E-05	0.00044
EABT4402	-5.1881	-0.650662	1.89E-05	0.00044
EABT7651	-3.2726	1.796846	1.91E-05	0.00044
EABT24491	3.14179	2.779577	1.93E-05	0.00044
EABT23286	-3.0436	4.580868	1.94E-05	0.00045
EABT33476	-3.0427	4.548808	1.96E-05	0.00045
EABT22025	3.8969	0.23734	1.97E-05	0.00045
EABT33392	3.8969	0.23734	1.97E-05	0.00045
EABT25094	3.8969	0.23734	1.97E-05	0.00045
EABT3718	-3.8886	0.234951	1.97E-05	0.00045

EABT28947	4.56407	-0.364839	1.98E-05	0.00045
EABT1768	-4.5559	-0.366942	1.98E-05	0.00045
EABT34232	-3.0421	4.4417	2.00E-05	0.00046
EABT1482	3.39025	1.295747	2.00E-05	0.00046
EABT29654	3.58419	0.758791	2.01E-05	0.00046
EABT4437	-3.1617	2.48345	2.02E-05	0.00046
EABT13581	3.78391	0.382568	2.04E-05	0.00047
EABT16031	3.78391	0.382568	2.04E-05	0.00047
EABT20701	-2.9993	9.398885	2.06E-05	0.00047
EABT31591	-3.0599	3.732064	2.08E-05	0.00047
EABT1017	4.02852	0.056726	2.08E-05	0.00047
EABT37562	4.02852	0.056726	2.08E-05	0.00047
EABT22445	4.02852	0.056726	2.08E-05	0.00047
EABT33271	-3.069	3.544569	2.09E-05	0.00047
EABT11608	-3.2753	1.670812	2.12E-05	0.00048
EABT15800	3.62746	0.635383	2.13E-05	0.00048
EABT25428	3.62746	0.635383	2.13E-05	0.00048
EABT5615	3.10988	2.913104	2.14E-05	0.00049
EABT37869	-3.1179	2.791005	2.15E-05	0.00049
EABT28274	3.14336	2.532173	2.16E-05	0.00049
EABT26557	3.87782	0.22026	2.18E-05	0.00049
EABT7194	3.87782	0.22026	2.18E-05	0.00049
EABT6299	4.22135	-0.149776	2.20E-05	0.0005
EABT17419	4.22135	-0.149776	2.20E-05	0.0005
EABT36618	3.76666	0.367131	2.24E-05	0.0005
EABT6548	-3.7583	0.364696	2.24E-05	0.0005
EABT25561	7.91499	-1.08493	2.25E-05	0.0005
EABT24333	7.91499	-1.08493	2.25E-05	0.0005
EABT31100	7.91499	-1.08493	2.25E-05	0.0005
EABT26467	7.91499	-1.08493	2.25E-05	0.0005
EABT14224	7.91499	-1.08493	2.25E-05	0.0005
EABT3580	7.91499	-1.08493	2.25E-05	0.0005
EABT5714	7.91499	-1.08493	2.25E-05	0.0005
EABT25077	7.91499	-1.08493	2.25E-05	0.0005
EABT29510	7.91499	-1.08493	2.25E-05	0.0005
EABT24747	7.91499	-1.08493	2.25E-05	0.0005
EABT15390	7.91499	-1.08493	2.25E-05	0.0005
EABT7317	7.91499	-1.08493	2.25E-05	0.0005
EABT22826	7.91499	-1.08493	2.25E-05	0.0005
EABT16787	7.91499	-1.08493	2.25E-05	0.0005
EABT20987	7.91499	-1.08493	2.25E-05	0.0005
EABT18369	7.91499	-1.08493	2.25E-05	0.0005
EABT7252	7.91499	-1.08493	2.25E-05	0.0005
EABT25683	-7.9108	-1.086636	2.25E-05	0.0005
EABT789	5.16047	-0.680427	2.25E-05	0.0005
EABT6003	5.16047	-0.680427	2.25E-05	0.0005
EABT32454	5.16047	-0.680427	2.25E-05	0.0005
EABT37692	5.16047	-0.680427	2.25E-05	0.0005
EABT15419	5.16047	-0.680427	2.25E-05	0.0005
EABT37509	5.16047	-0.680427	2.25E-05	0.0005
EABT10958	5.16047	-0.680427	2.25E-05	0.0005
EABT29271	-5.1525	-0.682358	2.25E-05	0.0005
EABT11753	4.535	-0.390845	2.29E-05	0.00051
EABT32526	4.535	-0.390845	2.29E-05	0.00051

EABT1162	4.535	-0.390845	2.29E-05	0.00051
EABT38012	4.535	-0.390845	2.29E-05	0.00051
EABT18060	4.535	-0.390845	2.29E-05	0.00051
EABT10782	4.535	-0.390845	2.29E-05	0.00051
EABT34594	4.535	-0.390845	2.29E-05	0.00051
EABT34166	3.61298	0.622438	2.30E-05	0.00051
EABT17302	-3.5492	0.732357	2.32E-05	0.00051
EABT6791	3.36261	1.271074	2.32E-05	0.00051
EABT10554	3.47243	0.936603	2.33E-05	0.00051
EABT27314	-3.0104	4.551374	2.33E-05	0.00051
EABT30136	4.00687	0.037352	2.33E-05	0.00051
EABT35021	3.12498	2.549366	2.35E-05	0.00052
EABT2780	-3.0418	3.583409	2.38E-05	0.00052
EABT33715	3.85849	0.202974	2.41E-05	0.00053
EABT29895	-3.1516	2.243121	2.41E-05	0.00053
EABT11794	-3.3449	1.26001	2.44E-05	0.00054
EABT180	-3.171	2.082672	2.45E-05	0.00054
EABT12466	4.19631	-0.172153	2.50E-05	0.00055
EABT14358	4.19631	-0.172153	2.50E-05	0.00055
EABT22161	4.19631	-0.172153	2.50E-05	0.00055
EABT6124	-4.1881	-0.174338	2.50E-05	0.00055
EABT23822	-3.0041	4.283573	2.52E-05	0.00055
EABT29982	3.00547	4.326195	2.53E-05	0.00055
EABT6650	-2.9982	4.332961	2.57E-05	0.00056
EABT17972	-3.0036	4.147099	2.58E-05	0.00056
EABT34478	-3.07	2.907372	2.60E-05	0.00057
EABT30849	-3.0139	3.799597	2.61E-05	0.00057
EABT3171	4.50533	-0.417329	2.65E-05	0.00058
EABT34768	4.50533	-0.417329	2.65E-05	0.00058
EABT21056	3.58357	0.596196	2.69E-05	0.00058
EABT9737	-3.1174	2.333061	2.69E-05	0.00058
EABT8142	-3.6407	0.469379	2.69E-05	0.00058
EABT18374	5.12406	-0.712862	2.69E-05	0.00058
EABT1365	5.12406	-0.712862	2.69E-05	0.00058
EABT22596	5.12406	-0.712862	2.69E-05	0.00058
EABT13444	5.12406	-0.712862	2.69E-05	0.00058
EABT12097	5.12406	-0.712862	2.69E-05	0.00058
EABT15051	5.12406	-0.712862	2.69E-05	0.00058
EABT3259	5.12406	-0.712862	2.69E-05	0.00058
EABT28870	5.12406	-0.712862	2.69E-05	0.00058
EABT17639	5.12406	-0.712862	2.69E-05	0.00058
EABT16546	5.12406	-0.712862	2.69E-05	0.00058
EABT19566	-5.1161	-0.714766	2.69E-05	0.00058
EABT23037	-5.1161	-0.714766	2.69E-05	0.00058
EABT1319	-5.1161	-0.714766	2.69E-05	0.00058
EABT12698	-2.9658	5.324001	2.74E-05	0.00059
EABT37627	3.27652	1.4502	2.75E-05	0.00059
EABT26364	3.15807	2.066624	2.75E-05	0.00059
EABT15410	-3.16	1.975048	2.80E-05	0.0006
EABT31823	7.86628	-1.128016	2.82E-05	0.0006
EABT2316	7.86628	-1.128016	2.82E-05	0.0006
EABT29151	7.86628	-1.128016	2.82E-05	0.0006
EABT13725	7.86628	-1.128016	2.82E-05	0.0006
EABT9772	7.86628	-1.128016	2.82E-05	0.0006

EABT16291	7.86628	-1.128016	2.82E-05	0.0006
EABT31961	7.86628	-1.128016	2.82E-05	0.0006
EABT34218	7.86628	-1.128016	2.82E-05	0.0006
EABT17810	7.86628	-1.128016	2.82E-05	0.0006
EABT32040	7.86628	-1.128016	2.82E-05	0.0006
EABT25763	7.86628	-1.128016	2.82E-05	0.0006
EABT10894	7.86628	-1.128016	2.82E-05	0.0006
EABT30443	7.86628	-1.128016	2.82E-05	0.0006
EABT17777	-7.8621	-1.129687	2.82E-05	0.0006
EABT33732	-7.8621	-1.129687	2.82E-05	0.0006
EABT29632	-3.2	1.732218	2.83E-05	0.0006
EABT37369	3.47329	0.805327	2.84E-05	0.0006
EABT32187	-2.988	4.081387	2.84E-05	0.0006
EABT2137	4.17083	-0.194883	2.84E-05	0.0006
EABT5150	4.17083	-0.194883	2.84E-05	0.0006
EABT33285	4.17083	-0.194883	2.84E-05	0.0006
EABT11154	-4.1626	-0.197048	2.84E-05	0.0006
EABT38145	-2.9482	6.555613	2.85E-05	0.0006
EABT38137	3.21695	1.679924	2.86E-05	0.00061
EABT4532	-3.1012	2.356649	2.87E-05	0.00061
EABT29496	3.96256	-0.002194	2.93E-05	0.00062
EABT2644	3.96256	-0.002194	2.93E-05	0.00062
EABT11090	3.63276	0.457337	2.93E-05	0.00062
EABT23980	-3.2015	1.670827	2.97E-05	0.00063
EABT21456	3.42478	0.89415	3.00E-05	0.00063
EABT24213	-2.9511	5.400694	3.01E-05	0.00064
EABT32907	2.93155	7.036597	3.05E-05	0.00064
EABT3498	2.9711	4.286739	3.06E-05	0.00064
EABT12334	4.47504	-0.444307	3.09E-05	0.00065
EABT24661	4.47504	-0.444307	3.09E-05	0.00065
EABT34490	4.47504	-0.444307	3.09E-05	0.00065
EABT9745	4.47504	-0.444307	3.09E-05	0.00065
EABT24508	-4.4669	-0.446343	3.09E-05	0.00065
EABT20469	-3.128	2.044467	3.09E-05	0.00065
EABT29915	3.23966	1.493485	3.09E-05	0.00065
EABT37952	-2.928	7.194198	3.10E-05	0.00065
EABT30670	3.55356	0.569467	3.14E-05	0.00066
EABT3842	3.55356	0.569467	3.14E-05	0.00066
EABT21357	-2.9393	5.647327	3.15E-05	0.00066
EABT30217	2.92016	8.939092	3.19E-05	0.00067
EABT36817	5.08671	-0.746043	3.23E-05	0.00067
EABT19608	5.08671	-0.746043	3.23E-05	0.00067
EABT34174	5.08671	-0.746043	3.23E-05	0.00067
EABT13712	5.08671	-0.746043	3.23E-05	0.00067
EABT19422	5.08671	-0.746043	3.23E-05	0.00067
EABT29848	5.08671	-0.746043	3.23E-05	0.00067
EABT18868	5.08671	-0.746043	3.23E-05	0.00067
EABT32297	5.08671	-0.746043	3.23E-05	0.00067
EABT20907	5.08671	-0.746043	3.23E-05	0.00067
EABT34212	5.08671	-0.746043	3.23E-05	0.00067
EABT12473	4.1449	-0.217977	3.24E-05	0.00067
EABT26470	4.1449	-0.217977	3.24E-05	0.00067
EABT12339	4.1449	-0.217977	3.24E-05	0.00067
EABT11580	4.1449	-0.217977	3.24E-05	0.00067

EABT13382	3.44733	0.782234	3.26E-05	0.00068
EABT19656	-3.439	0.779669	3.26E-05	0.00068
EABT16999	3.79888	0.14984	3.27E-05	0.00068
EABT23808	-2.9443	4.639755	3.28E-05	0.00068
EABT28315	3.93988	-0.02238	3.28E-05	0.00068
EABT13991	3.93988	-0.02238	3.28E-05	0.00068
EABT10659	-3.9316	-0.024616	3.28E-05	0.00068
EABT35256	-2.9226	6.029727	3.36E-05	0.00069
EABT2368	-2.9183	6.421343	3.37E-05	0.0007
EABT17097	-2.9405	4.586726	3.37E-05	0.0007
EABT25783	2.93112	5.284699	3.38E-05	0.0007
EABT18428	-3.0345	2.717032	3.38E-05	0.0007
EABT1316	-3.53	0.553436	3.40E-05	0.0007
EABT19471	-3.1153	1.985181	3.43E-05	0.0007
EABT30359	2.9099	6.897133	3.44E-05	0.00071
EABT14969	-2.9081	7.211553	3.45E-05	0.00071
EABT17917	-2.9231	5.147659	3.49E-05	0.00072
EABT6277	-3.0497	2.487044	3.52E-05	0.00072
EABT6891	-2.9016	8.347609	3.54E-05	0.00072
EABT31732	7.81588	-1.172428	3.57E-05	0.00072
EABT2694	7.81588	-1.172428	3.57E-05	0.00072
EABT7659	7.81588	-1.172428	3.57E-05	0.00072
EABT37036	7.81588	-1.172428	3.57E-05	0.00072
EABT32456	7.81588	-1.172428	3.57E-05	0.00072
EABT31314	7.81588	-1.172428	3.57E-05	0.00072
EABT3050	7.81588	-1.172428	3.57E-05	0.00072
EABT32325	7.81588	-1.172428	3.57E-05	0.00072
EABT24445	7.81588	-1.172428	3.57E-05	0.00072
EABT32363	7.81588	-1.172428	3.57E-05	0.00072
EABT32858	7.81588	-1.172428	3.57E-05	0.00072
EABT29325	7.81588	-1.172428	3.57E-05	0.00072
EABT25394	7.81588	-1.172428	3.57E-05	0.00072
EABT15577	7.81588	-1.172428	3.57E-05	0.00072
EABT4825	7.81588	-1.172428	3.57E-05	0.00072
EABT12751	7.81588	-1.172428	3.57E-05	0.00072
EABT2492	7.81588	-1.172428	3.57E-05	0.00072
EABT14708	7.81588	-1.172428	3.57E-05	0.00072
EABT21547	7.81588	-1.172428	3.57E-05	0.00072
EABT27042	7.81588	-1.172428	3.57E-05	0.00072
EABT13973	7.81588	-1.172428	3.57E-05	0.00072
EABT14712	7.81588	-1.172428	3.57E-05	0.00072
EABT7102	7.81588	-1.172428	3.57E-05	0.00072
EABT7199	-7.8117	-1.174064	3.57E-05	0.00072
EABT3967	-7.8117	-1.174064	3.57E-05	0.00072
EABT21185	4.44409	-0.4718	3.60E-05	0.00072
EABT16231	4.44409	-0.4718	3.60E-05	0.00072
EABT35292	4.44409	-0.4718	3.60E-05	0.00072
EABT27810	4.44409	-0.4718	3.60E-05	0.00072
EABT3462	-4.4359	-0.473812	3.60E-05	0.00072
EABT35682	2.90636	6.412609	3.60E-05	0.00072
EABT25301	3.5229	0.542233	3.69E-05	0.00074
EABT3790	-2.9138	5.0902	3.69E-05	0.00074
EABT7533	3.91685	-0.042853	3.69E-05	0.00074
EABT12269	-3.9086	-0.045071	3.69E-05	0.00074

EABT16400	2.92348	4.75685	3.70E-05	0.00074
EABT1073	4.11848	-0.241446	3.70E-05	0.00074
EABT941	3.16686	1.635512	3.74E-05	0.00075
EABT16895	3.18996	1.52163	3.75E-05	0.00075
EABT33379	-2.8881	7.244723	3.85E-05	0.00077
EABT22402	3.46001	0.648185	3.86E-05	0.00077
EABT18044	-3.3671	0.847836	3.88E-05	0.00077
EABT12392	5.04836	-0.780005	3.89E-05	0.00077
EABT31749	5.04836	-0.780005	3.89E-05	0.00077
EABT22406	5.04836	-0.780005	3.89E-05	0.00077
EABT18833	5.04836	-0.780005	3.89E-05	0.00077
EABT15710	5.04836	-0.780005	3.89E-05	0.00077
EABT2031	5.04836	-0.780005	3.89E-05	0.00077
EABT18391	5.04836	-0.780005	3.89E-05	0.00077
EABT11711	-5.0404	-0.781852	3.89E-05	0.00077
EABT21262	-2.9223	4.192607	3.95E-05	0.00079
EABT5343	-3.031	2.402708	3.95E-05	0.00079
EABT34206	3.00841	2.603029	4.08E-05	0.00081
EABT7534	2.94377	3.552807	4.10E-05	0.00081
EABT3822	-2.8714	10.06009	4.14E-05	0.00082
EABT20773	-3.5574	0.395411	4.15E-05	0.00082
EABT3043	3.89344	-0.063621	4.16E-05	0.00082
EABT7064	3.89344	-0.063621	4.16E-05	0.00082
EABT33344	3.89344	-0.063621	4.16E-05	0.00082
EABT10212	-3.8852	-0.065821	4.16E-05	0.00082
EABT2912	-3.2242	1.243262	4.17E-05	0.00083
EABT14856	4.41247	-0.499826	4.20E-05	0.00083
EABT5834	4.41247	-0.499826	4.20E-05	0.00083
EABT13094	4.41247	-0.499826	4.20E-05	0.00083
EABT38153	4.41247	-0.499826	4.20E-05	0.00083
EABT12069	4.09158	-0.265303	4.23E-05	0.00083
EABT15092	4.09158	-0.265303	4.23E-05	0.00083
EABT14453	-4.0833	-0.267407	4.23E-05	0.00083
EABT28508	-4.0833	-0.267407	4.23E-05	0.00083
EABT11623	2.87036	6.738797	4.27E-05	0.00084
EABT34672	-2.8749	6.165575	4.31E-05	0.00085
EABT37705	3.63975	0.254188	4.31E-05	0.00085
EABT33747	-2.874	6.169465	4.33E-05	0.00085
EABT13196	-3.4832	0.512034	4.34E-05	0.00085
EABT34038	3.05994	2.07137	4.37E-05	0.00086
EABT29284	3.35022	0.828032	4.43E-05	0.00087
EABT30036	2.92936	3.525255	4.45E-05	0.00087
EABT36669	3.73671	0.094674	4.50E-05	0.00087
EABT520	3.54849	0.382537	4.53E-05	0.00087
EABT17943	7.76365	-1.21825	4.53E-05	0.00087
EABT10854	7.76365	-1.21825	4.53E-05	0.00087
EABT4956	7.76365	-1.21825	4.53E-05	0.00087
EABT34079	7.76365	-1.21825	4.53E-05	0.00087
EABT28600	7.76365	-1.21825	4.53E-05	0.00087
EABT16584	7.76365	-1.21825	4.53E-05	0.00087
EABT14406	7.76365	-1.21825	4.53E-05	0.00087
EABT4723	7.76365	-1.21825	4.53E-05	0.00087
EABT1355	7.76365	-1.21825	4.53E-05	0.00087
EABT27491	7.76365	-1.21825	4.53E-05	0.00087

EABT4496	7.76365	-1.21825	4.53E-05	0.00087
EABT35603	7.76365	-1.21825	4.53E-05	0.00087
EABT23257	7.76365	-1.21825	4.53E-05	0.00087
EABT4725	7.76365	-1.21825	4.53E-05	0.00087
EABT16478	7.76365	-1.21825	4.53E-05	0.00087
EABT6343	7.76365	-1.21825	4.53E-05	0.00087
EABT6054	7.76365	-1.21825	4.53E-05	0.00087
EABT8501	7.76365	-1.21825	4.53E-05	0.00087
EABT36815	7.76365	-1.21825	4.53E-05	0.00087
EABT21288	7.76365	-1.21825	4.53E-05	0.00087
EABT23240	7.76365	-1.21825	4.53E-05	0.00087
EABT11357	7.76365	-1.21825	4.53E-05	0.00087
EABT37404	7.76365	-1.21825	4.53E-05	0.00087
EABT8866	7.76365	-1.21825	4.53E-05	0.00087
EABT752	7.76365	-1.21825	4.53E-05	0.00087
EABT15911	7.76365	-1.21825	4.53E-05	0.00087
EABT19590	7.76365	-1.21825	4.53E-05	0.00087
EABT7403	-7.7595	-1.21985	4.53E-05	0.00087
EABT36587	-7.7595	-1.21985	4.53E-05	0.00087
EABT4171	-7.7595	-1.21985	4.53E-05	0.00087
EABT29989	-7.7595	-1.21985	4.53E-05	0.00087
EABT32805	3.03531	2.176931	4.60E-05	0.00088
EABT16802	-3.0654	1.891424	4.64E-05	0.00089
EABT18101	3.86964	-0.084692	4.68E-05	0.00089
EABT3144	3.86964	-0.084692	4.68E-05	0.00089
EABT7524	2.86011	5.925047	4.69E-05	0.00089
EABT20936	5.00897	-0.814785	4.69E-05	0.00089
EABT10092	5.00897	-0.814785	4.69E-05	0.00089
EABT3191	5.00897	-0.814785	4.69E-05	0.00089
EABT1495	5.00897	-0.814785	4.69E-05	0.00089
EABT25930	5.00897	-0.814785	4.69E-05	0.00089
EABT23330	5.00897	-0.814785	4.69E-05	0.00089
EABT14073	5.00897	-0.814785	4.69E-05	0.00089
EABT37813	5.00897	-0.814785	4.69E-05	0.00089
EABT29403	5.00897	-0.814785	4.69E-05	0.00089
EABT33943	5.00897	-0.814785	4.69E-05	0.00089
EABT17707	5.00897	-0.814785	4.69E-05	0.00089
EABT19365	5.00897	-0.814785	4.69E-05	0.00089
EABT9614	5.00897	-0.814785	4.69E-05	0.00089
EABT16503	-5.001	-0.816603	4.69E-05	0.00089
EABT2082	-5.001	-0.816603	4.69E-05	0.00089
EABT12232	-2.8605	5.845809	4.74E-05	0.0009
EABT19807	3.62067	0.237307	4.76E-05	0.0009
EABT5563	3.62067	0.237307	4.76E-05	0.0009
EABT34459	-2.8871	4.16171	4.77E-05	0.00091
EABT33882	-2.8474	7.456005	4.78E-05	0.00091
EABT3309	-2.8514	6.524962	4.82E-05	0.00091
EABT36826	-2.9709	2.632627	4.83E-05	0.00092
EABT27578	-2.8559	5.92288	4.84E-05	0.00092
EABT29600	4.06417	-0.289561	4.85E-05	0.00092
EABT27273	4.06417	-0.289561	4.85E-05	0.00092
EABT19761	4.06417	-0.289561	4.85E-05	0.00092
EABT31250	2.96065	2.729108	4.89E-05	0.00093
EABT3396	4.38014	-0.528408	4.92E-05	0.00093

EABT35801	4.38014	-0.528408	4.92E-05	0.00093
EABT6711	4.38014	-0.528408	4.92E-05	0.00093
EABT18689	2.86875	4.794822	4.93E-05	0.00093
EABT3211	2.84179	7.51177	4.93E-05	0.00093
EABT36907	2.98053	2.486435	4.94E-05	0.00093
EABT9555	3.53103	0.3671	4.96E-05	0.00093
EABT6620	3.3665	0.710649	4.97E-05	0.00094
EABT36126	-2.8723	4.366298	4.99E-05	0.00094
EABT8468	3.71538	0.075806	5.01E-05	0.00094
EABT24339	3.71538	0.075806	5.01E-05	0.00094
EABT17052	3.71538	0.075806	5.01E-05	0.00094
EABT15113	2.93653	2.972913	5.04E-05	0.00095
EABT1559	-2.8515	5.262419	5.08E-05	0.00095
EABT6778	-3.1116	1.525821	5.09E-05	0.00096
EABT6963	2.92846	3.03038	5.14E-05	0.00097
EABT11540	3.84544	-0.106075	5.29E-05	0.00099
EABT35058	3.20556	1.131853	5.33E-05	0.001
EABT24916	-2.9031	3.245204	5.38E-05	0.00101
EABT14115	-2.9286	2.855931	5.41E-05	0.00101
EABT27493	3.51335	0.351496	5.42E-05	0.00101
EABT29659	3.51335	0.351496	5.42E-05	0.00101
EABT22421	-3.505	0.349137	5.42E-05	0.00101
EABT12712	3.14734	1.335895	5.45E-05	0.00102
EABT31230	3.44328	0.471813	5.56E-05	0.00104
EABT33613	4.03622	-0.314234	5.57E-05	0.00104
EABT14057	4.03622	-0.314234	5.57E-05	0.00104
EABT19183	4.03622	-0.314234	5.57E-05	0.00104
EABT28501	4.03622	-0.314234	5.57E-05	0.00104
EABT8527	-4.028	-0.316295	5.57E-05	0.00104
EABT33754	-3.6854	0.054458	5.59E-05	0.00104
EABT3003	-2.9169	2.915375	5.61E-05	0.00104
EABT15782	-2.8887	3.352718	5.61E-05	0.00104
EABT25636	2.816	8.481288	5.62E-05	0.00104
EABT25161	-3.036	1.814037	5.63E-05	0.00104
EABT6632	4.96847	-0.850424	5.69E-05	0.00104
EABT20276	4.96847	-0.850424	5.69E-05	0.00104
EABT11013	4.96847	-0.850424	5.69E-05	0.00104
EABT24033	4.96847	-0.850424	5.69E-05	0.00104
EABT14443	4.96847	-0.850424	5.69E-05	0.00104
EABT19663	4.96847	-0.850424	5.69E-05	0.00104
EABT32577	4.96847	-0.850424	5.69E-05	0.00104
EABT30237	4.96847	-0.850424	5.69E-05	0.00104
EABT21237	4.96847	-0.850424	5.69E-05	0.00104
EABT23062	4.96847	-0.850424	5.69E-05	0.00104
EABT34044	4.96847	-0.850424	5.69E-05	0.00104
EABT35302	4.96847	-0.850424	5.69E-05	0.00104
EABT36741	4.96847	-0.850424	5.69E-05	0.00104
EABT21172	4.96847	-0.850424	5.69E-05	0.00104
EABT22430	4.96847	-0.850424	5.69E-05	0.00104
EABT8168	2.98977	2.17692	5.71E-05	0.00104
EABT37552	2.81476	7.249603	5.72E-05	0.00104
EABT8217	-2.8232	6.03403	5.72E-05	0.00104
EABT28778	-2.8126	8.241579	5.73E-05	0.00104
EABT34361	2.81323	7.705229	5.74E-05	0.00104

EABT7956	-2.8192	6.430894	5.74E-05	0.00104
EABT13279	2.81953	6.239569	5.77E-05	0.00104
EABT6732	4.34707	-0.557567	5.78E-05	0.00104
EABT36315	4.34707	-0.557567	5.78E-05	0.00104
EABT5242	4.34707	-0.557567	5.78E-05	0.00104
EABT30024	4.34707	-0.557567	5.78E-05	0.00104
EABT4143	4.34707	-0.557567	5.78E-05	0.00104
EABT4139	4.34707	-0.557567	5.78E-05	0.00104
EABT35933	7.70945	-1.265574	5.79E-05	0.00104
EABT21233	7.70945	-1.265574	5.79E-05	0.00104
EABT10942	7.70945	-1.265574	5.79E-05	0.00104
EABT10608	7.70945	-1.265574	5.79E-05	0.00104
EABT29452	7.70945	-1.265574	5.79E-05	0.00104
EABT10520	7.70945	-1.265574	5.79E-05	0.00104
EABT9525	7.70945	-1.265574	5.79E-05	0.00104
EABT34795	7.70945	-1.265574	5.79E-05	0.00104
EABT1174	7.70945	-1.265574	5.79E-05	0.00104
EABT21531	7.70945	-1.265574	5.79E-05	0.00104
EABT35211	7.70945	-1.265574	5.79E-05	0.00104
EABT30660	7.70945	-1.265574	5.79E-05	0.00104
EABT37583	7.70945	-1.265574	5.79E-05	0.00104
EABT24414	7.70945	-1.265574	5.79E-05	0.00104
EABT221	7.70945	-1.265574	5.79E-05	0.00104
EABT1743	7.70945	-1.265574	5.79E-05	0.00104
EABT21139	7.70945	-1.265574	5.79E-05	0.00104
EABT31398	7.70945	-1.265574	5.79E-05	0.00104
EABT9962	7.70945	-1.265574	5.79E-05	0.00104
EABT25102	7.70945	-1.265574	5.79E-05	0.00104
EABT992	7.70945	-1.265574	5.79E-05	0.00104
EABT36236	7.70945	-1.265574	5.79E-05	0.00104
EABT4108	7.70945	-1.265574	5.79E-05	0.00104
EABT18969	7.70945	-1.265574	5.79E-05	0.00104
EABT31891	7.70945	-1.265574	5.79E-05	0.00104
EABT31972	-7.7053	-1.267137	5.79E-05	0.00104
EABT1604	3.58174	0.20294	5.79E-05	0.00104
EABT25994	3.58174	0.20294	5.79E-05	0.00104
EABT30640	3.58174	0.20294	5.79E-05	0.00104
EABT33225	3.06369	1.667343	5.81E-05	0.00105
EABT18959	3.49546	0.335722	5.94E-05	0.00107
EABT33415	3.82083	-0.12778	5.98E-05	0.00107
EABT35898	3.82083	-0.12778	5.98E-05	0.00107
EABT30011	-2.921	2.698724	6.00E-05	0.00108
EABT3369	-3.1058	1.379997	6.00E-05	0.00108
EABT22639	-3.0683	1.553307	6.02E-05	0.00108
EABT24832	-2.8114	6.186858	6.05E-05	0.00109
EABT31213	2.92281	2.642004	6.12E-05	0.0011
EABT24190	3.32432	0.67348	6.17E-05	0.00111
EABT33472	3.32432	0.67348	6.17E-05	0.00111
EABT11803	2.84947	3.890328	6.22E-05	0.00111
EABT11958	2.81825	5.136078	6.26E-05	0.00112
EABT33610	3.19663	1.027721	6.27E-05	0.00112
EABT35757	2.79833	6.889016	6.28E-05	0.00112
EABT24289	-2.8027	6.401437	6.28E-05	0.00112
EABT24386	2.79465	8.49416	6.30E-05	0.00112

EABT6327	-2.8315	4.238846	6.30E-05	0.00112
EABT16317	2.88998	2.975447	6.36E-05	0.00113
EABT30721	4.00772	-0.339336	6.41E-05	0.00114
EABT35219	4.00772	-0.339336	6.41E-05	0.00114
EABT27671	4.00772	-0.339336	6.41E-05	0.00114
EABT2872	-3.9995	-0.341375	6.41E-05	0.00114
EABT9622	-3.9995	-0.341375	6.41E-05	0.00114
EABT12726	-3.003	1.836585	6.45E-05	0.00115
EABT18970	-2.7984	6.242253	6.46E-05	0.00115
EABT15111	3.47734	0.319773	6.51E-05	0.00116
EABT17921	2.89561	2.822585	6.52E-05	0.00116
EABT13871	-2.9016	2.707927	6.58E-05	0.00117
EABT11	3.14398	1.167917	6.62E-05	0.00117
EABT9798	-2.8431	3.554864	6.72E-05	0.00119
EABT16159	-2.8127	4.467268	6.73E-05	0.00119
EABT2955	3.79579	-0.149816	6.77E-05	0.0012
EABT30369	3.79579	-0.149816	6.77E-05	0.0012
EABT5750	3.79579	-0.149816	6.77E-05	0.0012
EABT14106	3.79579	-0.149816	6.77E-05	0.0012
EABT20513	4.31322	-0.587327	6.80E-05	0.0012
EABT33545	4.31322	-0.587327	6.80E-05	0.0012
EABT36231	4.31322	-0.587327	6.80E-05	0.0012
EABT1429	4.31322	-0.587327	6.80E-05	0.0012
EABT30010	-4.3051	-0.589239	6.80E-05	0.0012
EABT22451	-2.9321	2.280646	6.81E-05	0.0012
EABT13148	-2.8718	2.985142	6.85E-05	0.00121
EABT4332	4.9268	-0.886965	6.91E-05	0.00121
EABT9505	4.9268	-0.886965	6.91E-05	0.00121
EABT1576	4.9268	-0.886965	6.91E-05	0.00121
EABT17606	4.9268	-0.886965	6.91E-05	0.00121
EABT25977	4.9268	-0.886965	6.91E-05	0.00121
EABT22188	4.9268	-0.886965	6.91E-05	0.00121
EABT33555	4.9268	-0.886965	6.91E-05	0.00121
EABT33925	4.9268	-0.886965	6.91E-05	0.00121
EABT1886	4.9268	-0.886965	6.91E-05	0.00121
EABT2224	4.9268	-0.886965	6.91E-05	0.00121
EABT21621	4.9268	-0.886965	6.91E-05	0.00121
EABT27033	4.9268	-0.886965	6.91E-05	0.00121
EABT37378	4.9268	-0.886965	6.91E-05	0.00121
EABT6744	2.93575	2.279401	6.98E-05	0.00122
EABT3234	-3.011	1.683359	6.99E-05	0.00122
EABT1490	3.15276	1.08544	7.01E-05	0.00123
EABT18381	-3.19	0.923515	7.08E-05	0.00124
EABT6655	-3.5334	0.165475	7.09E-05	0.00124
EABT3830	2.94886	2.14106	7.09E-05	0.00124
EABT15877	-2.8004	4.527392	7.12E-05	0.00124
EABT13857	-3.2497	0.744377	7.13E-05	0.00124
EABT27294	-3.2497	0.744377	7.13E-05	0.00124
EABT37253	-2.7976	4.565843	7.19E-05	0.00125
EABT31341	2.91626	2.398053	7.21E-05	0.00126
EABT3622	-3.0839	1.284862	7.26E-05	0.00126
EABT1527	2.88805	2.638785	7.27E-05	0.00126
EABT15358	3.10706	1.220387	7.31E-05	0.00127
EABT21538	2.92911	2.237574	7.37E-05	0.00127

EABT17812	3.97865	-0.364883	7.38E-05	0.00127
EABT19932	3.97865	-0.364883	7.38E-05	0.00127
EABT18359	-3.9704	-0.366898	7.38E-05	0.00127
EABT8493	7.65315	-1.314503	7.45E-05	0.00127
EABT6878	7.65315	-1.314503	7.45E-05	0.00127
EABT36087	7.65315	-1.314503	7.45E-05	0.00127
EABT25060	7.65315	-1.314503	7.45E-05	0.00127
EABT15824	7.65315	-1.314503	7.45E-05	0.00127
EABT32430	7.65315	-1.314503	7.45E-05	0.00127
EABT34541	7.65315	-1.314503	7.45E-05	0.00127
EABT23369	7.65315	-1.314503	7.45E-05	0.00127
EABT15480	7.65315	-1.314503	7.45E-05	0.00127
EABT17919	7.65315	-1.31E+00	7.45E-05	0.00127
EABT3797	7.65315	-1.314503	7.45E-05	0.00127
EABT35023	7.65315	-1.314503	7.45E-05	0.00127
EABT27039	7.65315	-1.314503	7.45E-05	0.00127
EABT10781	7.65315	-1.314503	7.45E-05	0.00127
EABT20043	7.65315	-1.314503	7.45E-05	0.00127
EABT7779	7.65315	-1.314503	7.45E-05	0.00127
EABT1820	7.65315	-1.314503	7.45E-05	0.00127
EABT18194	7.65315	-1.314503	7.45E-05	0.00127
EABT14385	7.65315	-1.314503	7.45E-05	0.00127
EABT18111	7.65315	-1.314503	7.45E-05	0.00127
EABT24746	7.65315	-1.314503	7.45E-05	0.00127
EABT15559	7.65315	-1.314503	7.45E-05	0.00127
EABT31469	7.65315	-1.314503	7.45E-05	0.00127
EABT36569	7.65315	-1.314503	7.45E-05	0.00127
EABT14985	7.65315	-1.314503	7.45E-05	0.00127
EABT26623	7.65315	-1.314503	7.45E-05	0.00127
EABT13090	7.65315	-1.314503	7.45E-05	0.00127
EABT12715	7.65315	-1.314503	7.45E-05	0.00127
EABT34477	7.65315	-1.314503	7.45E-05	0.00127
EABT28291	7.65315	-1.314503	7.45E-05	0.00127
EABT153	7.65315	-1.314503	7.45E-05	0.00127
EABT492	7.65315	-1.314503	7.45E-05	0.00127
EABT3425	7.65315	-1.314503	7.45E-05	0.00127
EABT7040	7.65315	-1.314503	7.45E-05	0.00127
EABT20991	7.65315	-1.314503	7.45E-05	0.00127
EABT19855	7.65315	-1.314503	7.45E-05	0.00127
EABT31969	7.65315	-1.314503	7.45E-05	0.00127
EABT33200	7.65315	-1.314503	7.45E-05	0.00127
EABT29523	-7.649	-1.316027	7.45E-05	0.00127
EABT2153	-7.649	-1.316027	7.45E-05	0.00127
EABT18425	-7.649	-1.316027	7.45E-05	0.00127
EABT10960	-7.649	-1.316027	7.45E-05	0.00127
EABT33226	2.9638	1.941897	7.49E-05	0.00127
EABT36412	-2.8971	2.41777	7.51E-05	0.00128
EABT24056	-2.8188	3.601681	7.53E-05	0.00128
EABT19339	-2.8312	3.335034	7.55E-05	0.00128
EABT19789	-2.8021	4.010111	7.57E-05	0.00128
EABT1944	2.86533	2.819744	7.59E-05	0.00128
EABT33725	-3.0042	1.619806	7.59E-05	0.00128
EABT26575	-2.7848	4.683201	7.59E-05	0.00128
EABT4680	3.77032	-0.172194	7.67E-05	0.0013

EABT20644	3.77032	-0.172194	7.67E-05	0.0013
EABT30506	-2.7673	5.888263	7.74E-05	0.00131
EABT7857	3.32413	0.528392	7.76E-05	0.00131
EABT17166	3.52131	0.149805	7.85E-05	0.00133
EABT30489	-2.8641	2.72616	7.85E-05	0.00133
EABT3138	4.27856	-0.617714	8.03E-05	0.00135
EABT15448	4.27856	-0.617714	8.03E-05	0.00135
EABT23245	4.27856	-0.617714	8.03E-05	0.00135
EABT31914	4.27856	-0.617714	8.03E-05	0.00135
EABT33606	4.27856	-0.617714	8.03E-05	0.00135
EABT32314	4.27856	-0.617714	8.03E-05	0.00135
EABT4645	4.27856	-0.617714	8.03E-05	0.00135
EABT22220	3.20031	0.816687	8.11E-05	0.00136
EABT36335	-2.7479	7.281428	8.17E-05	0.00137
EABT7239	2.81207	3.481096	8.19E-05	0.00138
EABT22996	3.23062	0.722803	8.21E-05	0.00138
EABT20217	2.74557	7.611202	8.24E-05	0.00138
EABT33425	-2.7754	4.397931	8.25E-05	0.00138
EABT8377	4.88389	-0.924455	8.44E-05	0.00141
EABT13424	4.88389	-0.924455	8.44E-05	0.00141
EABT9860	4.88389	-0.924455	8.44E-05	0.00141
EABT5887	4.88389	-0.924455	8.44E-05	0.00141
EABT8816	4.88389	-0.924455	8.44E-05	0.00141
EABT29072	4.88389	-0.924455	8.44E-05	0.00141
EABT37870	4.88389	-0.924455	8.44E-05	0.00141
EABT29624	4.88389	-0.924455	8.44E-05	0.00141
EABT20489	4.88389	-0.924455	8.44E-05	0.00141
EABT314	4.88389	-0.924455	8.44E-05	0.00141
EABT4970	-4.876	-0.926181	8.44E-05	0.00141
EABT35748	-2.7486	6.173383	8.44E-05	0.00141
EABT31441	2.77878	4.214135	8.47E-05	0.00141
EABT6767	3.94898	-0.390889	8.52E-05	0.00142
EABT18595	3.94898	-0.390889	8.52E-05	0.00142
EABT25083	3.94898	-0.390889	8.52E-05	0.00142
EABT12167	-3.9407	-0.392882	8.52E-05	0.00142
EABT23083	-3.9407	-0.392882	8.52E-05	0.00142
EABT12292	2.73699	9.637221	8.53E-05	0.00142
EABT4219	-2.9037	2.106118	8.60E-05	0.00143
EABT97	3.50059	0.131649	8.70E-05	0.00144
EABT21408	3.50059	0.131649	8.70E-05	0.00144
EABT11375	3.74438	-0.194924	8.71E-05	0.00144
EABT12120	3.6037	-0.022418	8.75E-05	0.00145
EABT3338	-3.5954	-0.024578	8.75E-05	0.00145
EABT13000	3.2167	0.710623	8.81E-05	0.00146
EABT19275	2.76273	4.584	8.81E-05	0.00146
EABT7470	-3.0459	1.25171	8.84E-05	0.00146
EABT7982	-2.834	2.796839	8.86E-05	0.00146
EABT3121	2.87662	2.363458	8.88E-05	0.00147
EABT4600	2.75242	5.064898	8.88E-05	0.00147
EABT3214	-2.7669	4.066035	9.05E-05	0.00149
EABT29886	3.29211	0.500365	9.13E-05	0.00151
EABT18659	-3.0734	1.110869	9.13E-05	0.00151
EABT5614	3.00526	1.427998	9.26E-05	0.00153
EABT20378	-3.1657	0.791303	9.26E-05	0.00153

EABT6682	3.09795	1.037484	9.30E-05	0.00153
EABT19036	-3.0896	1.034933	9.30E-05	0.00153
EABT17518	2.84508	2.518188	9.42E-05	0.00155
EABT34526	-2.7186	8.807978	9.43E-05	0.00155
EABT36798	-3.0488	1.174188	9.45E-05	0.00155
EABT21842	4.24304	-0.648754	9.50E-05	0.00155
EABT12254	4.24304	-0.648754	9.50E-05	0.00155
EABT23022	4.24304	-0.648754	9.50E-05	0.00155
EABT21527	4.24304	-0.648754	9.50E-05	0.00155
EABT8461	4.24304	-0.648754	9.50E-05	0.00155
EABT991	4.24304	-0.648754	9.50E-05	0.00155
EABT32696	4.24304	-0.648754	9.50E-05	0.00155
EABT11872	4.24304	-0.648754	9.50E-05	0.00155
EABT29884	4.24304	-0.648754	9.50E-05	0.00155
EABT21337	4.24304	-0.648754	9.50E-05	0.00155
EABT9611	4.24304	-0.648754	9.50E-05	0.00155
EABT37309	4.24304	-0.648754	9.50E-05	0.00155
EABT4756	-2.8822	2.08745	9.61E-05	0.00155
EABT28751	7.59455	-1.365149	9.64E-05	0.00155
EABT31847	7.59455	-1.365149	9.64E-05	0.00155
EABT26305	7.59455	-1.365149	9.64E-05	0.00155
EABT20536	7.59455	-1.365149	9.64E-05	0.00155
EABT34683	7.59455	-1.365149	9.64E-05	0.00155
EABT15030	7.59455	-1.365149	9.64E-05	0.00155
EABT32713	7.59455	-1.365149	9.64E-05	0.00155
EABT20450	7.59455	-1.365149	9.64E-05	0.00155
EABT21309	7.59455	-1.365149	9.64E-05	0.00155
EABT22700	7.59455	-1.365149	9.64E-05	0.00155
EABT33471	7.59455	-1.365149	9.64E-05	0.00155
EABT29083	7.59455	-1.365149	9.64E-05	0.00155
EABT7764	7.59455	-1.365149	9.64E-05	0.00155
EABT31001	7.59455	-1.365149	9.64E-05	0.00155
EABT36869	7.59455	-1.365149	9.64E-05	0.00155
EABT23720	7.59455	-1.365149	9.64E-05	0.00155
EABT13889	7.59455	-1.365149	9.64E-05	0.00155
EABT32267	7.59455	-1.365149	9.64E-05	0.00155
EABT36009	7.59455	-1.365149	9.64E-05	0.00155
EABT14481	7.59455	-1.365149	9.64E-05	0.00155
EABT7830	7.59455	-1.365149	9.64E-05	0.00155
EABT26103	7.59455	-1.365149	9.64E-05	0.00155
EABT29845	7.59455	-1.365149	9.64E-05	0.00155
EABT10180	7.59455	-1.365149	9.64E-05	0.00155
EABT8228	7.59455	-1.365149	9.64E-05	0.00155
EABT36407	7.59455	-1.365149	9.64E-05	0.00155
EABT7068	7.59455	-1.365149	9.64E-05	0.00155
EABT27365	7.59455	-1.365149	9.64E-05	0.00155
EABT14670	7.59455	-1.365149	9.64E-05	0.00155
EABT30339	7.59455	-1.365149	9.64E-05	0.00155
EABT1848	7.59455	-1.365149	9.64E-05	0.00155
EABT4052	7.59455	-1.365149	9.64E-05	0.00155
EABT2348	-7.5904	-1.366633	9.64E-05	0.00155
EABT2178	-7.5904	-1.366633	9.64E-05	0.00155
EABT101	-7.5904	-1.366633	9.64E-05	0.00155
EABT22149	3.07111	1.104163	9.64E-05	0.00155

EABT6374	3.07111	1.104163	9.64E-05	0.00155
EABT16542	3.47956	0.113262	9.66E-05	0.00155
EABT2111	-2.7417	4.427011	9.78E-05	0.00157
EABT6825	3.08673	1.027698	9.84E-05	0.00158
EABT18410	3.91868	-0.417373	9.86E-05	0.00158
EABT22749	-3.9104	-0.419343	9.86E-05	0.00158
EABT23023	3.71797	-0.218018	9.91E-05	0.00159
EABT35207	3.71797	-0.218018	9.91E-05	0.00159
EABT3506	3.71797	-0.218018	9.91E-05	0.00159
EABT24680	-2.7104	7.047708	9.99E-05	0.0016
EABT32544	-2.7199	5.664427	0.0001	0.00161
EABT26548	2.81241	2.750042	0.0001	0.00161
EABT18644	2.82411	2.583078	0.0001	0.00162
EABT5613	2.94653	1.62252	0.0001	0.00163
EABT7845	-2.7276	4.669944	0.0001	0.00163
EABT20560	-2.7534	3.707906	0.0001	0.00164
EABT20186	-2.7244	4.782339	0.0001	0.00164
EABT20642	4.83966	-0.962946	0.0001	0.00164
EABT15408	4.83966	-0.962946	0.0001	0.00164
EABT23015	4.83966	-0.962946	0.0001	0.00164
EABT11791	4.83966	-0.962946	0.0001	0.00164
EABT19452	4.83966	-0.962946	0.0001	0.00164
EABT9718	4.83966	-0.962946	0.0001	0.00164
EABT7541	4.83966	-0.962946	0.0001	0.00164
EABT10730	4.83966	-0.962946	0.0001	0.00164
EABT30614	4.83966	-0.962946	0.0001	0.00164
EABT22319	4.83966	-0.962946	0.0001	0.00164
EABT3588	4.83966	-0.962946	0.0001	0.00164
EABT18140	-4.8317	-0.964639	0.0001	0.00164
EABT20185	-4.8317	-0.964639	0.0001	0.00164
EABT425	-2.7103	6.065417	0.0001	0.00164
EABT19122	3.38316	0.237273	0.0001	0.00165
EABT23794	-2.7226	4.71854	0.0001	0.00165
EABT11615	-3.2125	0.580448	0.0001	0.00165
EABT12928	-2.7248	4.600028	0.0001	0.00165
EABT37105	-3.0283	1.156385	0.0001	0.00166
EABT36947	-2.7101	5.801073	0.00011	0.00166
EABT6207	-2.7076	5.898362	0.00011	0.00167
EABT18653	-2.8883	1.880663	0.00011	0.00168
EABT28270	-2.8523	2.13824	0.00011	0.0017
EABT21768	-2.6949	7.740537	0.00011	0.0017
EABT33100	-2.9555	1.454786	0.00011	0.0017
EABT24301	2.69346	7.057106	0.00011	0.00172
EABT29363	3.55649	-0.06366	0.00011	0.00174
EABT14369	-2.7776	2.964851	0.00011	0.00174
EABT25517	3.0264	1.149966	0.00011	0.00174
EABT7121	3.0264	1.149966	0.00011	0.00174
EABT236	2.70389	5.175893	0.00011	0.00175
EABT12056	-2.8971	1.744261	0.00011	0.00176
EABT20375	4.20663	-0.680477	0.00011	0.00176
EABT24918	4.20663	-0.680477	0.00011	0.00176
EABT5255	4.20663	-0.680477	0.00011	0.00176
EABT3130	-4.1985	-0.682308	0.00011	0.00176
EABT35616	-4.1985	-0.682308	0.00011	0.00176

EABT26328	3.20542	0.569409	0.00011	0.00176
EABT3609	3.69106	-0.241488	0.00011	0.00176
EABT12845	3.69106	-0.241488	0.00011	0.00176
EABT9219	3.69106	-0.241488	0.00011	0.00176
EABT12331	3.69106	-0.241488	0.00011	0.00176
EABT20707	3.69106	-0.241488	0.00011	0.00176
EABT36118	3.69106	-0.241488	0.00011	0.00176
EABT16842	3.69106	-0.241488	0.00011	0.00176
EABT13033	3.13389	0.758713	0.00011	0.00177
EABT10442	3.88774	-0.444353	0.00011	0.00178
EABT17631	3.88774	-0.444353	0.00011	0.00178
EABT11243	-3.8795	-0.446298	0.00011	0.00178
EABT2175	-3.8795	-0.446298	0.00011	0.00178
EABT18267	2.75556	3.264933	0.00011	0.00178
EABT32810	-2.7127	4.336016	0.00011	0.00179
EABT28454	-2.761	3.085039	0.00012	0.00179
EABT11462	-2.8429	2.092151	0.00012	0.00179
EABT22109	2.8322	2.258612	0.00012	0.0018
EABT22676	2.68036	6.803771	0.00012	0.00182
EABT11881	3.15962	0.660847	0.00012	0.00183
EABT1778	-2.9387	1.440146	0.00012	0.00183
EABT35041	-2.6753	8.965932	0.00012	0.00184
EABT17679	-2.8716	1.819747	0.00012	0.00185
EABT26133	3.43657	0.07577	0.00012	0.00185
EABT9208	3.43657	0.07577	0.00012	0.00185
EABT6937	3.43657	0.07577	0.00012	0.00185
EABT32480	-3.4283	0.073594	0.00012	0.00185
EABT6792	-3.0598	0.912975	0.00012	0.00187
EABT28995	-2.6737	6.954189	0.00012	0.00188
EABT19747	-3.2795	0.333409	0.00012	0.00188
EABT11046	-3.1119	0.744403	0.00012	0.00188
EABT10609	-2.7885	2.556649	0.00012	0.00188
EABT982	2.73386	3.413186	0.00012	0.00191
EABT31119	-2.7487	3.037191	0.00012	0.00191
EABT35160	2.66592	8.730139	0.00012	0.00191
EABT3316	-2.6652	8.940594	0.00012	0.00191
EABT11409	2.83496	2.080804	0.00013	0.00191
EABT38084	7.53348	-1.417636	0.00013	0.00191
EABT18387	7.53348	-1.417636	0.00013	0.00191
EABT22452	7.53348	-1.417636	0.00013	0.00191
EABT2465	7.53348	-1.417636	0.00013	0.00191
EABT32864	7.53348	-1.417636	0.00013	0.00191
EABT3702	7.53348	-1.417636	0.00013	0.00191
EABT23926	7.53348	-1.417636	0.00013	0.00191
EABT15339	7.53348	-1.417636	0.00013	0.00191
EABT35043	7.53348	-1.417636	0.00013	0.00191
EABT2559	7.53348	-1.417636	0.00013	0.00191
EABT36162	7.53348	-1.417636	0.00013	0.00191
EABT28727	7.53348	-1.417636	0.00013	0.00191
EABT17636	7.53348	-1.417636	0.00013	0.00191
EABT11150	7.53348	-1.417636	0.00013	0.00191
EABT34227	7.53348	-1.417636	0.00013	0.00191
EABT31353	7.53348	-1.417636	0.00013	0.00191
EABT32139	7.53348	-1.417636	0.00013	0.00191

EABT24939	7.53348	-1.417636	0.00013	0.00191
EABT34698	7.53348	-1.417636	0.00013	0.00191
EABT2770	7.53348	-1.417636	0.00013	0.00191
EABT9022	7.53348	-1.417636	0.00013	0.00191
EABT2855	7.53348	-1.417636	0.00013	0.00191
EABT25493	7.53348	-1.417636	0.00013	0.00191
EABT28821	7.53348	-1.417636	0.00013	0.00191
EABT9784	7.53348	-1.417636	0.00013	0.00191
EABT26571	7.53348	-1.417636	0.00013	0.00191
EABT10193	7.53348	-1.417636	0.00013	0.00191
EABT37520	7.53348	-1.417636	0.00013	0.00191
EABT20433	7.53348	-1.417636	0.00013	0.00191
EABT35178	7.53348	-1.417636	0.00013	0.00191
EABT6538	7.53348	-1.417636	0.00013	0.00191
EABT18651	-7.5293	-1.419081	0.00013	0.00191
EABT6450	-2.8489	1.891478	0.00013	0.00192
EABT5496	3.3437	0.202906	0.00013	0.00192
EABT16177	-3.3354	0.200683	0.00013	0.00192
EABT4374	-3.3354	0.200683	0.00013	0.00192
EABT16057	-3.2175	0.440306	0.00013	0.00192
EABT26226	4.79404	-1.00249	0.00013	0.00192
EABT16103	4.79404	-1.00249	0.00013	0.00192
EABT6627	4.79404	-1.00249	0.00013	0.00192
EABT12390	4.79404	-1.00249	0.00013	0.00192
EABT24527	4.79404	-1.00249	0.00013	0.00192
EABT24219	4.79404	-1.00249	0.00013	0.00192
EABT10066	4.79404	-1.00249	0.00013	0.00192
EABT37030	4.79404	-1.00249	0.00013	0.00192
EABT35146	4.79404	-1.00249	0.00013	0.00192
EABT17445	4.79404	-1.00249	0.00013	0.00192
EABT30367	-4.7861	-1.004151	0.00013	0.00192
EABT38057	-4.7861	-1.004151	0.00013	0.00192
EABT36547	-4.7861	-1.004151	0.00013	0.00192
EABT34275	2.65929	12.97923	0.00013	0.00193
EABT34567	-2.7206	3.434466	0.00013	0.00194
EABT689	3.66365	-0.265345	0.00013	0.00194
EABT8203	3.66365	-0.265345	0.00013	0.00194
EABT8668	3.66365	-0.265345	0.00013	0.00194
EABT7999	-3.6554	-0.267365	0.00013	0.00194
EABT11811	-2.6601	7.021219	0.00013	0.00196
EABT6267	3.10644	0.734855	0.00013	0.00196
EABT14687	2.91679	1.479125	0.00013	0.00196
EABT7196	-3.0209	0.975254	0.00013	0.00198
EABT9136	-3.1657	0.539825	0.00013	0.00198
EABT12035	-2.7171	3.374113	0.00013	0.00199
EABT18690	3.85612	-0.471846	0.00013	0.00199
EABT6610	3.85612	-0.471846	0.00013	0.00199
EABT24344	3.85612	-0.471846	0.00013	0.00199
EABT13673	3.41459	0.056653	0.00013	0.00199
EABT20089	3.26952	0.31974	0.00013	0.00199
EABT29939	2.7592	2.679929	0.00013	0.00201
EABT22643	4.16928	-0.712913	0.00013	0.00201
EABT33979	4.16928	-0.712913	0.00013	0.00201
EABT33530	-4.1611	-0.714716	0.00013	0.00201

EABT20429	2.83776	1.920876	0.00014	0.00203
EABT33689	2.6572	6.136323	0.00014	0.00204
EABT26248	-2.7474	2.767838	0.00014	0.00204
EABT12376	-2.8227	1.954671	0.00014	0.00205
EABT21270	-2.668	4.835083	0.00014	0.00205
EABT34673	-2.6627	5.06783	0.00014	0.00206
EABT30514	3.20881	0.427819	0.00014	0.00206
EABT8130	-2.6829	4.066062	0.00014	0.00207
EABT6793	3.50769	-0.106114	0.00014	0.00208
EABT34007	3.50769	-0.106114	0.00014	0.00208
EABT19087	2.64413	7.737228	0.00014	0.00209
EABT9448	2.66405	5.011271	0.00014	0.0021
EABT34833	2.87521	1.615987	0.00014	0.0021
EABT22979	-3.1498	0.526026	0.00014	0.00212
EABT27328	-3.1498	0.526026	0.00014	0.00212
EABT32002	-2.6835	3.793948	0.00014	0.00214
EABT25914	-2.6588	4.832285	0.00014	0.00214
EABT2213	2.84823	1.746961	0.00014	0.00215
EABT16170	2.65731	5.072023	0.00015	0.00215
EABT26299	3.6357	-0.289604	0.00015	0.00218
EABT9489	3.6357	-0.289604	0.00015	0.00218
EABT13833	3.6357	-0.289604	0.00015	0.00218
EABT21909	3.6357	-0.289604	0.00015	0.00218
EABT8225	3.6357	-0.289604	0.00015	0.00218
EABT29220	-3.6274	-0.291601	0.00015	0.00218
EABT6974	2.83291	1.828105	0.00015	0.00218
EABT28891	2.93786	1.228913	0.00015	0.00219
EABT26929	-2.914	1.284901	0.00015	0.00222
EABT24722	3.19156	0.412862	0.00015	0.00222
EABT4272	-2.6279	8.744427	0.00015	0.00224
EABT28532	-2.9544	1.092284	0.00015	0.00225
EABT18859	2.62455	8.638136	0.00015	0.00227
EABT25541	3.14209	0.514417	0.00015	0.00227
EABT36094	3.82378	-0.499873	0.00015	0.00227
EABT25570	3.82378	-0.499873	0.00015	0.00227
EABT399	3.82378	-0.499873	0.00015	0.00227
EABT13877	3.82378	-0.499873	0.00015	0.00227
EABT28136	3.82378	-0.499873	0.00015	0.00227
EABT3788	3.82378	-0.499873	0.00015	0.00227
EABT11343	-3.2948	0.16551	0.00015	0.00227
EABT10320	-2.6235	6.980696	0.00016	0.00228
EABT18224	4.74693	-1.043149	0.00016	0.00228
EABT1838	4.74693	-1.043149	0.00016	0.00228
EABT4768	4.74693	-1.043149	0.00016	0.00228
EABT28955	4.74693	-1.043149	0.00016	0.00228
EABT5181	4.74693	-1.043149	0.00016	0.00228
EABT29898	4.74693	-1.043149	0.00016	0.00228
EABT18500	4.74693	-1.043149	0.00016	0.00228
EABT23170	4.74693	-1.043149	0.00016	0.00228
EABT7290	4.74693	-1.043149	0.00016	0.00228
EABT15803	4.74693	-1.043149	0.00016	0.00228
EABT26799	4.74693	-1.043149	0.00016	0.00228
EABT27048	4.74693	-1.043149	0.00016	0.00228
EABT15610	4.74693	-1.043149	0.00016	0.00228

EABT18138	4.74693	-1.043149	0.00016	0.00228
EABT33586	4.74693	-1.043149	0.00016	0.00228
EABT15218	4.74693	-1.043149	0.00016	0.00228
EABT28151	4.74693	-1.043149	0.00016	0.00228
EABT23938	4.74693	-1.043149	0.00016	0.00228
EABT8677	4.74693	-1.043149	0.00016	0.00228
EABT2560	4.74693	-1.043149	0.00016	0.00228
EABT6673	4.74693	-1.043149	0.00016	0.00228
EABT4606	4.74693	-1.043149	0.00016	0.00228
EABT10124	4.74693	-1.043149	0.00016	0.00228
EABT36202	4.74693	-1.043149	0.00016	0.00228
EABT12041	-4.739	-1.044776	0.00016	0.00228
EABT18700	-4.739	-1.044776	0.00016	0.00228
EABT27959	-4.739	-1.044776	0.00016	0.00228
EABT17267	-4.739	-1.044776	0.00016	0.00228
EABT32937	2.91287	1.279248	0.00016	0.00229
EABT13852	2.97167	1.017823	0.00016	0.00231
EABT25201	4.13093	-0.746094	0.00016	0.00232
EABT31685	4.13093	-0.746094	0.00016	0.00232
EABT28446	4.13093	-0.746094	0.00016	0.00232
EABT7709	4.13093	-0.746094	0.00016	0.00232
EABT33278	-4.1228	-0.747868	0.00016	0.00232
EABT30814	-2.6384	4.724623	0.00016	0.00233
EABT6440	3.06427	0.698312	0.00016	0.00233
EABT35557	2.6172	7.972364	0.00016	0.00233
EABT37078	-2.6265	5.802891	0.00016	0.00233
EABT32433	-2.6179	6.843969	0.00016	0.00233
EABT12099	2.70725	2.858846	0.00016	0.00233
EABT9368	-2.7299	2.532709	0.00016	0.00233
EABT17447	2.62175	6.264709	0.00016	0.00233
EABT9441	-2.6716	3.447354	0.00016	0.00233
EABT11083	-3.1658	0.395473	0.00016	0.00233
EABT23523	7.4697	-1.472105	0.00016	0.00233
EABT683	7.4697	-1.472105	0.00016	0.00233
EABT6344	7.4697	-1.472105	0.00016	0.00233
EABT17605	7.4697	-1.472105	0.00016	0.00233
EABT8074	7.4697	-1.472105	0.00016	0.00233
EABT17630	7.4697	-1.472105	0.00016	0.00233
EABT6245	7.4697	-1.472105	0.00016	0.00233
EABT34727	7.4697	-1.472105	0.00016	0.00233
EABT13610	7.4697	-1.472105	0.00016	0.00233
EABT37774	7.4697	-1.472105	0.00016	0.00233
EABT33260	7.4697	-1.472105	0.00016	0.00233
EABT13062	7.4697	-1.472105	0.00016	0.00233
EABT26894	7.4697	-1.472105	0.00016	0.00233
EABT21534	7.4697	-1.472105	0.00016	0.00233
EABT2533	7.4697	-1.472105	0.00016	0.00233
EABT5519	7.4697	-1.472105	0.00016	0.00233
EABT27712	7.4697	-1.472105	0.00016	0.00233
EABT10794	7.4697	-1.472105	0.00016	0.00233
EABT9024	7.4697	-1.472105	0.00016	0.00233
EABT4820	7.4697	-1.472105	0.00016	0.00233
EABT12795	7.4697	-1.472105	0.00016	0.00233
EABT9272	7.4697	-1.472105	0.00016	0.00233

EABT34857	7.4697	-1.472105	0.00016	0.00233
EABT19810	7.4697	-1.472105	0.00016	0.00233
EABT31168	7.4697	-1.472105	0.00016	0.00233
EABT36484	7.4697	-1.472105	0.00016	0.00233
EABT5956	7.4697	-1.472105	0.00016	0.00233
EABT225	7.4697	-1.472105	0.00016	0.00233
EABT8428	7.4697	-1.472105	0.00016	0.00233
EABT14894	7.4697	-1.472105	0.00016	0.00233
EABT20619	7.4697	-1.472105	0.00016	0.00233
EABT25487	7.4697	-1.472105	0.00016	0.00233
EABT37663	7.4697	-1.472105	0.00016	0.00233
EABT22225	7.4697	-1.472105	0.00016	0.00233
EABT28319	7.4697	-1.472105	0.00016	0.00233
EABT19207	7.4697	-1.472105	0.00016	0.00233
EABT22442	7.4697	-1.472105	0.00016	0.00233
EABT26920	7.4697	-1.472105	0.00016	0.00233
EABT17255	7.4697	-1.472105	0.00016	0.00233
EABT1279	7.4697	-1.472105	0.00016	0.00233
EABT33594	-7.4655	-1.473507	0.00016	0.00233
EABT29107	-7.4655	-1.473507	0.00016	0.00233
EABT28609	-7.4655	-1.473507	0.00016	0.00233
EABT36975	-7.4655	-1.473507	0.00016	0.00233
EABT27778	-7.4655	-1.473507	0.00016	0.00233
EABT2920	-7.4655	-1.473507	0.00016	0.00233
EABT3585	-7.4655	-1.473507	0.00016	0.00233
EABT5257	-7.4655	-1.473507	0.00016	0.00233
EABT20451	3.36959	0.01764	0.00017	0.00233
EABT22071	-3.3613	0.015518	0.00017	0.00233
EABT31247	-3.3613	0.015518	0.00017	0.00233
EABT978	-2.6389	4.331088	0.00017	0.00236
EABT25719	-2.8049	1.762057	0.00017	0.00237
EABT30103	3.6072	-0.314277	0.00017	0.00238
EABT17969	3.6072	-0.314277	0.00017	0.00238
EABT20412	-2.615	6.165103	0.00017	0.00238
EABT17188	-2.6082	7.384626	0.00017	0.00239
EABT6773	2.74735	2.283465	0.00017	0.0024
EABT15893	3.28241	0.14977	0.00017	0.00241
EABT20175	-2.6353	4.269259	0.00017	0.00242
EABT36608	-2.6581	3.527475	0.00017	0.00242
EABT4710	2.61032	6.136876	0.00017	0.00244
EABT12062	3.01956	0.77047	0.00017	0.00245
EABT24327	2.60203	6.828606	0.00018	0.00247
EABT18394	2.62428	4.761927	0.00018	0.00248
EABT36301	3.45717	-0.149856	0.00018	0.0025
EABT30913	-2.6184	4.696409	0.00018	0.0025
EABT14554	2.65218	3.567986	0.00018	0.00252
EABT28953	2.59462	8.707266	0.00018	0.00253
EABT8720	3.79071	-0.528455	0.00018	0.00253
EABT1059	3.79071	-0.528455	0.00018	0.00253
EABT4664	3.79071	-0.528455	0.00018	0.00253
EABT13211	-3.7825	-0.530324	0.00018	0.00253
EABT161	-3.7825	-0.530324	0.00018	0.00253
EABT6353	2.70391	2.583042	0.00018	0.00253
EABT13605	-2.7537	2.015224	0.00018	0.00254

EABT16641	-2.8513	1.364629	0.00018	0.00256
EABT27195	2.6285	4.142238	0.00018	0.00257
EABT11826	3.06954	0.582808	0.00018	0.00257
EABT32445	2.62529	4.263894	0.00018	0.00257
EABT15055	3.34655	-0.002269	0.00018	0.00257
EABT17234	-3.3382	-0.004372	0.00018	0.00257
EABT31722	-3.3382	-0.004372	0.00018	0.00257
EABT2041	-2.7842	1.744291	0.00019	0.00259
EABT12345	3.26139	0.131614	0.00019	0.00264
EABT7077	-2.5909	6.17399	0.00019	0.00266
EABT18221	4.09154	-0.780056	0.00019	0.00266
EABT1217	4.09154	-0.780056	0.00019	0.00266
EABT2540	4.09154	-0.780056	0.00019	0.00266
EABT27300	4.09154	-0.780056	0.00019	0.00266
EABT34107	4.09154	-0.780056	0.00019	0.00266
EABT30970	4.09154	-0.780056	0.00019	0.00266
EABT18320	4.09154	-0.780056	0.00019	0.00266
EABT11819	4.09154	-0.780056	0.00019	0.00266
EABT9185	4.09154	-0.780056	0.00019	0.00266
EABT20997	4.09154	-0.780056	0.00019	0.00266
EABT24668	4.09154	-0.780056	0.00019	0.00266
EABT27349	-4.0834	-0.7818	0.00019	0.00266
EABT21448	-4.0834	-0.7818	0.00019	0.00266
EABT20873	-2.6427	3.339045	0.00019	0.00267
EABT9007	3.57813	-0.33938	0.00019	0.00267
EABT4321	-3.5699	-0.341331	0.00019	0.00267
EABT14255	-3.5699	-0.341331	0.00019	0.00267
EABT16786	2.58046	8.248212	0.00019	0.00268
EABT30277	4.69823	-1.084987	0.0002	0.00268
EABT31151	4.69823	-1.084987	0.0002	0.00268
EABT5043	4.69823	-1.084987	0.0002	0.00268
EABT10056	4.69823	-1.084987	0.0002	0.00268
EABT33414	4.69823	-1.084987	0.0002	0.00268
EABT21385	4.69823	-1.084987	0.0002	0.00268
EABT5001	4.69823	-1.084987	0.0002	0.00268
EABT15508	4.69823	-1.084987	0.0002	0.00268
EABT17963	4.69823	-1.084987	0.0002	0.00268
EABT22262	4.69823	-1.084987	0.0002	0.00268
EABT26130	4.69823	-1.084987	0.0002	0.00268
EABT15495	4.69823	-1.084987	0.0002	0.00268
EABT6257	4.69823	-1.084987	0.0002	0.00268
EABT37497	4.69823	-1.084987	0.0002	0.00268
EABT23507	4.69823	-1.084987	0.0002	0.00268
EABT12546	4.69823	-1.084987	0.0002	0.00268
EABT9914	-4.6903	-1.086579	0.0002	0.00268
EABT2400	-4.6903	-1.086579	0.0002	0.00268
EABT23912	3.09269	0.471753	0.0002	0.0027
EABT457	3.09269	0.471753	0.0002	0.0027
EABT2008	3.09269	0.471753	0.0002	0.0027
EABT12927	2.5912	5.279961	0.0002	0.00271
EABT19554	2.72413	2.198809	0.0002	0.00271
EABT25849	-2.9837	0.744429	0.0002	0.00274
EABT32981	2.8136	1.507532	0.0002	0.00274
EABT9816	-2.8138	1.454821	0.0002	0.00274

EABT18745	3.43123	-0.172234	0.0002	0.00275
EABT17704	3.43123	-0.172234	0.0002	0.00275
EABT24372	-2.5882	5.386082	0.0002	0.00276
EABT29630	2.60439	4.367379	0.0002	0.00276
EABT36675	-2.624	3.54812	0.0002	0.00277
EABT25238	2.63013	3.486405	0.0002	0.00277
EABT23901	-2.6038	4.149475	0.0002	0.00279
EABT15352	2.56818	8.999621	0.00021	0.00281
EABT33655	2.76615	1.770593	0.00021	0.00281
EABT33309	3.32314	-0.022456	0.00021	0.00282
EABT11447	3.75687	-0.557614	0.00021	0.00287
EABT5268	3.75687	-0.557614	0.00021	0.00287
EABT10026	3.75687	-0.557614	0.00021	0.00287
EABT18841	3.75687	-0.557614	0.00021	0.00287
EABT30867	3.75687	-0.557614	0.00021	0.00287
EABT9064	3.75687	-0.557614	0.00021	0.00287
EABT30388	3.75687	-0.557614	0.00021	0.00287
EABT24858	3.75687	-0.557614	0.00021	0.00287
EABT8425	-3.7486	-0.559457	0.00021	0.00287
EABT2248	-3.7486	-0.559457	0.00021	0.00287
EABT2046	-2.6114	3.655034	0.00021	0.00287
EABT11354	3.1741	0.237239	0.00021	0.00287
EABT12197	-3.1658	0.235052	0.00021	0.00287
EABT23527	-2.6345	3.09913	0.00021	0.00289
EABT23971	2.7593	1.764714	0.00021	0.00289
EABT33616	-3.0675	0.454976	0.00021	0.00289
EABT16642	-3.0675	0.454976	0.00021	0.00289
EABT11864	3.03822	0.555828	0.00021	0.00289
EABT1401	-2.6164	3.44003	0.00022	0.00289
EABT9877	7.40297	-1.52871	0.00022	0.00289
EABT9414	7.40297	-1.52871	0.00022	0.00289
EABT24941	7.40297	-1.52871	0.00022	0.00289
EABT4572	7.40297	-1.52871	0.00022	0.00289
EABT35739	7.40297	-1.52871	0.00022	0.00289
EABT1294	7.40297	-1.52871	0.00022	0.00289
EABT3979	7.40297	-1.52871	0.00022	0.00289
EABT27613	7.40297	-1.52871	0.00022	0.00289
EABT29638	7.40297	-1.52871	0.00022	0.00289
EABT10786	7.40297	-1.52871	0.00022	0.00289
EABT25090	7.40297	-1.52871	0.00022	0.00289
EABT13477	7.40297	-1.52871	0.00022	0.00289
EABT182	7.40297	-1.52871	0.00022	0.00289
EABT16043	7.40297	-1.52871	0.00022	0.00289
EABT23267	7.40297	-1.52871	0.00022	0.00289
EABT37893	7.40297	-1.52871	0.00022	0.00289
EABT1840	7.40297	-1.52871	0.00022	0.00289
EABT22084	7.40297	-1.52871	0.00022	0.00289
EABT24665	7.40297	-1.52871	0.00022	0.00289
EABT32453	7.40297	-1.52871	0.00022	0.00289
EABT30071	7.40297	-1.52871	0.00022	0.00289
EABT34285	7.40297	-1.52871	0.00022	0.00289
EABT15781	7.40297	-1.52871	0.00022	0.00289
EABT1627	7.40297	-1.52871	0.00022	0.00289
EABT11825	7.40297	-1.52871	0.00022	0.00289

EABT23724	7.40297	-1.52871	0.00022	0.00289
EABT25190	7.40297	-1.52871	0.00022	0.00289
EABT21021	7.40297	-1.52871	0.00022	0.00289
EABT7237	7.40297	-1.52871	0.00022	0.00289
EABT13846	7.40297	-1.52871	0.00022	0.00289
EABT16811	7.40297	-1.52871	0.00022	0.00289
EABT9224	7.40297	-1.52871	0.00022	0.00289
EABT23737	7.40297	-1.52871	0.00022	0.00289
EABT21407	7.40297	-1.52871	0.00022	0.00289
EABT4425	7.40297	-1.52871	0.00022	0.00289
EABT5546	7.40297	-1.52871	0.00022	0.00289
EABT11417	7.40297	-1.52871	0.00022	0.00289
EABT21225	7.40297	-1.52871	0.00022	0.00289
EABT34741	7.40297	-1.52871	0.00022	0.00289
EABT18904	7.40297	-1.52871	0.00022	0.00289
EABT33648	7.40297	-1.52871	0.00022	0.00289
EABT31641	7.40297	-1.52871	0.00022	0.00289
EABT9067	7.40297	-1.52871	0.00022	0.00289
EABT9323	-7.3988	-1.530069	0.00022	0.00289
EABT4932	-7.3988	-1.530069	0.00022	0.00289
EABT4566	-7.3988	-1.530069	0.00022	0.00289
EABT5954	-7.3988	-1.530069	0.00022	0.00289
EABT22895	-7.3988	-1.530069	0.00022	0.00289
EABT31820	-2.5576	7.794382	0.00022	0.00291
EABT12524	2.55417	9.7056	0.00022	0.00293
EABT24210	3.54846	-0.364927	0.00022	0.00294
EABT25631	3.54846	-0.364927	0.00022	0.00294
EABT32866	3.54846	-0.364927	0.00022	0.00294
EABT930	3.54846	-0.364927	0.00022	0.00294
EABT29141	2.72136	1.982913	0.00022	0.00296
EABT15544	-2.567	5.381306	0.00022	0.00298
EABT11149	-2.8765	1.025201	0.00023	0.00298
EABT21843	3.40482	-0.194965	0.00023	0.00302
EABT20483	-3.3965	-0.196966	0.00023	0.00302
EABT24271	2.94056	0.805226	0.00023	0.00302
EABT26039	4.05104	-0.814837	0.00023	0.00304
EABT22355	4.05104	-0.814837	0.00023	0.00304
EABT5144	4.05104	-0.814837	0.00023	0.00304
EABT36448	4.05104	-0.814837	0.00023	0.00304
EABT28078	4.05104	-0.814837	0.00023	0.00304
EABT5337	4.05104	-0.814837	0.00023	0.00304
EABT30391	-4.0429	-0.816551	0.00023	0.00304
EABT15865	-4.0429	-0.816551	0.00023	0.00304
EABT36958	-2.9827	0.632947	0.00023	0.00304
EABT20046	-2.7104	1.939164	0.00023	0.00304
EABT16868	-2.5494	6.645651	0.00023	0.00304
EABT15552	-3.0139	0.539854	0.00023	0.00304
EABT37166	-3.0139	0.539854	0.00023	0.00304
EABT20054	-3.0504	0.440337	0.00023	0.00306
EABT36260	3.2184	0.094602	0.00023	0.00306
EABT21988	-2.6716	2.284824	0.00023	0.00306
EABT24675	-3.1459	0.217987	0.00023	0.00306
EABT20230	2.5597	5.035234	0.00023	0.00307
EABT13807	2.64291	2.673633	0.00023	0.00308

EABT2011	-2.7652	1.525906	0.00024	0.00308
EABT193	-2.6844	2.101533	0.00024	0.00314
EABT8685	-2.7875	1.37238	0.00024	0.00314
EABT6036	2.68087	2.19443	0.00024	0.00317
EABT9811	2.53724	8.264362	0.00024	0.00317
EABT20591	4.64782	-1.128074	0.00024	0.00317
EABT17134	4.64782	-1.128074	0.00024	0.00317
EABT534	4.64782	-1.128074	0.00024	0.00317
EABT23898	4.64782	-1.128074	0.00024	0.00317
EABT15709	4.64782	-1.128074	0.00024	0.00317
EABT22335	4.64782	-1.128074	0.00024	0.00317
EABT2885	4.64782	-1.128074	0.00024	0.00317
EABT13386	4.64782	-1.128074	0.00024	0.00317
EABT20109	4.64782	-1.128074	0.00024	0.00317
EABT37150	4.64782	-1.128074	0.00024	0.00317
EABT18469	4.64782	-1.128074	0.00024	0.00317
EABT6210	4.64782	-1.128074	0.00024	0.00317
EABT32976	4.64782	-1.128074	0.00024	0.00317
EABT5503	4.64782	-1.128074	0.00024	0.00317
EABT27891	-4.6399	-1.12963	0.00024	0.00317
EABT30020	-4.6399	-1.12963	0.00024	0.00317
EABT32449	-4.6399	-1.12963	0.00024	0.00317
EABT31055	-2.5779	3.756056	0.00025	0.00319
EABT4212	2.58614	3.651554	0.00025	0.00319
EABT36895	2.94993	0.710569	0.00025	0.0032
EABT36997	-2.5463	5.588081	0.00025	0.0032
EABT29350	2.54575	5.477029	0.00025	0.0032
EABT24740	-2.588	3.441879	0.00025	0.00321
EABT3529	2.5431	5.727203	0.00025	0.00321
EABT26199	3.7222	-0.587375	0.00025	0.00321
EABT28081	3.7222	-0.587375	0.00025	0.00321
EABT26128	3.7222	-0.587375	0.00025	0.00321
EABT4125	3.7222	-0.587375	0.00025	0.00321
EABT34450	3.7222	-0.587375	0.00025	0.00321
EABT35508	-2.5416	5.920349	0.00025	0.00321
EABT35710	3.00621	0.528334	0.00025	0.00324
EABT34747	-2.5859	3.427102	0.00025	0.00324
EABT417	-2.5729	3.772226	0.00025	0.00324
EABT19802	2.79515	1.311829	0.00025	0.00327
EABT25389	-2.5621	4.089754	0.00025	0.00328
EABT32299	-2.8688	0.934074	0.00025	0.00329
EABT15058	-2.5278	7.777016	0.00026	0.00329
EABT31478	2.80421	1.25427	0.00026	0.0033
EABT21679	3.51816	-0.390934	0.00026	0.0033
EABT16592	3.51816	-0.390934	0.00026	0.0033
EABT36255	3.51816	-0.390934	0.00026	0.0033
EABT83	-2.7204	1.689639	0.00026	0.0033
EABT10757	3.19642	0.075734	0.00026	0.00333
EABT1607	3.19642	0.075734	0.00026	0.00333
EABT29905	3.37792	-0.218059	0.00026	0.00333
EABT17936	3.37792	-0.218059	0.00026	0.00333
EABT21500	-3.3696	-0.220039	0.00026	0.00333
EABT27762	3.27515	-0.063698	0.00026	0.00334
EABT29607	3.27515	-0.063698	0.00026	0.00334

EABT30000	3.27515	-0.063698	0.00026	0.00334
EABT17903	2.96078	0.609264	0.00027	0.00343
EABT18170	-2.9524	0.606965	0.00027	0.00343
EABT22889	-2.5196	7.104602	0.00027	0.00344
EABT25397	2.71042	1.722874	0.00027	0.0035
EABT37242	2.59026	3.005571	0.00027	0.00352
EABT26629	2.55882	3.75596	0.00028	0.00354
EABT3351	2.58462	3.068946	0.00028	0.00355
EABT28650	4.00937	-0.850476	0.00028	0.00355
EABT34524	4.00937	-0.850476	0.00028	0.00355
EABT7812	4.00937	-0.850476	0.00028	0.00355
EABT31908	4.00937	-0.850476	0.00028	0.00355
EABT19961	4.00937	-0.850476	0.00028	0.00355
EABT30407	4.00937	-0.850476	0.00028	0.00355
EABT5420	4.00937	-0.850476	0.00028	0.00355
EABT18130	4.00937	-0.850476	0.00028	0.00355
EABT1884	4.00937	-0.850476	0.00028	0.00355
EABT20703	4.00937	-0.850476	0.00028	0.00355
EABT29005	4.00937	-0.850476	0.00028	0.00355
EABT27808	-4.0012	-0.852159	0.00028	0.00355
EABT7111	-4.0012	-0.852159	0.00028	0.00355
EABT15763	-2.8916	0.768095	0.00028	0.00355
EABT23836	-2.5325	4.415898	0.00028	0.0036
EABT19346	2.92112	0.685895	0.00028	0.00361
EABT198	3.11366	0.185376	0.00028	0.00361
EABT8286	2.5451	3.961328	0.00029	0.00363
EABT20751	-2.5036	7.872664	0.00029	0.00363
EABT31394	7.33301	-1.587626	0.00029	0.00363
EABT2858	7.33301	-1.587626	0.00029	0.00363
EABT2367	7.33301	-1.587626	0.00029	0.00363
EABT4082	7.33301	-1.587626	0.00029	0.00363
EABT6782	7.33301	-1.587626	0.00029	0.00363
EABT33197	7.33301	-1.587626	0.00029	0.00363
EABT4439	7.33301	-1.587626	0.00029	0.00363
EABT27725	7.33301	-1.587626	0.00029	0.00363
EABT37106	7.33301	-1.587626	0.00029	0.00363
EABT30396	7.33301	-1.587626	0.00029	0.00363
EABT18078	7.33301	-1.587626	0.00029	0.00363
EABT10680	7.33301	-1.587626	0.00029	0.00363
EABT33949	7.33301	-1.587626	0.00029	0.00363
EABT35776	7.33301	-1.587626	0.00029	0.00363
EABT15267	7.33301	-1.587626	0.00029	0.00363
EABT23145	7.33301	-1.587626	0.00029	0.00363
EABT27019	7.33301	-1.587626	0.00029	0.00363
EABT10599	7.33301	-1.587626	0.00029	0.00363
EABT2497	7.33301	-1.587626	0.00029	0.00363
EABT19028	7.33301	-1.587626	0.00029	0.00363
EABT11114	7.33301	-1.587626	0.00029	0.00363
EABT13017	7.33301	-1.587626	0.00029	0.00363
EABT23278	7.33301	-1.587626	0.00029	0.00363
EABT25987	7.33301	-1.587626	0.00029	0.00363
EABT17798	7.33301	-1.587626	0.00029	0.00363
EABT11641	7.33301	-1.587626	0.00029	0.00363
EABT37295	7.33301	-1.587626	0.00029	0.00363

EABT28625	7.33301	-1.587626	0.00029	0.00363
EABT13209	7.33301	-1.587626	0.00029	0.00363
EABT1834	7.33301	-1.587626	0.00029	0.00363
EABT13016	7.33301	-1.587626	0.00029	0.00363
EABT1497	7.33301	-1.587626	0.00029	0.00363
EABT19195	7.33301	-1.587626	0.00029	0.00363
EABT1457	7.33301	-1.587626	0.00029	0.00363
EABT15326	7.33301	-1.587626	0.00029	0.00363
EABT22360	7.33301	-1.587626	0.00029	0.00363
EABT19652	7.33301	-1.587626	0.00029	0.00363
EABT27347	7.33301	-1.587626	0.00029	0.00363
EABT30476	7.33301	-1.587626	0.00029	0.00363
EABT21632	7.33301	-1.587626	0.00029	0.00363
EABT15748	7.33301	-1.587626	0.00029	0.00363
EABT7837	7.33301	-1.587626	0.00029	0.00363
EABT35223	7.33301	-1.587626	0.00029	0.00363
EABT5031	7.33301	-1.587626	0.00029	0.00363
EABT2330	7.33301	-1.587626	0.00029	0.00363
EABT11690	7.33301	-1.587626	0.00029	0.00363
EABT24229	7.33301	-1.587626	0.00029	0.00363
EABT27011	7.33301	-1.587626	0.00029	0.00363
EABT11786	7.33301	-1.587626	0.00029	0.00363
EABT18505	-7.3288	-1.588941	0.00029	0.00363
EABT33261	-7.3288	-1.588941	0.00029	0.00363
EABT2788	-7.3288	-1.588941	0.00029	0.00363
EABT20906	-7.3288	-1.588941	0.00029	0.00363
EABT5657	-7.3288	-1.588941	0.00029	0.00363
EABT10755	3.68669	-0.617762	0.00029	0.00363
EABT8156	3.68669	-0.617762	0.00029	0.00363
EABT10308	3.68669	-0.617762	0.00029	0.00363
EABT25481	3.68669	-0.617762	0.00029	0.00363
EABT22801	-3.6785	-0.619551	0.00029	0.00363
EABT32263	3.25054	-0.08477	0.00029	0.00363
EABT17819	3.25054	-0.08477	0.00029	0.00363
EABT33975	-2.5297	4.266186	0.00029	0.00363
EABT8595	-2.5333	4.032337	0.00029	0.00364
EABT13018	-2.5093	5.816462	0.00029	0.00365
EABT25857	2.97346	0.500306	0.00029	0.00365
EABT12055	2.51393	5.089712	0.00029	0.00365
EABT16751	3.3505	-0.241529	0.00029	0.00365
EABT31477	3.3505	-0.241529	0.00029	0.00365
EABT1317	3.3505	-0.241529	0.00029	0.00365
EABT37901	-3.3422	-0.243487	0.00029	0.00365
EABT8974	-3.3422	-0.243487	0.00029	0.00365
EABT5777	2.81439	1.047137	0.00029	0.00365
EABT25003	3.48722	-0.417418	0.0003	0.00365
EABT3878	3.48722	-0.417418	0.0003	0.00365
EABT3607	3.48722	-0.417418	0.0003	0.00365
EABT2676	3.48722	-0.417418	0.0003	0.00365
EABT25027	3.48722	-0.417418	0.0003	0.00365
EABT10999	-3.4789	-0.419298	0.0003	0.00365
EABT27830	-2.649	2.000357	0.0003	0.00366
EABT24510	2.50531	6.049197	0.0003	0.00367
EABT1352	-2.8778	0.756324	0.0003	0.00368

EABT35132	2.52859	4.2397	0.0003	0.00369
EABT13814	-2.998	0.395504	0.0003	0.0037
EABT37973	-2.8181	0.9753	0.0003	0.0037
EABT35228	2.67382	1.822413	0.0003	0.00374
EABT15627	-2.7209	1.432823	0.0003	0.00375
EABT37908	2.49082	10.55536	0.0003	0.00375
EABT15588	3.04557	0.287271	0.00031	0.00375
EABT7135	4.59559	-1.172486	0.00031	0.00375
EABT9225	4.59559	-1.172486	0.00031	0.00375
EABT2668	4.59559	-1.172486	0.00031	0.00375
EABT38000	4.59559	-1.172486	0.00031	0.00375
EABT17981	4.59559	-1.172486	0.00031	0.00375
EABT10945	4.59559	-1.172486	0.00031	0.00375
EABT32074	4.59559	-1.172486	0.00031	0.00375
EABT15043	4.59559	-1.172486	0.00031	0.00375
EABT17438	4.59559	-1.172486	0.00031	0.00375
EABT7596	4.59559	-1.172486	0.00031	0.00375
EABT5130	4.59559	-1.172486	0.00031	0.00375
EABT13106	4.59559	-1.172486	0.00031	0.00375
EABT24542	-4.5877	-1.174005	0.00031	0.00375
EABT4884	-4.5877	-1.174005	0.00031	0.00375
EABT5072	2.50501	5.162476	0.00031	0.00376
EABT31017	-2.5019	5.533091	0.00031	0.00379
EABT8810	2.48798	8.625323	0.00031	0.00381
EABT20352	-3.0846	0.165544	0.00031	0.00383
EABT30638	-2.5203	4.021315	0.00031	0.00383
EABT20996	-2.5078	4.493317	0.00032	0.00388
EABT24899	-2.8062	0.965109	0.00032	0.00389
EABT15872	-2.4827	9.23786	0.00032	0.0039
EABT22685	-2.5911	2.440111	0.00032	0.0039
EABT7936	-2.5142	4.127965	0.00032	0.00391
EABT18000	2.82743	0.894029	0.00032	0.00397
EABT31331	2.62428	2.113484	0.00033	0.00398
EABT6968	2.89171	0.660792	0.00033	0.00398
EABT6947	3.2255	-0.106154	0.00033	0.004
EABT29903	-3.2172	-0.108159	0.00033	0.004
EABT19635	2.55884	2.888829	0.00033	0.00401
EABT23949	-2.493	5.007706	0.00033	0.00407
EABT7435	2.47402	7.856377	0.00033	0.00407
EABT1373	3.32256	-0.265387	0.00034	0.00407
EABT30930	3.32256	-0.265387	0.00034	0.00407
EABT1713	-3.3143	-0.267322	0.00034	0.00407
EABT37138	3.96646	-0.887018	0.00034	0.00407
EABT4858	3.96646	-0.887018	0.00034	0.00407
EABT5841	3.96646	-0.887018	0.00034	0.00407
EABT26280	3.96646	-0.887018	0.00034	0.00407
EABT16745	3.96646	-0.887018	0.00034	0.00407
EABT17615	3.96646	-0.887018	0.00034	0.00407
EABT1169	3.96646	-0.887018	0.00034	0.00407
EABT1238	3.96646	-0.887018	0.00034	0.00407
EABT23402	3.96646	-0.887018	0.00034	0.00407
EABT10155	3.96646	-0.887018	0.00034	0.00407
EABT26042	3.96646	-0.887018	0.00034	0.00407
EABT28324	3.96646	-0.887018	0.00034	0.00407

EABT25526	-3.9583	-0.888669	0.00034	0.00407
EABT13026	-3.9583	-0.888669	0.00034	0.00407
EABT25760	2.48132	5.553992	0.00034	0.00411
EABT898	-2.4724	7.022823	0.00034	0.00412
EABT3990	-2.4788	5.839675	0.00034	0.00412
EABT296	3.4556	-0.444398	0.00034	0.00412
EABT28729	3.4556	-0.444398	0.00034	0.00412
EABT4040	3.4556	-0.444398	0.00034	0.00412
EABT26944	3.4556	-0.444398	0.00034	0.00412
EABT35861	3.4556	-0.444398	0.00034	0.00412
EABT37797	3.4556	-0.444398	0.00034	0.00412
EABT14279	3.65028	-0.648803	0.00034	0.00414
EABT7604	3.65028	-0.648803	0.00034	0.00414
EABT3409	3.65028	-0.648803	0.00034	0.00414
EABT29749	3.65028	-0.648803	0.00034	0.00414
EABT19276	3.65028	-0.648803	0.00034	0.00414
EABT2718	3.65028	-0.648803	0.00034	0.00414
EABT36170	-3.642	-0.650564	0.00034	0.00414
EABT1704	2.93996	0.471723	0.00034	0.00415
EABT9833	-2.9316	0.469499	0.00034	0.00415
EABT27899	2.65768	1.722859	0.00034	0.00415
EABT30731	-2.487	4.584379	0.00035	0.00419
EABT37487	-2.7716	1.015381	0.00035	0.00419
EABT32288	-2.4664	7.563603	0.00035	0.00419
EABT9674	2.52754	3.264858	0.00035	0.00421
EABT5407	-2.4656	7.401548	0.00035	0.00421
EABT11746	-2.8684	0.645793	0.00035	0.00421
EABT32324	-2.8201	0.802869	0.00035	0.00426
EABT23378	3.12838	0.017603	0.00036	0.00428
EABT37595	-2.4834	4.484431	0.00036	0.00429
EABT32063	2.54428	2.819645	0.00036	0.00431
EABT20408	-2.5052	3.553273	0.00036	0.00434
EABT489	-2.5618	2.515402	0.00036	0.00435
EABT7768	-2.5649	2.417869	0.00036	0.00435
EABT23682	2.80193	0.872323	0.00037	0.0044
EABT29805	3.20002	-0.127859	0.00037	0.00442
EABT29158	-2.7094	1.243381	0.00037	0.00443
EABT25426	2.92291	0.457216	0.00037	0.00447
EABT12199	-2.4549	6.389221	0.00037	0.00448
EABT33952	-2.4691	5.020077	0.00038	0.0045
EABT15437	-2.5448	2.63918	0.00038	0.0045
EABT28858	-2.4708	4.603406	0.00038	0.0045
EABT29655	2.49134	3.825341	0.00038	0.0045
EABT4903	3.05058	0.131578	0.00038	0.0045
EABT20078	-3.0422	0.129493	0.00038	0.0045
EABT3589	3.29406	-0.289646	0.00038	0.0045
EABT14737	3.29406	-0.289646	0.00038	0.0045
EABT30771	4.5414	-1.218309	0.00039	0.0045
EABT15584	4.5414	-1.218309	0.00039	0.0045
EABT10748	4.5414	-1.218309	0.00039	0.0045
EABT34078	4.5414	-1.218309	0.00039	0.0045
EABT12774	4.5414	-1.218309	0.00039	0.0045
EABT30471	4.5414	-1.218309	0.00039	0.0045
EABT26891	4.5414	-1.218309	0.00039	0.0045

EABT25939	4.5414	-1.218309	0.00039	0.0045
EABT942	4.5414	-1.218309	0.00039	0.0045
EABT10172	4.5414	-1.218309	0.00039	0.0045
EABT3537	4.5414	-1.218309	0.00039	0.0045
EABT3412	4.5414	-1.218309	0.00039	0.0045
EABT6375	4.5414	-1.218309	0.00039	0.0045
EABT28061	4.5414	-1.218309	0.00039	0.0045
EABT3540	4.5414	-1.218309	0.00039	0.0045
EABT20654	4.5414	-1.218309	0.00039	0.0045
EABT28629	4.5414	-1.218309	0.00039	0.0045
EABT30909	4.5414	-1.218309	0.00039	0.0045
EABT4915	4.5414	-1.218309	0.00039	0.0045
EABT673	4.5414	-1.218309	0.00039	0.0045
EABT12386	4.5414	-1.218309	0.00039	0.0045
EABT11534	4.5414	-1.218309	0.00039	0.0045
EABT26154	4.5414	-1.218309	0.00039	0.0045
EABT32330	-4.5335	-1.21979	0.00039	0.0045
EABT27126	-4.5335	-1.21979	0.00039	0.0045
EABT27069	-4.5335	-1.21979	0.00039	0.0045
EABT3834	2.67296	1.442713	0.00039	0.0045
EABT9942	-2.6646	1.440217	0.00039	0.0045
EABT15184	-2.4448	7.911277	0.00039	0.0045
EABT4836	2.58067	2.172371	0.00039	0.0045
EABT32182	2.95204	0.3514	0.00039	0.0045
EABT7243	2.95204	0.3514	0.00039	0.0045
EABT15716	2.88203	0.542117	0.00039	0.0045
EABT35294	2.789	0.861346	0.00039	0.0045
EABT18929	7.25948	-1.64905	0.00039	0.0045
EABT1282	7.25948	-1.64905	0.00039	0.0045
EABT29282	7.25948	-1.64905	0.00039	0.0045
EABT34985	7.25948	-1.64905	0.00039	0.0045
EABT3393	7.25948	-1.64905	0.00039	0.0045
EABT26640	7.25948	-1.64905	0.00039	0.0045
EABT16715	7.25948	-1.64905	0.00039	0.0045
EABT14299	7.25948	-1.64905	0.00039	0.0045
EABT11614	7.25948	-1.64905	0.00039	0.0045
EABT14284	7.25948	-1.64905	0.00039	0.0045
EABT23119	7.25948	-1.64905	0.00039	0.0045
EABT23116	7.25948	-1.64905	0.00039	0.0045
EABT11524	7.25948	-1.64905	0.00039	0.0045
EABT14049	7.25948	-1.64905	0.00039	0.0045
EABT34344	7.25948	-1.64905	0.00039	0.0045
EABT19257	7.25948	-1.64905	0.00039	0.0045
EABT24687	7.25948	-1.64905	0.00039	0.0045
EABT9337	7.25948	-1.64905	0.00039	0.0045
EABT17099	7.25948	-1.64905	0.00039	0.0045
EABT26188	7.25948	-1.64905	0.00039	0.0045
EABT688	7.25948	-1.64905	0.00039	0.0045
EABT815	7.25948	-1.64905	0.00039	0.0045
EABT6385	7.25948	-1.64905	0.00039	0.0045
EABT34511	7.25948	-1.64905	0.00039	0.0045
EABT21680	7.25948	-1.64905	0.00039	0.0045
EABT37038	7.25948	-1.64905	0.00039	0.0045
EABT25750	7.25948	-1.64905	0.00039	0.0045

EABT19611	7.25948	-1.64905	0.00039	0.0045
EABT11291	7.25948	-1.64905	0.00039	0.0045
EABT6706	7.25948	-1.64905	0.00039	0.0045
EABT16307	7.25948	-1.64905	0.00039	0.0045
EABT6733	7.25948	-1.64905	0.00039	0.0045
EABT3903	7.25948	-1.64905	0.00039	0.0045
EABT8893	7.25948	-1.64905	0.00039	0.0045
EABT28199	7.25948	-1.64905	0.00039	0.0045
EABT4076	7.25948	-1.64905	0.00039	0.0045
EABT28831	7.25948	-1.64905	0.00039	0.0045
EABT4774	7.25948	-1.64905	0.00039	0.0045
EABT29707	7.25948	-1.64905	0.00039	0.0045
EABT22041	7.25948	-1.64905	0.00039	0.0045
EABT9527	7.25948	-1.64905	0.00039	0.0045
EABT37588	7.25948	-1.64905	0.00039	0.0045
EABT20280	7.25948	-1.64905	0.00039	0.0045
EABT12417	7.25948	-1.64905	0.00039	0.0045
EABT29254	7.25948	-1.64905	0.00039	0.0045
EABT20592	7.25948	-1.64905	0.00039	0.0045
EABT18634	7.25948	-1.64905	0.00039	0.0045
EABT34149	7.25948	-1.64905	0.00039	0.0045
EABT27528	7.25948	-1.64905	0.00039	0.0045
EABT20521	7.25948	-1.64905	0.00039	0.0045
EABT12985	7.25948	-1.64905	0.00039	0.0045
EABT19784	7.25948	-1.64905	0.00039	0.0045
EABT6648	7.25948	-1.64905	0.00039	0.0045
EABT11744	7.25948	-1.64905	0.00039	0.0045
EABT26505	7.25948	-1.64905	0.00039	0.0045
EABT30937	7.25948	-1.64905	0.00039	0.0045
EABT469	7.25948	-1.64905	0.00039	0.0045
EABT32304	7.25948	-1.64905	0.00039	0.0045
EABT11483	7.25948	-1.64905	0.00039	0.0045
EABT11103	7.25948	-1.64905	0.00039	0.0045
EABT8580	7.25948	-1.64905	0.00039	0.0045
EABT30308	7.25948	-1.64905	0.00039	0.0045
EABT15602	7.25948	-1.64905	0.00039	0.0045
EABT34827	7.25948	-1.64905	0.00039	0.0045
EABT11950	-7.2553	-1.650319	0.00039	0.0045
EABT30620	-7.2553	-1.650319	0.00039	0.0045
EABT24350	-7.2553	-1.650319	0.00039	0.0045
EABT16277	-7.2553	-1.650319	0.00039	0.0045
EABT23054	-7.2553	-1.650319	0.00039	0.0045
EABT1642	-7.2553	-1.650319	0.00039	0.0045
EABT20360	-7.2553	-1.650319	0.00039	0.0045
EABT32967	2.61966	1.776405	0.0004	0.00454
EABT12436	3.42327	-0.471891	0.0004	0.00454
EABT31429	3.42327	-0.471891	0.0004	0.00454
EABT21711	3.42327	-0.471891	0.0004	0.00454
EABT31148	3.42327	-0.471891	0.0004	0.00454
EABT2455	2.4533	5.198326	0.0004	0.00455
EABT25706	2.45012	5.13082	0.0004	0.00463
EABT7476	-2.9784	0.235086	0.0004	0.00464
EABT2059	-2.8973	0.440367	0.00041	0.00465
EABT18522	3.61293	-0.680526	0.00041	0.00465

EABT27444	3.61293	-0.680526	0.00041	0.00465
EABT29596	3.61293	-0.680526	0.00041	0.00465
EABT16155	3.61293	-0.680526	0.00041	0.00465
EABT15567	3.61293	-0.680526	0.00041	0.00465
EABT28716	3.61293	-0.680526	0.00041	0.00465
EABT27000	3.61293	-0.680526	0.00041	0.00465
EABT33626	3.92224	-0.924509	0.00041	0.00466
EABT31786	3.92224	-0.924509	0.00041	0.00466
EABT9896	3.92224	-0.924509	0.00041	0.00466
EABT3645	3.92224	-0.924509	0.00041	0.00466
EABT34129	3.92224	-0.924509	0.00041	0.00466
EABT3029	3.92224	-0.924509	0.00041	0.00466
EABT16098	3.92224	-0.924509	0.00041	0.00466
EABT27269	3.92224	-0.924509	0.00041	0.00466
EABT7979	3.92224	-0.924509	0.00041	0.00466
EABT2357	3.92224	-0.924509	0.00041	0.00466
EABT37812	3.92224	-0.924509	0.00041	0.00466
EABT22975	-3.9141	-0.926127	0.00041	0.00466
EABT36541	-3.9141	-0.926127	0.00041	0.00466
EABT9949	-2.4329	9.17016	0.00041	0.00466
EABT6411	2.45939	4.500597	0.00041	0.00468
EABT14144	-2.446	5.402752	0.00041	0.00468
EABT31802	-2.557	2.187382	0.00041	0.0047
EABT2282	-2.4809	3.474622	0.00041	0.0047
EABT583	-2.4307	7.982659	0.00042	0.00473
EABT37569	3.17408	-0.149896	0.00042	0.00473
EABT352	3.17408	-0.149896	0.00042	0.00473
EABT15236	-2.4443	4.924679	0.00042	0.00477
EABT3612	-2.8574	0.526084	0.00042	0.00479
EABT5574	-2.429	7.056987	0.00042	0.0048
EABT7963	3.02893	0.113191	0.00042	0.0048
EABT16390	-2.9251	0.333474	0.00042	0.00481
EABT19070	-2.4364	5.701456	0.00042	0.00482
EABT28883	2.45026	4.634721	0.00042	0.00482
EABT16492	-2.4262	7.929994	0.00042	0.00482
EABT9903	2.75321	0.915389	0.00043	0.00484
EABT14933	-2.4484	4.400881	0.00043	0.00486
EABT24140	-2.4718	3.513617	0.00043	0.00486
EABT31202	-2.4378	5.342996	0.00043	0.00487
EABT24828	-2.4632	3.729242	0.00043	0.00488
EABT28180	2.65806	1.374882	0.00043	0.00489
EABT4702	-2.5357	2.313272	0.00043	0.00489
EABT17750	3.26498	-0.31432	0.00044	0.00494
EABT29339	-2.4367	5.152883	0.00044	0.00494
EABT11026	2.8882	0.427758	0.00044	0.00498
EABT8158	-2.5984	1.677239	0.00044	0.00499
EABT23311	2.41903	7.434016	0.00044	0.00499
EABT36388	-2.5705	1.896931	0.00044	0.00501
EABT12843	2.43173	5.004346	0.00044	0.00501
EABT18401	2.96663	0.220124	0.00044	0.00501
EABT37884	-2.6525	1.317384	0.00044	0.00501
EABT1016	-2.6525	1.317384	0.00044	0.00501
EABT35410	-2.669	1.20925	0.00045	0.00507
EABT16960	2.84928	0.514359	0.00046	0.00514

EABT20468	-2.6084	1.546606	0.00046	0.00516
EABT18814	3.3902	-0.499919	0.00046	0.00516
EABT8170	3.3902	-0.499919	0.00046	0.00516
EABT5195	-3.3819	-0.501722	0.00046	0.00516
EABT23280	-3.3819	-0.501722	0.00046	0.00516
EABT21935	2.72061	0.96748	0.00046	0.00522
EABT34574	-2.4101	6.90097	0.00046	0.00523
EABT21948	2.41514	6.020472	0.00047	0.00523
EABT27304	3.00694	0.094566	0.00047	0.00526
EABT32339	2.81546	0.596054	0.00047	0.00527
EABT2914	-2.8071	0.593824	0.00047	0.00527
EABT7634	3.14767	-0.172275	0.00047	0.00527
EABT1131	3.14767	-0.172275	0.00047	0.00527
EABT25632	3.14767	-0.172275	0.00047	0.00527
EABT10817	-2.409	6.323927	0.00047	0.00529
EABT36248	-2.6313	1.356912	0.00047	0.00529
EABT10409	2.41664	5.146147	0.00048	0.00533
EABT35796	-2.412	5.668468	0.00048	0.00536
EABT35017	2.87052	0.4128	0.00048	0.00536
EABT24184	2.87052	0.4128	0.00048	0.00536
EABT17262	3.57458	-0.712963	0.00048	0.00537
EABT1993	3.57458	-0.712963	0.00048	0.00537
EABT18899	3.57458	-0.712963	0.00048	0.00537
EABT2399	3.57458	-0.712963	0.00048	0.00537
EABT10931	3.57458	-0.712963	0.00048	0.00537
EABT24977	3.57458	-0.712963	0.00048	0.00537
EABT23778	3.57458	-0.712963	0.00048	0.00537
EABT17268	-3.5663	-0.714665	0.00048	0.00537
EABT3715	-3.5663	-0.714665	0.00048	0.00537
EABT16267	-2.4068	6.200369	0.00048	0.00538
EABT10674	-2.7196	0.891684	0.00048	0.00538
EABT35567	2.41229	5.215647	0.00048	0.0054
EABT3837	2.55969	1.883316	0.00049	0.0054
EABT24478	4.48509	-1.265634	0.00049	0.0054
EABT11763	4.48509	-1.265634	0.00049	0.0054
EABT10136	4.48509	-1.265634	0.00049	0.0054
EABT19893	4.48509	-1.265634	0.00049	0.0054
EABT3792	4.48509	-1.265634	0.00049	0.0054
EABT35065	4.48509	-1.265634	0.00049	0.0054
EABT25437	4.48509	-1.265634	0.00049	0.0054
EABT30966	4.48509	-1.265634	0.00049	0.0054
EABT13515	4.48509	-1.265634	0.00049	0.0054
EABT6414	4.48509	-1.265634	0.00049	0.0054
EABT4972	4.48509	-1.265634	0.00049	0.0054
EABT19102	4.48509	-1.265634	0.00049	0.0054
EABT33666	4.48509	-1.265634	0.00049	0.0054
EABT27155	4.48509	-1.265634	0.00049	0.0054
EABT34325	4.48509	-1.265634	0.00049	0.0054
EABT6412	4.48509	-1.265634	0.00049	0.0054
EABT16499	4.48509	-1.265634	0.00049	0.0054
EABT421	4.48509	-1.265634	0.00049	0.0054
EABT3641	4.48509	-1.265634	0.00049	0.0054
EABT37031	4.48509	-1.265634	0.00049	0.0054
EABT27168	4.48509	-1.265634	0.00049	0.0054

EABT3464	4.48509	-1.265634	0.00049	0.0054
EABT6196	4.48509	-1.265634	0.00049	0.0054
EABT8536	-4.4772	-1.267077	0.00049	0.0054
EABT4223	-4.4772	-1.267077	0.00049	0.0054
EABT11337	-4.4772	-1.267077	0.00049	0.0054
EABT29864	-4.4772	-1.267077	0.00049	0.0054
EABT11118	2.39642	10.43184	0.00049	0.00541
EABT19424	-2.4606	3.110795	0.00049	0.00542
EABT29566	-2.4251	4.170763	0.00049	0.00543
EABT16154	2.42182	4.508517	0.00049	0.00544
EABT36821	2.83263	0.500276	0.00049	0.00544
EABT2171	2.45384	3.297765	0.00049	0.00546
EABT11259	2.60047	1.535356	0.0005	0.00546
EABT24235	-2.5921	1.532872	0.0005	0.00546
EABT9884	-2.422	4.230498	0.0005	0.00546
EABT12162	-2.6484	1.191877	0.0005	0.00546
EABT21870	3.87661	-0.963	0.0005	0.00546
EABT26812	3.87661	-0.963	0.0005	0.00546
EABT35459	3.87661	-0.963	0.0005	0.00546
EABT9084	3.87661	-0.963	0.0005	0.00546
EABT32180	3.87661	-0.963	0.0005	0.00546
EABT29548	3.87661	-0.963	0.0005	0.00546
EABT37954	3.87661	-0.963	0.0005	0.00546
EABT19121	3.87661	-0.963	0.0005	0.00546
EABT16509	3.87661	-0.963	0.0005	0.00546
EABT6566	3.87661	-0.963	0.0005	0.00546
EABT4021	3.87661	-0.963	0.0005	0.00546
EABT20241	3.87661	-0.963	0.0005	0.00546
EABT25275	3.87661	-0.963	0.0005	0.00546
EABT21546	-3.8685	-0.964585	0.0005	0.00546
EABT8789	-3.8685	-0.964585	0.0005	0.00546
EABT38054	-3.8685	-0.964585	0.0005	0.00546
EABT32484	3.23531	-0.339423	0.0005	0.00547
EABT31164	3.23531	-0.339423	0.0005	0.00547
EABT5030	3.23531	-0.339423	0.0005	0.00547
EABT10449	3.23531	-0.339423	0.0005	0.00547
EABT36270	-3.227	-0.341288	0.0005	0.00547
EABT6535	2.69078	1.017756	0.0005	0.00547
EABT37059	-2.8872	0.301428	0.00051	0.00553
EABT21110	-2.3923	6.179585	0.00052	0.00565
EABT4214	-2.3873	7.551761	0.00052	0.00565
EABT10082	2.77085	0.660765	0.00052	0.00565
EABT17502	2.77085	0.660765	0.00052	0.00565
EABT5880	2.98462	0.075698	0.00052	0.00565
EABT11727	-2.4618	2.811255	0.00053	0.00565
EABT7856	7.182	-1.713204	0.00053	0.00565
EABT24656	7.182	-1.713204	0.00053	0.00565
EABT17444	7.182	-1.713204	0.00053	0.00565
EABT1968	7.182	-1.713204	0.00053	0.00565
EABT10586	7.182	-1.713204	0.00053	0.00565
EABT33720	7.182	-1.713204	0.00053	0.00565
EABT19755	7.182	-1.713204	0.00053	0.00565
EABT37189	7.182	-1.713204	0.00053	0.00565
EABT28325	7.182	-1.713204	0.00053	0.00565

EABT30654	7.182	-1.713204	0.00053	0.00565
EABT15990	7.182	-1.713204	0.00053	0.00565
EABT21855	7.182	-1.713204	0.00053	0.00565
EABT26495	7.182	-1.713204	0.00053	0.00565
EABT28552	7.182	-1.713204	0.00053	0.00565
EABT28041	7.182	-1.713204	0.00053	0.00565
EABT21931	7.182	-1.713204	0.00053	0.00565
EABT25074	7.182	-1.713204	0.00053	0.00565
EABT6629	7.182	-1.713204	0.00053	0.00565
EABT5023	7.182	-1.713204	0.00053	0.00565
EABT18398	7.182	-1.713204	0.00053	0.00565
EABT27060	7.182	-1.713204	0.00053	0.00565
EABT25453	7.182	-1.713204	0.00053	0.00565
EABT9341	7.182	-1.713204	0.00053	0.00565
EABT28972	7.182	-1.713204	0.00053	0.00565
EABT3056	7.182	-1.713204	0.00053	0.00565
EABT25972	7.182	-1.713204	0.00053	0.00565
EABT19220	7.182	-1.713204	0.00053	0.00565
EABT10964	7.182	-1.713204	0.00053	0.00565
EABT15820	7.182	-1.713204	0.00053	0.00565
EABT30600	7.182	-1.713204	0.00053	0.00565
EABT6810	7.182	-1.713204	0.00053	0.00565
EABT27312	7.182	-1.713204	0.00053	0.00565
EABT37747	7.182	-1.713204	0.00053	0.00565
EABT31670	7.182	-1.713204	0.00053	0.00565
EABT37621	7.182	-1.713204	0.00053	0.00565
EABT28021	7.182	-1.713204	0.00053	0.00565
EABT11184	7.182	-1.713204	0.00053	0.00565
EABT35437	7.182	-1.713204	0.00053	0.00565
EABT7146	7.182	-1.713204	0.00053	0.00565
EABT20073	7.182	-1.713204	0.00053	0.00565
EABT2925	7.182	-1.713204	0.00053	0.00565
EABT9615	7.182	-1.713204	0.00053	0.00565
EABT22546	7.182	-1.713204	0.00053	0.00565
EABT31368	7.182	-1.713204	0.00053	0.00565
EABT5790	7.182	-1.713204	0.00053	0.00565
EABT20006	7.182	-1.713204	0.00053	0.00565
EABT26156	7.182	-1.713204	0.00053	0.00565
EABT2131	7.182	-1.713204	0.00053	0.00565
EABT13330	7.182	-1.713204	0.00053	0.00565
EABT31324	7.182	-1.713204	0.00053	0.00565
EABT6842	7.182	-1.713204	0.00053	0.00565
EABT25743	7.182	-1.713204	0.00053	0.00565
EABT35666	7.182	-1.713204	0.00053	0.00565
EABT31027	7.182	-1.713204	0.00053	0.00565
EABT19367	7.182	-1.713204	0.00053	0.00565
EABT25756	7.182	-1.713204	0.00053	0.00565
EABT25590	7.182	-1.713204	0.00053	0.00565
EABT28982	7.182	-1.713204	0.00053	0.00565
EABT31870	7.182	-1.713204	0.00053	0.00565
EABT6486	7.182	-1.713204	0.00053	0.00565
EABT26007	7.182	-1.713204	0.00053	0.00565
EABT37949	7.182	-1.713204	0.00053	0.00565
EABT29359	7.182	-1.713204	0.00053	0.00565

EABT392	7.182	-1.713204	0.00053	0.00565
EABT34669	7.182	-1.713204	0.00053	0.00565
EABT16535	7.182	-1.713204	0.00053	0.00565
EABT4455	7.182	-1.713204	0.00053	0.00565
EABT34357	7.182	-1.713204	0.00053	0.00565
EABT3839	7.182	-1.713204	0.00053	0.00565
EABT4654	7.182	-1.713204	0.00053	0.00565
EABT28071	7.182	-1.713204	0.00053	0.00565
EABT21898	7.182	-1.713204	0.00053	0.00565
EABT16808	-7.1778	-1.714425	0.00053	0.00565
EABT31144	-7.1778	-1.714425	0.00053	0.00565
EABT4827	-7.1778	-1.714425	0.00053	0.00565
EABT5046	-7.1778	-1.714425	0.00053	0.00565
EABT13601	-7.1778	-1.714425	0.00053	0.00565
EABT8835	-7.1778	-1.714425	0.00053	0.00565
EABT13454	-7.1778	-1.714425	0.00053	0.00565
EABT25825	-7.1778	-1.714425	0.00053	0.00565
EABT9082	-7.1778	-1.714425	0.00053	0.00565
EABT24034	3.12077	-0.195006	0.00053	0.00565
EABT29808	3.12077	-0.195006	0.00053	0.00565
EABT13220	-2.4307	3.40847	0.00053	0.00566
EABT24294	3.35635	-0.528501	0.00053	0.00567
EABT36012	-3.3481	-0.530278	0.00053	0.00567
EABT3662	-3.3481	-0.530278	0.00053	0.00567
EABT31663	2.92549	0.185341	0.00054	0.00571
EABT27642	2.7022	0.872299	0.00054	0.00579
EABT17090	2.78379	0.569324	0.00054	0.00579
EABT11842	-2.5305	1.82549	0.00055	0.00583
EABT3808	-2.5841	1.425445	0.00055	0.00583
EABT36847	-2.3887	5.206891	0.00055	0.00583
EABT11736	-2.3744	7.503275	0.00055	0.00584
EABT2083	-2.3875	5.258933	0.00055	0.00584
EABT21755	-2.4024	4.056591	0.00055	0.00585
EABT15116	2.68382	0.936437	0.00055	0.00585
EABT19921	2.40984	3.972795	0.00055	0.00585
EABT7789	2.87622	0.287238	0.00055	0.00586
EABT8907	2.43005	3.303848	0.00055	0.00586
EABT24778	3.03236	-0.063737	0.00056	0.00588
EABT23969	3.03236	-0.063737	0.00056	0.00588
EABT36249	-2.6588	0.995514	0.00056	0.00592
EABT1740	2.37034	8.093348	0.00056	0.00592
EABT494	-2.8261	0.380276	0.00057	0.00599
EABT25224	2.36572	10.26625	0.00057	0.00601
EABT30425	-2.3865	4.537889	0.00057	0.00603
EABT34021	-2.3925	4.232636	0.00057	0.00603
EABT29963	3.53518	-0.746145	0.00057	0.00603
EABT17243	3.53518	-0.746145	0.00057	0.00603
EABT16419	3.53518	-0.746145	0.00057	0.00603
EABT11070	3.53518	-0.746145	0.00057	0.00603
EABT4363	-3.527	-0.747817	0.00057	0.00603
EABT5847	-3.527	-0.747817	0.00057	0.00603
EABT18145	3.20502	-0.36497	0.00057	0.00603
EABT10694	3.20502	-0.36497	0.00057	0.00603
EABT12614	2.96194	0.056579	0.00058	0.00607

EABT10340	2.38398	4.809004	0.00058	0.0061
EABT32997	2.90446	0.16763	0.00059	0.00623
EABT31150	-2.5031	1.91295	0.00059	0.00625
EABT12106	-2.3627	6.261277	0.0006	0.00627
EABT34016	-2.427	2.923449	0.0006	0.00632
EABT18933	3.09335	-0.218101	0.0006	0.00632
EABT10955	-2.3714	4.775918	0.0006	0.00634
EABT15688	2.85662	0.270742	0.00061	0.00636
EABT36233	3.8295	-1.002546	0.00061	0.00636
EABT1367	3.8295	-1.002546	0.00061	0.00636
EABT5676	3.8295	-1.002546	0.00061	0.00636
EABT9121	3.8295	-1.002546	0.00061	0.00636
EABT20779	3.8295	-1.002546	0.00061	0.00636
EABT18703	3.8295	-1.002546	0.00061	0.00636
EABT9377	3.8295	-1.002546	0.00061	0.00636
EABT20366	3.8295	-1.002546	0.00061	0.00636
EABT15993	3.8295	-1.002546	0.00061	0.00636
EABT591	3.8295	-1.002546	0.00061	0.00636
EABT33093	3.8295	-1.002546	0.00061	0.00636
EABT17185	3.8295	-1.002546	0.00061	0.00636
EABT17041	3.8295	-1.002546	0.00061	0.00636
EABT8144	3.8295	-1.002546	0.00061	0.00636
EABT18312	3.8295	-1.002546	0.00061	0.00636
EABT34356	-3.8214	-1.004096	0.00061	0.00636
EABT16539	-3.8214	-1.004096	0.00061	0.00636
EABT38143	-3.8214	-1.004096	0.00061	0.00636
EABT8026	-3.8214	-1.004096	0.00061	0.00636
EABT30513	2.42269	2.965082	0.00061	0.0064
EABT27567	-2.3658	5.111338	0.00062	0.00643
EABT2797	2.36282	5.061167	0.00062	0.00643
EABT29099	2.41314	3.111186	0.00062	0.00643
EABT29130	-2.3655	5.05658	0.00062	0.00643
EABT25791	4.42649	-1.314564	0.00062	0.00643
EABT28797	4.42649	-1.314564	0.00062	0.00643
EABT13139	4.42649	-1.314564	0.00062	0.00643
EABT30196	4.42649	-1.314564	0.00062	0.00643
EABT3570	4.42649	-1.314564	0.00062	0.00643
EABT34441	4.42649	-1.314564	0.00062	0.00643
EABT13864	4.42649	-1.314564	0.00062	0.00643
EABT23848	4.42649	-1.314564	0.00062	0.00643
EABT14328	4.42649	-1.314564	0.00062	0.00643
EABT37823	4.42649	-1.314564	0.00062	0.00643
EABT37143	4.42649	-1.314564	0.00062	0.00643
EABT21847	4.42649	-1.314564	0.00062	0.00643
EABT34655	4.42649	-1.314564	0.00062	0.00643
EABT21558	4.42649	-1.314564	0.00062	0.00643
EABT12148	4.42649	-1.314564	0.00062	0.00643
EABT20845	4.42649	-1.314564	0.00062	0.00643
EABT33367	4.42649	-1.314564	0.00062	0.00643
EABT37915	4.42649	-1.314564	0.00062	0.00643
EABT5324	4.42649	-1.314564	0.00062	0.00643
EABT26127	4.42649	-1.314564	0.00062	0.00643
EABT17849	4.42649	-1.314564	0.00062	0.00643
EABT13520	4.42649	-1.314564	0.00062	0.00643

EABT8825	4.42649	-1.314564	0.00062	0.00643
EABT29255	-4.4186	-1.315966	0.00062	0.00643
EABT14084	-4.4186	-1.315966	0.00062	0.00643
EABT833	3.32169	-0.557661	0.00062	0.00643
EABT36762	3.32169	-0.557661	0.00062	0.00643
EABT21622	3.32169	-0.557661	0.00062	0.00643
EABT21874	3.32169	-0.557661	0.00062	0.00643
EABT9894	3.32169	-0.557661	0.00062	0.00643
EABT15365	3.32169	-0.557661	0.00062	0.00643
EABT7693	-3.3134	-0.55941	0.00062	0.00643
EABT35698	-3.3134	-0.55941	0.00062	0.00643
EABT20564	2.42649	2.813924	0.00062	0.00644
EABT9156	-2.3519	6.076834	0.00063	0.00652
EABT24616	2.54355	1.535339	0.00063	0.00653
EABT11341	-2.3509	6.122268	0.00063	0.00654
EABT36812	-2.4178	2.864344	0.00064	0.00656
EABT25235	2.53093	1.615891	0.00064	0.00657
EABT34082	2.35695	4.992422	0.00064	0.00659
EABT12426	2.35366	5.383696	0.00064	0.00659
EABT36915	2.38389	3.780861	0.00064	0.00659
EABT8113	-2.3494	5.922069	0.00064	0.00662
EABT34195	2.51453	1.728866	0.00064	0.00662
EABT11656	-2.3849	3.518885	0.00065	0.00666
EABT30131	-2.3479	5.57439	0.00066	0.00676
EABT27965	3.17407	-0.390978	0.00066	0.00676
EABT26342	2.46069	2.189982	0.00066	0.0068
EABT28224	2.4047	2.998012	0.00066	0.0068
EABT20586	-2.8284	0.251984	0.00066	0.00682
EABT33129	2.41389	2.785177	0.00067	0.00684
EABT16993	-2.4124	2.788328	0.00067	0.00684
EABT29071	2.46612	2.075954	0.00068	0.00694
EABT28331	-2.3345	6.464244	0.00068	0.00696
EABT15118	-3.0571	-0.243445	0.00068	0.00698
EABT28913	3.49469	-0.780107	0.00068	0.00698
EABT33987	3.49469	-0.780107	0.00068	0.00698
EABT30675	3.49469	-0.780107	0.00068	0.00698
EABT23466	3.49469	-0.780107	0.00068	0.00698
EABT15170	3.49469	-0.780107	0.00068	0.00698
EABT37311	-3.4865	-0.781749	0.00068	0.00698
EABT18017	-2.5015	1.639306	0.00069	0.00704
EABT27732	-2.3902	3.051834	0.00069	0.00708
EABT3716	-2.4467	2.124689	0.00069	0.00711
EABT12302	-2.6588	0.756376	0.0007	0.00712
EABT32988	2.98185	-0.106193	0.0007	0.00715
EABT18662	2.98185	-0.106193	0.0007	0.00715
EABT7643	-2.9735	-0.108119	0.0007	0.00715
EABT13571	2.32541	6.861401	0.0007	0.0072
EABT26216	2.59342	1.075844	0.00071	0.00721
EABT7055	-2.5376	1.333356	0.00071	0.00721
EABT7769	2.53841	1.382543	0.00071	0.00721
EABT19166	2.91549	0.017566	0.00071	0.00721
EABT35116	-2.9072	0.015593	0.00071	0.00721
EABT9318	-2.3363	5.100913	0.00071	0.00721
EABT11629	-2.8531	0.129528	0.00072	0.00721

EABT10139	7.10012	-1.780343	0.00073	0.00721
EABT31077	7.10012	-1.780343	0.00073	0.00721
EABT1796	7.10012	-1.780343	0.00073	0.00721
EABT7823	7.10012	-1.780343	0.00073	0.00721
EABT30648	7.10012	-1.780343	0.00073	0.00721
EABT31507	7.10012	-1.780343	0.00073	0.00721
EABT9661	7.10012	-1.780343	0.00073	0.00721
EABT36167	7.10012	-1.780343	0.00073	0.00721
EABT15221	7.10012	-1.780343	0.00073	0.00721
EABT25542	7.10012	-1.780343	0.00073	0.00721
EABT8467	7.10012	-1.780343	0.00073	0.00721
EABT29052	7.10012	-1.780343	0.00073	0.00721
EABT37850	7.10012	-1.780343	0.00073	0.00721
EABT14575	7.10012	-1.780343	0.00073	0.00721
EABT25960	7.10012	-1.780343	0.00073	0.00721
EABT13677	7.10012	-1.780343	0.00073	0.00721
EABT12482	7.10012	-1.780343	0.00073	0.00721
EABT32055	7.10012	-1.780343	0.00073	0.00721
EABT37941	7.10012	-1.780343	0.00073	0.00721
EABT36479	7.10012	-1.780343	0.00073	0.00721
EABT37051	7.10012	-1.780343	0.00073	0.00721
EABT34848	7.10012	-1.780343	0.00073	0.00721
EABT21289	7.10012	-1.780343	0.00073	0.00721
EABT15271	7.10012	-1.780343	0.00073	0.00721
EABT7852	7.10012	-1.780343	0.00073	0.00721
EABT33896	7.10012	-1.780343	0.00073	0.00721
EABT20306	7.10012	-1.780343	0.00073	0.00721
EABT14395	7.10012	-1.780343	0.00073	0.00721
EABT26421	7.10012	-1.780343	0.00073	0.00721
EABT27268	7.10012	-1.780343	0.00073	0.00721
EABT18675	7.10012	-1.780343	0.00073	0.00721
EABT21578	7.10012	-1.780343	0.00073	0.00721
EABT27739	7.10012	-1.780343	0.00073	0.00721
EABT31424	7.10012	-1.780343	0.00073	0.00721
EABT10612	7.10012	-1.780343	0.00073	0.00721
EABT9865	7.10012	-1.780343	0.00073	0.00721
EABT12076	7.10012	-1.780343	0.00073	0.00721
EABT2790	7.10012	-1.780343	0.00073	0.00721
EABT24413	7.10012	-1.780343	0.00073	0.00721
EABT8706	7.10012	-1.780343	0.00073	0.00721
EABT30612	7.10012	-1.780343	0.00073	0.00721
EABT6457	7.10012	-1.780343	0.00073	0.00721
EABT34183	7.10012	-1.780343	0.00073	0.00721
EABT28856	7.10012	-1.780343	0.00073	0.00721
EABT5725	7.10012	-1.780343	0.00073	0.00721
EABT1392	7.10012	-1.780343	0.00073	0.00721
EABT37075	7.10012	-1.780343	0.00073	0.00721
EABT12319	7.10012	-1.780343	0.00073	0.00721
EABT7977	7.10012	-1.780343	0.00073	0.00721
EABT32233	7.10012	-1.780343	0.00073	0.00721
EABT29131	7.10012	-1.780343	0.00073	0.00721
EABT16017	7.10012	-1.780343	0.00073	0.00721
EABT28218	7.10012	-1.780343	0.00073	0.00721
EABT7517	7.10012	-1.780343	0.00073	0.00721

EABT27267	7.10012	-1.780343	0.00073	0.00721
EABT713	7.10012	-1.780343	0.00073	0.00721
EABT29791	7.10012	-1.780343	0.00073	0.00721
EABT32424	7.10012	-1.780343	0.00073	0.00721
EABT37307	7.10012	-1.780343	0.00073	0.00721
EABT27787	7.10012	-1.780343	0.00073	0.00721
EABT410	7.10012	-1.780343	0.00073	0.00721
EABT12090	7.10012	-1.780343	0.00073	0.00721
EABT28374	7.10012	-1.780343	0.00073	0.00721
EABT5240	7.10012	-1.780343	0.00073	0.00721
EABT33961	7.10012	-1.780343	0.00073	0.00721
EABT9058	7.10012	-1.780343	0.00073	0.00721
EABT14512	7.10012	-1.780343	0.00073	0.00721
EABT26311	7.10012	-1.780343	0.00073	0.00721
EABT25975	7.10012	-1.780343	0.00073	0.00721
EABT37592	7.10012	-1.780343	0.00073	0.00721
EABT36622	7.10012	-1.780343	0.00073	0.00721
EABT23562	7.10012	-1.780343	0.00073	0.00721
EABT23986	7.10012	-1.780343	0.00073	0.00721
EABT31636	7.10012	-1.780343	0.00073	0.00721
EABT10969	7.10012	-1.780343	0.00073	0.00721
EABT31856	7.10012	-1.780343	0.00073	0.00721
EABT8472	7.10012	-1.780343	0.00073	0.00721
EABT26446	7.10012	-1.780343	0.00073	0.00721
EABT18365	7.10012	-1.780343	0.00073	0.00721
EABT32409	7.10012	-1.780343	0.00073	0.00721
EABT6541	7.10012	-1.780343	0.00073	0.00721
EABT6984	7.10012	-1.780343	0.00073	0.00721
EABT21276	7.10012	-1.780343	0.00073	0.00721
EABT25151	7.10012	-1.780343	0.00073	0.00721
EABT11475	7.10012	-1.780343	0.00073	0.00721
EABT24371	-7.0959	-1.781515	0.00073	0.00721
EABT16625	-7.0959	-1.781515	0.00073	0.00721
EABT22809	-7.0959	-1.781515	0.00073	0.00721
EABT20716	-7.0959	-1.781515	0.00073	0.00721
EABT4681	-7.0959	-1.781515	0.00073	0.00721
EABT3092	-7.0959	-1.781515	0.00073	0.00721
EABT19920	-7.0959	-1.781515	0.00073	0.00721
EABT2619	3.28617	-0.587423	0.00073	0.00721
EABT2193	3.28617	-0.587423	0.00073	0.00721
EABT24528	3.28617	-0.587423	0.00073	0.00721
EABT13620	3.28617	-0.587423	0.00073	0.00721
EABT4465	-2.3406	4.209093	0.00073	0.00729
EABT16414	-2.4581	1.875355	0.00074	0.0073
EABT19109	-2.3275	5.256869	0.00074	0.0073
EABT8920	-2.3173	6.418054	0.00074	0.0073
EABT10429	2.7183	0.514329	0.00074	0.00732
EABT22594	2.7183	0.514329	0.00074	0.00732
EABT8476	2.31341	8.026287	0.00074	0.00732
EABT21902	-2.3157	6.650321	0.00074	0.00732
EABT19376	-2.598	0.944556	0.00074	0.00734
EABT28367	2.59363	1.007812	0.00074	0.00734
EABT30115	3.7808	-1.043206	0.00075	0.00734
EABT6011	3.7808	-1.043206	0.00075	0.00734

EABT27531	3.7808	-1.043206	0.00075	0.00734
EABT14566	3.7808	-1.043206	0.00075	0.00734
EABT7382	3.7808	-1.043206	0.00075	0.00734
EABT32202	3.7808	-1.043206	0.00075	0.00734
EABT21213	3.7808	-1.043206	0.00075	0.00734
EABT22932	3.7808	-1.043206	0.00075	0.00734
EABT2745	3.7808	-1.043206	0.00075	0.00734
EABT30146	3.7808	-1.043206	0.00075	0.00734
EABT19203	3.7808	-1.043206	0.00075	0.00734
EABT37416	3.7808	-1.043206	0.00075	0.00734
EABT3956	3.7808	-1.043206	0.00075	0.00734
EABT34677	3.7808	-1.043206	0.00075	0.00734
EABT4098	3.7808	-1.043206	0.00075	0.00734
EABT2501	3.7808	-1.043206	0.00075	0.00734
EABT21567	3.7808	-1.043206	0.00075	0.00734
EABT25864	3.7808	-1.043206	0.00075	0.00734
EABT7748	3.7808	-1.043206	0.00075	0.00734
EABT16804	3.7808	-1.043206	0.00075	0.00734
EABT36688	-3.7726	-1.04472	0.00075	0.00734
EABT6261	-3.7726	-1.04472	0.00075	0.00734
EABT3255	-2.3681	3.163045	0.00075	0.00735
EABT28474	-2.3214	5.392852	0.00075	0.0074
EABT23319	2.44572	2.027687	0.00075	0.00741
EABT19491	3.14245	-0.417463	0.00076	0.00741
EABT19584	3.14245	-0.417463	0.00076	0.00741
EABT29784	3.14245	-0.417463	0.00076	0.00741
EABT21371	3.14245	-0.417463	0.00076	0.00741
EABT31375	3.14245	-0.417463	0.00076	0.00741
EABT34413	-3.1342	-0.419253	0.00076	0.00741
EABT21190	-3.1342	-0.419253	0.00076	0.00741
EABT4549	-2.3476	3.651955	0.00076	0.00741
EABT36969	-2.3227	5.191696	0.00076	0.00741
EABT13051	-2.3321	4.272473	0.00076	0.00744
EABT8445	-2.388	2.692727	0.00076	0.00749
EABT36522	2.32485	4.811834	0.00077	0.00754
EABT23134	-2.4205	2.160876	0.00077	0.00754
EABT3175	3.03691	-0.265429	0.00077	0.00756
EABT3473	-3.0286	-0.26728	0.00077	0.00756
EABT30336	-3.0286	-0.26728	0.00077	0.00756
EABT8198	-2.3093	6.066458	0.00078	0.00759
EABT13285	2.5343	1.270878	0.00078	0.00761
EABT15980	2.8917	-0.002344	0.00079	0.0077
EABT13597	-2.3183	4.507506	0.00079	0.0077
EABT2454	-2.6302	0.732541	0.00079	0.0077
EABT30109	2.65708	0.660738	0.0008	0.0077
EABT20076	2.65708	0.660738	0.0008	0.0077
EABT14581	-2.8311	0.111159	0.0008	0.0077
EABT28860	-2.3165	4.892143	0.0008	0.0077
EABT31446	4.36542	-1.365211	0.0008	0.0077
EABT21194	4.36542	-1.365211	0.0008	0.0077
EABT24517	4.36542	-1.365211	0.0008	0.0077
EABT7666	4.36542	-1.365211	0.0008	0.0077
EABT19177	4.36542	-1.365211	0.0008	0.0077
EABT31461	4.36542	-1.365211	0.0008	0.0077

EABT35844	4.36542	-1.365211	0.0008	0.0077
EABT18946	4.36542	-1.365211	0.0008	0.0077
EABT19991	4.36542	-1.365211	0.0008	0.0077
EABT22730	4.36542	-1.365211	0.0008	0.0077
EABT30998	4.36542	-1.365211	0.0008	0.0077
EABT20926	4.36542	-1.365211	0.0008	0.0077
EABT13668	4.36542	-1.365211	0.0008	0.0077
EABT14337	4.36542	-1.365211	0.0008	0.0077
EABT2648	4.36542	-1.365211	0.0008	0.0077
EABT8958	4.36542	-1.365211	0.0008	0.0077
EABT27157	4.36542	-1.365211	0.0008	0.0077
EABT7644	4.36542	-1.365211	0.0008	0.0077
EABT22073	4.36542	-1.365211	0.0008	0.0077
EABT23634	4.36542	-1.365211	0.0008	0.0077
EABT35609	4.36542	-1.365211	0.0008	0.0077
EABT21719	4.36542	-1.365211	0.0008	0.0077
EABT1960	4.36542	-1.365211	0.0008	0.0077
EABT25752	4.36542	-1.365211	0.0008	0.0077
EABT16758	4.36542	-1.365211	0.0008	0.0077
EABT5897	4.36542	-1.365211	0.0008	0.0077
EABT3218	4.36542	-1.365211	0.0008	0.0077
EABT4471	4.36542	-1.365211	0.0008	0.0077
EABT4368	4.36542	-1.365211	0.0008	0.0077
EABT35598	4.36542	-1.365211	0.0008	0.0077
EABT24472	-4.3575	-1.366572	0.0008	0.0077
EABT2764	-4.3575	-1.366572	0.0008	0.0077
EABT540	-4.3575	-1.366572	0.0008	0.0077
EABT36344	-4.3575	-1.366572	0.0008	0.0077
EABT11755	-2.3126	5.126303	0.0008	0.0077
EABT17257	2.79618	0.22009	0.0008	0.00771
EABT7051	-2.6931	0.498114	0.0008	0.00771
EABT17022	-2.7201	0.410666	0.0008	0.00771
EABT17231	-2.7513	0.317572	0.0008	0.00771
EABT6160	-2.7513	0.317572	0.0008	0.00771
EABT16054	-2.2951	8.365432	0.00081	0.00777
EABT10651	-2.3022	5.771915	0.00081	0.00783
EABT10552	-2.3081	5.173001	0.00081	0.00783
EABT13471	-2.4115	2.124701	0.00081	0.00783
EABT16011	3.45302	-0.814889	0.00081	0.00783
EABT14798	3.45302	-0.814889	0.00081	0.00783
EABT30956	3.45302	-0.814889	0.00081	0.00783
EABT12775	3.45302	-0.814889	0.00081	0.00783
EABT34667	3.45302	-0.814889	0.00081	0.00783
EABT10396	3.45302	-0.814889	0.00081	0.00783
EABT36616	3.45302	-0.814889	0.00081	0.00783
EABT11339	3.45302	-0.814889	0.00081	0.00783
EABT38011	3.45302	-0.814889	0.00081	0.00783
EABT30557	-3.4448	-0.816499	0.00081	0.00783
EABT20891	-3.4448	-0.816499	0.00081	0.00783
EABT27641	-2.3406	3.333219	0.00082	0.0079
EABT33516	2.56961	0.987764	0.00083	0.00799
EABT29665	2.44015	1.855898	0.00083	0.008
EABT8803	-2.3111	4.316248	0.00084	0.00802
EABT11919	2.35117	3.044816	0.00084	0.00805

EABT1901	-2.2912	6.08659	0.00085	0.00811
EABT19482	2.45224	1.716721	0.00085	0.00811
EABT16489	-2.3738	2.560174	0.00085	0.00812
EABT14867	3.24976	-0.617811	0.00085	0.00815
EABT15844	3.24976	-0.617811	0.00085	0.00815
EABT29188	3.24976	-0.617811	0.00085	0.00815
EABT34301	3.24976	-0.617811	0.00085	0.00815
EABT20444	-3.2415	-0.619503	0.00085	0.00815
EABT31034	-2.4226	1.8808	0.00086	0.0082
EABT21989	-2.2854	6.277876	0.00086	0.00824
EABT2019	-2.2887	5.853285	0.00086	0.00825
EABT37197	3.11012	-0.444443	0.00087	0.00832
EABT17132	3.11012	-0.444443	0.00087	0.00832
EABT21444	-2.4334	1.744395	0.00087	0.00833
EABT17611	-2.3444	2.95215	0.00087	0.00835
EABT28571	-2.2849	5.831707	0.00088	0.00839
EABT26860	3.00783	-0.289689	0.00088	0.00839
EABT22797	-2.9995	-0.291516	0.00088	0.00839
EABT12047	-2.9995	-0.291516	0.00088	0.00839
EABT27655	-2.9995	-0.291516	0.00088	0.00839
EABT32742	-2.9995	-0.291516	0.00088	0.00839
EABT25142	2.81716	0.09453	0.00088	0.00839
EABT8463	-2.8088	0.092552	0.00088	0.00839
EABT17864	2.8675	-0.022532	0.00088	0.0084
EABT8600	2.9295	-0.149936	0.00088	0.0084
EABT29680	2.9295	-0.149936	0.00088	0.0084
EABT31865	2.9295	-0.149936	0.00088	0.0084
EABT20624	2.9295	-0.149936	0.00088	0.0084
EABT22326	-2.9212	-0.151819	0.00088	0.0084
EABT27286	-2.3029	4.082795	0.00088	0.00841
EABT31440	2.56858	0.915341	0.00089	0.00844
EABT36451	2.2746	7.690382	0.00089	0.00849
EABT1240	-2.3866	2.160888	0.00089	0.0085
EABT10752	2.50416	1.245774	0.0009	0.00852
EABT25192	2.29733	4.459285	0.0009	0.00852
EABT2505	2.39635	2.136349	0.0009	0.00853
EABT21326	2.59441	0.782054	0.0009	0.00855
EABT4806	-2.2726	6.91367	0.00091	0.00862
EABT24084	-2.2927	4.288929	0.00091	0.00867
EABT13086	-2.2688	7.903861	0.00092	0.0087
EABT32693	-2.2788	5.448812	0.00092	0.00871
EABT22031	3.73039	-1.085044	0.00092	0.00871
EABT21600	3.73039	-1.085044	0.00092	0.00871
EABT4304	3.73039	-1.085044	0.00092	0.00871
EABT2913	3.73039	-1.085044	0.00092	0.00871
EABT16442	3.73039	-1.085044	0.00092	0.00871
EABT9403	3.73039	-1.085044	0.00092	0.00871
EABT10981	3.73039	-1.085044	0.00092	0.00871
EABT38027	3.73039	-1.085044	0.00092	0.00871
EABT16139	3.73039	-1.085044	0.00092	0.00871
EABT13304	-3.7222	-1.086522	0.00092	0.00871
EABT31127	-2.3825	2.129283	0.00092	0.00872
EABT16580	2.26679	8.337782	0.00092	0.00873
EABT30741	-2.2807	5.182943	0.00092	0.00873

EABT14137	2.64549	0.555741	0.00093	0.00873
EABT33838	2.64549	0.555741	0.00093	0.00873
EABT25139	-2.6371	0.553609	0.00093	0.00873
EABT23871	2.54518	0.967434	0.00093	0.0088
EABT36929	2.27414	5.549218	0.00093	0.00881
EABT15787	-2.2657	7.359925	0.00093	0.00881
EABT23811	2.26298	8.472042	0.00094	0.00887
EABT34089	2.26389	7.618069	0.00094	0.00887
EABT25968	2.69198	0.38238	0.00094	0.00889
EABT21910	-2.6836	0.380308	0.00094	0.00889
EABT22212	-2.7461	0.183307	0.00096	0.00908
EABT141	2.25626	7.86129	0.00097	0.0091
EABT36513	2.79449	0.075661	0.00097	0.0091
EABT12007	2.79449	0.075661	0.00097	0.0091
EABT4018	3.41011	-0.850529	0.00098	0.0091
EABT31740	3.41011	-0.850529	0.00098	0.0091
EABT36723	3.41011	-0.850529	0.00098	0.0091
EABT19409	3.41011	-0.850529	0.00098	0.0091
EABT23799	3.41011	-0.850529	0.00098	0.0091
EABT11490	3.41011	-0.850529	0.00098	0.0091
EABT12583	3.41011	-0.850529	0.00098	0.0091
EABT12338	3.41011	-0.850529	0.00098	0.0091
EABT6936	-3.4019	-0.852106	0.00098	0.0091
EABT4423	-2.4416	1.454927	0.00098	0.0091
EABT36429	2.84289	-0.043006	0.00099	0.0091
EABT29183	2.42813	1.615858	0.00099	0.0091
EABT27790	2.90259	-0.172315	0.00099	0.0091
EABT22072	-2.8943	-0.174176	0.00099	0.0091
EABT26415	-2.8943	-0.174176	0.00099	0.0091
EABT21124	-2.2507	9.264953	0.00099	0.0091
EABT35144	-2.2921	3.546531	0.00099	0.0091
EABT31349	2.30171	3.367229	0.001	0.0091
EABT12957	2.62902	0.542059	0.001	0.0091
EABT15889	3.21241	-0.648852	0.001	0.0091
EABT1561	3.21241	-0.648852	0.001	0.0091
EABT24657	3.21241	-0.648852	0.001	0.0091
EABT1948	3.21241	-0.648852	0.001	0.0091
EABT20610	-3.2041	-0.650515	0.001	0.0091
EABT19578	-3.2041	-0.650515	0.001	0.0091
EABT17027	-3.2041	-0.650515	0.001	0.0091
EABT8178	-2.9698	-0.316166	0.001	0.0091
EABT4329	-2.2593	5.585282	0.001	0.0091
EABT22257	-2.2523	6.706693	0.001	0.0091
EABT24682	3.07705	-0.471937	0.00101	0.0091
EABT31147	3.07705	-0.471937	0.00101	0.0091
EABT37209	3.07705	-0.471937	0.00101	0.0091
EABT21615	3.07705	-0.471937	0.00101	0.0091
EABT36008	-3.0688	-0.473675	0.00101	0.0091
EABT23518	7.01332	-1.850757	0.00101	0.0091
EABT625	7.01332	-1.850757	0.00101	0.0091
EABT9105	7.01332	-1.850757	0.00101	0.0091
EABT15316	7.01332	-1.850757	0.00101	0.0091
EABT15596	7.01332	-1.850757	0.00101	0.0091
EABT26848	7.01332	-1.850757	0.00101	0.0091

EABT31512	7.01332	-1.850757	0.00101	0.0091
EABT34432	7.01332	-1.850757	0.00101	0.0091
EABT14697	7.01332	-1.850757	0.00101	0.0091
EABT34725	7.01332	-1.850757	0.00101	0.0091
EABT11816	7.01332	-1.850757	0.00101	0.0091
EABT22140	7.01332	-1.850757	0.00101	0.0091
EABT12009	7.01332	-1.850757	0.00101	0.0091
EABT17298	7.01332	-1.850757	0.00101	0.0091
EABT12108	7.01332	-1.850757	0.00101	0.0091
EABT22770	7.01332	-1.850757	0.00101	0.0091
EABT26272	7.01332	-1.850757	0.00101	0.0091
EABT26268	7.01332	-1.850757	0.00101	0.0091
EABT35823	7.01332	-1.850757	0.00101	0.0091
EABT24762	7.01332	-1.850757	0.00101	0.0091
EABT18059	7.01332	-1.850757	0.00101	0.0091
EABT27612	7.01332	-1.850757	0.00101	0.0091
EABT8474	7.01332	-1.850757	0.00101	0.0091
EABT24819	7.01332	-1.850757	0.00101	0.0091
EABT6380	7.01332	-1.850757	0.00101	0.0091
EABT6274	7.01332	-1.850757	0.00101	0.0091
EABT18788	7.01332	-1.850757	0.00101	0.0091
EABT22258	7.01332	-1.850757	0.00101	0.0091
EABT21819	7.01332	-1.850757	0.00101	0.0091
EABT34899	7.01332	-1.850757	0.00101	0.0091
EABT14655	7.01332	-1.850757	0.00101	0.0091
EABT6917	7.01332	-1.850757	0.00101	0.0091
EABT14172	7.01332	-1.850757	0.00101	0.0091
EABT2947	7.01332	-1.850757	0.00101	0.0091
EABT17747	7.01332	-1.850757	0.00101	0.0091
EABT32642	7.01332	-1.850757	0.00101	0.0091
EABT5202	7.01332	-1.850757	0.00101	0.0091
EABT35128	7.01332	-1.850757	0.00101	0.0091
EABT13592	7.01332	-1.850757	0.00101	0.0091
EABT36589	7.01332	-1.850757	0.00101	0.0091
EABT28450	7.01332	-1.850757	0.00101	0.0091
EABT37836	7.01332	-1.850757	0.00101	0.0091
EABT14129	7.01332	-1.850757	0.00101	0.0091
EABT37357	7.01332	-1.850757	0.00101	0.0091
EABT36314	7.01332	-1.850757	0.00101	0.0091
EABT13157	7.01332	-1.850757	0.00101	0.0091
EABT17720	7.01332	-1.850757	0.00101	0.0091
EABT31175	7.01332	-1.850757	0.00101	0.0091
EABT762	7.01332	-1.850757	0.00101	0.0091
EABT17098	7.01332	-1.850757	0.00101	0.0091
EABT12442	7.01332	-1.850757	0.00101	0.0091
EABT3821	7.01332	-1.850757	0.00101	0.0091
EABT27569	7.01332	-1.850757	0.00101	0.0091
EABT18115	7.01332	-1.850757	0.00101	0.0091
EABT24954	7.01332	-1.850757	0.00101	0.0091
EABT28132	7.01332	-1.850757	0.00101	0.0091
EABT17987	7.01332	-1.850757	0.00101	0.0091
EABT28536	7.01332	-1.850757	0.00101	0.0091
EABT21201	7.01332	-1.850757	0.00101	0.0091
EABT9518	7.01332	-1.850757	0.00101	0.0091

EABT33446	7.01332	-1.850757	0.00101	0.0091
EABT10213	7.01332	-1.850757	0.00101	0.0091
EABT17763	7.01332	-1.850757	0.00101	0.0091
EABT4047	7.01332	-1.850757	0.00101	0.0091
EABT7819	7.01332	-1.850757	0.00101	0.0091
EABT26778	7.01332	-1.850757	0.00101	0.0091
EABT31370	7.01332	-1.850757	0.00101	0.0091
EABT24753	7.01332	-1.850757	0.00101	0.0091
EABT31501	7.01332	-1.850757	0.00101	0.0091
EABT691	7.01332	-1.850757	0.00101	0.0091
EABT20720	7.01332	-1.850757	0.00101	0.0091
EABT21005	7.01332	-1.850757	0.00101	0.0091
EABT353	7.01332	-1.850757	0.00101	0.0091
EABT11889	7.01332	-1.850757	0.00101	0.0091
EABT8898	7.01332	-1.850757	0.00101	0.0091
EABT1894	7.01332	-1.850757	0.00101	0.0091
EABT21464	7.01332	-1.850757	0.00101	0.0091
EABT14650	7.01332	-1.850757	0.00101	0.0091
EABT28746	7.01332	-1.850757	0.00101	0.0091
EABT35865	7.01332	-1.850757	0.00101	0.0091
EABT24573	7.01332	-1.850757	0.00101	0.0091
EABT22210	7.01332	-1.850757	0.00101	0.0091
EABT35754	7.01332	-1.850757	0.00101	0.0091
EABT21587	7.01332	-1.850757	0.00101	0.0091
EABT36304	7.01332	-1.850757	0.00101	0.0091
EABT37603	7.01332	-1.850757	0.00101	0.0091
EABT33440	7.01332	-1.850757	0.00101	0.0091
EABT32246	7.01332	-1.850757	0.00101	0.0091
EABT4717	7.01332	-1.850757	0.00101	0.0091
EABT12142	7.01332	-1.850757	0.00101	0.0091
EABT11492	7.01332	-1.850757	0.00101	0.0091
EABT33524	7.01332	-1.850757	0.00101	0.0091
EABT36579	7.01332	-1.850757	0.00101	0.0091
EABT30198	7.01332	-1.850757	0.00101	0.0091
EABT35357	7.01332	-1.850757	0.00101	0.0091
EABT19850	7.01332	-1.850757	0.00101	0.0091
EABT33165	7.01332	-1.850757	0.00101	0.0091
EABT26136	7.01332	-1.850757	0.00101	0.0091
EABT12080	7.01332	-1.850757	0.00101	0.0091
EABT29200	7.01332	-1.850757	0.00101	0.0091
EABT7904	7.01332	-1.850757	0.00101	0.0091
EABT15191	7.01332	-1.850757	0.00101	0.0091
EABT1061	7.01332	-1.850757	0.00101	0.0091
EABT31521	7.01332	-1.850757	0.00101	0.0091
EABT19833	7.01332	-1.850757	0.00101	0.0091
EABT28968	7.01332	-1.850757	0.00101	0.0091
EABT1149	7.01332	-1.850757	0.00101	0.0091
EABT19958	7.01332	-1.850757	0.00101	0.0091
EABT30601	7.01332	-1.850757	0.00101	0.0091
EABT15070	-7.0091	-1.851879	0.00101	0.0091
EABT24378	-7.0091	-1.851879	0.00101	0.0091
EABT11849	-7.0091	-1.851879	0.00101	0.0091
EABT28566	-7.0091	-1.851879	0.00101	0.0091
EABT17109	-7.0091	-1.851879	0.00101	0.0091

EABT3697	-7.0091	-1.851879	0.00101	0.0091
EABT34146	-7.0091	-1.851879	0.00101	0.0091
EABT25242	-7.0091	-1.851879	0.00101	0.0091
EABT18337	-7.0091	-1.851879	0.00101	0.0091
EABT2805	2.64969	0.457156	0.00101	0.00914
EABT6965	2.24804	7.961987	0.00101	0.00915
EABT1732	-2.2495	6.942219	0.00101	0.00915
EABT27183	2.50381	1.066294	0.00102	0.00919
EABT15537	-2.3385	2.399138	0.00102	0.00919
EABT13977	-2.2945	3.295086	0.00102	0.00919
EABT19526	-2.2444	9.079528	0.00103	0.00919
EABT3453	-2.665	0.364886	0.00103	0.00919
EABT14974	2.31463	2.875169	0.00103	0.00919
EABT34527	-2.3023	3.094579	0.00103	0.00919
EABT945	4.30164	-1.417699	0.00103	0.00919
EABT31692	4.30164	-1.417699	0.00103	0.00919
EABT37606	4.30164	-1.417699	0.00103	0.00919
EABT28422	4.30164	-1.417699	0.00103	0.00919
EABT11342	4.30164	-1.417699	0.00103	0.00919
EABT35179	4.30164	-1.417699	0.00103	0.00919
EABT31504	4.30164	-1.417699	0.00103	0.00919
EABT26027	4.30164	-1.417699	0.00103	0.00919
EABT9325	4.30164	-1.417699	0.00103	0.00919
EABT13500	4.30164	-1.417699	0.00103	0.00919
EABT2309	4.30164	-1.417699	0.00103	0.00919
EABT8052	4.30164	-1.417699	0.00103	0.00919
EABT26975	4.30164	-1.417699	0.00103	0.00919
EABT13584	4.30164	-1.417699	0.00103	0.00919
EABT619	4.30164	-1.417699	0.00103	0.00919
EABT20883	4.30164	-1.417699	0.00103	0.00919
EABT32157	4.30164	-1.417699	0.00103	0.00919
EABT9492	4.30164	-1.417699	0.00103	0.00919
EABT15747	4.30164	-1.417699	0.00103	0.00919
EABT22168	4.30164	-1.417699	0.00103	0.00919
EABT23458	4.30164	-1.417699	0.00103	0.00919
EABT29978	4.30164	-1.417699	0.00103	0.00919
EABT35880	4.30164	-1.417699	0.00103	0.00919
EABT37914	4.30164	-1.417699	0.00103	0.00919
EABT120	4.30164	-1.417699	0.00103	0.00919
EABT6313	4.30164	-1.417699	0.00103	0.00919
EABT24580	4.30164	-1.417699	0.00103	0.00919
EABT11234	4.30164	-1.417699	0.00103	0.00919
EABT29212	4.30164	-1.417699	0.00103	0.00919
EABT11946	4.30164	-1.417699	0.00103	0.00919
EABT2663	-4.2937	-1.419018	0.00103	0.00919
EABT15281	-4.2937	-1.419018	0.00103	0.00919
EABT13019	-4.2937	-1.419018	0.00103	0.00919
EABT25884	-4.2937	-1.419018	0.00103	0.00919
EABT2142	-4.2937	-1.419018	0.00103	0.00919
EABT35863	-2.2901	3.315292	0.00104	0.00931
EABT25959	-2.2569	4.738298	0.00104	0.00931
EABT13617	-2.2519	5.471507	0.00104	0.00933
EABT36493	-2.3196	2.674098	0.00105	0.00935
EABT33767	2.29623	3.20527	0.00105	0.00936

EABT146	2.27209	3.966355	0.00106	0.00946
EABT22166	2.5298	0.883144	0.00106	0.00946
EABT10662	2.73311	0.167596	0.00106	0.00946
EABT17539	-2.7248	0.165614	0.00106	0.00946
EABT26263	-2.4496	1.260228	0.00107	0.00955
EABT13048	-2.2356	8.339364	0.00107	0.00955
EABT28986	-2.2698	3.717217	0.00108	0.00959
EABT25790	2.23448	8.467288	0.00108	0.00959
EABT22529	2.77145	0.056543	0.00108	0.00961
EABT13369	2.77145	0.056543	0.00108	0.00961
EABT22086	-2.2464	5.269383	0.00108	0.00962
EABT4666	-2.2424	5.585705	0.00109	0.00966
EABT5658	-2.2448	5.343565	0.00109	0.00968
EABT27751	-2.2644	3.765013	0.0011	0.00976
EABT17043	-2.4119	1.476628	0.0011	0.00977
EABT15294	-2.2861	3.122373	0.0011	0.00978
EABT26612	2.81785	-0.063775	0.0011	0.00978
EABT5236	2.81785	-0.063775	0.0011	0.00978
EABT10861	-2.2373	5.716046	0.00111	0.00984
EABT8022	-2.3081	2.64566	0.00111	0.00987
EABT32238	2.56471	0.673317	0.00111	0.00989
EABT29109	-2.2314	6.230275	0.00112	0.0099
EABT1500	-2.2367	5.534919	0.00112	0.00992
EABT29318	-2.2292	6.560743	0.00112	0.00994
EABT4369	-2.8668	-0.196884	0.00112	0.00995
