# Supplementary material for: Transcriptome Analysis of the Emerald Ash Borer (EAB), Agrilus planipennis: De Novo Assembly, Functional Annotation and Comparative Analysis
Source: PLoS One. 2015 Aug 5;10(8):e0134824. doi: 10.1371/journal.pone.0134824 (PMC4526369; doi:10.1371/journal.pone.0134824)
Supplement: S4 Table — (PDF) [file pone.0134824.s007.pdf]

GeneID	logFC	logCPM	PValue	FDR
EABT30825	-12.224	12.9088	6.27E-32	1.36E-27
EABT3117	11.6658	11.6832	5.06E-30	5.50E-26
EABT967	-11.42	13.7889	1.01E-29	7.10E-26
EABT26072	-11.632	11.058	1.31E-29	7.10E-26
EABT37729	11.9554	9.5995	1.79E-29	7.78E-26
EABT25720	11.3353	11.8659	3.44E-29	1.25E-25
EABT33854	11.1245	13.547	7.52E-29	2.33E-25
EABT3091	11.2725	10.4861	1.83E-28	4.96E-25
EABT275	-11.115	10.4216	4.05E-28	9.78E-25
EABT7214	11.9861	8.65341	6.47E-28	1.41E-24
EABT25798	-10.858	11.2814	1.02E-27	2.01E-24
EABT36743	11.5401	8.52023	4.88E-27	8.62E-24
EABT27263	11.3268	8.78179	5.16E-27	8.62E-24
EABT22472	10.736	9.61488	1.08E-26	1.68E-23
EABT18879	11.6538	7.92149	2.94E-26	4.25E-23
EABT14053	12.4172	7.21641	1.49E-25	1.90E-22
EABT16002	-10.596	8.71098	1.53E-25	1.90E-22
EABT22210	10.5511	8.51312	1.57E-25	1.90E-22
EABT1805	11.3916	7.65949	1.80E-25	2.06E-22
EABT2787	11.3178	7.58571	3.00E-25	3.26E-22
EABT13825	14.9144	6.61041	8.93E-25	9.24E-22
EABT16135	-10.592	7.72402	1.21E-24	1.20E-21
EABT21639	-9.6968	9.6483	4.27E-24	4.03E-21
EABT4709	9.53399	12.1226	4.50E-24	4.08E-21
EABT27049	-11.054	6.91374	7.27E-24	6.32E-21
EABT18209	9.7854	8.22816	1.40E-23	1.17E-20
EABT22914	-14.488	6.18681	1.69E-23	1.36E-20
EABT755	11.0522	6.76551	2.01E-23	1.56E-20
EABT33626	9.52296	8.85284	2.95E-23	2.21E-20
EABT32104	14.3764	6.07278	3.66E-23	2.65E-20
EABT36748	-9.4207	8.29585	1.00E-22	7.01E-20
EABT20883	-10.321	6.73992	1.05E-22	7.01E-20
EABT35227	9.88947	7.34566	1.07E-22	7.01E-20
EABT17510	14.2127	5.90914	1.13E-22	7.24E-20
EABT12768	9.88337	7.12168	2.07E-22	1.28E-19
EABT7798	-11.158	6.09811	3.41E-22	2.06E-19
EABT16001	9.99759	6.66639	6.02E-22	3.53E-19
EABT4529	11.1815	5.98167	7.51E-22	4.29E-19
EABT29247	10.1432	6.41214	9.97E-22	5.55E-19
EABT29991	9.54577	7.00248	1.14E-21	6.20E-19
EABT15251	13.8092	5.50599	1.82E-21	9.65E-19
EABT25343	-9.5251	6.65968	1.91E-21	9.73E-19
EABT10097	-9.6786	6.49961	1.93E-21	9.73E-19
EABT6482	8.58442	12.0666	2.88E-21	1.42E-18
EABT11139	8.6629	8.96278	3.89E-21	1.88E-18
EABT34995	-9.0116	7.38966	4.62E-21	2.18E-18
EABT33515	9.46693	6.44916	8.08E-21	3.73E-18
EABT6387	13.5318	5.22895	1.22E-20	5.53E-18
EABT13943	9.54546	5.81547	6.08E-20	2.70E-17
EABT1442	13.2854	4.98286	6.62E-20	2.88E-17
EABT17585	8.12444	10.313	8.03E-20	3.42E-17
EABT17216	-8.4569	7.24789	9.03E-20	3.77E-17

EABT9149	8.11302	8.23456	2.23E-19	9.14E-17
EABT453	-10.211	5.1539	2.30E-19	9.26E-17
EABT34539	-9.1883	5.61071	2.54E-19	9.87E-17
EABT30824	-10.196	5.13923	2.54E-19	9.87E-17
EABT33748	8.0224	7.69844	5.24E-19	2.00E-16
EABT13697	-12.967	4.67043	5.86E-19	2.20E-16
EABT32533	-10.043	4.98695	7.24E-19	2.66E-16
EABT30828	7.74635	10.8208	9.62E-19	3.48E-16
EABT2930	8.03106	7.12511	1.09E-18	3.90E-16
EABT6124	10.0492	4.85129	1.79E-18	6.26E-16
EABT997	7.55695	11.8573	3.19E-18	1.10E-15
EABT30380	-12.656	4.36214	4.85E-18	1.65E-15
EABT5445	-9.7421	4.68816	5.63E-18	1.88E-15
EABT2187	-12.613	4.31939	6.52E-18	2.15E-15
EABT14474	12.5081	4.2071	1.33E-17	4.30E-15
EABT9777	-8.1463	5.76167	1.35E-17	4.30E-15
EABT18649	-8.5544	4.98062	1.95E-17	6.10E-15
EABT22450	-8.3327	5.16005	1.97E-17	6.10E-15
EABT8671	7.84058	5.80881	2.02E-17	6.10E-15
EABT18711	-7.8809	6.00407	2.02E-17	6.10E-15
EABT35002	9.65773	4.46091	2.58E-17	7.68E-15
EABT10966	-12.33	4.03861	4.44E-17	1.30E-14
EABT2618	12.2422	3.94197	8.18E-17	2.37E-14
EABT16773	8.79938	4.5174	1.04E-16	2.97E-14
EABT36714	12.1187	3.81895	1.87E-16	5.27E-14
EABT19045	12.1105	3.81073	1.97E-16	5.49E-14
EABT1060	12.0882	3.78858	2.31E-16	6.36E-14
EABT25275	-9.18	4.13072	2.56E-16	6.96E-14
EABT22079	6.93233	7.83368	4.30E-16	1.15E-13
EABT19282	-6.9263	7.49863	5.26E-16	1.39E-13
EABT19342	11.9625	3.66331	5.36E-16	1.40E-13
EABT35119	6.90502	7.24156	6.68E-16	1.73E-13
EABT31821	-7.5655	5.18534	7.04E-16	1.80E-13
EABT37563	-8.0136	4.44453	7.72E-16	1.95E-13
EABT16046	-8.951	3.90413	1.21E-15	3.03E-13
EABT619	11.8381	3.53946	1.23E-15	3.05E-13
EABT30765	-6.8023	7.26712	1.35E-15	3.29E-13
EABT16683	-6.6583	11.2485	1.41E-15	3.42E-13
EABT16979	11.8078	3.50931	1.53E-15	3.65E-13
EABT20596	-8.9034	3.85707	1.67E-15	3.95E-13
EABT17412	-7.3845	4.78879	1.99E-15	4.64E-13
EABT15233	7.16062	5.1333	2.08E-15	4.76E-13
EABT34832	11.7629	3.4646	2.08E-15	4.76E-13
EABT16296	-6.7484	6.82825	2.37E-15	5.36E-13
EABT28591	6.76222	6.64904	2.59E-15	5.79E-13
EABT34185	-6.7926	6.49536	2.73E-15	6.05E-13
EABT18048	-7.5499	4.38459	4.03E-15	8.85E-13
EABT37184	-11.608	3.3248	5.72E-15	1.24E-12
EABT30012	-6.6002	6.94979	5.97E-15	1.28E-12
EABT22932	-8.7066	3.6628	6.28E-15	1.34E-12
EABT12947	6.99748	4.82189	8.33E-15	1.76E-12
EABT28763	-11.544	3.26115	8.79E-15	1.84E-12
EABT2149	6.39417	9.74948	9.57E-15	1.98E-12
EABT6078	-7.9376	3.81609	1.31E-14	2.68E-12

EABT26099	7.25122	4.4998	1.37E-14	2.76E-12
EABT18009	-6.4772	6.82822	1.37E-14	2.76E-12
EABT8268	11.4723	3.17559	1.45E-14	2.90E-12
EABT14276	6.41877	6.61485	1.89E-14	3.74E-12
EABT4501	6.70146	5.15793	2.11E-14	4.13E-12
EABT19694	8.64559	3.45376	2.40E-14	4.66E-12
EABT33313	-8.4949	3.4541	2.57E-14	4.95E-12
EABT27177	6.32681	7.32499	2.60E-14	4.96E-12
EABT11809	6.9188	4.38803	2.98E-14	5.63E-12
EABT38071	-11.356	3.07622	3.11E-14	5.82E-12
EABT23990	-6.4456	5.77171	3.55E-14	6.60E-12
EABT5736	-11.283	3.00458	5.02E-14	9.25E-12
EABT4485	6.74431	4.40433	6.37E-14	1.16E-11
EABT5943	-7.6993	3.58147	6.38E-14	1.16E-11
EABT9823	-7.3289	3.76884	7.71E-14	1.38E-11
EABT35564	-6.1141	7.66713	8.73E-14	1.55E-11
EABT29916	-6.1715	6.67205	9.41E-14	1.66E-11
EABT10896	6.38191	5.3521	9.52E-14	1.67E-11
EABT32856	11.1781	2.88342	1.01E-13	1.76E-11
EABT32687	6.04977	8.97759	1.04E-13	1.79E-11
EABT11026	6.48435	4.83763	1.07E-13	1.83E-11
EABT26183	11.1462	2.85174	1.28E-13	2.17E-11
EABT12604	-11.13	2.85446	1.37E-13	2.30E-11
EABT34850	11.0914	2.79735	1.80E-13	3.01E-11
EABT18893	-6.0958	6.35747	1.98E-13	3.29E-11
EABT14165	-6.553	4.54094	2.40E-13	3.96E-11
EABT18526	-11.013	2.73972	2.97E-13	4.85E-11
EABT25430	11.0109	2.71758	3.08E-13	5.00E-11
EABT9995	10.9628	2.66994	4.16E-13	6.64E-11
EABT19339	-10.959	2.68684	4.16E-13	6.64E-11
EABT4437	-5.9552	6.18624	5.27E-13	8.35E-11
EABT35032	-5.916	6.39286	5.37E-13	8.45E-11
EABT15948	5.71055	10.368	8.56E-13	1.34E-10
EABT19635	5.69031	9.8717	1.04E-12	1.61E-10
EABT33290	10.7024	2.41216	2.26E-12	3.48E-10
EABT22826	7.9195	2.73453	2.95E-12	4.51E-10
EABT25908	5.74911	5.21159	3.03E-12	4.60E-10
EABT1490	5.49426	9.96644	3.81E-12	5.75E-10
EABT3014	5.69943	5.1629	4.23E-12	6.33E-10
EABT13597	-5.8653	4.59161	4.64E-12	6.90E-10
EABT22013	5.81687	4.64532	4.93E-12	7.29E-10
EABT32157	-6.326	3.49284	5.10E-12	7.49E-10
EABT10867	-6.9918	2.88856	6.74E-12	9.83E-10
EABT1472	10.5316	2.24351	7.07E-12	1.02E-09
EABT27304	-5.569	5.47118	8.11E-12	1.16E-09
EABT21778	5.77252	4.43357	8.13E-12	1.16E-09
EABT10749	-6.6229	3.07746	8.26E-12	1.17E-09
EABT25456	-7.6154	2.59277	8.27E-12	1.17E-09
EABT7988	5.43221	6.62714	9.25E-12	1.30E-09
EABT18725	-5.43	6.84146	9.36E-12	1.30E-09
EABT14771	-5.4542	6.04925	1.05E-11	1.46E-09
EABT31440	5.38821	6.64483	1.24E-11	1.70E-09
EABT25426	5.32105	8.05451	1.38E-11	1.88E-09
EABT33425	-5.4875	5.34889	1.46E-11	1.98E-09

EABT1440	5.3041	7.59814	1.60E-11	2.14E-09
EABT25706	-6.5179	2.97534	1.60E-11	2.14E-09
EABT23189	5.44016	5.10088	2.09E-11	2.78E-09
EABT25148	-5.6096	4.43399	2.11E-11	2.79E-09
EABT8915	-5.974	3.40428	2.40E-11	3.15E-09
EABT7226	-7.4263	2.409	2.86E-11	3.75E-09
EABT11991	-5.3471	5.6245	2.89E-11	3.75E-09
EABT10777	-5.7427	3.89705	3.50E-11	4.52E-09
EABT28574	10.2705	1.98615	3.64E-11	4.68E-09
EABT2066	6.87089	2.60699	3.93E-11	5.02E-09
EABT31579	-6.38	2.84143	3.97E-11	5.04E-09
EABT24243	6.02681	3.29211	4.57E-11	5.77E-09
EABT8464	6.83174	2.56854	4.99E-11	6.26E-09
EABT27098	5.2299	5.4171	5.33E-11	6.66E-09
EABT8142	-6.3293	2.79221	5.41E-11	6.72E-09
EABT33660	5.18145	5.75198	6.63E-11	8.18E-09
EABT15541	-6.2979	2.76185	6.70E-11	8.18E-09
EABT19301	10.1787	1.89599	6.71E-11	8.18E-09
EABT32447	7.43669	2.259	6.74E-11	8.18E-09
EABT22492	-5.5049	4.03731	7.29E-11	8.79E-09
EABT11198	-5.1244	5.75427	9.06E-11	1.09E-08
EABT1778	-5.2118	5.03677	9.38E-11	1.12E-08
EABT15232	-5.7823	3.43378	1.02E-10	1.22E-08
EABT35543	-10.096	1.84804	1.05E-10	1.24E-08
EABT23281	10.092	1.81085	1.13E-10	1.32E-08
EABT32775	-5.1221	5.30476	1.15E-10	1.35E-08
EABT7622	7.33488	2.15912	1.25E-10	1.45E-08
EABT609	5.09443	5.34663	1.27E-10	1.47E-08
EABT14532	6.67835	2.41813	1.32E-10	1.52E-08
EABT1678	-5.9738	2.84191	1.35E-10	1.54E-08
EABT16770	10.0581	1.77759	1.38E-10	1.57E-08
EABT10391	-6.1825	2.65023	1.41E-10	1.59E-08
EABT28348	10.0466	1.76633	1.48E-10	1.66E-08
EABT30177	-5.5727	3.58358	1.52E-10	1.70E-08
EABT9267	4.92444	9.07947	1.64E-10	1.83E-08
EABT20525	-5.4341	3.73237	1.92E-10	2.13E-08
EABT31372	-10.003	1.75843	1.95E-10	2.15E-08
EABT14338	-4.923	7.46087	1.97E-10	2.16E-08
EABT36518	4.90728	6.74648	2.37E-10	2.58E-08
EABT22050	-4.8791	7.98894	2.38E-10	2.59E-08
EABT37324	-5.8807	2.75208	2.44E-10	2.64E-08
EABT33150	7.21582	2.04251	2.68E-10	2.89E-08
EABT36384	-9.9485	1.70612	2.78E-10	2.98E-08
EABT23838	9.93891	1.66085	2.99E-10	3.19E-08
EABT25095	9.92644	1.64864	3.22E-10	3.41E-08
EABT14728	5.72395	2.99618	3.24E-10	3.42E-08
EABT19672	-6.0318	2.50499	3.72E-10	3.91E-08
EABT2411	5.14706	3.99196	3.90E-10	4.07E-08
EABT8353	-5.645	2.83268	4.21E-10	4.37E-08
EABT29245	-5.3522	3.51883	4.41E-10	4.57E-08
EABT28855	4.75078	10.358	4.80E-10	4.95E-08
EABT1097	-5.1489	3.89596	4.98E-10	5.11E-08
EABT10688	-5.9735	2.44889	5.26E-10	5.37E-08
EABT31698	-9.8332	1.59542	5.48E-10	5.56E-08

EABT23217	-5.4806	2.92736	5.83E-10	5.89E-08
EABT28973	-5.3574	3.37553	6.00E-10	6.04E-08
EABT7732	-5.3504	3.36883	6.29E-10	6.30E-08
EABT36364	9.80899	1.53389	6.41E-10	6.38E-08
EABT7736	-4.9682	4.50451	6.60E-10	6.55E-08
EABT32870	-4.8217	5.58454	6.69E-10	6.61E-08
EABT38035	-9.7967	1.56047	6.94E-10	6.79E-08
EABT33770	9.79533	1.52056	6.94E-10	6.79E-08
EABT14637	7.065	1.89515	6.99E-10	6.81E-08
EABT5233	-5.5564	2.74743	7.36E-10	7.14E-08
EABT32980	-9.7843	1.54862	7.51E-10	7.25E-08
EABT24648	4.7994	5.42915	7.88E-10	7.56E-08
EABT4315	-4.7086	7.25399	7.89E-10	7.56E-08
EABT18701	-4.8829	4.67365	8.32E-10	7.92E-08
EABT29424	-4.753	5.94344	8.42E-10	7.99E-08
EABT5698	9.75356	1.47982	9.59E-10	9.04E-08
EABT30491	-4.8527	4.69	9.61E-10	9.04E-08
EABT32210	-6.198	2.12129	1.10E-09	1.03E-07
EABT14392	4.63683	7.38094	1.20E-09	1.12E-07
EABT15368	-6.8248	1.82945	1.25E-09	1.16E-07
EABT37802	9.69591	1.42366	1.34E-09	1.24E-07
EABT22719	4.72037	5.04382	1.40E-09	1.29E-07
EABT30473	-4.6162	7.10213	1.42E-09	1.29E-07
EABT4367	-5.045	3.59342	1.42E-09	1.29E-07
EABT29566	-9.6679	1.4374	1.46E-09	1.33E-07
EABT31707	-4.6538	5.71991	1.57E-09	1.43E-07
EABT27662	-4.9663	3.72038	1.59E-09	1.44E-07
EABT17212	-4.6506	5.86245	1.61E-09	1.44E-07
EABT32560	4.75825	4.4397	1.74E-09	1.56E-07
EABT36398	-6.7514	1.75932	2.01E-09	1.79E-07
EABT23572	-4.807	4.04542	2.07E-09	1.83E-07
EABT5363	-4.5076	9.83569	2.31E-09	2.04E-07
EABT8753	6.87254	1.70775	2.32E-09	2.04E-07
EABT14232	-4.5949	5.60478	2.39E-09	2.09E-07
EABT10562	-4.4959	10.956	2.47E-09	2.16E-07
EABT28053	-4.5015	8.7296	2.54E-09	2.21E-07
EABT29685	9.57321	1.30437	2.71E-09	2.35E-07
EABT11465	-4.4924	7.77436	2.85E-09	2.45E-07
EABT16054	-4.5251	6.16011	3.00E-09	2.57E-07
EABT4789	-4.8581	3.61681	3.16E-09	2.70E-07
EABT20217	-4.4898	6.86152	3.22E-09	2.75E-07
EABT8259	-4.5241	5.90477	3.24E-09	2.75E-07
EABT23144	5.83083	2.14032	3.28E-09	2.77E-07
EABT13053	-4.5538	5.48344	3.30E-09	2.78E-07
EABT9358	6.81067	1.64769	3.32E-09	2.78E-07
EABT23717	-5.0833	3.11238	3.47E-09	2.90E-07
EABT14865	-4.9782	3.29376	3.49E-09	2.91E-07
EABT30865	-9.5266	1.30278	3.58E-09	2.97E-07
EABT21737	-5.448	2.3372	3.67E-09	3.03E-07
EABT11799	4.76037	3.7692	3.93E-09	3.23E-07
EABT37380	-4.4507	7.15647	3.99E-09	3.27E-07
EABT26573	-5.6485	2.13788	4.06E-09	3.32E-07
EABT23284	-4.6487	4.35389	4.15E-09	3.37E-07
EABT13405	-5.2586	2.46274	4.69E-09	3.80E-07

EABT18084	-9.4659	1.24517	5.26E-09	4.25E-07
EABT6857	9.45656	1.1913	5.80E-09	4.65E-07
EABT15011	-9.4503	1.2304	5.80E-09	4.65E-07
EABT4989	-4.9373	3.12078	6.12E-09	4.89E-07
EABT18442	-4.6168	3.92988	6.28E-09	5.00E-07
EABT30158	-9.4346	1.21548	6.40E-09	5.08E-07
EABT12186	4.40497	6.01305	6.72E-09	5.31E-07
EABT23210	4.39909	5.90893	6.94E-09	5.46E-07
EABT21264	-9.4026	1.18515	7.08E-09	5.53E-07
EABT35586	-9.4026	1.18515	7.08E-09	5.53E-07
EABT20411	-6.5435	1.56145	7.11E-09	5.54E-07
EABT2432	6.67837	1.51954	7.70E-09	5.97E-07
EABT8523	9.3854	1.1225	8.67E-09	6.68E-07
EABT23918	9.3854	1.1225	8.67E-09	6.68E-07
EABT6035	-5.1558	2.36505	8.76E-09	6.72E-07
EABT14	-4.5342	3.97513	9.01E-09	6.89E-07
EABT10710	-6.5058	1.52564	9.04E-09	6.89E-07
EABT20512	5.1832	2.47225	9.72E-09	7.38E-07
EABT8751	-4.8055	3.12913	1.04E-08	7.85E-07
EABT5988	9.34846	1.08685	1.07E-08	8.02E-07
EABT23069	9.34846	1.08685	1.07E-08	8.02E-07
EABT22627	9.32963	1.06869	1.19E-08	8.88E-07
EABT35209	-5.1052	2.31709	1.22E-08	9.07E-07
EABT17676	-4.8714	2.91038	1.28E-08	9.53E-07
EABT8707	-4.2441	8.55339	1.30E-08	9.63E-07
EABT6530	-4.3111	5.76231	1.31E-08	9.69E-07
EABT4185	-5.2305	2.13069	1.43E-08	1.05E-06
EABT17392	-5.4441	1.94385	1.44E-08	1.06E-06
EABT19484	-4.2182	9.98021	1.45E-08	1.06E-06
EABT14420	-4.4669	3.85065	1.47E-08	1.07E-06
EABT4817	-4.5999	3.3709	1.60E-08	1.16E-06
EABT6969	5.90975	1.67107	1.61E-08	1.17E-06
EABT36242	4.56444	3.34987	1.71E-08	1.24E-06
EABT25601	-5.4054	1.90726	1.73E-08	1.24E-06
EABT12942	-5.4054	1.90726	1.73E-08	1.24E-06
EABT8158	-4.9687	2.65237	1.75E-08	1.25E-06
EABT32015	4.20835	7.25859	1.77E-08	1.26E-06
EABT5471	-9.249	1.04013	1.83E-08	1.30E-06
EABT7530	-6.3723	1.39939	1.93E-08	1.36E-06
EABT3543	5.53151	1.85148	2.09E-08	1.47E-06
EABT10669	-5.1619	2.06579	2.10E-08	1.47E-06
EABT13638	6.5017	1.34914	2.10E-08	1.47E-06
EABT21216	9.21118	0.95468	2.29E-08	1.60E-06
EABT928	-4.1681	7.17283	2.31E-08	1.61E-06
EABT29200	4.23565	4.97979	2.42E-08	1.68E-06
EABT12820	4.19913	5.68009	2.43E-08	1.68E-06
EABT15357	-4.9825	2.20105	2.55E-08	1.76E-06
EABT13818	-9.1939	0.98833	2.56E-08	1.76E-06
EABT10861	4.8053	2.32571	2.57E-08	1.76E-06
EABT33300	-4.5752	3.14569	2.69E-08	1.84E-06
EABT7615	-4.3877	3.64652	2.85E-08	1.94E-06
EABT26334	-4.8849	2.57295	2.90E-08	1.97E-06
EABT15437	4.31154	4.01279	2.92E-08	1.98E-06
EABT37986	-4.1285	6.87482	3.01E-08	2.03E-06

EABT30499	4.30852	3.96445	3.04E-08	2.05E-06
EABT14928	-5.6466	1.59734	3.15E-08	2.11E-06
EABT22850	-5.0898	1.99783	3.33E-08	2.22E-06
EABT33544	-5.6219	1.57416	3.67E-08	2.45E-06
EABT25444	-5.6095	1.56243	3.97E-08	2.64E-06
EABT29683	-4.2559	4.12141	4.10E-08	2.71E-06
EABT12927	-4.0917	6.26537	4.18E-08	2.76E-06
EABT3622	4.72242	2.24683	4.35E-08	2.86E-06
EABT7784	-4.0441	8.61024	4.48E-08	2.93E-06
EABT6528	-5.0428	1.95365	4.48E-08	2.93E-06
EABT32628	9.10445	0.85235	4.62E-08	2.99E-06
EABT27740	-9.0972	0.89759	4.62E-08	2.99E-06
EABT9354	-9.0771	0.87873	4.62E-08	2.99E-06
EABT24249	-4.1131	5.20566	4.74E-08	3.06E-06
EABT34438	-6.2251	1.26093	4.81E-08	3.10E-06
EABT7503	9.08213	0.83099	5.22E-08	3.35E-06
EABT32206	-4.0528	6.15451	5.25E-08	3.36E-06
EABT29635	4.01269	8.88469	5.27E-08	3.37E-06
EABT30241	-5.2283	1.74042	5.31E-08	3.38E-06
EABT28425	-5.0041	1.91731	5.38E-08	3.42E-06
EABT30134	-4.1369	4.67525	5.45E-08	3.45E-06
EABT4600	-4.7014	2.58512	5.46E-08	3.45E-06
EABT37640	-4.9943	1.90808	5.72E-08	3.60E-06
EABT31646	-4.3245	3.36858	5.79E-08	3.64E-06
EABT26001	-6.1938	1.23156	5.84E-08	3.65E-06
EABT18637	9.05945	0.80931	5.90E-08	3.69E-06
EABT29931	-4.5623	2.76534	6.21E-08	3.87E-06
EABT30518	9.03641	0.78731	6.69E-08	4.12E-06
EABT9454	9.03641	0.78731	6.69E-08	4.12E-06
EABT35196	-9.0149	0.82061	6.69E-08	4.12E-06
EABT24577	-9.0149	0.82061	6.69E-08	4.12E-06
EABT5253	4.54222	2.42816	6.92E-08	4.24E-06
EABT31892	-4.3622	3.14648	6.93E-08	4.24E-06
EABT2812	6.30035	1.15608	7.11E-08	4.33E-06
EABT19135	-4.358	3.14256	7.12E-08	4.33E-06
EABT21777	6.28223	1.13878	7.86E-08	4.74E-06
EABT12292	-6.129	1.17094	7.86E-08	4.74E-06
EABT19790	-6.129	1.17094	7.86E-08	4.74E-06
EABT22162	4.02226	5.31717	7.91E-08	4.76E-06
EABT11542	-4.4691	2.81104	8.12E-08	4.87E-06
EABT5749	-4.9338	1.85141	8.36E-08	5.01E-06
EABT2842	-8.972	0.78051	8.64E-08	5.14E-06
EABT30046	-8.972	0.78051	8.64E-08	5.14E-06
EABT21125	-4.4958	2.70276	9.17E-08	5.44E-06
EABT21251	-4.0866	4.1302	9.73E-08	5.76E-06
EABT3041	3.90128	8.54148	1.05E-07	6.21E-06
EABT30444	8.94038	0.69581	1.12E-07	6.57E-06
EABT24693	8.94038	0.69581	1.12E-07	6.57E-06
EABT21953	-8.9277	0.73924	1.12E-07	6.57E-06
EABT5614	-3.884	10.5597	1.15E-07	6.72E-06
EABT12935	-4.1285	3.53192	1.19E-07	6.90E-06
EABT25131	4.88406	1.93267	1.20E-07	6.96E-06
EABT27899	-4.5664	2.45823	1.24E-07	7.16E-06
EABT35594	-5.0764	1.5983	1.27E-07	7.33E-06

EABT12961	-5.4081	1.37383	1.27E-07	7.33E-06
EABT16982	-4.3501	2.82138	1.30E-07	7.49E-06
EABT26594	6.18805	1.04901	1.32E-07	7.54E-06
EABT24201	-5.0641	1.58676	1.37E-07	7.85E-06
EABT1357	-3.9351	5.03858	1.42E-07	8.10E-06
EABT25683	-6.0261	1.0749	1.46E-07	8.33E-06
EABT27427	3.86166	6.80296	1.52E-07	8.60E-06
EABT27934	-3.8736	6.07868	1.59E-07	8.97E-06
EABT27006	5.19599	1.53084	1.60E-07	9.04E-06
EABT5856	6.14858	1.01149	1.63E-07	9.18E-06
EABT13228	8.86392	0.62322	1.68E-07	9.43E-06
EABT32187	8.86392	0.62322	1.68E-07	9.43E-06
EABT31391	-5.9901	1.0414	1.82E-07	1.02E-05
EABT31921	-3.8205	7.40297	1.86E-07	1.04E-05
EABT2412	-4.0076	3.88848	1.88E-07	1.05E-05
EABT10535	-3.8172	7.25875	1.92E-07	1.07E-05
EABT37351	8.83751	0.59819	1.94E-07	1.07E-05
EABT7570	-3.9523	4.11891	1.99E-07	1.10E-05
EABT28043	3.81819	6.67286	2.00E-07	1.10E-05
EABT31108	-4.782	1.70976	2.00E-07	1.10E-05
EABT29575	-5.9717	1.02435	2.03E-07	1.11E-05
EABT27307	-3.8087	6.85029	2.11E-07	1.15E-05
EABT458	-4.4055	2.4714	2.18E-07	1.19E-05
EABT9692	3.7948	7.13461	2.20E-07	1.19E-05
EABT14616	3.83321	5.63074	2.20E-07	1.19E-05
EABT18946	3.8403	5.32941	2.22E-07	1.19E-05
EABT17326	-4.0118	3.48346	2.22E-07	1.19E-05
EABT5198	8.8106	0.57273	2.23E-07	1.19E-05
EABT10306	8.8106	0.57273	2.23E-07	1.19E-05
EABT5605	-8.8105	0.63055	2.23E-07	1.19E-05
EABT37124	5.12428	1.46283	2.38E-07	1.27E-05
EABT3973	5.12428	1.46283	2.38E-07	1.27E-05
EABT9152	-3.8432	5.00911	2.39E-07	1.27E-05
EABT3661	-4.1298	3.02006	2.41E-07	1.27E-05
EABT1942	-4.3145	2.53325	2.66E-07	1.41E-05
EABT3174	-4.589	1.83286	2.69E-07	1.42E-05
EABT6353	-5.9153	0.97194	2.84E-07	1.50E-05
EABT33803	-5.2722	1.24747	2.91E-07	1.53E-05
EABT8161	8.75523	0.52044	2.97E-07	1.56E-05
EABT2468	-5.8959	0.95404	3.19E-07	1.66E-05
EABT12161	-5.8959	0.95404	3.19E-07	1.66E-05
EABT10552	-4.1076	2.90851	3.20E-07	1.66E-05
EABT11118	-3.7161	8.93049	3.24E-07	1.68E-05
EABT17233	4.8511	1.59483	3.32E-07	1.72E-05
EABT26089	8.72673	0.49357	3.45E-07	1.78E-05
EABT20768	-4.8928	1.42765	3.60E-07	1.86E-05
EABT23916	3.69712	8.42754	3.65E-07	1.87E-05
EABT10866	-3.7032	7.24829	3.78E-07	1.94E-05
EABT26113	-8.7095	0.5372	4.00E-07	2.05E-05
EABT22356	6.00128	0.872	4.03E-07	2.05E-05
EABT24519	-5.8363	0.89894	4.03E-07	2.05E-05
EABT28025	-4.1211	2.72021	4.15E-07	2.11E-05
EABT9016	5.0175	1.36192	4.27E-07	2.16E-05
EABT14160	-3.9266	3.5178	4.30E-07	2.17E-05

EABT25041	3.68323	6.85555	4.44E-07	2.24E-05
EABT35490	3.75347	4.85653	4.49E-07	2.26E-05
EABT6069	-4.104	2.70432	4.62E-07	2.31E-05
EABT30568	4.32186	1.86925	4.62E-07	2.31E-05
EABT1551	8.69765	0.4662	4.66E-07	2.32E-05
EABT37035	-8.6831	0.51288	4.66E-07	2.32E-05
EABT34914	-3.6892	6.16792	4.69E-07	2.33E-05
EABT14874	-4.6377	1.57611	4.83E-07	2.40E-05
EABT37717	-4.2077	2.43395	5.17E-07	2.56E-05
EABT23521	-4.625	1.56438	5.22E-07	2.58E-05
EABT22462	-3.7247	4.93539	5.23E-07	2.58E-05
EABT5238	-3.6306	10.3347	5.35E-07	2.63E-05
EABT33362	-3.742	4.60905	5.41E-07	2.65E-05
EABT12363	8.66798	0.43831	5.43E-07	2.65E-05
EABT26818	-8.6562	0.48813	5.43E-07	2.65E-05
EABT8730	3.65431	6.23815	5.63E-07	2.74E-05
EABT141	-3.6979	5.01435	5.77E-07	2.80E-05
EABT26504	-4.4453	1.7	6.04E-07	2.93E-05
EABT37726	3.79525	3.65627	6.26E-07	3.02E-05
EABT8872	-3.6867	4.8267	6.26E-07	3.02E-05
EABT9758	8.63768	0.40987	6.35E-07	3.05E-05
EABT36043	-8.6008	0.43732	6.35E-07	3.05E-05
EABT9638	3.61948	6.10252	7.07E-07	3.39E-05
EABT11153	-5.1045	1.09259	7.16E-07	3.42E-05
EABT19564	-3.5955	7.45232	7.17E-07	3.42E-05
EABT23901	-5.7312	0.80211	7.42E-07	3.53E-05
EABT207	8.60673	0.38087	7.45E-07	3.53E-05
EABT23685	8.60673	0.38087	7.45E-07	3.53E-05
EABT27944	-8.5723	0.41121	7.45E-07	3.53E-05
EABT5357	3.59315	6.03743	8.21E-07	3.88E-05
EABT8476	-3.6301	5.01696	8.31E-07	3.92E-05
EABT37996	-3.7473	3.69084	8.54E-07	4.01E-05
EABT26515	3.68811	3.94847	8.54E-07	4.01E-05
EABT15009	-8.5433	0.38462	8.76E-07	4.08E-05
EABT12677	-8.5433	0.38462	8.76E-07	4.08E-05
EABT24178	-8.5433	0.38462	8.76E-07	4.08E-05
EABT8349	-5.0685	1.0595	8.84E-07	4.11E-05
EABT3897	4.67969	1.43364	9.09E-07	4.21E-05
EABT33384	-4.5192	1.46699	9.09E-07	4.21E-05
EABT34019	-3.7849	3.44038	9.18E-07	4.24E-05
EABT24220	-4.0152	2.51022	9.41E-07	4.34E-05
EABT14968	-3.5813	5.63707	9.47E-07	4.36E-05
EABT23738	5.83721	0.71774	9.57E-07	4.40E-05
EABT19190	3.52702	11.3223	9.90E-07	4.53E-05
EABT33237	-3.788	3.15316	9.91E-07	4.53E-05
EABT22395	3.69493	3.8244	1.02E-06	4.64E-05
EABT18342	3.53026	7.81176	1.03E-06	4.64E-05
EABT16857	8.54277	0.32108	1.03E-06	4.64E-05
EABT1965	8.54277	0.32108	1.03E-06	4.64E-05
EABT2380	-8.5136	0.35752	1.03E-06	4.64E-05
EABT31166	-8.5136	0.35752	1.03E-06	4.64E-05
EABT5757	-8.5136	0.35752	1.03E-06	4.64E-05
EABT19743	-4.2002	2.11765	1.03E-06	4.64E-05
EABT20811	4.86746	1.22096	1.05E-06	4.70E-05

EABT32222	-3.9952	2.49182	1.06E-06	4.76E-05
EABT21515	5.81217	0.69431	1.09E-06	4.87E-05
EABT7892	-3.528	6.30868	1.16E-06	5.18E-05
EABT33997	4.06317	1.97898	1.17E-06	5.22E-05
EABT16125	8.50969	0.29025	1.22E-06	5.39E-05
EABT1119	8.50969	0.29025	1.22E-06	5.39E-05
EABT7873	-8.4833	0.3299	1.22E-06	5.39E-05
EABT22092	-8.4833	0.3299	1.22E-06	5.39E-05
EABT17423	5.78669	0.67049	1.24E-06	5.46E-05
EABT27820	-5.6412	0.71961	1.24E-06	5.46E-05
EABT24636	-5.6412	0.71961	1.24E-06	5.46E-05
EABT8096	-3.4889	9.3261	1.25E-06	5.48E-05
EABT1129	3.48537	10.995	1.27E-06	5.53E-05
EABT37440	4.61771	1.37568	1.27E-06	5.53E-05
EABT29831	-5.6178	0.69822	1.42E-06	6.19E-05
EABT19111	8.47584	0.25875	1.45E-06	6.28E-05
EABT19259	-3.7983	2.79907	1.51E-06	6.55E-05
EABT24845	-3.4777	6.47221	1.53E-06	6.60E-05
EABT3532	3.45879	8.03306	1.54E-06	6.66E-05
EABT22740	3.47349	6.33844	1.56E-06	6.74E-05
EABT20383	-3.5106	5.15551	1.58E-06	6.78E-05
EABT32767	-4.067	2.15785	1.60E-06	6.86E-05
EABT12360	-3.6589	3.15784	1.78E-06	7.60E-05
EABT35365	3.56228	3.86338	1.78E-06	7.60E-05
EABT7273	-3.4697	5.37541	1.87E-06	7.96E-05
EABT2393	-3.4663	5.36106	1.91E-06	8.14E-05
EABT7366	-3.5179	4.28465	1.93E-06	8.23E-05
EABT16629	8.40565	0.19363	2.05E-06	8.69E-05
EABT35116	-4.5818	1.14205	2.07E-06	8.74E-05
EABT20028	-4.5818	1.14205	2.07E-06	8.74E-05
EABT36847	-3.406	7.70876	2.13E-06	8.98E-05
EABT16890	-4.9147	0.91888	2.16E-06	9.08E-05
EABT28206	-4.8943	0.90029	2.16E-06	9.08E-05
EABT12365	3.39305	10.4059	2.18E-06	9.16E-05
EABT1732	3.40059	7.79199	2.19E-06	9.16E-05
EABT17674	-3.8331	2.4549	2.25E-06	9.38E-05
EABT20790	-3.5103	4.12168	2.27E-06	9.46E-05
EABT34549	-3.5742	3.53203	2.34E-06	9.74E-05
EABT1030	-4.0637	1.99222	2.34E-06	9.74E-05
EABT19916	-3.5864	3.45441	2.40E-06	9.93E-05
EABT31247	-4.8736	0.88145	2.43E-06	0.0001
EABT7074	8.36924	0.15996	2.45E-06	0.0001
EABT31335	8.36924	0.15996	2.45E-06	0.0001
EABT37896	8.36924	0.15996	2.45E-06	0.0001
EABT28373	8.36924	0.15996	2.45E-06	0.0001
EABT18603	-8.3554	0.21372	2.45E-06	0.0001
EABT14270	5.65206	0.54521	2.47E-06	0.0001
EABT4723	5.65206	0.54521	2.47E-06	0.0001
EABT37496	4.35174	1.43258	2.52E-06	0.0001
EABT24462	-4.1918	1.46802	2.52E-06	0.0001
EABT35708	-3.3667	8.29543	2.59E-06	0.00011
EABT4811	-3.414	5.1582	2.63E-06	0.00011
EABT5243	-4.5288	1.09383	2.81E-06	0.00011
EABT17985	-4.5288	1.09383	2.81E-06	0.00011

EABT4901	8.33188	0.12549	2.94E-06	0.00012
EABT11060	8.33188	0.12549	2.94E-06	0.00012
EABT16648	8.33188	0.12549	2.94E-06	0.00012
EABT16210	8.33188	0.12549	2.94E-06	0.00012
EABT2454	-8.3215	0.18313	2.94E-06	0.00012
EABT9458	-3.4807	3.91969	3.12E-06	0.00012
EABT38034	-3.628	2.80005	3.25E-06	0.00013
EABT31441	-3.6809	2.60677	3.33E-06	0.00013
EABT9871	4.45	1.21977	3.35E-06	0.00013
EABT24497	-3.4759	3.64313	3.38E-06	0.00013
EABT4812	-3.3878	4.79525	3.43E-06	0.00014
EABT9829	-4.4923	1.06075	3.46E-06	0.00014
EABT23089	8.29352	0.09019	3.54E-06	0.00014
EABT9440	-8.2869	0.15187	3.54E-06	0.00014
EABT12493	-8.2869	0.15187	3.54E-06	0.00014
EABT193	-4.0282	1.56634	3.61E-06	0.00014
EABT9318	-4.2648	1.23502	3.68E-06	0.00014
EABT5353	-3.9751	1.91134	3.74E-06	0.00015
EABT16495	-3.3089	7.47652	3.79E-06	0.00015
EABT24244	-5.4423	0.53877	3.81E-06	0.00015
EABT3121	-4.0152	1.55454	3.89E-06	0.00015
EABT31783	-3.6991	2.43638	3.91E-06	0.00015
EABT29344	3.33991	5.06539	4.04E-06	0.00016
EABT4959	3.3202	5.83499	4.09E-06	0.00016
EABT145	-3.4293	3.67391	4.18E-06	0.00016
EABT20433	-3.2965	6.69741	4.26E-06	0.00016
EABT32264	8.25412	0.05402	4.28E-06	0.00016
EABT8044	8.25412	0.05402	4.28E-06	0.00016
EABT17893	8.25412	0.05402	4.28E-06	0.00016
EABT26543	8.25412	0.05402	4.28E-06	0.00016
EABT23919	-8.2514	0.1199	4.28E-06	0.00016
EABT29154	-8.2514	0.1199	4.28E-06	0.00016
EABT12355	3.32943	5.14514	4.31E-06	0.00016
EABT27931	-3.2938	6.56887	4.34E-06	0.00016
EABT7651	-3.3192	5.29345	4.35E-06	0.00016
EABT36436	5.5648	0.46454	4.42E-06	0.00017
EABT17955	5.5648	0.46454	4.42E-06	0.00017
EABT10268	-4.2316	1.20508	4.45E-06	0.00017
EABT1441	-3.5132	2.90246	4.69E-06	0.00018
EABT9487	3.26101	10.2952	4.69E-06	0.00018
EABT6582	-3.2939	5.6484	4.90E-06	0.00018
EABT33399	-3.2529	10.0446	4.92E-06	0.00018
EABT16222	-3.5491	2.72853	5.03E-06	0.00019
EABT18667	4.89866	0.71625	5.07E-06	0.00019
EABT7797	-4.7426	0.76289	5.07E-06	0.00019
EABT7914	5.5345	0.43663	5.15E-06	0.00019
EABT14294	-5.3601	0.46457	5.15E-06	0.00019
EABT7426	8.21361	0.01693	5.19E-06	0.00019
EABT32239	8.21361	0.01693	5.19E-06	0.00019
EABT28893	-3.2757	6.00997	5.19E-06	0.00019
EABT28570	3.59626	2.44457	5.39E-06	0.0002
EABT33129	3.30746	4.62602	5.51E-06	0.0002
EABT6965	-3.2512	6.6031	5.52E-06	0.0002
EABT25312	-3.6074	2.44975	5.61E-06	0.00021

EABT34904	5.50355	0.40817	6.00E-06	0.00022
EABT8628	-3.4789	2.806	6.13E-06	0.00022
EABT26199	3.21743	7.99092	6.14E-06	0.00022
EABT35072	3.21467	8.01484	6.24E-06	0.00023
EABT16069	-3.8177	1.93052	6.27E-06	0.00023
EABT16517	-8.1776	0.05374	6.32E-06	0.00023
EABT11388	-8.1776	0.05374	6.32E-06	0.00023
EABT9432	-8.1776	0.05374	6.32E-06	0.00023
EABT28645	8.17194	-0.0211	6.32E-06	0.00023
EABT21103	8.17194	-0.0211	6.32E-06	0.00023
EABT2673	-3.502	2.686	6.42E-06	0.00023
EABT23436	-4.6962	0.72107	6.56E-06	0.00024
EABT8569	-3.5184	2.62485	6.59E-06	0.00024
EABT13472	-5.3025	0.41287	7.02E-06	0.00025
EABT36241	-3.4449	2.84081	7.03E-06	0.00025
EABT23113	3.57618	2.24944	7.08E-06	0.00025
EABT7303	-8.1393	0.01946	7.72E-06	0.00027
EABT12037	-8.1393	0.01946	7.72E-06	0.00027
EABT4532	-8.1393	0.01946	7.72E-06	0.00027
EABT32143	8.12902	-0.0602	7.72E-06	0.00027
EABT32527	8.12902	-0.0602	7.72E-06	0.00027
EABT1933	8.12902	-0.0602	7.72E-06	0.00027
EABT32600	8.12902	-0.0602	7.72E-06	0.00027
EABT30857	-3.6085	2.25108	7.78E-06	0.00027
EABT5182	-4.1275	1.1113	8.02E-06	0.00028
EABT25835	-5.2728	0.38629	8.23E-06	0.00029
EABT748	-5.2728	0.38629	8.23E-06	0.00029
EABT20062	-3.4805	2.59067	8.24E-06	0.00029
EABT30436	3.67342	1.76866	8.37E-06	0.00029
EABT25644	-3.4012	2.80152	8.80E-06	0.00031
EABT13018	-3.584	2.22901	8.97E-06	0.00031
EABT10145	-4.2947	0.88281	9.39E-06	0.00033
EABT34240	-8.0999	-0.0157	9.48E-06	0.00033
EABT20633	8.08479	-0.1003	9.48E-06	0.00033
EABT19684	8.08479	-0.1003	9.48E-06	0.00033
EABT2927	8.08479	-0.1003	9.48E-06	0.00033
EABT31243	-3.8028	1.75522	9.53E-06	0.00033
EABT35331	-5.2425	0.35921	9.66E-06	0.00033
EABT3192	-5.2425	0.35921	9.66E-06	0.00033
EABT24387	-4.6236	0.65595	9.79E-06	0.00034
EABT5302	-3.3117	3.3368	9.81E-06	0.00034
EABT16317	-4.091	1.07863	9.85E-06	0.00034
EABT30353	-3.2858	3.64734	1.01E-05	0.00035
EABT15308	3.49273	2.26485	1.03E-05	0.00035
EABT37776	-3.1501	5.87753	1.03E-05	0.00035
EABT32371	-3.1391	6.25712	1.06E-05	0.00036
EABT18842	-4.0724	1.062	1.09E-05	0.00037
EABT21572	-3.918	1.22129	1.11E-05	0.00038
EABT15974	-3.4902	2.34425	1.11E-05	0.00038
EABT2939	-4.5985	0.63357	1.12E-05	0.00038
EABT36086	-3.5692	2.10448	1.16E-05	0.00039
EABT22877	-8.0594	-0.0517	1.17E-05	0.00039
EABT26196	8.03915	-0.1416	1.17E-05	0.00039
EABT12872	8.03915	-0.1416	1.17E-05	0.00039

EABT30171	8.03915	-0.1416	1.17E-05	0.00039
EABT28560	-3.2461	3.70782	1.17E-05	0.00039
EABT24403	3.42919	2.4504	1.18E-05	0.00039
EABT20443	-3.9012	1.20625	1.22E-05	0.00041
EABT29598	-3.1176	5.86767	1.27E-05	0.00042
EABT35276	-3.115	5.81332	1.27E-05	0.00042
EABT36177	4.71004	0.54361	1.29E-05	0.00043
EABT5227	3.25252	3.38632	1.30E-05	0.00043
EABT14577	3.16946	4.15795	1.33E-05	0.00044
EABT16561	-3.4516	2.30963	1.33E-05	0.00044
EABT4492	-3.8841	1.19105	1.34E-05	0.00044
EABT10408	4.39336	0.78296	1.34E-05	0.00044
EABT7641	5.338	0.25695	1.34E-05	0.00044
EABT36681	-5.18	0.30346	1.34E-05	0.00044
EABT26629	-3.0953	6.01647	1.39E-05	0.00046
EABT36369	-3.1093	5.27734	1.39E-05	0.00046
EABT33065	-3.101	5.55574	1.41E-05	0.00046
EABT31054	-3.7733	1.33733	1.43E-05	0.00047
EABT24716	-8.0177	-0.0886	1.45E-05	0.00047
EABT27746	-8.0177	-0.0886	1.45E-05	0.00047
EABT10378	-8.0177	-0.0886	1.45E-05	0.00047
EABT16467	-3.3926	2.43177	1.45E-05	0.00047
EABT34370	-3.6692	1.79674	1.47E-05	0.00048
EABT4585	3.05847	8.13712	1.50E-05	0.00049
EABT29496	-3.0613	6.86254	1.58E-05	0.00051
EABT7067	-5.1477	0.27474	1.59E-05	0.00051
EABT18591	-3.1789	3.70837	1.62E-05	0.00052
EABT16435	-3.0779	5.24937	1.65E-05	0.00053
EABT34302	-3.1738	3.51372	1.66E-05	0.00053
EABT25323	3.15846	3.64212	1.69E-05	0.00054
EABT24691	-3.0946	4.76592	1.70E-05	0.00054
EABT11629	-4.5207	0.56423	1.71E-05	0.00055
EABT14638	-4.5207	0.56423	1.71E-05	0.00055
EABT12972	-4.1848	0.78476	1.71E-05	0.00055
EABT23701	-3.1777	3.44784	1.71E-05	0.00055
EABT7328	-3.0763	5.10725	1.79E-05	0.00057
EABT21996	7.99203	-0.1841	1.80E-05	0.00057
EABT3214	-3.7261	1.29532	1.87E-05	0.00059
EABT33935	-3.0481	5.72005	1.87E-05	0.00059
EABT15390	-3.0892	4.63396	1.88E-05	0.0006
EABT15234	5.26606	0.19179	1.89E-05	0.0006
EABT10759	-5.1146	0.24543	1.89E-05	0.0006
EABT12809	-5.1146	0.24543	1.89E-05	0.0006
EABT7429	-3.0159	8.03664	1.91E-05	0.0006
EABT1130	-3.6937	1.26662	2.04E-05	0.00064
EABT25769	-3.0551	4.89666	2.07E-05	0.00065
EABT29794	3.76544	1.3575	2.10E-05	0.00066
EABT25373	-3.1892	3.14145	2.12E-05	0.00066
EABT27585	3.94749	1.0623	2.18E-05	0.00068
EABT36237	-4.1383	0.74358	2.20E-05	0.00069
EABT22712	-3.1346	3.57703	2.23E-05	0.00069
EABT23437	7.94332	-0.2278	2.25E-05	0.00069
EABT17003	7.94332	-0.2278	2.25E-05	0.00069
EABT13657	7.94332	-0.2278	2.25E-05	0.00069

EABT21457	7.94332	-0.2278	2.25E-05	0.00069
EABT1545	-7.9306	-0.1655	2.25E-05	0.00069
EABT21146	-7.9306	-0.1655	2.25E-05	0.00069
EABT32047	-7.9306	-0.1655	2.25E-05	0.00069
EABT21381	5.2287	0.15809	2.25E-05	0.00069
EABT10775	3.15994	3.1567	2.28E-05	0.0007
EABT5901	-3.2571	2.53897	2.29E-05	0.0007
EABT23616	-3.3275	2.2891	2.31E-05	0.00071
EABT34227	-3.6331	1.60311	2.49E-05	0.00076
EABT1145	-3.6331	1.60311	2.49E-05	0.00076
EABT13322	-3.3578	2.12998	2.57E-05	0.00078
EABT18035	-3.1067	3.34881	2.57E-05	0.00078
EABT33001	-3.003	4.9993	2.57E-05	0.00078
EABT30936	3.17577	2.66814	2.61E-05	0.0008
EABT4938	4.59004	0.43495	2.65E-05	0.00081
EABT8445	-4.41	0.46619	2.65E-05	0.00081
EABT20388	-3.688	1.47008	2.69E-05	0.00081
EABT2410	-3.688	1.47008	2.69E-05	0.00081
EABT23935	-3.0249	4.35357	2.80E-05	0.00085
EABT33821	7.8929	-0.2729	2.82E-05	0.00085
EABT31490	7.8929	-0.2729	2.82E-05	0.00085
EABT804	7.8929	-0.2729	2.82E-05	0.00085
EABT31500	7.8929	-0.2729	2.82E-05	0.00085
EABT33180	-7.8379	-0.2468	2.82E-05	0.00085
EABT12756	-3.1608	2.71001	2.87E-05	0.00086
EABT17775	3.71488	1.31191	2.93E-05	0.00088
EABT23153	-3.1798	2.60436	2.94E-05	0.00088
EABT7062	2.95488	5.87607	2.98E-05	0.00089
EABT19129	-3.0741	3.35595	2.98E-05	0.00089
EABT32208	-3.5284	1.67121	3.04E-05	0.00091
EABT3968	5.15095	0.08828	3.23E-05	0.00096
EABT28308	3.21122	2.4626	3.26E-05	0.00097
EABT20471	-2.9711	4.81022	3.32E-05	0.00099
EABT545	2.99977	4.15412	3.38E-05	0.001
EABT19534	-3.3425	2.01345	3.38E-05	0.001
EABT9992	-2.9774	4.42754	3.40E-05	0.001
EABT3502	-2.9855	4.19272	3.42E-05	0.00101
EABT24847	-3.0979	3.06056	3.48E-05	0.00103
EABT26518	7.84066	-0.3194	3.57E-05	0.00104
EABT6831	7.84066	-0.3194	3.57E-05	0.00104
EABT22838	7.84066	-0.3194	3.57E-05	0.00104
EABT22365	7.84066	-0.3194	3.57E-05	0.00104
EABT32652	-7.7892	-0.2893	3.57E-05	0.00104
EABT36796	-7.7892	-0.2893	3.57E-05	0.00104
EABT33859	-7.7892	-0.2893	3.57E-05	0.00104
EABT25868	-7.7892	-0.2893	3.57E-05	0.00104
EABT24211	4.52608	0.37744	3.60E-05	0.00105
EABT4986	-2.918	5.93127	3.69E-05	0.00107
EABT19402	-4.0407	0.65745	3.70E-05	0.00107
EABT23727	3.66249	1.26485	3.80E-05	0.0011
EABT12126	5.11044	0.05208	3.89E-05	0.00112
EABT746	-4.9742	0.12175	3.89E-05	0.00112
EABT2570	-4.9742	0.12175	3.89E-05	0.00112
EABT25442	-3.4783	1.6268	4.02E-05	0.00116

EABT20839	-2.9288	4.84399	4.04E-05	0.00116
EABT24301	-2.8894	6.28456	4.14E-05	0.00119
EABT26348	-3.4148	1.7152	4.15E-05	0.00119
EABT10340	-3.8061	0.82619	4.16E-05	0.00119
EABT15442	2.89499	5.72162	4.16E-05	0.00119
EABT3438	-4.0152	0.63508	4.23E-05	0.00121
EABT7326	-2.8959	5.69001	4.28E-05	0.00122
EABT25461	-3.6006	1.39255	4.34E-05	0.00124
EABT16400	-2.8579	8.58047	4.51E-05	0.00128
EABT32783	3.62647	1.23261	4.53E-05	0.00128
EABT6839	7.78645	-0.3674	4.53E-05	0.00128
EABT37633	7.78645	-0.3674	4.53E-05	0.00128
EABT35852	7.78645	-0.3674	4.53E-05	0.00128
EABT4099	-7.7388	-0.3331	4.53E-05	0.00128
EABT19318	-7.7388	-0.3331	4.53E-05	0.00128
EABT34771	5.06876	0.01497	4.69E-05	0.00132
EABT20057	-4.9368	0.08907	4.69E-05	0.00132
EABT6523	-4.9368	0.08907	4.69E-05	0.00132
EABT35148	2.85041	8.10634	4.72E-05	0.00133
EABT15371	-3.0642	2.89171	4.74E-05	0.00133
EABT10874	-3.0024	3.18218	4.82E-05	0.00135
EABT37458	-3.1363	2.36041	4.84E-05	0.00135
EABT1386	-2.9402	3.79126	4.84E-05	0.00135
EABT2867	-3.4983	1.48362	4.85E-05	0.00135
EABT24248	-3.9893	0.61236	4.85E-05	0.00135
EABT25122	-2.9529	3.70234	4.87E-05	0.00136
EABT32965	-2.8498	7.21238	4.88E-05	0.00136
EABT28595	-4.29	0.3609	4.92E-05	0.00137
EABT16403	3.67021	1.06103	5.25E-05	0.00146
EABT24863	-2.9749	3.40398	5.41E-05	0.0015
EABT15818	3.15542	2.12936	5.42E-05	0.0015
EABT23100	2.91189	3.84956	5.45E-05	0.00151
EABT536	-3.6008	0.94121	5.59E-05	0.00154
EABT5427	5.02584	-0.0231	5.69E-05	0.00156
EABT10302	5.02584	-0.0231	5.69E-05	0.00156
EABT3242	-4.8985	0.05563	5.69E-05	0.00156
EABT14545	-4.8985	0.05563	5.69E-05	0.00156
EABT35958	-2.833	6.13151	5.69E-05	0.00156
EABT16408	4.42448	0.28669	5.78E-05	0.00157
EABT15642	-4.2584	0.33331	5.78E-05	0.00157
EABT34089	-2.8327	5.8068	5.79E-05	0.00157
EABT25297	7.73013	-0.417	5.79E-05	0.00157
EABT9865	7.73013	-0.417	5.79E-05	0.00157
EABT27145	7.73013	-0.417	5.79E-05	0.00157
EABT2406	-7.6866	-0.3782	5.79E-05	0.00157
EABT20982	-7.6866	-0.3782	5.79E-05	0.00157
EABT31550	-7.6866	-0.3782	5.79E-05	0.00157
EABT32712	-7.6866	-0.3782	5.79E-05	0.00157
EABT10138	-2.9055	3.76088	5.89E-05	0.00159
EABT19795	-2.8296	5.71763	5.91E-05	0.0016
EABT26355	-3.2311	1.91541	5.92E-05	0.0016
EABT8870	3.38139	1.19844	5.94E-05	0.0016
EABT21415	-3.4825	1.08111	6.40E-05	0.00172
EABT2801	-3.936	0.56579	6.41E-05	0.00172

EABT33975	-2.8067	6.03985	6.57E-05	0.00176
EABT1631	-2.9941	2.87807	6.60E-05	0.00177
EABT18957	2.94426	3.21325	6.61E-05	0.00177
EABT3788	-3.4407	1.43289	6.62E-05	0.00177
EABT19013	-2.7833	9.14919	6.71E-05	0.00179
EABT9425	3.86609	0.68978	6.77E-05	0.0018
EABT1387	-2.9205	3.22105	6.77E-05	0.0018
EABT31468	4.38896	0.25515	6.80E-05	0.00181
EABT28744	4.38896	0.25515	6.80E-05	0.00181
EABT22848	4.98161	-0.0622	6.91E-05	0.00183
EABT9430	4.98161	-0.0622	6.91E-05	0.00183
EABT32629	2.79703	5.74762	7.02E-05	0.00186
EABT23449	3.30585	1.29411	7.17E-05	0.0019
EABT4830	2.77244	7.88926	7.19E-05	0.0019
EABT708	2.76981	8.38122	7.25E-05	0.00191
EABT9811	2.82484	4.35539	7.29E-05	0.00192
EABT5468	2.76816	7.65652	7.38E-05	0.00193
EABT17932	-2.7834	6.0971	7.41E-05	0.00193
EABT18349	-2.7733	7.00901	7.42E-05	0.00193
EABT6563	7.67152	-0.4684	7.45E-05	0.00193
EABT21455	7.67152	-0.4684	7.45E-05	0.00193
EABT35716	7.67152	-0.4684	7.45E-05	0.00193
EABT32367	7.67152	-0.4684	7.45E-05	0.00193
EABT2049	7.67152	-0.4684	7.45E-05	0.00193
EABT12275	-7.6324	-0.4249	7.45E-05	0.00193
EABT24875	-7.6324	-0.4249	7.45E-05	0.00193
EABT25767	-7.6324	-0.4249	7.45E-05	0.00193
EABT23278	-7.6324	-0.4249	7.45E-05	0.00193
EABT31349	-2.7789	6.26081	7.46E-05	0.00194
EABT29953	-2.9154	3.31925	7.64E-05	0.00198
EABT11225	-3.689	0.72399	7.67E-05	0.00199
EABT31320	3.10187	2.00166	7.77E-05	0.00201
EABT31473	-3.4753	1.28217	7.81E-05	0.00202
EABT31178	-2.8382	3.98631	7.87E-05	0.00203
EABT16432	-3.0593	2.21755	7.92E-05	0.00204
EABT16154	-2.9751	2.76413	8.04E-05	0.00207
EABT17254	-4.8186	-0.0137	8.44E-05	0.00216
EABT2844	-4.8186	-0.0137	8.44E-05	0.00216
EABT26256	-4.8186	-0.0137	8.44E-05	0.00216
EABT18507	-3.4588	1.26776	8.52E-05	0.00218
EABT27782	-3.8521	0.49294	8.52E-05	0.00218
EABT32718	-2.9898	2.61738	8.55E-05	0.00219
EABT12231	-2.7683	5.13958	8.67E-05	0.00221
EABT37663	3.58547	0.98575	8.70E-05	0.00222
EABT34978	3.81176	0.64166	8.71E-05	0.00222
EABT27863	-2.762	5.37017	8.85E-05	0.00225
EABT17895	-2.7527	5.63264	8.93E-05	0.00227
EABT24720	-2.7349	7.40647	8.98E-05	0.00228
EABT381	-3.1784	1.75877	9.09E-05	0.0023
EABT7211	-2.7444	6.10266	9.18E-05	0.00232
EABT28544	-2.7773	4.65254	9.28E-05	0.00235
EABT24514	3.03765	2.10202	9.28E-05	0.00235
EABT31432	-2.9395	2.73312	9.46E-05	0.00238
EABT37850	-3.2496	1.57025	9.46E-05	0.00238

EABT25535	-4.1591	0.24719	9.50E-05	0.00239
EABT3348	7.61043	-0.5217	9.64E-05	0.00239
EABT35959	7.61043	-0.5217	9.64E-05	0.00239
EABT1503	7.61043	-0.5217	9.64E-05	0.00239
EABT21756	7.61043	-0.5217	9.64E-05	0.00239
EABT2255	7.61043	-0.5217	9.64E-05	0.00239
EABT19583	7.61043	-0.5217	9.64E-05	0.00239
EABT18878	-7.5761	-0.4731	9.64E-05	0.00239
EABT25541	-7.5761	-0.4731	9.64E-05	0.00239
EABT13977	-7.5761	-0.4731	9.64E-05	0.00239
EABT23771	-7.5761	-0.4731	9.64E-05	0.00239
EABT11569	-7.5761	-0.4731	9.64E-05	0.00239
EABT25216	-7.5761	-0.4731	9.64E-05	0.00239
EABT18346	-2.7217	7.19956	9.68E-05	0.0024
EABT11928	-3.4925	0.84716	9.81E-05	0.00243
EABT13050	-3.4925	0.84716	9.81E-05	0.00243
EABT6131	-3.823	0.46781	9.86E-05	0.00244
EABT13351	2.72738	5.9826	9.98E-05	0.00246
EABT32558	-2.7692	4.27689	0.0001	0.0025
EABT31811	-2.8959	2.88372	0.0001	0.0025
EABT8495	-2.7231	6.04026	0.0001	0.00251
EABT16282	-2.7109	6.97106	0.0001	0.00253
EABT26149	-2.7135	6.6553	0.0001	0.00255
EABT26832	-2.7835	3.91888	0.00011	0.00258
EABT30170	2.9016	2.4804	0.00011	0.0026
EABT18804	2.70827	5.83038	0.00011	0.00269
EABT12176	3.63122	0.78006	0.00011	0.00269
EABT13871	3.63122	0.78006	0.00011	0.00269
EABT29939	-3.4699	0.82758	0.00011	0.00269
EABT8659	-4.1245	0.21728	0.00011	0.00275
EABT32487	-3.6139	0.65895	0.00011	0.00275
EABT34933	-3.0473	1.95344	0.00011	0.00276
EABT16795	3.97114	0.40478	0.00011	0.00278
EABT32838	-2.721	4.88714	0.00012	0.00281
EABT28786	-2.7739	3.64643	0.00012	0.00281
EABT22158	3.17361	1.32389	0.00012	0.00283
EABT12621	-3.1304	1.71701	0.00012	0.00284
EABT7775	-3.3434	0.96061	0.00012	0.00288
EABT30742	-2.6974	5.64946	0.00012	0.0029
EABT29332	-2.8636	2.81087	0.00012	0.00298
EABT11051	2.68829	5.64286	0.00012	0.00298
EABT37153	-2.9073	2.60084	0.00012	0.00299
EABT34288	2.8469	2.718	0.00012	0.003
EABT11248	7.54663	-0.5769	0.00013	0.003
EABT33889	7.54663	-0.5769	0.00013	0.003
EABT34144	7.54663	-0.5769	0.00013	0.003
EABT5770	7.54663	-0.5769	0.00013	0.003
EABT2353	-7.5175	-0.5231	0.00013	0.003
EABT933	-2.696	5.16802	0.00013	0.00304
EABT19381	2.66312	7.82945	0.00013	0.00305
EABT21493	-2.8582	2.76001	0.00013	0.0031
EABT23473	-2.6627	7.13452	0.00013	0.00315
EABT16142	-3.7631	0.41617	0.00013	0.00315
EABT6002	-3.3223	0.94253	0.00013	0.00315

EABT25388	-2.654	8.90358	0.00013	0.00315
EABT21560	-2.7163	4.37887	0.00013	0.00317
EABT29326	-4.089	0.18674	0.00013	0.00317
EABT10593	-2.6499	8.25621	0.00014	0.00323
EABT14617	2.6496	7.34323	0.00014	0.0033
EABT10426	-2.6607	5.67418	0.00014	0.00336
EABT4569	2.65304	6.39121	0.00014	0.00336
EABT31359	-2.6705	5.36867	0.00014	0.00337
EABT15563	-2.6595	5.75383	0.00014	0.00337
EABT9671	-2.8541	2.44244	0.00015	0.0034
EABT31769	-3.5616	0.61388	0.00015	0.00345
EABT5417	-3.301	0.92422	0.00015	0.00346
EABT15935	-3.301	0.92422	0.00015	0.00346
EABT1664	-2.6496	5.99007	0.00015	0.00348
EABT34962	-3.7321	0.38963	0.00015	0.00361
EABT20167	-3.7321	0.38963	0.00015	0.00361
EABT2478	-2.7301	3.50869	0.00016	0.00362
EABT12919	-2.6335	6.70018	0.00016	0.00363
EABT2193	-4.6898	-0.1245	0.00016	0.00365
EABT2038	-4.6442	-0.1635	0.00016	0.00365
EABT16994	-4.6442	-0.1635	0.00016	0.00365
EABT707	-3.3996	0.76718	0.00016	0.00365
EABT30699	-3.3996	0.76718	0.00016	0.00365
EABT25790	-2.7487	3.14149	0.00016	0.00365
EABT21898	2.61972	8.23325	0.00016	0.00369
EABT9408	-4.0526	0.15551	0.00016	0.00369
EABT16673	-4.0526	0.15551	0.00016	0.00369
EABT20393	-3.1001	1.57123	0.00016	0.0037
EABT33358	7.47989	-0.6344	0.00016	0.00375
EABT34276	7.47989	-0.6344	0.00016	0.00375
EABT32423	7.47989	-0.6344	0.00016	0.00375
EABT30112	7.47989	-0.6344	0.00016	0.00375
EABT11509	7.47989	-0.6344	0.00016	0.00375
EABT12110	7.47989	-0.6344	0.00016	0.00375
EABT26956	-7.4565	-0.5748	0.00016	0.00375
EABT24442	-7.4565	-0.5748	0.00016	0.00375
EABT16479	-7.4565	-0.5748	0.00016	0.00375
EABT3718	-7.4565	-0.5748	0.00016	0.00375
EABT26028	-7.4565	-0.5748	0.00016	0.00375
EABT31535	-3.2794	0.90567	0.00017	0.00375
EABT31061	-3.2794	0.90567	0.00017	0.00375
EABT3264	-2.6653	4.54362	0.00017	0.00379
EABT22396	3.01145	1.54716	0.00017	0.00391
EABT31972	2.80326	2.39526	0.00018	0.00401
EABT1229	-3.3754	0.74646	0.00018	0.00403
EABT30451	-2.6058	6.82603	0.00018	0.00403
EABT5429	2.7779	2.65831	0.00018	0.00406
EABT35042	-3.7005	0.36259	0.00018	0.00407
EABT25858	-3.7005	0.36259	0.00018	0.00407
EABT14590	2.66419	3.94615	0.00018	0.00407
EABT32288	-3.123	1.46055	0.00018	0.00413
EABT18508	2.58859	7.5694	0.00019	0.00424
EABT34275	-2.5833	11.0155	0.00019	0.00427
EABT30045	-2.6511	4.28002	0.00019	0.00428

EABT25224	2.58498	8.11259	0.00019	0.00428
EABT318	-4.0152	0.12359	0.00019	0.00428
EABT30523	-4.0152	0.12359	0.00019	0.00428
EABT12997	-2.8072	2.34253	0.00019	0.00429
EABT23051	-3.3015	1.13101	0.00019	0.0043
EABT19482	-3.5073	0.56734	0.00019	0.0043
EABT22859	-3.4793	0.54349	0.00019	0.0043
EABT16928	-3.2186	1.23963	0.0002	0.00434
EABT8276	-4.5971	-0.2035	0.0002	0.00434
EABT32806	2.57855	8.97722	0.0002	0.00435
EABT26866	2.69693	3.31728	0.0002	0.00435
EABT18479	2.58059	7.61531	0.0002	0.00436
EABT22632	2.75654	2.63992	0.0002	0.00441
EABT17156	-2.6623	3.79415	0.0002	0.00443
EABT25370	-2.8296	2.23447	0.0002	0.00443
EABT5319	-2.5881	6.31566	0.0002	0.00445
EABT18303	3.83402	0.28492	0.00021	0.00464
EABT16832	3.83402	0.28492	0.00021	0.00464
EABT32331	3.83402	0.28492	0.00021	0.00464
EABT5020	3.07489	1.09349	0.00021	0.00465
EABT32090	-2.6768	3.41028	0.00021	0.00467
EABT13161	-3.1343	1.32678	0.00021	0.00469
EABT10865	7.4099	-0.6941	0.00022	0.00472
EABT2184	7.4099	-0.6941	0.00022	0.00472
EABT10022	7.4099	-0.6941	0.00022	0.00472
EABT10939	7.4099	-0.6941	0.00022	0.00472
EABT17625	7.4099	-0.6941	0.00022	0.00472
EABT33680	-7.3927	-0.6285	0.00022	0.00472
EABT11981	-7.3927	-0.6285	0.00022	0.00472
EABT11302	-7.3927	-0.6285	0.00022	0.00472
EABT20587	-7.3927	-0.6285	0.00022	0.00472
EABT7894	-7.3927	-0.6285	0.00022	0.00472
EABT18031	-7.3927	-0.6285	0.00022	0.00472
EABT25357	-7.3927	-0.6285	0.00022	0.00472
EABT27143	-2.5791	5.4588	0.00022	0.0048
EABT9044	-2.7355	2.74555	0.00023	0.00488
EABT27503	-2.6615	3.39716	0.00023	0.00488
EABT34458	-2.9342	1.76054	0.00023	0.0049
EABT21604	-2.5814	5.10137	0.00023	0.00491
EABT31231	-3.3258	0.70411	0.00023	0.00491
EABT33540	-2.5598	6.28117	0.00023	0.00494
EABT3404	-3.9769	0.09094	0.00023	0.00494
EABT37157	-3.9769	0.09094	0.00023	0.00494
EABT35980	-2.549	7.51984	0.00023	0.00498
EABT26930	3.30052	0.94526	0.00023	0.00499
EABT32159	-3.2641	1.09876	0.00023	0.00499
EABT664	4.73748	-0.275	0.00024	0.00518
EABT8434	-4.5484	-0.2447	0.00024	0.00518
EABT617	-4.5484	-0.2447	0.00024	0.00518
EABT3234	-4.5484	-0.2447	0.00024	0.00518
EABT35631	-4.5484	-0.2447	0.00024	0.00518
EABT172	-4.5484	-0.2447	0.00024	0.00518
EABT7524	-2.5616	5.51546	0.00024	0.00519
EABT15126	-2.6094	3.8664	0.00025	0.00522

EABT35145	-2.7279	2.64809	0.00025	0.00535
EABT4110	-2.6676	3.16993	0.00026	0.00541
EABT9380	-3.4218	0.49455	0.00026	0.00541
EABT9950	2.52544	9.56096	0.00026	0.00543
EABT22249	-2.7304	2.6025	0.00026	0.00556
EABT31272	-2.6156	3.67041	0.00026	0.00556
EABT13086	-2.6299	3.34312	0.00027	0.00578
EABT14939	-2.6191	3.38759	0.00028	0.00582
EABT26259	-2.644	3.01792	0.00028	0.00583
EABT9510	4.0681	-0.0251	0.00028	0.00583
EABT1389	-3.9375	0.05751	0.00028	0.00583
EABT31681	-2.637	3.26474	0.00028	0.00587
EABT18291	-2.7262	2.54949	0.00029	0.00599
EABT11294	7.33635	-0.7564	0.00029	0.00602
EABT24422	7.33635	-0.7564	0.00029	0.00602
EABT17948	7.33635	-0.7564	0.00029	0.00602
EABT19614	7.33635	-0.7564	0.00029	0.00602
EABT26989	7.33635	-0.7564	0.00029	0.00602
EABT35951	7.33635	-0.7564	0.00029	0.00602
EABT2364	-7.326	-0.6844	0.00029	0.00602
EABT19861	-7.326	-0.6844	0.00029	0.00602
EABT15476	-7.326	-0.6844	0.00029	0.00602
EABT9521	-7.326	-0.6844	0.00029	0.00602
EABT37053	-7.326	-0.6844	0.00029	0.00602
EABT512	-3.6013	0.27823	0.00029	0.00603
EABT17270	-3.6013	0.27823	0.00029	0.00603
EABT22668	-2.5263	5.32552	0.00029	0.00603
EABT32495	3.41353	0.59033	0.00029	0.00607
EABT37241	-3.2744	0.66045	0.00029	0.00607
EABT13721	-2.5316	5.13539	0.0003	0.00608
EABT19435	-2.5519	4.47077	0.0003	0.00609
EABT4123	2.49862	7.83603	0.0003	0.00613
EABT6963	2.58218	3.50156	0.0003	0.00616
EABT19712	-2.7696	2.04362	0.0003	0.00617
EABT37320	-2.6274	3.19745	0.0003	0.00623
EABT33789	-2.7406	2.1586	0.00031	0.00623
EABT19033	-2.7862	1.9825	0.00031	0.00623
EABT26985	4.68328	-0.3216	0.00031	0.00623
EABT1886	4.68328	-0.3216	0.00031	0.00623
EABT808	-4.498	-0.2872	0.00031	0.00623
EABT16534	-4.498	-0.2872	0.00031	0.00623
EABT28858	-4.498	-0.2872	0.00031	0.00623
EABT30357	2.498	6.9725	0.00031	0.00625
EABT20331	-2.5477	4.13277	0.00031	0.00629
EABT11699	-2.9722	1.46158	0.00031	0.00631
EABT15633	-2.4821	8.1535	0.00032	0.00656
EABT2921	-2.7449	2.09413	0.00033	0.00662
EABT19847	-2.532	4.30298	0.00033	0.00666
EABT2455	-2.5759	3.42783	0.00033	0.00671
EABT7414	2.94569	1.12766	0.00034	0.00678
EABT801	-3.897	0.02329	0.00034	0.00679
EABT29677	-3.897	0.02329	0.00034	0.00679
EABT2005	-2.4695	10.9808	0.00034	0.00685
EABT13504	-3.3618	0.44386	0.00034	0.00687

EABT25789	-3.3618	0.44386	0.00034	0.00687
EABT5424	-3.5666	0.24895	0.00034	0.00691
EABT10550	-3.0003	1.35542	0.00034	0.00692
EABT7491	-3.0003	1.35542	0.00034	0.00692
EABT3166	-2.4849	5.88427	0.00035	0.007
EABT22310	-2.4673	7.03441	0.00036	0.00714
EABT19433	-2.4644	7.28338	0.00036	0.00716
EABT28978	-2.7034	2.37095	0.00036	0.00725
EABT9355	-3.118	0.76861	0.00037	0.00737
EABT33223	-2.5011	4.61759	0.00037	0.00738
EABT19095	-2.5837	3.19025	0.00037	0.00741
EABT20046	-2.4517	8.90125	0.00037	0.00745
EABT36005	-3.1457	0.99738	0.00038	0.0076
EABT21473	-3.2211	0.61541	0.00038	0.0076
EABT35668	2.44904	7.70351	0.00038	0.00761
EABT10404	-2.4689	5.5433	0.00038	0.00763
EABT20871	4.62695	-0.3696	0.00039	0.00763
EABT32396	-4.4458	-0.3309	0.00039	0.00763
EABT36393	-4.4458	-0.3309	0.00039	0.00763
EABT17611	-2.4438	10.8155	0.00039	0.00764
EABT31307	-2.7605	1.88135	0.00039	0.00764
EABT7586	7.25884	-0.8215	0.00039	0.00764
EABT985	7.25884	-0.8215	0.00039	0.00764
EABT22229	7.25884	-0.8215	0.00039	0.00764
EABT25850	7.25884	-0.8215	0.00039	0.00764
EABT25737	7.25884	-0.8215	0.00039	0.00764
EABT4581	7.25884	-0.8215	0.00039	0.00764
EABT22418	7.25884	-0.8215	0.00039	0.00764
EABT16512	-7.2561	-0.7425	0.00039	0.00764
EABT14614	-7.2561	-0.7425	0.00039	0.00764
EABT16220	-7.2561	-0.7425	0.00039	0.00764
EABT3509	-7.2561	-0.7425	0.00039	0.00764
EABT6409	-7.2561	-0.7425	0.00039	0.00764
EABT8344	-7.2561	-0.7425	0.00039	0.00764
EABT30189	2.4407	11.8483	0.00039	0.00765
EABT32579	-3.3309	0.41783	0.0004	0.0077
EABT25010	-3.3309	0.41783	0.0004	0.0077
EABT37792	-2.5232	3.69613	0.0004	0.00777
EABT28526	-2.4986	4.0761	0.0004	0.00777
EABT25091	-2.5822	3.06557	0.0004	0.00781
EABT22097	-3.8554	-0.0118	0.00041	0.00792
EABT13133	2.53529	3.30384	0.00043	0.00834
EABT2099	-2.4831	4.01666	0.00044	0.00847
EABT17491	-2.6023	2.63245	0.00044	0.00847
EABT6328	-2.5207	3.33043	0.00044	0.00857
EABT4249	2.48378	3.99228	0.00045	0.00863
EABT21671	-2.4401	5.29739	0.00046	0.00879
EABT17501	2.40939	8.68688	0.00046	0.00891
EABT21262	-2.9808	1.19576	0.00046	0.00891
EABT25138	3.21426	0.66287	0.00047	0.00904
EABT7830	-3.4947	0.18854	0.00048	0.00926
EABT7581	-2.5636	2.72622	0.00048	0.00928
EABT36880	4.56834	-0.4193	0.00049	0.00934
EABT37981	4.56834	-0.4193	0.00049	0.00934

EABT21365	4.56834	-0.4193	0.00049	0.00934
EABT17685	4.56834	-0.4193	0.00049	0.00934
EABT7664	4.56834	-0.4193	0.00049	0.00934
EABT8669	-4.3916	-0.3761	0.00049	0.00934
EABT18105	-2.4161	5.83054	0.00049	0.00937
EABT25213	3.97534	-0.1044	0.0005	0.00948
EABT23532	-3.8125	-0.0478	0.0005	0.00948
EABT9394	-3.8125	-0.0478	0.0005	0.00948
EABT30864	-3.1372	0.54506	0.0005	0.00951
EABT16575	2.56015	2.55625	0.00052	0.0098
EABT7715	2.40422	5.72316	0.00052	0.0099
EABT36172	-7.1826	-0.8031	0.00053	0.00993
EABT36065	-7.1826	-0.8031	0.00053	0.00993
EABT19938	-7.1826	-0.8031	0.00053	0.00993
EABT7393	-7.1826	-0.8031	0.00053	0.00993
EABT27691	-7.1826	-0.8031	0.00053	0.00993
EABT4775	-7.1826	-0.8031	0.00053	0.00993
EABT14061	-7.1826	-0.8031	0.00053	0.00993
EABT21301	-7.1826	-0.8031	0.00053	0.00993
EABT32742	-7.1826	-0.8031	0.00053	0.00993
EABT25895	-7.1826	-0.8031	0.00053	0.00993
EABT36639	7.17693	-0.8895	0.00053	0.00993
EABT12249	7.17693	-0.8895	0.00053	0.00993
EABT7376	7.17693	-0.8895	0.00053	0.00993
EABT18884	7.17693	-0.8895	0.00053	0.00993
EABT10096	7.17693	-0.8895	0.00053	0.00993
EABT23951	7.17693	-0.8895	0.00053	0.00993
EABT9067	7.17693	-0.8895	0.00053	0.00993
EABT7309	-2.4161	4.80405	0.00053	0.00995
EABT29038	2.38107	8.36008	0.00053	0.00996
EABT18680	-3.2669	0.36428	0.00053	0.00996
EABT996	-2.6009	2.16779	0.00053	0.00996
EABT14831	-2.573	2.56301	0.00053	0.00996
EABT20169	-2.6558	1.94763	0.00054	0.00998
EABT10584	2.71608	1.66899	0.00054	0.00999
