# Supplementary material for: Transcriptome Analysis of the Emerald Ash Borer (EAB), Agrilus planipennis: De Novo Assembly, Functional Annotation and Comparative Analysis
Source: PLoS One. 2015 Aug 5;10(8):e0134824. doi: 10.1371/journal.pone.0134824 (PMC4526369; doi:10.1371/journal.pone.0134824)
Supplement: S5 Table — (DOCX) [file pone.0134824.s008.docx]

| No. | Gene ID | Primers used to validate RNA-seq (5' to 3') | | PCR Product size(bp) | RNA-Seq  (Log_2_^FC^) | qRT-PCR  (Log_2_^FC^) |  |
| --- | --- | --- | --- | --- | --- | --- | --- |
|  |  | Forward | Reverse |  |  |  |  |
| 1 | EABT36748 | TTCGTGGCTGCTGACTTCGTA | AAGACAATACAGATGGCGGCAA | 142 | -9.18 | -10.39±0.20 | EAB3M/EAB4F |
| 2 | EABT26334 | CGGTATGTGGCGAATCATGTC | CGGTGTTAGCTGGAGAATCTTC | 158 | -3.41 | -3.24±0.71 |  |
| 3 | EABT1664 | ATCCAACCGAAGGACCGCATCA | CCTCCCTGAGCGTAAGCCTCTT | 131 | -6.08 | -1.44±0.36 |  |
| 4 | EABT37717 | GGATTGGACCGCTTCTGTGAAT | GAAAGAGCCCGAGGTTGAGGAA | 122 | -6.74 | -3.30±0.34 |  |
| 5 | EABT755 | GGCTCTTATCACCACACCACCT | GGGTTGTTTCCGCAGCTCACT | 104 | 11.75 | 12.75±0.18 |  |
| 6 | EABT22472 | TATTCACTCAGCGCCGCCAAT | GCCTTTCCAACCATGCACCAT | 124 | 9.29 | 10.27±0.08 |  |
| 7 | EABT11324 | CGACGACTTCTTCACCGACTG | TCCCTTAGCGCATTGGCTCAA | 100 | 8.82 | 6.88±0.28 |  |
| 8 | EABT14053 | TGTGATGAGCCGGAGCAGAAGT | GCGTCCTGTACGACAGTCACCT | 111 | 7.32 | 13.94±0.18 |  |
| 9 | EABT30570 | GAGCAGCGTAAGCAACTGGTG | CCATCTCCGAAAGCCGATCCAT | 136 | -9.95 | -15.68±0.42 | EABPP/EABP0 |
| 10 | EABT27511 | CCACGATGCTGTGTTCTGTGAC | CGAGGCCGTCTTGAGAATGGT | 117 | -9.63 | -10.93±0.35 |  |
| 11 | EABT36884 | CTACGCAGCACATCCAACACCT | CGTAAGCCAAAGGAGCCGATGC | 101 | -9.29 | -10.75±0.29 |  |
| 12 | EABT35689 | CAGAGCCTTGGCAGCTACAACT | CGGTCTTTAGAGCGGCGTCAT | 104 | -9.04 | -10.91±0.46 |  |
| 13 | EABT19583 | CGTCTTCACCCTTGTCGCACTT | ACCGTCACTACCATAGCCACCA | 127 | 10.97 | 10.18±0.33 |  |
| 14 | EABT23189 | GCCGCAAATGAACCAGACGATA | CGGAACCGCCAGCTTCTAATAG | 148 | 10.00 | 9.83±0.56 |  |
| 15 | EABT23473 | GTCGTGGTCACCGTCTCTTGAT | ACAACTCCGTCAGACTTAGCCA | 126 | 8.96 | 8.88±0.10 |  |
| 16 | EABT4817 | CGCTACCAGAACACGATAACA | CCGAATGTGGTTACTGATGGA | 134 | 7.27 | 7.63±0.23 |  |
| 17 | EABT14338 | CGTCACCTACGAGCAACTT | TCCACCTCAACAACTTCATCTT | 134 | -5.08 | -6.92±0.23 | EABLM/EABAM |
| 18 | EABT16135 | GTGGTAGTGGTCTCTGCGGATT | CCATGCCTTCTCTAAGCAACCT | 147 | -10.59 | -9.46±0.42 |  |
| 19 | EABT21639 | TCGCCTTGTTCGTCAGATGTGT | TCGCCGATTCCACCGAAGAA | 148 | -9.70 | -5.31±0.41 |  |
| 20 | EABT4315 | CCGCATTGGCTAAGTGACA | ATCTGTATGCTTCAACCTCGTT | 172 | -4.86 | -4.49±0.23 |  |
| 21 | EABT37729 | CCAGTCGAACAGTTGCCGTCAA | GGCGAAACAGTGGAGACAATGC | 115 | 11.96 | 11.98±0.26 |  |
| 22 | EABT33854 | AGCTTCCAATGCCTGGCCTATT | CGACTGTGTCTGCTGTGCTGAT | 128 | 11.12 | 7.77±0.78 |  |
| 23 | EABT7214 | TTGGTGGCGTTCTGACATAGTG | TGTTGACTTGGATCGAGGAGGA | 138 | 11.99 | 15.08±0.10 |  |
| 24 | EABT36743 | CCAATGTTGCCAATCCACTGGT | TGTAACGCTTAGTCCTGCCTCT | 138 | 11.54 | 13.00±0.38 |  |
| 25 | TEF-1α | CATTGAAACCTACGTTGTCGC | ACTGGAGTGCTTAAACCTGG | 130 |  | | Reference gene |
